# Meta-Prism 2.0: Enabling algorithm and web server for ultra-fast, memory-efficient, and accurate analysis among millions of microbial community samples

**DOI:** 10.1093/gigascience/giac073

**Published:** 2022-07-28

**Authors:** Kai Kang, Hui Chong, Kang Ning

**Affiliations:** Key Laboratory of Molecular Biophysics of the Ministry of Education, Hubei Key Laboratory of Bioinformatics and Molecular-imaging, Center of AI Biology, Department of Bioinformatics and Systems Biology, College of Life Science and Technology, Huazhong University of Science and Technology, Wuhan 430074, China; Center for Quantitative Biology, Academy for Advanced Interdisciplinary Studies, Peking University, Beijing 100871, China; Key Laboratory of Molecular Biophysics of the Ministry of Education, Hubei Key Laboratory of Bioinformatics and Molecular-imaging, Center of AI Biology, Department of Bioinformatics and Systems Biology, College of Life Science and Technology, Huazhong University of Science and Technology, Wuhan 430074, China; Key Laboratory of Molecular Biophysics of the Ministry of Education, Hubei Key Laboratory of Bioinformatics and Molecular-imaging, Center of AI Biology, Department of Bioinformatics and Systems Biology, College of Life Science and Technology, Huazhong University of Science and Technology, Wuhan 430074, China

## Abstract

**Background:**

Microbial community samples have been accumulating at a speed faster than ever, with hundreds of thousands of samples been sequenced each year. Mining such a huge amount of multisource heterogeneous data is becoming an increasingly difficult challenge, so efficient and accurate compare and search of samples is in urgent need: faced with millions of samples in the data repository, traditional sample comparison and search approaches fall short in speed and accuracy.

**Findings:**

Here we proposed Meta-Prism 2.0, a microbial community sample analysis method that has pushed the time and memory efficiency to a new limit without compromising accuracy. Based on sparse data structure, time-saving instruction pipeline, and SIMD optimization, Meta-Prism 2.0 has enabled ultra-fast, memory-efficient, flexible, and accurate search among millions of samples. Meta-Prism 2.0 was put to test on several data sets, with the largest containing 1 million samples. Results show that Meta-Prism 2.0’s 0.00001-s per sample pair compare speed and 8-GB memory needs for searching against 1 million samples have made it one of the most efficient sample analysis methods. Additionally, Meta-Prism 2.0 can achieve accuracy comparable with or better than other contemporary methods. Third, Meta-Prism 2.0 can precisely identify the original biome for samples, thus enabling sample source tracking. Finally, we have provided a web server for fast search of microbial community samples online.

**Conclusions:**

In summary, Meta-Prism 2.0 has changed the resource-intensive sample search scheme to an effective procedure, which could be conducted by researchers every day even on a laptop, for insightful sample search, similarity analysis, and knowledge discovery. Meta-Prism 2.0 can be accessed at https://github.com/HUST-NingKang-Lab/Meta-Prism-2.0, and the web server can be accessed at https://hust-ningkang-lab.github.io/Meta-Prism-2.0/.

## Background

Microbial communities have asserted great influences on health care, environment, and industry [1–4]. As such, an increasing number of projects have been conducted on microbial communities around the world, such as those from the “Human Microbiome Project” [1, 2] and the “Earth Microbiome Project” [3, 4]. Mining this massive amount of samples has already discovered knowledge about the microbial community and their effects on the environment and human health [5, 6], providing an opportunity to study the hidden evolution and ecology patterns among microbial communities.

A microbial community sample (also referred to as the sample) is represented by the hierarchically structured taxa (species, genus, families, etc.) and their relative abundances (also referred to as the community structure), and these species are functioning in concert to maintain stability and adapt to the specific environments (also referred to as the niches or biomes) where the microbial community is living. These samples’ community structures are often associated with the biomes and a variety of characteristics of the biomes. For example, the community structures of the human gut microbiome have been linked to multiple aspects of human life, such as health [6, 7], early development [8], immigration [9], and pregnancy [10]. Thus, there is a large amount of hidden information in the community structures and remains to be discovered. These challenges in current microbiome research are calling for a fast community-level comparison and search among the rapidly accumulating number of microbial communities.

Methods already existed for comparison and search of samples. The distance-based methods are the first batches designed for the purpose, whose primary strategy is to compare the similarity or distance between 2 samples. The simplest distance-based method is the Jensen–Shannon divergence (JSD) measurement [11], which only considered species abundances in the community. More advanced distance-based methods considered both species abundances and their phylogenetic relationships. For example, UniFrac [12] is a typical distance-based method, which first maps their respective sets of taxon abundances on the phylogenetic tree and, second, traverses the tree and executes operation at each node (each representing a taxon on the phylogenetic tree) to calculate their similarity. Fast UniFrac [13] and Meta-Storms [14] optimized such a procedure by changing tree traversal to an array loop. Striped UniFrac [15] further optimized matrix similarity comparison by reorganizing samples. Dynamic Meta-Storms enables species-level accuracy by introducing virtual nodes [16]. Previously, we designed Meta-Prism 1.0, a fast and accurate microbial community sample search tool [17]. Meta-Prism 1.0 generates an index to rapidly select samples with a similar biome and top phylum for comparison. Furthermore, Meta-Prism 1.0 uses GPU to accelerate comparison. However, given that more than a million community samples have already been deposited into public databases [18, 19], state-of-the-art methods including Meta-Prism 1.0 face difficulties in comparison and searching among these samples, while rendering knowledge discovery from samples formidable. Additionally, microbial community samples' data are very sparse. These methods use fixed-length arrays to save abundances with lengths equal to the entities number of the phylogenetic tree, wasting a considerable amount of memory. They also spend much time operating on these empty nodes.

To solve the large-scale microbial community sample search problem, we have redesigned and updated Meta-Prism to its second version (Meta-Prism 2.0). Facing the large and sparse characteristics of microbial samples, we adopted a special sparse storage format and a fast 1-N calculation method. This greatly reduces memory usage and time consumption. As the computing and storage efficiency increases, it adds a similarity matrix calculation function to analyze more than 100,000 samples' beta diversity. When searching samples, due to the efficiency improvement, Meta-Prism 2.0 no longer needs to generate the index system to select high-probability subsets for similarity comparison but conducts an exhaustive search against the entire database. Thus, it has higher flexibility (when searching among customized data sets) and robustness than Meta-Prism 1.0. More important, with these improvements of efficiency and space, Meta-Prism 2.0 now can deal with 1 million or even more microbial community samples and is one of the fastest microbial community sample search methods to date.

Using several data sets including the largest one containing a million samples, we demonstrated that it can achieve at least 20 times speed-up compared to the contemporary approach (e.g., Meta-Prism 1.0 and Striped UniFrac), and Meta-Prism 2.0 is the only method that can handle the search against a million samples. The memory utilization is also very efficient: compared with other methods including JSD, Striped UniFrac, and Dynamic Meta-Storms, when analyzing data set beta diversity, which has a size that exceeds 10,000, Meta-Prism 2.0 can at least save 80% of memory space needed. Though we have saved time and memory by magnitudes, the accuracy is not compromised. For example, Meta-Prism 2.0 obtained an area under the curve (AUC) of 0.99 in distinguishing samples from different biomes for more than 100,000 samples [20]. Meta-Prism 2.0 has changed the traditional computational resource-intensive sample search to a cheap and effective procedure that could be conducted by researchers every day, for the discovery of intricate relationships among samples. Meta-Prism 2.0 can be accessed at https://github.com/HUST-NingKang-Lab/Meta-Prism-2.0. Also, the fast and accurate microbial community sample search can be experienced on the web server at https://hust-ningkang-lab.github.io/Meta-Prism-2.0/.

## Findings

### Materials and execution environments used for evaluation

Through manual curation from the EBI MGnify database [18], we obtained a data set consisting of 126,727 microbial community samples belonging to 114 different biomes, defined as the Combined data set. We also generated a data set that consists of 10,270 samples, which have been used in the FEAST study [21], defined as the FEAST data set (Table 1). According to the biome annotation of the EBI MGnify database, we categorized these samples into 3 biomes: Fecal, Human (such as forehead, skin, oral, sebum), and Mixed (such as doorknobs, kitchen counter, light switch). Details of all samples in the FEAST data set are provided in Supplementary Table S1. To evaluate Meta-Prism 2.0’s speed and memory efficiency on the scale of 1 million samples, we synthesized a data set with 1,000,010 samples based on the Combined data set. All samples from these 3 data sets are accessible from https://github.com/HUST-NingKang-Lab/Meta-Prism-2.0. We used SILVA 132 LTPs132 SSU phylogenetic tree [22] in all experiments included in this study.

**Table 1:** The Combined data set and FEAST data set used in this study. Details are provided in Supplementary Table S1

Data set	Combined data set	FEAST data set
Top-level biome	Root	Human gut
Number of biomes involved	114	3
Number of samples	126,727	10,270
Number of species	45,477	5,762
Average number of species per sample	411.22	111.05
Notes	Selected samples from MGnify database	Selected samples from the FEAST study

Striped UniFrac, Dynamic Meta-Storms, and Meta-Prism 2.0 were compiled by GCC 4.8.5 and run on CentOS 6.7 with Intel Xeon CPU E5-2678 v3 @ 2.50 GHz and 252 GB memory. The JSD was calculated utilizing Python 3.7.3 and SciPy 1.4.1 and run on the same CentOS device. The executable Meta-Prism 2.0 steps’ time usage was compiled by clang-1100.0.33.16, evaluated by Xcode11.5 Instruments Time Profiler, and run on macOS 10.15 with Intel Core i7-9750H and 32 GB memory. Meta-Prism GPU version was compiled by NVCC 10.1 and run on RTX 2080Ti.

### Meta-Prism 2.0 outperforms other methods in source tracking accuracy

We assessed the search accuracy of different methods in the context of source tracking, namely by checking the consistency of the predicted biomes and query samples' actual biomes. This evaluation is based on the realization that the microbial communities collected from the same biome always share similar patterns in their taxonomical structures and relative abundances [20, 21]. Specifically, we used simple cross-validation for the evaluation, based on searching 12.5% randomly chosen samples (considered the query data set) against the rest of the samples (considered the target data set). For each query sample, we selected the top 100 most similar target samples. The similarity of these samples is then summed by biome and normalized by dividing by the total number of samples in the source data set for each biome. After the resulting values are normalized, it is the probability that the test sample belongs to each biome.

The evaluation performances are shown in Fig. 2. The varying classification threshold that generates different sensitivities and specificities ranges from 0.01 to 1 with a fixed step size of 0.01. On the FEAST data set, each method predicted the biome for testing samples according to the biomes included in the source data set (Fecal, Human, and Mixed). Distance-based phylogenetic tree approaches (Meta-Prism 2.0, Striped UniFrac, and Dynamic Meta-Storms) showed similarly good performance, while JSD obtained a lower AUC of 0.9512. On the Combined data set, each method predicted the biome for testing samples according to 114 biomes included in the source data set (87.5% of the Combined data set). JSD and Dynamic Meta-Storms could not finish the calculation within an acceptable time (10 days). We only compared Meta-Prism 2.0 and Striped UniFrac. Meta-Prism obtained a higher AUC result of 0.9934, while Striped UniFrac's AUC result was 0.9153.

### Meta-Prism 2.0 shows high efficiency with regard to time and memory

The time and memory efficiency are the most profound advantage of Meta-Prism 2.0. We first assessed Meta-Prism 2.0’s speed based on using data sets with different data set sizes and using different numbers of CPU threads (Fig. 3). The setting was matrix mode, which takes 1 data set as input and then calculates all sample pairs’ similarities, and the output is a similarity matrix. The time cost is split into several parts according to computational steps. Our 1-N module adds GenOrder and Convert steps, which increase linearly and quadratically with the increase of data set size, respectively.

We also evaluated Meta-Prism 2.0 performance on a data set with 1 million samples (see Materials for details). Meta-Prism 2.0 can efficiently package 1 million samples into a 369-MB-sized file for storage and load them within 27 seconds. We transferred the whole workload to a laptop and searched 100 samples against this data set with a single CPU thread. It took 324.96 seconds (less than 6 minutes) of CPU time to complete the search using only 6.9 GB memory. So far as we know, Meta-Prism 2.0 is the only method that can handle the search against a million samples.

We further selected data sets with different data set sizes (10, 100, 1,000, 10,000, 100,000, and 126,727) from the Combined data set to compare different methods. The setting was again matrix mode. We compared time and memory usage of Striped UniFrac, Dynamic Meta-Storms, JSD, Meta-Prism GPU, and Meta-Prism 2.0. Meta-Prism GPU is the only method that uses GPU for calculation, and we considered real-time usage for the measurement. In comparison, we took CPU core time usage as other methods’ time usage. JSD and Meta-Storms cannot calculate the similarity matrix when data set size is $\ge $10,000 within an acceptable time (10 days).

Results show that Meta-Prism 2.0 could achieve superior performance on both time usage and memory usage (Fig. 4). Specifically, when the data set sizes were no more than 1,000, Meta-Prism 2.0 used a similar core time compared with Striped UniFrac (Fig. 4A). When data set size became more extensive, the performance gap between Meta-Prism 2.0 and Striped UniFrac became larger. When calculating the similarity matrix for the Combined data set (generating 126,727 $\times $ 126,727 similarity matrix), Meta-Prism 2.0 was 55 times faster than Striped UniFrac. Meta-Prism GPU's real-time usage was smaller than Meta Prism 2.0's core time usage. However, when Meta-Prism 2.0 uses 3 CPU cores or more, it will be faster than Meta-Prism GPU.

Meta-Prism 2.0’s memory usage was only 11.1% of Striped UniFrac's when calculating the similarity matrix for the Combined data set with more than 100,000 samples. The utilization of a customized 16-bit floating point was the key reason because it can efficiently store the similarity matrix, which is the largest storage burden that increases quadratically when the data set size increases.

Wondering how far is the speed of Meta-Prism 2.0 to the theoretical lower bound for the sample search, we took IO Only (the time used solely for loading data and writing matrix calculation results, without any computation involved) as the lower bound for the sample search (Fig. 5). The result shows that the time cost of Meta-Prism 2.0 is only 2 times of IO Only but a magnitude smaller than that of Striped UniFrac.

### Meta-Prism 2.0 shows high accuracy in real data applications

Meta-Prism 2.0 can precisely identify the biome for samples of unknown origin, thus enabling the source tracking of samples. For example, it enables accurate differentiation of samples from close biomes such as “human skin” and “human oral” (the first application), identification of the biome for samples with unclear origin (the second application), and detection of microbial contamination (the third application). We published these applications' workflow at Code Ocean for researchers to track and reproduce (https://codeocean.com/capsule/3103931).

First, we tested Meta-Prism 2.0’s ability to accurately differentiate samples from close biomes. We obtained 1,261 skin metagenomic samples (MGYS00005172) [23] and 70 oral metagenomic samples (MGYS00005569) [24] from MGnify [18]. We used Meta-Prism 2.0 to calculate the similarities matrix of 1,331 samples on a laptop, which took only 3.75 seconds and 11 MB of memory. We also clustered samples based on their similarities by using affinity propagation from Scikit-learn (version 0.20.3). The samples were successfully clustered into 2 groups whose sizes were 1,260 and 71 (Fig. 6). Within 1,331 samples, only 9 samples (5 skin samples and 4 oral samples) were misclustered, proving Meta-Prism 2.0’s ability to quickly and accurately differentiate samples from close biomes.

Second, we evaluated the performance of Meta-Prism 2.0 on source tracking environmental samples from less-studied biomes, based on searching 11 groundwater samples curated from Saudi Arabia (MGYS00001601) [25] against the combined data set. The biome “groundwater” is less studied, with a handful of samples in the combined data set (MGYS00005245). Results show that Meta-Prism 2.0 could successfully identify source-related biomes for samples from “groundwater.” Within the top 100 most similar community samples for each “groundwater” query sample, there are on average 64 groundwater-related samples (from “root-Environmental-Terrestrial,” “root-Environmental-Aquatic,” “root-Engineered-Wastewater” and “root-Host-associated-Plants”) for each query sample (Table 2). Nevertheless, there is no “groundwater” sample in the top 100 similar samples searched by Meta-Prism 2.0, since “groundwater” samples in the combined data set are curated from New Zealand, which is in nature drastically different from our query samples. The result suggests that the geographic origins also influence the community structures, which was already confirmed by previous studies [26].

**Table 2:** The search results of 11 groundwater samples against the Combined data set. Count groundwater-related samples in the top 100 matching samples

Sum of groundwater-related samples:								
53	58	75	53	38	88	56	79	73

Finally, we evaluated the Meta-Prism 2.0’s power in detecting microbial contamination. We investigated the contamination of an indoor house surface community by selecting 611 samples from indoor house surfaces in Chicago as query samples and searching against 6,285 samples (899 + 3,773 + 721 + 692 + 200 from “human skin,” “environmental,” “mammal,” “plants,” and “insecta,” respectively). The analysis took only 6.16 seconds to complete. Our results show that the most closed biome source for indoor house surface samples is “human skin” (average similarity 0.889), indicating a large proportion of microbial community contamination from human skin, which agrees with previous analyses by SourceTracker [20] and FEAST [21] (Table 3). Again, it proved the ability of Meta-Prism 2.0 for accurate and fast microbial community contamination screening.

**Table 3:** Average similarities of biomes between 611 sink samples

	Environmental	Human skin	Insecta	Mammal	Plants
Average similarity	0.7886	0.8896	0.7849	0.8478	0.8313

### Meta-Prism 2.0 web server for fast and accurate sample search

For easy use of Meta-Prism 2.0, we also designed an online web server for Meta-Prism 2.0 (Fig. 7), with a precompiled Meta-Prism 2.0 executable file and a built-in data set containing more than 0.2 million microbiome samples. This data set includes major categories such as digestive system, aquatic, and soil, as well as subcategories such as oil-contaminated clay, thermal springs sediment, and bioreactor for biological phosphorus removal. The high efficiency of Meta-Prism 2.0 enables any query against this huge data set to be completed within 1 second, with high accuracy.

## Methods

**Figure 1:**
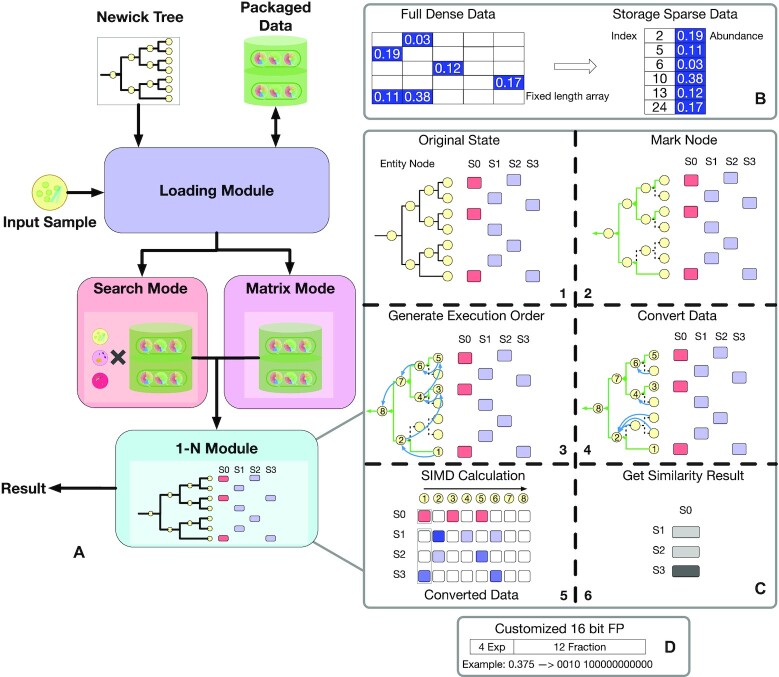
The Meta-Prism 2.0 pipeline with key optimization highlighted. (A) Meta-Prism 2.0 takes taxa abundance as input data, maps data to the phylogenetic tree, and converts data to sparse abundance data for space optimization. Meta-Prism 2.0 organizes data according to search mode or matrix mode, then uses the 1-N module to calculate similarities. (B) Space-saving scheme packages sample data to the sparse format for storage, cutting down both disk and memory usage. For example, when an evolutionary tree has a total of 25 nodes, and one of the samples has 5 nonzero nodes, a dense format will store all nodes in a fixed array, while a sparse format will only store the abundance and sum of 5 nonzero nodes. (C) The 1-N module saves resources to the maximum extent by removing redundant nodes without losing their abundances and fix the execution order for fast 1-against-N sample comparison (1–4), followed by SIMD optimization as a compiler-level optimization (5). The dashed lines indicate branches and nodes to be removed. The black arrows indicate an execution order to be recorded (postorder traversal), and the blue arrows indicate abundance aggregation from those to-be-removed nodes to their ancestors. (D) The similarities are saved in the format of a customized 16-bit floating point. Pseudocode about Meta-Prism 2.0 can be accessed from Supplementary Material 1.

Meta-Prism 2.0 calculates similarities between microbial communities using 2 calculation modes: search mode and matrix mode. The search mode takes 2 data sets (query and target) as input and then outputs each query sample's top N similar matches in the target data set. The matrix mode takes a data set as input and outputs a pairwise similarity matrix for all samples in the data set (Fig. 1A). These data sets can be produced by commonly used tools such as QIIME [27], MAPseq [28], and MetaPhlAn [29].

**Figure 2:**
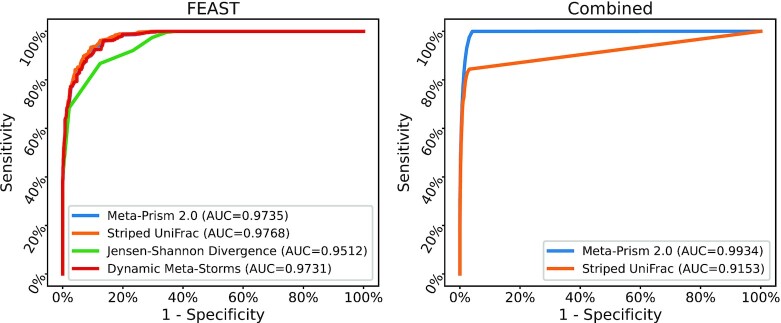
AUC of different methods for sample searches using the FEAST data set and the Combined data set. Note that all these methods could complete the analysis in due time and reach a good AUC on the FEAST data set, whereas Jensen–Shannon divergence and Dynamic Meta-Storms couldot complete the analysis on the Combined data set.

**Figure 3:**
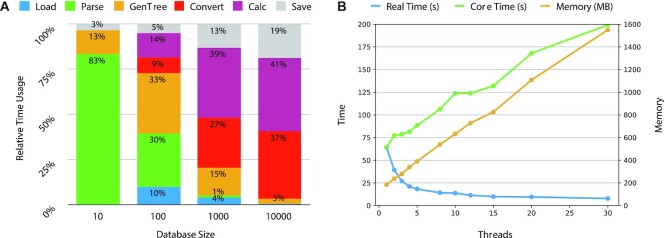
Time usage at different steps and multithread performance analysis of Meta-Prism 2.0. (A) Each step’s time usage with variate sample sizes. Load: load data. Save: save matrix result. Parse: load and parse phylogenetic tree. GenOrder: generates nonredundant phylogenetic tree (without redundant nodes) in 1-N module. Convert: convert sample data from spare format to dense format for the sample comparison. Calc: 1-against-N sample comparison. A higher proportion of total time was used by Convert and Calc steps when the number of sample pairs increased. (B) Time and memory usage for 10,000 samples’ pairwise similarity calculation using the different numbers of CPU threads. Real-time: the actual time usage of calculation. Core Time: the sum of each CPU core’s time usage.

**Figure 4:**
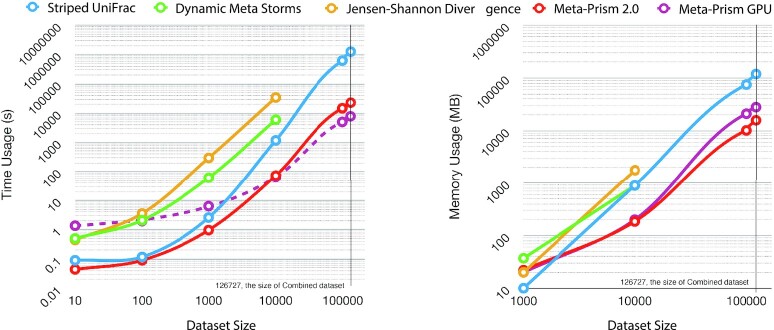
Time and memory usage of samples when calculating similarity matrix for data sets with different numbers of samples. (A) Time usage comparison. (B) Memory usage comparison. In (A), Meta-Prism GPU time usage with the dashed line is GPU time usage; others are CPU core time usage.

**Figure 5:**
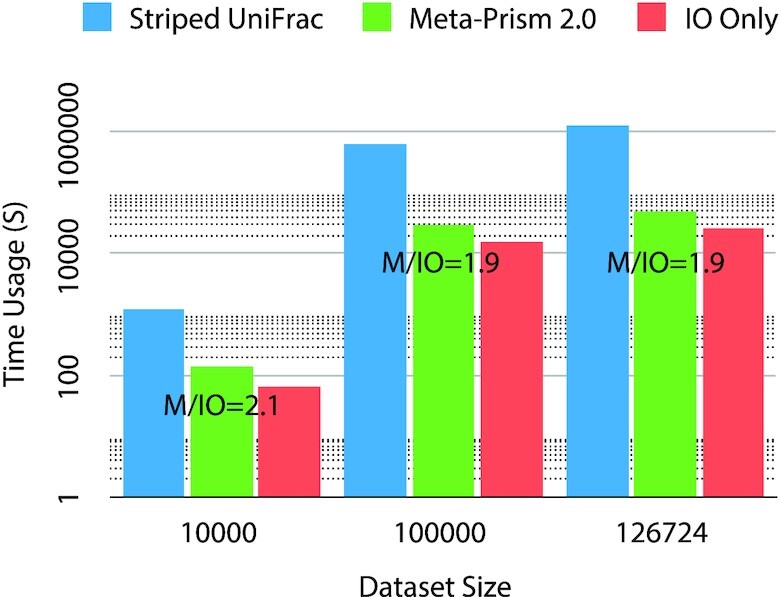
Time usage for different methods and IO Only on data sets with different sizes. “M/IO” is the ratio of time cost of Meta-Prism 2.0 over that of IO Only.

**Figure 6:**
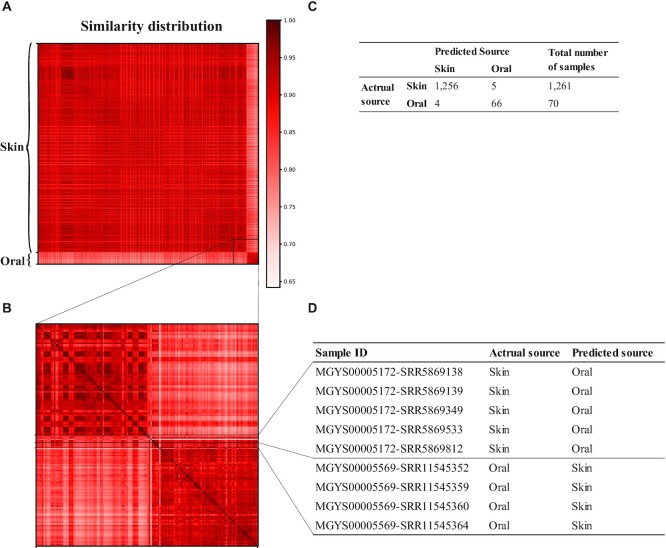
Clustering result of human samples from close biomes using similarities calculated by Meta-Prism 2.0. (A) Similarity distribution of 1,331 samples. The samples were successfully clustered into two groups, though we did not specify the number of clusters prior. (B) Similarity distribution of 9 samples that are not clustered with samples from the same biome (misclustered). (C) Confusion matrix and the number of samples within each actual source biome and predicted biome. (D) EBI MGnify study accession, run accession, actual biome source, and predicted biome source of 9 misclustered samples.

**Figure 7:**
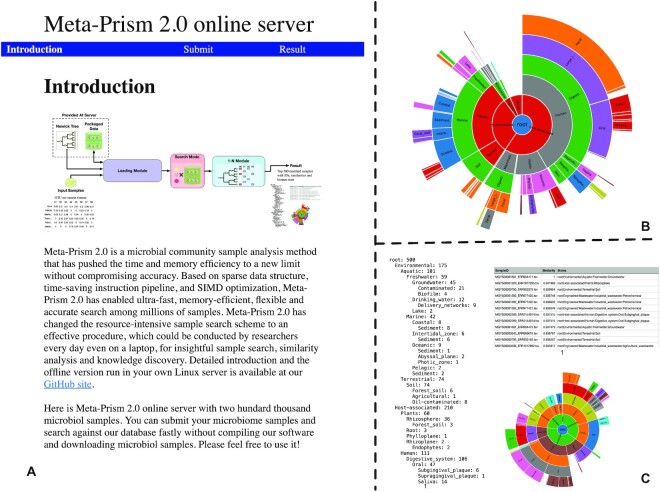
Screenshots for Meta-Prism 2.0 web server. (A) Meta-Prism 2.0 front page. (B) Statistics about sample source biomes for the built-in 200,000 microbial samples. (C) An example output consisting of  top 500 matched samples' IDs and similarity values in table format and statistics about sample source biomes of top 500 matched samples in text tree and sunburst format.

Meta-Prism 2.0 has unlocked several key computational techniques for efficient comparison (Fig. 1): first, it utilizes a sparse data structure to cut down the memory and disk usage (Fig. 1B). Second, to further cut down the memory usage, Meta-Prism 2.0 only stores essential taxa (taxa appeared in query samples) of the phylogenetic tree and abundances for similarity calculation (Fig. 1C (1–4)). Third, to cut down the time usage, Meta-Prism 2.0 discards redundant execution before diving into a similarity calculation (Fig. 1C (3)). Fourth, Meta-Prism 2.0 utilizes a fast 1-N compare module to enable further accelerations through the instruction pipeline [30] and single-instruction multiple-data (SIMD) optimization (Fig. 1C (5)). Last but not least, Meta-Prism 2.0 utilizes a customized 16-bit floating point to store the similarity matrix in a memory-saving manner (Fig. 1D).

### Space-saving data format

Each microbial community sample consists of classified taxa and their relative abundances. Meta-Prism 2.0 will find the taxa in the phylogenetic tree. For the representation of a single microbial community sample, most of the phylogenetic tree nodes are redundant. Unlike other methods that store in fixed-length arrays, Meta-Prism 2.0 stores taxonomic abundance data in a sparse format, that is, uses a variable-length list to store only relatively abundant nonzero nodes: the data include their relative abundance and the node ID of phylogenetic tree (Fig. 1B). When calculating similarities, Meta-Prism 2.0 converts sparse data back to dense data (Convert step, Fig. 1C (5), Algorithm 5 in Supplementary Material 1). The sparse data structure is applied to disk storage and memory cache to reduce space utilization globally.

The storage scheme is further optimized at the step of similarity result storage. To store similarity results for a sample pair, we designed a 16-bit floating point with 4 exponential bits and 12 mantissa bits. Considering that the similarities are between 0 and 1, we removed 2 sign bits of exponent and mantissa to increase the gamut and precision of the floating point (Fig. 1D).

### Similarity measurement independent of data type and sequencing depth

Our similarity is proposed to measure similarity between a pair of community samples, independent of data type and sequencing depth [14]. The details of similarity calculation are shown in Algorithm 6 in Supplementary Material 1 with the default execution order being generated from Algorithm 2 in Supplementary Material 1 with all nodes marked. To calculate the similarity of the 2 samples (*n* = 1 in pseudocode), we will recursively calculate the similarity of the relative abundance of the 2 samples at each node and deduce and then multiply the relative abundance remaining by 1 minus the evolutionary distance and send it to the parent node.

### Fast 1-N sample comparison

We further optimized the time usage through a fixed execution order and SIMD [31]. Current methods traverse the phylogenetic tree (with redundant nodes) and execute the operation during similarity calculation (Striped Unifrac calculates the difference at each node and divides it by the branch distance, Meta-Storm and Dynamic Meta-Storm accumulate the similarity at each node and pass the residual abundance to the parent node, and Meta-Prism 1.0 calculates the difference at each node, divides it by 1 minus the evolutionary distance, and passes it to the parent node), wasting time on redundant operations [15–17]. Meta-Prism 1.0 will wast time calculating at nodes with zero abundance (which also are the vast majority), without any influence on the result. Some nodes have only the relative abundance of 1 sample, and the most of the system's calculations on them are invalid, which are equivalent to multiplying their abundances by 1 minus the evolutionary distance and passing these to their parent nodes. To save the time wasted on such operations, when Meta-Prism 2.0 calculates a 1-N comparison (${S}_0$ against ${S}_n$), it will only consider nodes that are abundant in ${S}_0$ (marked node) and generate a fixed execution order based on them (GenOrder step, Fig. 1C (2 and 3), Algorithm 2 in Supplementary Material 1). To deal with nodes that are only abundant in ${S}_n$, Meta-Prism 2.0 will multiply the abundance on these nodes by the cumulative evolutionary distance factor and send them to the nearest labeled parent nodes (Convert step, Fig. 1C (4), Algorithm 5 in Supplementary Material 1). Which node to send and the factor value are calculated in Algorithm 4 in Supplementary Materials 1. The fixed execution order without branches and jumps will lead the CPU to use the instruction pipeline. Additionally, Meta-Prism 2.0 is implemented based on SIMD AVX intrinsic and thus can execute operations to compare a sample S0 with other multiple samples (referred to as Sn) at the same time (SimilarityCalculation step, Fig. 1C (5), Algorithm 6 in Supplementary Material 1). We packaged these steps as the “1-N module” and used the module to execute fast comparison and search.

## Discussions and Conclusion

In this work, we designed Meta-Prism 2.0 as an ultrafast and memory-efficient approach to analyze millions of microbial community samples. The sample compare and search problems have encountered great difficulties when faced with millions of samples, primarily due to the computational space and time limitations. Meta-Prism 2.0 was designed based on a sparse data structure, time-saving instruction pipeline, SIMD optimization, and exhaustive search strategy, enabling flexible, ultra-fast, memory-efficient, and added beta diversity analysis function.

Results show that compared to the current methods serving the same purpose, Meta-Prism 2.0 is at least 20 times faster, while memory cost is at least 4 times smaller. Additionally, the speed of Meta-Prism 2.0 is close to the lower bound of the search. Furthermore, according to our experiment, Meta-Prism 2.0 can even store all samples’ community structure from the EBI MGnify data set (300,000 in total as of October 2020) on a laptop and search against it at an unprecedented speed. Finally, we provided several concrete examples, which have proven the effectiveness and utility of Meta-Prism 2.0 in knowledge discovery. Also, the fast and accurate microbial community sample search could also be experienced on the web server, on which any query against this huge data set can be completed within 1 second, with high accuracy.

In summary, Meta-Prism 2.0 can perform searches among millions of samples with low memory cost and fast speed, enabling source tracking and knowledge discovery from sample mining at a massive scale. Meta-Prism 2.0 has optimized the traditional resource-intensive sample search and similarity matrix calculation into an affordable and effective procedure that researchers can conduct every day for mining intricate relationships among samples and discover previously unknown knowledge.

## Availability of Supporting Source Code and Requirements

Project name: Meta-Prism 2.0

Project home page: https://hust-ningkang-lab.github.io/Meta-Prism-2.0/

GitHub repository: https://github.com/HUST-NingKang-Lab/Meta-Prism-2.0

Operating systems: Platform independent

Programming language: C++

Other requirements: Compiler support C++11

License: GPL-3.0 License

RRID: SCR_021836

bio.tools ID: Meta-Prism 2.0

## Data Availability

For enhanced reproducibility, a CodeOcean computational capsule is available [32].

An archival copy of the code and other supporting data are also available via the *GigaScience* database GigaDB [33].

## Additional Files

**Supplementary Material 1**. Pseudocode about Meta-Prism 2.0.

**Supplementary Table S1**. Detailed information of the Combined data set and FEAST data set.

giac073_GIGA-D-21-00388_Original_Submission

giac073_GIGA-D-21-00388_Revision_1

giac073_GIGA-D-21-00388_Revision_2

giac073_Response_to_Reviewer_Comments_Original_Submission

giac073_Response_to_Reviewer_Comments_Revision_1

giac073_Reviewer_1_Report_Original_Submission Siyuan Ma -- 12/29/2021 Reviewed

giac073_Reviewer_1_Report_Revision_1 Siyuan Ma -- 5/15/2022 Reviewed

giac073_Supplemental_Files

## Abbreviations

AUC: area under the curve; AVX: Advanced Vector Extensions; EBI: European Bioinformatics Institute; FEAST: Fast Expectation-mAximization microbial Source Tracking; FP: Floating-Point; IO: input/output; JSD: Jensen–Shannon divergence; LTP: Living Tree Project; SIMD: Single Instruction Multiple Data; SSU: small subunit.

## Competing Interests

The authors declare that they have no competing interests.

## Funding

This work was partially supported by National Natural Science Foundation of China grants 32071465, 31871334, and 31671374; Ministry of Science and Technology's grant 2018YFC0910502; and National Undergraduate Training Program for Innovation and Entrepreneurship of China (Program No. 201910487071).

## Authors’ Contributions

K.N. conceived and supervised this study. K.K. designed and developed Meta-Prism 2.0 software and the web server. K.K and H.C. tested Meta-Prism 2.0. K.K, H.C., and K.N. wrote the manuscript. All authors read and approved the final manuscript.
