# Supplementary material for: Meta-Prism 2.0: Enabling algorithm and web server for ultra-fast, memory-efficient, and accurate analysis among millions of microbial community samples
Source: Gigascience. 2022 Jul 28;11:giac073. doi: 10.1093/gigascience/giac073 (PMC9334027; doi:10.1093/gigascience/giac073)
Supplement: giac073_GIGA-D-21-00388_Original_Submission [file giac073_giga-d-21-00388_original_submission.pdf]

GigaScience

# Meta-Prism 2.0: Enabling algorithm and web server for ultra-fast, memory-efficient, and accurate analysis among millions of microbial community samples

--Manuscript Draft--

| | |
|---|---|
| **Manuscript Number:** | GIGA-D-21-00388 |
| **Full Title:** | Meta-Prism 2.0: Enabling algorithm and web server for ultra-fast, memory-efficient, and accurate analysis among millions of microbial community samples |
| **Article Type:** | Research |
| **Funding Information:** | National Natural Science Foundation of China (32071465) — Mr Kang Ning<br>National Natural Science Foundation of China (31871334) — Mr Kang Ning<br>National Natural Science Foundation of China (31671374) — Mr Kang Ning<br>Ministry of Science and Technology of the People's Republic of China (2018YFC0910502) — Mr Kang Ning<br>National Undergraduate Training Program for Innovation and Entrepreneurship of China (201910487071) — Mr Kai Kang |

**Abstract:**

Background

Microbial community samples have been accumulated at a speed faster than ever, with hundreds of thousands of samples been sequenced each year. Mining such a huge amount of multi-source heterogeneous data is becoming an increasingly difficult challenge, so efficient and accurate compare and search of samples are in urgent need: Faced with millions of samples in the data repository, traditional sample comparison and search approaches fall short in speed and accuracy.

Findings

Here we proposed Meta-Prism 2.0, a microbial community sample analysis method that has pushed the time and memory efficiency to a new limit without compromising accuracy. Based on sparse data structure, time-saving instruction pipeline, and SIMD optimization, Meta-Prism 2.0 has enabled ultra-fast, memory-efficient, flexible and accurate search among millions of samples.

Conclusions

Meta-Prism 2.0 was put to test on several datasets, with the largest containing one million samples. Results show that Meta-Prism 2.0's 0.00001s per sample pair compare speed and 8GB memory needs for searching against one million samples have made it one of the most efficient sample analysis methods. Additionally, Meta-Prism 2.0 can achieve accuracy comparable with or better than other contemporary methods. Thirdly, Meta-Prism 2.0 can precisely identify the original biome for samples, thus enabling sample source tracking. Finally, we have provided a web server for fast search of microbial community samples online. In summary, Meta-Prism 2.0 has changed the resource-intensive sample search scheme to an effective procedure, which could be conducted by researchers every day even on a laptop, for insightful sample search, similarity analysis and knowledge discovery. Meta-Prism 2.0 can be accessed at: https://github.com/HUST-NingKang-Lab/Meta-Prism-2.0, and the web server can be accessed at: http://meta-prism2.aimicrobiome.cn/.

**Corresponding Author:** Kai Kang
Huazhong University of Science and Technology
Beijing, CHINA

Powered by Editorial Manager® and ProduXion Manager® from Aries Systems Corporation

| | |
|---|---|
| Corresponding Author Secondary Information: | |
| Corresponding Author's Institution: | Huazhong University of Science and Technology |
| Corresponding Author's Secondary Institution: | |
| First Author: | Kai Kang |
| First Author Secondary Information: | |
| Order of Authors: | Kai Kang |
| | Hui Chong |
| | Kang Ning |
| Order of Authors Secondary Information: | |
| Additional Information: | |
| **Question** | **Response** |
| Are you submitting this manuscript to a special series or article collection? | No |
| **Experimental design and statistics**<br><br>Full details of the experimental design and statistical methods used should be given in the Methods section, as detailed in our Minimum Standards Reporting Checklist. Information essential to interpreting the data presented should be made available in the figure legends.<br><br>Have you included all the information requested in your manuscript? | Yes |
| **Resources**<br><br>A description of all resources used, including antibodies, cell lines, animals and software tools, with enough information to allow them to be uniquely identified, should be included in the Methods section. Authors are strongly encouraged to cite Research Resource Identifiers (RRIDs) for antibodies, model organisms and tools, where possible.<br><br>Have you included the information requested as detailed in our Minimum Standards Reporting Checklist? | Yes |

| | |
|---|---|
| **Availability of data and materials**<br><br>All datasets and code on which the conclusions of the paper rely must be either included in your submission or deposited in publicly available repositories (where available and ethically appropriate), referencing such data using a unique identifier in the references and in the "Availability of Data and Materials" section of your manuscript.<br><br>Have you have met the above requirement as detailed in our Minimum Standards Reporting Checklist? | Yes |

Manuscript Click here to access/download;Manuscript;manuscript.docx

# Meta-Prism 2.0: Enabling algorithm and web server for ultra-fast, memory-efficient, and accurate analysis among millions of microbial community samples

Kai Kang[1,$], Hui Chong[1,$], Kang Ning[1,*]

*[1] Key Laboratory of Molecular Biophysics of the Ministry of Education, Hubei Key Laboratory of Bioinformatics and Molecular-imaging, Center of AI Biology, Department of Bioinformatics and Systems Biology, College of Life Science and Technology, Huazhong University of Science and Technology, Wuhan 430074, China*

*[$] These authors contributed equally to this work*
** Corresponding author*
*E-mail: ningkang@hust.edu.cn*

## Abstract

**Background**

Microbial community samples have been accumulated at a speed faster than ever, with hundreds of thousands of samples been sequenced each year. Mining such a huge amount of multi-source heterogeneous data is becoming an increasingly difficult challenge, so efficient and accurate compare and search of samples are in urgent need: Faced with millions of samples in the data repository, traditional sample comparison and search approaches fall short in speed and accuracy.

**Findings**

Here we proposed Meta-Prism 2.0, a microbial community sample analysis method that has pushed the time and memory efficiency to a new limit without compromising accuracy. Based on sparse data structure, time-saving instruction pipeline, and SIMD optimization, Meta-Prism 2.0 has enabled ultra-fast, memory-efficient, flexible and accurate search among millions of samples.

**Conclusions**

Meta-Prism 2.0 was put to test on several datasets, with the largest containing one million samples. Results show that Meta-Prism 2.0's 0.00001s per sample pair compare speed and 8GB memory needs for searching against one million samples have made it one of the most efficient sample analysis methods. Additionally, Meta-Prism 2.0 can achieve accuracy comparable with or better than other contemporary methods. Thirdly, Meta-Prism 2.0 can precisely identify the original biome for samples, thus enabling sample source tracking. Finally, we have provided a web server for fast search of

microbial community samples online. In summary, Meta-Prism 2.0 has changed the resource-intensive sample search scheme to an effective procedure, which could be conducted by researchers every day even on a laptop, for insightful sample search, similarity analysis and knowledge discovery. Meta-Prism 2.0 can be accessed at: https://github.com/HUST-NingKang-Lab/Meta-Prism-2.0, and the web server can be accessed at: http://meta-prism2.aimicrobiome.cn/.

# Introduction

Microbial communities have asserted great influences on healthcare, environment, and industry[1-4]. As such, an increasing number of projects have been conducted on microbial communities around the world, such as those from the "Human Microbiome Project"[1, 2] and the "Earth Microbiome Project"[3, 4]. Mining this massive amount of samples has already discovered knowledge about the microbial community and their effects on the environment and human health[5, 6], providing an opportunity to study the hidden evolution and ecology patterns among microbial communities.

A microbial community sample (also referred to as the sample) is represented by the hierarchically structured taxa (species, genus, families, etc.) and their relative abundances (also referred to as the community structure), and these species are functioning in concert to maintain stability and adapt to the specific environments (also referred to as the niches or biomes) where the microbial community is living. These samples' community structures are often associated with the biomes and a variety of characteristics of the biomes. For example, the community structures of the human gut microbiome have been linked to multiple aspects of human life, such as health[6, 7], early development[8], immigration[9], and pregnancy[10]. Thus, there is a large amount of hidden information in the community structures and remains to be discovered. These challenges in current microbiome researches are calling fast community-level comparison and search among the rapidly accumulating number of microbial communities.

There are already methods that existed for comparison and search of samples. The distance-based methods are the first batches designed for the purpose, whose primary strategy is to compare the similarity or distance between two samples. The simplest distance-based method is the Jensen-Shannon Divergence (JSD) measurement[11], which only considered species abundances in the community. More advanced distance-based methods considered both species abundances and their phylogenetic relationships. For example, UniFrac[12] is a typical distance-based method, which firstly maps their respective sets of taxon abundances on the phylogenetic tree, and secondly traverses the tree and executes operation at each node (each representing a taxon on the phylogenetic tree) to calculate their similarity. Fast UniFrac[13] and Meta-Storms[14]

optimized such a procedure by changing tree traversal to array loop. Striped UniFrac[15] further optimized matrix similarity comparison by reorganizing samples. Previously, we designed Meta-Prism 1.0, a fast and accurate microbial community sample search tool[16]. Meta-Prism 1.0 generates an index to rapidly select samples with similar biome and top phylum for comparison. Furthermore, Meta-Prism 1.0 uses GPU to accelerate comparison. However, given that more than a million community samples have already been deposited into public databases[17, 18], state-of-the-art methods including Meta-Prism 1.0 face difficulties in comparison and searching among these samples, while rendering knowledge discovery from samples formidable. Additionally, microbial community samples' data are very sparse. These methods use fixed-length arrays to save abundances with lengths equals to entities number of the phylogenetic tree, wasting a considerable amount of memory. They also spend much time operating on these empty nodes.

To solve the large-scale microbial community sample search problem, we have redesigned and updated Meta-Prism to its second version (Meta-Prism 2.0). Key improvements of Meta-Prism 2.0 include: (1) It removes redundant nodes to save memory and adopts a fast 1-against-N sample comparison strategy and thus is far faster than Meta-Prism 1.0. (2) It adds similarity matrix calculation function to analysis samples' beta diversity with high efficiency. (3) It exhaustively searches among all available samples and thus has higher flexibility (when searching among customized datasets) and robustness than Meta-Prism 1.0. More importantly, with these improvements of efficiency and space, Meta-Prism 2.0 now can deal with one million or even more microbial community samples and is one of the fastest microbial community sample search methods to date.

Using several datasets including the largest one containing a million samples, we demonstrated that it can achieve at least 20 times speed-up compared to the contemporary approach (e.g., Meta-Prism 1.0 and Striped UniFrac), and Meta-Prism 2.0 is the only method that could handle the search against a million samples. The memory utilization is also very efficient: Compared with other methods including JSD, Striped UniFrac, and Dynamic Meta Storms, when analyzing dataset beta diversity which size exceeds 10,000, Meta-Prism 2.0 can at least save 80% of memory space needed. Though we have saved time and memory by magnitudes, the accuracy is not compromised. For example, Meta-Prism 2.0 obtained 0.99 AUC in distinguishing samples from different biomes [19] while Striped UniFrac obtained 0.88 AUC on the same dataset. Meta-Prism 2.0 has changed the traditional computational resource-intensive sample search to a cheap and effective procedure that could be conducted by researchers every day, for the discovery of intricate relationships among samples. Meta-Prism 2.0 can be accessed at: https://github.com/HUST-NingKang-Lab/Meta-Prism-2.0. And the fast and accurate microbial community sample search could also be experienced on the web server at: http://meta-prism2.aimicrobiome.cn/.

# Methods

Meta-Prism 2.0 calculates similarities between microbial communities using two calculation modes: search mode and matrix mode. The search mode takes two datasets (query and target) as input and then outputs each query sample's top N similar matches in the target dataset. The matrix mode takes a dataset as input and outputs a pair-wise similarity matrix for all samples in the dataset (**Figure 1A**). These datasets can be produced by commonly used tools such as QIIME[20], MAPseq[21], and MetaPhlAn[22]. Each microbial community sample consists of classified taxa and their relative abundances. Meta-Prism 2.0 automatically maps the relative abundances to a phylogenetic tree (with nodes representing taxa of the community and phylogenetic distances between these taxa) and only stores the taxon name and relative abundances to reduce the memory and disk usage of the abundance data while keeping essential information.

Meta-Prism 2.0 has unlocked several key computational techniques for efficient comparison (**Figure 1**): Firstly, it utilizes a sparse data structure to cut down the memory and disk usage (**Figure 1B**). Secondly, to further cut down the memory usage, Meta-Prism 2.0 only stores essential taxa (taxa appeared in query samples) of the phylogenetic tree and abundances for similarity calculation. (**Figure 1C (1-4)**). Thirdly, to cut down the time usage, Meta-Prism 2.0 discards redundant execution before diving into similarity calculation (**Figure 1C (3)**). Fourthly, Meta-Prism 2.0 utilizes a fast 1-N compare module to enable further accelerations through the instruction pipeline[23] and single instruction multiple data (SIMD) optimization (**Figure 1C (5)**). Last but not least, Meta-Prism 2.0 utilizes a customized 16-bit floating-point to store the similarity matrix in a memory-saving manner (**Figure 1D**).

**Similarity independent of data type and sequencing depth**

Our similarity is proposed to measure similarity between a pair of community samples[14], independent of data type and sequencing depth. Precisely, our difference-based similarity[24] measures such similarity as follows:

$$Sim(S_1, S_2, t) = 1 - d(S_1, S_2, root(t), t)$$

Where

$$d(S_1, S_2, n, t) = \begin{cases} \left(1 - p(t, n)\right) \left|\dfrac{a(S_1, n) - a(S_2, n)}{2}\right|, if\ n\ is\ a\ leaf \\ \displaystyle\sum_{c \in C(n)} \left(1 - p(t, c)\right) d(S_1, S_2, c, t), otherwise \end{cases}$$

Where $S_1$ and $S_2$ are two community samples to be compared, $t$ is phylogenetic tree utilized, $root(t)$ is the root node for the phylogenetic tree $t$, $d(S_1,S_2,n,t)$ is a temporary difference between $S_1$ and $S_2$ at a specific node $n$, $C(n)$ is a set of children nodes for node $n$, $p(t,n)$ is the phylogenetic distance from $n$ to its parent on the tree $t$, and $a(S_1,n)$ is the relative abundance for $S_1$ at the node $n$.

**Space-saving data format**

For the representation of a single microbial community sample, most of the phylogenetic tree nodes are redundant. Meta-Prism 2.0 stores taxonomic abundance data in a sparse format (**Figure 1B**). When calculating similarities, Meta-Prism 2.0 converts sparse data back to dense data (Convert step, **Figure 1C (5),** Algorithm 5 in **Supplementary Materials**). The sparse data structure is applied to disk storage and memory cache to reduce space utilization globally.

The storage scheme is further optimized at the step of similarity result storage. To store similarity results for a sample pair, we designed 16 bits floating-point with four exponential bits and 12 mantissa bits. Considering that the similarities are between zero and one, we removed two sign bits of exponent and mantissa to increase the gamut and precision of the floating-point (**Figure 1D**).

**Fast 1-N sample comparison**

We further optimized the time usage to the minimum extend through a fixed execution order and SIMD[25] (**Figure 1E**). Current methods traverse phylogenetic tree (with redundant nodes) and execute operation during similarity calculation, wasting time on redundant operations. To save the time wasted on such operations, Meta-Prism 2.0 removes redundant nodes before diving into similarity calculation and fixes the execution order for each 1-against-N sample comparison to save the time of the tree traversal during similarity calculation, by storing the nodes in post-order (GenOrder step, **Figure 1C (3), Figure 1E,** Algorithm 2 in **Supplementary Materials**). The fixed execution order without branches and jumps will lead the CPU to use the instruction pipeline. Additionally, Meta-Prism 2.0 is implemented based on SIMD AVX intrinsic[26], thus can execute operations to compare a sample S0 with other multiple samples (referred to as Sn) at the same time (**Figure 1C (5), Figure 1E**). We packaged these steps as the “1-N module”, and use the module to execute fast comparison and search.

# Results

## Materials and execution environments used for evaluation

Through manual curation from the EBI MGnify database[17], we obtained a dataset consists of 126,727 microbial community samples belonging to 114 different biomes, defined as the Combined dataset. We also obtained a dataset consists of 10,270 samples belonging to three biomes: Fecal, Human, and Mixed, which have been used in the FEAST study[27], defined as the FEAST dataset (**Table 1**). To evaluate Meta-Prism 2.0's speed and memory efficiency on the scale of one million samples, we synthesized a dataset with 1,000,010 samples based on the Combined dataset. All samples from these three datasets are accessible from https://github.com/HUST-NingKang-Lab/Meta-Prism-2.0. We used SILVA 132 LTPs132 SSU phylogenetic tree[28] in all experiments included in this study.

Striped UniFrac, Dynamic Meta-Storms, Meta-Prism 2.0 were compiled by GCC 4.8.5 and ran on CentOS 6.7 with Intel(R) Xeon(R) CPU E5-2678 v3 @ 2.50GHz and 252GB memory. The Jensen-Shannon divergence was calculated utilizing Python 3.7.3 and SciPy 1.4.1 and ran on the same CentOS device. The executable Meta-Prism 2.0 steps' time usage was compiled by clang-1100.0.33.16, and evaluated by Xcode11.5 Instruments Time Profiler, ran on macOS 10.15 with Intel(R) Core (TM) i7-9750H and 32GB memory. Meta-Prism GPU version was compiled by NVCC 10.1 and ran on RTX 2080Ti.

## Accuracy evaluation

We assessed the search accuracy of different methods in the context of source tracking, namely by checking the consistency of the predicted biomes and query samples' actual biomes. This evaluation is based on the realization that the microbial communities collected from the same biome always share similar patterns in their taxonomical structures and relative abundances[19, 27]. Specifically, we used simple cross-validation for the evaluation, based on searching 12.5% randomly chosen samples (considered as query dataset) against the rest samples (considered as target dataset). For each query sample, we selected the top 100 most similar target samples, and the contributions of different source biomes of these 100 samples were assessed by SoftMax normalization.

The evaluation performances are shown in **Figure 2**. On the FEAST dataset, each method predicted biome for testing samples according to the biomes included in the source dataset (Fecal, Human, and Mixed). Distance-based phylogenetic tree approaches (Meta-Prism 2.0, Striped UniFrac, and Dynamic Meta-Storms) showed similarly good performance, while Jensen-Shannon Divergence (JSD) obtained a lower AUC of 0.9512. On the Combined dataset, each method predicted biome for testing samples according to 114 biomes included in the source dataset (87.5% of the

Combined dataset). JSD and Dynamic Meta-Storms cannot finish the calculation within an acceptable time (10 days). We only compared Meta-Prism 2.0 and Striped UniFrac. Meta-Prism obtained a higher AUC result of 0.9934, while Striped UniFrac's AUC result was 0.9153.

**Computational speed assessment**

The time and memory efficiency are the most profound advantage of Meta-Prism 2.0. We first assessed Meta-Prism 2.0's speed based on using datasets with different dataset sizes and using different numbers of CPU threads (**Figure 3**). The setting was matrix mode, which takes one dataset as input, then calculates all sample pairs' similarities, and the output is a similarity matrix. The time cost is split into several parts according to computational steps. Our 1-N module adds GenOrder and Convert steps, which increase linearly and quadratically with the increase of dataset size, respectively. Therefore, the GenOrder step takes more time when the dataset is small, while the Convert step takes more time when the dataset is big.

We also evaluated Meta-Prism 2.0 performance on a dataset with one million samples (see **Materials** for details). Meta-Prism 2.0 can efficiently package one million samples into a 369 MB-sized file for storage and load them within 27 seconds. We transferred the whole workload to a laptop and searched 100 samples against this dataset with a single CPU thread. It cost 324.96 seconds (less than 6 minutes) CPU time to complete the search using only 6.9 GB memory. So far as we know, Meta-Prism 2.0 is the only method that could handle the search against a million samples.

**Computational speed comparison**

We further selected datasets with different dataset sizes (10, 100, 1,000, 10,000, 100,000 and 126,727) from the Combined dataset to compared different methods. The setting is again matrix mode. We compared time and memory usage of Striped UniFrac, Dynamic Meta Storms, JSD, Meta-Prism GPU, and Meta-Prism 2.0. Meta-Prism GPU is the only method that uses GPU for calculation, and we considered real-time usage for the measurement. In comparison, we took CPU core time usage as other methods' time usage. JSD and Meta-Storms cannot calculate the similarity matrix when dataset size $\geq$ 10,000 within an acceptable time (10 days).

Results show that Meta-Prism 2.0 could achieve superior performance on both time usage and memory usage (**Figure 4)**. Specifically, when the dataset sizes are no more than one thousand, Meta-Prism 2.0 used a similar core time compared with Striped UniFrac **(Figure 4A)**. When dataset size became more extensive, the performance gap between Meta-Prism 2.0 and Striped UniFrac became larger. When calculating the similarity matrix for the Combined dataset (generating 126,727 $\times$ 126,727 similarity matrix), Meta-Prism 2.0 was 55 times faster than Striped UniFrac. Meta-Prism GPU's

real-time usage was smaller than Meta Prism 2.0 core time usage. However, when Meta-Prism 2.0 uses 3 CPU cores or more, it will be faster than Meta-Prism GPU.

When the dataset size is smaller than 1,000, all methods utilized similar memory space. However, Meta-Prism 2.0's memory usage was only 11.1% of Striped UniFrac's when calculating the similarity matrix for the Combined dataset with more than 100,000 samples. The utilization of customized 16 bits floating point was the key reason behind such efficient memory use: as the memory occupied by the similarity matrix increases quadratically when the dataset size increases, it would lead to an amplified reduction of memory usage.

Since Meta-Prism 2.0 is ultrafast, it is natural to wonder how far is the speed of Meta-Prism 2.0 to the theoretical lower bound for the sample search. To answer this question, we took IO Only as the lower bound for sample search, in which we only record the time used for loading data and writing matrix calculation results (**Figure 5**). The result shows that Meta-Prism 2.0 is already close to the limit of optimization: on datasets of different sizes, the time costs of Meta-Prism 2.0 is only two times of IO Only, while magnitude smaller than those of Striped UniFrac.

**Real data applications**

Meta-Prism 2.0 can precisely identify the biome for samples of unknown origin, thus enabling the source tracking of samples. For example, it enables accurate differentiation of samples from close biomes such as "human skin" and "human oral" (the first application), identification of the biome for samples with unclear origin (the second application), as well as detection of microbial contamination (the third application).

Firstly, we tested Meta-Prism 2.0's ability to accurately differentiating samples from close biomes. We obtained 1,261 skin metagenomic samples (MGYS00005172)[29] and 70 oral metagenomic samples (MGYS00005569)[30] from MGnify[17]. We used Meta-Prism 2.0 to calculate the similarities matrix of 1,331 samples on a laptop, which cost only 3.75 seconds and 11MB of memory. We also clustered samples based on their similarities by using affinity propagation from Scikit-learn (version 0.20.3). The samples were successfully clustered into two groups whose sizes are 1,260 and 71 (**Figure 6**). Within 1,331 samples, only nine samples (five skin samples and four oral samples) were miss-clustered, proving Meta-Prism 2.0's ability to fast and accurately differentiate samples from close biomes.

Secondly, we evaluated the performance of Meta-Prism 2.0 on source tracking environmental samples from less-studied biomes, based on searching 11 groundwater samples curated from Saudi Arabian (MGYS00001601)[31] against the combined dataset. The biome "groundwater" is less studied, with a handful of samples in the combined dataset (MGYS00005245). Results show that Meta-Prism 2.0 could

successfully identify source-related biomes for samples from "groundwater". Within the top 100 most similar community samples for each "groundwater" query sample, there are on average 64 groundwater-related samples (from "root-Environmental-Terrestrial", "root-Environmental-Aquatic", "root-Engineered-Wastewater" and "root-Host-associated-Plants") for each query sample. Nevertheless, there is no "groundwater" sample in the top 100 similar samples searched by Meta-Prism 2.0, since "groundwater" samples in the combined dataset are curated from New Zealand, which is in nature drastically different from our query samples. The result suggests that the geographic origins also influence the community structures, which was already confirmed by previous studies[32].

Finally, we evaluated the Meta-Prism 2.0's power in detecting microbial contamination. We investigated the contamination of indoor house surfaces community by selecting 611 samples from indoor house surfaces in Chicago as query samples and searching against 6,285 samples (899+3,773+721+692 from "human skin", "environmental", "mammal", and "plants", respectively). The analysis costs only 6.16 seconds to complete. Our results show that the most closed biome source for indoor house surface samples is "human skin" (average similarity 0.889), indicating a large proportion of microbial community contamination from human skin, which agrees with previous analyses by SourceTracker[19] and FEAST[27]. Again, it proved the ability of Meta-Prism 2.0 for accurate and fast microbial community contamination screening.

**Web server for fast and accurate microbial community sample search**

For easy use of Meta-Prism 2.0, we also designed an online web server for Meta-Prism 2.0 (**Figure 7**), with a pre-compiled Meta-Prism 2.0 executable file and a built-in dataset contains more than 0.2 million microbiome samples. This dataset including major categories such as digestive system, aquatic, and soil, as well as sub-categories such as oil-contaminated clay, thermal springs sediment, and bioreactor for biological phosphorus removal. The high efficiency of Meta-Prism 2.0 enables any query against this huge dataset to be completed within one second, with high accuracy.

# Discussions and Conclusion

In this work, we designed Meta-Prism 2.0 as an ultrafast and memory-efficient approach to analysis against millions of microbial community samples. The sample compare and search problems have encountered great difficulties when faced with millions of samples, primarily due to the computational space and time limitations. Meta-Prism 2.0 was designed based on sparse data structure, time-saving instruction pipeline, SIMD optimization, and exhaustive search strategy, enabling flexible, ultra-fast, memory-efficient, and added beta diversity analysis function.

Results show that compared to the current methods serving the same purpose, Meta-Prism 2.0 is at least 20 times faster, while memory cost is at least four times smaller. Additionally, the speed of Meta-Prism 2.0 is close to the lower bound of the search. Furthermore, according to our experiment, Meta-Prism 2.0 can even store all samples' community structure from the EBI MGnify dataset (300,000 in total as of Oct. 2020) on a laptop and searching against it at an unprecedented speed. Finally, we provided several concrete examples, which have proven the effectiveness and utility of Meta-Prism 2.0 in knowledge discovery. And the fast and accurate microbial community sample search could also be experienced on the web server, on which any query against this huge dataset to be completed within one second, with high accuracy

In summary, Meta-Prism 2.0 can perform searches among millions of samples with low memory cost and fast speed, enabling source tracking and knowledge discovery from sample mining at a massive scale. Meta-Prism 2.0 has optimized the traditional resource-intensive sample search and similarity matrix calculation into an affordable and effective procedure that researchers could conduct every day for mining intricate relationships among samples and discover previously unknown knowledge.

## Availability of Supporting Source Code and Requirements

Project name: Meta-Prism 2.0
Project home page: http://meta-prism2.aimicrobiome.cn/
GitHub repository: https://github.com/HUST-NingKang-Lab/Meta-Prism-2.0
Operating systems: Platform independent
Programming language: C++
Other requirements: Compiler support C++11
License: GPL-3.0 License
RRID: SCR_021836
bio.tools ID: Meta-Prism 2.0

## Availability of Data

Our code is published at https://github.com/HUST-NingKang-Lab/Meta-Prism-2.0. All the datasets curated for this study are available at this project's "Releases".

## Competing of Interest

The authors declare that they have no competing interests.

## Acknowledgments

The authors would like to thank Yi Zhan for insightful discussions about the project.

## Funding

This work was partially supported by National Natural Science Foundation of China grant 32071465, 31871334, and 31671374, Ministry of Science and Technology's grant 2018YFC0910502, and National Undergraduate Training Program for Innovation and Entrepreneurship of China (Program No. 201910487071).

## Authors' Contributions

K.N. conceived and supervised this study. K.K. designed and developed Meta-Prism 2.0 software and web server. K.K and H.C. tested Meta-Prism 2.0. K.K, H.C., and K.N. wrote the manuscript. All authors read and approved the final manuscript.

## Additional Files

**Supplementary Material 1**: Pseudocode about Meta-Prism 2.0.
**Supplementary Table 1**: Detail information of Combined dataset and FEAST dataset.

## References

1. Integrative HMPRNC: **The Integrative Human Microbiome Project: dynamic analysis of microbiome-host omics profiles during periods of human health and disease**. *Cell Host Microbe* 2014, **16**(3):276-289.
2. Turnbaugh PJ, Ley RE, Hamady M, Fraser-Liggett CM, Knight R, Gordon JI: **The human microbiome project**. *Nature* 2007, **449**(7164):804-810.
3. Gilbert JA, Jansson JK, Knight R: **The Earth Microbiome project: successes and aspirations**. *BMC Biol* 2014, **12**:69.
4. Thompson LR, Sanders JG, McDonald D, Amir A, Ladau J, Locey KJ, Prill RJ, Tripathi

A, Gibbons SM, Ackermann G *et al*: **A communal catalogue reveals Earth's multiscale microbial diversity**. *Nature* 2017, **551**(7681):457-463.

5. Dominguez-Bello MG, De Jesus-Laboy KM, Shen N, Cox LM, Amir A, Gonzalez A, Bokulich NA, Song SJ, Hoashi M, Rivera-Vinas JI: **Partial restoration of the microbiota of cesarean-born infants via vaginal microbial transfer**. *Nature medicine* 2016, **22**(3):250.
6. Thomas S, Izard J, Walsh E, Batich K, Chongsathidkiet P, Clarke G, Sela DA, Muller AJ, Mullin JM, Albert K: **The host microbiome regulates and maintains human health: a primer and perspective for non-microbiologists**. *Cancer research* 2017, **77**(8):1783-1812.
7. Zeller G, Tap J, Voigt AY, Sunagawa S, Kultima JR, Costea PI, Amiot A, Bohm J, Brunetti F, Habermann N *et al*: **Potential of fecal microbiota for early-stage detection of colorectal cancer**. *Mol Syst Biol* 2014, **10**:766.
8. Bäckhed F, Roswall J, Peng Y, Feng Q, Jia H, Kovatcheva-Datchary P, Li Y, Xia Y, Xie H, Zhong H: **Dynamics and stabilization of the human gut microbiome during the first year of life**. *Cell host & microbe* 2015, **17**(5):690-703.
9. Vangay P, Johnson AJ, Ward TL, Al-Ghalith GA, Shields-Cutler RR, Hillmann BM, Lucas SK, Beura LK, Thompson EA, Till LM: **US immigration westernizes the human gut microbiome**. *Cell* 2018, **175**(4):962-972. e910.
10. Koren O, Goodrich JK, Cullender TC, Spor A, Laitinen K, Bäckhed HK, Gonzalez A, Werner JJ, Angenent LT, Knight R: **Host remodeling of the gut microbiome and**

**metabolic changes during pregnancy**. *Cell* 2012, **150**(3):470-480.

11. Lin J: **Divergence measures based on the Shannon entropy**. *IEEE Transactions on Information theory* 1991, **37**(1):145-151.

12. Lozupone C, Knight R: **UniFrac: a New Phylogenetic Method for Comparing Microbial Communities**. *Applied and Environmental Microbiology* 2005, **71**(12):8228-8235.

13. Hamady M, Lozupone C, Knight R: **Fast UniFrac: facilitating high-throughput phylogenetic analyses of microbial communities including analysis of pyrosequencing and PhyloChip data**. *The ISME journal* 2010, **4**(1):17-27.

14. Su X, Xu J, Ning K: **Meta-Storms: efficient search for similar microbial communities based on a novel indexing scheme and similarity score for metagenomic data**. *Bioinformatics* 2012, **28**(19):2493-2501.

15. McDonald D, Vázquez-Baeza Y, Koslicki D, McClelland J, Reeve N, Xu Z, Gonzalez A, Knight R: **Striped UniFrac: enabling microbiome analysis at unprecedented scale**. *Nature Methods* 2018, **15**(11):847-848.

16. Zhu M, Kang K, Ning K: **Meta-Prism: Ultra-fast and highly accurate microbial community structure search utilizing dual indexing and parallel computation**. *Briefings in Bioinformatics* 2020, **00**(December 2019):1-11.

17. Mitchell AL, Almeida A, Beracochea M, Boland M, Burgin J, Cochrane G, Crusoe MR, Kale V, Potter SC, Richardson LJ: **MGnify: the microbiome analysis resource in 2020**. *Nucleic acids research* 2020, **48**(D1):D570-D578.

18. Coordinators NR: **Database resources of the National Center for Biotechnology**

**Information**. *Nucleic Acids Res* 2016, **44**(D1):D7-19.

19. Knights D, Kuczynski J, Charlson ES, Zaneveld J, Mozer MC, Collman RG, Bushman FD, Knight R, Kelley ST: **Bayesian community-wide culture-independent microbial source tracking**. *Nature methods* 2011, **8**(9):761-763.

20. Bolyen E, Rideout JR, Dillon MR, Bokulich NA, Abnet CC, Al-Ghalith GA, Alexander H, Alm EJ, Arumugam M, Asnicar F *et al*: **Reproducible, interactive, scalable and extensible microbiome data science using QIIME 2**. *Nature Biotechnology* 2019, **37**(8):852-857.

21. Matias Rodrigues JF, Schmidt TSB, Tackmann J, von Mering C: **MAPseq: highly efficient k-mer search with confidence estimates, for rRNA sequence analysis**. *Bioinformatics* 2017, **33**(23):3808-3810.

22. Truong DT, Franzosa EA, Tickle TL, Scholz M, Weingart G, Pasolli E, Tett A, Huttenhower C, Segata N: **MetaPhlAn2 for enhanced metagenomic taxonomic profiling**. *Nature Methods* 2015, **12**(10):902-903.

23. Finlayson I, Davis B, Gavin P, Uh G-R, Whalley D, Själander M, Tyson G: **Improving processor efficiency by statically pipelining instructions**. *ACM SIGPLAN Notices* 2013, **48**(5):33-44.

24. Zhu M, Kang K, Ning K: **Meta-Prism: Ultra-fast and highly accurate microbial community structure search utilizing dual indexing and parallel computation**. *Briefings in Bioinformatics* 2020.

25. Amiri H, Shahbahrami A: **SIMD programming using Intel vector extensions**. *Journal of*

*Parallel and Distributed Computing* 2020, **135**:83-100.

26. **Introduction to Intel Advanced Vector Extensions** [https://software.intel.com/content/www/us/en/develop/articles/introduction-to-intel-advanced-vector-extensions.html]

27. Shenhav L, Thompson M, Joseph TA, Briscoe L, Furman O, Bogumil D, Mizrahi I, Pe'er I, Halperin E: **FEAST: fast expectation-maximization for microbial source tracking**. *Nature Methods* 2019, **16**(7):627.

28. Yilmaz P, Parfrey LW, Yarza P, Gerken J, Pruesse E, Quast C, Schweer T, Peplies J, Ludwig W, Glöckner FO: **The SILVA and "all-species living tree project (LTP)" taxonomic frameworks**. *Nucleic acids research* 2014, **42**(D1):D643-D648.

29. SanMiguel AJ, Meisel JS, Horwinski J, Zheng Q, Bradley CW, Grice EA: **Antiseptic Agents Elicit Short-Term, Personalized, and Body Site–Specific Shifts in Resident Skin Bacterial Communities**. *Journal of Investigative Dermatology* 2018, **138**(10):2234-2243.

30. Shaiber A, Willis AD, Delmont TO, Roux S, Chen L-X, Schmid AC, Yousef M, Watson AR, Lolans K, Esen OC: **Functional and genetic markers of niche partitioning among enigmatic members of the human oral microbiome**. *bioRxiv* 2020.

31. Alsalah D, Al-Jassim N, Timraz K, Hong P-Y: **Assessing the Groundwater Quality at a Saudi Arabian Agricultural Site and the Occurrence of Opportunistic Pathogens on Irrigated Food Produce**. *International Journal of Environmental Research and Public Health* 2015, **12**(10):12391-12411.

32. Chai X, Yang Y, Wang X, Hao P, Wang L, Wu T, Zhang X, Xu X, Han Z, Wang Y:

**Spatial variation of the soil bacterial community in major apple producing regions of China**. *Journal of Applied Microbiology* 2020.

# Figures

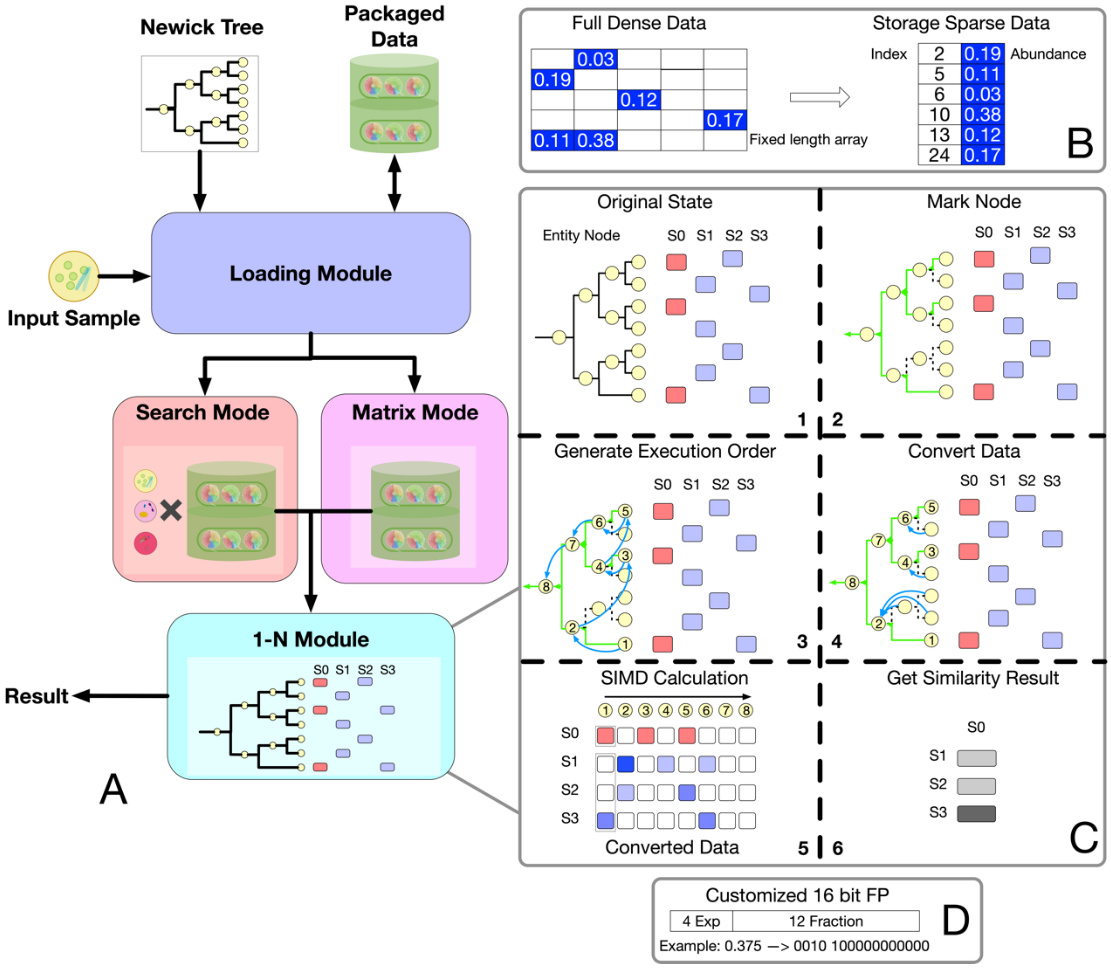


**Figure 1**

**Figure 1. The Meta-Prism 2.0 pipeline with key optimization highlighted.** (A) Meta-Prism 2.0 takes taxa abundance as input data, maps data to the phylogenetic tree, and converts data to sparse abundance data for space optimization. Meta-Prism 2.0 organizes data according to search mode or matrix mode, then uses the 1-N module to calculate similarities. Space-saving scheme packages sample data to the sparse format for storage, cuts down both disk and memory usage. (C) The 1-N module saves resources to the maximum extent by removing redundant nodes without losing their abundances, and fix the execution order for fast 1-against-N sample comparison (1-4), followed by SIMD optimization as a compiler-level optimization (5). The dashed lines indicate branches and nodes to be removed. The black arrows indicate an execution order to be recorded (post-order traversal), and the blue arrows indicate abundance aggregation from those to-be-removed nodes to their ancestors. (D) The similarities are saved in the format of a customized 16-bit floating-point. Pseudocode about Meta-Prism 2.0 can be accessed from **Supplementary Material 1**.

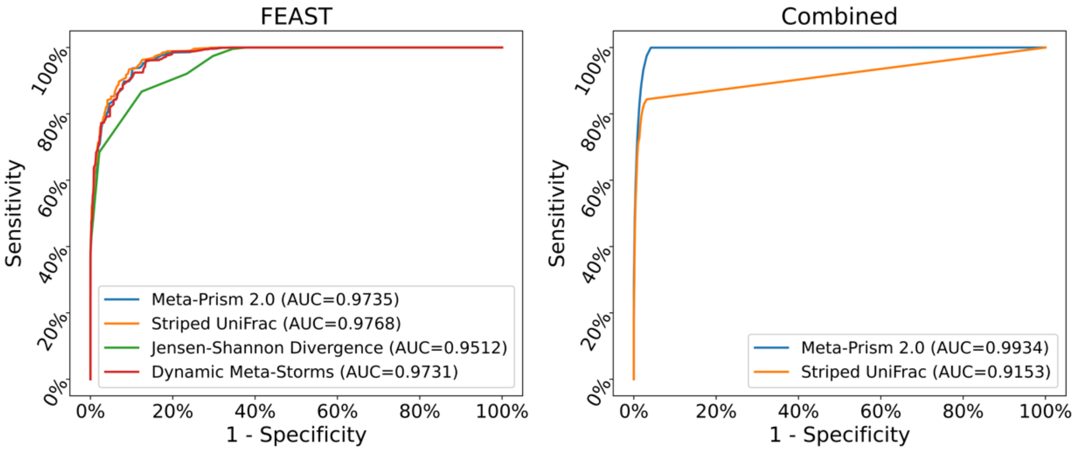


**Figure 2**

**Figure 2. AUC of different methods for sample searches using the FEAST dataset and the Combined dataset.** Note that all these methods could complete the analysis in due time and reached good AUC on the FEAST dataset, whereas Jensen-Shannon divergence and Dynamic Meta-Storms can not complete the analysis on the Combined dataset.

**Figure 3**

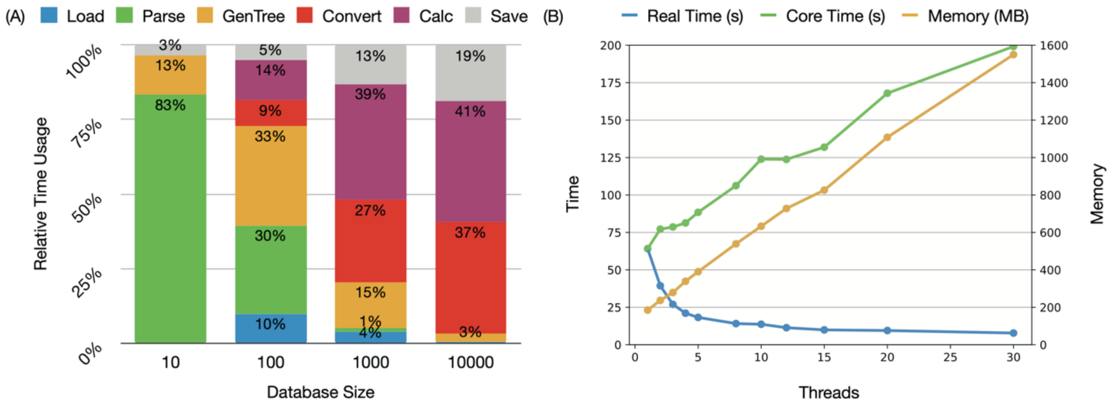


**Figure 3. Time usage at different steps and multi-threads performance analysis of Meta-Prism 2.0.** (A) Each steps' time usage with variate sample sizes. Load: load data, Save: save matrix result, Parse: load and parse phylogenetic tree, GenOrder generates non-redundant phylogenetic tree (without redundant nodes) in 1-N module, Convert: convert sample data from spare format to dense format for the sample comparison, Calc: 1-against-N sample comparison. A higher proportion of total time was used by Convert and Calc steps when the number of sample pairs increases. (B) Time and memory usage for 10,000 samples' pair-wise similarity calculation using the different numbers of CPU threads. Real-time: the actual time usage of calculation, Core Time: the sum of each CPU cores' time usage. Note that since each thread of Meta-Prism 2.0 removes redundancy in the phylogenetic tree and source samples separately, the total memory usage would increase as the number of threads increases.

**Figure 4**

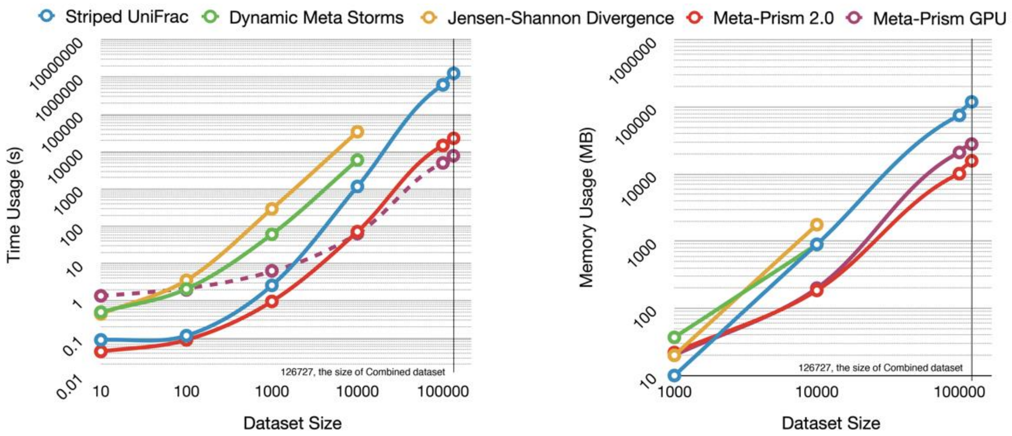


**Figure 4. Time and memory usage of samples when calculating similarity matrix for datasets with different numbers of samples.** (A) is for time usage comparison, and (B) is for memory usage comparison. In (A), Meta-Prism GPU time usage with dash line is GPU time usage, others are CPU core time usage.

**Figure 5**

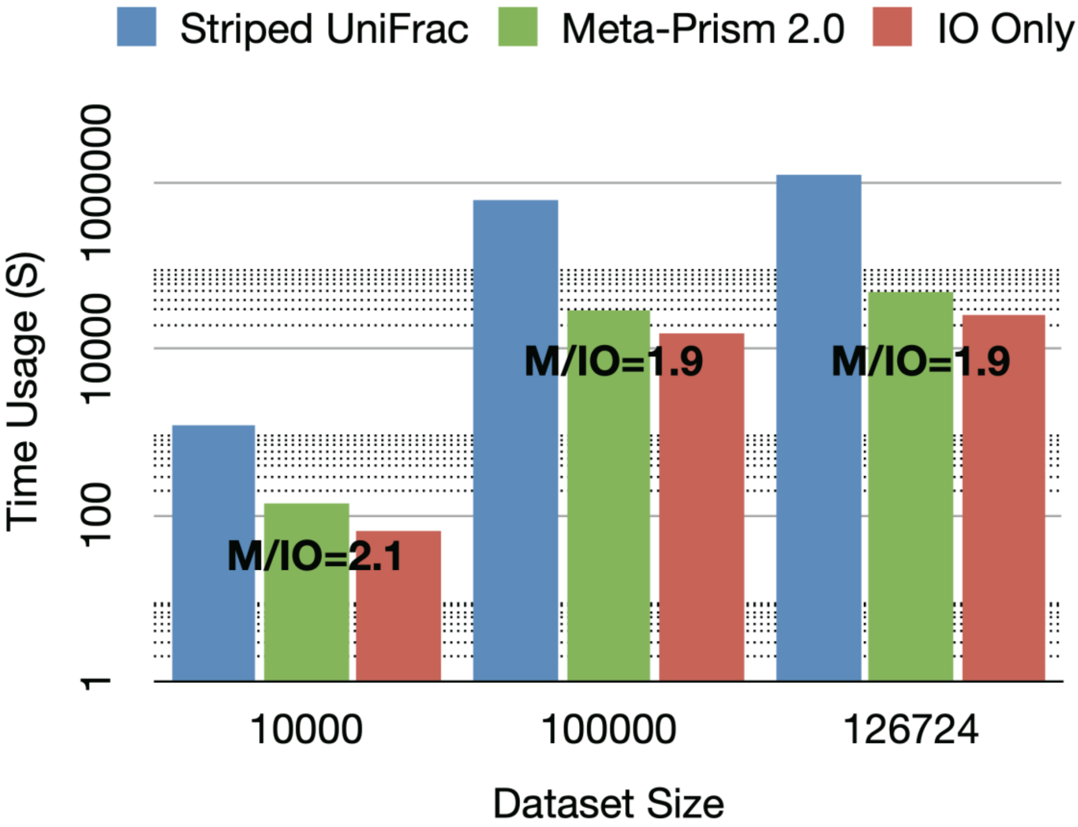


**Figure 5. Time usage for different methods and IO Only on datasets with different sizes.** "M/IO" is the ratio of time cost of Meta-Prism 2.0 over that of IO Only.

**Figure 6**

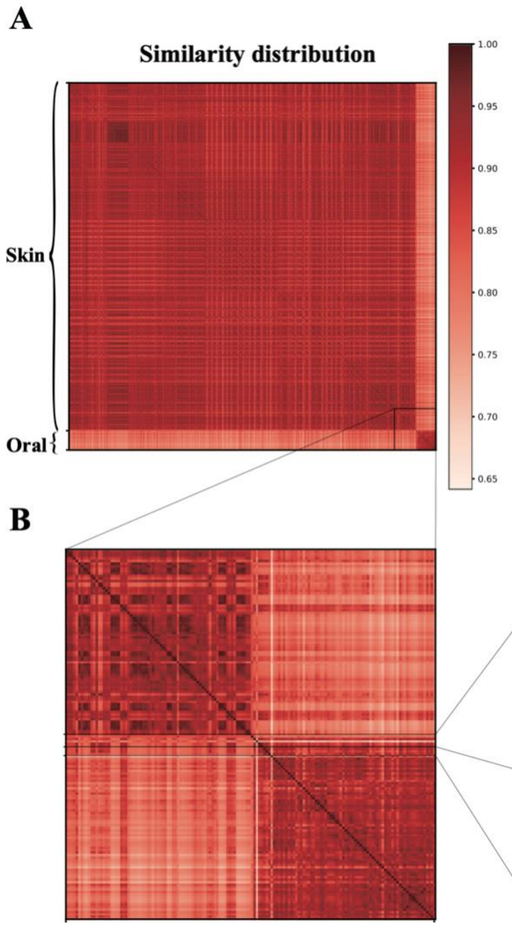


C

| | | Predicted Source | | Total number of samples |
|---|---|---|---|---|
| | | Skin | Oral | |
| Actrual source | Skin | 1,256 | 5 | 1,261 |
| | Oral | 4 | 66 | 70 |

D

| Sample ID | Actrual source | Predicted source |
|---|---|---|
| MGYS00005172-SRR5869138 | Skin | Oral |
| MGYS00005172-SRR5869139 | Skin | Oral |
| MGYS00005172-SRR5869349 | Skin | Oral |
| MGYS00005172-SRR5869533 | Skin | Oral |
| MGYS00005172-SRR5869812 | Skin | Oral |
| MGYS00005569-SRR11545352 | Oral | Skin |
| MGYS00005569-SRR11545359 | Oral | Skin |
| MGYS00005569-SRR11545360 | Oral | Skin |
| MGYS00005569-SRR11545364 | Oral | Skin |

**Figure 6. Clustering result of human samples from close biomes using similarities calculated by Meta-Prism 2.0.** (A) Similarity distribution of 1,331 samples. The samples were successfully clustered into two groups though we did not specify the number of clusters *prior*. (B) Similarity distribution of 9 samples that are not clustered with samples from the same biome (mis-clustered). (C) Confusion matrix and the number of samples within each actual source biome and predicted biome. (D) EBI MGnify study accession, run accession, actual biome source, and predicted biome source of 9 mis-clustered samples.

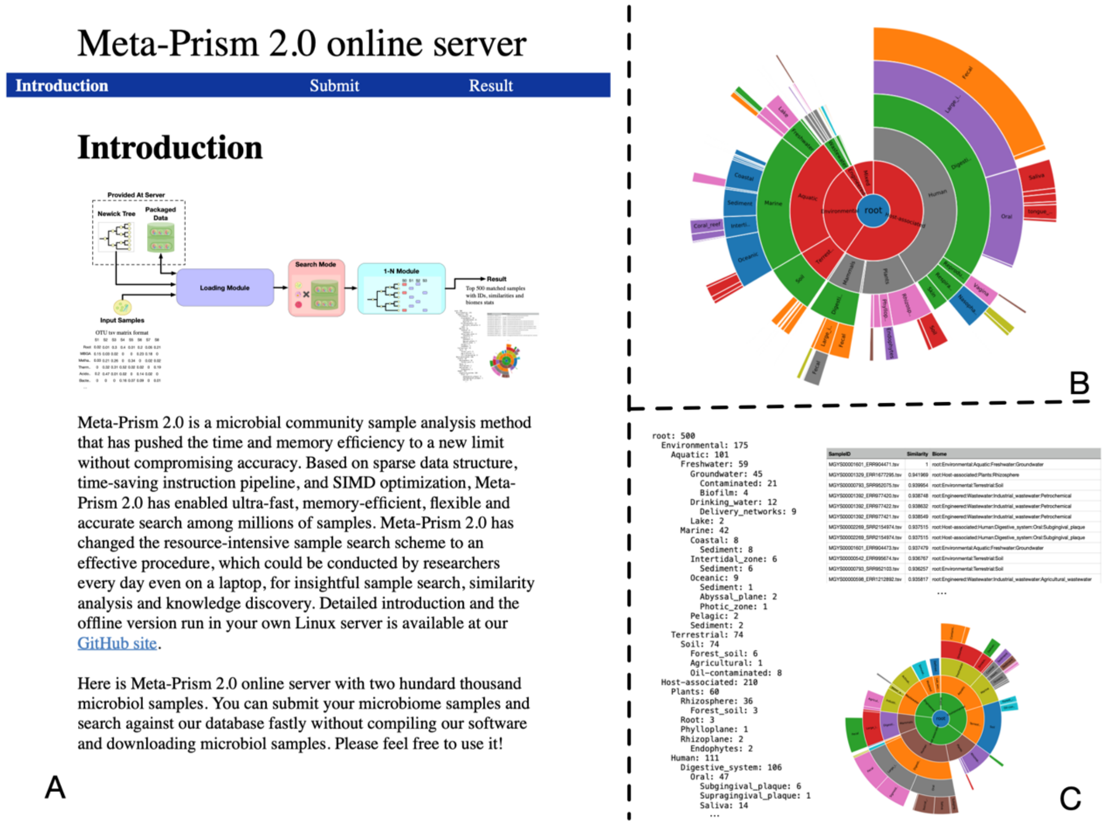


**Figure 7. Screenshots for Meta-Prism 2.0 web server.** (A) Meta-Prism 2.0 front page. (B) Statistics about sample source biomes for the built-in two hundred thousand microbial samples. (C) An example output consisting of top 500 matched samples' IDs and similarity values in table format, statistics about sample source biomes of top 500 matched samples in text tree and sunburst format.

# Tables

**Table 1**

**Table 1. The Combined dataset and FEAST dataset used in this study.** Details are provided in **Supplementary Table**.

| Dataset | Combined dataset | FEAST dataset |
|---|---|---|
| **Top-level biome** | Root | Human gut |
| **Number of biomes involved** | 114 | 3 |
| **Number of samples** | 126,727 | 10,270 |
| **Number of species** | 45,477 | 5,762 |
| **The average number of species per sample** | 411.22 | 111.05 |
| **Notes** | Selected samples from MGnify database | Selected samples from the FEAST study |

 Click here to access/download;Table;Table 1.xlsx

**Table 1. The Combined dataset and FEAST dataset used in this study**

| **Dataset** | **Combined dataset** | **FEAST dataset** |
|---|---|---|
| **Top-level biome** | Root | Human gut |
| **Number of biomes involved** | 114 | 3 |
| **Number of samples** | 126,727 | 10,270 |
| **Number of species** | 45,477 | 5,762 |
| **The average number of species per sample** | 411.22 | 111.05 |
| **Notes** | Selected samples from MGnify database | Selected samples from the FEAST study |

figure 1

Click here to access/download;Figure;Figure 1.eps

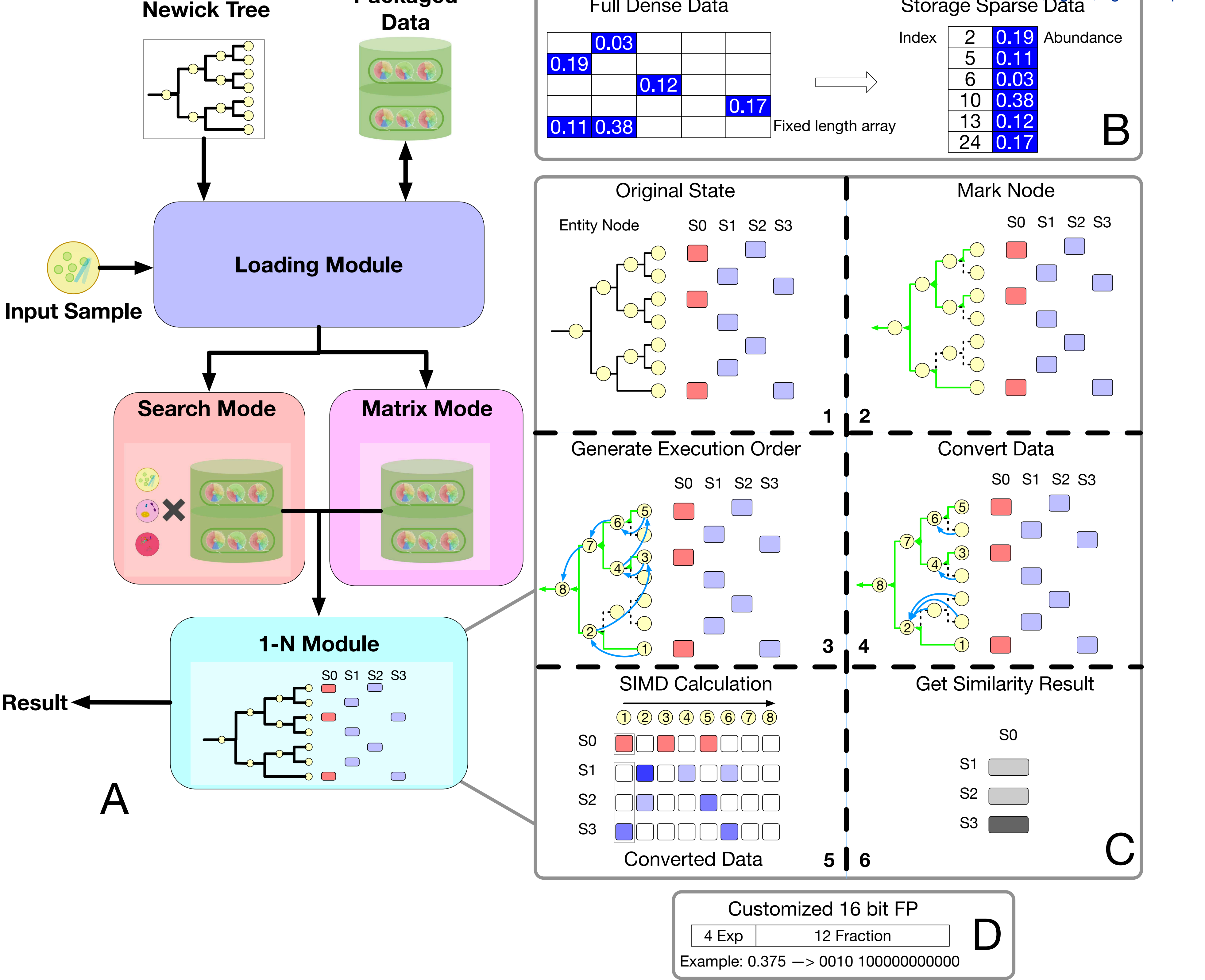

Click here to access/download;Figure;Figure 2.eps

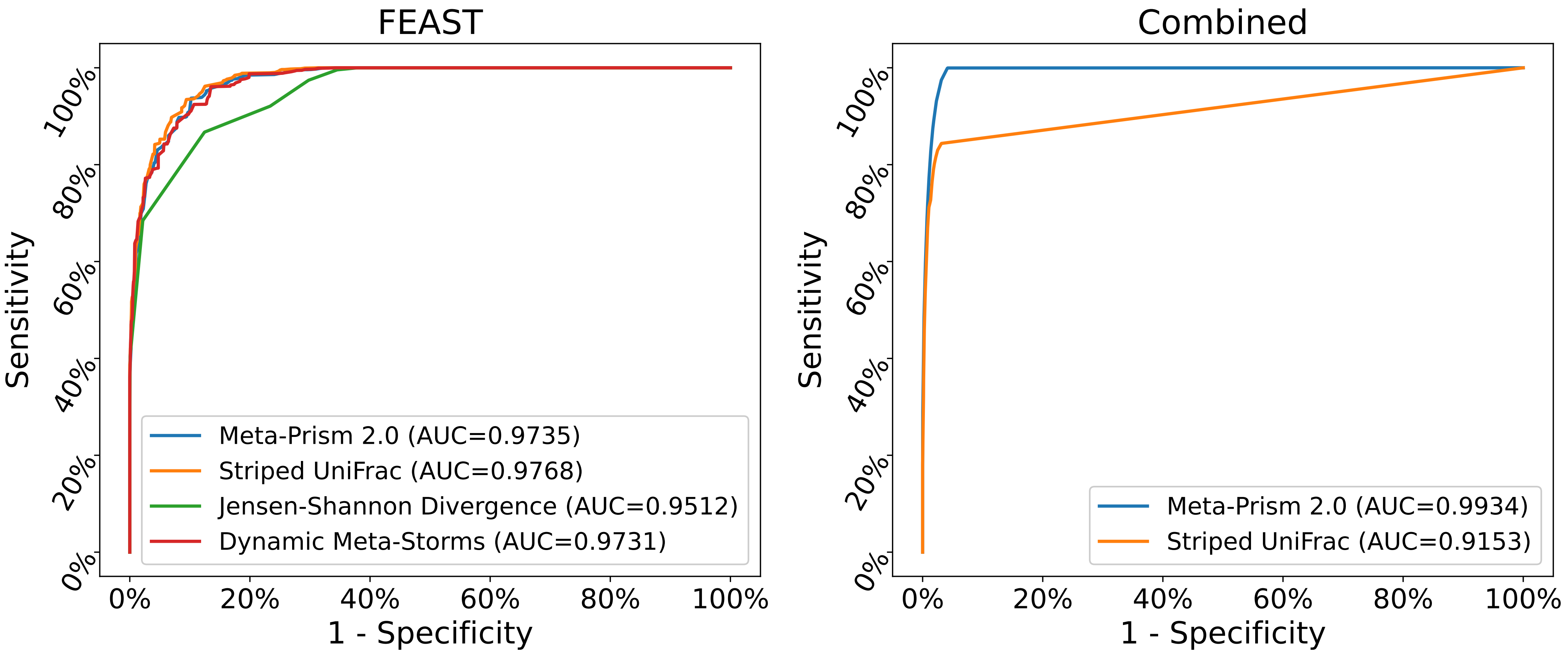

figure 3

Click here to access/download;Figure;Figure 3.eps

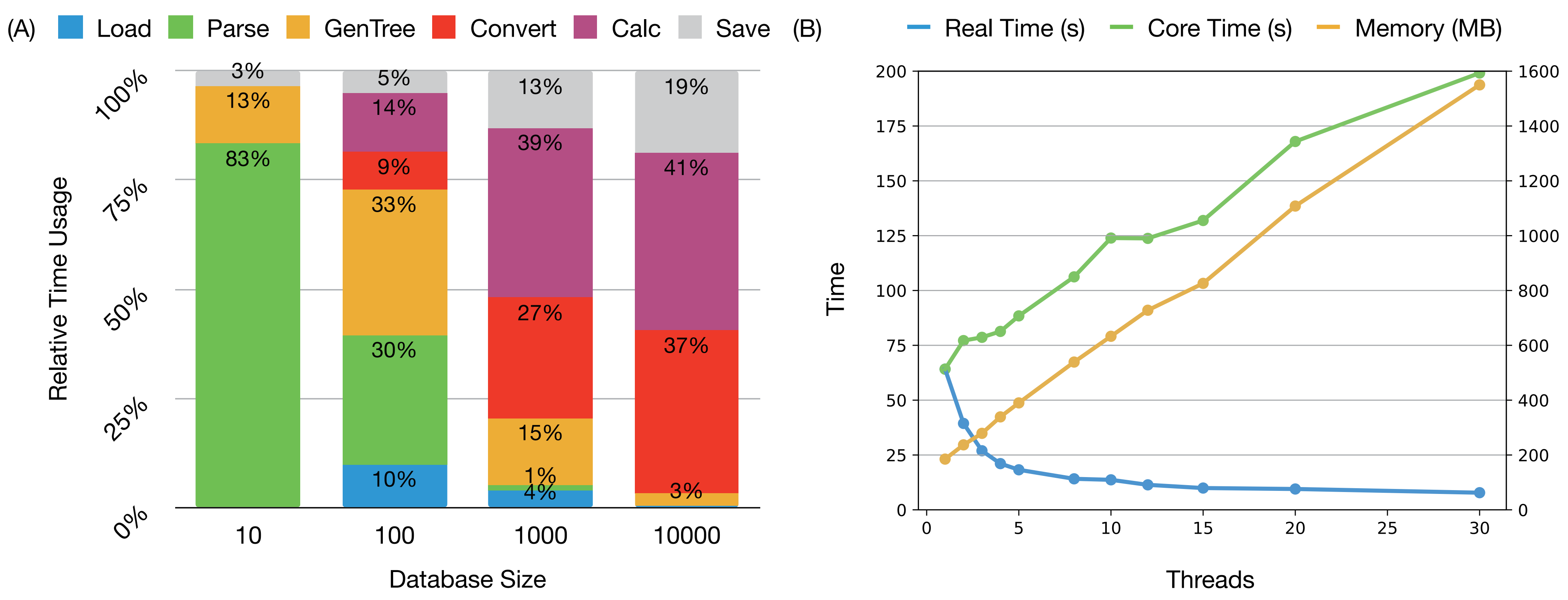

figure 4 Click here to access/download;Figure;Figure 4.eps

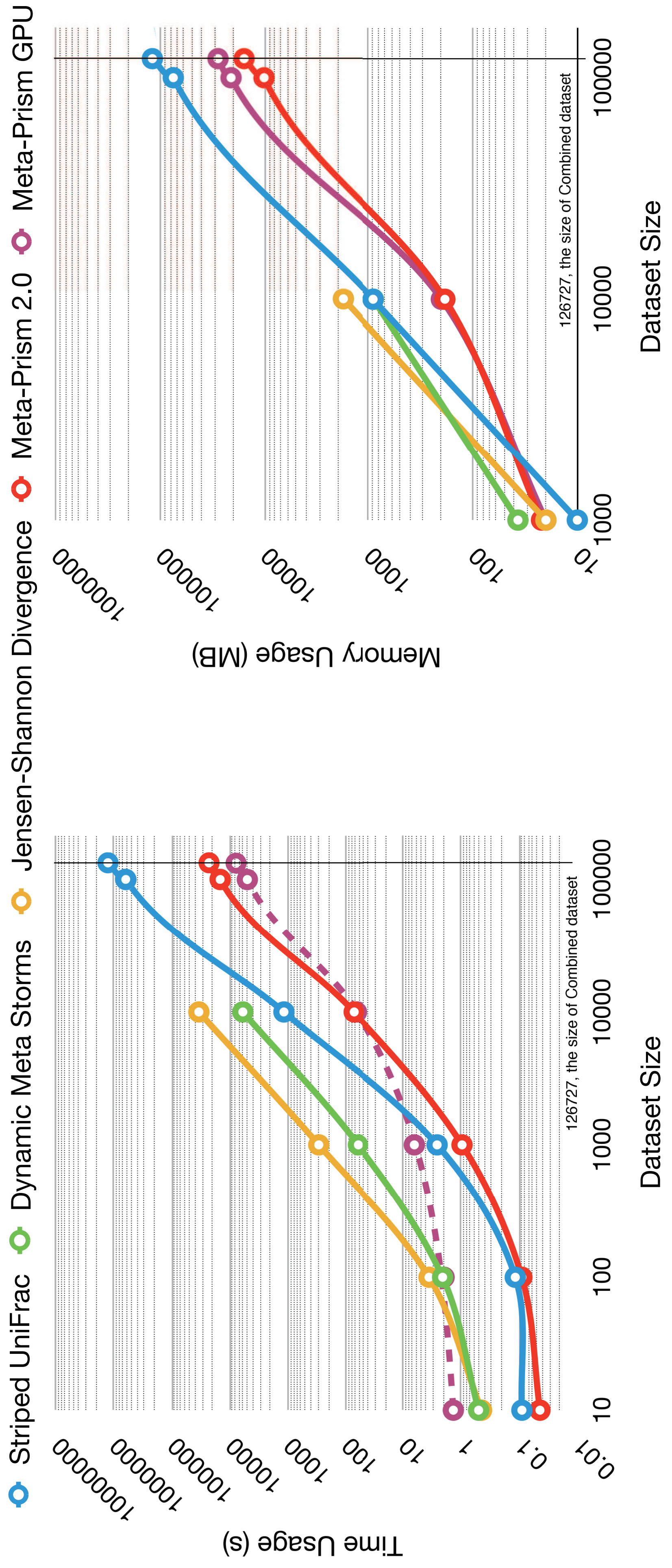

figure 5

Click here to access/download;Figure;Figure 5.eps

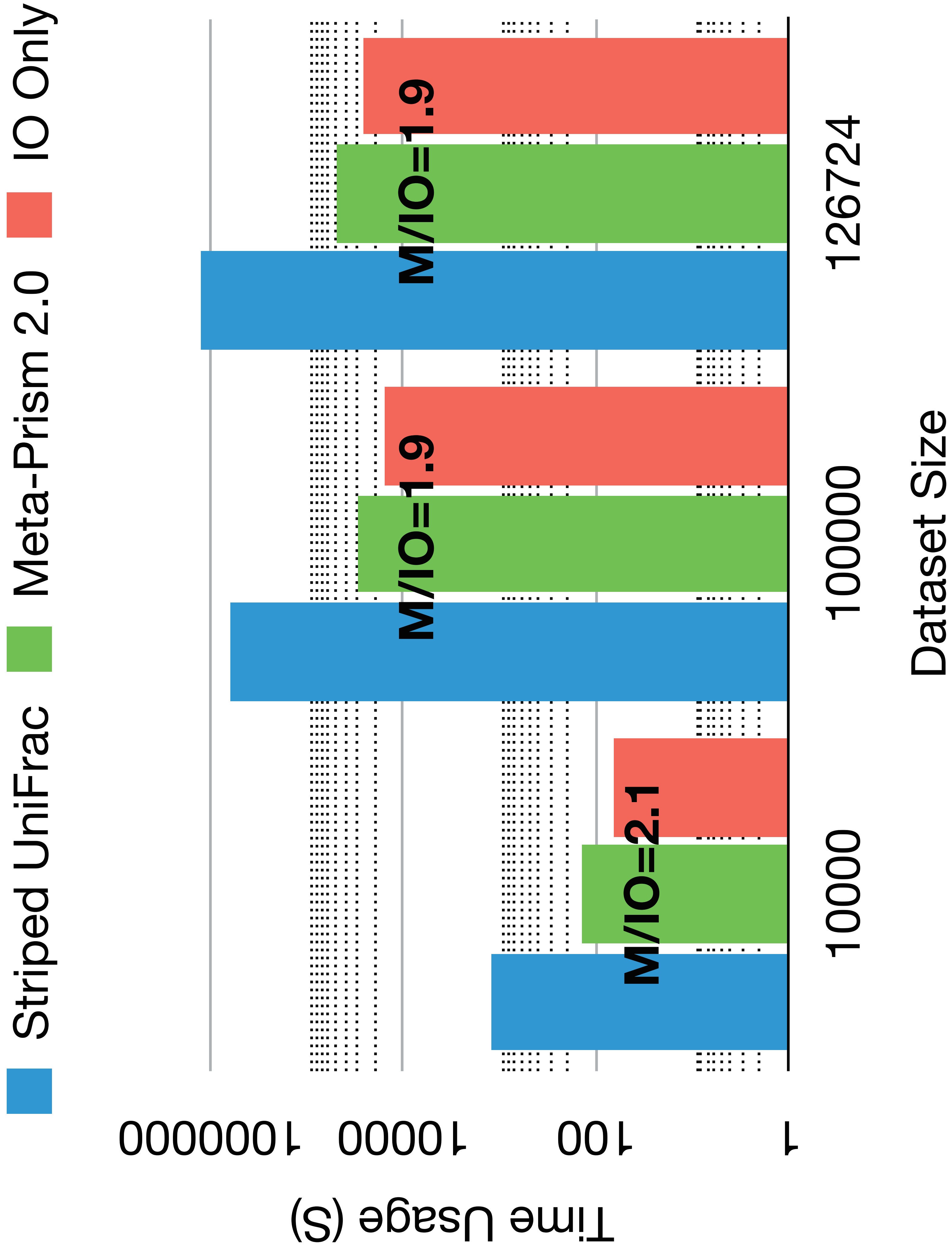

figure 6

Click here to access/download;Figure;Figure 6.eps

A

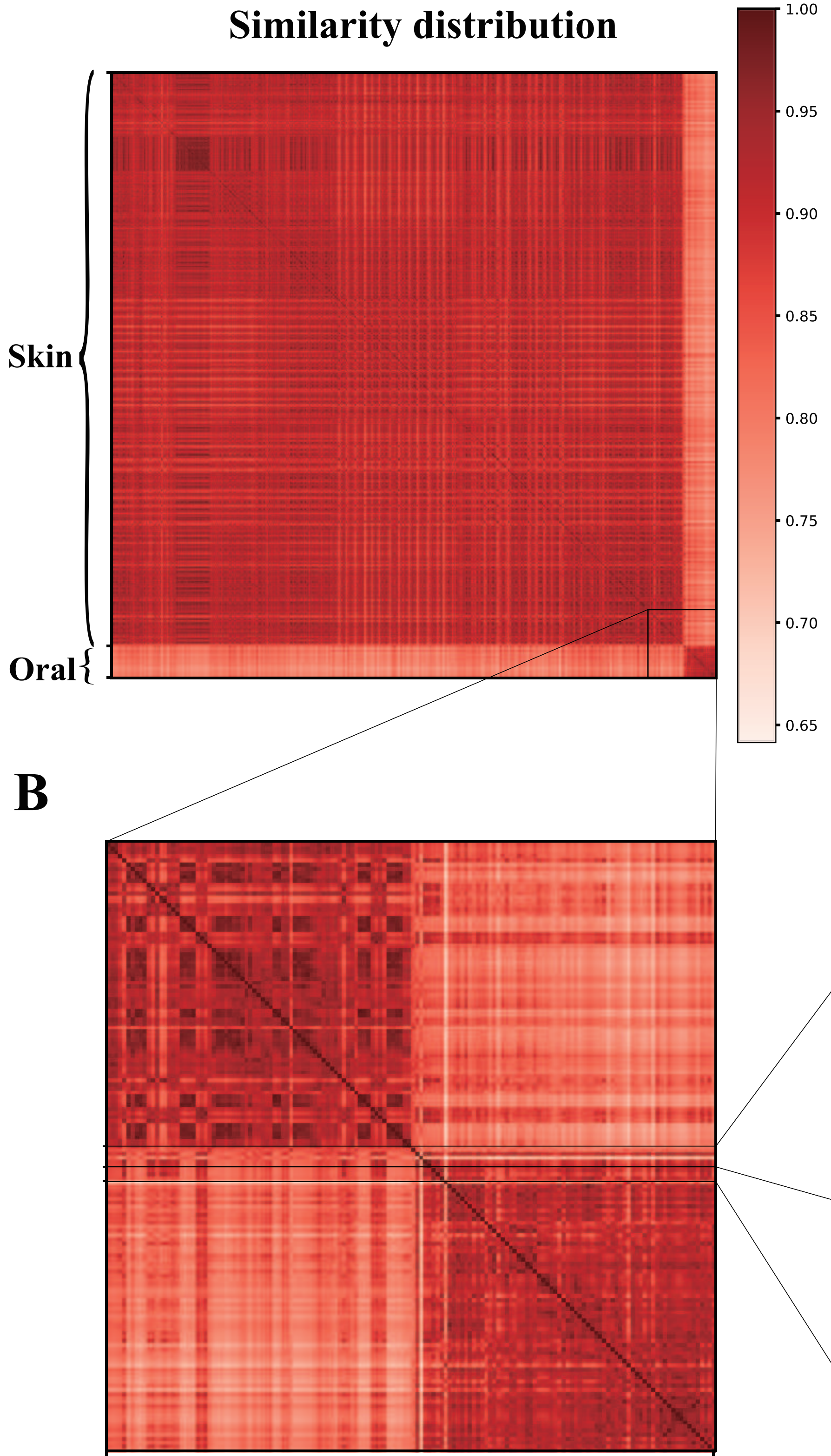


B

C

| | | Predicted Source | | Total number of samples |
|---|---|---|---|---|
| | | Skin | Oral | |
| Actrual source | Skin | 1,256 | 5 | 1,261 |
| | Oral | 4 | 66 | 70 |

D

| Sample ID | Actrual source | Predicted source |
|---|---|---|
| MGYS00005172-SRR5869138 | Skin | Oral |
| MGYS00005172-SRR5869139 | Skin | Oral |
| MGYS00005172-SRR5869349 | Skin | Oral |
| MGYS00005172-SRR5869533 | Skin | Oral |
| MGYS00005172-SRR5869812 | Skin | Oral |
| MGYS00005569-SRR11545352 | Oral | Skin |
| MGYS00005569-SRR11545359 | Oral | Skin |
| MGYS00005569-SRR11545360 | Oral | Skin |
| MGYS00005569-SRR11545364 | Oral | Skin |

figure 7

Click here to access/download;Figure;Figure 7.eps

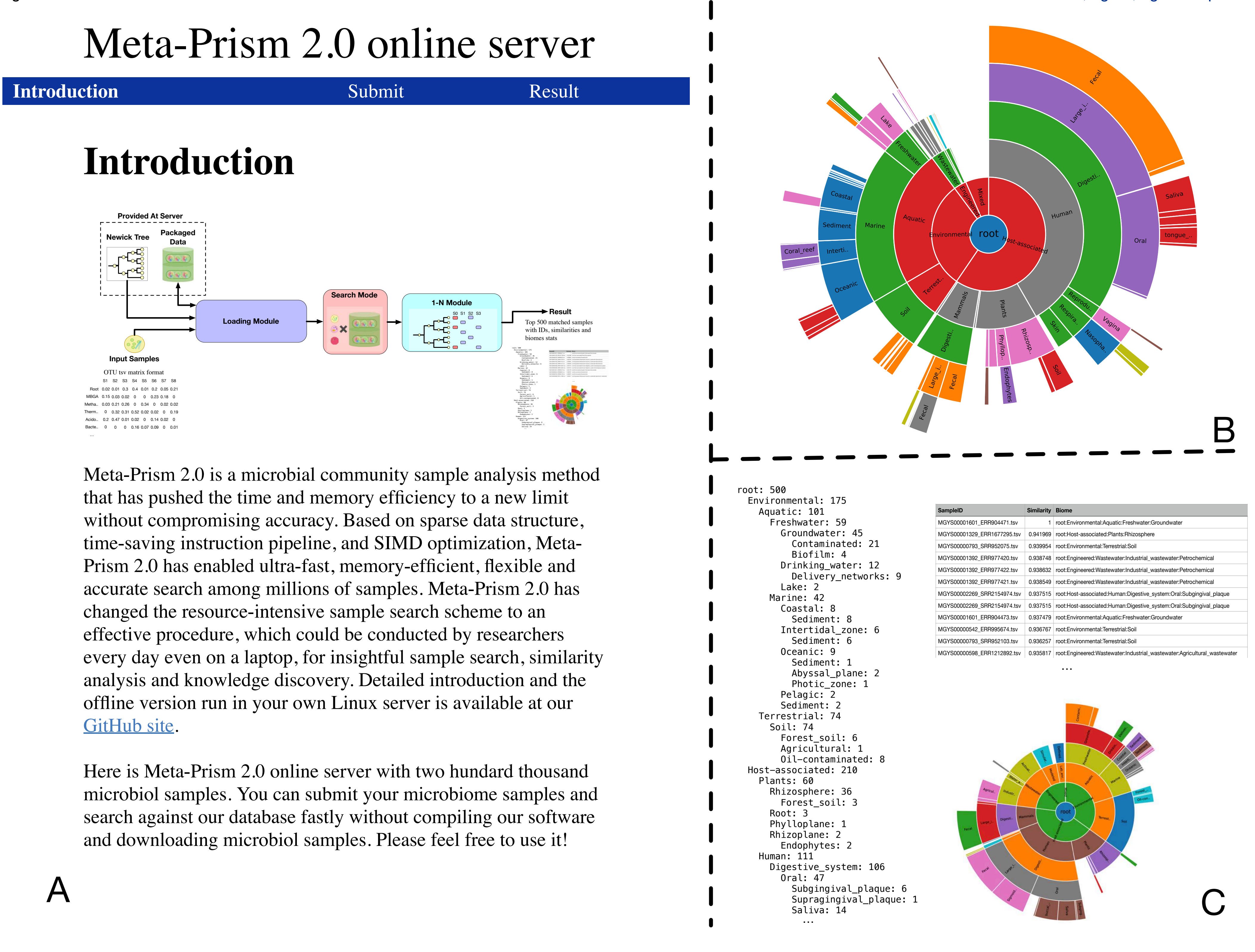

pseudocode about Meta-Prism 2.0

Click here to access/download
**Supplementary Material**
Supplementary Material 1.pdf

detail information of Combined dataset and FEAST dataset

Click here to access/download
**Supplementary Material**
Supplementary Table 1.pdf

detail information of Combined dataset and FEAST dataset

Click here to access/download
**Supplementary Material**
Supplementary Table 1.xlsx
