# Supplementary material for: Meta-Prism 2.0: Enabling algorithm and web server for ultra-fast, memory-efficient, and accurate analysis among millions of microbial community samples
Source: Gigascience. 2022 Jul 28;11:giac073. doi: 10.1093/gigascience/giac073 (PMC9334027; doi:10.1093/gigascience/giac073)
Supplement: giac073_GIGA-D-21-00388_Revision_1 [file giac073_giga-d-21-00388_revision_1.pdf]

GigaScience

Meta-Prism 2.0: Enabling algorithm and web server for ultra-fast, memory-efficient, and accurate analysis among millions of microbial community samples

--Manuscript Draft--

**Manuscript Number:**	GIGA-D-21-00388R1
**Full Title:**	Meta-Prism 2.0: Enabling algorithm and web server for ultra-fast, memory-efficient, and accurate analysis among millions of microbial community samples
**Article Type:**	Research
**Funding Information:**	National Natural Science Foundation of China (32071465) — Mr Kang Ning<br>National Natural Science Foundation of China (31871334) — Mr Kang Ning<br>National Natural Science Foundation of China (31671374) — Mr Kang Ning<br>Ministry of Science and Technology of the People's Republic of China (2018YFC0910502) — Mr Kang Ning<br>National Undergraduate Training Program for Innovation and Entrepreneurship of China (201910487071) — Mr Kai Kang

Abstract:

Background

Microbial community samples have been accumulated at a speed faster than ever, with hundreds of thousands of samples been sequenced each year. Mining such a huge amount of multi-source heterogeneous data is becoming an increasingly difficult challenge, so efficient and accurate compare and search of samples are in urgent need: Faced with millions of samples in the data repository, traditional sample comparison and search approaches fall short in speed and accuracy.

Findings

Here we proposed Meta-Prism 2.0, a microbial community sample analysis method that has pushed the time and memory efficiency to a new limit without compromising accuracy. Based on sparse data structure, time-saving instruction pipeline, and SIMD optimization, Meta-Prism 2.0 has enabled ultra-fast, memory-efficient, flexible and accurate search among millions of samples. Meta-Prism 2.0 was put to test on several datasets, with the largest containing one million samples. Results show that Meta-Prism 2.0's 0.00001s per sample pair compare speed and 8GB memory needs for searching against one million samples have made it one of the most efficient sample analysis methods. Additionally, Meta-Prism 2.0 can achieve accuracy comparable with or better than other contemporary methods. Thirdly, Meta-Prism 2.0 can precisely identify the original biome for samples, thus enabling sample source tracking. Finally, we have provided a web server for fast search of microbial community samples online.

Conclusions

In summary, Meta-Prism 2.0 has changed the resource-intensive sample search scheme to an effective procedure, which could be conducted by researchers every day even on a laptop, for insightful sample search, similarity analysis and knowledge discovery. Meta-Prism 2.0 can be accessed at: https://github.com/HUST-NingKang-Lab/Meta-Prism-2.0, and the web server can be accessed at: https://hust-ningkang-lab.github.io/Meta-Prism-2.0 /.

Corresponding Author:

Kai Kang
Huazhong University of Science and Technology
Beijing, CHINA

Powered by Editorial Manager® and ProduXion Manager® from Aries Systems Corporation

**Corresponding Author Secondary Information:**	
**Corresponding Author's Institution:**	Huazhong University of Science and Technology
**Corresponding Author's Secondary Institution:**	
**First Author:**	Kai Kang
**First Author Secondary Information:**	
**Order of Authors:**	Kai Kang
	Hui Chong
	Kang Ning
**Order of Authors Secondary Information:**	
**Response to Reviewers:**	We thank editor for these suggestions, and thank reviewers for questions and comments. We have updated the manuscript for clarity, as well as deployed all real data applications' workflow to the Code Ocean platform (https://codeocean.com/capsule/3103931). We also modified the program naming, added program documentation and did more tests to improve program robustness.<br><br>However, performance and accuracy analysis require similarity matrix calculations for the entire database, which require, as we show in Figure 4, more than 353 hours of CPU time to calculate. Therefore, we did not deploy this part into Code Ocean, but provided step introductions, intermediate data and supporting scripts in Google Drive (the FTP temporary account provided before seems to have been used by others), and the sharing link is https://drive.google.com/drive/folders/1BB7jyUBigw7ysjY_LCmVEdpaIZSlod-l?usp=sharing.<br><br>We have submitted a point to point docx formatted response file as "Respond.docx", please check it for details.
**Additional Information:**	
**Question**	**Response**
Are you submitting this manuscript to a special series or article collection?	No
**Experimental design and statistics**<br><br>Full details of the experimental design and statistical methods used should be given in the Methods section, as detailed in our Minimum Standards Reporting Checklist. Information essential to interpreting the data presented should be made available in the figure legends.<br><br>Have you included all the information requested in your manuscript?	Yes
**Resources**<br><br>A description of all resources used,	Yes

including antibodies, cell lines, animals and software tools, with enough information to allow them to be uniquely identified, should be included in the Methods section. Authors are strongly encouraged to cite Research Resource Identifiers (RRIDs) for antibodies, model organisms and tools, where possible.<br><br>Have you included the information requested as detailed in our Minimum Standards Reporting Checklist?	
**Availability of data and materials**<br><br>All datasets and code on which the conclusions of the paper rely must be either included in your submission or deposited in publicly available repositories (where available and ethically appropriate), referencing such data using a unique identifier in the references and in the "Availability of Data and Materials" section of your manuscript.<br><br>Have you have met the above requirement as detailed in our Minimum Standards Reporting Checklist?	Yes

Manuscript
Click here to access/download;Manuscript;manuscript.docx

Meta-Prism 2.0: Enabling algorithm and web server for ultra-fast, memory-efficient, and accurate analysis among millions of microbial community samples

Kai Kang[1,2,$], Hui Chong[1,$], Kang Ning[1,*]

[1] Key Laboratory of Molecular Biophysics of the Ministry of Education, Hubei Key Laboratory of Bioinformatics and Molecular-imaging, Center of AI Biology, Department of Bioinformatics and Systems Biology, College of Life Science and Technology, Huazhong University of Science and Technology, Wuhan 430074, China

[2] Center for Quantitative Biology, Academy for Advanced Interdisciplinary Studies, Peking University, Beijing 100871, China

[$] These authors contributed equally to this work

** Corresponding author*

E-mail: ningkang@hust.edu.cn

Abstract

Background

Microbial community samples have been accumulated at a speed faster than ever, with hundreds of thousands of samples been sequenced each year. Mining such a huge amount of multi-source heterogeneous data is becoming an increasingly difficult challenge, so efficient and accurate compare and search of samples are in urgent need: Faced with millions of samples in the data repository, traditional sample comparison and search approaches fall short in speed and accuracy.

Findings

Here we proposed Meta-Prism 2.0, a microbial community sample analysis method that has pushed the time and memory efficiency to a new limit without compromising accuracy. Based on sparse data structure, time-saving instruction pipeline, and SIMD optimization, Meta-Prism 2.0 has enabled ultra-fast, memory-efficient, flexible and accurate search among millions of samples. Meta-Prism 2.0 was put to test on several datasets, with the largest containing one million samples. Results show that Meta-Prism 2.0's 0.00001s per sample pair compare speed and 8GB memory needs for searching against one million samples have made it one of the most efficient sample analysis methods. Additionally, Meta-Prism 2.0 can achieve accuracy comparable with or better than other contemporary methods. Thirdly, Meta-Prism 2.0 can precisely identify the original biome for samples, thus enabling sample source tracking. Finally, we have provided a web server for fast search of microbial community samples online.

Conclusions

In summary, Meta-Prism 2.0 has changed the resource-intensive sample search scheme to an effective procedure, which could be conducted by researchers every day even on a laptop, for insightful sample search, similarity analysis and knowledge discovery. Meta-Prism 2.0 can be accessed at: https://github.com/HUST-NingKang-Lab/Meta-Prism-2.0, and the web server can be accessed at: https://hust-ningkang-lab.github.io/Meta-Prism-2.0/.

Introduction

Microbial communities have asserted great influences on healthcare, environment, and industry[1-4]. As such, an increasing number of projects have been conducted on microbial communities around the world, such as those from the “Human Microbiome Project”[1, 2] and the “Earth Microbiome Project”[3, 4]. Mining this massive amount of samples has already discovered knowledge about the microbial community and their effects on the environment and human health[5, 6], providing an opportunity to study the hidden evolution and ecology patterns among microbial communities.

A microbial community sample (also referred to as the sample) is represented by the hierarchically structured taxa (species, genus, families, etc.) and their relative abundances (also referred to as the community structure), and these species are functioning in concert to maintain stability and adapt to the specific environments (also referred to as the niches or biomes) where the microbial community is living. These samples’ community structures are often associated with the biomes and a variety of characteristics of the biomes. For example, the community structures of the human gut microbiome have been linked to multiple aspects of human life, such as health[6, 7], early development[8], immigration[9], and pregnancy[10]. Thus, there is a large amount of hidden information in the community structures and remains to be discovered. These challenges in current microbiome researches are calling fast community-level comparison and search among the rapidly accumulating number of microbial communities.

There are already methods that existed for comparison and search of samples. The distance-based methods are the first batches designed for the purpose, whose primary strategy is to compare the similarity or distance between two samples. The simplest distance-based method is the Jensen-Shannon Divergence (JSD) measurement[11], which only considered species abundances in the community. More advanced distance-based methods considered both species abundances and their phylogenetic relationships. For example, UniFrac[12] is a typical distance-based method, which firstly maps their respective sets of taxon abundances on the phylogenetic tree, and secondly traverses the tree and executes operation at each node (each representing a taxon on the

phylogenetic tree) to calculate their similarity. Fast UniFrac[13] and Meta-Storms[14] optimized such a procedure by changing tree traversal to array loop. Striped UniFrac[15] further optimized matrix similarity comparison by reorganizing samples. Dynamic Meta-Storms enables species-level accuracy by introducing virtual nodes[16]. Previously, we designed Meta-Prism 1.0, a fast and accurate microbial community sample search tool[17]. Meta-Prism 1.0 generates an index to rapidly select samples with similar biome and top phylum for comparison. Furthermore, Meta-Prism 1.0 uses GPU to accelerate comparison. However, given that more than a million community samples have already been deposited into public databases[18, 19], state-of-the-art methods including Meta-Prism 1.0 face difficulties in comparison and searching among these samples, while rendering knowledge discovery from samples formidable. Additionally, microbial community samples' data are very sparse. These methods use fixed-length arrays to save abundances with lengths equals to entities number of the phylogenetic tree, wasting a considerable amount of memory. They also spend much time operating on these empty nodes.

To solve the large-scale microbial community sample search problem, we have redesigned and updated Meta-Prism to its second version (Meta-Prism 2.0). Facing the large and sparse characteristics of microbial samples, we adopted a special sparse storage format and a fast 1-N calculation method. This greatly reduces memory usage and time consumption. As the computing and storage efficiency increased, it adds similarity matrix calculation function to analyze more than 100,000 samples' beta diversity. When searching samples, due to the efficiency improvement, Meta-Prism 2.0 no longer needs to generate the index system to select high-probability subsets for similarity comparison, but conduct an exhaustive search against the entire database. Thus it has higher flexibility (when searching among customized datasets) and robustness than Meta-Prism 1.0. More importantly, with these improvements of efficiency and space, Meta-Prism 2.0 now can deal with one million or even more microbial community samples and is one of the fastest microbial community sample search methods to date.

Using several datasets including the largest one containing a million samples, we demonstrated that it can achieve at least 20 times speed-up compared to the contemporary approach (e.g., Meta-Prism 1.0 and Striped UniFrac), and Meta-Prism 2.0 is the only method that could handle the search against a million samples. The memory utilization is also very efficient: Compared with other methods including JSD, Striped UniFrac, and Dynamic Meta Storms, when analyzing dataset beta diversity which size exceeds 10,000, Meta-Prism 2.0 can at least save 80% of memory space needed. Though we have saved time and memory by magnitudes, the accuracy is not compromised. For example, Meta-Prism 2.0 obtained 0.99 AUC in distinguishing samples from different biomes for more than one hundred thousand samples [20]. Meta-Prism 2.0 has changed the traditional computational resource-intensive sample search

to a cheap and effective procedure that could be conducted by researchers every day, for the discovery of intricate relationships among samples. Meta-Prism 2.0 can be accessed at: https://github.com/HUST-NingKang-Lab/Meta-Prism-2.0. And the fast and accurate microbial community sample search could also be experienced on the web server at: https://hust-ningkang-lab.github.io/Meta-Prism-2.0/.

Methods

Meta-Prism 2.0 calculates similarities between microbial communities using two calculation modes: search mode and matrix mode. The search mode takes two datasets (query and target) as input and then outputs each query sample's top N similar matches in the target dataset. The matrix mode takes a dataset as input and outputs a pair-wise similarity matrix for all samples in the dataset (**Figure 1A**). These datasets can be produced by commonly used tools such as QIIME[21], MAPseq[22], and MetaPhlAn[23].

Meta-Prism 2.0 has unlocked several key computational techniques for efficient comparison (**Figure 1**): Firstly, it utilizes a sparse data structure to cut down the memory and disk usage (**Figure 1B**). Secondly, to further cut down the memory usage, Meta-Prism 2.0 only stores essential taxa (taxa appeared in query samples) of the phylogenetic tree and abundances for similarity calculation. (**Figure 1C (1-4)**). Thirdly, to cut down the time usage, Meta-Prism 2.0 discards redundant execution before diving into similarity calculation (**Figure 1C (3)**). Fourthly, Meta-Prism 2.0 utilizes a fast 1-N compare module to enable further accelerations through the instruction pipeline[24] and single instruction multiple data (SIMD) optimization (**Figure 1C (5)**). Last but not least, Meta-Prism 2.0 utilizes a customized 16-bit floating-point to store the similarity matrix in a memory-saving manner (**Figure 1D**).

Space-saving data format

Each microbial community sample consists of classified taxa and their relative abundances. Meta-Prism 2.0 will find the taxa in the phylogenetic tree. For the representation of a single microbial community sample, most of the phylogenetic tree nodes are redundant. Unlike other methods that store in fixed-length arrays, Meta-Prism 2.0 stores taxonomic abundance data in a sparse format, that is, uses a variable-length list to store only relatively abundant non-zero nodes: the data includes their relative abundance and the node id of phylogenetic tree (**Figure 1B**). When calculating similarities, Meta-Prism 2.0 converts sparse data back to dense data (Convert step, **Figure 1C (5),** Algorithm 5 in **Supplementary Materials**). The sparse data structure is applied to disk storage and memory cache to reduce space utilization globally.

The storage scheme is further optimized at the step of similarity result storage. To store similarity results for a sample pair, we designed 16 bits floating-point with four exponential bits and 12 mantissa bits. Considering that the similarities are between zero and one, we removed two sign bits of exponent and mantissa to increase the gamut and precision of the floating-point (**Figure 1D**).

Similarity measurement independent of data type and sequencing depth

Our similarity is proposed to measure similarity between a pair of community samples, independent of data type and sequencing depth [14](section 2.2.2). The details of similarity calculation are shown at Algorithm 6 in **Supplementary Materials** with the default execution order being generated from Algorithm 2 in **Supplementary Materials** with all node marked**.** To calculate the similarity of the two samples (n=1 in pseudocode), we will recursively calculate the similarity of the relative abundance of the two samples at each node and deduce, and then multiply the relative abundance remained by one minus the evolutionary distance and send it to the parent node.

Fast 1-N sample comparison

We further optimized the time usage to the minimum extend through a fixed execution order and SIMD[25]. Current methods traverse phylogenetic tree (with redundant nodes) and execute operation during similarity calculation (Striped Unifrac calculates the difference at each node, divides it by the branch distance. Meta-Storm and Dynamic Meta-Storm accumulates the similarity at each node and passes the residual abundance to the parent node. Meta-Prism 1.0 calculates the difference at each node, divides it by 1 minus the evolutionary distance and passes it to the parent node), wasting time on redundant operations[15-17]. In Meta-Prism 1.0, for nodes with no abundance (which also are the vast majority), the system will calculate at variables value 0, without any influence on the result. Some nodes have only the relative abundance of one sample, and the most of the system's calculations on them are invalid which are equivalent to multiplying their abundances by one minus evolutionary distance and passing these to their parent nodes. To save the time wasted on such operations, when Meta-Prism 2.0 calculates 1-N comparison (S_0 against S_n), it will only consider nodes that are abundant in S_0 (marked node) and generate a fixed execution order based on them (GenOrder step, **Figure 1C (2 and 3),** Algorithm 2 in **Supplementary Materials**). To deal with nodes that are only contain abundant in S_n, Meta-Prism 2.0 will multiply the abundance on these nodes by the cumulative evolutionary distance factor and send them to the nearest labelled parent nodes (Convert step, **Figure 1C (4),** Algorithm 5 in **Supplementary Materials**). Which node to send and the factor value is calculated at Algorithm 4 in **Supplementary Materials**. The fixed execution order without branches and jumps will lead the CPU to use the instruction pipeline. Additionally, Meta-Prism 2.0 is implemented based on SIMD AVX intrinsic[26], thus can execute operations to compare a sample S0 with other multiple samples (referred to as Sn) at the same time (SimilarityCalculation step, **Figure 1C (5),** Algorithm 6 in **Supplementary Materials**).

We packaged these steps as the "1-N module", and use the module to execute fast comparison and search.

Results

Materials and execution environments used for evaluation

Through manual curation from the EBI MGnify database[18], we obtained a dataset consists of 126,727 microbial community samples belonging to 114 different biomes, defined as the Combined dataset. We also obtained a dataset that consists of 10,270 samples belonging to three biomes: Fecal, Human, and Mixed, which have been used in the FEAST study[27], defined as the FEAST dataset (**Table 1**). To evaluate Meta-Prism 2.0's speed and memory efficiency on the scale of one million samples, we synthesized a dataset with 1,000,010 samples based on the Combined dataset. All samples from these three datasets are accessible from https://github.com/HUST-NingKang-Lab/Meta-Prism-2.0. We used SILVA 132 LTPs132 SSU phylogenetic tree[28] in all experiments included in this study.

Striped UniFrac, Dynamic Meta-Storms, Meta-Prism 2.0 were compiled by GCC 4.8.5 and ran on CentOS 6.7 with Intel(R) Xeon(R) CPU E5-2678 v3 @ 2.50GHz and 252GB memory. The Jensen-Shannon divergence was calculated utilizing Python 3.7.3 and SciPy 1.4.1 and ran on the same CentOS device. The executable Meta-Prism 2.0 steps' time usage was compiled by clang-1100.0.33.16, and evaluated by Xcode11.5 Instruments Time Profiler, ran on macOS 10.15 with Intel(R) Core (TM) i7-9750H and 32GB memory. Meta-Prism GPU version was compiled by NVCC 10.1 and ran on RTX 2080Ti.

Accuracy evaluation

We assessed the search accuracy of different methods in the context of source tracking, namely by checking the consistency of the predicted biomes and query samples' actual biomes. This evaluation is based on the realization that the microbial communities collected from the same biome always share similar patterns in their taxonomical structures and relative abundances[20, 27]. Specifically, we used simple cross-validation for the evaluation, based on searching 12.5% randomly chosen samples (considered as query dataset) against the rest samples (considered as target dataset). For each query sample, we selected the top 100 most similar target samples. The similarity of these samples is then summed by biome and normalized by dividing by the total number of samples in the source dataset for each biome. After the resulting values are normalized, it is the probability that the test sample belongs to each biome.

The evaluation performances are shown in **Figure 2**. The varying classification threshold that generates different sensitivities and specificities range from 0.01 to 1

with a fixed step size of 0.01. On the FEAST dataset, each method predicted biome for testing samples according to the biomes included in the source dataset (Fecal, Human, and Mixed). Distance-based phylogenetic tree approaches (Meta-Prism 2.0, Striped UniFrac, and Dynamic Meta-Storms) showed similarly good performance, while Jensen-Shannon Divergence (JSD) obtained a lower AUC of 0.9512. On the Combined dataset, each method predicted biome for testing samples according to 114 biomes included in the source dataset (87.5% of the Combined dataset). JSD and Dynamic Meta-Storms cannot finish the calculation within an acceptable time (10 days). We only compared Meta-Prism 2.0 and Striped UniFrac. Meta-Prism obtained a higher AUC result of 0.9934, while Striped UniFrac's AUC result was 0.9153.

Computational speed assessment

The time and memory efficiency are the most profound advantage of Meta-Prism 2.0. We first assessed Meta-Prism 2.0's speed based on using datasets with different dataset sizes and using different numbers of CPU threads (**Figure 3**). The setting was matrix mode, which takes one dataset as input, then calculates all sample pairs' similarities, and the output is a similarity matrix. The time cost is split into several parts according to computational steps. Our 1-N module adds GenOrder and Convert steps, which increase linearly and quadratically with the increase of dataset size, respectively.

We also evaluated Meta-Prism 2.0 performance on a dataset with one million samples (see **Materials** for details). Meta-Prism 2.0 can efficiently package one million samples into a 369 MB-sized file for storage and load them within 27 seconds. We transferred the whole workload to a laptop and searched 100 samples against this dataset with a single CPU thread. It cost 324.96 seconds (less than 6 minutes) CPU time to complete the search using only 6.9 GB memory. So far as we know, Meta-Prism 2.0 is the only method that could handle the search against a million samples.

Computational speed comparison

We further selected datasets with different dataset sizes (10, 100, 1,000, 10,000, 100,000 and 126,727) from the Combined dataset to compared different methods. The setting is again matrix mode. We compared time and memory usage of Striped UniFrac, Dynamic Meta Storms, JSD, Meta-Prism GPU, and Meta-Prism 2.0. Meta-Prism GPU is the only method that uses GPU for calculation, and we considered real-time usage for the measurement. In comparison, we took CPU core time usage as other methods' time usage. JSD and Meta-Storms cannot calculate the similarity matrix when dataset size $\geq$ 10,000 within an acceptable time (10 days).

Results show that Meta-Prism 2.0 could achieve superior performance on both time usage and memory usage (**Figure 4)**. Specifically, when the dataset sizes are no more than one thousand, Meta-Prism 2.0 used a similar core time compared with Striped UniFrac **(Figure 4A)**. When dataset size became more extensive, the performance gap

between Meta-Prism 2.0 and Striped UniFrac became larger. When calculating the similarity matrix for the Combined dataset (generating 126,727 × 126,727 similarity matrix), Meta-Prism 2.0 was 55 times faster than Striped UniFrac. Meta-Prism GPU's real-time usage was smaller than Meta Prism 2.0 core time usage. However, when Meta-Prism 2.0 uses 3 CPU cores or more, it will be faster than Meta-Prism GPU.

Meta-Prism 2.0's memory usage was only 11.1% of Striped UniFrac's when calculating the similarity matrix for the Combined dataset with more than 100,000 samples. The utilization of customized 16 bits floating point was the key reason because it can efficiently store similarity matrix, which is the largest storage burden that increases quadratically when the dataset size increases.

Wondering how far is the speed of Meta-Prism 2.0 to the theoretical lower bound for the sample search, we took IO Only as the lower bound for sample search, in which we only record the time used for loading data and writing matrix calculation results (**Figure 5**). The result shows that the time costs of Meta-Prism 2.0 is only two times of IO Only, while magnitude smaller than those of Striped UniFrac.

Real data applications

Meta-Prism 2.0 can precisely identify the biome for samples of unknown origin, thus enabling the source tracking of samples. For example, it enables accurate differentiation of samples from close biomes such as "human skin" and "human oral" (the first application), identification of the biome for samples with unclear origin (the second application), as well as detection of microbial contamination (the third application). We published these applications' workflow at Code Ocean for researchers to track and reproduce (https://codeocean.com/capsule/3103931).

Firstly, we tested Meta-Prism 2.0's ability to accurately differentiating samples from close biomes. We obtained 1,261 skin metagenomic samples (MGYS00005172)[29] and 70 oral metagenomic samples (MGYS00005569)[30] from MGnify[18]. We used Meta-Prism 2.0 to calculate the similarities matrix of 1,331 samples on a laptop, which cost only 3.75 seconds and 11MB of memory. We also clustered samples based on their similarities by using affinity propagation from Scikit-learn (version 0.20.3). The samples were successfully clustered into two groups whose sizes are 1,260 and 71 (**Figure 6**). Within 1,331 samples, only nine samples (five skin samples and four oral samples) were miss-clustered, proving Meta-Prism 2.0's ability to fast and accurately differentiate samples from close biomes.

Secondly, we evaluated the performance of Meta-Prism 2.0 on source tracking environmental samples from less-studied biomes, based on searching 11 groundwater samples curated from Saudi Arabian (MGYS00001601)[31] against the combined dataset. The biome "groundwater" is less studied, with a handful of samples in the

combined dataset (MGYS00005245). Results show that Meta-Prism 2.0 could successfully identify source-related biomes for samples from “groundwater”. Within the top 100 most similar community samples for each “groundwater” query sample, there are on average 64 groundwater-related samples (from “root-Environmental-Terrestrial”, “root-Environmental-Aquatic”, “root-Engineered-Wastewater” and “root-Host-associated-Plants”) for each query sample (**Table 2)**. Nevertheless, there is no “groundwater” sample in the top 100 similar samples searched by Meta-Prism 2.0, since “groundwater” samples in the combined dataset are curated from New Zealand, which is in nature drastically different from our query samples. The result suggests that the geographic origins also influence the community structures, which was already confirmed by previous studies[32].

Finally, we evaluated the Meta-Prism 2.0’s power in detecting microbial contamination. We investigated the contamination of indoor house surfaces community by selecting 611 samples from indoor house surfaces in Chicago as query samples and searching against 6,285 samples (899+3,773+721+692+200 from “human skin”, “environmental”, “mammal”, “plants”, and "insecta", respectively). The analysis costs only 6.16 seconds to complete. Our results show that the most closed biome source for indoor house surface samples is “human skin” (average similarity 0.889), indicating a large proportion of microbial community contamination from human skin, which agrees with previous analyses by SourceTracker[20] and FEAST[27] (**Table 3**). Again, it proved the ability of Meta-Prism 2.0 for accurate and fast microbial community contamination screening.

Web server for fast and accurate microbial community sample search

For easy use of Meta-Prism 2.0, we also designed an online web server for Meta-Prism 2.0 (**Figure 7**), with a pre-compiled Meta-Prism 2.0 executable file and a built-in dataset contains more than 0.2 million microbiome samples. This dataset including major categories such as digestive system, aquatic, and soil, as well as sub-categories such as oil-contaminated clay, thermal springs sediment, and bioreactor for biological phosphorus removal. The high efficiency of Meta-Prism 2.0 enables any query against this huge dataset to be completed within one second, with high accuracy.

Discussions and Conclusion

In this work, we designed Meta-Prism 2.0 as an ultrafast and memory-efficient approach to analysis against millions of microbial community samples. The sample compare and search problems have encountered great difficulties when faced with millions of samples, primarily due to the computational space and time limitations. Meta-Prism 2.0 was designed based on sparse data structure, time-saving instruction pipeline, SIMD optimization, and exhaustive search strategy, enabling flexible, ultra-fast, memory-efficient, and added beta diversity analysis function.

Results show that compared to the current methods serving the same purpose, Meta-Prism 2.0 is at least 20 times faster, while memory cost is at least four times smaller. Additionally, the speed of Meta-Prism 2.0 is close to the lower bound of the search. Furthermore, according to our experiment, Meta-Prism 2.0 can even store all samples' community structure from the EBI MGnify dataset (300,000 in total as of Oct. 2020) on a laptop and searching against it at an unprecedented speed. Finally, we provided several concrete examples, which have proven the effectiveness and utility of Meta-Prism 2.0 in knowledge discovery. And the fast and accurate microbial community sample search could also be experienced on the web server, on which any query against this huge dataset to be completed within one second, with high accuracy

In summary, Meta-Prism 2.0 can perform searches among millions of samples with low memory cost and fast speed, enabling source tracking and knowledge discovery from sample mining at a massive scale. Meta-Prism 2.0 has optimized the traditional resource-intensive sample search and similarity matrix calculation into an affordable and effective procedure that researchers could conduct every day for mining intricate relationships among samples and discover previously unknown knowledge.

Availability of Supporting Source Code and Requirements

Project name: Meta-Prism 2.0
Project home page: https://hust-ningkang-lab.github.io/Meta-Prism-2.0/
GitHub repository: https://github.com/HUST-NingKang-Lab/Meta-Prism-2.0
Operating systems: Platform independent
Programming language: C++
Other requirements: Compiler support C++11
License: GPL-3.0 License
RRID: SCR_021836
bio.tools ID: Meta-Prism 2.0
Reproducible publication: https://codeocean.com/capsule/3103931

Availability of Data

Our code is published at https://github.com/HUST-NingKang-Lab/Meta-Prism-2.0. All the datasets curated for this study are available at this project's "Releases".

Competing of Interest

The authors declare that they have no competing interests.

Acknowledgments

The authors would like to thank Yi Zhan for insightful discussions about the project.

Funding

This work was partially supported by National Natural Science Foundation of China grant 32071465, 31871334, and 31671374, Ministry of Science and Technology's grant 2018YFC0910502, and National Undergraduate Training Program for Innovation and Entrepreneurship of China (Program No. 201910487071).

Authors' Contributions

K.N. conceived and supervised this study. K.K. designed and developed Meta-Prism 2.0 software and web server. K.K and H.C. tested Meta-Prism 2.0. K.K, H.C., and K.N. wrote the manuscript. All authors read and approved the final manuscript.

Additional Files

Supplementary Material 1: Pseudocode about Meta-Prism 2.0.
Supplementary Table 1: Detail information of Combined dataset and FEAST dataset.

References

1. Integrative HMPRNC: **The Integrative Human Microbiome Project: dynamic analysis of microbiome-host omics profiles during periods of human health and disease**. *Cell Host Microbe* 2014, **16**(3):276-289.
2. Turnbaugh PJ, Ley RE, Hamady M, Fraser-Liggett CM, Knight R, Gordon JI: **The human microbiome project**. *Nature* 2007, **449**(7164):804-810.
3. Gilbert JA, Jansson JK, Knight R: **The Earth Microbiome project: successes and aspirations**. *BMC Biol* 2014, **12**:69.
4. Thompson LR, Sanders JG, McDonald D, Amir A, Ladau J, Locey KJ, Prill RJ, Tripathi

A, Gibbons SM, Ackermann G *et al*: **A communal catalogue reveals Earth's multiscale microbial diversity**. *Nature* 2017, **551**(7681):457-463.

5. Dominguez-Bello MG, De Jesus-Laboy KM, Shen N, Cox LM, Amir A, Gonzalez A, Bokulich NA, Song SJ, Hoashi M, Rivera-Vinas JI: **Partial restoration of the microbiota of cesarean-born infants via vaginal microbial transfer**. *Nature medicine* 2016, **22**(3):250.

6. Thomas S, Izard J, Walsh E, Batich K, Chongsathidkiet P, Clarke G, Sela DA, Muller AJ, Mullin JM, Albert K: **The host microbiome regulates and maintains human health: a primer and perspective for non-microbiologists**. *Cancer research* 2017, **77**(8):1783-1812.

7. Zeller G, Tap J, Voigt AY, Sunagawa S, Kultima JR, Costea PI, Amiot A, Bohm J, Brunetti F, Habermann N *et al*: **Potential of fecal microbiota for early-stage detection of colorectal cancer**. *Mol Syst Biol* 2014, **10**:766.

8. Bäckhed F, Roswall J, Peng Y, Feng Q, Jia H, Kovatcheva-Datchary P, Li Y, Xia Y, Xie H, Zhong H: **Dynamics and stabilization of the human gut microbiome during the first year of life**. *Cell host & microbe* 2015, **17**(5):690-703.

9. Vangay P, Johnson AJ, Ward TL, Al-Ghalith GA, Shields-Cutler RR, Hillmann BM, Lucas SK, Beura LK, Thompson EA, Till LM: **US immigration westernizes the human gut microbiome**. *Cell* 2018, **175**(4):962-972. e910.

10. Koren O, Goodrich JK, Cullender TC, Spor A, Laitinen K, Bäckhed HK, Gonzalez A, Werner JJ, Angenent LT, Knight R: **Host remodeling of the gut microbiome and**

metabolic changes during pregnancy. *Cell* 2012, **150**(3):470-480.

11. Lin J: **Divergence measures based on the Shannon entropy**. *IEEE Transactions on Information theory* 1991, **37**(1):145-151.

12. Lozupone C, Knight R: **UniFrac: a New Phylogenetic Method for Comparing Microbial Communities**. *Applied and Environmental Microbiology* 2005, **71**(12):8228-8235.

13. Hamady M, Lozupone C, Knight R: **Fast UniFrac: facilitating high-throughput phylogenetic analyses of microbial communities including analysis of pyrosequencing and PhyloChip data**. *The ISME journal* 2010, **4**(1):17-27.

14. Su X, Xu J, Ning K: **Meta-Storms: efficient search for similar microbial communities based on a novel indexing scheme and similarity score for metagenomic data**. *Bioinformatics* 2012, **28**(19):2493-2501.

15. McDonald D, Vázquez-Baeza Y, Koslicki D, McClelland J, Reeve N, Xu Z, Gonzalez A, Knight R: **Striped UniFrac: enabling microbiome analysis at unprecedented scale**. *Nature Methods* 2018, **15**(11):847-848.

16. Jing G, Zhang Y, Yang M, Liu L, Xu J, Su X: **Dynamic Meta-Storms enables comprehensive taxonomic and phylogenetic comparison of shotgun metagenomes at the species level**. *Bioinformatics* 2019, **36**(7):2308-2310.

17. Zhu M, Kang K, Ning K: **Meta-Prism: Ultra-fast and highly accurate microbial community structure search utilizing dual indexing and parallel computation**. *Briefings in Bioinformatics* 2020, **00**(December 2019):1-11.

18. Mitchell AL, Almeida A, Beracochea M, Boland M, Burgin J, Cochrane G, Crusoe MR,

Kale V, Potter SC, Richardson LJ: **MGnify: the microbiome analysis resource in 2020**. *Nucleic acids research* 2020, **48**(D1):D570-D578.

19. Coordinators NR: **Database resources of the National Center for Biotechnology Information**. *Nucleic Acids Res* 2016, **44**(D1):D7-19.

20. Knights D, Kuczynski J, Charlson ES, Zaneveld J, Mozer MC, Collman RG, Bushman FD, Knight R, Kelley ST: **Bayesian community-wide culture-independent microbial source tracking**. *Nature methods* 2011, **8**(9):761-763.

21. Bolyen E, Rideout JR, Dillon MR, Bokulich NA, Abnet CC, Al-Ghalith GA, Alexander H, Alm EJ, Arumugam M, Asnicar F *et al*: **Reproducible, interactive, scalable and extensible microbiome data science using QIIME 2**. *Nature Biotechnology* 2019, **37**(8):852-857.

22. Matias Rodrigues JF, Schmidt TSB, Tackmann J, von Mering C: **MAPseq: highly efficient k-mer search with confidence estimates, for rRNA sequence analysis**. *Bioinformatics* 2017, **33**(23):3808-3810.

23. Truong DT, Franzosa EA, Tickle TL, Scholz M, Weingart G, Pasolli E, Tett A, Huttenhower C, Segata N: **MetaPhlAn2 for enhanced metagenomic taxonomic profiling**. *Nature Methods* 2015, **12**(10):902-903.

24. Finlayson I, Davis B, Gavin P, Uh G-R, Whalley D, Själander M, Tyson G: **Improving processor efficiency by statically pipelining instructions**. *ACM SIGPLAN Notices* 2013, **48**(5):33-44.

25. Amiri H, Shahbahrami A: **SIMD programming using Intel vector extensions**. *Journal of*

Parallel and Distributed Computing 2020, **135**:83-100.

26. **Introduction to Intel Advanced Vector Extensions** [https://software.intel.com/content/www/us/en/develop/articles/introduction-to-intel-advanced-vector-extensions.html]

27. Shenhav L, Thompson M, Joseph TA, Briscoe L, Furman O, Bogumil D, Mizrahi I, Pe'er I, Halperin E: **FEAST: fast expectation-maximization for microbial source tracking**. *Nature Methods* 2019, **16**(7):627.

28. Yilmaz P, Parfrey LW, Yarza P, Gerken J, Pruesse E, Quast C, Schweer T, Peplies J, Ludwig W, Glöckner FO: **The SILVA and "all-species living tree project (LTP)" taxonomic frameworks**. *Nucleic acids research* 2014, **42**(D1):D643-D648.

29. SanMiguel AJ, Meisel JS, Horwinski J, Zheng Q, Bradley CW, Grice EA: **Antiseptic Agents Elicit Short-Term, Personalized, and Body Site–Specific Shifts in Resident Skin Bacterial Communities**. *Journal of Investigative Dermatology* 2018, **138**(10):2234-2243.

30. Shaiber A, Willis AD, Delmont TO, Roux S, Chen L-X, Schmid AC, Yousef M, Watson AR, Lolans K, Esen OC: **Functional and genetic markers of niche partitioning among enigmatic members of the human oral microbiome**. *bioRxiv* 2020.

31. Alsalah D, Al-Jassim N, Timraz K, Hong P-Y: **Assessing the Groundwater Quality at a Saudi Arabian Agricultural Site and the Occurrence of Opportunistic Pathogens on Irrigated Food Produce**. *International Journal of Environmental Research and Public Health* 2015, **12**(10):12391-12411.

32. Chai X, Yang Y, Wang X, Hao P, Wang L, Wu T, Zhang X, Xu X, Han Z, Wang Y:

Spatial variation of the soil bacterial community in major apple producing regions of China. *Journal of Applied Microbiology* 2020.

Figures

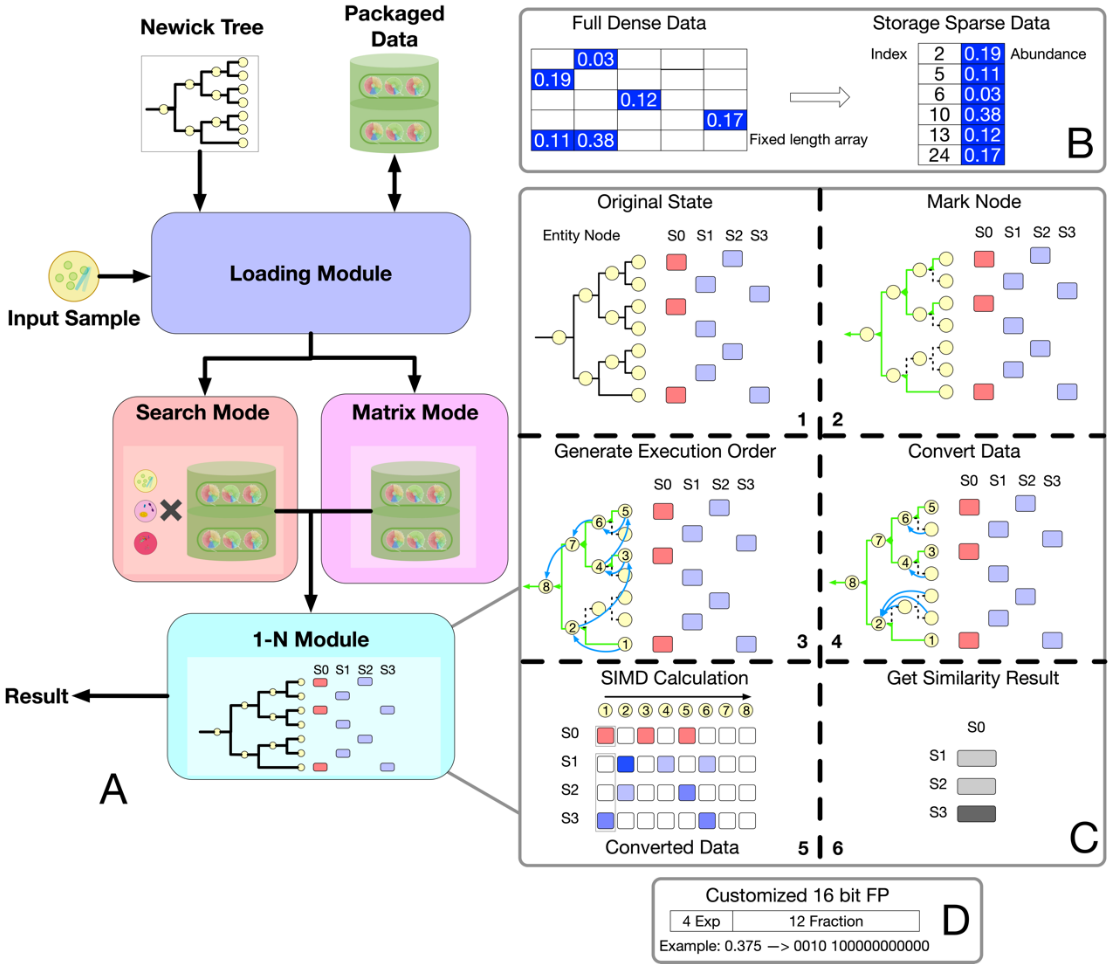

Figure 1

Figure 1. The Meta-Prism 2.0 pipeline with key optimization highlighted. (A) Meta-Prism 2.0 takes taxa abundance as input data, maps data to the phylogenetic tree, and converts data to sparse abundance data for space optimization. Meta-Prism 2.0 organizes data according to search mode or matrix mode, then uses the 1-N module to calculate similarities. (B) Space-saving scheme packages sample data to the sparse format for storage, cuts down both disk and memory usage. For example, when an evolutionary tree has a total of 25 nodes, and one of the samples has 5 non-zero nodes. Dense format will store all nodes in a fixed array, while sparse format will only store the abundance and sum of 5 non-zero nodes. (C) The 1-N module saves resources to the maximum extent by removing redundant nodes without losing their abundances, and fix the execution order for fast 1-against-N sample comparison (1-4), followed by SIMD optimization as a compiler-level optimization (5). The dashed lines indicate branches and nodes to be removed. The black arrows indicate an execution order to be recorded (post-order traversal), and the blue arrows indicate abundance aggregation from those to-be-removed nodes to their ancestors. (D) The similarities are saved in the

format of a customized 16-bit floating-point. Pseudocode about Meta-Prism 2.0 can be accessed from **Supplementary Material 1**.

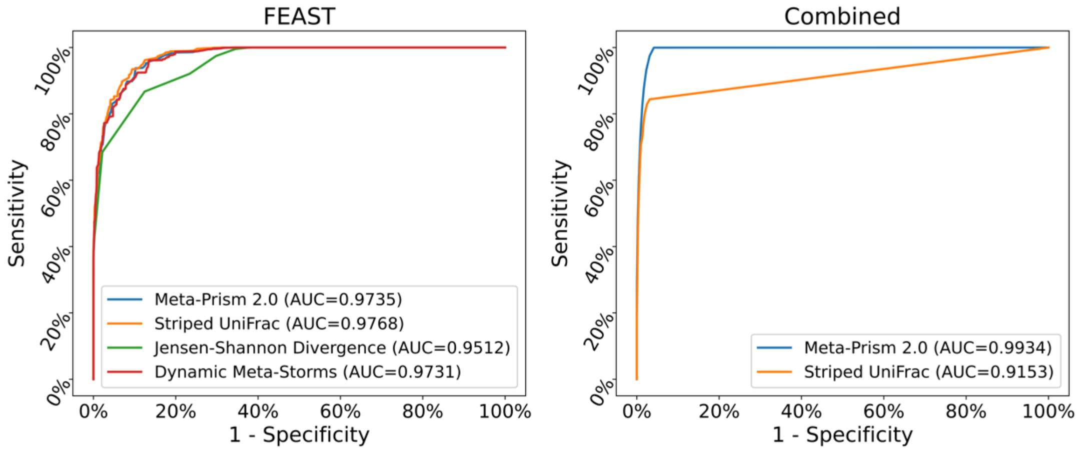

Figure 2

Figure 2. AUC of different methods for sample searches using the FEAST dataset and the Combined dataset. Note that all these methods could complete the analysis in due time and reached good AUC on the FEAST dataset, whereas Jensen-Shannon divergence and Dynamic Meta-Storms can not complete the analysis on the Combined dataset.

Figure 3

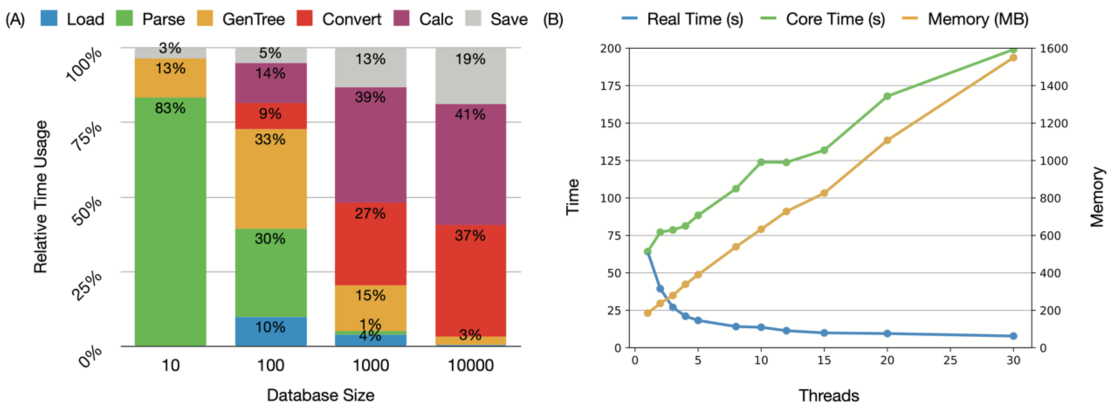

Figure 3. Time usage at different steps and multi-threads performance analysis of Meta-Prism 2.0. (A) Each steps' time usage with variate sample sizes. Load: load data, Save: save matrix result, Parse: load and parse phylogenetic tree, GenOrder generates non-redundant phylogenetic tree (without redundant nodes) in 1-N module, Convert: convert sample data from spare format to dense format for the sample comparison, Calc: 1-against-N sample comparison. A higher proportion of total time was used by Convert and Calc steps when the number of sample pairs increases. (B) Time and memory usage for 10,000 samples' pair-wise similarity calculation using the different numbers of CPU threads. Real-time: the actual time usage of calculation, Core Time: the sum of each CPU cores' time usage.

Figure 4

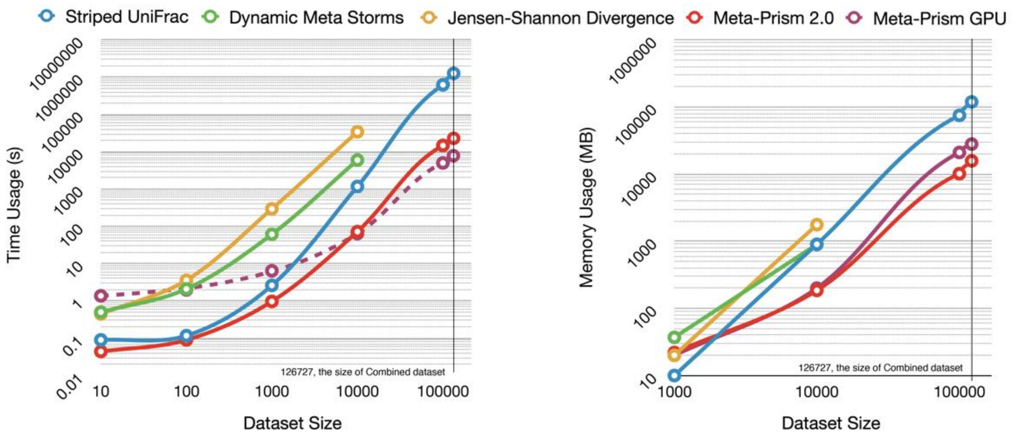

Figure 4. Time and memory usage of samples when calculating similarity matrix for datasets with different numbers of samples. (A) is for time usage comparison, and (B) is for memory usage comparison. In (A), Meta-Prism GPU time usage with dash line is GPU time usage, others are CPU core time usage.

Figure 5

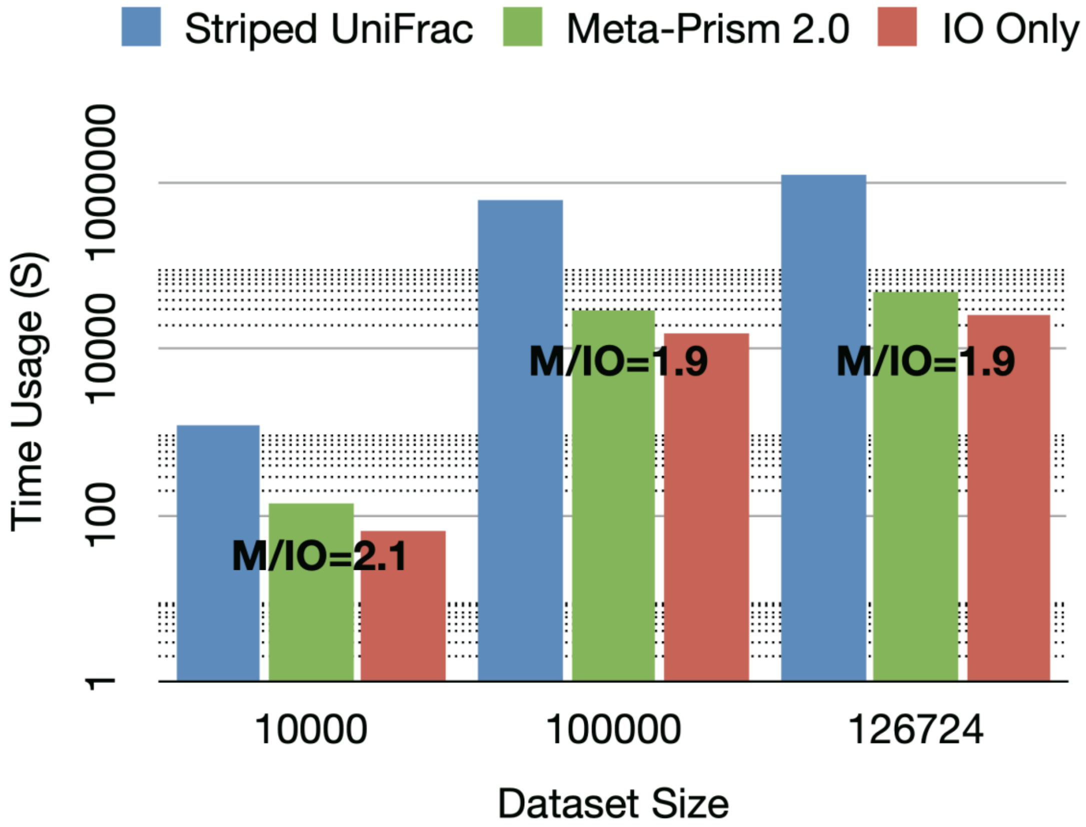

Figure 5. Time usage for different methods and IO Only on datasets with different sizes. “M/IO” is the ratio of time cost of Meta-Prism 2.0 over that of IO Only.

Figure 6

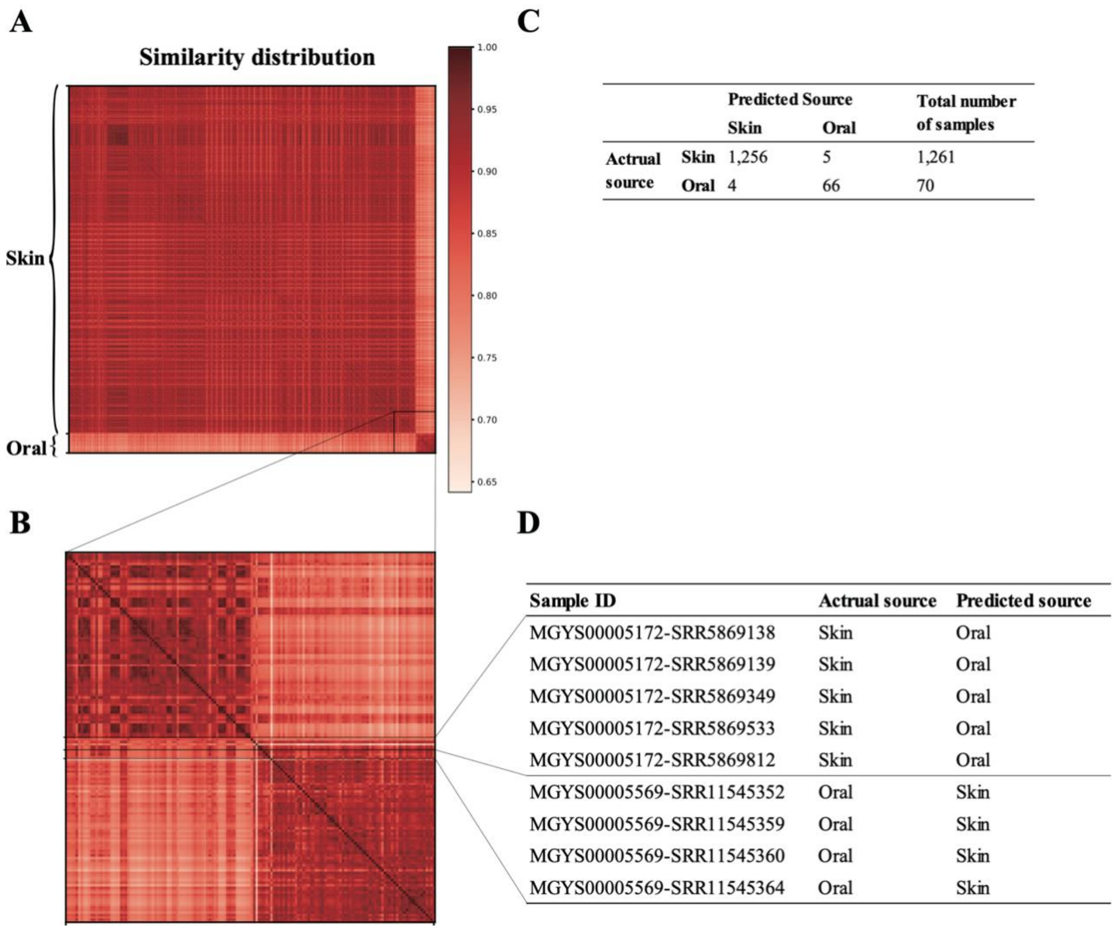

		Predicted Source		Total number of samples
		Skin	Oral	
Actrual source	Skin	1,256	5	1,261
	Oral	4	66	70

Sample ID	Actrual source	Predicted source
MGYS00005172-SRR5869138	Skin	Oral
MGYS00005172-SRR5869139	Skin	Oral
MGYS00005172-SRR5869349	Skin	Oral
MGYS00005172-SRR5869533	Skin	Oral
MGYS00005172-SRR5869812	Skin	Oral
MGYS00005569-SRR11545352	Oral	Skin
MGYS00005569-SRR11545359	Oral	Skin
MGYS00005569-SRR11545360	Oral	Skin
MGYS00005569-SRR11545364	Oral	Skin

Figure 6. Clustering result of human samples from close biomes using similarities calculated by Meta-Prism 2.0. (A) Similarity distribution of 1,331 samples. The samples were successfully clustered into two groups though we did not specify the number of clusters *prior*. (B) Similarity distribution of 9 samples that are not clustered with samples from the same biome (mis-clustered). (C) Confusion matrix and the number of samples within each actual source biome and predicted biome. (D) EBI MGnify study accession, run accession, actual biome source, and predicted biome source of 9 mis-clustered samples.

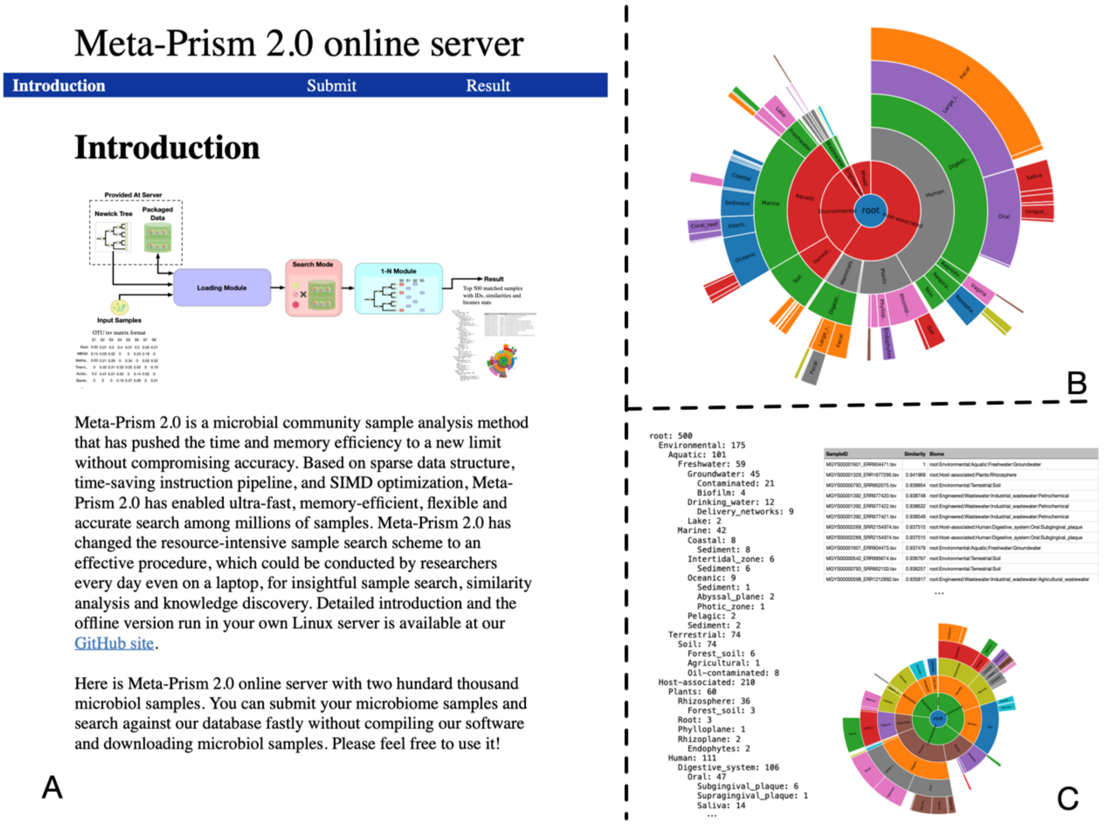

Figure 7. Screenshots for Meta-Prism 2.0 web server. (A) Meta-Prism 2.0 front page. (B) Statistics about sample source biomes for the built-in two hundred thousand microbial samples. (C) An example output consisting of top 500 matched samples' IDs and similarity values in table format, statistics about sample source biomes of top 500 matched samples in text tree and sunburst format.

Tables

Table 1

Table 1. The Combined dataset and FEAST dataset used in this study. Details are provided in **Supplementary Table**.

**Dataset**	**Combined dataset**	**FEAST dataset**
**Top-level biome**	Root	Human gut
**Number of biomes involved**	114	3
**Number of samples**	126,727	10,270
**Number of species**	45,477	5,762
**The average number of species per sample**	411.22	111.05
**Notes**	Selected samples from MGnify database	Selected samples from the FEAST study

Table 2

Table 2. The search results of 11 groundwater samples against Combined dataset.

Count groundwater-related samples in the top 100 matching samples

**Sum of groundwater-related samples:**								
53	58	75	53	38	88	56	79	73

Table 3

Table 3. Average similarities of biomes between 611 sink samples.

	**environmental**	**human skin**	**insecta**	**mammal**	**plants**
**Average Similarity**	0.7886	0.8896	0.7849	0.8478	0.8313

Manuscript (all revisions in red)

Click here to access/download;Manuscript;manuscript_Red.docx

Meta-Prism 2.0: Enabling algorithm and web server for ultra-fast, memory-efficient, and accurate analysis among millions of microbial community samples

Kai Kang[1,2,$], Hui Chong[1,$], Kang Ning[1,*]

[1] *Key Laboratory of Molecular Biophysics of the Ministry of Education, Hubei Key Laboratory of Bioinformatics and Molecular-imaging, Center of AI Biology, Department of Bioinformatics and Systems Biology, College of Life Science and Technology, Huazhong University of Science and Technology, Wuhan 430074, China*

[2] *Center for Quantitative Biology, Academy for Advanced Interdisciplinary Studies, Peking University, Beijing 100871, China*

[$] *These authors contributed equally to this work*

** Corresponding author*

E-mail: ningkang@hust.edu.cn

Abstract

Background

Microbial community samples have been accumulated at a speed faster than ever, with hundreds of thousands of samples been sequenced each year. Mining such a huge amount of multi-source heterogeneous data is becoming an increasingly difficult challenge, so efficient and accurate compare and search of samples are in urgent need: Faced with millions of samples in the data repository, traditional sample comparison and search approaches fall short in speed and accuracy.

Findings

Here we proposed Meta-Prism 2.0, a microbial community sample analysis method that has pushed the time and memory efficiency to a new limit without compromising accuracy. Based on sparse data structure, time-saving instruction pipeline, and SIMD optimization, Meta-Prism 2.0 has enabled ultra-fast, memory-efficient, flexible and accurate search among millions of samples. Meta-Prism 2.0 was put to test on several datasets, with the largest containing one million samples. Results show that Meta-Prism 2.0's 0.00001s per sample pair compare speed and 8GB memory needs for searching against one million samples have made it one of the most efficient sample analysis methods. Additionally, Meta-Prism 2.0 can achieve accuracy comparable with or better than other contemporary methods. Thirdly, Meta-Prism 2.0 can precisely identify the original biome for samples, thus enabling sample source tracking. Finally, we have provided a web server for fast search of microbial community samples online.

Conclusions

In summary, Meta-Prism 2.0 has changed the resource-intensive sample search scheme to an effective procedure, which could be conducted by researchers every day even on a laptop, for insightful sample search, similarity analysis and knowledge discovery. Meta-Prism 2.0 can be accessed at: https://github.com/HUST-NingKang-Lab/Meta-Prism-2.0, and the web server can be accessed at: https://hust-ningkang-lab.github.io/Meta-Prism-2.0/.

Introduction

Microbial communities have asserted great influences on healthcare, environment, and industry[1-4]. As such, an increasing number of projects have been conducted on microbial communities around the world, such as those from the "Human Microbiome Project"[1, 2] and the "Earth Microbiome Project"[3, 4]. Mining this massive amount of samples has already discovered knowledge about the microbial community and their effects on the environment and human health[5, 6], providing an opportunity to study the hidden evolution and ecology patterns among microbial communities.

A microbial community sample (also referred to as the sample) is represented by the hierarchically structured taxa (species, genus, families, etc.) and their relative abundances (also referred to as the community structure), and these species are functioning in concert to maintain stability and adapt to the specific environments (also referred to as the niches or biomes) where the microbial community is living. These samples' community structures are often associated with the biomes and a variety of characteristics of the biomes. For example, the community structures of the human gut microbiome have been linked to multiple aspects of human life, such as health[6, 7], early development[8], immigration[9], and pregnancy[10]. Thus, there is a large amount of hidden information in the community structures and remains to be discovered. These challenges in current microbiome researches are calling fast community-level comparison and search among the rapidly accumulating number of microbial communities.

There are already methods that existed for comparison and search of samples. The distance-based methods are the first batches designed for the purpose, whose primary strategy is to compare the similarity or distance between two samples. The simplest distance-based method is the Jensen-Shannon Divergence (JSD) measurement[11], which only considered species abundances in the community. More advanced distance-based methods considered both species abundances and their phylogenetic relationships. For example, UniFrac[12] is a typical distance-based method, which firstly maps their respective sets of taxon abundances on the phylogenetic tree, and secondly traverses the tree and executes operation at each node (each representing a taxon on the

phylogenetic tree) to calculate their similarity. Fast UniFrac[13] and Meta-Storms[14] optimized such a procedure by changing tree traversal to array loop. Striped UniFrac[15] further optimized matrix similarity comparison by reorganizing samples. Dynamic Meta-Storms enables species-level accuracy by introducing virtual nodes[16]. Previously, we designed Meta-Prism 1.0, a fast and accurate microbial community sample search tool[17]. Meta-Prism 1.0 generates an index to rapidly select samples with similar biome and top phylum for comparison. Furthermore, Meta-Prism 1.0 uses GPU to accelerate comparison. However, given that more than a million community samples have already been deposited into public databases[18, 19], state-of-the-art methods including Meta-Prism 1.0 face difficulties in comparison and searching among these samples, while rendering knowledge discovery from samples formidable. Additionally, microbial community samples' data are very sparse. These methods use fixed-length arrays to save abundances with lengths equals to entities number of the phylogenetic tree, wasting a considerable amount of memory. They also spend much time operating on these empty nodes.

To solve the large-scale microbial community sample search problem, we have redesigned and updated Meta-Prism to its second version (Meta-Prism 2.0). Facing the large and sparse characteristics of microbial samples, we adopted a special sparse storage format and a fast 1-N calculation method. This greatly reduces memory usage and time consumption. As the computing and storage efficiency increased, it adds similarity matrix calculation function to analyze more than 100,000 samples' beta diversity. When searching samples, due to the efficiency improvement, Meta-Prism 2.0 no longer needs to generate the index system to select high-probability subsets for similarity comparison, but conduct an exhaustive search against the entire database. Thus it has higher flexibility (when searching among customized datasets) and robustness than Meta-Prism 1.0. More importantly, with these improvements of efficiency and space, Meta-Prism 2.0 now can deal with one million or even more microbial community samples and is one of the fastest microbial community sample search methods to date.

Using several datasets including the largest one containing a million samples, we demonstrated that it can achieve at least 20 times speed-up compared to the contemporary approach (e.g., Meta-Prism 1.0 and Striped UniFrac), and Meta-Prism 2.0 is the only method that could handle the search against a million samples. The memory utilization is also very efficient: Compared with other methods including JSD, Striped UniFrac, and Dynamic Meta Storms, when analyzing dataset beta diversity which size exceeds 10,000, Meta-Prism 2.0 can at least save 80% of memory space needed. Though we have saved time and memory by magnitudes, the accuracy is not compromised. For example, Meta-Prism 2.0 obtained 0.99 AUC in distinguishing samples from different biomes for more than one hundred thousand samples [20]. Meta-Prism 2.0 has changed the traditional computational resource-intensive sample search

to a cheap and effective procedure that could be conducted by researchers every day, for the discovery of intricate relationships among samples. Meta-Prism 2.0 can be accessed at: https://github.com/HUST-NingKang-Lab/Meta-Prism-2.0. And the fast and accurate microbial community sample search could also be experienced on the web server at: https://hust-ningkang-lab.github.io/Meta-Prism-2.0/.

Methods

Meta-Prism 2.0 calculates similarities between microbial communities using two calculation modes: search mode and matrix mode. The search mode takes two datasets (query and target) as input and then outputs each query sample's top N similar matches in the target dataset. The matrix mode takes a dataset as input and outputs a pair-wise similarity matrix for all samples in the dataset (**Figure 1A**). These datasets can be produced by commonly used tools such as QIIME[21], MAPseq[22], and MetaPhlAn[23].

Meta-Prism 2.0 has unlocked several key computational techniques for efficient comparison (**Figure 1**): Firstly, it utilizes a sparse data structure to cut down the memory and disk usage (**Figure 1B**). Secondly, to further cut down the memory usage, Meta-Prism 2.0 only stores essential taxa (taxa appeared in query samples) of the phylogenetic tree and abundances for similarity calculation. (**Figure 1C (1-4)**). Thirdly, to cut down the time usage, Meta-Prism 2.0 discards redundant execution before diving into similarity calculation (**Figure 1C (3)**). Fourthly, Meta-Prism 2.0 utilizes a fast 1-N compare module to enable further accelerations through the instruction pipeline[24] and single instruction multiple data (SIMD) optimization (**Figure 1C (5)**). Last but not least, Meta-Prism 2.0 utilizes a customized 16-bit floating-point to store the similarity matrix in a memory-saving manner (**Figure 1D**).

Space-saving data format

Each microbial community sample consists of classified taxa and their relative abundances. Meta-Prism 2.0 will find the taxa in the phylogenetic tree. For the representation of a single microbial community sample, most of the phylogenetic tree nodes are redundant. Unlike other methods that store in fixed-length arrays, Meta-Prism 2.0 stores taxonomic abundance data in a sparse format, that is, uses a variable-length list to store only relatively abundant non-zero nodes: the data includes their relative abundance and the node id of phylogenetic tree (**Figure 1B**). When calculating similarities, Meta-Prism 2.0 converts sparse data back to dense data (Convert step, **Figure 1C (5),** Algorithm 5 in **Supplementary Materials**). The sparse data structure is applied to disk storage and memory cache to reduce space utilization globally.

The storage scheme is further optimized at the step of similarity result storage. To store similarity results for a sample pair, we designed 16 bits floating-point with four exponential bits and 12 mantissa bits. Considering that the similarities are between zero and one, we removed two sign bits of exponent and mantissa to increase the gamut and precision of the floating-point (**Figure 1D**).

Similarity measurement independent of data type and sequencing depth

Our similarity is proposed to measure similarity between a pair of community samples, independent of data type and sequencing depth [14](section 2.2.2). The details of similarity calculation are shown at Algorithm 6 in **Supplementary Materials** with the default execution order being generated from Algorithm 2 in **Supplementary Materials** with all node marked**.** To calculate the similarity of the two samples (n=1 in pseudocode), we will recursively calculate the similarity of the relative abundance of the two samples at each node and deduce, and then multiply the relative abundance remained by one minus the evolutionary distance and send it to the parent node.

Fast 1-N sample comparison

We further optimized the time usage to the minimum extend through a fixed execution order and SIMD[25]. Current methods traverse phylogenetic tree (with redundant nodes) and execute operation during similarity calculation (Striped Unifrac calculates the difference at each node, divides it by the branch distance. Meta-Storm and Dynamic Meta-Storm accumulates the similarity at each node and passes the residual abundance to the parent node. Meta-Prism 1.0 calculates the difference at each node, divides it by 1 minus the evolutionary distance and passes it to the parent node), wasting time on redundant operations[15-17]. In Meta-Prism 1.0, for nodes with no abundance (which also are the vast majority), the system will calculate at variables value 0, without any influence on the result. Some nodes have only the relative abundance of one sample, and the most of the system's calculations on them are invalid which are equivalent to multiplying their abundances by one minus evolutionary distance and passing these to their parent nodes. To save the time wasted on such operations, when Meta-Prism 2.0 calculates 1-N comparison (S_0 against S_n), it will only consider nodes that are abundant in S_0 (marked node) and generate a fixed execution order based on them (GenOrder step, **Figure 1C (2 and 3),** Algorithm 2 in **Supplementary Materials**). To deal with nodes that are only contain abundant in S_n, Meta-Prism 2.0 will multiply the abundance on these nodes by the cumulative evolutionary distance factor and send them to the nearest labelled parent nodes (Convert step, **Figure 1C (4),** Algorithm 5 in **Supplementary Materials**). Which node to send and the factor value is calculated at Algorithm 4 in **Supplementary Materials**. The fixed execution order without branches and jumps will lead the CPU to use the instruction pipeline. Additionally, Meta-Prism 2.0 is implemented based on SIMD AVX intrinsic[26], thus can execute operations to compare a sample S0 with other multiple samples (referred to as Sn) at the same time (SimilarityCalculation step, **Figure 1C (5),** Algorithm 6 in **Supplementary Materials**).

We packaged these steps as the "1-N module", and use the module to execute fast comparison and search.

Results

Materials and execution environments used for evaluation

Through manual curation from the EBI MGnify database[18], we obtained a dataset consists of 126,727 microbial community samples belonging to 114 different biomes, defined as the Combined dataset. We also obtained a dataset that consists of 10,270 samples belonging to three biomes: Fecal, Human, and Mixed, which have been used in the FEAST study[27], defined as the FEAST dataset (**Table 1**). To evaluate Meta-Prism 2.0's speed and memory efficiency on the scale of one million samples, we synthesized a dataset with 1,000,010 samples based on the Combined dataset. All samples from these three datasets are accessible from https://github.com/HUST-NingKang-Lab/Meta-Prism-2.0. We used SILVA 132 LTPs132 SSU phylogenetic tree[28] in all experiments included in this study.

Striped UniFrac, Dynamic Meta-Storms, Meta-Prism 2.0 were compiled by GCC 4.8.5 and ran on CentOS 6.7 with Intel(R) Xeon(R) CPU E5-2678 v3 @ 2.50GHz and 252GB memory. The Jensen-Shannon divergence was calculated utilizing Python 3.7.3 and SciPy 1.4.1 and ran on the same CentOS device. The executable Meta-Prism 2.0 steps' time usage was compiled by clang-1100.0.33.16, and evaluated by Xcode11.5 Instruments Time Profiler, ran on macOS 10.15 with Intel(R) Core (TM) i7-9750H and 32GB memory. Meta-Prism GPU version was compiled by NVCC 10.1 and ran on RTX 2080Ti.

Accuracy evaluation

We assessed the search accuracy of different methods in the context of source tracking, namely by checking the consistency of the predicted biomes and query samples' actual biomes. This evaluation is based on the realization that the microbial communities collected from the same biome always share similar patterns in their taxonomical structures and relative abundances[20, 27]. Specifically, we used simple cross-validation for the evaluation, based on searching 12.5% randomly chosen samples (considered as query dataset) against the rest samples (considered as target dataset). For each query sample, we selected the top 100 most similar target samples. The similarity of these samples is then summed by biome and normalized by dividing by the total number of samples in the source dataset for each biome. After the resulting values are normalized, it is the probability that the test sample belongs to each biome.

The evaluation performances are shown in **Figure 2**. The varying classification threshold that generates different sensitivities and specificities range from 0.01 to 1

with a fixed step size of 0.01. On the FEAST dataset, each method predicted biome for testing samples according to the biomes included in the source dataset (Fecal, Human, and Mixed). Distance-based phylogenetic tree approaches (Meta-Prism 2.0, Striped UniFrac, and Dynamic Meta-Storms) showed similarly good performance, while Jensen-Shannon Divergence (JSD) obtained a lower AUC of 0.9512. On the Combined dataset, each method predicted biome for testing samples according to 114 biomes included in the source dataset (87.5% of the Combined dataset). JSD and Dynamic Meta-Storms cannot finish the calculation within an acceptable time (10 days). We only compared Meta-Prism 2.0 and Striped UniFrac. Meta-Prism obtained a higher AUC result of 0.9934, while Striped UniFrac's AUC result was 0.9153.

Computational speed assessment

The time and memory efficiency are the most profound advantage of Meta-Prism 2.0. We first assessed Meta-Prism 2.0's speed based on using datasets with different dataset sizes and using different numbers of CPU threads (**Figure 3**). The setting was matrix mode, which takes one dataset as input, then calculates all sample pairs' similarities, and the output is a similarity matrix. The time cost is split into several parts according to computational steps. Our 1-N module adds GenOrder and Convert steps, which increase linearly and quadratically with the increase of dataset size, respectively.

We also evaluated Meta-Prism 2.0 performance on a dataset with one million samples (see **Materials** for details). Meta-Prism 2.0 can efficiently package one million samples into a 369 MB-sized file for storage and load them within 27 seconds. We transferred the whole workload to a laptop and searched 100 samples against this dataset with a single CPU thread. It cost 324.96 seconds (less than 6 minutes) CPU time to complete the search using only 6.9 GB memory. So far as we know, Meta-Prism 2.0 is the only method that could handle the search against a million samples.

Computational speed comparison

We further selected datasets with different dataset sizes (10, 100, 1,000, 10,000, 100,000 and 126,727) from the Combined dataset to compared different methods. The setting is again matrix mode. We compared time and memory usage of Striped UniFrac, Dynamic Meta Storms, JSD, Meta-Prism GPU, and Meta-Prism 2.0. Meta-Prism GPU is the only method that uses GPU for calculation, and we considered real-time usage for the measurement. In comparison, we took CPU core time usage as other methods' time usage. JSD and Meta-Storms cannot calculate the similarity matrix when dataset size $\geq$ 10,000 within an acceptable time (10 days).

Results show that Meta-Prism 2.0 could achieve superior performance on both time usage and memory usage (**Figure 4)**. Specifically, when the dataset sizes are no more than one thousand, Meta-Prism 2.0 used a similar core time compared with Striped UniFrac **(Figure 4A)**. When dataset size became more extensive, the performance gap

between Meta-Prism 2.0 and Striped UniFrac became larger. When calculating the similarity matrix for the Combined dataset (generating 126,727 × 126,727 similarity matrix), Meta-Prism 2.0 was 55 times faster than Striped UniFrac. Meta-Prism GPU's real-time usage was smaller than Meta Prism 2.0 core time usage. However, when Meta-Prism 2.0 uses 3 CPU cores or more, it will be faster than Meta-Prism GPU.

Meta-Prism 2.0's memory usage was only 11.1% of Striped UniFrac's when calculating the similarity matrix for the Combined dataset with more than 100,000 samples. The utilization of customized 16 bits floating point was the key reason because it can efficiently store similarity matrix, which is the largest storage burden that increases quadratically when the dataset size increases.

Wondering how far is the speed of Meta-Prism 2.0 to the theoretical lower bound for the sample search, we took IO Only as the lower bound for sample search, in which we only record the time used for loading data and writing matrix calculation results (**Figure 5**). The result shows that the time costs of Meta-Prism 2.0 is only two times of IO Only, while magnitude smaller than those of Striped UniFrac.

Real data applications

Meta-Prism 2.0 can precisely identify the biome for samples of unknown origin, thus enabling the source tracking of samples. For example, it enables accurate differentiation of samples from close biomes such as "human skin" and "human oral" (the first application), identification of the biome for samples with unclear origin (the second application), as well as detection of microbial contamination (the third application). We published these applications' workflow at Code Ocean for researchers to track and reproduce (https://codeocean.com/capsule/3103931).

Firstly, we tested Meta-Prism 2.0's ability to accurately differentiating samples from close biomes. We obtained 1,261 skin metagenomic samples (MGYS00005172)[29] and 70 oral metagenomic samples (MGYS00005569)[30] from MGnify[18]. We used Meta-Prism 2.0 to calculate the similarities matrix of 1,331 samples on a laptop, which cost only 3.75 seconds and 11MB of memory. We also clustered samples based on their similarities by using affinity propagation from Scikit-learn (version 0.20.3). The samples were successfully clustered into two groups whose sizes are 1,260 and 71 (**Figure 6**). Within 1,331 samples, only nine samples (five skin samples and four oral samples) were miss-clustered, proving Meta-Prism 2.0's ability to fast and accurately differentiate samples from close biomes.

Secondly, we evaluated the performance of Meta-Prism 2.0 on source tracking environmental samples from less-studied biomes, based on searching 11 groundwater samples curated from Saudi Arabian (MGYS00001601)[31] against the combined dataset. The biome "groundwater" is less studied, with a handful of samples in the

combined dataset (MGYS00005245). Results show that Meta-Prism 2.0 could successfully identify source-related biomes for samples from “groundwater”. Within the top 100 most similar community samples for each “groundwater” query sample, there are on average 64 groundwater-related samples (from “root-Environmental-Terrestrial”, “root-Environmental-Aquatic”, “root-Engineered-Wastewater” and “root-Host-associated-Plants”) for each query sample **(Table 2)**. Nevertheless, there is no “groundwater” sample in the top 100 similar samples searched by Meta-Prism 2.0, since “groundwater” samples in the combined dataset are curated from New Zealand, which is in nature drastically different from our query samples. The result suggests that the geographic origins also influence the community structures, which was already confirmed by previous studies[32].

Finally, we evaluated the Meta-Prism 2.0’s power in detecting microbial contamination. We investigated the contamination of indoor house surfaces community by selecting 611 samples from indoor house surfaces in Chicago as query samples and searching against 6,285 samples (899+3,773+721+692+200 from “human skin”, “environmental”, “mammal”, “plants”, and "insecta", respectively). The analysis costs only 6.16 seconds to complete. Our results show that the most closed biome source for indoor house surface samples is “human skin” (average similarity 0.889), indicating a large proportion of microbial community contamination from human skin, which agrees with previous analyses by SourceTracker[20] and FEAST[27] (**Table 3**). Again, it proved the ability of Meta-Prism 2.0 for accurate and fast microbial community contamination screening.

Web server for fast and accurate microbial community sample search

For easy use of Meta-Prism 2.0, we also designed an online web server for Meta-Prism 2.0 (**Figure 7**), with a pre-compiled Meta-Prism 2.0 executable file and a built-in dataset contains more than 0.2 million microbiome samples. This dataset including major categories such as digestive system, aquatic, and soil, as well as sub-categories such as oil-contaminated clay, thermal springs sediment, and bioreactor for biological phosphorus removal. The high efficiency of Meta-Prism 2.0 enables any query against this huge dataset to be completed within one second, with high accuracy.

Discussions and Conclusion

In this work, we designed Meta-Prism 2.0 as an ultrafast and memory-efficient approach to analysis against millions of microbial community samples. The sample compare and search problems have encountered great difficulties when faced with millions of samples, primarily due to the computational space and time limitations. Meta-Prism 2.0 was designed based on sparse data structure, time-saving instruction pipeline, SIMD optimization, and exhaustive search strategy, enabling flexible, ultra-fast, memory-efficient, and added beta diversity analysis function.

Results show that compared to the current methods serving the same purpose, Meta-Prism 2.0 is at least 20 times faster, while memory cost is at least four times smaller. Additionally, the speed of Meta-Prism 2.0 is close to the lower bound of the search. Furthermore, according to our experiment, Meta-Prism 2.0 can even store all samples' community structure from the EBI MGnify dataset (300,000 in total as of Oct. 2020) on a laptop and searching against it at an unprecedented speed. Finally, we provided several concrete examples, which have proven the effectiveness and utility of Meta-Prism 2.0 in knowledge discovery. And the fast and accurate microbial community sample search could also be experienced on the web server, on which any query against this huge dataset to be completed within one second, with high accuracy

In summary, Meta-Prism 2.0 can perform searches among millions of samples with low memory cost and fast speed, enabling source tracking and knowledge discovery from sample mining at a massive scale. Meta-Prism 2.0 has optimized the traditional resource-intensive sample search and similarity matrix calculation into an affordable and effective procedure that researchers could conduct every day for mining intricate relationships among samples and discover previously unknown knowledge.

Availability of Supporting Source Code and Requirements

Project name: Meta-Prism 2.0
Project home page: https://hust-ningkang-lab.github.io/Meta-Prism-2.0/
GitHub repository: https://github.com/HUST-NingKang-Lab/Meta-Prism-2.0
Operating systems: Platform independent
Programming language: C++
Other requirements: Compiler support C++11
License: GPL-3.0 License
RRID: SCR_021836
bio.tools ID: Meta-Prism 2.0
Reproducible publication: https://codeocean.com/capsule/3103931

Availability of Data

Our code is published at https://github.com/HUST-NingKang-Lab/Meta-Prism-2.0. All the datasets curated for this study are available at this project's "Releases".

Competing of Interest

The authors declare that they have no competing interests.

Acknowledgments

The authors would like to thank Yi Zhan for insightful discussions about the project.

Funding

This work was partially supported by National Natural Science Foundation of China grant 32071465, 31871334, and 31671374, Ministry of Science and Technology's grant 2018YFC0910502, and National Undergraduate Training Program for Innovation and Entrepreneurship of China (Program No. 201910487071).

Authors' Contributions

K.N. conceived and supervised this study. K.K. designed and developed Meta-Prism 2.0 software and web server. K.K and H.C. tested Meta-Prism 2.0. K.K, H.C., and K.N. wrote the manuscript. All authors read and approved the final manuscript.

Additional Files

Supplementary Material 1: Pseudocode about Meta-Prism 2.0.
Supplementary Table 1: Detail information of Combined dataset and FEAST dataset.

References

1. Integrative HMPRNC: **The Integrative Human Microbiome Project: dynamic analysis of microbiome-host omics profiles during periods of human health and disease**. *Cell Host Microbe* 2014, **16**(3):276-289.
2. Turnbaugh PJ, Ley RE, Hamady M, Fraser-Liggett CM, Knight R, Gordon JI: **The human microbiome project**. *Nature* 2007, **449**(7164):804-810.
3. Gilbert JA, Jansson JK, Knight R: **The Earth Microbiome project: successes and aspirations**. *BMC Biol* 2014, **12**:69.
4. Thompson LR, Sanders JG, McDonald D, Amir A, Ladau J, Locey KJ, Prill RJ, Tripathi

A, Gibbons SM, Ackermann G *et al*: **A communal catalogue reveals Earth's multiscale microbial diversity**. *Nature* 2017, **551**(7681):457-463.

5. Dominguez-Bello MG, De Jesus-Laboy KM, Shen N, Cox LM, Amir A, Gonzalez A, Bokulich NA, Song SJ, Hoashi M, Rivera-Vinas JI: **Partial restoration of the microbiota of cesarean-born infants via vaginal microbial transfer**. *Nature medicine* 2016, **22**(3):250.

6. Thomas S, Izard J, Walsh E, Batich K, Chongsathidkiet P, Clarke G, Sela DA, Muller AJ, Mullin JM, Albert K: **The host microbiome regulates and maintains human health: a primer and perspective for non-microbiologists**. *Cancer research* 2017, **77**(8):1783-1812.

7. Zeller G, Tap J, Voigt AY, Sunagawa S, Kultima JR, Costea PI, Amiot A, Bohm J, Brunetti F, Habermann N *et al*: **Potential of fecal microbiota for early-stage detection of colorectal cancer**. *Mol Syst Biol* 2014, **10**:766.

8. Bäckhed F, Roswall J, Peng Y, Feng Q, Jia H, Kovatcheva-Datchary P, Li Y, Xia Y, Xie H, Zhong H: **Dynamics and stabilization of the human gut microbiome during the first year of life**. *Cell host & microbe* 2015, **17**(5):690-703.

9. Vangay P, Johnson AJ, Ward TL, Al-Ghalith GA, Shields-Cutler RR, Hillmann BM, Lucas SK, Beura LK, Thompson EA, Till LM: **US immigration westernizes the human gut microbiome**. *Cell* 2018, **175**(4):962-972. e910.

10. Koren O, Goodrich JK, Cullender TC, Spor A, Laitinen K, Bäckhed HK, Gonzalez A, Werner JJ, Angenent LT, Knight R: **Host remodeling of the gut microbiome and**

metabolic changes during pregnancy. *Cell* 2012, **150**(3):470-480.

11. Lin J: **Divergence measures based on the Shannon entropy**. *IEEE Transactions on Information theory* 1991, **37**(1):145-151.

12. Lozupone C, Knight R: **UniFrac: a New Phylogenetic Method for Comparing Microbial Communities**. *Applied and Environmental Microbiology* 2005, **71**(12):8228-8235.

13. Hamady M, Lozupone C, Knight R: **Fast UniFrac: facilitating high-throughput phylogenetic analyses of microbial communities including analysis of pyrosequencing and PhyloChip data**. *The ISME journal* 2010, **4**(1):17-27.

14. Su X, Xu J, Ning K: **Meta-Storms: efficient search for similar microbial communities based on a novel indexing scheme and similarity score for metagenomic data**. *Bioinformatics* 2012, **28**(19):2493-2501.

15. McDonald D, Vázquez-Baeza Y, Koslicki D, McClelland J, Reeve N, Xu Z, Gonzalez A, Knight R: **Striped UniFrac: enabling microbiome analysis at unprecedented scale**. *Nature Methods* 2018, **15**(11):847-848.

16. Jing G, Zhang Y, Yang M, Liu L, Xu J, Su X: **Dynamic Meta-Storms enables comprehensive taxonomic and phylogenetic comparison of shotgun metagenomes at the species level**. *Bioinformatics* 2019, **36**(7):2308-2310.

17. Zhu M, Kang K, Ning K: **Meta-Prism: Ultra-fast and highly accurate microbial community structure search utilizing dual indexing and parallel computation**. *Briefings in Bioinformatics* 2020, **00**(December 2019):1-11.

18. Mitchell AL, Almeida A, Beracochea M, Boland M, Burgin J, Cochrane G, Crusoe MR,

Kale V, Potter SC, Richardson LJ: **MGnify: the microbiome analysis resource in 2020**. *Nucleic acids research* 2020, **48**(D1):D570-D578.

19. Coordinators NR: **Database resources of the National Center for Biotechnology Information**. *Nucleic Acids Res* 2016, **44**(D1):D7-19.

20. Knights D, Kuczynski J, Charlson ES, Zaneveld J, Mozer MC, Collman RG, Bushman FD, Knight R, Kelley ST: **Bayesian community-wide culture-independent microbial source tracking**. *Nature methods* 2011, **8**(9):761-763.

21. Bolyen E, Rideout JR, Dillon MR, Bokulich NA, Abnet CC, Al-Ghalith GA, Alexander H, Alm EJ, Arumugam M, Asnicar F *et al*: **Reproducible, interactive, scalable and extensible microbiome data science using QIIME 2**. *Nature Biotechnology* 2019, **37**(8):852-857.

22. Matias Rodrigues JF, Schmidt TSB, Tackmann J, von Mering C: **MAPseq: highly efficient k-mer search with confidence estimates, for rRNA sequence analysis**. *Bioinformatics* 2017, **33**(23):3808-3810.

23. Truong DT, Franzosa EA, Tickle TL, Scholz M, Weingart G, Pasolli E, Tett A, Huttenhower C, Segata N: **MetaPhlAn2 for enhanced metagenomic taxonomic profiling**. *Nature Methods* 2015, **12**(10):902-903.

24. Finlayson I, Davis B, Gavin P, Uh G-R, Whalley D, Själander M, Tyson G: **Improving processor efficiency by statically pipelining instructions**. *ACM SIGPLAN Notices* 2013, **48**(5):33-44.

25. Amiri H, Shahbahrami A: **SIMD programming using Intel vector extensions**. *Journal of*

Parallel and Distributed Computing 2020, **135**:83-100.

26. **Introduction to Intel Advanced Vector Extensions** [https://software.intel.com/content/www/us/en/develop/articles/introduction-to-intel-advanced-vector-extensions.html]

27. Shenhav L, Thompson M, Joseph TA, Briscoe L, Furman O, Bogumil D, Mizrahi I, Pe'er I, Halperin E: **FEAST: fast expectation-maximization for microbial source tracking**. *Nature Methods* 2019, **16**(7):627.

28. Yilmaz P, Parfrey LW, Yarza P, Gerken J, Pruesse E, Quast C, Schweer T, Peplies J, Ludwig W, Glöckner FO: **The SILVA and "all-species living tree project (LTP)" taxonomic frameworks**. *Nucleic acids research* 2014, **42**(D1):D643-D648.

29. SanMiguel AJ, Meisel JS, Horwinski J, Zheng Q, Bradley CW, Grice EA: **Antiseptic Agents Elicit Short-Term, Personalized, and Body Site–Specific Shifts in Resident Skin Bacterial Communities**. *Journal of Investigative Dermatology* 2018, **138**(10):2234-2243.

30. Shaiber A, Willis AD, Delmont TO, Roux S, Chen L-X, Schmid AC, Yousef M, Watson AR, Lolans K, Esen OC: **Functional and genetic markers of niche partitioning among enigmatic members of the human oral microbiome**. *bioRxiv* 2020.

31. Alsalah D, Al-Jassim N, Timraz K, Hong P-Y: **Assessing the Groundwater Quality at a Saudi Arabian Agricultural Site and the Occurrence of Opportunistic Pathogens on Irrigated Food Produce**. *International Journal of Environmental Research and Public Health* 2015, **12**(10):12391-12411.

32. Chai X, Yang Y, Wang X, Hao P, Wang L, Wu T, Zhang X, Xu X, Han Z, Wang Y:

Spatial variation of the soil bacterial community in major apple producing regions of China. *Journal of Applied Microbiology* 2020.

Figures

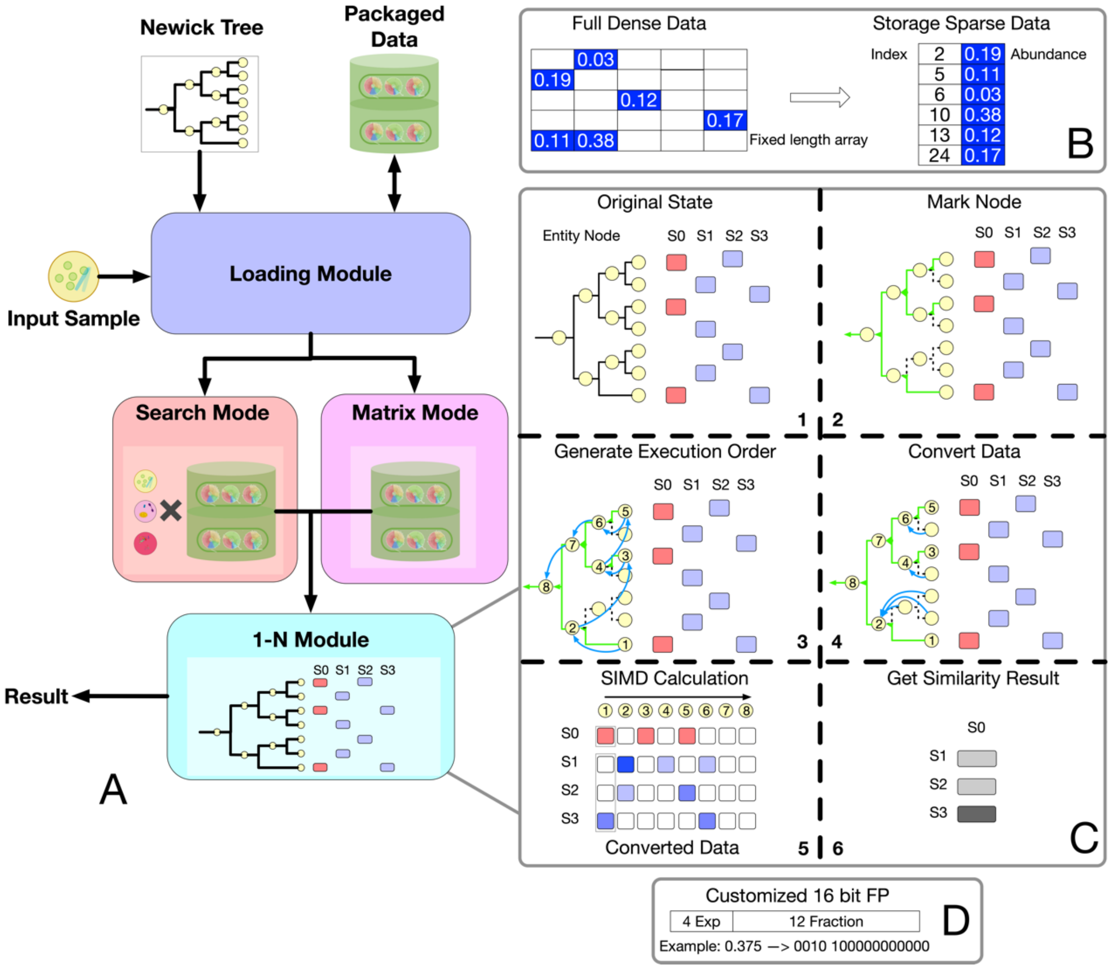

Figure 1

Figure 1. The Meta-Prism 2.0 pipeline with key optimization highlighted. (A) Meta-Prism 2.0 takes taxa abundance as input data, maps data to the phylogenetic tree, and converts data to sparse abundance data for space optimization. Meta-Prism 2.0 organizes data according to search mode or matrix mode, then uses the 1-N module to calculate similarities. (B) Space-saving scheme packages sample data to the sparse format for storage, cuts down both disk and memory usage. For example, when an evolutionary tree has a total of 25 nodes, and one of the samples has 5 non-zero nodes. Dense format will store all nodes in a fixed array, while sparse format will only store the abundance and sum of 5 non-zero nodes. (C) The 1-N module saves resources to the maximum extent by removing redundant nodes without losing their abundances, and fix the execution order for fast 1-against-N sample comparison (1-4), followed by SIMD optimization as a compiler-level optimization (5). The dashed lines indicate branches and nodes to be removed. The black arrows indicate an execution order to be recorded (post-order traversal), and the blue arrows indicate abundance aggregation from those to-be-removed nodes to their ancestors. (D) The similarities are saved in the

format of a customized 16-bit floating-point. Pseudocode about Meta-Prism 2.0 can be accessed from **Supplementary Material 1**.

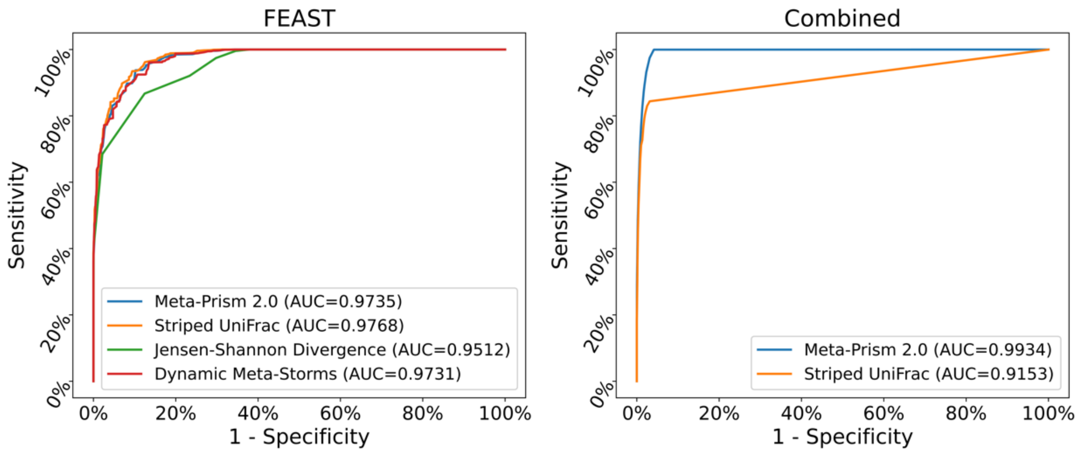

Figure 2

Figure 2. AUC of different methods for sample searches using the FEAST dataset and the Combined dataset. Note that all these methods could complete the analysis in due time and reached good AUC on the FEAST dataset, whereas Jensen-Shannon divergence and Dynamic Meta-Storms can not complete the analysis on the Combined dataset.

Figure 3

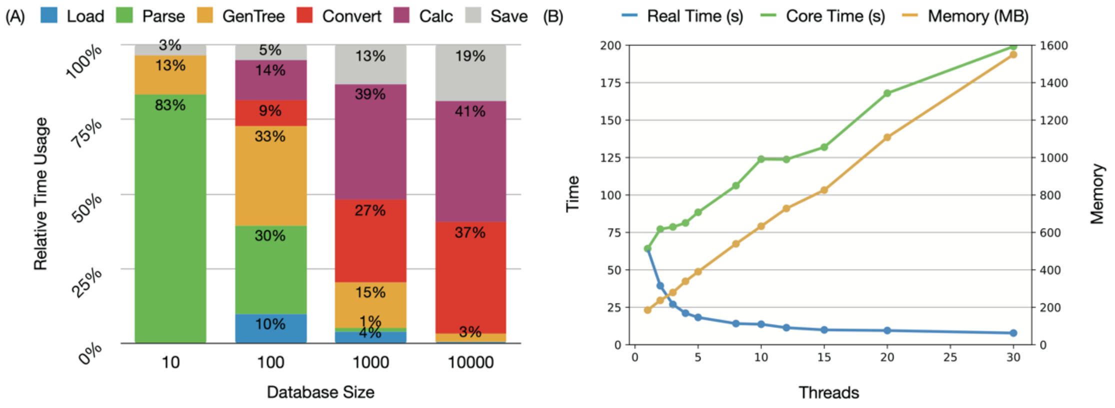

Figure 3. Time usage at different steps and multi-threads performance analysis of Meta-Prism 2.0. (A) Each steps' time usage with variate sample sizes. Load: load data, Save: save matrix result, Parse: load and parse phylogenetic tree, GenOrder generates non-redundant phylogenetic tree (without redundant nodes) in 1-N module, Convert: convert sample data from spare format to dense format for the sample comparison, Calc: 1-against-N sample comparison. A higher proportion of total time was used by Convert and Calc steps when the number of sample pairs increases. (B) Time and memory usage for 10,000 samples' pair-wise similarity calculation using the different numbers of CPU threads. Real-time: the actual time usage of calculation, Core Time: the sum of each CPU cores' time usage.

Figure 4

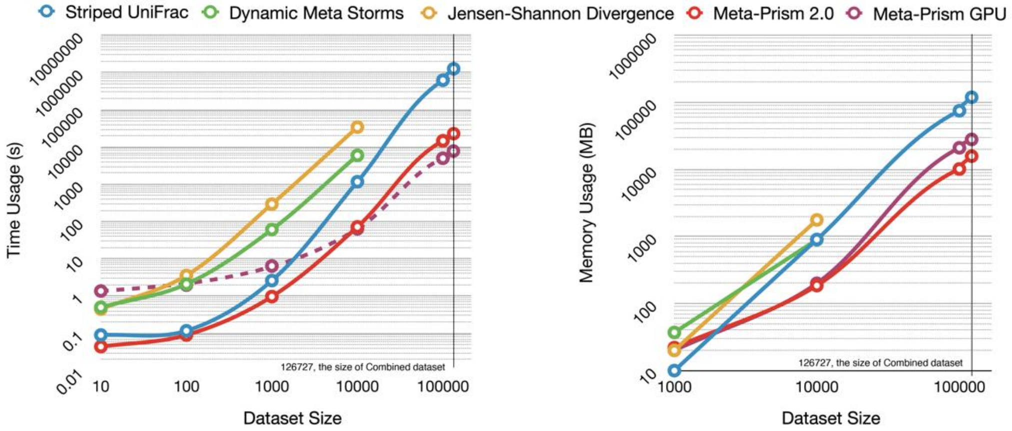

Figure 4. Time and memory usage of samples when calculating similarity matrix for datasets with different numbers of samples. (A) is for time usage comparison, and (B) is for memory usage comparison. In (A), Meta-Prism GPU time usage with dash line is GPU time usage, others are CPU core time usage.

Figure 5

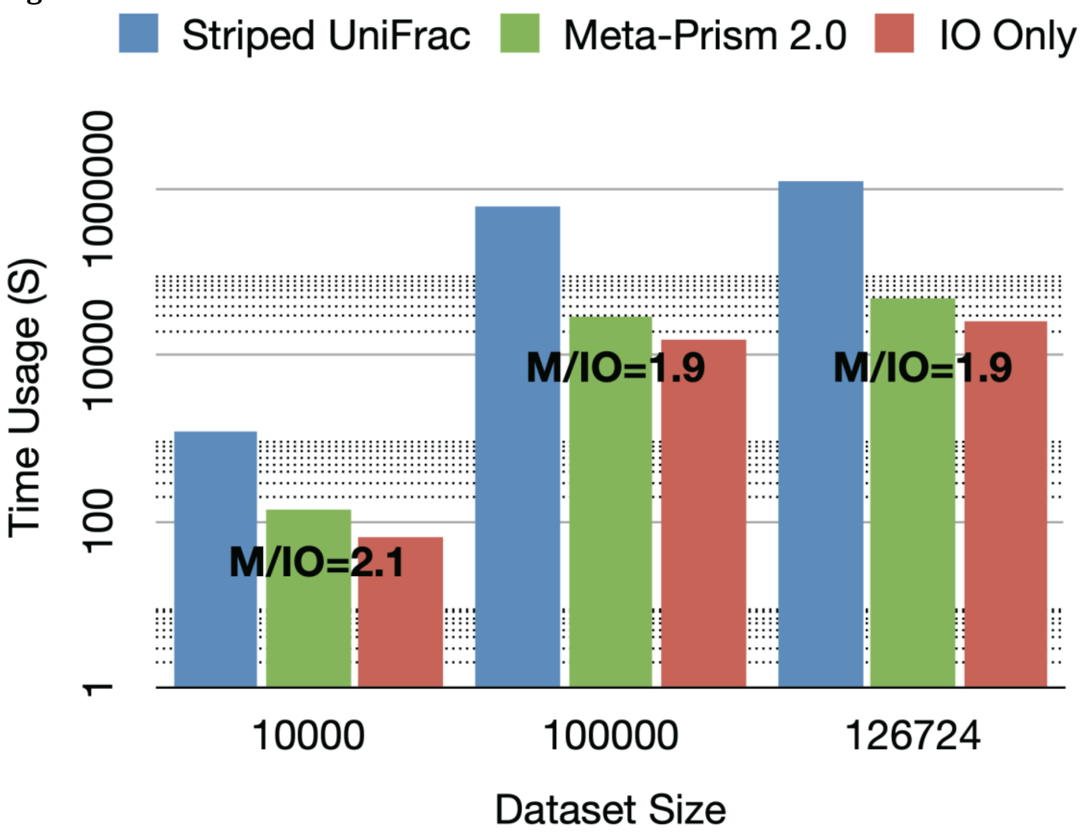

Figure 5. Time usage for different methods and IO Only on datasets with different sizes. “M/IO” is the ratio of time cost of Meta-Prism 2.0 over that of IO Only.

Figure 6

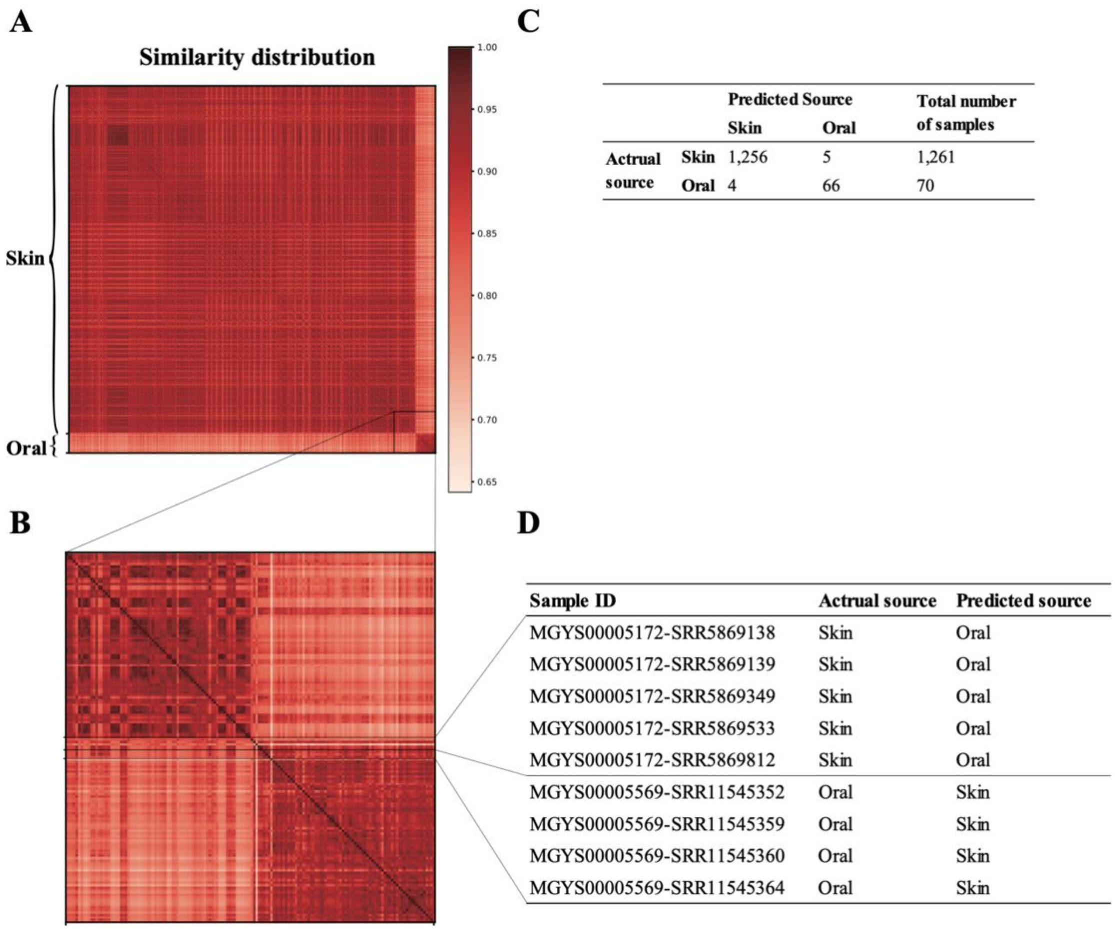

		Predicted Source		Total number of samples
		Skin	Oral	
Actrual source	Skin	1,256	5	1,261
	Oral	4	66	70

Sample ID	Actrual source	Predicted source
MGYS00005172-SRR5869138	Skin	Oral
MGYS00005172-SRR5869139	Skin	Oral
MGYS00005172-SRR5869349	Skin	Oral
MGYS00005172-SRR5869533	Skin	Oral
MGYS00005172-SRR5869812	Skin	Oral
MGYS00005569-SRR11545352	Oral	Skin
MGYS00005569-SRR11545359	Oral	Skin
MGYS00005569-SRR11545360	Oral	Skin
MGYS00005569-SRR11545364	Oral	Skin

Figure 6. Clustering result of human samples from close biomes using similarities calculated by Meta-Prism 2.0. (A) Similarity distribution of 1,331 samples. The samples were successfully clustered into two groups though we did not specify the number of clusters *prior*. (B) Similarity distribution of 9 samples that are not clustered with samples from the same biome (mis-clustered). (C) Confusion matrix and the number of samples within each actual source biome and predicted biome. (D) EBI MGnify study accession, run accession, actual biome source, and predicted biome source of 9 mis-clustered samples.

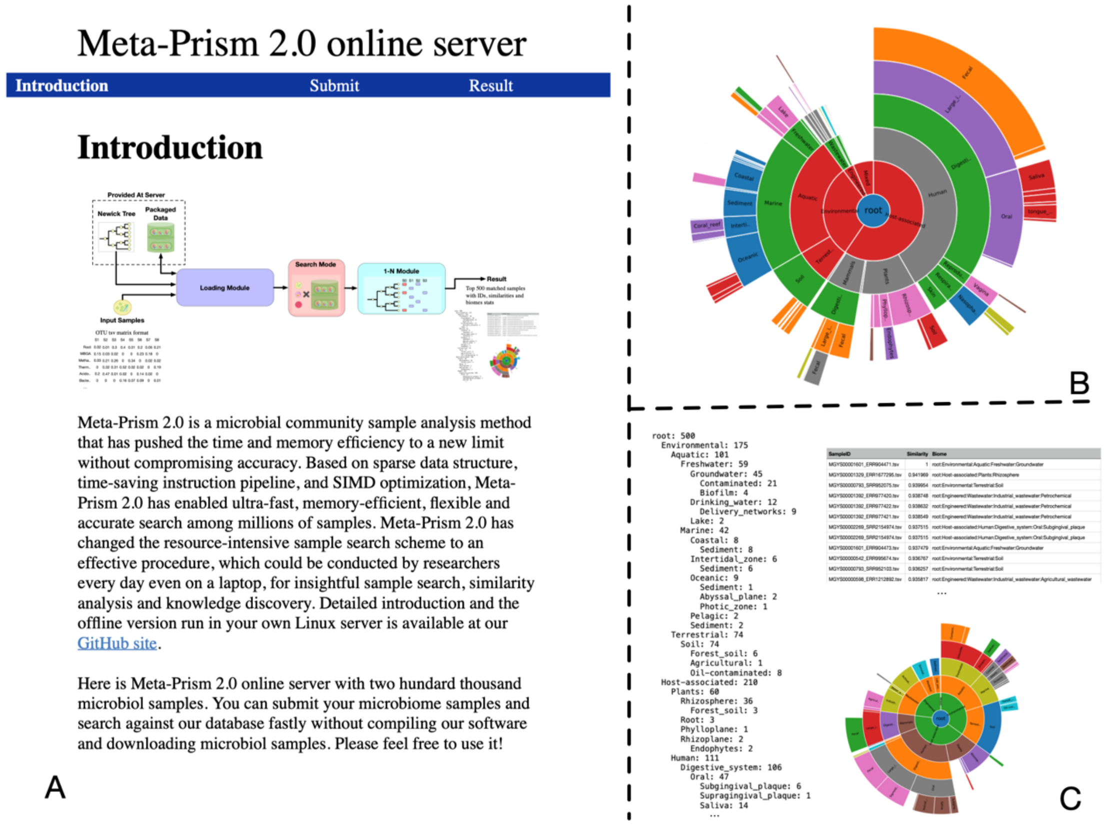

Figure 7. Screenshots for Meta-Prism 2.0 web server. (A) Meta-Prism 2.0 front page. (B) Statistics about sample source biomes for the built-in two hundred thousand microbial samples. (C) An example output consisting of top 500 matched samples' IDs and similarity values in table format, statistics about sample source biomes of top 500 matched samples in text tree and sunburst format.

Tables

Table 1

Table 1. The Combined dataset and FEAST dataset used in this study. Details are provided in **Supplementary Table**.

**Dataset**	**Combined dataset**	**FEAST dataset**
**Top-level biome**	Root	Human gut
**Number of biomes involved**	114	3
**Number of samples**	126,727	10,270
**Number of species**	45,477	5,762
**The average number of species per sample**	411.22	111.05
**Notes**	Selected samples from MGnify database	Selected samples from the FEAST study

Table 2

Table 2. The search results of 11 groundwater samples against Combined dataset.

Count groundwater-related samples in the top 100 matching samples

**Sum of groundwater-related samples:**								
53	58	75	53	38	88	56	79	73

Table 3

Table 3. Average similarities of biomes between 611 sink samples.

	**environmental**	**human skin**	**insecta**	**mammal**	**plants**
**Average Similarity**	0.7886	0.8896	0.7849	0.8478	0.8313

GIGA-D-21-00388

Meta-Prism 2.0: Enabling algorithm and web server for ultra-fast, memory-efficient, and accurate analysis among millions of microbial community samples

Kai Kang; Hui Chong; Kang Ning

GigaScience

Dear Mr Kang,

Your manuscript "Meta-Prism 2.0: Enabling algorithm and web server for ultra-fast, memory-efficient, and accurate analysis among millions of microbial community samples" (GIGA-D-21-00388) has been assessed by our reviewers. Although it could be of interest, we are unable to consider it for publication in its current form. The reviewers have raised a number of essential points which need careful consideration and some major revisions.

Their reports are below.

In particular, both reviewers point out that the methods and test cases need to be described in much more detail. I also fully agree with reviewer 2 that code, test data and supporting scripts need to be available in a form that allows full reproduction of all results and figures.

For this purpose, we recommend the use of reproducibility platforms such as Code Ocean https://codeocean.com/ or Gigantum https://docs.gigantum.com/, which allow to present code and data together in an executable format.

Answer:

We thank editor for these suggestions. We have updated the manuscript for clarity, as well as deployed all real data applications' workflow to the Code Ocean platform (https://codeocean.com/capsule/3103931). We also modified the program naming, added program documentation and did more tests to improve program robustness.

However, performance and accuracy analysis require similarity matrix calculations for the entire database, which require, as we show in Figure 4, more than 353 hours of CPU time to calculate. Therefore, we did not deploy this part into Code Ocean, but provided step introductions, intermediate data and supporting scripts in Google Drive (the FTP temporary account provided before seems to have been used by others), and the sharing link is https://drive.google.com/drive/folders/1BB7jyUBigw7ysjY_LCmVEdpaIZSlod-l?usp=sharing.

Please check our responses to reviewers for details.

Please note that we will need to re-review a revised manuscript before we can make a decision, and we can only consider the revised manuscript for publication if the reviewers are able to reproduce the work with the code and data provided.

If you are able to fully address the reviewers' points, we would encourage you to submit a revised manuscript to GigaScience. Once you have made the necessary corrections, please submit online at:

https://www.editorialmanager.com/giga/

If you have forgotten your username or password please use the "Send Login Details" link to get your login information. For security reasons, your password will be reset.

Please include a point-by-point within the 'Response to Reviewers' box in the submission system. Please ensure you describe additional experiments that were carried out and include a detailed rebuttal of any criticisms or requested revisions that you disagreed with. Please also ensure that your revised manuscript conforms to the journal style, which can be found in the Instructions for Authors on the journal homepage. If the data and code has been modified in the revision process please be sure to update the public versions of this too.

The due date for submitting the revised version of your article is 09 May 2022.

I look forward to receiving your revised manuscript soon.

Best wishes,

Hans

Hans Zauner
GigaScience
www.gigasciencejournal.com

Reviewer reports:

Reviewer #1: In Kang, Chong, and Ning, the authors present Meta-Prism 2, a microbial community analysis framework, which calculates sample-sample dissimilarities and queries microbial profiles similar to those of user-provided targets. Meta-Prism 2 adopts efficient algorithms to achieve the time and memory efficiency required for modern microbiome "big data" application scenarios. The

authors evaluated Meta-Prism 2's performance, both in terms of separating different biomes' microbial profiles and time/memory usage, on a variety of real-world studies.

I find the application target of Meta-Prism appealing: achieving efficient dissimilarity profiling is increasingly relevant for modern microbiome applications. However, I'm afraid the manuscript appears to be in poor state, with insufficient details for crucial methods and results components. Some display items are either missing or mis-referenced. As such, I cannot recommend for its acceptance, unless major improvements are made. My comments are detailed below.

Answer:

We thank the reviewer for these comments. Please check our detailed responses as below.

Major

1. The authors claim that from its previous iteration, the biggest improvements are: (1) removal of redundant nodes in 1-against-N sample comparisons. (2) functionality for similarity matrix calculation (3) exhaustive search among all available samples.

a. (1) seems the most crucial for the method's improved efficiency. However, the details on why these nodes can be eliminated, and how dissimilarity calculation is achieved post-elimination are not sufficient. The caption for Figure 1C, and relevant Methods texts (lines 173-188) should be expanded, to at least explain i) why it is valid to calculate (dis)similarity post elimination based on aggregation, ii) how aggregation is achieved for the target samples.

b. I may not have understood the authors on (2), but this improvement seems trivial? Is it simply that Meta-Prism 2 has a new function to calculate all pair-wise dissimilarities on a collection of microbial profiles?

c. For (3), it should be made clearer that Meta-Prism 1 does not do this. I needed to read the authors' previous paper to understand the comment about better flexibility in customized datasets. I assume that this improvement is enabled because Meta-Prism 2 is vastly faster compared to 1? If so, it might be helpful to point this out explicitly.

Answer:

Thanks for your questions. Removing the redundant nodes is the most crucial step for efficiency improvement in Meta-Prism 2.0. To better explain how our method works and why it is valid, we updated the explanation for our comparison details at " Fast 1-N sample comparison " section:

In Meta-Prism 1.0, for nodes with no abundance, the system will calculate at variables value 0, without any influence on the result. Some nodes only have the relative abundances for one sample, and the most of the system's calculations on them are invalid which are equivalent to multiplying their abundances by one minus evolutionary distance and passing these to their parent nodes. To

save the time wasted on such operations, when Meta-Prism 2.0 calculates 1-N comparison (S_0 against S_n), it will only consider nodes that are abundant in S_0 (marked node) and generate a fixed execution order based on them. To deal with nodes that only contain abundance information in S_n, Meta-Prism 2.0 will multiply the abundance on these nodes by the cumulative evolutionary distance factor and send them to the nearest labeled parent nodes.

(b, c) These two improvements are both based on the efficiency improvement. Meta-Prism 1.0 is designed to handle large-scale biome data, and the time and memory usage of similarity matrix calculation increases quadratically which is difficult for it to handle. Also because version 1.0 is inefficient, it takes too long to exhaustively compare each sample against the entire database. We built index system based on the database in advance, and then select a subset of samples that are more likely to match the input samples from the database. It's all because Meta-Prism 2.0 has become faster, and it is possible to add similarity matrix calculation and remove index system. We also revised the Introduction section to point this causal relationship out, and gave brief introduction to the index system in Meta-Prism 1.0: Facing the large and sparse characteristics of microbial samples, we adopted a special sparse storage format and a fast 1-N calculation method. This greatly reduces memory usage and time consumption. As the computing and storage efficiency increased, it adds similarity matrix calculation function to analysis more than 100,000 samples' beta diversity. When searching samples, due to the efficiency improvement, Meta-Prism 2.0 no longer needs to generate the index system to select high-probability subsets for similarity comparison, but conduct an exhaustive search against the entire database.

2. I am lost on the accuracy evaluation results for predicting different biomes (Figure 2).

a. How are biomes predicted for each microbial sample?

b. What is the varying classification threshold that generates different sensitivities and specificities?

c. Does "cross-validation" refer to e.g. selection of tuning parameters during model training, or for evaluation model performances?

d. What are the "Fecal", "Human", and "Combined" biomes for the Feast cohort? Such details were not provided in Shenhav et al.

Answer:

Thanks for you question. In order to predict the biomes of the test sample, we calculate the similarity between the test sample and each sample in the source dataset, and then find the samples with the top 100 similarity in the source dataset. The similarity of these samples is then summed by biome and normalized by dividing by the total number of samples in the source dataset for each biome. After the resulting values are normalized, it is the probability that the test sample belongs to each biome.

We then used DLMER (https://github.com/HUST-NingKang-Lab/DLMER-Bio), an R-based analysis tool, to evaluate the predicted ROC. The thresholds generated by DLMER range from 0.01 to 1 with a fixed step size of 0.01. We added this to "Result-Accuracy evaluation" section: The varying classification threshold that generates different sensitivities and specificities range from 0.01 to 1 with a fixed step size of 0.01.

About cross-valiation, the main purpose is for evaluation model performances.

The "Fecal", "Human", and "Combined" biomes refer to the biomes from which the microbiome samples were collected. This information is consistently used for all cohorts mentioned in this study, including the FEAST cohort.

Moderate

1. I understand that this was previously published, but could the authors comment on the intuitions behind their dissimilarity measure, and how it compares to similar measures such as the weighted UniFrac?

a. Does Meta-Storm and Meta-Prism share the same similarity definition? If so, why would they differ in terms of prediction accuracies?

Answer:

Thanks for your question. These methods are all based on evolutionary information with slightly difference. Meta-Storm proposes its similarity measure as:

$$\text{GetSimilarity}(X) = \begin{cases} \text{MIN}(X); \\ \text{Reduce}(X); & \textit{If X is a Leaf Node} \\ \\ \text{GetSimilarity}(X.\text{Left}) \\ +\text{GetSimilarity}(X.\text{Right}) \\ +\text{MIN}(X); & \textit{If X is an Internal Node} \\ \text{Reduce}(X); \end{cases} \quad (3)$$

$$\text{MIN}(X) = (X.P1 \le X.P2)\ ?\ X.P1 : X.P2; \quad (1)$$

$$\begin{aligned} &\text{Reduce}(X)\{ \\ &\qquad M = \text{MIN}(X); \\ &\qquad X.\text{Ancestor}.P1 += (X.P1 - M) * (1 - \text{Dist}); \quad (2) \\ &\qquad X.\text{Ancestor}.P2 += (X.P2 - M) * (1 - \text{Dist}); \\ &\qquad \} \end{aligned}$$

Dynamic Meta-Storm follows the Meta-Storm. For nodes that cannot be mapped to definite tip nodes in the phylogenic tree, Dynamic Meta-Storm introduces dynamic virtual nodes to make the comparison accuracy reach the species level.

Meta-Prism proposes its own new measure while retaining the Meta-Storms measure.

```
Difference-based-Scoring-Function(X)
---------------------------------------------
if X is an internal node:
        Difference-based-Scoring-Function(X.Left)
        Difference-based-Scoring-Function(X.Right)
```

```
        if X is not root:
                X.Ancestor.Difference+=X.Difference*(1-X.DistToAncestor)
        else: return 1-X.Difference
    if X is a leaf node:
        M=abs(X.S1-X.S2)/2
        X.Ancestor.Difference+=M*(1-X.DistToAncestor)
    return 0
```

Meta-Prism 2.0 has a faster implementation of Meta-Storm measure. Meta-Prism 2.0 allows nodes to be mapped to the middle of the phylogenic tree.

Meta-Storm was not involved in the comparison. Dynamic Meta-Storm and Meta-Prism 2.0 are both developed base Meta-Storm, but they have different solution for nodes that cannot be mapped to definite tip nodes in the phylogenic tree. So their results were very similar, but slightly different.

2. There seems to be some mis-referencing on the panels of Figure 1.

a. Panel B was not explained at all in the figure caption.

b. Line 185 references Figure 1E, which does not exist.

Answer:

Thanks for your comment. We modified the caption of Figure 1 and marked panel B. Figure 1 B is an example for dense format and sparse format. When an evolutionary tree has a total of 25 nodes, and one of the samples has 5 non-zero nodes. Dense format will store all nodes in a fixed array, while sparse format will only store the abundance and sum of 5 non-zero nodes. We added this to Figure 1B caption. We also removed the reference to Figure 1E (which was originally an introduction to SIMD)

Minor

1. The Meta-Prism 1 publication was referenced with duplicates (#16 and 24)

Answer:

Thanks for your comment. We deleted this duplicate reference.

2. There are minor language issues throughout the manuscript, but for they do not affect understanding of the materials. Examples:

a. Line 94: analysis -> analyze

b. Line 193: We also obtained a dataset that consists of …

Answer:

Thanks for your comment. We have corrected these language issues throughout the manuscript.

Reviewer #2: In this article the authors present Meta-Prism 2, a software to compute distances between metagenomic samples and also query a specific sample against a pool of samples.
They call "sample" a precomputed file with abundance of multiple taxa.
In the article they first succinctly present multiple aspects on the underlying algorithms.
Then they provide an extensive analysis on the precision, ram and time consumption of the software.
Finally, they show 3 applications of Meta-Prism 2.

I will start to say that the execution time of the tool looks very good compared to all other tools.

Answer:

We thank reviewer for these comments. We have updated the manuscript throughout for clarity. Please check the detailed answers as below.

But I have multiple concerns about these numbers.

- First, I like to reproduce the results of a paper before approving it. But I had a few problems doing so.
    * The tool do not compile as it is on git. I had to modify a line of code to compile it. This is nothing very bad but authors of tools should be sure that their main code branch is always compiling. See the end of the review for bug and fix.
    * The analysis are done using samples from MGnify. I found related OTU tsv files linked in the supplementary but no explanation on how to transform such files in pdata files that the software is processing.
    * The only way to directly reproduce the results is to trust the pdata files present on the github of the authors. I would like to make my own experiments and compare the time to transform OTU files into pdata with the actual run time of MP2.
- The authors evaluated the accuracy of their method (which is nice) but did not gave access on the scripts that were used for that. I would like to see the code and try to reproduce the figure by myself on my own data.

Answer:

Thanks for your questions. We fixed the bug that could not compile on some platforms, and completed verification on some platforms including macOS, Linux and Code Ocean server.
Meta-Prism2.0 supports the direct input of OTU tsv files and the transfer of OTUs as pdata files. We previously put a demonstration of how to take tsv files as input into the docs/usage.md file on GitHub (https://github.com/HUST-NingKang-Lab/Meta-Prism-2.0). We've put this part in the README of GitHub page for everyone's attention. How to pack multiple TSV files into pdata is described in the "Detailed Usage - Package data" chapter now. Users can use the packaged data for space efficiency, or use the same load method to directly read multiple TSV files and perform subsequent analysis and calculation.

We provided three real data applications on the Code Ocean platform (https://codeocean.com/capsule/3103931). In terms of performance and efficiency analysis, we used the time function (time usage) that comes with Linux, the htop tool (memory usage) and the XCode toolkit (time usage for each function). We provided accuracy analysis code and intermediate results for easy reproduction at Google Drive (https://drive.google.com/drive/folders/1BB7jyUBigw7ysjY_LCmVEdpaIZSlod-l?usp=sharing).

- The 2nd and 3rd applications are explained in plain text but there is no script related neither any table of graphics to reproduce or explain the results. The only way for me to evaluate this part is to trust the word of the authors. I would like the authors to show me clear and indisputable evidences.

Answer:

Thanks for your question. Because the results of the 2nd and 3rd applications can be well explained in plain text, we didn't provide data in tabular form in the manuscript. In order to convince researchers of our work, we published our source code, data, and processing scripts through the Code Ocean platform (https://codeocean.com/capsule/3103931). This will allow other researchers to reproduce our applications. We also provided two tables to show the results of the 2nd and 3rd applications.

For the methods part it is similar. We have hints on what the authors did, but not a full explanation:

- For the similarity function, I would like to know where it comes from. The cited papers [14] and [24] do not help on the comprehension of the formula. If the function is from another paper, I ask the authors to add a clear reference (paper + section in the paper) ; if not, I would like the authors to explain in details why this particular function, how they constructed it and how it behaves.

Answer:

We have added the citations about the similarity functions, and we have added text description and pseudocode in the method section: Our similarity is proposed to measure similarity between a pair of community samples, independent of data type and sequencing depth [14](section 2.2.2). The details of similarity calculation are shown at Algorithm 6 in Supplementary Materials with the default execution order being generated from Algorithm 2 in Supplementary Materials with all node marked. To calculate the similarity of the two samples (n=1 in pseudocode), we will recursively calculate the similarity of the relative abundance of the two samples at each node and deduce, and then multiply the relative abundance remained by one minus the evolutionary distance and send it to the parent node.

- The authors refer multiple times to "sparse format" applied to disk & cache but never defined what they mean by that. I would like to see in this section which exact data structure is used.

Answer:

It should be noted that our sparse data has no unique design, just a variable-length array that stores both abundance and node id. We have revised our introduction to sparse-data to be more explicit at

"Space-saving data format" in Method section: Unlike other methods that store in fixed-length arrays, Meta-Prism 2.0 stores taxonomic abundance data in a sparse format, that is, uses a variable-length list to store only relatively abundant non-zero nodes: the data includes their relative abundance and the node id of phylogenetic tree (Figure 1B).

The definition of structure is:

```
struct abdElement{
    uint32_t ID;float data;
};
struct SampleData{
    string name;
    vector<abdElement> data;
};
```

Taking Figure 1B as an example, when an evolutionary tree has a total of 25 nodes, and one of the samples has 5 non-zero nodes. Dense format will store all nodes in a fixed array, while sparse format will only store the abundance and sum of 5 non-zero nodes. We have added this to Figure 1 caption.

- In the Fast 1-N sample comparison, the authors write about "current methods" but without citing them. I would like the authors to refer to precise methods/software, succinctly describe them and then compare their methods on top of that.

Answer:

We have added the citations about the methods, and brief description at manuscript: Current methods traverse phylogenetic tree (with redundant nodes) and execute operation during similarity calculation (Striped Unifrac calculates the difference at each node, divides it by the branch distance. Meta-Storm and Dynamic Meta-Storm accumulates the similarity at each node and passes the residual abundance to the parent node. Meta-Prism 1.0 calculates the difference at each node, divides it by 1 minus the evolutionary distance and passes it to the parent node), wasting time on redundant operations[15-17].

Also in this part, the authors point at figure 1E that is not present in the manuscript.

- The figure 1 is not fully understandable without further details in the text. For example, what is Figure 1C4 ?

Answer:

Thanks for your comment. We have corrected this mistake. Now Figure 1 has only 4 subfigures.

We added a more detailed introduction in "Space-saving data format" in Method section, and referenced and explained the subplots in Fig1C: In Meta-Prism 1.0, for nodes with no abundance (which also are the vast majority), the system will calculate at variables value 0, without any influence on the result. Some nodes have only the relative abundance of one sample, and the most

of the system's calculations on them are invalid which are equivalent to multiplying their abundances by one minus evolutionary distance and passing these to their parent nodes. To save the time wasted on such operations, when Meta-Prism 2.0 calculates 1-N comparison (S_0 against S_n), it will only consider nodes that are abundant in S_0 (marked node) and generate a fixed execution order based on them (GenOrder step, Figure 1C (2 and 3), Algorithm 2 in Supplementary Materials). To deal with nodes that are only contain abundant in S_n, Meta-Prism 2.0 will multiply the abundance on these nodes by the cumulative evolutionary distance factor and send them to the nearest labeled parent nodes (Convert step, Figure 1C (4), Algorithm 5 in Supplementary Materials). Which node to send and the factor value is calculated at Algorithm 4 in Supplementary Materials. The fixed execution order without branches and jumps will lead the CPU to use the instruction pipeline. Additionally, Meta-Prism 2.0 is implemented based on SIMD AVX intrinsic[26], thus can execute operations to compare a sample S0 with other multiple samples (referred to as Sn) at the same time (SimilarityCalculation step, Figure 1C (5), Algorithm 6 in Supplementary Materials).

I want to point that the paper is not correctly balanced in term of content. 1.5 page for time execution analysis is too much compared to the 2 pages of methods and less than 1 page of real data applications.

Answer:

Thanks for your comment. We have added more contents in both methods and real data applications, and simplified the discussion of time execution analysis.

Finally, the authors are presenting a software but are not following the development standards. They should provide unit and functional tests of their software. I also strongly recommend them to create a continuous integration page with the git. With such a tool the compilation problem would not exist.

Answer:

Thanks for your comment. We modified the program naming and annotation to make the program more readable. We also did more tests to improve program robustness. In our server (https://hust-ningkang-lab.github.io/Meta-Prism-2.0/), there are more than 200,000 microbial data obtained from MGnify and FEAST. They are handled by different pipelines and have different data formats. Meta-Prism 2.0 can process these diverse inputs and give reliable results.

We have updated the manuscript for clarity, as well as providing reproducible codes and manuals on Code Ocean and GitHub.

To conclude, I think that the authors very well engineered the software but did not present it the right way. I suggest the authors to rewrite the paper with strong improvements of the "methods" and

"Real data application" sections. Also, to provide a long term useful software, they have to add guaranties to the code as tests and CI.

Answer:

Thanks for your comment. We have added more contents in both methods and real data applications, and simplified the discussion of time execution analysis.

For all these reasons, I recommend to reject this paper.

--- Bug & Fix ---

```
make
mkdir -p build
g++ -std=c++14 -O3 -m64 -march=native -pthread -c -o build/loader.o src/loader.cpp
g++ -std=c++14 -O3 -m64 -march=native -pthread -c -o build/newickParser.o src/newickParser.cpp
g++ -std=c++14 -O3 -m64 -march=native -pthread -c -o build/simCalc.o src/simCalc.cpp
g++ -std=c++14 -O3 -m64 -march=native -pthread -c -o build/structure.o src/structure.cpp
g++ -std=c++14 -O3 -m64 -march=native -pthread -c    -o build/main.o src/main.cpp
src/main.cpp: In function 'int main(int, const char**)':
src/main.cpp:128:31: error: 'class std::ios_base' has no member named 'clear'
  128 |                         buf.ios_base::clear();
      |                                       ^~~~~
make: *** [makefile:7: build/main.o] Error 1
```

To fix the bug:

src/main.cpp:128 => buf.ios.clear();

--

Please also take a moment to check our website at https://www.editorialmanager.com/giga/l.asp?i=103536&l=QFJVIVKQ for any additional comments that were saved as attachments. Please note that as GigaScience has a policy of open peer review, you will be able to see the names of the reviewers.

In compliance with data protection regulations, you may request that we remove your personal registration details at any time. (Use the following URL:

https://www.editorialmanager.com/giga/login.asp?a=r). Please contact the publication office if you have any questions.

Click here to access/download;Table;tables.xlsx

Table 1. The Combined dataset and FEAST dataset used in this study

Dataset	Combined dataset	FEAST dataset
**Top-level biome**	Root	Human gut
**Number of biomes involved**	114	3
**Number of samples**	126,727	10,270
**Number of species**	45,477	5,762
**The average number of species per sample**	411.22	111.05
**Notes**	Selected samples from MGnify database	Selected samples from the FEAST study

Table 2. The search results of 11 groundwater samples against Combined da

**Sum of groundwater-related samples:**						
53	58	75	53	38	88	56

ataset.

79	73

Table 3. Average similarities of biomes between 611 sink samples.

	environmental	human skin	insecta	mammal	plants
**Average Similarity**	0.7886	0.8896	0.7849	0.8478	0.8313

figure 1

Click here to access/download;Figure;Figure 1.eps

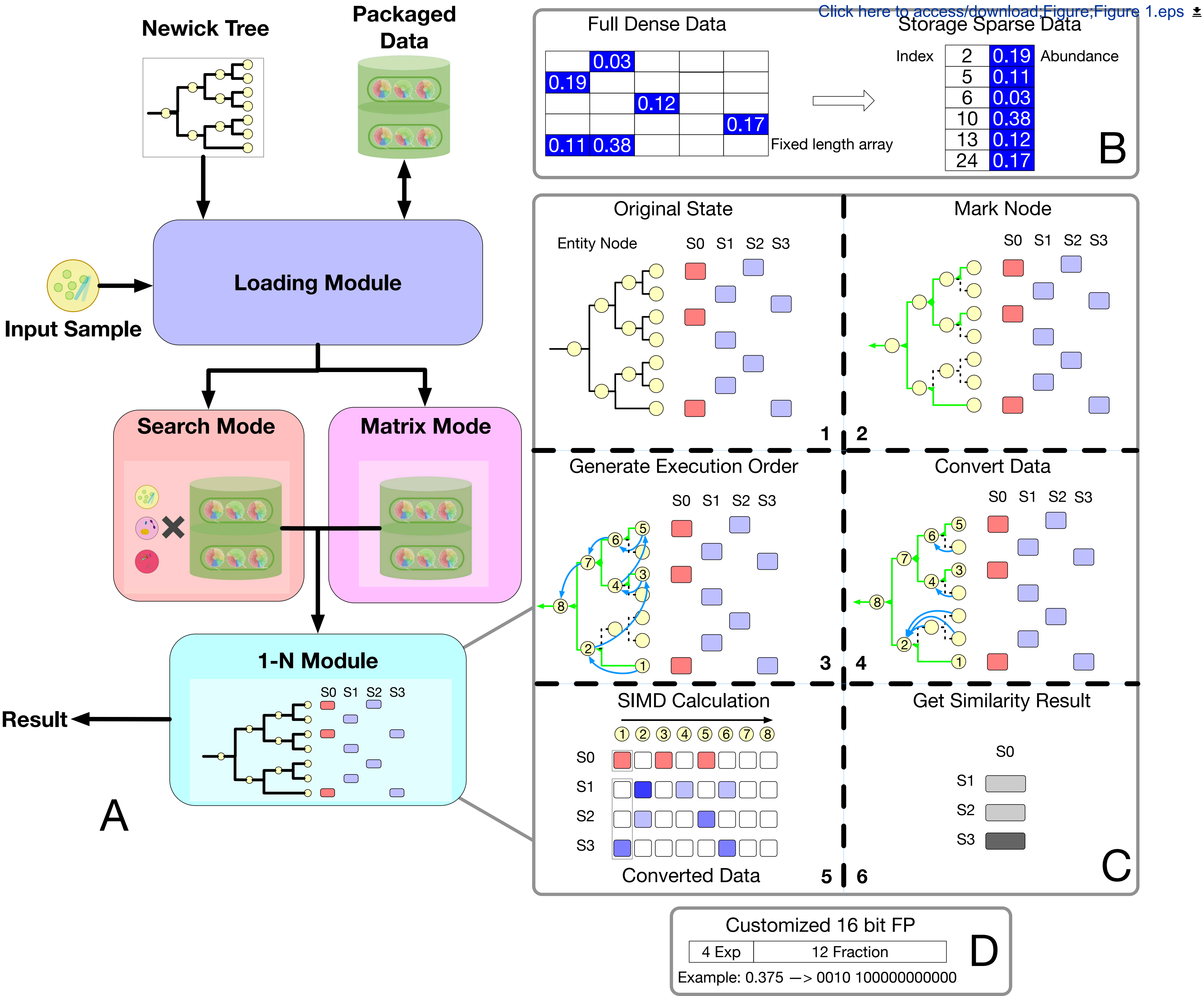

figure 2

Click here to access/download;Figure;Figure 2.eps

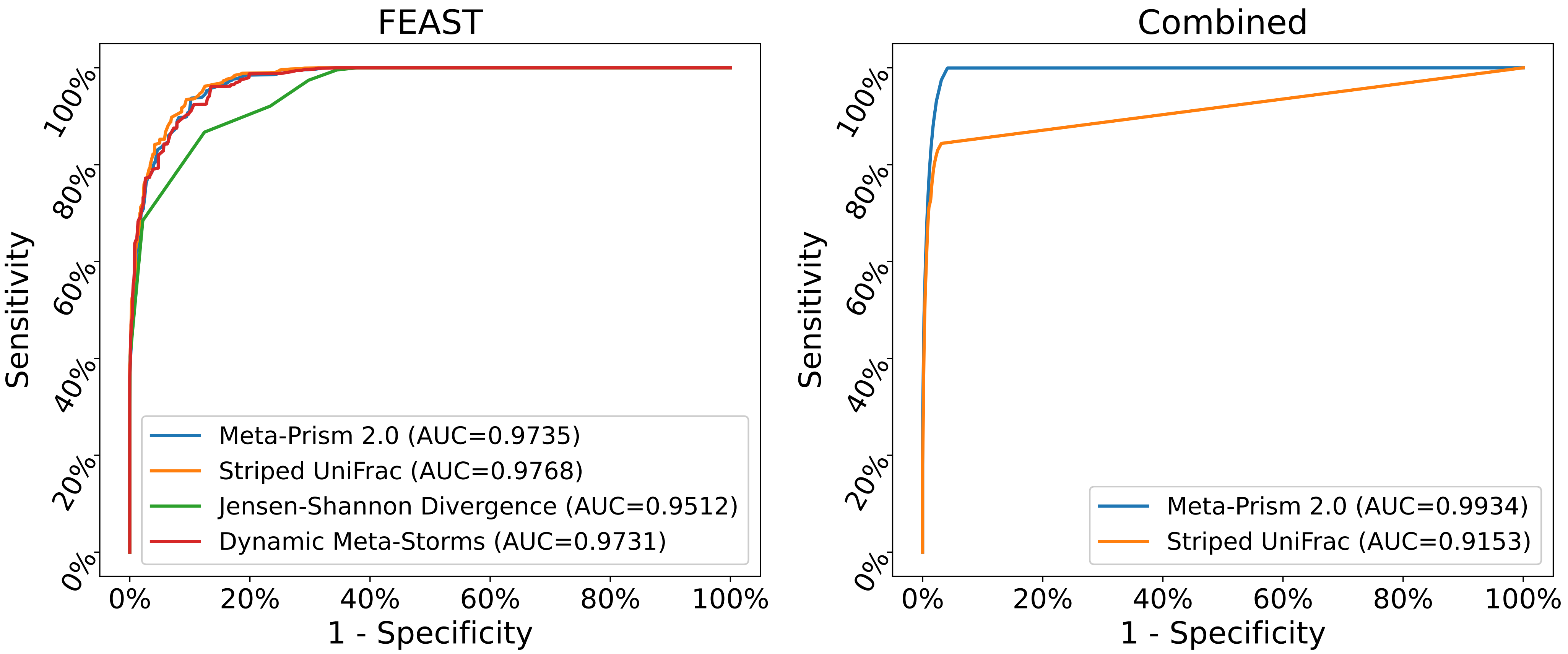

Click here to access/download;Figure;Figure 3.eps

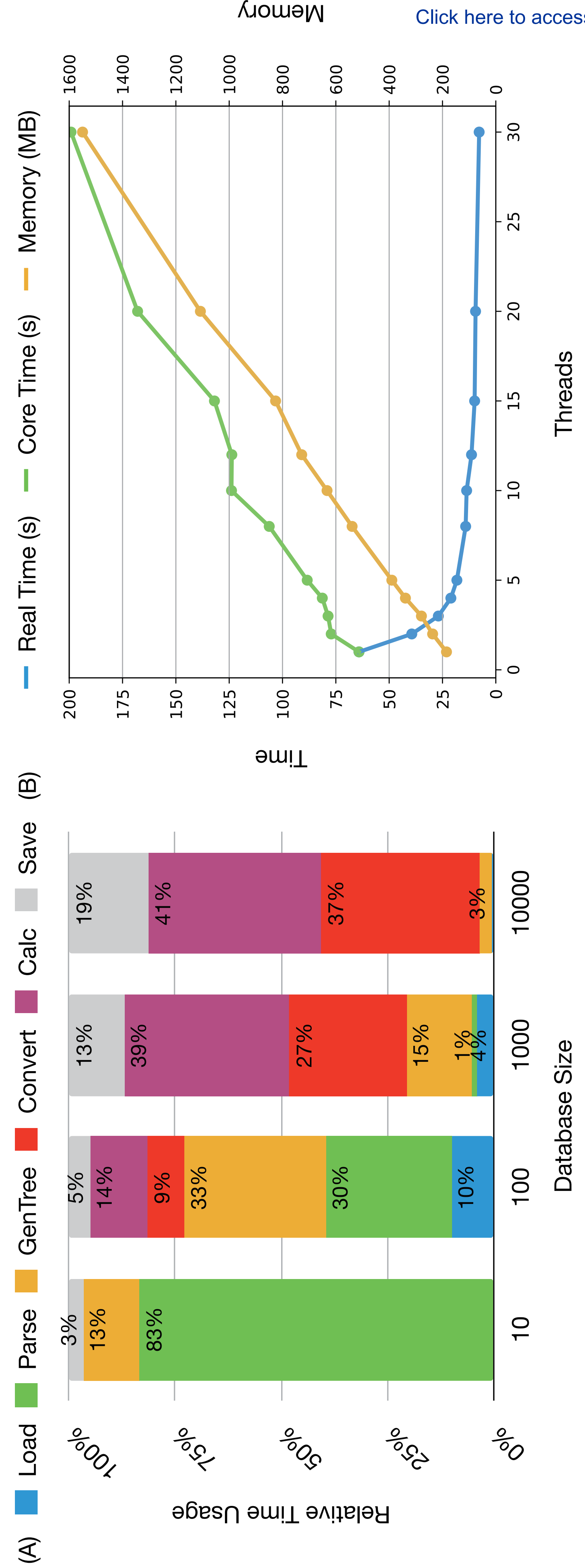

Click here to access/download;Figure;Figure 4.eps

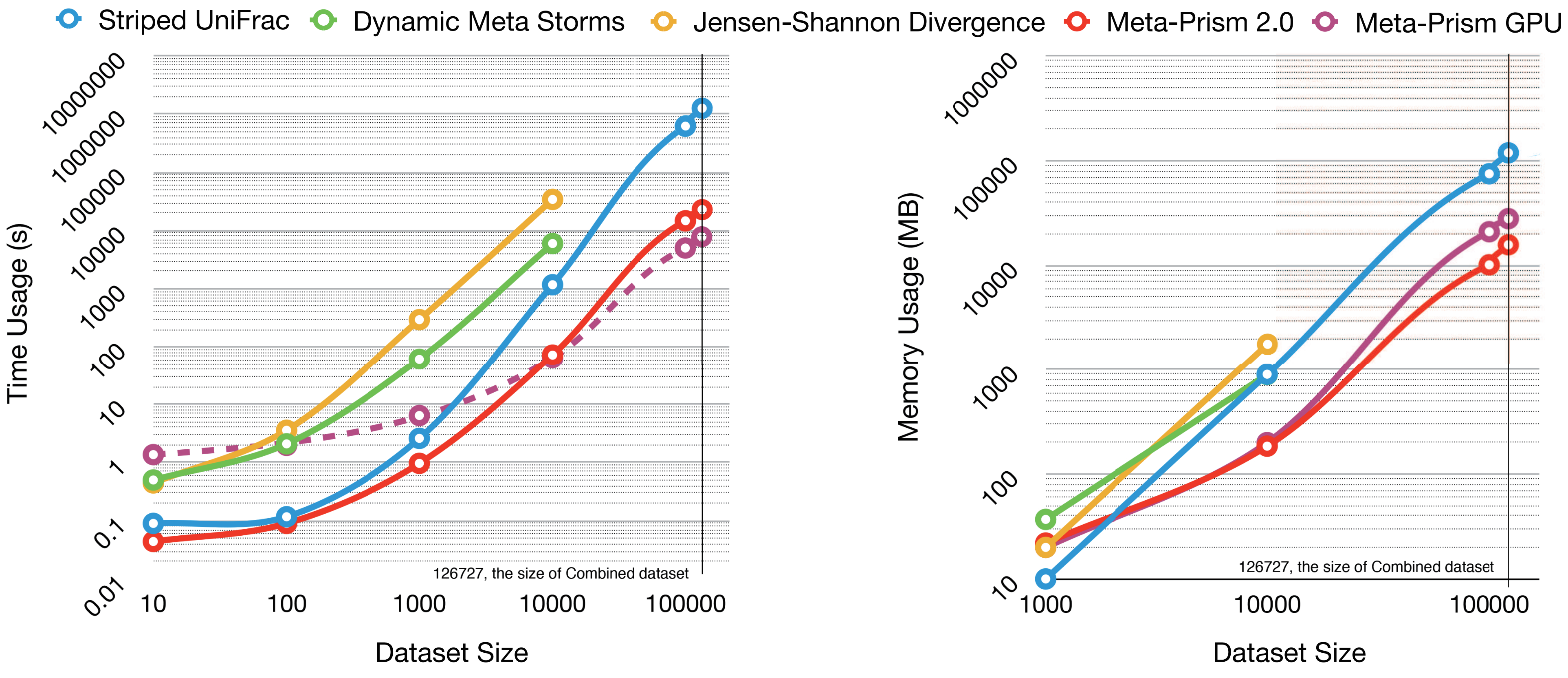

figure 5

Click here to access/download;Figure;Figure 5.eps

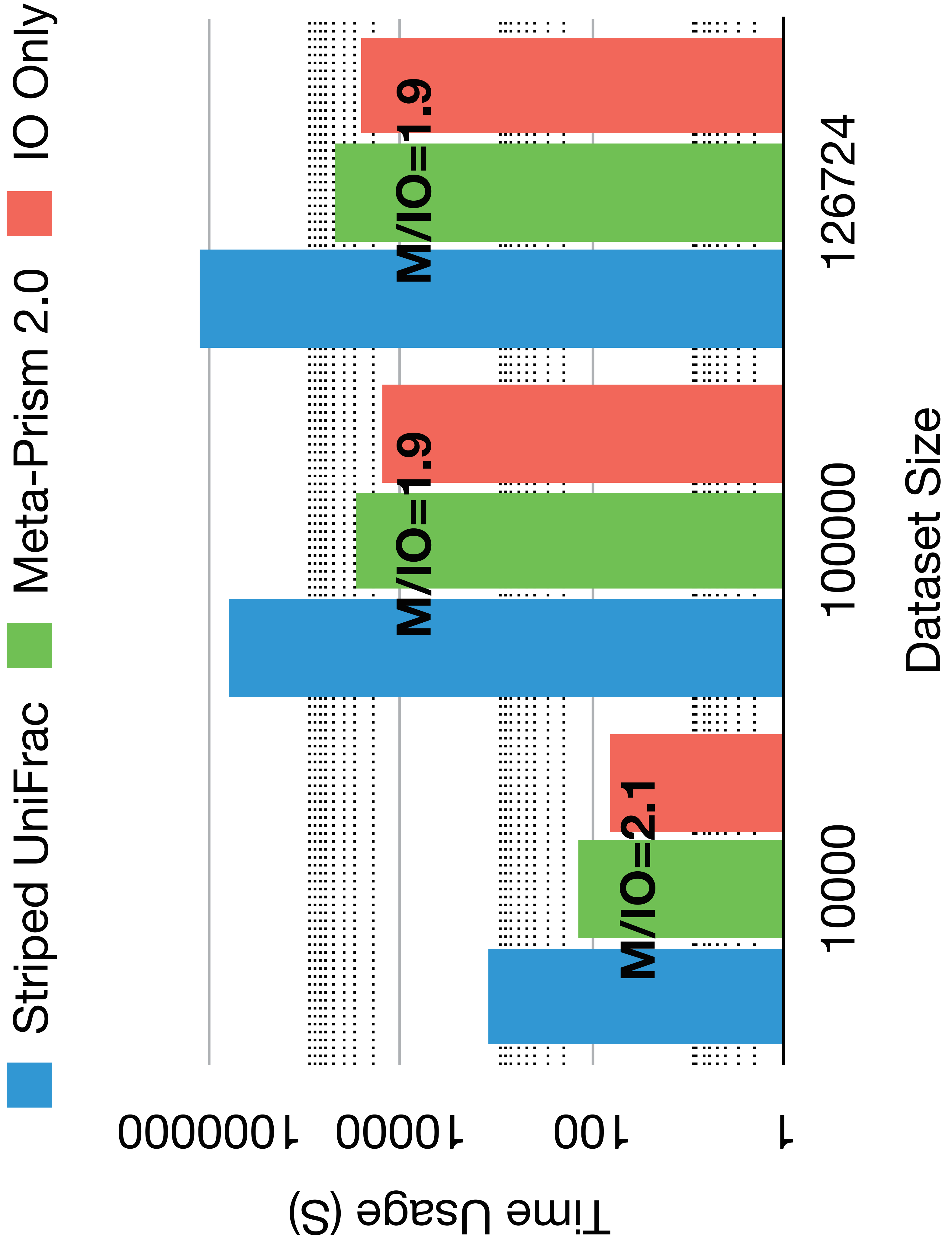

Click here to access/download;Figure;Figure 6.eps

A

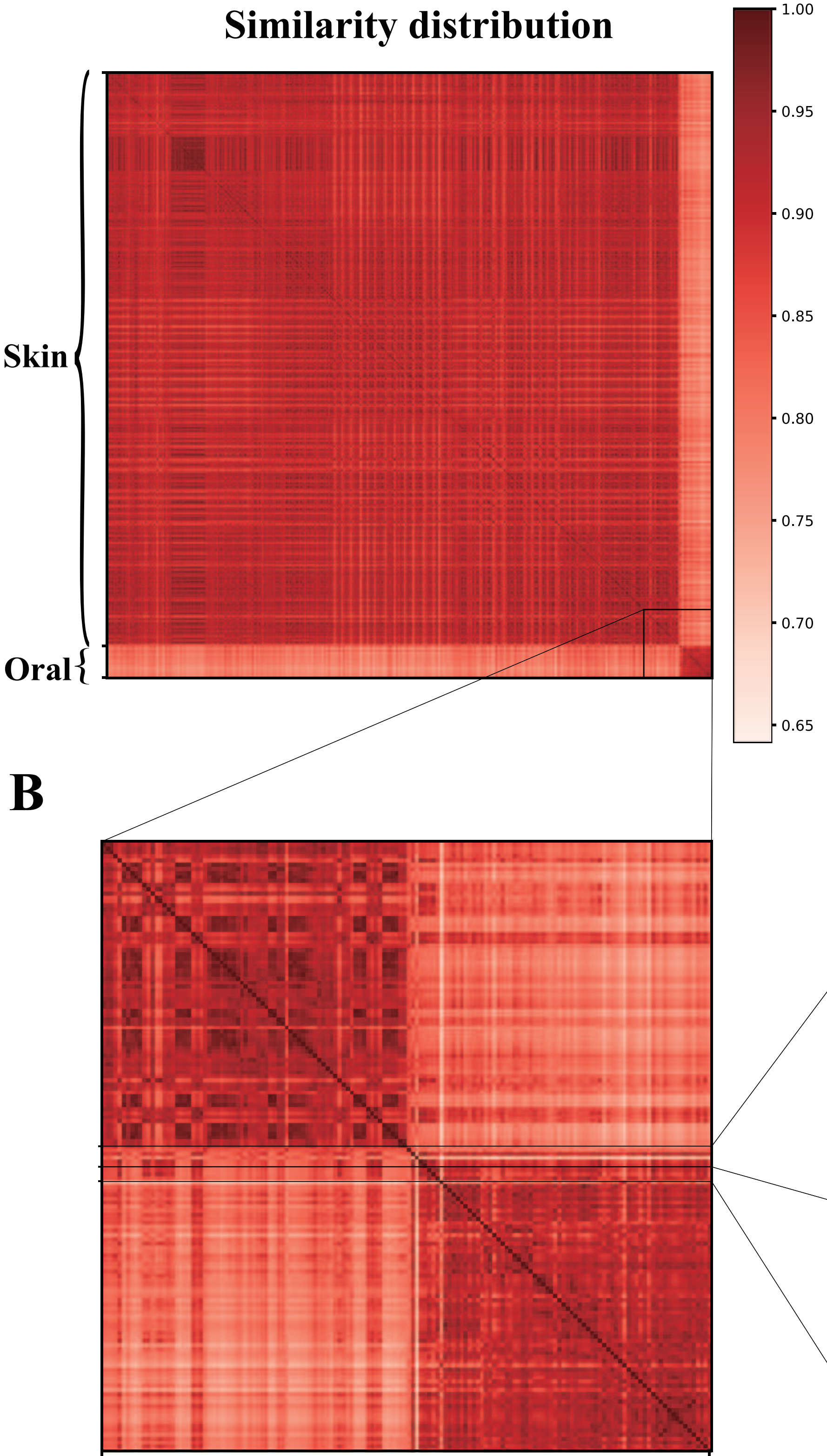

C

		Predicted Source		Total number of samples
		Skin	Oral	
Actrual source	Skin	1,256	5	1,261
	Oral	4	66	70

D

Sample ID	Actrual source	Predicted source
MGYS00005172-SRR5869138	Skin	Oral
MGYS00005172-SRR5869139	Skin	Oral
MGYS00005172-SRR5869349	Skin	Oral
MGYS00005172-SRR5869533	Skin	Oral
MGYS00005172-SRR5869812	Skin	Oral
MGYS00005569-SRR11545352	Oral	Skin
MGYS00005569-SRR11545359	Oral	Skin
MGYS00005569-SRR11545360	Oral	Skin
MGYS00005569-SRR11545364	Oral	Skin

figure 7

Click here to access/download;Figure;Figure 7.eps

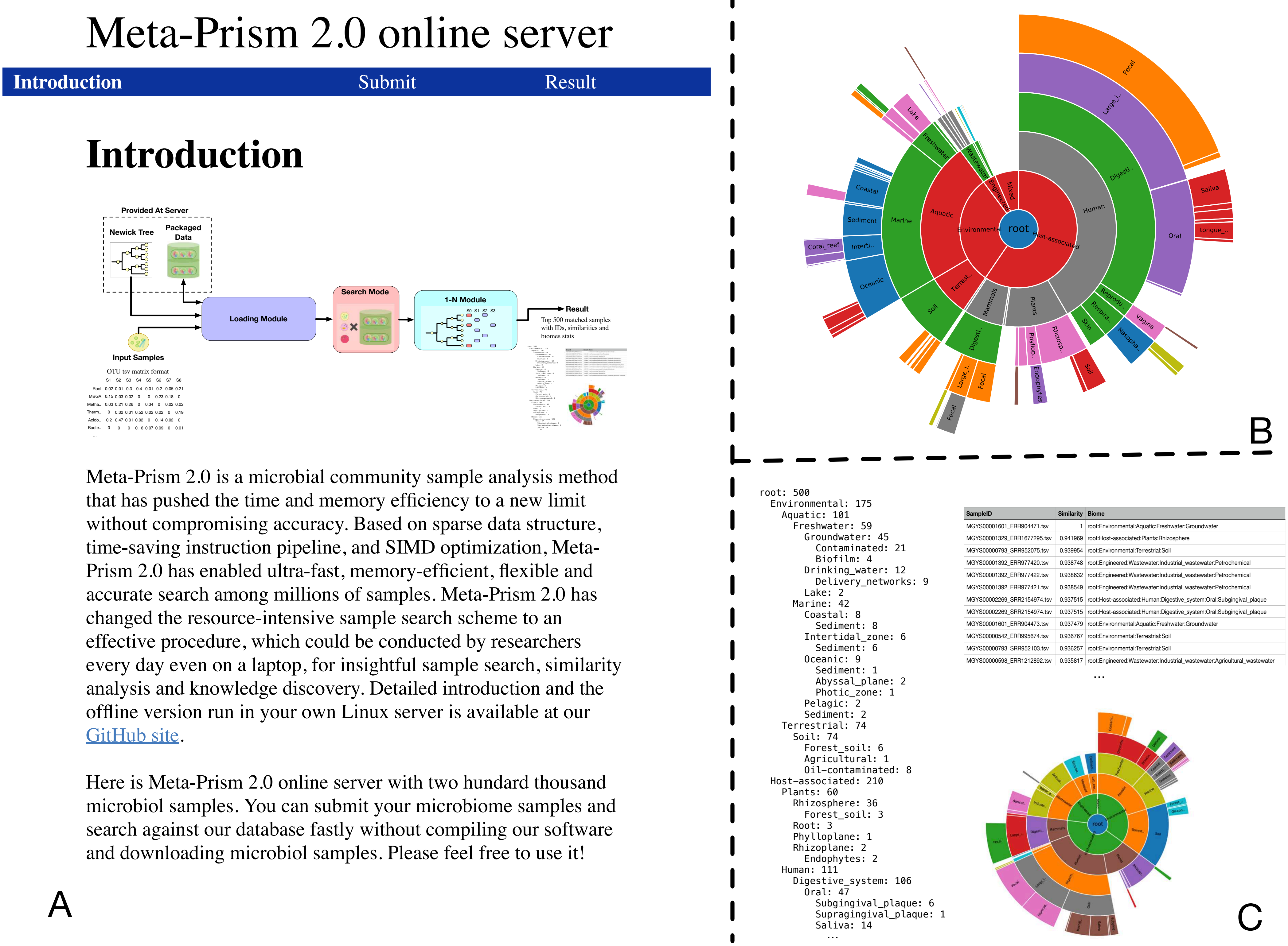

pseudocode about Meta-Prism 2.0

Click here to access/download
Supplementary Material
Supplementary Material 1.pdf

detail information of Combined dataset and FEAST dataset

Click here to access/download

Supplementary Material

Supplementary Table 1.pdf

detail information of Combined dataset and FEAST dataset

Click here to access/download
Supplementary Material
Supplementary Table 1.xlsx
