# Supplementary material for: Meta-Prism 2.0: Enabling algorithm and web server for ultra-fast, memory-efficient, and accurate analysis among millions of microbial community samples
Source: Gigascience. 2022 Jul 28;11:giac073. doi: 10.1093/gigascience/giac073 (PMC9334027; doi:10.1093/gigascience/giac073)
Supplement: giac073_Supplemental_Files [file giac073_supplemental_files.zip › Supplementary Material 1.pdf]

Algorithm 1 GenerateOrderTraversal

Input: $TreeNode$; $ExecutionOrder$; $NearestMarkedID$;

```
if TreeNode has left child then
    LeftID = GenerateOrderTraversal( TreeNode.LeftChild , ExecutionOrder)
end if
if TreeNode has right child then
    RightID = GenerateOrderTraversal( TreeNode.RightChild , ExecutionOrder)
end if
if TreeNode is marked then
    ExecutionOrder.append( TreeNode.MarkedID, LeftID, RightID, TreeNode.LeftDistance , TreeNode.RightDistance)
    return TreeNode.MarkedID
else
    return null
end if
```

Algorithm 2 GenerateExecutionOrder

Input: S_0;

Output: *ExecutionORder*: contains $FatherID$, $LeftChildID$, *RightChildID*, $LeftDistance$, *RightDistance* ;

```
Let ExecutionORder be new appendable list
GenerateOrderTraversal( PhylogeneticTree.root, ExecutionOrder) #PhylogeneticTree.root must be marked, and let its id =1
```

Algorithm 3 ConvertListTraversal

```
Input: TreeNode; ConvertList; NearestMarkedID; factor
if TreeNode is marked then
    NearestMarkedID = TreeNode.MarkedID
    factor = 1
end if
if TreeNode has left child then
    GenerateOrderTraversal( TreeNode.LeftChild, ConvertList, NearestMarkedID, factor* (1 − TreeNode.LeftDistance))
end if
if TreeNode has right child then
    GenerateOrderTraversal( TreeNode.RightChild, ConvertList, NearestMarkedID, factor* (1 − TreeNode.RightDistance))
end if
ConvertList[ TreeNode.TaxonName] =(TreeNode.MarkedID, factor)
```

Algorithm 4 GenerateConvertList

```
Output: ConvertList: contain MarkedID and factor
Let ConvertList [1... number of nodes( PhylogeneticTree) ] be new list
ConvertListTraversal( PhylogeneticTree.root, ConvertList, ExecutionOrder, 0, 1)
```

Algorithm 5 ConvertData

```
Input: S; ConvertList
Output: S′
Let S′ [1... number of nodes( PhylogeneticTree) ] be new array
for TaxonName, Abundance in S do
    S′[ ConvertList[ TaxonName].MarkedID] = abundance * ConvertList[ TaxonName].factor
end for
if S is a set of samples, do previous step at each sample
```

Algorithm 6 SimilarityCalculation

Input: S_0; S_n; *ExecutionOrder*

Output: *result*

```
Let result [1... sizeof(S_n)] be new array
for FatherID, LeftID, RightID, LeftDistance, RightDistance in ExecutionOrder do
    for i= 1...n do
        #Can accelerate this loop by SIMD
        MinLeft= min( S_0[LeftID], S_n[i][LeftID])
        MinRight= min( S_0[RightID], S_n[i][RightID])
        result[i]+= MinLeft+ MinRight
        S_0[FatherID]+= (1-LeftDistance)* (S_0[LeftID]- MinLeft)+ S_0[FatherID]+ (1-RightDistance)* (S_0[RightID]- MinRight)
        S_n[i][FatherID]+= (1-LeftDistance)* (S_n[i][LeftID]- MinLeft)+ S_n[i][FatherID]+ (1-RightDistance)* (S_n[i][RightID]- MinRight)
    end for
end for
```

Algorithm 7 MarkPhylogeneticTree

Input: S_0; *node*; *MarkID*

Output: *Marked*: node is marked or not; *MarkID*

```
if node is null then
    return False,MarkID
end if
node.marked = False
if node has relative abundance at S_0 then
    node.marked = True
end if
left, MarkID= MarkPhylogeneticTree( S_0, node.LeftChild, MarkID)
right, MarkID= MarkPhylogeneticTree( S_0, node.LeftChild, MarkID)
if left is True or right is True then
    node.Marked=True
end if
if node is marked then
    node.MarkedID= MarkID
    MarkID+= 1
end if
return node.Marked, MarkID
```

Algorithm 8 1-N Compare

Input: S_0; S_n;

MarkPhylogeneticTree(S_0, $PhylogeneticTree.root$, 0)
$ConvertList$= GenerateConvertList($S0$)
$ExecutionOrder$= GenerateExecutionORder(S_0)
Let S' [1... number of marked nodes] be new array storing taxa index and adjusted relative abundance
S'_0= ConvertData(S_0, $ConvertList$)
S'_n= ConvertData(S_0, $ConvertList$)
$result$= SimilarityCalculation(S'_0, S'_n, $ExecutionOrder$)

Algorithm 9 Matrix Mission

Input: S_n;
Output: Similarity matrix

for i in 0, 1,..., Size(S_n **do**
    1-N Compare(S_n[i],S_n[i+1:])
end for

Algorithm 10 Search Mission

Input: S_n_1;S_n_2;
Output: Similarity of S_n_1, S_n_2

for i in 0, 1,..., Size(S_n_1) **do**
    1-N Compare(S_n_1[i],S_n_2)
end for
