# Supplementary material for: Meta-Prism 2.0: Enabling algorithm and web server for ultra-fast, memory-efficient, and accurate analysis among millions of microbial community samples
Source: Gigascience. 2022 Jul 28;11:giac073. doi: 10.1093/gigascience/giac073 (PMC9334027; doi:10.1093/gigascience/giac073)
Supplement: giac073_Supplemental_Files [file Supplementary Table 1.pdf › Supplementary Table 1.pdf]

Combined dataset

RunID	AnalysisID	URL	Environment (as of January 2020)
SRR5178101	MGYA00148736	https://www.ebi.ac.uk/metagenomics/api/v1/analyses/MGYA00148736/file/SRR5178101_MERGED_FASTQ_SSU_OTU.tsv	Root-Host_associated-Human-Digestive_system-Oral-Subgingival_plaque
SRR2154895	MGYA00151072	https://www.ebi.ac.uk/metagenomics/api/v1/analyses/MGYA00151072/file/SRR2154895_FASTQ_SSU_OTU.tsv	Root-Host_associated-Human-Digestive_system-Oral-Subgingival_plaque
SRR5178088	MGYA00148800	https://www.ebi.ac.uk/metagenomics/api/v1/analyses/MGYA00148800/file/SRR5178088_MERGED_FASTQ_SSU_OTU.tsv	Root-Host_associated-Human-Digestive_system-Oral-Subgingival_plaque
SRR2155039	MGYA00150989	https://www.ebi.ac.uk/metagenomics/api/v1/analyses/MGYA00150989/file/SRR2155039_FASTQ_SSU_OTU.tsv	Root-Host_associated-Human-Digestive_system-Oral-Subgingival_plaque
SRR2086906	MGYA00143966	https://www.ebi.ac.uk/metagenomics/api/v1/analyses/MGYA00143966/file/SRR2086906_MERGED_FASTQ_SSU_OTU.tsv	Root-Host_associated-Human-Digestive_system-Oral-Subgingival_plaque
SRR2086796	MGYA00143974	https://www.ebi.ac.uk/metagenomics/api/v1/analyses/MGYA00143974/file/SRR2086796_MERGED_FASTQ_SSU_OTU.tsv	Root-Host_associated-Human-Digestive_system-Oral-Subgingival_plaque
SRR2086822	MGYA00143826	https://www.ebi.ac.uk/metagenomics/api/v1/analyses/MGYA00143826/file/SRR2086822_MERGED_FASTQ_SSU_OTU.tsv	Root-Host_associated-Human-Digestive_system-Oral-Subgingival_plaque
SRR2086747	MGYA00143923	https://www.ebi.ac.uk/metagenomics/api/v1/analyses/MGYA00143923/file/SRR2086747_MERGED_FASTQ_SSU_OTU.tsv	Root-Host_associated-Human-Digestive_system-Oral-Subgingival_plaque
DRR049465	MGYA00160355	https://www.ebi.ac.uk/metagenomics/api/v1/analyses/MGYA00160355/file/DRR049465_MERGED_FASTQ_SSU_OTU.tsv	Root-Host_associated-Human-Digestive_system-Oral-Subgingival_plaque
SRR2154960	MGYA00151035	https://www.ebi.ac.uk/metagenomics/api/v1/analyses/MGYA00151035/file/SRR2154960_FASTQ_SSU_OTU.tsv	Root-Host_associated-Human-Digestive_system-Oral-Subgingival_plaque

SRR6239877	MGYA00168158	https://www.ebi.ac.uk/metagenomics/api/v1/analyses/MGYA00168158/file/SRR6239877_FASTQ_SSU_OTU.tsv	Root-Host_associated-Human-Digestive_system-Oral-Subgingival_plaque
SRR2086791	MGYA00143973	https://www.ebi.ac.uk/metagenomics/api/v1/analyses/MGYA00143973/file/SRR2086791_MERGED_FASTQ_SSU_OTU.tsv	Root-Host_associated-Human-Digestive_system-Oral-Subgingival_plaque
SRR2086825	MGYA00143902	https://www.ebi.ac.uk/metagenomics/api/v1/analyses/MGYA00143902/file/SRR2086825_MERGED_FASTQ_SSU_OTU.tsv	Root-Host_associated-Human-Digestive_system-Oral-Subgingival_plaque
SRR2086740	MGYA00144033	https://www.ebi.ac.uk/metagenomics/api/v1/analyses/MGYA00144033/file/SRR2086740_MERGED_FASTQ_SSU_OTU.tsv	Root-Host_associated-Human-Digestive_system-Oral-Subgingival_plaque
DRR049462	MGYA00160343	https://www.ebi.ac.uk/metagenomics/api/v1/analyses/MGYA00160343/file/DRR049462_MERGED_FASTQ_SSU_OTU.tsv	Root-Host_associated-Human-Digestive_system-Oral-Subgingival_plaque
SRR1715670	MGYA00148945	https://www.ebi.ac.uk/metagenomics/api/v1/analyses/MGYA00148945/file/SRR1715670_FASTQ_SSU_OTU.tsv	Root-Host_associated-Human-Digestive_system-Oral-Subgingival_plaque
SRR2154967	MGYA00151003	https://www.ebi.ac.uk/metagenomics/api/v1/analyses/MGYA00151003/file/SRR2154967_FASTQ_SSU_OTU.tsv	Root-Host_associated-Human-Digestive_system-Oral-Subgingival_plaque
SRR6239870	MGYA00168172	https://www.ebi.ac.uk/metagenomics/api/v1/analyses/MGYA00168172/file/SRR6239870_FASTQ_SSU_OTU.tsv	Root-Host_associated-Human-Digestive_system-Oral-Subgingival_plaque
SRR2086859	MGYA00143855	https://www.ebi.ac.uk/metagenomics/api/v1/analyses/MGYA00143855/file/SRR2086859_MERGED_FASTQ_SSU_OTU.tsv	Root-Host_associated-Human-Digestive_system-Oral-Subgingival_plaque
SRR2086888	MGYA00144025	https://www.ebi.ac.uk/metagenomics/api/v1/analyses/MGYA00144025/file/SRR2086888_MERGED_FASTQ_SSU_OTU.tsv	Root-Host_associated-Human-Digestive_system-Oral-Subgingival_plaque
SRR2086901	MGYA00143991	https://www.ebi.ac.uk/metagenomics/api/v1/analyses/MGYA00143991/file/SRR2086901_MERGED_FASTQ_SSU_OTU.tsv	Root-Host_associated-Human-Digestive_system-Oral-Subgingival_plaque
SRR6239926	MGYA00168164	https://www.ebi.ac.uk/metagenomics/api/v1/analyses/MGYA00168164/file/SRR6239926_FASTQ_SSU_OTU.tsv	Root-Host_associated-Human-Digestive_system-Oral-Subgingival_plaque

SRR3381583	MGYA00151185	https://www.ebi.ac.uk/metagenomics/api/v1/analyses/MGYA00151185/file/SRR3381583_FASTQ_SSU_OTU.tsv	Root-Host_associated-Human-Digestive_system-Oral-Subgingival_plaque
SRR3381552	MGYA00151156	https://www.ebi.ac.uk/metagenomics/api/v1/analyses/MGYA00151156/file/SRR3381552_FASTQ_SSU_OTU.tsv	Root-Host_associated-Human-Digestive_system-Oral-Subgingival_plaque
SRR2154969	MGYA00151046	https://www.ebi.ac.uk/metagenomics/api/v1/analyses/MGYA00151046/file/SRR2154969_FASTQ_SSU_OTU.tsv	Root-Host_associated-Human-Digestive_system-Oral-Subgingival_plaque
SRR2086857	MGYA00143851	https://www.ebi.ac.uk/metagenomics/api/v1/analyses/MGYA00143851/file/SRR2086857_MERGED_FASTQ_SSU_OTU.tsv	Root-Host_associated-Human-Digestive_system-Oral-Subgingival_plaque
SRR5178108	MGYA00148795	https://www.ebi.ac.uk/metagenomics/api/v1/analyses/MGYA00148795/file/SRR5178108_MERGED_FASTQ_SSU_OTU.tsv	Root-Host_associated-Human-Digestive_system-Oral-Subgingival_plaque
SRR2086886	MGYA00143930	https://www.ebi.ac.uk/metagenomics/api/v1/analyses/MGYA00143930/file/SRR2086886_MERGED_FASTQ_SSU_OTU.tsv	Root-Host_associated-Human-Digestive_system-Oral-Subgingival_plaque
SRR2086732	MGYA00143936	https://www.ebi.ac.uk/metagenomics/api/v1/analyses/MGYA00143936/file/SRR2086732_MERGED_FASTQ_SSU_OTU.tsv	Root-Host_associated-Human-Digestive_system-Oral-Subgingival_plaque
SRR5178081	MGYA00148769	https://www.ebi.ac.uk/metagenomics/api/v1/analyses/MGYA00148769/file/SRR5178081_MERGED_FASTQ_SSU_OTU.tsv	Root-Host_associated-Human-Digestive_system-Oral-Subgingival_plaque
SRR2155030	MGYA00151022	https://www.ebi.ac.uk/metagenomics/api/v1/analyses/MGYA00151022/file/SRR2155030_FASTQ_SSU_OTU.tsv	Root-Host_associated-Human-Digestive_system-Oral-Subgingival_plaque
SRR1715602	MGYA00148906	https://www.ebi.ac.uk/metagenomics/api/v1/analyses/MGYA00148906/file/SRR1715602_FASTQ_SSU_OTU.tsv	Root-Host_associated-Human-Digestive_system-Oral-Subgingival_plaque
SRR2154915	MGYA00151048	https://www.ebi.ac.uk/metagenomics/api/v1/analyses/MGYA00151048/file/SRR2154915_FASTQ_SSU_OTU.tsv	Root-Host_associated-Human-Digestive_system-Oral-Subgingival_plaque
SRR2086850	MGYA00144027	https://www.ebi.ac.uk/metagenomics/api/v1/analyses/MGYA00144027/file/SRR2086850_MERGED_FASTQ_SSU_OTU.tsv	Root-Host_associated-Human-Digestive_system-Oral-Subgingival_plaque

SRR2086881	MGYA00143949	https://www.ebi.ac.uk/metagenomics/api/v1/analyses/MGYA00143949/file/SRR2086881_MERGED_FASTQ_SSU_OTU.tsv	Root-Host_associated-Human-Digestive_system-Oral-Subgingival_plaque
SRR2086735	MGYA00143843	https://www.ebi.ac.uk/metagenomics/api/v1/analyses/MGYA00143843/file/SRR2086735_MERGED_FASTQ_SSU_OTU.tsv	Root-Host_associated-Human-Digestive_system-Oral-Subgingival_plaque
SRR5178086	MGYA00148775	https://www.ebi.ac.uk/metagenomics/api/v1/analyses/MGYA00148775/file/SRR5178086_MERGED_FASTQ_SSU_OTU.tsv	Root-Host_associated-Human-Digestive_system-Oral-Subgingival_plaque
SRR2155037	MGYA00151056	https://www.ebi.ac.uk/metagenomics/api/v1/analyses/MGYA00151056/file/SRR2155037_FASTQ_SSU_OTU.tsv	Root-Host_associated-Human-Digestive_system-Oral-Subgingival_plaque
SRR1715605	MGYA00148938	https://www.ebi.ac.uk/metagenomics/api/v1/analyses/MGYA00148938/file/SRR1715605_FASTQ_SSU_OTU.tsv	Root-Host_associated-Human-Digestive_system-Oral-Subgingival_plaque
SRR2154912	MGYA00151063	https://www.ebi.ac.uk/metagenomics/api/v1/analyses/MGYA00151063/file/SRR2154912_FASTQ_SSU_OTU.tsv	Root-Host_associated-Human-Digestive_system-Oral-Subgingival_plaque
SRR2086908	MGYA00144040	https://www.ebi.ac.uk/metagenomics/api/v1/analyses/MGYA00144040/file/SRR2086908_MERGED_FASTQ_SSU_OTU.tsv	Root-Host_associated-Human-Digestive_system-Oral-Subgingival_plaque
SRR2086798	MGYA00143994	https://www.ebi.ac.uk/metagenomics/api/v1/analyses/MGYA00143994/file/SRR2086798_MERGED_FASTQ_SSU_OTU.tsv	Root-Host_associated-Human-Digestive_system-Oral-Subgingival_plaque
SRR6239921	MGYA00168170	https://www.ebi.ac.uk/metagenomics/api/v1/analyses/MGYA00168170/file/SRR6239921_FASTQ_SSU_OTU.tsv	Root-Host_associated-Human-Digestive_system-Oral-Subgingival_plaque
SRR2086749	MGYA00143904	https://www.ebi.ac.uk/metagenomics/api/v1/analyses/MGYA00143904/file/SRR2086749_MERGED_FASTQ_SSU_OTU.tsv	Root-Host_associated-Human-Digestive_system-Oral-Subgingival_plaque
SRR3381584	MGYA00151176	https://www.ebi.ac.uk/metagenomics/api/v1/analyses/MGYA00151176/file/SRR3381584_FASTQ_SSU_OTU.tsv	Root-Host_associated-Human-Digestive_system-Oral-Subgingival_plaque
SRR6239879	MGYA00168206	https://www.ebi.ac.uk/metagenomics/api/v1/analyses/MGYA00168206/file/SRR6239879_FASTQ_SSU_OTU.tsv	Root-Host_associated-Human-Digestive_system-Oral-Subgingival_plaque

SRR3381555	MGYA00151166	https://www.ebi.ac.uk/metagenomics/api/v1/analyses/MGYA00151166/file/SRR3381555_FASTQ_SSU_OTU.tsv	Root-Host_associated-Human-Digestive_system-Oral-Subgingival_plaque
SRR2086865	MGYA00143840	https://www.ebi.ac.uk/metagenomics/api/v1/analyses/MGYA00143840/file/SRR2086865_MERGED_FASTQ_SSU_OTU.tsv	Root-Host_associated-Human-Digestive_system-Oral-Subgingival_plaque
SRR2154927	MGYA00151070	https://www.ebi.ac.uk/metagenomics/api/v1/analyses/MGYA00151070/file/SRR2154927_FASTQ_SSU_OTU.tsv	Root-Host_associated-Human-Digestive_system-Oral-Subgingival_plaque
SRR1715630	MGYA00148888	https://www.ebi.ac.uk/metagenomics/api/v1/analyses/MGYA00148888/file/SRR1715630_FASTQ_SSU_OTU.tsv	Root-Host_associated-Human-Digestive_system-Oral-Subgingival_plaque
SRR1745942	MGYA00168151	https://www.ebi.ac.uk/metagenomics/api/v1/analyses/MGYA00168151/file/SRR1745942_FASTQ_SSU_OTU.tsv	Root-Host_associated-Human-Digestive_system-Oral-Subgingival_plaque
SRR2155002	MGYA00151071	https://www.ebi.ac.uk/metagenomics/api/v1/analyses/MGYA00151071/file/SRR2155002_FASTQ_SSU_OTU.tsv	Root-Host_associated-Human-Digestive_system-Oral-Subgingival_plaque
SRR5178146	MGYA00148808	https://www.ebi.ac.uk/metagenomics/api/v1/analyses/MGYA00148808/file/SRR5178146_MERGED_FASTQ_SSU_OTU.tsv	Root-Host_associated-Human-Digestive_system-Oral-Subgingival_plaque
SRR6239914	MGYA00168178	https://www.ebi.ac.uk/metagenomics/api/v1/analyses/MGYA00168178/file/SRR6239914_FASTQ_SSU_OTU.tsv	Root-Host_associated-Human-Digestive_system-Oral-Subgingival_plaque
SRR2086819	MGYA00143959	https://www.ebi.ac.uk/metagenomics/api/v1/analyses/MGYA00143959/file/SRR2086819_MERGED_FASTQ_SSU_OTU.tsv	Root-Host_associated-Human-Digestive_system-Oral-Subgingival_plaque
SRR2086941	MGYA00143927	https://www.ebi.ac.uk/metagenomics/api/v1/analyses/MGYA00143927/file/SRR2086941_MERGED_FASTQ_SSU_OTU.tsv	Root-Host_associated-Human-Digestive_system-Oral-Subgingival_plaque
SRR3381560	MGYA00151179	https://www.ebi.ac.uk/metagenomics/api/v1/analyses/MGYA00151179/file/SRR3381560_FASTQ_SSU_OTU.tsv	Root-Host_associated-Human-Digestive_system-Oral-Subgingival_plaque
SRR6316009	MGYA00293725	https://www.ebi.ac.uk/metagenomics/api/v1/analyses/MGYA00293725/file/SRR6316009_MERGED_FASTQ_SSU_OTU.tsv	Root-Host_associated-Human-Digestive_system-Oral-Subgingival_plaque

SRR5178141	MGYA00148782	https://www.ebi.ac.uk/metagenomics/api/v1/analyses/MGYA00148782/file/SRR5178141_MERGED_FASTQ_SSU_OTU.tsv	Root-Host_associated-Human-Digestive_system-Oral-Subgingival_plaque
SRR6239913	MGYA00168188	https://www.ebi.ac.uk/metagenomics/api/v1/analyses/MGYA00168188/file/SRR6239913_FASTQ_SSU_OTU.tsv	Root-Host_associated-Human-Digestive_system-Oral-Subgingival_plaque
SRR2086946	MGYA00143924	https://www.ebi.ac.uk/metagenomics/api/v1/analyses/MGYA00143924/file/SRR2086946_MERGED_FASTQ_SSU_OTU.tsv	Root-Host_associated-Human-Digestive_system-Oral-Subgingival_plaque
DRR049488	MGYA00160359	https://www.ebi.ac.uk/metagenomics/api/v1/analyses/MGYA00160359/file/DRR049488_MERGED_FASTQ_SSU_OTU.tsv	Root-Host_associated-Human-Digestive_system-Oral-Subgingival_plaque
SRR1745939	MGYA00168144	https://www.ebi.ac.uk/metagenomics/api/v1/analyses/MGYA00168144/file/SRR1745939_FASTQ_SSU_OTU.tsv	Root-Host_associated-Human-Digestive_system-Oral-Subgingival_plaque
DRR049459	MGYA00160371	https://www.ebi.ac.uk/metagenomics/api/v1/analyses/MGYA00160371/file/DRR049459_MERGED_FASTQ_SSU_OTU.tsv	Root-Host_associated-Human-Digestive_system-Oral-Subgingival_plaque
SRR2086862	MGYA00143848	https://www.ebi.ac.uk/metagenomics/api/v1/analyses/MGYA00143848/file/SRR2086862_MERGED_FASTQ_SSU_OTU.tsv	Root-Host_associated-Human-Digestive_system-Oral-Subgingival_plaque
SRR2154920	MGYA00151013	https://www.ebi.ac.uk/metagenomics/api/v1/analyses/MGYA00151013/file/SRR2154920_FASTQ_SSU_OTU.tsv	Root-Host_associated-Human-Digestive_system-Oral-Subgingival_plaque
SRR1715637	MGYA00148944	https://www.ebi.ac.uk/metagenomics/api/v1/analyses/MGYA00148944/file/SRR1715637_FASTQ_SSU_OTU.tsv	Root-Host_associated-Human-Digestive_system-Oral-Subgingival_plaque
SRR1745945	MGYA00168146	https://www.ebi.ac.uk/metagenomics/api/v1/analyses/MGYA00168146/file/SRR1745945_FASTQ_SSU_OTU.tsv	Root-Host_associated-Human-Digestive_system-Oral-Subgingival_plaque
SRR2155005	MGYA00151090	https://www.ebi.ac.uk/metagenomics/api/v1/analyses/MGYA00151090/file/SRR2155005_FASTQ_SSU_OTU.tsv	Root-Host_associated-Human-Digestive_system-Oral-Subgingival_plaque
SRR2086775	MGYA00143990	https://www.ebi.ac.uk/metagenomics/api/v1/analyses/MGYA00143990/file/SRR2086775_MERGED_FASTQ_SSU_OTU.tsv	Root-Host_associated-Human-Digestive_system-Oral-Subgingival_plaque

SRR2086810	MGYA00144004	https://www.ebi.ac.uk/metagenomics/api/v1/analyses/MGYA00144004/file/SRR2086810_MERGED_FASTQ_SSU_OTU.tsv	Root-Host_associated-Human-Digestive_system-Oral-Subgingival_plaque
SRR1715583	MGYA00148941	https://www.ebi.ac.uk/metagenomics/api/v1/analyses/MGYA00148941/file/SRR1715583_FASTQ_SSU_OTU.tsv	Root-Host_associated-Human-Digestive_system-Oral-Subgingival_plaque
DRR049486	MGYA00160362	https://www.ebi.ac.uk/metagenomics/api/v1/analyses/MGYA00160362/file/DRR049486_MERGED_FASTQ_SSU_OTU.tsv	Root-Host_associated-Human-Digestive_system-Oral-Subgingival_plaque
SRR2154952	MGYA00151084	https://www.ebi.ac.uk/metagenomics/api/v1/analyses/MGYA00151084/file/SRR2154952_FASTQ_SSU_OTU.tsv	Root-Host_associated-Human-Digestive_system-Oral-Subgingival_plaque
SRR3381569	MGYA00151154	https://www.ebi.ac.uk/metagenomics/api/v1/analyses/MGYA00151154/file/SRR3381569_FASTQ_SSU_OTU.tsv	Root-Host_associated-Human-Digestive_system-Oral-Subgingival_plaque
SRR2086948	MGYA00143932	https://www.ebi.ac.uk/metagenomics/api/v1/analyses/MGYA00143932/file/SRR2086948_MERGED_FASTQ_SSU_OTU.tsv	Root-Host_associated-Human-Digestive_system-Oral-Subgingival_plaque
SRR1715645	MGYA00148968	https://www.ebi.ac.uk/metagenomics/api/v1/analyses/MGYA00148968/file/SRR1715645_FASTQ_SSU_OTU.tsv	Root-Host_associated-Human-Digestive_system-Oral-Subgingival_plaque
SRR3992170	MGYA00151249	https://www.ebi.ac.uk/metagenomics/api/v1/analyses/MGYA00151249/file/SRR3992170_FASTQ_SSU_OTU.tsv	Root-Host_associated-Human-Digestive_system-Oral-Subgingival_plaque
SRR1745937	MGYA00168148	https://www.ebi.ac.uk/metagenomics/api/v1/analyses/MGYA00168148/file/SRR1745937_FASTQ_SSU_OTU.tsv	Root-Host_associated-Human-Digestive_system-Oral-Subgingival_plaque
SRR2154983	MGYA00151061	https://www.ebi.ac.uk/metagenomics/api/v1/analyses/MGYA00151061/file/SRR2154983_FASTQ_SSU_OTU.tsv	Root-Host_associated-Human-Digestive_system-Oral-Subgingival_plaque
SRR6239894	MGYA00168160	https://www.ebi.ac.uk/metagenomics/api/v1/analyses/MGYA00168160/file/SRR6239894_FASTQ_SSU_OTU.tsv	Root-Host_associated-Human-Digestive_system-Oral-Subgingival_plaque
DRR049457	MGYA00160330	https://www.ebi.ac.uk/metagenomics/api/v1/analyses/MGYA00160330/file/DRR049457_MERGED_FASTQ_SSU_OTU.tsv	Root-Host_associated-Human-Digestive_system-Oral-Subgingival_plaque

SRR5178133	MGYA00148807	https://www.ebi.ac.uk/metagenomics/api/v1/analyses/MGYA00148807/file/SRR5178133_MERGED_FASTQ_SSU_OTU.tsv	Root-Host_associated-Human-Digestive_system-Oral-Subgingival_plaque
SRR2086934	MGYA00143856	https://www.ebi.ac.uk/metagenomics/api/v1/analyses/MGYA00143856/file/SRR2086934_MERGED_FASTQ_SSU_OTU.tsv	Root-Host_associated-Human-Digestive_system-Oral-Subgingival_plaque
SRR1715639	MGYA00148890	https://www.ebi.ac.uk/metagenomics/api/v1/analyses/MGYA00148890/file/SRR1715639_FASTQ_SSU_OTU.tsv	Root-Host_associated-Human-Digestive_system-Oral-Subgingival_plaque
SRR5178134	MGYA00148741	https://www.ebi.ac.uk/metagenomics/api/v1/analyses/MGYA00148741/file/SRR5178134_MERGED_FASTQ_SSU_OTU.tsv	Root-Host_associated-Human-Digestive_system-Oral-Subgingival_plaque
SRR2086933	MGYA00144023	https://www.ebi.ac.uk/metagenomics/api/v1/analyses/MGYA00144023/file/SRR2086933_MERGED_FASTQ_SSU_OTU.tsv	Root-Host_associated-Human-Digestive_system-Oral-Subgingival_plaque
SRR2154929	MGYA00151033	https://www.ebi.ac.uk/metagenomics/api/v1/analyses/MGYA00151033/file/SRR2154929_FASTQ_SSU_OTU.tsv	Root-Host_associated-Human-Digestive_system-Oral-Subgingival_plaque
SRR2086772	MGYA00143828	https://www.ebi.ac.uk/metagenomics/api/v1/analyses/MGYA00143828/file/SRR2086772_MERGED_FASTQ_SSU_OTU.tsv	Root-Host_associated-Human-Digestive_system-Oral-Subgingival_plaque
SRR2086817	MGYA00143983	https://www.ebi.ac.uk/metagenomics/api/v1/analyses/MGYA00143983/file/SRR2086817_MERGED_FASTQ_SSU_OTU.tsv	Root-Host_associated-Human-Digestive_system-Oral-Subgingival_plaque
SRR5178148	MGYA00148781	https://www.ebi.ac.uk/metagenomics/api/v1/analyses/MGYA00148781/file/SRR5178148_MERGED_FASTQ_SSU_OTU.tsv	Root-Host_associated-Human-Digestive_system-Oral-Subgingival_plaque
SRR1715584	MGYA00148956	https://www.ebi.ac.uk/metagenomics/api/v1/analyses/MGYA00148956/file/SRR1715584_FASTQ_SSU_OTU.tsv	Root-Host_associated-Human-Digestive_system-Oral-Subgingival_plaque
DRR049481	MGYA00160363	https://www.ebi.ac.uk/metagenomics/api/v1/analyses/MGYA00160363/file/DRR049481_MERGED_FASTQ_SSU_OTU.tsv	Root-Host_associated-Human-Digestive_system-Oral-Subgingival_plaque
SRR2154955	MGYA00150956	https://www.ebi.ac.uk/metagenomics/api/v1/analyses/MGYA00150956/file/SRR2154955_FASTQ_SSU_OTU.tsv	Root-Host_associated-Human-Digestive_system-Oral-Subgingival_plaque

SRR1715642	MGYA00148967	https://www.ebi.ac.uk/metagenomics/api/v1/analyses/MGYA00148967/file/SRR1715642_FASTQ_SSU_OTU.tsv	Root-Host_associated-Human-Digestive_system-Oral-Subgingival_plaque
SRR1745930	MGYA00168139	https://www.ebi.ac.uk/metagenomics/api/v1/analyses/MGYA00168139/file/SRR1745930_FASTQ_SSU_OTU.tsv	Root-Host_associated-Human-Digestive_system-Oral-Subgingival_plaque
SRR6316007	MGYA00293723	https://www.ebi.ac.uk/metagenomics/api/v1/analyses/MGYA00293723/file/SRR6316007_MERGED_FASTQ_SSU_OTU.tsv	Root-Host_associated-Human-Digestive_system-Oral-Subgingival_plaque
SRR2154984	MGYA00151087	https://www.ebi.ac.uk/metagenomics/api/v1/analyses/MGYA00151087/file/SRR2154984_FASTQ_SSU_OTU.tsv	Root-Host_associated-Human-Digestive_system-Oral-Subgingival_plaque
SRR6239893	MGYA00168176	https://www.ebi.ac.uk/metagenomics/api/v1/analyses/MGYA00168176/file/SRR6239893_FASTQ_SSU_OTU.tsv	Root-Host_associated-Human-Digestive_system-Oral-Subgingival_plaque
SRR2086922	MGYA00143941	https://www.ebi.ac.uk/metagenomics/api/v1/analyses/MGYA00143941/file/SRR2086922_MERGED_FASTQ_SSU_OTU.tsv	Root-Host_associated-Human-Digestive_system-Oral-Subgingival_plaque
SRR2154938	MGYA00150968	https://www.ebi.ac.uk/metagenomics/api/v1/analyses/MGYA00150968/file/SRR2154938_FASTQ_SSU_OTU.tsv	Root-Host_associated-Human-Digestive_system-Oral-Subgingival_plaque
SRR5178125	MGYA00148772	https://www.ebi.ac.uk/metagenomics/api/v1/analyses/MGYA00148772/file/SRR5178125_MERGED_FASTQ_SSU_OTU.tsv	Root-Host_associated-Human-Digestive_system-Oral-Subgingival_plaque
SRR2154995	MGYA00150961	https://www.ebi.ac.uk/metagenomics/api/v1/analyses/MGYA00150961/file/SRR2154995_FASTQ_SSU_OTU.tsv	Root-Host_associated-Human-Digestive_system-Oral-Subgingival_plaque
SRR6239882	MGYA00168212	https://www.ebi.ac.uk/metagenomics/api/v1/analyses/MGYA00168212/file/SRR6239882_FASTQ_SSU_OTU.tsv	Root-Host_associated-Human-Digestive_system-Oral-Subgingival_plaque
SRR2154944	MGYA00151091	https://www.ebi.ac.uk/metagenomics/api/v1/analyses/MGYA00151091/file/SRR2154944_FASTQ_SSU_OTU.tsv	Root-Host_associated-Human-Digestive_system-Oral-Subgingival_plaque
SRR1715595	MGYA00148899	https://www.ebi.ac.uk/metagenomics/api/v1/analyses/MGYA00148899/file/SRR1715595_FASTQ_SSU_OTU.tsv	Root-Host_associated-Human-Digestive_system-Oral-Subgingival_plaque

DRR049490	MGYA00160341	https://www.ebi.ac.uk/metagenomics/api/v1/analyses/MGYA00160341/file/DRR049490_MERGED_FASTQ_SSU_OTU.tsv	Root-Host_associated-Human-Digestive_system-Oral-Subgingival_plaque
SRR3992166	MGYA00151248	https://www.ebi.ac.uk/metagenomics/api/v1/analyses/MGYA00151248/file/SRR3992166_FASTQ_SSU_OTU.tsv	Root-Host_associated-Human-Digestive_system-Oral-Subgingival_plaque
SRR6316016	MGYA00293732	https://www.ebi.ac.uk/metagenomics/api/v1/analyses/MGYA00293732/file/SRR6316016_MERGED_FASTQ_SSU_OTU.tsv	Root-Host_associated-Human-Digestive_system-Oral-Subgingival_plaque
SRR1715653	MGYA00148883	https://www.ebi.ac.uk/metagenomics/api/v1/analyses/MGYA00148883/file/SRR1715653_FASTQ_SSU_OTU.tsv	Root-Host_associated-Human-Digestive_system-Oral-Subgingival_plaque
SRR5178159	MGYA00148796	https://www.ebi.ac.uk/metagenomics/api/v1/analyses/MGYA00148796/file/SRR5178159_MERGED_FASTQ_SSU_OTU.tsv	Root-Host_associated-Human-Digestive_system-Oral-Subgingival_plaque
SRR2086806	MGYA00144038	https://www.ebi.ac.uk/metagenomics/api/v1/analyses/MGYA00144038/file/SRR2086806_MERGED_FASTQ_SSU_OTU.tsv	Root-Host_associated-Human-Digestive_system-Oral-Subgingival_plaque
SRR2086763	MGYA00144036	https://www.ebi.ac.uk/metagenomics/api/v1/analyses/MGYA00144036/file/SRR2086763_MERGED_FASTQ_SSU_OTU.tsv	Root-Host_associated-Human-Digestive_system-Oral-Subgingival_plaque
SRR2154992	MGYA00151040	https://www.ebi.ac.uk/metagenomics/api/v1/analyses/MGYA00151040/file/SRR2154992_FASTQ_SSU_OTU.tsv	Root-Host_associated-Human-Digestive_system-Oral-Subgingival_plaque
SRR6239885	MGYA00168195	https://www.ebi.ac.uk/metagenomics/api/v1/analyses/MGYA00168195/file/SRR6239885_FASTQ_SSU_OTU.tsv	Root-Host_associated-Human-Digestive_system-Oral-Subgingival_plaque
SRR2154943	MGYA00150991	https://www.ebi.ac.uk/metagenomics/api/v1/analyses/MGYA00150991/file/SRR2154943_FASTQ_SSU_OTU.tsv	Root-Host_associated-Human-Digestive_system-Oral-Subgingival_plaque
SRR3381578	MGYA00151177	https://www.ebi.ac.uk/metagenomics/api/v1/analyses/MGYA00151177/file/SRR3381578_FASTQ_SSU_OTU.tsv	Root-Host_associated-Human-Digestive_system-Oral-Subgingival_plaque
SRR1715592	MGYA00148936	https://www.ebi.ac.uk/metagenomics/api/v1/analyses/MGYA00148936/file/SRR1715592_FASTQ_SSU_OTU.tsv	Root-Host_associated-Human-Digestive_system-Oral-Subgingival_plaque

DRR049497	MGYA00160358	https://www.ebi.ac.uk/metagenomics/api/v1/analyses/MGYA00160358/file/DRR049497_MERGED_FASTQ_SSU_OTU.tsv	Root-Host_associated-Human-Digestive_system-Oral-Subgingival_plaque
SRR6316011	MGYA00293727	https://www.ebi.ac.uk/metagenomics/api/v1/analyses/MGYA00293727/file/SRR6316011_MERGED_FASTQ_SSU_OTU.tsv	Root-Host_associated-Human-Digestive_system-Oral-Subgingival_plaque
SRR1745926	MGYA00168138	https://www.ebi.ac.uk/metagenomics/api/v1/analyses/MGYA00168138/file/SRR1745926_FASTQ_SSU_OTU.tsv	Root-Host_associated-Human-Digestive_system-Oral-Subgingival_plaque
SRR1715654	MGYA00148961	https://www.ebi.ac.uk/metagenomics/api/v1/analyses/MGYA00148961/file/SRR1715654_FASTQ_SSU_OTU.tsv	Root-Host_associated-Human-Digestive_system-Oral-Subgingival_plaque
SRR2086801	MGYA00144021	https://www.ebi.ac.uk/metagenomics/api/v1/analyses/MGYA00144021/file/SRR2086801_MERGED_FASTQ_SSU_OTU.tsv	Root-Host_associated-Human-Digestive_system-Oral-Subgingival_plaque
SRR1178323	MGYA00151887	https://www.ebi.ac.uk/metagenomics/api/v1/analyses/MGYA00151887/file/SRR1178323_FASTQ_SSU_OTU.tsv	Root-Host_associated-Human-Digestive_system-Oral-Subgingival_plaque
SRR2086764	MGYA00143967	https://www.ebi.ac.uk/metagenomics/api/v1/analyses/MGYA00143967/file/SRR2086764_MERGED_FASTQ_SSU_OTU.tsv	Root-Host_associated-Human-Digestive_system-Oral-Subgingival_plaque
SRR2086925	MGYA00144034	https://www.ebi.ac.uk/metagenomics/api/v1/analyses/MGYA00144034/file/SRR2086925_MERGED_FASTQ_SSU_OTU.tsv	Root-Host_associated-Human-Digestive_system-Oral-Subgingival_plaque
SRR1715628	MGYA00148955	https://www.ebi.ac.uk/metagenomics/api/v1/analyses/MGYA00148955/file/SRR1715628_FASTQ_SSU_OTU.tsv	Root-Host_associated-Human-Digestive_system-Oral-Subgingival_plaque
SRR5178122	MGYA00148728	https://www.ebi.ac.uk/metagenomics/api/v1/analyses/MGYA00148728/file/SRR5178122_MERGED_FASTQ_SSU_OTU.tsv	Root-Host_associated-Human-Digestive_system-Oral-Subgingival_plaque
DRR049499	MGYA00160361	https://www.ebi.ac.uk/metagenomics/api/v1/analyses/MGYA00160361/file/DRR049499_MERGED_FASTQ_SSU_OTU.tsv	Root-Host_associated-Human-Digestive_system-Oral-Subgingival_plaque
SRR2086957	MGYA00143881	https://www.ebi.ac.uk/metagenomics/api/v1/analyses/MGYA00143881/file/SRR2086957_MERGED_FASTQ_SSU_OTU.tsv	Root-Host_associated-Human-Digestive_system-Oral-Subgingival_plaque

SRR3381576	MGYA00151188	https://www.ebi.ac.uk/metagenomics/api/v1/analyses/MGYA00151188/file/SRR3381576_FASTQ_SSU_OTU.tsv	Root-Host_associated-Human-Digestive_system-Oral-Subgingival_plaque
SRR1745928	MGYA00168145	https://www.ebi.ac.uk/metagenomics/api/v1/analyses/MGYA00168145/file/SRR1745928_FASTQ_SSU_OTU.tsv	Root-Host_associated-Human-Digestive_system-Oral-Subgingival_plaque
SRR6239902	MGYA00168211	https://www.ebi.ac.uk/metagenomics/api/v1/analyses/MGYA00168211/file/SRR6239902_FASTQ_SSU_OTU.tsv	Root-Host_associated-Human-Digestive_system-Oral-Subgingival_plaque
SRR5178150	MGYA00148793	https://www.ebi.ac.uk/metagenomics/api/v1/analyses/MGYA00148793/file/SRR5178150_MERGED_FASTQ_SSU_OTU.tsv	Root-Host_associated-Human-Digestive_system-Oral-Subgingival_plaque
SRR2155014	MGYA00150996	https://www.ebi.ac.uk/metagenomics/api/v1/analyses/MGYA00150996/file/SRR2155014_FASTQ_SSU_OTU.tsv	Root-Host_associated-Human-Digestive_system-Oral-Subgingival_plaque
SRR2154931	MGYA00150971	https://www.ebi.ac.uk/metagenomics/api/v1/analyses/MGYA00150971/file/SRR2154931_FASTQ_SSU_OTU.tsv	Root-Host_associated-Human-Digestive_system-Oral-Subgingival_plaque
SRR5178074	MGYA00148804	https://www.ebi.ac.uk/metagenomics/api/v1/analyses/MGYA00148804/file/SRR5178074_MERGED_FASTQ_SSU_OTU.tsv	Root-Host_associated-Human-Digestive_system-Oral-Subgingival_plaque
SRR1715626	MGYA00148927	https://www.ebi.ac.uk/metagenomics/api/v1/analyses/MGYA00148927/file/SRR1715626_FASTQ_SSU_OTU.tsv	Root-Host_associated-Human-Digestive_system-Oral-Subgingival_plaque
SRR2086873	MGYA00143977	https://www.ebi.ac.uk/metagenomics/api/v1/analyses/MGYA00143977/file/SRR2086873_MERGED_FASTQ_SSU_OTU.tsv	Root-Host_associated-Human-Digestive_system-Oral-Subgingival_plaque
SRR2155013	MGYA00150986	https://www.ebi.ac.uk/metagenomics/api/v1/analyses/MGYA00150986/file/SRR2155013_FASTQ_SSU_OTU.tsv	Root-Host_associated-Human-Digestive_system-Oral-Subgingival_plaque
SRR2154936	MGYA00151089	https://www.ebi.ac.uk/metagenomics/api/v1/analyses/MGYA00151089/file/SRR2154936_FASTQ_SSU_OTU.tsv	Root-Host_associated-Human-Digestive_system-Oral-Subgingival_plaque
SRR5178073	MGYA00148805	https://www.ebi.ac.uk/metagenomics/api/v1/analyses/MGYA00148805/file/SRR5178073_MERGED_FASTQ_SSU_OTU.tsv	Root-Host_associated-Human-Digestive_system-Oral-Subgingival_plaque

SRR1715621	MGYA00148908	https://www.ebi.ac.uk/metagenomics/api/v1/analyses/MGYA00148908/file/SRR1715621_FASTQ_SSU_OTU.tsv	Root-Host_associated-Human-Digestive_system-Oral-Subgingival_plaque
SRR2086874	MGYA00144002	https://www.ebi.ac.uk/metagenomics/api/v1/analyses/MGYA00144002/file/SRR2086874_MERGED_FASTQ_SSU_OTU.tsv	Root-Host_associated-Human-Digestive_system-Oral-Subgingival_plaque
SRR3381571	MGYA00151182	https://www.ebi.ac.uk/metagenomics/api/v1/analyses/MGYA00151182/file/SRR3381571_FASTQ_SSU_OTU.tsv	Root-Host_associated-Human-Digestive_system-Oral-Subgingival_plaque
SRR2086950	MGYA00143987	https://www.ebi.ac.uk/metagenomics/api/v1/analyses/MGYA00143987/file/SRR2086950_MERGED_FASTQ_SSU_OTU.tsv	Root-Host_associated-Human-Digestive_system-Oral-Subgingival_plaque
SRR6316018	MGYA00293734	https://www.ebi.ac.uk/metagenomics/api/v1/analyses/MGYA00293734/file/SRR6316018_MERGED_FASTQ_SSU_OTU.tsv	Root-Host_associated-Human-Digestive_system-Oral-Subgingival_plaque
SRR3992168	MGYA00151243	https://www.ebi.ac.uk/metagenomics/api/v1/analyses/MGYA00151243/file/SRR3992168_FASTQ_SSU_OTU.tsv	Root-Host_associated-Human-Digestive_system-Oral-Subgingival_plaque
SRR6239905	MGYA00168205	https://www.ebi.ac.uk/metagenomics/api/v1/analyses/MGYA00168205/file/SRR6239905_FASTQ_SSU_OTU.tsv	Root-Host_associated-Human-Digestive_system-Oral-Subgingival_plaque
SRR2086808	MGYA00143854	https://www.ebi.ac.uk/metagenomics/api/v1/analyses/MGYA00143854/file/SRR2086808_MERGED_FASTQ_SSU_OTU.tsv	Root-Host_associated-Human-Digestive_system-Oral-Subgingival_plaque
SRR5178157	MGYA00148745	https://www.ebi.ac.uk/metagenomics/api/v1/analyses/MGYA00148745/file/SRR5178157_MERGED_FASTQ_SSU_OTU.tsv	Root-Host_associated-Human-Digestive_system-Oral-Subgingival_plaque
SRR1715614	MGYA00148895	https://www.ebi.ac.uk/metagenomics/api/v1/analyses/MGYA00148895/file/SRR1715614_FASTQ_SSU_OTU.tsv	Root-Host_associated-Human-Digestive_system-Oral-Subgingival_plaque
SRR2154903	MGYA00151094	https://www.ebi.ac.uk/metagenomics/api/v1/analyses/MGYA00151094/file/SRR2154903_FASTQ_SSU_OTU.tsv	Root-Host_associated-Human-Digestive_system-Oral-Subgingival_plaque
SRR2086919	MGYA00143943	https://www.ebi.ac.uk/metagenomics/api/v1/analyses/MGYA00143943/file/SRR2086919_MERGED_FASTQ_SSU_OTU.tsv	Root-Host_associated-Human-Digestive_system-Oral-Subgingival_plaque

SRR5178097	MGYA00148714	https://www.ebi.ac.uk/metagenomics/api/v1/analyses/MGYA00148714/file/SRR5178097_MERGED_FASTQ_SSU_OTU.tsv	Root-Host_associated-Human-Digestive_system-Oral-Subgingival_plaque
SRR2155026	MGYA00150998	https://www.ebi.ac.uk/metagenomics/api/v1/analyses/MGYA00150998/file/SRR2155026_FASTQ_SSU_OTU.tsv	Root-Host_associated-Human-Digestive_system-Oral-Subgingival_plaque
SRR2086890	MGYA00143945	https://www.ebi.ac.uk/metagenomics/api/v1/analyses/MGYA00143945/file/SRR2086890_MERGED_FASTQ_SSU_OTU.tsv	Root-Host_associated-Human-Digestive_system-Oral-Subgingival_plaque
SRR2086724	MGYA00143914	https://www.ebi.ac.uk/metagenomics/api/v1/analyses/MGYA00143914/file/SRR2086724_MERGED_FASTQ_SSU_OTU.tsv	Root-Host_associated-Human-Digestive_system-Oral-Subgingival_plaque
SRR2086841	MGYA00143871	https://www.ebi.ac.uk/metagenomics/api/v1/analyses/MGYA00143871/file/SRR2086841_MERGED_FASTQ_SSU_OTU.tsv	Root-Host_associated-Human-Digestive_system-Oral-Subgingival_plaque
SRR1715668	MGYA00148911	https://www.ebi.ac.uk/metagenomics/api/v1/analyses/MGYA00148911/file/SRR1715668_FASTQ_SSU_OTU.tsv	Root-Host_associated-Human-Digestive_system-Oral-Subgingival_plaque
SRR6239868	MGYA00168166	https://www.ebi.ac.uk/metagenomics/api/v1/analyses/MGYA00168166/file/SRR6239868_FASTQ_SSU_OTU.tsv	Root-Host_associated-Human-Digestive_system-Oral-Subgingival_plaque
SRR2086758	MGYA00144041	https://www.ebi.ac.uk/metagenomics/api/v1/analyses/MGYA00144041/file/SRR2086758_MERGED_FASTQ_SSU_OTU.tsv	Root-Host_associated-Human-Digestive_system-Oral-Subgingival_plaque
SRR2086789	MGYA00144031	https://www.ebi.ac.uk/metagenomics/api/v1/analyses/MGYA00144031/file/SRR2086789_MERGED_FASTQ_SSU_OTU.tsv	Root-Host_associated-Human-Digestive_system-Oral-Subgingival_plaque
SRR5178162	MGYA00148715	https://www.ebi.ac.uk/metagenomics/api/v1/analyses/MGYA00148715/file/SRR5178162_MERGED_FASTQ_SSU_OTU.tsv	Root-Host_associated-Human-Digestive_system-Oral-Subgingival_plaque
SRR2154978	MGYA00150977	https://www.ebi.ac.uk/metagenomics/api/v1/analyses/MGYA00150977/file/SRR2154978_FASTQ_SSU_OTU.tsv	Root-Host_associated-Human-Digestive_system-Oral-Subgingival_plaque
SRR1715578	MGYA00148902	https://www.ebi.ac.uk/metagenomics/api/v1/analyses/MGYA00148902/file/SRR1715578_FASTQ_SSU_OTU.tsv	Root-Host_associated-Human-Digestive_system-Oral-Subgingival_plaque

SRR5178165	MGYA00148716	https://www.ebi.ac.uk/metagenomics/api/v1/analyses/MGYA00148716/file/SRR5178165_MERGED_FASTQ_SSU_OTU.tsv	Root-Host_associated-Human-Digestive_system-Oral-Subgingival_plaque
SRR1715613	MGYA00148881	https://www.ebi.ac.uk/metagenomics/api/v1/analyses/MGYA00148881/file/SRR1715613_FASTQ_SSU_OTU.tsv	Root-Host_associated-Human-Digestive_system-Oral-Subgingival_plaque
SRR2154904	MGYA00150995	https://www.ebi.ac.uk/metagenomics/api/v1/analyses/MGYA00150995/file/SRR2154904_FASTQ_SSU_OTU.tsv	Root-Host_associated-Human-Digestive_system-Oral-Subgingival_plaque
SRR5178090	MGYA00148766	https://www.ebi.ac.uk/metagenomics/api/v1/analyses/MGYA00148766/file/SRR5178090_MERGED_FASTQ_SSU_OTU.tsv	Root-Host_associated-Human-Digestive_system-Oral-Subgingival_plaque
SRR2155021	MGYA00151075	https://www.ebi.ac.uk/metagenomics/api/v1/analyses/MGYA00151075/file/SRR2155021_FASTQ_SSU_OTU.tsv	Root-Host_associated-Human-Digestive_system-Oral-Subgingival_plaque
SRR2086723	MGYA00143916	https://www.ebi.ac.uk/metagenomics/api/v1/analyses/MGYA00143916/file/SRR2086723_MERGED_FASTQ_SSU_OTU.tsv	Root-Host_associated-Human-Digestive_system-Oral-Subgingival_plaque
SRR5178119	MGYA00148765	https://www.ebi.ac.uk/metagenomics/api/v1/analyses/MGYA00148765/file/SRR5178119_MERGED_FASTQ_SSU_OTU.tsv	Root-Host_associated-Human-Digestive_system-Oral-Subgingival_plaque
SRR2086846	MGYA00143917	https://www.ebi.ac.uk/metagenomics/api/v1/analyses/MGYA00143917/file/SRR2086846_MERGED_FASTQ_SSU_OTU.tsv	Root-Host_associated-Human-Digestive_system-Oral-Subgingival_plaque
SRR6316024	MGYA00293740	https://www.ebi.ac.uk/metagenomics/api/v1/analyses/MGYA00293740/file/SRR6316024_MERGED_FASTQ_SSU_OTU.tsv	Root-Host_associated-Human-Digestive_system-Oral-Subgingival_plaque
SRR1715661	MGYA00148939	https://www.ebi.ac.uk/metagenomics/api/v1/analyses/MGYA00148939/file/SRR1715661_FASTQ_SSU_OTU.tsv	Root-Host_associated-Human-Digestive_system-Oral-Subgingival_plaque
SRR2154976	MGYA00150958	https://www.ebi.ac.uk/metagenomics/api/v1/analyses/MGYA00150958/file/SRR2154976_FASTQ_SSU_OTU.tsv	Root-Host_associated-Human-Digestive_system-Oral-Subgingival_plaque
SRR6239861	MGYA00168189	https://www.ebi.ac.uk/metagenomics/api/v1/analyses/MGYA00168189/file/SRR6239861_FASTQ_SSU_OTU.tsv	Root-Host_associated-Human-Digestive_system-Oral-Subgingival_plaque

SRR1715576	MGYA00148887	https://www.ebi.ac.uk/metagenomics/api/v1/analyses/MGYA00148887/file/SRR1715576_FASTQ_SSU_OTU.tsv	Root-Host_associated-Human-Digestive_system-Oral-Subgingival_plaque
DRR049473	MGYA00160334	https://www.ebi.ac.uk/metagenomics/api/v1/analyses/MGYA00160334/file/DRR049473_MERGED_FASTQ_SSU_OTU.tsv	Root-Host_associated-Human-Digestive_system-Oral-Subgingival_plaque
SRR2086751	MGYA00143906	https://www.ebi.ac.uk/metagenomics/api/v1/analyses/MGYA00143906/file/SRR2086751_MERGED_FASTQ_SSU_OTU.tsv	Root-Host_associated-Human-Digestive_system-Oral-Subgingival_plaque
SRR2086780	MGYA00143876	https://www.ebi.ac.uk/metagenomics/api/v1/analyses/MGYA00143876/file/SRR2086780_MERGED_FASTQ_SSU_OTU.tsv	Root-Host_associated-Human-Digestive_system-Oral-Subgingival_plaque
SRR2086834	MGYA00143833	https://www.ebi.ac.uk/metagenomics/api/v1/analyses/MGYA00143833/file/SRR2086834_MERGED_FASTQ_SSU_OTU.tsv	Root-Host_associated-Human-Digestive_system-Oral-Subgingival_plaque
SRR2086848	MGYA00143899	https://www.ebi.ac.uk/metagenomics/api/v1/analyses/MGYA00143899/file/SRR2086848_MERGED_FASTQ_SSU_OTU.tsv	Root-Host_associated-Human-Digestive_system-Oral-Subgingival_plaque
SRR5178117	MGYA00148773	https://www.ebi.ac.uk/metagenomics/api/v1/analyses/MGYA00148773/file/SRR5178117_MERGED_FASTQ_SSU_OTU.tsv	Root-Host_associated-Human-Digestive_system-Oral-Subgingival_plaque
SRR2086917	MGYA00143933	https://www.ebi.ac.uk/metagenomics/api/v1/analyses/MGYA00143933/file/SRR2086917_MERGED_FASTQ_SSU_OTU.tsv	Root-Host_associated-Human-Digestive_system-Oral-Subgingival_plaque
SRR5178099	MGYA00148786	https://www.ebi.ac.uk/metagenomics/api/v1/analyses/MGYA00148786/file/SRR5178099_MERGED_FASTQ_SSU_OTU.tsv	Root-Host_associated-Human-Digestive_system-Oral-Subgingival_plaque
SRR2155028	MGYA00151015	https://www.ebi.ac.uk/metagenomics/api/v1/analyses/MGYA00151015/file/SRR2155028_FASTQ_SSU_OTU.tsv	Root-Host_associated-Human-Digestive_system-Oral-Subgingival_plaque
SRR5178110	MGYA00148718	https://www.ebi.ac.uk/metagenomics/api/v1/analyses/MGYA00148718/file/SRR5178110_MERGED_FASTQ_SSU_OTU.tsv	Root-Host_associated-Human-Digestive_system-Oral-Subgingival_plaque
SRR6316023	MGYA00293739	https://www.ebi.ac.uk/metagenomics/api/v1/analyses/MGYA00293739/file/SRR6316023_MERGED_FASTQ_SSU_OTU.tsv	Root-Host_associated-Human-Digestive_system-Oral-Subgingival_plaque

SRR1715666	MGYA00148948	https://www.ebi.ac.uk/metagenomics/api/v1/analyses/MGYA00148948/file/SRR1715666_FASTQ_SSU_OTU.tsv	Root-Host_associated-Human-Digestive_system-Oral-Subgingival_plaque
SRR2154971	MGYA00151019	https://www.ebi.ac.uk/metagenomics/api/v1/analyses/MGYA00151019/file/SRR2154971_FASTQ_SSU_OTU.tsv	Root-Host_associated-Human-Digestive_system-Oral-Subgingival_plaque
SRR6239866	MGYA00168215	https://www.ebi.ac.uk/metagenomics/api/v1/analyses/MGYA00168215/file/SRR6239866_FASTQ_SSU_OTU.tsv	Root-Host_associated-Human-Digestive_system-Oral-Subgingival_plaque
DRR049474	MGYA00160338	https://www.ebi.ac.uk/metagenomics/api/v1/analyses/MGYA00160338/file/DRR049474_MERGED_FASTQ_SSU_OTU.tsv	Root-Host_associated-Human-Digestive_system-Oral-Subgingival_plaque
SRR2086756	MGYA00143946	https://www.ebi.ac.uk/metagenomics/api/v1/analyses/MGYA00143946/file/SRR2086756_MERGED_FASTQ_SSU_OTU.tsv	Root-Host_associated-Human-Digestive_system-Oral-Subgingival_plaque
SRR2086787	MGYA00143884	https://www.ebi.ac.uk/metagenomics/api/v1/analyses/MGYA00143884/file/SRR2086787_MERGED_FASTQ_SSU_OTU.tsv	Root-Host_associated-Human-Digestive_system-Oral-Subgingival_plaque
SRR2086833	MGYA00143918	https://www.ebi.ac.uk/metagenomics/api/v1/analyses/MGYA00143918/file/SRR2086833_MERGED_FASTQ_SSU_OTU.tsv	Root-Host_associated-Human-Digestive_system-Oral-Subgingival_plaque
SRR5178135	MGYA00148711	https://www.ebi.ac.uk/metagenomics/api/v1/analyses/MGYA00148711/file/SRR5178135_MERGED_FASTQ_SSU_OTU.tsv	Root-Host_associated-Human-Digestive_system-Oral-Subgingival_plaque
SRR2086932	MGYA00143877	https://www.ebi.ac.uk/metagenomics/api/v1/analyses/MGYA00143877/file/SRR2086932_MERGED_FASTQ_SSU_OTU.tsv	Root-Host_associated-Human-Digestive_system-Oral-Subgingival_plaque
SRR2154928	MGYA00150983	https://www.ebi.ac.uk/metagenomics/api/v1/analyses/MGYA00150983/file/SRR2154928_FASTQ_SSU_OTU.tsv	Root-Host_associated-Human-Digestive_system-Oral-Subgingival_plaque
SRR2086773	MGYA00143971	https://www.ebi.ac.uk/metagenomics/api/v1/analyses/MGYA00143971/file/SRR2086773_MERGED_FASTQ_SSU_OTU.tsv	Root-Host_associated-Human-Digestive_system-Oral-Subgingival_plaque
SRR5178149	MGYA00148788	https://www.ebi.ac.uk/metagenomics/api/v1/analyses/MGYA00148788/file/SRR5178149_MERGED_FASTQ_SSU_OTU.tsv	Root-Host_associated-Human-Digestive_system-Oral-Subgingival_plaque

SRR1178334	MGYA00151877	https://www.ebi.ac.uk/metagenomics/api/v1/analyses/MGYA00151877/file/SRR1178334_FASTQ_SSU_OTU.tsv	Root-Host_associated-Human-Digestive_system-Oral-Subgingival_plaque
SRR2086816	MGYA00143985	https://www.ebi.ac.uk/metagenomics/api/v1/analyses/MGYA00143985/file/SRR2086816_MERGED_FASTQ_SSU_OTU.tsv	Root-Host_associated-Human-Digestive_system-Oral-Subgingival_plaque
SRR2154954	MGYA00150955	https://www.ebi.ac.uk/metagenomics/api/v1/analyses/MGYA00150955/file/SRR2154954_FASTQ_SSU_OTU.tsv	Root-Host_associated-Human-Digestive_system-Oral-Subgingival_plaque
DRR049480	MGYA00160349	https://www.ebi.ac.uk/metagenomics/api/v1/analyses/MGYA00160349/file/DRR049480_MERGED_FASTQ_SSU_OTU.tsv	Root-Host_associated-Human-Digestive_system-Oral-Subgingival_plaque
SRR1715585	MGYA00148966	https://www.ebi.ac.uk/metagenomics/api/v1/analyses/MGYA00148966/file/SRR1715585_FASTQ_SSU_OTU.tsv	Root-Host_associated-Human-Digestive_system-Oral-Subgingival_plaque
SRR1745931	MGYA00168153	https://www.ebi.ac.uk/metagenomics/api/v1/analyses/MGYA00168153/file/SRR1745931_FASTQ_SSU_OTU.tsv	Root-Host_associated-Human-Digestive_system-Oral-Subgingival_plaque
SRR6316006	MGYA00293722	https://www.ebi.ac.uk/metagenomics/api/v1/analyses/MGYA00293722/file/SRR6316006_MERGED_FASTQ_SSU_OTU.tsv	Root-Host_associated-Human-Digestive_system-Oral-Subgingival_plaque
SRR1715643	MGYA00148929	https://www.ebi.ac.uk/metagenomics/api/v1/analyses/MGYA00148929/file/SRR1715643_FASTQ_SSU_OTU.tsv	Root-Host_associated-Human-Digestive_system-Oral-Subgingival_plaque
SRR6239892	MGYA00168192	https://www.ebi.ac.uk/metagenomics/api/v1/analyses/MGYA00168192/file/SRR6239892_FASTQ_SSU_OTU.tsv	Root-Host_associated-Human-Digestive_system-Oral-Subgingival_plaque
SRR2154985	MGYA00151074	https://www.ebi.ac.uk/metagenomics/api/v1/analyses/MGYA00151074/file/SRR2154985_FASTQ_SSU_OTU.tsv	Root-Host_associated-Human-Digestive_system-Oral-Subgingival_plaque
SRR2086774	MGYA00143888	https://www.ebi.ac.uk/metagenomics/api/v1/analyses/MGYA00143888/file/SRR2086774_MERGED_FASTQ_SSU_OTU.tsv	Root-Host_associated-Human-Digestive_system-Oral-Subgingival_plaque
SRR2086811	MGYA00143866	https://www.ebi.ac.uk/metagenomics/api/v1/analyses/MGYA00143866/file/SRR2086811_MERGED_FASTQ_SSU_OTU.tsv	Root-Host_associated-Human-Digestive_system-Oral-Subgingival_plaque

SRR1178333	MGYA00151890	https://www.ebi.ac.uk/metagenomics/api/v1/analyses/MGYA00151890/file/SRR1178333_FASTQ_SSU_OTU.tsv	Root-Host_associated-Human-Digestive_system-Oral-Subgingival_plaque
SRR2154953	MGYA00150972	https://www.ebi.ac.uk/metagenomics/api/v1/analyses/MGYA00150972/file/SRR2154953_FASTQ_SSU_OTU.tsv	Root-Host_associated-Human-Digestive_system-Oral-Subgingival_plaque
SRR2086949	MGYA00143860	https://www.ebi.ac.uk/metagenomics/api/v1/analyses/MGYA00143860/file/SRR2086949_MERGED_FASTQ_SSU_OTU.tsv	Root-Host_associated-Human-Digestive_system-Oral-Subgingival_plaque
SRR3381568	MGYA00151158	https://www.ebi.ac.uk/metagenomics/api/v1/analyses/MGYA00151158/file/SRR3381568_FASTQ_SSU_OTU.tsv	Root-Host_associated-Human-Digestive_system-Oral-Subgingival_plaque
DRR049487	MGYA00160353	https://www.ebi.ac.uk/metagenomics/api/v1/analyses/MGYA00160353/file/DRR049487_MERGED_FASTQ_SSU_OTU.tsv	Root-Host_associated-Human-Digestive_system-Oral-Subgingival_plaque
SRR1715582	MGYA00148975	https://www.ebi.ac.uk/metagenomics/api/v1/analyses/MGYA00148975/file/SRR1715582_FASTQ_SSU_OTU.tsv	Root-Host_associated-Human-Digestive_system-Oral-Subgingival_plaque
SRR3992171	MGYA00151251	https://www.ebi.ac.uk/metagenomics/api/v1/analyses/MGYA00151251/file/SRR3992171_FASTQ_SSU_OTU.tsv	Root-Host_associated-Human-Digestive_system-Oral-Subgingival_plaque
SRR1745936	MGYA00168147	https://www.ebi.ac.uk/metagenomics/api/v1/analyses/MGYA00168147/file/SRR1745936_FASTQ_SSU_OTU.tsv	Root-Host_associated-Human-Digestive_system-Oral-Subgingival_plaque
SRR1715644	MGYA00148915	https://www.ebi.ac.uk/metagenomics/api/v1/analyses/MGYA00148915/file/SRR1715644_FASTQ_SSU_OTU.tsv	Root-Host_associated-Human-Digestive_system-Oral-Subgingival_plaque
DRR049456	MGYA00160367	https://www.ebi.ac.uk/metagenomics/api/v1/analyses/MGYA00160367/file/DRR049456_MERGED_FASTQ_SSU_OTU.tsv	Root-Host_associated-Human-Digestive_system-Oral-Subgingival_plaque
SRR6239895	MGYA00168162	https://www.ebi.ac.uk/metagenomics/api/v1/analyses/MGYA00168162/file/SRR6239895_FASTQ_SSU_OTU.tsv	Root-Host_associated-Human-Digestive_system-Oral-Subgingival_plaque
SRR2154982	MGYA00151050	https://www.ebi.ac.uk/metagenomics/api/v1/analyses/MGYA00151050/file/SRR2154982_FASTQ_SSU_OTU.tsv	Root-Host_associated-Human-Digestive_system-Oral-Subgingival_plaque

SRR5178132	MGYA00148744	https://www.ebi.ac.uk/metagenomics/api/v1/analyses/MGYA00148744/file/SRR5178132_MERGED_FASTQ_SSU_OTU.tsv	Root-Host_associated-Human-Digestive_system-Oral-Subgingival_plaque
SRR2086935	MGYA00143986	https://www.ebi.ac.uk/metagenomics/api/v1/analyses/MGYA00143986/file/SRR2086935_MERGED_FASTQ_SSU_OTU.tsv	Root-Host_associated-Human-Digestive_system-Oral-Subgingival_plaque
SRR1715638	MGYA00148920	https://www.ebi.ac.uk/metagenomics/api/v1/analyses/MGYA00148920/file/SRR1715638_FASTQ_SSU_OTU.tsv	Root-Host_associated-Human-Digestive_system-Oral-Subgingival_plaque
DRR049500	MGYA00160342	https://www.ebi.ac.uk/metagenomics/api/v1/analyses/MGYA00160342/file/DRR049500_MERGED_FASTQ_SSU_OTU.tsv	Root-Host_associated-Human-Digestive_system-Oral-Subgingival_plaque
SRR6239912	MGYA00168185	https://www.ebi.ac.uk/metagenomics/api/v1/analyses/MGYA00168185/file/SRR6239912_FASTQ_SSU_OTU.tsv	Root-Host_associated-Human-Digestive_system-Oral-Subgingival_plaque
SRR5178140	MGYA00148789	https://www.ebi.ac.uk/metagenomics/api/v1/analyses/MGYA00148789/file/SRR5178140_MERGED_FASTQ_SSU_OTU.tsv	Root-Host_associated-Human-Digestive_system-Oral-Subgingival_plaque
DRR049489	MGYA00160372	https://www.ebi.ac.uk/metagenomics/api/v1/analyses/MGYA00160372/file/DRR049489_MERGED_FASTQ_SSU_OTU.tsv	Root-Host_associated-Human-Digestive_system-Oral-Subgingival_plaque
SRR2086947	MGYA00143853	https://www.ebi.ac.uk/metagenomics/api/v1/analyses/MGYA00143853/file/SRR2086947_MERGED_FASTQ_SSU_OTU.tsv	Root-Host_associated-Human-Digestive_system-Oral-Subgingival_plaque
SRR3381566	MGYA00151190	https://www.ebi.ac.uk/metagenomics/api/v1/analyses/MGYA00151190/file/SRR3381566_FASTQ_SSU_OTU.tsv	Root-Host_associated-Human-Digestive_system-Oral-Subgingival_plaque
SRR1745938	MGYA00168154	https://www.ebi.ac.uk/metagenomics/api/v1/analyses/MGYA00168154/file/SRR1745938_FASTQ_SSU_OTU.tsv	Root-Host_associated-Human-Digestive_system-Oral-Subgingival_plaque
DRR049458	MGYA00160333	https://www.ebi.ac.uk/metagenomics/api/v1/analyses/MGYA00160333/file/DRR049458_MERGED_FASTQ_SSU_OTU.tsv	Root-Host_associated-Human-Digestive_system-Oral-Subgingival_plaque
SRR2086863	MGYA00143931	https://www.ebi.ac.uk/metagenomics/api/v1/analyses/MGYA00143931/file/SRR2086863_MERGED_FASTQ_SSU_OTU.tsv	Root-Host_associated-Human-Digestive_system-Oral-Subgingival_plaque

SRR2154921	MGYA00150973	https://www.ebi.ac.uk/metagenomics/api/v1/analyses/MGYA00150973/file/SRR2154921_FASTQ_SSU_OTU.tsv	Root-Host_associated-Human-Digestive_system-Oral-Subgingival_plaque
SRR1745944	MGYA00168150	https://www.ebi.ac.uk/metagenomics/api/v1/analyses/MGYA00168150/file/SRR1745944_FASTQ_SSU_OTU.tsv	Root-Host_associated-Human-Digestive_system-Oral-Subgingival_plaque
SRR1715636	MGYA00148914	https://www.ebi.ac.uk/metagenomics/api/v1/analyses/MGYA00148914/file/SRR1715636_FASTQ_SSU_OTU.tsv	Root-Host_associated-Human-Digestive_system-Oral-Subgingival_plaque
SRR2155004	MGYA00151008	https://www.ebi.ac.uk/metagenomics/api/v1/analyses/MGYA00151008/file/SRR2155004_FASTQ_SSU_OTU.tsv	Root-Host_associated-Human-Digestive_system-Oral-Subgingival_plaque
SRR2086864	MGYA00143965	https://www.ebi.ac.uk/metagenomics/api/v1/analyses/MGYA00143965/file/SRR2086864_MERGED_FASTQ_SSU_OTU.tsv	Root-Host_associated-Human-Digestive_system-Oral-Subgingival_plaque
SRR2154926	MGYA00151043	https://www.ebi.ac.uk/metagenomics/api/v1/analyses/MGYA00151043/file/SRR2154926_FASTQ_SSU_OTU.tsv	Root-Host_associated-Human-Digestive_system-Oral-Subgingival_plaque
SRR1745943	MGYA00168149	https://www.ebi.ac.uk/metagenomics/api/v1/analyses/MGYA00168149/file/SRR1745943_FASTQ_SSU_OTU.tsv	Root-Host_associated-Human-Digestive_system-Oral-Subgingival_plaque
SRR1715631	MGYA00148974	https://www.ebi.ac.uk/metagenomics/api/v1/analyses/MGYA00148974/file/SRR1715631_FASTQ_SSU_OTU.tsv	Root-Host_associated-Human-Digestive_system-Oral-Subgingival_plaque
SRR2155003	MGYA00150951	https://www.ebi.ac.uk/metagenomics/api/v1/analyses/MGYA00150951/file/SRR2155003_FASTQ_SSU_OTU.tsv	Root-Host_associated-Human-Digestive_system-Oral-Subgingival_plaque
SRR6239915	MGYA00168199	https://www.ebi.ac.uk/metagenomics/api/v1/analyses/MGYA00168199/file/SRR6239915_FASTQ_SSU_OTU.tsv	Root-Host_associated-Human-Digestive_system-Oral-Subgingival_plaque
SRR2086818	MGYA00143999	https://www.ebi.ac.uk/metagenomics/api/v1/analyses/MGYA00143999/file/SRR2086818_MERGED_FASTQ_SSU_OTU.tsv	Root-Host_associated-Human-Digestive_system-Oral-Subgingival_plaque
SRR5178147	MGYA00148751	https://www.ebi.ac.uk/metagenomics/api/v1/analyses/MGYA00148751/file/SRR5178147_MERGED_FASTQ_SSU_OTU.tsv	Root-Host_associated-Human-Digestive_system-Oral-Subgingival_plaque

SRR3381561	MGYA00151168	https://www.ebi.ac.uk/metagenomics/api/v1/analyses/MGYA00151168/file/SRR3381561_FASTQ_SSU_OTU.tsv	Root-Host_associated-Human-Digestive_system-Oral-Subgingival_plaque
SRR2086940	MGYA00143953	https://www.ebi.ac.uk/metagenomics/api/v1/analyses/MGYA00143953/file/SRR2086940_MERGED_FASTQ_SSU_OTU.tsv	Root-Host_associated-Human-Digestive_system-Oral-Subgingival_plaque
SRR6316008	MGYA00293724	https://www.ebi.ac.uk/metagenomics/api/v1/analyses/MGYA00293724/file/SRR6316008_MERGED_FASTQ_SSU_OTU.tsv	Root-Host_associated-Human-Digestive_system-Oral-Subgingival_plaque
SRR2086851	MGYA00143873	https://www.ebi.ac.uk/metagenomics/api/v1/analyses/MGYA00143873/file/SRR2086851_MERGED_FASTQ_SSU_OTU.tsv	Root-Host_associated-Human-Digestive_system-Oral-Subgingival_plaque
SRR2086880	MGYA00143864	https://www.ebi.ac.uk/metagenomics/api/v1/analyses/MGYA00143864/file/SRR2086880_MERGED_FASTQ_SSU_OTU.tsv	Root-Host_associated-Human-Digestive_system-Oral-Subgingival_plaque
SRR2086734	MGYA00144017	https://www.ebi.ac.uk/metagenomics/api/v1/analyses/MGYA00144017/file/SRR2086734_MERGED_FASTQ_SSU_OTU.tsv	Root-Host_associated-Human-Digestive_system-Oral-Subgingival_plaque
SRR5178087	MGYA00148738	https://www.ebi.ac.uk/metagenomics/api/v1/analyses/MGYA00148738/file/SRR5178087_MERGED_FASTQ_SSU_OTU.tsv	Root-Host_associated-Human-Digestive_system-Oral-Subgingival_plaque
SRR2155036	MGYA00151034	https://www.ebi.ac.uk/metagenomics/api/v1/analyses/MGYA00151034/file/SRR2155036_FASTQ_SSU_OTU.tsv	Root-Host_associated-Human-Digestive_system-Oral-Subgingival_plaque
SRR1715604	MGYA00148907	https://www.ebi.ac.uk/metagenomics/api/v1/analyses/MGYA00148907/file/SRR1715604_FASTQ_SSU_OTU.tsv	Root-Host_associated-Human-Digestive_system-Oral-Subgingival_plaque
SRR2154913	MGYA00151059	https://www.ebi.ac.uk/metagenomics/api/v1/analyses/MGYA00151059/file/SRR2154913_FASTQ_SSU_OTU.tsv	Root-Host_associated-Human-Digestive_system-Oral-Subgingival_plaque
SRR2086799	MGYA00143968	https://www.ebi.ac.uk/metagenomics/api/v1/analyses/MGYA00143968/file/SRR2086799_MERGED_FASTQ_SSU_OTU.tsv	Root-Host_associated-Human-Digestive_system-Oral-Subgingival_plaque
SRR6239920	MGYA00168183	https://www.ebi.ac.uk/metagenomics/api/v1/analyses/MGYA00168183/file/SRR6239920_FASTQ_SSU_OTU.tsv	Root-Host_associated-Human-Digestive_system-Oral-Subgingival_plaque

SRR2086748	MGYA00143895	https://www.ebi.ac.uk/metagenomics/api/v1/analyses/MGYA00143895/file/SRR2086748_MERGED_FASTQ_SSU_OTU.tsv	Root-Host_associated-Human-Digestive_system-Oral-Subgingival_plaque
SRR1178521	MGYA00151884	https://www.ebi.ac.uk/metagenomics/api/v1/analyses/MGYA00151884/file/SRR1178521_FASTQ_SSU_OTU.tsv	Root-Host_associated-Human-Digestive_system-Oral-Subgingival_plaque
SRR3381585	MGYA00151175	https://www.ebi.ac.uk/metagenomics/api/v1/analyses/MGYA00151175/file/SRR3381585_FASTQ_SSU_OTU.tsv	Root-Host_associated-Human-Digestive_system-Oral-Subgingival_plaque
SRR6239878	MGYA00168177	https://www.ebi.ac.uk/metagenomics/api/v1/analyses/MGYA00168177/file/SRR6239878_FASTQ_SSU_OTU.tsv	Root-Host_associated-Human-Digestive_system-Oral-Subgingival_plaque
SRR3381554	MGYA00151186	https://www.ebi.ac.uk/metagenomics/api/v1/analyses/MGYA00151186/file/SRR3381554_FASTQ_SSU_OTU.tsv	Root-Host_associated-Human-Digestive_system-Oral-Subgingival_plaque
SRR3381582	MGYA00151171	https://www.ebi.ac.uk/metagenomics/api/v1/analyses/MGYA00151171/file/SRR3381582_FASTQ_SSU_OTU.tsv	Root-Host_associated-Human-Digestive_system-Oral-Subgingival_plaque
SRR3381553	MGYA00151187	https://www.ebi.ac.uk/metagenomics/api/v1/analyses/MGYA00151187/file/SRR3381553_FASTQ_SSU_OTU.tsv	Root-Host_associated-Human-Digestive_system-Oral-Subgingival_plaque
SRR2154968	MGYA00150988	https://www.ebi.ac.uk/metagenomics/api/v1/analyses/MGYA00150988/file/SRR2154968_FASTQ_SSU_OTU.tsv	Root-Host_associated-Human-Digestive_system-Oral-Subgingival_plaque
SRR5178109	MGYA00148806	https://www.ebi.ac.uk/metagenomics/api/v1/analyses/MGYA00148806/file/SRR5178109_MERGED_FASTQ_SSU_OTU.tsv	Root-Host_associated-Human-Digestive_system-Oral-Subgingival_plaque
SRR2086856	MGYA00143905	https://www.ebi.ac.uk/metagenomics/api/v1/analyses/MGYA00143905/file/SRR2086856_MERGED_FASTQ_SSU_OTU.tsv	Root-Host_associated-Human-Digestive_system-Oral-Subgingival_plaque
SRR2086887	MGYA00143825	https://www.ebi.ac.uk/metagenomics/api/v1/analyses/MGYA00143825/file/SRR2086887_MERGED_FASTQ_SSU_OTU.tsv	Root-Host_associated-Human-Digestive_system-Oral-Subgingival_plaque
SRR2086733	MGYA00143844	https://www.ebi.ac.uk/metagenomics/api/v1/analyses/MGYA00143844/file/SRR2086733_MERGED_FASTQ_SSU_OTU.tsv	Root-Host_associated-Human-Digestive_system-Oral-Subgingival_plaque

SRR5178080	MGYA00148746	https://www.ebi.ac.uk/metagenomics/api/v1/analyses/MGYA00148746/file/SRR5178080_MERGED_FASTQ_SSU_OTU.tsv	Root-Host_associated-Human-Digestive_system-Oral-Subgingival_plaque
SRR2155031	MGYA00150957	https://www.ebi.ac.uk/metagenomics/api/v1/analyses/MGYA00150957/file/SRR2155031_FASTQ_SSU_OTU.tsv	Root-Host_associated-Human-Digestive_system-Oral-Subgingival_plaque
SRR1715603	MGYA00148960	https://www.ebi.ac.uk/metagenomics/api/v1/analyses/MGYA00148960/file/SRR1715603_FASTQ_SSU_OTU.tsv	Root-Host_associated-Human-Digestive_system-Oral-Subgingival_plaque
SRR2154914	MGYA00151005	https://www.ebi.ac.uk/metagenomics/api/v1/analyses/MGYA00151005/file/SRR2154914_FASTQ_SSU_OTU.tsv	Root-Host_associated-Human-Digestive_system-Oral-Subgingival_plaque
SRR2086790	MGYA00143850	https://www.ebi.ac.uk/metagenomics/api/v1/analyses/MGYA00143850/file/SRR2086790_MERGED_FASTQ_SSU_OTU.tsv	Root-Host_associated-Human-Digestive_system-Oral-Subgingival_plaque
SRR2086824	MGYA00143908	https://www.ebi.ac.uk/metagenomics/api/v1/analyses/MGYA00143908/file/SRR2086824_MERGED_FASTQ_SSU_OTU.tsv	Root-Host_associated-Human-Digestive_system-Oral-Subgingival_plaque
SRR2086741	MGYA00143934	https://www.ebi.ac.uk/metagenomics/api/v1/analyses/MGYA00143934/file/SRR2086741_MERGED_FASTQ_SSU_OTU.tsv	Root-Host_associated-Human-Digestive_system-Oral-Subgingival_plaque
DRR049463	MGYA00160366	https://www.ebi.ac.uk/metagenomics/api/v1/analyses/MGYA00160366/file/DRR049463_MERGED_FASTQ_SSU_OTU.tsv	Root-Host_associated-Human-Digestive_system-Oral-Subgingival_plaque
SRR1715671	MGYA00148949	https://www.ebi.ac.uk/metagenomics/api/v1/analyses/MGYA00148949/file/SRR1715671_FASTQ_SSU_OTU.tsv	Root-Host_associated-Human-Digestive_system-Oral-Subgingival_plaque
SRR6239871	MGYA00168207	https://www.ebi.ac.uk/metagenomics/api/v1/analyses/MGYA00168207/file/SRR6239871_FASTQ_SSU_OTU.tsv	Root-Host_associated-Human-Digestive_system-Oral-Subgingival_plaque
SRR2154966	MGYA00151010	https://www.ebi.ac.uk/metagenomics/api/v1/analyses/MGYA00151010/file/SRR2154966_FASTQ_SSU_OTU.tsv	Root-Host_associated-Human-Digestive_system-Oral-Subgingival_plaque
SRR2086858	MGYA00143898	https://www.ebi.ac.uk/metagenomics/api/v1/analyses/MGYA00143898/file/SRR2086858_MERGED_FASTQ_SSU_OTU.tsv	Root-Host_associated-Human-Digestive_system-Oral-Subgingival_plaque

SRR5178107	MGYA00148768	https://www.ebi.ac.uk/metagenomics/api/v1/analyses/MGYA00148768/file/SRR5178107_MERGED_FASTQ_SSU_OTU.tsv	Root-Host_associated-Human-Digestive_system-Oral-Subgingival_plaque
SRR2086889	MGYA00143978	https://www.ebi.ac.uk/metagenomics/api/v1/analyses/MGYA00143978/file/SRR2086889_MERGED_FASTQ_SSU_OTU.tsv	Root-Host_associated-Human-Digestive_system-Oral-Subgingival_plaque
SRR2154893	MGYA00151066	https://www.ebi.ac.uk/metagenomics/api/v1/analyses/MGYA00151066/file/SRR2154893_FASTQ_SSU_OTU.tsv	Root-Host_associated-Human-Digestive_system-Oral-Subgingival_plaque
SRR2086900	MGYA00144032	https://www.ebi.ac.uk/metagenomics/api/v1/analyses/MGYA00144032/file/SRR2086900_MERGED_FASTQ_SSU_OTU.tsv	Root-Host_associated-Human-Digestive_system-Oral-Subgingival_plaque
SRR5178100	MGYA00148767	https://www.ebi.ac.uk/metagenomics/api/v1/analyses/MGYA00148767/file/SRR5178100_MERGED_FASTQ_SSU_OTU.tsv	Root-Host_associated-Human-Digestive_system-Oral-Subgingival_plaque
SRR2154894	MGYA00151041	https://www.ebi.ac.uk/metagenomics/api/v1/analyses/MGYA00151041/file/SRR2154894_FASTQ_SSU_OTU.tsv	Root-Host_associated-Human-Digestive_system-Oral-Subgingival_plaque
SRR5178089	MGYA00148739	https://www.ebi.ac.uk/metagenomics/api/v1/analyses/MGYA00148739/file/SRR5178089_MERGED_FASTQ_SSU_OTU.tsv	Root-Host_associated-Human-Digestive_system-Oral-Subgingival_plaque
SRR2155038	MGYA00151012	https://www.ebi.ac.uk/metagenomics/api/v1/analyses/MGYA00151012/file/SRR2155038_FASTQ_SSU_OTU.tsv	Root-Host_associated-Human-Digestive_system-Oral-Subgingival_plaque
SRR2086907	MGYA00144013	https://www.ebi.ac.uk/metagenomics/api/v1/analyses/MGYA00144013/file/SRR2086907_MERGED_FASTQ_SSU_OTU.tsv	Root-Host_associated-Human-Digestive_system-Oral-Subgingival_plaque
SRR2086797	MGYA00143901	https://www.ebi.ac.uk/metagenomics/api/v1/analyses/MGYA00143901/file/SRR2086797_MERGED_FASTQ_SSU_OTU.tsv	Root-Host_associated-Human-Digestive_system-Oral-Subgingival_plaque
SRR2086823	MGYA00143882	https://www.ebi.ac.uk/metagenomics/api/v1/analyses/MGYA00143882/file/SRR2086823_MERGED_FASTQ_SSU_OTU.tsv	Root-Host_associated-Human-Digestive_system-Oral-Subgingival_plaque
SRR2086746	MGYA00143915	https://www.ebi.ac.uk/metagenomics/api/v1/analyses/MGYA00143915/file/SRR2086746_MERGED_FASTQ_SSU_OTU.tsv	Root-Host_associated-Human-Digestive_system-Oral-Subgingival_plaque

DRR049464	MGYA00160352	https://www.ebi.ac.uk/metagenomics/api/v1/analyses/MGYA00160352/file/DRR049464_MERGED_FASTQ_SSU_OTU.tsv	Root-Host_associated-Human-Digestive_system-Oral-Subgingival_plaque
SRR6239876	MGYA00168202	https://www.ebi.ac.uk/metagenomics/api/v1/analyses/MGYA00168202/file/SRR6239876_FASTQ_SSU_OTU.tsv	Root-Host_associated-Human-Digestive_system-Oral-Subgingival_plaque
SRR2154961	MGYA00151018	https://www.ebi.ac.uk/metagenomics/api/v1/analyses/MGYA00151018/file/SRR2154961_FASTQ_SSU_OTU.tsv	Root-Host_associated-Human-Digestive_system-Oral-Subgingival_plaque
SRR1226615	MGYA00150635	https://www.ebi.ac.uk/metagenomics/api/v1/analyses/MGYA00150635/file/SRR1226615_FASTQ_SSU_OTU.tsv	Root-Host_associated-Human-Digestive_system-Oral-Subgingival_plaque
SRR2086916	MGYA00143921	https://www.ebi.ac.uk/metagenomics/api/v1/analyses/MGYA00143921/file/SRR2086916_MERGED_FASTQ_SSU_OTU.tsv	Root-Host_associated-Human-Digestive_system-Oral-Subgingival_plaque
SRR5178098	MGYA00148726	https://www.ebi.ac.uk/metagenomics/api/v1/analyses/MGYA00148726/file/SRR5178098_MERGED_FASTQ_SSU_OTU.tsv	Root-Host_associated-Human-Digestive_system-Oral-Subgingival_plaque
SRR2155029	MGYA00150970	https://www.ebi.ac.uk/metagenomics/api/v1/analyses/MGYA00150970/file/SRR2155029_FASTQ_SSU_OTU.tsv	Root-Host_associated-Human-Digestive_system-Oral-Subgingival_plaque
SRR5178111	MGYA00148732	https://www.ebi.ac.uk/metagenomics/api/v1/analyses/MGYA00148732/file/SRR5178111_MERGED_FASTQ_SSU_OTU.tsv	Root-Host_associated-Human-Digestive_system-Oral-Subgingival_plaque
SRR1715667	MGYA00148904	https://www.ebi.ac.uk/metagenomics/api/v1/analyses/MGYA00148904/file/SRR1715667_FASTQ_SSU_OTU.tsv	Root-Host_associated-Human-Digestive_system-Oral-Subgingival_plaque
SRR6316022	MGYA00293738	https://www.ebi.ac.uk/metagenomics/api/v1/analyses/MGYA00293738/file/SRR6316022_MERGED_FASTQ_SSU_OTU.tsv	Root-Host_associated-Human-Digestive_system-Oral-Subgingival_plaque
SRR6239867	MGYA00168208	https://www.ebi.ac.uk/metagenomics/api/v1/analyses/MGYA00168208/file/SRR6239867_FASTQ_SSU_OTU.tsv	Root-Host_associated-Human-Digestive_system-Oral-Subgingival_plaque
SRR2154970	MGYA00151081	https://www.ebi.ac.uk/metagenomics/api/v1/analyses/MGYA00151081/file/SRR2154970_FASTQ_SSU_OTU.tsv	Root-Host_associated-Human-Digestive_system-Oral-Subgingival_plaque

DRR049475	MGYA00160337	https://www.ebi.ac.uk/metagenomics/api/v1/analyses/MGYA00160337/file/DRR049475_MERGED_FASTQ_SSU_OTU.tsv	Root-Host_associated-Human-Digestive_system-Oral-Subgingival_plaque
SRR2086757	MGYA00144016	https://www.ebi.ac.uk/metagenomics/api/v1/analyses/MGYA00144016/file/SRR2086757_MERGED_FASTQ_SSU_OTU.tsv	Root-Host_associated-Human-Digestive_system-Oral-Subgingival_plaque
SRR2086786	MGYA00143841	https://www.ebi.ac.uk/metagenomics/api/v1/analyses/MGYA00143841/file/SRR2086786_MERGED_FASTQ_SSU_OTU.tsv	Root-Host_associated-Human-Digestive_system-Oral-Subgingival_plaque
SRR2086832	MGYA00143837	https://www.ebi.ac.uk/metagenomics/api/v1/analyses/MGYA00143837/file/SRR2086832_MERGED_FASTQ_SSU_OTU.tsv	Root-Host_associated-Human-Digestive_system-Oral-Subgingival_plaque
SRR1715660	MGYA00148971	https://www.ebi.ac.uk/metagenomics/api/v1/analyses/MGYA00148971/file/SRR1715660_FASTQ_SSU_OTU.tsv	Root-Host_associated-Human-Digestive_system-Oral-Subgingival_plaque
SRR6239860	MGYA00168175	https://www.ebi.ac.uk/metagenomics/api/v1/analyses/MGYA00168175/file/SRR6239860_FASTQ_SSU_OTU.tsv	Root-Host_associated-Human-Digestive_system-Oral-Subgingival_plaque
SRR2154977	MGYA00150990	https://www.ebi.ac.uk/metagenomics/api/v1/analyses/MGYA00150990/file/SRR2154977_FASTQ_SSU_OTU.tsv	Root-Host_associated-Human-Digestive_system-Oral-Subgingival_plaque
DRR049472	MGYA00160329	https://www.ebi.ac.uk/metagenomics/api/v1/analyses/MGYA00160329/file/DRR049472_MERGED_FASTQ_SSU_OTU.tsv	Root-Host_associated-Human-Digestive_system-Oral-Subgingival_plaque
SRR1715577	MGYA00148893	https://www.ebi.ac.uk/metagenomics/api/v1/analyses/MGYA00148893/file/SRR1715577_FASTQ_SSU_OTU.tsv	Root-Host_associated-Human-Digestive_system-Oral-Subgingival_plaque
SRR2086750	MGYA00143909	https://www.ebi.ac.uk/metagenomics/api/v1/analyses/MGYA00143909/file/SRR2086750_MERGED_FASTQ_SSU_OTU.tsv	Root-Host_associated-Human-Digestive_system-Oral-Subgingival_plaque
SRR2086781	MGYA00143862	https://www.ebi.ac.uk/metagenomics/api/v1/analyses/MGYA00143862/file/SRR2086781_MERGED_FASTQ_SSU_OTU.tsv	Root-Host_associated-Human-Digestive_system-Oral-Subgingival_plaque
SRR2086835	MGYA00143948	https://www.ebi.ac.uk/metagenomics/api/v1/analyses/MGYA00143948/file/SRR2086835_MERGED_FASTQ_SSU_OTU.tsv	Root-Host_associated-Human-Digestive_system-Oral-Subgingival_plaque

SRR5178116	MGYA00148725	https://www.ebi.ac.uk/metagenomics/api/v1/analyses/MGYA00148725/file/SRR5178116_MERGED_FASTQ_SSU_OTU.tsv	Root-Host_associated-Human-Digestive_system-Oral-Subgingival_plaque
SRR2086849	MGYA00143995	https://www.ebi.ac.uk/metagenomics/api/v1/analyses/MGYA00143995/file/SRR2086849_MERGED_FASTQ_SSU_OTU.tsv	Root-Host_associated-Human-Digestive_system-Oral-Subgingival_plaque
SRR2154979	MGYA00151047	https://www.ebi.ac.uk/metagenomics/api/v1/analyses/MGYA00151047/file/SRR2154979_FASTQ_SSU_OTU.tsv	Root-Host_associated-Human-Digestive_system-Oral-Subgingival_plaque
SRR1715579	MGYA00148879	https://www.ebi.ac.uk/metagenomics/api/v1/analyses/MGYA00148879/file/SRR1715579_FASTQ_SSU_OTU.tsv	Root-Host_associated-Human-Digestive_system-Oral-Subgingival_plaque
SRR5178164	MGYA00148750	https://www.ebi.ac.uk/metagenomics/api/v1/analyses/MGYA00148750/file/SRR5178164_MERGED_FASTQ_SSU_OTU.tsv	Root-Host_associated-Human-Digestive_system-Oral-Subgingival_plaque
SRR1715612	MGYA00148905	https://www.ebi.ac.uk/metagenomics/api/v1/analyses/MGYA00148905/file/SRR1715612_FASTQ_SSU_OTU.tsv	Root-Host_associated-Human-Digestive_system-Oral-Subgingival_plaque
SRR2154905	MGYA00151017	https://www.ebi.ac.uk/metagenomics/api/v1/analyses/MGYA00151017/file/SRR2154905_FASTQ_SSU_OTU.tsv	Root-Host_associated-Human-Digestive_system-Oral-Subgingival_plaque
SRR5178091	MGYA00148784	https://www.ebi.ac.uk/metagenomics/api/v1/analyses/MGYA00148784/file/SRR5178091_MERGED_FASTQ_SSU_OTU.tsv	Root-Host_associated-Human-Digestive_system-Oral-Subgingival_plaque
SRR2155020	MGYA00150975	https://www.ebi.ac.uk/metagenomics/api/v1/analyses/MGYA00150975/file/SRR2155020_FASTQ_SSU_OTU.tsv	Root-Host_associated-Human-Digestive_system-Oral-Subgingival_plaque
SRR2086847	MGYA00143857	https://www.ebi.ac.uk/metagenomics/api/v1/analyses/MGYA00143857/file/SRR2086847_MERGED_FASTQ_SSU_OTU.tsv	Root-Host_associated-Human-Digestive_system-Oral-Subgingival_plaque
SRR5178118	MGYA00148735	https://www.ebi.ac.uk/metagenomics/api/v1/analyses/MGYA00148735/file/SRR5178118_MERGED_FASTQ_SSU_OTU.tsv	Root-Host_associated-Human-Digestive_system-Oral-Subgingival_plaque
SRR1715615	MGYA00148924	https://www.ebi.ac.uk/metagenomics/api/v1/analyses/MGYA00148924/file/SRR1715615_FASTQ_SSU_OTU.tsv	Root-Host_associated-Human-Digestive_system-Oral-Subgingival_plaque

SRR2154902	MGYA00151032	https://www.ebi.ac.uk/metagenomics/api/v1/analyses/MGYA00151032/file/SRR2154902_FASTQ_SSU_OTU.tsv	Root-Host_associated-Human-Digestive_system-Oral-Subgingival_plaque
SRR2086918	MGYA00143939	https://www.ebi.ac.uk/metagenomics/api/v1/analyses/MGYA00143939/file/SRR2086918_MERGED_FASTQ_SSU_OTU.tsv	Root-Host_associated-Human-Digestive_system-Oral-Subgingival_plaque
SRR5178096	MGYA00148717	https://www.ebi.ac.uk/metagenomics/api/v1/analyses/MGYA00148717/file/SRR5178096_MERGED_FASTQ_SSU_OTU.tsv	Root-Host_associated-Human-Digestive_system-Oral-Subgingival_plaque
SRR2155027	MGYA00151042	https://www.ebi.ac.uk/metagenomics/api/v1/analyses/MGYA00151042/file/SRR2155027_FASTQ_SSU_OTU.tsv	Root-Host_associated-Human-Digestive_system-Oral-Subgingival_plaque
SRR2086891	MGYA00143891	https://www.ebi.ac.uk/metagenomics/api/v1/analyses/MGYA00143891/file/SRR2086891_MERGED_FASTQ_SSU_OTU.tsv	Root-Host_associated-Human-Digestive_system-Oral-Subgingival_plaque
SRR2086725	MGYA00144011	https://www.ebi.ac.uk/metagenomics/api/v1/analyses/MGYA00144011/file/SRR2086725_MERGED_FASTQ_SSU_OTU.tsv	Root-Host_associated-Human-Digestive_system-Oral-Subgingival_plaque
SRR2086840	MGYA00144014	https://www.ebi.ac.uk/metagenomics/api/v1/analyses/MGYA00144014/file/SRR2086840_MERGED_FASTQ_SSU_OTU.tsv	Root-Host_associated-Human-Digestive_system-Oral-Subgingival_plaque
SRR1715669	MGYA00148954	https://www.ebi.ac.uk/metagenomics/api/v1/analyses/MGYA00148954/file/SRR1715669_FASTQ_SSU_OTU.tsv	Root-Host_associated-Human-Digestive_system-Oral-Subgingival_plaque
SRR6239869	MGYA00168190	https://www.ebi.ac.uk/metagenomics/api/v1/analyses/MGYA00168190/file/SRR6239869_FASTQ_SSU_OTU.tsv	Root-Host_associated-Human-Digestive_system-Oral-Subgingival_plaque
SRR3381545	MGYA00151155	https://www.ebi.ac.uk/metagenomics/api/v1/analyses/MGYA00151155/file/SRR3381545_FASTQ_SSU_OTU.tsv	Root-Host_associated-Human-Digestive_system-Oral-Subgingival_plaque
SRR1178530	MGYA00151883	https://www.ebi.ac.uk/metagenomics/api/v1/analyses/MGYA00151883/file/SRR1178530_FASTQ_SSU_OTU.tsv	Root-Host_associated-Human-Digestive_system-Oral-Subgingival_plaque
SRR2086759	MGYA00143829	https://www.ebi.ac.uk/metagenomics/api/v1/analyses/MGYA00143829/file/SRR2086759_MERGED_FASTQ_SSU_OTU.tsv	Root-Host_associated-Human-Digestive_system-Oral-Subgingival_plaque

SRR2086788	MGYA00143972	https://www.ebi.ac.uk/metagenomics/api/v1/analyses/MGYA00143972/file/SRR2086788_MERGED_FASTQ_SSU_OTU.tsv	Root-Host_associated-Human-Digestive_system-Oral-Subgingival_plaque
SRR5178163	MGYA00148721	https://www.ebi.ac.uk/metagenomics/api/v1/analyses/MGYA00148721/file/SRR5178163_MERGED_FASTQ_SSU_OTU.tsv	Root-Host_associated-Human-Digestive_system-Oral-Subgingival_plaque
SRR2155012	MGYA00151079	https://www.ebi.ac.uk/metagenomics/api/v1/analyses/MGYA00151079/file/SRR2155012_FASTQ_SSU_OTU.tsv	Root-Host_associated-Human-Digestive_system-Oral-Subgingival_plaque
SRR5178072	MGYA00148771	https://www.ebi.ac.uk/metagenomics/api/v1/analyses/MGYA00148771/file/SRR5178072_MERGED_FASTQ_SSU_OTU.tsv	Root-Host_associated-Human-Digestive_system-Oral-Subgingival_plaque
SRR2154937	MGYA00150954	https://www.ebi.ac.uk/metagenomics/api/v1/analyses/MGYA00150954/file/SRR2154937_FASTQ_SSU_OTU.tsv	Root-Host_associated-Human-Digestive_system-Oral-Subgingival_plaque
SRR1715620	MGYA00148918	https://www.ebi.ac.uk/metagenomics/api/v1/analyses/MGYA00148918/file/SRR1715620_FASTQ_SSU_OTU.tsv	Root-Host_associated-Human-Digestive_system-Oral-Subgingival_plaque
SRR2086875	MGYA00143845	https://www.ebi.ac.uk/metagenomics/api/v1/analyses/MGYA00143845/file/SRR2086875_MERGED_FASTQ_SSU_OTU.tsv	Root-Host_associated-Human-Digestive_system-Oral-Subgingival_plaque
SRR2086951	MGYA00144048	https://www.ebi.ac.uk/metagenomics/api/v1/analyses/MGYA00144048/file/SRR2086951_MERGED_FASTQ_SSU_OTU.tsv	Root-Host_associated-Human-Digestive_system-Oral-Subgingival_plaque
SRR3381570	MGYA00151163	https://www.ebi.ac.uk/metagenomics/api/v1/analyses/MGYA00151163/file/SRR3381570_FASTQ_SSU_OTU.tsv	Root-Host_associated-Human-Digestive_system-Oral-Subgingival_plaque
SRR1178273	MGYA00151889	https://www.ebi.ac.uk/metagenomics/api/v1/analyses/MGYA00151889/file/SRR1178273_FASTQ_SSU_OTU.tsv	Root-Host_associated-Human-Digestive_system-Oral-Subgingival_plaque
SRR3992169	MGYA00151247	https://www.ebi.ac.uk/metagenomics/api/v1/analyses/MGYA00151247/file/SRR3992169_FASTQ_SSU_OTU.tsv	Root-Host_associated-Human-Digestive_system-Oral-Subgingival_plaque
SRR6316019	MGYA00293735	https://www.ebi.ac.uk/metagenomics/api/v1/analyses/MGYA00293735/file/SRR6316019_MERGED_FASTQ_SSU_OTU.tsv	Root-Host_associated-Human-Digestive_system-Oral-Subgingival_plaque

SRR6239904	MGYA00168196	https://www.ebi.ac.uk/metagenomics/api/v1/analyses/MGYA00168196/file/SRR6239904_FASTQ_SSU_OTU.tsv	Root-Host_associated-Human-Digestive_system-Oral-Subgingival_plaque
SRR2086809	MGYA00143956	https://www.ebi.ac.uk/metagenomics/api/v1/analyses/MGYA00143956/file/SRR2086809_MERGED_FASTQ_SSU_OTU.tsv	Root-Host_associated-Human-Digestive_system-Oral-Subgingival_plaque
SRR1178274	MGYA00151881	https://www.ebi.ac.uk/metagenomics/api/v1/analyses/MGYA00151881/file/SRR1178274_FASTQ_SSU_OTU.tsv	Root-Host_associated-Human-Digestive_system-Oral-Subgingival_plaque
SRR3381577	MGYA00151173	https://www.ebi.ac.uk/metagenomics/api/v1/analyses/MGYA00151173/file/SRR3381577_FASTQ_SSU_OTU.tsv	Root-Host_associated-Human-Digestive_system-Oral-Subgingival_plaque
SRR2086956	MGYA00143883	https://www.ebi.ac.uk/metagenomics/api/v1/analyses/MGYA00143883/file/SRR2086956_MERGED_FASTQ_SSU_OTU.tsv	Root-Host_associated-Human-Digestive_system-Oral-Subgingival_plaque
DRR049498	MGYA00160346	https://www.ebi.ac.uk/metagenomics/api/v1/analyses/MGYA00160346/file/DRR049498_MERGED_FASTQ_SSU_OTU.tsv	Root-Host_associated-Human-Digestive_system-Oral-Subgingival_plaque
SRR1745929	MGYA00168155	https://www.ebi.ac.uk/metagenomics/api/v1/analyses/MGYA00168155/file/SRR1745929_FASTQ_SSU_OTU.tsv	Root-Host_associated-Human-Digestive_system-Oral-Subgingival_plaque
SRR5178151	MGYA00148803	https://www.ebi.ac.uk/metagenomics/api/v1/analyses/MGYA00148803/file/SRR5178151_MERGED_FASTQ_SSU_OTU.tsv	Root-Host_associated-Human-Digestive_system-Oral-Subgingival_plaque
SRR6239903	MGYA00168179	https://www.ebi.ac.uk/metagenomics/api/v1/analyses/MGYA00168179/file/SRR6239903_FASTQ_SSU_OTU.tsv	Root-Host_associated-Human-Digestive_system-Oral-Subgingival_plaque
SRR2155015	MGYA00151083	https://www.ebi.ac.uk/metagenomics/api/v1/analyses/MGYA00151083/file/SRR2155015_FASTQ_SSU_OTU.tsv	Root-Host_associated-Human-Digestive_system-Oral-Subgingival_plaque
SRR5178075	MGYA00148770	https://www.ebi.ac.uk/metagenomics/api/v1/analyses/MGYA00148770/file/SRR5178075_MERGED_FASTQ_SSU_OTU.tsv	Root-Host_associated-Human-Digestive_system-Oral-Subgingival_plaque
SRR2154930	MGYA00151049	https://www.ebi.ac.uk/metagenomics/api/v1/analyses/MGYA00151049/file/SRR2154930_FASTQ_SSU_OTU.tsv	Root-Host_associated-Human-Digestive_system-Oral-Subgingival_plaque

SRR1715627	MGYA00148930	https://www.ebi.ac.uk/metagenomics/api/v1/analyses/MGYA00148930/file/SRR1715627_FASTQ_SSU_OTU.tsv	Root-Host_associated-Human-Digestive_system-Oral-Subgingival_plaque
SRR2086872	MGYA00143926	https://www.ebi.ac.uk/metagenomics/api/v1/analyses/MGYA00143926/file/SRR2086872_MERGED_FASTQ_SSU_OTU.tsv	Root-Host_associated-Human-Digestive_system-Oral-Subgingival_plaque
SRR6239884	MGYA00168180	https://www.ebi.ac.uk/metagenomics/api/v1/analyses/MGYA00168180/file/SRR6239884_FASTQ_SSU_OTU.tsv	Root-Host_associated-Human-Digestive_system-Oral-Subgingival_plaque
SRR2154993	MGYA00151030	https://www.ebi.ac.uk/metagenomics/api/v1/analyses/MGYA00151030/file/SRR2154993_FASTQ_SSU_OTU.tsv	Root-Host_associated-Human-Digestive_system-Oral-Subgingival_plaque
DRR049496	MGYA00160354	https://www.ebi.ac.uk/metagenomics/api/v1/analyses/MGYA00160354/file/DRR049496_MERGED_FASTQ_SSU_OTU.tsv	Root-Host_associated-Human-Digestive_system-Oral-Subgingival_plaque
SRR1715593	MGYA00148953	https://www.ebi.ac.uk/metagenomics/api/v1/analyses/MGYA00148953/file/SRR1715593_FASTQ_SSU_OTU.tsv	Root-Host_associated-Human-Digestive_system-Oral-Subgingival_plaque
SRR2154942	MGYA00150960	https://www.ebi.ac.uk/metagenomics/api/v1/analyses/MGYA00150960/file/SRR2154942_FASTQ_SSU_OTU.tsv	Root-Host_associated-Human-Digestive_system-Oral-Subgingival_plaque
SRR3381579	MGYA00151172	https://www.ebi.ac.uk/metagenomics/api/v1/analyses/MGYA00151172/file/SRR3381579_FASTQ_SSU_OTU.tsv	Root-Host_associated-Human-Digestive_system-Oral-Subgingival_plaque
SRR2086958	MGYA00143878	https://www.ebi.ac.uk/metagenomics/api/v1/analyses/MGYA00143878/file/SRR2086958_MERGED_FASTQ_SSU_OTU.tsv	Root-Host_associated-Human-Digestive_system-Oral-Subgingival_plaque
SRR1715655	MGYA00148896	https://www.ebi.ac.uk/metagenomics/api/v1/analyses/MGYA00148896/file/SRR1715655_FASTQ_SSU_OTU.tsv	Root-Host_associated-Human-Digestive_system-Oral-Subgingival_plaque
SRR6316010	MGYA00293726	https://www.ebi.ac.uk/metagenomics/api/v1/analyses/MGYA00293726/file/SRR6316010_MERGED_FASTQ_SSU_OTU.tsv	Root-Host_associated-Human-Digestive_system-Oral-Subgingival_plaque
SRR1745927	MGYA00168137	https://www.ebi.ac.uk/metagenomics/api/v1/analyses/MGYA00168137/file/SRR1745927_FASTQ_SSU_OTU.tsv	Root-Host_associated-Human-Digestive_system-Oral-Subgingival_plaque

SRR2086800	MGYA00143896	https://www.ebi.ac.uk/metagenomics/api/v1/analyses/MGYA00143896/file/SRR2086800_MERGED_FASTQ_SSU_OTU.tsv	Root-Host_associated-Human-Digestive_system-Oral-Subgingival_plaque
SRR2086765	MGYA00143830	https://www.ebi.ac.uk/metagenomics/api/v1/analyses/MGYA00143830/file/SRR2086765_MERGED_FASTQ_SSU_OTU.tsv	Root-Host_associated-Human-Digestive_system-Oral-Subgingival_plaque
SRR2086924	MGYA00144005	https://www.ebi.ac.uk/metagenomics/api/v1/analyses/MGYA00144005/file/SRR2086924_MERGED_FASTQ_SSU_OTU.tsv	Root-Host_associated-Human-Digestive_system-Oral-Subgingival_plaque
SRR1715629	MGYA00148912	https://www.ebi.ac.uk/metagenomics/api/v1/analyses/MGYA00148912/file/SRR1715629_FASTQ_SSU_OTU.tsv	Root-Host_associated-Human-Digestive_system-Oral-Subgingival_plaque
SRR5178123	MGYA00148797	https://www.ebi.ac.uk/metagenomics/api/v1/analyses/MGYA00148797/file/SRR5178123_MERGED_FASTQ_SSU_OTU.tsv	Root-Host_associated-Human-Digestive_system-Oral-Subgingival_plaque
SRR2086923	MGYA00143859	https://www.ebi.ac.uk/metagenomics/api/v1/analyses/MGYA00143859/file/SRR2086923_MERGED_FASTQ_SSU_OTU.tsv	Root-Host_associated-Human-Digestive_system-Oral-Subgingival_plaque
SRR2154939	MGYA00151077	https://www.ebi.ac.uk/metagenomics/api/v1/analyses/MGYA00151077/file/SRR2154939_FASTQ_SSU_OTU.tsv	Root-Host_associated-Human-Digestive_system-Oral-Subgingival_plaque
SRR5178124	MGYA00148755	https://www.ebi.ac.uk/metagenomics/api/v1/analyses/MGYA00148755/file/SRR5178124_MERGED_FASTQ_SSU_OTU.tsv	Root-Host_associated-Human-Digestive_system-Oral-Subgingival_plaque
SRR6239883	MGYA00168220	https://www.ebi.ac.uk/metagenomics/api/v1/analyses/MGYA00168220/file/SRR6239883_FASTQ_SSU_OTU.tsv	Root-Host_associated-Human-Digestive_system-Oral-Subgingival_plaque
SRR2154994	MGYA00151002	https://www.ebi.ac.uk/metagenomics/api/v1/analyses/MGYA00151002/file/SRR2154994_FASTQ_SSU_OTU.tsv	Root-Host_associated-Human-Digestive_system-Oral-Subgingival_plaque
DRR049491	MGYA00160364	https://www.ebi.ac.uk/metagenomics/api/v1/analyses/MGYA00160364/file/DRR049491_MERGED_FASTQ_SSU_OTU.tsv	Root-Host_associated-Human-Digestive_system-Oral-Subgingival_plaque
SRR1715594	MGYA00148922	https://www.ebi.ac.uk/metagenomics/api/v1/analyses/MGYA00148922/file/SRR1715594_FASTQ_SSU_OTU.tsv	Root-Host_associated-Human-Digestive_system-Oral-Subgingival_plaque

SRR2154945	MGYA00151004	https://www.ebi.ac.uk/metagenomics/api/v1/analyses/MGYA00151004/file/SRR2154945_FASTQ_SSU_OTU.tsv	Root-Host_associated-Human-Digestive_system-Oral-Subgingival_plaque
SRR1715652	MGYA00148972	https://www.ebi.ac.uk/metagenomics/api/v1/analyses/MGYA00148972/file/SRR1715652_FASTQ_SSU_OTU.tsv	Root-Host_associated-Human-Digestive_system-Oral-Subgingival_plaque
SRR6316017	MGYA00293733	https://www.ebi.ac.uk/metagenomics/api/v1/analyses/MGYA00293733/file/SRR6316017_MERGED_FASTQ_SSU_OTU.tsv	Root-Host_associated-Human-Digestive_system-Oral-Subgingival_plaque
SRR3992167	MGYA00151246	https://www.ebi.ac.uk/metagenomics/api/v1/analyses/MGYA00151246/file/SRR3992167_FASTQ_SSU_OTU.tsv	Root-Host_associated-Human-Digestive_system-Oral-Subgingival_plaque
SRR2086807	MGYA00143998	https://www.ebi.ac.uk/metagenomics/api/v1/analyses/MGYA00143998/file/SRR2086807_MERGED_FASTQ_SSU_OTU.tsv	Root-Host_associated-Human-Digestive_system-Oral-Subgingival_plaque
SRR1178325	MGYA00151882	https://www.ebi.ac.uk/metagenomics/api/v1/analyses/MGYA00151882/file/SRR1178325_FASTQ_SSU_OTU.tsv	Root-Host_associated-Human-Digestive_system-Oral-Subgingival_plaque
SRR5178158	MGYA00148758	https://www.ebi.ac.uk/metagenomics/api/v1/analyses/MGYA00148758/file/SRR5178158_MERGED_FASTQ_SSU_OTU.tsv	Root-Host_associated-Human-Digestive_system-Oral-Subgingival_plaque
SRR2086762	MGYA00143935	https://www.ebi.ac.uk/metagenomics/api/v1/analyses/MGYA00143935/file/SRR2086762_MERGED_FASTQ_SSU_OTU.tsv	Root-Host_associated-Human-Digestive_system-Oral-Subgingival_plaque
SRR2154974	MGYA00151088	https://www.ebi.ac.uk/metagenomics/api/v1/analyses/MGYA00151088/file/SRR2154974_FASTQ_SSU_OTU.tsv	Root-Host_associated-Human-Digestive_system-Oral-Subgingival_plaque
SRR6239863	MGYA00168219	https://www.ebi.ac.uk/metagenomics/api/v1/analyses/MGYA00168219/file/SRR6239863_FASTQ_SSU_OTU.tsv	Root-Host_associated-Human-Digestive_system-Oral-Subgingival_plaque
SRR1715663	MGYA00148950	https://www.ebi.ac.uk/metagenomics/api/v1/analyses/MGYA00148950/file/SRR1715663_FASTQ_SSU_OTU.tsv	Root-Host_associated-Human-Digestive_system-Oral-Subgingival_plaque
SRR1715574	MGYA00148947	https://www.ebi.ac.uk/metagenomics/api/v1/analyses/MGYA00148947/file/SRR1715574_FASTQ_SSU_OTU.tsv	Root-Host_associated-Human-Digestive_system-Oral-Subgingival_plaque

DRR049471	MGYA00160340	https://www.ebi.ac.uk/metagenomics/api/v1/analyses/MGYA00160340/file/DRR049471_MERGED_FASTQ_SSU_OTU.tsv	Root-Host_associated-Human-Digestive_system-Oral-Subgingival_plaque
SRR2086753	MGYA00143988	https://www.ebi.ac.uk/metagenomics/api/v1/analyses/MGYA00143988/file/SRR2086753_MERGED_FASTQ_SSU_OTU.tsv	Root-Host_associated-Human-Digestive_system-Oral-Subgingival_plaque
SRR5178169	MGYA00148724	https://www.ebi.ac.uk/metagenomics/api/v1/analyses/MGYA00148724/file/SRR5178169_MERGED_FASTQ_SSU_OTU.tsv	Root-Host_associated-Human-Digestive_system-Oral-Subgingival_plaque
SRR2086836	MGYA00143922	https://www.ebi.ac.uk/metagenomics/api/v1/analyses/MGYA00143922/file/SRR2086836_MERGED_FASTQ_SSU_OTU.tsv	Root-Host_associated-Human-Digestive_system-Oral-Subgingival_plaque
SRR2086782	MGYA00143827	https://www.ebi.ac.uk/metagenomics/api/v1/analyses/MGYA00143827/file/SRR2086782_MERGED_FASTQ_SSU_OTU.tsv	Root-Host_associated-Human-Digestive_system-Oral-Subgingival_plaque
SRR2154908	MGYA00151007	https://www.ebi.ac.uk/metagenomics/api/v1/analyses/MGYA00151007/file/SRR2154908_FASTQ_SSU_OTU.tsv	Root-Host_associated-Human-Digestive_system-Oral-Subgingival_plaque
SRR5178115	MGYA00148799	https://www.ebi.ac.uk/metagenomics/api/v1/analyses/MGYA00148799/file/SRR5178115_MERGED_FASTQ_SSU_OTU.tsv	Root-Host_associated-Human-Digestive_system-Oral-Subgingival_plaque
SRR2086915	MGYA00144009	https://www.ebi.ac.uk/metagenomics/api/v1/analyses/MGYA00144009/file/SRR2086915_MERGED_FASTQ_SSU_OTU.tsv	Root-Host_associated-Human-Digestive_system-Oral-Subgingival_plaque
SRR1715618	MGYA00148913	https://www.ebi.ac.uk/metagenomics/api/v1/analyses/MGYA00148913/file/SRR1715618_FASTQ_SSU_OTU.tsv	Root-Host_associated-Human-Digestive_system-Oral-Subgingival_plaque
SRR2086728	MGYA00143849	https://www.ebi.ac.uk/metagenomics/api/v1/analyses/MGYA00143849/file/SRR2086728_MERGED_FASTQ_SSU_OTU.tsv	Root-Host_associated-Human-Digestive_system-Oral-Subgingival_plaque
SRR5178112	MGYA00148762	https://www.ebi.ac.uk/metagenomics/api/v1/analyses/MGYA00148762/file/SRR5178112_MERGED_FASTQ_SSU_OTU.tsv	Root-Host_associated-Human-Digestive_system-Oral-Subgingival_plaque
SRR2154973	MGYA00151069	https://www.ebi.ac.uk/metagenomics/api/v1/analyses/MGYA00151069/file/SRR2154973_FASTQ_SSU_OTU.tsv	Root-Host_associated-Human-Digestive_system-Oral-Subgingival_plaque

SRR6239864	MGYA00168181	https://www.ebi.ac.uk/metagenomics/api/v1/analyses/MGYA00168181/file/SRR6239864_FASTQ_SSU_OTU.tsv	Root-Host_associated-Human-Digestive_system-Oral-Subgingival_plaque
SRR3381548	MGYA00151189	https://www.ebi.ac.uk/metagenomics/api/v1/analyses/MGYA00151189/file/SRR3381548_FASTQ_SSU_OTU.tsv	Root-Host_associated-Human-Digestive_system-Oral-Subgingival_plaque
SRR6316021	MGYA00293737	https://www.ebi.ac.uk/metagenomics/api/v1/analyses/MGYA00293737/file/SRR6316021_MERGED_FASTQ_SSU_OTU.tsv	Root-Host_associated-Human-Digestive_system-Oral-Subgingival_plaque
SRR1715664	MGYA00148882	https://www.ebi.ac.uk/metagenomics/api/v1/analyses/MGYA00148882/file/SRR1715664_FASTQ_SSU_OTU.tsv	Root-Host_associated-Human-Digestive_system-Oral-Subgingival_plaque
SRR1715573	MGYA00148925	https://www.ebi.ac.uk/metagenomics/api/v1/analyses/MGYA00148925/file/SRR1715573_FASTQ_SSU_OTU.tsv	Root-Host_associated-Human-Digestive_system-Oral-Subgingival_plaque
DRR049476	MGYA00160335	https://www.ebi.ac.uk/metagenomics/api/v1/analyses/MGYA00160335/file/DRR049476_MERGED_FASTQ_SSU_OTU.tsv	Root-Host_associated-Human-Digestive_system-Oral-Subgingival_plaque
SRR2086754	MGYA00144020	https://www.ebi.ac.uk/metagenomics/api/v1/analyses/MGYA00144020/file/SRR2086754_MERGED_FASTQ_SSU_OTU.tsv	Root-Host_associated-Human-Digestive_system-Oral-Subgingival_plaque
SRR2086831	MGYA00143897	https://www.ebi.ac.uk/metagenomics/api/v1/analyses/MGYA00143897/file/SRR2086831_MERGED_FASTQ_SSU_OTU.tsv	Root-Host_associated-Human-Digestive_system-Oral-Subgingival_plaque
SRR2086785	MGYA00143879	https://www.ebi.ac.uk/metagenomics/api/v1/analyses/MGYA00143879/file/SRR2086785_MERGED_FASTQ_SSU_OTU.tsv	Root-Host_associated-Human-Digestive_system-Oral-Subgingival_plaque
SRR2154901	MGYA00150994	https://www.ebi.ac.uk/metagenomics/api/v1/analyses/MGYA00150994/file/SRR2154901_FASTQ_SSU_OTU.tsv	Root-Host_associated-Human-Digestive_system-Oral-Subgingival_plaque
SRR1715616	MGYA00148970	https://www.ebi.ac.uk/metagenomics/api/v1/analyses/MGYA00148970/file/SRR1715616_FASTQ_SSU_OTU.tsv	Root-Host_associated-Human-Digestive_system-Oral-Subgingival_plaque
SRR2155024	MGYA00151064	https://www.ebi.ac.uk/metagenomics/api/v1/analyses/MGYA00151064/file/SRR2155024_FASTQ_SSU_OTU.tsv	Root-Host_associated-Human-Digestive_system-Oral-Subgingival_plaque

SRR5178095	MGYA00148756	https://www.ebi.ac.uk/metagenomics/api/v1/analyses/MGYA00148756/file/SRR5178095_MERGED_FASTQ_SSU_OTU.tsv	Root-Host_associated-Human-Digestive_system-Oral-Subgingival_plaque
SRR2086726	MGYA00143955	https://www.ebi.ac.uk/metagenomics/api/v1/analyses/MGYA00143955/file/SRR2086726_MERGED_FASTQ_SSU_OTU.tsv	Root-Host_associated-Human-Digestive_system-Oral-Subgingival_plaque
SRR2086843	MGYA00143870	https://www.ebi.ac.uk/metagenomics/api/v1/analyses/MGYA00143870/file/SRR2086843_MERGED_FASTQ_SSU_OTU.tsv	Root-Host_associated-Human-Digestive_system-Oral-Subgingival_plaque
SRR3381546	MGYA00151184	https://www.ebi.ac.uk/metagenomics/api/v1/analyses/MGYA00151184/file/SRR3381546_FASTQ_SSU_OTU.tsv	Root-Host_associated-Human-Digestive_system-Oral-Subgingival_plaque
SRR1178533	MGYA00151888	https://www.ebi.ac.uk/metagenomics/api/v1/analyses/MGYA00151888/file/SRR1178533_FASTQ_SSU_OTU.tsv	Root-Host_associated-Human-Digestive_system-Oral-Subgingival_plaque
DRR049478	MGYA00160360	https://www.ebi.ac.uk/metagenomics/api/v1/analyses/MGYA00160360/file/DRR049478_MERGED_FASTQ_SSU_OTU.tsv	Root-Host_associated-Human-Digestive_system-Oral-Subgingival_plaque
SRR5178160	MGYA00148712	https://www.ebi.ac.uk/metagenomics/api/v1/analyses/MGYA00148712/file/SRR5178160_MERGED_FASTQ_SSU_OTU.tsv	Root-Host_associated-Human-Digestive_system-Oral-Subgingival_plaque
SRR2086838	MGYA00143868	https://www.ebi.ac.uk/metagenomics/api/v1/analyses/MGYA00143868/file/SRR2086838_MERGED_FASTQ_SSU_OTU.tsv	Root-Host_associated-Human-Digestive_system-Oral-Subgingival_plaque
SRR5178167	MGYA00148733	https://www.ebi.ac.uk/metagenomics/api/v1/analyses/MGYA00148733/file/SRR5178167_MERGED_FASTQ_SSU_OTU.tsv	Root-Host_associated-Human-Digestive_system-Oral-Subgingival_plaque
SRR2154906	MGYA00151021	https://www.ebi.ac.uk/metagenomics/api/v1/analyses/MGYA00151021/file/SRR2154906_FASTQ_SSU_OTU.tsv	Root-Host_associated-Human-Digestive_system-Oral-Subgingival_plaque
SRR1715611	MGYA00148900	https://www.ebi.ac.uk/metagenomics/api/v1/analyses/MGYA00148900/file/SRR1715611_FASTQ_SSU_OTU.tsv	Root-Host_associated-Human-Digestive_system-Oral-Subgingival_plaque
SRR2155023	MGYA00150953	https://www.ebi.ac.uk/metagenomics/api/v1/analyses/MGYA00150953/file/SRR2155023_FASTQ_SSU_OTU.tsv	Root-Host_associated-Human-Digestive_system-Oral-Subgingival_plaque

SRR5178092	MGYA00148752	https://www.ebi.ac.uk/metagenomics/api/v1/analyses/MGYA00148752/file/SRR5178092_MERGED_FASTQ_SSU_OTU.tsv	Root-Host_associated-Human-Digestive_system-Oral-Subgingival_plaque
SRR2086895	MGYA00143861	https://www.ebi.ac.uk/metagenomics/api/v1/analyses/MGYA00143861/file/SRR2086895_MERGED_FASTQ_SSU_OTU.tsv	Root-Host_associated-Human-Digestive_system-Oral-Subgingival_plaque
SRR2086844	MGYA00143834	https://www.ebi.ac.uk/metagenomics/api/v1/analyses/MGYA00143834/file/SRR2086844_MERGED_FASTQ_SSU_OTU.tsv	Root-Host_associated-Human-Digestive_system-Oral-Subgingival_plaque
SRR6239889	MGYA00168194	https://www.ebi.ac.uk/metagenomics/api/v1/analyses/MGYA00168194/file/SRR6239889_FASTQ_SSU_OTU.tsv	Root-Host_associated-Human-Digestive_system-Oral-Subgingival_plaque
SRR1715658	MGYA00148951	https://www.ebi.ac.uk/metagenomics/api/v1/analyses/MGYA00148951/file/SRR1715658_FASTQ_SSU_OTU.tsv	Root-Host_associated-Human-Digestive_system-Oral-Subgingival_plaque
SRR3381574	MGYA00151164	https://www.ebi.ac.uk/metagenomics/api/v1/analyses/MGYA00151164/file/SRR3381574_FASTQ_SSU_OTU.tsv	Root-Host_associated-Human-Digestive_system-Oral-Subgingival_plaque
SRR2086955	MGYA00144045	https://www.ebi.ac.uk/metagenomics/api/v1/analyses/MGYA00144045/file/SRR2086955_MERGED_FASTQ_SSU_OTU.tsv	Root-Host_associated-Human-Digestive_system-Oral-Subgingival_plaque
SRR6239900	MGYA00168221	https://www.ebi.ac.uk/metagenomics/api/v1/analyses/MGYA00168221/file/SRR6239900_FASTQ_SSU_OTU.tsv	Root-Host_associated-Human-Digestive_system-Oral-Subgingival_plaque
SRR5178152	MGYA00148763	https://www.ebi.ac.uk/metagenomics/api/v1/analyses/MGYA00148763/file/SRR5178152_MERGED_FASTQ_SSU_OTU.tsv	Root-Host_associated-Human-Digestive_system-Oral-Subgingival_plaque
SRR2086768	MGYA00143961	https://www.ebi.ac.uk/metagenomics/api/v1/analyses/MGYA00143961/file/SRR2086768_MERGED_FASTQ_SSU_OTU.tsv	Root-Host_associated-Human-Digestive_system-Oral-Subgingival_plaque
SRR2155016	MGYA00150981	https://www.ebi.ac.uk/metagenomics/api/v1/analyses/MGYA00150981/file/SRR2155016_FASTQ_SSU_OTU.tsv	Root-Host_associated-Human-Digestive_system-Oral-Subgingival_plaque
SRR1715624	MGYA00148916	https://www.ebi.ac.uk/metagenomics/api/v1/analyses/MGYA00148916/file/SRR1715624_FASTQ_SSU_OTU.tsv	Root-Host_associated-Human-Digestive_system-Oral-Subgingival_plaque

SRR2154933	MGYA00150974	https://www.ebi.ac.uk/metagenomics/api/v1/analyses/MGYA00150974/file/SRR2154933_FASTQ_SSU_OTU.tsv	Root-Host_associated-Human-Digestive_system-Oral-Subgingival_plaque
SRR2086929	MGYA00143996	https://www.ebi.ac.uk/metagenomics/api/v1/analyses/MGYA00143996/file/SRR2086929_MERGED_FASTQ_SSU_OTU.tsv	Root-Host_associated-Human-Digestive_system-Oral-Subgingival_plaque
SRR5178076	MGYA00148740	https://www.ebi.ac.uk/metagenomics/api/v1/analyses/MGYA00148740/file/SRR5178076_MERGED_FASTQ_SSU_OTU.tsv	Root-Host_associated-Human-Digestive_system-Oral-Subgingival_plaque
SRR2086871	MGYA00143869	https://www.ebi.ac.uk/metagenomics/api/v1/analyses/MGYA00143869/file/SRR2086871_MERGED_FASTQ_SSU_OTU.tsv	Root-Host_associated-Human-Digestive_system-Oral-Subgingival_plaque
SRR2155011	MGYA00150979	https://www.ebi.ac.uk/metagenomics/api/v1/analyses/MGYA00150979/file/SRR2155011_FASTQ_SSU_OTU.tsv	Root-Host_associated-Human-Digestive_system-Oral-Subgingival_plaque
SRR1715623	MGYA00148952	https://www.ebi.ac.uk/metagenomics/api/v1/analyses/MGYA00148952/file/SRR1715623_FASTQ_SSU_OTU.tsv	Root-Host_associated-Human-Digestive_system-Oral-Subgingival_plaque
SRR2154934	MGYA00150997	https://www.ebi.ac.uk/metagenomics/api/v1/analyses/MGYA00150997/file/SRR2154934_FASTQ_SSU_OTU.tsv	Root-Host_associated-Human-Digestive_system-Oral-Subgingival_plaque
SRR5178071	MGYA00148798	https://www.ebi.ac.uk/metagenomics/api/v1/analyses/MGYA00148798/file/SRR5178071_MERGED_FASTQ_SSU_OTU.tsv	Root-Host_associated-Human-Digestive_system-Oral-Subgingival_plaque
SRR5178129	MGYA00148783	https://www.ebi.ac.uk/metagenomics/api/v1/analyses/MGYA00148783/file/SRR5178129_MERGED_FASTQ_SSU_OTU.tsv	Root-Host_associated-Human-Digestive_system-Oral-Subgingival_plaque
SRR2086876	MGYA00144030	https://www.ebi.ac.uk/metagenomics/api/v1/analyses/MGYA00144030/file/SRR2086876_MERGED_FASTQ_SSU_OTU.tsv	Root-Host_associated-Human-Digestive_system-Oral-Subgingival_plaque
SRR2154999	MGYA00150980	https://www.ebi.ac.uk/metagenomics/api/v1/analyses/MGYA00150980/file/SRR2154999_FASTQ_SSU_OTU.tsv	Root-Host_associated-Human-Digestive_system-Oral-Subgingival_plaque
SRR1715599	MGYA00148884	https://www.ebi.ac.uk/metagenomics/api/v1/analyses/MGYA00148884/file/SRR1715599_FASTQ_SSU_OTU.tsv	Root-Host_associated-Human-Digestive_system-Oral-Subgingival_plaque

SRR2086952	MGYA00143835	https://www.ebi.ac.uk/metagenomics/api/v1/analyses/MGYA00143835/file/SRR2086952_MERGED_FASTQ_SSU_OTU.tsv	Root-Host_associated-Human-Digestive_system-Oral-Subgingival_plaque
SRR3381573	MGYA00151159	https://www.ebi.ac.uk/metagenomics/api/v1/analyses/MGYA00151159/file/SRR3381573_FASTQ_SSU_OTU.tsv	Root-Host_associated-Human-Digestive_system-Oral-Subgingival_plaque
SRR2154948	MGYA00151093	https://www.ebi.ac.uk/metagenomics/api/v1/analyses/MGYA00151093/file/SRR2154948_FASTQ_SSU_OTU.tsv	Root-Host_associated-Human-Digestive_system-Oral-Subgingival_plaque
SRR6239907	MGYA00168165	https://www.ebi.ac.uk/metagenomics/api/v1/analyses/MGYA00168165/file/SRR6239907_FASTQ_SSU_OTU.tsv	Root-Host_associated-Human-Digestive_system-Oral-Subgingival_plaque
SRR5178155	MGYA00148760	https://www.ebi.ac.uk/metagenomics/api/v1/analyses/MGYA00148760/file/SRR5178155_MERGED_FASTQ_SSU_OTU.tsv	Root-Host_associated-Human-Digestive_system-Oral-Subgingival_plaque
SRR2086920	MGYA00143889	https://www.ebi.ac.uk/metagenomics/api/v1/analyses/MGYA00143889/file/SRR2086920_MERGED_FASTQ_SSU_OTU.tsv	Root-Host_associated-Human-Digestive_system-Oral-Subgingival_plaque
SRR2086878	MGYA00143842	https://www.ebi.ac.uk/metagenomics/api/v1/analyses/MGYA00143842/file/SRR2086878_MERGED_FASTQ_SSU_OTU.tsv	Root-Host_associated-Human-Digestive_system-Oral-Subgingival_plaque
SRR5178127	MGYA00148727	https://www.ebi.ac.uk/metagenomics/api/v1/analyses/MGYA00148727/file/SRR5178127_MERGED_FASTQ_SSU_OTU.tsv	Root-Host_associated-Human-Digestive_system-Oral-Subgingival_plaque
SRR2154997	MGYA00151076	https://www.ebi.ac.uk/metagenomics/api/v1/analyses/MGYA00151076/file/SRR2154997_FASTQ_SSU_OTU.tsv	Root-Host_associated-Human-Digestive_system-Oral-Subgingival_plaque
SRR6239880	MGYA00168161	https://www.ebi.ac.uk/metagenomics/api/v1/analyses/MGYA00168161/file/SRR6239880_FASTQ_SSU_OTU.tsv	Root-Host_associated-Human-Digestive_system-Oral-Subgingival_plaque
SRR6316014	MGYA00293730	https://www.ebi.ac.uk/metagenomics/api/v1/analyses/MGYA00293730/file/SRR6316014_MERGED_FASTQ_SSU_OTU.tsv	Root-Host_associated-Human-Digestive_system-Oral-Subgingival_plaque
SRR1715651	MGYA00148969	https://www.ebi.ac.uk/metagenomics/api/v1/analyses/MGYA00148969/file/SRR1715651_FASTQ_SSU_OTU.tsv	Root-Host_associated-Human-Digestive_system-Oral-Subgingival_plaque

SRR2154946	MGYA00151092	https://www.ebi.ac.uk/metagenomics/api/v1/analyses/MGYA00151092/file/SRR2154946_FASTQ_SSU_OTU.tsv	Root-Host_associated-Human-Digestive_system-Oral-Subgingival_plaque
SRR1715597	MGYA00148901	https://www.ebi.ac.uk/metagenomics/api/v1/analyses/MGYA00148901/file/SRR1715597_FASTQ_SSU_OTU.tsv	Root-Host_associated-Human-Digestive_system-Oral-Subgingival_plaque
DRR049492	MGYA00160365	https://www.ebi.ac.uk/metagenomics/api/v1/analyses/MGYA00160365/file/DRR049492_MERGED_FASTQ_SSU_OTU.tsv	Root-Host_associated-Human-Digestive_system-Oral-Subgingival_plaque
SRR6239909	MGYA00168197	https://www.ebi.ac.uk/metagenomics/api/v1/analyses/MGYA00168197/file/SRR6239909_FASTQ_SSU_OTU.tsv	Root-Host_associated-Human-Digestive_system-Oral-Subgingival_plaque
SRR2086804	MGYA00143907	https://www.ebi.ac.uk/metagenomics/api/v1/analyses/MGYA00143907/file/SRR2086804_MERGED_FASTQ_SSU_OTU.tsv	Root-Host_associated-Human-Digestive_system-Oral-Subgingival_plaque
SRR2086761	MGYA00143913	https://www.ebi.ac.uk/metagenomics/api/v1/analyses/MGYA00143913/file/SRR2086761_MERGED_FASTQ_SSU_OTU.tsv	Root-Host_associated-Human-Digestive_system-Oral-Subgingival_plaque
SRR2154990	MGYA00151096	https://www.ebi.ac.uk/metagenomics/api/v1/analyses/MGYA00151096/file/SRR2154990_FASTQ_SSU_OTU.tsv	Root-Host_associated-Human-Digestive_system-Oral-Subgingival_plaque
SRR6239887	MGYA00168203	https://www.ebi.ac.uk/metagenomics/api/v1/analyses/MGYA00168203/file/SRR6239887_FASTQ_SSU_OTU.tsv	Root-Host_associated-Human-Digestive_system-Oral-Subgingival_plaque
SRR6316013	MGYA00293729	https://www.ebi.ac.uk/metagenomics/api/v1/analyses/MGYA00293729/file/SRR6316013_MERGED_FASTQ_SSU_OTU.tsv	Root-Host_associated-Human-Digestive_system-Oral-Subgingival_plaque
SRR1715656	MGYA00148885	https://www.ebi.ac.uk/metagenomics/api/v1/analyses/MGYA00148885/file/SRR1715656_FASTQ_SSU_OTU.tsv	Root-Host_associated-Human-Digestive_system-Oral-Subgingival_plaque
SRR2154941	MGYA00151000	https://www.ebi.ac.uk/metagenomics/api/v1/analyses/MGYA00151000/file/SRR2154941_FASTQ_SSU_OTU.tsv	Root-Host_associated-Human-Digestive_system-Oral-Subgingival_plaque
SRR1715590	MGYA00148892	https://www.ebi.ac.uk/metagenomics/api/v1/analyses/MGYA00148892/file/SRR1715590_FASTQ_SSU_OTU.tsv	Root-Host_associated-Human-Digestive_system-Oral-Subgingival_plaque

DRR049495	MGYA00160339	https://www.ebi.ac.uk/metagenomics/api/v1/analyses/MGYA00160339/file/DRR049495_MERGED_FASTQ_SSU_OTU.tsv	Root-Host_associated-Human-Digestive_system-Oral-Subgingival_plaque
SRR2086803	MGYA00143875	https://www.ebi.ac.uk/metagenomics/api/v1/analyses/MGYA00143875/file/SRR2086803_MERGED_FASTQ_SSU_OTU.tsv	Root-Host_associated-Human-Digestive_system-Oral-Subgingival_plaque
SRR2086766	MGYA00143950	https://www.ebi.ac.uk/metagenomics/api/v1/analyses/MGYA00143950/file/SRR2086766_MERGED_FASTQ_SSU_OTU.tsv	Root-Host_associated-Human-Digestive_system-Oral-Subgingival_plaque
SRR2155018	MGYA00151044	https://www.ebi.ac.uk/metagenomics/api/v1/analyses/MGYA00151044/file/SRR2155018_FASTQ_SSU_OTU.tsv	Root-Host_associated-Human-Digestive_system-Oral-Subgingival_plaque
SRR5178078	MGYA00148731	https://www.ebi.ac.uk/metagenomics/api/v1/analyses/MGYA00148731/file/SRR5178078_MERGED_FASTQ_SSU_OTU.tsv	Root-Host_associated-Human-Digestive_system-Oral-Subgingival_plaque
SRR2086927	MGYA00144007	https://www.ebi.ac.uk/metagenomics/api/v1/analyses/MGYA00144007/file/SRR2086927_MERGED_FASTQ_SSU_OTU.tsv	Root-Host_associated-Human-Digestive_system-Oral-Subgingival_plaque
SRR5178120	MGYA00148737	https://www.ebi.ac.uk/metagenomics/api/v1/analyses/MGYA00148737/file/SRR5178120_MERGED_FASTQ_SSU_OTU.tsv	Root-Host_associated-Human-Digestive_system-Oral-Subgingival_plaque
SRR2086777	MGYA00143951	https://www.ebi.ac.uk/metagenomics/api/v1/analyses/MGYA00143951/file/SRR2086777_MERGED_FASTQ_SSU_OTU.tsv	Root-Host_associated-Human-Digestive_system-Oral-Subgingival_plaque
SRR2086812	MGYA00143886	https://www.ebi.ac.uk/metagenomics/api/v1/analyses/MGYA00143886/file/SRR2086812_MERGED_FASTQ_SSU_OTU.tsv	Root-Host_associated-Human-Digestive_system-Oral-Subgingival_plaque
SRR1715647	MGYA00148923	https://www.ebi.ac.uk/metagenomics/api/v1/analyses/MGYA00148923/file/SRR1715647_FASTQ_SSU_OTU.tsv	Root-Host_associated-Human-Digestive_system-Oral-Subgingival_plaque
SRR1745935	MGYA00168136	https://www.ebi.ac.uk/metagenomics/api/v1/analyses/MGYA00168136/file/SRR1745935_FASTQ_SSU_OTU.tsv	Root-Host_associated-Human-Digestive_system-Oral-Subgingival_plaque
SRR3992172	MGYA00151244	https://www.ebi.ac.uk/metagenomics/api/v1/analyses/MGYA00151244/file/SRR3992172_FASTQ_SSU_OTU.tsv	Root-Host_associated-Human-Digestive_system-Oral-Subgingival_plaque

SRR1715581	MGYA00148931	https://www.ebi.ac.uk/metagenomics/api/v1/analyses/MGYA00148931/file/SRR1715581_FASTQ_SSU_OTU.tsv	Root-Host_associated-Human-Digestive_system-Oral-Subgingival_plaque
DRR049484	MGYA00160344	https://www.ebi.ac.uk/metagenomics/api/v1/analyses/MGYA00160344/file/DRR049484_MERGED_FASTQ_SSU_OTU.tsv	Root-Host_associated-Human-Digestive_system-Oral-Subgingival_plaque
SRR2154950	MGYA00151095	https://www.ebi.ac.uk/metagenomics/api/v1/analyses/MGYA00151095/file/SRR2154950_FASTQ_SSU_OTU.tsv	Root-Host_associated-Human-Digestive_system-Oral-Subgingival_plaque
SRR2154981	MGYA00151031	https://www.ebi.ac.uk/metagenomics/api/v1/analyses/MGYA00151031/file/SRR2154981_FASTQ_SSU_OTU.tsv	Root-Host_associated-Human-Digestive_system-Oral-Subgingival_plaque
SRR6239896	MGYA00168210	https://www.ebi.ac.uk/metagenomics/api/v1/analyses/MGYA00168210/file/SRR6239896_FASTQ_SSU_OTU.tsv	Root-Host_associated-Human-Digestive_system-Oral-Subgingival_plaque
DRR049455	MGYA00160331	https://www.ebi.ac.uk/metagenomics/api/v1/analyses/MGYA00160331/file/DRR049455_MERGED_FASTQ_SSU_OTU.tsv	Root-Host_associated-Human-Digestive_system-Oral-Subgingival_plaque
SRR5178131	MGYA00148792	https://www.ebi.ac.uk/metagenomics/api/v1/analyses/MGYA00148792/file/SRR5178131_MERGED_FASTQ_SSU_OTU.tsv	Root-Host_associated-Human-Digestive_system-Oral-Subgingival_plaque
SRR2086936	MGYA00143919	https://www.ebi.ac.uk/metagenomics/api/v1/analyses/MGYA00143919/file/SRR2086936_MERGED_FASTQ_SSU_OTU.tsv	Root-Host_associated-Human-Digestive_system-Oral-Subgingival_plaque
SRR2155009	MGYA00151023	https://www.ebi.ac.uk/metagenomics/api/v1/analyses/MGYA00151023/file/SRR2155009_FASTQ_SSU_OTU.tsv	Root-Host_associated-Human-Digestive_system-Oral-Subgingival_plaque
SRR5178136	MGYA00148713	https://www.ebi.ac.uk/metagenomics/api/v1/analyses/MGYA00148713/file/SRR5178136_MERGED_FASTQ_SSU_OTU.tsv	Root-Host_associated-Human-Digestive_system-Oral-Subgingival_plaque
SRR2086869	MGYA00143903	https://www.ebi.ac.uk/metagenomics/api/v1/analyses/MGYA00143903/file/SRR2086869_MERGED_FASTQ_SSU_OTU.tsv	Root-Host_associated-Human-Digestive_system-Oral-Subgingival_plaque
SRR2086931	MGYA00143964	https://www.ebi.ac.uk/metagenomics/api/v1/analyses/MGYA00143964/file/SRR2086931_MERGED_FASTQ_SSU_OTU.tsv	Root-Host_associated-Human-Digestive_system-Oral-Subgingival_plaque

SRR2086770	MGYA00144042	https://www.ebi.ac.uk/metagenomics/api/v1/analyses/MGYA00144042/file/SRR2086770_MERGED_FASTQ_SSU_OTU.tsv	Root-Host_associated-Human-Digestive_system-Oral-Subgingival_plaque
SRR6239918	MGYA00168218	https://www.ebi.ac.uk/metagenomics/api/v1/analyses/MGYA00168218/file/SRR6239918_FASTQ_SSU_OTU.tsv	Root-Host_associated-Human-Digestive_system-Oral-Subgingival_plaque
SRR2086815	MGYA00143929	https://www.ebi.ac.uk/metagenomics/api/v1/analyses/MGYA00143929/file/SRR2086815_MERGED_FASTQ_SSU_OTU.tsv	Root-Host_associated-Human-Digestive_system-Oral-Subgingival_plaque
SRR1715640	MGYA00148964	https://www.ebi.ac.uk/metagenomics/api/v1/analyses/MGYA00148964/file/SRR1715640_FASTQ_SSU_OTU.tsv	Root-Host_associated-Human-Digestive_system-Oral-Subgingival_plaque
SRR6316005	MGYA00293721	https://www.ebi.ac.uk/metagenomics/api/v1/analyses/MGYA00293721/file/SRR6316005_MERGED_FASTQ_SSU_OTU.tsv	Root-Host_associated-Human-Digestive_system-Oral-Subgingival_plaque
SRR1745932	MGYA00168142	https://www.ebi.ac.uk/metagenomics/api/v1/analyses/MGYA00168142/file/SRR1745932_FASTQ_SSU_OTU.tsv	Root-Host_associated-Human-Digestive_system-Oral-Subgingival_plaque
SRR1715586	MGYA00148880	https://www.ebi.ac.uk/metagenomics/api/v1/analyses/MGYA00148880/file/SRR1715586_FASTQ_SSU_OTU.tsv	Root-Host_associated-Human-Digestive_system-Oral-Subgingival_plaque
DRR049483	MGYA00160348	https://www.ebi.ac.uk/metagenomics/api/v1/analyses/MGYA00160348/file/DRR049483_MERGED_FASTQ_SSU_OTU.tsv	Root-Host_associated-Human-Digestive_system-Oral-Subgingival_plaque
SRR2154957	MGYA00150984	https://www.ebi.ac.uk/metagenomics/api/v1/analyses/MGYA00150984/file/SRR2154957_FASTQ_SSU_OTU.tsv	Root-Host_associated-Human-Digestive_system-Oral-Subgingival_plaque
SRR2154986	MGYA00151006	https://www.ebi.ac.uk/metagenomics/api/v1/analyses/MGYA00151006/file/SRR2154986_FASTQ_SSU_OTU.tsv	Root-Host_associated-Human-Digestive_system-Oral-Subgingival_plaque
SRR6239891	MGYA00168182	https://www.ebi.ac.uk/metagenomics/api/v1/analyses/MGYA00168182/file/SRR6239891_FASTQ_SSU_OTU.tsv	Root-Host_associated-Human-Digestive_system-Oral-Subgingival_plaque
SRR2086867	MGYA00143900	https://www.ebi.ac.uk/metagenomics/api/v1/analyses/MGYA00143900/file/SRR2086867_MERGED_FASTQ_SSU_OTU.tsv	Root-Host_associated-Human-Digestive_system-Oral-Subgingival_plaque

SRR5178138	MGYA00148785	https://www.ebi.ac.uk/metagenomics/api/v1/analyses/MGYA00148785/file/SRR5178138_MERGED_FASTQ_SSU_OTU.tsv	Root-Host_associated-Human-Digestive_system-Oral-Subgingival_plaque
SRR1715632	MGYA00148976	https://www.ebi.ac.uk/metagenomics/api/v1/analyses/MGYA00148976/file/SRR1715632_FASTQ_SSU_OTU.tsv	Root-Host_associated-Human-Digestive_system-Oral-Subgingival_plaque
SRR1745940	MGYA00168143	https://www.ebi.ac.uk/metagenomics/api/v1/analyses/MGYA00168143/file/SRR1745940_FASTQ_SSU_OTU.tsv	Root-Host_associated-Human-Digestive_system-Oral-Subgingival_plaque
SRR2154925	MGYA00151052	https://www.ebi.ac.uk/metagenomics/api/v1/analyses/MGYA00151052/file/SRR2154925_FASTQ_SSU_OTU.tsv	Root-Host_associated-Human-Digestive_system-Oral-Subgingival_plaque
SRR2155000	MGYA00151082	https://www.ebi.ac.uk/metagenomics/api/v1/analyses/MGYA00151082/file/SRR2155000_FASTQ_SSU_OTU.tsv	Root-Host_associated-Human-Digestive_system-Oral-Subgingival_plaque
SRR5178144	MGYA00148794	https://www.ebi.ac.uk/metagenomics/api/v1/analyses/MGYA00148794/file/SRR5178144_MERGED_FASTQ_SSU_OTU.tsv	Root-Host_associated-Human-Digestive_system-Oral-Subgingival_plaque
SRR6239916	MGYA00168173	https://www.ebi.ac.uk/metagenomics/api/v1/analyses/MGYA00168173/file/SRR6239916_FASTQ_SSU_OTU.tsv	Root-Host_associated-Human-Digestive_system-Oral-Subgingival_plaque
SRR3381562	MGYA00151162	https://www.ebi.ac.uk/metagenomics/api/v1/analyses/MGYA00151162/file/SRR3381562_FASTQ_SSU_OTU.tsv	Root-Host_associated-Human-Digestive_system-Oral-Subgingival_plaque
SRR2086943	MGYA00143887	https://www.ebi.ac.uk/metagenomics/api/v1/analyses/MGYA00143887/file/SRR2086943_MERGED_FASTQ_SSU_OTU.tsv	Root-Host_associated-Human-Digestive_system-Oral-Subgingival_plaque
SRR2154959	MGYA00151001	https://www.ebi.ac.uk/metagenomics/api/v1/analyses/MGYA00151001/file/SRR2154959_FASTQ_SSU_OTU.tsv	Root-Host_associated-Human-Digestive_system-Oral-Subgingival_plaque
SRR1715588	MGYA00148921	https://www.ebi.ac.uk/metagenomics/api/v1/analyses/MGYA00148921/file/SRR1715588_FASTQ_SSU_OTU.tsv	Root-Host_associated-Human-Digestive_system-Oral-Subgingival_plaque
SRR2154988	MGYA00150993	https://www.ebi.ac.uk/metagenomics/api/v1/analyses/MGYA00150993/file/SRR2154988_FASTQ_SSU_OTU.tsv	Root-Host_associated-Human-Digestive_system-Oral-Subgingival_plaque

SRR2086779	MGYA00143982	https://www.ebi.ac.uk/metagenomics/api/v1/analyses/MGYA00143982/file/SRR2086779_MERGED_FASTQ_SSU_OTU.tsv	Root-Host_associated-Human-Digestive_system-Oral-Subgingival_plaque
SRR5178143	MGYA00148734	https://www.ebi.ac.uk/metagenomics/api/v1/analyses/MGYA00148734/file/SRR5178143_MERGED_FASTQ_SSU_OTU.tsv	Root-Host_associated-Human-Digestive_system-Oral-Subgingival_plaque
SRR6239911	MGYA00168198	https://www.ebi.ac.uk/metagenomics/api/v1/analyses/MGYA00168198/file/SRR6239911_FASTQ_SSU_OTU.tsv	Root-Host_associated-Human-Digestive_system-Oral-Subgingival_plaque
SRR3745301	MGYA00148774	https://www.ebi.ac.uk/metagenomics/api/v1/analyses/MGYA00148774/file/SRR3745301_MERGED_FASTQ_SSU_OTU.tsv	Root-Host_associated-Human-Digestive_system-Oral-Subgingival_plaque
SRR1715649	MGYA00148886	https://www.ebi.ac.uk/metagenomics/api/v1/analyses/MGYA00148886/file/SRR1715649_FASTQ_SSU_OTU.tsv	Root-Host_associated-Human-Digestive_system-Oral-Subgingival_plaque
SRR2086944	MGYA00144026	https://www.ebi.ac.uk/metagenomics/api/v1/analyses/MGYA00144026/file/SRR2086944_MERGED_FASTQ_SSU_OTU.tsv	Root-Host_associated-Human-Digestive_system-Oral-Subgingival_plaque
SRR3381565	MGYA00151153	https://www.ebi.ac.uk/metagenomics/api/v1/analyses/MGYA00151153/file/SRR3381565_FASTQ_SSU_OTU.tsv	Root-Host_associated-Human-Digestive_system-Oral-Subgingival_plaque
SRR6239898	MGYA00168168	https://www.ebi.ac.uk/metagenomics/api/v1/analyses/MGYA00168168/file/SRR6239898_FASTQ_SSU_OTU.tsv	Root-Host_associated-Human-Digestive_system-Oral-Subgingival_plaque
SRR2086860	MGYA00143910	https://www.ebi.ac.uk/metagenomics/api/v1/analyses/MGYA00143910/file/SRR2086860_MERGED_FASTQ_SSU_OTU.tsv	Root-Host_associated-Human-Digestive_system-Oral-Subgingival_plaque
SRR1715635	MGYA00148898	https://www.ebi.ac.uk/metagenomics/api/v1/analyses/MGYA00148898/file/SRR1715635_FASTQ_SSU_OTU.tsv	Root-Host_associated-Human-Digestive_system-Oral-Subgingival_plaque
SRR2154922	MGYA00150976	https://www.ebi.ac.uk/metagenomics/api/v1/analyses/MGYA00150976/file/SRR2154922_FASTQ_SSU_OTU.tsv	Root-Host_associated-Human-Digestive_system-Oral-Subgingival_plaque
SRR2086938	MGYA00143839	https://www.ebi.ac.uk/metagenomics/api/v1/analyses/MGYA00143839/file/SRR2086938_MERGED_FASTQ_SSU_OTU.tsv	Root-Host_associated-Human-Digestive_system-Oral-Subgingival_plaque

SRR2155007	MGYA00150967	https://www.ebi.ac.uk/metagenomics/api/v1/analyses/MGYA00150967/file/SRR2155007_FASTQ_SSU_OTU.tsv	Root-Host_associated-Human-Digestive_system-Oral-Subgingival_plaque
SRR6239924	MGYA00168157	https://www.ebi.ac.uk/metagenomics/api/v1/analyses/MGYA00168157/file/SRR6239924_FASTQ_SSU_OTU.tsv	Root-Host_associated-Human-Digestive_system-Oral-Subgingival_plaque
SRR2086829	MGYA00144006	https://www.ebi.ac.uk/metagenomics/api/v1/analyses/MGYA00144006/file/SRR2086829_MERGED_FASTQ_SSU_OTU.tsv	Root-Host_associated-Human-Digestive_system-Oral-Subgingival_plaque
SRR3381581	MGYA00151191	https://www.ebi.ac.uk/metagenomics/api/v1/analyses/MGYA00151191/file/SRR3381581_FASTQ_SSU_OTU.tsv	Root-Host_associated-Human-Digestive_system-Oral-Subgingival_plaque
SRR3381550	MGYA00151157	https://www.ebi.ac.uk/metagenomics/api/v1/analyses/MGYA00151157/file/SRR3381550_FASTQ_SSU_OTU.tsv	Root-Host_associated-Human-Digestive_system-Oral-Subgingival_plaque
SRR2086855	MGYA00144047	https://www.ebi.ac.uk/metagenomics/api/v1/analyses/MGYA00144047/file/SRR2086855_MERGED_FASTQ_SSU_OTU.tsv	Root-Host_associated-Human-Digestive_system-Oral-Subgingival_plaque
SRR2086730	MGYA00143836	https://www.ebi.ac.uk/metagenomics/api/v1/analyses/MGYA00143836/file/SRR2086730_MERGED_FASTQ_SSU_OTU.tsv	Root-Host_associated-Human-Digestive_system-Oral-Subgingival_plaque
SRR1178401	MGYA00151878	https://www.ebi.ac.uk/metagenomics/api/v1/analyses/MGYA00151878/file/SRR1178401_FASTQ_SSU_OTU.tsv	Root-Host_associated-Human-Digestive_system-Oral-Subgingival_plaque
SRR2086884	MGYA00143911	https://www.ebi.ac.uk/metagenomics/api/v1/analyses/MGYA00143911/file/SRR2086884_MERGED_FASTQ_SSU_OTU.tsv	Root-Host_associated-Human-Digestive_system-Oral-Subgingival_plaque
SRR2155032	MGYA00151080	https://www.ebi.ac.uk/metagenomics/api/v1/analyses/MGYA00151080/file/SRR2155032_FASTQ_SSU_OTU.tsv	Root-Host_associated-Human-Digestive_system-Oral-Subgingival_plaque
SRR5178083	MGYA00148790	https://www.ebi.ac.uk/metagenomics/api/v1/analyses/MGYA00148790/file/SRR5178083_MERGED_FASTQ_SSU_OTU.tsv	Root-Host_associated-Human-Digestive_system-Oral-Subgingival_plaque
SRR2154917	MGYA00151024	https://www.ebi.ac.uk/metagenomics/api/v1/analyses/MGYA00151024/file/SRR2154917_FASTQ_SSU_OTU.tsv	Root-Host_associated-Human-Digestive_system-Oral-Subgingival_plaque

SRR1715600	MGYA00148891	https://www.ebi.ac.uk/metagenomics/api/v1/analyses/MGYA00148891/file/SRR1715600_FASTQ_SSU_OTU.tsv	Root-Host_associated-Human-Digestive_system-Oral-Subgingival_plaque
SRR2086852	MGYA00143981	https://www.ebi.ac.uk/metagenomics/api/v1/analyses/MGYA00143981/file/SRR2086852_MERGED_FASTQ_SSU_OTU.tsv	Root-Host_associated-Human-Digestive_system-Oral-Subgingival_plaque
SRR2086737	MGYA00143970	https://www.ebi.ac.uk/metagenomics/api/v1/analyses/MGYA00143970/file/SRR2086737_MERGED_FASTQ_SSU_OTU.tsv	Root-Host_associated-Human-Digestive_system-Oral-Subgingival_plaque
SRR2154899	MGYA00150999	https://www.ebi.ac.uk/metagenomics/api/v1/analyses/MGYA00150999/file/SRR2154899_FASTQ_SSU_OTU.tsv	Root-Host_associated-Human-Digestive_system-Oral-Subgingival_plaque
SRR2086883	MGYA00143962	https://www.ebi.ac.uk/metagenomics/api/v1/analyses/MGYA00143962/file/SRR2086883_MERGED_FASTQ_SSU_OTU.tsv	Root-Host_associated-Human-Digestive_system-Oral-Subgingival_plaque
SRR2155035	MGYA00151027	https://www.ebi.ac.uk/metagenomics/api/v1/analyses/MGYA00151027/file/SRR2155035_FASTQ_SSU_OTU.tsv	Root-Host_associated-Human-Digestive_system-Oral-Subgingival_plaque
SRR5178084	MGYA00148753	https://www.ebi.ac.uk/metagenomics/api/v1/analyses/MGYA00148753/file/SRR5178084_MERGED_FASTQ_SSU_OTU.tsv	Root-Host_associated-Human-Digestive_system-Oral-Subgingival_plaque
SRR2154910	MGYA00151039	https://www.ebi.ac.uk/metagenomics/api/v1/analyses/MGYA00151039/file/SRR2154910_FASTQ_SSU_OTU.tsv	Root-Host_associated-Human-Digestive_system-Oral-Subgingival_plaque
SRR1715607	MGYA00148957	https://www.ebi.ac.uk/metagenomics/api/v1/analyses/MGYA00148957/file/SRR1715607_FASTQ_SSU_OTU.tsv	Root-Host_associated-Human-Digestive_system-Oral-Subgingival_plaque
SRR5178171	MGYA00148776	https://www.ebi.ac.uk/metagenomics/api/v1/analyses/MGYA00148776/file/SRR5178171_MERGED_FASTQ_SSU_OTU.tsv	Root-Host_associated-Human-Digestive_system-Oral-Subgingival_plaque
SRR6239923	MGYA00168214	https://www.ebi.ac.uk/metagenomics/api/v1/analyses/MGYA00168214/file/SRR6239923_FASTQ_SSU_OTU.tsv	Root-Host_associated-Human-Digestive_system-Oral-Subgingival_plaque
DRR049469	MGYA00160332	https://www.ebi.ac.uk/metagenomics/api/v1/analyses/MGYA00160332/file/DRR049469_MERGED_FASTQ_SSU_OTU.tsv	Root-Host_associated-Human-Digestive_system-Oral-Subgingival_plaque

SRR3381557	MGYA00151167	https://www.ebi.ac.uk/metagenomics/api/v1/analyses/MGYA00151167/file/SRR3381557_FASTQ_SSU_OTU.tsv	Root-Host_associated-Human-Digestive_system-Oral-Subgingival_plaque
SRR5178103	MGYA00148757	https://www.ebi.ac.uk/metagenomics/api/v1/analyses/MGYA00148757/file/SRR5178103_MERGED_FASTQ_SSU_OTU.tsv	Root-Host_associated-Human-Digestive_system-Oral-Subgingival_plaque
SRR2086739	MGYA00143872	https://www.ebi.ac.uk/metagenomics/api/v1/analyses/MGYA00143872/file/SRR2086739_MERGED_FASTQ_SSU_OTU.tsv	Root-Host_associated-Human-Digestive_system-Oral-Subgingival_plaque
SRR2154897	MGYA00150950	https://www.ebi.ac.uk/metagenomics/api/v1/analyses/MGYA00150950/file/SRR2154897_FASTQ_SSU_OTU.tsv	Root-Host_associated-Human-Digestive_system-Oral-Subgingival_plaque
SRR2086904	MGYA00143863	https://www.ebi.ac.uk/metagenomics/api/v1/analyses/MGYA00143863/file/SRR2086904_MERGED_FASTQ_SSU_OTU.tsv	Root-Host_associated-Human-Digestive_system-Oral-Subgingival_plaque
SRR1715609	MGYA00148937	https://www.ebi.ac.uk/metagenomics/api/v1/analyses/MGYA00148937/file/SRR1715609_FASTQ_SSU_OTU.tsv	Root-Host_associated-Human-Digestive_system-Oral-Subgingival_plaque
SRR2086820	MGYA00144046	https://www.ebi.ac.uk/metagenomics/api/v1/analyses/MGYA00144046/file/SRR2086820_MERGED_FASTQ_SSU_OTU.tsv	Root-Host_associated-Human-Digestive_system-Oral-Subgingival_plaque
SRR2086794	MGYA00143952	https://www.ebi.ac.uk/metagenomics/api/v1/analyses/MGYA00143952/file/SRR2086794_MERGED_FASTQ_SSU_OTU.tsv	Root-Host_associated-Human-Digestive_system-Oral-Subgingival_plaque
SRR2086745	MGYA00144019	https://www.ebi.ac.uk/metagenomics/api/v1/analyses/MGYA00144019/file/SRR2086745_MERGED_FASTQ_SSU_OTU.tsv	Root-Host_associated-Human-Digestive_system-Oral-Subgingival_plaque
DRR049467	MGYA00160336	https://www.ebi.ac.uk/metagenomics/api/v1/analyses/MGYA00160336/file/DRR049467_MERGED_FASTQ_SSU_OTU.tsv	Root-Host_associated-Human-Digestive_system-Oral-Subgingival_plaque
SRR2154962	MGYA00151068	https://www.ebi.ac.uk/metagenomics/api/v1/analyses/MGYA00151068/file/SRR2154962_FASTQ_SSU_OTU.tsv	Root-Host_associated-Human-Digestive_system-Oral-Subgingival_plaque
SRR6239875	MGYA00168171	https://www.ebi.ac.uk/metagenomics/api/v1/analyses/MGYA00168171/file/SRR6239875_FASTQ_SSU_OTU.tsv	Root-Host_associated-Human-Digestive_system-Oral-Subgingival_plaque

SRR3381559	MGYA00151165	https://www.ebi.ac.uk/metagenomics/api/v1/analyses/MGYA00151165/file/SRR3381559_FASTQ_SSU_OTU.tsv	Root-Host_associated-Human-Digestive_system-Oral-Subgingival_plaque
SRR2086827	MGYA00143958	https://www.ebi.ac.uk/metagenomics/api/v1/analyses/MGYA00143958/file/SRR2086827_MERGED_FASTQ_SSU_OTU.tsv	Root-Host_associated-Human-Digestive_system-Oral-Subgingival_plaque
SRR2086793	MGYA00143993	https://www.ebi.ac.uk/metagenomics/api/v1/analyses/MGYA00143993/file/SRR2086793_MERGED_FASTQ_SSU_OTU.tsv	Root-Host_associated-Human-Digestive_system-Oral-Subgingival_plaque
SRR2086742	MGYA00143912	https://www.ebi.ac.uk/metagenomics/api/v1/analyses/MGYA00143912/file/SRR2086742_MERGED_FASTQ_SSU_OTU.tsv	Root-Host_associated-Human-Digestive_system-Oral-Subgingival_plaque
DRR049460	MGYA00160350	https://www.ebi.ac.uk/metagenomics/api/v1/analyses/MGYA00160350/file/DRR049460_MERGED_FASTQ_SSU_OTU.tsv	Root-Host_associated-Human-Digestive_system-Oral-Subgingival_plaque
SRR2154965	MGYA00151067	https://www.ebi.ac.uk/metagenomics/api/v1/analyses/MGYA00151067/file/SRR2154965_FASTQ_SSU_OTU.tsv	Root-Host_associated-Human-Digestive_system-Oral-Subgingival_plaque
SRR6239872	MGYA00168184	https://www.ebi.ac.uk/metagenomics/api/v1/analyses/MGYA00168184/file/SRR6239872_FASTQ_SSU_OTU.tsv	Root-Host_associated-Human-Digestive_system-Oral-Subgingival_plaque
SRR5178104	MGYA00148754	https://www.ebi.ac.uk/metagenomics/api/v1/analyses/MGYA00148754/file/SRR5178104_MERGED_FASTQ_SSU_OTU.tsv	Root-Host_associated-Human-Digestive_system-Oral-Subgingival_plaque
SRR2086903	MGYA00144043	https://www.ebi.ac.uk/metagenomics/api/v1/analyses/MGYA00144043/file/SRR2086903_MERGED_FASTQ_SSU_OTU.tsv	Root-Host_associated-Human-Digestive_system-Oral-Subgingival_plaque
SRR2154919	MGYA00150969	https://www.ebi.ac.uk/metagenomics/api/v1/analyses/MGYA00150969/file/SRR2154919_FASTQ_SSU_OTU.tsv	Root-Host_associated-Human-Digestive_system-Oral-Subgingival_plaque
SRR6239886	MGYA00168187	https://www.ebi.ac.uk/metagenomics/api/v1/analyses/MGYA00168187/file/SRR6239886_FASTQ_SSU_OTU.tsv	Root-Host_associated-Human-Digestive_system-Oral-Subgingival_plaque
SRR2154991	MGYA00151020	https://www.ebi.ac.uk/metagenomics/api/v1/analyses/MGYA00151020/file/SRR2154991_FASTQ_SSU_OTU.tsv	Root-Host_associated-Human-Digestive_system-Oral-Subgingival_plaque

SRR1715657	MGYA00148942	https://www.ebi.ac.uk/metagenomics/api/v1/analyses/MGYA00148942/file/SRR1715657_FASTQ_SSU_OTU.tsv	Root-Host_associated-Human-Digestive_system-Oral-Subgingival_plaque
SRR6316012	MGYA00293728	https://www.ebi.ac.uk/metagenomics/api/v1/analyses/MGYA00293728/file/SRR6316012_MERGED_FASTQ_SSU_OTU.tsv	Root-Host_associated-Human-Digestive_system-Oral-Subgingival_plaque
DRR049494	MGYA00160375	https://www.ebi.ac.uk/metagenomics/api/v1/analyses/MGYA00160375/file/DRR049494_MERGED_FASTQ_SSU_OTU.tsv	Root-Host_associated-Human-Digestive_system-Oral-Subgingival_plaque
SRR1715591	MGYA00148932	https://www.ebi.ac.uk/metagenomics/api/v1/analyses/MGYA00148932/file/SRR1715591_FASTQ_SSU_OTU.tsv	Root-Host_associated-Human-Digestive_system-Oral-Subgingival_plaque
SRR2154940	MGYA00151009	https://www.ebi.ac.uk/metagenomics/api/v1/analyses/MGYA00151009/file/SRR2154940_FASTQ_SSU_OTU.tsv	Root-Host_associated-Human-Digestive_system-Oral-Subgingival_plaque
SRR2086802	MGYA00144024	https://www.ebi.ac.uk/metagenomics/api/v1/analyses/MGYA00144024/file/SRR2086802_MERGED_FASTQ_SSU_OTU.tsv	Root-Host_associated-Human-Digestive_system-Oral-Subgingival_plaque
SRR2086767	MGYA00143940	https://www.ebi.ac.uk/metagenomics/api/v1/analyses/MGYA00143940/file/SRR2086767_MERGED_FASTQ_SSU_OTU.tsv	Root-Host_associated-Human-Digestive_system-Oral-Subgingival_plaque
SRR2155019	MGYA00151051	https://www.ebi.ac.uk/metagenomics/api/v1/analyses/MGYA00151051/file/SRR2155019_FASTQ_SSU_OTU.tsv	Root-Host_associated-Human-Digestive_system-Oral-Subgingival_plaque
SRR2086926	MGYA00143963	https://www.ebi.ac.uk/metagenomics/api/v1/analyses/MGYA00143963/file/SRR2086926_MERGED_FASTQ_SSU_OTU.tsv	Root-Host_associated-Human-Digestive_system-Oral-Subgingival_plaque
SRR5178079	MGYA00148742	https://www.ebi.ac.uk/metagenomics/api/v1/analyses/MGYA00148742/file/SRR5178079_MERGED_FASTQ_SSU_OTU.tsv	Root-Host_associated-Human-Digestive_system-Oral-Subgingival_plaque
SRR5178121	MGYA00148778	https://www.ebi.ac.uk/metagenomics/api/v1/analyses/MGYA00148778/file/SRR5178121_MERGED_FASTQ_SSU_OTU.tsv	Root-Host_associated-Human-Digestive_system-Oral-Subgingival_plaque
SRR2086921	MGYA00143890	https://www.ebi.ac.uk/metagenomics/api/v1/analyses/MGYA00143890/file/SRR2086921_MERGED_FASTQ_SSU_OTU.tsv	Root-Host_associated-Human-Digestive_system-Oral-Subgingival_plaque

SRR5178126	MGYA00148780	https://www.ebi.ac.uk/metagenomics/api/v1/analyses/MGYA00148780/file/SRR5178126_MERGED_FASTQ_SSU_OTU.tsv	Root-Host_associated-Human-Digestive_system-Oral-Subgingival_plaque
SRR2086879	MGYA00143942	https://www.ebi.ac.uk/metagenomics/api/v1/analyses/MGYA00143942/file/SRR2086879_MERGED_FASTQ_SSU_OTU.tsv	Root-Host_associated-Human-Digestive_system-Oral-Subgingival_plaque
SRR6239881	MGYA00168223	https://www.ebi.ac.uk/metagenomics/api/v1/analyses/MGYA00168223/file/SRR6239881_FASTQ_SSU_OTU.tsv	Root-Host_associated-Human-Digestive_system-Oral-Subgingival_plaque
SRR2154996	MGYA00151086	https://www.ebi.ac.uk/metagenomics/api/v1/analyses/MGYA00151086/file/SRR2154996_FASTQ_SSU_OTU.tsv	Root-Host_associated-Human-Digestive_system-Oral-Subgingival_plaque
SRR1715650	MGYA00148946	https://www.ebi.ac.uk/metagenomics/api/v1/analyses/MGYA00148946/file/SRR1715650_FASTQ_SSU_OTU.tsv	Root-Host_associated-Human-Digestive_system-Oral-Subgingival_plaque
SRR6316015	MGYA00293731	https://www.ebi.ac.uk/metagenomics/api/v1/analyses/MGYA00293731/file/SRR6316015_MERGED_FASTQ_SSU_OTU.tsv	Root-Host_associated-Human-Digestive_system-Oral-Subgingival_plaque
SRR3992165	MGYA00151252	https://www.ebi.ac.uk/metagenomics/api/v1/analyses/MGYA00151252/file/SRR3992165_FASTQ_SSU_OTU.tsv	Root-Host_associated-Human-Digestive_system-Oral-Subgingival_plaque
DRR049493	MGYA00160370	https://www.ebi.ac.uk/metagenomics/api/v1/analyses/MGYA00160370/file/DRR049493_MERGED_FASTQ_SSU_OTU.tsv	Root-Host_associated-Human-Digestive_system-Oral-Subgingival_plaque
SRR1715596	MGYA00148878	https://www.ebi.ac.uk/metagenomics/api/v1/analyses/MGYA00148878/file/SRR1715596_FASTQ_SSU_OTU.tsv	Root-Host_associated-Human-Digestive_system-Oral-Subgingival_plaque
SRR2154947	MGYA00150966	https://www.ebi.ac.uk/metagenomics/api/v1/analyses/MGYA00150966/file/SRR2154947_FASTQ_SSU_OTU.tsv	Root-Host_associated-Human-Digestive_system-Oral-Subgingival_plaque
SRR6239908	MGYA00168193	https://www.ebi.ac.uk/metagenomics/api/v1/analyses/MGYA00168193/file/SRR6239908_FASTQ_SSU_OTU.tsv	Root-Host_associated-Human-Digestive_system-Oral-Subgingival_plaque
SRR1178327	MGYA00151885	https://www.ebi.ac.uk/metagenomics/api/v1/analyses/MGYA00151885/file/SRR1178327_FASTQ_SSU_OTU.tsv	Root-Host_associated-Human-Digestive_system-Oral-Subgingival_plaque

SRR2086805	MGYA00144022	https://www.ebi.ac.uk/metagenomics/api/v1/analyses/MGYA00144022/file/SRR2086805_MERGED_FASTQ_SSU_OTU.tsv	Root-Host_associated-Human-Digestive_system-Oral-Subgingival_plaque
SRR2086760	MGYA00143831	https://www.ebi.ac.uk/metagenomics/api/v1/analyses/MGYA00143831/file/SRR2086760_MERGED_FASTQ_SSU_OTU.tsv	Root-Host_associated-Human-Digestive_system-Oral-Subgingival_plaque
SRR2155010	MGYA00151045	https://www.ebi.ac.uk/metagenomics/api/v1/analyses/MGYA00151045/file/SRR2155010_FASTQ_SSU_OTU.tsv	Root-Host_associated-Human-Digestive_system-Oral-Subgingival_plaque
SRR1715622	MGYA00148934	https://www.ebi.ac.uk/metagenomics/api/v1/analyses/MGYA00148934/file/SRR1715622_FASTQ_SSU_OTU.tsv	Root-Host_associated-Human-Digestive_system-Oral-Subgingival_plaque
SRR5178070	MGYA00148749	https://www.ebi.ac.uk/metagenomics/api/v1/analyses/MGYA00148749/file/SRR5178070_MERGED_FASTQ_SSU_OTU.tsv	Root-Host_associated-Human-Digestive_system-Oral-Subgingival_plaque
SRR2154935	MGYA00151016	https://www.ebi.ac.uk/metagenomics/api/v1/analyses/MGYA00151016/file/SRR2154935_FASTQ_SSU_OTU.tsv	Root-Host_associated-Human-Digestive_system-Oral-Subgingival_plaque
SRR2086877	MGYA00143947	https://www.ebi.ac.uk/metagenomics/api/v1/analyses/MGYA00143947/file/SRR2086877_MERGED_FASTQ_SSU_OTU.tsv	Root-Host_associated-Human-Digestive_system-Oral-Subgingival_plaque
SRR5178128	MGYA00148729	https://www.ebi.ac.uk/metagenomics/api/v1/analyses/MGYA00148729/file/SRR5178128_MERGED_FASTQ_SSU_OTU.tsv	Root-Host_associated-Human-Digestive_system-Oral-Subgingival_plaque
SRR2154998	MGYA00150959	https://www.ebi.ac.uk/metagenomics/api/v1/analyses/MGYA00150959/file/SRR2154998_FASTQ_SSU_OTU.tsv	Root-Host_associated-Human-Digestive_system-Oral-Subgingival_plaque
SRR3381572	MGYA00151161	https://www.ebi.ac.uk/metagenomics/api/v1/analyses/MGYA00151161/file/SRR3381572_FASTQ_SSU_OTU.tsv	Root-Host_associated-Human-Digestive_system-Oral-Subgingival_plaque
SRR2086953	MGYA00143880	https://www.ebi.ac.uk/metagenomics/api/v1/analyses/MGYA00143880/file/SRR2086953_MERGED_FASTQ_SSU_OTU.tsv	Root-Host_associated-Human-Digestive_system-Oral-Subgingival_plaque
SRR2154949	MGYA00151037	https://www.ebi.ac.uk/metagenomics/api/v1/analyses/MGYA00151037/file/SRR2154949_FASTQ_SSU_OTU.tsv	Root-Host_associated-Human-Digestive_system-Oral-Subgingival_plaque

SRR1715598	MGYA00148928	https://www.ebi.ac.uk/metagenomics/api/v1/analyses/MGYA00148928/file/SRR1715598_FASTQ_SSU_OTU.tsv	Root-Host_associated-Human-Digestive_system-Oral-Subgingival_plaque
SRR5178154	MGYA00148802	https://www.ebi.ac.uk/metagenomics/api/v1/analyses/MGYA00148802/file/SRR5178154_MERGED_FASTQ_SSU_OTU.tsv	Root-Host_associated-Human-Digestive_system-Oral-Subgingival_plaque
SRR6239906	MGYA00168213	https://www.ebi.ac.uk/metagenomics/api/v1/analyses/MGYA00168213/file/SRR6239906_FASTQ_SSU_OTU.tsv	Root-Host_associated-Human-Digestive_system-Oral-Subgingival_plaque
SRR6239888	MGYA00168156	https://www.ebi.ac.uk/metagenomics/api/v1/analyses/MGYA00168156/file/SRR6239888_FASTQ_SSU_OTU.tsv	Root-Host_associated-Human-Digestive_system-Oral-Subgingival_plaque
SRR1715659	MGYA00148965	https://www.ebi.ac.uk/metagenomics/api/v1/analyses/MGYA00148965/file/SRR1715659_FASTQ_SSU_OTU.tsv	Root-Host_associated-Human-Digestive_system-Oral-Subgingival_plaque
SRR6239859	MGYA00168222	https://www.ebi.ac.uk/metagenomics/api/v1/analyses/MGYA00168222/file/SRR6239859_FASTQ_SSU_OTU.tsv	Root-Host_associated-Human-Digestive_system-Oral-Subgingival_plaque
SRR2086954	MGYA00143925	https://www.ebi.ac.uk/metagenomics/api/v1/analyses/MGYA00143925/file/SRR2086954_MERGED_FASTQ_SSU_OTU.tsv	Root-Host_associated-Human-Digestive_system-Oral-Subgingival_plaque
SRR3381575	MGYA00151180	https://www.ebi.ac.uk/metagenomics/api/v1/analyses/MGYA00151180/file/SRR3381575_FASTQ_SSU_OTU.tsv	Root-Host_associated-Human-Digestive_system-Oral-Subgingival_plaque
SRR5178153	MGYA00148764	https://www.ebi.ac.uk/metagenomics/api/v1/analyses/MGYA00148764/file/SRR5178153_MERGED_FASTQ_SSU_OTU.tsv	Root-Host_associated-Human-Digestive_system-Oral-Subgingival_plaque
SRR6239901	MGYA00168159	https://www.ebi.ac.uk/metagenomics/api/v1/analyses/MGYA00168159/file/SRR6239901_FASTQ_SSU_OTU.tsv	Root-Host_associated-Human-Digestive_system-Oral-Subgingival_plaque
SRR2086769	MGYA00143920	https://www.ebi.ac.uk/metagenomics/api/v1/analyses/MGYA00143920/file/SRR2086769_MERGED_FASTQ_SSU_OTU.tsv	Root-Host_associated-Human-Digestive_system-Oral-Subgingival_plaque
SRR2155017	MGYA00151014	https://www.ebi.ac.uk/metagenomics/api/v1/analyses/MGYA00151014/file/SRR2155017_FASTQ_SSU_OTU.tsv	Root-Host_associated-Human-Digestive_system-Oral-Subgingival_plaque

SRR1715625	MGYA00148910	https://www.ebi.ac.uk/metagenomics/api/v1/analyses/MGYA00148910/file/SRR1715625_FASTQ_SSU_OTU.tsv	Root-Host_associated-Human-Digestive_system-Oral-Subgingival_plaque
SRR5178077	MGYA00148801	https://www.ebi.ac.uk/metagenomics/api/v1/analyses/MGYA00148801/file/SRR5178077_MERGED_FASTQ_SSU_OTU.tsv	Root-Host_associated-Human-Digestive_system-Oral-Subgingival_plaque
SRR2154932	MGYA00150963	https://www.ebi.ac.uk/metagenomics/api/v1/analyses/MGYA00150963/file/SRR2154932_FASTQ_SSU_OTU.tsv	Root-Host_associated-Human-Digestive_system-Oral-Subgingival_plaque
SRR2086928	MGYA00143865	https://www.ebi.ac.uk/metagenomics/api/v1/analyses/MGYA00143865/file/SRR2086928_MERGED_FASTQ_SSU_OTU.tsv	Root-Host_associated-Human-Digestive_system-Oral-Subgingival_plaque
SRR2086870	MGYA00144039	https://www.ebi.ac.uk/metagenomics/api/v1/analyses/MGYA00144039/file/SRR2086870_MERGED_FASTQ_SSU_OTU.tsv	Root-Host_associated-Human-Digestive_system-Oral-Subgingival_plaque
SRR5178166	MGYA00148787	https://www.ebi.ac.uk/metagenomics/api/v1/analyses/MGYA00148787/file/SRR5178166_MERGED_FASTQ_SSU_OTU.tsv	Root-Host_associated-Human-Digestive_system-Oral-Subgingival_plaque
SRR2086839	MGYA00143847	https://www.ebi.ac.uk/metagenomics/api/v1/analyses/MGYA00143847/file/SRR2086839_MERGED_FASTQ_SSU_OTU.tsv	Root-Host_associated-Human-Digestive_system-Oral-Subgingival_plaque
SRR2154907	MGYA00151062	https://www.ebi.ac.uk/metagenomics/api/v1/analyses/MGYA00151062/file/SRR2154907_FASTQ_SSU_OTU.tsv	Root-Host_associated-Human-Digestive_system-Oral-Subgingival_plaque
SRR1715610	MGYA00148917	https://www.ebi.ac.uk/metagenomics/api/v1/analyses/MGYA00148917/file/SRR1715610_FASTQ_SSU_OTU.tsv	Root-Host_associated-Human-Digestive_system-Oral-Subgingival_plaque
SRR2155022	MGYA00151057	https://www.ebi.ac.uk/metagenomics/api/v1/analyses/MGYA00151057/file/SRR2155022_FASTQ_SSU_OTU.tsv	Root-Host_associated-Human-Digestive_system-Oral-Subgingival_plaque
SRR5178093	MGYA00148810	https://www.ebi.ac.uk/metagenomics/api/v1/analyses/MGYA00148810/file/SRR5178093_MERGED_FASTQ_SSU_OTU.tsv	Root-Host_associated-Human-Digestive_system-Oral-Subgingival_plaque
SRR2086845	MGYA00143984	https://www.ebi.ac.uk/metagenomics/api/v1/analyses/MGYA00143984/file/SRR2086845_MERGED_FASTQ_SSU_OTU.tsv	Root-Host_associated-Human-Digestive_system-Oral-Subgingival_plaque

SRR2154900	MGYA00151029	https://www.ebi.ac.uk/metagenomics/api/v1/analyses/MGYA00151029/file/SRR2154900_FASTQ_SSU_OTU.tsv	Root-Host_associated-Human-Digestive_system-Oral-Subgingival_plaque
SRR1715617	MGYA00148894	https://www.ebi.ac.uk/metagenomics/api/v1/analyses/MGYA00148894/file/SRR1715617_FASTQ_SSU_OTU.tsv	Root-Host_associated-Human-Digestive_system-Oral-Subgingival_plaque
SRR2155025	MGYA00151085	https://www.ebi.ac.uk/metagenomics/api/v1/analyses/MGYA00151085/file/SRR2155025_FASTQ_SSU_OTU.tsv	Root-Host_associated-Human-Digestive_system-Oral-Subgingival_plaque
SRR5178094	MGYA00148759	https://www.ebi.ac.uk/metagenomics/api/v1/analyses/MGYA00148759/file/SRR5178094_MERGED_FASTQ_SSU_OTU.tsv	Root-Host_associated-Human-Digestive_system-Oral-Subgingival_plaque
SRR2086727	MGYA00144018	https://www.ebi.ac.uk/metagenomics/api/v1/analyses/MGYA00144018/file/SRR2086727_MERGED_FASTQ_SSU_OTU.tsv	Root-Host_associated-Human-Digestive_system-Oral-Subgingival_plaque
SRR2086842	MGYA00144012	https://www.ebi.ac.uk/metagenomics/api/v1/analyses/MGYA00144012/file/SRR2086842_MERGED_FASTQ_SSU_OTU.tsv	Root-Host_associated-Human-Digestive_system-Oral-Subgingival_plaque
SRR3381547	MGYA00151170	https://www.ebi.ac.uk/metagenomics/api/v1/analyses/MGYA00151170/file/SRR3381547_FASTQ_SSU_OTU.tsv	Root-Host_associated-Human-Digestive_system-Oral-Subgingival_plaque
DRR049479	MGYA00160373	https://www.ebi.ac.uk/metagenomics/api/v1/analyses/MGYA00160373/file/DRR049479_MERGED_FASTQ_SSU_OTU.tsv	Root-Host_associated-Human-Digestive_system-Oral-Subgingival_plaque
SRR5178161	MGYA00148747	https://www.ebi.ac.uk/metagenomics/api/v1/analyses/MGYA00148747/file/SRR5178161_MERGED_FASTQ_SSU_OTU.tsv	Root-Host_associated-Human-Digestive_system-Oral-Subgingival_plaque
SRR1715619	MGYA00148973	https://www.ebi.ac.uk/metagenomics/api/v1/analyses/MGYA00148973/file/SRR1715619_FASTQ_SSU_OTU.tsv	Root-Host_associated-Human-Digestive_system-Oral-Subgingival_plaque
SRR2086729	MGYA00143969	https://www.ebi.ac.uk/metagenomics/api/v1/analyses/MGYA00143969/file/SRR2086729_MERGED_FASTQ_SSU_OTU.tsv	Root-Host_associated-Human-Digestive_system-Oral-Subgingival_plaque
SRR5178113	MGYA00148777	https://www.ebi.ac.uk/metagenomics/api/v1/analyses/MGYA00148777/file/SRR5178113_MERGED_FASTQ_SSU_OTU.tsv	Root-Host_associated-Human-Digestive_system-Oral-Subgingival_plaque

SRR6239865	MGYA00168169	https://www.ebi.ac.uk/metagenomics/api/v1/analyses/MGYA00168169/file/SRR6239865_FASTQ_SSU_OTU.tsv	Root-Host_associated-Human-Digestive_system-Oral-Subgingival_plaque
SRR2154972	MGYA00151026	https://www.ebi.ac.uk/metagenomics/api/v1/analyses/MGYA00151026/file/SRR2154972_FASTQ_SSU_OTU.tsv	Root-Host_associated-Human-Digestive_system-Oral-Subgingival_plaque
SRR3381549	MGYA00151169	https://www.ebi.ac.uk/metagenomics/api/v1/analyses/MGYA00151169/file/SRR3381549_FASTQ_SSU_OTU.tsv	Root-Host_associated-Human-Digestive_system-Oral-Subgingival_plaque
SRR1715665	MGYA00148943	https://www.ebi.ac.uk/metagenomics/api/v1/analyses/MGYA00148943/file/SRR1715665_FASTQ_SSU_OTU.tsv	Root-Host_associated-Human-Digestive_system-Oral-Subgingival_plaque
SRR6316020	MGYA00293736	https://www.ebi.ac.uk/metagenomics/api/v1/analyses/MGYA00293736/file/SRR6316020_MERGED_FASTQ_SSU_OTU.tsv	Root-Host_associated-Human-Digestive_system-Oral-Subgingival_plaque
DRR049477	MGYA00160369	https://www.ebi.ac.uk/metagenomics/api/v1/analyses/MGYA00160369/file/DRR049477_MERGED_FASTQ_SSU_OTU.tsv	Root-Host_associated-Human-Digestive_system-Oral-Subgingival_plaque
SRR1715572	MGYA00148940	https://www.ebi.ac.uk/metagenomics/api/v1/analyses/MGYA00148940/file/SRR1715572_FASTQ_SSU_OTU.tsv	Root-Host_associated-Human-Digestive_system-Oral-Subgingival_plaque
SRR2086755	MGYA00143858	https://www.ebi.ac.uk/metagenomics/api/v1/analyses/MGYA00143858/file/SRR2086755_MERGED_FASTQ_SSU_OTU.tsv	Root-Host_associated-Human-Digestive_system-Oral-Subgingival_plaque
SRR2086830	MGYA00143992	https://www.ebi.ac.uk/metagenomics/api/v1/analyses/MGYA00143992/file/SRR2086830_MERGED_FASTQ_SSU_OTU.tsv	Root-Host_associated-Human-Digestive_system-Oral-Subgingival_plaque
SRR2086784	MGYA00143867	https://www.ebi.ac.uk/metagenomics/api/v1/analyses/MGYA00143867/file/SRR2086784_MERGED_FASTQ_SSU_OTU.tsv	Root-Host_associated-Human-Digestive_system-Oral-Subgingival_plaque
SRR6239862	MGYA00168201	https://www.ebi.ac.uk/metagenomics/api/v1/analyses/MGYA00168201/file/SRR6239862_FASTQ_SSU_OTU.tsv	Root-Host_associated-Human-Digestive_system-Oral-Subgingival_plaque
SRR2154975	MGYA00151054	https://www.ebi.ac.uk/metagenomics/api/v1/analyses/MGYA00151054/file/SRR2154975_FASTQ_SSU_OTU.tsv	Root-Host_associated-Human-Digestive_system-Oral-Subgingival_plaque

SRR1715662	MGYA00148903	https://www.ebi.ac.uk/metagenomics/api/v1/analyses/MGYA00148903/file/SRR1715662_FASTQ_SSU_OTU.tsv	Root-Host_associated-Human-Digestive_system-Oral-Subgingival_plaque
DRR049470	MGYA00160345	https://www.ebi.ac.uk/metagenomics/api/v1/analyses/MGYA00160345/file/DRR049470_MERGED_FASTQ_SSU_OTU.tsv	Root-Host_associated-Human-Digestive_system-Oral-Subgingival_plaque
SRR1715575	MGYA00148897	https://www.ebi.ac.uk/metagenomics/api/v1/analyses/MGYA00148897/file/SRR1715575_FASTQ_SSU_OTU.tsv	Root-Host_associated-Human-Digestive_system-Oral-Subgingival_plaque
SRR2086752	MGYA00143979	https://www.ebi.ac.uk/metagenomics/api/v1/analyses/MGYA00143979/file/SRR2086752_MERGED_FASTQ_SSU_OTU.tsv	Root-Host_associated-Human-Digestive_system-Oral-Subgingival_plaque
SRR2086837	MGYA00143894	https://www.ebi.ac.uk/metagenomics/api/v1/analyses/MGYA00143894/file/SRR2086837_MERGED_FASTQ_SSU_OTU.tsv	Root-Host_associated-Human-Digestive_system-Oral-Subgingival_plaque
SRR5178168	MGYA00148791	https://www.ebi.ac.uk/metagenomics/api/v1/analyses/MGYA00148791/file/SRR5178168_MERGED_FASTQ_SSU_OTU.tsv	Root-Host_associated-Human-Digestive_system-Oral-Subgingival_plaque
SRR2086783	MGYA00143980	https://www.ebi.ac.uk/metagenomics/api/v1/analyses/MGYA00143980/file/SRR2086783_MERGED_FASTQ_SSU_OTU.tsv	Root-Host_associated-Human-Digestive_system-Oral-Subgingival_plaque
SRR2086913	MGYA00144015	https://www.ebi.ac.uk/metagenomics/api/v1/analyses/MGYA00144015/file/SRR2086913_MERGED_FASTQ_SSU_OTU.tsv	Root-Host_associated-Human-Digestive_system-Oral-Subgingival_plaque
SRR2154909	MGYA00151060	https://www.ebi.ac.uk/metagenomics/api/v1/analyses/MGYA00151060/file/SRR2154909_FASTQ_SSU_OTU.tsv	Root-Host_associated-Human-Digestive_system-Oral-Subgingival_plaque
SRR5178114	MGYA00148811	https://www.ebi.ac.uk/metagenomics/api/v1/analyses/MGYA00148811/file/SRR5178114_MERGED_FASTQ_SSU_OTU.tsv	Root-Host_associated-Human-Digestive_system-Oral-Subgingival_plaque
SRR2086826	MGYA00143874	https://www.ebi.ac.uk/metagenomics/api/v1/analyses/MGYA00143874/file/SRR2086826_MERGED_FASTQ_SSU_OTU.tsv	Root-Host_associated-Human-Digestive_system-Oral-Subgingival_plaque
SRR2086792	MGYA00143892	https://www.ebi.ac.uk/metagenomics/api/v1/analyses/MGYA00143892/file/SRR2086792_MERGED_FASTQ_SSU_OTU.tsv	Root-Host_associated-Human-Digestive_system-Oral-Subgingival_plaque

SRR2086743	MGYA00144035	https://www.ebi.ac.uk/metagenomics/api/v1/analyses/MGYA00144035/file/SRR2086743_MERGED_FASTQ_SSU_OTU.tsv	Root-Host_associated-Human-Digestive_system-Oral-Subgingival_plaque
DRR049461	MGYA00160357	https://www.ebi.ac.uk/metagenomics/api/v1/analyses/MGYA00160357/file/DRR049461_MERGED_FASTQ_SSU_OTU.tsv	Root-Host_associated-Human-Digestive_system-Oral-Subgingival_plaque
SRR6239873	MGYA00168216	https://www.ebi.ac.uk/metagenomics/api/v1/analyses/MGYA00168216/file/SRR6239873_FASTQ_SSU_OTU.tsv	Root-Host_associated-Human-Digestive_system-Oral-Subgingival_plaque
SRR2154964	MGYA00151055	https://www.ebi.ac.uk/metagenomics/api/v1/analyses/MGYA00151055/file/SRR2154964_FASTQ_SSU_OTU.tsv	Root-Host_associated-Human-Digestive_system-Oral-Subgingival_plaque
SRR5178105	MGYA00148761	https://www.ebi.ac.uk/metagenomics/api/v1/analyses/MGYA00148761/file/SRR5178105_MERGED_FASTQ_SSU_OTU.tsv	Root-Host_associated-Human-Digestive_system-Oral-Subgingival_plaque
SRR2086902	MGYA00143960	https://www.ebi.ac.uk/metagenomics/api/v1/analyses/MGYA00143960/file/SRR2086902_MERGED_FASTQ_SSU_OTU.tsv	Root-Host_associated-Human-Digestive_system-Oral-Subgingival_plaque
SRR2154918	MGYA00151078	https://www.ebi.ac.uk/metagenomics/api/v1/analyses/MGYA00151078/file/SRR2154918_FASTQ_SSU_OTU.tsv	Root-Host_associated-Human-Digestive_system-Oral-Subgingival_plaque
SRR5178102	MGYA00148809	https://www.ebi.ac.uk/metagenomics/api/v1/analyses/MGYA00148809/file/SRR5178102_MERGED_FASTQ_SSU_OTU.tsv	Root-Host_associated-Human-Digestive_system-Oral-Subgingival_plaque
SRR2086738	MGYA00143997	https://www.ebi.ac.uk/metagenomics/api/v1/analyses/MGYA00143997/file/SRR2086738_MERGED_FASTQ_SSU_OTU.tsv	Root-Host_associated-Human-Digestive_system-Oral-Subgingival_plaque
SRR2154896	MGYA00151038	https://www.ebi.ac.uk/metagenomics/api/v1/analyses/MGYA00151038/file/SRR2154896_FASTQ_SSU_OTU.tsv	Root-Host_associated-Human-Digestive_system-Oral-Subgingival_plaque
SRR2086905	MGYA00143885	https://www.ebi.ac.uk/metagenomics/api/v1/analyses/MGYA00143885/file/SRR2086905_MERGED_FASTQ_SSU_OTU.tsv	Root-Host_associated-Human-Digestive_system-Oral-Subgingival_plaque
SRR1715608	MGYA00148963	https://www.ebi.ac.uk/metagenomics/api/v1/analyses/MGYA00148963/file/SRR1715608_FASTQ_SSU_OTU.tsv	Root-Host_associated-Human-Digestive_system-Oral-Subgingival_plaque

SRR2086821	MGYA00143989	https://www.ebi.ac.uk/metagenomics/api/v1/analyses/MGYA00143989/file/SRR2086821_MERGED_FASTQ_SSU_OTU.tsv	Root-Host_associated-Human-Digestive_system-Oral-Subgingival_plaque
SRR2086795	MGYA00144029	https://www.ebi.ac.uk/metagenomics/api/v1/analyses/MGYA00144029/file/SRR2086795_MERGED_FASTQ_SSU_OTU.tsv	Root-Host_associated-Human-Digestive_system-Oral-Subgingival_plaque
SRR2086744	MGYA00143893	https://www.ebi.ac.uk/metagenomics/api/v1/analyses/MGYA00143893/file/SRR2086744_MERGED_FASTQ_SSU_OTU.tsv	Root-Host_associated-Human-Digestive_system-Oral-Subgingival_plaque
DRR049466	MGYA00160347	https://www.ebi.ac.uk/metagenomics/api/v1/analyses/MGYA00160347/file/DRR049466_MERGED_FASTQ_SSU_OTU.tsv	Root-Host_associated-Human-Digestive_system-Oral-Subgingival_plaque
SRR6239874	MGYA00168204	https://www.ebi.ac.uk/metagenomics/api/v1/analyses/MGYA00168204/file/SRR6239874_FASTQ_SSU_OTU.tsv	Root-Host_associated-Human-Digestive_system-Oral-Subgingival_plaque
SRR2154963	MGYA00150952	https://www.ebi.ac.uk/metagenomics/api/v1/analyses/MGYA00150952/file/SRR2154963_FASTQ_SSU_OTU.tsv	Root-Host_associated-Human-Digestive_system-Oral-Subgingival_plaque
SRR3381558	MGYA00151178	https://www.ebi.ac.uk/metagenomics/api/v1/analyses/MGYA00151178/file/SRR3381558_FASTQ_SSU_OTU.tsv	Root-Host_associated-Human-Digestive_system-Oral-Subgingival_plaque
SRR2086853	MGYA00143938	https://www.ebi.ac.uk/metagenomics/api/v1/analyses/MGYA00143938/file/SRR2086853_MERGED_FASTQ_SSU_OTU.tsv	Root-Host_associated-Human-Digestive_system-Oral-Subgingival_plaque
SRR2086736	MGYA00143975	https://www.ebi.ac.uk/metagenomics/api/v1/analyses/MGYA00143975/file/SRR2086736_MERGED_FASTQ_SSU_OTU.tsv	Root-Host_associated-Human-Digestive_system-Oral-Subgingival_plaque
SRR2154898	MGYA00150985	https://www.ebi.ac.uk/metagenomics/api/v1/analyses/MGYA00150985/file/SRR2154898_FASTQ_SSU_OTU.tsv	Root-Host_associated-Human-Digestive_system-Oral-Subgingival_plaque
SRR2086882	MGYA00144003	https://www.ebi.ac.uk/metagenomics/api/v1/analyses/MGYA00144003/file/SRR2086882_MERGED_FASTQ_SSU_OTU.tsv	Root-Host_associated-Human-Digestive_system-Oral-Subgingival_plaque
SRR2155034	MGYA00151058	https://www.ebi.ac.uk/metagenomics/api/v1/analyses/MGYA00151058/file/SRR2155034_FASTQ_SSU_OTU.tsv	Root-Host_associated-Human-Digestive_system-Oral-Subgingival_plaque

SRR5178085	MGYA00148723	https://www.ebi.ac.uk/metagenomics/api/v1/analyses/MGYA00148723/file/SRR5178085_MERGED_FASTQ_SSU_OTU.tsv	Root-Host_associated-Human-Digestive_system-Oral-Subgingival_plaque
SRR2154911	MGYA00150982	https://www.ebi.ac.uk/metagenomics/api/v1/analyses/MGYA00150982/file/SRR2154911_FASTQ_SSU_OTU.tsv	Root-Host_associated-Human-Digestive_system-Oral-Subgingival_plaque
SRR1715606	MGYA00148958	https://www.ebi.ac.uk/metagenomics/api/v1/analyses/MGYA00148958/file/SRR1715606_FASTQ_SSU_OTU.tsv	Root-Host_associated-Human-Digestive_system-Oral-Subgingival_plaque
SRR6239922	MGYA00168186	https://www.ebi.ac.uk/metagenomics/api/v1/analyses/MGYA00168186/file/SRR6239922_FASTQ_SSU_OTU.tsv	Root-Host_associated-Human-Digestive_system-Oral-Subgingival_plaque
SRR5178170	MGYA00148748	https://www.ebi.ac.uk/metagenomics/api/v1/analyses/MGYA00148748/file/SRR5178170_MERGED_FASTQ_SSU_OTU.tsv	Root-Host_associated-Human-Digestive_system-Oral-Subgingival_plaque
DRR049468	MGYA00160368	https://www.ebi.ac.uk/metagenomics/api/v1/analyses/MGYA00160368/file/DRR049468_MERGED_FASTQ_SSU_OTU.tsv	Root-Host_associated-Human-Digestive_system-Oral-Subgingival_plaque
SRR3381556	MGYA00151183	https://www.ebi.ac.uk/metagenomics/api/v1/analyses/MGYA00151183/file/SRR3381556_FASTQ_SSU_OTU.tsv	Root-Host_associated-Human-Digestive_system-Oral-Subgingival_plaque
SRR6239925	MGYA00168191	https://www.ebi.ac.uk/metagenomics/api/v1/analyses/MGYA00168191/file/SRR6239925_FASTQ_SSU_OTU.tsv	Root-Host_associated-Human-Digestive_system-Oral-Subgingival_plaque
SRR2086828	MGYA00143832	https://www.ebi.ac.uk/metagenomics/api/v1/analyses/MGYA00143832/file/SRR2086828_MERGED_FASTQ_SSU_OTU.tsv	Root-Host_associated-Human-Digestive_system-Oral-Subgingival_plaque
SRR1178524	MGYA00151879	https://www.ebi.ac.uk/metagenomics/api/v1/analyses/MGYA00151879/file/SRR1178524_FASTQ_SSU_OTU.tsv	Root-Host_associated-Human-Digestive_system-Oral-Subgingival_plaque
SRR3381580	MGYA00151181	https://www.ebi.ac.uk/metagenomics/api/v1/analyses/MGYA00151181/file/SRR3381580_FASTQ_SSU_OTU.tsv	Root-Host_associated-Human-Digestive_system-Oral-Subgingival_plaque
SRR3381551	MGYA00151192	https://www.ebi.ac.uk/metagenomics/api/v1/analyses/MGYA00151192/file/SRR3381551_FASTQ_SSU_OTU.tsv	Root-Host_associated-Human-Digestive_system-Oral-Subgingival_plaque

SRR2086854	MGYA00143944	https://www.ebi.ac.uk/metagenomics/api/v1/analyses/MGYA00143944/file/SRR2086854_MERGED_FASTQ_SSU_OTU.tsv	Root-Host_associated-Human-Digestive_system-Oral-Subgingival_plaque
SRR2086731	MGYA00143846	https://www.ebi.ac.uk/metagenomics/api/v1/analyses/MGYA00143846/file/SRR2086731_MERGED_FASTQ_SSU_OTU.tsv	Root-Host_associated-Human-Digestive_system-Oral-Subgingival_plaque
SRR2086885	MGYA00144028	https://www.ebi.ac.uk/metagenomics/api/v1/analyses/MGYA00144028/file/SRR2086885_MERGED_FASTQ_SSU_OTU.tsv	Root-Host_associated-Human-Digestive_system-Oral-Subgingival_plaque
SRR2155033	MGYA00150962	https://www.ebi.ac.uk/metagenomics/api/v1/analyses/MGYA00150962/file/SRR2155033_FASTQ_SSU_OTU.tsv	Root-Host_associated-Human-Digestive_system-Oral-Subgingival_plaque
SRR5178082	MGYA00148730	https://www.ebi.ac.uk/metagenomics/api/v1/analyses/MGYA00148730/file/SRR5178082_MERGED_FASTQ_SSU_OTU.tsv	Root-Host_associated-Human-Digestive_system-Oral-Subgingival_plaque
SRR2154916	MGYA00150965	https://www.ebi.ac.uk/metagenomics/api/v1/analyses/MGYA00150965/file/SRR2154916_FASTQ_SSU_OTU.tsv	Root-Host_associated-Human-Digestive_system-Oral-Subgingival_plaque
SRR1715601	MGYA00148877	https://www.ebi.ac.uk/metagenomics/api/v1/analyses/MGYA00148877/file/SRR1715601_FASTQ_SSU_OTU.tsv	Root-Host_associated-Human-Digestive_system-Oral-Subgingival_plaque
SRR2086778	MGYA00143838	https://www.ebi.ac.uk/metagenomics/api/v1/analyses/MGYA00143838/file/SRR2086778_MERGED_FASTQ_SSU_OTU.tsv	Root-Host_associated-Human-Digestive_system-Oral-Subgingival_plaque
SRR6239910	MGYA00168217	https://www.ebi.ac.uk/metagenomics/api/v1/analyses/MGYA00168217/file/SRR6239910_FASTQ_SSU_OTU.tsv	Root-Host_associated-Human-Digestive_system-Oral-Subgingival_plaque
SRR5178142	MGYA00148722	https://www.ebi.ac.uk/metagenomics/api/v1/analyses/MGYA00148722/file/SRR5178142_MERGED_FASTQ_SSU_OTU.tsv	Root-Host_associated-Human-Digestive_system-Oral-Subgingival_plaque
SRR1715648	MGYA00148962	https://www.ebi.ac.uk/metagenomics/api/v1/analyses/MGYA00148962/file/SRR1715648_FASTQ_SSU_OTU.tsv	Root-Host_associated-Human-Digestive_system-Oral-Subgingival_plaque
SRR3381564	MGYA00151174	https://www.ebi.ac.uk/metagenomics/api/v1/analyses/MGYA00151174/file/SRR3381564_FASTQ_SSU_OTU.tsv	Root-Host_associated-Human-Digestive_system-Oral-Subgingival_plaque

SRR2086945	MGYA00144010	https://www.ebi.ac.uk/metagenomics/api/v1/analyses/MGYA00144010/file/SRR2086945_MERGED_FASTQ_SSU_OTU.tsv	Root-Host_associated-Human-Digestive_system-Oral-Subgingival_plaque
SRR6239899	MGYA00168200	https://www.ebi.ac.uk/metagenomics/api/v1/analyses/MGYA00168200/file/SRR6239899_FASTQ_SSU_OTU.tsv	Root-Host_associated-Human-Digestive_system-Oral-Subgingival_plaque
SRR1178392	MGYA00151886	https://www.ebi.ac.uk/metagenomics/api/v1/analyses/MGYA00151886/file/SRR1178392_FASTQ_SSU_OTU.tsv	Root-Host_associated-Human-Digestive_system-Oral-Subgingival_plaque
SRR2086861	MGYA00143852	https://www.ebi.ac.uk/metagenomics/api/v1/analyses/MGYA00143852/file/SRR2086861_MERGED_FASTQ_SSU_OTU.tsv	Root-Host_associated-Human-Digestive_system-Oral-Subgingival_plaque
SRR1715634	MGYA00148926	https://www.ebi.ac.uk/metagenomics/api/v1/analyses/MGYA00148926/file/SRR1715634_FASTQ_SSU_OTU.tsv	Root-Host_associated-Human-Digestive_system-Oral-Subgingival_plaque
SRR2154923	MGYA00151073	https://www.ebi.ac.uk/metagenomics/api/v1/analyses/MGYA00151073/file/SRR2154923_FASTQ_SSU_OTU.tsv	Root-Host_associated-Human-Digestive_system-Oral-Subgingival_plaque
SRR2086939	MGYA00143957	https://www.ebi.ac.uk/metagenomics/api/v1/analyses/MGYA00143957/file/SRR2086939_MERGED_FASTQ_SSU_OTU.tsv	Root-Host_associated-Human-Digestive_system-Oral-Subgingival_plaque
SRR2155006	MGYA00150992	https://www.ebi.ac.uk/metagenomics/api/v1/analyses/MGYA00150992/file/SRR2155006_FASTQ_SSU_OTU.tsv	Root-Host_associated-Human-Digestive_system-Oral-Subgingival_plaque
SRR5178139	MGYA00148719	https://www.ebi.ac.uk/metagenomics/api/v1/analyses/MGYA00148719/file/SRR5178139_MERGED_FASTQ_SSU_OTU.tsv	Root-Host_associated-Human-Digestive_system-Oral-Subgingival_plaque
SRR2086866	MGYA00144000	https://www.ebi.ac.uk/metagenomics/api/v1/analyses/MGYA00144000/file/SRR2086866_MERGED_FASTQ_SSU_OTU.tsv	Root-Host_associated-Human-Digestive_system-Oral-Subgingival_plaque
SRR1745941	MGYA00168140	https://www.ebi.ac.uk/metagenomics/api/v1/analyses/MGYA00168140/file/SRR1745941_FASTQ_SSU_OTU.tsv	Root-Host_associated-Human-Digestive_system-Oral-Subgingival_plaque
SRR1715633	MGYA00148933	https://www.ebi.ac.uk/metagenomics/api/v1/analyses/MGYA00148933/file/SRR1715633_FASTQ_SSU_OTU.tsv	Root-Host_associated-Human-Digestive_system-Oral-Subgingival_plaque

SRR2154924	MGYA00151025	https://www.ebi.ac.uk/metagenomics/api/v1/analyses/MGYA00151025/file/SRR2154924_FASTQ_SSU_OTU.tsv	Root-Host_associated-Human-Digestive_system-Oral-Subgingival_plaque
SRR2155001	MGYA00151036	https://www.ebi.ac.uk/metagenomics/api/v1/analyses/MGYA00151036/file/SRR2155001_FASTQ_SSU_OTU.tsv	Root-Host_associated-Human-Digestive_system-Oral-Subgingival_plaque
SRR6239917	MGYA00168163	https://www.ebi.ac.uk/metagenomics/api/v1/analyses/MGYA00168163/file/SRR6239917_FASTQ_SSU_OTU.tsv	Root-Host_associated-Human-Digestive_system-Oral-Subgingival_plaque
SRR5178145	MGYA00148743	https://www.ebi.ac.uk/metagenomics/api/v1/analyses/MGYA00148743/file/SRR5178145_MERGED_FASTQ_SSU_OTU.tsv	Root-Host_associated-Human-Digestive_system-Oral-Subgingival_plaque
SRR1715589	MGYA00148959	https://www.ebi.ac.uk/metagenomics/api/v1/analyses/MGYA00148959/file/SRR1715589_FASTQ_SSU_OTU.tsv	Root-Host_associated-Human-Digestive_system-Oral-Subgingival_plaque
SRR2086942	MGYA00144037	https://www.ebi.ac.uk/metagenomics/api/v1/analyses/MGYA00144037/file/SRR2086942_MERGED_FASTQ_SSU_OTU.tsv	Root-Host_associated-Human-Digestive_system-Oral-Subgingival_plaque
SRR3381563	MGYA00151160	https://www.ebi.ac.uk/metagenomics/api/v1/analyses/MGYA00151160/file/SRR3381563_FASTQ_SSU_OTU.tsv	Root-Host_associated-Human-Digestive_system-Oral-Subgingival_plaque
SRR2154958	MGYA00150978	https://www.ebi.ac.uk/metagenomics/api/v1/analyses/MGYA00150978/file/SRR2154958_FASTQ_SSU_OTU.tsv	Root-Host_associated-Human-Digestive_system-Oral-Subgingival_plaque
SRR2154989	MGYA00151053	https://www.ebi.ac.uk/metagenomics/api/v1/analyses/MGYA00151053/file/SRR2154989_FASTQ_SSU_OTU.tsv	Root-Host_associated-Human-Digestive_system-Oral-Subgingival_plaque
SRR2086868	MGYA00144008	https://www.ebi.ac.uk/metagenomics/api/v1/analyses/MGYA00144008/file/SRR2086868_MERGED_FASTQ_SSU_OTU.tsv	Root-Host_associated-Human-Digestive_system-Oral-Subgingival_plaque
SRR5178137	MGYA00148779	https://www.ebi.ac.uk/metagenomics/api/v1/analyses/MGYA00148779/file/SRR5178137_MERGED_FASTQ_SSU_OTU.tsv	Root-Host_associated-Human-Digestive_system-Oral-Subgingival_plaque
SRR2086930	MGYA00143928	https://www.ebi.ac.uk/metagenomics/api/v1/analyses/MGYA00143928/file/SRR2086930_MERGED_FASTQ_SSU_OTU.tsv	Root-Host_associated-Human-Digestive_system-Oral-Subgingival_plaque

SRR2086771	MGYA00144001	https://www.ebi.ac.uk/metagenomics/api/v1/analyses/MGYA00144001/file/SRR2086771_MERGED_FASTQ_SSU_OTU.tsv	Root-Host_associated-Human-Digestive_system-Oral-Subgingival_plaque
SRR6239919	MGYA00168167	https://www.ebi.ac.uk/metagenomics/api/v1/analyses/MGYA00168167/file/SRR6239919_FASTQ_SSU_OTU.tsv	Root-Host_associated-Human-Digestive_system-Oral-Subgingival_plaque
SRR2086814	MGYA00144044	https://www.ebi.ac.uk/metagenomics/api/v1/analyses/MGYA00144044/file/SRR2086814_MERGED_FASTQ_SSU_OTU.tsv	Root-Host_associated-Human-Digestive_system-Oral-Subgingival_plaque
SRR6316004	MGYA00293720	https://www.ebi.ac.uk/metagenomics/api/v1/analyses/MGYA00293720/file/SRR6316004_MERGED_FASTQ_SSU_OTU.tsv	Root-Host_associated-Human-Digestive_system-Oral-Subgingival_plaque
SRR3992174	MGYA00151245	https://www.ebi.ac.uk/metagenomics/api/v1/analyses/MGYA00151245/file/SRR3992174_FASTQ_SSU_OTU.tsv	Root-Host_associated-Human-Digestive_system-Oral-Subgingival_plaque
SRR1745933	MGYA00168141	https://www.ebi.ac.uk/metagenomics/api/v1/analyses/MGYA00168141/file/SRR1745933_FASTQ_SSU_OTU.tsv	Root-Host_associated-Human-Digestive_system-Oral-Subgingival_plaque
SRR1715641	MGYA00148919	https://www.ebi.ac.uk/metagenomics/api/v1/analyses/MGYA00148919/file/SRR1715641_FASTQ_SSU_OTU.tsv	Root-Host_associated-Human-Digestive_system-Oral-Subgingival_plaque
SRR2154956	MGYA00150987	https://www.ebi.ac.uk/metagenomics/api/v1/analyses/MGYA00150987/file/SRR2154956_FASTQ_SSU_OTU.tsv	Root-Host_associated-Human-Digestive_system-Oral-Subgingival_plaque
DRR049482	MGYA00160376	https://www.ebi.ac.uk/metagenomics/api/v1/analyses/MGYA00160376/file/DRR049482_MERGED_FASTQ_SSU_OTU.tsv	Root-Host_associated-Human-Digestive_system-Oral-Subgingival_plaque
SRR1715587	MGYA00148909	https://www.ebi.ac.uk/metagenomics/api/v1/analyses/MGYA00148909/file/SRR1715587_FASTQ_SSU_OTU.tsv	Root-Host_associated-Human-Digestive_system-Oral-Subgingival_plaque
DRR049453	MGYA00160356	https://www.ebi.ac.uk/metagenomics/api/v1/analyses/MGYA00160356/file/DRR049453_MERGED_FASTQ_SSU_OTU.tsv	Root-Host_associated-Human-Digestive_system-Oral-Subgingival_plaque
SRR1178518	MGYA00151880	https://www.ebi.ac.uk/metagenomics/api/v1/analyses/MGYA00151880/file/SRR1178518_FASTQ_SSU_OTU.tsv	Root-Host_associated-Human-Digestive_system-Oral-Subgingival_plaque

SRR6239890	MGYA00168209	https://www.ebi.ac.uk/metagenomics/api/v1/analyses/MGYA00168209/file/SRR6239890_FASTQ_SSU_OTU.tsv	Root-Host_associated-Human-Digestive_system-Oral-Subgingival_plaque
SRR2154987	MGYA00151011	https://www.ebi.ac.uk/metagenomics/api/v1/analyses/MGYA00151011/file/SRR2154987_FASTQ_SSU_OTU.tsv	Root-Host_associated-Human-Digestive_system-Oral-Subgingival_plaque
SRR2086776	MGYA00143954	https://www.ebi.ac.uk/metagenomics/api/v1/analyses/MGYA00143954/file/SRR2086776_MERGED_FASTQ_SSU_OTU.tsv	Root-Host_associated-Human-Digestive_system-Oral-Subgingival_plaque
SRR2086813	MGYA00143937	https://www.ebi.ac.uk/metagenomics/api/v1/analyses/MGYA00143937/file/SRR2086813_MERGED_FASTQ_SSU_OTU.tsv	Root-Host_associated-Human-Digestive_system-Oral-Subgingival_plaque
SRR1745934	MGYA00168152	https://www.ebi.ac.uk/metagenomics/api/v1/analyses/MGYA00168152/file/SRR1745934_FASTQ_SSU_OTU.tsv	Root-Host_associated-Human-Digestive_system-Oral-Subgingival_plaque
SRR3992173	MGYA00151250	https://www.ebi.ac.uk/metagenomics/api/v1/analyses/MGYA00151250/file/SRR3992173_FASTQ_SSU_OTU.tsv	Root-Host_associated-Human-Digestive_system-Oral-Subgingival_plaque
SRR1715646	MGYA00148889	https://www.ebi.ac.uk/metagenomics/api/v1/analyses/MGYA00148889/file/SRR1715646_FASTQ_SSU_OTU.tsv	Root-Host_associated-Human-Digestive_system-Oral-Subgingival_plaque
SRR2154951	MGYA00150964	https://www.ebi.ac.uk/metagenomics/api/v1/analyses/MGYA00150964/file/SRR2154951_FASTQ_SSU_OTU.tsv	Root-Host_associated-Human-Digestive_system-Oral-Subgingival_plaque
DRR049485	MGYA00160351	https://www.ebi.ac.uk/metagenomics/api/v1/analyses/MGYA00160351/file/DRR049485_MERGED_FASTQ_SSU_OTU.tsv	Root-Host_associated-Human-Digestive_system-Oral-Subgingival_plaque
SRR1715580	MGYA00148935	https://www.ebi.ac.uk/metagenomics/api/v1/analyses/MGYA00148935/file/SRR1715580_FASTQ_SSU_OTU.tsv	Root-Host_associated-Human-Digestive_system-Oral-Subgingival_plaque
DRR049454	MGYA00160374	https://www.ebi.ac.uk/metagenomics/api/v1/analyses/MGYA00160374/file/DRR049454_MERGED_FASTQ_SSU_OTU.tsv	Root-Host_associated-Human-Digestive_system-Oral-Subgingival_plaque
SRR6239897	MGYA00168174	https://www.ebi.ac.uk/metagenomics/api/v1/analyses/MGYA00168174/file/SRR6239897_FASTQ_SSU_OTU.tsv	Root-Host_associated-Human-Digestive_system-Oral-Subgingival_plaque

SRR2154980	MGYA00151028	https://www.ebi.ac.uk/metagenomics/api/v1/analyses/MGYA00151028/file/SRR2154980_FASTQ_SSU_OTU.tsv	Root-Host_associated-Human-Digestive_system-Oral-Subgingival_plaque
SRR5178130	MGYA00148720	https://www.ebi.ac.uk/metagenomics/api/v1/analyses/MGYA00148720/file/SRR5178130_MERGED_FASTQ_SSU_OTU.tsv	Root-Host_associated-Human-Digestive_system-Oral-Subgingival_plaque
SRR2086937	MGYA00143976	https://www.ebi.ac.uk/metagenomics/api/v1/analyses/MGYA00143976/file/SRR2086937_MERGED_FASTQ_SSU_OTU.tsv	Root-Host_associated-Human-Digestive_system-Oral-Subgingival_plaque
SRR2155008	MGYA00151065	https://www.ebi.ac.uk/metagenomics/api/v1/analyses/MGYA00151065/file/SRR2155008_FASTQ_SSU_OTU.tsv	Root-Host_associated-Human-Digestive_system-Oral-Subgingival_plaque
SRR6746548	MGYA00202646	https://www.ebi.ac.uk/metagenomics/api/v1/analyses/MGYA00202646/file/SRR6746548_MERGED_FASTQ_SSU_OTU.tsv	Root-Environmental-Aquatic-Marine-Coastal-Sediment
SRR5097531	MGYA00360543	https://www.ebi.ac.uk/metagenomics/api/v1/analyses/MGYA00360543/file/SRR5097531_MERGED_FASTQ_SSU_OTU.tsv	Root-Environmental-Aquatic-Marine-Coastal-Sediment
SRR6746129	MGYA00202563	https://www.ebi.ac.uk/metagenomics/api/v1/analyses/MGYA00202563/file/SRR6746129_MERGED_FASTQ_SSU_OTU.tsv	Root-Environmental-Aquatic-Marine-Coastal-Sediment
SRR6745977	MGYA00202901	https://www.ebi.ac.uk/metagenomics/api/v1/analyses/MGYA00202901/file/SRR6745977_MERGED_FASTQ_SSU_OTU.tsv	Root-Environmental-Aquatic-Marine-Coastal-Sediment
SRR6746071	MGYA00202531	https://www.ebi.ac.uk/metagenomics/api/v1/analyses/MGYA00202531/file/SRR6746071_MERGED_FASTQ_SSU_OTU.tsv	Root-Environmental-Aquatic-Marine-Coastal-Sediment
SRR6745386	MGYA00202792	https://www.ebi.ac.uk/metagenomics/api/v1/analyses/MGYA00202792/file/SRR6745386_MERGED_FASTQ_SSU_OTU.tsv	Root-Environmental-Aquatic-Marine-Coastal-Sediment
SRR6745853	MGYA00202949	https://www.ebi.ac.uk/metagenomics/api/v1/analyses/MGYA00202949/file/SRR6745853_MERGED_FASTQ_SSU_OTU.tsv	Root-Environmental-Aquatic-Marine-Coastal-Sediment
SRR6745421	MGYA00202641	https://www.ebi.ac.uk/metagenomics/api/v1/analyses/MGYA00202641/file/SRR6745421_MERGED_FASTQ_SSU_OTU.tsv	Root-Environmental-Aquatic-Marine-Coastal-Sediment

SRR6746534	MGYA00202521	https://www.ebi.ac.uk/metagenomics/api/v1/analyses/MGYA00202521/file/SRR6746534_MERGED_FASTQ_SSU_OTU.tsv	Root-Environmental-Aquatic-Marine-Coastal-Sediment
SRR2297161	MGYA00358925	https://www.ebi.ac.uk/metagenomics/api/v1/analyses/MGYA00358925/file/SRR2297161_MERGED_FASTQ_SSU_OTU.tsv	Root-Environmental-Aquatic-Marine-Coastal-Sediment
SRR6746155	MGYA00202794	https://www.ebi.ac.uk/metagenomics/api/v1/analyses/MGYA00202794/file/SRR6746155_MERGED_FASTQ_SSU_OTU.tsv	Root-Environmental-Aquatic-Marine-Coastal-Sediment
SRR6746184	MGYA00202691	https://www.ebi.ac.uk/metagenomics/api/v1/analyses/MGYA00202691/file/SRR6746184_MERGED_FASTQ_SSU_OTU.tsv	Root-Environmental-Aquatic-Marine-Coastal-Sediment
SRR6745357	MGYA00202898	https://www.ebi.ac.uk/metagenomics/api/v1/analyses/MGYA00202898/file/SRR6745357_MERGED_FASTQ_SSU_OTU.tsv	Root-Environmental-Aquatic-Marine-Coastal-Sediment
SRR1503983	MGYA00358538	https://www.ebi.ac.uk/metagenomics/api/v1/analyses/MGYA00358538/file/SRR1503983_FASTQ_SSU_OTU.tsv	Root-Environmental-Aquatic-Marine-Coastal-Sediment
SRR6745882	MGYA00202533	https://www.ebi.ac.uk/metagenomics/api/v1/analyses/MGYA00202533/file/SRR6745882_MERGED_FASTQ_SSU_OTU.tsv	Root-Environmental-Aquatic-Marine-Coastal-Sediment
SRR2856424	MGYA00359221	https://www.ebi.ac.uk/metagenomics/api/v1/analyses/MGYA00359221/file/SRR2856424_MERGED_FASTQ_SSU_OTU.tsv	Root-Environmental-Aquatic-Marine-Coastal-Sediment
SRR6745426	MGYA00203045	https://www.ebi.ac.uk/metagenomics/api/v1/analyses/MGYA00203045/file/SRR6745426_MERGED_FASTQ_SSU_OTU.tsv	Root-Environmental-Aquatic-Marine-Coastal-Sediment
SRR6745854	MGYA00202706	https://www.ebi.ac.uk/metagenomics/api/v1/analyses/MGYA00202706/file/SRR6745854_MERGED_FASTQ_SSU_OTU.tsv	Root-Environmental-Aquatic-Marine-Coastal-Sediment
SRR6745381	MGYA00202580	https://www.ebi.ac.uk/metagenomics/api/v1/analyses/MGYA00202580/file/SRR6745381_MERGED_FASTQ_SSU_OTU.tsv	Root-Environmental-Aquatic-Marine-Coastal-Sediment
SRR6746533	MGYA00202937	https://www.ebi.ac.uk/metagenomics/api/v1/analyses/MGYA00202937/file/SRR6746533_MERGED_FASTQ_SSU_OTU.tsv	Root-Environmental-Aquatic-Marine-Coastal-Sediment

SRR6746152	MGYA00202789	https://www.ebi.ac.uk/metagenomics/api/v1/analyses/MGYA00202789/file/SRR6746152_MERGED_FASTQ_SSU_OTU.tsv	Root-Environmental-Aquatic-Marine-Coastal-Sediment
SRR2297166	MGYA00358930	https://www.ebi.ac.uk/metagenomics/api/v1/analyses/MGYA00358930/file/SRR2297166_MERGED_FASTQ_SSU_OTU.tsv	Root-Environmental-Aquatic-Marine-Coastal-Sediment
SRR4300278	MGYA00360239	https://www.ebi.ac.uk/metagenomics/api/v1/analyses/MGYA00360239/file/SRR4300278_MERGED_FASTQ_SSU_OTU.tsv	Root-Environmental-Aquatic-Marine-Coastal-Sediment
SRR6746183	MGYA00202614	https://www.ebi.ac.uk/metagenomics/api/v1/analyses/MGYA00202614/file/SRR6746183_MERGED_FASTQ_SSU_OTU.tsv	Root-Environmental-Aquatic-Marine-Coastal-Sediment
SRR1503984	MGYA00358539	https://www.ebi.ac.uk/metagenomics/api/v1/analyses/MGYA00358539/file/SRR1503984_FASTQ_SSU_OTU.tsv	Root-Environmental-Aquatic-Marine-Coastal-Sediment
SRR6745885	MGYA00202822	https://www.ebi.ac.uk/metagenomics/api/v1/analyses/MGYA00202822/file/SRR6745885_MERGED_FASTQ_SSU_OTU.tsv	Root-Environmental-Aquatic-Marine-Coastal-Sediment
SRR6745350	MGYA00202497	https://www.ebi.ac.uk/metagenomics/api/v1/analyses/MGYA00202497/file/SRR6745350_MERGED_FASTQ_SSU_OTU.tsv	Root-Environmental-Aquatic-Marine-Coastal-Sediment
SRR2856423	MGYA00359220	https://www.ebi.ac.uk/metagenomics/api/v1/analyses/MGYA00359220/file/SRR2856423_MERGED_FASTQ_SSU_OTU.tsv	Root-Environmental-Aquatic-Marine-Coastal-Sediment
SRR5097536	MGYA00360548	https://www.ebi.ac.uk/metagenomics/api/v1/analyses/MGYA00360548/file/SRR5097536_MERGED_FASTQ_SSU_OTU.tsv	Root-Environmental-Aquatic-Marine-Coastal-Sediment
SRR6745828	MGYA00202606	https://www.ebi.ac.uk/metagenomics/api/v1/analyses/MGYA00202606/file/SRR6745828_MERGED_FASTQ_SSU_OTU.tsv	Root-Environmental-Aquatic-Marine-Coastal-Sediment
SRR5227106	MGYA00360665	https://www.ebi.ac.uk/metagenomics/api/v1/analyses/MGYA00360665/file/SRR5227106_FASTQ_SSU_OTU.tsv	Root-Environmental-Aquatic-Marine-Coastal-Sediment
SRR6745970	MGYA00202797	https://www.ebi.ac.uk/metagenomics/api/v1/analyses/MGYA00202797/file/SRR6745970_MERGED_FASTQ_SSU_OTU.tsv	Root-Environmental-Aquatic-Marine-Coastal-Sediment

SRR6746076	MGYA00202769	https://www.ebi.ac.uk/metagenomics/api/v1/analyses/MGYA00202769/file/SRR6746076_MERGED_FASTQ_SSU_OTU.tsv	Root-Environmental-Aquatic-Marine-Coastal-Sediment
SRR6745428	MGYA00203050	https://www.ebi.ac.uk/metagenomics/api/v1/analyses/MGYA00203050/file/SRR6745428_MERGED_FASTQ_SSU_OTU.tsv	Root-Environmental-Aquatic-Marine-Coastal-Sediment
SRR4300276	MGYA00360237	https://www.ebi.ac.uk/metagenomics/api/v1/analyses/MGYA00360237/file/SRR4300276_MERGED_FASTQ_SSU_OTU.tsv	Root-Environmental-Aquatic-Marine-Coastal-Sediment
SRR2297168	MGYA00358932	https://www.ebi.ac.uk/metagenomics/api/v1/analyses/MGYA00358932/file/SRR2297168_MERGED_FASTQ_SSU_OTU.tsv	Root-Environmental-Aquatic-Marine-Coastal-Sediment
SRR6746004	MGYA00203087	https://www.ebi.ac.uk/metagenomics/api/v1/analyses/MGYA00203087/file/SRR6746004_MERGED_FASTQ_SSU_OTU.tsv	Root-Environmental-Aquatic-Marine-Coastal-Sediment
SRR6745826	MGYA00202727	https://www.ebi.ac.uk/metagenomics/api/v1/analyses/MGYA00202727/file/SRR6745826_MERGED_FASTQ_SSU_OTU.tsv	Root-Environmental-Aquatic-Marine-Coastal-Sediment
SRR5097538	MGYA00360550	https://www.ebi.ac.uk/metagenomics/api/v1/analyses/MGYA00360550/file/SRR5097538_MERGED_FASTQ_SSU_OTU.tsv	Root-Environmental-Aquatic-Marine-Coastal-Sediment
SRR6746541	MGYA00202859	https://www.ebi.ac.uk/metagenomics/api/v1/analyses/MGYA00202859/file/SRR6746541_MERGED_FASTQ_SSU_OTU.tsv	Root-Environmental-Aquatic-Marine-Coastal-Sediment
SRR6745792	MGYA00203068	https://www.ebi.ac.uk/metagenomics/api/v1/analyses/MGYA00203068/file/SRR6745792_MERGED_FASTQ_SSU_OTU.tsv	Root-Environmental-Aquatic-Marine-Coastal-Sediment
SRR2297114	MGYA00358878	https://www.ebi.ac.uk/metagenomics/api/v1/analyses/MGYA00358878/file/SRR2297114_MERGED_FASTQ_SSU_OTU.tsv	Root-Environmental-Aquatic-Marine-Coastal-Sediment
SRR6746120	MGYA00202910	https://www.ebi.ac.uk/metagenomics/api/v1/analyses/MGYA00202910/file/SRR6746120_MERGED_FASTQ_SSU_OTU.tsv	Root-Environmental-Aquatic-Marine-Coastal-Sediment
SRR5227108	MGYA00360667	https://www.ebi.ac.uk/metagenomics/api/v1/analyses/MGYA00360667/file/SRR5227108_FASTQ_SSU_OTU.tsv	Root-Environmental-Aquatic-Marine-Coastal-Sediment

SRR2856451	MGYA00359227	https://www.ebi.ac.uk/metagenomics/api/v1/analyses/MGYA00359227/file/SRR2856451_MERGED_FASTQ_SSU_OTU.tsv	Root-Environmental-Aquatic-Marine-Coastal-Sediment
SRR6746078	MGYA00202990	https://www.ebi.ac.uk/metagenomics/api/v1/analyses/MGYA00202990/file/SRR6746078_MERGED_FASTQ_SSU_OTU.tsv	Root-Environmental-Aquatic-Marine-Coastal-Sediment
SRR6745821	MGYA00202585	https://www.ebi.ac.uk/metagenomics/api/v1/analyses/MGYA00202585/file/SRR6745821_MERGED_FASTQ_SSU_OTU.tsv	Root-Environmental-Aquatic-Marine-Coastal-Sediment
SRR6746546	MGYA00202842	https://www.ebi.ac.uk/metagenomics/api/v1/analyses/MGYA00202842/file/SRR6746546_MERGED_FASTQ_SSU_OTU.tsv	Root-Environmental-Aquatic-Marine-Coastal-Sediment
SRR6745795	MGYA00202535	https://www.ebi.ac.uk/metagenomics/api/v1/analyses/MGYA00202535/file/SRR6745795_MERGED_FASTQ_SSU_OTU.tsv	Root-Environmental-Aquatic-Marine-Coastal-Sediment
SRR6746127	MGYA00202682	https://www.ebi.ac.uk/metagenomics/api/v1/analyses/MGYA00202682/file/SRR6746127_MERGED_FASTQ_SSU_OTU.tsv	Root-Environmental-Aquatic-Marine-Coastal-Sediment
SRR2297113	MGYA00358877	https://www.ebi.ac.uk/metagenomics/api/v1/analyses/MGYA00358877/file/SRR2297113_MERGED_FASTQ_SSU_OTU.tsv	Root-Environmental-Aquatic-Marine-Coastal-Sediment
SRR2856456	MGYA00359231	https://www.ebi.ac.uk/metagenomics/api/v1/analyses/MGYA00359231/file/SRR2856456_MERGED_FASTQ_SSU_OTU.tsv	Root-Environmental-Aquatic-Marine-Coastal-Sediment
SRR6745979	MGYA00203079	https://www.ebi.ac.uk/metagenomics/api/v1/analyses/MGYA00203079/file/SRR6745979_MERGED_FASTQ_SSU_OTU.tsv	Root-Environmental-Aquatic-Marine-Coastal-Sediment
SRR6745388	MGYA00202790	https://www.ebi.ac.uk/metagenomics/api/v1/analyses/MGYA00202790/file/SRR6745388_MERGED_FASTQ_SSU_OTU.tsv	Root-Environmental-Aquatic-Marine-Coastal-Sediment
SRR650765	MGYA00356175	https://www.ebi.ac.uk/metagenomics/api/v1/analyses/MGYA00356175/file/SRR650765_FASTQ_SSU_OTU.tsv	Root-Environmental-Aquatic-Marine-Coastal-Sediment
SRR4300271	MGYA00360233	https://www.ebi.ac.uk/metagenomics/api/v1/analyses/MGYA00360233/file/SRR4300271_MERGED_FASTQ_SSU_OTU.tsv	Root-Environmental-Aquatic-Marine-Coastal-Sediment

SRR6745359	MGYA00202622	https://www.ebi.ac.uk/metagenomics/api/v1/analyses/MGYA00202622/file/SRR6745359_MERGED_FASTQ_SSU_OTU.tsv	Root-Environmental-Aquatic-Marine-Coastal-Sediment
SRR6745905	MGYA00203037	https://www.ebi.ac.uk/metagenomics/api/v1/analyses/MGYA00203037/file/SRR6745905_MERGED_FASTQ_SSU_OTU.tsv	Root-Environmental-Aquatic-Marine-Coastal-Sediment
SRR6746003	MGYA00203095	https://www.ebi.ac.uk/metagenomics/api/v1/analyses/MGYA00203095/file/SRR6746003_MERGED_FASTQ_SSU_OTU.tsv	Root-Environmental-Aquatic-Marine-Coastal-Sediment
SRR6746205	MGYA00202932	https://www.ebi.ac.uk/metagenomics/api/v1/analyses/MGYA00202932/file/SRR6746205_MERGED_FASTQ_SSU_OTU.tsv	Root-Environmental-Aquatic-Marine-Coastal-Sediment
ERR330032	MGYA00357990	https://www.ebi.ac.uk/metagenomics/api/v1/analyses/MGYA00357990/file/ERR330032_FASTQ_SSU_OTU.tsv	Root-Environmental-Aquatic-Marine-Coastal-Sediment
SRR6172341	MGYA00362303	https://www.ebi.ac.uk/metagenomics/api/v1/analyses/MGYA00362303/file/SRR6172341_MERGED_FASTQ_SSU_OTU.tsv	Root-Environmental-Aquatic-Marine-Coastal-Sediment
SRR2297126	MGYA00358890	https://www.ebi.ac.uk/metagenomics/api/v1/analyses/MGYA00358890/file/SRR2297126_MERGED_FASTQ_SSU_OTU.tsv	Root-Environmental-Aquatic-Marine-Coastal-Sediment
SRR6746112	MGYA00202554	https://www.ebi.ac.uk/metagenomics/api/v1/analyses/MGYA00202554/file/SRR6746112_MERGED_FASTQ_SSU_OTU.tsv	Root-Environmental-Aquatic-Marine-Coastal-Sediment
SRR6745814	MGYA00202965	https://www.ebi.ac.uk/metagenomics/api/v1/analyses/MGYA00202965/file/SRR6745814_MERGED_FASTQ_SSU_OTU.tsv	Root-Environmental-Aquatic-Marine-Coastal-Sediment
SRR4300295	MGYA00360254	https://www.ebi.ac.uk/metagenomics/api/v1/analyses/MGYA00360254/file/SRR4300295_MERGED_FASTQ_SSU_OTU.tsv	Root-Environmental-Aquatic-Marine-Coastal-Sediment
SRR6745868	MGYA00202574	https://www.ebi.ac.uk/metagenomics/api/v1/analyses/MGYA00202574/file/SRR6745868_MERGED_FASTQ_SSU_OTU.tsv	Root-Environmental-Aquatic-Marine-Coastal-Sediment
SRR6746036	MGYA00202619	https://www.ebi.ac.uk/metagenomics/api/v1/analyses/MGYA00202619/file/SRR6746036_MERGED_FASTQ_SSU_OTU.tsv	Root-Environmental-Aquatic-Marine-Coastal-Sediment

SRR6745930	MGYA00202722	https://www.ebi.ac.uk/metagenomics/api/v1/analyses/MGYA00202722/file/SRR6745930_MERGED_FASTQ_SSU_OTU.tsv	Root-Environmental-Aquatic-Marine-Coastal-Sediment
SRR6746486	MGYA00202501	https://www.ebi.ac.uk/metagenomics/api/v1/analyses/MGYA00202501/file/SRR6746486_MERGED_FASTQ_SSU_OTU.tsv	Root-Environmental-Aquatic-Marine-Coastal-Sediment
SRR6517606	MGYA00362471	https://www.ebi.ac.uk/metagenomics/api/v1/analyses/MGYA00362471/file/SRR6517606_MERGED_FASTQ_SSU_OTU.tsv	Root-Environmental-Aquatic-Marine-Coastal-Sediment
ERR330049	MGYA00358007	https://www.ebi.ac.uk/metagenomics/api/v1/analyses/MGYA00358007/file/ERR330049_FASTQ_SSU_OTU.tsv	Root-Environmental-Aquatic-Marine-Coastal-Sediment
SRR650832	MGYA00356181	https://www.ebi.ac.uk/metagenomics/api/v1/analyses/MGYA00356181/file/SRR650832_FASTQ_SSU_OTU.tsv	Root-Environmental-Aquatic-Marine-Coastal-Sediment
SRR4300292	MGYA00360252	https://www.ebi.ac.uk/metagenomics/api/v1/analyses/MGYA00360252/file/SRR4300292_MERGED_FASTQ_SSU_OTU.tsv	Root-Environmental-Aquatic-Marine-Coastal-Sediment
SRR6746169	MGYA00202633	https://www.ebi.ac.uk/metagenomics/api/v1/analyses/MGYA00202633/file/SRR6746169_MERGED_FASTQ_SSU_OTU.tsv	Root-Environmental-Aquatic-Marine-Coastal-Sediment
SRR6746508	MGYA00202741	https://www.ebi.ac.uk/metagenomics/api/v1/analyses/MGYA00202741/file/SRR6746508_MERGED_FASTQ_SSU_OTU.tsv	Root-Environmental-Aquatic-Marine-Coastal-Sediment
SRR6746031	MGYA00202752	https://www.ebi.ac.uk/metagenomics/api/v1/analyses/MGYA00202752/file/SRR6746031_MERGED_FASTQ_SSU_OTU.tsv	Root-Environmental-Aquatic-Marine-Coastal-Sediment
SRR6745937	MGYA00202749	https://www.ebi.ac.uk/metagenomics/api/v1/analyses/MGYA00202749/file/SRR6745937_MERGED_FASTQ_SSU_OTU.tsv	Root-Environmental-Aquatic-Marine-Coastal-Sediment
SRR6746481	MGYA00202760	https://www.ebi.ac.uk/metagenomics/api/v1/analyses/MGYA00202760/file/SRR6746481_MERGED_FASTQ_SSU_OTU.tsv	Root-Environmental-Aquatic-Marine-Coastal-Sediment
SRR6746202	MGYA00202559	https://www.ebi.ac.uk/metagenomics/api/v1/analyses/MGYA00202559/file/SRR6746202_MERGED_FASTQ_SSU_OTU.tsv	Root-Environmental-Aquatic-Marine-Coastal-Sediment

ERR330035	MGYA00357993	https://www.ebi.ac.uk/metagenomics/api/v1/analyses/MGYA00357993/file/ERR330035_FASTQ_SSU_OTU.tsv	Root-Environmental-Aquatic-Marine-Coastal-Sediment
SRR6172346	MGYA00362308	https://www.ebi.ac.uk/metagenomics/api/v1/analyses/MGYA00362308/file/SRR6172346_MERGED_FASTQ_SSU_OTU.tsv	Root-Environmental-Aquatic-Marine-Coastal-Sediment
SRR6746115	MGYA00202658	https://www.ebi.ac.uk/metagenomics/api/v1/analyses/MGYA00202658/file/SRR6746115_MERGED_FASTQ_SSU_OTU.tsv	Root-Environmental-Aquatic-Marine-Coastal-Sediment
SRR2297121	MGYA00358885	https://www.ebi.ac.uk/metagenomics/api/v1/analyses/MGYA00358885/file/SRR2297121_MERGED_FASTQ_SSU_OTU.tsv	Root-Environmental-Aquatic-Marine-Coastal-Sediment
SRR6745813	MGYA00202550	https://www.ebi.ac.uk/metagenomics/api/v1/analyses/MGYA00202550/file/SRR6745813_MERGED_FASTQ_SSU_OTU.tsv	Root-Environmental-Aquatic-Marine-Coastal-Sediment
SRR2855903	MGYA00359219	https://www.ebi.ac.uk/metagenomics/api/v1/analyses/MGYA00359219/file/SRR2855903_MERGED_FASTQ_SSU_OTU.tsv	Root-Environmental-Aquatic-Marine-Coastal-Sediment
ERR330047	MGYA00358005	https://www.ebi.ac.uk/metagenomics/api/v1/analyses/MGYA00358005/file/ERR330047_FASTQ_SSU_OTU.tsv	Root-Environmental-Aquatic-Marine-Coastal-Sediment
SRR6745365	MGYA00203026	https://www.ebi.ac.uk/metagenomics/api/v1/analyses/MGYA00203026/file/SRR6745365_MERGED_FASTQ_SSU_OTU.tsv	Root-Environmental-Aquatic-Marine-Coastal-Sediment
SRR6172334	MGYA00362296	https://www.ebi.ac.uk/metagenomics/api/v1/analyses/MGYA00362296/file/SRR6172334_MERGED_FASTQ_SSU_OTU.tsv	Root-Environmental-Aquatic-Marine-Coastal-Sediment
SRR2297182	MGYA00358946	https://www.ebi.ac.uk/metagenomics/api/v1/analyses/MGYA00358946/file/SRR2297182_MERGED_FASTQ_SSU_OTU.tsv	Root-Environmental-Aquatic-Marine-Coastal-Sediment
SRR2297153	MGYA00358917	https://www.ebi.ac.uk/metagenomics/api/v1/analyses/MGYA00358917/file/SRR2297153_MERGED_FASTQ_SSU_OTU.tsv	Root-Environmental-Aquatic-Marine-Coastal-Sediment
SRR6746167	MGYA00202608	https://www.ebi.ac.uk/metagenomics/api/v1/analyses/MGYA00202608/file/SRR6746167_MERGED_FASTQ_SSU_OTU.tsv	Root-Environmental-Aquatic-Marine-Coastal-Sediment

SRR6745413	MGYA00203039	https://www.ebi.ac.uk/metagenomics/api/v1/analyses/MGYA00203039/file/SRR6745413_MERGED_FASTQ_SSU_OTU.tsv	Root-Environmental-Aquatic-Marine-Coastal-Sediment
SRR6745861	MGYA00202813	https://www.ebi.ac.uk/metagenomics/api/v1/analyses/MGYA00202813/file/SRR6745861_MERGED_FASTQ_SSU_OTU.tsv	Root-Environmental-Aquatic-Marine-Coastal-Sediment
SRR6746506	MGYA00202978	https://www.ebi.ac.uk/metagenomics/api/v1/analyses/MGYA00202978/file/SRR6746506_MERGED_FASTQ_SSU_OTU.tsv	Root-Environmental-Aquatic-Marine-Coastal-Sediment
SRR6745939	MGYA00202721	https://www.ebi.ac.uk/metagenomics/api/v1/analyses/MGYA00202721/file/SRR6745939_MERGED_FASTQ_SSU_OTU.tsv	Root-Environmental-Aquatic-Marine-Coastal-Sediment
SRR6172348	MGYA00362310	https://www.ebi.ac.uk/metagenomics/api/v1/analyses/MGYA00362310/file/SRR6172348_MERGED_FASTQ_SSU_OTU.tsv	Root-Environmental-Aquatic-Marine-Coastal-Sediment
SRR4436303	MGYA00213053	https://www.ebi.ac.uk/metagenomics/api/v1/analyses/MGYA00213053/file/SRR4436303_FASTQ_SSU_OTU.tsv	Root-Environmental-Aquatic-Marine-Coastal-Sediment
SRR6746043	MGYA00202700	https://www.ebi.ac.uk/metagenomics/api/v1/analyses/MGYA00202700/file/SRR6746043_MERGED_FASTQ_SSU_OTU.tsv	Root-Environmental-Aquatic-Marine-Coastal-Sediment
SRR6745945	MGYA00202944	https://www.ebi.ac.uk/metagenomics/api/v1/analyses/MGYA00202944/file/SRR6745945_MERGED_FASTQ_SSU_OTU.tsv	Root-Environmental-Aquatic-Marine-Coastal-Sediment
SRR6745994	MGYA00202509	https://www.ebi.ac.uk/metagenomics/api/v1/analyses/MGYA00202509/file/SRR6745994_MERGED_FASTQ_SSU_OTU.tsv	Root-Environmental-Aquatic-Marine-Coastal-Sediment
SRR6746092	MGYA00203055	https://www.ebi.ac.uk/metagenomics/api/v1/analyses/MGYA00203055/file/SRR6746092_MERGED_FASTQ_SSU_OTU.tsv	Root-Environmental-Aquatic-Marine-Coastal-Sediment
SRR2297128	MGYA00358892	https://www.ebi.ac.uk/metagenomics/api/v1/analyses/MGYA00358892/file/SRR2297128_MERGED_FASTQ_SSU_OTU.tsv	Root-Environmental-Aquatic-Marine-Coastal-Sediment
SRR4436304	MGYA00213042	https://www.ebi.ac.uk/metagenomics/api/v1/analyses/MGYA00213042/file/SRR4436304_FASTQ_SSU_OTU.tsv	Root-Environmental-Aquatic-Marine-Coastal-Sediment

SRR6746044	MGYA00203023	https://www.ebi.ac.uk/metagenomics/api/v1/analyses/MGYA00203023/file/SRR6746044_MERGED_FASTQ_SSU_OTU.tsv	Root-Environmental-Aquatic-Marine-Coastal-Sediment
SRR6745942	MGYA00202933	https://www.ebi.ac.uk/metagenomics/api/v1/analyses/MGYA00202933/file/SRR6745942_MERGED_FASTQ_SSU_OTU.tsv	Root-Environmental-Aquatic-Marine-Coastal-Sediment
SRR6745993	MGYA00202714	https://www.ebi.ac.uk/metagenomics/api/v1/analyses/MGYA00202714/file/SRR6745993_MERGED_FASTQ_SSU_OTU.tsv	Root-Environmental-Aquatic-Marine-Coastal-Sediment
SRR6746095	MGYA00202508	https://www.ebi.ac.uk/metagenomics/api/v1/analyses/MGYA00202508/file/SRR6746095_MERGED_FASTQ_SSU_OTU.tsv	Root-Environmental-Aquatic-Marine-Coastal-Sediment
ERR330040	MGYA00357998	https://www.ebi.ac.uk/metagenomics/api/v1/analyses/MGYA00357998/file/ERR330040_FASTQ_SSU_OTU.tsv	Root-Environmental-Aquatic-Marine-Coastal-Sediment
SRR6172333	MGYA00362295	https://www.ebi.ac.uk/metagenomics/api/v1/analyses/MGYA00362295/file/SRR6172333_MERGED_FASTQ_SSU_OTU.tsv	Root-Environmental-Aquatic-Marine-Coastal-Sediment
SRR6745362	MGYA00202783	https://www.ebi.ac.uk/metagenomics/api/v1/analyses/MGYA00202783/file/SRR6745362_MERGED_FASTQ_SSU_OTU.tsv	Root-Environmental-Aquatic-Marine-Coastal-Sediment
SRR2297185	MGYA00358949	https://www.ebi.ac.uk/metagenomics/api/v1/analyses/MGYA00358949/file/SRR2297185_MERGED_FASTQ_SSU_OTU.tsv	Root-Environmental-Aquatic-Marine-Coastal-Sediment
SRR6746160	MGYA00203064	https://www.ebi.ac.uk/metagenomics/api/v1/analyses/MGYA00203064/file/SRR6746160_MERGED_FASTQ_SSU_OTU.tsv	Root-Environmental-Aquatic-Marine-Coastal-Sediment
SRR2297154	MGYA00358918	https://www.ebi.ac.uk/metagenomics/api/v1/analyses/MGYA00358918/file/SRR2297154_MERGED_FASTQ_SSU_OTU.tsv	Root-Environmental-Aquatic-Marine-Coastal-Sediment
SRR6745866	MGYA00203001	https://www.ebi.ac.uk/metagenomics/api/v1/analyses/MGYA00203001/file/SRR6745866_MERGED_FASTQ_SSU_OTU.tsv	Root-Environmental-Aquatic-Marine-Coastal-Sediment
SRR6745414	MGYA00202773	https://www.ebi.ac.uk/metagenomics/api/v1/analyses/MGYA00202773/file/SRR6745414_MERGED_FASTQ_SSU_OTU.tsv	Root-Environmental-Aquatic-Marine-Coastal-Sediment

SRR6746501	MGYA00202709	https://www.ebi.ac.uk/metagenomics/api/v1/analyses/MGYA00202709/file/SRR6746501_MERGED_FASTQ_SSU_OTU.tsv	Root-Environmental-Aquatic-Marine-Coastal-Sediment
SRR6746038	MGYA00203034	https://www.ebi.ac.uk/metagenomics/api/v1/analyses/MGYA00203034/file/SRR6746038_MERGED_FASTQ_SSU_OTU.tsv	Root-Environmental-Aquatic-Marine-Coastal-Sediment
SRR6517608	MGYA00362473	https://www.ebi.ac.uk/metagenomics/api/v1/analyses/MGYA00362473/file/SRR6517608_MERGED_FASTQ_SSU_OTU.tsv	Root-Environmental-Aquatic-Marine-Coastal-Sediment
SRR6746488	MGYA00202686	https://www.ebi.ac.uk/metagenomics/api/v1/analyses/MGYA00202686/file/SRR6746488_MERGED_FASTQ_SSU_OTU.tsv	Root-Environmental-Aquatic-Marine-Coastal-Sediment
SRR6745982	MGYA00202643	https://www.ebi.ac.uk/metagenomics/api/v1/analyses/MGYA00202643/file/SRR6745982_MERGED_FASTQ_SSU_OTU.tsv	Root-Environmental-Aquatic-Marine-Coastal-Sediment
SRR6746084	MGYA00203032	https://www.ebi.ac.uk/metagenomics/api/v1/analyses/MGYA00203032/file/SRR6746084_MERGED_FASTQ_SSU_OTU.tsv	Root-Environmental-Aquatic-Marine-Coastal-Sediment
SRR6746055	MGYA00202927	https://www.ebi.ac.uk/metagenomics/api/v1/analyses/MGYA00202927/file/SRR6746055_MERGED_FASTQ_SSU_OTU.tsv	Root-Environmental-Aquatic-Marine-Coastal-Sediment
SRR6745953	MGYA00203018	https://www.ebi.ac.uk/metagenomics/api/v1/analyses/MGYA00203018/file/SRR6745953_MERGED_FASTQ_SSU_OTU.tsv	Root-Environmental-Aquatic-Marine-Coastal-Sediment
SRR2297139	MGYA00358903	https://www.ebi.ac.uk/metagenomics/api/v1/analyses/MGYA00358903/file/SRR2297139_MERGED_FASTQ_SSU_OTU.tsv	Root-Environmental-Aquatic-Marine-Coastal-Sediment
SRR6746499	MGYA00202570	https://www.ebi.ac.uk/metagenomics/api/v1/analyses/MGYA00202570/file/SRR6746499_MERGED_FASTQ_SSU_OTU.tsv	Root-Environmental-Aquatic-Marine-Coastal-Sediment
SRR1258344	MGYA00358467	https://www.ebi.ac.uk/metagenomics/api/v1/analyses/MGYA00358467/file/SRR1258344_FASTQ_SSU_OTU.tsv	Root-Environmental-Aquatic-Marine-Coastal-Sediment
SRR4300303	MGYA00360262	https://www.ebi.ac.uk/metagenomics/api/v1/analyses/MGYA00360262/file/SRR4300303_MERGED_FASTQ_SSU_OTU.tsv	Root-Environmental-Aquatic-Marine-Coastal-Sediment

SRR6746029	MGYA00202729	https://www.ebi.ac.uk/metagenomics/api/v1/analyses/MGYA00202729/file/SRR6746029_MERGED_FASTQ_SSU_OTU.tsv	Root-Environmental-Aquatic-Marine-Coastal-Sediment
SRR2297145	MGYA00358909	https://www.ebi.ac.uk/metagenomics/api/v1/analyses/MGYA00358909/file/SRR2297145_MERGED_FASTQ_SSU_OTU.tsv	Root-Environmental-Aquatic-Marine-Coastal-Sediment
SRR6746171	MGYA00202974	https://www.ebi.ac.uk/metagenomics/api/v1/analyses/MGYA00202974/file/SRR6746171_MERGED_FASTQ_SSU_OTU.tsv	Root-Environmental-Aquatic-Marine-Coastal-Sediment
SRR6746510	MGYA00203056	https://www.ebi.ac.uk/metagenomics/api/v1/analyses/MGYA00203056/file/SRR6746510_MERGED_FASTQ_SSU_OTU.tsv	Root-Environmental-Aquatic-Marine-Coastal-Sediment
SRR6745405	MGYA00202546	https://www.ebi.ac.uk/metagenomics/api/v1/analyses/MGYA00202546/file/SRR6745405_MERGED_FASTQ_SSU_OTU.tsv	Root-Environmental-Aquatic-Marine-Coastal-Sediment
SRR6745877	MGYA00203086	https://www.ebi.ac.uk/metagenomics/api/v1/analyses/MGYA00203086/file/SRR6745877_MERGED_FASTQ_SSU_OTU.tsv	Root-Environmental-Aquatic-Marine-Coastal-Sediment
SRR1503976	MGYA00358531	https://www.ebi.ac.uk/metagenomics/api/v1/analyses/MGYA00358531/file/SRR1503976_FASTQ_SSU_OTU.tsv	Root-Environmental-Aquatic-Marine-Coastal-Sediment
SRR6745373	MGYA00202980	https://www.ebi.ac.uk/metagenomics/api/v1/analyses/MGYA00202980/file/SRR6745373_MERGED_FASTQ_SSU_OTU.tsv	Root-Environmental-Aquatic-Marine-Coastal-Sediment
ERR330051	MGYA00358009	https://www.ebi.ac.uk/metagenomics/api/v1/analyses/MGYA00358009/file/ERR330051_FASTQ_SSU_OTU.tsv	Root-Environmental-Aquatic-Marine-Coastal-Sediment
SRR1258343	MGYA00358466	https://www.ebi.ac.uk/metagenomics/api/v1/analyses/MGYA00358466/file/SRR1258343_FASTQ_SSU_OTU.tsv	Root-Environmental-Aquatic-Marine-Coastal-Sediment
SRR6745928	MGYA00203027	https://www.ebi.ac.uk/metagenomics/api/v1/analyses/MGYA00203027/file/SRR6745928_MERGED_FASTQ_SSU_OTU.tsv	Root-Environmental-Aquatic-Marine-Coastal-Sediment
SRR6746176	MGYA00203076	https://www.ebi.ac.uk/metagenomics/api/v1/analyses/MGYA00203076/file/SRR6746176_MERGED_FASTQ_SSU_OTU.tsv	Root-Environmental-Aquatic-Marine-Coastal-Sediment

SRR2297142	MGYA00358906	https://www.ebi.ac.uk/metagenomics/api/v1/analyses/MGYA00358906/file/SRR2297142_MERGED_FASTQ_SSU_OTU.tsv	Root-Environmental-Aquatic-Marine-Coastal-Sediment
SRR6746517	MGYA00202610	https://www.ebi.ac.uk/metagenomics/api/v1/analyses/MGYA00202610/file/SRR6746517_MERGED_FASTQ_SSU_OTU.tsv	Root-Environmental-Aquatic-Marine-Coastal-Sediment
SRR6745870	MGYA00203119	https://www.ebi.ac.uk/metagenomics/api/v1/analyses/MGYA00203119/file/SRR6745870_MERGED_FASTQ_SSU_OTU.tsv	Root-Environmental-Aquatic-Marine-Coastal-Sediment
SRR6745402	MGYA00202560	https://www.ebi.ac.uk/metagenomics/api/v1/analyses/MGYA00202560/file/SRR6745402_MERGED_FASTQ_SSU_OTU.tsv	Root-Environmental-Aquatic-Marine-Coastal-Sediment
SRR6745374	MGYA00202923	https://www.ebi.ac.uk/metagenomics/api/v1/analyses/MGYA00202923/file/SRR6745374_MERGED_FASTQ_SSU_OTU.tsv	Root-Environmental-Aquatic-Marine-Coastal-Sediment
ERR330056	MGYA00358014	https://www.ebi.ac.uk/metagenomics/api/v1/analyses/MGYA00358014/file/ERR330056_FASTQ_SSU_OTU.tsv	Root-Environmental-Aquatic-Marine-Coastal-Sediment
SRR6745985	MGYA00202785	https://www.ebi.ac.uk/metagenomics/api/v1/analyses/MGYA00202785/file/SRR6745985_MERGED_FASTQ_SSU_OTU.tsv	Root-Environmental-Aquatic-Marine-Coastal-Sediment
SRR6746083	MGYA00202528	https://www.ebi.ac.uk/metagenomics/api/v1/analyses/MGYA00202528/file/SRR6746083_MERGED_FASTQ_SSU_OTU.tsv	Root-Environmental-Aquatic-Marine-Coastal-Sediment
SRR6746052	MGYA00203118	https://www.ebi.ac.uk/metagenomics/api/v1/analyses/MGYA00203118/file/SRR6746052_MERGED_FASTQ_SSU_OTU.tsv	Root-Environmental-Aquatic-Marine-Coastal-Sediment
SRR6745954	MGYA00203106	https://www.ebi.ac.uk/metagenomics/api/v1/analyses/MGYA00203106/file/SRR6745954_MERGED_FASTQ_SSU_OTU.tsv	Root-Environmental-Aquatic-Marine-Coastal-Sediment
SRR6172359	MGYA00362321	https://www.ebi.ac.uk/metagenomics/api/v1/analyses/MGYA00362321/file/SRR6172359_MERGED_FASTQ_SSU_OTU.tsv	Root-Environmental-Aquatic-Marine-Coastal-Sediment
SRR6517610	MGYA00362475	https://www.ebi.ac.uk/metagenomics/api/v1/analyses/MGYA00362475/file/SRR6517610_MERGED_FASTQ_SSU_OTU.tsv	Root-Environmental-Aquatic-Marine-Coastal-Sediment

SRR6746490	MGYA00202840	https://www.ebi.ac.uk/metagenomics/api/v1/analyses/MGYA00202840/file/SRR6746490_MERGED_FASTQ_SSU_OTU.tsv	Root-Environmental-Aquatic-Marine-Coastal-Sediment
SRR6746020	MGYA00202504	https://www.ebi.ac.uk/metagenomics/api/v1/analyses/MGYA00202504/file/SRR6746020_MERGED_FASTQ_SSU_OTU.tsv	Root-Environmental-Aquatic-Marine-Coastal-Sediment
SRR6745926	MGYA00203021	https://www.ebi.ac.uk/metagenomics/api/v1/analyses/MGYA00203021/file/SRR6745926_MERGED_FASTQ_SSU_OTU.tsv	Root-Environmental-Aquatic-Marine-Coastal-Sediment
SRR6746178	MGYA00203000	https://www.ebi.ac.uk/metagenomics/api/v1/analyses/MGYA00203000/file/SRR6746178_MERGED_FASTQ_SSU_OTU.tsv	Root-Environmental-Aquatic-Marine-Coastal-Sediment
SRR6746519	MGYA00203004	https://www.ebi.ac.uk/metagenomics/api/v1/analyses/MGYA00203004/file/SRR6746519_MERGED_FASTQ_SSU_OTU.tsv	Root-Environmental-Aquatic-Marine-Coastal-Sediment
SRR4300283	MGYA00360244	https://www.ebi.ac.uk/metagenomics/api/v1/analyses/MGYA00360244/file/SRR4300283_MERGED_FASTQ_SSU_OTU.tsv	Root-Environmental-Aquatic-Marine-Coastal-Sediment
SRR4436295	MGYA00213052	https://www.ebi.ac.uk/metagenomics/api/v1/analyses/MGYA00213052/file/SRR4436295_FASTQ_SSU_OTU.tsv	Root-Environmental-Aquatic-Marine-Coastal-Sediment
SRR2297130	MGYA00358894	https://www.ebi.ac.uk/metagenomics/api/v1/analyses/MGYA00358894/file/SRR2297130_MERGED_FASTQ_SSU_OTU.tsv	Root-Environmental-Aquatic-Marine-Coastal-Sediment
SRR6746104	MGYA00203038	https://www.ebi.ac.uk/metagenomics/api/v1/analyses/MGYA00203038/file/SRR6746104_MERGED_FASTQ_SSU_OTU.tsv	Root-Environmental-Aquatic-Marine-Coastal-Sediment
SRR6746565	MGYA00202524	https://www.ebi.ac.uk/metagenomics/api/v1/analyses/MGYA00202524/file/SRR6746565_MERGED_FASTQ_SSU_OTU.tsv	Root-Environmental-Aquatic-Marine-Coastal-Sediment
SRR6745802	MGYA00202745	https://www.ebi.ac.uk/metagenomics/api/v1/analyses/MGYA00202745/file/SRR6745802_MERGED_FASTQ_SSU_OTU.tsv	Root-Environmental-Aquatic-Marine-Coastal-Sediment
SRR6172357	MGYA00362319	https://www.ebi.ac.uk/metagenomics/api/v1/analyses/MGYA00362319/file/SRR6172357_MERGED_FASTQ_SSU_OTU.tsv	Root-Environmental-Aquatic-Marine-Coastal-Sediment

SRR6746213	MGYA00202804	https://www.ebi.ac.uk/metagenomics/api/v1/analyses/MGYA00202804/file/SRR6746213_MERGED_FASTQ_SSU_OTU.tsv	Root-Environmental-Aquatic-Marine-Coastal-Sediment
ERR330024	MGYA00357982	https://www.ebi.ac.uk/metagenomics/api/v1/analyses/MGYA00357982/file/ERR330024_FASTQ_SSU_OTU.tsv	Root-Environmental-Aquatic-Marine-Coastal-Sediment
SRR4436292	MGYA00213044	https://www.ebi.ac.uk/metagenomics/api/v1/analyses/MGYA00213044/file/SRR4436292_FASTQ_SSU_OTU.tsv	Root-Environmental-Aquatic-Marine-Coastal-Sediment
SRR6746103	MGYA00202959	https://www.ebi.ac.uk/metagenomics/api/v1/analyses/MGYA00202959/file/SRR6746103_MERGED_FASTQ_SSU_OTU.tsv	Root-Environmental-Aquatic-Marine-Coastal-Sediment
SRR2297137	MGYA00358901	https://www.ebi.ac.uk/metagenomics/api/v1/analyses/MGYA00358901/file/SRR2297137_MERGED_FASTQ_SSU_OTU.tsv	Root-Environmental-Aquatic-Marine-Coastal-Sediment
SRR6746562	MGYA00202647	https://www.ebi.ac.uk/metagenomics/api/v1/analyses/MGYA00202647/file/SRR6746562_MERGED_FASTQ_SSU_OTU.tsv	Root-Environmental-Aquatic-Marine-Coastal-Sediment
SRR6745805	MGYA00202604	https://www.ebi.ac.uk/metagenomics/api/v1/analyses/MGYA00202604/file/SRR6745805_MERGED_FASTQ_SSU_OTU.tsv	Root-Environmental-Aquatic-Marine-Coastal-Sediment
SRR6172350	MGYA00362312	https://www.ebi.ac.uk/metagenomics/api/v1/analyses/MGYA00362312/file/SRR6172350_MERGED_FASTQ_SSU_OTU.tsv	Root-Environmental-Aquatic-Marine-Coastal-Sediment
SRR6746214	MGYA00202536	https://www.ebi.ac.uk/metagenomics/api/v1/analyses/MGYA00202536/file/SRR6746214_MERGED_FASTQ_SSU_OTU.tsv	Root-Environmental-Aquatic-Marine-Coastal-Sediment
ERR330023	MGYA00357981	https://www.ebi.ac.uk/metagenomics/api/v1/analyses/MGYA00357981/file/ERR330023_FASTQ_SSU_OTU.tsv	Root-Environmental-Aquatic-Marine-Coastal-Sediment
SRR6746497	MGYA00202806	https://www.ebi.ac.uk/metagenomics/api/v1/analyses/MGYA00202806/file/SRR6746497_MERGED_FASTQ_SSU_OTU.tsv	Root-Environmental-Aquatic-Marine-Coastal-Sediment
SRR6746027	MGYA00202873	https://www.ebi.ac.uk/metagenomics/api/v1/analyses/MGYA00202873/file/SRR6746027_MERGED_FASTQ_SSU_OTU.tsv	Root-Environmental-Aquatic-Marine-Coastal-Sediment

SRR2712919	MGYA00355957	https://www.ebi.ac.uk/metagenomics/api/v1/analyses/MGYA00355957/file/SRR2712919_FASTQ_SSU_OTU.tsv	Root-Environmental-Aquatic-Marine-Coastal-Sediment
SRR6745921	MGYA00202635	https://www.ebi.ac.uk/metagenomics/api/v1/analyses/MGYA00202635/file/SRR6745921_MERGED_FASTQ_SSU_OTU.tsv	Root-Environmental-Aquatic-Marine-Coastal-Sediment
SRR6745879	MGYA00203085	https://www.ebi.ac.uk/metagenomics/api/v1/analyses/MGYA00203085/file/SRR6745879_MERGED_FASTQ_SSU_OTU.tsv	Root-Environmental-Aquatic-Marine-Coastal-Sediment
SRR1503978	MGYA00358533	https://www.ebi.ac.uk/metagenomics/api/v1/analyses/MGYA00358533/file/SRR1503978_FASTQ_SSU_OTU.tsv	Root-Environmental-Aquatic-Marine-Coastal-Sediment
SRR6745968	MGYA00202616	https://www.ebi.ac.uk/metagenomics/api/v1/analyses/MGYA00202616/file/SRR6745968_MERGED_FASTQ_SSU_OTU.tsv	Root-Environmental-Aquatic-Marine-Coastal-Sediment
SRR2712981	MGYA00355960	https://www.ebi.ac.uk/metagenomics/api/v1/analyses/MGYA00355960/file/SRR2712981_FASTQ_SSU_OTU.tsv	Root-Environmental-Aquatic-Marine-Coastal-Sediment
SRR6745334	MGYA00202740	https://www.ebi.ac.uk/metagenomics/api/v1/analyses/MGYA00202740/file/SRR6745334_MERGED_FASTQ_SSU_OTU.tsv	Root-Environmental-Aquatic-Marine-Coastal-Sediment
SRR6746557	MGYA00202787	https://www.ebi.ac.uk/metagenomics/api/v1/analyses/MGYA00202787/file/SRR6746557_MERGED_FASTQ_SSU_OTU.tsv	Root-Environmental-Aquatic-Marine-Coastal-Sediment
SRR6745830	MGYA00203036	https://www.ebi.ac.uk/metagenomics/api/v1/analyses/MGYA00203036/file/SRR6745830_MERGED_FASTQ_SSU_OTU.tsv	Root-Environmental-Aquatic-Marine-Coastal-Sediment
SRR2297102	MGYA00358866	https://www.ebi.ac.uk/metagenomics/api/v1/analyses/MGYA00358866/file/SRR2297102_MERGED_FASTQ_SSU_OTU.tsv	Root-Environmental-Aquatic-Marine-Coastal-Sediment
SRR6746136	MGYA00202512	https://www.ebi.ac.uk/metagenomics/api/v1/analyses/MGYA00202512/file/SRR6746136_MERGED_FASTQ_SSU_OTU.tsv	Root-Environmental-Aquatic-Marine-Coastal-Sediment
SRR6746473	MGYA00202520	https://www.ebi.ac.uk/metagenomics/api/v1/analyses/MGYA00202520/file/SRR6746473_MERGED_FASTQ_SSU_OTU.tsv	Root-Environmental-Aquatic-Marine-Coastal-Sediment

SRR6745914	MGYA00202731	https://www.ebi.ac.uk/metagenomics/api/v1/analyses/MGYA00202731/file/SRR6745914_MERGED_FASTQ_SSU_OTU.tsv	Root-Environmental-Aquatic-Marine-Coastal-Sediment
SRR6746012	MGYA00202688	https://www.ebi.ac.uk/metagenomics/api/v1/analyses/MGYA00202688/file/SRR6746012_MERGED_FASTQ_SSU_OTU.tsv	Root-Environmental-Aquatic-Marine-Coastal-Sediment
SRR6745348	MGYA00202880	https://www.ebi.ac.uk/metagenomics/api/v1/analyses/MGYA00202880/file/SRR6745348_MERGED_FASTQ_SSU_OTU.tsv	Root-Environmental-Aquatic-Marine-Coastal-Sediment
SRR6745399	MGYA00203059	https://www.ebi.ac.uk/metagenomics/api/v1/analyses/MGYA00203059/file/SRR6745399_MERGED_FASTQ_SSU_OTU.tsv	Root-Environmental-Aquatic-Marine-Coastal-Sediment
SRR3358605	MGYA00364493	https://www.ebi.ac.uk/metagenomics/api/v1/analyses/MGYA00364493/file/SRR3358605_FASTQ_SSU_OTU.tsv	Root-Environmental-Aquatic-Marine-Coastal-Sediment
SRR6746474	MGYA00203063	https://www.ebi.ac.uk/metagenomics/api/v1/analyses/MGYA00203063/file/SRR6746474_MERGED_FASTQ_SSU_OTU.tsv	Root-Environmental-Aquatic-Marine-Coastal-Sediment
SRR6745913	MGYA00202888	https://www.ebi.ac.uk/metagenomics/api/v1/analyses/MGYA00202888/file/SRR6745913_MERGED_FASTQ_SSU_OTU.tsv	Root-Environmental-Aquatic-Marine-Coastal-Sediment
SRR6746015	MGYA00202547	https://www.ebi.ac.uk/metagenomics/api/v1/analyses/MGYA00202547/file/SRR6746015_MERGED_FASTQ_SSU_OTU.tsv	Root-Environmental-Aquatic-Marine-Coastal-Sediment
SRR2240289	MGYA00358470	https://www.ebi.ac.uk/metagenomics/api/v1/analyses/MGYA00358470/file/SRR2240289_FASTQ_SSU_OTU.tsv	Root-Environmental-Aquatic-Marine-Coastal-Sediment
SRR4300267	MGYA00360229	https://www.ebi.ac.uk/metagenomics/api/v1/analyses/MGYA00360229/file/SRR4300267_MERGED_FASTQ_SSU_OTU.tsv	Root-Environmental-Aquatic-Marine-Coastal-Sediment
SRR2297179	MGYA00358943	https://www.ebi.ac.uk/metagenomics/api/v1/analyses/MGYA00358943/file/SRR2297179_MERGED_FASTQ_SSU_OTU.tsv	Root-Environmental-Aquatic-Marine-Coastal-Sediment
SRR6746069	MGYA00203015	https://www.ebi.ac.uk/metagenomics/api/v1/analyses/MGYA00203015/file/SRR6746069_MERGED_FASTQ_SSU_OTU.tsv	Root-Environmental-Aquatic-Marine-Coastal-Sediment

SRR6745333	MGYA00203103	https://www.ebi.ac.uk/metagenomics/api/v1/analyses/MGYA00203103/file/SRR6745333_MERGED_FASTQ_SSU_OTU.tsv	Root-Environmental-Aquatic-Marine-Coastal-Sediment
SRR6746550	MGYA00203041	https://www.ebi.ac.uk/metagenomics/api/v1/analyses/MGYA00203041/file/SRR6746550_MERGED_FASTQ_SSU_OTU.tsv	Root-Environmental-Aquatic-Marine-Coastal-Sediment
SRR5097529	MGYA00360541	https://www.ebi.ac.uk/metagenomics/api/v1/analyses/MGYA00360541/file/SRR5097529_MERGED_FASTQ_SSU_OTU.tsv	Root-Environmental-Aquatic-Marine-Coastal-Sediment
SRR6745837	MGYA00202984	https://www.ebi.ac.uk/metagenomics/api/v1/analyses/MGYA00202984/file/SRR6745837_MERGED_FASTQ_SSU_OTU.tsv	Root-Environmental-Aquatic-Marine-Coastal-Sediment
SRR6746131	MGYA00202660	https://www.ebi.ac.uk/metagenomics/api/v1/analyses/MGYA00202660/file/SRR6746131_MERGED_FASTQ_SSU_OTU.tsv	Root-Environmental-Aquatic-Marine-Coastal-Sediment
SRR2297105	MGYA00358869	https://www.ebi.ac.uk/metagenomics/api/v1/analyses/MGYA00358869/file/SRR2297105_MERGED_FASTQ_SSU_OTU.tsv	Root-Environmental-Aquatic-Marine-Coastal-Sediment
SRR3355074	MGYA00359645	https://www.ebi.ac.uk/metagenomics/api/v1/analyses/MGYA00359645/file/SRR3355074_MERGED_FASTQ_SSU_OTU.tsv	Root-Environmental-Aquatic-Marine-Coastal-Sediment
SRR2855280	MGYA00359212	https://www.ebi.ac.uk/metagenomics/api/v1/analyses/MGYA00359212/file/SRR2855280_MERGED_FASTQ_SSU_OTU.tsv	Root-Environmental-Aquatic-Marine-Coastal-Sediment
SRR6746192	MGYA00202877	https://www.ebi.ac.uk/metagenomics/api/v1/analyses/MGYA00202877/file/SRR6746192_MERGED_FASTQ_SSU_OTU.tsv	Root-Environmental-Aquatic-Marine-Coastal-Sediment
SRR6745341	MGYA00202819	https://www.ebi.ac.uk/metagenomics/api/v1/analyses/MGYA00202819/file/SRR6745341_MERGED_FASTQ_SSU_OTU.tsv	Root-Environmental-Aquatic-Marine-Coastal-Sediment
SRR6746522	MGYA00203107	https://www.ebi.ac.uk/metagenomics/api/v1/analyses/MGYA00203107/file/SRR6746522_MERGED_FASTQ_SSU_OTU.tsv	Root-Environmental-Aquatic-Marine-Coastal-Sediment
SRR6745390	MGYA00202505	https://www.ebi.ac.uk/metagenomics/api/v1/analyses/MGYA00202505/file/SRR6745390_MERGED_FASTQ_SSU_OTU.tsv	Root-Environmental-Aquatic-Marine-Coastal-Sediment

SRR6745845	MGYA00202776	https://www.ebi.ac.uk/metagenomics/api/v1/analyses/MGYA00202776/file/SRR6745845_MERGED_FASTQ_SSU_OTU.tsv	Root-Environmental-Aquatic-Marine-Coastal-Sediment
SRR2297177	MGYA00358941	https://www.ebi.ac.uk/metagenomics/api/v1/analyses/MGYA00358941/file/SRR2297177_MERGED_FASTQ_SSU_OTU.tsv	Root-Environmental-Aquatic-Marine-Coastal-Sediment
SRR6746143	MGYA00203006	https://www.ebi.ac.uk/metagenomics/api/v1/analyses/MGYA00203006/file/SRR6746143_MERGED_FASTQ_SSU_OTU.tsv	Root-Environmental-Aquatic-Marine-Coastal-Sediment
SRR4300269	MGYA00360231	https://www.ebi.ac.uk/metagenomics/api/v1/analyses/MGYA00360231/file/SRR4300269_MERGED_FASTQ_SSU_OTU.tsv	Root-Environmental-Aquatic-Marine-Coastal-Sediment
SRR6745961	MGYA00202543	https://www.ebi.ac.uk/metagenomics/api/v1/analyses/MGYA00202543/file/SRR6745961_MERGED_FASTQ_SSU_OTU.tsv	Root-Environmental-Aquatic-Marine-Coastal-Sediment
SRR6746067	MGYA00203105	https://www.ebi.ac.uk/metagenomics/api/v1/analyses/MGYA00203105/file/SRR6746067_MERGED_FASTQ_SSU_OTU.tsv	Root-Environmental-Aquatic-Marine-Coastal-Sediment
SRR6745839	MGYA00202993	https://www.ebi.ac.uk/metagenomics/api/v1/analyses/MGYA00202993/file/SRR6745839_MERGED_FASTQ_SSU_OTU.tsv	Root-Environmental-Aquatic-Marine-Coastal-Sediment
SRR5097527	MGYA00360539	https://www.ebi.ac.uk/metagenomics/api/v1/analyses/MGYA00360539/file/SRR5097527_MERGED_FASTQ_SSU_OTU.tsv	Root-Environmental-Aquatic-Marine-Coastal-Sediment
SRR6745966	MGYA00202861	https://www.ebi.ac.uk/metagenomics/api/v1/analyses/MGYA00202861/file/SRR6745966_MERGED_FASTQ_SSU_OTU.tsv	Root-Environmental-Aquatic-Marine-Coastal-Sediment
SRR6746060	MGYA00202620	https://www.ebi.ac.uk/metagenomics/api/v1/analyses/MGYA00202620/file/SRR6746060_MERGED_FASTQ_SSU_OTU.tsv	Root-Environmental-Aquatic-Marine-Coastal-Sediment
SRR2856449	MGYA00359225	https://www.ebi.ac.uk/metagenomics/api/v1/analyses/MGYA00359225/file/SRR2856449_MERGED_FASTQ_SSU_OTU.tsv	Root-Environmental-Aquatic-Marine-Coastal-Sediment
SRR5227110	MGYA00360669	https://www.ebi.ac.uk/metagenomics/api/v1/analyses/MGYA00360669/file/SRR5227110_FASTQ_SSU_OTU.tsv	Root-Environmental-Aquatic-Marine-Coastal-Sediment

ERR330018	MGYA00357976	https://www.ebi.ac.uk/metagenomics/api/v1/analyses/MGYA00357976/file/ERR330018_FASTQ_SSU_OTU.tsv	Root-Environmental-Aquatic-Marine-Coastal-Sediment
SRR6746559	MGYA00202827	https://www.ebi.ac.uk/metagenomics/api/v1/analyses/MGYA00202827/file/SRR6746559_MERGED_FASTQ_SSU_OTU.tsv	Root-Environmental-Aquatic-Marine-Coastal-Sediment
SRR6746138	MGYA00203030	https://www.ebi.ac.uk/metagenomics/api/v1/analyses/MGYA00203030/file/SRR6746138_MERGED_FASTQ_SSU_OTU.tsv	Root-Environmental-Aquatic-Marine-Coastal-Sediment
SRR6746195	MGYA00202969	https://www.ebi.ac.uk/metagenomics/api/v1/analyses/MGYA00202969/file/SRR6746195_MERGED_FASTQ_SSU_OTU.tsv	Root-Environmental-Aquatic-Marine-Coastal-Sediment
SRR6745346	MGYA00202836	https://www.ebi.ac.uk/metagenomics/api/v1/analyses/MGYA00202836/file/SRR6745346_MERGED_FASTQ_SSU_OTU.tsv	Root-Environmental-Aquatic-Marine-Coastal-Sediment
SRR6746525	MGYA00202851	https://www.ebi.ac.uk/metagenomics/api/v1/analyses/MGYA00202851/file/SRR6746525_MERGED_FASTQ_SSU_OTU.tsv	Root-Environmental-Aquatic-Marine-Coastal-Sediment
SRR6745430	MGYA00202987	https://www.ebi.ac.uk/metagenomics/api/v1/analyses/MGYA00202987/file/SRR6745430_MERGED_FASTQ_SSU_OTU.tsv	Root-Environmental-Aquatic-Marine-Coastal-Sediment
SRR6745842	MGYA00202650	https://www.ebi.ac.uk/metagenomics/api/v1/analyses/MGYA00202650/file/SRR6745842_MERGED_FASTQ_SSU_OTU.tsv	Root-Environmental-Aquatic-Marine-Coastal-Sediment
SRR6745397	MGYA00203074	https://www.ebi.ac.uk/metagenomics/api/v1/analyses/MGYA00203074/file/SRR6745397_MERGED_FASTQ_SSU_OTU.tsv	Root-Environmental-Aquatic-Marine-Coastal-Sediment
SRR6746144	MGYA00202545	https://www.ebi.ac.uk/metagenomics/api/v1/analyses/MGYA00202545/file/SRR6746144_MERGED_FASTQ_SSU_OTU.tsv	Root-Environmental-Aquatic-Marine-Coastal-Sediment
SRR2297170	MGYA00358934	https://www.ebi.ac.uk/metagenomics/api/v1/analyses/MGYA00358934/file/SRR2297170_MERGED_FASTQ_SSU_OTU.tsv	Root-Environmental-Aquatic-Marine-Coastal-Sediment
SRR3355020	MGYA00359637	https://www.ebi.ac.uk/metagenomics/api/v1/analyses/MGYA00359637/file/SRR3355020_MERGED_FASTQ_SSU_OTU.tsv	Root-Environmental-Aquatic-Marine-Coastal-Sediment

SRR2297129	MGYA00358893	https://www.ebi.ac.uk/metagenomics/api/v1/analyses/MGYA00358893/file/SRR2297129_MERGED_FASTQ_SSU_OTU.tsv	Root-Environmental-Aquatic-Marine-Coastal-Sediment
SRR4436305	MGYA00213051	https://www.ebi.ac.uk/metagenomics/api/v1/analyses/MGYA00213051/file/SRR4436305_FASTQ_SSU_OTU.tsv	Root-Environmental-Aquatic-Marine-Coastal-Sediment
SRR6746045	MGYA00202930	https://www.ebi.ac.uk/metagenomics/api/v1/analyses/MGYA00202930/file/SRR6746045_MERGED_FASTQ_SSU_OTU.tsv	Root-Environmental-Aquatic-Marine-Coastal-Sediment
SRR6745943	MGYA00202958	https://www.ebi.ac.uk/metagenomics/api/v1/analyses/MGYA00202958/file/SRR6745943_MERGED_FASTQ_SSU_OTU.tsv	Root-Environmental-Aquatic-Marine-Coastal-Sediment
SRR6745992	MGYA00202561	https://www.ebi.ac.uk/metagenomics/api/v1/analyses/MGYA00202561/file/SRR6745992_MERGED_FASTQ_SSU_OTU.tsv	Root-Environmental-Aquatic-Marine-Coastal-Sediment
SRR6746094	MGYA00202832	https://www.ebi.ac.uk/metagenomics/api/v1/analyses/MGYA00202832/file/SRR6746094_MERGED_FASTQ_SSU_OTU.tsv	Root-Environmental-Aquatic-Marine-Coastal-Sediment
SRR6745363	MGYA00202941	https://www.ebi.ac.uk/metagenomics/api/v1/analyses/MGYA00202941/file/SRR6745363_MERGED_FASTQ_SSU_OTU.tsv	Root-Environmental-Aquatic-Marine-Coastal-Sediment
SRR6172332	MGYA00362294	https://www.ebi.ac.uk/metagenomics/api/v1/analyses/MGYA00362294/file/SRR6172332_MERGED_FASTQ_SSU_OTU.tsv	Root-Environmental-Aquatic-Marine-Coastal-Sediment
ERR330041	MGYA00357999	https://www.ebi.ac.uk/metagenomics/api/v1/analyses/MGYA00357999/file/ERR330041_FASTQ_SSU_OTU.tsv	Root-Environmental-Aquatic-Marine-Coastal-Sediment
SRR2297184	MGYA00358948	https://www.ebi.ac.uk/metagenomics/api/v1/analyses/MGYA00358948/file/SRR2297184_MERGED_FASTQ_SSU_OTU.tsv	Root-Environmental-Aquatic-Marine-Coastal-Sediment
SRR2297155	MGYA00358919	https://www.ebi.ac.uk/metagenomics/api/v1/analyses/MGYA00358919/file/SRR2297155_MERGED_FASTQ_SSU_OTU.tsv	Root-Environmental-Aquatic-Marine-Coastal-Sediment
SRR6746161	MGYA00202913	https://www.ebi.ac.uk/metagenomics/api/v1/analyses/MGYA00202913/file/SRR6746161_MERGED_FASTQ_SSU_OTU.tsv	Root-Environmental-Aquatic-Marine-Coastal-Sediment

SRR6746500	MGYA00202972	https://www.ebi.ac.uk/metagenomics/api/v1/analyses/MGYA00202972/file/SRR6746500_MERGED_FASTQ_SSU_OTU.tsv	Root-Environmental-Aquatic-Marine-Coastal-Sediment
SRR6745867	MGYA00202636	https://www.ebi.ac.uk/metagenomics/api/v1/analyses/MGYA00202636/file/SRR6745867_MERGED_FASTQ_SSU_OTU.tsv	Root-Environmental-Aquatic-Marine-Coastal-Sediment
SRR6745415	MGYA00202735	https://www.ebi.ac.uk/metagenomics/api/v1/analyses/MGYA00202735/file/SRR6745415_MERGED_FASTQ_SSU_OTU.tsv	Root-Environmental-Aquatic-Marine-Coastal-Sediment
SRR6746039	MGYA00202831	https://www.ebi.ac.uk/metagenomics/api/v1/analyses/MGYA00202831/file/SRR6746039_MERGED_FASTQ_SSU_OTU.tsv	Root-Environmental-Aquatic-Marine-Coastal-Sediment
SRR6517609	MGYA00362474	https://www.ebi.ac.uk/metagenomics/api/v1/analyses/MGYA00362474/file/SRR6517609_MERGED_FASTQ_SSU_OTU.tsv	Root-Environmental-Aquatic-Marine-Coastal-Sediment
SRR2239573	MGYA00358469	https://www.ebi.ac.uk/metagenomics/api/v1/analyses/MGYA00358469/file/SRR2239573_FASTQ_SSU_OTU.tsv	Root-Environmental-Aquatic-Marine-Coastal-Sediment
SRR6746489	MGYA00202825	https://www.ebi.ac.uk/metagenomics/api/v1/analyses/MGYA00202825/file/SRR6746489_MERGED_FASTQ_SSU_OTU.tsv	Root-Environmental-Aquatic-Marine-Coastal-Sediment
SRR6172335	MGYA00362297	https://www.ebi.ac.uk/metagenomics/api/v1/analyses/MGYA00362297/file/SRR6172335_MERGED_FASTQ_SSU_OTU.tsv	Root-Environmental-Aquatic-Marine-Coastal-Sediment
SRR6745364	MGYA00202812	https://www.ebi.ac.uk/metagenomics/api/v1/analyses/MGYA00202812/file/SRR6745364_MERGED_FASTQ_SSU_OTU.tsv	Root-Environmental-Aquatic-Marine-Coastal-Sediment
ERR330046	MGYA00358004	https://www.ebi.ac.uk/metagenomics/api/v1/analyses/MGYA00358004/file/ERR330046_FASTQ_SSU_OTU.tsv	Root-Environmental-Aquatic-Marine-Coastal-Sediment
SRR2297183	MGYA00358947	https://www.ebi.ac.uk/metagenomics/api/v1/analyses/MGYA00358947/file/SRR2297183_MERGED_FASTQ_SSU_OTU.tsv	Root-Environmental-Aquatic-Marine-Coastal-Sediment
SRR6746166	MGYA00203077	https://www.ebi.ac.uk/metagenomics/api/v1/analyses/MGYA00203077/file/SRR6746166_MERGED_FASTQ_SSU_OTU.tsv	Root-Environmental-Aquatic-Marine-Coastal-Sediment

SRR2297152	MGYA00358916	https://www.ebi.ac.uk/metagenomics/api/v1/analyses/MGYA00358916/file/SRR2297152_MERGED_FASTQ_SSU_OTU.tsv	Root-Environmental-Aquatic-Marine-Coastal-Sediment
SRR6746507	MGYA00202679	https://www.ebi.ac.uk/metagenomics/api/v1/analyses/MGYA00202679/file/SRR6746507_MERGED_FASTQ_SSU_OTU.tsv	Root-Environmental-Aquatic-Marine-Coastal-Sediment
SRR6745412	MGYA00202950	https://www.ebi.ac.uk/metagenomics/api/v1/analyses/MGYA00202950/file/SRR6745412_MERGED_FASTQ_SSU_OTU.tsv	Root-Environmental-Aquatic-Marine-Coastal-Sediment
SRR6745860	MGYA00203100	https://www.ebi.ac.uk/metagenomics/api/v1/analyses/MGYA00203100/file/SRR6745860_MERGED_FASTQ_SSU_OTU.tsv	Root-Environmental-Aquatic-Marine-Coastal-Sediment
SRR6745938	MGYA00202736	https://www.ebi.ac.uk/metagenomics/api/v1/analyses/MGYA00202736/file/SRR6745938_MERGED_FASTQ_SSU_OTU.tsv	Root-Environmental-Aquatic-Marine-Coastal-Sediment
SRR6172349	MGYA00362311	https://www.ebi.ac.uk/metagenomics/api/v1/analyses/MGYA00362311/file/SRR6172349_MERGED_FASTQ_SSU_OTU.tsv	Root-Environmental-Aquatic-Marine-Coastal-Sediment
SRR4436302	MGYA00213048	https://www.ebi.ac.uk/metagenomics/api/v1/analyses/MGYA00213048/file/SRR4436302_FASTQ_SSU_OTU.tsv	Root-Environmental-Aquatic-Marine-Coastal-Sediment
SRR6746042	MGYA00202963	https://www.ebi.ac.uk/metagenomics/api/v1/analyses/MGYA00202963/file/SRR6746042_MERGED_FASTQ_SSU_OTU.tsv	Root-Environmental-Aquatic-Marine-Coastal-Sediment
SRR6745944	MGYA00202510	https://www.ebi.ac.uk/metagenomics/api/v1/analyses/MGYA00202510/file/SRR6745944_MERGED_FASTQ_SSU_OTU.tsv	Root-Environmental-Aquatic-Marine-Coastal-Sediment
SRR6745995	MGYA00202839	https://www.ebi.ac.uk/metagenomics/api/v1/analyses/MGYA00202839/file/SRR6745995_MERGED_FASTQ_SSU_OTU.tsv	Root-Environmental-Aquatic-Marine-Coastal-Sediment
SRR6746093	MGYA00202781	https://www.ebi.ac.uk/metagenomics/api/v1/analyses/MGYA00202781/file/SRR6746093_MERGED_FASTQ_SSU_OTU.tsv	Root-Environmental-Aquatic-Marine-Coastal-Sediment
SRR650833	MGYA00356182	https://www.ebi.ac.uk/metagenomics/api/v1/analyses/MGYA00356182/file/SRR650833_FASTQ_SSU_OTU.tsv	Root-Environmental-Aquatic-Marine-Coastal-Sediment

ERR330048	MGYA00358006	https://www.ebi.ac.uk/metagenomics/api/v1/analyses/MGYA00358006/file/ERR330048_FASTQ_SSU_OTU.tsv	Root-Environmental-Aquatic-Marine-Coastal-Sediment
SRR4300293	MGYA00360253	https://www.ebi.ac.uk/metagenomics/api/v1/analyses/MGYA00360253/file/SRR4300293_MERGED_FASTQ_SSU_OTU.tsv	Root-Environmental-Aquatic-Marine-Coastal-Sediment
SRR6746168	MGYA00203083	https://www.ebi.ac.uk/metagenomics/api/v1/analyses/MGYA00203083/file/SRR6746168_MERGED_FASTQ_SSU_OTU.tsv	Root-Environmental-Aquatic-Marine-Coastal-Sediment
SRR6746509	MGYA00203031	https://www.ebi.ac.uk/metagenomics/api/v1/analyses/MGYA00203031/file/SRR6746509_MERGED_FASTQ_SSU_OTU.tsv	Root-Environmental-Aquatic-Marine-Coastal-Sediment
SRR6746030	MGYA00202793	https://www.ebi.ac.uk/metagenomics/api/v1/analyses/MGYA00202793/file/SRR6746030_MERGED_FASTQ_SSU_OTU.tsv	Root-Environmental-Aquatic-Marine-Coastal-Sediment
SRR6745936	MGYA00202967	https://www.ebi.ac.uk/metagenomics/api/v1/analyses/MGYA00202967/file/SRR6745936_MERGED_FASTQ_SSU_OTU.tsv	Root-Environmental-Aquatic-Marine-Coastal-Sediment
SRR6746480	MGYA00202748	https://www.ebi.ac.uk/metagenomics/api/v1/analyses/MGYA00202748/file/SRR6746480_MERGED_FASTQ_SSU_OTU.tsv	Root-Environmental-Aquatic-Marine-Coastal-Sediment
SRR6172347	MGYA00362309	https://www.ebi.ac.uk/metagenomics/api/v1/analyses/MGYA00362309/file/SRR6172347_MERGED_FASTQ_SSU_OTU.tsv	Root-Environmental-Aquatic-Marine-Coastal-Sediment
ERR330034	MGYA00357992	https://www.ebi.ac.uk/metagenomics/api/v1/analyses/MGYA00357992/file/ERR330034_FASTQ_SSU_OTU.tsv	Root-Environmental-Aquatic-Marine-Coastal-Sediment
SRR6746203	MGYA00202994	https://www.ebi.ac.uk/metagenomics/api/v1/analyses/MGYA00202994/file/SRR6746203_MERGED_FASTQ_SSU_OTU.tsv	Root-Environmental-Aquatic-Marine-Coastal-Sediment
SRR3355080	MGYA00359648	https://www.ebi.ac.uk/metagenomics/api/v1/analyses/MGYA00359648/file/SRR3355080_MERGED_FASTQ_SSU_OTU.tsv	Root-Environmental-Aquatic-Marine-Coastal-Sediment
SRR2297120	MGYA00358884	https://www.ebi.ac.uk/metagenomics/api/v1/analyses/MGYA00358884/file/SRR2297120_MERGED_FASTQ_SSU_OTU.tsv	Root-Environmental-Aquatic-Marine-Coastal-Sediment

SRR6746114	MGYA00202626	https://www.ebi.ac.uk/metagenomics/api/v1/analyses/MGYA00202626/file/SRR6746114_MERGED_FASTQ_SSU_OTU.tsv	Root-Environmental-Aquatic-Marine-Coastal-Sediment
SRR3355029	MGYA00359641	https://www.ebi.ac.uk/metagenomics/api/v1/analyses/MGYA00359641/file/SRR3355029_MERGED_FASTQ_SSU_OTU.tsv	Root-Environmental-Aquatic-Marine-Coastal-Sediment
SRR6745812	MGYA00202759	https://www.ebi.ac.uk/metagenomics/api/v1/analyses/MGYA00202759/file/SRR6745812_MERGED_FASTQ_SSU_OTU.tsv	Root-Environmental-Aquatic-Marine-Coastal-Sediment
SRR4436285	MGYA00213047	https://www.ebi.ac.uk/metagenomics/api/v1/analyses/MGYA00213047/file/SRR4436285_FASTQ_SSU_OTU.tsv	Root-Environmental-Aquatic-Marine-Coastal-Sediment
SRR6172340	MGYA00362302	https://www.ebi.ac.uk/metagenomics/api/v1/analyses/MGYA00362302/file/SRR6172340_MERGED_FASTQ_SSU_OTU.tsv	Root-Environmental-Aquatic-Marine-Coastal-Sediment
ERR330033	MGYA00357991	https://www.ebi.ac.uk/metagenomics/api/v1/analyses/MGYA00357991/file/ERR330033_FASTQ_SSU_OTU.tsv	Root-Environmental-Aquatic-Marine-Coastal-Sediment
SRR6746204	MGYA00202672	https://www.ebi.ac.uk/metagenomics/api/v1/analyses/MGYA00202672/file/SRR6746204_MERGED_FASTQ_SSU_OTU.tsv	Root-Environmental-Aquatic-Marine-Coastal-Sediment
SRR6746113	MGYA00202663	https://www.ebi.ac.uk/metagenomics/api/v1/analyses/MGYA00202663/file/SRR6746113_MERGED_FASTQ_SSU_OTU.tsv	Root-Environmental-Aquatic-Marine-Coastal-Sediment
SRR2297127	MGYA00358891	https://www.ebi.ac.uk/metagenomics/api/v1/analyses/MGYA00358891/file/SRR2297127_MERGED_FASTQ_SSU_OTU.tsv	Root-Environmental-Aquatic-Marine-Coastal-Sediment
SRR6745815	MGYA00202920	https://www.ebi.ac.uk/metagenomics/api/v1/analyses/MGYA00202920/file/SRR6745815_MERGED_FASTQ_SSU_OTU.tsv	Root-Environmental-Aquatic-Marine-Coastal-Sediment
SRR6745869	MGYA00202921	https://www.ebi.ac.uk/metagenomics/api/v1/analyses/MGYA00202921/file/SRR6745869_MERGED_FASTQ_SSU_OTU.tsv	Root-Environmental-Aquatic-Marine-Coastal-Sediment
SRR6746037	MGYA00202568	https://www.ebi.ac.uk/metagenomics/api/v1/analyses/MGYA00202568/file/SRR6746037_MERGED_FASTQ_SSU_OTU.tsv	Root-Environmental-Aquatic-Marine-Coastal-Sediment

SRR6745931	MGYA00202820	https://www.ebi.ac.uk/metagenomics/api/v1/analyses/MGYA00202820/file/SRR6745931_MERGED_FASTQ_SSU_OTU.tsv	Root-Environmental-Aquatic-Marine-Coastal-Sediment
SRR6746487	MGYA00202917	https://www.ebi.ac.uk/metagenomics/api/v1/analyses/MGYA00202917/file/SRR6746487_MERGED_FASTQ_SSU_OTU.tsv	Root-Environmental-Aquatic-Marine-Coastal-Sediment
SRR6517607	MGYA00362472	https://www.ebi.ac.uk/metagenomics/api/v1/analyses/MGYA00362472/file/SRR6517607_MERGED_FASTQ_SSU_OTU.tsv	Root-Environmental-Aquatic-Marine-Coastal-Sediment
SRR6746547	MGYA00202693	https://www.ebi.ac.uk/metagenomics/api/v1/analyses/MGYA00202693/file/SRR6746547_MERGED_FASTQ_SSU_OTU.tsv	Root-Environmental-Aquatic-Marine-Coastal-Sediment
SRR6745820	MGYA00202925	https://www.ebi.ac.uk/metagenomics/api/v1/analyses/MGYA00202925/file/SRR6745820_MERGED_FASTQ_SSU_OTU.tsv	Root-Environmental-Aquatic-Marine-Coastal-Sediment
SRR2297112	MGYA00358876	https://www.ebi.ac.uk/metagenomics/api/v1/analyses/MGYA00358876/file/SRR2297112_MERGED_FASTQ_SSU_OTU.tsv	Root-Environmental-Aquatic-Marine-Coastal-Sediment
SRR6746126	MGYA00202534	https://www.ebi.ac.uk/metagenomics/api/v1/analyses/MGYA00202534/file/SRR6746126_MERGED_FASTQ_SSU_OTU.tsv	Root-Environmental-Aquatic-Marine-Coastal-Sediment
SRR6745794	MGYA00202588	https://www.ebi.ac.uk/metagenomics/api/v1/analyses/MGYA00202588/file/SRR6745794_MERGED_FASTQ_SSU_OTU.tsv	Root-Environmental-Aquatic-Marine-Coastal-Sediment
SRR2856457	MGYA00359232	https://www.ebi.ac.uk/metagenomics/api/v1/analyses/MGYA00359232/file/SRR2856457_MERGED_FASTQ_SSU_OTU.tsv	Root-Environmental-Aquatic-Marine-Coastal-Sediment
SRR6745978	MGYA00203022	https://www.ebi.ac.uk/metagenomics/api/v1/analyses/MGYA00203022/file/SRR6745978_MERGED_FASTQ_SSU_OTU.tsv	Root-Environmental-Aquatic-Marine-Coastal-Sediment
SRR6745389	MGYA00202699	https://www.ebi.ac.uk/metagenomics/api/v1/analyses/MGYA00202699/file/SRR6745389_MERGED_FASTQ_SSU_OTU.tsv	Root-Environmental-Aquatic-Marine-Coastal-Sediment
SRR4300270	MGYA00360232	https://www.ebi.ac.uk/metagenomics/api/v1/analyses/MGYA00360232/file/SRR4300270_MERGED_FASTQ_SSU_OTU.tsv	Root-Environmental-Aquatic-Marine-Coastal-Sediment

SRR6745358	MGYA00202852	https://www.ebi.ac.uk/metagenomics/api/v1/analyses/MGYA00202852/file/SRR6745358_MERGED_FASTQ_SSU_OTU.tsv	Root-Environmental-Aquatic-Marine-Coastal-Sediment
SRR6746002	MGYA00202939	https://www.ebi.ac.uk/metagenomics/api/v1/analyses/MGYA00202939/file/SRR6746002_MERGED_FASTQ_SSU_OTU.tsv	Root-Environmental-Aquatic-Marine-Coastal-Sediment
SRR6745429	MGYA00202935	https://www.ebi.ac.uk/metagenomics/api/v1/analyses/MGYA00202935/file/SRR6745429_MERGED_FASTQ_SSU_OTU.tsv	Root-Environmental-Aquatic-Marine-Coastal-Sediment
SRR4300277	MGYA00360238	https://www.ebi.ac.uk/metagenomics/api/v1/analyses/MGYA00360238/file/SRR4300277_MERGED_FASTQ_SSU_OTU.tsv	Root-Environmental-Aquatic-Marine-Coastal-Sediment
SRR2297169	MGYA00358933	https://www.ebi.ac.uk/metagenomics/api/v1/analyses/MGYA00358933/file/SRR2297169_MERGED_FASTQ_SSU_OTU.tsv	Root-Environmental-Aquatic-Marine-Coastal-Sediment
SRR6746005	MGYA00202876	https://www.ebi.ac.uk/metagenomics/api/v1/analyses/MGYA00202876/file/SRR6746005_MERGED_FASTQ_SSU_OTU.tsv	Root-Environmental-Aquatic-Marine-Coastal-Sediment
SRR6746540	MGYA00203003	https://www.ebi.ac.uk/metagenomics/api/v1/analyses/MGYA00203003/file/SRR6746540_MERGED_FASTQ_SSU_OTU.tsv	Root-Environmental-Aquatic-Marine-Coastal-Sediment
SRR5097539	MGYA00360551	https://www.ebi.ac.uk/metagenomics/api/v1/analyses/MGYA00360551/file/SRR5097539_MERGED_FASTQ_SSU_OTU.tsv	Root-Environmental-Aquatic-Marine-Coastal-Sediment
SRR6745827	MGYA00202802	https://www.ebi.ac.uk/metagenomics/api/v1/analyses/MGYA00202802/file/SRR6745827_MERGED_FASTQ_SSU_OTU.tsv	Root-Environmental-Aquatic-Marine-Coastal-Sediment
SRR6746121	MGYA00202589	https://www.ebi.ac.uk/metagenomics/api/v1/analyses/MGYA00202589/file/SRR6746121_MERGED_FASTQ_SSU_OTU.tsv	Root-Environmental-Aquatic-Marine-Coastal-Sediment
SRR2297115	MGYA00358879	https://www.ebi.ac.uk/metagenomics/api/v1/analyses/MGYA00358879/file/SRR2297115_MERGED_FASTQ_SSU_OTU.tsv	Root-Environmental-Aquatic-Marine-Coastal-Sediment
SRR6745793	MGYA00202940	https://www.ebi.ac.uk/metagenomics/api/v1/analyses/MGYA00202940/file/SRR6745793_MERGED_FASTQ_SSU_OTU.tsv	Root-Environmental-Aquatic-Marine-Coastal-Sediment

SRR5227109	MGYA00360668	https://www.ebi.ac.uk/metagenomics/api/v1/analyses/MGYA00360668/file/SRR5227109_FASTQ_SSU_OTU.tsv	Root-Environmental-Aquatic-Marine-Coastal-Sediment
SRR2856450	MGYA00359226	https://www.ebi.ac.uk/metagenomics/api/v1/analyses/MGYA00359226/file/SRR2856450_MERGED_FASTQ_SSU_OTU.tsv	Root-Environmental-Aquatic-Marine-Coastal-Sediment
SRR6746079	MGYA00202492	https://www.ebi.ac.uk/metagenomics/api/v1/analyses/MGYA00202492/file/SRR6746079_MERGED_FASTQ_SSU_OTU.tsv	Root-Environmental-Aquatic-Marine-Coastal-Sediment
SRR6746532	MGYA00202684	https://www.ebi.ac.uk/metagenomics/api/v1/analyses/MGYA00202684/file/SRR6746532_MERGED_FASTQ_SSU_OTU.tsv	Root-Environmental-Aquatic-Marine-Coastal-Sediment
SRR6745380	MGYA00202973	https://www.ebi.ac.uk/metagenomics/api/v1/analyses/MGYA00202973/file/SRR6745380_MERGED_FASTQ_SSU_OTU.tsv	Root-Environmental-Aquatic-Marine-Coastal-Sediment
SRR6745427	MGYA00202814	https://www.ebi.ac.uk/metagenomics/api/v1/analyses/MGYA00202814/file/SRR6745427_MERGED_FASTQ_SSU_OTU.tsv	Root-Environmental-Aquatic-Marine-Coastal-Sediment
SRR6745855	MGYA00203099	https://www.ebi.ac.uk/metagenomics/api/v1/analyses/MGYA00203099/file/SRR6745855_MERGED_FASTQ_SSU_OTU.tsv	Root-Environmental-Aquatic-Marine-Coastal-Sediment
SRR2297167	MGYA00358931	https://www.ebi.ac.uk/metagenomics/api/v1/analyses/MGYA00358931/file/SRR2297167_MERGED_FASTQ_SSU_OTU.tsv	Root-Environmental-Aquatic-Marine-Coastal-Sediment
SRR6746153	MGYA00202800	https://www.ebi.ac.uk/metagenomics/api/v1/analyses/MGYA00202800/file/SRR6746153_MERGED_FASTQ_SSU_OTU.tsv	Root-Environmental-Aquatic-Marine-Coastal-Sediment
SRR4300279	MGYA00360240	https://www.ebi.ac.uk/metagenomics/api/v1/analyses/MGYA00360240/file/SRR4300279_MERGED_FASTQ_SSU_OTU.tsv	Root-Environmental-Aquatic-Marine-Coastal-Sediment
SRR6746182	MGYA00202737	https://www.ebi.ac.uk/metagenomics/api/v1/analyses/MGYA00202737/file/SRR6746182_MERGED_FASTQ_SSU_OTU.tsv	Root-Environmental-Aquatic-Marine-Coastal-Sediment
SRR6745351	MGYA00202634	https://www.ebi.ac.uk/metagenomics/api/v1/analyses/MGYA00202634/file/SRR6745351_MERGED_FASTQ_SSU_OTU.tsv	Root-Environmental-Aquatic-Marine-Coastal-Sediment

SRR1503985	MGYA00358540	https://www.ebi.ac.uk/metagenomics/api/v1/analyses/MGYA00358540/file/SRR1503985_FASTQ_SSU_OTU.tsv	Root-Environmental-Aquatic-Marine-Coastal-Sediment
SRR6745884	MGYA00202710	https://www.ebi.ac.uk/metagenomics/api/v1/analyses/MGYA00202710/file/SRR6745884_MERGED_FASTQ_SSU_OTU.tsv	Root-Environmental-Aquatic-Marine-Coastal-Sediment
SRR6745829	MGYA00202578	https://www.ebi.ac.uk/metagenomics/api/v1/analyses/MGYA00202578/file/SRR6745829_MERGED_FASTQ_SSU_OTU.tsv	Root-Environmental-Aquatic-Marine-Coastal-Sediment
SRR5097537	MGYA00360549	https://www.ebi.ac.uk/metagenomics/api/v1/analyses/MGYA00360549/file/SRR5097537_MERGED_FASTQ_SSU_OTU.tsv	Root-Environmental-Aquatic-Marine-Coastal-Sediment
SRR5957811	MGYA00365448	https://www.ebi.ac.uk/metagenomics/api/v1/analyses/MGYA00365448/file/SRR5957811_FASTQ_SSU_OTU.tsv	Root-Environmental-Aquatic-Marine-Coastal-Sediment
SRR5227107	MGYA00360666	https://www.ebi.ac.uk/metagenomics/api/v1/analyses/MGYA00360666/file/SRR5227107_FASTQ_SSU_OTU.tsv	Root-Environmental-Aquatic-Marine-Coastal-Sediment
SRR6745971	MGYA00202956	https://www.ebi.ac.uk/metagenomics/api/v1/analyses/MGYA00202956/file/SRR6745971_MERGED_FASTQ_SSU_OTU.tsv	Root-Environmental-Aquatic-Marine-Coastal-Sediment
SRR6746077	MGYA00203092	https://www.ebi.ac.uk/metagenomics/api/v1/analyses/MGYA00203092/file/SRR6746077_MERGED_FASTQ_SSU_OTU.tsv	Root-Environmental-Aquatic-Marine-Coastal-Sediment
SRR5097530	MGYA00360542	https://www.ebi.ac.uk/metagenomics/api/v1/analyses/MGYA00360542/file/SRR5097530_MERGED_FASTQ_SSU_OTU.tsv	Root-Environmental-Aquatic-Marine-Coastal-Sediment
SRR6746549	MGYA00202514	https://www.ebi.ac.uk/metagenomics/api/v1/analyses/MGYA00202514/file/SRR6746549_MERGED_FASTQ_SSU_OTU.tsv	Root-Environmental-Aquatic-Marine-Coastal-Sediment
SRR6746128	MGYA00203014	https://www.ebi.ac.uk/metagenomics/api/v1/analyses/MGYA00203014/file/SRR6746128_MERGED_FASTQ_SSU_OTU.tsv	Root-Environmental-Aquatic-Marine-Coastal-Sediment
SRR5957816	MGYA00365453	https://www.ebi.ac.uk/metagenomics/api/v1/analyses/MGYA00365453/file/SRR5957816_FASTQ_SSU_OTU.tsv	Root-Environmental-Aquatic-Marine-Coastal-Sediment

SRR6745976	MGYA00202599	https://www.ebi.ac.uk/metagenomics/api/v1/analyses/MGYA00202599/file/SRR6745976_MERGED_FASTQ_SSU_OTU.tsv	Root-Environmental-Aquatic-Marine-Coastal-Sediment
SRR6746070	MGYA00202598	https://www.ebi.ac.uk/metagenomics/api/v1/analyses/MGYA00202598/file/SRR6746070_MERGED_FASTQ_SSU_OTU.tsv	Root-Environmental-Aquatic-Marine-Coastal-Sediment
SRR6746535	MGYA00203028	https://www.ebi.ac.uk/metagenomics/api/v1/analyses/MGYA00203028/file/SRR6746535_MERGED_FASTQ_SSU_OTU.tsv	Root-Environmental-Aquatic-Marine-Coastal-Sediment
SRR6745852	MGYA00203071	https://www.ebi.ac.uk/metagenomics/api/v1/analyses/MGYA00203071/file/SRR6745852_MERGED_FASTQ_SSU_OTU.tsv	Root-Environmental-Aquatic-Marine-Coastal-Sediment
SRR6745420	MGYA00202562	https://www.ebi.ac.uk/metagenomics/api/v1/analyses/MGYA00202562/file/SRR6745420_MERGED_FASTQ_SSU_OTU.tsv	Root-Environmental-Aquatic-Marine-Coastal-Sediment
SRR6745387	MGYA00203009	https://www.ebi.ac.uk/metagenomics/api/v1/analyses/MGYA00203009/file/SRR6745387_MERGED_FASTQ_SSU_OTU.tsv	Root-Environmental-Aquatic-Marine-Coastal-Sediment
SRR6746154	MGYA00202931	https://www.ebi.ac.uk/metagenomics/api/v1/analyses/MGYA00202931/file/SRR6746154_MERGED_FASTQ_SSU_OTU.tsv	Root-Environmental-Aquatic-Marine-Coastal-Sediment
SRR2297160	MGYA00358924	https://www.ebi.ac.uk/metagenomics/api/v1/analyses/MGYA00358924/file/SRR2297160_MERGED_FASTQ_SSU_OTU.tsv	Root-Environmental-Aquatic-Marine-Coastal-Sediment
SRR6746185	MGYA00202998	https://www.ebi.ac.uk/metagenomics/api/v1/analyses/MGYA00202998/file/SRR6746185_MERGED_FASTQ_SSU_OTU.tsv	Root-Environmental-Aquatic-Marine-Coastal-Sediment
SRR1503982	MGYA00358537	https://www.ebi.ac.uk/metagenomics/api/v1/analyses/MGYA00358537/file/SRR1503982_FASTQ_SSU_OTU.tsv	Root-Environmental-Aquatic-Marine-Coastal-Sediment
SRR6745883	MGYA00203066	https://www.ebi.ac.uk/metagenomics/api/v1/analyses/MGYA00203066/file/SRR6745883_MERGED_FASTQ_SSU_OTU.tsv	Root-Environmental-Aquatic-Marine-Coastal-Sediment
SRR6745356	MGYA00202592	https://www.ebi.ac.uk/metagenomics/api/v1/analyses/MGYA00202592/file/SRR6745356_MERGED_FASTQ_SSU_OTU.tsv	Root-Environmental-Aquatic-Marine-Coastal-Sediment

SRR2856425	MGYA00359222	https://www.ebi.ac.uk/metagenomics/api/v1/analyses/MGYA00359222/file/SRR2856425_MERGED_FASTQ_SSU_OTU.tsv	Root-Environmental-Aquatic-Marine-Coastal-Sediment
SRR1257386	MGYA00356848	https://www.ebi.ac.uk/metagenomics/api/v1/analyses/MGYA00356848/file/SRR1257386_MERGED_FASTQ_SSU_OTU.tsv	Root-Environmental-Aquatic-Marine-Coastal-Sediment
SRR6745967	MGYA00202542	https://www.ebi.ac.uk/metagenomics/api/v1/analyses/MGYA00202542/file/SRR6745967_MERGED_FASTQ_SSU_OTU.tsv	Root-Environmental-Aquatic-Marine-Coastal-Sediment
SRR6746061	MGYA00203065	https://www.ebi.ac.uk/metagenomics/api/v1/analyses/MGYA00203065/file/SRR6746061_MERGED_FASTQ_SSU_OTU.tsv	Root-Environmental-Aquatic-Marine-Coastal-Sediment
SRR5227111	MGYA00360670	https://www.ebi.ac.uk/metagenomics/api/v1/analyses/MGYA00360670/file/SRR5227111_FASTQ_SSU_OTU.tsv	Root-Environmental-Aquatic-Marine-Coastal-Sediment
ERR330019	MGYA00357977	https://www.ebi.ac.uk/metagenomics/api/v1/analyses/MGYA00357977/file/ERR330019_FASTQ_SSU_OTU.tsv	Root-Environmental-Aquatic-Marine-Coastal-Sediment
SRR6746558	MGYA00203011	https://www.ebi.ac.uk/metagenomics/api/v1/analyses/MGYA00203011/file/SRR6746558_MERGED_FASTQ_SSU_OTU.tsv	Root-Environmental-Aquatic-Marine-Coastal-Sediment
SRR6746139	MGYA00202828	https://www.ebi.ac.uk/metagenomics/api/v1/analyses/MGYA00202828/file/SRR6746139_MERGED_FASTQ_SSU_OTU.tsv	Root-Environmental-Aquatic-Marine-Coastal-Sediment
SRR6746194	MGYA00202757	https://www.ebi.ac.uk/metagenomics/api/v1/analyses/MGYA00202757/file/SRR6746194_MERGED_FASTQ_SSU_OTU.tsv	Root-Environmental-Aquatic-Marine-Coastal-Sediment
SRR6745347	MGYA00202808	https://www.ebi.ac.uk/metagenomics/api/v1/analyses/MGYA00202808/file/SRR6745347_MERGED_FASTQ_SSU_OTU.tsv	Root-Environmental-Aquatic-Marine-Coastal-Sediment
SRR6745892	MGYA00202523	https://www.ebi.ac.uk/metagenomics/api/v1/analyses/MGYA00202523/file/SRR6745892_MERGED_FASTQ_SSU_OTU.tsv	Root-Environmental-Aquatic-Marine-Coastal-Sediment
SRR6745396	MGYA00202803	https://www.ebi.ac.uk/metagenomics/api/v1/analyses/MGYA00202803/file/SRR6745396_MERGED_FASTQ_SSU_OTU.tsv	Root-Environmental-Aquatic-Marine-Coastal-Sediment

SRR6745431	MGYA00202739	https://www.ebi.ac.uk/metagenomics/api/v1/analyses/MGYA00202739/file/SRR6745431_MERGED_FASTQ_SSU_OTU.tsv	Root-Environmental-Aquatic-Marine-Coastal-Sediment
SRR6745843	MGYA00202659	https://www.ebi.ac.uk/metagenomics/api/v1/analyses/MGYA00202659/file/SRR6745843_MERGED_FASTQ_SSU_OTU.tsv	Root-Environmental-Aquatic-Marine-Coastal-Sediment
SRR6746524	MGYA00202799	https://www.ebi.ac.uk/metagenomics/api/v1/analyses/MGYA00202799/file/SRR6746524_MERGED_FASTQ_SSU_OTU.tsv	Root-Environmental-Aquatic-Marine-Coastal-Sediment
SRR2297171	MGYA00358935	https://www.ebi.ac.uk/metagenomics/api/v1/analyses/MGYA00358935/file/SRR2297171_MERGED_FASTQ_SSU_OTU.tsv	Root-Environmental-Aquatic-Marine-Coastal-Sediment
SRR6746145	MGYA00202493	https://www.ebi.ac.uk/metagenomics/api/v1/analyses/MGYA00202493/file/SRR6746145_MERGED_FASTQ_SSU_OTU.tsv	Root-Environmental-Aquatic-Marine-Coastal-Sediment
SRR6746193	MGYA00202698	https://www.ebi.ac.uk/metagenomics/api/v1/analyses/MGYA00202698/file/SRR6746193_MERGED_FASTQ_SSU_OTU.tsv	Root-Environmental-Aquatic-Marine-Coastal-Sediment
SRR6745340	MGYA00202960	https://www.ebi.ac.uk/metagenomics/api/v1/analyses/MGYA00202960/file/SRR6745340_MERGED_FASTQ_SSU_OTU.tsv	Root-Environmental-Aquatic-Marine-Coastal-Sediment
SRR6745844	MGYA00202648	https://www.ebi.ac.uk/metagenomics/api/v1/analyses/MGYA00202648/file/SRR6745844_MERGED_FASTQ_SSU_OTU.tsv	Root-Environmental-Aquatic-Marine-Coastal-Sediment
SRR6745391	MGYA00202905	https://www.ebi.ac.uk/metagenomics/api/v1/analyses/MGYA00202905/file/SRR6745391_MERGED_FASTQ_SSU_OTU.tsv	Root-Environmental-Aquatic-Marine-Coastal-Sediment
SRR6746523	MGYA00202817	https://www.ebi.ac.uk/metagenomics/api/v1/analyses/MGYA00202817/file/SRR6746523_MERGED_FASTQ_SSU_OTU.tsv	Root-Environmental-Aquatic-Marine-Coastal-Sediment
SRR6746142	MGYA00202981	https://www.ebi.ac.uk/metagenomics/api/v1/analyses/MGYA00202981/file/SRR6746142_MERGED_FASTQ_SSU_OTU.tsv	Root-Environmental-Aquatic-Marine-Coastal-Sediment
SRR2297176	MGYA00358940	https://www.ebi.ac.uk/metagenomics/api/v1/analyses/MGYA00358940/file/SRR2297176_MERGED_FASTQ_SSU_OTU.tsv	Root-Environmental-Aquatic-Marine-Coastal-Sediment

SRR4300268	MGYA00360230	https://www.ebi.ac.uk/metagenomics/api/v1/analyses/MGYA00360230/file/SRR4300268_MERGED_FASTQ_SSU_OTU.tsv	Root-Environmental-Aquatic-Marine-Coastal-Sediment
SRR6745960	MGYA00203008	https://www.ebi.ac.uk/metagenomics/api/v1/analyses/MGYA00203008/file/SRR6745960_MERGED_FASTQ_SSU_OTU.tsv	Root-Environmental-Aquatic-Marine-Coastal-Sediment
SRR6746066	MGYA00202656	https://www.ebi.ac.uk/metagenomics/api/v1/analyses/MGYA00202656/file/SRR6746066_MERGED_FASTQ_SSU_OTU.tsv	Root-Environmental-Aquatic-Marine-Coastal-Sediment
SRR5227116	MGYA00360675	https://www.ebi.ac.uk/metagenomics/api/v1/analyses/MGYA00360675/file/SRR5227116_FASTQ_SSU_OTU.tsv	Root-Environmental-Aquatic-Marine-Coastal-Sediment
SRR6745838	MGYA00202867	https://www.ebi.ac.uk/metagenomics/api/v1/analyses/MGYA00202867/file/SRR6745838_MERGED_FASTQ_SSU_OTU.tsv	Root-Environmental-Aquatic-Marine-Coastal-Sediment
SRR6745912	MGYA00202744	https://www.ebi.ac.uk/metagenomics/api/v1/analyses/MGYA00202744/file/SRR6745912_MERGED_FASTQ_SSU_OTU.tsv	Root-Environmental-Aquatic-Marine-Coastal-Sediment
SRR6746475	MGYA00202838	https://www.ebi.ac.uk/metagenomics/api/v1/analyses/MGYA00202838/file/SRR6746475_MERGED_FASTQ_SSU_OTU.tsv	Root-Environmental-Aquatic-Marine-Coastal-Sediment
SRR6746014	MGYA00203048	https://www.ebi.ac.uk/metagenomics/api/v1/analyses/MGYA00203048/file/SRR6746014_MERGED_FASTQ_SSU_OTU.tsv	Root-Environmental-Aquatic-Marine-Coastal-Sediment
SRR3358603	MGYA00364491	https://www.ebi.ac.uk/metagenomics/api/v1/analyses/MGYA00364491/file/SRR3358603_FASTQ_SSU_OTU.tsv	Root-Environmental-Aquatic-Marine-Coastal-Sediment
SRR4300266	MGYA00360228	https://www.ebi.ac.uk/metagenomics/api/v1/analyses/MGYA00360228/file/SRR4300266_MERGED_FASTQ_SSU_OTU.tsv	Root-Environmental-Aquatic-Marine-Coastal-Sediment
SRR2297178	MGYA00358942	https://www.ebi.ac.uk/metagenomics/api/v1/analyses/MGYA00358942/file/SRR2297178_MERGED_FASTQ_SSU_OTU.tsv	Root-Environmental-Aquatic-Marine-Coastal-Sediment
SRR6746068	MGYA00202824	https://www.ebi.ac.uk/metagenomics/api/v1/analyses/MGYA00202824/file/SRR6746068_MERGED_FASTQ_SSU_OTU.tsv	Root-Environmental-Aquatic-Marine-Coastal-Sediment

SRR6745332	MGYA00202904	https://www.ebi.ac.uk/metagenomics/api/v1/analyses/MGYA00202904/file/SRR6745332_MERGED_FASTQ_SSU_OTU.tsv	Root-Environmental-Aquatic-Marine-Coastal-Sediment
SRR6745836	MGYA00202586	https://www.ebi.ac.uk/metagenomics/api/v1/analyses/MGYA00202586/file/SRR6745836_MERGED_FASTQ_SSU_OTU.tsv	Root-Environmental-Aquatic-Marine-Coastal-Sediment
SRR5097528	MGYA00360540	https://www.ebi.ac.uk/metagenomics/api/v1/analyses/MGYA00360540/file/SRR5097528_MERGED_FASTQ_SSU_OTU.tsv	Root-Environmental-Aquatic-Marine-Coastal-Sediment
SRR6746551	MGYA00202725	https://www.ebi.ac.uk/metagenomics/api/v1/analyses/MGYA00202725/file/SRR6746551_MERGED_FASTQ_SSU_OTU.tsv	Root-Environmental-Aquatic-Marine-Coastal-Sediment
SRR2297104	MGYA00358868	https://www.ebi.ac.uk/metagenomics/api/v1/analyses/MGYA00358868/file/SRR2297104_MERGED_FASTQ_SSU_OTU.tsv	Root-Environmental-Aquatic-Marine-Coastal-Sediment
SRR6746130	MGYA00203116	https://www.ebi.ac.uk/metagenomics/api/v1/analyses/MGYA00203116/file/SRR6746130_MERGED_FASTQ_SSU_OTU.tsv	Root-Environmental-Aquatic-Marine-Coastal-Sediment
SRR6745969	MGYA00202862	https://www.ebi.ac.uk/metagenomics/api/v1/analyses/MGYA00202862/file/SRR6745969_MERGED_FASTQ_SSU_OTU.tsv	Root-Environmental-Aquatic-Marine-Coastal-Sediment
SRR2856446	MGYA00359224	https://www.ebi.ac.uk/metagenomics/api/v1/analyses/MGYA00359224/file/SRR2856446_MERGED_FASTQ_SSU_OTU.tsv	Root-Environmental-Aquatic-Marine-Coastal-Sediment
SRR6745335	MGYA00202743	https://www.ebi.ac.uk/metagenomics/api/v1/analyses/MGYA00202743/file/SRR6745335_MERGED_FASTQ_SSU_OTU.tsv	Root-Environmental-Aquatic-Marine-Coastal-Sediment
SRR6745831	MGYA00202711	https://www.ebi.ac.uk/metagenomics/api/v1/analyses/MGYA00202711/file/SRR6745831_MERGED_FASTQ_SSU_OTU.tsv	Root-Environmental-Aquatic-Marine-Coastal-Sediment
SRR6746556	MGYA00202671	https://www.ebi.ac.uk/metagenomics/api/v1/analyses/MGYA00202671/file/SRR6746556_MERGED_FASTQ_SSU_OTU.tsv	Root-Environmental-Aquatic-Marine-Coastal-Sediment
SRR3355072	MGYA00359644	https://www.ebi.ac.uk/metagenomics/api/v1/analyses/MGYA00359644/file/SRR3355072_MERGED_FASTQ_SSU_OTU.tsv	Root-Environmental-Aquatic-Marine-Coastal-Sediment

SRR6746137	MGYA00203005	https://www.ebi.ac.uk/metagenomics/api/v1/analyses/MGYA00203005/file/SRR6746137_MERGED_FASTQ_SSU_OTU.tsv	Root-Environmental-Aquatic-Marine-Coastal-Sediment
SRR2297103	MGYA00358867	https://www.ebi.ac.uk/metagenomics/api/v1/analyses/MGYA00358867/file/SRR2297103_MERGED_FASTQ_SSU_OTU.tsv	Root-Environmental-Aquatic-Marine-Coastal-Sediment
SRR6745915	MGYA00202916	https://www.ebi.ac.uk/metagenomics/api/v1/analyses/MGYA00202916/file/SRR6745915_MERGED_FASTQ_SSU_OTU.tsv	Root-Environmental-Aquatic-Marine-Coastal-Sediment
SRR6746472	MGYA00202701	https://www.ebi.ac.uk/metagenomics/api/v1/analyses/MGYA00202701/file/SRR6746472_MERGED_FASTQ_SSU_OTU.tsv	Root-Environmental-Aquatic-Marine-Coastal-Sediment
SRR6746013	MGYA00203078	https://www.ebi.ac.uk/metagenomics/api/v1/analyses/MGYA00203078/file/SRR6746013_MERGED_FASTQ_SSU_OTU.tsv	Root-Environmental-Aquatic-Marine-Coastal-Sediment
SRR6745349	MGYA00203114	https://www.ebi.ac.uk/metagenomics/api/v1/analyses/MGYA00203114/file/SRR6745349_MERGED_FASTQ_SSU_OTU.tsv	Root-Environmental-Aquatic-Marine-Coastal-Sediment
SRR6745398	MGYA00202780	https://www.ebi.ac.uk/metagenomics/api/v1/analyses/MGYA00202780/file/SRR6745398_MERGED_FASTQ_SSU_OTU.tsv	Root-Environmental-Aquatic-Marine-Coastal-Sediment
SRR4436293	MGYA00213049	https://www.ebi.ac.uk/metagenomics/api/v1/analyses/MGYA00213049/file/SRR4436293_FASTQ_SSU_OTU.tsv	Root-Environmental-Aquatic-Marine-Coastal-Sediment
SRR2297136	MGYA00358900	https://www.ebi.ac.uk/metagenomics/api/v1/analyses/MGYA00358900/file/SRR2297136_MERGED_FASTQ_SSU_OTU.tsv	Root-Environmental-Aquatic-Marine-Coastal-Sediment
SRR6746102	MGYA00203117	https://www.ebi.ac.uk/metagenomics/api/v1/analyses/MGYA00203117/file/SRR6746102_MERGED_FASTQ_SSU_OTU.tsv	Root-Environmental-Aquatic-Marine-Coastal-Sediment
SRR6745804	MGYA00202772	https://www.ebi.ac.uk/metagenomics/api/v1/analyses/MGYA00202772/file/SRR6745804_MERGED_FASTQ_SSU_OTU.tsv	Root-Environmental-Aquatic-Marine-Coastal-Sediment
SRR6746563	MGYA00202895	https://www.ebi.ac.uk/metagenomics/api/v1/analyses/MGYA00202895/file/SRR6746563_MERGED_FASTQ_SSU_OTU.tsv	Root-Environmental-Aquatic-Marine-Coastal-Sediment

ERR330022	MGYA00357980	https://www.ebi.ac.uk/metagenomics/api/v1/analyses/MGYA00357980/file/ERR330022_FASTQ_SSU_OTU.tsv	Root-Environmental-Aquatic-Marine-Coastal-Sediment
SRR6746215	MGYA00203024	https://www.ebi.ac.uk/metagenomics/api/v1/analyses/MGYA00203024/file/SRR6746215_MERGED_FASTQ_SSU_OTU.tsv	Root-Environmental-Aquatic-Marine-Coastal-Sediment
SRR6172351	MGYA00362313	https://www.ebi.ac.uk/metagenomics/api/v1/analyses/MGYA00362313/file/SRR6172351_MERGED_FASTQ_SSU_OTU.tsv	Root-Environmental-Aquatic-Marine-Coastal-Sediment
SRR6746496	MGYA00203067	https://www.ebi.ac.uk/metagenomics/api/v1/analyses/MGYA00203067/file/SRR6746496_MERGED_FASTQ_SSU_OTU.tsv	Root-Environmental-Aquatic-Marine-Coastal-Sediment
SRR2712918	MGYA00355956	https://www.ebi.ac.uk/metagenomics/api/v1/analyses/MGYA00355956/file/SRR2712918_FASTQ_SSU_OTU.tsv	Root-Environmental-Aquatic-Marine-Coastal-Sediment
SRR6746026	MGYA00202908	https://www.ebi.ac.uk/metagenomics/api/v1/analyses/MGYA00202908/file/SRR6746026_MERGED_FASTQ_SSU_OTU.tsv	Root-Environmental-Aquatic-Marine-Coastal-Sediment
SRR6745920	MGYA00202756	https://www.ebi.ac.uk/metagenomics/api/v1/analyses/MGYA00202756/file/SRR6745920_MERGED_FASTQ_SSU_OTU.tsv	Root-Environmental-Aquatic-Marine-Coastal-Sediment
SRR6745878	MGYA00203094	https://www.ebi.ac.uk/metagenomics/api/v1/analyses/MGYA00203094/file/SRR6745878_MERGED_FASTQ_SSU_OTU.tsv	Root-Environmental-Aquatic-Marine-Coastal-Sediment
SRR1503979	MGYA00358534	https://www.ebi.ac.uk/metagenomics/api/v1/analyses/MGYA00358534/file/SRR1503979_FASTQ_SSU_OTU.tsv	Root-Environmental-Aquatic-Marine-Coastal-Sediment
SRR4300285	MGYA00360245	https://www.ebi.ac.uk/metagenomics/api/v1/analyses/MGYA00360245/file/SRR4300285_MERGED_FASTQ_SSU_OTU.tsv	Root-Environmental-Aquatic-Marine-Coastal-Sediment
SRR6517611	MGYA00362476	https://www.ebi.ac.uk/metagenomics/api/v1/analyses/MGYA00362476/file/SRR6517611_MERGED_FASTQ_SSU_OTU.tsv	Root-Environmental-Aquatic-Marine-Coastal-Sediment
SRR6746491	MGYA00202805	https://www.ebi.ac.uk/metagenomics/api/v1/analyses/MGYA00202805/file/SRR6746491_MERGED_FASTQ_SSU_OTU.tsv	Root-Environmental-Aquatic-Marine-Coastal-Sediment

SRR6746021	MGYA00202596	https://www.ebi.ac.uk/metagenomics/api/v1/analyses/MGYA00202596/file/SRR6746021_MERGED_FASTQ_SSU_OTU.tsv	Root-Environmental-Aquatic-Marine-Coastal-Sediment
SRR6745927	MGYA00202703	https://www.ebi.ac.uk/metagenomics/api/v1/analyses/MGYA00202703/file/SRR6745927_MERGED_FASTQ_SSU_OTU.tsv	Root-Environmental-Aquatic-Marine-Coastal-Sediment
SRR6746179	MGYA00202763	https://www.ebi.ac.uk/metagenomics/api/v1/analyses/MGYA00202763/file/SRR6746179_MERGED_FASTQ_SSU_OTU.tsv	Root-Environmental-Aquatic-Marine-Coastal-Sediment
SRR6746518	MGYA00202942	https://www.ebi.ac.uk/metagenomics/api/v1/analyses/MGYA00202942/file/SRR6746518_MERGED_FASTQ_SSU_OTU.tsv	Root-Environmental-Aquatic-Marine-Coastal-Sediment
SRR4300282	MGYA00360243	https://www.ebi.ac.uk/metagenomics/api/v1/analyses/MGYA00360243/file/SRR4300282_MERGED_FASTQ_SSU_OTU.tsv	Root-Environmental-Aquatic-Marine-Coastal-Sediment
SRR4436294	MGYA00213045	https://www.ebi.ac.uk/metagenomics/api/v1/analyses/MGYA00213045/file/SRR4436294_FASTQ_SSU_OTU.tsv	Root-Environmental-Aquatic-Marine-Coastal-Sediment
SRR6746105	MGYA00202715	https://www.ebi.ac.uk/metagenomics/api/v1/analyses/MGYA00202715/file/SRR6746105_MERGED_FASTQ_SSU_OTU.tsv	Root-Environmental-Aquatic-Marine-Coastal-Sediment
SRR2297131	MGYA00358895	https://www.ebi.ac.uk/metagenomics/api/v1/analyses/MGYA00358895/file/SRR2297131_MERGED_FASTQ_SSU_OTU.tsv	Root-Environmental-Aquatic-Marine-Coastal-Sediment
SRR6745803	MGYA00202924	https://www.ebi.ac.uk/metagenomics/api/v1/analyses/MGYA00202924/file/SRR6745803_MERGED_FASTQ_SSU_OTU.tsv	Root-Environmental-Aquatic-Marine-Coastal-Sediment
SRR6746564	MGYA00202516	https://www.ebi.ac.uk/metagenomics/api/v1/analyses/MGYA00202516/file/SRR6746564_MERGED_FASTQ_SSU_OTU.tsv	Root-Environmental-Aquatic-Marine-Coastal-Sediment
ERR330025	MGYA00357983	https://www.ebi.ac.uk/metagenomics/api/v1/analyses/MGYA00357983/file/ERR330025_FASTQ_SSU_OTU.tsv	Root-Environmental-Aquatic-Marine-Coastal-Sediment
SRR6746212	MGYA00202982	https://www.ebi.ac.uk/metagenomics/api/v1/analyses/MGYA00202982/file/SRR6746212_MERGED_FASTQ_SSU_OTU.tsv	Root-Environmental-Aquatic-Marine-Coastal-Sediment

SRR6172356	MGYA00362318	https://www.ebi.ac.uk/metagenomics/api/v1/analyses/MGYA00362318/file/SRR6172356_MERGED_FASTQ_SSU_OTU.tsv	Root-Environmental-Aquatic-Marine-Coastal-Sediment
SRR1258342	MGYA00358465	https://www.ebi.ac.uk/metagenomics/api/v1/analyses/MGYA00358465/file/SRR1258342_FASTQ_SSU_OTU.tsv	Root-Environmental-Aquatic-Marine-Coastal-Sediment
SRR6745929	MGYA00202593	https://www.ebi.ac.uk/metagenomics/api/v1/analyses/MGYA00202593/file/SRR6745929_MERGED_FASTQ_SSU_OTU.tsv	Root-Environmental-Aquatic-Marine-Coastal-Sediment
SRR2297143	MGYA00358907	https://www.ebi.ac.uk/metagenomics/api/v1/analyses/MGYA00358907/file/SRR2297143_MERGED_FASTQ_SSU_OTU.tsv	Root-Environmental-Aquatic-Marine-Coastal-Sediment
SRR6746177	MGYA00202702	https://www.ebi.ac.uk/metagenomics/api/v1/analyses/MGYA00202702/file/SRR6746177_MERGED_FASTQ_SSU_OTU.tsv	Root-Environmental-Aquatic-Marine-Coastal-Sediment
SRR6745871	MGYA00202538	https://www.ebi.ac.uk/metagenomics/api/v1/analyses/MGYA00202538/file/SRR6745871_MERGED_FASTQ_SSU_OTU.tsv	Root-Environmental-Aquatic-Marine-Coastal-Sediment
SRR6745403	MGYA00202751	https://www.ebi.ac.uk/metagenomics/api/v1/analyses/MGYA00202751/file/SRR6745403_MERGED_FASTQ_SSU_OTU.tsv	Root-Environmental-Aquatic-Marine-Coastal-Sediment
SRR6746516	MGYA00202594	https://www.ebi.ac.uk/metagenomics/api/v1/analyses/MGYA00202594/file/SRR6746516_MERGED_FASTQ_SSU_OTU.tsv	Root-Environmental-Aquatic-Marine-Coastal-Sediment
ERR330057	MGYA00358015	https://www.ebi.ac.uk/metagenomics/api/v1/analyses/MGYA00358015/file/ERR330057_FASTQ_SSU_OTU.tsv	Root-Environmental-Aquatic-Marine-Coastal-Sediment
SRR6745375	MGYA00202537	https://www.ebi.ac.uk/metagenomics/api/v1/analyses/MGYA00202537/file/SRR6745375_MERGED_FASTQ_SSU_OTU.tsv	Root-Environmental-Aquatic-Marine-Coastal-Sediment
SRR6745984	MGYA00202918	https://www.ebi.ac.uk/metagenomics/api/v1/analyses/MGYA00202918/file/SRR6745984_MERGED_FASTQ_SSU_OTU.tsv	Root-Environmental-Aquatic-Marine-Coastal-Sediment
SRR6746082	MGYA00202645	https://www.ebi.ac.uk/metagenomics/api/v1/analyses/MGYA00202645/file/SRR6746082_MERGED_FASTQ_SSU_OTU.tsv	Root-Environmental-Aquatic-Marine-Coastal-Sediment

SRR6746053	MGYA00202975	https://www.ebi.ac.uk/metagenomics/api/v1/analyses/MGYA00202975/file/SRR6746053_MERGED_FASTQ_SSU_OTU.tsv	Root-Environmental-Aquatic-Marine-Coastal-Sediment
SRR6745955	MGYA00202697	https://www.ebi.ac.uk/metagenomics/api/v1/analyses/MGYA00202697/file/SRR6745955_MERGED_FASTQ_SSU_OTU.tsv	Root-Environmental-Aquatic-Marine-Coastal-Sediment
SRR6172358	MGYA00362320	https://www.ebi.ac.uk/metagenomics/api/v1/analyses/MGYA00362320/file/SRR6172358_MERGED_FASTQ_SSU_OTU.tsv	Root-Environmental-Aquatic-Marine-Coastal-Sediment
SRR6745983	MGYA00202573	https://www.ebi.ac.uk/metagenomics/api/v1/analyses/MGYA00202573/file/SRR6745983_MERGED_FASTQ_SSU_OTU.tsv	Root-Environmental-Aquatic-Marine-Coastal-Sediment
SRR6746085	MGYA00202826	https://www.ebi.ac.uk/metagenomics/api/v1/analyses/MGYA00202826/file/SRR6746085_MERGED_FASTQ_SSU_OTU.tsv	Root-Environmental-Aquatic-Marine-Coastal-Sediment
SRR6746054	MGYA00202801	https://www.ebi.ac.uk/metagenomics/api/v1/analyses/MGYA00202801/file/SRR6746054_MERGED_FASTQ_SSU_OTU.tsv	Root-Environmental-Aquatic-Marine-Coastal-Sediment
SRR6745952	MGYA00202961	https://www.ebi.ac.uk/metagenomics/api/v1/analyses/MGYA00202961/file/SRR6745952_MERGED_FASTQ_SSU_OTU.tsv	Root-Environmental-Aquatic-Marine-Coastal-Sediment
SRR2297138	MGYA00358902	https://www.ebi.ac.uk/metagenomics/api/v1/analyses/MGYA00358902/file/SRR2297138_MERGED_FASTQ_SSU_OTU.tsv	Root-Environmental-Aquatic-Marine-Coastal-Sediment
SRR2855893	MGYA00359215	https://www.ebi.ac.uk/metagenomics/api/v1/analyses/MGYA00359215/file/SRR2855893_MERGED_FASTQ_SSU_OTU.tsv	Root-Environmental-Aquatic-Marine-Coastal-Sediment
SRR6746498	MGYA00202964	https://www.ebi.ac.uk/metagenomics/api/v1/analyses/MGYA00202964/file/SRR6746498_MERGED_FASTQ_SSU_OTU.tsv	Root-Environmental-Aquatic-Marine-Coastal-Sediment
SRR4300302	MGYA00360261	https://www.ebi.ac.uk/metagenomics/api/v1/analyses/MGYA00360261/file/SRR4300302_MERGED_FASTQ_SSU_OTU.tsv	Root-Environmental-Aquatic-Marine-Coastal-Sediment
SRR6746028	MGYA00202979	https://www.ebi.ac.uk/metagenomics/api/v1/analyses/MGYA00202979/file/SRR6746028_MERGED_FASTQ_SSU_OTU.tsv	Root-Environmental-Aquatic-Marine-Coastal-Sediment

SRR1258345	MGYA00358468	https://www.ebi.ac.uk/metagenomics/api/v1/analyses/MGYA00358468/file/SRR1258345_FASTQ_SSU_OTU.tsv	Root-Environmental-Aquatic-Marine-Coastal-Sediment
SRR6746170	MGYA00202841	https://www.ebi.ac.uk/metagenomics/api/v1/analyses/MGYA00202841/file/SRR6746170_MERGED_FASTQ_SSU_OTU.tsv	Root-Environmental-Aquatic-Marine-Coastal-Sediment
SRR2297144	MGYA00358908	https://www.ebi.ac.uk/metagenomics/api/v1/analyses/MGYA00358908/file/SRR2297144_MERGED_FASTQ_SSU_OTU.tsv	Root-Environmental-Aquatic-Marine-Coastal-Sediment
SRR6745404	MGYA00202766	https://www.ebi.ac.uk/metagenomics/api/v1/analyses/MGYA00202766/file/SRR6745404_MERGED_FASTQ_SSU_OTU.tsv	Root-Environmental-Aquatic-Marine-Coastal-Sediment
SRR6745876	MGYA00202771	https://www.ebi.ac.uk/metagenomics/api/v1/analyses/MGYA00202771/file/SRR6745876_MERGED_FASTQ_SSU_OTU.tsv	Root-Environmental-Aquatic-Marine-Coastal-Sediment
SRR1503977	MGYA00358532	https://www.ebi.ac.uk/metagenomics/api/v1/analyses/MGYA00358532/file/SRR1503977_FASTQ_SSU_OTU.tsv	Root-Environmental-Aquatic-Marine-Coastal-Sediment
SRR6746511	MGYA00202541	https://www.ebi.ac.uk/metagenomics/api/v1/analyses/MGYA00202541/file/SRR6746511_MERGED_FASTQ_SSU_OTU.tsv	Root-Environmental-Aquatic-Marine-Coastal-Sediment
ERR330050	MGYA00358008	https://www.ebi.ac.uk/metagenomics/api/v1/analyses/MGYA00358008/file/ERR330050_FASTQ_SSU_OTU.tsv	Root-Environmental-Aquatic-Marine-Coastal-Sediment
SRR6745372	MGYA00203010	https://www.ebi.ac.uk/metagenomics/api/v1/analyses/MGYA00203010/file/SRR6745372_MERGED_FASTQ_SSU_OTU.tsv	Root-Environmental-Aquatic-Marine-Coastal-Sediment
SRR6746019	MGYA00202609	https://www.ebi.ac.uk/metagenomics/api/v1/analyses/MGYA00202609/file/SRR6746019_MERGED_FASTQ_SSU_OTU.tsv	Root-Environmental-Aquatic-Marine-Coastal-Sediment
SRR6746478	MGYA00202600	https://www.ebi.ac.uk/metagenomics/api/v1/analyses/MGYA00202600/file/SRR6746478_MERGED_FASTQ_SSU_OTU.tsv	Root-Environmental-Aquatic-Marine-Coastal-Sediment
SRR6745343	MGYA00202807	https://www.ebi.ac.uk/metagenomics/api/v1/analyses/MGYA00202807/file/SRR6745343_MERGED_FASTQ_SSU_OTU.tsv	Root-Environmental-Aquatic-Marine-Coastal-Sediment

SRR6746190	MGYA00202746	https://www.ebi.ac.uk/metagenomics/api/v1/analyses/MGYA00202746/file/SRR6746190_MERGED_FASTQ_SSU_OTU.tsv	Root-Environmental-Aquatic-Marine-Coastal-Sediment
SRR6746141	MGYA00202903	https://www.ebi.ac.uk/metagenomics/api/v1/analyses/MGYA00202903/file/SRR6746141_MERGED_FASTQ_SSU_OTU.tsv	Root-Environmental-Aquatic-Marine-Coastal-Sediment
SRR2297175	MGYA00358939	https://www.ebi.ac.uk/metagenomics/api/v1/analyses/MGYA00358939/file/SRR2297175_MERGED_FASTQ_SSU_OTU.tsv	Root-Environmental-Aquatic-Marine-Coastal-Sediment
SRR6746520	MGYA00202758	https://www.ebi.ac.uk/metagenomics/api/v1/analyses/MGYA00202758/file/SRR6746520_MERGED_FASTQ_SSU_OTU.tsv	Root-Environmental-Aquatic-Marine-Coastal-Sediment
SRR6745435	MGYA00202689	https://www.ebi.ac.uk/metagenomics/api/v1/analyses/MGYA00202689/file/SRR6745435_MERGED_FASTQ_SSU_OTU.tsv	Root-Environmental-Aquatic-Marine-Coastal-Sediment
SRR6745847	MGYA00202997	https://www.ebi.ac.uk/metagenomics/api/v1/analyses/MGYA00202997/file/SRR6745847_MERGED_FASTQ_SSU_OTU.tsv	Root-Environmental-Aquatic-Marine-Coastal-Sediment
SRR6745392	MGYA00203046	https://www.ebi.ac.uk/metagenomics/api/v1/analyses/MGYA00203046/file/SRR6745392_MERGED_FASTQ_SSU_OTU.tsv	Root-Environmental-Aquatic-Marine-Coastal-Sediment
SRR6746065	MGYA00202995	https://www.ebi.ac.uk/metagenomics/api/v1/analyses/MGYA00202995/file/SRR6746065_MERGED_FASTQ_SSU_OTU.tsv	Root-Environmental-Aquatic-Marine-Coastal-Sediment
SRR6745963	MGYA00203112	https://www.ebi.ac.uk/metagenomics/api/v1/analyses/MGYA00203112/file/SRR6745963_MERGED_FASTQ_SSU_OTU.tsv	Root-Environmental-Aquatic-Marine-Coastal-Sediment
SRR5227115	MGYA00360674	https://www.ebi.ac.uk/metagenomics/api/v1/analyses/MGYA00360674/file/SRR5227115_FASTQ_SSU_OTU.tsv	Root-Environmental-Aquatic-Marine-Coastal-Sediment
SRR3355078	MGYA00359647	https://www.ebi.ac.uk/metagenomics/api/v1/analyses/MGYA00359647/file/SRR3355078_MERGED_FASTQ_SSU_OTU.tsv	Root-Environmental-Aquatic-Marine-Coastal-Sediment
SRR2297109	MGYA00358873	https://www.ebi.ac.uk/metagenomics/api/v1/analyses/MGYA00358873/file/SRR2297109_MERGED_FASTQ_SSU_OTU.tsv	Root-Environmental-Aquatic-Marine-Coastal-Sediment

SRR6746062	MGYA00202552	https://www.ebi.ac.uk/metagenomics/api/v1/analyses/MGYA00202552/file/SRR6746062_MERGED_FASTQ_SSU_OTU.tsv	Root-Environmental-Aquatic-Marine-Coastal-Sediment
SRR6745964	MGYA00202549	https://www.ebi.ac.uk/metagenomics/api/v1/analyses/MGYA00202549/file/SRR6745964_MERGED_FASTQ_SSU_OTU.tsv	Root-Environmental-Aquatic-Marine-Coastal-Sediment
SRR6745338	MGYA00202499	https://www.ebi.ac.uk/metagenomics/api/v1/analyses/MGYA00202499/file/SRR6745338_MERGED_FASTQ_SSU_OTU.tsv	Root-Environmental-Aquatic-Marine-Coastal-Sediment
SRR5227112	MGYA00360671	https://www.ebi.ac.uk/metagenomics/api/v1/analyses/MGYA00360671/file/SRR5227112_FASTQ_SSU_OTU.tsv	Root-Environmental-Aquatic-Marine-Coastal-Sediment
SRR6745788	MGYA00202775	https://www.ebi.ac.uk/metagenomics/api/v1/analyses/MGYA00202775/file/SRR6745788_MERGED_FASTQ_SSU_OTU.tsv	Root-Environmental-Aquatic-Marine-Coastal-Sediment
SRR2712920	MGYA00355958	https://www.ebi.ac.uk/metagenomics/api/v1/analyses/MGYA00355958/file/SRR2712920_FASTQ_SSU_OTU.tsv	Root-Environmental-Aquatic-Marine-Coastal-Sediment
SRR6745918	MGYA00202669	https://www.ebi.ac.uk/metagenomics/api/v1/analyses/MGYA00202669/file/SRR6745918_MERGED_FASTQ_SSU_OTU.tsv	Root-Environmental-Aquatic-Marine-Coastal-Sediment
SRR6745344	MGYA00203060	https://www.ebi.ac.uk/metagenomics/api/v1/analyses/MGYA00203060/file/SRR6745344_MERGED_FASTQ_SSU_OTU.tsv	Root-Environmental-Aquatic-Marine-Coastal-Sediment
SRR6745891	MGYA00202551	https://www.ebi.ac.uk/metagenomics/api/v1/analyses/MGYA00202551/file/SRR6745891_MERGED_FASTQ_SSU_OTU.tsv	Root-Environmental-Aquatic-Marine-Coastal-Sediment
SRR6746197	MGYA00202496	https://www.ebi.ac.uk/metagenomics/api/v1/analyses/MGYA00202496/file/SRR6746197_MERGED_FASTQ_SSU_OTU.tsv	Root-Environmental-Aquatic-Marine-Coastal-Sediment
SRR2297172	MGYA00358936	https://www.ebi.ac.uk/metagenomics/api/v1/analyses/MGYA00358936/file/SRR2297172_MERGED_FASTQ_SSU_OTU.tsv	Root-Environmental-Aquatic-Marine-Coastal-Sediment
SRR6746146	MGYA00202889	https://www.ebi.ac.uk/metagenomics/api/v1/analyses/MGYA00202889/file/SRR6746146_MERGED_FASTQ_SSU_OTU.tsv	Root-Environmental-Aquatic-Marine-Coastal-Sediment

SRR6746527	MGYA00202951	https://www.ebi.ac.uk/metagenomics/api/v1/analyses/MGYA00202951/file/SRR6746527_MERGED_FASTQ_SSU_OTU.tsv	Root-Environmental-Aquatic-Marine-Coastal-Sediment
SRR6745395	MGYA00202695	https://www.ebi.ac.uk/metagenomics/api/v1/analyses/MGYA00202695/file/SRR6745395_MERGED_FASTQ_SSU_OTU.tsv	Root-Environmental-Aquatic-Marine-Coastal-Sediment
SRR6745840	MGYA00203120	https://www.ebi.ac.uk/metagenomics/api/v1/analyses/MGYA00203120/file/SRR6745840_MERGED_FASTQ_SSU_OTU.tsv	Root-Environmental-Aquatic-Marine-Coastal-Sediment
SRR6745432	MGYA00203020	https://www.ebi.ac.uk/metagenomics/api/v1/analyses/MGYA00203020/file/SRR6745432_MERGED_FASTQ_SSU_OTU.tsv	Root-Environmental-Aquatic-Marine-Coastal-Sediment
SRR6745336	MGYA00202863	https://www.ebi.ac.uk/metagenomics/api/v1/analyses/MGYA00202863/file/SRR6745336_MERGED_FASTQ_SSU_OTU.tsv	Root-Environmental-Aquatic-Marine-Coastal-Sediment
SRR6746134	MGYA00202992	https://www.ebi.ac.uk/metagenomics/api/v1/analyses/MGYA00202992/file/SRR6746134_MERGED_FASTQ_SSU_OTU.tsv	Root-Environmental-Aquatic-Marine-Coastal-Sediment
SRR6746555	MGYA00202705	https://www.ebi.ac.uk/metagenomics/api/v1/analyses/MGYA00202705/file/SRR6746555_MERGED_FASTQ_SSU_OTU.tsv	Root-Environmental-Aquatic-Marine-Coastal-Sediment
SRR6745832	MGYA00202673	https://www.ebi.ac.uk/metagenomics/api/v1/analyses/MGYA00202673/file/SRR6745832_MERGED_FASTQ_SSU_OTU.tsv	Root-Environmental-Aquatic-Marine-Coastal-Sediment
SRR6746010	MGYA00202976	https://www.ebi.ac.uk/metagenomics/api/v1/analyses/MGYA00202976/file/SRR6746010_MERGED_FASTQ_SSU_OTU.tsv	Root-Environmental-Aquatic-Marine-Coastal-Sediment
SRR6746471	MGYA00202892	https://www.ebi.ac.uk/metagenomics/api/v1/analyses/MGYA00202892/file/SRR6746471_MERGED_FASTQ_SSU_OTU.tsv	Root-Environmental-Aquatic-Marine-Coastal-Sediment
SRR6745916	MGYA00202639	https://www.ebi.ac.uk/metagenomics/api/v1/analyses/MGYA00202639/file/SRR6745916_MERGED_FASTQ_SSU_OTU.tsv	Root-Environmental-Aquatic-Marine-Coastal-Sediment
SRR6746199	MGYA00202754	https://www.ebi.ac.uk/metagenomics/api/v1/analyses/MGYA00202754/file/SRR6746199_MERGED_FASTQ_SSU_OTU.tsv	Root-Environmental-Aquatic-Marine-Coastal-Sediment

SRR6746148	MGYA00202946	https://www.ebi.ac.uk/metagenomics/api/v1/analyses/MGYA00202946/file/SRR6746148_MERGED_FASTQ_SSU_OTU.tsv	Root-Environmental-Aquatic-Marine-Coastal-Sediment
SRR6746529	MGYA00203035	https://www.ebi.ac.uk/metagenomics/api/v1/analyses/MGYA00203035/file/SRR6746529_MERGED_FASTQ_SSU_OTU.tsv	Root-Environmental-Aquatic-Marine-Coastal-Sediment
SRR6746017	MGYA00202507	https://www.ebi.ac.uk/metagenomics/api/v1/analyses/MGYA00202507/file/SRR6746017_MERGED_FASTQ_SSU_OTU.tsv	Root-Environmental-Aquatic-Marine-Coastal-Sediment
SRR6746476	MGYA00202591	https://www.ebi.ac.uk/metagenomics/api/v1/analyses/MGYA00202591/file/SRR6746476_MERGED_FASTQ_SSU_OTU.tsv	Root-Environmental-Aquatic-Marine-Coastal-Sediment
SRR6745911	MGYA00202566	https://www.ebi.ac.uk/metagenomics/api/v1/analyses/MGYA00202566/file/SRR6745911_MERGED_FASTQ_SSU_OTU.tsv	Root-Environmental-Aquatic-Marine-Coastal-Sediment
SRR4300265	MGYA00360227	https://www.ebi.ac.uk/metagenomics/api/v1/analyses/MGYA00360227/file/SRR4300265_MERGED_FASTQ_SSU_OTU.tsv	Root-Environmental-Aquatic-Marine-Coastal-Sediment
SRR6745849	MGYA00202569	https://www.ebi.ac.uk/metagenomics/api/v1/analyses/MGYA00202569/file/SRR6745849_MERGED_FASTQ_SSU_OTU.tsv	Root-Environmental-Aquatic-Marine-Coastal-Sediment
SRR2855305	MGYA00359213	https://www.ebi.ac.uk/metagenomics/api/v1/analyses/MGYA00359213/file/SRR2855305_MERGED_FASTQ_SSU_OTU.tsv	Root-Environmental-Aquatic-Marine-Coastal-Sediment
SRR6745331	MGYA00202506	https://www.ebi.ac.uk/metagenomics/api/v1/analyses/MGYA00202506/file/SRR6745331_MERGED_FASTQ_SSU_OTU.tsv	Root-Environmental-Aquatic-Marine-Coastal-Sediment
SRR6172360	MGYA00362322	https://www.ebi.ac.uk/metagenomics/api/v1/analyses/MGYA00362322/file/SRR6172360_MERGED_FASTQ_SSU_OTU.tsv	Root-Environmental-Aquatic-Marine-Coastal-Sediment
SRR2297107	MGYA00358871	https://www.ebi.ac.uk/metagenomics/api/v1/analyses/MGYA00358871/file/SRR2297107_MERGED_FASTQ_SSU_OTU.tsv	Root-Environmental-Aquatic-Marine-Coastal-Sediment
SRR6746133	MGYA00202676	https://www.ebi.ac.uk/metagenomics/api/v1/analyses/MGYA00202676/file/SRR6746133_MERGED_FASTQ_SSU_OTU.tsv	Root-Environmental-Aquatic-Marine-Coastal-Sediment

SRR3355076	MGYA00359646	https://www.ebi.ac.uk/metagenomics/api/v1/analyses/MGYA00359646/file/SRR3355076_MERGED_FASTQ_SSU_OTU.tsv	Root-Environmental-Aquatic-Marine-Coastal-Sediment
SRR6746552	MGYA00202909	https://www.ebi.ac.uk/metagenomics/api/v1/analyses/MGYA00202909/file/SRR6746552_MERGED_FASTQ_SSU_OTU.tsv	Root-Environmental-Aquatic-Marine-Coastal-Sediment
SRR6745835	MGYA00202816	https://www.ebi.ac.uk/metagenomics/api/v1/analyses/MGYA00202816/file/SRR6745835_MERGED_FASTQ_SSU_OTU.tsv	Root-Environmental-Aquatic-Marine-Coastal-Sediment
SRR6746492	MGYA00202576	https://www.ebi.ac.uk/metagenomics/api/v1/analyses/MGYA00202576/file/SRR6746492_MERGED_FASTQ_SSU_OTU.tsv	Root-Environmental-Aquatic-Marine-Coastal-Sediment
SRR6517612	MGYA00362477	https://www.ebi.ac.uk/metagenomics/api/v1/analyses/MGYA00362477/file/SRR6517612_MERGED_FASTQ_SSU_OTU.tsv	Root-Environmental-Aquatic-Marine-Coastal-Sediment
SRR6745924	MGYA00202815	https://www.ebi.ac.uk/metagenomics/api/v1/analyses/MGYA00202815/file/SRR6745924_MERGED_FASTQ_SSU_OTU.tsv	Root-Environmental-Aquatic-Marine-Coastal-Sediment
SRR6746022	MGYA00202879	https://www.ebi.ac.uk/metagenomics/api/v1/analyses/MGYA00202879/file/SRR6746022_MERGED_FASTQ_SSU_OTU.tsv	Root-Environmental-Aquatic-Marine-Coastal-Sediment
SRR4300281	MGYA00360242	https://www.ebi.ac.uk/metagenomics/api/v1/analyses/MGYA00360242/file/SRR4300281_MERGED_FASTQ_SSU_OTU.tsv	Root-Environmental-Aquatic-Marine-Coastal-Sediment
SRR6745378	MGYA00202625	https://www.ebi.ac.uk/metagenomics/api/v1/analyses/MGYA00202625/file/SRR6745378_MERGED_FASTQ_SSU_OTU.tsv	Root-Environmental-Aquatic-Marine-Coastal-Sediment
SRR6172329	MGYA00362291	https://www.ebi.ac.uk/metagenomics/api/v1/analyses/MGYA00362291/file/SRR6172329_MERGED_FASTQ_SSU_OTU.tsv	Root-Environmental-Aquatic-Marine-Coastal-Sediment
SRR4436297	MGYA00213055	https://www.ebi.ac.uk/metagenomics/api/v1/analyses/MGYA00213055/file/SRR4436297_FASTQ_SSU_OTU.tsv	Root-Environmental-Aquatic-Marine-Coastal-Sediment
SRR6745989	MGYA00202871	https://www.ebi.ac.uk/metagenomics/api/v1/analyses/MGYA00202871/file/SRR6745989_MERGED_FASTQ_SSU_OTU.tsv	Root-Environmental-Aquatic-Marine-Coastal-Sediment

SRR6745958	MGYA00202934	https://www.ebi.ac.uk/metagenomics/api/v1/analyses/MGYA00202934/file/SRR6745958_MERGED_FASTQ_SSU_OTU.tsv	Root-Environmental-Aquatic-Marine-Coastal-Sediment
SRR1258333	MGYA00358462	https://www.ebi.ac.uk/metagenomics/api/v1/analyses/MGYA00358462/file/SRR1258333_FASTQ_SSU_OTU.tsv	Root-Environmental-Aquatic-Marine-Coastal-Sediment
SRR6746567	MGYA00203070	https://www.ebi.ac.uk/metagenomics/api/v1/analyses/MGYA00203070/file/SRR6746567_MERGED_FASTQ_SSU_OTU.tsv	Root-Environmental-Aquatic-Marine-Coastal-Sediment
SRR2855899	MGYA00359217	https://www.ebi.ac.uk/metagenomics/api/v1/analyses/MGYA00359217/file/SRR2855899_MERGED_FASTQ_SSU_OTU.tsv	Root-Environmental-Aquatic-Marine-Coastal-Sediment
SRR6745800	MGYA00202728	https://www.ebi.ac.uk/metagenomics/api/v1/analyses/MGYA00202728/file/SRR6745800_MERGED_FASTQ_SSU_OTU.tsv	Root-Environmental-Aquatic-Marine-Coastal-Sediment
SRR6746106	MGYA00202615	https://www.ebi.ac.uk/metagenomics/api/v1/analyses/MGYA00202615/file/SRR6746106_MERGED_FASTQ_SSU_OTU.tsv	Root-Environmental-Aquatic-Marine-Coastal-Sediment
SRR2297132	MGYA00358896	https://www.ebi.ac.uk/metagenomics/api/v1/analyses/MGYA00358896/file/SRR2297132_MERGED_FASTQ_SSU_OTU.tsv	Root-Environmental-Aquatic-Marine-Coastal-Sediment
SRR6172355	MGYA00362317	https://www.ebi.ac.uk/metagenomics/api/v1/analyses/MGYA00362317/file/SRR6172355_MERGED_FASTQ_SSU_OTU.tsv	Root-Environmental-Aquatic-Marine-Coastal-Sediment
SRR6746211	MGYA00202624	https://www.ebi.ac.uk/metagenomics/api/v1/analyses/MGYA00202624/file/SRR6746211_MERGED_FASTQ_SSU_OTU.tsv	Root-Environmental-Aquatic-Marine-Coastal-Sediment
ERR330026	MGYA00357984	https://www.ebi.ac.uk/metagenomics/api/v1/analyses/MGYA00357984/file/ERR330026_FASTQ_SSU_OTU.tsv	Root-Environmental-Aquatic-Marine-Coastal-Sediment
SRR6746088	MGYA00203033	https://www.ebi.ac.uk/metagenomics/api/v1/analyses/MGYA00203033/file/SRR6746088_MERGED_FASTQ_SSU_OTU.tsv	Root-Environmental-Aquatic-Marine-Coastal-Sediment
SRR4436290	MGYA00213037	https://www.ebi.ac.uk/metagenomics/api/v1/analyses/MGYA00213037/file/SRR4436290_FASTQ_SSU_OTU.tsv	Root-Environmental-Aquatic-Marine-Coastal-Sediment

SRR1258334	MGYA00358463	https://www.ebi.ac.uk/metagenomics/api/v1/analyses/MGYA00358463/file/SRR1258334_FASTQ_SSU_OTU.tsv	Root-Environmental-Aquatic-Marine-Coastal-Sediment
SRR6746560	MGYA00202515	https://www.ebi.ac.uk/metagenomics/api/v1/analyses/MGYA00202515/file/SRR6746560_MERGED_FASTQ_SSU_OTU.tsv	Root-Environmental-Aquatic-Marine-Coastal-Sediment
SRR6745807	MGYA00203088	https://www.ebi.ac.uk/metagenomics/api/v1/analyses/MGYA00203088/file/SRR6745807_MERGED_FASTQ_SSU_OTU.tsv	Root-Environmental-Aquatic-Marine-Coastal-Sediment
SRR2297135	MGYA00358899	https://www.ebi.ac.uk/metagenomics/api/v1/analyses/MGYA00358899/file/SRR2297135_MERGED_FASTQ_SSU_OTU.tsv	Root-Environmental-Aquatic-Marine-Coastal-Sediment
SRR6746101	MGYA00202878	https://www.ebi.ac.uk/metagenomics/api/v1/analyses/MGYA00202878/file/SRR6746101_MERGED_FASTQ_SSU_OTU.tsv	Root-Environmental-Aquatic-Marine-Coastal-Sediment
SRR6172352	MGYA00362314	https://www.ebi.ac.uk/metagenomics/api/v1/analyses/MGYA00362314/file/SRR6172352_MERGED_FASTQ_SSU_OTU.tsv	Root-Environmental-Aquatic-Marine-Coastal-Sediment
SRR6746216	MGYA00202907	https://www.ebi.ac.uk/metagenomics/api/v1/analyses/MGYA00202907/file/SRR6746216_MERGED_FASTQ_SSU_OTU.tsv	Root-Environmental-Aquatic-Marine-Coastal-Sediment
ERR330021	MGYA00357979	https://www.ebi.ac.uk/metagenomics/api/v1/analyses/MGYA00357979/file/ERR330021_FASTQ_SSU_OTU.tsv	Root-Environmental-Aquatic-Marine-Coastal-Sediment
SRR6746495	MGYA00202685	https://www.ebi.ac.uk/metagenomics/api/v1/analyses/MGYA00202685/file/SRR6746495_MERGED_FASTQ_SSU_OTU.tsv	Root-Environmental-Aquatic-Marine-Coastal-Sediment
SRR6745923	MGYA00202857	https://www.ebi.ac.uk/metagenomics/api/v1/analyses/MGYA00202857/file/SRR6745923_MERGED_FASTQ_SSU_OTU.tsv	Root-Environmental-Aquatic-Marine-Coastal-Sediment
SRR6746025	MGYA00202864	https://www.ebi.ac.uk/metagenomics/api/v1/analyses/MGYA00202864/file/SRR6746025_MERGED_FASTQ_SSU_OTU.tsv	Root-Environmental-Aquatic-Marine-Coastal-Sediment
SRR6745409	MGYA00202681	https://www.ebi.ac.uk/metagenomics/api/v1/analyses/MGYA00202681/file/SRR6745409_MERGED_FASTQ_SSU_OTU.tsv	Root-Environmental-Aquatic-Marine-Coastal-Sediment

SRR2297149	MGYA00358913	https://www.ebi.ac.uk/metagenomics/api/v1/analyses/MGYA00358913/file/SRR2297149_MERGED_FASTQ_SSU_OTU.tsv	Root-Environmental-Aquatic-Marine-Coastal-Sediment
SRR4300286	MGYA00360246	https://www.ebi.ac.uk/metagenomics/api/v1/analyses/MGYA00360246/file/SRR4300286_MERGED_FASTQ_SSU_OTU.tsv	Root-Environmental-Aquatic-Marine-Coastal-Sediment
SRR6746086	MGYA00202613	https://www.ebi.ac.uk/metagenomics/api/v1/analyses/MGYA00202613/file/SRR6746086_MERGED_FASTQ_SSU_OTU.tsv	Root-Environmental-Aquatic-Marine-Coastal-Sediment
SRR6745980	MGYA00203096	https://www.ebi.ac.uk/metagenomics/api/v1/analyses/MGYA00203096/file/SRR6745980_MERGED_FASTQ_SSU_OTU.tsv	Root-Environmental-Aquatic-Marine-Coastal-Sediment
SRR6745951	MGYA00202611	https://www.ebi.ac.uk/metagenomics/api/v1/analyses/MGYA00202611/file/SRR6745951_MERGED_FASTQ_SSU_OTU.tsv	Root-Environmental-Aquatic-Marine-Coastal-Sediment
SRR6746057	MGYA00202764	https://www.ebi.ac.uk/metagenomics/api/v1/analyses/MGYA00202764/file/SRR6746057_MERGED_FASTQ_SSU_OTU.tsv	Root-Environmental-Aquatic-Marine-Coastal-Sediment
SRR6745809	MGYA00202869	https://www.ebi.ac.uk/metagenomics/api/v1/analyses/MGYA00202869/file/SRR6745809_MERGED_FASTQ_SSU_OTU.tsv	Root-Environmental-Aquatic-Marine-Coastal-Sediment
SRR6746218	MGYA00202926	https://www.ebi.ac.uk/metagenomics/api/v1/analyses/MGYA00202926/file/SRR6746218_MERGED_FASTQ_SSU_OTU.tsv	Root-Environmental-Aquatic-Marine-Coastal-Sediment
SRR4300301	MGYA00360260	https://www.ebi.ac.uk/metagenomics/api/v1/analyses/MGYA00360260/file/SRR4300301_MERGED_FASTQ_SSU_OTU.tsv	Root-Environmental-Aquatic-Marine-Coastal-Sediment
SRR6746512	MGYA00202955	https://www.ebi.ac.uk/metagenomics/api/v1/analyses/MGYA00202955/file/SRR6746512_MERGED_FASTQ_SSU_OTU.tsv	Root-Environmental-Aquatic-Marine-Coastal-Sediment
SRR1503974	MGYA00358529	https://www.ebi.ac.uk/metagenomics/api/v1/analyses/MGYA00358529/file/SRR1503974_FASTQ_SSU_OTU.tsv	Root-Environmental-Aquatic-Marine-Coastal-Sediment
SRR6745875	MGYA00202865	https://www.ebi.ac.uk/metagenomics/api/v1/analyses/MGYA00202865/file/SRR6745875_MERGED_FASTQ_SSU_OTU.tsv	Root-Environmental-Aquatic-Marine-Coastal-Sediment

SRR6745407	MGYA00202670	https://www.ebi.ac.uk/metagenomics/api/v1/analyses/MGYA00202670/file/SRR6745407_MERGED_FASTQ_SSU_OTU.tsv	Root-Environmental-Aquatic-Marine-Coastal-Sediment
SRR6746173	MGYA00202768	https://www.ebi.ac.uk/metagenomics/api/v1/analyses/MGYA00202768/file/SRR6746173_MERGED_FASTQ_SSU_OTU.tsv	Root-Environmental-Aquatic-Marine-Coastal-Sediment
SRR2297147	MGYA00358911	https://www.ebi.ac.uk/metagenomics/api/v1/analyses/MGYA00358911/file/SRR2297147_MERGED_FASTQ_SSU_OTU.tsv	Root-Environmental-Aquatic-Marine-Coastal-Sediment
SRR4300288	MGYA00360248	https://www.ebi.ac.uk/metagenomics/api/v1/analyses/MGYA00360248/file/SRR4300288_MERGED_FASTQ_SSU_OTU.tsv	Root-Environmental-Aquatic-Marine-Coastal-Sediment
SRR6745371	MGYA00202500	https://www.ebi.ac.uk/metagenomics/api/v1/analyses/MGYA00202500/file/SRR6745371_MERGED_FASTQ_SSU_OTU.tsv	Root-Environmental-Aquatic-Marine-Coastal-Sediment
SRR650828	MGYA00356177	https://www.ebi.ac.uk/metagenomics/api/v1/analyses/MGYA00356177/file/SRR650828_FASTQ_SSU_OTU.tsv	Root-Environmental-Aquatic-Marine-Coastal-Sediment
ERR330053	MGYA00358011	https://www.ebi.ac.uk/metagenomics/api/v1/analyses/MGYA00358011/file/ERR330053_FASTQ_SSU_OTU.tsv	Root-Environmental-Aquatic-Marine-Coastal-Sediment
SRR2713037	MGYA00355961	https://www.ebi.ac.uk/metagenomics/api/v1/analyses/MGYA00355961/file/SRR2713037_FASTQ_SSU_OTU.tsv	Root-Environmental-Aquatic-Marine-Coastal-Sediment
SRR1258341	MGYA00358464	https://www.ebi.ac.uk/metagenomics/api/v1/analyses/MGYA00358464/file/SRR1258341_FASTQ_SSU_OTU.tsv	Root-Environmental-Aquatic-Marine-Coastal-Sediment
SRR6746515	MGYA00202936	https://www.ebi.ac.uk/metagenomics/api/v1/analyses/MGYA00202936/file/SRR6746515_MERGED_FASTQ_SSU_OTU.tsv	Root-Environmental-Aquatic-Marine-Coastal-Sediment
SRR6745400	MGYA00202553	https://www.ebi.ac.uk/metagenomics/api/v1/analyses/MGYA00202553/file/SRR6745400_MERGED_FASTQ_SSU_OTU.tsv	Root-Environmental-Aquatic-Marine-Coastal-Sediment
SRR1503973	MGYA00358528	https://www.ebi.ac.uk/metagenomics/api/v1/analyses/MGYA00358528/file/SRR1503973_FASTQ_SSU_OTU.tsv	Root-Environmental-Aquatic-Marine-Coastal-Sediment

SRR6745872	MGYA00202678	https://www.ebi.ac.uk/metagenomics/api/v1/analyses/MGYA00202678/file/SRR6745872_MERGED_FASTQ_SSU_OTU.tsv	Root-Environmental-Aquatic-Marine-Coastal-Sediment
SRR2297140	MGYA00358904	https://www.ebi.ac.uk/metagenomics/api/v1/analyses/MGYA00358904/file/SRR2297140_MERGED_FASTQ_SSU_OTU.tsv	Root-Environmental-Aquatic-Marine-Coastal-Sediment
SRR6746174	MGYA00202844	https://www.ebi.ac.uk/metagenomics/api/v1/analyses/MGYA00202844/file/SRR6746174_MERGED_FASTQ_SSU_OTU.tsv	Root-Environmental-Aquatic-Marine-Coastal-Sediment
SRR6745376	MGYA00202583	https://www.ebi.ac.uk/metagenomics/api/v1/analyses/MGYA00202583/file/SRR6745376_MERGED_FASTQ_SSU_OTU.tsv	Root-Environmental-Aquatic-Marine-Coastal-Sediment
SRR6172327	MGYA00362289	https://www.ebi.ac.uk/metagenomics/api/v1/analyses/MGYA00362289/file/SRR6172327_MERGED_FASTQ_SSU_OTU.tsv	Root-Environmental-Aquatic-Marine-Coastal-Sediment
ERR330054	MGYA00358012	https://www.ebi.ac.uk/metagenomics/api/v1/analyses/MGYA00358012/file/ERR330054_FASTQ_SSU_OTU.tsv	Root-Environmental-Aquatic-Marine-Coastal-Sediment
SRR6746081	MGYA00202915	https://www.ebi.ac.uk/metagenomics/api/v1/analyses/MGYA00202915/file/SRR6746081_MERGED_FASTQ_SSU_OTU.tsv	Root-Environmental-Aquatic-Marine-Coastal-Sediment
SRR6745987	MGYA00203019	https://www.ebi.ac.uk/metagenomics/api/v1/analyses/MGYA00203019/file/SRR6745987_MERGED_FASTQ_SSU_OTU.tsv	Root-Environmental-Aquatic-Marine-Coastal-Sediment
SRR4436299	MGYA00213039	https://www.ebi.ac.uk/metagenomics/api/v1/analyses/MGYA00213039/file/SRR4436299_FASTQ_SSU_OTU.tsv	Root-Environmental-Aquatic-Marine-Coastal-Sediment
SRR6745956	MGYA00202629	https://www.ebi.ac.uk/metagenomics/api/v1/analyses/MGYA00202629/file/SRR6745956_MERGED_FASTQ_SSU_OTU.tsv	Root-Environmental-Aquatic-Marine-Coastal-Sediment
SRR6746050	MGYA00202848	https://www.ebi.ac.uk/metagenomics/api/v1/analyses/MGYA00202848/file/SRR6746050_MERGED_FASTQ_SSU_OTU.tsv	Root-Environmental-Aquatic-Marine-Coastal-Sediment
SRR6746569	MGYA00203044	https://www.ebi.ac.uk/metagenomics/api/v1/analyses/MGYA00203044/file/SRR6746569_MERGED_FASTQ_SSU_OTU.tsv	Root-Environmental-Aquatic-Marine-Coastal-Sediment

SRR6746108	MGYA00202821	https://www.ebi.ac.uk/metagenomics/api/v1/analyses/MGYA00202821/file/SRR6746108_MERGED_FASTQ_SSU_OTU.tsv	Root-Environmental-Aquatic-Marine-Coastal-Sediment
ERR330028	MGYA00357986	https://www.ebi.ac.uk/metagenomics/api/v1/analyses/MGYA00357986/file/ERR330028_FASTQ_SSU_OTU.tsv	Root-Environmental-Aquatic-Marine-Coastal-Sediment
SRR2297180	MGYA00358944	https://www.ebi.ac.uk/metagenomics/api/v1/analyses/MGYA00358944/file/SRR2297180_MERGED_FASTQ_SSU_OTU.tsv	Root-Environmental-Aquatic-Marine-Coastal-Sediment
ERR330045	MGYA00358003	https://www.ebi.ac.uk/metagenomics/api/v1/analyses/MGYA00358003/file/ERR330045_FASTQ_SSU_OTU.tsv	Root-Environmental-Aquatic-Marine-Coastal-Sediment
SRR6172336	MGYA00362298	https://www.ebi.ac.uk/metagenomics/api/v1/analyses/MGYA00362298/file/SRR6172336_MERGED_FASTQ_SSU_OTU.tsv	Root-Environmental-Aquatic-Marine-Coastal-Sediment
SRR6745367	MGYA00202742	https://www.ebi.ac.uk/metagenomics/api/v1/analyses/MGYA00202742/file/SRR6745367_MERGED_FASTQ_SSU_OTU.tsv	Root-Environmental-Aquatic-Marine-Coastal-Sediment
SRR6745863	MGYA00202612	https://www.ebi.ac.uk/metagenomics/api/v1/analyses/MGYA00202612/file/SRR6745863_MERGED_FASTQ_SSU_OTU.tsv	Root-Environmental-Aquatic-Marine-Coastal-Sediment
SRR6745411	MGYA00203081	https://www.ebi.ac.uk/metagenomics/api/v1/analyses/MGYA00203081/file/SRR6745411_MERGED_FASTQ_SSU_OTU.tsv	Root-Environmental-Aquatic-Marine-Coastal-Sediment
SRR6746504	MGYA00203090	https://www.ebi.ac.uk/metagenomics/api/v1/analyses/MGYA00203090/file/SRR6746504_MERGED_FASTQ_SSU_OTU.tsv	Root-Environmental-Aquatic-Marine-Coastal-Sediment
SRR6746165	MGYA00202602	https://www.ebi.ac.uk/metagenomics/api/v1/analyses/MGYA00202602/file/SRR6746165_MERGED_FASTQ_SSU_OTU.tsv	Root-Environmental-Aquatic-Marine-Coastal-Sediment
SRR2297151	MGYA00358915	https://www.ebi.ac.uk/metagenomics/api/v1/analyses/MGYA00358915/file/SRR2297151_MERGED_FASTQ_SSU_OTU.tsv	Root-Environmental-Aquatic-Marine-Coastal-Sediment
ERR330039	MGYA00357997	https://www.ebi.ac.uk/metagenomics/api/v1/analyses/MGYA00357997/file/ERR330039_FASTQ_SSU_OTU.tsv	Root-Environmental-Aquatic-Marine-Coastal-Sediment

SRR4436301	MGYA00213040	https://www.ebi.ac.uk/metagenomics/api/v1/analyses/MGYA00213040/file/SRR4436301_FASTQ_SSU_OTU.tsv	Root-Environmental-Aquatic-Marine-Coastal-Sediment
SRR6746119	MGYA00202503	https://www.ebi.ac.uk/metagenomics/api/v1/analyses/MGYA00202503/file/SRR6746119_MERGED_FASTQ_SSU_OTU.tsv	Root-Environmental-Aquatic-Marine-Coastal-Sediment
SRR3355024	MGYA00359639	https://www.ebi.ac.uk/metagenomics/api/v1/analyses/MGYA00359639/file/SRR3355024_MERGED_FASTQ_SSU_OTU.tsv	Root-Environmental-Aquatic-Marine-Coastal-Sediment
SRR6745947	MGYA00202657	https://www.ebi.ac.uk/metagenomics/api/v1/analyses/MGYA00202657/file/SRR6745947_MERGED_FASTQ_SSU_OTU.tsv	Root-Environmental-Aquatic-Marine-Coastal-Sediment
SRR6746041	MGYA00202674	https://www.ebi.ac.uk/metagenomics/api/v1/analyses/MGYA00202674/file/SRR6746041_MERGED_FASTQ_SSU_OTU.tsv	Root-Environmental-Aquatic-Marine-Coastal-Sediment
SRR6746090	MGYA00202947	https://www.ebi.ac.uk/metagenomics/api/v1/analyses/MGYA00202947/file/SRR6746090_MERGED_FASTQ_SSU_OTU.tsv	Root-Environmental-Aquatic-Marine-Coastal-Sediment
SRR4436288	MGYA00213043	https://www.ebi.ac.uk/metagenomics/api/v1/analyses/MGYA00213043/file/SRR4436288_FASTQ_SSU_OTU.tsv	Root-Environmental-Aquatic-Marine-Coastal-Sediment
SRR6745996	MGYA00202761	https://www.ebi.ac.uk/metagenomics/api/v1/analyses/MGYA00202761/file/SRR6745996_MERGED_FASTQ_SSU_OTU.tsv	Root-Environmental-Aquatic-Marine-Coastal-Sediment
SRR6746209	MGYA00203007	https://www.ebi.ac.uk/metagenomics/api/v1/analyses/MGYA00203007/file/SRR6746209_MERGED_FASTQ_SSU_OTU.tsv	Root-Environmental-Aquatic-Marine-Coastal-Sediment
SRR2856994	MGYA00359234	https://www.ebi.ac.uk/metagenomics/api/v1/analyses/MGYA00359234/file/SRR2856994_MERGED_FASTQ_SSU_OTU.tsv	Root-Environmental-Aquatic-Marine-Coastal-Sediment
SRR4436306	MGYA00213050	https://www.ebi.ac.uk/metagenomics/api/v1/analyses/MGYA00213050/file/SRR4436306_FASTQ_SSU_OTU.tsv	Root-Environmental-Aquatic-Marine-Coastal-Sediment
SRR6745818	MGYA00202762	https://www.ebi.ac.uk/metagenomics/api/v1/analyses/MGYA00202762/file/SRR6745818_MERGED_FASTQ_SSU_OTU.tsv	Root-Environmental-Aquatic-Marine-Coastal-Sediment

SRR2856620	MGYA00359233	https://www.ebi.ac.uk/metagenomics/api/v1/analyses/MGYA00359233/file/SRR2856620_MERGED_FASTQ_SSU_OTU.tsv	Root-Environmental-Aquatic-Marine-Coastal-Sediment
SRR6745940	MGYA00202914	https://www.ebi.ac.uk/metagenomics/api/v1/analyses/MGYA00202914/file/SRR6745940_MERGED_FASTQ_SSU_OTU.tsv	Root-Environmental-Aquatic-Marine-Coastal-Sediment
SRR6746046	MGYA00202707	https://www.ebi.ac.uk/metagenomics/api/v1/analyses/MGYA00202707/file/SRR6746046_MERGED_FASTQ_SSU_OTU.tsv	Root-Environmental-Aquatic-Marine-Coastal-Sediment
SRR6746097	MGYA00202750	https://www.ebi.ac.uk/metagenomics/api/v1/analyses/MGYA00202750/file/SRR6746097_MERGED_FASTQ_SSU_OTU.tsv	Root-Environmental-Aquatic-Marine-Coastal-Sediment
SRR6745991	MGYA00202809	https://www.ebi.ac.uk/metagenomics/api/v1/analyses/MGYA00202809/file/SRR6745991_MERGED_FASTQ_SSU_OTU.tsv	Root-Environmental-Aquatic-Marine-Coastal-Sediment
SRR4300299	MGYA00360258	https://www.ebi.ac.uk/metagenomics/api/v1/analyses/MGYA00360258/file/SRR4300299_MERGED_FASTQ_SSU_OTU.tsv	Root-Environmental-Aquatic-Marine-Coastal-Sediment
SRR2297187	MGYA00358951	https://www.ebi.ac.uk/metagenomics/api/v1/analyses/MGYA00358951/file/SRR2297187_MERGED_FASTQ_SSU_OTU.tsv	Root-Environmental-Aquatic-Marine-Coastal-Sediment
ERR330042	MGYA00358000	https://www.ebi.ac.uk/metagenomics/api/v1/analyses/MGYA00358000/file/ERR330042_FASTQ_SSU_OTU.tsv	Root-Environmental-Aquatic-Marine-Coastal-Sediment
SRR6745360	MGYA00202717	https://www.ebi.ac.uk/metagenomics/api/v1/analyses/MGYA00202717/file/SRR6745360_MERGED_FASTQ_SSU_OTU.tsv	Root-Environmental-Aquatic-Marine-Coastal-Sediment
SRR6172331	MGYA00362293	https://www.ebi.ac.uk/metagenomics/api/v1/analyses/MGYA00362293/file/SRR6172331_MERGED_FASTQ_SSU_OTU.tsv	Root-Environmental-Aquatic-Marine-Coastal-Sediment
SRR6745416	MGYA00202680	https://www.ebi.ac.uk/metagenomics/api/v1/analyses/MGYA00202680/file/SRR6745416_MERGED_FASTQ_SSU_OTU.tsv	Root-Environmental-Aquatic-Marine-Coastal-Sediment
SRR6745864	MGYA00202900	https://www.ebi.ac.uk/metagenomics/api/v1/analyses/MGYA00202900/file/SRR6745864_MERGED_FASTQ_SSU_OTU.tsv	Root-Environmental-Aquatic-Marine-Coastal-Sediment

SRR6746503	MGYA00203069	https://www.ebi.ac.uk/metagenomics/api/v1/analyses/MGYA00203069/file/SRR6746503_MERGED_FASTQ_SSU_OTU.tsv	Root-Environmental-Aquatic-Marine-Coastal-Sediment
SRR2297156	MGYA00358920	https://www.ebi.ac.uk/metagenomics/api/v1/analyses/MGYA00358920/file/SRR2297156_MERGED_FASTQ_SSU_OTU.tsv	Root-Environmental-Aquatic-Marine-Coastal-Sediment
SRR6746162	MGYA00203075	https://www.ebi.ac.uk/metagenomics/api/v1/analyses/MGYA00203075/file/SRR6746162_MERGED_FASTQ_SSU_OTU.tsv	Root-Environmental-Aquatic-Marine-Coastal-Sediment
SRR6746207	MGYA00203113	https://www.ebi.ac.uk/metagenomics/api/v1/analyses/MGYA00203113/file/SRR6746207_MERGED_FASTQ_SSU_OTU.tsv	Root-Environmental-Aquatic-Marine-Coastal-Sediment
ERR330030	MGYA00357988	https://www.ebi.ac.uk/metagenomics/api/v1/analyses/MGYA00357988/file/ERR330030_FASTQ_SSU_OTU.tsv	Root-Environmental-Aquatic-Marine-Coastal-Sediment
SRR6172343	MGYA00362305	https://www.ebi.ac.uk/metagenomics/api/v1/analyses/MGYA00362305/file/SRR6172343_MERGED_FASTQ_SSU_OTU.tsv	Root-Environmental-Aquatic-Marine-Coastal-Sediment
SRR6745816	MGYA00202912	https://www.ebi.ac.uk/metagenomics/api/v1/analyses/MGYA00202912/file/SRR6745816_MERGED_FASTQ_SSU_OTU.tsv	Root-Environmental-Aquatic-Marine-Coastal-Sediment
SRR6746571	MGYA00202661	https://www.ebi.ac.uk/metagenomics/api/v1/analyses/MGYA00202661/file/SRR6746571_MERGED_FASTQ_SSU_OTU.tsv	Root-Environmental-Aquatic-Marine-Coastal-Sediment
SRR6746110	MGYA00202716	https://www.ebi.ac.uk/metagenomics/api/v1/analyses/MGYA00202716/file/SRR6746110_MERGED_FASTQ_SSU_OTU.tsv	Root-Environmental-Aquatic-Marine-Coastal-Sediment
SRR2297124	MGYA00358888	https://www.ebi.ac.uk/metagenomics/api/v1/analyses/MGYA00358888/file/SRR2297124_MERGED_FASTQ_SSU_OTU.tsv	Root-Environmental-Aquatic-Marine-Coastal-Sediment
SRR826483	MGYA00357573	https://www.ebi.ac.uk/metagenomics/api/v1/analyses/MGYA00357573/file/SRR826483_FASTQ_SSU_OTU.tsv	Root-Environmental-Aquatic-Marine-Coastal-Sediment
SRR6746048	MGYA00202511	https://www.ebi.ac.uk/metagenomics/api/v1/analyses/MGYA00202511/file/SRR6746048_MERGED_FASTQ_SSU_OTU.tsv	Root-Environmental-Aquatic-Marine-Coastal-Sediment

SRR6746099	MGYA00203040	https://www.ebi.ac.uk/metagenomics/api/v1/analyses/MGYA00203040/file/SRR6746099_MERGED_FASTQ_SSU_OTU.tsv	Root-Environmental-Aquatic-Marine-Coastal-Sediment
SRR2297189	MGYA00358953	https://www.ebi.ac.uk/metagenomics/api/v1/analyses/MGYA00358953/file/SRR2297189_MERGED_FASTQ_SSU_OTU.tsv	Root-Environmental-Aquatic-Marine-Coastal-Sediment
SRR4300297	MGYA00360256	https://www.ebi.ac.uk/metagenomics/api/v1/analyses/MGYA00360256/file/SRR4300297_MERGED_FASTQ_SSU_OTU.tsv	Root-Environmental-Aquatic-Marine-Coastal-Sediment
SRR6745418	MGYA00202664	https://www.ebi.ac.uk/metagenomics/api/v1/analyses/MGYA00202664/file/SRR6745418_MERGED_FASTQ_SSU_OTU.tsv	Root-Environmental-Aquatic-Marine-Coastal-Sediment
SRR2297158	MGYA00358922	https://www.ebi.ac.uk/metagenomics/api/v1/analyses/MGYA00358922/file/SRR2297158_MERGED_FASTQ_SSU_OTU.tsv	Root-Environmental-Aquatic-Marine-Coastal-Sediment
SRR6745932	MGYA00202986	https://www.ebi.ac.uk/metagenomics/api/v1/analyses/MGYA00202986/file/SRR6745932_MERGED_FASTQ_SSU_OTU.tsv	Root-Environmental-Aquatic-Marine-Coastal-Sediment
SRR6746034	MGYA00202786	https://www.ebi.ac.uk/metagenomics/api/v1/analyses/MGYA00202786/file/SRR6746034_MERGED_FASTQ_SSU_OTU.tsv	Root-Environmental-Aquatic-Marine-Coastal-Sediment
SRR6517604	MGYA00362469	https://www.ebi.ac.uk/metagenomics/api/v1/analyses/MGYA00362469/file/SRR6517604_MERGED_FASTQ_SSU_OTU.tsv	Root-Environmental-Aquatic-Marine-Coastal-Sediment
SRR6746484	MGYA00202943	https://www.ebi.ac.uk/metagenomics/api/v1/analyses/MGYA00202943/file/SRR6746484_MERGED_FASTQ_SSU_OTU.tsv	Root-Environmental-Aquatic-Marine-Coastal-Sediment
SRR4300290	MGYA00360250	https://www.ebi.ac.uk/metagenomics/api/v1/analyses/MGYA00360250/file/SRR4300290_MERGED_FASTQ_SSU_OTU.tsv	Root-Environmental-Aquatic-Marine-Coastal-Sediment
SRR6172338	MGYA00362300	https://www.ebi.ac.uk/metagenomics/api/v1/analyses/MGYA00362300/file/SRR6172338_MERGED_FASTQ_SSU_OTU.tsv	Root-Environmental-Aquatic-Marine-Coastal-Sediment
SRR6745369	MGYA00202617	https://www.ebi.ac.uk/metagenomics/api/v1/analyses/MGYA00202617/file/SRR6745369_MERGED_FASTQ_SSU_OTU.tsv	Root-Environmental-Aquatic-Marine-Coastal-Sediment

SRR650830	MGYA00356179	https://www.ebi.ac.uk/metagenomics/api/v1/analyses/MGYA00356179/file/SRR650830_FASTQ_SSU_OTU.tsv	Root-Environmental-Aquatic-Marine-Coastal-Sediment
SRR6745935	MGYA00202850	https://www.ebi.ac.uk/metagenomics/api/v1/analyses/MGYA00202850/file/SRR6745935_MERGED_FASTQ_SSU_OTU.tsv	Root-Environmental-Aquatic-Marine-Coastal-Sediment
SRR6746033	MGYA00202527	https://www.ebi.ac.uk/metagenomics/api/v1/analyses/MGYA00202527/file/SRR6746033_MERGED_FASTQ_SSU_OTU.tsv	Root-Environmental-Aquatic-Marine-Coastal-Sediment
SRR6746483	MGYA00203002	https://www.ebi.ac.uk/metagenomics/api/v1/analyses/MGYA00203002/file/SRR6746483_MERGED_FASTQ_SSU_OTU.tsv	Root-Environmental-Aquatic-Marine-Coastal-Sediment
SRR6517603	MGYA00362468	https://www.ebi.ac.uk/metagenomics/api/v1/analyses/MGYA00362468/file/SRR6517603_MERGED_FASTQ_SSU_OTU.tsv	Root-Environmental-Aquatic-Marine-Coastal-Sediment
SRR6746200	MGYA00202558	https://www.ebi.ac.uk/metagenomics/api/v1/analyses/MGYA00202558/file/SRR6746200_MERGED_FASTQ_SSU_OTU.tsv	Root-Environmental-Aquatic-Marine-Coastal-Sediment
ERR330037	MGYA00357995	https://www.ebi.ac.uk/metagenomics/api/v1/analyses/MGYA00357995/file/ERR330037_FASTQ_SSU_OTU.tsv	Root-Environmental-Aquatic-Marine-Coastal-Sediment
SRR6172344	MGYA00362306	https://www.ebi.ac.uk/metagenomics/api/v1/analyses/MGYA00362306/file/SRR6172344_MERGED_FASTQ_SSU_OTU.tsv	Root-Environmental-Aquatic-Marine-Coastal-Sediment
SRR6745811	MGYA00202724	https://www.ebi.ac.uk/metagenomics/api/v1/analyses/MGYA00202724/file/SRR6745811_MERGED_FASTQ_SSU_OTU.tsv	Root-Environmental-Aquatic-Marine-Coastal-Sediment
SRR2297123	MGYA00358887	https://www.ebi.ac.uk/metagenomics/api/v1/analyses/MGYA00358887/file/SRR2297123_MERGED_FASTQ_SSU_OTU.tsv	Root-Environmental-Aquatic-Marine-Coastal-Sediment
SRR6746117	MGYA00203052	https://www.ebi.ac.uk/metagenomics/api/v1/analyses/MGYA00203052/file/SRR6746117_MERGED_FASTQ_SSU_OTU.tsv	Root-Environmental-Aquatic-Marine-Coastal-Sediment
SRR6745949	MGYA00202623	https://www.ebi.ac.uk/metagenomics/api/v1/analyses/MGYA00202623/file/SRR6745949_MERGED_FASTQ_SSU_OTU.tsv	Root-Environmental-Aquatic-Marine-Coastal-Sediment

SRR6745998	MGYA00202630	https://www.ebi.ac.uk/metagenomics/api/v1/analyses/MGYA00202630/file/SRR6745998_MERGED_FASTQ_SSU_OTU.tsv	Root-Environmental-Aquatic-Marine-Coastal-Sediment
SRR2855901	MGYA00359218	https://www.ebi.ac.uk/metagenomics/api/v1/analyses/MGYA00359218/file/SRR2855901_MERGED_FASTQ_SSU_OTU.tsv	Root-Environmental-Aquatic-Marine-Coastal-Sediment
SRR4436286	MGYA00213038	https://www.ebi.ac.uk/metagenomics/api/v1/analyses/MGYA00213038/file/SRR4436286_FASTQ_SSU_OTU.tsv	Root-Environmental-Aquatic-Marine-Coastal-Sediment
SRR4300274	MGYA00360235	https://www.ebi.ac.uk/metagenomics/api/v1/analyses/MGYA00360235/file/SRR4300274_MERGED_FASTQ_SSU_OTU.tsv	Root-Environmental-Aquatic-Marine-Coastal-Sediment
SRR2855775	MGYA00359214	https://www.ebi.ac.uk/metagenomics/api/v1/analyses/MGYA00359214/file/SRR2855775_MERGED_FASTQ_SSU_OTU.tsv	Root-Environmental-Aquatic-Marine-Coastal-Sediment
SRR6745858	MGYA00202644	https://www.ebi.ac.uk/metagenomics/api/v1/analyses/MGYA00202644/file/SRR6745858_MERGED_FASTQ_SSU_OTU.tsv	Root-Environmental-Aquatic-Marine-Coastal-Sediment
SRR6745889	MGYA00202605	https://www.ebi.ac.uk/metagenomics/api/v1/analyses/MGYA00202605/file/SRR6745889_MERGED_FASTQ_SSU_OTU.tsv	Root-Environmental-Aquatic-Marine-Coastal-Sediment
SRR6746006	MGYA00203115	https://www.ebi.ac.uk/metagenomics/api/v1/analyses/MGYA00203115/file/SRR6746006_MERGED_FASTQ_SSU_OTU.tsv	Root-Environmental-Aquatic-Marine-Coastal-Sediment
SRR6746467	MGYA00202518	https://www.ebi.ac.uk/metagenomics/api/v1/analyses/MGYA00202518/file/SRR6746467_MERGED_FASTQ_SSU_OTU.tsv	Root-Environmental-Aquatic-Marine-Coastal-Sediment
SRR3355067	MGYA00359642	https://www.ebi.ac.uk/metagenomics/api/v1/analyses/MGYA00359642/file/SRR3355067_MERGED_FASTQ_SSU_OTU.tsv	Root-Environmental-Aquatic-Marine-Coastal-Sediment
SRR6745790	MGYA00202833	https://www.ebi.ac.uk/metagenomics/api/v1/analyses/MGYA00202833/file/SRR6745790_MERGED_FASTQ_SSU_OTU.tsv	Root-Environmental-Aquatic-Marine-Coastal-Sediment
SRR6746122	MGYA00203016	https://www.ebi.ac.uk/metagenomics/api/v1/analyses/MGYA00203016/file/SRR6746122_MERGED_FASTQ_SSU_OTU.tsv	Root-Environmental-Aquatic-Marine-Coastal-Sediment

SRR2297116	MGYA00358880	https://www.ebi.ac.uk/metagenomics/api/v1/analyses/MGYA00358880/file/SRR2297116_MERGED_FASTQ_SSU_OTU.tsv	Root-Environmental-Aquatic-Marine-Coastal-Sediment
SRR6745824	MGYA00202782	https://www.ebi.ac.uk/metagenomics/api/v1/analyses/MGYA00202782/file/SRR6745824_MERGED_FASTQ_SSU_OTU.tsv	Root-Environmental-Aquatic-Marine-Coastal-Sediment
SRR6746543	MGYA00202791	https://www.ebi.ac.uk/metagenomics/api/v1/analyses/MGYA00202791/file/SRR6746543_MERGED_FASTQ_SSU_OTU.tsv	Root-Environmental-Aquatic-Marine-Coastal-Sediment
SRR2856453	MGYA00359228	https://www.ebi.ac.uk/metagenomics/api/v1/analyses/MGYA00359228/file/SRR2856453_MERGED_FASTQ_SSU_OTU.tsv	Root-Environmental-Aquatic-Marine-Coastal-Sediment
SRR6745797	MGYA00202999	https://www.ebi.ac.uk/metagenomics/api/v1/analyses/MGYA00202999/file/SRR6745797_MERGED_FASTQ_SSU_OTU.tsv	Root-Environmental-Aquatic-Marine-Coastal-Sediment
SRR2297111	MGYA00358875	https://www.ebi.ac.uk/metagenomics/api/v1/analyses/MGYA00358875/file/SRR2297111_MERGED_FASTQ_SSU_OTU.tsv	Root-Environmental-Aquatic-Marine-Coastal-Sediment
SRR6746125	MGYA00202866	https://www.ebi.ac.uk/metagenomics/api/v1/analyses/MGYA00202866/file/SRR6746125_MERGED_FASTQ_SSU_OTU.tsv	Root-Environmental-Aquatic-Marine-Coastal-Sediment
SRR6745823	MGYA00202977	https://www.ebi.ac.uk/metagenomics/api/v1/analyses/MGYA00202977/file/SRR6745823_MERGED_FASTQ_SSU_OTU.tsv	Root-Environmental-Aquatic-Marine-Coastal-Sediment
SRR6746544	MGYA00202966	https://www.ebi.ac.uk/metagenomics/api/v1/analyses/MGYA00202966/file/SRR6746544_MERGED_FASTQ_SSU_OTU.tsv	Root-Environmental-Aquatic-Marine-Coastal-Sediment
SRR2856454	MGYA00359229	https://www.ebi.ac.uk/metagenomics/api/v1/analyses/MGYA00359229/file/SRR2856454_MERGED_FASTQ_SSU_OTU.tsv	Root-Environmental-Aquatic-Marine-Coastal-Sediment
SRR4300273	MGYA00360234	https://www.ebi.ac.uk/metagenomics/api/v1/analyses/MGYA00360234/file/SRR4300273_MERGED_FASTQ_SSU_OTU.tsv	Root-Environmental-Aquatic-Marine-Coastal-Sediment
SRR6746159	MGYA00202860	https://www.ebi.ac.uk/metagenomics/api/v1/analyses/MGYA00202860/file/SRR6746159_MERGED_FASTQ_SSU_OTU.tsv	Root-Environmental-Aquatic-Marine-Coastal-Sediment

SRR6746538	MGYA00202723	https://www.ebi.ac.uk/metagenomics/api/v1/analyses/MGYA00202723/file/SRR6746538_MERGED_FASTQ_SSU_OTU.tsv	Root-Environmental-Aquatic-Marine-Coastal-Sediment
SRR5097541	MGYA00360553	https://www.ebi.ac.uk/metagenomics/api/v1/analyses/MGYA00360553/file/SRR5097541_MERGED_FASTQ_SSU_OTU.tsv	Root-Environmental-Aquatic-Marine-Coastal-Sediment
SRR6746188	MGYA00202953	https://www.ebi.ac.uk/metagenomics/api/v1/analyses/MGYA00202953/file/SRR6746188_MERGED_FASTQ_SSU_OTU.tsv	Root-Environmental-Aquatic-Marine-Coastal-Sediment
SRR6746001	MGYA00202548	https://www.ebi.ac.uk/metagenomics/api/v1/analyses/MGYA00202548/file/SRR6746001_MERGED_FASTQ_SSU_OTU.tsv	Root-Environmental-Aquatic-Marine-Coastal-Sediment
SRR6745907	MGYA00202540	https://www.ebi.ac.uk/metagenomics/api/v1/analyses/MGYA00202540/file/SRR6745907_MERGED_FASTQ_SSU_OTU.tsv	Root-Environmental-Aquatic-Marine-Coastal-Sediment
SRR6745799	MGYA00202868	https://www.ebi.ac.uk/metagenomics/api/v1/analyses/MGYA00202868/file/SRR6745799_MERGED_FASTQ_SSU_OTU.tsv	Root-Environmental-Aquatic-Marine-Coastal-Sediment
SRR5097533	MGYA00360545	https://www.ebi.ac.uk/metagenomics/api/v1/analyses/MGYA00360545/file/SRR5097533_MERGED_FASTQ_SSU_OTU.tsv	Root-Environmental-Aquatic-Marine-Coastal-Sediment
SRR5227103	MGYA00360662	https://www.ebi.ac.uk/metagenomics/api/v1/analyses/MGYA00360662/file/SRR5227103_FASTQ_SSU_OTU.tsv	Root-Environmental-Aquatic-Marine-Coastal-Sediment
SRR6746073	MGYA00202929	https://www.ebi.ac.uk/metagenomics/api/v1/analyses/MGYA00202929/file/SRR6746073_MERGED_FASTQ_SSU_OTU.tsv	Root-Environmental-Aquatic-Marine-Coastal-Sediment
SRR6745975	MGYA00202526	https://www.ebi.ac.uk/metagenomics/api/v1/analyses/MGYA00202526/file/SRR6745975_MERGED_FASTQ_SSU_OTU.tsv	Root-Environmental-Aquatic-Marine-Coastal-Sediment
SRR6746157	MGYA00203042	https://www.ebi.ac.uk/metagenomics/api/v1/analyses/MGYA00203042/file/SRR6746157_MERGED_FASTQ_SSU_OTU.tsv	Root-Environmental-Aquatic-Marine-Coastal-Sediment
SRR2297163	MGYA00358927	https://www.ebi.ac.uk/metagenomics/api/v1/analyses/MGYA00358927/file/SRR2297163_MERGED_FASTQ_SSU_OTU.tsv	Root-Environmental-Aquatic-Marine-Coastal-Sediment

SRR6745423	MGYA00202632	https://www.ebi.ac.uk/metagenomics/api/v1/analyses/MGYA00202632/file/SRR6745423_MERGED_FASTQ_SSU_OTU.tsv	Root-Environmental-Aquatic-Marine-Coastal-Sediment
SRR6745851	MGYA00202696	https://www.ebi.ac.uk/metagenomics/api/v1/analyses/MGYA00202696/file/SRR6745851_MERGED_FASTQ_SSU_OTU.tsv	Root-Environmental-Aquatic-Marine-Coastal-Sediment
SRR6745384	MGYA00203062	https://www.ebi.ac.uk/metagenomics/api/v1/analyses/MGYA00203062/file/SRR6745384_MERGED_FASTQ_SSU_OTU.tsv	Root-Environmental-Aquatic-Marine-Coastal-Sediment
SRR6746536	MGYA00202906	https://www.ebi.ac.uk/metagenomics/api/v1/analyses/MGYA00202906/file/SRR6746536_MERGED_FASTQ_SSU_OTU.tsv	Root-Environmental-Aquatic-Marine-Coastal-Sediment
SRR6745880	MGYA00202962	https://www.ebi.ac.uk/metagenomics/api/v1/analyses/MGYA00202962/file/SRR6745880_MERGED_FASTQ_SSU_OTU.tsv	Root-Environmental-Aquatic-Marine-Coastal-Sediment
SRR1503981	MGYA00358536	https://www.ebi.ac.uk/metagenomics/api/v1/analyses/MGYA00358536/file/SRR1503981_FASTQ_SSU_OTU.tsv	Root-Environmental-Aquatic-Marine-Coastal-Sediment
SRR6745355	MGYA00202694	https://www.ebi.ac.uk/metagenomics/api/v1/analyses/MGYA00202694/file/SRR6745355_MERGED_FASTQ_SSU_OTU.tsv	Root-Environmental-Aquatic-Marine-Coastal-Sediment
SRR6746186	MGYA00202779	https://www.ebi.ac.uk/metagenomics/api/v1/analyses/MGYA00202779/file/SRR6746186_MERGED_FASTQ_SSU_OTU.tsv	Root-Environmental-Aquatic-Marine-Coastal-Sediment
SRR2856426	MGYA00359223	https://www.ebi.ac.uk/metagenomics/api/v1/analyses/MGYA00359223/file/SRR2856426_MERGED_FASTQ_SSU_OTU.tsv	Root-Environmental-Aquatic-Marine-Coastal-Sediment
SRR6745909	MGYA00203061	https://www.ebi.ac.uk/metagenomics/api/v1/analyses/MGYA00203061/file/SRR6745909_MERGED_FASTQ_SSU_OTU.tsv	Root-Environmental-Aquatic-Marine-Coastal-Sediment
SRR2297164	MGYA00358928	https://www.ebi.ac.uk/metagenomics/api/v1/analyses/MGYA00358928/file/SRR2297164_MERGED_FASTQ_SSU_OTU.tsv	Root-Environmental-Aquatic-Marine-Coastal-Sediment
SRR6746150	MGYA00202556	https://www.ebi.ac.uk/metagenomics/api/v1/analyses/MGYA00202556/file/SRR6746150_MERGED_FASTQ_SSU_OTU.tsv	Root-Environmental-Aquatic-Marine-Coastal-Sediment

SRR6745383	MGYA00203051	https://www.ebi.ac.uk/metagenomics/api/v1/analyses/MGYA00203051/file/SRR6745383_MERGED_FASTQ_SSU_OTU.tsv	Root-Environmental-Aquatic-Marine-Coastal-Sediment
SRR6745856	MGYA00202692	https://www.ebi.ac.uk/metagenomics/api/v1/analyses/MGYA00202692/file/SRR6745856_MERGED_FASTQ_SSU_OTU.tsv	Root-Environmental-Aquatic-Marine-Coastal-Sediment
SRR6745424	MGYA00202637	https://www.ebi.ac.uk/metagenomics/api/v1/analyses/MGYA00202637/file/SRR6745424_MERGED_FASTQ_SSU_OTU.tsv	Root-Environmental-Aquatic-Marine-Coastal-Sediment
SRR6746531	MGYA00202530	https://www.ebi.ac.uk/metagenomics/api/v1/analyses/MGYA00202530/file/SRR6746531_MERGED_FASTQ_SSU_OTU.tsv	Root-Environmental-Aquatic-Marine-Coastal-Sediment
SRR6745352	MGYA00202991	https://www.ebi.ac.uk/metagenomics/api/v1/analyses/MGYA00202991/file/SRR6745352_MERGED_FASTQ_SSU_OTU.tsv	Root-Environmental-Aquatic-Marine-Coastal-Sediment
SRR6745887	MGYA00202770	https://www.ebi.ac.uk/metagenomics/api/v1/analyses/MGYA00202770/file/SRR6745887_MERGED_FASTQ_SSU_OTU.tsv	Root-Environmental-Aquatic-Marine-Coastal-Sediment
SRR6746181	MGYA00202572	https://www.ebi.ac.uk/metagenomics/api/v1/analyses/MGYA00202572/file/SRR6746181_MERGED_FASTQ_SSU_OTU.tsv	Root-Environmental-Aquatic-Marine-Coastal-Sediment
SRR6746008	MGYA00202872	https://www.ebi.ac.uk/metagenomics/api/v1/analyses/MGYA00202872/file/SRR6746008_MERGED_FASTQ_SSU_OTU.tsv	Root-Environmental-Aquatic-Marine-Coastal-Sediment
SRR6746469	MGYA00203089	https://www.ebi.ac.uk/metagenomics/api/v1/analyses/MGYA00203089/file/SRR6746469_MERGED_FASTQ_SSU_OTU.tsv	Root-Environmental-Aquatic-Marine-Coastal-Sediment
SRR2297118	MGYA00358882	https://www.ebi.ac.uk/metagenomics/api/v1/analyses/MGYA00358882/file/SRR2297118_MERGED_FASTQ_SSU_OTU.tsv	Root-Environmental-Aquatic-Marine-Coastal-Sediment
SRR5097534	MGYA00360546	https://www.ebi.ac.uk/metagenomics/api/v1/analyses/MGYA00360546/file/SRR5097534_MERGED_FASTQ_SSU_OTU.tsv	Root-Environmental-Aquatic-Marine-Coastal-Sediment
SRR2713137	MGYA00355964	https://www.ebi.ac.uk/metagenomics/api/v1/analyses/MGYA00355964/file/SRR2713137_FASTQ_SSU_OTU.tsv	Root-Environmental-Aquatic-Marine-Coastal-Sediment

SRR5227104	MGYA00360663	https://www.ebi.ac.uk/metagenomics/api/v1/analyses/MGYA00360663/file/SRR5227104_FASTQ_SSU_OTU.tsv	Root-Environmental-Aquatic-Marine-Coastal-Sediment
SRR6746074	MGYA00203102	https://www.ebi.ac.uk/metagenomics/api/v1/analyses/MGYA00203102/file/SRR6746074_MERGED_FASTQ_SSU_OTU.tsv	Root-Environmental-Aquatic-Marine-Coastal-Sediment
SRR6745972	MGYA00203047	https://www.ebi.ac.uk/metagenomics/api/v1/analyses/MGYA00203047/file/SRR6745972_MERGED_FASTQ_SSU_OTU.tsv	Root-Environmental-Aquatic-Marine-Coastal-Sediment
SRR6745401	MGYA00203017	https://www.ebi.ac.uk/metagenomics/api/v1/analyses/MGYA00203017/file/SRR6745401_MERGED_FASTQ_SSU_OTU.tsv	Root-Environmental-Aquatic-Marine-Coastal-Sediment
SRR1503972	MGYA00358527	https://www.ebi.ac.uk/metagenomics/api/v1/analyses/MGYA00358527/file/SRR1503972_FASTQ_SSU_OTU.tsv	Root-Environmental-Aquatic-Marine-Coastal-Sediment
SRR6745873	MGYA00202881	https://www.ebi.ac.uk/metagenomics/api/v1/analyses/MGYA00202881/file/SRR6745873_MERGED_FASTQ_SSU_OTU.tsv	Root-Environmental-Aquatic-Marine-Coastal-Sediment
SRR6746514	MGYA00202849	https://www.ebi.ac.uk/metagenomics/api/v1/analyses/MGYA00202849/file/SRR6746514_MERGED_FASTQ_SSU_OTU.tsv	Root-Environmental-Aquatic-Marine-Coastal-Sediment
SRR6746175	MGYA00202665	https://www.ebi.ac.uk/metagenomics/api/v1/analyses/MGYA00202665/file/SRR6746175_MERGED_FASTQ_SSU_OTU.tsv	Root-Environmental-Aquatic-Marine-Coastal-Sediment
SRR2297141	MGYA00358905	https://www.ebi.ac.uk/metagenomics/api/v1/analyses/MGYA00358905/file/SRR2297141_MERGED_FASTQ_SSU_OTU.tsv	Root-Environmental-Aquatic-Marine-Coastal-Sediment
ERR330055	MGYA00358013	https://www.ebi.ac.uk/metagenomics/api/v1/analyses/MGYA00358013/file/ERR330055_FASTQ_SSU_OTU.tsv	Root-Environmental-Aquatic-Marine-Coastal-Sediment
SRR6172326	MGYA00362288	https://www.ebi.ac.uk/metagenomics/api/v1/analyses/MGYA00362288/file/SRR6172326_MERGED_FASTQ_SSU_OTU.tsv	Root-Environmental-Aquatic-Marine-Coastal-Sediment
SRR6745377	MGYA00202818	https://www.ebi.ac.uk/metagenomics/api/v1/analyses/MGYA00202818/file/SRR6745377_MERGED_FASTQ_SSU_OTU.tsv	Root-Environmental-Aquatic-Marine-Coastal-Sediment

SRR6746080	MGYA00202584	https://www.ebi.ac.uk/metagenomics/api/v1/analyses/MGYA00202584/file/SRR6746080_MERGED_FASTQ_SSU_OTU.tsv	Root-Environmental-Aquatic-Marine-Coastal-Sediment
SRR4436298	MGYA00213041	https://www.ebi.ac.uk/metagenomics/api/v1/analyses/MGYA00213041/file/SRR4436298_FASTQ_SSU_OTU.tsv	Root-Environmental-Aquatic-Marine-Coastal-Sediment
SRR6745986	MGYA00202582	https://www.ebi.ac.uk/metagenomics/api/v1/analyses/MGYA00202582/file/SRR6745986_MERGED_FASTQ_SSU_OTU.tsv	Root-Environmental-Aquatic-Marine-Coastal-Sediment
SRR6745957	MGYA00202713	https://www.ebi.ac.uk/metagenomics/api/v1/analyses/MGYA00202713/file/SRR6745957_MERGED_FASTQ_SSU_OTU.tsv	Root-Environmental-Aquatic-Marine-Coastal-Sediment
SRR6746051	MGYA00202796	https://www.ebi.ac.uk/metagenomics/api/v1/analyses/MGYA00202796/file/SRR6746051_MERGED_FASTQ_SSU_OTU.tsv	Root-Environmental-Aquatic-Marine-Coastal-Sediment
SRR6746568	MGYA00202718	https://www.ebi.ac.uk/metagenomics/api/v1/analyses/MGYA00202718/file/SRR6746568_MERGED_FASTQ_SSU_OTU.tsv	Root-Environmental-Aquatic-Marine-Coastal-Sediment
SRR2855896	MGYA00359216	https://www.ebi.ac.uk/metagenomics/api/v1/analyses/MGYA00359216/file/SRR2855896_MERGED_FASTQ_SSU_OTU.tsv	Root-Environmental-Aquatic-Marine-Coastal-Sediment
SRR6746109	MGYA00202911	https://www.ebi.ac.uk/metagenomics/api/v1/analyses/MGYA00202911/file/SRR6746109_MERGED_FASTQ_SSU_OTU.tsv	Root-Environmental-Aquatic-Marine-Coastal-Sediment
ERR330029	MGYA00357987	https://www.ebi.ac.uk/metagenomics/api/v1/analyses/MGYA00357987/file/ERR330029_FASTQ_SSU_OTU.tsv	Root-Environmental-Aquatic-Marine-Coastal-Sediment
SRR6746087	MGYA00202870	https://www.ebi.ac.uk/metagenomics/api/v1/analyses/MGYA00202870/file/SRR6746087_MERGED_FASTQ_SSU_OTU.tsv	Root-Environmental-Aquatic-Marine-Coastal-Sediment
SRR6745981	MGYA00202885	https://www.ebi.ac.uk/metagenomics/api/v1/analyses/MGYA00202885/file/SRR6745981_MERGED_FASTQ_SSU_OTU.tsv	Root-Environmental-Aquatic-Marine-Coastal-Sediment
SRR6745950	MGYA00203053	https://www.ebi.ac.uk/metagenomics/api/v1/analyses/MGYA00203053/file/SRR6745950_MERGED_FASTQ_SSU_OTU.tsv	Root-Environmental-Aquatic-Marine-Coastal-Sediment

SRR6746056	MGYA00203082	https://www.ebi.ac.uk/metagenomics/api/v1/analyses/MGYA00203082/file/SRR6746056_MERGED_FASTQ_SSU_OTU.tsv	Root-Environmental-Aquatic-Marine-Coastal-Sediment
SRR6745808	MGYA00203043	https://www.ebi.ac.uk/metagenomics/api/v1/analyses/MGYA00203043/file/SRR6745808_MERGED_FASTQ_SSU_OTU.tsv	Root-Environmental-Aquatic-Marine-Coastal-Sediment
SRR6746219	MGYA00202922	https://www.ebi.ac.uk/metagenomics/api/v1/analyses/MGYA00202922/file/SRR6746219_MERGED_FASTQ_SSU_OTU.tsv	Root-Environmental-Aquatic-Marine-Coastal-Sediment
SRR4300300	MGYA00360259	https://www.ebi.ac.uk/metagenomics/api/v1/analyses/MGYA00360259/file/SRR4300300_MERGED_FASTQ_SSU_OTU.tsv	Root-Environmental-Aquatic-Marine-Coastal-Sediment
SRR3354757	MGYA00359635	https://www.ebi.ac.uk/metagenomics/api/v1/analyses/MGYA00359635/file/SRR3354757_MERGED_FASTQ_SSU_OTU.tsv	Root-Environmental-Aquatic-Marine-Coastal-Sediment
SRR1503975	MGYA00358530	https://www.ebi.ac.uk/metagenomics/api/v1/analyses/MGYA00358530/file/SRR1503975_FASTQ_SSU_OTU.tsv	Root-Environmental-Aquatic-Marine-Coastal-Sediment
SRR6745874	MGYA00202733	https://www.ebi.ac.uk/metagenomics/api/v1/analyses/MGYA00202733/file/SRR6745874_MERGED_FASTQ_SSU_OTU.tsv	Root-Environmental-Aquatic-Marine-Coastal-Sediment
SRR6745406	MGYA00202971	https://www.ebi.ac.uk/metagenomics/api/v1/analyses/MGYA00202971/file/SRR6745406_MERGED_FASTQ_SSU_OTU.tsv	Root-Environmental-Aquatic-Marine-Coastal-Sediment
SRR6746513	MGYA00202539	https://www.ebi.ac.uk/metagenomics/api/v1/analyses/MGYA00202539/file/SRR6746513_MERGED_FASTQ_SSU_OTU.tsv	Root-Environmental-Aquatic-Marine-Coastal-Sediment
SRR2297146	MGYA00358910	https://www.ebi.ac.uk/metagenomics/api/v1/analyses/MGYA00358910/file/SRR2297146_MERGED_FASTQ_SSU_OTU.tsv	Root-Environmental-Aquatic-Marine-Coastal-Sediment
SRR6746172	MGYA00202666	https://www.ebi.ac.uk/metagenomics/api/v1/analyses/MGYA00202666/file/SRR6746172_MERGED_FASTQ_SSU_OTU.tsv	Root-Environmental-Aquatic-Marine-Coastal-Sediment
SRR4300289	MGYA00360249	https://www.ebi.ac.uk/metagenomics/api/v1/analyses/MGYA00360249/file/SRR4300289_MERGED_FASTQ_SSU_OTU.tsv	Root-Environmental-Aquatic-Marine-Coastal-Sediment

ERR330052	MGYA00358010	https://www.ebi.ac.uk/metagenomics/api/v1/analyses/MGYA00358010/file/ERR330052_FASTQ_SSU_OTU.tsv	Root-Environmental-Aquatic-Marine-Coastal-Sediment
SRR650829	MGYA00356178	https://www.ebi.ac.uk/metagenomics/api/v1/analyses/MGYA00356178/file/SRR650829_FASTQ_SSU_OTU.tsv	Root-Environmental-Aquatic-Marine-Coastal-Sediment
SRR6745370	MGYA00202655	https://www.ebi.ac.uk/metagenomics/api/v1/analyses/MGYA00202655/file/SRR6745370_MERGED_FASTQ_SSU_OTU.tsv	Root-Environmental-Aquatic-Marine-Coastal-Sediment
SRR6746089	MGYA00202603	https://www.ebi.ac.uk/metagenomics/api/v1/analyses/MGYA00202603/file/SRR6746089_MERGED_FASTQ_SSU_OTU.tsv	Root-Environmental-Aquatic-Marine-Coastal-Sediment
SRR4436291	MGYA00213057	https://www.ebi.ac.uk/metagenomics/api/v1/analyses/MGYA00213057/file/SRR4436291_FASTQ_SSU_OTU.tsv	Root-Environmental-Aquatic-Marine-Coastal-Sediment
SRR6746058	MGYA00202883	https://www.ebi.ac.uk/metagenomics/api/v1/analyses/MGYA00202883/file/SRR6746058_MERGED_FASTQ_SSU_OTU.tsv	Root-Environmental-Aquatic-Marine-Coastal-Sediment
SRR6745806	MGYA00202875	https://www.ebi.ac.uk/metagenomics/api/v1/analyses/MGYA00202875/file/SRR6745806_MERGED_FASTQ_SSU_OTU.tsv	Root-Environmental-Aquatic-Marine-Coastal-Sediment
SRR6746561	MGYA00203097	https://www.ebi.ac.uk/metagenomics/api/v1/analyses/MGYA00203097/file/SRR6746561_MERGED_FASTQ_SSU_OTU.tsv	Root-Environmental-Aquatic-Marine-Coastal-Sediment
SRR6746100	MGYA00202690	https://www.ebi.ac.uk/metagenomics/api/v1/analyses/MGYA00202690/file/SRR6746100_MERGED_FASTQ_SSU_OTU.tsv	Root-Environmental-Aquatic-Marine-Coastal-Sediment
SRR2297134	MGYA00358898	https://www.ebi.ac.uk/metagenomics/api/v1/analyses/MGYA00358898/file/SRR2297134_MERGED_FASTQ_SSU_OTU.tsv	Root-Environmental-Aquatic-Marine-Coastal-Sediment
ERR330020	MGYA00357978	https://www.ebi.ac.uk/metagenomics/api/v1/analyses/MGYA00357978/file/ERR330020_FASTQ_SSU_OTU.tsv	Root-Environmental-Aquatic-Marine-Coastal-Sediment
SRR6746217	MGYA00202640	https://www.ebi.ac.uk/metagenomics/api/v1/analyses/MGYA00202640/file/SRR6746217_MERGED_FASTQ_SSU_OTU.tsv	Root-Environmental-Aquatic-Marine-Coastal-Sediment

SRR6172353	MGYA00362315	https://www.ebi.ac.uk/metagenomics/api/v1/analyses/MGYA00362315/file/SRR6172353_MERGED_FASTQ_SSU_OTU.tsv	Root-Environmental-Aquatic-Marine-Coastal-Sediment
SRR6746494	MGYA00202597	https://www.ebi.ac.uk/metagenomics/api/v1/analyses/MGYA00202597/file/SRR6746494_MERGED_FASTQ_SSU_OTU.tsv	Root-Environmental-Aquatic-Marine-Coastal-Sediment
SRR6745922	MGYA00202996	https://www.ebi.ac.uk/metagenomics/api/v1/analyses/MGYA00202996/file/SRR6745922_MERGED_FASTQ_SSU_OTU.tsv	Root-Environmental-Aquatic-Marine-Coastal-Sediment
SRR6746024	MGYA00202874	https://www.ebi.ac.uk/metagenomics/api/v1/analyses/MGYA00202874/file/SRR6746024_MERGED_FASTQ_SSU_OTU.tsv	Root-Environmental-Aquatic-Marine-Coastal-Sediment
SRR6745408	MGYA00202491	https://www.ebi.ac.uk/metagenomics/api/v1/analyses/MGYA00202491/file/SRR6745408_MERGED_FASTQ_SSU_OTU.tsv	Root-Environmental-Aquatic-Marine-Coastal-Sediment
SRR2297148	MGYA00358912	https://www.ebi.ac.uk/metagenomics/api/v1/analyses/MGYA00358912/file/SRR2297148_MERGED_FASTQ_SSU_OTU.tsv	Root-Environmental-Aquatic-Marine-Coastal-Sediment
SRR4300287	MGYA00360247	https://www.ebi.ac.uk/metagenomics/api/v1/analyses/MGYA00360247/file/SRR4300287_MERGED_FASTQ_SSU_OTU.tsv	Root-Environmental-Aquatic-Marine-Coastal-Sediment
SRR6746493	MGYA00202571	https://www.ebi.ac.uk/metagenomics/api/v1/analyses/MGYA00202571/file/SRR6746493_MERGED_FASTQ_SSU_OTU.tsv	Root-Environmental-Aquatic-Marine-Coastal-Sediment
SRR6745925	MGYA00202823	https://www.ebi.ac.uk/metagenomics/api/v1/analyses/MGYA00202823/file/SRR6745925_MERGED_FASTQ_SSU_OTU.tsv	Root-Environmental-Aquatic-Marine-Coastal-Sediment
SRR6746023	MGYA00202928	https://www.ebi.ac.uk/metagenomics/api/v1/analyses/MGYA00202928/file/SRR6746023_MERGED_FASTQ_SSU_OTU.tsv	Root-Environmental-Aquatic-Marine-Coastal-Sediment
SRR4300280	MGYA00360241	https://www.ebi.ac.uk/metagenomics/api/v1/analyses/MGYA00360241/file/SRR4300280_MERGED_FASTQ_SSU_OTU.tsv	Root-Environmental-Aquatic-Marine-Coastal-Sediment
SRR6172328	MGYA00362290	https://www.ebi.ac.uk/metagenomics/api/v1/analyses/MGYA00362290/file/SRR6172328_MERGED_FASTQ_SSU_OTU.tsv	Root-Environmental-Aquatic-Marine-Coastal-Sediment

SRR6745379	MGYA00202952	https://www.ebi.ac.uk/metagenomics/api/v1/analyses/MGYA00202952/file/SRR6745379_MERGED_FASTQ_SSU_OTU.tsv	Root-Environmental-Aquatic-Marine-Coastal-Sediment
SRR6745988	MGYA00203108	https://www.ebi.ac.uk/metagenomics/api/v1/analyses/MGYA00203108/file/SRR6745988_MERGED_FASTQ_SSU_OTU.tsv	Root-Environmental-Aquatic-Marine-Coastal-Sediment
SRR4436296	MGYA00213056	https://www.ebi.ac.uk/metagenomics/api/v1/analyses/MGYA00213056/file/SRR4436296_FASTQ_SSU_OTU.tsv	Root-Environmental-Aquatic-Marine-Coastal-Sediment
SRR6745959	MGYA00202564	https://www.ebi.ac.uk/metagenomics/api/v1/analyses/MGYA00202564/file/SRR6745959_MERGED_FASTQ_SSU_OTU.tsv	Root-Environmental-Aquatic-Marine-Coastal-Sediment
SRR2713095	MGYA00355962	https://www.ebi.ac.uk/metagenomics/api/v1/analyses/MGYA00355962/file/SRR2713095_FASTQ_SSU_OTU.tsv	Root-Environmental-Aquatic-Marine-Coastal-Sediment
SRR6745801	MGYA00203110	https://www.ebi.ac.uk/metagenomics/api/v1/analyses/MGYA00203110/file/SRR6745801_MERGED_FASTQ_SSU_OTU.tsv	Root-Environmental-Aquatic-Marine-Coastal-Sediment
SRR6746566	MGYA00202631	https://www.ebi.ac.uk/metagenomics/api/v1/analyses/MGYA00202631/file/SRR6746566_MERGED_FASTQ_SSU_OTU.tsv	Root-Environmental-Aquatic-Marine-Coastal-Sediment
SRR2297133	MGYA00358897	https://www.ebi.ac.uk/metagenomics/api/v1/analyses/MGYA00358897/file/SRR2297133_MERGED_FASTQ_SSU_OTU.tsv	Root-Environmental-Aquatic-Marine-Coastal-Sediment
SRR6746107	MGYA00203091	https://www.ebi.ac.uk/metagenomics/api/v1/analyses/MGYA00203091/file/SRR6746107_MERGED_FASTQ_SSU_OTU.tsv	Root-Environmental-Aquatic-Marine-Coastal-Sediment
ERR330027	MGYA00357985	https://www.ebi.ac.uk/metagenomics/api/v1/analyses/MGYA00357985/file/ERR330027_FASTQ_SSU_OTU.tsv	Root-Environmental-Aquatic-Marine-Coastal-Sediment
SRR6746210	MGYA00202732	https://www.ebi.ac.uk/metagenomics/api/v1/analyses/MGYA00202732/file/SRR6746210_MERGED_FASTQ_SSU_OTU.tsv	Root-Environmental-Aquatic-Marine-Coastal-Sediment
SRR6172354	MGYA00362316	https://www.ebi.ac.uk/metagenomics/api/v1/analyses/MGYA00362316/file/SRR6172354_MERGED_FASTQ_SSU_OTU.tsv	Root-Environmental-Aquatic-Marine-Coastal-Sediment

SRR6746016	MGYA00202968	https://www.ebi.ac.uk/metagenomics/api/v1/analyses/MGYA00202968/file/SRR6746016_MERGED_FASTQ_SSU_OTU.tsv	Root-Environmental-Aquatic-Marine-Coastal-Sediment
SRR6745910	MGYA00202607	https://www.ebi.ac.uk/metagenomics/api/v1/analyses/MGYA00202607/file/SRR6745910_MERGED_FASTQ_SSU_OTU.tsv	Root-Environmental-Aquatic-Marine-Coastal-Sediment
SRR6746477	MGYA00202788	https://www.ebi.ac.uk/metagenomics/api/v1/analyses/MGYA00202788/file/SRR6746477_MERGED_FASTQ_SSU_OTU.tsv	Root-Environmental-Aquatic-Marine-Coastal-Sediment
SRR3358601	MGYA00364489	https://www.ebi.ac.uk/metagenomics/api/v1/analyses/MGYA00364489/file/SRR3358601_FASTQ_SSU_OTU.tsv	Root-Environmental-Aquatic-Marine-Coastal-Sediment
SRR6745848	MGYA00202882	https://www.ebi.ac.uk/metagenomics/api/v1/analyses/MGYA00202882/file/SRR6745848_MERGED_FASTQ_SSU_OTU.tsv	Root-Environmental-Aquatic-Marine-Coastal-Sediment
SRR6172361	MGYA00362323	https://www.ebi.ac.uk/metagenomics/api/v1/analyses/MGYA00362323/file/SRR6172361_MERGED_FASTQ_SSU_OTU.tsv	Root-Environmental-Aquatic-Marine-Coastal-Sediment
SRR6746132	MGYA00202919	https://www.ebi.ac.uk/metagenomics/api/v1/analyses/MGYA00202919/file/SRR6746132_MERGED_FASTQ_SSU_OTU.tsv	Root-Environmental-Aquatic-Marine-Coastal-Sediment
SRR2297106	MGYA00358870	https://www.ebi.ac.uk/metagenomics/api/v1/analyses/MGYA00358870/file/SRR2297106_MERGED_FASTQ_SSU_OTU.tsv	Root-Environmental-Aquatic-Marine-Coastal-Sediment
SRR6745834	MGYA00202642	https://www.ebi.ac.uk/metagenomics/api/v1/analyses/MGYA00202642/file/SRR6745834_MERGED_FASTQ_SSU_OTU.tsv	Root-Environmental-Aquatic-Marine-Coastal-Sediment
SRR6746553	MGYA00202784	https://www.ebi.ac.uk/metagenomics/api/v1/analyses/MGYA00202784/file/SRR6746553_MERGED_FASTQ_SSU_OTU.tsv	Root-Environmental-Aquatic-Marine-Coastal-Sediment
SRR6745337	MGYA00202856	https://www.ebi.ac.uk/metagenomics/api/v1/analyses/MGYA00202856/file/SRR6745337_MERGED_FASTQ_SSU_OTU.tsv	Root-Environmental-Aquatic-Marine-Coastal-Sediment
SRR3355070	MGYA00359643	https://www.ebi.ac.uk/metagenomics/api/v1/analyses/MGYA00359643/file/SRR3355070_MERGED_FASTQ_SSU_OTU.tsv	Root-Environmental-Aquatic-Marine-Coastal-Sediment

SRR6746135	MGYA00202854	https://www.ebi.ac.uk/metagenomics/api/v1/analyses/MGYA00202854/file/SRR6746135_MERGED_FASTQ_SSU_OTU.tsv	Root-Environmental-Aquatic-Marine-Coastal-Sediment
SRR6745833	MGYA00202662	https://www.ebi.ac.uk/metagenomics/api/v1/analyses/MGYA00202662/file/SRR6745833_MERGED_FASTQ_SSU_OTU.tsv	Root-Environmental-Aquatic-Marine-Coastal-Sediment
SRR6746554	MGYA00203058	https://www.ebi.ac.uk/metagenomics/api/v1/analyses/MGYA00203058/file/SRR6746554_MERGED_FASTQ_SSU_OTU.tsv	Root-Environmental-Aquatic-Marine-Coastal-Sediment
SRR6746011	MGYA00202712	https://www.ebi.ac.uk/metagenomics/api/v1/analyses/MGYA00202712/file/SRR6746011_MERGED_FASTQ_SSU_OTU.tsv	Root-Environmental-Aquatic-Marine-Coastal-Sediment
SRR6745917	MGYA00202778	https://www.ebi.ac.uk/metagenomics/api/v1/analyses/MGYA00202778/file/SRR6745917_MERGED_FASTQ_SSU_OTU.tsv	Root-Environmental-Aquatic-Marine-Coastal-Sediment
SRR6746470	MGYA00202587	https://www.ebi.ac.uk/metagenomics/api/v1/analyses/MGYA00202587/file/SRR6746470_MERGED_FASTQ_SSU_OTU.tsv	Root-Environmental-Aquatic-Marine-Coastal-Sediment
SRR6746198	MGYA00202581	https://www.ebi.ac.uk/metagenomics/api/v1/analyses/MGYA00202581/file/SRR6746198_MERGED_FASTQ_SSU_OTU.tsv	Root-Environmental-Aquatic-Marine-Coastal-Sediment
SRR6746149	MGYA00202677	https://www.ebi.ac.uk/metagenomics/api/v1/analyses/MGYA00202677/file/SRR6746149_MERGED_FASTQ_SSU_OTU.tsv	Root-Environmental-Aquatic-Marine-Coastal-Sediment
SRR6746528	MGYA00202891	https://www.ebi.ac.uk/metagenomics/api/v1/analyses/MGYA00202891/file/SRR6746528_MERGED_FASTQ_SSU_OTU.tsv	Root-Environmental-Aquatic-Marine-Coastal-Sediment
SRR6746063	MGYA00202899	https://www.ebi.ac.uk/metagenomics/api/v1/analyses/MGYA00202899/file/SRR6746063_MERGED_FASTQ_SSU_OTU.tsv	Root-Environmental-Aquatic-Marine-Coastal-Sediment
SRR6745965	MGYA00202896	https://www.ebi.ac.uk/metagenomics/api/v1/analyses/MGYA00202896/file/SRR6745965_MERGED_FASTQ_SSU_OTU.tsv	Root-Environmental-Aquatic-Marine-Coastal-Sediment
ERR3063408	MGYA00293719	https://www.ebi.ac.uk/metagenomics/api/v1/analyses/MGYA00293719/file/ERR3063408_FASTQ_SSU_OTU.tsv	Root-Environmental-Aquatic-Marine-Coastal-Sediment

SRR6745339	MGYA00202513	https://www.ebi.ac.uk/metagenomics/api/v1/analyses/MGYA00202513/file/SRR6745339_MERGED_FASTQ_SSU_OTU.tsv	Root-Environmental-Aquatic-Marine-Coastal-Sediment
SRR5227113	MGYA00360672	https://www.ebi.ac.uk/metagenomics/api/v1/analyses/MGYA00360672/file/SRR5227113_FASTQ_SSU_OTU.tsv	Root-Environmental-Aquatic-Marine-Coastal-Sediment
SRR6745789	MGYA00202628	https://www.ebi.ac.uk/metagenomics/api/v1/analyses/MGYA00202628/file/SRR6745789_MERGED_FASTQ_SSU_OTU.tsv	Root-Environmental-Aquatic-Marine-Coastal-Sediment
SRR2712921	MGYA00355959	https://www.ebi.ac.uk/metagenomics/api/v1/analyses/MGYA00355959/file/SRR2712921_FASTQ_SSU_OTU.tsv	Root-Environmental-Aquatic-Marine-Coastal-Sediment
SRR6745919	MGYA00202835	https://www.ebi.ac.uk/metagenomics/api/v1/analyses/MGYA00202835/file/SRR6745919_MERGED_FASTQ_SSU_OTU.tsv	Root-Environmental-Aquatic-Marine-Coastal-Sediment
SRR6745890	MGYA00202798	https://www.ebi.ac.uk/metagenomics/api/v1/analyses/MGYA00202798/file/SRR6745890_MERGED_FASTQ_SSU_OTU.tsv	Root-Environmental-Aquatic-Marine-Coastal-Sediment
SRR6745345	MGYA00202575	https://www.ebi.ac.uk/metagenomics/api/v1/analyses/MGYA00202575/file/SRR6745345_MERGED_FASTQ_SSU_OTU.tsv	Root-Environmental-Aquatic-Marine-Coastal-Sediment
SRR6746196	MGYA00202810	https://www.ebi.ac.uk/metagenomics/api/v1/analyses/MGYA00202810/file/SRR6746196_MERGED_FASTQ_SSU_OTU.tsv	Root-Environmental-Aquatic-Marine-Coastal-Sediment
SRR6746147	MGYA00203072	https://www.ebi.ac.uk/metagenomics/api/v1/analyses/MGYA00203072/file/SRR6746147_MERGED_FASTQ_SSU_OTU.tsv	Root-Environmental-Aquatic-Marine-Coastal-Sediment
SRR2297173	MGYA00358937	https://www.ebi.ac.uk/metagenomics/api/v1/analyses/MGYA00358937/file/SRR2297173_MERGED_FASTQ_SSU_OTU.tsv	Root-Environmental-Aquatic-Marine-Coastal-Sediment
SRR6745841	MGYA00203013	https://www.ebi.ac.uk/metagenomics/api/v1/analyses/MGYA00203013/file/SRR6745841_MERGED_FASTQ_SSU_OTU.tsv	Root-Environmental-Aquatic-Marine-Coastal-Sediment
SRR6745433	MGYA00202858	https://www.ebi.ac.uk/metagenomics/api/v1/analyses/MGYA00202858/file/SRR6745433_MERGED_FASTQ_SSU_OTU.tsv	Root-Environmental-Aquatic-Marine-Coastal-Sediment

SRR6745394	MGYA00202618	https://www.ebi.ac.uk/metagenomics/api/v1/analyses/MGYA00202618/file/SRR6745394_MERGED_FASTQ_SSU_OTU.tsv	Root-Environmental-Aquatic-Marine-Coastal-Sediment
SRR6746526	MGYA00202726	https://www.ebi.ac.uk/metagenomics/api/v1/analyses/MGYA00202726/file/SRR6746526_MERGED_FASTQ_SSU_OTU.tsv	Root-Environmental-Aquatic-Marine-Coastal-Sediment
SRR6746018	MGYA00202494	https://www.ebi.ac.uk/metagenomics/api/v1/analyses/MGYA00202494/file/SRR6746018_MERGED_FASTQ_SSU_OTU.tsv	Root-Environmental-Aquatic-Marine-Coastal-Sediment
SRR6746479	MGYA00203012	https://www.ebi.ac.uk/metagenomics/api/v1/analyses/MGYA00203012/file/SRR6746479_MERGED_FASTQ_SSU_OTU.tsv	Root-Environmental-Aquatic-Marine-Coastal-Sediment
SRR3211973	MGYA00357597	https://www.ebi.ac.uk/metagenomics/api/v1/analyses/MGYA00357597/file/SRR3211973_MERGED_FASTQ_SSU_OTU.tsv	Root-Environmental-Aquatic-Marine-Coastal-Sediment
SRR6745342	MGYA00203104	https://www.ebi.ac.uk/metagenomics/api/v1/analyses/MGYA00203104/file/SRR6745342_MERGED_FASTQ_SSU_OTU.tsv	Root-Environmental-Aquatic-Marine-Coastal-Sediment
SRR6746191	MGYA00203029	https://www.ebi.ac.uk/metagenomics/api/v1/analyses/MGYA00203029/file/SRR6746191_MERGED_FASTQ_SSU_OTU.tsv	Root-Environmental-Aquatic-Marine-Coastal-Sediment
SRR2297174	MGYA00358938	https://www.ebi.ac.uk/metagenomics/api/v1/analyses/MGYA00358938/file/SRR2297174_MERGED_FASTQ_SSU_OTU.tsv	Root-Environmental-Aquatic-Marine-Coastal-Sediment
SRR6746140	MGYA00202522	https://www.ebi.ac.uk/metagenomics/api/v1/analyses/MGYA00202522/file/SRR6746140_MERGED_FASTQ_SSU_OTU.tsv	Root-Environmental-Aquatic-Marine-Coastal-Sediment
SRR3354989	MGYA00359636	https://www.ebi.ac.uk/metagenomics/api/v1/analyses/MGYA00359636/file/SRR3354989_MERGED_FASTQ_SSU_OTU.tsv	Root-Environmental-Aquatic-Marine-Coastal-Sediment
SRR6745393	MGYA00202595	https://www.ebi.ac.uk/metagenomics/api/v1/analyses/MGYA00202595/file/SRR6745393_MERGED_FASTQ_SSU_OTU.tsv	Root-Environmental-Aquatic-Marine-Coastal-Sediment
SRR6745434	MGYA00202834	https://www.ebi.ac.uk/metagenomics/api/v1/analyses/MGYA00202834/file/SRR6745434_MERGED_FASTQ_SSU_OTU.tsv	Root-Environmental-Aquatic-Marine-Coastal-Sediment

SRR6745846	MGYA00202902	https://www.ebi.ac.uk/metagenomics/api/v1/analyses/MGYA00202902/file/SRR6745846_MERGED_FASTQ_SSU_OTU.tsv	Root-Environmental-Aquatic-Marine-Coastal-Sediment
SRR6746521	MGYA00202747	https://www.ebi.ac.uk/metagenomics/api/v1/analyses/MGYA00202747/file/SRR6746521_MERGED_FASTQ_SSU_OTU.tsv	Root-Environmental-Aquatic-Marine-Coastal-Sediment
SRR6746064	MGYA00202675	https://www.ebi.ac.uk/metagenomics/api/v1/analyses/MGYA00202675/file/SRR6746064_MERGED_FASTQ_SSU_OTU.tsv	Root-Environmental-Aquatic-Marine-Coastal-Sediment
SRR6745962	MGYA00202765	https://www.ebi.ac.uk/metagenomics/api/v1/analyses/MGYA00202765/file/SRR6745962_MERGED_FASTQ_SSU_OTU.tsv	Root-Environmental-Aquatic-Marine-Coastal-Sediment
SRR5227114	MGYA00360673	https://www.ebi.ac.uk/metagenomics/api/v1/analyses/MGYA00360673/file/SRR5227114_FASTQ_SSU_OTU.tsv	Root-Environmental-Aquatic-Marine-Coastal-Sediment
SRR2297108	MGYA00358872	https://www.ebi.ac.uk/metagenomics/api/v1/analyses/MGYA00358872/file/SRR2297108_MERGED_FASTQ_SSU_OTU.tsv	Root-Environmental-Aquatic-Marine-Coastal-Sediment
SRR6746151	MGYA00202853	https://www.ebi.ac.uk/metagenomics/api/v1/analyses/MGYA00202853/file/SRR6746151_MERGED_FASTQ_SSU_OTU.tsv	Root-Environmental-Aquatic-Marine-Coastal-Sediment
SRR2297165	MGYA00358929	https://www.ebi.ac.uk/metagenomics/api/v1/analyses/MGYA00358929/file/SRR2297165_MERGED_FASTQ_SSU_OTU.tsv	Root-Environmental-Aquatic-Marine-Coastal-Sediment
SRR6746530	MGYA00202970	https://www.ebi.ac.uk/metagenomics/api/v1/analyses/MGYA00202970/file/SRR6746530_MERGED_FASTQ_SSU_OTU.tsv	Root-Environmental-Aquatic-Marine-Coastal-Sediment
SRR6745857	MGYA00202627	https://www.ebi.ac.uk/metagenomics/api/v1/analyses/MGYA00202627/file/SRR6745857_MERGED_FASTQ_SSU_OTU.tsv	Root-Environmental-Aquatic-Marine-Coastal-Sediment
SRR6745425	MGYA00202846	https://www.ebi.ac.uk/metagenomics/api/v1/analyses/MGYA00202846/file/SRR6745425_MERGED_FASTQ_SSU_OTU.tsv	Root-Environmental-Aquatic-Marine-Coastal-Sediment
SRR6745382	MGYA00202777	https://www.ebi.ac.uk/metagenomics/api/v1/analyses/MGYA00202777/file/SRR6745382_MERGED_FASTQ_SSU_OTU.tsv	Root-Environmental-Aquatic-Marine-Coastal-Sediment

SRR6745886	MGYA00202957	https://www.ebi.ac.uk/metagenomics/api/v1/analyses/MGYA00202957/file/SRR6745886_MERGED_FASTQ_SSU_OTU.tsv	Root-Environmental-Aquatic-Marine-Coastal-Sediment
SRR6745353	MGYA00202738	https://www.ebi.ac.uk/metagenomics/api/v1/analyses/MGYA00202738/file/SRR6745353_MERGED_FASTQ_SSU_OTU.tsv	Root-Environmental-Aquatic-Marine-Coastal-Sediment
SRR6746180	MGYA00202502	https://www.ebi.ac.uk/metagenomics/api/v1/analyses/MGYA00202502/file/SRR6746180_MERGED_FASTQ_SSU_OTU.tsv	Root-Environmental-Aquatic-Marine-Coastal-Sediment
SRR6746009	MGYA00202734	https://www.ebi.ac.uk/metagenomics/api/v1/analyses/MGYA00202734/file/SRR6746009_MERGED_FASTQ_SSU_OTU.tsv	Root-Environmental-Aquatic-Marine-Coastal-Sediment
SRR6746468	MGYA00202687	https://www.ebi.ac.uk/metagenomics/api/v1/analyses/MGYA00202687/file/SRR6746468_MERGED_FASTQ_SSU_OTU.tsv	Root-Environmental-Aquatic-Marine-Coastal-Sediment
SRR2297119	MGYA00358883	https://www.ebi.ac.uk/metagenomics/api/v1/analyses/MGYA00358883/file/SRR2297119_MERGED_FASTQ_SSU_OTU.tsv	Root-Environmental-Aquatic-Marine-Coastal-Sediment
SRR5097535	MGYA00360547	https://www.ebi.ac.uk/metagenomics/api/v1/analyses/MGYA00360547/file/SRR5097535_MERGED_FASTQ_SSU_OTU.tsv	Root-Environmental-Aquatic-Marine-Coastal-Sediment
SRR2713136	MGYA00355963	https://www.ebi.ac.uk/metagenomics/api/v1/analyses/MGYA00355963/file/SRR2713136_FASTQ_SSU_OTU.tsv	Root-Environmental-Aquatic-Marine-Coastal-Sediment
SRR5227105	MGYA00360664	https://www.ebi.ac.uk/metagenomics/api/v1/analyses/MGYA00360664/file/SRR5227105_FASTQ_SSU_OTU.tsv	Root-Environmental-Aquatic-Marine-Coastal-Sediment
SRR6746075	MGYA00202652	https://www.ebi.ac.uk/metagenomics/api/v1/analyses/MGYA00202652/file/SRR6746075_MERGED_FASTQ_SSU_OTU.tsv	Root-Environmental-Aquatic-Marine-Coastal-Sediment
SRR6745973	MGYA00202753	https://www.ebi.ac.uk/metagenomics/api/v1/analyses/MGYA00202753/file/SRR6745973_MERGED_FASTQ_SSU_OTU.tsv	Root-Environmental-Aquatic-Marine-Coastal-Sediment
SRR6745798	MGYA00202654	https://www.ebi.ac.uk/metagenomics/api/v1/analyses/MGYA00202654/file/SRR6745798_MERGED_FASTQ_SSU_OTU.tsv	Root-Environmental-Aquatic-Marine-Coastal-Sediment

SRR5097532	MGYA00360544	https://www.ebi.ac.uk/metagenomics/api/v1/analyses/MGYA00360544/file/SRR5097532_MERGED_FASTQ_SSU_OTU.tsv	Root-Environmental-Aquatic-Marine-Coastal-Sediment
SRR5227102	MGYA00360661	https://www.ebi.ac.uk/metagenomics/api/v1/analyses/MGYA00360661/file/SRR5227102_FASTQ_SSU_OTU.tsv	Root-Environmental-Aquatic-Marine-Coastal-Sediment
SRR6746072	MGYA00202601	https://www.ebi.ac.uk/metagenomics/api/v1/analyses/MGYA00202601/file/SRR6746072_MERGED_FASTQ_SSU_OTU.tsv	Root-Environmental-Aquatic-Marine-Coastal-Sediment
SRR6745974	MGYA00203098	https://www.ebi.ac.uk/metagenomics/api/v1/analyses/MGYA00203098/file/SRR6745974_MERGED_FASTQ_SSU_OTU.tsv	Root-Environmental-Aquatic-Marine-Coastal-Sediment
SRR2297162	MGYA00358926	https://www.ebi.ac.uk/metagenomics/api/v1/analyses/MGYA00358926/file/SRR2297162_MERGED_FASTQ_SSU_OTU.tsv	Root-Environmental-Aquatic-Marine-Coastal-Sediment
SRR6746156	MGYA00202577	https://www.ebi.ac.uk/metagenomics/api/v1/analyses/MGYA00202577/file/SRR6746156_MERGED_FASTQ_SSU_OTU.tsv	Root-Environmental-Aquatic-Marine-Coastal-Sediment
SRR6746537	MGYA00202954	https://www.ebi.ac.uk/metagenomics/api/v1/analyses/MGYA00202954/file/SRR6746537_MERGED_FASTQ_SSU_OTU.tsv	Root-Environmental-Aquatic-Marine-Coastal-Sediment
SRR6745385	MGYA00202649	https://www.ebi.ac.uk/metagenomics/api/v1/analyses/MGYA00202649/file/SRR6745385_MERGED_FASTQ_SSU_OTU.tsv	Root-Environmental-Aquatic-Marine-Coastal-Sediment
SRR6745422	MGYA00202894	https://www.ebi.ac.uk/metagenomics/api/v1/analyses/MGYA00202894/file/SRR6745422_MERGED_FASTQ_SSU_OTU.tsv	Root-Environmental-Aquatic-Marine-Coastal-Sediment
SRR6745850	MGYA00202774	https://www.ebi.ac.uk/metagenomics/api/v1/analyses/MGYA00202774/file/SRR6745850_MERGED_FASTQ_SSU_OTU.tsv	Root-Environmental-Aquatic-Marine-Coastal-Sediment
SRR6745354	MGYA00203093	https://www.ebi.ac.uk/metagenomics/api/v1/analyses/MGYA00203093/file/SRR6745354_MERGED_FASTQ_SSU_OTU.tsv	Root-Environmental-Aquatic-Marine-Coastal-Sediment
SRR6745881	MGYA00202830	https://www.ebi.ac.uk/metagenomics/api/v1/analyses/MGYA00202830/file/SRR6745881_MERGED_FASTQ_SSU_OTU.tsv	Root-Environmental-Aquatic-Marine-Coastal-Sediment

SRR1503980	MGYA00358535	https://www.ebi.ac.uk/metagenomics/api/v1/analyses/MGYA00358535/file/SRR1503980_FASTQ_SSU_OTU.tsv	Root-Environmental-Aquatic-Marine-Coastal-Sediment
SRR6746187	MGYA00202893	https://www.ebi.ac.uk/metagenomics/api/v1/analyses/MGYA00202893/file/SRR6746187_MERGED_FASTQ_SSU_OTU.tsv	Root-Environmental-Aquatic-Marine-Coastal-Sediment
SRR6745908	MGYA00203073	https://www.ebi.ac.uk/metagenomics/api/v1/analyses/MGYA00203073/file/SRR6745908_MERGED_FASTQ_SSU_OTU.tsv	Root-Environmental-Aquatic-Marine-Coastal-Sediment
SRR6746124	MGYA00202837	https://www.ebi.ac.uk/metagenomics/api/v1/analyses/MGYA00202837/file/SRR6746124_MERGED_FASTQ_SSU_OTU.tsv	Root-Environmental-Aquatic-Marine-Coastal-Sediment
SRR2297110	MGYA00358874	https://www.ebi.ac.uk/metagenomics/api/v1/analyses/MGYA00358874/file/SRR2297110_MERGED_FASTQ_SSU_OTU.tsv	Root-Environmental-Aquatic-Marine-Coastal-Sediment
SRR6745796	MGYA00202897	https://www.ebi.ac.uk/metagenomics/api/v1/analyses/MGYA00202897/file/SRR6745796_MERGED_FASTQ_SSU_OTU.tsv	Root-Environmental-Aquatic-Marine-Coastal-Sediment
SRR6746545	MGYA00202886	https://www.ebi.ac.uk/metagenomics/api/v1/analyses/MGYA00202886/file/SRR6746545_MERGED_FASTQ_SSU_OTU.tsv	Root-Environmental-Aquatic-Marine-Coastal-Sediment
SRR6745822	MGYA00202795	https://www.ebi.ac.uk/metagenomics/api/v1/analyses/MGYA00202795/file/SRR6745822_MERGED_FASTQ_SSU_OTU.tsv	Root-Environmental-Aquatic-Marine-Coastal-Sediment
SRR2856455	MGYA00359230	https://www.ebi.ac.uk/metagenomics/api/v1/analyses/MGYA00359230/file/SRR2856455_MERGED_FASTQ_SSU_OTU.tsv	Root-Environmental-Aquatic-Marine-Coastal-Sediment
SRR650766	MGYA00356176	https://www.ebi.ac.uk/metagenomics/api/v1/analyses/MGYA00356176/file/SRR650766_FASTQ_SSU_OTU.tsv	Root-Environmental-Aquatic-Marine-Coastal-Sediment
SRR6746158	MGYA00203049	https://www.ebi.ac.uk/metagenomics/api/v1/analyses/MGYA00203049/file/SRR6746158_MERGED_FASTQ_SSU_OTU.tsv	Root-Environmental-Aquatic-Marine-Coastal-Sediment
SRR5097540	MGYA00360552	https://www.ebi.ac.uk/metagenomics/api/v1/analyses/MGYA00360552/file/SRR5097540_MERGED_FASTQ_SSU_OTU.tsv	Root-Environmental-Aquatic-Marine-Coastal-Sediment

SRR6746539	MGYA00202708	https://www.ebi.ac.uk/metagenomics/api/v1/analyses/MGYA00202708/file/SRR6746539_MERGED_FASTQ_SSU_OTU.tsv	Root-Environmental-Aquatic-Marine-Coastal-Sediment
SRR6746189	MGYA00202755	https://www.ebi.ac.uk/metagenomics/api/v1/analyses/MGYA00202755/file/SRR6746189_MERGED_FASTQ_SSU_OTU.tsv	Root-Environmental-Aquatic-Marine-Coastal-Sediment
SRR6746000	MGYA00202668	https://www.ebi.ac.uk/metagenomics/api/v1/analyses/MGYA00202668/file/SRR6746000_MERGED_FASTQ_SSU_OTU.tsv	Root-Environmental-Aquatic-Marine-Coastal-Sediment
SRR6745906	MGYA00202988	https://www.ebi.ac.uk/metagenomics/api/v1/analyses/MGYA00202988/file/SRR6745906_MERGED_FASTQ_SSU_OTU.tsv	Root-Environmental-Aquatic-Marine-Coastal-Sediment
SRR4300275	MGYA00360236	https://www.ebi.ac.uk/metagenomics/api/v1/analyses/MGYA00360236/file/SRR4300275_MERGED_FASTQ_SSU_OTU.tsv	Root-Environmental-Aquatic-Marine-Coastal-Sediment
SRR6745859	MGYA00202843	https://www.ebi.ac.uk/metagenomics/api/v1/analyses/MGYA00202843/file/SRR6745859_MERGED_FASTQ_SSU_OTU.tsv	Root-Environmental-Aquatic-Marine-Coastal-Sediment
SRR6745888	MGYA00202579	https://www.ebi.ac.uk/metagenomics/api/v1/analyses/MGYA00202579/file/SRR6745888_MERGED_FASTQ_SSU_OTU.tsv	Root-Environmental-Aquatic-Marine-Coastal-Sediment
SRR6746007	MGYA00202555	https://www.ebi.ac.uk/metagenomics/api/v1/analyses/MGYA00202555/file/SRR6746007_MERGED_FASTQ_SSU_OTU.tsv	Root-Environmental-Aquatic-Marine-Coastal-Sediment
SRR2297117	MGYA00358881	https://www.ebi.ac.uk/metagenomics/api/v1/analyses/MGYA00358881/file/SRR2297117_MERGED_FASTQ_SSU_OTU.tsv	Root-Environmental-Aquatic-Marine-Coastal-Sediment
SRR6746123	MGYA00202651	https://www.ebi.ac.uk/metagenomics/api/v1/analyses/MGYA00202651/file/SRR6746123_MERGED_FASTQ_SSU_OTU.tsv	Root-Environmental-Aquatic-Marine-Coastal-Sediment
SRR6745791	MGYA00202565	https://www.ebi.ac.uk/metagenomics/api/v1/analyses/MGYA00202565/file/SRR6745791_MERGED_FASTQ_SSU_OTU.tsv	Root-Environmental-Aquatic-Marine-Coastal-Sediment
SRR6746542	MGYA00202525	https://www.ebi.ac.uk/metagenomics/api/v1/analyses/MGYA00202525/file/SRR6746542_MERGED_FASTQ_SSU_OTU.tsv	Root-Environmental-Aquatic-Marine-Coastal-Sediment

SRR6745825	MGYA00202829	https://www.ebi.ac.uk/metagenomics/api/v1/analyses/MGYA00202829/file/SRR6745825_MERGED_FASTQ_SSU_OTU.tsv	Root-Environmental-Aquatic-Marine-Coastal-Sediment
SRR2713138	MGYA00355965	https://www.ebi.ac.uk/metagenomics/api/v1/analyses/MGYA00355965/file/SRR2713138_FASTQ_SSU_OTU.tsv	Root-Environmental-Aquatic-Marine-Coastal-Sediment
SRR4300291	MGYA00360251	https://www.ebi.ac.uk/metagenomics/api/v1/analyses/MGYA00360251/file/SRR4300291_MERGED_FASTQ_SSU_OTU.tsv	Root-Environmental-Aquatic-Marine-Coastal-Sediment
SRR650831	MGYA00356180	https://www.ebi.ac.uk/metagenomics/api/v1/analyses/MGYA00356180/file/SRR650831_FASTQ_SSU_OTU.tsv	Root-Environmental-Aquatic-Marine-Coastal-Sediment
SRR6745368	MGYA00202890	https://www.ebi.ac.uk/metagenomics/api/v1/analyses/MGYA00202890/file/SRR6745368_MERGED_FASTQ_SSU_OTU.tsv	Root-Environmental-Aquatic-Marine-Coastal-Sediment
SRR6172339	MGYA00362301	https://www.ebi.ac.uk/metagenomics/api/v1/analyses/MGYA00362301/file/SRR6172339_MERGED_FASTQ_SSU_OTU.tsv	Root-Environmental-Aquatic-Marine-Coastal-Sediment
SRR6745934	MGYA00202985	https://www.ebi.ac.uk/metagenomics/api/v1/analyses/MGYA00202985/file/SRR6745934_MERGED_FASTQ_SSU_OTU.tsv	Root-Environmental-Aquatic-Marine-Coastal-Sediment
SRR6746032	MGYA00202529	https://www.ebi.ac.uk/metagenomics/api/v1/analyses/MGYA00202529/file/SRR6746032_MERGED_FASTQ_SSU_OTU.tsv	Root-Environmental-Aquatic-Marine-Coastal-Sediment
SRR6746482	MGYA00202811	https://www.ebi.ac.uk/metagenomics/api/v1/analyses/MGYA00202811/file/SRR6746482_MERGED_FASTQ_SSU_OTU.tsv	Root-Environmental-Aquatic-Marine-Coastal-Sediment
SRR6172345	MGYA00362307	https://www.ebi.ac.uk/metagenomics/api/v1/analyses/MGYA00362307/file/SRR6172345_MERGED_FASTQ_SSU_OTU.tsv	Root-Environmental-Aquatic-Marine-Coastal-Sediment
ERR330036	MGYA00357994	https://www.ebi.ac.uk/metagenomics/api/v1/analyses/MGYA00357994/file/ERR330036_FASTQ_SSU_OTU.tsv	Root-Environmental-Aquatic-Marine-Coastal-Sediment
SRR6746201	MGYA00203057	https://www.ebi.ac.uk/metagenomics/api/v1/analyses/MGYA00203057/file/SRR6746201_MERGED_FASTQ_SSU_OTU.tsv	Root-Environmental-Aquatic-Marine-Coastal-Sediment

SRR6745810	MGYA00202495	https://www.ebi.ac.uk/metagenomics/api/v1/analyses/MGYA00202495/file/SRR6745810_MERGED_FASTQ_SSU_OTU.tsv	Root-Environmental-Aquatic-Marine-Coastal-Sediment
SRR6746116	MGYA00202517	https://www.ebi.ac.uk/metagenomics/api/v1/analyses/MGYA00202517/file/SRR6746116_MERGED_FASTQ_SSU_OTU.tsv	Root-Environmental-Aquatic-Marine-Coastal-Sediment
SRR2297122	MGYA00358886	https://www.ebi.ac.uk/metagenomics/api/v1/analyses/MGYA00358886/file/SRR2297122_MERGED_FASTQ_SSU_OTU.tsv	Root-Environmental-Aquatic-Marine-Coastal-Sediment
SRR6745948	MGYA00202532	https://www.ebi.ac.uk/metagenomics/api/v1/analyses/MGYA00202532/file/SRR6745948_MERGED_FASTQ_SSU_OTU.tsv	Root-Environmental-Aquatic-Marine-Coastal-Sediment
SRR4436287	MGYA00213036	https://www.ebi.ac.uk/metagenomics/api/v1/analyses/MGYA00213036/file/SRR4436287_FASTQ_SSU_OTU.tsv	Root-Environmental-Aquatic-Marine-Coastal-Sediment
SRR6745999	MGYA00202730	https://www.ebi.ac.uk/metagenomics/api/v1/analyses/MGYA00202730/file/SRR6745999_MERGED_FASTQ_SSU_OTU.tsv	Root-Environmental-Aquatic-Marine-Coastal-Sediment
SRR6172342	MGYA00362304	https://www.ebi.ac.uk/metagenomics/api/v1/analyses/MGYA00362304/file/SRR6172342_MERGED_FASTQ_SSU_OTU.tsv	Root-Environmental-Aquatic-Marine-Coastal-Sediment
ERR330031	MGYA00357989	https://www.ebi.ac.uk/metagenomics/api/v1/analyses/MGYA00357989/file/ERR330031_FASTQ_SSU_OTU.tsv	Root-Environmental-Aquatic-Marine-Coastal-Sediment
SRR6746206	MGYA00202519	https://www.ebi.ac.uk/metagenomics/api/v1/analyses/MGYA00202519/file/SRR6746206_MERGED_FASTQ_SSU_OTU.tsv	Root-Environmental-Aquatic-Marine-Coastal-Sediment
SRR6746570	MGYA00202983	https://www.ebi.ac.uk/metagenomics/api/v1/analyses/MGYA00202983/file/SRR6746570_MERGED_FASTQ_SSU_OTU.tsv	Root-Environmental-Aquatic-Marine-Coastal-Sediment
SRR6745817	MGYA00202667	https://www.ebi.ac.uk/metagenomics/api/v1/analyses/MGYA00202667/file/SRR6745817_MERGED_FASTQ_SSU_OTU.tsv	Root-Environmental-Aquatic-Marine-Coastal-Sediment
SRR2297125	MGYA00358889	https://www.ebi.ac.uk/metagenomics/api/v1/analyses/MGYA00358889/file/SRR2297125_MERGED_FASTQ_SSU_OTU.tsv	Root-Environmental-Aquatic-Marine-Coastal-Sediment

SRR6746111	MGYA00202498	https://www.ebi.ac.uk/metagenomics/api/v1/analyses/MGYA00202498/file/SRR6746111_MERGED_FASTQ_SSU_OTU.tsv	Root-Environmental-Aquatic-Marine-Coastal-Sediment
SRR6746049	MGYA00202557	https://www.ebi.ac.uk/metagenomics/api/v1/analyses/MGYA00202557/file/SRR6746049_MERGED_FASTQ_SSU_OTU.tsv	Root-Environmental-Aquatic-Marine-Coastal-Sediment
SRR6746098	MGYA00202989	https://www.ebi.ac.uk/metagenomics/api/v1/analyses/MGYA00202989/file/SRR6746098_MERGED_FASTQ_SSU_OTU.tsv	Root-Environmental-Aquatic-Marine-Coastal-Sediment
SRR2297188	MGYA00358952	https://www.ebi.ac.uk/metagenomics/api/v1/analyses/MGYA00358952/file/SRR2297188_MERGED_FASTQ_SSU_OTU.tsv	Root-Environmental-Aquatic-Marine-Coastal-Sediment
SRR4300296	MGYA00360255	https://www.ebi.ac.uk/metagenomics/api/v1/analyses/MGYA00360255/file/SRR4300296_MERGED_FASTQ_SSU_OTU.tsv	Root-Environmental-Aquatic-Marine-Coastal-Sediment
SRR6745419	MGYA00202884	https://www.ebi.ac.uk/metagenomics/api/v1/analyses/MGYA00202884/file/SRR6745419_MERGED_FASTQ_SSU_OTU.tsv	Root-Environmental-Aquatic-Marine-Coastal-Sediment
SRR2297159	MGYA00358923	https://www.ebi.ac.uk/metagenomics/api/v1/analyses/MGYA00358923/file/SRR2297159_MERGED_FASTQ_SSU_OTU.tsv	Root-Environmental-Aquatic-Marine-Coastal-Sediment
SRR6745933	MGYA00202621	https://www.ebi.ac.uk/metagenomics/api/v1/analyses/MGYA00202621/file/SRR6745933_MERGED_FASTQ_SSU_OTU.tsv	Root-Environmental-Aquatic-Marine-Coastal-Sediment
SRR6746035	MGYA00203109	https://www.ebi.ac.uk/metagenomics/api/v1/analyses/MGYA00203109/file/SRR6746035_MERGED_FASTQ_SSU_OTU.tsv	Root-Environmental-Aquatic-Marine-Coastal-Sediment
SRR6517605	MGYA00362470	https://www.ebi.ac.uk/metagenomics/api/v1/analyses/MGYA00362470/file/SRR6517605_MERGED_FASTQ_SSU_OTU.tsv	Root-Environmental-Aquatic-Marine-Coastal-Sediment
SRR6746485	MGYA00203111	https://www.ebi.ac.uk/metagenomics/api/v1/analyses/MGYA00203111/file/SRR6746485_MERGED_FASTQ_SSU_OTU.tsv	Root-Environmental-Aquatic-Marine-Coastal-Sediment
SRR6746208	MGYA00202945	https://www.ebi.ac.uk/metagenomics/api/v1/analyses/MGYA00202945/file/SRR6746208_MERGED_FASTQ_SSU_OTU.tsv	Root-Environmental-Aquatic-Marine-Coastal-Sediment

SRR6745819	MGYA00202948	https://www.ebi.ac.uk/metagenomics/api/v1/analyses/MGYA00202948/file/SRR6745819_MERGED_FASTQ_SSU_OTU.tsv	Root-Environmental-Aquatic-Marine-Coastal-Sediment
SRR2856995	MGYA00359235	https://www.ebi.ac.uk/metagenomics/api/v1/analyses/MGYA00359235/file/SRR2856995_MERGED_FASTQ_SSU_OTU.tsv	Root-Environmental-Aquatic-Marine-Coastal-Sediment
SRR3355022	MGYA00359638	https://www.ebi.ac.uk/metagenomics/api/v1/analyses/MGYA00359638/file/SRR3355022_MERGED_FASTQ_SSU_OTU.tsv	Root-Environmental-Aquatic-Marine-Coastal-Sediment
SRR6745941	MGYA00203101	https://www.ebi.ac.uk/metagenomics/api/v1/analyses/MGYA00203101/file/SRR6745941_MERGED_FASTQ_SSU_OTU.tsv	Root-Environmental-Aquatic-Marine-Coastal-Sediment
SRR6746047	MGYA00203054	https://www.ebi.ac.uk/metagenomics/api/v1/analyses/MGYA00203054/file/SRR6746047_MERGED_FASTQ_SSU_OTU.tsv	Root-Environmental-Aquatic-Marine-Coastal-Sediment
SRR6746096	MGYA00202855	https://www.ebi.ac.uk/metagenomics/api/v1/analyses/MGYA00202855/file/SRR6746096_MERGED_FASTQ_SSU_OTU.tsv	Root-Environmental-Aquatic-Marine-Coastal-Sediment
SRR6745990	MGYA00202704	https://www.ebi.ac.uk/metagenomics/api/v1/analyses/MGYA00202704/file/SRR6745990_MERGED_FASTQ_SSU_OTU.tsv	Root-Environmental-Aquatic-Marine-Coastal-Sediment
SRR4300298	MGYA00360257	https://www.ebi.ac.uk/metagenomics/api/v1/analyses/MGYA00360257/file/SRR4300298_MERGED_FASTQ_SSU_OTU.tsv	Root-Environmental-Aquatic-Marine-Coastal-Sediment
SRR2297186	MGYA00358950	https://www.ebi.ac.uk/metagenomics/api/v1/analyses/MGYA00358950/file/SRR2297186_MERGED_FASTQ_SSU_OTU.tsv	Root-Environmental-Aquatic-Marine-Coastal-Sediment
SRR6172330	MGYA00362292	https://www.ebi.ac.uk/metagenomics/api/v1/analyses/MGYA00362292/file/SRR6172330_MERGED_FASTQ_SSU_OTU.tsv	Root-Environmental-Aquatic-Marine-Coastal-Sediment
SRR6745361	MGYA00202719	https://www.ebi.ac.uk/metagenomics/api/v1/analyses/MGYA00202719/file/SRR6745361_MERGED_FASTQ_SSU_OTU.tsv	Root-Environmental-Aquatic-Marine-Coastal-Sediment
ERR330043	MGYA00358001	https://www.ebi.ac.uk/metagenomics/api/v1/analyses/MGYA00358001/file/ERR330043_FASTQ_SSU_OTU.tsv	Root-Environmental-Aquatic-Marine-Coastal-Sediment

SRR6746502	MGYA00202683	https://www.ebi.ac.uk/metagenomics/api/v1/analyses/MGYA00202683/file/SRR6746502_MERGED_FASTQ_SSU_OTU.tsv	Root-Environmental-Aquatic-Marine-Coastal-Sediment
SRR6745417	MGYA00202938	https://www.ebi.ac.uk/metagenomics/api/v1/analyses/MGYA00202938/file/SRR6745417_MERGED_FASTQ_SSU_OTU.tsv	Root-Environmental-Aquatic-Marine-Coastal-Sediment
SRR6745865	MGYA00202845	https://www.ebi.ac.uk/metagenomics/api/v1/analyses/MGYA00202845/file/SRR6745865_MERGED_FASTQ_SSU_OTU.tsv	Root-Environmental-Aquatic-Marine-Coastal-Sediment
SRR6746163	MGYA00202590	https://www.ebi.ac.uk/metagenomics/api/v1/analyses/MGYA00202590/file/SRR6746163_MERGED_FASTQ_SSU_OTU.tsv	Root-Environmental-Aquatic-Marine-Coastal-Sediment
SRR2297157	MGYA00358921	https://www.ebi.ac.uk/metagenomics/api/v1/analyses/MGYA00358921/file/SRR2297157_MERGED_FASTQ_SSU_OTU.tsv	Root-Environmental-Aquatic-Marine-Coastal-Sediment
SRR2297181	MGYA00358945	https://www.ebi.ac.uk/metagenomics/api/v1/analyses/MGYA00358945/file/SRR2297181_MERGED_FASTQ_SSU_OTU.tsv	Root-Environmental-Aquatic-Marine-Coastal-Sediment
SRR6745366	MGYA00202638	https://www.ebi.ac.uk/metagenomics/api/v1/analyses/MGYA00202638/file/SRR6745366_MERGED_FASTQ_SSU_OTU.tsv	Root-Environmental-Aquatic-Marine-Coastal-Sediment
SRR6172337	MGYA00362299	https://www.ebi.ac.uk/metagenomics/api/v1/analyses/MGYA00362299/file/SRR6172337_MERGED_FASTQ_SSU_OTU.tsv	Root-Environmental-Aquatic-Marine-Coastal-Sediment
ERR330044	MGYA00358002	https://www.ebi.ac.uk/metagenomics/api/v1/analyses/MGYA00358002/file/ERR330044_FASTQ_SSU_OTU.tsv	Root-Environmental-Aquatic-Marine-Coastal-Sediment
SRR6746505	MGYA00202544	https://www.ebi.ac.uk/metagenomics/api/v1/analyses/MGYA00202544/file/SRR6746505_MERGED_FASTQ_SSU_OTU.tsv	Root-Environmental-Aquatic-Marine-Coastal-Sediment
SRR6745862	MGYA00202847	https://www.ebi.ac.uk/metagenomics/api/v1/analyses/MGYA00202847/file/SRR6745862_MERGED_FASTQ_SSU_OTU.tsv	Root-Environmental-Aquatic-Marine-Coastal-Sediment
SRR6745410	MGYA00202767	https://www.ebi.ac.uk/metagenomics/api/v1/analyses/MGYA00202767/file/SRR6745410_MERGED_FASTQ_SSU_OTU.tsv	Root-Environmental-Aquatic-Marine-Coastal-Sediment

SRR2297150	MGYA00358914	https://www.ebi.ac.uk/metagenomics/api/v1/analyses/MGYA00358914/file/SRR2297150_MERGED_FASTQ_SSU_OTU.tsv	Root-Environmental-Aquatic-Marine-Coastal-Sediment
SRR6746164	MGYA00202720	https://www.ebi.ac.uk/metagenomics/api/v1/analyses/MGYA00202720/file/SRR6746164_MERGED_FASTQ_SSU_OTU.tsv	Root-Environmental-Aquatic-Marine-Coastal-Sediment
ERR330038	MGYA00357996	https://www.ebi.ac.uk/metagenomics/api/v1/analyses/MGYA00357996/file/ERR330038_FASTQ_SSU_OTU.tsv	Root-Environmental-Aquatic-Marine-Coastal-Sediment
SRR4436300	MGYA00213046	https://www.ebi.ac.uk/metagenomics/api/v1/analyses/MGYA00213046/file/SRR4436300_FASTQ_SSU_OTU.tsv	Root-Environmental-Aquatic-Marine-Coastal-Sediment
SRR3355025	MGYA00359640	https://www.ebi.ac.uk/metagenomics/api/v1/analyses/MGYA00359640/file/SRR3355025_MERGED_FASTQ_SSU_OTU.tsv	Root-Environmental-Aquatic-Marine-Coastal-Sediment
SRR6746118	MGYA00203025	https://www.ebi.ac.uk/metagenomics/api/v1/analyses/MGYA00203025/file/SRR6746118_MERGED_FASTQ_SSU_OTU.tsv	Root-Environmental-Aquatic-Marine-Coastal-Sediment
SRR6745946	MGYA00202567	https://www.ebi.ac.uk/metagenomics/api/v1/analyses/MGYA00202567/file/SRR6745946_MERGED_FASTQ_SSU_OTU.tsv	Root-Environmental-Aquatic-Marine-Coastal-Sediment
SRR6746040	MGYA00202653	https://www.ebi.ac.uk/metagenomics/api/v1/analyses/MGYA00202653/file/SRR6746040_MERGED_FASTQ_SSU_OTU.tsv	Root-Environmental-Aquatic-Marine-Coastal-Sediment
SRR6746091	MGYA00203080	https://www.ebi.ac.uk/metagenomics/api/v1/analyses/MGYA00203080/file/SRR6746091_MERGED_FASTQ_SSU_OTU.tsv	Root-Environmental-Aquatic-Marine-Coastal-Sediment
SRR6745997	MGYA00202887	https://www.ebi.ac.uk/metagenomics/api/v1/analyses/MGYA00202887/file/SRR6745997_MERGED_FASTQ_SSU_OTU.tsv	Root-Environmental-Aquatic-Marine-Coastal-Sediment
SRR4436289	MGYA00213054	https://www.ebi.ac.uk/metagenomics/api/v1/analyses/MGYA00213054/file/SRR4436289_FASTQ_SSU_OTU.tsv	Root-Environmental-Aquatic-Marine-Coastal-Sediment
SRR5081941	MGYA00360535	https://www.ebi.ac.uk/metagenomics/api/v1/analyses/MGYA00360535/file/SRR5081941_MERGED_FASTQ_SSU_OTU.tsv	Root-Environmental-Aquatic-Marine-Wetlands

SRR5078075	MGYA00360534	https://www.ebi.ac.uk/metagenomics/api/v1/analyses/MGYA00360534/file/SRR5078075_FASTQ_SSU_OTU.tsv	Root-Environmental-Aquatic-Marine-Wetlands
SRR5078074	MGYA00360533	https://www.ebi.ac.uk/metagenomics/api/v1/analyses/MGYA00360533/file/SRR5078074_FASTQ_SSU_OTU.tsv	Root-Environmental-Aquatic-Marine-Wetlands
SRR5081943	MGYA00360537	https://www.ebi.ac.uk/metagenomics/api/v1/analyses/MGYA00360537/file/SRR5081943_MERGED_FASTQ_SSU_OTU.tsv	Root-Environmental-Aquatic-Marine-Wetlands
SRR5081944	MGYA00360538	https://www.ebi.ac.uk/metagenomics/api/v1/analyses/MGYA00360538/file/SRR5081944_MERGED_FASTQ_SSU_OTU.tsv	Root-Environmental-Aquatic-Marine-Wetlands
SRR5081942	MGYA00360536	https://www.ebi.ac.uk/metagenomics/api/v1/analyses/MGYA00360536/file/SRR5081942_MERGED_FASTQ_SSU_OTU.tsv	Root-Environmental-Aquatic-Marine-Wetlands
ERR537776	MGYA00218277	https://www.ebi.ac.uk/metagenomics/api/v1/analyses/MGYA00218277/file/ERR537776_MERGED_FASTQ_SSU_OTU.tsv	Root-Host_associated-Mammals-Digestive_system-Large_intestine-Cecum
SRR4125143	MGYA00099283	https://www.ebi.ac.uk/metagenomics/api/v1/analyses/MGYA00099283/file/SRR4125143_MERGED_FASTQ_otu.tsv	Root-Host_associated-Mammals-Digestive_system-Large_intestine-Cecum
ERR537683	MGYA00218362	https://www.ebi.ac.uk/metagenomics/api/v1/analyses/MGYA00218362/file/ERR537683_MERGED_FASTQ_SSU_OTU.tsv	Root-Host_associated-Mammals-Digestive_system-Large_intestine-Cecum
ERR537652	MGYA00218324	https://www.ebi.ac.uk/metagenomics/api/v1/analyses/MGYA00218324/file/ERR537652_MERGED_FASTQ_SSU_OTU.tsv	Root-Host_associated-Mammals-Digestive_system-Large_intestine-Cecum
ERR537684	MGYA00218341	https://www.ebi.ac.uk/metagenomics/api/v1/analyses/MGYA00218341/file/ERR537684_MERGED_FASTQ_SSU_OTU.tsv	Root-Host_associated-Mammals-Digestive_system-Large_intestine-Cecum
SRR4125138	MGYA00099335	https://www.ebi.ac.uk/metagenomics/api/v1/analyses/MGYA00099335/file/SRR4125138_MERGED_FASTQ_otu.tsv	Root-Host_associated-Mammals-Digestive_system-Large_intestine-Cecum
ERR537655	MGYA00218293	https://www.ebi.ac.uk/metagenomics/api/v1/analyses/MGYA00218293/file/ERR537655_MERGED_FASTQ_SSU_OTU.tsv	Root-Host_associated-Mammals-Digestive_system-Large_intestine-Cecum

ERR537771	MGYA00218320	https://www.ebi.ac.uk/metagenomics/api/v1/analyses/MGYA00218320/file/ERR537771_MERGED_FASTQ_SSU_OTU.tsv	Root-Host_associated-Mammals-Digestive_system-Large_intestine-Cecum
SRR4125144	MGYA00099315	https://www.ebi.ac.uk/metagenomics/api/v1/analyses/MGYA00099315/file/SRR4125144_MERGED_FASTQ_otu.tsv	Root-Host_associated-Mammals-Digestive_system-Large_intestine-Cecum
ERR537703	MGYA00218367	https://www.ebi.ac.uk/metagenomics/api/v1/analyses/MGYA00218367/file/ERR537703_MERGED_FASTQ_SSU_OTU.tsv	Root-Host_associated-Mammals-Digestive_system-Large_intestine-Cecum
SRR4125136	MGYA00099312	https://www.ebi.ac.uk/metagenomics/api/v1/analyses/MGYA00099312/file/SRR4125136_MERGED_FASTQ_otu.tsv	Root-Host_associated-Mammals-Digestive_system-Large_intestine-Cecum
SRR8553588	MGYA00412312	https://www.ebi.ac.uk/metagenomics/api/v1/analyses/MGYA00412312/file/SRR8553588_MERGED_FASTQ_SSU_OTU.tsv	Root-Host_associated-Mammals-Digestive_system-Large_intestine-Cecum
ERR537704	MGYA00218301	https://www.ebi.ac.uk/metagenomics/api/v1/analyses/MGYA00218301/file/ERR537704_MERGED_FASTQ_SSU_OTU.tsv	Root-Host_associated-Mammals-Digestive_system-Large_intestine-Cecum
SRR4125131	MGYA00099305	https://www.ebi.ac.uk/metagenomics/api/v1/analyses/MGYA00099305/file/SRR4125131_MERGED_FASTQ_otu.tsv	Root-Host_associated-Mammals-Digestive_system-Large_intestine-Cecum
ERR537731	MGYA00218339	https://www.ebi.ac.uk/metagenomics/api/v1/analyses/MGYA00218339/file/ERR537731_MERGED_FASTQ_SSU_OTU.tsv	Root-Host_associated-Mammals-Digestive_system-Large_intestine-Cecum
ERR537669	MGYA00218383	https://www.ebi.ac.uk/metagenomics/api/v1/analyses/MGYA00218383/file/ERR537669_MERGED_FASTQ_SSU_OTU.tsv	Root-Host_associated-Mammals-Digestive_system-Large_intestine-Cecum
SRR4125104	MGYA00099298	https://www.ebi.ac.uk/metagenomics/api/v1/analyses/MGYA00099298/file/SRR4125104_MERGED_FASTQ_otu.tsv	Root-Host_associated-Mammals-Digestive_system-Large_intestine-Cecum
ERR537736	MGYA00218334	https://www.ebi.ac.uk/metagenomics/api/v1/analyses/MGYA00218334/file/ERR537736_MERGED_FASTQ_SSU_OTU.tsv	Root-Host_associated-Mammals-Digestive_system-Large_intestine-Cecum
SRR4125103	MGYA00099332	https://www.ebi.ac.uk/metagenomics/api/v1/analyses/MGYA00099332/file/SRR4125103_MERGED_FASTQ_otu.tsv	Root-Host_associated-Mammals-Digestive_system-Large_intestine-Cecum

ERR958528	MGYA00126328	https://www.ebi.ac.uk/metagenomics/api/v1/analyses/MGYA00126328/file/ERR958528_MERGED_FASTQ_otu.tsv	Root-Host_associated-Mammals-Digestive_system-Large_intestine-Cecum
ERR537738	MGYA00218316	https://www.ebi.ac.uk/metagenomics/api/v1/analyses/MGYA00218316/file/ERR537738_MERGED_FASTQ_SSU_OTU.tsv	Root-Host_associated-Mammals-Digestive_system-Large_intestine-Cecum
ERR537660	MGYA00218402	https://www.ebi.ac.uk/metagenomics/api/v1/analyses/MGYA00218402/file/ERR537660_MERGED_FASTQ_SSU_OTU.tsv	Root-Host_associated-Mammals-Digestive_system-Large_intestine-Cecum
ERR537744	MGYA00218338	https://www.ebi.ac.uk/metagenomics/api/v1/analyses/MGYA00218338/file/ERR537744_MERGED_FASTQ_SSU_OTU.tsv	Root-Host_associated-Mammals-Digestive_system-Large_intestine-Cecum
ERR537743	MGYA00218350	https://www.ebi.ac.uk/metagenomics/api/v1/analyses/MGYA00218350/file/ERR537743_MERGED_FASTQ_SSU_OTU.tsv	Root-Host_associated-Mammals-Digestive_system-Large_intestine-Cecum
ERR537667	MGYA00218312	https://www.ebi.ac.uk/metagenomics/api/v1/analyses/MGYA00218312/file/ERR537667_MERGED_FASTQ_SSU_OTU.tsv	Root-Host_associated-Mammals-Digestive_system-Large_intestine-Cecum
ERR537752	MGYA00218299	https://www.ebi.ac.uk/metagenomics/api/v1/analyses/MGYA00218299/file/ERR537752_MERGED_FASTQ_SSU_OTU.tsv	Root-Host_associated-Mammals-Digestive_system-Large_intestine-Cecum
ERR537676	MGYA00218286	https://www.ebi.ac.uk/metagenomics/api/v1/analyses/MGYA00218286/file/ERR537676_MERGED_FASTQ_SSU_OTU.tsv	Root-Host_associated-Mammals-Digestive_system-Large_intestine-Cecum
ERR537671	MGYA00218388	https://www.ebi.ac.uk/metagenomics/api/v1/analyses/MGYA00218388/file/ERR537671_MERGED_FASTQ_SSU_OTU.tsv	Root-Host_associated-Mammals-Digestive_system-Large_intestine-Cecum
ERR537729	MGYA00218329	https://www.ebi.ac.uk/metagenomics/api/v1/analyses/MGYA00218329/file/ERR537729_MERGED_FASTQ_SSU_OTU.tsv	Root-Host_associated-Mammals-Digestive_system-Large_intestine-Cecum
SRR4125095	MGYA00099282	https://www.ebi.ac.uk/metagenomics/api/v1/analyses/MGYA00099282/file/SRR4125095_MERGED_FASTQ_otu.tsv	Root-Host_associated-Mammals-Digestive_system-Large_intestine-Cecum
ERR537755	MGYA00218352	https://www.ebi.ac.uk/metagenomics/api/v1/analyses/MGYA00218352/file/ERR537755_MERGED_FASTQ_SSU_OTU.tsv	Root-Host_associated-Mammals-Digestive_system-Large_intestine-Cecum

SRR4125112	MGYA00099319	https://www.ebi.ac.uk/metagenomics/api/v1/analyses/MGYA00099319/file/SRR4125112_MERGED_FASTQ_otu.tsv	Root-Host_associated-Mammals-Digestive_system-Large_intestine-Cecum
ERR537727	MGYA00218392	https://www.ebi.ac.uk/metagenomics/api/v1/analyses/MGYA00218392/file/ERR537727_MERGED_FASTQ_SSU_OTU.tsv	Root-Host_associated-Mammals-Digestive_system-Large_intestine-Cecum
SRR4125115	MGYA00099313	https://www.ebi.ac.uk/metagenomics/api/v1/analyses/MGYA00099313/file/SRR4125115_MERGED_FASTQ_otu.tsv	Root-Host_associated-Mammals-Digestive_system-Large_intestine-Cecum
ERR537678	MGYA00218319	https://www.ebi.ac.uk/metagenomics/api/v1/analyses/MGYA00218319/file/ERR537678_MERGED_FASTQ_SSU_OTU.tsv	Root-Host_associated-Mammals-Digestive_system-Large_intestine-Cecum
ERR537720	MGYA00218292	https://www.ebi.ac.uk/metagenomics/api/v1/analyses/MGYA00218292/file/ERR537720_MERGED_FASTQ_SSU_OTU.tsv	Root-Host_associated-Mammals-Digestive_system-Large_intestine-Cecum
ERR537769	MGYA00218327	https://www.ebi.ac.uk/metagenomics/api/v1/analyses/MGYA00218327/file/ERR537769_MERGED_FASTQ_SSU_OTU.tsv	Root-Host_associated-Mammals-Digestive_system-Large_intestine-Cecum
SRR4125120	MGYA00099336	https://www.ebi.ac.uk/metagenomics/api/v1/analyses/MGYA00099336/file/SRR4125120_MERGED_FASTQ_otu.tsv	Root-Host_associated-Mammals-Digestive_system-Large_intestine-Cecum
ERR537715	MGYA00218376	https://www.ebi.ac.uk/metagenomics/api/v1/analyses/MGYA00218376/file/ERR537715_MERGED_FASTQ_SSU_OTU.tsv	Root-Host_associated-Mammals-Digestive_system-Large_intestine-Cecum
SRR4125127	MGYA00099325	https://www.ebi.ac.uk/metagenomics/api/v1/analyses/MGYA00099325/file/SRR4125127_MERGED_FASTQ_otu.tsv	Root-Host_associated-Mammals-Digestive_system-Large_intestine-Cecum
ERR537712	MGYA00218381	https://www.ebi.ac.uk/metagenomics/api/v1/analyses/MGYA00218381/file/ERR537712_MERGED_FASTQ_SSU_OTU.tsv	Root-Host_associated-Mammals-Digestive_system-Large_intestine-Cecum
ERR537644	MGYA00218370	https://www.ebi.ac.uk/metagenomics/api/v1/analyses/MGYA00218370/file/ERR537644_MERGED_FASTQ_SSU_OTU.tsv	Root-Host_associated-Mammals-Digestive_system-Large_intestine-Cecum
ERR537695	MGYA00218404	https://www.ebi.ac.uk/metagenomics/api/v1/analyses/MGYA00218404/file/ERR537695_MERGED_FASTQ_SSU_OTU.tsv	Root-Host_associated-Mammals-Digestive_system-Large_intestine-Cecum

SRR4125129	MGYA00099317	https://www.ebi.ac.uk/metagenomics/api/v1/analyses/MGYA00099317/file/SRR4125129_MERGED_FASTQ_otu.tsv	Root-Host_associated-Mammals-Digestive_system-Large_intestine-Cecum
ERR537638	MGYA00218306	https://www.ebi.ac.uk/metagenomics/api/v1/analyses/MGYA00218306/file/ERR537638_MERGED_FASTQ_SSU_OTU.tsv	Root-Host_associated-Mammals-Digestive_system-Large_intestine-Cecum
ERR537760	MGYA00218371	https://www.ebi.ac.uk/metagenomics/api/v1/analyses/MGYA00218371/file/ERR537760_MERGED_FASTQ_SSU_OTU.tsv	Root-Host_associated-Mammals-Digestive_system-Large_intestine-Cecum
ERR537767	MGYA00218343	https://www.ebi.ac.uk/metagenomics/api/v1/analyses/MGYA00218343/file/ERR537767_MERGED_FASTQ_SSU_OTU.tsv	Root-Host_associated-Mammals-Digestive_system-Large_intestine-Cecum
ERR537643	MGYA00218283	https://www.ebi.ac.uk/metagenomics/api/v1/analyses/MGYA00218283/file/ERR537643_MERGED_FASTQ_SSU_OTU.tsv	Root-Host_associated-Mammals-Digestive_system-Large_intestine-Cecum
ERR537692	MGYA00218313	https://www.ebi.ac.uk/metagenomics/api/v1/analyses/MGYA00218313/file/ERR537692_MERGED_FASTQ_SSU_OTU.tsv	Root-Host_associated-Mammals-Digestive_system-Large_intestine-Cecum
ERR537742	MGYA00218298	https://www.ebi.ac.uk/metagenomics/api/v1/analyses/MGYA00218298/file/ERR537742_MERGED_FASTQ_SSU_OTU.tsv	Root-Host_associated-Mammals-Digestive_system-Large_intestine-Cecum
ERR537666	MGYA00218337	https://www.ebi.ac.uk/metagenomics/api/v1/analyses/MGYA00218337/file/ERR537666_MERGED_FASTQ_SSU_OTU.tsv	Root-Host_associated-Mammals-Digestive_system-Large_intestine-Cecum
ERR537739	MGYA00218378	https://www.ebi.ac.uk/metagenomics/api/v1/analyses/MGYA00218378/file/ERR537739_MERGED_FASTQ_SSU_OTU.tsv	Root-Host_associated-Mammals-Digestive_system-Large_intestine-Cecum
ERR537661	MGYA00218280	https://www.ebi.ac.uk/metagenomics/api/v1/analyses/MGYA00218280/file/ERR537661_MERGED_FASTQ_SSU_OTU.tsv	Root-Host_associated-Mammals-Digestive_system-Large_intestine-Cecum
ERR537745	MGYA00218387	https://www.ebi.ac.uk/metagenomics/api/v1/analyses/MGYA00218387/file/ERR537745_MERGED_FASTQ_SSU_OTU.tsv	Root-Host_associated-Mammals-Digestive_system-Large_intestine-Cecum
ERR537737	MGYA00218318	https://www.ebi.ac.uk/metagenomics/api/v1/analyses/MGYA00218318/file/ERR537737_MERGED_FASTQ_SSU_OTU.tsv	Root-Host_associated-Mammals-Digestive_system-Large_intestine-Cecum

SRR4125102	MGYA00099327	https://www.ebi.ac.uk/metagenomics/api/v1/analyses/MGYA000993<br>27/file/SRR4125102_MERGED_FASTQ_otu.tsv	Root-Host_associated-Mammals-Digestive_system-Large_intestine-Cecum
ERR537730	MGYA00218413	https://www.ebi.ac.uk/metagenomics/api/v1/analyses/MGYA002184<br>13/file/ERR537730_MERGED_FASTQ_SSU_OTU.tsv	Root-Host_associated-Mammals-Digestive_system-Large_intestine-Cecum
ERR537668	MGYA00218403	https://www.ebi.ac.uk/metagenomics/api/v1/analyses/MGYA002184<br>03/file/ERR537668_MERGED_FASTQ_SSU_OTU.tsv	Root-Host_associated-Mammals-Digestive_system-Large_intestine-Cecum
SRR4125105	MGYA00099334	https://www.ebi.ac.uk/metagenomics/api/v1/analyses/MGYA000993<br>34/file/SRR4125105_MERGED_FASTQ_otu.tsv	Root-Host_associated-Mammals-Digestive_system-Large_intestine-Cecum
ERR537705	MGYA00218281	https://www.ebi.ac.uk/metagenomics/api/v1/analyses/MGYA002182<br>81/file/ERR537705_MERGED_FASTQ_SSU_OTU.tsv	Root-Host_associated-Mammals-Digestive_system-Large_intestine-Cecum
SRR4125130	MGYA00099279	https://www.ebi.ac.uk/metagenomics/api/v1/analyses/MGYA000992<br>79/file/SRR4125130_MERGED_FASTQ_otu.tsv	Root-Host_associated-Mammals-Digestive_system-Large_intestine-Cecum
ERR537702	MGYA00218363	https://www.ebi.ac.uk/metagenomics/api/v1/analyses/MGYA002183<br>63/file/ERR537702_MERGED_FASTQ_SSU_OTU.tsv	Root-Host_associated-Mammals-Digestive_system-Large_intestine-Cecum
SRR4125137	MGYA00099311	https://www.ebi.ac.uk/metagenomics/api/v1/analyses/MGYA000993<br>11/file/SRR4125137_MERGED_FASTQ_otu.tsv	Root-Host_associated-Mammals-Digestive_system-Large_intestine-Cecum
ERR537685	MGYA00218379	https://www.ebi.ac.uk/metagenomics/api/v1/analyses/MGYA002183<br>79/file/ERR537685_MERGED_FASTQ_SSU_OTU.tsv	Root-Host_associated-Mammals-Digestive_system-Large_intestine-Cecum
SRR4125139	MGYA00099308	https://www.ebi.ac.uk/metagenomics/api/v1/analyses/MGYA000993<br>08/file/SRR4125139_MERGED_FASTQ_otu.tsv	Root-Host_associated-Mammals-Digestive_system-Large_intestine-Cecum
ERR537654	MGYA00218284	https://www.ebi.ac.uk/metagenomics/api/v1/analyses/MGYA002182<br>84/file/ERR537654_MERGED_FASTQ_SSU_OTU.tsv	Root-Host_associated-Mammals-Digestive_system-Large_intestine-Cecum
ERR537770	MGYA00218282	https://www.ebi.ac.uk/metagenomics/api/v1/analyses/MGYA002182<br>82/file/ERR537770_MERGED_FASTQ_SSU_OTU.tsv	Root-Host_associated-Mammals-Digestive_system-Large_intestine-Cecum

SRR4125145	MGYA00099289	https://www.ebi.ac.uk/metagenomics/api/v1/analyses/MGYA00099289/file/SRR4125145_MERGED_FASTQ_otu.tsv	Root-Host_associated-Mammals-Digestive_system-Large_intestine-Cecum
ERR537777	MGYA00218351	https://www.ebi.ac.uk/metagenomics/api/v1/analyses/MGYA00218351/file/ERR537777_MERGED_FASTQ_SSU_OTU.tsv	Root-Host_associated-Mammals-Digestive_system-Large_intestine-Cecum
SRR4125142	MGYA00099303	https://www.ebi.ac.uk/metagenomics/api/v1/analyses/MGYA00099303/file/SRR4125142_MERGED_FASTQ_otu.tsv	Root-Host_associated-Mammals-Digestive_system-Large_intestine-Cecum
ERR537682	MGYA00218385	https://www.ebi.ac.uk/metagenomics/api/v1/analyses/MGYA00218385/file/ERR537682_MERGED_FASTQ_SSU_OTU.tsv	Root-Host_associated-Mammals-Digestive_system-Large_intestine-Cecum
ERR537653	MGYA00218357	https://www.ebi.ac.uk/metagenomics/api/v1/analyses/MGYA00218357/file/ERR537653_MERGED_FASTQ_SSU_OTU.tsv	Root-Host_associated-Mammals-Digestive_system-Large_intestine-Cecum
ERR537766	MGYA00218285	https://www.ebi.ac.uk/metagenomics/api/v1/analyses/MGYA00218285/file/ERR537766_MERGED_FASTQ_SSU_OTU.tsv	Root-Host_associated-Mammals-Digestive_system-Large_intestine-Cecum
ERR537642	MGYA00218391	https://www.ebi.ac.uk/metagenomics/api/v1/analyses/MGYA00218391/file/ERR537642_MERGED_FASTQ_SSU_OTU.tsv	Root-Host_associated-Mammals-Digestive_system-Large_intestine-Cecum
ERR537693	MGYA00218384	https://www.ebi.ac.uk/metagenomics/api/v1/analyses/MGYA00218384/file/ERR537693_MERGED_FASTQ_SSU_OTU.tsv	Root-Host_associated-Mammals-Digestive_system-Large_intestine-Cecum
ERR537645	MGYA00218330	https://www.ebi.ac.uk/metagenomics/api/v1/analyses/MGYA00218330/file/ERR537645_MERGED_FASTQ_SSU_OTU.tsv	Root-Host_associated-Mammals-Digestive_system-Large_intestine-Cecum
ERR537694	MGYA00218365	https://www.ebi.ac.uk/metagenomics/api/v1/analyses/MGYA00218365/file/ERR537694_MERGED_FASTQ_SSU_OTU.tsv	Root-Host_associated-Mammals-Digestive_system-Large_intestine-Cecum
SRR4125128	MGYA00099297	https://www.ebi.ac.uk/metagenomics/api/v1/analyses/MGYA00099297/file/SRR4125128_MERGED_FASTQ_otu.tsv	Root-Host_associated-Mammals-Digestive_system-Large_intestine-Cecum
ERR537639	MGYA00218374	https://www.ebi.ac.uk/metagenomics/api/v1/analyses/MGYA00218374/file/ERR537639_MERGED_FASTQ_SSU_OTU.tsv	Root-Host_associated-Mammals-Digestive_system-Large_intestine-Cecum

ERR537761	MGYA00218311	https://www.ebi.ac.uk/metagenomics/api/v1/analyses/MGYA00218311/file/ERR537761_MERGED_FASTQ_SSU_OTU.tsv	Root-Host_associated-Mammals-Digestive_system-Large_intestine-Cecum
SRR4125126	MGYA00099304	https://www.ebi.ac.uk/metagenomics/api/v1/analyses/MGYA00099304/file/SRR4125126_MERGED_FASTQ_otu.tsv	Root-Host_associated-Mammals-Digestive_system-Large_intestine-Cecum
ERR537713	MGYA00218335	https://www.ebi.ac.uk/metagenomics/api/v1/analyses/MGYA00218335/file/ERR537713_MERGED_FASTQ_SSU_OTU.tsv	Root-Host_associated-Mammals-Digestive_system-Large_intestine-Cecum
ERR537768	MGYA00218406	https://www.ebi.ac.uk/metagenomics/api/v1/analyses/MGYA00218406/file/ERR537768_MERGED_FASTQ_SSU_OTU.tsv	Root-Host_associated-Mammals-Digestive_system-Large_intestine-Cecum
SRR4125121	MGYA00099323	https://www.ebi.ac.uk/metagenomics/api/v1/analyses/MGYA00099323/file/SRR4125121_MERGED_FASTQ_otu.tsv	Root-Host_associated-Mammals-Digestive_system-Large_intestine-Cecum
ERR537714	MGYA00218290	https://www.ebi.ac.uk/metagenomics/api/v1/analyses/MGYA00218290/file/ERR537714_MERGED_FASTQ_SSU_OTU.tsv	Root-Host_associated-Mammals-Digestive_system-Large_intestine-Cecum
SRR4125114	MGYA00099333	https://www.ebi.ac.uk/metagenomics/api/v1/analyses/MGYA00099333/file/SRR4125114_MERGED_FASTQ_otu.tsv	Root-Host_associated-Mammals-Digestive_system-Large_intestine-Cecum
ERR537679	MGYA00218359	https://www.ebi.ac.uk/metagenomics/api/v1/analyses/MGYA00218359/file/ERR537679_MERGED_FASTQ_SSU_OTU.tsv	Root-Host_associated-Mammals-Digestive_system-Large_intestine-Cecum
ERR537721	MGYA00218368	https://www.ebi.ac.uk/metagenomics/api/v1/analyses/MGYA00218368/file/ERR537721_MERGED_FASTQ_SSU_OTU.tsv	Root-Host_associated-Mammals-Digestive_system-Large_intestine-Cecum
SRR4125113	MGYA00099296	https://www.ebi.ac.uk/metagenomics/api/v1/analyses/MGYA00099296/file/SRR4125113_MERGED_FASTQ_otu.tsv	Root-Host_associated-Mammals-Digestive_system-Large_intestine-Cecum
ERR537726	MGYA00218326	https://www.ebi.ac.uk/metagenomics/api/v1/analyses/MGYA00218326/file/ERR537726_MERGED_FASTQ_SSU_OTU.tsv	Root-Host_associated-Mammals-Digestive_system-Large_intestine-Cecum
ERR537670	MGYA00218412	https://www.ebi.ac.uk/metagenomics/api/v1/analyses/MGYA00218412/file/ERR537670_MERGED_FASTQ_SSU_OTU.tsv	Root-Host_associated-Mammals-Digestive_system-Large_intestine-Cecum

ERR537728	MGYA00218375	https://www.ebi.ac.uk/metagenomics/api/v1/analyses/MGYA00218375/file/ERR537728_MERGED_FASTQ_SSU_OTU.tsv	Root-Host_associated-Mammals-Digestive_system-Large_intestine-Cecum
SRR4125094	MGYA00099322	https://www.ebi.ac.uk/metagenomics/api/v1/analyses/MGYA00099322/file/SRR4125094_MERGED_FASTQ_otu.tsv	Root-Host_associated-Mammals-Digestive_system-Large_intestine-Cecum
ERR537754	MGYA00218380	https://www.ebi.ac.uk/metagenomics/api/v1/analyses/MGYA00218380/file/ERR537754_MERGED_FASTQ_SSU_OTU.tsv	Root-Host_associated-Mammals-Digestive_system-Large_intestine-Cecum
ERR537753	MGYA00218369	https://www.ebi.ac.uk/metagenomics/api/v1/analyses/MGYA00218369/file/ERR537753_MERGED_FASTQ_SSU_OTU.tsv	Root-Host_associated-Mammals-Digestive_system-Large_intestine-Cecum
ERR537677	MGYA00218314	https://www.ebi.ac.uk/metagenomics/api/v1/analyses/MGYA00218314/file/ERR537677_MERGED_FASTQ_SSU_OTU.tsv	Root-Host_associated-Mammals-Digestive_system-Large_intestine-Cecum
SRR4125093	MGYA00099309	https://www.ebi.ac.uk/metagenomics/api/v1/analyses/MGYA00099309/file/SRR4125093_MERGED_FASTQ_otu.tsv	Root-Host_associated-Mammals-Digestive_system-Large_intestine-Cecum
ERR537646	MGYA00218307	https://www.ebi.ac.uk/metagenomics/api/v1/analyses/MGYA00218307/file/ERR537646_MERGED_FASTQ_SSU_OTU.tsv	Root-Host_associated-Mammals-Digestive_system-Large_intestine-Cecum
ERR537697	MGYA00218333	https://www.ebi.ac.uk/metagenomics/api/v1/analyses/MGYA00218333/file/ERR537697_MERGED_FASTQ_SSU_OTU.tsv	Root-Host_associated-Mammals-Digestive_system-Large_intestine-Cecum
ERR537762	MGYA00218336	https://www.ebi.ac.uk/metagenomics/api/v1/analyses/MGYA00218336/file/ERR537762_MERGED_FASTQ_SSU_OTU.tsv	Root-Host_associated-Mammals-Digestive_system-Large_intestine-Cecum
SRR4125150	MGYA00099291	https://www.ebi.ac.uk/metagenomics/api/v1/analyses/MGYA00099291/file/SRR4125150_MERGED_FASTQ_otu.tsv	Root-Host_associated-Mammals-Digestive_system-Large_intestine-Cecum
ERR537765	MGYA00218398	https://www.ebi.ac.uk/metagenomics/api/v1/analyses/MGYA00218398/file/ERR537765_MERGED_FASTQ_SSU_OTU.tsv	Root-Host_associated-Mammals-Digestive_system-Large_intestine-Cecum
ERR537641	MGYA00218274	https://www.ebi.ac.uk/metagenomics/api/v1/analyses/MGYA00218274/file/ERR537641_MERGED_FASTQ_SSU_OTU.tsv	Root-Host_associated-Mammals-Digestive_system-Large_intestine-Cecum

ERR537690	MGYA00218289	https://www.ebi.ac.uk/metagenomics/api/v1/analyses/MGYA00218289/file/ERR537690_MERGED_FASTQ_SSU_OTU.tsv	Root-Host_associated-Mammals-Digestive_system-Large_intestine-Cecum
ERR537719	MGYA00218310	https://www.ebi.ac.uk/metagenomics/api/v1/analyses/MGYA00218310/file/ERR537719_MERGED_FASTQ_SSU_OTU.tsv	Root-Host_associated-Mammals-Digestive_system-Large_intestine-Cecum
SRR4125122	MGYA00099281	https://www.ebi.ac.uk/metagenomics/api/v1/analyses/MGYA00099281/file/SRR4125122_MERGED_FASTQ_otu.tsv	Root-Host_associated-Mammals-Digestive_system-Large_intestine-Cecum
ERR537717	MGYA00218410	https://www.ebi.ac.uk/metagenomics/api/v1/analyses/MGYA00218410/file/ERR537717_MERGED_FASTQ_SSU_OTU.tsv	Root-Host_associated-Mammals-Digestive_system-Large_intestine-Cecum
ERR537648	MGYA00218354	https://www.ebi.ac.uk/metagenomics/api/v1/analyses/MGYA00218354/file/ERR537648_MERGED_FASTQ_SSU_OTU.tsv	Root-Host_associated-Mammals-Digestive_system-Large_intestine-Cecum
SRR4125125	MGYA00099285	https://www.ebi.ac.uk/metagenomics/api/v1/analyses/MGYA00099285/file/SRR4125125_MERGED_FASTQ_otu.tsv	Root-Host_associated-Mammals-Digestive_system-Large_intestine-Cecum
ERR537699	MGYA00218317	https://www.ebi.ac.uk/metagenomics/api/v1/analyses/MGYA00218317/file/ERR537699_MERGED_FASTQ_SSU_OTU.tsv	Root-Host_associated-Mammals-Digestive_system-Large_intestine-Cecum
ERR537710	MGYA00218308	https://www.ebi.ac.uk/metagenomics/api/v1/analyses/MGYA00218308/file/ERR537710_MERGED_FASTQ_SSU_OTU.tsv	Root-Host_associated-Mammals-Digestive_system-Large_intestine-Cecum
SRR4125110	MGYA00099295	https://www.ebi.ac.uk/metagenomics/api/v1/analyses/MGYA00099295/file/SRR4125110_MERGED_FASTQ_otu.tsv	Root-Host_associated-Mammals-Digestive_system-Large_intestine-Cecum
SRR4125099	MGYA00099310	https://www.ebi.ac.uk/metagenomics/api/v1/analyses/MGYA00099310/file/SRR4125099_MERGED_FASTQ_otu.tsv	Root-Host_associated-Mammals-Digestive_system-Large_intestine-Cecum
ERR537725	MGYA00218401	https://www.ebi.ac.uk/metagenomics/api/v1/analyses/MGYA00218401/file/ERR537725_MERGED_FASTQ_SSU_OTU.tsv	Root-Host_associated-Mammals-Digestive_system-Large_intestine-Cecum
ERR537759	MGYA00218345	https://www.ebi.ac.uk/metagenomics/api/v1/analyses/MGYA00218345/file/ERR537759_MERGED_FASTQ_SSU_OTU.tsv	Root-Host_associated-Mammals-Digestive_system-Large_intestine-Cecum

SRR4125117	MGYA00099284	https://www.ebi.ac.uk/metagenomics/api/v1/analyses/MGYA00099284/file/SRR4125117_MERGED_FASTQ_otu.tsv	Root-Host_associated-Mammals-Digestive_system-Large_intestine-Cecum
ERR537722	MGYA00218278	https://www.ebi.ac.uk/metagenomics/api/v1/analyses/MGYA00218278/file/ERR537722_MERGED_FASTQ_SSU_OTU.tsv	Root-Host_associated-Mammals-Digestive_system-Large_intestine-Cecum
ERR537750	MGYA00218396	https://www.ebi.ac.uk/metagenomics/api/v1/analyses/MGYA00218396/file/ERR537750_MERGED_FASTQ_SSU_OTU.tsv	Root-Host_associated-Mammals-Digestive_system-Large_intestine-Cecum
SRR4125119	MGYA00099324	https://www.ebi.ac.uk/metagenomics/api/v1/analyses/MGYA00099324/file/SRR4125119_MERGED_FASTQ_otu.tsv	Root-Host_associated-Mammals-Digestive_system-Large_intestine-Cecum
ERR537674	MGYA00218405	https://www.ebi.ac.uk/metagenomics/api/v1/analyses/MGYA00218405/file/ERR537674_MERGED_FASTQ_SSU_OTU.tsv	Root-Host_associated-Mammals-Digestive_system-Large_intestine-Cecum
ERR537673	MGYA00218304	https://www.ebi.ac.uk/metagenomics/api/v1/analyses/MGYA00218304/file/ERR537673_MERGED_FASTQ_SSU_OTU.tsv	Root-Host_associated-Mammals-Digestive_system-Large_intestine-Cecum
SRR4125097	MGYA00099302	https://www.ebi.ac.uk/metagenomics/api/v1/analyses/MGYA00099302/file/SRR4125097_MERGED_FASTQ_otu.tsv	Root-Host_associated-Mammals-Digestive_system-Large_intestine-Cecum
ERR537757	MGYA00218346	https://www.ebi.ac.uk/metagenomics/api/v1/analyses/MGYA00218346/file/ERR537757_MERGED_FASTQ_SSU_OTU.tsv	Root-Host_associated-Mammals-Digestive_system-Large_intestine-Cecum
ERR537662	MGYA00218295	https://www.ebi.ac.uk/metagenomics/api/v1/analyses/MGYA00218295/file/ERR537662_MERGED_FASTQ_SSU_OTU.tsv	Root-Host_associated-Mammals-Digestive_system-Large_intestine-Cecum
ERR537746	MGYA00218411	https://www.ebi.ac.uk/metagenomics/api/v1/analyses/MGYA00218411/file/ERR537746_MERGED_FASTQ_SSU_OTU.tsv	Root-Host_associated-Mammals-Digestive_system-Large_intestine-Cecum
ERR537741	MGYA00218373	https://www.ebi.ac.uk/metagenomics/api/v1/analyses/MGYA00218373/file/ERR537741_MERGED_FASTQ_SSU_OTU.tsv	Root-Host_associated-Mammals-Digestive_system-Large_intestine-Cecum
ERR537665	MGYA00218397	https://www.ebi.ac.uk/metagenomics/api/v1/analyses/MGYA00218397/file/ERR537665_MERGED_FASTQ_SSU_OTU.tsv	Root-Host_associated-Mammals-Digestive_system-Large_intestine-Cecum

SRR4125108	MGYA00099292	https://www.ebi.ac.uk/metagenomics/api/v1/analyses/MGYA00099292/file/SRR4125108_MERGED_FASTQ_otu.tsv	Root-Host_associated-Mammals-Digestive_system-Large_intestine-Cecum
ERR537733	MGYA00218303	https://www.ebi.ac.uk/metagenomics/api/v1/analyses/MGYA00218303/file/ERR537733_MERGED_FASTQ_SSU_OTU.tsv	Root-Host_associated-Mammals-Digestive_system-Large_intestine-Cecum
SRR4125106	MGYA00099326	https://www.ebi.ac.uk/metagenomics/api/v1/analyses/MGYA00099326/file/SRR4125106_MERGED_FASTQ_otu.tsv	Root-Host_associated-Mammals-Digestive_system-Large_intestine-Cecum
ERR537734	MGYA00218399	https://www.ebi.ac.uk/metagenomics/api/v1/analyses/MGYA00218399/file/ERR537734_MERGED_FASTQ_SSU_OTU.tsv	Root-Host_associated-Mammals-Digestive_system-Large_intestine-Cecum
SRR4125101	MGYA00099328	https://www.ebi.ac.uk/metagenomics/api/v1/analyses/MGYA00099328/file/SRR4125101_MERGED_FASTQ_otu.tsv	Root-Host_associated-Mammals-Digestive_system-Large_intestine-Cecum
ERR537748	MGYA00218302	https://www.ebi.ac.uk/metagenomics/api/v1/analyses/MGYA00218302/file/ERR537748_MERGED_FASTQ_SSU_OTU.tsv	Root-Host_associated-Mammals-Digestive_system-Large_intestine-Cecum
ERR537701	MGYA00218361	https://www.ebi.ac.uk/metagenomics/api/v1/analyses/MGYA00218361/file/ERR537701_MERGED_FASTQ_SSU_OTU.tsv	Root-Host_associated-Mammals-Digestive_system-Large_intestine-Cecum
SRR4125134	MGYA00099290	https://www.ebi.ac.uk/metagenomics/api/v1/analyses/MGYA00099290/file/SRR4125134_MERGED_FASTQ_otu.tsv	Root-Host_associated-Mammals-Digestive_system-Large_intestine-Cecum
ERR537688	MGYA00218358	https://www.ebi.ac.uk/metagenomics/api/v1/analyses/MGYA00218358/file/ERR537688_MERGED_FASTQ_SSU_OTU.tsv	Root-Host_associated-Mammals-Digestive_system-Large_intestine-Cecum
ERR537659	MGYA00218389	https://www.ebi.ac.uk/metagenomics/api/v1/analyses/MGYA00218389/file/ERR537659_MERGED_FASTQ_SSU_OTU.tsv	Root-Host_associated-Mammals-Digestive_system-Large_intestine-Cecum
SRR4125148	MGYA00099306	https://www.ebi.ac.uk/metagenomics/api/v1/analyses/MGYA00099306/file/SRR4125148_MERGED_FASTQ_otu.tsv	Root-Host_associated-Mammals-Digestive_system-Large_intestine-Cecum
ERR537706	MGYA00218294	https://www.ebi.ac.uk/metagenomics/api/v1/analyses/MGYA00218294/file/ERR537706_MERGED_FASTQ_SSU_OTU.tsv	Root-Host_associated-Mammals-Digestive_system-Large_intestine-Cecum

SRR4125133	MGYA00099318	https://www.ebi.ac.uk/metagenomics/api/v1/analyses/MGYA00099318/file/SRR4125133_MERGED_FASTQ_otu.tsv	Root-Host_associated-Mammals-Digestive_system-Large_intestine-Cecum
ERR537774	MGYA00218409	https://www.ebi.ac.uk/metagenomics/api/v1/analyses/MGYA00218409/file/ERR537774_MERGED_FASTQ_SSU_OTU.tsv	Root-Host_associated-Mammals-Digestive_system-Large_intestine-Cecum
SRR4125141	MGYA00099314	https://www.ebi.ac.uk/metagenomics/api/v1/analyses/MGYA00099314/file/SRR4125141_MERGED_FASTQ_otu.tsv	Root-Host_associated-Mammals-Digestive_system-Large_intestine-Cecum
ERR537708	MGYA00218348	https://www.ebi.ac.uk/metagenomics/api/v1/analyses/MGYA00218348/file/ERR537708_MERGED_FASTQ_SSU_OTU.tsv	Root-Host_associated-Mammals-Digestive_system-Large_intestine-Cecum
ERR537681	MGYA00218349	https://www.ebi.ac.uk/metagenomics/api/v1/analyses/MGYA00218349/file/ERR537681_MERGED_FASTQ_SSU_OTU.tsv	Root-Host_associated-Mammals-Digestive_system-Large_intestine-Cecum
ERR537650	MGYA00218276	https://www.ebi.ac.uk/metagenomics/api/v1/analyses/MGYA00218276/file/ERR537650_MERGED_FASTQ_SSU_OTU.tsv	Root-Host_associated-Mammals-Digestive_system-Large_intestine-Cecum
ERR537686	MGYA00218364	https://www.ebi.ac.uk/metagenomics/api/v1/analyses/MGYA00218364/file/ERR537686_MERGED_FASTQ_SSU_OTU.tsv	Root-Host_associated-Mammals-Digestive_system-Large_intestine-Cecum
ERR537657	MGYA00218322	https://www.ebi.ac.uk/metagenomics/api/v1/analyses/MGYA00218322/file/ERR537657_MERGED_FASTQ_SSU_OTU.tsv	Root-Host_associated-Mammals-Digestive_system-Large_intestine-Cecum
ERR537773	MGYA00218325	https://www.ebi.ac.uk/metagenomics/api/v1/analyses/MGYA00218325/file/ERR537773_MERGED_FASTQ_SSU_OTU.tsv	Root-Host_associated-Mammals-Digestive_system-Large_intestine-Cecum
SRR4125146	MGYA00099294	https://www.ebi.ac.uk/metagenomics/api/v1/analyses/MGYA00099294/file/SRR4125146_MERGED_FASTQ_otu.tsv	Root-Host_associated-Mammals-Digestive_system-Large_intestine-Cecum
ERR537672	MGYA00218297	https://www.ebi.ac.uk/metagenomics/api/v1/analyses/MGYA00218297/file/ERR537672_MERGED_FASTQ_SSU_OTU.tsv	Root-Host_associated-Mammals-Digestive_system-Large_intestine-Cecum
SRR4125096	MGYA00099287	https://www.ebi.ac.uk/metagenomics/api/v1/analyses/MGYA00099287/file/SRR4125096_MERGED_FASTQ_otu.tsv	Root-Host_associated-Mammals-Digestive_system-Large_intestine-Cecum

ERR537756	MGYA00218309	https://www.ebi.ac.uk/metagenomics/api/v1/analyses/MGYA00218309/file/ERR537756_MERGED_FASTQ_SSU_OTU.tsv	Root-Host_associated-Mammals-Digestive_system-Large_intestine-Cecum
ERR537751	MGYA00218340	https://www.ebi.ac.uk/metagenomics/api/v1/analyses/MGYA00218340/file/ERR537751_MERGED_FASTQ_SSU_OTU.tsv	Root-Host_associated-Mammals-Digestive_system-Large_intestine-Cecum
SRR4125118	MGYA00099337	https://www.ebi.ac.uk/metagenomics/api/v1/analyses/MGYA00099337/file/SRR4125118_MERGED_FASTQ_otu.tsv	Root-Host_associated-Mammals-Digestive_system-Large_intestine-Cecum
ERR537675	MGYA00218394	https://www.ebi.ac.uk/metagenomics/api/v1/analyses/MGYA00218394/file/ERR537675_MERGED_FASTQ_SSU_OTU.tsv	Root-Host_associated-Mammals-Digestive_system-Large_intestine-Cecum
SRR4125116	MGYA00099293	https://www.ebi.ac.uk/metagenomics/api/v1/analyses/MGYA00099293/file/SRR4125116_MERGED_FASTQ_otu.tsv	Root-Host_associated-Mammals-Digestive_system-Large_intestine-Cecum
ERR537723	MGYA00218366	https://www.ebi.ac.uk/metagenomics/api/v1/analyses/MGYA00218366/file/ERR537723_MERGED_FASTQ_SSU_OTU.tsv	Root-Host_associated-Mammals-Digestive_system-Large_intestine-Cecum
SRR4125111	MGYA00099330	https://www.ebi.ac.uk/metagenomics/api/v1/analyses/MGYA00099330/file/SRR4125111_MERGED_FASTQ_otu.tsv	Root-Host_associated-Mammals-Digestive_system-Large_intestine-Cecum
SRR4125098	MGYA00099301	https://www.ebi.ac.uk/metagenomics/api/v1/analyses/MGYA00099301/file/SRR4125098_MERGED_FASTQ_otu.tsv	Root-Host_associated-Mammals-Digestive_system-Large_intestine-Cecum
ERR537724	MGYA00218400	https://www.ebi.ac.uk/metagenomics/api/v1/analyses/MGYA00218400/file/ERR537724_MERGED_FASTQ_SSU_OTU.tsv	Root-Host_associated-Mammals-Digestive_system-Large_intestine-Cecum
ERR537758	MGYA00218279	https://www.ebi.ac.uk/metagenomics/api/v1/analyses/MGYA00218279/file/ERR537758_MERGED_FASTQ_SSU_OTU.tsv	Root-Host_associated-Mammals-Digestive_system-Large_intestine-Cecum
ERR537649	MGYA00218288	https://www.ebi.ac.uk/metagenomics/api/v1/analyses/MGYA00218288/file/ERR537649_MERGED_FASTQ_SSU_OTU.tsv	Root-Host_associated-Mammals-Digestive_system-Large_intestine-Cecum
SRR4125124	MGYA00099307	https://www.ebi.ac.uk/metagenomics/api/v1/analyses/MGYA00099307/file/SRR4125124_MERGED_FASTQ_otu.tsv	Root-Host_associated-Mammals-Digestive_system-Large_intestine-Cecum

ERR537698	MGYA00218353	https://www.ebi.ac.uk/metagenomics/api/v1/analyses/MGYA00218353/file/ERR537698_MERGED_FASTQ_SSU_OTU.tsv	Root-Host_associated-Mammals-Digestive_system-Large_intestine-Cecum
ERR537711	MGYA00218344	https://www.ebi.ac.uk/metagenomics/api/v1/analyses/MGYA00218344/file/ERR537711_MERGED_FASTQ_SSU_OTU.tsv	Root-Host_associated-Mammals-Digestive_system-Large_intestine-Cecum
SRR4125123	MGYA00099280	https://www.ebi.ac.uk/metagenomics/api/v1/analyses/MGYA00099280/file/SRR4125123_MERGED_FASTQ_otu.tsv	Root-Host_associated-Mammals-Digestive_system-Large_intestine-Cecum
ERR537716	MGYA00218342	https://www.ebi.ac.uk/metagenomics/api/v1/analyses/MGYA00218342/file/ERR537716_MERGED_FASTQ_SSU_OTU.tsv	Root-Host_associated-Mammals-Digestive_system-Large_intestine-Cecum
SRR4125151	MGYA00099321	https://www.ebi.ac.uk/metagenomics/api/v1/analyses/MGYA00099321/file/SRR4125151_MERGED_FASTQ_otu.tsv	Root-Host_associated-Mammals-Digestive_system-Large_intestine-Cecum
ERR537764	MGYA00218296	https://www.ebi.ac.uk/metagenomics/api/v1/analyses/MGYA00218296/file/ERR537764_MERGED_FASTQ_SSU_OTU.tsv	Root-Host_associated-Mammals-Digestive_system-Large_intestine-Cecum
ERR537640	MGYA00218355	https://www.ebi.ac.uk/metagenomics/api/v1/analyses/MGYA00218355/file/ERR537640_MERGED_FASTQ_SSU_OTU.tsv	Root-Host_associated-Mammals-Digestive_system-Large_intestine-Cecum
ERR537691	MGYA00218328	https://www.ebi.ac.uk/metagenomics/api/v1/analyses/MGYA00218328/file/ERR537691_MERGED_FASTQ_SSU_OTU.tsv	Root-Host_associated-Mammals-Digestive_system-Large_intestine-Cecum
ERR537718	MGYA00218291	https://www.ebi.ac.uk/metagenomics/api/v1/analyses/MGYA00218291/file/ERR537718_MERGED_FASTQ_SSU_OTU.tsv	Root-Host_associated-Mammals-Digestive_system-Large_intestine-Cecum
ERR537647	MGYA00218287	https://www.ebi.ac.uk/metagenomics/api/v1/analyses/MGYA00218287/file/ERR537647_MERGED_FASTQ_SSU_OTU.tsv	Root-Host_associated-Mammals-Digestive_system-Large_intestine-Cecum
ERR537696	MGYA00218372	https://www.ebi.ac.uk/metagenomics/api/v1/analyses/MGYA00218372/file/ERR537696_MERGED_FASTQ_SSU_OTU.tsv	Root-Host_associated-Mammals-Digestive_system-Large_intestine-Cecum
ERR537763	MGYA00218382	https://www.ebi.ac.uk/metagenomics/api/v1/analyses/MGYA00218382/file/ERR537763_MERGED_FASTQ_SSU_OTU.tsv	Root-Host_associated-Mammals-Digestive_system-Large_intestine-Cecum

ERR537687	MGYA00218305	https://www.ebi.ac.uk/metagenomics/api/v1/analyses/MGYA00218305/file/ERR537687_MERGED_FASTQ_SSU_OTU.tsv	Root-Host_associated-Mammals-Digestive_system-Large_intestine-Cecum
ERR537656	MGYA00218356	https://www.ebi.ac.uk/metagenomics/api/v1/analyses/MGYA00218356/file/ERR537656_MERGED_FASTQ_SSU_OTU.tsv	Root-Host_associated-Mammals-Digestive_system-Large_intestine-Cecum
ERR537772	MGYA00218332	https://www.ebi.ac.uk/metagenomics/api/v1/analyses/MGYA00218332/file/ERR537772_MERGED_FASTQ_SSU_OTU.tsv	Root-Host_associated-Mammals-Digestive_system-Large_intestine-Cecum
SRR4125147	MGYA00099331	https://www.ebi.ac.uk/metagenomics/api/v1/analyses/MGYA00099331/file/SRR4125147_MERGED_FASTQ_otu.tsv	Root-Host_associated-Mammals-Digestive_system-Large_intestine-Cecum
ERR537775	MGYA00218315	https://www.ebi.ac.uk/metagenomics/api/v1/analyses/MGYA00218315/file/ERR537775_MERGED_FASTQ_SSU_OTU.tsv	Root-Host_associated-Mammals-Digestive_system-Large_intestine-Cecum
SRR4125140	MGYA00099288	https://www.ebi.ac.uk/metagenomics/api/v1/analyses/MGYA00099288/file/SRR4125140_MERGED_FASTQ_otu.tsv	Root-Host_associated-Mammals-Digestive_system-Large_intestine-Cecum
ERR537709	MGYA00218393	https://www.ebi.ac.uk/metagenomics/api/v1/analyses/MGYA00218393/file/ERR537709_MERGED_FASTQ_SSU_OTU.tsv	Root-Host_associated-Mammals-Digestive_system-Large_intestine-Cecum
ERR537680	MGYA00218331	https://www.ebi.ac.uk/metagenomics/api/v1/analyses/MGYA00218331/file/ERR537680_MERGED_FASTQ_SSU_OTU.tsv	Root-Host_associated-Mammals-Digestive_system-Large_intestine-Cecum
ERR537651	MGYA00218407	https://www.ebi.ac.uk/metagenomics/api/v1/analyses/MGYA00218407/file/ERR537651_MERGED_FASTQ_SSU_OTU.tsv	Root-Host_associated-Mammals-Digestive_system-Large_intestine-Cecum
ERR537707	MGYA00218408	https://www.ebi.ac.uk/metagenomics/api/v1/analyses/MGYA00218408/file/ERR537707_MERGED_FASTQ_SSU_OTU.tsv	Root-Host_associated-Mammals-Digestive_system-Large_intestine-Cecum
SRR4125132	MGYA00099299	https://www.ebi.ac.uk/metagenomics/api/v1/analyses/MGYA00099299/file/SRR4125132_MERGED_FASTQ_otu.tsv	Root-Host_associated-Mammals-Digestive_system-Large_intestine-Cecum
ERR537700	MGYA00218390	https://www.ebi.ac.uk/metagenomics/api/v1/analyses/MGYA00218390/file/ERR537700_MERGED_FASTQ_SSU_OTU.tsv	Root-Host_associated-Mammals-Digestive_system-Large_intestine-Cecum

SRR4125135	MGYA00099329	https://www.ebi.ac.uk/metagenomics/api/v1/analyses/MGYA00099329/file/SRR4125135_MERGED_FASTQ_otu.tsv	Root-Host_associated-Mammals-Digestive_system-Large_intestine-Cecum
ERR537689	MGYA00218300	https://www.ebi.ac.uk/metagenomics/api/v1/analyses/MGYA00218300/file/ERR537689_MERGED_FASTQ_SSU_OTU.tsv	Root-Host_associated-Mammals-Digestive_system-Large_intestine-Cecum
ERR537658	MGYA00218395	https://www.ebi.ac.uk/metagenomics/api/v1/analyses/MGYA00218395/file/ERR537658_MERGED_FASTQ_SSU_OTU.tsv	Root-Host_associated-Mammals-Digestive_system-Large_intestine-Cecum
SRR4125149	MGYA00099286	https://www.ebi.ac.uk/metagenomics/api/v1/analyses/MGYA00099286/file/SRR4125149_MERGED_FASTQ_otu.tsv	Root-Host_associated-Mammals-Digestive_system-Large_intestine-Cecum
ERR537735	MGYA00218386	https://www.ebi.ac.uk/metagenomics/api/v1/analyses/MGYA00218386/file/ERR537735_MERGED_FASTQ_SSU_OTU.tsv	Root-Host_associated-Mammals-Digestive_system-Large_intestine-Cecum
SRR4125100	MGYA00099320	https://www.ebi.ac.uk/metagenomics/api/v1/analyses/MGYA00099320/file/SRR4125100_MERGED_FASTQ_otu.tsv	Root-Host_associated-Mammals-Digestive_system-Large_intestine-Cecum
ERR537749	MGYA00218275	https://www.ebi.ac.uk/metagenomics/api/v1/analyses/MGYA00218275/file/ERR537749_MERGED_FASTQ_SSU_OTU.tsv	Root-Host_associated-Mammals-Digestive_system-Large_intestine-Cecum
ERR537732	MGYA00218323	https://www.ebi.ac.uk/metagenomics/api/v1/analyses/MGYA00218323/file/ERR537732_MERGED_FASTQ_SSU_OTU.tsv	Root-Host_associated-Mammals-Digestive_system-Large_intestine-Cecum
SRR4125107	MGYA00099300	https://www.ebi.ac.uk/metagenomics/api/v1/analyses/MGYA00099300/file/SRR4125107_MERGED_FASTQ_otu.tsv	Root-Host_associated-Mammals-Digestive_system-Large_intestine-Cecum
ERR537740	MGYA00218377	https://www.ebi.ac.uk/metagenomics/api/v1/analyses/MGYA00218377/file/ERR537740_MERGED_FASTQ_SSU_OTU.tsv	Root-Host_associated-Mammals-Digestive_system-Large_intestine-Cecum
ERR537664	MGYA00218347	https://www.ebi.ac.uk/metagenomics/api/v1/analyses/MGYA00218347/file/ERR537664_MERGED_FASTQ_SSU_OTU.tsv	Root-Host_associated-Mammals-Digestive_system-Large_intestine-Cecum
SRR4125109	MGYA00099316	https://www.ebi.ac.uk/metagenomics/api/v1/analyses/MGYA00099316/file/SRR4125109_MERGED_FASTQ_otu.tsv	Root-Host_associated-Mammals-Digestive_system-Large_intestine-Cecum

ERR537663	MGYA00218321	https://www.ebi.ac.uk/metagenomics/api/v1/analyses/MGYA00218321/file/ERR537663_MERGED_FASTQ_SSU_OTU.tsv	Root-Host_associated-Mammals-Digestive_system-Large_intestine-Cecum
ERR537747	MGYA00218360	https://www.ebi.ac.uk/metagenomics/api/v1/analyses/MGYA00218360/file/ERR537747_MERGED_FASTQ_SSU_OTU.tsv	Root-Host_associated-Mammals-Digestive_system-Large_intestine-Cecum
SRR5870132	MGYA00416415	https://www.ebi.ac.uk/metagenomics/api/v1/analyses/MGYA00416415/file/SRR5870132_MERGED_FASTQ_SSU_OTU.tsv	Root-Host_associated-Human-Skin
SRR995142	MGYA00024138	https://www.ebi.ac.uk/metagenomics/api/v1/analyses/MGYA00024138/file/SRR995142_FASTQ_otu.tsv	Root-Host_associated-Human-Skin
SRR996391	MGYA00024979	https://www.ebi.ac.uk/metagenomics/api/v1/analyses/MGYA00024979/file/SRR996391_FASTQ_otu.tsv	Root-Host_associated-Human-Skin
SRR919592	MGYA00024951	https://www.ebi.ac.uk/metagenomics/api/v1/analyses/MGYA00024951/file/SRR919592_FASTQ_otu.tsv	Root-Host_associated-Human-Skin
SRR996436	MGYA00024473	https://www.ebi.ac.uk/metagenomics/api/v1/analyses/MGYA00024473/file/SRR996436_FASTQ_otu.tsv	Root-Host_associated-Human-Skin
SRR995523	MGYA00023621	https://www.ebi.ac.uk/metagenomics/api/v1/analyses/MGYA00023621/file/SRR995523_FASTQ_otu.tsv	Root-Host_associated-Human-Skin
SRR1632408	MGYA00023545	https://www.ebi.ac.uk/metagenomics/api/v1/analyses/MGYA00023545/file/SRR1632408_MERGED_FASTQ_otu.tsv	Root-Host_associated-Human-Skin
SRR5870225	MGYA00426194	https://www.ebi.ac.uk/metagenomics/api/v1/analyses/MGYA00426194/file/SRR5870225_MERGED_FASTQ_SSU_OTU.tsv	Root-Host_associated-Human-Skin
SRR994867	MGYA00022393	https://www.ebi.ac.uk/metagenomics/api/v1/analyses/MGYA00022393/file/SRR994867_FASTQ_otu.tsv	Root-Host_associated-Human-Skin
ERR443769	MGYA00413310	https://www.ebi.ac.uk/metagenomics/api/v1/analyses/MGYA00413310/file/ERR443769_FASTQ_SSU_OTU.tsv	Root-Host_associated-Human-Skin

SRR995980	MGYA00022935	https://www.ebi.ac.uk/metagenomics/api/v1/analyses/MGYA00022935/file/SRR995980_FASTQ_otu.tsv	Root-Host_associated-Human-Skin
ERR442021	MGYA00385219	https://www.ebi.ac.uk/metagenomics/api/v1/analyses/MGYA00385219/file/ERR442021_FASTQ_SSU_OTU.tsv	Root-Host_associated-Human-Skin
SRR996340	MGYA00022277	https://www.ebi.ac.uk/metagenomics/api/v1/analyses/MGYA00022277/file/SRR996340_FASTQ_otu.tsv	Root-Host_associated-Human-Skin
SRR996895	MGYA00022794	https://www.ebi.ac.uk/metagenomics/api/v1/analyses/MGYA00022794/file/SRR996895_FASTQ_otu.tsv	Root-Host_associated-Human-Skin
SRR1704508	MGYA00426927	https://www.ebi.ac.uk/metagenomics/api/v1/analyses/MGYA00426927/file/SRR1704508_FASTQ_SSU_OTU.tsv	Root-Host_associated-Human-Skin
SRR921019	MGYA00023332	https://www.ebi.ac.uk/metagenomics/api/v1/analyses/MGYA00023332/file/SRR921019_FASTQ_otu.tsv	Root-Host_associated-Human-Skin
SRR995634	MGYA00022341	https://www.ebi.ac.uk/metagenomics/api/v1/analyses/MGYA00022341/file/SRR995634_FASTQ_otu.tsv	Root-Host_associated-Human-Skin
SRR920659	MGYA00023729	https://www.ebi.ac.uk/metagenomics/api/v1/analyses/MGYA00023729/file/SRR920659_FASTQ_otu.tsv	Root-Host_associated-Human-Skin
SRR996721	MGYA00023432	https://www.ebi.ac.uk/metagenomics/api/v1/analyses/MGYA00023432/file/SRR996721_FASTQ_otu.tsv	Root-Host_associated-Human-Skin
SRR5869388	MGYA00418741	https://www.ebi.ac.uk/metagenomics/api/v1/analyses/MGYA00418741/file/SRR5869388_MERGED_FASTQ_SSU_OTU.tsv	Root-Host_associated-Human-Skin
SRR996086	MGYA00024360	https://www.ebi.ac.uk/metagenomics/api/v1/analyses/MGYA00024360/file/SRR996086_FASTQ_otu.tsv	Root-Host_associated-Human-Skin
SRR919869	MGYA00023905	https://www.ebi.ac.uk/metagenomics/api/v1/analyses/MGYA00023905/file/SRR919869_FASTQ_otu.tsv	Root-Host_associated-Human-Skin

SRR1704450	MGYA00424944	https://www.ebi.ac.uk/metagenomics/api/v1/analyses/MGYA00424944/file/SRR1704450_FASTQ_SSU_OTU.tsv	Root-Host_associated-Human-Skin
SRR1620021	MGYA00024919	https://www.ebi.ac.uk/metagenomics/api/v1/analyses/MGYA00024919/file/SRR1620021_MERGED_FASTQ_otu.tsv	Root-Host_associated-Human-Skin
SRR1922543	MGYA00028406	https://www.ebi.ac.uk/metagenomics/api/v1/analyses/MGYA00028406/file/SRR1922543_FASTQ_otu.tsv	Root-Host_associated-Human-Skin
ERR1960823	MGYA00130738	https://www.ebi.ac.uk/metagenomics/api/v1/analyses/MGYA00130738/file/ERR1960823_MERGED_FASTQ_otu.tsv	Root-Host_associated-Human-Skin
ERR981213	MGYA00010281	https://www.ebi.ac.uk/metagenomics/api/v1/analyses/MGYA00010281/file/ERR981213_MERGED_FASTQ_otu.tsv	Root-Host_associated-Human-Skin
SRR997039	MGYA00023609	https://www.ebi.ac.uk/metagenomics/api/v1/analyses/MGYA00023609/file/SRR997039_FASTQ_otu.tsv	Root-Host_associated-Human-Skin
ERR2538413	MGYA00381420	https://www.ebi.ac.uk/metagenomics/api/v1/analyses/MGYA00381420/file/ERR2538413_FASTQ_SSU_OTU.tsv	Root-Host_associated-Human-Skin
SRR921141	MGYA00024585	https://www.ebi.ac.uk/metagenomics/api/v1/analyses/MGYA00024585/file/SRR921141_FASTQ_otu.tsv	Root-Host_associated-Human-Skin
ERR1960797	MGYA00130802	https://www.ebi.ac.uk/metagenomics/api/v1/analyses/MGYA00130802/file/ERR1960797_MERGED_FASTQ_otu.tsv	Root-Host_associated-Human-Skin
SRR920701	MGYA00024016	https://www.ebi.ac.uk/metagenomics/api/v1/analyses/MGYA00024016/file/SRR920701_FASTQ_otu.tsv	Root-Host_associated-Human-Skin
ERR1701234	MGYA00084325	https://www.ebi.ac.uk/metagenomics/api/v1/analyses/MGYA00084325/file/ERR1701234_FASTQ_otu.tsv	Root-Host_associated-Human-Skin
ERR443281	MGYA00416671	https://www.ebi.ac.uk/metagenomics/api/v1/analyses/MGYA00416671/file/ERR443281_FASTQ_SSU_OTU.tsv	Root-Host_associated-Human-Skin

ERR1960746	MGYA00130715	https://www.ebi.ac.uk/metagenomics/api/v1/analyses/MGYA00130715/file/ERR1960746_MERGED_FASTQ_otu.tsv	Root-Host_associated-Human-Skin
ERR2538365	MGYA00381372	https://www.ebi.ac.uk/metagenomics/api/v1/analyses/MGYA00381372/file/ERR2538365_FASTQ_SSU_OTU.tsv	Root-Host_associated-Human-Skin
SRR1622811	MGYA00023981	https://www.ebi.ac.uk/metagenomics/api/v1/analyses/MGYA00023981/file/SRR1622811_MERGED_FASTQ_otu.tsv	Root-Host_associated-Human-Skin
SRR6757632	MGYA00380749	https://www.ebi.ac.uk/metagenomics/api/v1/analyses/MGYA00380749/file/SRR6757632_MERGED_FASTQ_SSU_OTU.tsv	Root-Host_associated-Human-Skin
SRR5868833	MGYA00427135	https://www.ebi.ac.uk/metagenomics/api/v1/analyses/MGYA00427135/file/SRR5868833_MERGED_FASTQ_SSU_OTU.tsv	Root-Host_associated-Human-Skin
ERR451164	MGYA00426089	https://www.ebi.ac.uk/metagenomics/api/v1/analyses/MGYA00426089/file/ERR451164_FASTQ_SSU_OTU.tsv	Root-Host_associated-Human-Skin
SRR1704326	MGYA00421506	https://www.ebi.ac.uk/metagenomics/api/v1/analyses/MGYA00421506/file/SRR1704326_FASTQ_SSU_OTU.tsv	Root-Host_associated-Human-Skin
SRR5868787	MGYA00421648	https://www.ebi.ac.uk/metagenomics/api/v1/analyses/MGYA00421648/file/SRR5868787_MERGED_FASTQ_SSU_OTU.tsv	Root-Host_associated-Human-Skin
SRR1950693	MGYA00376860	https://www.ebi.ac.uk/metagenomics/api/v1/analyses/MGYA00376860/file/SRR1950693_MERGED_FASTQ_SSU_OTU.tsv	Root-Host_associated-Human-Skin
SRR2005645	MGYA00381304	https://www.ebi.ac.uk/metagenomics/api/v1/analyses/MGYA00381304/file/SRR2005645_MERGED_FASTQ_SSU_OTU.tsv	Root-Host_associated-Human-Skin
SRR920153	MGYA00024516	https://www.ebi.ac.uk/metagenomics/api/v1/analyses/MGYA00024516/file/SRR920153_FASTQ_otu.tsv	Root-Host_associated-Human-Skin
ERR1960662	MGYA00130780	https://www.ebi.ac.uk/metagenomics/api/v1/analyses/MGYA00130780/file/ERR1960662_MERGED_FASTQ_otu.tsv	Root-Host_associated-Human-Skin

SRR995699	MGYA00023001	https://www.ebi.ac.uk/metagenomics/api/v1/analyses/MGYA00023001/file/SRR995699_FASTQ_otu.tsv	Root-Host_associated-Human-Skin
SRR1622935	MGYA00025291	https://www.ebi.ac.uk/metagenomics/api/v1/analyses/MGYA00025291/file/SRR1622935_MERGED_FASTQ_otu.tsv	Root-Host_associated-Human-Skin
SRR1923923	MGYA00028273	https://www.ebi.ac.uk/metagenomics/api/v1/analyses/MGYA00028273/file/SRR1923923_FASTQ_otu.tsv	Root-Host_associated-Human-Skin
SRR1632474	MGYA00022486	https://www.ebi.ac.uk/metagenomics/api/v1/analyses/MGYA00022486/file/SRR1632474_MERGED_FASTQ_otu.tsv	Root-Host_associated-Human-Skin
SRR996838	MGYA00023873	https://www.ebi.ac.uk/metagenomics/api/v1/analyses/MGYA00023873/file/SRR996838_FASTQ_otu.tsv	Root-Host_associated-Human-Skin
SRR5869744	MGYA00415886	https://www.ebi.ac.uk/metagenomics/api/v1/analyses/MGYA00415886/file/SRR5869744_MERGED_FASTQ_SSU_OTU.tsv	Root-Host_associated-Human-Skin
SRR5869032	MGYA00420727	https://www.ebi.ac.uk/metagenomics/api/v1/analyses/MGYA00420727/file/SRR5869032_MERGED_FASTQ_SSU_OTU.tsv	Root-Host_associated-Human-Skin
SRR1950766	MGYA00376933	https://www.ebi.ac.uk/metagenomics/api/v1/analyses/MGYA00376933/file/SRR1950766_MERGED_FASTQ_SSU_OTU.tsv	Root-Host_associated-Human-Skin
ERR1759930	MGYA00140526	https://www.ebi.ac.uk/metagenomics/api/v1/analyses/MGYA00140526/file/ERR1759930_MERGED_FASTQ_SSU_OTU.tsv	Root-Host_associated-Human-Skin
SRR920991	MGYA00023249	https://www.ebi.ac.uk/metagenomics/api/v1/analyses/MGYA00023249/file/SRR920991_FASTQ_otu.tsv	Root-Host_associated-Human-Skin
SRR1632302	MGYA00023270	https://www.ebi.ac.uk/metagenomics/api/v1/analyses/MGYA00023270/file/SRR1632302_MERGED_FASTQ_otu.tsv	Root-Host_associated-Human-Skin
SRR920244	MGYA00023492	https://www.ebi.ac.uk/metagenomics/api/v1/analyses/MGYA00023492/file/SRR920244_FASTQ_otu.tsv	Root-Host_associated-Human-Skin

SRR1631676	MGYA00023829	https://www.ebi.ac.uk/metagenomics/api/v1/analyses/MGYA00023829/file/SRR1631676_MERGED_FASTQ_otu.tsv	Root-Host_associated-Human-Skin
SRR5869453	MGYA00426634	https://www.ebi.ac.uk/metagenomics/api/v1/analyses/MGYA00426634/file/SRR5869453_MERGED_FASTQ_SSU_OTU.tsv	Root-Host_associated-Human-Skin
SRR1624808	MGYA00024676	https://www.ebi.ac.uk/metagenomics/api/v1/analyses/MGYA00024676/file/SRR1624808_MERGED_FASTQ_otu.tsv	Root-Host_associated-Human-Skin
ERR451357	MGYA00419156	https://www.ebi.ac.uk/metagenomics/api/v1/analyses/MGYA00419156/file/ERR451357_FASTQ_SSU_OTU.tsv	Root-Host_associated-Human-Skin
SRR995648	MGYA00024626	https://www.ebi.ac.uk/metagenomics/api/v1/analyses/MGYA00024626/file/SRR995648_FASTQ_otu.tsv	Root-Host_associated-Human-Skin
SRR920625	MGYA00022767	https://www.ebi.ac.uk/metagenomics/api/v1/analyses/MGYA00022767/file/SRR920625_FASTQ_otu.tsv	Root-Host_associated-Human-Skin
ERR443374	MGYA00426040	https://www.ebi.ac.uk/metagenomics/api/v1/analyses/MGYA00426040/file/ERR443374_FASTQ_SSU_OTU.tsv	Root-Host_associated-Human-Skin
SRR5870301	MGYA00418683	https://www.ebi.ac.uk/metagenomics/api/v1/analyses/MGYA00418683/file/SRR5870301_MERGED_FASTQ_SSU_OTU.tsv	Root-Host_associated-Human-Skin
SRR996264	MGYA00023509	https://www.ebi.ac.uk/metagenomics/api/v1/analyses/MGYA00023509/file/SRR996264_FASTQ_otu.tsv	Root-Host_associated-Human-Skin
SRR995710	MGYA00023134	https://www.ebi.ac.uk/metagenomics/api/v1/analyses/MGYA00023134/file/SRR995710_FASTQ_otu.tsv	Root-Host_associated-Human-Skin
ERR1701248	MGYA00084298	https://www.ebi.ac.uk/metagenomics/api/v1/analyses/MGYA00084298/file/ERR1701248_FASTQ_otu.tsv	Root-Host_associated-Human-Skin
SRR1631089	MGYA00023782	https://www.ebi.ac.uk/metagenomics/api/v1/analyses/MGYA00023782/file/SRR1631089_MERGED_FASTQ_otu.tsv	Root-Host_associated-Human-Skin

SRR996605	MGYA00024576	https://www.ebi.ac.uk/metagenomics/api/v1/analyses/MGYA00024576/file/SRR996605_FASTQ_otu.tsv	Root-Host_associated-Human-Skin
SRR997094	MGYA00022511	https://www.ebi.ac.uk/metagenomics/api/v1/analyses/MGYA00022511/file/SRR997094_FASTQ_otu.tsv	Root-Host_associated-Human-Skin
ERR1701299	MGYA00084330	https://www.ebi.ac.uk/metagenomics/api/v1/analyses/MGYA00084330/file/ERR1701299_FASTQ_otu.tsv	Root-Host_associated-Human-Skin
ERR1760076	MGYA00140519	https://www.ebi.ac.uk/metagenomics/api/v1/analyses/MGYA00140519/file/ERR1760076_MERGED_FASTQ_SSU_OTU.tsv	Root-Host_associated-Human-Skin
SRR995066	MGYA00024062	https://www.ebi.ac.uk/metagenomics/api/v1/analyses/MGYA00024062/file/SRR995066_FASTQ_otu.tsv	Root-Host_associated-Human-Skin
SRR1631439	MGYA00025315	https://www.ebi.ac.uk/metagenomics/api/v1/analyses/MGYA00025315/file/SRR1631439_MERGED_FASTQ_otu.tsv	Root-Host_associated-Human-Skin
SRR996512	MGYA00023074	https://www.ebi.ac.uk/metagenomics/api/v1/analyses/MGYA00023074/file/SRR996512_FASTQ_otu.tsv	Root-Host_associated-Human-Skin
SRR2005639	MGYA00381299	https://www.ebi.ac.uk/metagenomics/api/v1/analyses/MGYA00381299/file/SRR2005639_MERGED_FASTQ_SSU_OTU.tsv	Root-Host_associated-Human-Skin
SRR994992	MGYA00023931	https://www.ebi.ac.uk/metagenomics/api/v1/analyses/MGYA00023931/file/SRR994992_FASTQ_otu.tsv	Root-Host_associated-Human-Skin
ERR451426	MGYA00428710	https://www.ebi.ac.uk/metagenomics/api/v1/analyses/MGYA00428710/file/ERR451426_FASTQ_SSU_OTU.tsv	Root-Host_associated-Human-Skin
ERR1760129	MGYA00140449	https://www.ebi.ac.uk/metagenomics/api/v1/analyses/MGYA00140449/file/ERR1760129_MERGED_FASTQ_SSU_OTU.tsv	Root-Host_associated-Human-Skin
ERR1960665	MGYA00130775	https://www.ebi.ac.uk/metagenomics/api/v1/analyses/MGYA00130775/file/ERR1960665_MERGED_FASTQ_otu.tsv	Root-Host_associated-Human-Skin

ERR443405	MGYA00421413	https://www.ebi.ac.uk/metagenomics/api/v1/analyses/MGYA00421413/file/ERR443405_FASTQ_SSU_OTU.tsv	Root-Host_associated-Human-Skin
SRR920154	MGYA00024511	https://www.ebi.ac.uk/metagenomics/api/v1/analyses/MGYA00024511/file/SRR920154_FASTQ_otu.tsv	Root-Host_associated-Human-Skin
SRR994779	MGYA00023031	https://www.ebi.ac.uk/metagenomics/api/v1/analyses/MGYA00023031/file/SRR994779_FASTQ_otu.tsv	Root-Host_associated-Human-Skin
SRR5869322	MGYA00417564	https://www.ebi.ac.uk/metagenomics/api/v1/analyses/MGYA00417564/file/SRR5869322_MERGED_FASTQ_SSU_OTU.tsv	Root-Host_associated-Human-Skin
SRR5869485	MGYA00414860	https://www.ebi.ac.uk/metagenomics/api/v1/analyses/MGYA00414860/file/SRR5869485_MERGED_FASTQ_SSU_OTU.tsv	Root-Host_associated-Human-Skin
SRR1622932	MGYA00025288	https://www.ebi.ac.uk/metagenomics/api/v1/analyses/MGYA00025288/file/SRR1622932_MERGED_FASTQ_otu.tsv	Root-Host_associated-Human-Skin
SRR1632473	MGYA00023589	https://www.ebi.ac.uk/metagenomics/api/v1/analyses/MGYA00023589/file/SRR1632473_MERGED_FASTQ_otu.tsv	Root-Host_associated-Human-Skin
SRR1923924	MGYA00028245	https://www.ebi.ac.uk/metagenomics/api/v1/analyses/MGYA00028245/file/SRR1923924_FASTQ_otu.tsv	Root-Host_associated-Human-Skin
ERR1759937	MGYA00140418	https://www.ebi.ac.uk/metagenomics/api/v1/analyses/MGYA00140418/file/ERR1759937_MERGED_FASTQ_SSU_OTU.tsv	Root-Host_associated-Human-Skin
SRR919538	MGYA00024308	https://www.ebi.ac.uk/metagenomics/api/v1/analyses/MGYA00024308/file/SRR919538_FASTQ_otu.tsv	Root-Host_associated-Human-Skin
SRR5869035	MGYA00426249	https://www.ebi.ac.uk/metagenomics/api/v1/analyses/MGYA00426249/file/SRR5869035_MERGED_FASTQ_SSU_OTU.tsv	Root-Host_associated-Human-Skin
SRR1950761	MGYA00376928	https://www.ebi.ac.uk/metagenomics/api/v1/analyses/MGYA00376928/file/SRR1950761_MERGED_FASTQ_SSU_OTU.tsv	Root-Host_associated-Human-Skin

SRR995589	MGYA00022326	https://www.ebi.ac.uk/metagenomics/api/v1/analyses/MGYA00022326/file/SRR995589_FASTQ_otu.tsv	Root-Host_associated-Human-Skin
SRR920243	MGYA00023497	https://www.ebi.ac.uk/metagenomics/api/v1/analyses/MGYA00023497/file/SRR920243_FASTQ_otu.tsv	Root-Host_associated-Human-Skin
SRR920996	MGYA00023256	https://www.ebi.ac.uk/metagenomics/api/v1/analyses/MGYA00023256/file/SRR920996_FASTQ_otu.tsv	Root-Host_associated-Human-Skin
SRR1632305	MGYA00023277	https://www.ebi.ac.uk/metagenomics/api/v1/analyses/MGYA00023277/file/SRR1632305_MERGED_FASTQ_otu.tsv	Root-Host_associated-Human-Skin
SRR5869826	MGYA00427592	https://www.ebi.ac.uk/metagenomics/api/v1/analyses/MGYA00427592/file/SRR5869826_MERGED_FASTQ_SSU_OTU.tsv	Root-Host_associated-Human-Skin
SRR1631671	MGYA00023834	https://www.ebi.ac.uk/metagenomics/api/v1/analyses/MGYA00023834/file/SRR1631671_MERGED_FASTQ_otu.tsv	Root-Host_associated-Human-Skin
SRR920622	MGYA00022762	https://www.ebi.ac.uk/metagenomics/api/v1/analyses/MGYA00022762/file/SRR920622_FASTQ_otu.tsv	Root-Host_associated-Human-Skin
ERR451350	MGYA00426514	https://www.ebi.ac.uk/metagenomics/api/v1/analyses/MGYA00426514/file/ERR451350_FASTQ_SSU_OTU.tsv	Root-Host_associated-Human-Skin
SRR1632988	MGYA00025160	https://www.ebi.ac.uk/metagenomics/api/v1/analyses/MGYA00025160/file/SRR1632988_MERGED_FASTQ_otu.tsv	Root-Host_associated-Human-Skin
SRR1631348	MGYA00022813	https://www.ebi.ac.uk/metagenomics/api/v1/analyses/MGYA00022813/file/SRR1631348_MERGED_FASTQ_otu.tsv	Root-Host_associated-Human-Skin
SRR996263	MGYA00023504	https://www.ebi.ac.uk/metagenomics/api/v1/analyses/MGYA00023504/file/SRR996263_FASTQ_otu.tsv	Root-Host_associated-Human-Skin
SRR995717	MGYA00023357	https://www.ebi.ac.uk/metagenomics/api/v1/analyses/MGYA00023357/file/SRR995717_FASTQ_otu.tsv	Root-Host_associated-Human-Skin

SRR996602	MGYA00024573	https://www.ebi.ac.uk/metagenomics/api/v1/analyses/MGYA00024573/file/SRR996602_FASTQ_otu.tsv	Root-Host_associated-Human-Skin
ERR2538468	MGYA00381475	https://www.ebi.ac.uk/metagenomics/api/v1/analyses/MGYA00381475/file/ERR2538468_FASTQ_SSU_OTU.tsv	Root-Host_associated-Human-Skin
SRR6757698	MGYA00380811	https://www.ebi.ac.uk/metagenomics/api/v1/analyses/MGYA00380811/file/SRR6757698_MERGED_FASTQ_SSU_OTU.tsv	Root-Host_associated-Human-Skin
SRR997093	MGYA00022514	https://www.ebi.ac.uk/metagenomics/api/v1/analyses/MGYA00022514/file/SRR997093_FASTQ_otu.tsv	Root-Host_associated-Human-Skin
SRR6757649	MGYA00380764	https://www.ebi.ac.uk/metagenomics/api/v1/analyses/MGYA00380764/file/SRR6757649_MERGED_FASTQ_SSU_OTU.tsv	Root-Host_associated-Human-Skin
ERR1760071	MGYA00140583	https://www.ebi.ac.uk/metagenomics/api/v1/analyses/MGYA00140583/file/ERR1760071_MERGED_FASTQ_SSU_OTU.tsv	Root-Host_associated-Human-Skin
SRR995061	MGYA00024055	https://www.ebi.ac.uk/metagenomics/api/v1/analyses/MGYA00024055/file/SRR995061_FASTQ_otu.tsv	Root-Host_associated-Human-Skin
SRR994995	MGYA00023930	https://www.ebi.ac.uk/metagenomics/api/v1/analyses/MGYA00023930/file/SRR994995_FASTQ_otu.tsv	Root-Host_associated-Human-Skin
SRR1704618	MGYA00426149	https://www.ebi.ac.uk/metagenomics/api/v1/analyses/MGYA00426149/file/SRR1704618_FASTQ_SSU_OTU.tsv	Root-Host_associated-Human-Skin
SRR995145	MGYA00024137	https://www.ebi.ac.uk/metagenomics/api/v1/analyses/MGYA00024137/file/SRR995145_FASTQ_otu.tsv	Root-Host_associated-Human-Skin
SRR996431	MGYA00024478	https://www.ebi.ac.uk/metagenomics/api/v1/analyses/MGYA00024478/file/SRR996431_FASTQ_otu.tsv	Root-Host_associated-Human-Skin
SRR996396	MGYA00024972	https://www.ebi.ac.uk/metagenomics/api/v1/analyses/MGYA00024972/file/SRR996396_FASTQ_otu.tsv	Root-Host_associated-Human-Skin

SRR919595	MGYA00024952	https://www.ebi.ac.uk/metagenomics/api/v1/analyses/MGYA00024952/file/SRR919595_FASTQ_otu.tsv	Root-Host_associated-Human-Skin
SRR995524	MGYA00023418	https://www.ebi.ac.uk/metagenomics/api/v1/analyses/MGYA00023418/file/SRR995524_FASTQ_otu.tsv	Root-Host_associated-Human-Skin
SRR1923958	MGYA00028364	https://www.ebi.ac.uk/metagenomics/api/v1/analyses/MGYA00028364/file/SRR1923958_FASTQ_otu.tsv	Root-Host_associated-Human-Skin
ERR442026	MGYA00385207	https://www.ebi.ac.uk/metagenomics/api/v1/analyses/MGYA00385207/file/ERR442026_FASTQ_SSU_OTU.tsv	Root-Host_associated-Human-Skin
SRR995987	MGYA00022938	https://www.ebi.ac.uk/metagenomics/api/v1/analyses/MGYA00022938/file/SRR995987_FASTQ_otu.tsv	Root-Host_associated-Human-Skin
SRR1923989	MGYA00028283	https://www.ebi.ac.uk/metagenomics/api/v1/analyses/MGYA00028283/file/SRR1923989_FASTQ_otu.tsv	Root-Host_associated-Human-Skin
SRR994860	MGYA00022390	https://www.ebi.ac.uk/metagenomics/api/v1/analyses/MGYA00022390/file/SRR994860_FASTQ_otu.tsv	Root-Host_associated-Human-Skin
SRR5868609	MGYA00417182	https://www.ebi.ac.uk/metagenomics/api/v1/analyses/MGYA00417182/file/SRR5868609_MERGED_FASTQ_SSU_OTU.tsv	Root-Host_associated-Human-Skin
SRR996892	MGYA00022801	https://www.ebi.ac.uk/metagenomics/api/v1/analyses/MGYA00022801/file/SRR996892_FASTQ_otu.tsv	Root-Host_associated-Human-Skin
SRR996347	MGYA00022282	https://www.ebi.ac.uk/metagenomics/api/v1/analyses/MGYA00022282/file/SRR996347_FASTQ_otu.tsv	Root-Host_associated-Human-Skin
SRR5869428	MGYA00419684	https://www.ebi.ac.uk/metagenomics/api/v1/analyses/MGYA00419684/file/SRR5869428_MERGED_FASTQ_SSU_OTU.tsv	Root-Host_associated-Human-Skin
SRR996081	MGYA00024367	https://www.ebi.ac.uk/metagenomics/api/v1/analyses/MGYA00024367/file/SRR996081_FASTQ_otu.tsv	Root-Host_associated-Human-Skin

SRR996726	MGYA00023427	https://www.ebi.ac.uk/metagenomics/api/v1/analyses/MGYA00023427/file/SRR996726_FASTQ_otu.tsv	Root-Host_associated-Human-Skin
SRR1704457	MGYA00422058	https://www.ebi.ac.uk/metagenomics/api/v1/analyses/MGYA00422058/file/SRR1704457_FASTQ_SSU_OTU.tsv	Root-Host_associated-Human-Skin
ERR1960824	MGYA00130778	https://www.ebi.ac.uk/metagenomics/api/v1/analyses/MGYA00130778/file/ERR1960824_MERGED_FASTQ_otu.tsv	Root-Host_associated-Human-Skin
SRR1922544	MGYA00028407	https://www.ebi.ac.uk/metagenomics/api/v1/analyses/MGYA00028407/file/SRR1922544_FASTQ_otu.tsv	Root-Host_associated-Human-Skin
ERR443191	MGYA00420142	https://www.ebi.ac.uk/metagenomics/api/v1/analyses/MGYA00420142/file/ERR443191_FASTQ_SSU_OTU.tsv	Root-Host_associated-Human-Skin
SRR994938	MGYA00024968	https://www.ebi.ac.uk/metagenomics/api/v1/analyses/MGYA00024968/file/SRR994938_FASTQ_otu.tsv	Root-Host_associated-Human-Skin
SRR5869902	MGYA00416946	https://www.ebi.ac.uk/metagenomics/api/v1/analyses/MGYA00416946/file/SRR5869902_MERGED_FASTQ_SSU_OTU.tsv	Root-Host_associated-Human-Skin
ERR2538414	MGYA00381421	https://www.ebi.ac.uk/metagenomics/api/v1/analyses/MGYA00381421/file/ERR2538414_FASTQ_SSU_OTU.tsv	Root-Host_associated-Human-Skin
ERR1701233	MGYA00084266	https://www.ebi.ac.uk/metagenomics/api/v1/analyses/MGYA00084266/file/ERR1701233_FASTQ_otu.tsv	Root-Host_associated-Human-Skin
ERR1960790	MGYA00130825	https://www.ebi.ac.uk/metagenomics/api/v1/analyses/MGYA00130825/file/ERR1960790_MERGED_FASTQ_otu.tsv	Root-Host_associated-Human-Skin
SRR921146	MGYA00024592	https://www.ebi.ac.uk/metagenomics/api/v1/analyses/MGYA00024592/file/SRR921146_FASTQ_otu.tsv	Root-Host_associated-Human-Skin
ERR450897	MGYA00414558	https://www.ebi.ac.uk/metagenomics/api/v1/analyses/MGYA00414558/file/ERR450897_FASTQ_SSU_OTU.tsv	Root-Host_associated-Human-Skin

ERR1960741	MGYA00130823	https://www.ebi.ac.uk/metagenomics/api/v1/analyses/MGYA00130823/file/ERR1960741_MERGED_FASTQ_otu.tsv	Root-Host_associated-Human-Skin
SRR1704740	MGYA00426571	https://www.ebi.ac.uk/metagenomics/api/v1/analyses/MGYA00426571/file/SRR1704740_FASTQ_SSU_OTU.tsv	Root-Host_associated-Human-Skin
SRR6757635	MGYA00380751	https://www.ebi.ac.uk/metagenomics/api/v1/analyses/MGYA00380751/file/SRR6757635_MERGED_FASTQ_SSU_OTU.tsv	Root-Host_associated-Human-Skin
SRR1622816	MGYA00023984	https://www.ebi.ac.uk/metagenomics/api/v1/analyses/MGYA00023984/file/SRR1622816_MERGED_FASTQ_otu.tsv	Root-Host_associated-Human-Skin
ERR2538362	MGYA00381369	https://www.ebi.ac.uk/metagenomics/api/v1/analyses/MGYA00381369/file/ERR2538362_FASTQ_SSU_OTU.tsv	Root-Host_associated-Human-Skin
SRR1950694	MGYA00376861	https://www.ebi.ac.uk/metagenomics/api/v1/analyses/MGYA00376861/file/SRR1950694_MERGED_FASTQ_SSU_OTU.tsv	Root-Host_associated-Human-Skin
SRR996569	MGYA00025294	https://www.ebi.ac.uk/metagenomics/api/v1/analyses/MGYA00025294/file/SRR996569_FASTQ_otu.tsv	Root-Host_associated-Human-Skin
SRR2005642	MGYA00381301	https://www.ebi.ac.uk/metagenomics/api/v1/analyses/MGYA00381301/file/SRR2005642_MERGED_FASTQ_SSU_OTU.tsv	Root-Host_associated-Human-Skin
SRR5868780	MGYA00417106	https://www.ebi.ac.uk/metagenomics/api/v1/analyses/MGYA00417106/file/SRR5868780_MERGED_FASTQ_SSU_OTU.tsv	Root-Host_associated-Human-Skin
SRR1704321	MGYA00413830	https://www.ebi.ac.uk/metagenomics/api/v1/analyses/MGYA00413830/file/SRR1704321_FASTQ_SSU_OTU.tsv	Root-Host_associated-Human-Skin
SRR996785	MGYA00024078	https://www.ebi.ac.uk/metagenomics/api/v1/analyses/MGYA00024078/file/SRR996785_FASTQ_otu.tsv	Root-Host_associated-Human-Skin
SRR996022	MGYA00023727	https://www.ebi.ac.uk/metagenomics/api/v1/analyses/MGYA00023727/file/SRR996022_FASTQ_otu.tsv	Root-Host_associated-Human-Skin

ERR441983	MGYA00385194	https://www.ebi.ac.uk/metagenomics/api/v1/analyses/MGYA00385194/file/ERR441983_FASTQ_SSU_OTU.tsv	Root-Host_associated-Human-Skin
SRR5870147	MGYA00415834	https://www.ebi.ac.uk/metagenomics/api/v1/analyses/MGYA00415834/file/SRR5870147_MERGED_FASTQ_SSU_OTU.tsv	Root-Host_associated-Human-Skin
SRR995690	MGYA00023008	https://www.ebi.ac.uk/metagenomics/api/v1/analyses/MGYA00023008/file/SRR995690_FASTQ_otu.tsv	Root-Host_associated-Human-Skin
ERR1760127	MGYA00140426	https://www.ebi.ac.uk/metagenomics/api/v1/analyses/MGYA00140426/file/ERR1760127_MERGED_FASTQ_SSU_OTU.tsv	Root-Host_associated-Human-Skin
SRR996831	MGYA00023866	https://www.ebi.ac.uk/metagenomics/api/v1/analyses/MGYA00023866/file/SRR996831_FASTQ_otu.tsv	Root-Host_associated-Human-Skin
SRR995924	MGYA00024069	https://www.ebi.ac.uk/metagenomics/api/v1/analyses/MGYA00024069/file/SRR995924_FASTQ_otu.tsv	Root-Host_associated-Human-Skin
SRR920998	MGYA00023258	https://www.ebi.ac.uk/metagenomics/api/v1/analyses/MGYA00023258/file/SRR920998_FASTQ_otu.tsv	Root-Host_associated-Human-Skin
ERR442054	MGYA00385201	https://www.ebi.ac.uk/metagenomics/api/v1/analyses/MGYA00385201/file/ERR442054_FASTQ_SSU_OTU.tsv	Root-Host_associated-Human-Skin
SRR995587	MGYA00022318	https://www.ebi.ac.uk/metagenomics/api/v1/analyses/MGYA00022318/file/SRR995587_FASTQ_otu.tsv	Root-Host_associated-Human-Skin
ERR451098	MGYA00421425	https://www.ebi.ac.uk/metagenomics/api/v1/analyses/MGYA00421425/file/ERR451098_FASTQ_SSU_OTU.tsv	Root-Host_associated-Human-Skin
SRR919944	MGYA00024623	https://www.ebi.ac.uk/metagenomics/api/v1/analyses/MGYA00024623/file/SRR919944_FASTQ_otu.tsv	Root-Host_associated-Human-Skin
SRR919536	MGYA00024310	https://www.ebi.ac.uk/metagenomics/api/v1/analyses/MGYA00024310/file/SRR919536_FASTQ_otu.tsv	Root-Host_associated-Human-Skin

SRR996492	MGYA00023402	https://www.ebi.ac.uk/metagenomics/api/v1/analyses/MGYA00023402/file/SRR996492_FASTQ_otu.tsv	Root-Host_associated-Human-Skin
ERR1759939	MGYA00140468	https://www.ebi.ac.uk/metagenomics/api/v1/analyses/MGYA00140468/file/ERR1759939_MERGED_FASTQ_SSU_OTU.tsv	Root-Host_associated-Human-Skin
SRR995641	MGYA00024627	https://www.ebi.ac.uk/metagenomics/api/v1/analyses/MGYA00024627/file/SRR995641_FASTQ_otu.tsv	Root-Host_associated-Human-Skin
SRR1624801	MGYA00024675	https://www.ebi.ac.uk/metagenomics/api/v1/analyses/MGYA00024675/file/SRR1624801_MERGED_FASTQ_otu.tsv	Root-Host_associated-Human-Skin
SRR997114	MGYA00024565	https://www.ebi.ac.uk/metagenomics/api/v1/analyses/MGYA00024565/file/SRR997114_FASTQ_otu.tsv	Root-Host_associated-Human-Skin
SRR1630974	MGYA00024350	https://www.ebi.ac.uk/metagenomics/api/v1/analyses/MGYA00024350/file/SRR1630974_MERGED_FASTQ_otu.tsv	Root-Host_associated-Human-Skin
SRR1631346	MGYA00022809	https://www.ebi.ac.uk/metagenomics/api/v1/analyses/MGYA00022809/file/SRR1631346_MERGED_FASTQ_otu.tsv	Root-Host_associated-Human-Skin
SRR5868723	MGYA00421204	https://www.ebi.ac.uk/metagenomics/api/v1/analyses/MGYA00421204/file/SRR5868723_MERGED_FASTQ_SSU_OTU.tsv	Root-Host_associated-Human-Skin
SRR1632986	MGYA00025158	https://www.ebi.ac.uk/metagenomics/api/v1/analyses/MGYA00025158/file/SRR1632986_MERGED_FASTQ_otu.tsv	Root-Host_associated-Human-Skin
SRR1631080	MGYA00023787	https://www.ebi.ac.uk/metagenomics/api/v1/analyses/MGYA00023787/file/SRR1631080_MERGED_FASTQ_otu.tsv	Root-Host_associated-Human-Skin
SRR6757696	MGYA00380809	https://www.ebi.ac.uk/metagenomics/api/v1/analyses/MGYA00380809/file/SRR6757696_MERGED_FASTQ_SSU_OTU.tsv	Root-Host_associated-Human-Skin
ERR2538466	MGYA00381473	https://www.ebi.ac.uk/metagenomics/api/v1/analyses/MGYA00381473/file/ERR2538466_FASTQ_SSU_OTU.tsv	Root-Host_associated-Human-Skin

SRR921134	MGYA00022298	https://www.ebi.ac.uk/metagenomics/api/v1/analyses/MGYA00022298/file/SRR921134_FASTQ_otu.tsv	Root-Host_associated-Human-Skin
SRR995719	MGYA00023128	https://www.ebi.ac.uk/metagenomics/api/v1/analyses/MGYA00023128/file/SRR995719_FASTQ_otu.tsv	Root-Host_associated-Human-Skin
ERR450834	MGYA00416716	https://www.ebi.ac.uk/metagenomics/api/v1/analyses/MGYA00416716/file/ERR450834_FASTQ_SSU_OTU.tsv	Root-Host_associated-Human-Skin
ERR1701241	MGYA00084247	https://www.ebi.ac.uk/metagenomics/api/v1/analyses/MGYA00084247/file/ERR1701241_FASTQ_otu.tsv	Root-Host_associated-Human-Skin
ERR1960733	MGYA00130878	https://www.ebi.ac.uk/metagenomics/api/v1/analyses/MGYA00130878/file/ERR1960733_MERGED_FASTQ_otu.tsv	Root-Host_associated-Human-Skin
ERR1701290	MGYA00084264	https://www.ebi.ac.uk/metagenomics/api/v1/analyses/MGYA00084264/file/ERR1701290_FASTQ_otu.tsv	Root-Host_associated-Human-Skin
ERR451570	MGYA00426502	https://www.ebi.ac.uk/metagenomics/api/v1/analyses/MGYA00426502/file/ERR451570_FASTQ_SSU_OTU.tsv	Root-Host_associated-Human-Skin
SRR6757647	MGYA00380762	https://www.ebi.ac.uk/metagenomics/api/v1/analyses/MGYA00380762/file/SRR6757647_MERGED_FASTQ_SSU_OTU.tsv	Root-Host_associated-Human-Skin
SRR1704732	MGYA00421462	https://www.ebi.ac.uk/metagenomics/api/v1/analyses/MGYA00421462/file/SRR1704732_FASTQ_SSU_OTU.tsv	Root-Host_associated-Human-Skin
SRR1923872	MGYA00028254	https://www.ebi.ac.uk/metagenomics/api/v1/analyses/MGYA00028254/file/SRR1923872_FASTQ_otu.tsv	Root-Host_associated-Human-Skin
ERR443132	MGYA00412637	https://www.ebi.ac.uk/metagenomics/api/v1/analyses/MGYA00412637/file/ERR443132_FASTQ_SSU_OTU.tsv	Root-Host_associated-Human-Skin
SRR1704353	MGYA00413828	https://www.ebi.ac.uk/metagenomics/api/v1/analyses/MGYA00413828/file/SRR1704353_FASTQ_SSU_OTU.tsv	Root-Host_associated-Human-Skin

ERR443477	MGYA00419483	https://www.ebi.ac.uk/metagenomics/api/v1/analyses/MGYA00419483/file/ERR443477_FASTQ_SSU_OTU.tsv	Root-Host_associated-Human-Skin
SRR920935	MGYA00022574	https://www.ebi.ac.uk/metagenomics/api/v1/analyses/MGYA00022574/file/SRR920935_FASTQ_otu.tsv	Root-Host_associated-Human-Skin
SRR1632401	MGYA00023550	https://www.ebi.ac.uk/metagenomics/api/v1/analyses/MGYA00023550/file/SRR1632401_MERGED_FASTQ_otu.tsv	Root-Host_associated-Human-Skin
ERR1759994	MGYA00140536	https://www.ebi.ac.uk/metagenomics/api/v1/analyses/MGYA00140536/file/ERR1759994_MERGED_FASTQ_SSU_OTU.tsv	Root-Host_associated-Human-Skin
SRR1631514	MGYA00023385	https://www.ebi.ac.uk/metagenomics/api/v1/analyses/MGYA00023385/file/SRR1631514_MERGED_FASTQ_otu.tsv	Root-Host_associated-Human-Skin
SRR1704277	MGYA00426987	https://www.ebi.ac.uk/metagenomics/api/v1/analyses/MGYA00426987/file/SRR1704277_FASTQ_SSU_OTU.tsv	Root-Host_associated-Human-Skin
SRR2005714	MGYA00381343	https://www.ebi.ac.uk/metagenomics/api/v1/analyses/MGYA00381343/file/SRR2005714_MERGED_FASTQ_SSU_OTU.tsv	Root-Host_associated-Human-Skin
SRR996398	MGYA00024980	https://www.ebi.ac.uk/metagenomics/api/v1/analyses/MGYA00024980/file/SRR996398_FASTQ_otu.tsv	Root-Host_associated-Human-Skin
SRR1950713	MGYA00376880	https://www.ebi.ac.uk/metagenomics/api/v1/analyses/MGYA00376880/file/SRR1950713_MERGED_FASTQ_SSU_OTU.tsv	Root-Host_associated-Human-Skin
SRR996349	MGYA00022284	https://www.ebi.ac.uk/metagenomics/api/v1/analyses/MGYA00022284/file/SRR996349_FASTQ_otu.tsv	Root-Host_associated-Human-Skin
ERR1759945	MGYA00140397	https://www.ebi.ac.uk/metagenomics/api/v1/analyses/MGYA00140397/file/ERR1759945_MERGED_FASTQ_SSU_OTU.tsv	Root-Host_associated-Human-Skin
SRR1623065	MGYA00024411	https://www.ebi.ac.uk/metagenomics/api/v1/analyses/MGYA00024411/file/SRR1623065_MERGED_FASTQ_otu.tsv	Root-Host_associated-Human-Skin

ERR442028	MGYA00385206	https://www.ebi.ac.uk/metagenomics/api/v1/analyses/MGYA00385206/file/ERR442028_FASTQ_SSU_OTU.tsv	Root-Host_associated-Human-Skin
SRR995989	MGYA00022932	https://www.ebi.ac.uk/metagenomics/api/v1/analyses/MGYA00022932/file/SRR995989_FASTQ_otu.tsv	Root-Host_associated-Human-Skin
SRR996728	MGYA00023425	https://www.ebi.ac.uk/metagenomics/api/v1/analyses/MGYA00023425/file/SRR996728_FASTQ_otu.tsv	Root-Host_associated-Human-Skin
SRR921010	MGYA00023327	https://www.ebi.ac.uk/metagenomics/api/v1/analyses/MGYA00023327/file/SRR921010_FASTQ_otu.tsv	Root-Host_associated-Human-Skin
SRR920650	MGYA00023674	https://www.ebi.ac.uk/metagenomics/api/v1/analyses/MGYA00023674/file/SRR920650_FASTQ_otu.tsv	Root-Host_associated-Human-Skin
SRR994936	MGYA00024960	https://www.ebi.ac.uk/metagenomics/api/v1/analyses/MGYA00024960/file/SRR994936_FASTQ_otu.tsv	Root-Host_associated-Human-Skin
SRR994544	MGYA00024259	https://www.ebi.ac.uk/metagenomics/api/v1/analyses/MGYA00024259/file/SRR994544_FASTQ_otu.tsv	Root-Host_associated-Human-Skin
SRR995304	MGYA00022699	https://www.ebi.ac.uk/metagenomics/api/v1/analyses/MGYA00022699/file/SRR995304_FASTQ_otu.tsv	Root-Host_associated-Human-Skin
SRR919860	MGYA00023901	https://www.ebi.ac.uk/metagenomics/api/v1/analyses/MGYA00023901/file/SRR919860_FASTQ_otu.tsv	Root-Host_associated-Human-Skin
SRR921148	MGYA00024594	https://www.ebi.ac.uk/metagenomics/api/v1/analyses/MGYA00024594/file/SRR921148_FASTQ_otu.tsv	Root-Host_associated-Human-Skin
SRR920708	MGYA00024021	https://www.ebi.ac.uk/metagenomics/api/v1/analyses/MGYA00024021/file/SRR920708_FASTQ_otu.tsv	Root-Host_associated-Human-Skin
SRR997030	MGYA00023604	https://www.ebi.ac.uk/metagenomics/api/v1/analyses/MGYA00023604/file/SRR997030_FASTQ_otu.tsv	Root-Host_associated-Human-Skin

SRR1622818	MGYA00023988	https://www.ebi.ac.uk/metagenomics/api/v1/analyses/MGYA00023988/file/SRR1622818_MERGED_FASTQ_otu.tsv	Root-Host_associated-Human-Skin
SRR1924016	MGYA00028353	https://www.ebi.ac.uk/metagenomics/api/v1/analyses/MGYA00028353/file/SRR1924016_FASTQ_otu.tsv	Root-Host_associated-Human-Skin
SRR994653	MGYA00023450	https://www.ebi.ac.uk/metagenomics/api/v1/analyses/MGYA00023450/file/SRR994653_FASTQ_otu.tsv	Root-Host_associated-Human-Skin
ERR1760003	MGYA00140384	https://www.ebi.ac.uk/metagenomics/api/v1/analyses/MGYA00140384/file/ERR1760003_MERGED_FASTQ_SSU_OTU.tsv	Root-Host_associated-Human-Skin
SRR996567	MGYA00025302	https://www.ebi.ac.uk/metagenomics/api/v1/analyses/MGYA00025302/file/SRR996567_FASTQ_otu.tsv	Root-Host_associated-Human-Skin
SRR996915	MGYA00022442	https://www.ebi.ac.uk/metagenomics/api/v1/analyses/MGYA00022442/file/SRR996915_FASTQ_otu.tsv	Root-Host_associated-Human-Skin
SRR995472	MGYA00025102	https://www.ebi.ac.uk/metagenomics/api/v1/analyses/MGYA00025102/file/SRR995472_FASTQ_otu.tsv	Root-Host_associated-Human-Skin
ERR451453	MGYA00422025	https://www.ebi.ac.uk/metagenomics/api/v1/analyses/MGYA00422025/file/ERR451453_FASTQ_SSU_OTU.tsv	Root-Host_associated-Human-Skin
SRR1704611	MGYA00421469	https://www.ebi.ac.uk/metagenomics/api/v1/analyses/MGYA00421469/file/SRR1704611_FASTQ_SSU_OTU.tsv	Root-Host_associated-Human-Skin
SRR1632406	MGYA00023549	https://www.ebi.ac.uk/metagenomics/api/v1/analyses/MGYA00023549/file/SRR1632406_MERGED_FASTQ_otu.tsv	Root-Host_associated-Human-Skin
SRR920932	MGYA00022577	https://www.ebi.ac.uk/metagenomics/api/v1/analyses/MGYA00022577/file/SRR920932_FASTQ_otu.tsv	Root-Host_associated-Human-Skin
SRR1923951	MGYA00028388	https://www.ebi.ac.uk/metagenomics/api/v1/analyses/MGYA00028388/file/SRR1923951_FASTQ_otu.tsv	Root-Host_associated-Human-Skin

SRR2005713	MGYA00381342	https://www.ebi.ac.uk/metagenomics/api/v1/analyses/MGYA00381342/file/SRR2005713_MERGED_FASTQ_SSU_OTU.tsv	Root-Host_associated-Human-Skin
SRR996438	MGYA00024481	https://www.ebi.ac.uk/metagenomics/api/v1/analyses/MGYA00024481/file/SRR996438_FASTQ_otu.tsv	Root-Host_associated-Human-Skin
SRR5869091	MGYA00418117	https://www.ebi.ac.uk/metagenomics/api/v1/analyses/MGYA00418117/file/SRR5869091_MERGED_FASTQ_SSU_OTU.tsv	Root-Host_associated-Human-Skin
SRR1704270	MGYA00426988	https://www.ebi.ac.uk/metagenomics/api/v1/analyses/MGYA00426988/file/SRR1704270_FASTQ_SSU_OTU.tsv	Root-Host_associated-Human-Skin
ERR1759993	MGYA00140406	https://www.ebi.ac.uk/metagenomics/api/v1/analyses/MGYA00140406/file/ERR1759993_MERGED_FASTQ_SSU_OTU.tsv	Root-Host_associated-Human-Skin
SRR1631513	MGYA00023380	https://www.ebi.ac.uk/metagenomics/api/v1/analyses/MGYA00023380/file/SRR1631513_MERGED_FASTQ_otu.tsv	Root-Host_associated-Human-Skin
ERR1759942	MGYA00140609	https://www.ebi.ac.uk/metagenomics/api/v1/analyses/MGYA00140609/file/ERR1759942_MERGED_FASTQ_SSU_OTU.tsv	Root-Host_associated-Human-Skin
SRR1623062	MGYA00024418	https://www.ebi.ac.uk/metagenomics/api/v1/analyses/MGYA00024418/file/SRR1623062_MERGED_FASTQ_otu.tsv	Root-Host_associated-Human-Skin
SRR5869040	MGYA00418133	https://www.ebi.ac.uk/metagenomics/api/v1/analyses/MGYA00418133/file/SRR5869040_MERGED_FASTQ_SSU_OTU.tsv	Root-Host_associated-Human-Skin
SRR1950714	MGYA00376881	https://www.ebi.ac.uk/metagenomics/api/v1/analyses/MGYA00376881/file/SRR1950714_MERGED_FASTQ_SSU_OTU.tsv	Root-Host_associated-Human-Skin
ERR443767	MGYA00425243	https://www.ebi.ac.uk/metagenomics/api/v1/analyses/MGYA00425243/file/ERR443767_FASTQ_SSU_OTU.tsv	Root-Host_associated-Human-Skin
SRR994869	MGYA00022397	https://www.ebi.ac.uk/metagenomics/api/v1/analyses/MGYA00022397/file/SRR994869_FASTQ_otu.tsv	Root-Host_associated-Human-Skin

SRR996088	MGYA00024358	https://www.ebi.ac.uk/metagenomics/api/v1/analyses/MGYA00024358/file/SRR996088_FASTQ_otu.tsv	Root-Host_associated-Human-Skin
ERR443306	MGYA00428756	https://www.ebi.ac.uk/metagenomics/api/v1/analyses/MGYA00428756/file/ERR443306_FASTQ_SSU_OTU.tsv	Root-Host_associated-Human-Skin
SRR920657	MGYA00025216	https://www.ebi.ac.uk/metagenomics/api/v1/analyses/MGYA00025216/file/SRR920657_FASTQ_otu.tsv	Root-Host_associated-Human-Skin
SRR921017	MGYA00023328	https://www.ebi.ac.uk/metagenomics/api/v1/analyses/MGYA00023328/file/SRR921017_FASTQ_otu.tsv	Root-Host_associated-Human-Skin
ERR451482	MGYA00422358	https://www.ebi.ac.uk/metagenomics/api/v1/analyses/MGYA00422358/file/ERR451482_FASTQ_SSU_OTU.tsv	Root-Host_associated-Human-Skin
SRR995303	MGYA00022698	https://www.ebi.ac.uk/metagenomics/api/v1/analyses/MGYA00022698/file/SRR995303_FASTQ_otu.tsv	Root-Host_associated-Human-Skin
SRR994543	MGYA00024264	https://www.ebi.ac.uk/metagenomics/api/v1/analyses/MGYA00024264/file/SRR994543_FASTQ_otu.tsv	Root-Host_associated-Human-Skin
SRR5868758	MGYA00417114	https://www.ebi.ac.uk/metagenomics/api/v1/analyses/MGYA00417114/file/SRR5868758_MERGED_FASTQ_SSU_OTU.tsv	Root-Host_associated-Human-Skin
SRR919867	MGYA00023898	https://www.ebi.ac.uk/metagenomics/api/v1/analyses/MGYA00023898/file/SRR919867_FASTQ_otu.tsv	Root-Host_associated-Human-Skin
ERR1960799	MGYA00130896	https://www.ebi.ac.uk/metagenomics/api/v1/analyses/MGYA00130896/file/ERR1960799_MERGED_FASTQ_otu.tsv	Root-Host_associated-Human-Skin
SRR997037	MGYA00023603	https://www.ebi.ac.uk/metagenomics/api/v1/analyses/MGYA00023603/file/SRR997037_FASTQ_otu.tsv	Root-Host_associated-Human-Skin
SRR1704749	MGYA00413802	https://www.ebi.ac.uk/metagenomics/api/v1/analyses/MGYA00413802/file/SRR1704749_FASTQ_SSU_OTU.tsv	Root-Host_associated-Human-Skin

ERR1760004	MGYA00140564	https://www.ebi.ac.uk/metagenomics/api/v1/analyses/MGYA00140564/file/ERR1760004_MERGED_FASTQ_SSU_OTU.tsv	Root-Host_associated-Human-Skin
ERR1960748	MGYA00130763	https://www.ebi.ac.uk/metagenomics/api/v1/analyses/MGYA00130763/file/ERR1960748_MERGED_FASTQ_otu.tsv	Root-Host_associated-Human-Skin
SRR994654	MGYA00023445	https://www.ebi.ac.uk/metagenomics/api/v1/analyses/MGYA00023445/file/SRR994654_FASTQ_otu.tsv	Root-Host_associated-Human-Skin
SRR996912	MGYA00022439	https://www.ebi.ac.uk/metagenomics/api/v1/analyses/MGYA00022439/file/SRR996912_FASTQ_otu.tsv	Root-Host_associated-Human-Skin
SRR996560	MGYA00025297	https://www.ebi.ac.uk/metagenomics/api/v1/analyses/MGYA00025297/file/SRR996560_FASTQ_otu.tsv	Root-Host_associated-Human-Skin
SRR1704328	MGYA00426967	https://www.ebi.ac.uk/metagenomics/api/v1/analyses/MGYA00426967/file/SRR1704328_FASTQ_SSU_OTU.tsv	Root-Host_associated-Human-Skin
SRR5868789	MGYA00427173	https://www.ebi.ac.uk/metagenomics/api/v1/analyses/MGYA00427173/file/SRR5868789_MERGED_FASTQ_SSU_OTU.tsv	Root-Host_associated-Human-Skin
ERR443149	MGYA00412636	https://www.ebi.ac.uk/metagenomics/api/v1/analyses/MGYA00412636/file/ERR443149_FASTQ_SSU_OTU.tsv	Root-Host_associated-Human-Skin
SRR995475	MGYA00025101	https://www.ebi.ac.uk/metagenomics/api/v1/analyses/MGYA00025101/file/SRR995475_FASTQ_otu.tsv	Root-Host_associated-Human-Skin
ERR441984	MGYA00385182	https://www.ebi.ac.uk/metagenomics/api/v1/analyses/MGYA00385182/file/ERR441984_FASTQ_SSU_OTU.tsv	Root-Host_associated-Human-Skin
SRR996025	MGYA00023722	https://www.ebi.ac.uk/metagenomics/api/v1/analyses/MGYA00023722/file/SRR996025_FASTQ_otu.tsv	Root-Host_associated-Human-Skin
SRR996782	MGYA00024077	https://www.ebi.ac.uk/metagenomics/api/v1/analyses/MGYA00024077/file/SRR996782_FASTQ_otu.tsv	Root-Host_associated-Human-Skin

ERR1760120	MGYA00140459	https://www.ebi.ac.uk/metagenomics/api/v1/analyses/MGYA00140459/file/ERR1760120_MERGED_FASTQ_SSU_OTU.tsv	Root-Host_associated-Human-Skin
ERR442999	MGYA00423314	https://www.ebi.ac.uk/metagenomics/api/v1/analyses/MGYA00423314/file/ERR442999_FASTQ_SSU_OTU.tsv	Root-Host_associated-Human-Skin
ERR451388	MGYA00414269	https://www.ebi.ac.uk/metagenomics/api/v1/analyses/MGYA00414269/file/ERR451388_FASTQ_SSU_OTU.tsv	Root-Host_associated-Human-Skin
SRR995697	MGYA00023003	https://www.ebi.ac.uk/metagenomics/api/v1/analyses/MGYA00023003/file/SRR995697_FASTQ_otu.tsv	Root-Host_associated-Human-Skin
SRR996836	MGYA00023869	https://www.ebi.ac.uk/metagenomics/api/v1/analyses/MGYA00023869/file/SRR996836_FASTQ_otu.tsv	Root-Host_associated-Human-Skin
SRR995923	MGYA00024068	https://www.ebi.ac.uk/metagenomics/api/v1/analyses/MGYA00024068/file/SRR995923_FASTQ_otu.tsv	Root-Host_associated-Human-Skin
ERR442053	MGYA00385237	https://www.ebi.ac.uk/metagenomics/api/v1/analyses/MGYA00385237/file/ERR442053_FASTQ_SSU_OTU.tsv	Root-Host_associated-Human-Skin
SRR5870257	MGYA00425415	https://www.ebi.ac.uk/metagenomics/api/v1/analyses/MGYA00425415/file/SRR5870257_MERGED_FASTQ_SSU_OTU.tsv	Root-Host_associated-Human-Skin
SRR1950768	MGYA00376935	https://www.ebi.ac.uk/metagenomics/api/v1/analyses/MGYA00376935/file/SRR1950768_MERGED_FASTQ_SSU_OTU.tsv	Root-Host_associated-Human-Skin
SRR996495	MGYA00023403	https://www.ebi.ac.uk/metagenomics/api/v1/analyses/MGYA00023403/file/SRR996495_FASTQ_otu.tsv	Root-Host_associated-Human-Skin
SRR919531	MGYA00024315	https://www.ebi.ac.uk/metagenomics/api/v1/analyses/MGYA00024315/file/SRR919531_FASTQ_otu.tsv	Root-Host_associated-Human-Skin
SRR919943	MGYA00024618	https://www.ebi.ac.uk/metagenomics/api/v1/analyses/MGYA00024618/file/SRR919943_FASTQ_otu.tsv	Root-Host_associated-Human-Skin

SRR1624806	MGYA00024668	https://www.ebi.ac.uk/metagenomics/api/v1/analyses/MGYA00024668/file/SRR1624806_MERGED_FASTQ_otu.tsv	Root-Host_associated-Human-Skin
SRR995646	MGYA00024634	https://www.ebi.ac.uk/metagenomics/api/v1/analyses/MGYA00024634/file/SRR995646_FASTQ_otu.tsv	Root-Host_associated-Human-Skin
ERR451359	MGYA00426016	https://www.ebi.ac.uk/metagenomics/api/v1/analyses/MGYA00426016/file/ERR451359_FASTQ_SSU_OTU.tsv	Root-Host_associated-Human-Skin
SRR1631678	MGYA00023455	https://www.ebi.ac.uk/metagenomics/api/v1/analyses/MGYA00023455/file/SRR1631678_MERGED_FASTQ_otu.tsv	Root-Host_associated-Human-Skin
SRR997113	MGYA00024558	https://www.ebi.ac.uk/metagenomics/api/v1/analyses/MGYA00024558/file/SRR997113_FASTQ_otu.tsv	Root-Host_associated-Human-Skin
SRR1631341	MGYA00022806	https://www.ebi.ac.uk/metagenomics/api/v1/analyses/MGYA00022806/file/SRR1631341_MERGED_FASTQ_otu.tsv	Root-Host_associated-Human-Skin
SRR1704422	MGYA00428856	https://www.ebi.ac.uk/metagenomics/api/v1/analyses/MGYA00428856/file/SRR1704422_FASTQ_SSU_OTU.tsv	Root-Host_associated-Human-Skin
SRR1630973	MGYA00024351	https://www.ebi.ac.uk/metagenomics/api/v1/analyses/MGYA00024351/file/SRR1630973_MERGED_FASTQ_otu.tsv	Root-Host_associated-Human-Skin
ERR443643	MGYA00415113	https://www.ebi.ac.uk/metagenomics/api/v1/analyses/MGYA00415113/file/ERR443643_FASTQ_SSU_OTU.tsv	Root-Host_associated-Human-Skin
SRR1632981	MGYA00025153	https://www.ebi.ac.uk/metagenomics/api/v1/analyses/MGYA00025153/file/SRR1632981_MERGED_FASTQ_otu.tsv	Root-Host_associated-Human-Skin
ERR2538461	MGYA00381468	https://www.ebi.ac.uk/metagenomics/api/v1/analyses/MGYA00381468/file/ERR2538461_FASTQ_SSU_OTU.tsv	Root-Host_associated-Human-Skin
SRR1631087	MGYA00023786	https://www.ebi.ac.uk/metagenomics/api/v1/analyses/MGYA00023786/file/SRR1631087_MERGED_FASTQ_otu.tsv	Root-Host_associated-Human-Skin

SRR6757691	MGYA00380804	https://www.ebi.ac.uk/metagenomics/api/v1/analyses/MGYA00380804/file/SRR6757691_MERGED_FASTQ_SSU_OTU.tsv	Root-Host_associated-Human-Skin
ERR443585	MGYA00413334	https://www.ebi.ac.uk/metagenomics/api/v1/analyses/MGYA00413334/file/ERR443585_FASTQ_SSU_OTU.tsv	Root-Host_associated-Human-Skin
ERR1701246	MGYA00084329	https://www.ebi.ac.uk/metagenomics/api/v1/analyses/MGYA00084329/file/ERR1701246_FASTQ_otu.tsv	Root-Host_associated-Human-Skin
SRR921133	MGYA00022305	https://www.ebi.ac.uk/metagenomics/api/v1/analyses/MGYA00022305/file/SRR921133_FASTQ_otu.tsv	Root-Host_associated-Human-Skin
SRR995068	MGYA00023500	https://www.ebi.ac.uk/metagenomics/api/v1/analyses/MGYA00023500/file/SRR995068_FASTQ_otu.tsv	Root-Host_associated-Human-Skin
ERR1760078	MGYA00140517	https://www.ebi.ac.uk/metagenomics/api/v1/analyses/MGYA00140517/file/ERR1760078_MERGED_FASTQ_SSU_OTU.tsv	Root-Host_associated-Human-Skin
SRR5870018	MGYA00421580	https://www.ebi.ac.uk/metagenomics/api/v1/analyses/MGYA00421580/file/SRR5870018_MERGED_FASTQ_SSU_OTU.tsv	Root-Host_associated-Human-Skin
ERR1701297	MGYA00084293	https://www.ebi.ac.uk/metagenomics/api/v1/analyses/MGYA00084293/file/ERR1701297_FASTQ_otu.tsv	Root-Host_associated-Human-Skin
ERR1960734	MGYA00130885	https://www.ebi.ac.uk/metagenomics/api/v1/analyses/MGYA00130885/file/ERR1960734_MERGED_FASTQ_otu.tsv	Root-Host_associated-Human-Skin
SRR6757640	MGYA00380755	https://www.ebi.ac.uk/metagenomics/api/v1/analyses/MGYA00380755/file/SRR6757640_MERGED_FASTQ_SSU_OTU.tsv	Root-Host_associated-Human-Skin
SRR1704735	MGYA00426572	https://www.ebi.ac.uk/metagenomics/api/v1/analyses/MGYA00426572/file/SRR1704735_FASTQ_SSU_OTU.tsv	Root-Host_associated-Human-Skin
ERR450756	MGYA00416736	https://www.ebi.ac.uk/metagenomics/api/v1/analyses/MGYA00416736/file/ERR450756_FASTQ_SSU_OTU.tsv	Root-Host_associated-Human-Skin

SRR1923875	MGYA00028350	https://www.ebi.ac.uk/metagenomics/api/v1/analyses/MGYA00028350/file/SRR1923875_FASTQ_otu.tsv	Root-Host_associated-Human-Skin
SRR2005637	MGYA00381297	https://www.ebi.ac.uk/metagenomics/api/v1/analyses/MGYA00381297/file/SRR2005637_MERGED_FASTQ_SSU_OTU.tsv	Root-Host_associated-Human-Skin
SRR5869612	MGYA00418042	https://www.ebi.ac.uk/metagenomics/api/v1/analyses/MGYA00418042/file/SRR5869612_MERGED_FASTQ_SSU_OTU.tsv	Root-Host_associated-Human-Skin
SRR1631437	MGYA00025319	https://www.ebi.ac.uk/metagenomics/api/v1/analyses/MGYA00025319/file/SRR1631437_MERGED_FASTQ_otu.tsv	Root-Host_associated-Human-Skin
ERR450922	MGYA00415184	https://www.ebi.ac.uk/metagenomics/api/v1/analyses/MGYA00415184/file/ERR450922_FASTQ_SSU_OTU.tsv	Root-Host_associated-Human-Skin
SRR1633164	MGYA00022381	https://www.ebi.ac.uk/metagenomics/api/v1/analyses/MGYA00022381/file/SRR1633164_MERGED_FASTQ_otu.tsv	Root-Host_associated-Human-Skin
ERR451310	MGYA00421415	https://www.ebi.ac.uk/metagenomics/api/v1/analyses/MGYA00421415/file/ERR451310_FASTQ_SSU_OTU.tsv	Root-Host_associated-Human-Skin
SRR5868635	MGYA00421656	https://www.ebi.ac.uk/metagenomics/api/v1/analyses/MGYA00421656/file/SRR5868635_MERGED_FASTQ_SSU_OTU.tsv	Root-Host_associated-Human-Skin
SRR1631250	MGYA00024541	https://www.ebi.ac.uk/metagenomics/api/v1/analyses/MGYA00024541/file/SRR1631250_MERGED_FASTQ_otu.tsv	Root-Host_associated-Human-Skin
ERR1759977	MGYA00140560	https://www.ebi.ac.uk/metagenomics/api/v1/analyses/MGYA00140560/file/ERR1759977_MERGED_FASTQ_SSU_OTU.tsv	Root-Host_associated-Human-Skin
SRR1950721	MGYA00376888	https://www.ebi.ac.uk/metagenomics/api/v1/analyses/MGYA00376888/file/SRR1950721_MERGED_FASTQ_SSU_OTU.tsv	Root-Host_associated-Human-Skin
SRR919578	MGYA00024719	https://www.ebi.ac.uk/metagenomics/api/v1/analyses/MGYA00024719/file/SRR919578_FASTQ_otu.tsv	Root-Host_associated-Human-Skin

ERR450696	MGYA00415585	https://www.ebi.ac.uk/metagenomics/api/v1/analyses/MGYA00415585/file/ERR450696_FASTQ_SSU_OTU.tsv	Root-Host_associated-Human-Skin
SRR1632345	MGYA00025165	https://www.ebi.ac.uk/metagenomics/api/v1/analyses/MGYA00025165/file/SRR1632345_MERGED_FASTQ_otu.tsv	Root-Host_associated-Human-Skin
SRR920203	MGYA00023058	https://www.ebi.ac.uk/metagenomics/api/v1/analyses/MGYA00023058/file/SRR920203_FASTQ_otu.tsv	Root-Host_associated-Human-Skin
ERR443024	MGYA00426791	https://www.ebi.ac.uk/metagenomics/api/v1/analyses/MGYA00426791/file/ERR443024_FASTQ_SSU_OTU.tsv	Root-Host_associated-Human-Skin
ERR443783	MGYA00414250	https://www.ebi.ac.uk/metagenomics/api/v1/analyses/MGYA00414250/file/ERR443783_FASTQ_SSU_OTU.tsv	Root-Host_associated-Human-Skin
SRR5869703	MGYA00418036	https://www.ebi.ac.uk/metagenomics/api/v1/analyses/MGYA00418036/file/SRR5869703_MERGED_FASTQ_SSU_OTU.tsv	Root-Host_associated-Human-Skin
SRR1631281	MGYA00025274	https://www.ebi.ac.uk/metagenomics/api/v1/analyses/MGYA00025274/file/SRR1631281_MERGED_FASTQ_otu.tsv	Root-Host_associated-Human-Skin
SRR1704245	MGYA00428455	https://www.ebi.ac.uk/metagenomics/api/v1/analyses/MGYA00428455/file/SRR1704245_FASTQ_SSU_OTU.tsv	Root-Host_associated-Human-Skin
SRR995179	MGYA00022314	https://www.ebi.ac.uk/metagenomics/api/v1/analyses/MGYA00022314/file/SRR995179_FASTQ_otu.tsv	Root-Host_associated-Human-Skin
SRR994739	MGYA00023466	https://www.ebi.ac.uk/metagenomics/api/v1/analyses/MGYA00023466/file/SRR994739_FASTQ_otu.tsv	Root-Host_associated-Human-Skin
SRR920114	MGYA00024129	https://www.ebi.ac.uk/metagenomics/api/v1/analyses/MGYA00024129/file/SRR920114_FASTQ_otu.tsv	Root-Host_associated-Human-Skin
SRR1704183	MGYA00419551	https://www.ebi.ac.uk/metagenomics/api/v1/analyses/MGYA00419551/file/SRR1704183_FASTQ_SSU_OTU.tsv	Root-Host_associated-Human-Skin

SRR996927	MGYA00025132	https://www.ebi.ac.uk/metagenomics/api/v1/analyses/MGYA00025132/file/SRR996927_FASTQ_otu.tsv	Root-Host_associated-Human-Skin
SRR996555	MGYA00022622	https://www.ebi.ac.uk/metagenomics/api/v1/analyses/MGYA00022622/file/SRR996555_FASTQ_otu.tsv	Root-Host_associated-Human-Skin
SRR919883	MGYA00024500	https://www.ebi.ac.uk/metagenomics/api/v1/analyses/MGYA00024500/file/SRR919883_FASTQ_otu.tsv	Root-Host_associated-Human-Skin
SRR1623218	MGYA00023915	https://www.ebi.ac.uk/metagenomics/api/v1/analyses/MGYA00023915/file/SRR1623218_MERGED_FASTQ_otu.tsv	Root-Host_associated-Human-Skin
SRR6757609	MGYA00380727	https://www.ebi.ac.uk/metagenomics/api/v1/analyses/MGYA00380727/file/SRR6757609_MERGED_FASTQ_SSU_OTU.tsv	Root-Host_associated-Human-Skin
SRR996134	MGYA00024995	https://www.ebi.ac.uk/metagenomics/api/v1/analyses/MGYA00024995/file/SRR996134_FASTQ_otu.tsv	Root-Host_associated-Human-Skin
SRR996693	MGYA00022290	https://www.ebi.ac.uk/metagenomics/api/v1/analyses/MGYA00022290/file/SRR996693_FASTQ_otu.tsv	Root-Host_associated-Human-Skin
ERR1760031	MGYA00140365	https://www.ebi.ac.uk/metagenomics/api/v1/analyses/MGYA00140365/file/ERR1760031_MERGED_FASTQ_SSU_OTU.tsv	Root-Host_associated-Human-Skin
SRR995021	MGYA00023949	https://www.ebi.ac.uk/metagenomics/api/v1/analyses/MGYA00023949/file/SRR995021_FASTQ_otu.tsv	Root-Host_associated-Human-Skin
ERR451299	MGYA00428741	https://www.ebi.ac.uk/metagenomics/api/v1/analyses/MGYA00428741/file/ERR451299_FASTQ_SSU_OTU.tsv	Root-Host_associated-Human-Skin
ERR2538428	MGYA00381435	https://www.ebi.ac.uk/metagenomics/api/v1/analyses/MGYA00381435/file/ERR2538428_FASTQ_SSU_OTU.tsv	Root-Host_associated-Human-Skin
SRR995491	MGYA00024112	https://www.ebi.ac.uk/metagenomics/api/v1/analyses/MGYA00024112/file/SRR995491_FASTQ_otu.tsv	Root-Host_associated-Human-Skin

ERR1960818	MGYA00130720	https://www.ebi.ac.uk/metagenomics/api/v1/analyses/MGYA00130720/file/ERR1960818_MERGED_FASTQ_otu.tsv	Root-Host_associated-Human-Skin
SRR1631308	MGYA00023226	https://www.ebi.ac.uk/metagenomics/api/v1/analyses/MGYA00023226/file/SRR1631308_MERGED_FASTQ_otu.tsv	Root-Host_associated-Human-Skin
SRR919852	MGYA00024740	https://www.ebi.ac.uk/metagenomics/api/v1/analyses/MGYA00024740/file/SRR919852_FASTQ_otu.tsv	Root-Host_associated-Human-Skin
SRR996223	MGYA00023976	https://www.ebi.ac.uk/metagenomics/api/v1/analyses/MGYA00023976/file/SRR996223_FASTQ_otu.tsv	Root-Host_associated-Human-Skin
ERR1960688	MGYA00130730	https://www.ebi.ac.uk/metagenomics/api/v1/analyses/MGYA00130730/file/ERR1960688_MERGED_FASTQ_otu.tsv	Root-Host_associated-Human-Skin
SRR994794	MGYA00022828	https://www.ebi.ac.uk/metagenomics/api/v1/analyses/MGYA00022828/file/SRR994794_FASTQ_otu.tsv	Root-Host_associated-Human-Skin
SRR996766	MGYA00023861	https://www.ebi.ac.uk/metagenomics/api/v1/analyses/MGYA00023861/file/SRR996766_FASTQ_otu.tsv	Root-Host_associated-Human-Skin
ERR441960	MGYA00385177	https://www.ebi.ac.uk/metagenomics/api/v1/analyses/MGYA00385177/file/ERR441960_FASTQ_SSU_OTU.tsv	Root-Host_associated-Human-Skin
SRR1704689	MGYA00426585	https://www.ebi.ac.uk/metagenomics/api/v1/analyses/MGYA00426585/file/SRR1704689_FASTQ_SSU_OTU.tsv	Root-Host_associated-Human-Skin
SRR1624787	MGYA00022546	https://www.ebi.ac.uk/metagenomics/api/v1/analyses/MGYA00022546/file/SRR1624787_MERGED_FASTQ_otu.tsv	Root-Host_associated-Human-Skin
ERR442066	MGYA00385196	https://www.ebi.ac.uk/metagenomics/api/v1/analyses/MGYA00385196/file/ERR442066_FASTQ_SSU_OTU.tsv	Root-Host_associated-Human-Skin
SRR994452	MGYA00022456	https://www.ebi.ac.uk/metagenomics/api/v1/analyses/MGYA00022456/file/SRR994452_FASTQ_otu.tsv	Root-Host_associated-Human-Skin

SRR5868649	MGYA00411943	https://www.ebi.ac.uk/metagenomics/api/v1/analyses/MGYA00411943/file/SRR5868649_MERGED_FASTQ_SSU_OTU.tsv	Root-Host_associated-Human-Skin
SRR996307	MGYA00022709	https://www.ebi.ac.uk/metagenomics/api/v1/analyses/MGYA00022709/file/SRR996307_FASTQ_otu.tsv	Root-Host_associated-Human-Skin
SRR994483	MGYA00023139	https://www.ebi.ac.uk/metagenomics/api/v1/analyses/MGYA00023139/file/SRR994483_FASTQ_otu.tsv	Root-Host_associated-Human-Skin
SRR1923918	MGYA00028302	https://www.ebi.ac.uk/metagenomics/api/v1/analyses/MGYA00028302/file/SRR1923918_FASTQ_otu.tsv	Root-Host_associated-Human-Skin
SRR995564	MGYA00022982	https://www.ebi.ac.uk/metagenomics/api/v1/analyses/MGYA00022982/file/SRR995564_FASTQ_otu.tsv	Root-Host_associated-Human-Skin
SRR919613	MGYA00023525	https://www.ebi.ac.uk/metagenomics/api/v1/analyses/MGYA00023525/file/SRR919613_FASTQ_otu.tsv	Root-Host_associated-Human-Skin
SRR995105	MGYA00023671	https://www.ebi.ac.uk/metagenomics/api/v1/analyses/MGYA00023671/file/SRR995105_FASTQ_otu.tsv	Root-Host_associated-Human-Skin
ERR1760115	MGYA00140574	https://www.ebi.ac.uk/metagenomics/api/v1/analyses/MGYA00140574/file/ERR1760115_MERGED_FASTQ_SSU_OTU.tsv	Root-Host_associated-Human-Skin
ERR1960659	MGYA00130810	https://www.ebi.ac.uk/metagenomics/api/v1/analyses/MGYA00130810/file/ERR1960659_MERGED_FASTQ_otu.tsv	Root-Host_associated-Human-Skin
ERR443439	MGYA00428397	https://www.ebi.ac.uk/metagenomics/api/v1/analyses/MGYA00428397/file/ERR443439_FASTQ_SSU_OTU.tsv	Root-Host_associated-Human-Skin
SRR1632789	MGYA00024073	https://www.ebi.ac.uk/metagenomics/api/v1/analyses/MGYA00024073/file/SRR1632789_MERGED_FASTQ_otu.tsv	Root-Host_associated-Human-Skin
ERR443100	MGYA00415174	https://www.ebi.ac.uk/metagenomics/api/v1/analyses/MGYA00415174/file/ERR443100_FASTQ_SSU_OTU.tsv	Root-Host_associated-Human-Skin

ERR450763	MGYA00416733	https://www.ebi.ac.uk/metagenomics/api/v1/analyses/MGYA00416733/file/ERR450763_FASTQ_SSU_OTU.tsv	Root-Host_associated-Human-Skin
SRR5869180	MGYA00420259	https://www.ebi.ac.uk/metagenomics/api/v1/analyses/MGYA00420259/file/SRR5869180_MERGED_FASTQ_SSU_OTU.tsv	Root-Host_associated-Human-Skin
SRR1631402	MGYA00022433	https://www.ebi.ac.uk/metagenomics/api/v1/analyses/MGYA00022433/file/SRR1631402_MERGED_FASTQ_otu.tsv	Root-Host_associated-Human-Skin
ERR1960701	MGYA00130809	https://www.ebi.ac.uk/metagenomics/api/v1/analyses/MGYA00130809/file/ERR1960701_MERGED_FASTQ_otu.tsv	Root-Host_associated-Human-Skin
SRR1704700	MGYA00420586	https://www.ebi.ac.uk/metagenomics/api/v1/analyses/MGYA00420586/file/SRR1704700_FASTQ_SSU_OTU.tsv	Root-Host_associated-Human-Skin
SRR1631063	MGYA00024811	https://www.ebi.ac.uk/metagenomics/api/v1/analyses/MGYA00024811/file/SRR1631063_MERGED_FASTQ_otu.tsv	Root-Host_associated-Human-Skin
SRR6757675	MGYA00380788	https://www.ebi.ac.uk/metagenomics/api/v1/analyses/MGYA00380788/file/SRR6757675_MERGED_FASTQ_SSU_OTU.tsv	Root-Host_associated-Human-Skin
SRR5868874	MGYA00426652	https://www.ebi.ac.uk/metagenomics/api/v1/analyses/MGYA00426652/file/SRR5868874_MERGED_FASTQ_SSU_OTU.tsv	Root-Host_associated-Human-Skin
SRR1622856	MGYA00024381	https://www.ebi.ac.uk/metagenomics/api/v1/analyses/MGYA00024381/file/SRR1622856_MERGED_FASTQ_otu.tsv	Root-Host_associated-Human-Skin
SRR996148	MGYA00022523	https://www.ebi.ac.uk/metagenomics/api/v1/analyses/MGYA00022523/file/SRR996148_FASTQ_otu.tsv	Root-Host_associated-Human-Skin
ERR2538485	MGYA00381492	https://www.ebi.ac.uk/metagenomics/api/v1/analyses/MGYA00381492/file/ERR2538485_FASTQ_SSU_OTU.tsv	Root-Host_associated-Human-Skin
ERR2538454	MGYA00381461	https://www.ebi.ac.uk/metagenomics/api/v1/analyses/MGYA00381461/file/ERR2538454_FASTQ_SSU_OTU.tsv	Root-Host_associated-Human-Skin

SRR1631715	MGYA00024036	https://www.ebi.ac.uk/metagenomics/api/v1/analyses/MGYA00024036/file/SRR1631715_MERGED_FASTQ_otu.tsv	Root-Host_associated-Human-Skin
SRR920746	MGYA00023542	https://www.ebi.ac.uk/metagenomics/api/v1/analyses/MGYA00023542/file/SRR920746_FASTQ_otu.tsv	Root-Host_associated-Human-Skin
ERR450806	MGYA00423891	https://www.ebi.ac.uk/metagenomics/api/v1/analyses/MGYA00423891/file/ERR450806_FASTQ_SSU_OTU.tsv	Root-Host_associated-Human-Skin
ERR1701273	MGYA00084308	https://www.ebi.ac.uk/metagenomics/api/v1/analyses/MGYA00084308/file/ERR1701273_FASTQ_otu.tsv	Root-Host_associated-Human-Skin
SRR921106	MGYA00025002	https://www.ebi.ac.uk/metagenomics/api/v1/analyses/MGYA00025002/file/SRR921106_FASTQ_otu.tsv	Root-Host_associated-Human-Skin
SRR1923891	MGYA00028367	https://www.ebi.ac.uk/metagenomics/api/v1/analyses/MGYA00028367/file/SRR1923891_FASTQ_otu.tsv	Root-Host_associated-Human-Skin
SRR994978	MGYA00024548	https://www.ebi.ac.uk/metagenomics/api/v1/analyses/MGYA00024548/file/SRR994978_FASTQ_otu.tsv	Root-Host_associated-Human-Skin
ERR443676	MGYA00422019	https://www.ebi.ac.uk/metagenomics/api/v1/analyses/MGYA00422019/file/ERR443676_FASTQ_SSU_OTU.tsv	Root-Host_associated-Human-Skin
ERR443348	MGYA00428744	https://www.ebi.ac.uk/metagenomics/api/v1/analyses/MGYA00428744/file/ERR443348_FASTQ_SSU_OTU.tsv	Root-Host_associated-Human-Skin
ERR441967	MGYA00385234	https://www.ebi.ac.uk/metagenomics/api/v1/analyses/MGYA00385234/file/ERR441967_FASTQ_SSU_OTU.tsv	Root-Host_associated-Human-Skin
SRR996761	MGYA00023858	https://www.ebi.ac.uk/metagenomics/api/v1/analyses/MGYA00023858/file/SRR996761_FASTQ_otu.tsv	Root-Host_associated-Human-Skin
SRR994455	MGYA00022461	https://www.ebi.ac.uk/metagenomics/api/v1/analyses/MGYA00022461/file/SRR994455_FASTQ_otu.tsv	Root-Host_associated-Human-Skin

SRR994827	MGYA00022829	https://www.ebi.ac.uk/metagenomics/api/v1/analyses/MGYA00022829/file/SRR994827_FASTQ_otu.tsv	Root-Host_associated-Human-Skin
ERR442061	MGYA00385199	https://www.ebi.ac.uk/metagenomics/api/v1/analyses/MGYA00385199/file/ERR442061_FASTQ_SSU_OTU.tsv	Root-Host_associated-Human-Skin
SRR996300	MGYA00022712	https://www.ebi.ac.uk/metagenomics/api/v1/analyses/MGYA00022712/file/SRR996300_FASTQ_otu.tsv	Root-Host_associated-Human-Skin
SRR1704548	MGYA00413075	https://www.ebi.ac.uk/metagenomics/api/v1/analyses/MGYA00413075/file/SRR1704548_FASTQ_SSU_OTU.tsv	Root-Host_associated-Human-Skin
SRR995563	MGYA00022981	https://www.ebi.ac.uk/metagenomics/api/v1/analyses/MGYA00022981/file/SRR995563_FASTQ_otu.tsv	Root-Host_associated-Human-Skin
SRR1632448	MGYA00023105	https://www.ebi.ac.uk/metagenomics/api/v1/analyses/MGYA00023105/file/SRR1632448_MERGED_FASTQ_otu.tsv	Root-Host_associated-Human-Skin
SRR994484	MGYA00023141	https://www.ebi.ac.uk/metagenomics/api/v1/analyses/MGYA00023141/file/SRR994484_FASTQ_otu.tsv	Root-Host_associated-Human-Skin
SRR1622909	MGYA00022371	https://www.ebi.ac.uk/metagenomics/api/v1/analyses/MGYA00022371/file/SRR1622909_MERGED_FASTQ_otu.tsv	Root-Host_associated-Human-Skin
SRR919614	MGYA00023530	https://www.ebi.ac.uk/metagenomics/api/v1/analyses/MGYA00023530/file/SRR919614_FASTQ_otu.tsv	Root-Host_associated-Human-Skin
SRR995102	MGYA00023664	https://www.ebi.ac.uk/metagenomics/api/v1/analyses/MGYA00023664/file/SRR995102_FASTQ_otu.tsv	Root-Host_associated-Human-Skin
ERR1760112	MGYA00140357	https://www.ebi.ac.uk/metagenomics/api/v1/analyses/MGYA00140357/file/ERR1760112_MERGED_FASTQ_SSU_OTU.tsv	Root-Host_associated-Human-Skin
ERR443107	MGYA00413007	https://www.ebi.ac.uk/metagenomics/api/v1/analyses/MGYA00413007/file/ERR443107_FASTQ_SSU_OTU.tsv	Root-Host_associated-Human-Skin

SRR1704366	MGYA00428879	https://www.ebi.ac.uk/metagenomics/api/v1/analyses/MGYA00428879/file/SRR1704366_FASTQ_SSU_OTU.tsv	Root-Host_associated-Human-Skin
ERR1759885	MGYA00140541	https://www.ebi.ac.uk/metagenomics/api/v1/analyses/MGYA00140541/file/ERR1759885_MERGED_FASTQ_SSU_OTU.tsv	Root-Host_associated-Human-Skin
SRR1630990	MGYA00022850	https://www.ebi.ac.uk/metagenomics/api/v1/analyses/MGYA00022850/file/SRR1630990_MERGED_FASTQ_otu.tsv	Root-Host_associated-Human-Skin
SRR996289	MGYA00023303	https://www.ebi.ac.uk/metagenomics/api/v1/analyses/MGYA00023303/file/SRR996289_FASTQ_otu.tsv	Root-Host_associated-Human-Skin
ERR443566	MGYA00415121	https://www.ebi.ac.uk/metagenomics/api/v1/analyses/MGYA00415121/file/ERR443566_FASTQ_SSU_OTU.tsv	Root-Host_associated-Human-Skin
ERR1960706	MGYA00130808	https://www.ebi.ac.uk/metagenomics/api/v1/analyses/MGYA00130808/file/ERR1960706_MERGED_FASTQ_otu.tsv	Root-Host_associated-Human-Skin
ERR451545	MGYA00422355	https://www.ebi.ac.uk/metagenomics/api/v1/analyses/MGYA00422355/file/ERR451545_FASTQ_SSU_OTU.tsv	Root-Host_associated-Human-Skin
SRR1622851	MGYA00024386	https://www.ebi.ac.uk/metagenomics/api/v1/analyses/MGYA00024386/file/SRR1622851_MERGED_FASTQ_otu.tsv	Root-Host_associated-Human-Skin
ERR2538482	MGYA00381489	https://www.ebi.ac.uk/metagenomics/api/v1/analyses/MGYA00381489/file/ERR2538482_FASTQ_SSU_OTU.tsv	Root-Host_associated-Human-Skin
SRR5869241	MGYA00419730	https://www.ebi.ac.uk/metagenomics/api/v1/analyses/MGYA00419730/file/SRR5869241_MERGED_FASTQ_SSU_OTU.tsv	Root-Host_associated-Human-Skin
SRR1631064	MGYA00024808	https://www.ebi.ac.uk/metagenomics/api/v1/analyses/MGYA00024808/file/SRR1631064_MERGED_FASTQ_otu.tsv	Root-Host_associated-Human-Skin
SRR6757672	MGYA00380785	https://www.ebi.ac.uk/metagenomics/api/v1/analyses/MGYA00380785/file/SRR6757672_MERGED_FASTQ_SSU_OTU.tsv	Root-Host_associated-Human-Skin

SRR1631712	MGYA00024041	https://www.ebi.ac.uk/metagenomics/api/v1/analyses/MGYA00024041/file/SRR1631712_MERGED_FASTQ_otu.tsv	Root-Host_associated-Human-Skin
SRR997079	MGYA00023157	https://www.ebi.ac.uk/metagenomics/api/v1/analyses/MGYA00023157/file/SRR997079_FASTQ_otu.tsv	Root-Host_associated-Human-Skin
ERR2538453	MGYA00381460	https://www.ebi.ac.uk/metagenomics/api/v1/analyses/MGYA00381460/file/ERR2538453_FASTQ_SSU_OTU.tsv	Root-Host_associated-Human-Skin
SRR921101	MGYA00025003	https://www.ebi.ac.uk/metagenomics/api/v1/analyses/MGYA00025003/file/SRR921101_FASTQ_otu.tsv	Root-Host_associated-Human-Skin
ERR1701274	MGYA00084275	https://www.ebi.ac.uk/metagenomics/api/v1/analyses/MGYA00084275/file/ERR1701274_FASTQ_otu.tsv	Root-Host_associated-Human-Skin
ERR450801	MGYA00426833	https://www.ebi.ac.uk/metagenomics/api/v1/analyses/MGYA00426833/file/ERR450801_FASTQ_SSU_OTU.tsv	Root-Host_associated-Human-Skin
SRR920741	MGYA00023537	https://www.ebi.ac.uk/metagenomics/api/v1/analyses/MGYA00023537/file/SRR920741_FASTQ_otu.tsv	Root-Host_associated-Human-Skin
SRR5869156	MGYA00429114	https://www.ebi.ac.uk/metagenomics/api/v1/analyses/MGYA00429114/file/SRR5869156_MERGED_FASTQ_SSU_OTU.tsv	Root-Host_associated-Human-Skin
ERR443671	MGYA00419446	https://www.ebi.ac.uk/metagenomics/api/v1/analyses/MGYA00419446/file/ERR443671_FASTQ_SSU_OTU.tsv	Root-Host_associated-Human-Skin
SRR1923896	MGYA00028374	https://www.ebi.ac.uk/metagenomics/api/v1/analyses/MGYA00028374/file/SRR1923896_FASTQ_otu.tsv	Root-Host_associated-Human-Skin
SRR995608	MGYA00025046	https://www.ebi.ac.uk/metagenomics/api/v1/analyses/MGYA00025046/file/SRR995608_FASTQ_otu.tsv	Root-Host_associated-Human-Skin
SRR1624848	MGYA00024287	https://www.ebi.ac.uk/metagenomics/api/v1/analyses/MGYA00024287/file/SRR1624848_MERGED_FASTQ_otu.tsv	Root-Host_associated-Human-Skin

SRR1633163	MGYA00022386	https://www.ebi.ac.uk/metagenomics/api/v1/analyses/MGYA00022386/file/SRR1633163_MERGED_FASTQ_otu.tsv	Root-Host_associated-Human-Skin
SRR920665	MGYA00022335	https://www.ebi.ac.uk/metagenomics/api/v1/analyses/MGYA00022335/file/SRR920665_FASTQ_otu.tsv	Root-Host_associated-Human-Skin
ERR443334	MGYA00426050	https://www.ebi.ac.uk/metagenomics/api/v1/analyses/MGYA00426050/file/ERR443334_FASTQ_SSU_OTU.tsv	Root-Host_associated-Human-Skin
SRR1950726	MGYA00376893	https://www.ebi.ac.uk/metagenomics/api/v1/analyses/MGYA00376893/file/SRR1950726_MERGED_FASTQ_SSU_OTU.tsv	Root-Host_associated-Human-Skin
SRR5869072	MGYA00417045	https://www.ebi.ac.uk/metagenomics/api/v1/analyses/MGYA00417045/file/SRR5869072_MERGED_FASTQ_SSU_OTU.tsv	Root-Host_associated-Human-Skin
ERR1759970	MGYA00140471	https://www.ebi.ac.uk/metagenomics/api/v1/analyses/MGYA00140471/file/ERR1759970_MERGED_FASTQ_SSU_OTU.tsv	Root-Host_associated-Human-Skin
SRR5868632	MGYA00418799	https://www.ebi.ac.uk/metagenomics/api/v1/analyses/MGYA00418799/file/SRR5868632_MERGED_FASTQ_SSU_OTU.tsv	Root-Host_associated-Human-Skin
SRR1631257	MGYA00024546	https://www.ebi.ac.uk/metagenomics/api/v1/analyses/MGYA00024546/file/SRR1631257_MERGED_FASTQ_otu.tsv	Root-Host_associated-Human-Skin
SRR1704534	MGYA00417446	https://www.ebi.ac.uk/metagenomics/api/v1/analyses/MGYA00417446/file/SRR1704534_FASTQ_SSU_OTU.tsv	Root-Host_associated-Human-Skin
SRR994429	MGYA00024967	https://www.ebi.ac.uk/metagenomics/api/v1/analyses/MGYA00024967/file/SRR994429_FASTQ_otu.tsv	Root-Host_associated-Human-Skin
SRR920204	MGYA00023061	https://www.ebi.ac.uk/metagenomics/api/v1/analyses/MGYA00023061/file/SRR920204_FASTQ_otu.tsv	Root-Host_associated-Human-Skin
SRR1632342	MGYA00025166	https://www.ebi.ac.uk/metagenomics/api/v1/analyses/MGYA00025166/file/SRR1632342_MERGED_FASTQ_otu.tsv	Root-Host_associated-Human-Skin

SRR1923963	MGYA00028222	https://www.ebi.ac.uk/metagenomics/api/v1/analyses/MGYA00028222/file/SRR1923963_FASTQ_otu.tsv	Root-Host_associated-Human-Skin
ERR451000	MGYA00415177	https://www.ebi.ac.uk/metagenomics/api/v1/analyses/MGYA00415177/file/ERR451000_FASTQ_SSU_OTU.tsv	Root-Host_associated-Human-Skin
SRR1631286	MGYA00022656	https://www.ebi.ac.uk/metagenomics/api/v1/analyses/MGYA00022656/file/SRR1631286_MERGED_FASTQ_otu.tsv	Root-Host_associated-Human-Skin
SRR5869704	MGYA00416979	https://www.ebi.ac.uk/metagenomics/api/v1/analyses/MGYA00416979/file/SRR5869704_MERGED_FASTQ_SSU_OTU.tsv	Root-Host_associated-Human-Skin
SRR996878	MGYA00023443	https://www.ebi.ac.uk/metagenomics/api/v1/analyses/MGYA00023443/file/SRR996878_FASTQ_otu.tsv	Root-Host_associated-Human-Skin
SRR2005721	MGYA00381350	https://www.ebi.ac.uk/metagenomics/api/v1/analyses/MGYA00381350/file/SRR2005721_MERGED_FASTQ_SSU_OTU.tsv	Root-Host_associated-Human-Skin
ERR443442	MGYA00426010	https://www.ebi.ac.uk/metagenomics/api/v1/analyses/MGYA00426010/file/ERR443442_FASTQ_SSU_OTU.tsv	Root-Host_associated-Human-Skin
SRR920113	MGYA00024126	https://www.ebi.ac.uk/metagenomics/api/v1/analyses/MGYA00024126/file/SRR920113_FASTQ_otu.tsv	Root-Host_associated-Human-Skin
SRR1704623	MGYA00413812	https://www.ebi.ac.uk/metagenomics/api/v1/analyses/MGYA00413812/file/SRR1704623_FASTQ_SSU_OTU.tsv	Root-Host_associated-Human-Skin
SRR1704184	MGYA00428932	https://www.ebi.ac.uk/metagenomics/api/v1/analyses/MGYA00428932/file/SRR1704184_FASTQ_SSU_OTU.tsv	Root-Host_associated-Human-Skin
SRR919884	MGYA00024499	https://www.ebi.ac.uk/metagenomics/api/v1/analyses/MGYA00024499/file/SRR919884_FASTQ_otu.tsv	Root-Host_associated-Human-Skin
SRR996552	MGYA00022619	https://www.ebi.ac.uk/metagenomics/api/v1/analyses/MGYA00022619/file/SRR996552_FASTQ_otu.tsv	Root-Host_associated-Human-Skin

SRR996920	MGYA00025127	https://www.ebi.ac.uk/metagenomics/api/v1/analyses/MGYA00025127/file/SRR996920_FASTQ_otu.tsv	Root-Host_associated-Human-Skin
ERR2538359	MGYA00381366	https://www.ebi.ac.uk/metagenomics/api/v1/analyses/MGYA00381366/file/ERR2538359_FASTQ_SSU_OTU.tsv	Root-Host_associated-Human-Skin
SRR996694	MGYA00022289	https://www.ebi.ac.uk/metagenomics/api/v1/analyses/MGYA00022289/file/SRR996694_FASTQ_otu.tsv	Root-Host_associated-Human-Skin
SRR996133	MGYA00024990	https://www.ebi.ac.uk/metagenomics/api/v1/analyses/MGYA00024990/file/SRR996133_FASTQ_otu.tsv	Root-Host_associated-Human-Skin
SRR1924023	MGYA00028380	https://www.ebi.ac.uk/metagenomics/api/v1/analyses/MGYA00028380/file/SRR1924023_FASTQ_otu.tsv	Root-Host_associated-Human-Skin
SRR3217222	MGYA00412222	https://www.ebi.ac.uk/metagenomics/api/v1/analyses/MGYA00412222/file/SRR3217222_FASTQ_SSU_OTU.tsv	Root-Host_associated-Human-Skin
SRR5870056	MGYA00422530	https://www.ebi.ac.uk/metagenomics/api/v1/analyses/MGYA00422530/file/SRR5870056_MERGED_FASTQ_SSU_OTU.tsv	Root-Host_associated-Human-Skin
ERR1760036	MGYA00140563	https://www.ebi.ac.uk/metagenomics/api/v1/analyses/MGYA00140563/file/ERR1760036_MERGED_FASTQ_SSU_OTU.tsv	Root-Host_associated-Human-Skin
SRR995026	MGYA00023948	https://www.ebi.ac.uk/metagenomics/api/v1/analyses/MGYA00023948/file/SRR995026_FASTQ_otu.tsv	Root-Host_associated-Human-Skin
SRR995750	MGYA00022896	https://www.ebi.ac.uk/metagenomics/api/v1/analyses/MGYA00022896/file/SRR995750_FASTQ_otu.tsv	Root-Host_associated-Human-Skin
ERR1701208	MGYA00084311	https://www.ebi.ac.uk/metagenomics/api/v1/analyses/MGYA00084311/file/ERR1701208_FASTQ_otu.tsv	Root-Host_associated-Human-Skin
SRR5870087	MGYA00418703	https://www.ebi.ac.uk/metagenomics/api/v1/analyses/MGYA00418703/file/SRR5870087_MERGED_FASTQ_SSU_OTU.tsv	Root-Host_associated-Human-Skin

ERR2538388	MGYA00381395	https://www.ebi.ac.uk/metagenomics/api/v1/analyses/MGYA00381395/file/ERR2538388_FASTQ_SSU_OTU.tsv	Root-Host_associated-Human-Skin
SRR5869939	MGYA00415310	https://www.ebi.ac.uk/metagenomics/api/v1/analyses/MGYA00415310/file/SRR5869939_MERGED_FASTQ_SSU_OTU.tsv	Root-Host_associated-Human-Skin
SRR995496	MGYA00024117	https://www.ebi.ac.uk/metagenomics/api/v1/analyses/MGYA00024117/file/SRR995496_FASTQ_otu.tsv	Root-Host_associated-Human-Skin
SRR996224	MGYA00023973	https://www.ebi.ac.uk/metagenomics/api/v1/analyses/MGYA00023973/file/SRR996224_FASTQ_otu.tsv	Root-Host_associated-Human-Skin
ERR2538505	MGYA00381512	https://www.ebi.ac.uk/metagenomics/api/v1/analyses/MGYA00381512/file/ERR2538505_FASTQ_SSU_OTU.tsv	Root-Host_associated-Human-Skin
ERR441969	MGYA00385249	https://www.ebi.ac.uk/metagenomics/api/v1/analyses/MGYA00385249/file/ERR441969_FASTQ_SSU_OTU.tsv	Root-Host_associated-Human-Skin
ERR1960681	MGYA00130806	https://www.ebi.ac.uk/metagenomics/api/v1/analyses/MGYA00130806/file/ERR1960681_MERGED_FASTQ_otu.tsv	Root-Host_associated-Human-Skin
ERR450957	MGYA00415558	https://www.ebi.ac.uk/metagenomics/api/v1/analyses/MGYA00415558/file/ERR450957_FASTQ_SSU_OTU.tsv	Root-Host_associated-Human-Skin
SRR921057	MGYA00022857	https://www.ebi.ac.uk/metagenomics/api/v1/analyses/MGYA00022857/file/SRR921057_FASTQ_otu.tsv	Root-Host_associated-Human-Skin
ERR442974	MGYA00425261	https://www.ebi.ac.uk/metagenomics/api/v1/analyses/MGYA00425261/file/ERR442974_FASTQ_SSU_OTU.tsv	Root-Host_associated-Human-Skin
SRR5868640	MGYA00411946	https://www.ebi.ac.uk/metagenomics/api/v1/analyses/MGYA00411946/file/SRR5868640_MERGED_FASTQ_SSU_OTU.tsv	Root-Host_associated-Human-Skin
ERR1759902	MGYA00140528	https://www.ebi.ac.uk/metagenomics/api/v1/analyses/MGYA00140528/file/ERR1759902_MERGED_FASTQ_SSU_OTU.tsv	Root-Host_associated-Human-Skin

SRR1950754	MGYA00376921	https://www.ebi.ac.uk/metagenomics/api/v1/analyses/MGYA00376921/file/SRR1950754_MERGED_FASTQ_SSU_OTU.tsv	Root-Host_associated-Human-Skin
SRR1632330	MGYA00024334	https://www.ebi.ac.uk/metagenomics/api/v1/analyses/MGYA00024334/file/SRR1632330_MERGED_FASTQ_otu.tsv	Root-Host_associated-Human-Skin
SRR994829	MGYA00022827	https://www.ebi.ac.uk/metagenomics/api/v1/analyses/MGYA00022827/file/SRR994829_FASTQ_otu.tsv	Root-Host_associated-Human-Skin
SRR920972	MGYA00023037	https://www.ebi.ac.uk/metagenomics/api/v1/analyses/MGYA00023037/file/SRR920972_FASTQ_otu.tsv	Root-Host_associated-Human-Skin
SRR1632834	MGYA00022814	https://www.ebi.ac.uk/metagenomics/api/v1/analyses/MGYA00022814/file/SRR1632834_MERGED_FASTQ_otu.tsv	Root-Host_associated-Human-Skin
ERR451072	MGYA00424909	https://www.ebi.ac.uk/metagenomics/api/v1/analyses/MGYA00424909/file/ERR451072_FASTQ_SSU_OTU.tsv	Root-Host_associated-Human-Skin
SRR1923911	MGYA00028299	https://www.ebi.ac.uk/metagenomics/api/v1/analyses/MGYA00028299/file/SRR1923911_FASTQ_otu.tsv	Root-Host_associated-Human-Skin
SRR996478	MGYA00024913	https://www.ebi.ac.uk/metagenomics/api/v1/analyses/MGYA00024913/file/SRR996478_FASTQ_otu.tsv	Root-Host_associated-Human-Skin
SRR1950785	MGYA00376952	https://www.ebi.ac.uk/metagenomics/api/v1/analyses/MGYA00376952/file/SRR1950785_MERGED_FASTQ_SSU_OTU.tsv	Root-Host_associated-Human-Skin
SRR5868691	MGYA00423065	https://www.ebi.ac.uk/metagenomics/api/v1/analyses/MGYA00423065/file/SRR5868691_MERGED_FASTQ_SSU_OTU.tsv	Root-Host_associated-Human-Skin
ERR1960650	MGYA00130743	https://www.ebi.ac.uk/metagenomics/api/v1/analyses/MGYA00130743/file/ERR1960650_MERGED_FASTQ_otu.tsv	Root-Host_associated-Human-Skin
ERR443397	MGYA00428726	https://www.ebi.ac.uk/metagenomics/api/v1/analyses/MGYA00428726/file/ERR443397_FASTQ_SSU_OTU.tsv	Root-Host_associated-Human-Skin

SRR1622907	MGYA00024368	https://www.ebi.ac.uk/metagenomics/api/v1/analyses/MGYA00024368/file/SRR1622907_MERGED_FASTQ_otu.tsv	Root-Host_associated-Human-Skin
SRR996952	MGYA00022876	https://www.ebi.ac.uk/metagenomics/api/v1/analyses/MGYA00022876/file/SRR996952_FASTQ_otu.tsv	Root-Host_associated-Human-Skin
SRR996287	MGYA00023307	https://www.ebi.ac.uk/metagenomics/api/v1/analyses/MGYA00023307/file/SRR996287_FASTQ_otu.tsv	Root-Host_associated-Human-Skin
SRR1704368	MGYA00423390	https://www.ebi.ac.uk/metagenomics/api/v1/analyses/MGYA00423390/file/SRR1704368_FASTQ_SSU_OTU.tsv	Root-Host_associated-Human-Skin
SRR995392	MGYA00024759	https://www.ebi.ac.uk/metagenomics/api/v1/analyses/MGYA00024759/file/SRR995392_FASTQ_otu.tsv	Root-Host_associated-Human-Skin
SRR996141	MGYA00022526	https://www.ebi.ac.uk/metagenomics/api/v1/analyses/MGYA00022526/file/SRR996141_FASTQ_otu.tsv	Root-Host_associated-Human-Skin
ERR1760044	MGYA00140458	https://www.ebi.ac.uk/metagenomics/api/v1/analyses/MGYA00140458/file/ERR1760044_MERGED_FASTQ_SSU_OTU.tsv	Root-Host_associated-Human-Skin
SRR994614	MGYA00023017	https://www.ebi.ac.uk/metagenomics/api/v1/analyses/MGYA00023017/file/SRR994614_FASTQ_otu.tsv	Root-Host_associated-Human-Skin
ERR443568	MGYA00412359	https://www.ebi.ac.uk/metagenomics/api/v1/analyses/MGYA00412359/file/ERR443568_FASTQ_SSU_OTU.tsv	Root-Host_associated-Human-Skin
ERR1960708	MGYA00130774	https://www.ebi.ac.uk/metagenomics/api/v1/analyses/MGYA00130774/file/ERR1960708_MERGED_FASTQ_otu.tsv	Root-Host_associated-Human-Skin
SRR995085	MGYA00022722	https://www.ebi.ac.uk/metagenomics/api/v1/analyses/MGYA00022722/file/SRR995085_FASTQ_otu.tsv	Root-Host_associated-Human-Skin
ERR1760095	MGYA00140524	https://www.ebi.ac.uk/metagenomics/api/v1/analyses/MGYA00140524/file/ERR1760095_MERGED_FASTQ_SSU_OTU.tsv	Root-Host_associated-Human-Skin

ERR981250	MGYA00010233	https://www.ebi.ac.uk/metagenomics/api/v1/analyses/MGYA00010233/file/ERR981250_MERGED_FASTQ_otu.tsv	Root-Host_associated-Human-Skin
SRR997077	MGYA00023165	https://www.ebi.ac.uk/metagenomics/api/v1/analyses/MGYA00023165/file/SRR997077_FASTQ_otu.tsv	Root-Host_associated-Human-Skin
SRR995343	MGYA00023125	https://www.ebi.ac.uk/metagenomics/api/v1/analyses/MGYA00023125/file/SRR995343_FASTQ_otu.tsv	Root-Host_associated-Human-Skin
SRR1923898	MGYA00028366	https://www.ebi.ac.uk/metagenomics/api/v1/analyses/MGYA00028366/file/SRR1923898_FASTQ_otu.tsv	Root-Host_associated-Human-Skin
SRR994971	MGYA00024551	https://www.ebi.ac.uk/metagenomics/api/v1/analyses/MGYA00024551/file/SRR994971_FASTQ_otu.tsv	Root-Host_associated-Human-Skin
SRR994503	MGYA00023817	https://www.ebi.ac.uk/metagenomics/api/v1/analyses/MGYA00023817/file/SRR994503_FASTQ_otu.tsv	Root-Host_associated-Human-Skin
SRR5868718	MGYA00417127	https://www.ebi.ac.uk/metagenomics/api/v1/analyses/MGYA00417127/file/SRR5868718_MERGED_FASTQ_SSU_OTU.tsv	Root-Host_associated-Human-Skin
SRR996983	MGYA00024534	https://www.ebi.ac.uk/metagenomics/api/v1/analyses/MGYA00024534/file/SRR996983_FASTQ_otu.tsv	Root-Host_associated-Human-Skin
SRR5869158	MGYA00429108	https://www.ebi.ac.uk/metagenomics/api/v1/analyses/MGYA00429108/file/SRR5869158_MERGED_FASTQ_SSU_OTU.tsv	Root-Host_associated-Human-Skin
SRR995606	MGYA00025039	https://www.ebi.ac.uk/metagenomics/api/v1/analyses/MGYA00025039/file/SRR995606_FASTQ_otu.tsv	Root-Host_associated-Human-Skin
SRR1624846	MGYA00024293	https://www.ebi.ac.uk/metagenomics/api/v1/analyses/MGYA00024293/file/SRR1624846_MERGED_FASTQ_otu.tsv	Root-Host_associated-Human-Skin
ERR442908	MGYA00422392	https://www.ebi.ac.uk/metagenomics/api/v1/analyses/MGYA00422392/file/ERR442908_FASTQ_SSU_OTU.tsv	Root-Host_associated-Human-Skin

ERR442013	MGYA00385246	https://www.ebi.ac.uk/metagenomics/api/v1/analyses/MGYA00385246/file/ERR442013_FASTQ_SSU_OTU.tsv	Root-Host_associated-Human-Skin
SRR994427	MGYA00024963	https://www.ebi.ac.uk/metagenomics/api/v1/analyses/MGYA00024963/file/SRR994427_FASTQ_otu.tsv	Root-Host_associated-Human-Skin
SRR1631259	MGYA00024538	https://www.ebi.ac.uk/metagenomics/api/v1/analyses/MGYA00024538/file/SRR1631259_MERGED_FASTQ_otu.tsv	Root-Host_associated-Human-Skin
SRR919903	MGYA00025029	https://www.ebi.ac.uk/metagenomics/api/v1/analyses/MGYA00025029/file/SRR919903_FASTQ_otu.tsv	Root-Host_associated-Human-Skin
SRR919571	MGYA00024724	https://www.ebi.ac.uk/metagenomics/api/v1/analyses/MGYA00024724/file/SRR919571_FASTQ_otu.tsv	Root-Host_associated-Human-Skin
SRR1950728	MGYA00376895	https://www.ebi.ac.uk/metagenomics/api/v1/analyses/MGYA00376895/file/SRR1950728_MERGED_FASTQ_SSU_OTU.tsv	Root-Host_associated-Human-Skin
SRR996876	MGYA00023435	https://www.ebi.ac.uk/metagenomics/api/v1/analyses/MGYA00023435/file/SRR996876_FASTQ_otu.tsv	Root-Host_associated-Human-Skin
SRR1631288	MGYA00025250	https://www.ebi.ac.uk/metagenomics/api/v1/analyses/MGYA00025250/file/SRR1631288_MERGED_FASTQ_otu.tsv	Root-Host_associated-Human-Skin
SRR994884	MGYA00022182	https://www.ebi.ac.uk/metagenomics/api/v1/analyses/MGYA00022182/file/SRR994884_FASTQ_otu.tsv	Root-Host_associated-Human-Skin
SRR995963	MGYA00023853	https://www.ebi.ac.uk/metagenomics/api/v1/analyses/MGYA00023853/file/SRR995963_FASTQ_otu.tsv	Root-Host_associated-Human-Skin
SRR996065	MGYA00023319	https://www.ebi.ac.uk/metagenomics/api/v1/analyses/MGYA00023319/file/SRR996065_FASTQ_otu.tsv	Root-Host_associated-Human-Skin
SRR995170	MGYA00022163	https://www.ebi.ac.uk/metagenomics/api/v1/analyses/MGYA00022163/file/SRR995170_FASTQ_otu.tsv	Root-Host_associated-Human-Skin

SRR994730	MGYA00023893	https://www.ebi.ac.uk/metagenomics/api/v1/analyses/MGYA00023893/file/SRR994730_FASTQ_otu.tsv	Root-Host_associated-Human-Skin
SRR995028	MGYA00023942	https://www.ebi.ac.uk/metagenomics/api/v1/analyses/MGYA00023942/file/SRR995028_FASTQ_otu.tsv	Root-Host_associated-Human-Skin
ERR1760038	MGYA00140567	https://www.ebi.ac.uk/metagenomics/api/v1/analyses/MGYA00140567/file/ERR1760038_MERGED_FASTQ_SSU_OTU.tsv	Root-Host_associated-Human-Skin
SRR1923781	MGYA00028235	https://www.ebi.ac.uk/metagenomics/api/v1/analyses/MGYA00028235/file/SRR1923781_FASTQ_otu.tsv	Root-Host_associated-Human-Skin
ERR1960774	MGYA00130736	https://www.ebi.ac.uk/metagenomics/api/v1/analyses/MGYA00130736/file/ERR1960774_MERGED_FASTQ_otu.tsv	Root-Host_associated-Human-Skin
SRR5868801	MGYA00416053	https://www.ebi.ac.uk/metagenomics/api/v1/analyses/MGYA00416053/file/SRR5868801_MERGED_FASTQ_SSU_OTU.tsv	Root-Host_associated-Human-Skin
SRR6757600	MGYA00380719	https://www.ebi.ac.uk/metagenomics/api/v1/analyses/MGYA00380719/file/SRR6757600_MERGED_FASTQ_SSU_OTU.tsv	Root-Host_associated-Human-Skin
SRR1623211	MGYA00023910	https://www.ebi.ac.uk/metagenomics/api/v1/analyses/MGYA00023910/file/SRR1623211_MERGED_FASTQ_otu.tsv	Root-Host_associated-Human-Skin
ERR2538357	MGYA00381364	https://www.ebi.ac.uk/metagenomics/api/v1/analyses/MGYA00381364/file/ERR2538357_FASTQ_SSU_OTU.tsv	Root-Host_associated-Human-Skin
SRR5869233	MGYA00415374	https://www.ebi.ac.uk/metagenomics/api/v1/analyses/MGYA00415374/file/SRR5869233_MERGED_FASTQ_SSU_OTU.tsv	Root-Host_associated-Human-Skin
ERR2538386	MGYA00381393	https://www.ebi.ac.uk/metagenomics/api/v1/analyses/MGYA00381393/file/ERR2538386_FASTQ_SSU_OTU.tsv	Root-Host_associated-Human-Skin
ERR2538421	MGYA00381428	https://www.ebi.ac.uk/metagenomics/api/v1/analyses/MGYA00381428/file/ERR2538421_FASTQ_SSU_OTU.tsv	Root-Host_associated-Human-Skin

ERR1701206	MGYA00084254	https://www.ebi.ac.uk/metagenomics/api/v1/analyses/MGYA00084254/file/ERR1701206_FASTQ_otu.tsv	Root-Host_associated-Human-Skin
SRR1704462	MGYA00426929	https://www.ebi.ac.uk/metagenomics/api/v1/analyses/MGYA00426929/file/SRR1704462_FASTQ_SSU_OTU.tsv	Root-Host_associated-Human-Skin
SRR1631301	MGYA00023233	https://www.ebi.ac.uk/metagenomics/api/v1/analyses/MGYA00023233/file/SRR1631301_MERGED_FASTQ_otu.tsv	Root-Host_associated-Human-Skin
SRR5869683	MGYA00415892	https://www.ebi.ac.uk/metagenomics/api/v1/analyses/MGYA00415892/file/SRR5869683_MERGED_FASTQ_SSU_OTU.tsv	Root-Host_associated-Human-Skin
SRR5869124	MGYA00417018	https://www.ebi.ac.uk/metagenomics/api/v1/analyses/MGYA00417018/file/SRR5869124_MERGED_FASTQ_SSU_OTU.tsv	Root-Host_associated-Human-Skin
SRR995498	MGYA00024119	https://www.ebi.ac.uk/metagenomics/api/v1/analyses/MGYA00024119/file/SRR995498_FASTQ_otu.tsv	Root-Host_associated-Human-Skin
ERR1960811	MGYA00130877	https://www.ebi.ac.uk/metagenomics/api/v1/analyses/MGYA00130877/file/ERR1960811_MERGED_FASTQ_otu.tsv	Root-Host_associated-Human-Skin
SRR1624841	MGYA00024292	https://www.ebi.ac.uk/metagenomics/api/v1/analyses/MGYA00024292/file/SRR1624841_MERGED_FASTQ_otu.tsv	Root-Host_associated-Human-Skin
SRR995601	MGYA00025040	https://www.ebi.ac.uk/metagenomics/api/v1/analyses/MGYA00025040/file/SRR995601_FASTQ_otu.tsv	Root-Host_associated-Human-Skin
ERR1551331	MGYA00069424	https://www.ebi.ac.uk/metagenomics/api/v1/analyses/MGYA00069424/file/ERR1551331_MERGED_FASTQ_otu.tsv	Root-Host_associated-Human-Skin
ERR442014	MGYA00385191	https://www.ebi.ac.uk/metagenomics/api/v1/analyses/MGYA00385191/file/ERR442014_FASTQ_SSU_OTU.tsv	Root-Host_associated-Human-Skin
SRR919576	MGYA00024727	https://www.ebi.ac.uk/metagenomics/api/v1/analyses/MGYA00024727/file/SRR919576_FASTQ_otu.tsv	Root-Host_associated-Human-Skin

SRR919904	MGYA00025026	https://www.ebi.ac.uk/metagenomics/api/v1/analyses/MGYA00025026/file/SRR919904_FASTQ_otu.tsv	Root-Host_associated-Human-Skin
ERR1759979	MGYA00140607	https://www.ebi.ac.uk/metagenomics/api/v1/analyses/MGYA00140607/file/ERR1759979_MERGED_FASTQ_SSU_OTU.tsv	Root-Host_associated-Human-Skin
SRR996871	MGYA00023440	https://www.ebi.ac.uk/metagenomics/api/v1/analyses/MGYA00023440/file/SRR996871_FASTQ_otu.tsv	Root-Host_associated-Human-Skin
ERR451009	MGYA00426530	https://www.ebi.ac.uk/metagenomics/api/v1/analyses/MGYA00426530/file/ERR451009_FASTQ_SSU_OTU.tsv	Root-Host_associated-Human-Skin
SRR994883	MGYA00022189	https://www.ebi.ac.uk/metagenomics/api/v1/analyses/MGYA00022189/file/SRR994883_FASTQ_otu.tsv	Root-Host_associated-Human-Skin
SRR996062	MGYA00023316	https://www.ebi.ac.uk/metagenomics/api/v1/analyses/MGYA00023316/file/SRR996062_FASTQ_otu.tsv	Root-Host_associated-Human-Skin
SRR994737	MGYA00023468	https://www.ebi.ac.uk/metagenomics/api/v1/analyses/MGYA00023468/file/SRR994737_FASTQ_otu.tsv	Root-Host_associated-Human-Skin
SRR5870107	MGYA00422526	https://www.ebi.ac.uk/metagenomics/api/v1/analyses/MGYA00422526/file/SRR5870107_MERGED_FASTQ_SSU_OTU.tsv	Root-Host_associated-Human-Skin
ERR443172	MGYA00415548	https://www.ebi.ac.uk/metagenomics/api/v1/analyses/MGYA00415548/file/ERR443172_FASTQ_SSU_OTU.tsv	Root-Host_associated-Human-Skin
SRR2005670	MGYA00381327	https://www.ebi.ac.uk/metagenomics/api/v1/analyses/MGYA00381327/file/SRR2005670_MERGED_FASTQ_SSU_OTU.tsv	Root-Host_associated-Human-Skin
SRR996929	MGYA00025134	https://www.ebi.ac.uk/metagenomics/api/v1/analyses/MGYA00025134/file/SRR996929_FASTQ_otu.tsv	Root-Host_associated-Human-Skin
SRR5869655	MGYA00416998	https://www.ebi.ac.uk/metagenomics/api/v1/analyses/MGYA00416998/file/SRR5869655_MERGED_FASTQ_SSU_OTU.tsv	Root-Host_associated-Human-Skin

ERR1960773	MGYA00130795	https://www.ebi.ac.uk/metagenomics/api/v1/analyses/MGYA00130795/file/ERR1960773_MERGED_FASTQ_otu.tsv	Root-Host_associated-Human-Skin
SRR1923786	MGYA00028234	https://www.ebi.ac.uk/metagenomics/api/v1/analyses/MGYA00028234/file/SRR1923786_FASTQ_otu.tsv	Root-Host_associated-Human-Skin
ERR451297	MGYA00412624	https://www.ebi.ac.uk/metagenomics/api/v1/analyses/MGYA00412624/file/ERR451297_FASTQ_SSU_OTU.tsv	Root-Host_associated-Human-Skin
ERR2538350	MGYA00381357	https://www.ebi.ac.uk/metagenomics/api/v1/analyses/MGYA00381357/file/ERR2538350_FASTQ_SSU_OTU.tsv	Root-Host_associated-Human-Skin
SRR5869234	MGYA00418780	https://www.ebi.ac.uk/metagenomics/api/v1/analyses/MGYA00418780/file/SRR5869234_MERGED_FASTQ_SSU_OTU.tsv	Root-Host_associated-Human-Skin
SRR1623216	MGYA00023911	https://www.ebi.ac.uk/metagenomics/api/v1/analyses/MGYA00023911/file/SRR1623216_MERGED_FASTQ_otu.tsv	Root-Host_associated-Human-Skin
SRR6757607	MGYA00380813	https://www.ebi.ac.uk/metagenomics/api/v1/analyses/MGYA00380813/file/SRR6757607_MERGED_FASTQ_SSU_OTU.tsv	Root-Host_associated-Human-Skin
SRR5868806	MGYA00420762	https://www.ebi.ac.uk/metagenomics/api/v1/analyses/MGYA00420762/file/SRR5868806_MERGED_FASTQ_SSU_OTU.tsv	Root-Host_associated-Human-Skin
ERR2538426	MGYA00381433	https://www.ebi.ac.uk/metagenomics/api/v1/analyses/MGYA00381433/file/ERR2538426_FASTQ_SSU_OTU.tsv	Root-Host_associated-Human-Skin
SRR5869930	MGYA00414821	https://www.ebi.ac.uk/metagenomics/api/v1/analyses/MGYA00414821/file/SRR5869930_MERGED_FASTQ_SSU_OTU.tsv	Root-Host_associated-Human-Skin
ERR2538381	MGYA00381388	https://www.ebi.ac.uk/metagenomics/api/v1/analyses/MGYA00381388/file/ERR2538381_FASTQ_SSU_OTU.tsv	Root-Host_associated-Human-Skin
SRR1633032	MGYA00024155	https://www.ebi.ac.uk/metagenomics/api/v1/analyses/MGYA00024155/file/SRR1633032_MERGED_FASTQ_otu.tsv	Root-Host_associated-Human-Skin

SRR920734	MGYA00025018	https://www.ebi.ac.uk/metagenomics/api/v1/analyses/MGYA00025018/file/SRR920734_FASTQ_otu.tsv	Root-Host_associated-Human-Skin
SRR1630934	MGYA00023968	https://www.ebi.ac.uk/metagenomics/api/v1/analyses/MGYA00023968/file/SRR1630934_MERGED_FASTQ_otu.tsv	Root-Host_associated-Human-Skin
SRR1631306	MGYA00023228	https://www.ebi.ac.uk/metagenomics/api/v1/analyses/MGYA00023228/file/SRR1631306_MERGED_FASTQ_otu.tsv	Root-Host_associated-Human-Skin
ERR1960816	MGYA00130728	https://www.ebi.ac.uk/metagenomics/api/v1/analyses/MGYA00130728/file/ERR1960816_MERGED_FASTQ_otu.tsv	Root-Host_associated-Human-Skin
SRR996768	MGYA00023855	https://www.ebi.ac.uk/metagenomics/api/v1/analyses/MGYA00023855/file/SRR996768_FASTQ_otu.tsv	Root-Host_associated-Human-Skin
ERR2538502	MGYA00381509	https://www.ebi.ac.uk/metagenomics/api/v1/analyses/MGYA00381509/file/ERR2538502_FASTQ_SSU_OTU.tsv	Root-Host_associated-Human-Skin
SRR921050	MGYA00022864	https://www.ebi.ac.uk/metagenomics/api/v1/analyses/MGYA00022864/file/SRR921050_FASTQ_otu.tsv	Root-Host_associated-Human-Skin
ERR450950	MGYA00412392	https://www.ebi.ac.uk/metagenomics/api/v1/analyses/MGYA00412392/file/ERR450950_FASTQ_SSU_OTU.tsv	Root-Host_associated-Human-Skin
ERR1960686	MGYA00130812	https://www.ebi.ac.uk/metagenomics/api/v1/analyses/MGYA00130812/file/ERR1960686_MERGED_FASTQ_otu.tsv	Root-Host_associated-Human-Skin
SRR996309	MGYA00022704	https://www.ebi.ac.uk/metagenomics/api/v1/analyses/MGYA00022704/file/SRR996309_FASTQ_otu.tsv	Root-Host_associated-Human-Skin
SRR1950753	MGYA00376920	https://www.ebi.ac.uk/metagenomics/api/v1/analyses/MGYA00376920/file/SRR1950753_MERGED_FASTQ_SSU_OTU.tsv	Root-Host_associated-Human-Skin
ERR1759905	MGYA00140422	https://www.ebi.ac.uk/metagenomics/api/v1/analyses/MGYA00140422/file/ERR1759905_MERGED_FASTQ_SSU_OTU.tsv	Root-Host_associated-Human-Skin

SRR5868647	MGYA00411944	https://www.ebi.ac.uk/metagenomics/api/v1/analyses/MGYA00411944/file/SRR5868647_MERGED_FASTQ_SSU_OTU.tsv	Root-Host_associated-Human-Skin
ERR443720	MGYA00415500	https://www.ebi.ac.uk/metagenomics/api/v1/analyses/MGYA00415500/file/ERR443720_FASTQ_SSU_OTU.tsv	Root-Host_associated-Human-Skin
ERR443087	MGYA00422036	https://www.ebi.ac.uk/metagenomics/api/v1/analyses/MGYA00422036/file/ERR443087_FASTQ_SSU_OTU.tsv	Root-Host_associated-Human-Skin
SRR1624789	MGYA00022543	https://www.ebi.ac.uk/metagenomics/api/v1/analyses/MGYA00022543/file/SRR1624789_MERGED_FASTQ_otu.tsv	Root-Host_associated-Human-Skin
SRR1923916	MGYA00028296	https://www.ebi.ac.uk/metagenomics/api/v1/analyses/MGYA00028296/file/SRR1923916_FASTQ_otu.tsv	Root-Host_associated-Human-Skin
ERR451075	MGYA00422033	https://www.ebi.ac.uk/metagenomics/api/v1/analyses/MGYA00422033/file/ERR451075_FASTQ_SSU_OTU.tsv	Root-Host_associated-Human-Skin
SRR1632833	MGYA00022821	https://www.ebi.ac.uk/metagenomics/api/v1/analyses/MGYA00022821/file/SRR1632833_MERGED_FASTQ_otu.tsv	Root-Host_associated-Human-Skin
SRR920975	MGYA00023042	https://www.ebi.ac.uk/metagenomics/api/v1/analyses/MGYA00023042/file/SRR920975_FASTQ_otu.tsv	Root-Host_associated-Human-Skin
SRR1704237	MGYA00426998	https://www.ebi.ac.uk/metagenomics/api/v1/analyses/MGYA00426998/file/SRR1704237_FASTQ_SSU_OTU.tsv	Root-Host_associated-Human-Skin
SRR1631554	MGYA00023799	https://www.ebi.ac.uk/metagenomics/api/v1/analyses/MGYA00023799/file/SRR1631554_MERGED_FASTQ_otu.tsv	Root-Host_associated-Human-Skin
SRR1704590	MGYA00426915	https://www.ebi.ac.uk/metagenomics/api/v1/analyses/MGYA00426915/file/SRR1704590_FASTQ_SSU_OTU.tsv	Root-Host_associated-Human-Skin
SRR1950782	MGYA00376949	https://www.ebi.ac.uk/metagenomics/api/v1/analyses/MGYA00376949/file/SRR1950782_MERGED_FASTQ_SSU_OTU.tsv	Root-Host_associated-Human-Skin

ERR1960657	MGYA00130785	https://www.ebi.ac.uk/metagenomics/api/v1/analyses/MGYA00130785/file/ERR1960657_MERGED_FASTQ_otu.tsv	Root-Host_associated-Human-Skin
SRR1622900	MGYA00022365	https://www.ebi.ac.uk/metagenomics/api/v1/analyses/MGYA00022365/file/SRR1622900_MERGED_FASTQ_otu.tsv	Root-Host_associated-Human-Skin
SRR996280	MGYA00023308	https://www.ebi.ac.uk/metagenomics/api/v1/analyses/MGYA00023308/file/SRR996280_FASTQ_otu.tsv	Root-Host_associated-Human-Skin
SRR1630999	MGYA00022847	https://www.ebi.ac.uk/metagenomics/api/v1/analyses/MGYA00022847/file/SRR1630999_MERGED_FASTQ_otu.tsv	Root-Host_associated-Human-Skin
SRR996955	MGYA00022871	https://www.ebi.ac.uk/metagenomics/api/v1/analyses/MGYA00022871/file/SRR996955_FASTQ_otu.tsv	Root-Host_associated-Human-Skin
SRR995395	MGYA00024756	https://www.ebi.ac.uk/metagenomics/api/v1/analyses/MGYA00024756/file/SRR995395_FASTQ_otu.tsv	Root-Host_associated-Human-Skin
SRR5869248	MGYA00415941	https://www.ebi.ac.uk/metagenomics/api/v1/analyses/MGYA00415941/file/SRR5869248_MERGED_FASTQ_SSU_OTU.tsv	Root-Host_associated-Human-Skin
SRR996146	MGYA00022529	https://www.ebi.ac.uk/metagenomics/api/v1/analyses/MGYA00022529/file/SRR996146_FASTQ_otu.tsv	Root-Host_associated-Human-Skin
SRR1622858	MGYA00024379	https://www.ebi.ac.uk/metagenomics/api/v1/analyses/MGYA00024379/file/SRR1622858_MERGED_FASTQ_otu.tsv	Root-Host_associated-Human-Skin
SRR994613	MGYA00023014	https://www.ebi.ac.uk/metagenomics/api/v1/analyses/MGYA00023014/file/SRR994613_FASTQ_otu.tsv	Root-Host_associated-Human-Skin
ERR1760043	MGYA00140353	https://www.ebi.ac.uk/metagenomics/api/v1/analyses/MGYA00140353/file/ERR1760043_MERGED_FASTQ_SSU_OTU.tsv	Root-Host_associated-Human-Skin
SRR921108	MGYA00024998	https://www.ebi.ac.uk/metagenomics/api/v1/analyses/MGYA00024998/file/SRR921108_FASTQ_otu.tsv	Root-Host_associated-Human-Skin

SRR995082	MGYA00022717	https://www.ebi.ac.uk/metagenomics/api/v1/analyses/MGYA00022717/file/SRR995082_FASTQ_otu.tsv	Root-Host_associated-Human-Skin
ERR1760092	MGYA00140606	https://www.ebi.ac.uk/metagenomics/api/v1/analyses/MGYA00140606/file/ERR1760092_MERGED_FASTQ_SSU_OTU.tsv	Root-Host_associated-Human-Skin
SRR920748	MGYA00023534	https://www.ebi.ac.uk/metagenomics/api/v1/analyses/MGYA00023534/file/SRR920748_FASTQ_otu.tsv	Root-Host_associated-Human-Skin
SRR997070	MGYA00023158	https://www.ebi.ac.uk/metagenomics/api/v1/analyses/MGYA00023158/file/SRR997070_FASTQ_otu.tsv	Root-Host_associated-Human-Skin
ERR443678	MGYA00415509	https://www.ebi.ac.uk/metagenomics/api/v1/analyses/MGYA00415509/file/ERR443678_FASTQ_SSU_OTU.tsv	Root-Host_associated-Human-Skin
SRR994504	MGYA00023820	https://www.ebi.ac.uk/metagenomics/api/v1/analyses/MGYA00023820/file/SRR994504_FASTQ_otu.tsv	Root-Host_associated-Human-Skin
SRR994976	MGYA00024556	https://www.ebi.ac.uk/metagenomics/api/v1/analyses/MGYA00024556/file/SRR994976_FASTQ_otu.tsv	Root-Host_associated-Human-Skin
SRR995344	MGYA00023118	https://www.ebi.ac.uk/metagenomics/api/v1/analyses/MGYA00023118/file/SRR995344_FASTQ_otu.tsv	Root-Host_associated-Human-Skin
SRR996984	MGYA00024533	https://www.ebi.ac.uk/metagenomics/api/v1/analyses/MGYA00024533/file/SRR996984_FASTQ_otu.tsv	Root-Host_associated-Human-Skin
ERR451257	MGYA00424893	https://www.ebi.ac.uk/metagenomics/api/v1/analyses/MGYA00424893/file/ERR451257_FASTQ_SSU_OTU.tsv	Root-Host_associated-Human-Skin
ERR443274	MGYA00423301	https://www.ebi.ac.uk/metagenomics/api/v1/analyses/MGYA00423301/file/ERR443274_FASTQ_SSU_OTU.tsv	Root-Host_associated-Human-Skin
SRR920725	MGYA00023747	https://www.ebi.ac.uk/metagenomics/api/v1/analyses/MGYA00023747/file/SRR920725_FASTQ_otu.tsv	Root-Host_associated-Human-Skin

ERR1701210	MGYA00084307	https://www.ebi.ac.uk/metagenomics/api/v1/analyses/MGYA00084307/file/ERR1701210_FASTQ_otu.tsv	Root-Host_associated-Human-Skin
ERR2538390	MGYA00381397	https://www.ebi.ac.uk/metagenomics/api/v1/analyses/MGYA00381397/file/ERR2538390_FASTQ_SSU_OTU.tsv	Root-Host_associated-Human-Skin
SRR5869553	MGYA00419650	https://www.ebi.ac.uk/metagenomics/api/v1/analyses/MGYA00419650/file/SRR5869553_MERGED_FASTQ_SSU_OTU.tsv	Root-Host_associated-Human-Skin
SRR5869921	MGYA00420706	https://www.ebi.ac.uk/metagenomics/api/v1/analyses/MGYA00420706/file/SRR5869921_MERGED_FASTQ_SSU_OTU.tsv	Root-Host_associated-Human-Skin
ERR2538437	MGYA00381444	https://www.ebi.ac.uk/metagenomics/api/v1/analyses/MGYA00381444/file/ERR2538437_FASTQ_SSU_OTU.tsv	Root-Host_associated-Human-Skin
ERR443615	MGYA00415117	https://www.ebi.ac.uk/metagenomics/api/v1/analyses/MGYA00415117/file/ERR443615_FASTQ_SSU_OTU.tsv	Root-Host_associated-Human-Skin
SRR994569	MGYA00024438	https://www.ebi.ac.uk/metagenomics/api/v1/analyses/MGYA00024438/file/SRR994569_FASTQ_otu.tsv	Root-Host_associated-Human-Skin
SRR1922567	MGYA00028305	https://www.ebi.ac.uk/metagenomics/api/v1/analyses/MGYA00028305/file/SRR1922567_FASTQ_otu.tsv	Root-Host_associated-Human-Skin
ERR1960807	MGYA00130801	https://www.ebi.ac.uk/metagenomics/api/v1/analyses/MGYA00130801/file/ERR1960807_MERGED_FASTQ_otu.tsv	Root-Host_associated-Human-Skin
SRR995329	MGYA00022484	https://www.ebi.ac.uk/metagenomics/api/v1/analyses/MGYA00022484/file/SRR995329_FASTQ_otu.tsv	Root-Host_associated-Human-Skin
SRR1704474	MGYA00417452	https://www.ebi.ac.uk/metagenomics/api/v1/analyses/MGYA00417452/file/SRR1704474_FASTQ_SSU_OTU.tsv	Root-Host_associated-Human-Skin
SRR5869644	MGYA00414835	https://www.ebi.ac.uk/metagenomics/api/v1/analyses/MGYA00414835/file/SRR5869644_MERGED_FASTQ_SSU_OTU.tsv	Root-Host_associated-Human-Skin

SRR2005661	MGYA00381319	https://www.ebi.ac.uk/metagenomics/api/v1/analyses/MGYA00381319/file/SRR2005661_MERGED_FASTQ_SSU_OTU.tsv	Root-Host_associated-Human-Skin
SRR996938	MGYA00022232	https://www.ebi.ac.uk/metagenomics/api/v1/analyses/MGYA00022232/file/SRR996938_FASTQ_otu.tsv	Root-Host_associated-Human-Skin
ERR450700	MGYA00413026	https://www.ebi.ac.uk/metagenomics/api/v1/analyses/MGYA00413026/file/ERR450700_FASTQ_SSU_OTU.tsv	Root-Host_associated-Human-Skin
SRR1622835	MGYA00023715	https://www.ebi.ac.uk/metagenomics/api/v1/analyses/MGYA00023715/file/SRR1622835_MERGED_FASTQ_otu.tsv	Root-Host_associated-Human-Skin
SRR5869582	MGYA00415919	https://www.ebi.ac.uk/metagenomics/api/v1/analyses/MGYA00415919/file/SRR5869582_MERGED_FASTQ_SSU_OTU.tsv	Root-Host_associated-Human-Skin
SRR6757616	MGYA00380734	https://www.ebi.ac.uk/metagenomics/api/v1/analyses/MGYA00380734/file/SRR6757616_MERGED_FASTQ_SSU_OTU.tsv	Root-Host_associated-Human-Skin
SRR5868817	MGYA00424495	https://www.ebi.ac.uk/metagenomics/api/v1/analyses/MGYA00424495/file/SRR5868817_MERGED_FASTQ_SSU_OTU.tsv	Root-Host_associated-Human-Skin
ERR1960762	MGYA00130830	https://www.ebi.ac.uk/metagenomics/api/v1/analyses/MGYA00130830/file/ERR1960762_MERGED_FASTQ_otu.tsv	Root-Host_associated-Human-Skin
SRR995507	MGYA00023171	https://www.ebi.ac.uk/metagenomics/api/v1/analyses/MGYA00023171/file/SRR995507_FASTQ_otu.tsv	Root-Host_associated-Human-Skin
SRR920918	MGYA00022350	https://www.ebi.ac.uk/metagenomics/api/v1/analyses/MGYA00022350/file/SRR920918_FASTQ_otu.tsv	Root-Host_associated-Human-Skin
SRR1631539	MGYA00023576	https://www.ebi.ac.uk/metagenomics/api/v1/analyses/MGYA00023576/file/SRR1631539_MERGED_FASTQ_otu.tsv	Root-Host_associated-Human-Skin
SRR996412	MGYA00024300	https://www.ebi.ac.uk/metagenomics/api/v1/analyses/MGYA00024300/file/SRR996412_FASTQ_otu.tsv	Root-Host_associated-Human-Skin

SRR995166	MGYA00024327	https://www.ebi.ac.uk/metagenomics/api/v1/analyses/MGYA00024327/file/SRR995166_FASTQ_otu.tsv	Root-Host_associated-Human-Skin
SRR996705	MGYA00022331	https://www.ebi.ac.uk/metagenomics/api/v1/analyses/MGYA00022331/file/SRR996705_FASTQ_otu.tsv	Root-Host_associated-Human-Skin
SRR996364	MGYA00022501	https://www.ebi.ac.uk/metagenomics/api/v1/analyses/MGYA00022501/file/SRR996364_FASTQ_otu.tsv	Root-Host_associated-Human-Skin
ERR1759968	MGYA00140388	https://www.ebi.ac.uk/metagenomics/api/v1/analyses/MGYA00140388/file/ERR1759968_MERGED_FASTQ_SSU_OTU.tsv	Root-Host_associated-Human-Skin
SRR1623048	MGYA00024636	https://www.ebi.ac.uk/metagenomics/api/v1/analyses/MGYA00024636/file/SRR1623048_MERGED_FASTQ_otu.tsv	Root-Host_associated-Human-Skin
SRR994843	MGYA00022612	https://www.ebi.ac.uk/metagenomics/api/v1/analyses/MGYA00022612/file/SRR994843_FASTQ_otu.tsv	Root-Host_associated-Human-Skin
ERR450689	MGYA00425290	https://www.ebi.ac.uk/metagenomics/api/v1/analyses/MGYA00425290/file/ERR450689_FASTQ_SSU_OTU.tsv	Root-Host_associated-Human-Skin
SRR995271	MGYA00025063	https://www.ebi.ac.uk/metagenomics/api/v1/analyses/MGYA00025063/file/SRR995271_FASTQ_otu.tsv	Root-Host_associated-Human-Skin
ERR442005	MGYA00385226	https://www.ebi.ac.uk/metagenomics/api/v1/analyses/MGYA00385226/file/ERR442005_FASTQ_SSU_OTU.tsv	Root-Host_associated-Human-Skin
SRR996186	MGYA00022955	https://www.ebi.ac.uk/metagenomics/api/v1/analyses/MGYA00022955/file/SRR996186_FASTQ_otu.tsv	Root-Host_associated-Human-Skin
SRR1622898	MGYA00024763	https://www.ebi.ac.uk/metagenomics/api/v1/analyses/MGYA00024763/file/SRR1622898_MERGED_FASTQ_otu.tsv	Root-Host_associated-Human-Skin
ERR981246	MGYA00010236	https://www.ebi.ac.uk/metagenomics/api/v1/analyses/MGYA00010236/file/ERR981246_MERGED_FASTQ_otu.tsv	Root-Host_associated-Human-Skin

ERR1760083	MGYA00140367	https://www.ebi.ac.uk/metagenomics/api/v1/analyses/MGYA00140367/file/ERR1760083_MERGED_FASTQ_SSU_OTU.tsv	Root-Host_associated-Human-Skin
SRR995734	MGYA00023097	https://www.ebi.ac.uk/metagenomics/api/v1/analyses/MGYA00023097/file/SRR995734_FASTQ_otu.tsv	Root-Host_associated-Human-Skin
SRR1630959	MGYA00024158	https://www.ebi.ac.uk/metagenomics/api/v1/analyses/MGYA00024158/file/SRR1630959_MERGED_FASTQ_otu.tsv	Root-Host_associated-Human-Skin
SRR996240	MGYA00023704	https://www.ebi.ac.uk/metagenomics/api/v1/analyses/MGYA00023704/file/SRR996240_FASTQ_otu.tsv	Root-Host_associated-Human-Skin
ERR443669	MGYA00418473	https://www.ebi.ac.uk/metagenomics/api/v1/analyses/MGYA00418473/file/ERR443669_FASTQ_SSU_OTU.tsv	Root-Host_associated-Human-Skin
SRR996536	MGYA00022837	https://www.ebi.ac.uk/metagenomics/api/v1/analyses/MGYA00022837/file/SRR996536_FASTQ_otu.tsv	Root-Host_associated-Human-Skin
ERR1760052	MGYA00140477	https://www.ebi.ac.uk/metagenomics/api/v1/analyses/MGYA00140477/file/ERR1760052_MERGED_FASTQ_SSU_OTU.tsv	Root-Host_associated-Human-Skin
SRR995042	MGYA00023697	https://www.ebi.ac.uk/metagenomics/api/v1/analyses/MGYA00023697/file/SRR995042_FASTQ_otu.tsv	Root-Host_associated-Human-Skin
SRR5868687	MGYA00428060	https://www.ebi.ac.uk/metagenomics/api/v1/analyses/MGYA00428060/file/SRR5868687_MERGED_FASTQ_SSU_OTU.tsv	Root-Host_associated-Human-Skin
SRR1704581	MGYA00414631	https://www.ebi.ac.uk/metagenomics/api/v1/analyses/MGYA00414631/file/SRR1704581_FASTQ_SSU_OTU.tsv	Root-Host_associated-Human-Skin
SRR1704226	MGYA00412728	https://www.ebi.ac.uk/metagenomics/api/v1/analyses/MGYA00412728/file/SRR1704226_FASTQ_SSU_OTU.tsv	Root-Host_associated-Human-Skin
SRR995909	MGYA00023845	https://www.ebi.ac.uk/metagenomics/api/v1/analyses/MGYA00023845/file/SRR995909_FASTQ_otu.tsv	Root-Host_associated-Human-Skin

ERR451064	MGYA00413005	https://www.ebi.ac.uk/metagenomics/api/v1/analyses/MGYA00413005/file/ERR451064_FASTQ_SSU_OTU.tsv	Root-Host_associated-Human-Skin
SRR1923907	MGYA00028329	https://www.ebi.ac.uk/metagenomics/api/v1/analyses/MGYA00028329/file/SRR1923907_FASTQ_otu.tsv	Root-Host_associated-Human-Skin
SRR1632450	MGYA00023812	https://www.ebi.ac.uk/metagenomics/api/v1/analyses/MGYA00023812/file/SRR1632450_MERGED_FASTQ_otu.tsv	Root-Host_associated-Human-Skin
SRR1622911	MGYA00025087	https://www.ebi.ac.uk/metagenomics/api/v1/analyses/MGYA00025087/file/SRR1622911_MERGED_FASTQ_otu.tsv	Root-Host_associated-Human-Skin
SRR1631683	MGYA00024827	https://www.ebi.ac.uk/metagenomics/api/v1/analyses/MGYA00024827/file/SRR1631683_MERGED_FASTQ_otu.tsv	Root-Host_associated-Human-Skin
SRR920177	MGYA00024712	https://www.ebi.ac.uk/metagenomics/api/v1/analyses/MGYA00024712/file/SRR920177_FASTQ_otu.tsv	Root-Host_associated-Human-Skin
SRR1632796	MGYA00023372	https://www.ebi.ac.uk/metagenomics/api/v1/analyses/MGYA00023372/file/SRR1632796_MERGED_FASTQ_otu.tsv	Root-Host_associated-Human-Skin
ERR1960646	MGYA00130893	https://www.ebi.ac.uk/metagenomics/api/v1/analyses/MGYA00130893/file/ERR1960646_MERGED_FASTQ_otu.tsv	Root-Host_associated-Human-Skin
ERR451405	MGYA00428720	https://www.ebi.ac.uk/metagenomics/api/v1/analyses/MGYA00428720/file/ERR451405_FASTQ_SSU_OTU.tsv	Root-Host_associated-Human-Skin
SRR921090	MGYA00022426	https://www.ebi.ac.uk/metagenomics/api/v1/analyses/MGYA00022426/file/SRR921090_FASTQ_otu.tsv	Root-Host_associated-Human-Skin
ERR451373	MGYA00426012	https://www.ebi.ac.uk/metagenomics/api/v1/analyses/MGYA00426012/file/ERR451373_FASTQ_SSU_OTU.tsv	Root-Host_associated-Human-Skin
ERR442962	MGYA00412394	https://www.ebi.ac.uk/metagenomics/api/v1/analyses/MGYA00412394/file/ERR442962_FASTQ_SSU_OTU.tsv	Root-Host_associated-Human-Skin

SRR920601	MGYA00022564	https://www.ebi.ac.uk/metagenomics/api/v1/analyses/MGYA00022564/file/SRR920601_FASTQ_otu.tsv	Root-Host_associated-Human-Skin
ERR1960697	MGYA00130733	https://www.ebi.ac.uk/metagenomics/api/v1/analyses/MGYA00130733/file/ERR1960697_MERGED_FASTQ_otu.tsv	Root-Host_associated-Human-Skin
ERR2538513	MGYA00381520	https://www.ebi.ac.uk/metagenomics/api/v1/analyses/MGYA00381520/file/ERR2538513_FASTQ_SSU_OTU.tsv	Root-Host_associated-Human-Skin
SRR1632326	MGYA00023477	https://www.ebi.ac.uk/metagenomics/api/v1/analyses/MGYA00023477/file/SRR1632326_MERGED_FASTQ_otu.tsv	Root-Host_associated-Human-Skin
SRR1632481	MGYA00022678	https://www.ebi.ac.uk/metagenomics/api/v1/analyses/MGYA00022678/file/SRR1632481_MERGED_FASTQ_otu.tsv	Root-Host_associated-Human-Skin
SRR920260	MGYA00023687	https://www.ebi.ac.uk/metagenomics/api/v1/analyses/MGYA00023687/file/SRR920260_FASTQ_otu.tsv	Root-Host_associated-Human-Skin
SRR1950742	MGYA00376909	https://www.ebi.ac.uk/metagenomics/api/v1/analyses/MGYA00376909/file/SRR1950742_MERGED_FASTQ_SSU_OTU.tsv	Root-Host_associated-Human-Skin
SRR5868656	MGYA00416067	https://www.ebi.ac.uk/metagenomics/api/v1/analyses/MGYA00416067/file/SRR5868656_MERGED_FASTQ_SSU_OTU.tsv	Root-Host_associated-Human-Skin
ERR1759914	MGYA00140527	https://www.ebi.ac.uk/metagenomics/api/v1/analyses/MGYA00140527/file/ERR1759914_MERGED_FASTQ_SSU_OTU.tsv	Root-Host_associated-Human-Skin
SRR996181	MGYA00022960	https://www.ebi.ac.uk/metagenomics/api/v1/analyses/MGYA00022960/file/SRR996181_FASTQ_otu.tsv	Root-Host_associated-Human-Skin
SRR5869528	MGYA00414856	https://www.ebi.ac.uk/metagenomics/api/v1/analyses/MGYA00414856/file/SRR5869528_MERGED_FASTQ_SSU_OTU.tsv	Root-Host_associated-Human-Skin
SRR995733	MGYA00023102	https://www.ebi.ac.uk/metagenomics/api/v1/analyses/MGYA00023102/file/SRR995733_FASTQ_otu.tsv	Root-Host_associated-Human-Skin

SRR995094	MGYA00023390	https://www.ebi.ac.uk/metagenomics/api/v1/analyses/MGYA00023390/file/SRR995094_FASTQ_otu.tsv	Root-Host_associated-Human-Skin
ERR1760084	MGYA00140364	https://www.ebi.ac.uk/metagenomics/api/v1/analyses/MGYA00140364/file/ERR1760084_MERGED_FASTQ_SSU_OTU.tsv	Root-Host_associated-Human-Skin
SRR996247	MGYA00023711	https://www.ebi.ac.uk/metagenomics/api/v1/analyses/MGYA00023711/file/SRR996247_FASTQ_otu.tsv	Root-Host_associated-Human-Skin
SRR1923889	MGYA00028336	https://www.ebi.ac.uk/metagenomics/api/v1/analyses/MGYA00028336/file/SRR1923889_FASTQ_otu.tsv	Root-Host_associated-Human-Skin
SRR1923858	MGYA00028289	https://www.ebi.ac.uk/metagenomics/api/v1/analyses/MGYA00028289/file/SRR1923858_FASTQ_otu.tsv	Root-Host_associated-Human-Skin
SRR996531	MGYA00022844	https://www.ebi.ac.uk/metagenomics/api/v1/analyses/MGYA00022844/file/SRR996531_FASTQ_otu.tsv	Root-Host_associated-Human-Skin
SRR5869198	MGYA00415967	https://www.ebi.ac.uk/metagenomics/api/v1/analyses/MGYA00415967/file/SRR5869198_MERGED_FASTQ_SSU_OTU.tsv	Root-Host_associated-Human-Skin
ERR1760055	MGYA00140368	https://www.ebi.ac.uk/metagenomics/api/v1/analyses/MGYA00140368/file/ERR1760055_MERGED_FASTQ_SSU_OTU.tsv	Root-Host_associated-Human-Skin
SRR995045	MGYA00023694	https://www.ebi.ac.uk/metagenomics/api/v1/analyses/MGYA00023694/file/SRR995045_FASTQ_otu.tsv	Root-Host_associated-Human-Skin
ERR1960719	MGYA00130726	https://www.ebi.ac.uk/metagenomics/api/v1/analyses/MGYA00130726/file/ERR1960719_MERGED_FASTQ_otu.tsv	Root-Host_associated-Human-Skin
ERR443579	MGYA00414259	https://www.ebi.ac.uk/metagenomics/api/v1/analyses/MGYA00414259/file/ERR443579_FASTQ_SSU_OTU.tsv	Root-Host_associated-Human-Skin
SRR1924040	MGYA00028328	https://www.ebi.ac.uk/metagenomics/api/v1/analyses/MGYA00028328/file/SRR1924040_FASTQ_otu.tsv	Root-Host_associated-Human-Skin

SRR1704586	MGYA00426150	https://www.ebi.ac.uk/metagenomics/api/v1/analyses/MGYA00426150/file/SRR1704586_FASTQ_SSU_OTU.tsv	Root-Host_associated-Human-Skin
ERR443040	MGYA00415178	https://www.ebi.ac.uk/metagenomics/api/v1/analyses/MGYA00415178/file/ERR443040_FASTQ_SSU_OTU.tsv	Root-Host_associated-Human-Skin
SRR1923900	MGYA00028282	https://www.ebi.ac.uk/metagenomics/api/v1/analyses/MGYA00028282/file/SRR1923900_FASTQ_otu.tsv	Root-Host_associated-Human-Skin
SRR1621010	MGYA00024796	https://www.ebi.ac.uk/metagenomics/api/v1/analyses/MGYA00024796/file/SRR1621010_MERGED_FASTQ_otu.tsv	Root-Host_associated-Human-Skin
SRR1631684	MGYA00024832	https://www.ebi.ac.uk/metagenomics/api/v1/analyses/MGYA00024832/file/SRR1631684_MERGED_FASTQ_otu.tsv	Root-Host_associated-Human-Skin
SRR5869306	MGYA00419705	https://www.ebi.ac.uk/metagenomics/api/v1/analyses/MGYA00419705/file/SRR5869306_MERGED_FASTQ_SSU_OTU.tsv	Root-Host_associated-Human-Skin
SRR1622916	MGYA00025080	https://www.ebi.ac.uk/metagenomics/api/v1/analyses/MGYA00025080/file/SRR1622916_MERGED_FASTQ_otu.tsv	Root-Host_associated-Human-Skin
SRR996008	MGYA00023521	https://www.ebi.ac.uk/metagenomics/api/v1/analyses/MGYA00023521/file/SRR996008_FASTQ_otu.tsv	Root-Host_associated-Human-Skin
SRR921097	MGYA00022427	https://www.ebi.ac.uk/metagenomics/api/v1/analyses/MGYA00022427/file/SRR921097_FASTQ_otu.tsv	Root-Host_associated-Human-Skin
ERR1960641	MGYA00130753	https://www.ebi.ac.uk/metagenomics/api/v1/analyses/MGYA00130753/file/ERR1960641_MERGED_FASTQ_otu.tsv	Root-Host_associated-Human-Skin
SRR920170	MGYA00024711	https://www.ebi.ac.uk/metagenomics/api/v1/analyses/MGYA00024711/file/SRR920170_FASTQ_otu.tsv	Root-Host_associated-Human-Skin
SRR1632791	MGYA00023373	https://www.ebi.ac.uk/metagenomics/api/v1/analyses/MGYA00023373/file/SRR1632791_MERGED_FASTQ_otu.tsv	Root-Host_associated-Human-Skin

SRR920606	MGYA00022563	https://www.ebi.ac.uk/metagenomics/api/v1/analyses/MGYA00022563/file/SRR920606_FASTQ_otu.tsv	Root-Host_associated-Human-Skin
ERR1960690	MGYA00130803	https://www.ebi.ac.uk/metagenomics/api/v1/analyses/MGYA00130803/file/ERR1960690_MERGED_FASTQ_otu.tsv	Root-Host_associated-Human-Skin
ERR442965	MGYA00416701	https://www.ebi.ac.uk/metagenomics/api/v1/analyses/MGYA00416701/file/ERR442965_FASTQ_SSU_OTU.tsv	Root-Host_associated-Human-Skin
SRR2005455	MGYA00381292	https://www.ebi.ac.uk/metagenomics/api/v1/analyses/MGYA00381292/file/SRR2005455_MERGED_FASTQ_SSU_OTU.tsv	Root-Host_associated-Human-Skin
ERR2538514	MGYA00381521	https://www.ebi.ac.uk/metagenomics/api/v1/analyses/MGYA00381521/file/ERR2538514_FASTQ_SSU_OTU.tsv	Root-Host_associated-Human-Skin
ERR441978	MGYA00385172	https://www.ebi.ac.uk/metagenomics/api/v1/analyses/MGYA00385172/file/ERR441978_FASTQ_SSU_OTU.tsv	Root-Host_associated-Human-Skin
SRR1704136	MGYA00413115	https://www.ebi.ac.uk/metagenomics/api/v1/analyses/MGYA00413115/file/SRR1704136_FASTQ_SSU_OTU.tsv	Root-Host_associated-Human-Skin
SRR1632486	MGYA00022677	https://www.ebi.ac.uk/metagenomics/api/v1/analyses/MGYA00022677/file/SRR1632486_MERGED_FASTQ_otu.tsv	Root-Host_associated-Human-Skin
SRR1632321	MGYA00023480	https://www.ebi.ac.uk/metagenomics/api/v1/analyses/MGYA00023480/file/SRR1632321_MERGED_FASTQ_otu.tsv	Root-Host_associated-Human-Skin
ERR1759913	MGYA00140390	https://www.ebi.ac.uk/metagenomics/api/v1/analyses/MGYA00140390/file/ERR1759913_MERGED_FASTQ_SSU_OTU.tsv	Root-Host_associated-Human-Skin
SRR5868651	MGYA00417151	https://www.ebi.ac.uk/metagenomics/api/v1/analyses/MGYA00417151/file/SRR5868651_MERGED_FASTQ_SSU_OTU.tsv	Root-Host_associated-Human-Skin
SRR1950745	MGYA00376912	https://www.ebi.ac.uk/metagenomics/api/v1/analyses/MGYA00376912/file/SRR1950745_MERGED_FASTQ_SSU_OTU.tsv	Root-Host_associated-Human-Skin

ERR1701217	MGYA00084328	https://www.ebi.ac.uk/metagenomics/api/v1/analyses/MGYA00084328/file/ERR1701217_FASTQ_otu.tsv	Root-Host_associated-Human-Skin
ERR2538430	MGYA00381437	https://www.ebi.ac.uk/metagenomics/api/v1/analyses/MGYA00381437/file/ERR2538430_FASTQ_SSU_OTU.tsv	Root-Host_associated-Human-Skin
ERR2538397	MGYA00381404	https://www.ebi.ac.uk/metagenomics/api/v1/analyses/MGYA00381404/file/ERR2538397_FASTQ_SSU_OTU.tsv	Root-Host_associated-Human-Skin
ERR451196	MGYA00415162	https://www.ebi.ac.uk/metagenomics/api/v1/analyses/MGYA00415162/file/ERR451196_FASTQ_SSU_OTU.tsv	Root-Host_associated-Human-Skin
ERR1960800	MGYA00130781	https://www.ebi.ac.uk/metagenomics/api/v1/analyses/MGYA00130781/file/ERR1960800_MERGED_FASTQ_otu.tsv	Root-Host_associated-Human-Skin
ERR443612	MGYA00416637	https://www.ebi.ac.uk/metagenomics/api/v1/analyses/MGYA00416637/file/ERR443612_FASTQ_SSU_OTU.tsv	Root-Host_associated-Human-Skin
SRR5868775	MGYA00416057	https://www.ebi.ac.uk/metagenomics/api/v1/analyses/MGYA00416057/file/SRR5868775_MERGED_FASTQ_SSU_OTU.tsv	Root-Host_associated-Human-Skin
SRR2005666	MGYA00381324	https://www.ebi.ac.uk/metagenomics/api/v1/analyses/MGYA00381324/file/SRR2005666_MERGED_FASTQ_SSU_OTU.tsv	Root-Host_associated-Human-Skin
SRR5869643	MGYA00415903	https://www.ebi.ac.uk/metagenomics/api/v1/analyses/MGYA00415903/file/SRR5869643_MERGED_FASTQ_SSU_OTU.tsv	Root-Host_associated-Human-Skin
SRR1631466	MGYA00024982	https://www.ebi.ac.uk/metagenomics/api/v1/analyses/MGYA00024982/file/SRR1631466_MERGED_FASTQ_otu.tsv	Root-Host_associated-Human-Skin
SRR995458	MGYA00025313	https://www.ebi.ac.uk/metagenomics/api/v1/analyses/MGYA00025313/file/SRR995458_FASTQ_otu.tsv	Root-Host_associated-Human-Skin
SRR6757611	MGYA00380729	https://www.ebi.ac.uk/metagenomics/api/v1/analyses/MGYA00380729/file/SRR6757611_MERGED_FASTQ_SSU_OTU.tsv	Root-Host_associated-Human-Skin

SRR1631007	MGYA00024598	https://www.ebi.ac.uk/metagenomics/api/v1/analyses/MGYA00024598/file/SRR1631007_MERGED_FASTQ_otu.tsv	Root-Host_associated-Human-Skin
SRR5869585	MGYA00417532	https://www.ebi.ac.uk/metagenomics/api/v1/analyses/MGYA00417532/file/SRR5869585_MERGED_FASTQ_SSU_OTU.tsv	Root-Host_associated-Human-Skin
ERR1760029	MGYA00140401	https://www.ebi.ac.uk/metagenomics/api/v1/analyses/MGYA00140401/file/ERR1760029_MERGED_FASTQ_SSU_OTU.tsv	Root-Host_associated-Human-Skin
ERR451526	MGYA00416249	https://www.ebi.ac.uk/metagenomics/api/v1/analyses/MGYA00416249/file/ERR451526_FASTQ_SSU_OTU.tsv	Root-Host_associated-Human-Skin
SRR994679	MGYA00023634	https://www.ebi.ac.uk/metagenomics/api/v1/analyses/MGYA00023634/file/SRR994679_FASTQ_otu.tsv	Root-Host_associated-Human-Skin
ERR1960765	MGYA00130777	https://www.ebi.ac.uk/metagenomics/api/v1/analyses/MGYA00130777/file/ERR1960765_MERGED_FASTQ_otu.tsv	Root-Host_associated-Human-Skin
SRR1632859	MGYA00023045	https://www.ebi.ac.uk/metagenomics/api/v1/analyses/MGYA00023045/file/SRR1632859_MERGED_FASTQ_otu.tsv	Root-Host_associated-Human-Skin
SRR995500	MGYA00023176	https://www.ebi.ac.uk/metagenomics/api/v1/analyses/MGYA00023176/file/SRR995500_FASTQ_otu.tsv	Root-Host_associated-Human-Skin
SRR996415	MGYA00024301	https://www.ebi.ac.uk/metagenomics/api/v1/analyses/MGYA00024301/file/SRR996415_FASTQ_otu.tsv	Root-Host_associated-Human-Skin
SRR996867	MGYA00024914	https://www.ebi.ac.uk/metagenomics/api/v1/analyses/MGYA00024914/file/SRR996867_FASTQ_otu.tsv	Root-Host_associated-Human-Skin
SRR995161	MGYA00024330	https://www.ebi.ac.uk/metagenomics/api/v1/analyses/MGYA00024330/file/SRR995161_FASTQ_otu.tsv	Root-Host_associated-Human-Skin
SRR996702	MGYA00022330	https://www.ebi.ac.uk/metagenomics/api/v1/analyses/MGYA00022330/file/SRR996702_FASTQ_otu.tsv	Root-Host_associated-Human-Skin

ERR1551327	MGYA00069428	https://www.ebi.ac.uk/metagenomics/api/v1/analyses/MGYA00069428/file/ERR1551327_MERGED_FASTQ_otu.tsv	Root-Host_associated-Human-Skin
SRR1950739	MGYA00376906	https://www.ebi.ac.uk/metagenomics/api/v1/analyses/MGYA00376906/file/SRR1950739_MERGED_FASTQ_SSU_OTU.tsv	Root-Host_associated-Human-Skin
SRR996363	MGYA00022502	https://www.ebi.ac.uk/metagenomics/api/v1/analyses/MGYA00022502/file/SRR996363_FASTQ_otu.tsv	Root-Host_associated-Human-Skin
ERR442002	MGYA00385181	https://www.ebi.ac.uk/metagenomics/api/v1/analyses/MGYA00385181/file/ERR442002_FASTQ_SSU_OTU.tsv	Root-Host_associated-Human-Skin
SRR995276	MGYA00025060	https://www.ebi.ac.uk/metagenomics/api/v1/analyses/MGYA00025060/file/SRR995276_FASTQ_otu.tsv	Root-Host_associated-Human-Skin
SRR994844	MGYA00022613	https://www.ebi.ac.uk/metagenomics/api/v1/analyses/MGYA00022613/file/SRR994844_FASTQ_otu.tsv	Root-Host_associated-Human-Skin
SRR5870206	MGYA00426198	https://www.ebi.ac.uk/metagenomics/api/v1/analyses/MGYA00426198/file/SRR5870206_MERGED_FASTQ_SSU_OTU.tsv	Root-Host_associated-Human-Skin
ERR443201	MGYA00414544	https://www.ebi.ac.uk/metagenomics/api/v1/analyses/MGYA00414544/file/ERR443201_FASTQ_SSU_OTU.tsv	Root-Host_associated-Human-Skin
ERR1701265	MGYA00084261	https://www.ebi.ac.uk/metagenomics/api/v1/analyses/MGYA00084261/file/ERR1701265_FASTQ_otu.tsv	Root-Host_associated-Human-Skin
SRR5869954	MGYA00414818	https://www.ebi.ac.uk/metagenomics/api/v1/analyses/MGYA00414818/file/SRR5869954_MERGED_FASTQ_SSU_OTU.tsv	Root-Host_associated-Human-Skin
SRR1622891	MGYA00024766	https://www.ebi.ac.uk/metagenomics/api/v1/analyses/MGYA00024766/file/SRR1622891_MERGED_FASTQ_otu.tsv	Root-Host_associated-Human-Skin
ERR2538442	MGYA00381449	https://www.ebi.ac.uk/metagenomics/api/v1/analyses/MGYA00381449/file/ERR2538442_FASTQ_SSU_OTU.tsv	Root-Host_associated-Human-Skin

SRR1923887	MGYA00028344	https://www.ebi.ac.uk/metagenomics/api/v1/analyses/MGYA00028344/file/SRR1923887_FASTQ_otu.tsv	Root-Host_associated-Human-Skin
SRR996249	MGYA00023703	https://www.ebi.ac.uk/metagenomics/api/v1/analyses/MGYA00023703/file/SRR996249_FASTQ_otu.tsv	Root-Host_associated-Human-Skin
SRR1630950	MGYA00024165	https://www.ebi.ac.uk/metagenomics/api/v1/analyses/MGYA00024165/file/SRR1630950_MERGED_FASTQ_otu.tsv	Root-Host_associated-Human-Skin
SRR1623165	MGYA00023488	https://www.ebi.ac.uk/metagenomics/api/v1/analyses/MGYA00023488/file/SRR1623165_MERGED_FASTQ_otu.tsv	Root-Host_associated-Human-Skin
SRR1631362	MGYA00022585	https://www.ebi.ac.uk/metagenomics/api/v1/analyses/MGYA00022585/file/SRR1631362_MERGED_FASTQ_otu.tsv	Root-Host_associated-Human-Skin
SRR1704377	MGYA00426950	https://www.ebi.ac.uk/metagenomics/api/v1/analyses/MGYA00426950/file/SRR1704377_FASTQ_SSU_OTU.tsv	Root-Host_associated-Human-Skin
ERR1759894	MGYA00140572	https://www.ebi.ac.uk/metagenomics/api/v1/analyses/MGYA00140572/file/ERR1759894_MERGED_FASTQ_SSU_OTU.tsv	Root-Host_associated-Human-Skin
SRR1923856	MGYA00028292	https://www.ebi.ac.uk/metagenomics/api/v1/analyses/MGYA00028292/file/SRR1923856_FASTQ_otu.tsv	Root-Host_associated-Human-Skin
ERR451135	MGYA00412632	https://www.ebi.ac.uk/metagenomics/api/v1/analyses/MGYA00412632/file/ERR451135_FASTQ_SSU_OTU.tsv	Root-Host_associated-Human-Skin
ERR2538493	MGYA00381500	https://www.ebi.ac.uk/metagenomics/api/v1/analyses/MGYA00381500/file/ERR2538493_FASTQ_SSU_OTU.tsv	Root-Host_associated-Human-Skin
SRR1704716	MGYA00424366	https://www.ebi.ac.uk/metagenomics/api/v1/analyses/MGYA00424366/file/SRR1704716_FASTQ_SSU_OTU.tsv	Root-Host_associated-Human-Skin
SRR5868862	MGYA00420730	https://www.ebi.ac.uk/metagenomics/api/v1/analyses/MGYA00420730/file/SRR5868862_MERGED_FASTQ_SSU_OTU.tsv	Root-Host_associated-Human-Skin

SRR6757663	MGYA00380776	https://www.ebi.ac.uk/metagenomics/api/v1/analyses/MGYA00380776/file/SRR6757663_MERGED_FASTQ_SSU_OTU.tsv	Root-Host_associated-Human-Skin
ERR1960717	MGYA00130886	https://www.ebi.ac.uk/metagenomics/api/v1/analyses/MGYA00130886/file/ERR1960717_MERGED_FASTQ_otu.tsv	Root-Host_associated-Human-Skin
ERR443577	MGYA00425758	https://www.ebi.ac.uk/metagenomics/api/v1/analyses/MGYA00425758/file/ERR443577_FASTQ_SSU_OTU.tsv	Root-Host_associated-Human-Skin
SRR995900	MGYA00023838	https://www.ebi.ac.uk/metagenomics/api/v1/analyses/MGYA00023838/file/SRR995900_FASTQ_otu.tsv	Root-Host_associated-Human-Skin
SRR996815	MGYA00023626	https://www.ebi.ac.uk/metagenomics/api/v1/analyses/MGYA00023626/file/SRR996815_FASTQ_otu.tsv	Root-Host_associated-Human-Skin
ERR443388	MGYA00422946	https://www.ebi.ac.uk/metagenomics/api/v1/analyses/MGYA00422946/file/ERR443388_FASTQ_SSU_OTU.tsv	Root-Host_associated-Human-Skin
SRR994753	MGYA00023244	https://www.ebi.ac.uk/metagenomics/api/v1/analyses/MGYA00023244/file/SRR994753_FASTQ_otu.tsv	Root-Host_associated-Human-Skin
SRR921099	MGYA00022421	https://www.ebi.ac.uk/metagenomics/api/v1/analyses/MGYA00022421/file/SRR921099_FASTQ_otu.tsv	Root-Host_associated-Human-Skin
ERR1760103	MGYA00140470	https://www.ebi.ac.uk/metagenomics/api/v1/analyses/MGYA00140470/file/ERR1760103_MERGED_FASTQ_SSU_OTU.tsv	Root-Host_associated-Human-Skin
SRR996006	MGYA00023513	https://www.ebi.ac.uk/metagenomics/api/v1/analyses/MGYA00023513/file/SRR996006_FASTQ_otu.tsv	Root-Host_associated-Human-Skin
SRR1622918	MGYA00025090	https://www.ebi.ac.uk/metagenomics/api/v1/analyses/MGYA00025090/file/SRR1622918_MERGED_FASTQ_otu.tsv	Root-Host_associated-Human-Skin
SRR5868599	MGYA00428119	https://www.ebi.ac.uk/metagenomics/api/v1/analyses/MGYA00428119/file/SRR5868599_MERGED_FASTQ_SSU_OTU.tsv	Root-Host_associated-Human-Skin

ERR441976	MGYA00385192	https://www.ebi.ac.uk/metagenomics/api/v1/analyses/MGYA00385192/file/ERR441976_FASTQ_SSU_OTU.tsv	Root-Host_associated-Human-Skin
SRR995665	MGYA00024844	https://www.ebi.ac.uk/metagenomics/api/v1/analyses/MGYA00024844/file/SRR995665_FASTQ_otu.tsv	Root-Host_associated-Human-Skin
SRR1624825	MGYA00024888	https://www.ebi.ac.uk/metagenomics/api/v1/analyses/MGYA00024888/file/SRR1624825_MERGED_FASTQ_otu.tsv	Root-Host_associated-Human-Skin
SRR920608	MGYA00022559	https://www.ebi.ac.uk/metagenomics/api/v1/analyses/MGYA00022559/file/SRR920608_FASTQ_otu.tsv	Root-Host_associated-Human-Skin
ERR450948	MGYA00426796	https://www.ebi.ac.uk/metagenomics/api/v1/analyses/MGYA00426796/file/ERR450948_FASTQ_SSU_OTU.tsv	Root-Host_associated-Human-Skin
SRR1632488	MGYA00022683	https://www.ebi.ac.uk/metagenomics/api/v1/analyses/MGYA00022683/file/SRR1632488_MERGED_FASTQ_otu.tsv	Root-Host_associated-Human-Skin
SRR997014	MGYA00023412	https://www.ebi.ac.uk/metagenomics/api/v1/analyses/MGYA00023412/file/SRR997014_FASTQ_otu.tsv	Root-Host_associated-Human-Skin
SRR5869928	MGYA00413953	https://www.ebi.ac.uk/metagenomics/api/v1/analyses/MGYA00413953/file/SRR5869928_MERGED_FASTQ_SSU_OTU.tsv	Root-Host_associated-Human-Skin
ERR2538399	MGYA00381406	https://www.ebi.ac.uk/metagenomics/api/v1/analyses/MGYA00381406/file/ERR2538399_FASTQ_SSU_OTU.tsv	Root-Host_associated-Human-Skin
ERR1701219	MGYA00084259	https://www.ebi.ac.uk/metagenomics/api/v1/analyses/MGYA00084259/file/ERR1701219_FASTQ_otu.tsv	Root-Host_associated-Human-Skin
SRR996592	MGYA00023995	https://www.ebi.ac.uk/metagenomics/api/v1/analyses/MGYA00023995/file/SRR996592_FASTQ_otu.tsv	Root-Host_associated-Human-Skin
SRR994560	MGYA00024433	https://www.ebi.ac.uk/metagenomics/api/v1/analyses/MGYA00024433/file/SRR994560_FASTQ_otu.tsv	Root-Host_associated-Human-Skin

SRR994912	MGYA00024748	https://www.ebi.ac.uk/metagenomics/api/v1/analyses/MGYA00024748/file/SRR994912_FASTQ_otu.tsv	Root-Host_associated-Human-Skin
SRR995320	MGYA00022477	https://www.ebi.ac.uk/metagenomics/api/v1/analyses/MGYA00022477/file/SRR995320_FASTQ_otu.tsv	Root-Host_associated-Human-Skin
SRR995456	MGYA00025307	https://www.ebi.ac.uk/metagenomics/api/v1/analyses/MGYA00025307/file/SRR995456_FASTQ_otu.tsv	Root-Host_associated-Human-Skin
ERR450709	MGYA00422961	https://www.ebi.ac.uk/metagenomics/api/v1/analyses/MGYA00422961/file/ERR450709_FASTQ_SSU_OTU.tsv	Root-Host_associated-Human-Skin
SRR996931	MGYA00022225	https://www.ebi.ac.uk/metagenomics/api/v1/analyses/MGYA00022225/file/SRR996931_FASTQ_otu.tsv	Root-Host_associated-Human-Skin
SRR994677	MGYA00023640	https://www.ebi.ac.uk/metagenomics/api/v1/analyses/MGYA00023640/file/SRR994677_FASTQ_otu.tsv	Root-Host_associated-Human-Skin
ERR1760027	MGYA00140485	https://www.ebi.ac.uk/metagenomics/api/v1/analyses/MGYA00140485/file/ERR1760027_MERGED_FASTQ_SSU_OTU.tsv	Root-Host_associated-Human-Skin
SRR1631009	MGYA00024604	https://www.ebi.ac.uk/metagenomics/api/v1/analyses/MGYA00024604/file/SRR1631009_MERGED_FASTQ_otu.tsv	Root-Host_associated-Human-Skin
SRR1631530	MGYA00023582	https://www.ebi.ac.uk/metagenomics/api/v1/analyses/MGYA00023582/file/SRR1631530_MERGED_FASTQ_otu.tsv	Root-Host_associated-Human-Skin
SRR5869715	MGYA00416974	https://www.ebi.ac.uk/metagenomics/api/v1/analyses/MGYA00416974/file/SRR5869715_MERGED_FASTQ_SSU_OTU.tsv	Root-Host_associated-Human-Skin
SRR1632382	MGYA00022370	https://www.ebi.ac.uk/metagenomics/api/v1/analyses/MGYA00022370/file/SRR1632382_MERGED_FASTQ_otu.tsv	Root-Host_associated-Human-Skin
ERR443032	MGYA00423310	https://www.ebi.ac.uk/metagenomics/api/v1/analyses/MGYA00423310/file/ERR443032_FASTQ_SSU_OTU.tsv	Root-Host_associated-Human-Skin

SRR920911	MGYA00022354	https://www.ebi.ac.uk/metagenomics/api/v1/analyses/MGYA00022354/file/SRR920911_FASTQ_otu.tsv	Root-Host_associated-Human-Skin
SRR1632857	MGYA00023047	https://www.ebi.ac.uk/metagenomics/api/v1/analyses/MGYA00023047/file/SRR1632857_MERGED_FASTQ_otu.tsv	Root-Host_associated-Human-Skin
SRR1632425	MGYA00023754	https://www.ebi.ac.uk/metagenomics/api/v1/analyses/MGYA00023754/file/SRR1632425_MERGED_FASTQ_otu.tsv	Root-Host_associated-Human-Skin
SRR1704632	MGYA00413811	https://www.ebi.ac.uk/metagenomics/api/v1/analyses/MGYA00413811/file/SRR1704632_FASTQ_SSU_OTU.tsv	Root-Host_associated-Human-Skin
ERR1960633	MGYA00130765	https://www.ebi.ac.uk/metagenomics/api/v1/analyses/MGYA00130765/file/ERR1960633_MERGED_FASTQ_otu.tsv	Root-Host_associated-Human-Skin
SRR1633172	MGYA00025260	https://www.ebi.ac.uk/metagenomics/api/v1/analyses/MGYA00025260/file/SRR1633172_MERGED_FASTQ_otu.tsv	Root-Host_associated-Human-Skin
SRR921034	MGYA00023079	https://www.ebi.ac.uk/metagenomics/api/v1/analyses/MGYA00023079/file/SRR921034_FASTQ_otu.tsv	Root-Host_associated-Human-Skin
ERR1551329	MGYA00069432	https://www.ebi.ac.uk/metagenomics/api/v1/analyses/MGYA00069432/file/ERR1551329_MERGED_FASTQ_otu.tsv	Root-Host_associated-Human-Skin
SRR2005380	MGYA00381289	https://www.ebi.ac.uk/metagenomics/api/v1/analyses/MGYA00381289/file/SRR2005380_MERGED_FASTQ_SSU_OTU.tsv	Root-Host_associated-Human-Skin
SRR5869870	MGYA00416452	https://www.ebi.ac.uk/metagenomics/api/v1/analyses/MGYA00416452/file/SRR5869870_MERGED_FASTQ_SSU_OTU.tsv	Root-Host_associated-Human-Skin
ERR450680	MGYA00426875	https://www.ebi.ac.uk/metagenomics/api/v1/analyses/MGYA00426875/file/ERR450680_FASTQ_SSU_OTU.tsv	Root-Host_associated-Human-Skin
ERR443744	MGYA00412953	https://www.ebi.ac.uk/metagenomics/api/v1/analyses/MGYA00412953/file/ERR443744_FASTQ_SSU_OTU.tsv	Root-Host_associated-Human-Skin

SRR995278	MGYA00025066	https://www.ebi.ac.uk/metagenomics/api/v1/analyses/MGYA00025066/file/SRR995278_FASTQ_otu.tsv	Root-Host_associated-Human-Skin
SRR1950737	MGYA00376904	https://www.ebi.ac.uk/metagenomics/api/v1/analyses/MGYA00376904/file/SRR1950737_MERGED_FASTQ_SSU_OTU.tsv	Root-Host_associated-Human-Skin
SRR5868623	MGYA00411953	https://www.ebi.ac.uk/metagenomics/api/v1/analyses/MGYA00411953/file/SRR5868623_MERGED_FASTQ_SSU_OTU.tsv	Root-Host_associated-Human-Skin
ERR1759961	MGYA00140571	https://www.ebi.ac.uk/metagenomics/api/v1/analyses/MGYA00140571/file/ERR1759961_MERGED_FASTQ_SSU_OTU.tsv	Root-Host_associated-Human-Skin
ERR2538439	MGYA00381446	https://www.ebi.ac.uk/metagenomics/api/v1/analyses/MGYA00381446/file/ERR2538439_FASTQ_SSU_OTU.tsv	Root-Host_associated-Human-Skin
SRR997013	MGYA00023415	https://www.ebi.ac.uk/metagenomics/api/v1/analyses/MGYA00023415/file/SRR997013_FASTQ_otu.tsv	Root-Host_associated-Human-Skin
SRR996595	MGYA00023990	https://www.ebi.ac.uk/metagenomics/api/v1/analyses/MGYA00023990/file/SRR996595_FASTQ_otu.tsv	Root-Host_associated-Human-Skin
SRR995327	MGYA00022480	https://www.ebi.ac.uk/metagenomics/api/v1/analyses/MGYA00022480/file/SRR995327_FASTQ_otu.tsv	Root-Host_associated-Human-Skin
SRR994915	MGYA00024747	https://www.ebi.ac.uk/metagenomics/api/v1/analyses/MGYA00024747/file/SRR994915_FASTQ_otu.tsv	Root-Host_associated-Human-Skin
SRR994567	MGYA00024434	https://www.ebi.ac.uk/metagenomics/api/v1/analyses/MGYA00024434/file/SRR994567_FASTQ_otu.tsv	Root-Host_associated-Human-Skin
SRR1922569	MGYA00028315	https://www.ebi.ac.uk/metagenomics/api/v1/analyses/MGYA00028315/file/SRR1922569_FASTQ_otu.tsv	Root-Host_associated-Human-Skin
ERR1960809	MGYA00130837	https://www.ebi.ac.uk/metagenomics/api/v1/analyses/MGYA00130837/file/ERR1960809_MERGED_FASTQ_otu.tsv	Root-Host_associated-Human-Skin

SRR996936	MGYA00022226	https://www.ebi.ac.uk/metagenomics/api/v1/analyses/MGYA00022226/file/SRR996936_FASTQ_otu.tsv	Root-Host_associated-Human-Skin
ERR1760020	MGYA00140447	https://www.ebi.ac.uk/metagenomics/api/v1/analyses/MGYA00140447/file/ERR1760020_MERGED_FASTQ_SSU_OTU.tsv	Root-Host_associated-Human-Skin
SRR5870040	MGYA00416929	https://www.ebi.ac.uk/metagenomics/api/v1/analyses/MGYA00416929/file/SRR5870040_MERGED_FASTQ_SSU_OTU.tsv	Root-Host_associated-Human-Skin
SRR994670	MGYA00023639	https://www.ebi.ac.uk/metagenomics/api/v1/analyses/MGYA00023639/file/SRR994670_FASTQ_otu.tsv	Root-Host_associated-Human-Skin
SRR6757618	MGYA00380814	https://www.ebi.ac.uk/metagenomics/api/v1/analyses/MGYA00380814/file/SRR6757618_MERGED_FASTQ_SSU_OTU.tsv	Root-Host_associated-Human-Skin
SRR1924035	MGYA00028359	https://www.ebi.ac.uk/metagenomics/api/v1/analyses/MGYA00028359/file/SRR1924035_FASTQ_otu.tsv	Root-Host_associated-Human-Skin
SRR5868819	MGYA00417087	https://www.ebi.ac.uk/metagenomics/api/v1/analyses/MGYA00417087/file/SRR5868819_MERGED_FASTQ_SSU_OTU.tsv	Root-Host_associated-Human-Skin
SRR996125	MGYA00024891	https://www.ebi.ac.uk/metagenomics/api/v1/analyses/MGYA00024891/file/SRR996125_FASTQ_otu.tsv	Root-Host_associated-Human-Skin
SRR1631537	MGYA00023581	https://www.ebi.ac.uk/metagenomics/api/v1/analyses/MGYA00023581/file/SRR1631537_MERGED_FASTQ_otu.tsv	Root-Host_associated-Human-Skin
ERR443792	MGYA00424254	https://www.ebi.ac.uk/metagenomics/api/v1/analyses/MGYA00424254/file/ERR443792_FASTQ_SSU_OTU.tsv	Root-Host_associated-Human-Skin
SRR1632422	MGYA00023756	https://www.ebi.ac.uk/metagenomics/api/v1/analyses/MGYA00023756/file/SRR1632422_MERGED_FASTQ_otu.tsv	Root-Host_associated-Human-Skin
SRR1632850	MGYA00023052	https://www.ebi.ac.uk/metagenomics/api/v1/analyses/MGYA00023052/file/SRR1632850_MERGED_FASTQ_otu.tsv	Root-Host_associated-Human-Skin

SRR1632385	MGYA00023523	https://www.ebi.ac.uk/metagenomics/api/v1/analyses/MGYA00023523/file/SRR1632385_MERGED_FASTQ_otu.tsv	Root-Host_associated-Human-Skin
ERR443035	MGYA00416322	https://www.ebi.ac.uk/metagenomics/api/v1/analyses/MGYA00416322/file/ERR443035_FASTQ_SSU_OTU.tsv	Root-Host_associated-Human-Skin
SRR920916	MGYA00022361	https://www.ebi.ac.uk/metagenomics/api/v1/analyses/MGYA00022361/file/SRR920916_FASTQ_otu.tsv	Root-Host_associated-Human-Skin
SRR995509	MGYA00023169	https://www.ebi.ac.uk/metagenomics/api/v1/analyses/MGYA00023169/file/SRR995509_FASTQ_otu.tsv	Root-Host_associated-Human-Skin
ERR451016	MGYA00416317	https://www.ebi.ac.uk/metagenomics/api/v1/analyses/MGYA00416317/file/ERR451016_FASTQ_SSU_OTU.tsv	Root-Host_associated-Human-Skin
SRR1704192	MGYA00428926	https://www.ebi.ac.uk/metagenomics/api/v1/analyses/MGYA00428926/file/SRR1704192_FASTQ_SSU_OTU.tsv	Root-Host_associated-Human-Skin
SRR995168	MGYA00024321	https://www.ebi.ac.uk/metagenomics/api/v1/analyses/MGYA00024321/file/SRR995168_FASTQ_otu.tsv	Root-Host_associated-Human-Skin
ERR1960634	MGYA00130769	https://www.ebi.ac.uk/metagenomics/api/v1/analyses/MGYA00130769/file/ERR1960634_MERGED_FASTQ_otu.tsv	Root-Host_associated-Human-Skin
ERR443454	MGYA00424887	https://www.ebi.ac.uk/metagenomics/api/v1/analyses/MGYA00424887/file/ERR443454_FASTQ_SSU_OTU.tsv	Root-Host_associated-Human-Skin
ERR450933	MGYA00421439	https://www.ebi.ac.uk/metagenomics/api/v1/analyses/MGYA00421439/file/ERR450933_FASTQ_SSU_OTU.tsv	Root-Host_associated-Human-Skin
SRR921033	MGYA00023084	https://www.ebi.ac.uk/metagenomics/api/v1/analyses/MGYA00023084/file/SRR921033_FASTQ_otu.tsv	Root-Host_associated-Human-Skin
SRR1633175	MGYA00025259	https://www.ebi.ac.uk/metagenomics/api/v1/analyses/MGYA00025259/file/SRR1633175_MERGED_FASTQ_otu.tsv	Root-Host_associated-Human-Skin

SRR5869877	MGYA00414828	https://www.ebi.ac.uk/metagenomics/api/v1/analyses/MGYA00414828/file/SRR5869877_MERGED_FASTQ_SSU_OTU.tsv	Root-Host_associated-Human-Skin
ERR443743	MGYA00425247	https://www.ebi.ac.uk/metagenomics/api/v1/analyses/MGYA00425247/file/ERR443743_FASTQ_SSU_OTU.tsv	Root-Host_associated-Human-Skin
SRR1623046	MGYA00024638	https://www.ebi.ac.uk/metagenomics/api/v1/analyses/MGYA00024638/file/SRR1623046_MERGED_FASTQ_otu.tsv	Root-Host_associated-Human-Skin
SRR1704285	MGYA00415664	https://www.ebi.ac.uk/metagenomics/api/v1/analyses/MGYA00415664/file/SRR1704285_FASTQ_SSU_OTU.tsv	Root-Host_associated-Human-Skin
ERR1759966	MGYA00140523	https://www.ebi.ac.uk/metagenomics/api/v1/analyses/MGYA00140523/file/ERR1759966_MERGED_FASTQ_SSU_OTU.tsv	Root-Host_associated-Human-Skin
SRR1950730	MGYA00376897	https://www.ebi.ac.uk/metagenomics/api/v1/analyses/MGYA00376897/file/SRR1950730_MERGED_FASTQ_SSU_OTU.tsv	Root-Host_associated-Human-Skin
ERR1701262	MGYA00084318	https://www.ebi.ac.uk/metagenomics/api/v1/analyses/MGYA00084318/file/ERR1701262_FASTQ_otu.tsv	Root-Host_associated-Human-Skin
ERR450817	MGYA00426830	https://www.ebi.ac.uk/metagenomics/api/v1/analyses/MGYA00426830/file/ERR450817_FASTQ_SSU_OTU.tsv	Root-Host_associated-Human-Skin
ERR981248	MGYA00010239	https://www.ebi.ac.uk/metagenomics/api/v1/analyses/MGYA00010239/file/ERR981248_MERGED_FASTQ_otu.tsv	Root-Host_associated-Human-Skin
SRR1622896	MGYA00024769	https://www.ebi.ac.uk/metagenomics/api/v1/analyses/MGYA00024769/file/SRR1622896_MERGED_FASTQ_otu.tsv	Root-Host_associated-Human-Skin
SRR5869953	MGYA00427104	https://www.ebi.ac.uk/metagenomics/api/v1/analyses/MGYA00427104/file/SRR5869953_MERGED_FASTQ_SSU_OTU.tsv	Root-Host_associated-Human-Skin
SRR996188	MGYA00022963	https://www.ebi.ac.uk/metagenomics/api/v1/analyses/MGYA00022963/file/SRR996188_FASTQ_otu.tsv	Root-Host_associated-Human-Skin

ERR2538445	MGYA00381452	https://www.ebi.ac.uk/metagenomics/api/v1/analyses/MGYA00381452/file/ERR2538445_FASTQ_SSU_OTU.tsv	Root-Host_associated-Human-Skin
SRR1923880	MGYA00028341	https://www.ebi.ac.uk/metagenomics/api/v1/analyses/MGYA00028341/file/SRR1923880_FASTQ_otu.tsv	Root-Host_associated-Human-Skin
SRR1631365	MGYA00022586	https://www.ebi.ac.uk/metagenomics/api/v1/analyses/MGYA00022586/file/SRR1631365_MERGED_FASTQ_otu.tsv	Root-Host_associated-Human-Skin
SRR1623162	MGYA00023485	https://www.ebi.ac.uk/metagenomics/api/v1/analyses/MGYA00023485/file/SRR1623162_MERGED_FASTQ_otu.tsv	Root-Host_associated-Human-Skin
SRR1630957	MGYA00024162	https://www.ebi.ac.uk/metagenomics/api/v1/analyses/MGYA00024162/file/SRR1630957_MERGED_FASTQ_otu.tsv	Root-Host_associated-Human-Skin
SRR5869140	MGYA00415989	https://www.ebi.ac.uk/metagenomics/api/v1/analyses/MGYA00415989/file/SRR5869140_MERGED_FASTQ_SSU_OTU.tsv	Root-Host_associated-Human-Skin
SRR996538	MGYA00022845	https://www.ebi.ac.uk/metagenomics/api/v1/analyses/MGYA00022845/file/SRR996538_FASTQ_otu.tsv	Root-Host_associated-Human-Skin
SRR5869636	MGYA00421604	https://www.ebi.ac.uk/metagenomics/api/v1/analyses/MGYA00421604/file/SRR5869636_MERGED_FASTQ_SSU_OTU.tsv	Root-Host_associated-Human-Skin
ERR1759893	MGYA00140580	https://www.ebi.ac.uk/metagenomics/api/v1/analyses/MGYA00140580/file/ERR1759893_MERGED_FASTQ_SSU_OTU.tsv	Root-Host_associated-Human-Skin
SRR6757664	MGYA00380777	https://www.ebi.ac.uk/metagenomics/api/v1/analyses/MGYA00380777/file/SRR6757664_MERGED_FASTQ_SSU_OTU.tsv	Root-Host_associated-Human-Skin
SRR5868865	MGYA00427117	https://www.ebi.ac.uk/metagenomics/api/v1/analyses/MGYA00427117/file/SRR5868865_MERGED_FASTQ_SSU_OTU.tsv	Root-Host_associated-Human-Skin
ERR2538494	MGYA00381501	https://www.ebi.ac.uk/metagenomics/api/v1/analyses/MGYA00381501/file/ERR2538494_FASTQ_SSU_OTU.tsv	Root-Host_associated-Human-Skin

SRR5869257	MGYA00419721	https://www.ebi.ac.uk/metagenomics/api/v1/analyses/MGYA00419721/file/SRR5869257_MERGED_FASTQ_SSU_OTU.tsv	Root-Host_associated-Human-Skin
ERR451553	MGYA00426505	https://www.ebi.ac.uk/metagenomics/api/v1/analyses/MGYA00426505/file/ERR451553_FASTQ_SSU_OTU.tsv	Root-Host_associated-Human-Skin
ERR1960710	MGYA00130881	https://www.ebi.ac.uk/metagenomics/api/v1/analyses/MGYA00130881/file/ERR1960710_MERGED_FASTQ_otu.tsv	Root-Host_associated-Human-Skin
SRR995907	MGYA00023841	https://www.ebi.ac.uk/metagenomics/api/v1/analyses/MGYA00023841/file/SRR995907_FASTQ_otu.tsv	Root-Host_associated-Human-Skin
SRR1923909	MGYA00028284	https://www.ebi.ac.uk/metagenomics/api/v1/analyses/MGYA00028284/file/SRR1923909_FASTQ_otu.tsv	Root-Host_associated-Human-Skin
SRR996812	MGYA00023633	https://www.ebi.ac.uk/metagenomics/api/v1/analyses/MGYA00023633/file/SRR996812_FASTQ_otu.tsv	Root-Host_associated-Human-Skin
SRR5868689	MGYA00421652	https://www.ebi.ac.uk/metagenomics/api/v1/analyses/MGYA00421652/file/SRR5868689_MERGED_FASTQ_SSU_OTU.tsv	Root-Host_associated-Human-Skin
SRR1704228	MGYA00428911	https://www.ebi.ac.uk/metagenomics/api/v1/analyses/MGYA00428911/file/SRR1704228_FASTQ_SSU_OTU.tsv	Root-Host_associated-Human-Skin
ERR1760104	MGYA00140552	https://www.ebi.ac.uk/metagenomics/api/v1/analyses/MGYA00140552/file/ERR1760104_MERGED_FASTQ_SSU_OTU.tsv	Root-Host_associated-Human-Skin
SRR1632798	MGYA00023213	https://www.ebi.ac.uk/metagenomics/api/v1/analyses/MGYA00023213/file/SRR1632798_MERGED_FASTQ_otu.tsv	Root-Host_associated-Human-Skin
ERR443428	MGYA00428399	https://www.ebi.ac.uk/metagenomics/api/v1/analyses/MGYA00428399/file/ERR443428_FASTQ_SSU_OTU.tsv	Root-Host_associated-Human-Skin
SRR994754	MGYA00023245	https://www.ebi.ac.uk/metagenomics/api/v1/analyses/MGYA00023245/file/SRR994754_FASTQ_otu.tsv	Root-Host_associated-Human-Skin

SRR920179	MGYA00024716	https://www.ebi.ac.uk/metagenomics/api/v1/analyses/MGYA00024716/file/SRR920179_FASTQ_otu.tsv	Root-Host_associated-Human-Skin
ERR1960648	MGYA00130852	https://www.ebi.ac.uk/metagenomics/api/v1/analyses/MGYA00130852/file/ERR1960648_MERGED_FASTQ_otu.tsv	Root-Host_associated-Human-Skin
SRR1704649	MGYA00426909	https://www.ebi.ac.uk/metagenomics/api/v1/analyses/MGYA00426909/file/SRR1704649_FASTQ_SSU_OTU.tsv	Root-Host_associated-Human-Skin
SRR996001	MGYA00023520	https://www.ebi.ac.uk/metagenomics/api/v1/analyses/MGYA00023520/file/SRR996001_FASTQ_otu.tsv	Root-Host_associated-Human-Skin
ERR441971	MGYA00385257	https://www.ebi.ac.uk/metagenomics/api/v1/analyses/MGYA00385257/file/ERR441971_FASTQ_SSU_OTU.tsv	Root-Host_associated-Human-Skin
SRR1704698	MGYA00428444	https://www.ebi.ac.uk/metagenomics/api/v1/analyses/MGYA00428444/file/SRR1704698_FASTQ_SSU_OTU.tsv	Root-Host_associated-Human-Skin
ERR1960699	MGYA00130895	https://www.ebi.ac.uk/metagenomics/api/v1/analyses/MGYA00130895/file/ERR1960699_MERGED_FASTQ_otu.tsv	Root-Host_associated-Human-Skin
SRR1624822	MGYA00024885	https://www.ebi.ac.uk/metagenomics/api/v1/analyses/MGYA00024885/file/SRR1624822_MERGED_FASTQ_otu.tsv	Root-Host_associated-Human-Skin
SRR995662	MGYA00024847	https://www.ebi.ac.uk/metagenomics/api/v1/analyses/MGYA00024847/file/SRR995662_FASTQ_otu.tsv	Root-Host_associated-Human-Skin
SRR5868658	MGYA00423511	https://www.ebi.ac.uk/metagenomics/api/v1/analyses/MGYA00423511/file/SRR5868658_MERGED_FASTQ_SSU_OTU.tsv	Root-Host_associated-Human-Skin
SRR1632328	MGYA00024023	https://www.ebi.ac.uk/metagenomics/api/v1/analyses/MGYA00024023/file/SRR1632328_MERGED_FASTQ_otu.tsv	Root-Host_associated-Human-Skin
ERR443098	MGYA00413702	https://www.ebi.ac.uk/metagenomics/api/v1/analyses/MGYA00413702/file/ERR443098_FASTQ_SSU_OTU.tsv	Root-Host_associated-Human-Skin

ERR1760015	MGYA00140378	https://www.ebi.ac.uk/metagenomics/api/v1/analyses/MGYA00140378/file/ERR1760015_MERGED_FASTQ_SSU_OTU.tsv	Root-Host_associated-Human-Skin
SRR995005	MGYA00024150	https://www.ebi.ac.uk/metagenomics/api/v1/analyses/MGYA00024150/file/SRR995005_FASTQ_otu.tsv	Root-Host_associated-Human-Skin
SRR5870075	MGYA00426215	https://www.ebi.ac.uk/metagenomics/api/v1/analyses/MGYA00426215/file/SRR5870075_MERGED_FASTQ_SSU_OTU.tsv	Root-Host_associated-Human-Skin
ERR443539	MGYA00415124	https://www.ebi.ac.uk/metagenomics/api/v1/analyses/MGYA00415124/file/ERR443539_FASTQ_SSU_OTU.tsv	Root-Host_associated-Human-Skin
ERR1960759	MGYA00130838	https://www.ebi.ac.uk/metagenomics/api/v1/analyses/MGYA00130838/file/ERR1960759_MERGED_FASTQ_otu.tsv	Root-Host_associated-Human-Skin
SRR1924000	MGYA00028231	https://www.ebi.ac.uk/metagenomics/api/v1/analyses/MGYA00028231/file/SRR1924000_FASTQ_otu.tsv	Root-Host_associated-Human-Skin
SRR996110	MGYA00025171	https://www.ebi.ac.uk/metagenomics/api/v1/analyses/MGYA00025171/file/SRR996110_FASTQ_otu.tsv	Root-Host_associated-Human-Skin
SRR994583	MGYA00022925	https://www.ebi.ac.uk/metagenomics/api/v1/analyses/MGYA00022925/file/SRR994583_FASTQ_otu.tsv	Root-Host_associated-Human-Skin
SRR1923818	MGYA00028405	https://www.ebi.ac.uk/metagenomics/api/v1/analyses/MGYA00028405/file/SRR1923818_FASTQ_otu.tsv	Root-Host_associated-Human-Skin
SRR996571	MGYA00022416	https://www.ebi.ac.uk/metagenomics/api/v1/analyses/MGYA00022416/file/SRR996571_FASTQ_otu.tsv	Root-Host_associated-Human-Skin
SRR996903	MGYA00025325	https://www.ebi.ac.uk/metagenomics/api/v1/analyses/MGYA00025325/file/SRR996903_FASTQ_otu.tsv	Root-Host_associated-Human-Skin
SRR5868798	MGYA00412501	https://www.ebi.ac.uk/metagenomics/api/v1/analyses/MGYA00412501/file/SRR5868798_MERGED_FASTQ_SSU_OTU.tsv	Root-Host_associated-Human-Skin

SRR996207	MGYA00024171	https://www.ebi.ac.uk/metagenomics/api/v1/analyses/MGYA00024171/file/SRR996207_FASTQ_otu.tsv	Root-Host_associated-Human-Skin
SRR996666	MGYA00024772	https://www.ebi.ac.uk/metagenomics/api/v1/analyses/MGYA00024772/file/SRR996666_FASTQ_otu.tsv	Root-Host_associated-Human-Skin
SRR994694	MGYA00023879	https://www.ebi.ac.uk/metagenomics/api/v1/analyses/MGYA00023879/file/SRR994694_FASTQ_otu.tsv	Root-Host_associated-Human-Skin
ERR1960788	MGYA00130907	https://www.ebi.ac.uk/metagenomics/api/v1/analyses/MGYA00130907/file/ERR1960788_MERGED_FASTQ_otu.tsv	Root-Host_associated-Human-Skin
SRR1633018	MGYA00023962	https://www.ebi.ac.uk/metagenomics/api/v1/analyses/MGYA00023962/file/SRR1633018_MERGED_FASTQ_otu.tsv	Root-Host_associated-Human-Skin
SRR920580	MGYA00024254	https://www.ebi.ac.uk/metagenomics/api/v1/analyses/MGYA00024254/file/SRR920580_FASTQ_otu.tsv	Root-Host_associated-Human-Skin
SRR920227	MGYA00023284	https://www.ebi.ac.uk/metagenomics/api/v1/analyses/MGYA00023284/file/SRR920227_FASTQ_otu.tsv	Root-Host_associated-Human-Skin
SRR994878	MGYA00025273	https://www.ebi.ac.uk/metagenomics/api/v1/analyses/MGYA00025273/file/SRR994878_FASTQ_otu.tsv	Root-Host_associated-Human-Skin
SRR1631274	MGYA00024737	https://www.ebi.ac.uk/metagenomics/api/v1/analyses/MGYA00024737/file/SRR1631274_MERGED_FASTQ_otu.tsv	Root-Host_associated-Human-Skin
SRR5868611	MGYA00428105	https://www.ebi.ac.uk/metagenomics/api/v1/analyses/MGYA00428105/file/SRR5868611_MERGED_FASTQ_SSU_OTU.tsv	Root-Host_associated-Human-Skin
ERR1759953	MGYA00140381	https://www.ebi.ac.uk/metagenomics/api/v1/analyses/MGYA00140381/file/ERR1759953_MERGED_FASTQ_SSU_OTU.tsv	Root-Host_associated-Human-Skin
SRR1950705	MGYA00376872	https://www.ebi.ac.uk/metagenomics/api/v1/analyses/MGYA00376872/file/SRR1950705_MERGED_FASTQ_SSU_OTU.tsv	Root-Host_associated-Human-Skin

ERR451493	MGYA00413656	https://www.ebi.ac.uk/metagenomics/api/v1/analyses/MGYA00413656/file/ERR451493_FASTQ_SSU_OTU.tsv	Root-Host_associated-Human-Skin
SRR1633140	MGYA00022608	https://www.ebi.ac.uk/metagenomics/api/v1/analyses/MGYA00022608/file/SRR1633140_MERGED_FASTQ_otu.tsv	Root-Host_associated-Human-Skin
ERR442925	MGYA00422956	https://www.ebi.ac.uk/metagenomics/api/v1/analyses/MGYA00422956/file/ERR442925_FASTQ_SSU_OTU.tsv	Root-Host_associated-Human-Skin
SRR5869842	MGYA00416955	https://www.ebi.ac.uk/metagenomics/api/v1/analyses/MGYA00416955/file/SRR5869842_MERGED_FASTQ_SSU_OTU.tsv	Root-Host_associated-Human-Skin
SRR1704176	MGYA00422061	https://www.ebi.ac.uk/metagenomics/api/v1/analyses/MGYA00422061/file/SRR1704176_FASTQ_SSU_OTU.tsv	Root-Host_associated-Human-Skin
SRR5868974	MGYA00421646	https://www.ebi.ac.uk/metagenomics/api/v1/analyses/MGYA00421646/file/SRR5868974_MERGED_FASTQ_SSU_OTU.tsv	Root-Host_associated-Human-Skin
SRR996048	MGYA00023064	https://www.ebi.ac.uk/metagenomics/api/v1/analyses/MGYA00023064/file/SRR996048_FASTQ_otu.tsv	Root-Host_associated-Human-Skin
SRR920130	MGYA00024324	https://www.ebi.ac.uk/metagenomics/api/v1/analyses/MGYA00024324/file/SRR920130_FASTQ_otu.tsv	Root-Host_associated-Human-Skin
SRR920697	MGYA00024835	https://www.ebi.ac.uk/metagenomics/api/v1/analyses/MGYA00024835/file/SRR920697_FASTQ_otu.tsv	Root-Host_associated-Human-Skin
ERR1759982	MGYA00140434	https://www.ebi.ac.uk/metagenomics/api/v1/analyses/MGYA00140434/file/ERR1759982_MERGED_FASTQ_SSU_OTU.tsv	Root-Host_associated-Human-Skin
SRR1632417	MGYA00023813	https://www.ebi.ac.uk/metagenomics/api/v1/analyses/MGYA00023813/file/SRR1632417_MERGED_FASTQ_otu.tsv	Root-Host_associated-Human-Skin
SRR1923940	MGYA00028269	https://www.ebi.ac.uk/metagenomics/api/v1/analyses/MGYA00028269/file/SRR1923940_FASTQ_otu.tsv	Root-Host_associated-Human-Skin

SRR6757651	MGYA00380766	https://www.ebi.ac.uk/metagenomics/api/v1/analyses/MGYA00380766/file/SRR6757651_MERGED_FASTQ_SSU_OTU.tsv	Root-Host_associated-Human-Skin
SRR5868850	MGYA00417069	https://www.ebi.ac.uk/metagenomics/api/v1/analyses/MGYA00417069/file/SRR5868850_MERGED_FASTQ_SSU_OTU.tsv	Root-Host_associated-Human-Skin
SRR1704724	MGYA00426576	https://www.ebi.ac.uk/metagenomics/api/v1/analyses/MGYA00426576/file/SRR1704724_FASTQ_SSU_OTU.tsv	Root-Host_associated-Human-Skin
SRR1631047	MGYA00025014	https://www.ebi.ac.uk/metagenomics/api/v1/analyses/MGYA00025014/file/SRR1631047_MERGED_FASTQ_otu.tsv	Root-Host_associated-Human-Skin
SRR1622872	MGYA00024185	https://www.ebi.ac.uk/metagenomics/api/v1/analyses/MGYA00024185/file/SRR1622872_MERGED_FASTQ_otu.tsv	Root-Host_associated-Human-Skin
ERR1760069	MGYA00140455	https://www.ebi.ac.uk/metagenomics/api/v1/analyses/MGYA00140455/file/ERR1760069_MERGED_FASTQ_SSU_OTU.tsv	Root-Host_associated-Human-Skin
ERR1960725	MGYA00130742	https://www.ebi.ac.uk/metagenomics/api/v1/analyses/MGYA00130742/file/ERR1960725_MERGED_FASTQ_otu.tsv	Root-Host_associated-Human-Skin
ERR1701286	MGYA00084240	https://www.ebi.ac.uk/metagenomics/api/v1/analyses/MGYA00084240/file/ERR1701286_FASTQ_otu.tsv	Root-Host_associated-Human-Skin
SRR5870009	MGYA00418718	https://www.ebi.ac.uk/metagenomics/api/v1/analyses/MGYA00418718/file/SRR5870009_MERGED_FASTQ_SSU_OTU.tsv	Root-Host_associated-Human-Skin
SRR994639	MGYA00023225	https://www.ebi.ac.uk/metagenomics/api/v1/analyses/MGYA00023225/file/SRR994639_FASTQ_otu.tsv	Root-Host_associated-Human-Skin
SRR1623186	MGYA00023676	https://www.ebi.ac.uk/metagenomics/api/v1/analyses/MGYA00023676/file/SRR1623186_MERGED_FASTQ_otu.tsv	Root-Host_associated-Human-Skin
SRR1704345	MGYA00417462	https://www.ebi.ac.uk/metagenomics/api/v1/analyses/MGYA00417462/file/SRR1704345_FASTQ_SSU_OTU.tsv	Root-Host_associated-Human-Skin

SRR1631381	MGYA00024090	https://www.ebi.ac.uk/metagenomics/api/v1/analyses/MGYA00024090/file/SRR1631381_MERGED_FASTQ_otu.tsv	Root-Host_associated-Human-Skin
SRR1923864	MGYA00028321	https://www.ebi.ac.uk/metagenomics/api/v1/analyses/MGYA00028321/file/SRR1923864_FASTQ_otu.tsv	Root-Host_associated-Human-Skin
SRR995418	MGYA00024922	https://www.ebi.ac.uk/metagenomics/api/v1/analyses/MGYA00024922/file/SRR995418_FASTQ_otu.tsv	Root-Host_associated-Human-Skin
ERR451107	MGYA00416304	https://www.ebi.ac.uk/metagenomics/api/v1/analyses/MGYA00416304/file/ERR451107_FASTQ_SSU_OTU.tsv	Root-Host_associated-Human-Skin
SRR1632990	MGYA00022271	https://www.ebi.ac.uk/metagenomics/api/v1/analyses/MGYA00022271/file/SRR1632990_MERGED_FASTQ_otu.tsv	Root-Host_associated-Human-Skin
SRR1704394	MGYA00424947	https://www.ebi.ac.uk/metagenomics/api/v1/analyses/MGYA00424947/file/SRR1704394_FASTQ_SSU_OTU.tsv	Root-Host_associated-Human-Skin
SRR5868735	MGYA00421197	https://www.ebi.ac.uk/metagenomics/api/v1/analyses/MGYA00421197/file/SRR5868735_MERGED_FASTQ_SSU_OTU.tsv	Root-Host_associated-Human-Skin
ERR443594	MGYA00416639	https://www.ebi.ac.uk/metagenomics/api/v1/analyses/MGYA00416639/file/ERR443594_FASTQ_SSU_OTU.tsv	Root-Host_associated-Human-Skin
ERR1701257	MGYA00084269	https://www.ebi.ac.uk/metagenomics/api/v1/analyses/MGYA00084269/file/ERR1701257_FASTQ_otu.tsv	Root-Host_associated-Human-Skin
SRR921122	MGYA00025182	https://www.ebi.ac.uk/metagenomics/api/v1/analyses/MGYA00025182/file/SRR921122_FASTQ_otu.tsv	Root-Host_associated-Human-Skin
ERR2538470	MGYA00381477	https://www.ebi.ac.uk/metagenomics/api/v1/analyses/MGYA00381477/file/ERR2538470_FASTQ_SSU_OTU.tsv	Root-Host_associated-Human-Skin
SRR6757680	MGYA00380793	https://www.ebi.ac.uk/metagenomics/api/v1/analyses/MGYA00380793/file/SRR6757680_MERGED_FASTQ_SSU_OTU.tsv	Root-Host_associated-Human-Skin

SRR996323	MGYA00022942	https://www.ebi.ac.uk/metagenomics/api/v1/analyses/MGYA00022942/file/SRR996323_FASTQ_otu.tsv	Root-Host_associated-Human-Skin
SRR1950779	MGYA00376946	https://www.ebi.ac.uk/metagenomics/api/v1/analyses/MGYA00376946/file/SRR1950779_MERGED_FASTQ_SSU_OTU.tsv	Root-Host_associated-Human-Skin
SRR995591	MGYA00025205	https://www.ebi.ac.uk/metagenomics/api/v1/analyses/MGYA00025205/file/SRR995591_FASTQ_otu.tsv	Root-Host_associated-Human-Skin
ERR442042	MGYA00385245	https://www.ebi.ac.uk/metagenomics/api/v1/analyses/MGYA00385245/file/ERR442042_FASTQ_SSU_OTU.tsv	Root-Host_associated-Human-Skin
SRR994476	MGYA00022249	https://www.ebi.ac.uk/metagenomics/api/v1/analyses/MGYA00022249/file/SRR994476_FASTQ_otu.tsv	Root-Host_associated-Human-Skin
SRR994804	MGYA00023022	https://www.ebi.ac.uk/metagenomics/api/v1/analyses/MGYA00023022/file/SRR994804_FASTQ_otu.tsv	Root-Host_associated-Human-Skin
SRR996742	MGYA00023617	https://www.ebi.ac.uk/metagenomics/api/v1/analyses/MGYA00023617/file/SRR996742_FASTQ_otu.tsv	Root-Host_associated-Human-Skin
SRR1631669	MGYA00023155	https://www.ebi.ac.uk/metagenomics/api/v1/analyses/MGYA00023155/file/SRR1631669_MERGED_FASTQ_otu.tsv	Root-Host_associated-Human-Skin
SRR5870180	MGYA00418693	https://www.ebi.ac.uk/metagenomics/api/v1/analyses/MGYA00418693/file/SRR5870180_MERGED_FASTQ_SSU_OTU.tsv	Root-Host_associated-Human-Skin
ERR451399	MGYA00416263	https://www.ebi.ac.uk/metagenomics/api/v1/analyses/MGYA00416263/file/ERR451399_FASTQ_SSU_OTU.tsv	Root-Host_associated-Human-Skin
SRR995121	MGYA00023918	https://www.ebi.ac.uk/metagenomics/api/v1/analyses/MGYA00023918/file/SRR995121_FASTQ_otu.tsv	Root-Host_associated-Human-Skin
ERR1760131	MGYA00140604	https://www.ebi.ac.uk/metagenomics/api/v1/analyses/MGYA00140604/file/ERR1760131_MERGED_FASTQ_SSU_OTU.tsv	Root-Host_associated-Human-Skin

SRR919637	MGYA00023770	https://www.ebi.ac.uk/metagenomics/api/v1/analyses/MGYA00023770/file/SRR919637_FASTQ_otu.tsv	Root-Host_associated-Human-Skin
ERR441995	MGYA00385186	https://www.ebi.ac.uk/metagenomics/api/v1/analyses/MGYA00385186/file/ERR441995_FASTQ_SSU_OTU.tsv	Root-Host_associated-Human-Skin
SRR1632819	MGYA00022599	https://www.ebi.ac.uk/metagenomics/api/v1/analyses/MGYA00022599/file/SRR1632819_MERGED_FASTQ_otu.tsv	Root-Host_associated-Human-Skin
SRR995540	MGYA00022742	https://www.ebi.ac.uk/metagenomics/api/v1/analyses/MGYA00022742/file/SRR995540_FASTQ_otu.tsv	Root-Host_associated-Human-Skin
SRR1622875	MGYA00024178	https://www.ebi.ac.uk/metagenomics/api/v1/analyses/MGYA00024178/file/SRR1622875_MERGED_FASTQ_otu.tsv	Root-Host_associated-Human-Skin
SRR5868857	MGYA00424493	https://www.ebi.ac.uk/metagenomics/api/v1/analyses/MGYA00424493/file/SRR5868857_MERGED_FASTQ_SSU_OTU.tsv	Root-Host_associated-Human-Skin
SRR6757656	MGYA00380771	https://www.ebi.ac.uk/metagenomics/api/v1/analyses/MGYA00380771/file/SRR6757656_MERGED_FASTQ_SSU_OTU.tsv	Root-Host_associated-Human-Skin
SRR1631040	MGYA00025007	https://www.ebi.ac.uk/metagenomics/api/v1/analyses/MGYA00025007/file/SRR1631040_MERGED_FASTQ_otu.tsv	Root-Host_associated-Human-Skin
ERR1701281	MGYA00084326	https://www.ebi.ac.uk/metagenomics/api/v1/analyses/MGYA00084326/file/ERR1701281_FASTQ_otu.tsv	Root-Host_associated-Human-Skin
ERR1960722	MGYA00130821	https://www.ebi.ac.uk/metagenomics/api/v1/analyses/MGYA00130821/file/ERR1960722_MERGED_FASTQ_otu.tsv	Root-Host_associated-Human-Skin
SRR1631386	MGYA00024085	https://www.ebi.ac.uk/metagenomics/api/v1/analyses/MGYA00024085/file/SRR1631386_MERGED_FASTQ_otu.tsv	Root-Host_associated-Human-Skin
SRR1623181	MGYA00023675	https://www.ebi.ac.uk/metagenomics/api/v1/analyses/MGYA00023675/file/SRR1623181_MERGED_FASTQ_otu.tsv	Root-Host_associated-Human-Skin

SRR1704342	MGYA00417847	https://www.ebi.ac.uk/metagenomics/api/v1/analyses/MGYA00417847/file/SRR1704342_FASTQ_SSU_OTU.tsv	Root-Host_associated-Human-Skin
SRR996978	MGYA00022638	https://www.ebi.ac.uk/metagenomics/api/v1/analyses/MGYA00022638/file/SRR996978_FASTQ_otu.tsv	Root-Host_associated-Human-Skin
SRR5869604	MGYA00425424	https://www.ebi.ac.uk/metagenomics/api/v1/analyses/MGYA00425424/file/SRR5869604_MERGED_FASTQ_SSU_OTU.tsv	Root-Host_associated-Human-Skin
SRR1923863	MGYA00028320	https://www.ebi.ac.uk/metagenomics/api/v1/analyses/MGYA00028320/file/SRR1923863_FASTQ_otu.tsv	Root-Host_associated-Human-Skin
ERR443123	MGYA00415551	https://www.ebi.ac.uk/metagenomics/api/v1/analyses/MGYA00415551/file/ERR443123_FASTQ_SSU_OTU.tsv	Root-Host_associated-Human-Skin
SRR1632997	MGYA00022264	https://www.ebi.ac.uk/metagenomics/api/v1/analyses/MGYA00022264/file/SRR1632997_MERGED_FASTQ_otu.tsv	Root-Host_associated-Human-Skin
ERR443655	MGYA00414256	https://www.ebi.ac.uk/metagenomics/api/v1/analyses/MGYA00414256/file/ERR443655_FASTQ_SSU_OTU.tsv	Root-Host_associated-Human-Skin
SRR994529	MGYA00024053	https://www.ebi.ac.uk/metagenomics/api/v1/analyses/MGYA00024053/file/SRR994529_FASTQ_otu.tsv	Root-Host_associated-Human-Skin
SRR995369	MGYA00022912	https://www.ebi.ac.uk/metagenomics/api/v1/analyses/MGYA00022912/file/SRR995369_FASTQ_otu.tsv	Root-Host_associated-Human-Skin
SRR921125	MGYA00025187	https://www.ebi.ac.uk/metagenomics/api/v1/analyses/MGYA00025187/file/SRR921125_FASTQ_otu.tsv	Root-Host_associated-Human-Skin
SRR995708	MGYA00023805	https://www.ebi.ac.uk/metagenomics/api/v1/analyses/MGYA00023805/file/SRR995708_FASTQ_otu.tsv	Root-Host_associated-Human-Skin
ERR1701250	MGYA00084302	https://www.ebi.ac.uk/metagenomics/api/v1/analyses/MGYA00084302/file/ERR1701250_FASTQ_otu.tsv	Root-Host_associated-Human-Skin

ERR443234	MGYA00412630	https://www.ebi.ac.uk/metagenomics/api/v1/analyses/MGYA00412630/file/ERR443234_FASTQ_SSU_OTU.tsv	Root-Host_associated-Human-Skin
SRR6757687	MGYA00380800	https://www.ebi.ac.uk/metagenomics/api/v1/analyses/MGYA00380800/file/SRR6757687_MERGED_FASTQ_SSU_OTU.tsv	Root-Host_associated-Human-Skin
ERR2538477	MGYA00381484	https://www.ebi.ac.uk/metagenomics/api/v1/analyses/MGYA00381484/file/ERR2538477_FASTQ_SSU_OTU.tsv	Root-Host_associated-Human-Skin
SRR5869513	MGYA00414857	https://www.ebi.ac.uk/metagenomics/api/v1/analyses/MGYA00414857/file/SRR5869513_MERGED_FASTQ_SSU_OTU.tsv	Root-Host_associated-Human-Skin
SRR996324	MGYA00022949	https://www.ebi.ac.uk/metagenomics/api/v1/analyses/MGYA00022949/file/SRR996324_FASTQ_otu.tsv	Root-Host_associated-Human-Skin
ERR1759928	MGYA00140553	https://www.ebi.ac.uk/metagenomics/api/v1/analyses/MGYA00140553/file/ERR1759928_MERGED_FASTQ_SSU_OTU.tsv	Root-Host_associated-Human-Skin
SRR994471	MGYA00022250	https://www.ebi.ac.uk/metagenomics/api/v1/analyses/MGYA00022250/file/SRR994471_FASTQ_otu.tsv	Root-Host_associated-Human-Skin
ERR442045	MGYA00385155	https://www.ebi.ac.uk/metagenomics/api/v1/analyses/MGYA00385155/file/ERR442045_FASTQ_SSU_OTU.tsv	Root-Host_associated-Human-Skin
SRR995596	MGYA00025204	https://www.ebi.ac.uk/metagenomics/api/v1/analyses/MGYA00025204/file/SRR995596_FASTQ_otu.tsv	Root-Host_associated-Human-Skin
SRR996745	MGYA00023610	https://www.ebi.ac.uk/metagenomics/api/v1/analyses/MGYA00023610/file/SRR996745_FASTQ_otu.tsv	Root-Host_associated-Human-Skin
SRR995126	MGYA00023923	https://www.ebi.ac.uk/metagenomics/api/v1/analyses/MGYA00023923/file/SRR995126_FASTQ_otu.tsv	Root-Host_associated-Human-Skin
ERR1760136	MGYA00140593	https://www.ebi.ac.uk/metagenomics/api/v1/analyses/MGYA00140593/file/ERR1760136_MERGED_FASTQ_SSU_OTU.tsv	Root-Host_associated-Human-Skin

SRR919630	MGYA00023763	https://www.ebi.ac.uk/metagenomics/api/v1/analyses/MGYA00023763/file/SRR919630_FASTQ_otu.tsv	Root-Host_associated-Human-Skin
ERR441992	MGYA00385228	https://www.ebi.ac.uk/metagenomics/api/v1/analyses/MGYA00385228/file/ERR441992_FASTQ_SSU_OTU.tsv	Root-Host_associated-Human-Skin
SRR995547	MGYA00022745	https://www.ebi.ac.uk/metagenomics/api/v1/analyses/MGYA00022745/file/SRR995547_FASTQ_otu.tsv	Root-Host_associated-Human-Skin
SRR920958	MGYA00022791	https://www.ebi.ac.uk/metagenomics/api/v1/analyses/MGYA00022791/file/SRR920958_FASTQ_otu.tsv	Root-Host_associated-Human-Skin
SRR5870290	MGYA00422521	https://www.ebi.ac.uk/metagenomics/api/v1/analyses/MGYA00422521/file/SRR5870290_MERGED_FASTQ_SSU_OTU.tsv	Root-Host_associated-Human-Skin
ERR1760012	MGYA00140393	https://www.ebi.ac.uk/metagenomics/api/v1/analyses/MGYA00140393/file/ERR1760012_MERGED_FASTQ_SSU_OTU.tsv	Root-Host_associated-Human-Skin
SRR995002	MGYA00024145	https://www.ebi.ac.uk/metagenomics/api/v1/analyses/MGYA00024145/file/SRR995002_FASTQ_otu.tsv	Root-Host_associated-Human-Skin
SRR996117	MGYA00025178	https://www.ebi.ac.uk/metagenomics/api/v1/analyses/MGYA00025178/file/SRR996117_FASTQ_otu.tsv	Root-Host_associated-Human-Skin
SRR994584	MGYA00022928	https://www.ebi.ac.uk/metagenomics/api/v1/analyses/MGYA00022928/file/SRR994584_FASTQ_otu.tsv	Root-Host_associated-Human-Skin
SRR996904	MGYA00025324	https://www.ebi.ac.uk/metagenomics/api/v1/analyses/MGYA00025324/file/SRR996904_FASTQ_otu.tsv	Root-Host_associated-Human-Skin
SRR996576	MGYA00022417	https://www.ebi.ac.uk/metagenomics/api/v1/analyses/MGYA00022417/file/SRR996576_FASTQ_otu.tsv	Root-Host_associated-Human-Skin
SRR996200	MGYA00024172	https://www.ebi.ac.uk/metagenomics/api/v1/analyses/MGYA00024172/file/SRR996200_FASTQ_otu.tsv	Root-Host_associated-Human-Skin

SRR996661	MGYA00024779	https://www.ebi.ac.uk/metagenomics/api/v1/analyses/MGYA00024779/file/SRR996661_FASTQ_otu.tsv	Root-Host_associated-Human-Skin
SRR994693	MGYA00023876	https://www.ebi.ac.uk/metagenomics/api/v1/analyses/MGYA00023876/file/SRR994693_FASTQ_otu.tsv	Root-Host_associated-Human-Skin
ERR443771	MGYA00422349	https://www.ebi.ac.uk/metagenomics/api/v1/analyses/MGYA00422349/file/ERR443771_FASTQ_SSU_OTU.tsv	Root-Host_associated-Human-Skin
SRR920220	MGYA00023291	https://www.ebi.ac.uk/metagenomics/api/v1/analyses/MGYA00023291/file/SRR920220_FASTQ_otu.tsv	Root-Host_associated-Human-Skin
SRR920587	MGYA00024249	https://www.ebi.ac.uk/metagenomics/api/v1/analyses/MGYA00024249/file/SRR920587_FASTQ_otu.tsv	Root-Host_associated-Human-Skin
ERR442039	MGYA00385241	https://www.ebi.ac.uk/metagenomics/api/v1/analyses/MGYA00385241/file/ERR442039_FASTQ_SSU_OTU.tsv	Root-Host_associated-Human-Skin
SRR919929	MGYA00025192	https://www.ebi.ac.uk/metagenomics/api/v1/analyses/MGYA00025192/file/SRR919929_FASTQ_otu.tsv	Root-Host_associated-Human-Skin
SRR1950702	MGYA00376869	https://www.ebi.ac.uk/metagenomics/api/v1/analyses/MGYA00376869/file/SRR1950702_MERGED_FASTQ_SSU_OTU.tsv	Root-Host_associated-Human-Skin
ERR1759954	MGYA00140480	https://www.ebi.ac.uk/metagenomics/api/v1/analyses/MGYA00140480/file/ERR1759954_MERGED_FASTQ_SSU_OTU.tsv	Root-Host_associated-Human-Skin
SRR1631273	MGYA00024730	https://www.ebi.ac.uk/metagenomics/api/v1/analyses/MGYA00024730/file/SRR1631273_MERGED_FASTQ_otu.tsv	Root-Host_associated-Human-Skin
SRR1704510	MGYA00413076	https://www.ebi.ac.uk/metagenomics/api/v1/analyses/MGYA00413076/file/SRR1704510_FASTQ_SSU_OTU.tsv	Root-Host_associated-Human-Skin
SRR1633147	MGYA00024782	https://www.ebi.ac.uk/metagenomics/api/v1/analyses/MGYA00024782/file/SRR1633147_MERGED_FASTQ_otu.tsv	Root-Host_associated-Human-Skin

SRR5869437	MGYA00419679	https://www.ebi.ac.uk/metagenomics/api/v1/analyses/MGYA00419679/file/SRR5869437_MERGED_FASTQ_SSU_OTU.tsv	Root-Host_associated-Human-Skin
SRR920690	MGYA00024840	https://www.ebi.ac.uk/metagenomics/api/v1/analyses/MGYA00024840/file/SRR920690_FASTQ_otu.tsv	Root-Host_associated-Human-Skin
SRR920137	MGYA00024317	https://www.ebi.ac.uk/metagenomics/api/v1/analyses/MGYA00024317/file/SRR920137_FASTQ_otu.tsv	Root-Host_associated-Human-Skin
ERR443814	MGYA00416628	https://www.ebi.ac.uk/metagenomics/api/v1/analyses/MGYA00416628/file/ERR443814_FASTQ_SSU_OTU.tsv	Root-Host_associated-Human-Skin
ERR443466	MGYA00419487	https://www.ebi.ac.uk/metagenomics/api/v1/analyses/MGYA00419487/file/ERR443466_FASTQ_SSU_OTU.tsv	Root-Host_associated-Human-Skin
ERR1759985	MGYA00140452	https://www.ebi.ac.uk/metagenomics/api/v1/analyses/MGYA00140452/file/ERR1759985_MERGED_FASTQ_SSU_OTU.tsv	Root-Host_associated-Human-Skin
SRR1923947	MGYA00028224	https://www.ebi.ac.uk/metagenomics/api/v1/analyses/MGYA00028224/file/SRR1923947_FASTQ_otu.tsv	Root-Host_associated-Human-Skin
ERR451024	MGYA00423307	https://www.ebi.ac.uk/metagenomics/api/v1/analyses/MGYA00423307/file/ERR451024_FASTQ_SSU_OTU.tsv	Root-Host_associated-Human-Skin
SRR995949	MGYA00024278	https://www.ebi.ac.uk/metagenomics/api/v1/analyses/MGYA00024278/file/SRR995949_FASTQ_otu.tsv	Root-Host_associated-Human-Skin
SRR1632410	MGYA00023334	https://www.ebi.ac.uk/metagenomics/api/v1/analyses/MGYA00023334/file/SRR1632410_MERGED_FASTQ_otu.tsv	Root-Host_associated-Human-Skin
ERR1760060	MGYA00140513	https://www.ebi.ac.uk/metagenomics/api/v1/analyses/MGYA00140513/file/ERR1760060_MERGED_FASTQ_SSU_OTU.tsv	Root-Host_associated-Human-Skin
SRR995070	MGYA00024642	https://www.ebi.ac.uk/metagenomics/api/v1/analyses/MGYA00024642/file/SRR995070_FASTQ_otu.tsv	Root-Host_associated-Human-Skin

SRR994630	MGYA00023234	https://www.ebi.ac.uk/metagenomics/api/v1/analyses/MGYA00023234/file/SRR994630_FASTQ_otu.tsv	Root-Host_associated-Human-Skin
SRR5868859	MGYA00416040	https://www.ebi.ac.uk/metagenomics/api/v1/analyses/MGYA00416040/file/SRR5868859_MERGED_FASTQ_SSU_OTU.tsv	Root-Host_associated-Human-Skin
SRR6757658	MGYA00380817	https://www.ebi.ac.uk/metagenomics/api/v1/analyses/MGYA00380817/file/SRR6757658_MERGED_FASTQ_SSU_OTU.tsv	Root-Host_associated-Human-Skin
SRR996165	MGYA00022286	https://www.ebi.ac.uk/metagenomics/api/v1/analyses/MGYA00022286/file/SRR996165_FASTQ_otu.tsv	Root-Host_associated-Human-Skin
SRR995411	MGYA00024925	https://www.ebi.ac.uk/metagenomics/api/v1/analyses/MGYA00024925/file/SRR995411_FASTQ_otu.tsv	Root-Host_associated-Human-Skin
SRR996976	MGYA00022640	https://www.ebi.ac.uk/metagenomics/api/v1/analyses/MGYA00022640/file/SRR996976_FASTQ_otu.tsv	Root-Host_associated-Human-Skin
SRR1631388	MGYA00024093	https://www.ebi.ac.uk/metagenomics/api/v1/analyses/MGYA00024093/file/SRR1631388_MERGED_FASTQ_otu.tsv	Root-Host_associated-Human-Skin
SRR995367	MGYA00022914	https://www.ebi.ac.uk/metagenomics/api/v1/analyses/MGYA00022914/file/SRR995367_FASTQ_otu.tsv	Root-Host_associated-Human-Skin
SRR994527	MGYA00024045	https://www.ebi.ac.uk/metagenomics/api/v1/analyses/MGYA00024045/file/SRR994527_FASTQ_otu.tsv	Root-Host_associated-Human-Skin
SRR994955	MGYA00024342	https://www.ebi.ac.uk/metagenomics/api/v1/analyses/MGYA00024342/file/SRR994955_FASTQ_otu.tsv	Root-Host_associated-Human-Skin
SRR1632999	MGYA00022272	https://www.ebi.ac.uk/metagenomics/api/v1/analyses/MGYA00022272/file/SRR1632999_MERGED_FASTQ_otu.tsv	Root-Host_associated-Human-Skin
ERR2538479	MGYA00381486	https://www.ebi.ac.uk/metagenomics/api/v1/analyses/MGYA00381486/file/ERR2538479_FASTQ_SSU_OTU.tsv	Root-Host_associated-Human-Skin

SRR2005538	MGYA00381294	https://www.ebi.ac.uk/metagenomics/api/v1/analyses/MGYA00381294/file/SRR2005538_MERGED_FASTQ_SSU_OTU.tsv	Root-Host_associated-Human-Skin
SRR997053	MGYA00022965	https://www.ebi.ac.uk/metagenomics/api/v1/analyses/MGYA00022965/file/SRR997053_FASTQ_otu.tsv	Root-Host_associated-Human-Skin
SRR6757689	MGYA00380802	https://www.ebi.ac.uk/metagenomics/api/v1/analyses/MGYA00380802/file/SRR6757689_MERGED_FASTQ_SSU_OTU.tsv	Root-Host_associated-Human-Skin
SRR995598	MGYA00025210	https://www.ebi.ac.uk/metagenomics/api/v1/analyses/MGYA00025210/file/SRR995598_FASTQ_otu.tsv	Root-Host_associated-Human-Skin
ERR451087	MGYA00421426	https://www.ebi.ac.uk/metagenomics/api/v1/analyses/MGYA00421426/file/ERR451087_FASTQ_SSU_OTU.tsv	Root-Host_associated-Human-Skin
ERR1759926	MGYA00140500	https://www.ebi.ac.uk/metagenomics/api/v1/analyses/MGYA00140500/file/ERR1759926_MERGED_FASTQ_SSU_OTU.tsv	Root-Host_associated-Human-Skin
SRR1950770	MGYA00376937	https://www.ebi.ac.uk/metagenomics/api/v1/analyses/MGYA00376937/file/SRR1950770_MERGED_FASTQ_SSU_OTU.tsv	Root-Host_associated-Human-Skin
SRR920194	MGYA00023267	https://www.ebi.ac.uk/metagenomics/api/v1/analyses/MGYA00023267/file/SRR920194_FASTQ_otu.tsv	Root-Host_associated-Human-Skin
SRR1633135	MGYA00024878	https://www.ebi.ac.uk/metagenomics/api/v1/analyses/MGYA00024878/file/SRR1633135_MERGED_FASTQ_otu.tsv	Root-Host_associated-Human-Skin
ERR442950	MGYA00416706	https://www.ebi.ac.uk/metagenomics/api/v1/analyses/MGYA00416706/file/ERR442950_FASTQ_SSU_OTU.tsv	Root-Host_associated-Human-Skin
SRR921073	MGYA00022630	https://www.ebi.ac.uk/metagenomics/api/v1/analyses/MGYA00022630/file/SRR921073_FASTQ_otu.tsv	Root-Host_associated-Human-Skin
ERR2538521	MGYA00381528	https://www.ebi.ac.uk/metagenomics/api/v1/analyses/MGYA00381528/file/ERR2538521_FASTQ_SSU_OTU.tsv	Root-Host_associated-Human-Skin

SRR1631660	MGYA00023144	https://www.ebi.ac.uk/metagenomics/api/v1/analyses/MGYA00023144/file/SRR1631660_MERGED_FASTQ_otu.tsv	Root-Host_associated-Human-Skin
SRR1622923	MGYA00023795	https://www.ebi.ac.uk/metagenomics/api/v1/analyses/MGYA00023795/file/SRR1622923_MERGED_FASTQ_otu.tsv	Root-Host_associated-Human-Skin
SRR5869333	MGYA00414864	https://www.ebi.ac.uk/metagenomics/api/v1/analyses/MGYA00414864/file/SRR5869333_MERGED_FASTQ_SSU_OTU.tsv	Root-Host_associated-Human-Skin
ERR1760138	MGYA00140392	https://www.ebi.ac.uk/metagenomics/api/v1/analyses/MGYA00140392/file/ERR1760138_MERGED_FASTQ_SSU_OTU.tsv	Root-Host_associated-Human-Skin
SRR995128	MGYA00023925	https://www.ebi.ac.uk/metagenomics/api/v1/analyses/MGYA00023925/file/SRR995128_FASTQ_otu.tsv	Root-Host_associated-Human-Skin
ERR451390	MGYA00419492	https://www.ebi.ac.uk/metagenomics/api/v1/analyses/MGYA00419492/file/ERR451390_FASTQ_SSU_OTU.tsv	Root-Host_associated-Human-Skin
ERR1960674	MGYA00130734	https://www.ebi.ac.uk/metagenomics/api/v1/analyses/MGYA00130734/file/ERR1960674_MERGED_FASTQ_otu.tsv	Root-Host_associated-Human-Skin
SRR920956	MGYA00022785	https://www.ebi.ac.uk/metagenomics/api/v1/analyses/MGYA00022785/file/SRR920956_FASTQ_otu.tsv	Root-Host_associated-Human-Skin
SRR1632810	MGYA00022592	https://www.ebi.ac.uk/metagenomics/api/v1/analyses/MGYA00022592/file/SRR1632810_MERGED_FASTQ_otu.tsv	Root-Host_associated-Human-Skin
SRR1632462	MGYA00023131	https://www.ebi.ac.uk/metagenomics/api/v1/analyses/MGYA00023131/file/SRR1632462_MERGED_FASTQ_otu.tsv	Root-Host_associated-Human-Skin
SRR995549	MGYA00022749	https://www.ebi.ac.uk/metagenomics/api/v1/analyses/MGYA00022749/file/SRR995549_FASTQ_otu.tsv	Root-Host_associated-Human-Skin
SRR1923935	MGYA00028262	https://www.ebi.ac.uk/metagenomics/api/v1/analyses/MGYA00028262/file/SRR1923935_FASTQ_otu.tsv	Root-Host_associated-Human-Skin

SRR6757624	MGYA00380741	https://www.ebi.ac.uk/metagenomics/api/v1/analyses/MGYA00380741/file/SRR6757624_MERGED_FASTQ_SSU_OTU.tsv	Root-Host_associated-Human-Skin
ERR2538373	MGYA00381380	https://www.ebi.ac.uk/metagenomics/api/v1/analyses/MGYA00381380/file/ERR2538373_FASTQ_SSU_OTU.tsv	Root-Host_associated-Human-Skin
SRR5869217	MGYA00413472	https://www.ebi.ac.uk/metagenomics/api/v1/analyses/MGYA00413472/file/SRR5869217_MERGED_FASTQ_SSU_OTU.tsv	Root-Host_associated-Human-Skin
SRR996119	MGYA00025180	https://www.ebi.ac.uk/metagenomics/api/v1/analyses/MGYA00025180/file/SRR996119_FASTQ_otu.tsv	Root-Host_associated-Human-Skin
ERR1960750	MGYA00130757	https://www.ebi.ac.uk/metagenomics/api/v1/analyses/MGYA00130757/file/ERR1960750_MERGED_FASTQ_otu.tsv	Root-Host_associated-Human-Skin
SRR5869676	MGYA00416987	https://www.ebi.ac.uk/metagenomics/api/v1/analyses/MGYA00416987/file/SRR5869676_MERGED_FASTQ_SSU_OTU.tsv	Root-Host_associated-Human-Skin
SRR1920904	MGYA00028253	https://www.ebi.ac.uk/metagenomics/api/v1/analyses/MGYA00028253/file/SRR1920904_FASTQ_otu.tsv	Root-Host_associated-Human-Skin
SRR996578	MGYA00022411	https://www.ebi.ac.uk/metagenomics/api/v1/analyses/MGYA00022411/file/SRR996578_FASTQ_otu.tsv	Root-Host_associated-Human-Skin
SRR1631453	MGYA00025118	https://www.ebi.ac.uk/metagenomics/api/v1/analyses/MGYA00025118/file/SRR1631453_MERGED_FASTQ_otu.tsv	Root-Host_associated-Human-Skin
SRR1704497	MGYA00414640	https://www.ebi.ac.uk/metagenomics/api/v1/analyses/MGYA00414640/file/SRR1704497_FASTQ_SSU_OTU.tsv	Root-Host_associated-Human-Skin
SRR5868791	MGYA00427171	https://www.ebi.ac.uk/metagenomics/api/v1/analyses/MGYA00427171/file/SRR5868791_MERGED_FASTQ_SSU_OTU.tsv	Root-Host_associated-Human-Skin
ERR450732	MGYA00416744	https://www.ebi.ac.uk/metagenomics/api/v1/analyses/MGYA00416744/file/ERR450732_FASTQ_SSU_OTU.tsv	Root-Host_associated-Human-Skin

ERR443627	MGYA00428659	https://www.ebi.ac.uk/metagenomics/api/v1/analyses/MGYA00428659/file/ERR443627_FASTQ_SSU_OTU.tsv	Root-Host_associated-Human-Skin
SRR1631482	MGYA00024934	https://www.ebi.ac.uk/metagenomics/api/v1/analyses/MGYA00024934/file/SRR1631482_MERGED_FASTQ_otu.tsv	Root-Host_associated-Human-Skin
ERR1960781	MGYA00130884	https://www.ebi.ac.uk/metagenomics/api/v1/analyses/MGYA00130884/file/ERR1960781_MERGED_FASTQ_otu.tsv	Root-Host_associated-Human-Skin
ERR443246	MGYA00426080	https://www.ebi.ac.uk/metagenomics/api/v1/analyses/MGYA00426080/file/ERR443246_FASTQ_SSU_OTU.tsv	Root-Host_associated-Human-Skin
ERR1701222	MGYA00084305	https://www.ebi.ac.uk/metagenomics/api/v1/analyses/MGYA00084305/file/ERR1701222_FASTQ_otu.tsv	Root-Host_associated-Human-Skin
SRR1633011	MGYA00023953	https://www.ebi.ac.uk/metagenomics/api/v1/analyses/MGYA00023953/file/SRR1633011_MERGED_FASTQ_otu.tsv	Root-Host_associated-Human-Skin
SRR1923774	MGYA00028309	https://www.ebi.ac.uk/metagenomics/api/v1/analyses/MGYA00028309/file/SRR1923774_FASTQ_otu.tsv	Root-Host_associated-Human-Skin
ERR2538405	MGYA00381412	https://www.ebi.ac.uk/metagenomics/api/v1/analyses/MGYA00381412/file/ERR2538405_FASTQ_SSU_OTU.tsv	Root-Host_associated-Human-Skin
SRR1921021	MGYA00028248	https://www.ebi.ac.uk/metagenomics/api/v1/analyses/MGYA00028248/file/SRR1921021_FASTQ_otu.tsv	Root-Host_associated-Human-Skin
SRR5868618	MGYA00428099	https://www.ebi.ac.uk/metagenomics/api/v1/analyses/MGYA00428099/file/SRR5868618_MERGED_FASTQ_SSU_OTU.tsv	Root-Host_associated-Human-Skin
SRR919555	MGYA00024496	https://www.ebi.ac.uk/metagenomics/api/v1/analyses/MGYA00024496/file/SRR919555_FASTQ_otu.tsv	Root-Host_associated-Human-Skin
SRR919927	MGYA00025196	https://www.ebi.ac.uk/metagenomics/api/v1/analyses/MGYA00025196/file/SRR919927_FASTQ_otu.tsv	Root-Host_associated-Human-Skin

SRR1923998	MGYA00028265	https://www.ebi.ac.uk/metagenomics/api/v1/analyses/MGYA00028265/file/SRR1923998_FASTQ_otu.tsv	Root-Host_associated-Human-Skin
ERR442037	MGYA00384600	https://www.ebi.ac.uk/metagenomics/api/v1/analyses/MGYA00384600/file/ERR442037_FASTQ_SSU_OTU.tsv	Root-Host_associated-Human-Skin
SRR994871	MGYA00025264	https://www.ebi.ac.uk/metagenomics/api/v1/analyses/MGYA00025264/file/SRR994871_FASTQ_otu.tsv	Root-Host_associated-Human-Skin
SRR920589	MGYA00024247	https://www.ebi.ac.uk/metagenomics/api/v1/analyses/MGYA00024247/file/SRR920589_FASTQ_otu.tsv	Root-Host_associated-Human-Skin
SRR5869439	MGYA00429047	https://www.ebi.ac.uk/metagenomics/api/v1/analyses/MGYA00429047/file/SRR5869439_MERGED_FASTQ_SSU_OTU.tsv	Root-Host_associated-Human-Skin
SRR995622	MGYA00025220	https://www.ebi.ac.uk/metagenomics/api/v1/analyses/MGYA00025220/file/SRR995622_FASTQ_otu.tsv	Root-Host_associated-Human-Skin
SRR1624862	MGYA00024470	https://www.ebi.ac.uk/metagenomics/api/v1/analyses/MGYA00024470/file/SRR1624862_MERGED_FASTQ_otu.tsv	Root-Host_associated-Human-Skin
SRR1633149	MGYA00024399	https://www.ebi.ac.uk/metagenomics/api/v1/analyses/MGYA00024399/file/SRR1633149_MERGED_FASTQ_otu.tsv	Root-Host_associated-Human-Skin
SRR920139	MGYA00023577	https://www.ebi.ac.uk/metagenomics/api/v1/analyses/MGYA00023577/file/SRR920139_FASTQ_otu.tsv	Root-Host_associated-Human-Skin
SRR994714	MGYA00023644	https://www.ebi.ac.uk/metagenomics/api/v1/analyses/MGYA00023644/file/SRR994714_FASTQ_otu.tsv	Root-Host_associated-Human-Skin
SRR996041	MGYA00023071	https://www.ebi.ac.uk/metagenomics/api/v1/analyses/MGYA00023071/file/SRR996041_FASTQ_otu.tsv	Root-Host_associated-Human-Skin
ERR443009	MGYA00416328	https://www.ebi.ac.uk/metagenomics/api/v1/analyses/MGYA00416328/file/ERR443009_FASTQ_SSU_OTU.tsv	Root-Host_associated-Human-Skin

SRR995292	MGYA00023592	https://www.ebi.ac.uk/metagenomics/api/v1/analyses/MGYA00023592/file/SRR995292_FASTQ_otu.tsv	Root-Host_associated-Human-Skin
SRR995947	MGYA00024280	https://www.ebi.ac.uk/metagenomics/api/v1/analyses/MGYA00024280/file/SRR995947_FASTQ_otu.tsv	Root-Host_associated-Human-Skin
SRR996852	MGYA00022485	https://www.ebi.ac.uk/metagenomics/api/v1/analyses/MGYA00022485/file/SRR996852_FASTQ_otu.tsv	Root-Host_associated-Human-Skin
ERR2538374	MGYA00381381	https://www.ebi.ac.uk/metagenomics/api/v1/analyses/MGYA00381381/file/ERR2538374_FASTQ_SSU_OTU.tsv	Root-Host_associated-Human-Skin
SRR1623232	MGYA00024123	https://www.ebi.ac.uk/metagenomics/api/v1/analyses/MGYA00024123/file/SRR1623232_MERGED_FASTQ_otu.tsv	Root-Host_associated-Human-Skin
SRR6757623	MGYA00380740	https://www.ebi.ac.uk/metagenomics/api/v1/analyses/MGYA00380740/file/SRR6757623_MERGED_FASTQ_SSU_OTU.tsv	Root-Host_associated-Human-Skin
SRR5868822	MGYA00420755	https://www.ebi.ac.uk/metagenomics/api/v1/analyses/MGYA00420755/file/SRR5868822_MERGED_FASTQ_SSU_OTU.tsv	Root-Host_associated-Human-Skin
ERR1960757	MGYA00130828	https://www.ebi.ac.uk/metagenomics/api/v1/analyses/MGYA00130828/file/ERR1960757_MERGED_FASTQ_otu.tsv	Root-Host_associated-Human-Skin
ERR451514	MGYA00425759	https://www.ebi.ac.uk/metagenomics/api/v1/analyses/MGYA00425759/file/ERR451514_FASTQ_SSU_OTU.tsv	Root-Host_associated-Human-Skin
SRR5868796	MGYA00419731	https://www.ebi.ac.uk/metagenomics/api/v1/analyses/MGYA00419731/file/SRR5868796_MERGED_FASTQ_SSU_OTU.tsv	Root-Host_associated-Human-Skin
SRR1631454	MGYA00025125	https://www.ebi.ac.uk/metagenomics/api/v1/analyses/MGYA00025125/file/SRR1631454_MERGED_FASTQ_otu.tsv	Root-Host_associated-Human-Skin
SRR2005654	MGYA00381312	https://www.ebi.ac.uk/metagenomics/api/v1/analyses/MGYA00381312/file/SRR2005654_MERGED_FASTQ_SSU_OTU.tsv	Root-Host_associated-Human-Skin

SRR5869671	MGYA00421593	https://www.ebi.ac.uk/metagenomics/api/v1/analyses/MGYA00421593/file/SRR5869671_MERGED_FASTQ_SSU_OTU.tsv	Root-Host_associated-Human-Skin
SRR1920903	MGYA00028250	https://www.ebi.ac.uk/metagenomics/api/v1/analyses/MGYA00028250/file/SRR1920903_FASTQ_otu.tsv	Root-Host_associated-Human-Skin
SRR1922552	MGYA00028243	https://www.ebi.ac.uk/metagenomics/api/v1/analyses/MGYA00028243/file/SRR1922552_FASTQ_otu.tsv	Root-Host_associated-Human-Skin
ERR1960832	MGYA00130724	https://www.ebi.ac.uk/metagenomics/api/v1/analyses/MGYA00130724/file/ERR1960832_MERGED_FASTQ_otu.tsv	Root-Host_associated-Human-Skin
ERR443620	MGYA00415522	https://www.ebi.ac.uk/metagenomics/api/v1/analyses/MGYA00415522/file/ERR443620_FASTQ_SSU_OTU.tsv	Root-Host_associated-Human-Skin
SRR5869107	MGYA00416469	https://www.ebi.ac.uk/metagenomics/api/v1/analyses/MGYA00416469/file/SRR5869107_MERGED_FASTQ_SSU_OTU.tsv	Root-Host_associated-Human-Skin
SRR996209	MGYA00024177	https://www.ebi.ac.uk/metagenomics/api/v1/analyses/MGYA00024177/file/SRR996209_FASTQ_otu.tsv	Root-Host_associated-Human-Skin
SRR1631485	MGYA00024937	https://www.ebi.ac.uk/metagenomics/api/v1/analyses/MGYA00024937/file/SRR1631485_MERGED_FASTQ_otu.tsv	Root-Host_associated-Human-Skin
SRR1633016	MGYA00023960	https://www.ebi.ac.uk/metagenomics/api/v1/analyses/MGYA00023960/file/SRR1633016_MERGED_FASTQ_otu.tsv	Root-Host_associated-Human-Skin
SRR1923773	MGYA00028363	https://www.ebi.ac.uk/metagenomics/api/v1/analyses/MGYA00028363/file/SRR1923773_FASTQ_otu.tsv	Root-Host_associated-Human-Skin
ERR451262	MGYA00412989	https://www.ebi.ac.uk/metagenomics/api/v1/analyses/MGYA00412989/file/ERR451262_FASTQ_SSU_OTU.tsv	Root-Host_associated-Human-Skin
ERR1701225	MGYA00084331	https://www.ebi.ac.uk/metagenomics/api/v1/analyses/MGYA00084331/file/ERR1701225_FASTQ_otu.tsv	Root-Host_associated-Human-Skin

ERR1960786	MGYA00130831	https://www.ebi.ac.uk/metagenomics/api/v1/analyses/MGYA00130831/file/ERR1960786_MERGED_FASTQ_otu.tsv	Root-Host_associated-Human-Skin
ERR450850	MGYA00426824	https://www.ebi.ac.uk/metagenomics/api/v1/analyses/MGYA00426824/file/ERR450850_FASTQ_SSU_OTU.tsv	Root-Host_associated-Human-Skin
ERR2538402	MGYA00381409	https://www.ebi.ac.uk/metagenomics/api/v1/analyses/MGYA00381409/file/ERR2538402_FASTQ_SSU_OTU.tsv	Root-Host_associated-Human-Skin
SRR919920	MGYA00025197	https://www.ebi.ac.uk/metagenomics/api/v1/analyses/MGYA00025197/file/SRR919920_FASTQ_otu.tsv	Root-Host_associated-Human-Skin
SRR919552	MGYA00024503	https://www.ebi.ac.uk/metagenomics/api/v1/analyses/MGYA00024503/file/SRR919552_FASTQ_otu.tsv	Root-Host_associated-Human-Skin
SRR920229	MGYA00023282	https://www.ebi.ac.uk/metagenomics/api/v1/analyses/MGYA00023282/file/SRR920229_FASTQ_otu.tsv	Root-Host_associated-Human-Skin
SRR994404	MGYA00025138	https://www.ebi.ac.uk/metagenomics/api/v1/analyses/MGYA00025138/file/SRR994404_FASTQ_otu.tsv	Root-Host_associated-Human-Skin
SRR994876	MGYA00025271	https://www.ebi.ac.uk/metagenomics/api/v1/analyses/MGYA00025271/file/SRR994876_FASTQ_otu.tsv	Root-Host_associated-Human-Skin
ERR442030	MGYA00385216	https://www.ebi.ac.uk/metagenomics/api/v1/analyses/MGYA00385216/file/ERR442030_FASTQ_SSU_OTU.tsv	Root-Host_associated-Human-Skin
SRR1624865	MGYA00024465	https://www.ebi.ac.uk/metagenomics/api/v1/analyses/MGYA00024465/file/SRR1624865_MERGED_FASTQ_otu.tsv	Root-Host_associated-Human-Skin
SRR995625	MGYA00025225	https://www.ebi.ac.uk/metagenomics/api/v1/analyses/MGYA00025225/file/SRR995625_FASTQ_otu.tsv	Root-Host_associated-Human-Skin
ERR450908	MGYA00414283	https://www.ebi.ac.uk/metagenomics/api/v1/analyses/MGYA00414283/file/ERR450908_FASTQ_SSU_OTU.tsv	Root-Host_associated-Human-Skin

SRR994713	MGYA00025093	https://www.ebi.ac.uk/metagenomics/api/v1/analyses/MGYA00025093/file/SRR994713_FASTQ_otu.tsv	Root-Host_associated-Human-Skin
SRR920699	MGYA00024849	https://www.ebi.ac.uk/metagenomics/api/v1/analyses/MGYA00024849/file/SRR920699_FASTQ_otu.tsv	Root-Host_associated-Human-Skin
SRR995940	MGYA00024285	https://www.ebi.ac.uk/metagenomics/api/v1/analyses/MGYA00024285/file/SRR995940_FASTQ_otu.tsv	Root-Host_associated-Human-Skin
SRR995295	MGYA00023599	https://www.ebi.ac.uk/metagenomics/api/v1/analyses/MGYA00023599/file/SRR995295_FASTQ_otu.tsv	Root-Host_associated-Human-Skin
SRR1632419	MGYA00023810	https://www.ebi.ac.uk/metagenomics/api/v1/analyses/MGYA00023810/file/SRR1632419_MERGED_FASTQ_otu.tsv	Root-Host_associated-Human-Skin
SRR996855	MGYA00023219	https://www.ebi.ac.uk/metagenomics/api/v1/analyses/MGYA00023219/file/SRR996855_FASTQ_otu.tsv	Root-Host_associated-Human-Skin
ERR1701288	MGYA00084322	https://www.ebi.ac.uk/metagenomics/api/v1/analyses/MGYA00084322/file/ERR1701288_FASTQ_otu.tsv	Root-Host_associated-Human-Skin
SRR994637	MGYA00022542	https://www.ebi.ac.uk/metagenomics/api/v1/analyses/MGYA00022542/file/SRR994637_FASTQ_otu.tsv	Root-Host_associated-Human-Skin
ERR1760067	MGYA00140554	https://www.ebi.ac.uk/metagenomics/api/v1/analyses/MGYA00140554/file/ERR1760067_MERGED_FASTQ_SSU_OTU.tsv	Root-Host_associated-Human-Skin
SRR995077	MGYA00024645	https://www.ebi.ac.uk/metagenomics/api/v1/analyses/MGYA00024645/file/SRR995077_FASTQ_otu.tsv	Root-Host_associated-Human-Skin
SRR996162	MGYA00022293	https://www.ebi.ac.uk/metagenomics/api/v1/analyses/MGYA00022293/file/SRR996162_FASTQ_otu.tsv	Root-Host_associated-Human-Skin
SRR1631049	MGYA00025016	https://www.ebi.ac.uk/metagenomics/api/v1/analyses/MGYA00025016/file/SRR1631049_MERGED_FASTQ_otu.tsv	Root-Host_associated-Human-Skin

SRR995416	MGYA00024930	https://www.ebi.ac.uk/metagenomics/api/v1/analyses/MGYA00024930/file/SRR995416_FASTQ_otu.tsv	Root-Host_associated-Human-Skin
SRR1623188	MGYA00023680	https://www.ebi.ac.uk/metagenomics/api/v1/analyses/MGYA00023680/file/SRR1623188_MERGED_FASTQ_otu.tsv	Root-Host_associated-Human-Skin
SRR6757599	MGYA00380718	https://www.ebi.ac.uk/metagenomics/api/v1/analyses/MGYA00380718/file/SRR6757599_MERGED_FASTQ_SSU_OTU.tsv	Root-Host_associated-Human-Skin
SRR996971	MGYA00022647	https://www.ebi.ac.uk/metagenomics/api/v1/analyses/MGYA00022647/file/SRR996971_FASTQ_otu.tsv	Root-Host_associated-Human-Skin
SRR994952	MGYA00024335	https://www.ebi.ac.uk/metagenomics/api/v1/analyses/MGYA00024335/file/SRR994952_FASTQ_otu.tsv	Root-Host_associated-Human-Skin
SRR994520	MGYA00024052	https://www.ebi.ac.uk/metagenomics/api/v1/analyses/MGYA00024052/file/SRR994520_FASTQ_otu.tsv	Root-Host_associated-Human-Skin
SRR995360	MGYA00022919	https://www.ebi.ac.uk/metagenomics/api/v1/analyses/MGYA00022919/file/SRR995360_FASTQ_otu.tsv	Root-Host_associated-Human-Skin
SRR997054	MGYA00022970	https://www.ebi.ac.uk/metagenomics/api/v1/analyses/MGYA00022970/file/SRR997054_FASTQ_otu.tsv	Root-Host_associated-Human-Skin
ERR1701259	MGYA00084296	https://www.ebi.ac.uk/metagenomics/api/v1/analyses/MGYA00084296/file/ERR1701259_FASTQ_otu.tsv	Root-Host_associated-Human-Skin
SRR994478	MGYA00022245	https://www.ebi.ac.uk/metagenomics/api/v1/analyses/MGYA00022245/file/SRR994478_FASTQ_otu.tsv	Root-Host_associated-Human-Skin
SRR1950777	MGYA00376944	https://www.ebi.ac.uk/metagenomics/api/v1/analyses/MGYA00376944/file/SRR1950777_MERGED_FASTQ_SSU_OTU.tsv	Root-Host_associated-Human-Skin
ERR1759921	MGYA00140435	https://www.ebi.ac.uk/metagenomics/api/v1/analyses/MGYA00140435/file/ERR1759921_MERGED_FASTQ_SSU_OTU.tsv	Root-Host_associated-Human-Skin

SRR5868663	MGYA00423071	https://www.ebi.ac.uk/metagenomics/api/v1/analyses/MGYA00423071/file/SRR5868663_MERGED_FASTQ_SSU_OTU.tsv	Root-Host_associated-Human-Skin
SRR1704565	MGYA00421476	https://www.ebi.ac.uk/metagenomics/api/v1/analyses/MGYA00421476/file/SRR1704565_FASTQ_SSU_OTU.tsv	Root-Host_associated-Human-Skin
SRR921074	MGYA00022633	https://www.ebi.ac.uk/metagenomics/api/v1/analyses/MGYA00022633/file/SRR921074_FASTQ_otu.tsv	Root-Host_associated-Human-Skin
ERR451346	MGYA00428403	https://www.ebi.ac.uk/metagenomics/api/v1/analyses/MGYA00428403/file/ERR451346_FASTQ_SSU_OTU.tsv	Root-Host_associated-Human-Skin
ERR450974	MGYA00415556	https://www.ebi.ac.uk/metagenomics/api/v1/analyses/MGYA00415556/file/ERR450974_FASTQ_SSU_OTU.tsv	Root-Host_associated-Human-Skin
ERR443365	MGYA00428739	https://www.ebi.ac.uk/metagenomics/api/v1/analyses/MGYA00428739/file/ERR443365_FASTQ_SSU_OTU.tsv	Root-Host_associated-Human-Skin
ERR1701301	MGYA00084310	https://www.ebi.ac.uk/metagenomics/api/v1/analyses/MGYA00084310/file/ERR1701301_FASTQ_otu.tsv	Root-Host_associated-Human-Skin
SRR920193	MGYA00023264	https://www.ebi.ac.uk/metagenomics/api/v1/analyses/MGYA00023264/file/SRR920193_FASTQ_otu.tsv	Root-Host_associated-Human-Skin
SRR1631667	MGYA00023153	https://www.ebi.ac.uk/metagenomics/api/v1/analyses/MGYA00023153/file/SRR1631667_MERGED_FASTQ_otu.tsv	Root-Host_associated-Human-Skin
ERR2538526	MGYA00381533	https://www.ebi.ac.uk/metagenomics/api/v1/analyses/MGYA00381533/file/ERR2538526_FASTQ_SSU_OTU.tsv	Root-Host_associated-Human-Skin
SRR1622924	MGYA00023794	https://www.ebi.ac.uk/metagenomics/api/v1/analyses/MGYA00023794/file/SRR1622924_MERGED_FASTQ_otu.tsv	Root-Host_associated-Human-Skin
SRR919639	MGYA00023762	https://www.ebi.ac.uk/metagenomics/api/v1/analyses/MGYA00023762/file/SRR919639_FASTQ_otu.tsv	Root-Host_associated-Human-Skin

SRR1631111	MGYA00022869	https://www.ebi.ac.uk/metagenomics/api/v1/analyses/MGYA00022869/file/SRR1631111_MERGED_FASTQ_otu.tsv	Root-Host_associated-Human-Skin
SRR1704672	MGYA00422984	https://www.ebi.ac.uk/metagenomics/api/v1/analyses/MGYA00422984/file/SRR1704672_FASTQ_SSU_OTU.tsv	Root-Host_associated-Human-Skin
ERR1960673	MGYA00130853	https://www.ebi.ac.uk/metagenomics/api/v1/analyses/MGYA00130853/file/ERR1960673_MERGED_FASTQ_otu.tsv	Root-Host_associated-Human-Skin
ERR443413	MGYA00428722	https://www.ebi.ac.uk/metagenomics/api/v1/analyses/MGYA00428722/file/ERR443413_FASTQ_SSU_OTU.tsv	Root-Host_associated-Human-Skin
ERR442986	MGYA00420163	https://www.ebi.ac.uk/metagenomics/api/v1/analyses/MGYA00420163/file/ERR442986_FASTQ_SSU_OTU.tsv	Root-Host_associated-Human-Skin
ERR451430	MGYA00426008	https://www.ebi.ac.uk/metagenomics/api/v1/analyses/MGYA00426008/file/ERR451430_FASTQ_SSU_OTU.tsv	Root-Host_associated-Human-Skin
SRR1923932	MGYA00028257	https://www.ebi.ac.uk/metagenomics/api/v1/analyses/MGYA00028257/file/SRR1923932_FASTQ_otu.tsv	Root-Host_associated-Human-Skin
SRR1632465	MGYA00023140	https://www.ebi.ac.uk/metagenomics/api/v1/analyses/MGYA00023140/file/SRR1632465_MERGED_FASTQ_otu.tsv	Root-Host_associated-Human-Skin
SRR1632817	MGYA00022595	https://www.ebi.ac.uk/metagenomics/api/v1/analyses/MGYA00022595/file/SRR1632817_MERGED_FASTQ_otu.tsv	Root-Host_associated-Human-Skin
SRR920951	MGYA00022786	https://www.ebi.ac.uk/metagenomics/api/v1/analyses/MGYA00022786/file/SRR920951_FASTQ_otu.tsv	Root-Host_associated-Human-Skin
SRR5869869	MGYA00415858	https://www.ebi.ac.uk/metagenomics/api/v1/analyses/MGYA00415858/file/SRR5869869_MERGED_FASTQ_SSU_OTU.tsv	Root-Host_associated-Human-Skin
SRR1624840	MGYA00024291	https://www.ebi.ac.uk/metagenomics/api/v1/analyses/MGYA00024291/file/SRR1624840_MERGED_FASTQ_otu.tsv	Root-Host_associated-Human-Skin

SRR995600	MGYA00025041	https://www.ebi.ac.uk/metagenomics/api/v1/analyses/MGYA00025041/file/SRR995600_FASTQ_otu.tsv	Root-Host_associated-Human-Skin
ERR1551330	MGYA00069426	https://www.ebi.ac.uk/metagenomics/api/v1/analyses/MGYA00069426/file/ERR1551330_MERGED_FASTQ_otu.tsv	Root-Host_associated-Human-Skin
SRR919577	MGYA00024726	https://www.ebi.ac.uk/metagenomics/api/v1/analyses/MGYA00024726/file/SRR919577_FASTQ_otu.tsv	Root-Host_associated-Human-Skin
SRR919905	MGYA00025027	https://www.ebi.ac.uk/metagenomics/api/v1/analyses/MGYA00025027/file/SRR919905_FASTQ_otu.tsv	Root-Host_associated-Human-Skin
ERR1759978	MGYA00140376	https://www.ebi.ac.uk/metagenomics/api/v1/analyses/MGYA00140376/file/ERR1759978_MERGED_FASTQ_SSU_OTU.tsv	Root-Host_associated-Human-Skin
SRR994421	MGYA00024961	https://www.ebi.ac.uk/metagenomics/api/v1/analyses/MGYA00024961/file/SRR994421_FASTQ_otu.tsv	Root-Host_associated-Human-Skin
ERR442015	MGYA00385209	https://www.ebi.ac.uk/metagenomics/api/v1/analyses/MGYA00385209/file/ERR442015_FASTQ_SSU_OTU.tsv	Root-Host_associated-Human-Skin
SRR995965	MGYA00024451	https://www.ebi.ac.uk/metagenomics/api/v1/analyses/MGYA00024451/file/SRR995965_FASTQ_otu.tsv	Root-Host_associated-Human-Skin
SRR994882	MGYA00022188	https://www.ebi.ac.uk/metagenomics/api/v1/analyses/MGYA00022188/file/SRR994882_FASTQ_otu.tsv	Root-Host_associated-Human-Skin
SRR996870	MGYA00023441	https://www.ebi.ac.uk/metagenomics/api/v1/analyses/MGYA00023441/file/SRR996870_FASTQ_otu.tsv	Root-Host_associated-Human-Skin
SRR996063	MGYA00023317	https://www.ebi.ac.uk/metagenomics/api/v1/analyses/MGYA00023317/file/SRR996063_FASTQ_otu.tsv	Root-Host_associated-Human-Skin
SRR996928	MGYA00025135	https://www.ebi.ac.uk/metagenomics/api/v1/analyses/MGYA00025135/file/SRR996928_FASTQ_otu.tsv	Root-Host_associated-Human-Skin

SRR2005671	MGYA00381328	https://www.ebi.ac.uk/metagenomics/api/v1/analyses/MGYA00381328/file/SRR2005671_MERGED_FASTQ_SSU_OTU.tsv	Root-Host_associated-Human-Skin
ERR450710	MGYA00413025	https://www.ebi.ac.uk/metagenomics/api/v1/analyses/MGYA00413025/file/ERR450710_FASTQ_SSU_OTU.tsv	Root-Host_associated-Human-Skin
ERR2538351	MGYA00381358	https://www.ebi.ac.uk/metagenomics/api/v1/analyses/MGYA00381358/file/ERR2538351_FASTQ_SSU_OTU.tsv	Root-Host_associated-Human-Skin
SRR6757606	MGYA00380725	https://www.ebi.ac.uk/metagenomics/api/v1/analyses/MGYA00380725/file/SRR6757606_MERGED_FASTQ_SSU_OTU.tsv	Root-Host_associated-Human-Skin
SRR5868807	MGYA00427155	https://www.ebi.ac.uk/metagenomics/api/v1/analyses/MGYA00427155/file/SRR5868807_MERGED_FASTQ_SSU_OTU.tsv	Root-Host_associated-Human-Skin
SRR1623217	MGYA00023912	https://www.ebi.ac.uk/metagenomics/api/v1/analyses/MGYA00023912/file/SRR1623217_MERGED_FASTQ_otu.tsv	Root-Host_associated-Human-Skin
ERR1960772	MGYA00130903	https://www.ebi.ac.uk/metagenomics/api/v1/analyses/MGYA00130903/file/ERR1960772_MERGED_FASTQ_otu.tsv	Root-Host_associated-Human-Skin
ERR443512	MGYA00428704	https://www.ebi.ac.uk/metagenomics/api/v1/analyses/MGYA00428704/file/ERR443512_FASTQ_SSU_OTU.tsv	Root-Host_associated-Human-Skin
SRR1923787	MGYA00028233	https://www.ebi.ac.uk/metagenomics/api/v1/analyses/MGYA00028233/file/SRR1923787_FASTQ_otu.tsv	Root-Host_associated-Human-Skin
SRR1633033	MGYA00024154	https://www.ebi.ac.uk/metagenomics/api/v1/analyses/MGYA00024154/file/SRR1633033_MERGED_FASTQ_otu.tsv	Root-Host_associated-Human-Skin
SRR920735	MGYA00025017	https://www.ebi.ac.uk/metagenomics/api/v1/analyses/MGYA00025017/file/SRR920735_FASTQ_otu.tsv	Root-Host_associated-Human-Skin
ERR2538380	MGYA00381387	https://www.ebi.ac.uk/metagenomics/api/v1/analyses/MGYA00381387/file/ERR2538380_FASTQ_SSU_OTU.tsv	Root-Host_associated-Human-Skin

SRR5869543	MGYA00417536	https://www.ebi.ac.uk/metagenomics/api/v1/analyses/MGYA00417536/file/SRR5869543_MERGED_FASTQ_SSU_OTU.tsv	Root-Host_associated-Human-Skin
ERR2538427	MGYA00381434	https://www.ebi.ac.uk/metagenomics/api/v1/analyses/MGYA00381434/file/ERR2538427_FASTQ_SSU_OTU.tsv	Root-Host_associated-Human-Skin
ERR443605	MGYA00421388	https://www.ebi.ac.uk/metagenomics/api/v1/analyses/MGYA00421388/file/ERR443605_FASTQ_SSU_OTU.tsv	Root-Host_associated-Human-Skin
ERR1960817	MGYA00130891	https://www.ebi.ac.uk/metagenomics/api/v1/analyses/MGYA00130891/file/ERR1960817_MERGED_FASTQ_otu.tsv	Root-Host_associated-Human-Skin
SRR1630935	MGYA00023967	https://www.ebi.ac.uk/metagenomics/api/v1/analyses/MGYA00023967/file/SRR1630935_MERGED_FASTQ_otu.tsv	Root-Host_associated-Human-Skin
SRR1631307	MGYA00023227	https://www.ebi.ac.uk/metagenomics/api/v1/analyses/MGYA00023227/file/SRR1631307_MERGED_FASTQ_otu.tsv	Root-Host_associated-Human-Skin
ERR442972	MGYA00421100	https://www.ebi.ac.uk/metagenomics/api/v1/analyses/MGYA00421100/file/ERR442972_FASTQ_SSU_OTU.tsv	Root-Host_associated-Human-Skin
SRR921051	MGYA00022863	https://www.ebi.ac.uk/metagenomics/api/v1/analyses/MGYA00022863/file/SRR921051_FASTQ_otu.tsv	Root-Host_associated-Human-Skin
ERR451363	MGYA00420124	https://www.ebi.ac.uk/metagenomics/api/v1/analyses/MGYA00420124/file/ERR451363_FASTQ_SSU_OTU.tsv	Root-Host_associated-Human-Skin
ERR1960687	MGYA00130857	https://www.ebi.ac.uk/metagenomics/api/v1/analyses/MGYA00130857/file/ERR1960687_MERGED_FASTQ_otu.tsv	Root-Host_associated-Human-Skin
ERR2538503	MGYA00381510	https://www.ebi.ac.uk/metagenomics/api/v1/analyses/MGYA00381510/file/ERR2538503_FASTQ_SSU_OTU.tsv	Root-Host_associated-Human-Skin
SRR5869467	MGYA00419665	https://www.ebi.ac.uk/metagenomics/api/v1/analyses/MGYA00419665/file/SRR5869467_MERGED_FASTQ_SSU_OTU.tsv	Root-Host_associated-Human-Skin

SRR5869815	MGYA00425838	https://www.ebi.ac.uk/metagenomics/api/v1/analyses/MGYA00425838/file/SRR5869815_MERGED_FASTQ_SSU_OTU.tsv	Root-Host_associated-Human-Skin
SRR996769	MGYA00023854	https://www.ebi.ac.uk/metagenomics/api/v1/analyses/MGYA00023854/file/SRR996769_FASTQ_otu.tsv	Root-Host_associated-Human-Skin
SRR1632336	MGYA00024581	https://www.ebi.ac.uk/metagenomics/api/v1/analyses/MGYA00024581/file/SRR1632336_MERGED_FASTQ_otu.tsv	Root-Host_associated-Human-Skin
SRR1624788	MGYA00022544	https://www.ebi.ac.uk/metagenomics/api/v1/analyses/MGYA00022544/file/SRR1624788_MERGED_FASTQ_otu.tsv	Root-Host_associated-Human-Skin
SRR1950752	MGYA00376919	https://www.ebi.ac.uk/metagenomics/api/v1/analyses/MGYA00376919/file/SRR1950752_MERGED_FASTQ_SSU_OTU.tsv	Root-Host_associated-Human-Skin
SRR996308	MGYA00022705	https://www.ebi.ac.uk/metagenomics/api/v1/analyses/MGYA00022705/file/SRR996308_FASTQ_otu.tsv	Root-Host_associated-Human-Skin
SRR5868646	MGYA00421225	https://www.ebi.ac.uk/metagenomics/api/v1/analyses/MGYA00421225/file/SRR5868646_MERGED_FASTQ_SSU_OTU.tsv	Root-Host_associated-Human-Skin
SRR1704540	MGYA00428835	https://www.ebi.ac.uk/metagenomics/api/v1/analyses/MGYA00428835/file/SRR1704540_FASTQ_SSU_OTU.tsv	Root-Host_associated-Human-Skin
ERR1759904	MGYA00140573	https://www.ebi.ac.uk/metagenomics/api/v1/analyses/MGYA00140573/file/ERR1759904_MERGED_FASTQ_SSU_OTU.tsv	Root-Host_associated-Human-Skin
SRR1704591	MGYA00424936	https://www.ebi.ac.uk/metagenomics/api/v1/analyses/MGYA00424936/file/SRR1704591_FASTQ_SSU_OTU.tsv	Root-Host_associated-Human-Skin
SRR5868697	MGYA00420808	https://www.ebi.ac.uk/metagenomics/api/v1/analyses/MGYA00420808/file/SRR5868697_MERGED_FASTQ_SSU_OTU.tsv	Root-Host_associated-Human-Skin
SRR1631555	MGYA00023800	https://www.ebi.ac.uk/metagenomics/api/v1/analyses/MGYA00023800/file/SRR1631555_MERGED_FASTQ_otu.tsv	Root-Host_associated-Human-Skin

SRR1950783	MGYA00376950	https://www.ebi.ac.uk/metagenomics/api/v1/analyses/MGYA00376950/file/SRR1950783_MERGED_FASTQ_SSU_OTU.tsv	Root-Host_associated-Human-Skin
SRR1923917	MGYA00028297	https://www.ebi.ac.uk/metagenomics/api/v1/analyses/MGYA00028297/file/SRR1923917_FASTQ_otu.tsv	Root-Host_associated-Human-Skin
SRR920974	MGYA00023043	https://www.ebi.ac.uk/metagenomics/api/v1/analyses/MGYA00023043/file/SRR920974_FASTQ_otu.tsv	Root-Host_associated-Human-Skin
SRR1632832	MGYA00022820	https://www.ebi.ac.uk/metagenomics/api/v1/analyses/MGYA00022820/file/SRR1632832_MERGED_FASTQ_otu.tsv	Root-Host_associated-Human-Skin
SRR1622901	MGYA00022364	https://www.ebi.ac.uk/metagenomics/api/v1/analyses/MGYA00022364/file/SRR1622901_MERGED_FASTQ_otu.tsv	Root-Host_associated-Human-Skin
ERR443436	MGYA00428398	https://www.ebi.ac.uk/metagenomics/api/v1/analyses/MGYA00428398/file/ERR443436_FASTQ_SSU_OTU.tsv	Root-Host_associated-Human-Skin
ERR443391	MGYA00426033	https://www.ebi.ac.uk/metagenomics/api/v1/analyses/MGYA00426033/file/ERR443391_FASTQ_SSU_OTU.tsv	Root-Host_associated-Human-Skin
ERR1960656	MGYA00130859	https://www.ebi.ac.uk/metagenomics/api/v1/analyses/MGYA00130859/file/ERR1960656_MERGED_FASTQ_otu.tsv	Root-Host_associated-Human-Skin
ERR451415	MGYA00421402	https://www.ebi.ac.uk/metagenomics/api/v1/analyses/MGYA00421402/file/ERR451415_FASTQ_SSU_OTU.tsv	Root-Host_associated-Human-Skin
SRR995394	MGYA00024757	https://www.ebi.ac.uk/metagenomics/api/v1/analyses/MGYA00024757/file/SRR995394_FASTQ_otu.tsv	Root-Host_associated-Human-Skin
SRR996954	MGYA00022870	https://www.ebi.ac.uk/metagenomics/api/v1/analyses/MGYA00022870/file/SRR996954_FASTQ_otu.tsv	Root-Host_associated-Human-Skin
SRR5869628	MGYA00414839	https://www.ebi.ac.uk/metagenomics/api/v1/analyses/MGYA00414839/file/SRR5869628_MERGED_FASTQ_SSU_OTU.tsv	Root-Host_associated-Human-Skin

SRR996281	MGYA00023309	https://www.ebi.ac.uk/metagenomics/api/v1/analyses/MGYA00023309/file/SRR996281_FASTQ_otu.tsv	Root-Host_associated-Human-Skin
SRR5870022	MGYA00418715	https://www.ebi.ac.uk/metagenomics/api/v1/analyses/MGYA00418715/file/SRR5870022_MERGED_FASTQ_SSU_OTU.tsv	Root-Host_associated-Human-Skin
SRR994612	MGYA00023015	https://www.ebi.ac.uk/metagenomics/api/v1/analyses/MGYA00023015/file/SRR994612_FASTQ_otu.tsv	Root-Host_associated-Human-Skin
ERR1760042	MGYA00140550	https://www.ebi.ac.uk/metagenomics/api/v1/analyses/MGYA00140550/file/ERR1760042_MERGED_FASTQ_SSU_OTU.tsv	Root-Host_associated-Human-Skin
SRR996147	MGYA00022528	https://www.ebi.ac.uk/metagenomics/api/v1/analyses/MGYA00022528/file/SRR996147_FASTQ_otu.tsv	Root-Host_associated-Human-Skin
SRR5869249	MGYA00422540	https://www.ebi.ac.uk/metagenomics/api/v1/analyses/MGYA00422540/file/SRR5869249_MERGED_FASTQ_SSU_OTU.tsv	Root-Host_associated-Human-Skin
SRR997071	MGYA00023159	https://www.ebi.ac.uk/metagenomics/api/v1/analyses/MGYA00023159/file/SRR997071_FASTQ_otu.tsv	Root-Host_associated-Human-Skin
ERR1760093	MGYA00140433	https://www.ebi.ac.uk/metagenomics/api/v1/analyses/MGYA00140433/file/ERR1760093_MERGED_FASTQ_SSU_OTU.tsv	Root-Host_associated-Human-Skin
SRR995083	MGYA00022715	https://www.ebi.ac.uk/metagenomics/api/v1/analyses/MGYA00022715/file/SRR995083_FASTQ_otu.tsv	Root-Host_associated-Human-Skin
SRR921109	MGYA00024997	https://www.ebi.ac.uk/metagenomics/api/v1/analyses/MGYA00024997/file/SRR921109_FASTQ_otu.tsv	Root-Host_associated-Human-Skin
SRR996985	MGYA00024532	https://www.ebi.ac.uk/metagenomics/api/v1/analyses/MGYA00024532/file/SRR996985_FASTQ_otu.tsv	Root-Host_associated-Human-Skin
SRR994505	MGYA00023819	https://www.ebi.ac.uk/metagenomics/api/v1/analyses/MGYA00023819/file/SRR994505_FASTQ_otu.tsv	Root-Host_associated-Human-Skin

SRR994977	MGYA00024557	https://www.ebi.ac.uk/metagenomics/api/v1/analyses/MGYA00024557/file/SRR994977_FASTQ_otu.tsv	Root-Host_associated-Human-Skin
SRR995345	MGYA00023119	https://www.ebi.ac.uk/metagenomics/api/v1/analyses/MGYA00023119/file/SRR995345_FASTQ_otu.tsv	Root-Host_associated-Human-Skin
ERR1960680	MGYA00130784	https://www.ebi.ac.uk/metagenomics/api/v1/analyses/MGYA00130784/file/ERR1960680_MERGED_FASTQ_otu.tsv	Root-Host_associated-Human-Skin
ERR443347	MGYA00426047	https://www.ebi.ac.uk/metagenomics/api/v1/analyses/MGYA00426047/file/ERR443347_FASTQ_SSU_OTU.tsv	Root-Host_associated-Human-Skin
SRR921056	MGYA00022858	https://www.ebi.ac.uk/metagenomics/api/v1/analyses/MGYA00022858/file/SRR921056_FASTQ_otu.tsv	Root-Host_associated-Human-Skin
ERR2538504	MGYA00381511	https://www.ebi.ac.uk/metagenomics/api/v1/analyses/MGYA00381511/file/ERR2538504_FASTQ_SSU_OTU.tsv	Root-Host_associated-Human-Skin
SRR5869812	MGYA00415875	https://www.ebi.ac.uk/metagenomics/api/v1/analyses/MGYA00415875/file/SRR5869812_MERGED_FASTQ_SSU_OTU.tsv	Root-Host_associated-Human-Skin
SRR5869460	MGYA00419667	https://www.ebi.ac.uk/metagenomics/api/v1/analyses/MGYA00419667/file/SRR5869460_MERGED_FASTQ_SSU_OTU.tsv	Root-Host_associated-Human-Skin
ERR441968	MGYA00385250	https://www.ebi.ac.uk/metagenomics/api/v1/analyses/MGYA00385250/file/ERR441968_FASTQ_SSU_OTU.tsv	Root-Host_associated-Human-Skin
SRR994828	MGYA00022826	https://www.ebi.ac.uk/metagenomics/api/v1/analyses/MGYA00022826/file/SRR994828_FASTQ_otu.tsv	Root-Host_associated-Human-Skin
SRR1632331	MGYA00024570	https://www.ebi.ac.uk/metagenomics/api/v1/analyses/MGYA00024570/file/SRR1632331_MERGED_FASTQ_otu.tsv	Root-Host_associated-Human-Skin
ERR1759903	MGYA00140429	https://www.ebi.ac.uk/metagenomics/api/v1/analyses/MGYA00140429/file/ERR1759903_MERGED_FASTQ_SSU_OTU.tsv	Root-Host_associated-Human-Skin

SRR5868641	MGYA00428073	https://www.ebi.ac.uk/metagenomics/api/v1/analyses/MGYA00428073/file/SRR5868641_MERGED_FASTQ_SSU_OTU.tsv	Root-Host_associated-Human-Skin
SRR1950755	MGYA00376922	https://www.ebi.ac.uk/metagenomics/api/v1/analyses/MGYA00376922/file/SRR1950755_MERGED_FASTQ_SSU_OTU.tsv	Root-Host_associated-Human-Skin
SRR996479	MGYA00024912	https://www.ebi.ac.uk/metagenomics/api/v1/analyses/MGYA00024912/file/SRR996479_FASTQ_otu.tsv	Root-Host_associated-Human-Skin
SRR1950784	MGYA00376951	https://www.ebi.ac.uk/metagenomics/api/v1/analyses/MGYA00376951/file/SRR1950784_MERGED_FASTQ_SSU_OTU.tsv	Root-Host_associated-Human-Skin
SRR1631552	MGYA00023801	https://www.ebi.ac.uk/metagenomics/api/v1/analyses/MGYA00023801/file/SRR1631552_MERGED_FASTQ_otu.tsv	Root-Host_associated-Human-Skin
SRR1704596	MGYA00414320	https://www.ebi.ac.uk/metagenomics/api/v1/analyses/MGYA00414320/file/SRR1704596_FASTQ_SSU_OTU.tsv	Root-Host_associated-Human-Skin
SRR1632835	MGYA00022815	https://www.ebi.ac.uk/metagenomics/api/v1/analyses/MGYA00022815/file/SRR1632835_MERGED_FASTQ_otu.tsv	Root-Host_associated-Human-Skin
ERR443050	MGYA00426532	https://www.ebi.ac.uk/metagenomics/api/v1/analyses/MGYA00426532/file/ERR443050_FASTQ_SSU_OTU.tsv	Root-Host_associated-Human-Skin
SRR920973	MGYA00023036	https://www.ebi.ac.uk/metagenomics/api/v1/analyses/MGYA00023036/file/SRR920973_FASTQ_otu.tsv	Root-Host_associated-Human-Skin
SRR1923910	MGYA00028298	https://www.ebi.ac.uk/metagenomics/api/v1/analyses/MGYA00028298/file/SRR1923910_FASTQ_otu.tsv	Root-Host_associated-Human-Skin
SRR5869316	MGYA00412813	https://www.ebi.ac.uk/metagenomics/api/v1/analyses/MGYA00412813/file/SRR5869316_MERGED_FASTQ_SSU_OTU.tsv	Root-Host_associated-Human-Skin
SRR1622906	MGYA00022362	https://www.ebi.ac.uk/metagenomics/api/v1/analyses/MGYA00022362/file/SRR1622906_MERGED_FASTQ_otu.tsv	Root-Host_associated-Human-Skin

ERR1960651	MGYA00130899	https://www.ebi.ac.uk/metagenomics/api/v1/analyses/MGYA00130899/file/ERR1960651_MERGED_FASTQ_otu.tsv	Root-Host_associated-Human-Skin
ERR450987	MGYA00413012	https://www.ebi.ac.uk/metagenomics/api/v1/analyses/MGYA00413012/file/ERR450987_FASTQ_SSU_OTU.tsv	Root-Host_associated-Human-Skin
ERR443431	MGYA00412979	https://www.ebi.ac.uk/metagenomics/api/v1/analyses/MGYA00412979/file/ERR443431_FASTQ_SSU_OTU.tsv	Root-Host_associated-Human-Skin
SRR995393	MGYA00024758	https://www.ebi.ac.uk/metagenomics/api/v1/analyses/MGYA00024758/file/SRR995393_FASTQ_otu.tsv	Root-Host_associated-Human-Skin
SRR996286	MGYA00023306	https://www.ebi.ac.uk/metagenomics/api/v1/analyses/MGYA00023306/file/SRR996286_FASTQ_otu.tsv	Root-Host_associated-Human-Skin
SRR996953	MGYA00022877	https://www.ebi.ac.uk/metagenomics/api/v1/analyses/MGYA00022877/file/SRR996953_FASTQ_otu.tsv	Root-Host_associated-Human-Skin
ERR1760045	MGYA00140491	https://www.ebi.ac.uk/metagenomics/api/v1/analyses/MGYA00140491/file/ERR1760045_MERGED_FASTQ_SSU_OTU.tsv	Root-Host_associated-Human-Skin
ERR1960709	MGYA00130798	https://www.ebi.ac.uk/metagenomics/api/v1/analyses/MGYA00130798/file/ERR1960709_MERGED_FASTQ_otu.tsv	Root-Host_associated-Human-Skin
ERR443569	MGYA00424288	https://www.ebi.ac.uk/metagenomics/api/v1/analyses/MGYA00424288/file/ERR443569_FASTQ_SSU_OTU.tsv	Root-Host_associated-Human-Skin
SRR994615	MGYA00023016	https://www.ebi.ac.uk/metagenomics/api/v1/analyses/MGYA00023016/file/SRR994615_FASTQ_otu.tsv	Root-Host_associated-Human-Skin
SRR5870025	MGYA00413454	https://www.ebi.ac.uk/metagenomics/api/v1/analyses/MGYA00413454/file/SRR5870025_MERGED_FASTQ_SSU_OTU.tsv	Root-Host_associated-Human-Skin
SRR1704708	MGYA00421463	https://www.ebi.ac.uk/metagenomics/api/v1/analyses/MGYA00421463/file/SRR1704708_FASTQ_SSU_OTU.tsv	Root-Host_associated-Human-Skin

SRR996140	MGYA00022527	https://www.ebi.ac.uk/metagenomics/api/v1/analyses/MGYA00022527/file/SRR996140_FASTQ_otu.tsv	Root-Host_associated-Human-Skin
ERR981251	MGYA00010232	https://www.ebi.ac.uk/metagenomics/api/v1/analyses/MGYA00010232/file/ERR981251_MERGED_FASTQ_otu.tsv	Root-Host_associated-Human-Skin
SRR997076	MGYA00023164	https://www.ebi.ac.uk/metagenomics/api/v1/analyses/MGYA00023164/file/SRR997076_FASTQ_otu.tsv	Root-Host_associated-Human-Skin
ERR1760094	MGYA00140416	https://www.ebi.ac.uk/metagenomics/api/v1/analyses/MGYA00140416/file/ERR1760094_MERGED_FASTQ_SSU_OTU.tsv	Root-Host_associated-Human-Skin
SRR995084	MGYA00022723	https://www.ebi.ac.uk/metagenomics/api/v1/analyses/MGYA00022723/file/SRR995084_FASTQ_otu.tsv	Root-Host_associated-Human-Skin
SRR5869159	MGYA00420264	https://www.ebi.ac.uk/metagenomics/api/v1/analyses/MGYA00420264/file/SRR5869159_MERGED_FASTQ_SSU_OTU.tsv	Root-Host_associated-Human-Skin
SRR996982	MGYA00024535	https://www.ebi.ac.uk/metagenomics/api/v1/analyses/MGYA00024535/file/SRR996982_FASTQ_otu.tsv	Root-Host_associated-Human-Skin
SRR995342	MGYA00023124	https://www.ebi.ac.uk/metagenomics/api/v1/analyses/MGYA00023124/file/SRR995342_FASTQ_otu.tsv	Root-Host_associated-Human-Skin
SRR1923899	MGYA00028365	https://www.ebi.ac.uk/metagenomics/api/v1/analyses/MGYA00028365/file/SRR1923899_FASTQ_otu.tsv	Root-Host_associated-Human-Skin
SRR994970	MGYA00024550	https://www.ebi.ac.uk/metagenomics/api/v1/analyses/MGYA00024550/file/SRR994970_FASTQ_otu.tsv	Root-Host_associated-Human-Skin
SRR994502	MGYA00023818	https://www.ebi.ac.uk/metagenomics/api/v1/analyses/MGYA00023818/file/SRR994502_FASTQ_otu.tsv	Root-Host_associated-Human-Skin
SRR995607	MGYA00025038	https://www.ebi.ac.uk/metagenomics/api/v1/analyses/MGYA00025038/file/SRR995607_FASTQ_otu.tsv	Root-Host_associated-Human-Skin

SRR1624847	MGYA00024294	https://www.ebi.ac.uk/metagenomics/api/v1/analyses/MGYA00024294/file/SRR1624847_MERGED_FASTQ_otu.tsv	Root-Host_associated-Human-Skin
SRR1631258	MGYA00024539	https://www.ebi.ac.uk/metagenomics/api/v1/analyses/MGYA00024539/file/SRR1631258_MERGED_FASTQ_otu.tsv	Root-Host_associated-Human-Skin
SRR1950729	MGYA00376896	https://www.ebi.ac.uk/metagenomics/api/v1/analyses/MGYA00376896/file/SRR1950729_MERGED_FASTQ_SSU_OTU.tsv	Root-Host_associated-Human-Skin
SRR919902	MGYA00025028	https://www.ebi.ac.uk/metagenomics/api/v1/analyses/MGYA00025028/file/SRR919902_FASTQ_otu.tsv	Root-Host_associated-Human-Skin
SRR919570	MGYA00024725	https://www.ebi.ac.uk/metagenomics/api/v1/analyses/MGYA00024725/file/SRR919570_FASTQ_otu.tsv	Root-Host_associated-Human-Skin
ERR442012	MGYA00385180	https://www.ebi.ac.uk/metagenomics/api/v1/analyses/MGYA00385180/file/ERR442012_FASTQ_SSU_OTU.tsv	Root-Host_associated-Human-Skin
SRR994426	MGYA00024962	https://www.ebi.ac.uk/metagenomics/api/v1/analyses/MGYA00024962/file/SRR994426_FASTQ_otu.tsv	Root-Host_associated-Human-Skin
SRR994885	MGYA00022183	https://www.ebi.ac.uk/metagenomics/api/v1/analyses/MGYA00022183/file/SRR994885_FASTQ_otu.tsv	Root-Host_associated-Human-Skin
SRR995962	MGYA00023852	https://www.ebi.ac.uk/metagenomics/api/v1/analyses/MGYA00023852/file/SRR995962_FASTQ_otu.tsv	Root-Host_associated-Human-Skin
SRR996877	MGYA00023434	https://www.ebi.ac.uk/metagenomics/api/v1/analyses/MGYA00023434/file/SRR996877_FASTQ_otu.tsv	Root-Host_associated-Human-Skin
SRR1631289	MGYA00025251	https://www.ebi.ac.uk/metagenomics/api/v1/analyses/MGYA00025251/file/SRR1631289_MERGED_FASTQ_otu.tsv	Root-Host_associated-Human-Skin
SRR5870101	MGYA00416417	https://www.ebi.ac.uk/metagenomics/api/v1/analyses/MGYA00416417/file/SRR5870101_MERGED_FASTQ_SSU_OTU.tsv	Root-Host_associated-Human-Skin

SRR994731	MGYA00023474	https://www.ebi.ac.uk/metagenomics/api/v1/analyses/MGYA00023474/file/SRR994731_FASTQ_otu.tsv	Root-Host_associated-Human-Skin
SRR5868958	MGYA00412818	https://www.ebi.ac.uk/metagenomics/api/v1/analyses/MGYA00412818/file/SRR5868958_MERGED_FASTQ_SSU_OTU.tsv	Root-Host_associated-Human-Skin
SRR996064	MGYA00023318	https://www.ebi.ac.uk/metagenomics/api/v1/analyses/MGYA00023318/file/SRR996064_FASTQ_otu.tsv	Root-Host_associated-Human-Skin
SRR2005676	MGYA00381332	https://www.ebi.ac.uk/metagenomics/api/v1/analyses/MGYA00381332/file/SRR2005676_MERGED_FASTQ_SSU_OTU.tsv	Root-Host_associated-Human-Skin
SRR5869653	MGYA00416999	https://www.ebi.ac.uk/metagenomics/api/v1/analyses/MGYA00416999/file/SRR5869653_MERGED_FASTQ_SSU_OTU.tsv	Root-Host_associated-Human-Skin
ERR443174	MGYA00414545	https://www.ebi.ac.uk/metagenomics/api/v1/analyses/MGYA00414545/file/ERR443174_FASTQ_SSU_OTU.tsv	Root-Host_associated-Human-Skin
ERR450717	MGYA00423896	https://www.ebi.ac.uk/metagenomics/api/v1/analyses/MGYA00423896/file/ERR450717_FASTQ_SSU_OTU.tsv	Root-Host_associated-Human-Skin
SRR1623210	MGYA00023909	https://www.ebi.ac.uk/metagenomics/api/v1/analyses/MGYA00023909/file/SRR1623210_MERGED_FASTQ_otu.tsv	Root-Host_associated-Human-Skin
SRR6757601	MGYA00380720	https://www.ebi.ac.uk/metagenomics/api/v1/analyses/MGYA00380720/file/SRR6757601_MERGED_FASTQ_SSU_OTU.tsv	Root-Host_associated-Human-Skin
ERR2538356	MGYA00381363	https://www.ebi.ac.uk/metagenomics/api/v1/analyses/MGYA00381363/file/ERR2538356_FASTQ_SSU_OTU.tsv	Root-Host_associated-Human-Skin
ERR1760039	MGYA00140461	https://www.ebi.ac.uk/metagenomics/api/v1/analyses/MGYA00140461/file/ERR1760039_MERGED_FASTQ_SSU_OTU.tsv	Root-Host_associated-Human-Skin
SRR995029	MGYA00023941	https://www.ebi.ac.uk/metagenomics/api/v1/analyses/MGYA00023941/file/SRR995029_FASTQ_otu.tsv	Root-Host_associated-Human-Skin

ERR451536	MGYA00414535	https://www.ebi.ac.uk/metagenomics/api/v1/analyses/MGYA00414535/file/ERR451536_FASTQ_SSU_OTU.tsv	Root-Host_associated-Human-Skin
ERR451291	MGYA00419505	https://www.ebi.ac.uk/metagenomics/api/v1/analyses/MGYA00419505/file/ERR451291_FASTQ_SSU_OTU.tsv	Root-Host_associated-Human-Skin
ERR1960775	MGYA00130760	https://www.ebi.ac.uk/metagenomics/api/v1/analyses/MGYA00130760/file/ERR1960775_MERGED_FASTQ_otu.tsv	Root-Host_associated-Human-Skin
ERR1701207	MGYA00084274	https://www.ebi.ac.uk/metagenomics/api/v1/analyses/MGYA00084274/file/ERR1701207_FASTQ_otu.tsv	Root-Host_associated-Human-Skin
SRR920732	MGYA00025032	https://www.ebi.ac.uk/metagenomics/api/v1/analyses/MGYA00025032/file/SRR920732_FASTQ_otu.tsv	Root-Host_associated-Human-Skin
SRR5869936	MGYA00424482	https://www.ebi.ac.uk/metagenomics/api/v1/analyses/MGYA00424482/file/SRR5869936_MERGED_FASTQ_SSU_OTU.tsv	Root-Host_associated-Human-Skin
ERR2538420	MGYA00381427	https://www.ebi.ac.uk/metagenomics/api/v1/analyses/MGYA00381427/file/ERR2538420_FASTQ_SSU_OTU.tsv	Root-Host_associated-Human-Skin
ERR2538387	MGYA00381394	https://www.ebi.ac.uk/metagenomics/api/v1/analyses/MGYA00381394/file/ERR2538387_FASTQ_SSU_OTU.tsv	Root-Host_associated-Human-Skin
SRR995499	MGYA00024120	https://www.ebi.ac.uk/metagenomics/api/v1/analyses/MGYA00024120/file/SRR995499_FASTQ_otu.tsv	Root-Host_associated-Human-Skin
ERR451186	MGYA00416674	https://www.ebi.ac.uk/metagenomics/api/v1/analyses/MGYA00416674/file/ERR451186_FASTQ_SSU_OTU.tsv	Root-Host_associated-Human-Skin
ERR1960810	MGYA00130842	https://www.ebi.ac.uk/metagenomics/api/v1/analyses/MGYA00130842/file/ERR1960810_MERGED_FASTQ_otu.tsv	Root-Host_associated-Human-Skin
SRR1922570	MGYA00028338	https://www.ebi.ac.uk/metagenomics/api/v1/analyses/MGYA00028338/file/SRR1922570_FASTQ_otu.tsv	Root-Host_associated-Human-Skin

ERR443602	MGYA00428665	https://www.ebi.ac.uk/metagenomics/api/v1/analyses/MGYA00428665/file/ERR443602_FASTQ_SSU_OTU.tsv	Root-Host_associated-Human-Skin
SRR1631300	MGYA00022521	https://www.ebi.ac.uk/metagenomics/api/v1/analyses/MGYA00022521/file/SRR1631300_MERGED_FASTQ_otu.tsv	Root-Host_associated-Human-Skin
SRR996760	MGYA00023859	https://www.ebi.ac.uk/metagenomics/api/v1/analyses/MGYA00023859/file/SRR996760_FASTQ_otu.tsv	Root-Host_associated-Human-Skin
ERR441966	MGYA00385169	https://www.ebi.ac.uk/metagenomics/api/v1/analyses/MGYA00385169/file/ERR441966_FASTQ_SSU_OTU.tsv	Root-Host_associated-Human-Skin
ERR443349	MGYA00412378	https://www.ebi.ac.uk/metagenomics/api/v1/analyses/MGYA00412378/file/ERR443349_FASTQ_SSU_OTU.tsv	Root-Host_associated-Human-Skin
SRR994792	MGYA00022834	https://www.ebi.ac.uk/metagenomics/api/v1/analyses/MGYA00022834/file/SRR994792_FASTQ_otu.tsv	Root-Host_associated-Human-Skin
SRR996301	MGYA00022711	https://www.ebi.ac.uk/metagenomics/api/v1/analyses/MGYA00022711/file/SRR996301_FASTQ_otu.tsv	Root-Host_associated-Human-Skin
ERR443728	MGYA00414252	https://www.ebi.ac.uk/metagenomics/api/v1/analyses/MGYA00414252/file/ERR443728_FASTQ_SSU_OTU.tsv	Root-Host_associated-Human-Skin
SRR994454	MGYA00022462	https://www.ebi.ac.uk/metagenomics/api/v1/analyses/MGYA00022462/file/SRR994454_FASTQ_otu.tsv	Root-Host_associated-Human-Skin
ERR442060	MGYA00385178	https://www.ebi.ac.uk/metagenomics/api/v1/analyses/MGYA00385178/file/ERR442060_FASTQ_SSU_OTU.tsv	Root-Host_associated-Human-Skin
SRR995562	MGYA00022980	https://www.ebi.ac.uk/metagenomics/api/v1/analyses/MGYA00022980/file/SRR995562_FASTQ_otu.tsv	Root-Host_associated-Human-Skin
SRR994485	MGYA00023143	https://www.ebi.ac.uk/metagenomics/api/v1/analyses/MGYA00023143/file/SRR994485_FASTQ_otu.tsv	Root-Host_associated-Human-Skin

SRR1632449	MGYA00023104	https://www.ebi.ac.uk/metagenomics/api/v1/analyses/MGYA00023104/file/SRR1632449_MERGED_FASTQ_otu.tsv	Root-Host_associated-Human-Skin
ERR1760113	MGYA00140385	https://www.ebi.ac.uk/metagenomics/api/v1/analyses/MGYA00140385/file/ERR1760113_MERGED_FASTQ_SSU_OTU.tsv	Root-Host_associated-Human-Skin
SRR995103	MGYA00023665	https://www.ebi.ac.uk/metagenomics/api/v1/analyses/MGYA00023665/file/SRR995103_FASTQ_otu.tsv	Root-Host_associated-Human-Skin
SRR5869318	MGYA00412491	https://www.ebi.ac.uk/metagenomics/api/v1/analyses/MGYA00412491/file/SRR5869318_MERGED_FASTQ_SSU_OTU.tsv	Root-Host_associated-Human-Skin
SRR919615	MGYA00023531	https://www.ebi.ac.uk/metagenomics/api/v1/analyses/MGYA00023531/file/SRR919615_FASTQ_otu.tsv	Root-Host_associated-Human-Skin
SRR1622908	MGYA00022377	https://www.ebi.ac.uk/metagenomics/api/v1/analyses/MGYA00022377/file/SRR1622908_MERGED_FASTQ_otu.tsv	Root-Host_associated-Human-Skin
SRR1704367	MGYA00421499	https://www.ebi.ac.uk/metagenomics/api/v1/analyses/MGYA00421499/file/SRR1704367_FASTQ_SSU_OTU.tsv	Root-Host_associated-Human-Skin
ERR1759884	MGYA00140359	https://www.ebi.ac.uk/metagenomics/api/v1/analyses/MGYA00140359/file/ERR1759884_MERGED_FASTQ_SSU_OTU.tsv	Root-Host_associated-Human-Skin
SRR996288	MGYA00023302	https://www.ebi.ac.uk/metagenomics/api/v1/analyses/MGYA00023302/file/SRR996288_FASTQ_otu.tsv	Root-Host_associated-Human-Skin
SRR1630991	MGYA00022849	https://www.ebi.ac.uk/metagenomics/api/v1/analyses/MGYA00022849/file/SRR1630991_MERGED_FASTQ_otu.tsv	Root-Host_associated-Human-Skin
ERR2538483	MGYA00381490	https://www.ebi.ac.uk/metagenomics/api/v1/analyses/MGYA00381490/file/ERR2538483_FASTQ_SSU_OTU.tsv	Root-Host_associated-Human-Skin
SRR1622850	MGYA00024387	https://www.ebi.ac.uk/metagenomics/api/v1/analyses/MGYA00024387/file/SRR1622850_MERGED_FASTQ_otu.tsv	Root-Host_associated-Human-Skin

SRR6757673	MGYA00380786	https://www.ebi.ac.uk/metagenomics/api/v1/analyses/MGYA00380786/file/SRR6757673_MERGED_FASTQ_SSU_OTU.tsv	Root-Host_associated-Human-Skin
SRR1631065	MGYA00024809	https://www.ebi.ac.uk/metagenomics/api/v1/analyses/MGYA00024809/file/SRR1631065_MERGED_FASTQ_otu.tsv	Root-Host_associated-Human-Skin
ERR1960707	MGYA00130790	https://www.ebi.ac.uk/metagenomics/api/v1/analyses/MGYA00130790/file/ERR1960707_MERGED_FASTQ_otu.tsv	Root-Host_associated-Human-Skin
SRR921100	MGYA00025004	https://www.ebi.ac.uk/metagenomics/api/v1/analyses/MGYA00025004/file/SRR921100_FASTQ_otu.tsv	Root-Host_associated-Human-Skin
SRR920740	MGYA00023536	https://www.ebi.ac.uk/metagenomics/api/v1/analyses/MGYA00023536/file/SRR920740_FASTQ_otu.tsv	Root-Host_associated-Human-Skin
ERR1701275	MGYA00084285	https://www.ebi.ac.uk/metagenomics/api/v1/analyses/MGYA00084285/file/ERR1701275_FASTQ_otu.tsv	Root-Host_associated-Human-Skin
SRR997078	MGYA00023156	https://www.ebi.ac.uk/metagenomics/api/v1/analyses/MGYA00023156/file/SRR997078_FASTQ_otu.tsv	Root-Host_associated-Human-Skin
SRR1631713	MGYA00024042	https://www.ebi.ac.uk/metagenomics/api/v1/analyses/MGYA00024042/file/SRR1631713_MERGED_FASTQ_otu.tsv	Root-Host_associated-Human-Skin
SRR5869291	MGYA00415360	https://www.ebi.ac.uk/metagenomics/api/v1/analyses/MGYA00415360/file/SRR5869291_MERGED_FASTQ_SSU_OTU.tsv	Root-Host_associated-Human-Skin
SRR5869944	MGYA00416936	https://www.ebi.ac.uk/metagenomics/api/v1/analyses/MGYA00416936/file/SRR5869944_MERGED_FASTQ_SSU_OTU.tsv	Root-Host_associated-Human-Skin
ERR2538452	MGYA00381459	https://www.ebi.ac.uk/metagenomics/api/v1/analyses/MGYA00381459/file/ERR2538452_FASTQ_SSU_OTU.tsv	Root-Host_associated-Human-Skin
SRR1923897	MGYA00028373	https://www.ebi.ac.uk/metagenomics/api/v1/analyses/MGYA00028373/file/SRR1923897_FASTQ_otu.tsv	Root-Host_associated-Human-Skin

SRR5869157	MGYA00429110	https://www.ebi.ac.uk/metagenomics/api/v1/analyses/MGYA00429110/file/SRR5869157_MERGED_FASTQ_SSU_OTU.tsv	Root-Host_associated-Human-Skin
SRR1704411	MGYA00417455	https://www.ebi.ac.uk/metagenomics/api/v1/analyses/MGYA00417455/file/SRR1704411_FASTQ_SSU_OTU.tsv	Root-Host_associated-Human-Skin
SRR5868717	MGYA00421206	https://www.ebi.ac.uk/metagenomics/api/v1/analyses/MGYA00421206/file/SRR5868717_MERGED_FASTQ_SSU_OTU.tsv	Root-Host_associated-Human-Skin
SRR1633162	MGYA00022387	https://www.ebi.ac.uk/metagenomics/api/v1/analyses/MGYA00022387/file/SRR1633162_MERGED_FASTQ_otu.tsv	Root-Host_associated-Human-Skin
SRR995609	MGYA00025045	https://www.ebi.ac.uk/metagenomics/api/v1/analyses/MGYA00025045/file/SRR995609_FASTQ_otu.tsv	Root-Host_associated-Human-Skin
SRR1624849	MGYA00024288	https://www.ebi.ac.uk/metagenomics/api/v1/analyses/MGYA00024288/file/SRR1624849_MERGED_FASTQ_otu.tsv	Root-Host_associated-Human-Skin
SRR920664	MGYA00022336	https://www.ebi.ac.uk/metagenomics/api/v1/analyses/MGYA00022336/file/SRR920664_FASTQ_otu.tsv	Root-Host_associated-Human-Skin
SRR1632343	MGYA00025167	https://www.ebi.ac.uk/metagenomics/api/v1/analyses/MGYA00025167/file/SRR1632343_MERGED_FASTQ_otu.tsv	Root-Host_associated-Human-Skin
ERR450690	MGYA00426871	https://www.ebi.ac.uk/metagenomics/api/v1/analyses/MGYA00426871/file/ERR450690_FASTQ_SSU_OTU.tsv	Root-Host_associated-Human-Skin
SRR994428	MGYA00024966	https://www.ebi.ac.uk/metagenomics/api/v1/analyses/MGYA00024966/file/SRR994428_FASTQ_otu.tsv	Root-Host_associated-Human-Skin
SRR920205	MGYA00023060	https://www.ebi.ac.uk/metagenomics/api/v1/analyses/MGYA00023060/file/SRR920205_FASTQ_otu.tsv	Root-Host_associated-Human-Skin
SRR1950727	MGYA00376894	https://www.ebi.ac.uk/metagenomics/api/v1/analyses/MGYA00376894/file/SRR1950727_MERGED_FASTQ_SSU_OTU.tsv	Root-Host_associated-Human-Skin

SRR1631256	MGYA00024547	https://www.ebi.ac.uk/metagenomics/api/v1/analyses/MGYA00024547/file/SRR1631256_MERGED_FASTQ_otu.tsv	Root-Host_associated-Human-Skin
SRR5868633	MGYA00417164	https://www.ebi.ac.uk/metagenomics/api/v1/analyses/MGYA00417164/file/SRR5868633_MERGED_FASTQ_SSU_OTU.tsv	Root-Host_associated-Human-Skin
ERR1759971	MGYA00140561	https://www.ebi.ac.uk/metagenomics/api/v1/analyses/MGYA00140561/file/ERR1759971_MERGED_FASTQ_SSU_OTU.tsv	Root-Host_associated-Human-Skin
SRR1631287	MGYA00022657	https://www.ebi.ac.uk/metagenomics/api/v1/analyses/MGYA00022657/file/SRR1631287_MERGED_FASTQ_otu.tsv	Root-Host_associated-Human-Skin
SRR1623080	MGYA00025044	https://www.ebi.ac.uk/metagenomics/api/v1/analyses/MGYA00025044/file/SRR1623080_MERGED_FASTQ_otu.tsv	Root-Host_associated-Human-Skin
SRR1704243	MGYA00426996	https://www.ebi.ac.uk/metagenomics/api/v1/analyses/MGYA00426996/file/SRR1704243_FASTQ_SSU_OTU.tsv	Root-Host_associated-Human-Skin
SRR2005720	MGYA00381349	https://www.ebi.ac.uk/metagenomics/api/v1/analyses/MGYA00381349/file/SRR2005720_MERGED_FASTQ_SSU_OTU.tsv	Root-Host_associated-Human-Skin
SRR996879	MGYA00023442	https://www.ebi.ac.uk/metagenomics/api/v1/analyses/MGYA00023442/file/SRR996879_FASTQ_otu.tsv	Root-Host_associated-Human-Skin
SRR920901	MGYA00024100	https://www.ebi.ac.uk/metagenomics/api/v1/analyses/MGYA00024100/file/SRR920901_FASTQ_otu.tsv	Root-Host_associated-Human-Skin
ERR443785	MGYA00424255	https://www.ebi.ac.uk/metagenomics/api/v1/analyses/MGYA00424255/file/ERR443785_FASTQ_SSU_OTU.tsv	Root-Host_associated-Human-Skin
SRR1704622	MGYA00421133	https://www.ebi.ac.uk/metagenomics/api/v1/analyses/MGYA00421133/file/SRR1704622_FASTQ_SSU_OTU.tsv	Root-Host_associated-Human-Skin
SRR920112	MGYA00024127	https://www.ebi.ac.uk/metagenomics/api/v1/analyses/MGYA00024127/file/SRR920112_FASTQ_otu.tsv	Root-Host_associated-Human-Skin

ERR443831	MGYA00428371	https://www.ebi.ac.uk/metagenomics/api/v1/analyses/MGYA00428371/file/ERR443831_FASTQ_SSU_OTU.tsv	Root-Host_associated-Human-Skin
ERR450719	MGYA00426544	https://www.ebi.ac.uk/metagenomics/api/v1/analyses/MGYA00426544/file/ERR450719_FASTQ_SSU_OTU.tsv	Root-Host_associated-Human-Skin
SRR2005678	MGYA00381333	https://www.ebi.ac.uk/metagenomics/api/v1/analyses/MGYA00381333/file/SRR2005678_MERGED_FASTQ_SSU_OTU.tsv	Root-Host_associated-Human-Skin
SRR996553	MGYA00022620	https://www.ebi.ac.uk/metagenomics/api/v1/analyses/MGYA00022620/file/SRR996553_FASTQ_otu.tsv	Root-Host_associated-Human-Skin
SRR996921	MGYA00025126	https://www.ebi.ac.uk/metagenomics/api/v1/analyses/MGYA00025126/file/SRR996921_FASTQ_otu.tsv	Root-Host_associated-Human-Skin
SRR919885	MGYA00024200	https://www.ebi.ac.uk/metagenomics/api/v1/analyses/MGYA00024200/file/SRR919885_FASTQ_otu.tsv	Root-Host_associated-Human-Skin
SRR3217223	MGYA00412196	https://www.ebi.ac.uk/metagenomics/api/v1/analyses/MGYA00412196/file/SRR3217223_FASTQ_SSU_OTU.tsv	Root-Host_associated-Human-Skin
SRR5870057	MGYA00412472	https://www.ebi.ac.uk/metagenomics/api/v1/analyses/MGYA00412472/file/SRR5870057_MERGED_FASTQ_SSU_OTU.tsv	Root-Host_associated-Human-Skin
SRR995027	MGYA00023947	https://www.ebi.ac.uk/metagenomics/api/v1/analyses/MGYA00023947/file/SRR995027_FASTQ_otu.tsv	Root-Host_associated-Human-Skin
ERR1760037	MGYA00140379	https://www.ebi.ac.uk/metagenomics/api/v1/analyses/MGYA00140379/file/ERR1760037_MERGED_FASTQ_SSU_OTU.tsv	Root-Host_associated-Human-Skin
SRR996132	MGYA00024989	https://www.ebi.ac.uk/metagenomics/api/v1/analyses/MGYA00024989/file/SRR996132_FASTQ_otu.tsv	Root-Host_associated-Human-Skin
SRR996695	MGYA00022288	https://www.ebi.ac.uk/metagenomics/api/v1/analyses/MGYA00022288/file/SRR996695_FASTQ_otu.tsv	Root-Host_associated-Human-Skin

ERR2538358	MGYA00381365	https://www.ebi.ac.uk/metagenomics/api/v1/analyses/MGYA00381365/file/ERR2538358_FASTQ_SSU_OTU.tsv	Root-Host_associated-Human-Skin
ERR2538389	MGYA00381396	https://www.ebi.ac.uk/metagenomics/api/v1/analyses/MGYA00381396/file/ERR2538389_FASTQ_SSU_OTU.tsv	Root-Host_associated-Human-Skin
SRR995751	MGYA00022895	https://www.ebi.ac.uk/metagenomics/api/v1/analyses/MGYA00022895/file/SRR995751_FASTQ_otu.tsv	Root-Host_associated-Human-Skin
ERR1701209	MGYA00084273	https://www.ebi.ac.uk/metagenomics/api/v1/analyses/MGYA00084273/file/ERR1701209_FASTQ_otu.tsv	Root-Host_associated-Human-Skin
SRR996225	MGYA00023974	https://www.ebi.ac.uk/metagenomics/api/v1/analyses/MGYA00023974/file/SRR996225_FASTQ_otu.tsv	Root-Host_associated-Human-Skin
SRR995497	MGYA00024118	https://www.ebi.ac.uk/metagenomics/api/v1/analyses/MGYA00024118/file/SRR995497_FASTQ_otu.tsv	Root-Host_associated-Human-Skin
SRR920663	MGYA00023393	https://www.ebi.ac.uk/metagenomics/api/v1/analyses/MGYA00023393/file/SRR920663_FASTQ_otu.tsv	Root-Host_associated-Human-Skin
ERR451311	MGYA00413676	https://www.ebi.ac.uk/metagenomics/api/v1/analyses/MGYA00413676/file/ERR451311_FASTQ_SSU_OTU.tsv	Root-Host_associated-Human-Skin
SRR1633165	MGYA00022380	https://www.ebi.ac.uk/metagenomics/api/v1/analyses/MGYA00022380/file/SRR1633165_MERGED_FASTQ_otu.tsv	Root-Host_associated-Human-Skin
SRR5869415	MGYA00422113	https://www.ebi.ac.uk/metagenomics/api/v1/analyses/MGYA00422113/file/SRR5869415_MERGED_FASTQ_SSU_OTU.tsv	Root-Host_associated-Human-Skin
SRR920202	MGYA00023059	https://www.ebi.ac.uk/metagenomics/api/v1/analyses/MGYA00023059/file/SRR920202_FASTQ_otu.tsv	Root-Host_associated-Human-Skin
SRR1632344	MGYA00025164	https://www.ebi.ac.uk/metagenomics/api/v1/analyses/MGYA00025164/file/SRR1632344_MERGED_FASTQ_otu.tsv	Root-Host_associated-Human-Skin

ERR1759976	MGYA00140394	https://www.ebi.ac.uk/metagenomics/api/v1/analyses/MGYA00140394/file/ERR1759976_MERGED_FASTQ_SSU_OTU.tsv	Root-Host_associated-Human-Skin
SRR1631251	MGYA00024540	https://www.ebi.ac.uk/metagenomics/api/v1/analyses/MGYA00024540/file/SRR1631251_MERGED_FASTQ_otu.tsv	Root-Host_associated-Human-Skin
SRR5868634	MGYA00417162	https://www.ebi.ac.uk/metagenomics/api/v1/analyses/MGYA00417162/file/SRR5868634_MERGED_FASTQ_SSU_OTU.tsv	Root-Host_associated-Human-Skin
SRR919579	MGYA00024718	https://www.ebi.ac.uk/metagenomics/api/v1/analyses/MGYA00024718/file/SRR919579_FASTQ_otu.tsv	Root-Host_associated-Human-Skin
SRR1950720	MGYA00376887	https://www.ebi.ac.uk/metagenomics/api/v1/analyses/MGYA00376887/file/SRR1950720_MERGED_FASTQ_SSU_OTU.tsv	Root-Host_associated-Human-Skin
SRR5869702	MGYA00416980	https://www.ebi.ac.uk/metagenomics/api/v1/analyses/MGYA00416980/file/SRR5869702_MERGED_FASTQ_SSU_OTU.tsv	Root-Host_associated-Human-Skin
SRR2005727	MGYA00381355	https://www.ebi.ac.uk/metagenomics/api/v1/analyses/MGYA00381355/file/SRR2005727_MERGED_FASTQ_SSU_OTU.tsv	Root-Host_associated-Human-Skin
SRR1631280	MGYA00022662	https://www.ebi.ac.uk/metagenomics/api/v1/analyses/MGYA00022662/file/SRR1631280_MERGED_FASTQ_otu.tsv	Root-Host_associated-Human-Skin
ERR443782	MGYA00415091	https://www.ebi.ac.uk/metagenomics/api/v1/analyses/MGYA00415091/file/ERR443782_FASTQ_SSU_OTU.tsv	Root-Host_associated-Human-Skin
SRR920906	MGYA00024102	https://www.ebi.ac.uk/metagenomics/api/v1/analyses/MGYA00024102/file/SRR920906_FASTQ_otu.tsv	Root-Host_associated-Human-Skin
SRR1923965	MGYA00028376	https://www.ebi.ac.uk/metagenomics/api/v1/analyses/MGYA00028376/file/SRR1923965_FASTQ_otu.tsv	Root-Host_associated-Human-Skin
ERR451467	MGYA00412966	https://www.ebi.ac.uk/metagenomics/api/v1/analyses/MGYA00412966/file/ERR451467_FASTQ_SSU_OTU.tsv	Root-Host_associated-Human-Skin

SRR994738	MGYA00023465	https://www.ebi.ac.uk/metagenomics/api/v1/analyses/MGYA00023465/file/SRR994738_FASTQ_otu.tsv	Root-Host_associated-Human-Skin
SRR920115	MGYA00024128	https://www.ebi.ac.uk/metagenomics/api/v1/analyses/MGYA00024128/file/SRR920115_FASTQ_otu.tsv	Root-Host_associated-Human-Skin
SRR919882	MGYA00024203	https://www.ebi.ac.uk/metagenomics/api/v1/analyses/MGYA00024203/file/SRR919882_FASTQ_otu.tsv	Root-Host_associated-Human-Skin
SRR996926	MGYA00025133	https://www.ebi.ac.uk/metagenomics/api/v1/analyses/MGYA00025133/file/SRR996926_FASTQ_otu.tsv	Root-Host_associated-Human-Skin
SRR996554	MGYA00022621	https://www.ebi.ac.uk/metagenomics/api/v1/analyses/MGYA00022621/file/SRR996554_FASTQ_otu.tsv	Root-Host_associated-Human-Skin
ERR451298	MGYA00412377	https://www.ebi.ac.uk/metagenomics/api/v1/analyses/MGYA00412377/file/ERR451298_FASTQ_SSU_OTU.tsv	Root-Host_associated-Human-Skin
SRR995020	MGYA00023950	https://www.ebi.ac.uk/metagenomics/api/v1/analyses/MGYA00023950/file/SRR995020_FASTQ_otu.tsv	Root-Host_associated-Human-Skin
ERR1760030	MGYA00140498	https://www.ebi.ac.uk/metagenomics/api/v1/analyses/MGYA00140498/file/ERR1760030_MERGED_FASTQ_SSU_OTU.tsv	Root-Host_associated-Human-Skin
SRR1923789	MGYA00028286	https://www.ebi.ac.uk/metagenomics/api/v1/analyses/MGYA00028286/file/SRR1923789_FASTQ_otu.tsv	Root-Host_associated-Human-Skin
SRR6757608	MGYA00380726	https://www.ebi.ac.uk/metagenomics/api/v1/analyses/MGYA00380726/file/SRR6757608_MERGED_FASTQ_SSU_OTU.tsv	Root-Host_associated-Human-Skin
SRR1623219	MGYA00023916	https://www.ebi.ac.uk/metagenomics/api/v1/analyses/MGYA00023916/file/SRR1623219_MERGED_FASTQ_otu.tsv	Root-Host_associated-Human-Skin
SRR996692	MGYA00022291	https://www.ebi.ac.uk/metagenomics/api/v1/analyses/MGYA00022291/file/SRR996692_FASTQ_otu.tsv	Root-Host_associated-Human-Skin

SRR996135	MGYA00024996	https://www.ebi.ac.uk/metagenomics/api/v1/analyses/MGYA00024996/file/SRR996135_FASTQ_otu.tsv	Root-Host_associated-Human-Skin
ERR2538429	MGYA00381436	https://www.ebi.ac.uk/metagenomics/api/v1/analyses/MGYA00381436/file/ERR2538429_FASTQ_SSU_OTU.tsv	Root-Host_associated-Human-Skin
SRR1631309	MGYA00022496	https://www.ebi.ac.uk/metagenomics/api/v1/analyses/MGYA00022496/file/SRR1631309_MERGED_FASTQ_otu.tsv	Root-Host_associated-Human-Skin
SRR996222	MGYA00023975	https://www.ebi.ac.uk/metagenomics/api/v1/analyses/MGYA00023975/file/SRR996222_FASTQ_otu.tsv	Root-Host_associated-Human-Skin
SRR919853	MGYA00024741	https://www.ebi.ac.uk/metagenomics/api/v1/analyses/MGYA00024741/file/SRR919853_FASTQ_otu.tsv	Root-Host_associated-Human-Skin
SRR995490	MGYA00024111	https://www.ebi.ac.uk/metagenomics/api/v1/analyses/MGYA00024111/file/SRR995490_FASTQ_otu.tsv	Root-Host_associated-Human-Skin
ERR1960819	MGYA00130755	https://www.ebi.ac.uk/metagenomics/api/v1/analyses/MGYA00130755/file/ERR1960819_MERGED_FASTQ_otu.tsv	Root-Host_associated-Human-Skin
ERR441961	MGYA00385190	https://www.ebi.ac.uk/metagenomics/api/v1/analyses/MGYA00385190/file/ERR441961_FASTQ_SSU_OTU.tsv	Root-Host_associated-Human-Skin
SRR996767	MGYA00023860	https://www.ebi.ac.uk/metagenomics/api/v1/analyses/MGYA00023860/file/SRR996767_FASTQ_otu.tsv	Root-Host_associated-Human-Skin
ERR1960689	MGYA00130751	https://www.ebi.ac.uk/metagenomics/api/v1/analyses/MGYA00130751/file/ERR1960689_MERGED_FASTQ_otu.tsv	Root-Host_associated-Human-Skin
SRR5868648	MGYA00423517	https://www.ebi.ac.uk/metagenomics/api/v1/analyses/MGYA00423517/file/SRR5868648_MERGED_FASTQ_SSU_OTU.tsv	Root-Host_associated-Human-Skin
SRR996306	MGYA00022710	https://www.ebi.ac.uk/metagenomics/api/v1/analyses/MGYA00022710/file/SRR996306_FASTQ_otu.tsv	Root-Host_associated-Human-Skin

SRR1624786	MGYA00022547	https://www.ebi.ac.uk/metagenomics/api/v1/analyses/MGYA00022547/file/SRR1624786_MERGED_FASTQ_otu.tsv	Root-Host_associated-Human-Skin
ERR442067	MGYA00385195	https://www.ebi.ac.uk/metagenomics/api/v1/analyses/MGYA00385195/file/ERR442067_FASTQ_SSU_OTU.tsv	Root-Host_associated-Human-Skin
SRR994821	MGYA00022835	https://www.ebi.ac.uk/metagenomics/api/v1/analyses/MGYA00022835/file/SRR994821_FASTQ_otu.tsv	Root-Host_associated-Human-Skin
SRR994453	MGYA00022455	https://www.ebi.ac.uk/metagenomics/api/v1/analyses/MGYA00022455/file/SRR994453_FASTQ_otu.tsv	Root-Host_associated-Human-Skin
SRR994482	MGYA00023137	https://www.ebi.ac.uk/metagenomics/api/v1/analyses/MGYA00023137/file/SRR994482_FASTQ_otu.tsv	Root-Host_associated-Human-Skin
SRR995565	MGYA00022983	https://www.ebi.ac.uk/metagenomics/api/v1/analyses/MGYA00022983/file/SRR995565_FASTQ_otu.tsv	Root-Host_associated-Human-Skin
SRR1923919	MGYA00028303	https://www.ebi.ac.uk/metagenomics/api/v1/analyses/MGYA00028303/file/SRR1923919_FASTQ_otu.tsv	Root-Host_associated-Human-Skin
SRR5868699	MGYA00420807	https://www.ebi.ac.uk/metagenomics/api/v1/analyses/MGYA00420807/file/SRR5868699_MERGED_FASTQ_SSU_OTU.tsv	Root-Host_associated-Human-Skin
ERR1760114	MGYA00140590	https://www.ebi.ac.uk/metagenomics/api/v1/analyses/MGYA00140590/file/ERR1760114_MERGED_FASTQ_SSU_OTU.tsv	Root-Host_associated-Human-Skin
SRR995104	MGYA00023670	https://www.ebi.ac.uk/metagenomics/api/v1/analyses/MGYA00023670/file/SRR995104_FASTQ_otu.tsv	Root-Host_associated-Human-Skin
SRR1632788	MGYA00022569	https://www.ebi.ac.uk/metagenomics/api/v1/analyses/MGYA00022569/file/SRR1632788_MERGED_FASTQ_otu.tsv	Root-Host_associated-Human-Skin
ERR1960658	MGYA00130719	https://www.ebi.ac.uk/metagenomics/api/v1/analyses/MGYA00130719/file/ERR1960658_MERGED_FASTQ_otu.tsv	Root-Host_associated-Human-Skin

SRR919612	MGYA00023524	https://www.ebi.ac.uk/metagenomics/api/v1/analyses/MGYA00023524/file/SRR919612_FASTQ_otu.tsv	Root-Host_associated-Human-Skin
SRR1630996	MGYA00022856	https://www.ebi.ac.uk/metagenomics/api/v1/analyses/MGYA00022856/file/SRR1630996_MERGED_FASTQ_otu.tsv	Root-Host_associated-Human-Skin
ERR443101	MGYA00416314	https://www.ebi.ac.uk/metagenomics/api/v1/analyses/MGYA00416314/file/ERR443101_FASTQ_SSU_OTU.tsv	Root-Host_associated-Human-Skin
ERR451122	MGYA00413000	https://www.ebi.ac.uk/metagenomics/api/v1/analyses/MGYA00413000/file/ERR451122_FASTQ_SSU_OTU.tsv	Root-Host_associated-Human-Skin
SRR6757674	MGYA00380787	https://www.ebi.ac.uk/metagenomics/api/v1/analyses/MGYA00380787/file/SRR6757674_MERGED_FASTQ_SSU_OTU.tsv	Root-Host_associated-Human-Skin
SRR5868875	MGYA00426265	https://www.ebi.ac.uk/metagenomics/api/v1/analyses/MGYA00426265/file/SRR5868875_MERGED_FASTQ_SSU_OTU.tsv	Root-Host_associated-Human-Skin
SRR1631062	MGYA00024810	https://www.ebi.ac.uk/metagenomics/api/v1/analyses/MGYA00024810/file/SRR1631062_MERGED_FASTQ_otu.tsv	Root-Host_associated-Human-Skin
ERR2538484	MGYA00381491	https://www.ebi.ac.uk/metagenomics/api/v1/analyses/MGYA00381491/file/ERR2538484_FASTQ_SSU_OTU.tsv	Root-Host_associated-Human-Skin
SRR996149	MGYA00022522	https://www.ebi.ac.uk/metagenomics/api/v1/analyses/MGYA00022522/file/SRR996149_FASTQ_otu.tsv	Root-Host_associated-Human-Skin
SRR1622857	MGYA00024380	https://www.ebi.ac.uk/metagenomics/api/v1/analyses/MGYA00024380/file/SRR1622857_MERGED_FASTQ_otu.tsv	Root-Host_associated-Human-Skin
ERR451543	MGYA00428663	https://www.ebi.ac.uk/metagenomics/api/v1/analyses/MGYA00428663/file/ERR451543_FASTQ_SSU_OTU.tsv	Root-Host_associated-Human-Skin
ERR443560	MGYA00416644	https://www.ebi.ac.uk/metagenomics/api/v1/analyses/MGYA00416644/file/ERR443560_FASTQ_SSU_OTU.tsv	Root-Host_associated-Human-Skin

ERR1960700	MGYA00130799	https://www.ebi.ac.uk/metagenomics/api/v1/analyses/MGYA00130799/file/ERR1960700_MERGED_FASTQ_otu.tsv	Root-Host_associated-Human-Skin
ERR1701272	MGYA00084287	https://www.ebi.ac.uk/metagenomics/api/v1/analyses/MGYA00084287/file/ERR1701272_FASTQ_otu.tsv	Root-Host_associated-Human-Skin
SRR920747	MGYA00023543	https://www.ebi.ac.uk/metagenomics/api/v1/analyses/MGYA00023543/file/SRR920747_FASTQ_otu.tsv	Root-Host_associated-Human-Skin
ERR450807	MGYA00423329	https://www.ebi.ac.uk/metagenomics/api/v1/analyses/MGYA00423329/file/ERR450807_FASTQ_SSU_OTU.tsv	Root-Host_associated-Human-Skin
ERR451235	MGYA00426515	https://www.ebi.ac.uk/metagenomics/api/v1/analyses/MGYA00426515/file/ERR451235_FASTQ_SSU_OTU.tsv	Root-Host_associated-Human-Skin
SRR921107	MGYA00025001	https://www.ebi.ac.uk/metagenomics/api/v1/analyses/MGYA00025001/file/SRR921107_FASTQ_otu.tsv	Root-Host_associated-Human-Skin
ERR2538455	MGYA00381462	https://www.ebi.ac.uk/metagenomics/api/v1/analyses/MGYA00381462/file/ERR2538455_FASTQ_SSU_OTU.tsv	Root-Host_associated-Human-Skin
SRR1631714	MGYA00024035	https://www.ebi.ac.uk/metagenomics/api/v1/analyses/MGYA00024035/file/SRR1631714_MERGED_FASTQ_otu.tsv	Root-Host_associated-Human-Skin
SRR1923890	MGYA00028368	https://www.ebi.ac.uk/metagenomics/api/v1/analyses/MGYA00028368/file/SRR1923890_FASTQ_otu.tsv	Root-Host_associated-Human-Skin
SRR994979	MGYA00024549	https://www.ebi.ac.uk/metagenomics/api/v1/analyses/MGYA00024549/file/SRR994979_FASTQ_otu.tsv	Root-Host_associated-Human-Skin
SRR1704416	MGYA00415214	https://www.ebi.ac.uk/metagenomics/api/v1/analyses/MGYA00415214/file/SRR1704416_FASTQ_SSU_OTU.tsv	Root-Host_associated-Human-Skin
SRR1704610	MGYA00428825	https://www.ebi.ac.uk/metagenomics/api/v1/analyses/MGYA00428825/file/SRR1704610_FASTQ_SSU_OTU.tsv	Root-Host_associated-Human-Skin

SRR5868964	MGYA00416498	https://www.ebi.ac.uk/metagenomics/api/v1/analyses/MGYA00416498/file/SRR5868964_MERGED_FASTQ_SSU_OTU.tsv	Root-Host_associated-Human-Skin
SRR2005373	MGYA00381284	https://www.ebi.ac.uk/metagenomics/api/v1/analyses/MGYA00381284/file/SRR2005373_MERGED_FASTQ_SSU_OTU.tsv	Root-Host_associated-Human-Skin
ERR443471	MGYA00428719	https://www.ebi.ac.uk/metagenomics/api/v1/analyses/MGYA00428719/file/ERR443471_FASTQ_SSU_OTU.tsv	Root-Host_associated-Human-Skin
SRR5869090	MGYA00418118	https://www.ebi.ac.uk/metagenomics/api/v1/analyses/MGYA00418118/file/SRR5869090_MERGED_FASTQ_SSU_OTU.tsv	Root-Host_associated-Human-Skin
SRR996439	MGYA00024480	https://www.ebi.ac.uk/metagenomics/api/v1/analyses/MGYA00024480/file/SRR996439_FASTQ_otu.tsv	Root-Host_associated-Human-Skin
SRR2005712	MGYA00381341	https://www.ebi.ac.uk/metagenomics/api/v1/analyses/MGYA00381341/file/SRR2005712_MERGED_FASTQ_SSU_OTU.tsv	Root-Host_associated-Human-Skin
SRR1631512	MGYA00023379	https://www.ebi.ac.uk/metagenomics/api/v1/analyses/MGYA00023379/file/SRR1631512_MERGED_FASTQ_otu.tsv	Root-Host_associated-Human-Skin
ERR1759992	MGYA00140466	https://www.ebi.ac.uk/metagenomics/api/v1/analyses/MGYA00140466/file/ERR1759992_MERGED_FASTQ_SSU_OTU.tsv	Root-Host_associated-Human-Skin
ERR443010	MGYA00420161	https://www.ebi.ac.uk/metagenomics/api/v1/analyses/MGYA00420161/file/ERR443010_FASTQ_SSU_OTU.tsv	Root-Host_associated-Human-Skin
SRR920933	MGYA00022576	https://www.ebi.ac.uk/metagenomics/api/v1/analyses/MGYA00022576/file/SRR920933_FASTQ_otu.tsv	Root-Host_associated-Human-Skin
SRR1632407	MGYA00023548	https://www.ebi.ac.uk/metagenomics/api/v1/analyses/MGYA00023548/file/SRR1632407_MERGED_FASTQ_otu.tsv	Root-Host_associated-Human-Skin
SRR1923950	MGYA00028334	https://www.ebi.ac.uk/metagenomics/api/v1/analyses/MGYA00028334/file/SRR1923950_FASTQ_otu.tsv	Root-Host_associated-Human-Skin

SRR994868	MGYA00022396	https://www.ebi.ac.uk/metagenomics/api/v1/analyses/MGYA00022396/file/SRR994868_FASTQ_otu.tsv	Root-Host_associated-Human-Skin
ERR443766	MGYA00421372	https://www.ebi.ac.uk/metagenomics/api/v1/analyses/MGYA00421372/file/ERR443766_FASTQ_SSU_OTU.tsv	Root-Host_associated-Human-Skin
SRR5868601	MGYA00411960	https://www.ebi.ac.uk/metagenomics/api/v1/analyses/MGYA00411960/file/SRR5868601_MERGED_FASTQ_SSU_OTU.tsv	Root-Host_associated-Human-Skin
SRR1704507	MGYA00424942	https://www.ebi.ac.uk/metagenomics/api/v1/analyses/MGYA00424942/file/SRR1704507_FASTQ_SSU_OTU.tsv	Root-Host_associated-Human-Skin
SRR1623063	MGYA00024417	https://www.ebi.ac.uk/metagenomics/api/v1/analyses/MGYA00024417/file/SRR1623063_MERGED_FASTQ_otu.tsv	Root-Host_associated-Human-Skin
ERR1759943	MGYA00140533	https://www.ebi.ac.uk/metagenomics/api/v1/analyses/MGYA00140533/file/ERR1759943_MERGED_FASTQ_SSU_OTU.tsv	Root-Host_associated-Human-Skin
SRR1950715	MGYA00376882	https://www.ebi.ac.uk/metagenomics/api/v1/analyses/MGYA00376882/file/SRR1950715_MERGED_FASTQ_SSU_OTU.tsv	Root-Host_associated-Human-Skin
SRR920656	MGYA00025215	https://www.ebi.ac.uk/metagenomics/api/v1/analyses/MGYA00025215/file/SRR920656_FASTQ_otu.tsv	Root-Host_associated-Human-Skin
SRR921016	MGYA00023329	https://www.ebi.ac.uk/metagenomics/api/v1/analyses/MGYA00023329/file/SRR921016_FASTQ_otu.tsv	Root-Host_associated-Human-Skin
ERR451324	MGYA00426034	https://www.ebi.ac.uk/metagenomics/api/v1/analyses/MGYA00426034/file/ERR451324_FASTQ_SSU_OTU.tsv	Root-Host_associated-Human-Skin
SRR996089	MGYA00024359	https://www.ebi.ac.uk/metagenomics/api/v1/analyses/MGYA00024359/file/SRR996089_FASTQ_otu.tsv	Root-Host_associated-Human-Skin
SRR5869387	MGYA00415350	https://www.ebi.ac.uk/metagenomics/api/v1/analyses/MGYA00415350/file/SRR5869387_MERGED_FASTQ_SSU_OTU.tsv	Root-Host_associated-Human-Skin

SRR919866	MGYA00023899	https://www.ebi.ac.uk/metagenomics/api/v1/analyses/MGYA00023899/file/SRR919866_FASTQ_otu.tsv	Root-Host_associated-Human-Skin
SRR995302	MGYA00022697	https://www.ebi.ac.uk/metagenomics/api/v1/analyses/MGYA00022697/file/SRR995302_FASTQ_otu.tsv	Root-Host_associated-Human-Skin
ERR443199	MGYA00421091	https://www.ebi.ac.uk/metagenomics/api/v1/analyses/MGYA00421091/file/ERR443199_FASTQ_SSU_OTU.tsv	Root-Host_associated-Human-Skin
SRR994542	MGYA00024265	https://www.ebi.ac.uk/metagenomics/api/v1/analyses/MGYA00024265/file/SRR994542_FASTQ_otu.tsv	Root-Host_associated-Human-Skin
SRR997036	MGYA00023602	https://www.ebi.ac.uk/metagenomics/api/v1/analyses/MGYA00023602/file/SRR997036_FASTQ_otu.tsv	Root-Host_associated-Human-Skin
ERR1960798	MGYA00130844	https://www.ebi.ac.uk/metagenomics/api/v1/analyses/MGYA00130844/file/ERR1960798_MERGED_FASTQ_otu.tsv	Root-Host_associated-Human-Skin
ERR1760005	MGYA00140478	https://www.ebi.ac.uk/metagenomics/api/v1/analyses/MGYA00140478/file/ERR1760005_MERGED_FASTQ_SSU_OTU.tsv	Root-Host_associated-Human-Skin
ERR443529	MGYA00428696	https://www.ebi.ac.uk/metagenomics/api/v1/analyses/MGYA00428696/file/ERR443529_FASTQ_SSU_OTU.tsv	Root-Host_associated-Human-Skin
SRR994655	MGYA00023444	https://www.ebi.ac.uk/metagenomics/api/v1/analyses/MGYA00023444/file/SRR994655_FASTQ_otu.tsv	Root-Host_associated-Human-Skin
ERR1960749	MGYA00130855	https://www.ebi.ac.uk/metagenomics/api/v1/analyses/MGYA00130855/file/ERR1960749_MERGED_FASTQ_otu.tsv	Root-Host_associated-Human-Skin
SRR1704748	MGYA00428434	https://www.ebi.ac.uk/metagenomics/api/v1/analyses/MGYA00428434/file/SRR1704748_FASTQ_SSU_OTU.tsv	Root-Host_associated-Human-Skin
SRR1924010	MGYA00028387	https://www.ebi.ac.uk/metagenomics/api/v1/analyses/MGYA00028387/file/SRR1924010_FASTQ_otu.tsv	Root-Host_associated-Human-Skin

SRR995474	MGYA00025100	https://www.ebi.ac.uk/metagenomics/api/v1/analyses/MGYA00025100/file/SRR995474_FASTQ_otu.tsv	Root-Host_associated-Human-Skin
SRR996913	MGYA00022440	https://www.ebi.ac.uk/metagenomics/api/v1/analyses/MGYA00022440/file/SRR996913_FASTQ_otu.tsv	Root-Host_associated-Human-Skin
SRR996561	MGYA00025296	https://www.ebi.ac.uk/metagenomics/api/v1/analyses/MGYA00025296/file/SRR996561_FASTQ_otu.tsv	Root-Host_associated-Human-Skin
SRR995696	MGYA00023002	https://www.ebi.ac.uk/metagenomics/api/v1/analyses/MGYA00023002/file/SRR995696_FASTQ_otu.tsv	Root-Host_associated-Human-Skin
ERR1760121	MGYA00140582	https://www.ebi.ac.uk/metagenomics/api/v1/analyses/MGYA00140582/file/ERR1760121_MERGED_FASTQ_SSU_OTU.tsv	Root-Host_associated-Human-Skin
SRR996783	MGYA00024076	https://www.ebi.ac.uk/metagenomics/api/v1/analyses/MGYA00024076/file/SRR996783_FASTQ_otu.tsv	Root-Host_associated-Human-Skin
ERR441985	MGYA00385230	https://www.ebi.ac.uk/metagenomics/api/v1/analyses/MGYA00385230/file/ERR441985_FASTQ_SSU_OTU.tsv	Root-Host_associated-Human-Skin
SRR996024	MGYA00023721	https://www.ebi.ac.uk/metagenomics/api/v1/analyses/MGYA00023721/file/SRR996024_FASTQ_otu.tsv	Root-Host_associated-Human-Skin
SRR995922	MGYA00024067	https://www.ebi.ac.uk/metagenomics/api/v1/analyses/MGYA00024067/file/SRR995922_FASTQ_otu.tsv	Root-Host_associated-Human-Skin
SRR996837	MGYA00023868	https://www.ebi.ac.uk/metagenomics/api/v1/analyses/MGYA00023868/file/SRR996837_FASTQ_otu.tsv	Root-Host_associated-Human-Skin
SRR919530	MGYA00024316	https://www.ebi.ac.uk/metagenomics/api/v1/analyses/MGYA00024316/file/SRR919530_FASTQ_otu.tsv	Root-Host_associated-Human-Skin
SRR919942	MGYA00024617	https://www.ebi.ac.uk/metagenomics/api/v1/analyses/MGYA00024617/file/SRR919942_FASTQ_otu.tsv	Root-Host_associated-Human-Skin

SRR1950769	MGYA00376936	https://www.ebi.ac.uk/metagenomics/api/v1/analyses/MGYA00376936/file/SRR1950769_MERGED_FASTQ_SSU_OTU.tsv	Root-Host_associated-Human-Skin
SRR996494	MGYA00023404	https://www.ebi.ac.uk/metagenomics/api/v1/analyses/MGYA00023404/file/SRR996494_FASTQ_otu.tsv	Root-Host_associated-Human-Skin
ERR442052	MGYA00385213	https://www.ebi.ac.uk/metagenomics/api/v1/analyses/MGYA00385213/file/ERR442052_FASTQ_SSU_OTU.tsv	Root-Host_associated-Human-Skin
SRR997112	MGYA00024559	https://www.ebi.ac.uk/metagenomics/api/v1/analyses/MGYA00024559/file/SRR997112_FASTQ_otu.tsv	Root-Host_associated-Human-Skin
SRR1631679	MGYA00023454	https://www.ebi.ac.uk/metagenomics/api/v1/analyses/MGYA00023454/file/SRR1631679_MERGED_FASTQ_otu.tsv	Root-Host_associated-Human-Skin
SRR5870190	MGYA00414806	https://www.ebi.ac.uk/metagenomics/api/v1/analyses/MGYA00414806/file/SRR5870190_MERGED_FASTQ_SSU_OTU.tsv	Root-Host_associated-Human-Skin
SRR1624807	MGYA00024669	https://www.ebi.ac.uk/metagenomics/api/v1/analyses/MGYA00024669/file/SRR1624807_MERGED_FASTQ_otu.tsv	Root-Host_associated-Human-Skin
ERR451358	MGYA00427803	https://www.ebi.ac.uk/metagenomics/api/v1/analyses/MGYA00427803/file/ERR451358_FASTQ_SSU_OTU.tsv	Root-Host_associated-Human-Skin
SRR995647	MGYA00024633	https://www.ebi.ac.uk/metagenomics/api/v1/analyses/MGYA00024633/file/SRR995647_FASTQ_otu.tsv	Root-Host_associated-Human-Skin
SRR1632980	MGYA00025152	https://www.ebi.ac.uk/metagenomics/api/v1/analyses/MGYA00025152/file/SRR1632980_MERGED_FASTQ_otu.tsv	Root-Host_associated-Human-Skin
ERR443642	MGYA00425743	https://www.ebi.ac.uk/metagenomics/api/v1/analyses/MGYA00425743/file/ERR443642_FASTQ_SSU_OTU.tsv	Root-Host_associated-Human-Skin
SRR1704384	MGYA00428872	https://www.ebi.ac.uk/metagenomics/api/v1/analyses/MGYA00428872/file/SRR1704384_FASTQ_SSU_OTU.tsv	Root-Host_associated-Human-Skin

SRR1631340	MGYA00022807	https://www.ebi.ac.uk/metagenomics/api/v1/analyses/MGYA00022807/file/SRR1631340_MERGED_FASTQ_otu.tsv	Root-Host_associated-Human-Skin
SRR1630972	MGYA00024352	https://www.ebi.ac.uk/metagenomics/api/v1/analyses/MGYA00024352/file/SRR1630972_MERGED_FASTQ_otu.tsv	Root-Host_associated-Human-Skin
ERR450832	MGYA00422049	https://www.ebi.ac.uk/metagenomics/api/v1/analyses/MGYA00422049/file/ERR450832_FASTQ_SSU_OTU.tsv	Root-Host_associated-Human-Skin
ERR1701247	MGYA00084277	https://www.ebi.ac.uk/metagenomics/api/v1/analyses/MGYA00084277/file/ERR1701247_FASTQ_otu.tsv	Root-Host_associated-Human-Skin
ERR443584	MGYA00412357	https://www.ebi.ac.uk/metagenomics/api/v1/analyses/MGYA00412357/file/ERR443584_FASTQ_SSU_OTU.tsv	Root-Host_associated-Human-Skin
SRR921132	MGYA00022304	https://www.ebi.ac.uk/metagenomics/api/v1/analyses/MGYA00022304/file/SRR921132_FASTQ_otu.tsv	Root-Host_associated-Human-Skin
ERR2538460	MGYA00381467	https://www.ebi.ac.uk/metagenomics/api/v1/analyses/MGYA00381467/file/ERR2538460_FASTQ_SSU_OTU.tsv	Root-Host_associated-Human-Skin
SRR6757690	MGYA00380803	https://www.ebi.ac.uk/metagenomics/api/v1/analyses/MGYA00380803/file/SRR6757690_MERGED_FASTQ_SSU_OTU.tsv	Root-Host_associated-Human-Skin
SRR1631086	MGYA00023785	https://www.ebi.ac.uk/metagenomics/api/v1/analyses/MGYA00023785/file/SRR1631086_MERGED_FASTQ_otu.tsv	Root-Host_associated-Human-Skin
SRR6757641	MGYA00380756	https://www.ebi.ac.uk/metagenomics/api/v1/analyses/MGYA00380756/file/SRR6757641_MERGED_FASTQ_SSU_OTU.tsv	Root-Host_associated-Human-Skin
SRR5868840	MGYA00426663	https://www.ebi.ac.uk/metagenomics/api/v1/analyses/MGYA00426663/file/SRR5868840_MERGED_FASTQ_SSU_OTU.tsv	Root-Host_associated-Human-Skin
SRR5869272	MGYA00418762	https://www.ebi.ac.uk/metagenomics/api/v1/analyses/MGYA00418762/file/SRR5869272_MERGED_FASTQ_SSU_OTU.tsv	Root-Host_associated-Human-Skin

ERR1760079	MGYA00140518	https://www.ebi.ac.uk/metagenomics/api/v1/analyses/MGYA00140518/file/ERR1760079_MERGED_FASTQ_SSU_OTU.tsv	Root-Host_associated-Human-Skin
SRR995069	MGYA00023499	https://www.ebi.ac.uk/metagenomics/api/v1/analyses/MGYA00023499/file/SRR995069_FASTQ_otu.tsv	Root-Host_associated-Human-Skin
ERR1960735	MGYA00130714	https://www.ebi.ac.uk/metagenomics/api/v1/analyses/MGYA00130714/file/ERR1960735_MERGED_FASTQ_otu.tsv	Root-Host_associated-Human-Skin
SRR5870019	MGYA00420683	https://www.ebi.ac.uk/metagenomics/api/v1/analyses/MGYA00420683/file/SRR5870019_MERGED_FASTQ_SSU_OTU.tsv	Root-Host_associated-Human-Skin
ERR1701296	MGYA00084260	https://www.ebi.ac.uk/metagenomics/api/v1/analyses/MGYA00084260/file/ERR1701296_FASTQ_otu.tsv	Root-Host_associated-Human-Skin
SRR5869613	MGYA00421609	https://www.ebi.ac.uk/metagenomics/api/v1/analyses/MGYA00421609/file/SRR5869613_MERGED_FASTQ_SSU_OTU.tsv	Root-Host_associated-Human-Skin
SRR2005636	MGYA00381296	https://www.ebi.ac.uk/metagenomics/api/v1/analyses/MGYA00381296/file/SRR2005636_MERGED_FASTQ_SSU_OTU.tsv	Root-Host_associated-Human-Skin
SRR1631436	MGYA00025320	https://www.ebi.ac.uk/metagenomics/api/v1/analyses/MGYA00025320/file/SRR1631436_MERGED_FASTQ_otu.tsv	Root-Host_associated-Human-Skin
SRR1631391	MGYA00022726	https://www.ebi.ac.uk/metagenomics/api/v1/analyses/MGYA00022726/file/SRR1631391_MERGED_FASTQ_otu.tsv	Root-Host_associated-Human-Skin
ERR443134	MGYA00421093	https://www.ebi.ac.uk/metagenomics/api/v1/analyses/MGYA00421093/file/ERR443134_FASTQ_SSU_OTU.tsv	Root-Host_associated-Human-Skin
ERR450757	MGYA00425280	https://www.ebi.ac.uk/metagenomics/api/v1/analyses/MGYA00425280/file/ERR450757_FASTQ_SSU_OTU.tsv	Root-Host_associated-Human-Skin
SRR1923874	MGYA00028351	https://www.ebi.ac.uk/metagenomics/api/v1/analyses/MGYA00028351/file/SRR1923874_FASTQ_otu.tsv	Root-Host_associated-Human-Skin

ERR1760126	MGYA00140506	https://www.ebi.ac.uk/metagenomics/api/v1/analyses/MGYA00140506/file/ERR1760126_MERGED_FASTQ_SSU_OTU.tsv	Root-Host_associated-Human-Skin
SRR995691	MGYA00023009	https://www.ebi.ac.uk/metagenomics/api/v1/analyses/MGYA00023009/file/SRR995691_FASTQ_otu.tsv	Root-Host_associated-Human-Skin
SRR996023	MGYA00023728	https://www.ebi.ac.uk/metagenomics/api/v1/analyses/MGYA00023728/file/SRR996023_FASTQ_otu.tsv	Root-Host_associated-Human-Skin
ERR441982	MGYA00385162	https://www.ebi.ac.uk/metagenomics/api/v1/analyses/MGYA00385162/file/ERR441982_FASTQ_SSU_OTU.tsv	Root-Host_associated-Human-Skin
SRR996784	MGYA00024079	https://www.ebi.ac.uk/metagenomics/api/v1/analyses/MGYA00024079/file/SRR996784_FASTQ_otu.tsv	Root-Host_associated-Human-Skin
SRR995925	MGYA00024070	https://www.ebi.ac.uk/metagenomics/api/v1/analyses/MGYA00024070/file/SRR995925_FASTQ_otu.tsv	Root-Host_associated-Human-Skin
ERR451048	MGYA00426103	https://www.ebi.ac.uk/metagenomics/api/v1/analyses/MGYA00426103/file/ERR451048_FASTQ_SSU_OTU.tsv	Root-Host_associated-Human-Skin
SRR996830	MGYA00023867	https://www.ebi.ac.uk/metagenomics/api/v1/analyses/MGYA00023867/file/SRR996830_FASTQ_otu.tsv	Root-Host_associated-Human-Skin
SRR996493	MGYA00023401	https://www.ebi.ac.uk/metagenomics/api/v1/analyses/MGYA00023401/file/SRR996493_FASTQ_otu.tsv	Root-Host_associated-Human-Skin
SRR919945	MGYA00024624	https://www.ebi.ac.uk/metagenomics/api/v1/analyses/MGYA00024624/file/SRR919945_FASTQ_otu.tsv	Root-Host_associated-Human-Skin
SRR919537	MGYA00024309	https://www.ebi.ac.uk/metagenomics/api/v1/analyses/MGYA00024309/file/SRR919537_FASTQ_otu.tsv	Root-Host_associated-Human-Skin
ERR1759938	MGYA00140373	https://www.ebi.ac.uk/metagenomics/api/v1/analyses/MGYA00140373/file/ERR1759938_MERGED_FASTQ_SSU_OTU.tsv	Root-Host_associated-Human-Skin

SRR920999	MGYA00023257	https://www.ebi.ac.uk/metagenomics/api/v1/analyses/MGYA00023257/file/SRR920999_FASTQ_otu.tsv	Root-Host_associated-Human-Skin
ERR442055	MGYA00385255	https://www.ebi.ac.uk/metagenomics/api/v1/analyses/MGYA00385255/file/ERR442055_FASTQ_SSU_OTU.tsv	Root-Host_associated-Human-Skin
SRR995586	MGYA00022317	https://www.ebi.ac.uk/metagenomics/api/v1/analyses/MGYA00022317/file/SRR995586_FASTQ_otu.tsv	Root-Host_associated-Human-Skin
SRR997115	MGYA00024564	https://www.ebi.ac.uk/metagenomics/api/v1/analyses/MGYA00024564/file/SRR997115_FASTQ_otu.tsv	Root-Host_associated-Human-Skin
SRR995640	MGYA00024628	https://www.ebi.ac.uk/metagenomics/api/v1/analyses/MGYA00024628/file/SRR995640_FASTQ_otu.tsv	Root-Host_associated-Human-Skin
SRR1624800	MGYA00024674	https://www.ebi.ac.uk/metagenomics/api/v1/analyses/MGYA00024674/file/SRR1624800_MERGED_FASTQ_otu.tsv	Root-Host_associated-Human-Skin
SRR1632987	MGYA00025159	https://www.ebi.ac.uk/metagenomics/api/v1/analyses/MGYA00025159/file/SRR1632987_MERGED_FASTQ_otu.tsv	Root-Host_associated-Human-Skin
SRR1630975	MGYA00024349	https://www.ebi.ac.uk/metagenomics/api/v1/analyses/MGYA00024349/file/SRR1630975_MERGED_FASTQ_otu.tsv	Root-Host_associated-Human-Skin
SRR1631347	MGYA00022808	https://www.ebi.ac.uk/metagenomics/api/v1/analyses/MGYA00022808/file/SRR1631347_MERGED_FASTQ_otu.tsv	Root-Host_associated-Human-Skin
SRR1704383	MGYA00414649	https://www.ebi.ac.uk/metagenomics/api/v1/analyses/MGYA00414649/file/SRR1704383_FASTQ_SSU_OTU.tsv	Root-Host_associated-Human-Skin
SRR921135	MGYA00022299	https://www.ebi.ac.uk/metagenomics/api/v1/analyses/MGYA00022299/file/SRR921135_FASTQ_otu.tsv	Root-Host_associated-Human-Skin
ERR451207	MGYA00424898	https://www.ebi.ac.uk/metagenomics/api/v1/analyses/MGYA00424898/file/ERR451207_FASTQ_SSU_OTU.tsv	Root-Host_associated-Human-Skin

SRR995718	MGYA00023129	https://www.ebi.ac.uk/metagenomics/api/v1/analyses/MGYA00023129/file/SRR995718_FASTQ_otu.tsv	Root-Host_associated-Human-Skin
ERR1701240	MGYA00084319	https://www.ebi.ac.uk/metagenomics/api/v1/analyses/MGYA00084319/file/ERR1701240_FASTQ_otu.tsv	Root-Host_associated-Human-Skin
ERR443583	MGYA00416641	https://www.ebi.ac.uk/metagenomics/api/v1/analyses/MGYA00416641/file/ERR443583_FASTQ_SSU_OTU.tsv	Root-Host_associated-Human-Skin
SRR5868896	MGYA00426259	https://www.ebi.ac.uk/metagenomics/api/v1/analyses/MGYA00426259/file/SRR5868896_MERGED_FASTQ_SSU_OTU.tsv	Root-Host_associated-Human-Skin
SRR6757697	MGYA00380810	https://www.ebi.ac.uk/metagenomics/api/v1/analyses/MGYA00380810/file/SRR6757697_MERGED_FASTQ_SSU_OTU.tsv	Root-Host_associated-Human-Skin
SRR1631081	MGYA00023788	https://www.ebi.ac.uk/metagenomics/api/v1/analyses/MGYA00023788/file/SRR1631081_MERGED_FASTQ_otu.tsv	Root-Host_associated-Human-Skin
ERR2538467	MGYA00381474	https://www.ebi.ac.uk/metagenomics/api/v1/analyses/MGYA00381474/file/ERR2538467_FASTQ_SSU_OTU.tsv	Root-Host_associated-Human-Skin
SRR5869971	MGYA00413952	https://www.ebi.ac.uk/metagenomics/api/v1/analyses/MGYA00413952/file/SRR5869971_MERGED_FASTQ_SSU_OTU.tsv	Root-Host_associated-Human-Skin
SRR6757646	MGYA00380761	https://www.ebi.ac.uk/metagenomics/api/v1/analyses/MGYA00380761/file/SRR6757646_MERGED_FASTQ_SSU_OTU.tsv	Root-Host_associated-Human-Skin
ERR1701291	MGYA00084237	https://www.ebi.ac.uk/metagenomics/api/v1/analyses/MGYA00084237/file/ERR1701291_FASTQ_otu.tsv	Root-Host_associated-Human-Skin
ERR1960732	MGYA00130870	https://www.ebi.ac.uk/metagenomics/api/v1/analyses/MGYA00130870/file/ERR1960732_MERGED_FASTQ_otu.tsv	Root-Host_associated-Human-Skin
SRR1631396	MGYA00022771	https://www.ebi.ac.uk/metagenomics/api/v1/analyses/MGYA00022771/file/SRR1631396_MERGED_FASTQ_otu.tsv	Root-Host_associated-Human-Skin

SRR1923873	MGYA00028352	https://www.ebi.ac.uk/metagenomics/api/v1/analyses/MGYA00028352/file/SRR1923873_FASTQ_otu.tsv	Root-Host_associated-Human-Skin
ERR443133	MGYA00416683	https://www.ebi.ac.uk/metagenomics/api/v1/analyses/MGYA00416683/file/ERR443133_FASTQ_SSU_OTU.tsv	Root-Host_associated-Human-Skin
ERR450750	MGYA00426851	https://www.ebi.ac.uk/metagenomics/api/v1/analyses/MGYA00426851/file/ERR450750_FASTQ_SSU_OTU.tsv	Root-Host_associated-Human-Skin
SRR2005374	MGYA00381285	https://www.ebi.ac.uk/metagenomics/api/v1/analyses/MGYA00381285/file/SRR2005374_MERGED_FASTQ_SSU_OTU.tsv	Root-Host_associated-Human-Skin
ERR443476	MGYA00424299	https://www.ebi.ac.uk/metagenomics/api/v1/analyses/MGYA00424299/file/ERR443476_FASTQ_SSU_OTU.tsv	Root-Host_associated-Human-Skin
ERR443804	MGYA00419415	https://www.ebi.ac.uk/metagenomics/api/v1/analyses/MGYA00419415/file/ERR443804_FASTQ_SSU_OTU.tsv	Root-Host_associated-Human-Skin
SRR1631515	MGYA00023386	https://www.ebi.ac.uk/metagenomics/api/v1/analyses/MGYA00023386/file/SRR1631515_MERGED_FASTQ_otu.tsv	Root-Host_associated-Human-Skin
ERR1759995	MGYA00140557	https://www.ebi.ac.uk/metagenomics/api/v1/analyses/MGYA00140557/file/ERR1759995_MERGED_FASTQ_SSU_OTU.tsv	Root-Host_associated-Human-Skin
SRR996399	MGYA00024981	https://www.ebi.ac.uk/metagenomics/api/v1/analyses/MGYA00024981/file/SRR996399_FASTQ_otu.tsv	Root-Host_associated-Human-Skin
SRR2005715	MGYA00381344	https://www.ebi.ac.uk/metagenomics/api/v1/analyses/MGYA00381344/file/SRR2005715_MERGED_FASTQ_SSU_OTU.tsv	Root-Host_associated-Human-Skin
SRR1923957	MGYA00028393	https://www.ebi.ac.uk/metagenomics/api/v1/analyses/MGYA00028393/file/SRR1923957_FASTQ_otu.tsv	Root-Host_associated-Human-Skin
SRR1632400	MGYA00023551	https://www.ebi.ac.uk/metagenomics/api/v1/analyses/MGYA00023551/file/SRR1632400_MERGED_FASTQ_otu.tsv	Root-Host_associated-Human-Skin

SRR920934	MGYA00022575	https://www.ebi.ac.uk/metagenomics/api/v1/analyses/MGYA00022575/file/SRR920934_FASTQ_otu.tsv	Root-Host_associated-Human-Skin
ERR443761	MGYA00424261	https://www.ebi.ac.uk/metagenomics/api/v1/analyses/MGYA00424261/file/ERR443761_FASTQ_SSU_OTU.tsv	Root-Host_associated-Human-Skin
ERR442029	MGYA00385170	https://www.ebi.ac.uk/metagenomics/api/v1/analyses/MGYA00385170/file/ERR442029_FASTQ_SSU_OTU.tsv	Root-Host_associated-Human-Skin
SRR995988	MGYA00022931	https://www.ebi.ac.uk/metagenomics/api/v1/analyses/MGYA00022931/file/SRR995988_FASTQ_otu.tsv	Root-Host_associated-Human-Skin
SRR996348	MGYA00022285	https://www.ebi.ac.uk/metagenomics/api/v1/analyses/MGYA00022285/file/SRR996348_FASTQ_otu.tsv	Root-Host_associated-Human-Skin
SRR1950712	MGYA00376879	https://www.ebi.ac.uk/metagenomics/api/v1/analyses/MGYA00376879/file/SRR1950712_MERGED_FASTQ_SSU_OTU.tsv	Root-Host_associated-Human-Skin
SRR1623064	MGYA00024412	https://www.ebi.ac.uk/metagenomics/api/v1/analyses/MGYA00024412/file/SRR1623064_MERGED_FASTQ_otu.tsv	Root-Host_associated-Human-Skin
ERR1759944	MGYA00140512	https://www.ebi.ac.uk/metagenomics/api/v1/analyses/MGYA00140512/file/ERR1759944_MERGED_FASTQ_SSU_OTU.tsv	Root-Host_associated-Human-Skin
SRR5868606	MGYA00417184	https://www.ebi.ac.uk/metagenomics/api/v1/analyses/MGYA00417184/file/SRR5868606_MERGED_FASTQ_SSU_OTU.tsv	Root-Host_associated-Human-Skin
SRR1704500	MGYA00419542	https://www.ebi.ac.uk/metagenomics/api/v1/analyses/MGYA00419542/file/SRR1704500_FASTQ_SSU_OTU.tsv	Root-Host_associated-Human-Skin
SRR921011	MGYA00023326	https://www.ebi.ac.uk/metagenomics/api/v1/analyses/MGYA00023326/file/SRR921011_FASTQ_otu.tsv	Root-Host_associated-Human-Skin
SRR920651	MGYA00025214	https://www.ebi.ac.uk/metagenomics/api/v1/analyses/MGYA00025214/file/SRR920651_FASTQ_otu.tsv	Root-Host_associated-Human-Skin

ERR450911	MGYA00425262	https://www.ebi.ac.uk/metagenomics/api/v1/analyses/MGYA00425262/file/ERR450911_FASTQ_SSU_OTU.tsv	Root-Host_associated-Human-Skin
SRR996729	MGYA00023424	https://www.ebi.ac.uk/metagenomics/api/v1/analyses/MGYA00023424/file/SRR996729_FASTQ_otu.tsv	Root-Host_associated-Human-Skin
SRR919861	MGYA00023900	https://www.ebi.ac.uk/metagenomics/api/v1/analyses/MGYA00023900/file/SRR919861_FASTQ_otu.tsv	Root-Host_associated-Human-Skin
SRR994545	MGYA00024258	https://www.ebi.ac.uk/metagenomics/api/v1/analyses/MGYA00024258/file/SRR994545_FASTQ_otu.tsv	Root-Host_associated-Human-Skin
SRR995305	MGYA00022700	https://www.ebi.ac.uk/metagenomics/api/v1/analyses/MGYA00022700/file/SRR995305_FASTQ_otu.tsv	Root-Host_associated-Human-Skin
SRR997031	MGYA00023605	https://www.ebi.ac.uk/metagenomics/api/v1/analyses/MGYA00023605/file/SRR997031_FASTQ_otu.tsv	Root-Host_associated-Human-Skin
SRR921149	MGYA00024593	https://www.ebi.ac.uk/metagenomics/api/v1/analyses/MGYA00024593/file/SRR921149_FASTQ_otu.tsv	Root-Host_associated-Human-Skin
SRR920709	MGYA00024022	https://www.ebi.ac.uk/metagenomics/api/v1/analyses/MGYA00024022/file/SRR920709_FASTQ_otu.tsv	Root-Host_associated-Human-Skin
ERR443258	MGYA00412992	https://www.ebi.ac.uk/metagenomics/api/v1/analyses/MGYA00412992/file/ERR443258_FASTQ_SSU_OTU.tsv	Root-Host_associated-Human-Skin
ERR443289	MGYA00420131	https://www.ebi.ac.uk/metagenomics/api/v1/analyses/MGYA00420131/file/ERR443289_FASTQ_SSU_OTU.tsv	Root-Host_associated-Human-Skin
ERR450898	MGYA00415562	https://www.ebi.ac.uk/metagenomics/api/v1/analyses/MGYA00415562/file/ERR450898_FASTQ_SSU_OTU.tsv	Root-Host_associated-Human-Skin
SRR994652	MGYA00023451	https://www.ebi.ac.uk/metagenomics/api/v1/analyses/MGYA00023451/file/SRR994652_FASTQ_otu.tsv	Root-Host_associated-Human-Skin

ERR1760002	MGYA00140568	https://www.ebi.ac.uk/metagenomics/api/v1/analyses/MGYA00140568/file/ERR1760002_MERGED_FASTQ_SSU_OTU.tsv	Root-Host_associated-Human-Skin
SRR5869209	MGYA00420253	https://www.ebi.ac.uk/metagenomics/api/v1/analyses/MGYA00420253/file/SRR5869209_MERGED_FASTQ_SSU_OTU.tsv	Root-Host_associated-Human-Skin
SRR1622819	MGYA00023987	https://www.ebi.ac.uk/metagenomics/api/v1/analyses/MGYA00023987/file/SRR1622819_MERGED_FASTQ_otu.tsv	Root-Host_associated-Human-Skin
SRR995473	MGYA00025103	https://www.ebi.ac.uk/metagenomics/api/v1/analyses/MGYA00025103/file/SRR995473_FASTQ_otu.tsv	Root-Host_associated-Human-Skin
SRR996566	MGYA00025303	https://www.ebi.ac.uk/metagenomics/api/v1/analyses/MGYA00025303/file/SRR996566_FASTQ_otu.tsv	Root-Host_associated-Human-Skin
SRR996914	MGYA00022441	https://www.ebi.ac.uk/metagenomics/api/v1/analyses/MGYA00022441/file/SRR996914_FASTQ_otu.tsv	Root-Host_associated-Human-Skin
SRR5868911	MGYA00418145	https://www.ebi.ac.uk/metagenomics/api/v1/analyses/MGYA00418145/file/SRR5868911_MERGED_FASTQ_SSU_OTU.tsv	Root-Host_associated-Human-Skin
SRR5869484	MGYA00419660	https://www.ebi.ac.uk/metagenomics/api/v1/analyses/MGYA00419660/file/SRR5869484_MERGED_FASTQ_SSU_OTU.tsv	Root-Host_associated-Human-Skin
SRR1622933	MGYA00025289	https://www.ebi.ac.uk/metagenomics/api/v1/analyses/MGYA00025289/file/SRR1622933_MERGED_FASTQ_otu.tsv	Root-Host_associated-Human-Skin
ERR1760128	MGYA00140474	https://www.ebi.ac.uk/metagenomics/api/v1/analyses/MGYA00140474/file/ERR1760128_MERGED_FASTQ_SSU_OTU.tsv	Root-Host_associated-Human-Skin
ERR451427	MGYA00419478	https://www.ebi.ac.uk/metagenomics/api/v1/analyses/MGYA00419478/file/ERR451427_FASTQ_SSU_OTU.tsv	Root-Host_associated-Human-Skin
ERR442991	MGYA00412648	https://www.ebi.ac.uk/metagenomics/api/v1/analyses/MGYA00412648/file/ERR442991_FASTQ_SSU_OTU.tsv	Root-Host_associated-Human-Skin

SRR920155	MGYA00024510	https://www.ebi.ac.uk/metagenomics/api/v1/analyses/MGYA00024510/file/SRR920155_FASTQ_otu.tsv	Root-Host_associated-Human-Skin
SRR994778	MGYA00023030	https://www.ebi.ac.uk/metagenomics/api/v1/analyses/MGYA00023030/file/SRR994778_FASTQ_otu.tsv	Root-Host_associated-Human-Skin
ERR1960664	MGYA00130792	https://www.ebi.ac.uk/metagenomics/api/v1/analyses/MGYA00130792/file/ERR1960664_MERGED_FASTQ_otu.tsv	Root-Host_associated-Human-Skin
SRR1632472	MGYA00023587	https://www.ebi.ac.uk/metagenomics/api/v1/analyses/MGYA00023587/file/SRR1632472_MERGED_FASTQ_otu.tsv	Root-Host_associated-Human-Skin
SRR1923925	MGYA00028241	https://www.ebi.ac.uk/metagenomics/api/v1/analyses/MGYA00028241/file/SRR1923925_FASTQ_otu.tsv	Root-Host_associated-Human-Skin
SRR995588	MGYA00022325	https://www.ebi.ac.uk/metagenomics/api/v1/analyses/MGYA00022325/file/SRR995588_FASTQ_otu.tsv	Root-Host_associated-Human-Skin
ERR451097	MGYA00412635	https://www.ebi.ac.uk/metagenomics/api/v1/analyses/MGYA00412635/file/ERR451097_FASTQ_SSU_OTU.tsv	Root-Host_associated-Human-Skin
SRR1632304	MGYA00023276	https://www.ebi.ac.uk/metagenomics/api/v1/analyses/MGYA00023276/file/SRR1632304_MERGED_FASTQ_otu.tsv	Root-Host_associated-Human-Skin
SRR920997	MGYA00023255	https://www.ebi.ac.uk/metagenomics/api/v1/analyses/MGYA00023255/file/SRR920997_FASTQ_otu.tsv	Root-Host_associated-Human-Skin
SRR920242	MGYA00023498	https://www.ebi.ac.uk/metagenomics/api/v1/analyses/MGYA00023498/file/SRR920242_FASTQ_otu.tsv	Root-Host_associated-Human-Skin
SRR1704572	MGYA00421475	https://www.ebi.ac.uk/metagenomics/api/v1/analyses/MGYA00421475/file/SRR1704572_FASTQ_SSU_OTU.tsv	Root-Host_associated-Human-Skin
ERR1759936	MGYA00140537	https://www.ebi.ac.uk/metagenomics/api/v1/analyses/MGYA00140537/file/ERR1759936_MERGED_FASTQ_SSU_OTU.tsv	Root-Host_associated-Human-Skin

SRR1950760	MGYA00376927	https://www.ebi.ac.uk/metagenomics/api/v1/analyses/MGYA00376927/file/SRR1950760_MERGED_FASTQ_SSU_OTU.tsv	Root-Host_associated-Human-Skin
SRR5869034	MGYA00414879	https://www.ebi.ac.uk/metagenomics/api/v1/analyses/MGYA00414879/file/SRR5869034_MERGED_FASTQ_SSU_OTU.tsv	Root-Host_associated-Human-Skin
SRR5869793	MGYA00415881	https://www.ebi.ac.uk/metagenomics/api/v1/analyses/MGYA00415881/file/SRR5869793_MERGED_FASTQ_SSU_OTU.tsv	Root-Host_associated-Human-Skin
SRR919539	MGYA00024307	https://www.ebi.ac.uk/metagenomics/api/v1/analyses/MGYA00024307/file/SRR919539_FASTQ_otu.tsv	Root-Host_associated-Human-Skin
SRR5870199	MGYA00413948	https://www.ebi.ac.uk/metagenomics/api/v1/analyses/MGYA00413948/file/SRR5870199_MERGED_FASTQ_SSU_OTU.tsv	Root-Host_associated-Human-Skin
SRR920623	MGYA00022761	https://www.ebi.ac.uk/metagenomics/api/v1/analyses/MGYA00022761/file/SRR920623_FASTQ_otu.tsv	Root-Host_associated-Human-Skin
ERR442940	MGYA00412395	https://www.ebi.ac.uk/metagenomics/api/v1/analyses/MGYA00412395/file/ERR442940_FASTQ_SSU_OTU.tsv	Root-Host_associated-Human-Skin
SRR5869455	MGYA00412811	https://www.ebi.ac.uk/metagenomics/api/v1/analyses/MGYA00412811/file/SRR5869455_MERGED_FASTQ_SSU_OTU.tsv	Root-Host_associated-Human-Skin
SRR1631670	MGYA00023835	https://www.ebi.ac.uk/metagenomics/api/v1/analyses/MGYA00023835/file/SRR1631670_MERGED_FASTQ_otu.tsv	Root-Host_associated-Human-Skin
SRR1631349	MGYA00022812	https://www.ebi.ac.uk/metagenomics/api/v1/analyses/MGYA00022812/file/SRR1631349_MERGED_FASTQ_otu.tsv	Root-Host_associated-Human-Skin
SRR996262	MGYA00023503	https://www.ebi.ac.uk/metagenomics/api/v1/analyses/MGYA00023503/file/SRR996262_FASTQ_otu.tsv	Root-Host_associated-Human-Skin
SRR1632989	MGYA00025161	https://www.ebi.ac.uk/metagenomics/api/v1/analyses/MGYA00025161/file/SRR1632989_MERGED_FASTQ_otu.tsv	Root-Host_associated-Human-Skin

ERR2538469	MGYA00381476	https://www.ebi.ac.uk/metagenomics/api/v1/analyses/MGYA00381476/file/ERR2538469_FASTQ_SSU_OTU.tsv	Root-Host_associated-Human-Skin
SRR996603	MGYA00024574	https://www.ebi.ac.uk/metagenomics/api/v1/analyses/MGYA00024574/file/SRR996603_FASTQ_otu.tsv	Root-Host_associated-Human-Skin
SRR6757699	MGYA00380812	https://www.ebi.ac.uk/metagenomics/api/v1/analyses/MGYA00380812/file/SRR6757699_MERGED_FASTQ_SSU_OTU.tsv	Root-Host_associated-Human-Skin
SRR995716	MGYA00023358	https://www.ebi.ac.uk/metagenomics/api/v1/analyses/MGYA00023358/file/SRR995716_FASTQ_otu.tsv	Root-Host_associated-Human-Skin
SRR995060	MGYA00024056	https://www.ebi.ac.uk/metagenomics/api/v1/analyses/MGYA00024056/file/SRR995060_FASTQ_otu.tsv	Root-Host_associated-Human-Skin
ERR1760070	MGYA00140354	https://www.ebi.ac.uk/metagenomics/api/v1/analyses/MGYA00140354/file/ERR1760070_MERGED_FASTQ_SSU_OTU.tsv	Root-Host_associated-Human-Skin
SRR997092	MGYA00022513	https://www.ebi.ac.uk/metagenomics/api/v1/analyses/MGYA00022513/file/SRR997092_FASTQ_otu.tsv	Root-Host_associated-Human-Skin
SRR6757648	MGYA00380763	https://www.ebi.ac.uk/metagenomics/api/v1/analyses/MGYA00380763/file/SRR6757648_MERGED_FASTQ_SSU_OTU.tsv	Root-Host_associated-Human-Skin
SRR994994	MGYA00023929	https://www.ebi.ac.uk/metagenomics/api/v1/analyses/MGYA00023929/file/SRR994994_FASTQ_otu.tsv	Root-Host_associated-Human-Skin
SRR996514	MGYA00023068	https://www.ebi.ac.uk/metagenomics/api/v1/analyses/MGYA00023068/file/SRR996514_FASTQ_otu.tsv	Root-Host_associated-Human-Skin
SRR995144	MGYA00024136	https://www.ebi.ac.uk/metagenomics/api/v1/analyses/MGYA00024136/file/SRR995144_FASTQ_otu.tsv	Root-Host_associated-Human-Skin
SRR995525	MGYA00023419	https://www.ebi.ac.uk/metagenomics/api/v1/analyses/MGYA00023419/file/SRR995525_FASTQ_otu.tsv	Root-Host_associated-Human-Skin

SRR919594	MGYA00024953	https://www.ebi.ac.uk/metagenomics/api/v1/analyses/MGYA00024953/file/SRR919594_FASTQ_otu.tsv	Root-Host_associated-Human-Skin
SRR996397	MGYA00024973	https://www.ebi.ac.uk/metagenomics/api/v1/analyses/MGYA00024973/file/SRR996397_FASTQ_otu.tsv	Root-Host_associated-Human-Skin
SRR996430	MGYA00024479	https://www.ebi.ac.uk/metagenomics/api/v1/analyses/MGYA00024479/file/SRR996430_FASTQ_otu.tsv	Root-Host_associated-Human-Skin
SRR1704278	MGYA00428891	https://www.ebi.ac.uk/metagenomics/api/v1/analyses/MGYA00428891/file/SRR1704278_FASTQ_SSU_OTU.tsv	Root-Host_associated-Human-Skin
SRR5868608	MGYA00421672	https://www.ebi.ac.uk/metagenomics/api/v1/analyses/MGYA00421672/file/SRR5868608_MERGED_FASTQ_SSU_OTU.tsv	Root-Host_associated-Human-Skin
SRR996346	MGYA00022283	https://www.ebi.ac.uk/metagenomics/api/v1/analyses/MGYA00022283/file/SRR996346_FASTQ_otu.tsv	Root-Host_associated-Human-Skin
SRR996893	MGYA00022800	https://www.ebi.ac.uk/metagenomics/api/v1/analyses/MGYA00022800/file/SRR996893_FASTQ_otu.tsv	Root-Host_associated-Human-Skin
ERR442027	MGYA00385152	https://www.ebi.ac.uk/metagenomics/api/v1/analyses/MGYA00385152/file/ERR442027_FASTQ_SSU_OTU.tsv	Root-Host_associated-Human-Skin
SRR995986	MGYA00022937	https://www.ebi.ac.uk/metagenomics/api/v1/analyses/MGYA00022937/file/SRR995986_FASTQ_otu.tsv	Root-Host_associated-Human-Skin
SRR994861	MGYA00022391	https://www.ebi.ac.uk/metagenomics/api/v1/analyses/MGYA00022391/file/SRR994861_FASTQ_otu.tsv	Root-Host_associated-Human-Skin
SRR996727	MGYA00023426	https://www.ebi.ac.uk/metagenomics/api/v1/analyses/MGYA00023426/file/SRR996727_FASTQ_otu.tsv	Root-Host_associated-Human-Skin
SRR996080	MGYA00024366	https://www.ebi.ac.uk/metagenomics/api/v1/analyses/MGYA00024366/file/SRR996080_FASTQ_otu.tsv	Root-Host_associated-Human-Skin

SRR994939	MGYA00024969	https://www.ebi.ac.uk/metagenomics/api/v1/analyses/MGYA00024969/file/SRR994939_FASTQ_otu.tsv	Root-Host_associated-Human-Skin
ERR1960825	MGYA00130783	https://www.ebi.ac.uk/metagenomics/api/v1/analyses/MGYA00130783/file/ERR1960825_MERGED_FASTQ_otu.tsv	Root-Host_associated-Human-Skin
SRR1922545	MGYA00028408	https://www.ebi.ac.uk/metagenomics/api/v1/analyses/MGYA00028408/file/SRR1922545_FASTQ_otu.tsv	Root-Host_associated-Human-Skin
SRR5869110	MGYA00422565	https://www.ebi.ac.uk/metagenomics/api/v1/analyses/MGYA00422565/file/SRR5869110_MERGED_FASTQ_SSU_OTU.tsv	Root-Host_associated-Human-Skin
ERR1960791	MGYA00130706	https://www.ebi.ac.uk/metagenomics/api/v1/analyses/MGYA00130706/file/ERR1960791_MERGED_FASTQ_otu.tsv	Root-Host_associated-Human-Skin
ERR1701232	MGYA00084252	https://www.ebi.ac.uk/metagenomics/api/v1/analyses/MGYA00084252/file/ERR1701232_FASTQ_otu.tsv	Root-Host_associated-Human-Skin
SRR921147	MGYA00024591	https://www.ebi.ac.uk/metagenomics/api/v1/analyses/MGYA00024591/file/SRR921147_FASTQ_otu.tsv	Root-Host_associated-Human-Skin
ERR2538415	MGYA00381422	https://www.ebi.ac.uk/metagenomics/api/v1/analyses/MGYA00381422/file/ERR2538415_FASTQ_SSU_OTU.tsv	Root-Host_associated-Human-Skin
SRR5869571	MGYA00419648	https://www.ebi.ac.uk/metagenomics/api/v1/analyses/MGYA00419648/file/SRR5869571_MERGED_FASTQ_SSU_OTU.tsv	Root-Host_associated-Human-Skin
SRR5869903	MGYA00423468	https://www.ebi.ac.uk/metagenomics/api/v1/analyses/MGYA00423468/file/SRR5869903_MERGED_FASTQ_SSU_OTU.tsv	Root-Host_associated-Human-Skin
SRR6757634	MGYA00380815	https://www.ebi.ac.uk/metagenomics/api/v1/analyses/MGYA00380815/file/SRR6757634_MERGED_FASTQ_SSU_OTU.tsv	Root-Host_associated-Human-Skin
SRR1924019	MGYA00028395	https://www.ebi.ac.uk/metagenomics/api/v1/analyses/MGYA00028395/file/SRR1924019_FASTQ_otu.tsv	Root-Host_associated-Human-Skin

SRR1704741	MGYA00425791	https://www.ebi.ac.uk/metagenomics/api/v1/analyses/MGYA00425791/file/SRR1704741_FASTQ_SSU_OTU.tsv	Root-Host_associated-Human-Skin
ERR2538363	MGYA00381370	https://www.ebi.ac.uk/metagenomics/api/v1/analyses/MGYA00381370/file/ERR2538363_FASTQ_SSU_OTU.tsv	Root-Host_associated-Human-Skin
SRR1622817	MGYA00023983	https://www.ebi.ac.uk/metagenomics/api/v1/analyses/MGYA00023983/file/SRR1622817_MERGED_FASTQ_otu.tsv	Root-Host_associated-Human-Skin
ERR1960740	MGYA00130762	https://www.ebi.ac.uk/metagenomics/api/v1/analyses/MGYA00130762/file/ERR1960740_MERGED_FASTQ_otu.tsv	Root-Host_associated-Human-Skin
ERR443520	MGYA00414264	https://www.ebi.ac.uk/metagenomics/api/v1/analyses/MGYA00414264/file/ERR443520_FASTQ_SSU_OTU.tsv	Root-Host_associated-Human-Skin
SRR2005643	MGYA00381302	https://www.ebi.ac.uk/metagenomics/api/v1/analyses/MGYA00381302/file/SRR2005643_MERGED_FASTQ_SSU_OTU.tsv	Root-Host_associated-Human-Skin
SRR1950695	MGYA00376862	https://www.ebi.ac.uk/metagenomics/api/v1/analyses/MGYA00376862/file/SRR1950695_MERGED_FASTQ_SSU_OTU.tsv	Root-Host_associated-Human-Skin
SRR996568	MGYA00025295	https://www.ebi.ac.uk/metagenomics/api/v1/analyses/MGYA00025295/file/SRR996568_FASTQ_otu.tsv	Root-Host_associated-Human-Skin
ERR450722	MGYA00416746	https://www.ebi.ac.uk/metagenomics/api/v1/analyses/MGYA00416746/file/ERR450722_FASTQ_SSU_OTU.tsv	Root-Host_associated-Human-Skin
SRR1923801	MGYA00028381	https://www.ebi.ac.uk/metagenomics/api/v1/analyses/MGYA00028381/file/SRR1923801_FASTQ_otu.tsv	Root-Host_associated-Human-Skin
SRR995143	MGYA00024139	https://www.ebi.ac.uk/metagenomics/api/v1/analyses/MGYA00024139/file/SRR995143_FASTQ_otu.tsv	Root-Host_associated-Human-Skin
SRR995522	MGYA00023620	https://www.ebi.ac.uk/metagenomics/api/v1/analyses/MGYA00023620/file/SRR995522_FASTQ_otu.tsv	Root-Host_associated-Human-Skin

SRR1632409	MGYA00023544	https://www.ebi.ac.uk/metagenomics/api/v1/analyses/MGYA00023544/file/SRR1632409_MERGED_FASTQ_otu.tsv	Root-Host_associated-Human-Skin
SRR996437	MGYA00024472	https://www.ebi.ac.uk/metagenomics/api/v1/analyses/MGYA00024472/file/SRR996437_FASTQ_otu.tsv	Root-Host_associated-Human-Skin
SRR919593	MGYA00024950	https://www.ebi.ac.uk/metagenomics/api/v1/analyses/MGYA00024950/file/SRR919593_FASTQ_otu.tsv	Root-Host_associated-Human-Skin
SRR996390	MGYA00024978	https://www.ebi.ac.uk/metagenomics/api/v1/analyses/MGYA00024978/file/SRR996390_FASTQ_otu.tsv	Root-Host_associated-Human-Skin
SRR996894	MGYA00022795	https://www.ebi.ac.uk/metagenomics/api/v1/analyses/MGYA00022795/file/SRR996894_FASTQ_otu.tsv	Root-Host_associated-Human-Skin
SRR996341	MGYA00022276	https://www.ebi.ac.uk/metagenomics/api/v1/analyses/MGYA00022276/file/SRR996341_FASTQ_otu.tsv	Root-Host_associated-Human-Skin
SRR994866	MGYA00022392	https://www.ebi.ac.uk/metagenomics/api/v1/analyses/MGYA00022392/file/SRR994866_FASTQ_otu.tsv	Root-Host_associated-Human-Skin
SRR995981	MGYA00022936	https://www.ebi.ac.uk/metagenomics/api/v1/analyses/MGYA00022936/file/SRR995981_FASTQ_otu.tsv	Root-Host_associated-Human-Skin
ERR442020	MGYA00385175	https://www.ebi.ac.uk/metagenomics/api/v1/analyses/MGYA00385175/file/ERR442020_FASTQ_SSU_OTU.tsv	Root-Host_associated-Human-Skin
SRR1704168	MGYA00415676	https://www.ebi.ac.uk/metagenomics/api/v1/analyses/MGYA00415676/file/SRR1704168_FASTQ_SSU_OTU.tsv	Root-Host_associated-Human-Skin
SRR996720	MGYA00023433	https://www.ebi.ac.uk/metagenomics/api/v1/analyses/MGYA00023433/file/SRR996720_FASTQ_otu.tsv	Root-Host_associated-Human-Skin
SRR921018	MGYA00023333	https://www.ebi.ac.uk/metagenomics/api/v1/analyses/MGYA00023333/file/SRR921018_FASTQ_otu.tsv	Root-Host_associated-Human-Skin

SRR995635	MGYA00022342	https://www.ebi.ac.uk/metagenomics/api/v1/analyses/MGYA00022342/file/SRR995635_FASTQ_otu.tsv	Root-Host_associated-Human-Skin
SRR920658	MGYA00025221	https://www.ebi.ac.uk/metagenomics/api/v1/analyses/MGYA00025221/file/SRR920658_FASTQ_otu.tsv	Root-Host_associated-Human-Skin
ERR1960822	MGYA00130788	https://www.ebi.ac.uk/metagenomics/api/v1/analyses/MGYA00130788/file/ERR1960822_MERGED_FASTQ_otu.tsv	Root-Host_associated-Human-Skin
ERR443630	MGYA00421385	https://www.ebi.ac.uk/metagenomics/api/v1/analyses/MGYA00421385/file/ERR443630_FASTQ_SSU_OTU.tsv	Root-Host_associated-Human-Skin
SRR1620020	MGYA00024918	https://www.ebi.ac.uk/metagenomics/api/v1/analyses/MGYA00024918/file/SRR1620020_MERGED_FASTQ_otu.tsv	Root-Host_associated-Human-Skin
ERR981212	MGYA00010282	https://www.ebi.ac.uk/metagenomics/api/v1/analyses/MGYA00010282/file/ERR981212_MERGED_FASTQ_otu.tsv	Root-Host_associated-Human-Skin
SRR919868	MGYA00023906	https://www.ebi.ac.uk/metagenomics/api/v1/analyses/MGYA00023906/file/SRR919868_FASTQ_otu.tsv	Root-Host_associated-Human-Skin
SRR921140	MGYA00024586	https://www.ebi.ac.uk/metagenomics/api/v1/analyses/MGYA00024586/file/SRR921140_FASTQ_otu.tsv	Root-Host_associated-Human-Skin
ERR1701235	MGYA00084297	https://www.ebi.ac.uk/metagenomics/api/v1/analyses/MGYA00084297/file/ERR1701235_FASTQ_otu.tsv	Root-Host_associated-Human-Skin
ERR1960796	MGYA00130875	https://www.ebi.ac.uk/metagenomics/api/v1/analyses/MGYA00130875/file/ERR1960796_MERGED_FASTQ_otu.tsv	Root-Host_associated-Human-Skin
SRR997038	MGYA00023608	https://www.ebi.ac.uk/metagenomics/api/v1/analyses/MGYA00023608/file/SRR997038_FASTQ_otu.tsv	Root-Host_associated-Human-Skin
ERR2538412	MGYA00381419	https://www.ebi.ac.uk/metagenomics/api/v1/analyses/MGYA00381419/file/ERR2538412_FASTQ_SSU_OTU.tsv	Root-Host_associated-Human-Skin

SRR5869904	MGYA00416945	https://www.ebi.ac.uk/metagenomics/api/v1/analyses/MGYA00416945/file/SRR5869904_MERGED_FASTQ_SSU_OTU.tsv	Root-Host_associated-Human-Skin
SRR1622810	MGYA00023982	https://www.ebi.ac.uk/metagenomics/api/v1/analyses/MGYA00023982/file/SRR1622810_MERGED_FASTQ_otu.tsv	Root-Host_associated-Human-Skin
ERR2538364	MGYA00381371	https://www.ebi.ac.uk/metagenomics/api/v1/analyses/MGYA00381371/file/ERR2538364_FASTQ_SSU_OTU.tsv	Root-Host_associated-Human-Skin
SRR6757633	MGYA00380750	https://www.ebi.ac.uk/metagenomics/api/v1/analyses/MGYA00380750/file/SRR6757633_MERGED_FASTQ_SSU_OTU.tsv	Root-Host_associated-Human-Skin
ERR443527	MGYA00422945	https://www.ebi.ac.uk/metagenomics/api/v1/analyses/MGYA00422945/file/ERR443527_FASTQ_SSU_OTU.tsv	Root-Host_associated-Human-Skin
ERR1960747	MGYA00130856	https://www.ebi.ac.uk/metagenomics/api/v1/analyses/MGYA00130856/file/ERR1960747_MERGED_FASTQ_otu.tsv	Root-Host_associated-Human-Skin
ERR443280	MGYA00425768	https://www.ebi.ac.uk/metagenomics/api/v1/analyses/MGYA00425768/file/ERR443280_FASTQ_SSU_OTU.tsv	Root-Host_associated-Human-Skin
ERR451504	MGYA00424289	https://www.ebi.ac.uk/metagenomics/api/v1/analyses/MGYA00424289/file/ERR451504_FASTQ_SSU_OTU.tsv	Root-Host_associated-Human-Skin
SRR2005644	MGYA00381303	https://www.ebi.ac.uk/metagenomics/api/v1/analyses/MGYA00381303/file/SRR2005644_MERGED_FASTQ_SSU_OTU.tsv	Root-Host_associated-Human-Skin
SRR1950692	MGYA00376859	https://www.ebi.ac.uk/metagenomics/api/v1/analyses/MGYA00376859/file/SRR1950692_MERGED_FASTQ_SSU_OTU.tsv	Root-Host_associated-Human-Skin
SRR5869661	MGYA00416992	https://www.ebi.ac.uk/metagenomics/api/v1/analyses/MGYA00416992/file/SRR5869661_MERGED_FASTQ_SSU_OTU.tsv	Root-Host_associated-Human-Skin
ERR450725	MGYA00418480	https://www.ebi.ac.uk/metagenomics/api/v1/analyses/MGYA00418480/file/ERR450725_FASTQ_SSU_OTU.tsv	Root-Host_associated-Human-Skin

ERR443146	MGYA00420147	https://www.ebi.ac.uk/metagenomics/api/v1/analyses/MGYA00420147/file/ERR443146_FASTQ_SSU_OTU.tsv	Root-Host_associated-Human-Skin
SRR1622934	MGYA00025290	https://www.ebi.ac.uk/metagenomics/api/v1/analyses/MGYA00025290/file/SRR1622934_MERGED_FASTQ_otu.tsv	Root-Host_associated-Human-Skin
SRR5869483	MGYA00414861	https://www.ebi.ac.uk/metagenomics/api/v1/analyses/MGYA00414861/file/SRR5869483_MERGED_FASTQ_SSU_OTU.tsv	Root-Host_associated-Human-Skin
SRR1704662	MGYA00421465	https://www.ebi.ac.uk/metagenomics/api/v1/analyses/MGYA00421465/file/SRR1704662_FASTQ_SSU_OTU.tsv	Root-Host_associated-Human-Skin
ERR1960663	MGYA00130740	https://www.ebi.ac.uk/metagenomics/api/v1/analyses/MGYA00130740/file/ERR1960663_MERGED_FASTQ_otu.tsv	Root-Host_associated-Human-Skin
SRR920152	MGYA00024517	https://www.ebi.ac.uk/metagenomics/api/v1/analyses/MGYA00024517/file/SRR920152_FASTQ_otu.tsv	Root-Host_associated-Human-Skin
SRR995698	MGYA00023000	https://www.ebi.ac.uk/metagenomics/api/v1/analyses/MGYA00023000/file/SRR995698_FASTQ_otu.tsv	Root-Host_associated-Human-Skin
SRR1920445	MGYA00028277	https://www.ebi.ac.uk/metagenomics/api/v1/analyses/MGYA00028277/file/SRR1920445_FASTQ_otu.tsv	Root-Host_associated-Human-Skin
SRR996839	MGYA00023872	https://www.ebi.ac.uk/metagenomics/api/v1/analyses/MGYA00023872/file/SRR996839_FASTQ_otu.tsv	Root-Host_associated-Human-Skin
SRR1923922	MGYA00028274	https://www.ebi.ac.uk/metagenomics/api/v1/analyses/MGYA00028274/file/SRR1923922_FASTQ_otu.tsv	Root-Host_associated-Human-Skin
ERR451041	MGYA00428773	https://www.ebi.ac.uk/metagenomics/api/v1/analyses/MGYA00428773/file/ERR451041_FASTQ_SSU_OTU.tsv	Root-Host_associated-Human-Skin
SRR1632475	MGYA00022488	https://www.ebi.ac.uk/metagenomics/api/v1/analyses/MGYA00022488/file/SRR1632475_MERGED_FASTQ_otu.tsv	Root-Host_associated-Human-Skin

ERR443714	MGYA00419436	https://www.ebi.ac.uk/metagenomics/api/v1/analyses/MGYA00419436/file/ERR443714_FASTQ_SSU_OTU.tsv	Root-Host_associated-Human-Skin
SRR920245	MGYA00023491	https://www.ebi.ac.uk/metagenomics/api/v1/analyses/MGYA00023491/file/SRR920245_FASTQ_otu.tsv	Root-Host_associated-Human-Skin
SRR5870258	MGYA00416926	https://www.ebi.ac.uk/metagenomics/api/v1/analyses/MGYA00416926/file/SRR5870258_MERGED_FASTQ_SSU_OTU.tsv	Root-Host_associated-Human-Skin
SRR1632303	MGYA00023271	https://www.ebi.ac.uk/metagenomics/api/v1/analyses/MGYA00023271/file/SRR1632303_MERGED_FASTQ_otu.tsv	Root-Host_associated-Human-Skin
SRR920990	MGYA00023250	https://www.ebi.ac.uk/metagenomics/api/v1/analyses/MGYA00023250/file/SRR920990_FASTQ_otu.tsv	Root-Host_associated-Human-Skin
SRR1950767	MGYA00376934	https://www.ebi.ac.uk/metagenomics/api/v1/analyses/MGYA00376934/file/SRR1950767_MERGED_FASTQ_SSU_OTU.tsv	Root-Host_associated-Human-Skin
SRR5869033	MGYA00416495	https://www.ebi.ac.uk/metagenomics/api/v1/analyses/MGYA00416495/file/SRR5869033_MERGED_FASTQ_SSU_OTU.tsv	Root-Host_associated-Human-Skin
ERR1759931	MGYA00140403	https://www.ebi.ac.uk/metagenomics/api/v1/analyses/MGYA00140403/file/ERR1759931_MERGED_FASTQ_SSU_OTU.tsv	Root-Host_associated-Human-Skin
SRR1624809	MGYA00024677	https://www.ebi.ac.uk/metagenomics/api/v1/analyses/MGYA00024677/file/SRR1624809_MERGED_FASTQ_otu.tsv	Root-Host_associated-Human-Skin
SRR995649	MGYA00024625	https://www.ebi.ac.uk/metagenomics/api/v1/analyses/MGYA00024625/file/SRR995649_FASTQ_otu.tsv	Root-Host_associated-Human-Skin
SRR920624	MGYA00022768	https://www.ebi.ac.uk/metagenomics/api/v1/analyses/MGYA00022768/file/SRR920624_FASTQ_otu.tsv	Root-Host_associated-Human-Skin
SRR1631677	MGYA00023828	https://www.ebi.ac.uk/metagenomics/api/v1/analyses/MGYA00023828/file/SRR1631677_MERGED_FASTQ_otu.tsv	Root-Host_associated-Human-Skin

SRR5869820	MGYA00420716	https://www.ebi.ac.uk/metagenomics/api/v1/analyses/MGYA00420716/file/SRR5869820_MERGED_FASTQ_SSU_OTU.tsv	Root-Host_associated-Human-Skin
SRR996265	MGYA00023510	https://www.ebi.ac.uk/metagenomics/api/v1/analyses/MGYA00023510/file/SRR996265_FASTQ_otu.tsv	Root-Host_associated-Human-Skin
ERR450788	MGYA00426839	https://www.ebi.ac.uk/metagenomics/api/v1/analyses/MGYA00426839/file/ERR450788_FASTQ_SSU_OTU.tsv	Root-Host_associated-Human-Skin
SRR1631088	MGYA00023781	https://www.ebi.ac.uk/metagenomics/api/v1/analyses/MGYA00023781/file/SRR1631088_MERGED_FASTQ_otu.tsv	Root-Host_associated-Human-Skin
SRR996604	MGYA00024575	https://www.ebi.ac.uk/metagenomics/api/v1/analyses/MGYA00024575/file/SRR996604_FASTQ_otu.tsv	Root-Host_associated-Human-Skin
SRR995711	MGYA00023355	https://www.ebi.ac.uk/metagenomics/api/v1/analyses/MGYA00023355/file/SRR995711_FASTQ_otu.tsv	Root-Host_associated-Human-Skin
ERR1701249	MGYA00084244	https://www.ebi.ac.uk/metagenomics/api/v1/analyses/MGYA00084244/file/ERR1701249_FASTQ_otu.tsv	Root-Host_associated-Human-Skin
ERR1701298	MGYA00084249	https://www.ebi.ac.uk/metagenomics/api/v1/analyses/MGYA00084249/file/ERR1701298_FASTQ_otu.tsv	Root-Host_associated-Human-Skin
SRR995067	MGYA00024061	https://www.ebi.ac.uk/metagenomics/api/v1/analyses/MGYA00024061/file/SRR995067_FASTQ_otu.tsv	Root-Host_associated-Human-Skin
ERR1760077	MGYA00140514	https://www.ebi.ac.uk/metagenomics/api/v1/analyses/MGYA00140514/file/ERR1760077_MERGED_FASTQ_SSU_OTU.tsv	Root-Host_associated-Human-Skin
SRR997095	MGYA00022512	https://www.ebi.ac.uk/metagenomics/api/v1/analyses/MGYA00022512/file/SRR997095_FASTQ_otu.tsv	Root-Host_associated-Human-Skin
SRR994993	MGYA00023932	https://www.ebi.ac.uk/metagenomics/api/v1/analyses/MGYA00023932/file/SRR994993_FASTQ_otu.tsv	Root-Host_associated-Human-Skin

SRR1631438	MGYA00025316	https://www.ebi.ac.uk/metagenomics/api/v1/analyses/MGYA00025316/file/SRR1631438_MERGED_FASTQ_otu.tsv	Root-Host_associated-Human-Skin
SRR2005638	MGYA00381298	https://www.ebi.ac.uk/metagenomics/api/v1/analyses/MGYA00381298/file/SRR2005638_MERGED_FASTQ_SSU_OTU.tsv	Root-Host_associated-Human-Skin
SRR996513	MGYA00023075	https://www.ebi.ac.uk/metagenomics/api/v1/analyses/MGYA00023075/file/SRR996513_FASTQ_otu.tsv	Root-Host_associated-Human-Skin
ERR1960756	MGYA00130861	https://www.ebi.ac.uk/metagenomics/api/v1/analyses/MGYA00130861/file/ERR1960756_MERGED_FASTQ_otu.tsv	Root-Host_associated-Human-Skin
ERR451515	MGYA00415526	https://www.ebi.ac.uk/metagenomics/api/v1/analyses/MGYA00415526/file/ERR451515_FASTQ_SSU_OTU.tsv	Root-Host_associated-Human-Skin
SRR5869211	MGYA00429063	https://www.ebi.ac.uk/metagenomics/api/v1/analyses/MGYA00429063/file/SRR5869211_MERGED_FASTQ_SSU_OTU.tsv	Root-Host_associated-Human-Skin
ERR2538375	MGYA00381382	https://www.ebi.ac.uk/metagenomics/api/v1/analyses/MGYA00381382/file/ERR2538375_FASTQ_SSU_OTU.tsv	Root-Host_associated-Human-Skin
SRR6757622	MGYA00380739	https://www.ebi.ac.uk/metagenomics/api/v1/analyses/MGYA00380739/file/SRR6757622_MERGED_FASTQ_SSU_OTU.tsv	Root-Host_associated-Human-Skin
SRR5868823	MGYA00420288	https://www.ebi.ac.uk/metagenomics/api/v1/analyses/MGYA00420288/file/SRR5868823_MERGED_FASTQ_SSU_OTU.tsv	Root-Host_associated-Human-Skin
SRR1923817	MGYA00028403	https://www.ebi.ac.uk/metagenomics/api/v1/analyses/MGYA00028403/file/SRR1923817_FASTQ_otu.tsv	Root-Host_associated-Human-Skin
SRR1631455	MGYA00025124	https://www.ebi.ac.uk/metagenomics/api/v1/analyses/MGYA00025124/file/SRR1631455_MERGED_FASTQ_otu.tsv	Root-Host_associated-Human-Skin
SRR1704336	MGYA00426963	https://www.ebi.ac.uk/metagenomics/api/v1/analyses/MGYA00426963/file/SRR1704336_FASTQ_SSU_OTU.tsv	Root-Host_associated-Human-Skin

SRR5868797	MGYA00427165	https://www.ebi.ac.uk/metagenomics/api/v1/analyses/MGYA00427165/file/SRR5868797_MERGED_FASTQ_SSU_OTU.tsv	Root-Host_associated-Human-Skin
SRR1920902	MGYA00028249	https://www.ebi.ac.uk/metagenomics/api/v1/analyses/MGYA00028249/file/SRR1920902_FASTQ_otu.tsv	Root-Host_associated-Human-Skin
SRR2005655	MGYA00381313	https://www.ebi.ac.uk/metagenomics/api/v1/analyses/MGYA00381313/file/SRR2005655_MERGED_FASTQ_SSU_OTU.tsv	Root-Host_associated-Human-Skin
SRR996208	MGYA00024176	https://www.ebi.ac.uk/metagenomics/api/v1/analyses/MGYA00024176/file/SRR996208_FASTQ_otu.tsv	Root-Host_associated-Human-Skin
SRR5869106	MGYA00414877	https://www.ebi.ac.uk/metagenomics/api/v1/analyses/MGYA00414877/file/SRR5869106_MERGED_FASTQ_SSU_OTU.tsv	Root-Host_associated-Human-Skin
SRR1631484	MGYA00024936	https://www.ebi.ac.uk/metagenomics/api/v1/analyses/MGYA00024936/file/SRR1631484_MERGED_FASTQ_otu.tsv	Root-Host_associated-Human-Skin
ERR1960833	MGYA00130832	https://www.ebi.ac.uk/metagenomics/api/v1/analyses/MGYA00130832/file/ERR1960833_MERGED_FASTQ_otu.tsv	Root-Host_associated-Human-Skin
SRR996669	MGYA00024781	https://www.ebi.ac.uk/metagenomics/api/v1/analyses/MGYA00024781/file/SRR996669_FASTQ_otu.tsv	Root-Host_associated-Human-Skin
ERR2538403	MGYA00381410	https://www.ebi.ac.uk/metagenomics/api/v1/analyses/MGYA00381410/file/ERR2538403_FASTQ_SSU_OTU.tsv	Root-Host_associated-Human-Skin
SRR1633017	MGYA00023959	https://www.ebi.ac.uk/metagenomics/api/v1/analyses/MGYA00023959/file/SRR1633017_MERGED_FASTQ_otu.tsv	Root-Host_associated-Human-Skin
ERR1701224	MGYA00084304	https://www.ebi.ac.uk/metagenomics/api/v1/analyses/MGYA00084304/file/ERR1701224_FASTQ_otu.tsv	Root-Host_associated-Human-Skin
ERR443779	MGYA00428389	https://www.ebi.ac.uk/metagenomics/api/v1/analyses/MGYA00428389/file/ERR443779_FASTQ_SSU_OTU.tsv	Root-Host_associated-Human-Skin

SRR920228	MGYA00023283	https://www.ebi.ac.uk/metagenomics/api/v1/analyses/MGYA00023283/file/SRR920228_FASTQ_otu.tsv	Root-Host_associated-Human-Skin
SRR994405	MGYA00025139	https://www.ebi.ac.uk/metagenomics/api/v1/analyses/MGYA00025139/file/SRR994405_FASTQ_otu.tsv	Root-Host_associated-Human-Skin
SRR994877	MGYA00025270	https://www.ebi.ac.uk/metagenomics/api/v1/analyses/MGYA00025270/file/SRR994877_FASTQ_otu.tsv	Root-Host_associated-Human-Skin
ERR442031	MGYA00385166	https://www.ebi.ac.uk/metagenomics/api/v1/analyses/MGYA00385166/file/ERR442031_FASTQ_SSU_OTU.tsv	Root-Host_associated-Human-Skin
SRR919921	MGYA00025198	https://www.ebi.ac.uk/metagenomics/api/v1/analyses/MGYA00025198/file/SRR919921_FASTQ_otu.tsv	Root-Host_associated-Human-Skin
SRR919553	MGYA00024502	https://www.ebi.ac.uk/metagenomics/api/v1/analyses/MGYA00024502/file/SRR919553_FASTQ_otu.tsv	Root-Host_associated-Human-Skin
SRR1624864	MGYA00024464	https://www.ebi.ac.uk/metagenomics/api/v1/analyses/MGYA00024464/file/SRR1624864_MERGED_FASTQ_otu.tsv	Root-Host_associated-Human-Skin
SRR995624	MGYA00025226	https://www.ebi.ac.uk/metagenomics/api/v1/analyses/MGYA00025226/file/SRR995624_FASTQ_otu.tsv	Root-Host_associated-Human-Skin
ERR443318	MGYA00416291	https://www.ebi.ac.uk/metagenomics/api/v1/analyses/MGYA00416291/file/ERR443318_FASTQ_SSU_OTU.tsv	Root-Host_associated-Human-Skin
SRR920698	MGYA00024848	https://www.ebi.ac.uk/metagenomics/api/v1/analyses/MGYA00024848/file/SRR920698_FASTQ_otu.tsv	Root-Host_associated-Human-Skin
SRR994712	MGYA00025092	https://www.ebi.ac.uk/metagenomics/api/v1/analyses/MGYA00025092/file/SRR994712_FASTQ_otu.tsv	Root-Host_associated-Human-Skin
SRR996854	MGYA00023220	https://www.ebi.ac.uk/metagenomics/api/v1/analyses/MGYA00023220/file/SRR996854_FASTQ_otu.tsv	Root-Host_associated-Human-Skin

SRR995294	MGYA00023598	https://www.ebi.ac.uk/metagenomics/api/v1/analyses/MGYA00023598/file/SRR995294_FASTQ_otu.tsv	Root-Host_associated-Human-Skin
SRR1632418	MGYA00023809	https://www.ebi.ac.uk/metagenomics/api/v1/analyses/MGYA00023809/file/SRR1632418_MERGED_FASTQ_otu.tsv	Root-Host_associated-Human-Skin
SRR996163	MGYA00023203	https://www.ebi.ac.uk/metagenomics/api/v1/analyses/MGYA00023203/file/SRR996163_FASTQ_otu.tsv	Root-Host_associated-Human-Skin
SRR1631048	MGYA00025015	https://www.ebi.ac.uk/metagenomics/api/v1/analyses/MGYA00025015/file/SRR1631048_MERGED_FASTQ_otu.tsv	Root-Host_associated-Human-Skin
ERR1701289	MGYA00084248	https://www.ebi.ac.uk/metagenomics/api/v1/analyses/MGYA00084248/file/ERR1701289_FASTQ_otu.tsv	Root-Host_associated-Human-Skin
SRR994636	MGYA00022545	https://www.ebi.ac.uk/metagenomics/api/v1/analyses/MGYA00022545/file/SRR994636_FASTQ_otu.tsv	Root-Host_associated-Human-Skin
SRR995076	MGYA00024644	https://www.ebi.ac.uk/metagenomics/api/v1/analyses/MGYA00024644/file/SRR995076_FASTQ_otu.tsv	Root-Host_associated-Human-Skin
ERR1760066	MGYA00140521	https://www.ebi.ac.uk/metagenomics/api/v1/analyses/MGYA00140521/file/ERR1760066_MERGED_FASTQ_SSU_OTU.tsv	Root-Host_associated-Human-Skin
SRR996970	MGYA00022646	https://www.ebi.ac.uk/metagenomics/api/v1/analyses/MGYA00022646/file/SRR996970_FASTQ_otu.tsv	Root-Host_associated-Human-Skin
SRR995417	MGYA00024931	https://www.ebi.ac.uk/metagenomics/api/v1/analyses/MGYA00024931/file/SRR995417_FASTQ_otu.tsv	Root-Host_associated-Human-Skin
SRR994953	MGYA00024336	https://www.ebi.ac.uk/metagenomics/api/v1/analyses/MGYA00024336/file/SRR994953_FASTQ_otu.tsv	Root-Host_associated-Human-Skin
SRR994521	MGYA00024051	https://www.ebi.ac.uk/metagenomics/api/v1/analyses/MGYA00024051/file/SRR994521_FASTQ_otu.tsv	Root-Host_associated-Human-Skin

SRR995361	MGYA00022920	https://www.ebi.ac.uk/metagenomics/api/v1/analyses/MGYA00022920/file/SRR995361_FASTQ_otu.tsv	Root-Host_associated-Human-Skin
ERR1701258	MGYA00084268	https://www.ebi.ac.uk/metagenomics/api/v1/analyses/MGYA00084268/file/ERR1701258_FASTQ_otu.tsv	Root-Host_associated-Human-Skin
SRR997055	MGYA00022971	https://www.ebi.ac.uk/metagenomics/api/v1/analyses/MGYA00022971/file/SRR997055_FASTQ_otu.tsv	Root-Host_associated-Human-Skin
SRR1950776	MGYA00376943	https://www.ebi.ac.uk/metagenomics/api/v1/analyses/MGYA00376943/file/SRR1950776_MERGED_FASTQ_SSU_OTU.tsv	Root-Host_associated-Human-Skin
SRR5868662	MGYA00427619	https://www.ebi.ac.uk/metagenomics/api/v1/analyses/MGYA00427619/file/SRR5868662_MERGED_FASTQ_SSU_OTU.tsv	Root-Host_associated-Human-Skin
ERR1759920	MGYA00140454	https://www.ebi.ac.uk/metagenomics/api/v1/analyses/MGYA00140454/file/ERR1759920_MERGED_FASTQ_SSU_OTU.tsv	Root-Host_associated-Human-Skin
SRR994479	MGYA00022244	https://www.ebi.ac.uk/metagenomics/api/v1/analyses/MGYA00022244/file/SRR994479_FASTQ_otu.tsv	Root-Host_associated-Human-Skin
SRR1631666	MGYA00023152	https://www.ebi.ac.uk/metagenomics/api/v1/analyses/MGYA00023152/file/SRR1631666_MERGED_FASTQ_otu.tsv	Root-Host_associated-Human-Skin
ERR2538527	MGYA00381534	https://www.ebi.ac.uk/metagenomics/api/v1/analyses/MGYA00381534/file/ERR2538527_FASTQ_SSU_OTU.tsv	Root-Host_associated-Human-Skin
SRR5869443	MGYA00426642	https://www.ebi.ac.uk/metagenomics/api/v1/analyses/MGYA00426642/file/SRR5869443_MERGED_FASTQ_SSU_OTU.tsv	Root-Host_associated-Human-Skin
SRR1633133	MGYA00024874	https://www.ebi.ac.uk/metagenomics/api/v1/analyses/MGYA00024874/file/SRR1633133_MERGED_FASTQ_otu.tsv	Root-Host_associated-Human-Skin
SRR921075	MGYA00022632	https://www.ebi.ac.uk/metagenomics/api/v1/analyses/MGYA00022632/file/SRR921075_FASTQ_otu.tsv	Root-Host_associated-Human-Skin

ERR442956	MGYA00421443	https://www.ebi.ac.uk/metagenomics/api/v1/analyses/MGYA00421443/file/ERR442956_FASTQ_SSU_OTU.tsv	Root-Host_associated-Human-Skin
ERR1701300	MGYA00084256	https://www.ebi.ac.uk/metagenomics/api/v1/analyses/MGYA00084256/file/ERR1701300_FASTQ_otu.tsv	Root-Host_associated-Human-Skin
SRR920192	MGYA00023265	https://www.ebi.ac.uk/metagenomics/api/v1/analyses/MGYA00023265/file/SRR920192_FASTQ_otu.tsv	Root-Host_associated-Human-Skin
ERR443364	MGYA00426043	https://www.ebi.ac.uk/metagenomics/api/v1/analyses/MGYA00426043/file/ERR443364_FASTQ_SSU_OTU.tsv	Root-Host_associated-Human-Skin
ERR1960672	MGYA00130727	https://www.ebi.ac.uk/metagenomics/api/v1/analyses/MGYA00130727/file/ERR1960672_MERGED_FASTQ_otu.tsv	Root-Host_associated-Human-Skin
ERR451431	MGYA00428709	https://www.ebi.ac.uk/metagenomics/api/v1/analyses/MGYA00428709/file/ERR451431_FASTQ_SSU_OTU.tsv	Root-Host_associated-Human-Skin
ERR442987	MGYA00413713	https://www.ebi.ac.uk/metagenomics/api/v1/analyses/MGYA00413713/file/ERR442987_FASTQ_SSU_OTU.tsv	Root-Host_associated-Human-Skin
ERR451396	MGYA00413345	https://www.ebi.ac.uk/metagenomics/api/v1/analyses/MGYA00413345/file/ERR451396_FASTQ_SSU_OTU.tsv	Root-Host_associated-Human-Skin
SRR919638	MGYA00023761	https://www.ebi.ac.uk/metagenomics/api/v1/analyses/MGYA00023761/file/SRR919638_FASTQ_otu.tsv	Root-Host_associated-Human-Skin
SRR1622925	MGYA00023793	https://www.ebi.ac.uk/metagenomics/api/v1/analyses/MGYA00023793/file/SRR1622925_MERGED_FASTQ_otu.tsv	Root-Host_associated-Human-Skin
SRR1631110	MGYA00022868	https://www.ebi.ac.uk/metagenomics/api/v1/analyses/MGYA00022868/file/SRR1631110_MERGED_FASTQ_otu.tsv	Root-Host_associated-Human-Skin
SRR1923933	MGYA00028258	https://www.ebi.ac.uk/metagenomics/api/v1/analyses/MGYA00028258/file/SRR1923933_FASTQ_otu.tsv	Root-Host_associated-Human-Skin

SRR920950	MGYA00022787	https://www.ebi.ac.uk/metagenomics/api/v1/analyses/MGYA00022787/file/SRR920950_FASTQ_otu.tsv	Root-Host_associated-Human-Skin
SRR5870298	MGYA00426187	https://www.ebi.ac.uk/metagenomics/api/v1/analyses/MGYA00426187/file/SRR5870298_MERGED_FASTQ_SSU_OTU.tsv	Root-Host_associated-Human-Skin
SRR1632464	MGYA00023142	https://www.ebi.ac.uk/metagenomics/api/v1/analyses/MGYA00023142/file/SRR1632464_MERGED_FASTQ_otu.tsv	Root-Host_associated-Human-Skin
SRR1632816	MGYA00022594	https://www.ebi.ac.uk/metagenomics/api/v1/analyses/MGYA00022594/file/SRR1632816_MERGED_FASTQ_otu.tsv	Root-Host_associated-Human-Skin
SRR6757659	MGYA00380773	https://www.ebi.ac.uk/metagenomics/api/v1/analyses/MGYA00380773/file/SRR6757659_MERGED_FASTQ_SSU_OTU.tsv	Root-Host_associated-Human-Skin
SRR996164	MGYA00022287	https://www.ebi.ac.uk/metagenomics/api/v1/analyses/MGYA00022287/file/SRR996164_FASTQ_otu.tsv	Root-Host_associated-Human-Skin
SRR995071	MGYA00024643	https://www.ebi.ac.uk/metagenomics/api/v1/analyses/MGYA00024643/file/SRR995071_FASTQ_otu.tsv	Root-Host_associated-Human-Skin
ERR1760061	MGYA00140502	https://www.ebi.ac.uk/metagenomics/api/v1/analyses/MGYA00140502/file/ERR1760061_MERGED_FASTQ_SSU_OTU.tsv	Root-Host_associated-Human-Skin
SRR994631	MGYA00022520	https://www.ebi.ac.uk/metagenomics/api/v1/analyses/MGYA00022520/file/SRR994631_FASTQ_otu.tsv	Root-Host_associated-Human-Skin
SRR996977	MGYA00022641	https://www.ebi.ac.uk/metagenomics/api/v1/analyses/MGYA00022641/file/SRR996977_FASTQ_otu.tsv	Root-Host_associated-Human-Skin
SRR1631389	MGYA00024092	https://www.ebi.ac.uk/metagenomics/api/v1/analyses/MGYA00024092/file/SRR1631389_MERGED_FASTQ_otu.tsv	Root-Host_associated-Human-Skin
SRR995410	MGYA00024924	https://www.ebi.ac.uk/metagenomics/api/v1/analyses/MGYA00024924/file/SRR995410_FASTQ_otu.tsv	Root-Host_associated-Human-Skin

SRR995366	MGYA00022913	https://www.ebi.ac.uk/metagenomics/api/v1/analyses/MGYA00022913/file/SRR995366_FASTQ_otu.tsv	Root-Host_associated-Human-Skin
SRR1632998	MGYA00022273	https://www.ebi.ac.uk/metagenomics/api/v1/analyses/MGYA00022273/file/SRR1632998_MERGED_FASTQ_otu.tsv	Root-Host_associated-Human-Skin
SRR994526	MGYA00024046	https://www.ebi.ac.uk/metagenomics/api/v1/analyses/MGYA00024046/file/SRR994526_FASTQ_otu.tsv	Root-Host_associated-Human-Skin
SRR994954	MGYA00024341	https://www.ebi.ac.uk/metagenomics/api/v1/analyses/MGYA00024341/file/SRR994954_FASTQ_otu.tsv	Root-Host_associated-Human-Skin
ERR451218	MGYA00415160	https://www.ebi.ac.uk/metagenomics/api/v1/analyses/MGYA00415160/file/ERR451218_FASTQ_SSU_OTU.tsv	Root-Host_associated-Human-Skin
ERR2538478	MGYA00381485	https://www.ebi.ac.uk/metagenomics/api/v1/analyses/MGYA00381485/file/ERR2538478_FASTQ_SSU_OTU.tsv	Root-Host_associated-Human-Skin
SRR997052	MGYA00022964	https://www.ebi.ac.uk/metagenomics/api/v1/analyses/MGYA00022964/file/SRR997052_FASTQ_otu.tsv	Root-Host_associated-Human-Skin
SRR6757688	MGYA00380801	https://www.ebi.ac.uk/metagenomics/api/v1/analyses/MGYA00380801/file/SRR6757688_MERGED_FASTQ_SSU_OTU.tsv	Root-Host_associated-Human-Skin
ERR1759927	MGYA00140421	https://www.ebi.ac.uk/metagenomics/api/v1/analyses/MGYA00140421/file/ERR1759927_MERGED_FASTQ_SSU_OTU.tsv	Root-Host_associated-Human-Skin
SRR1704563	MGYA00414635	https://www.ebi.ac.uk/metagenomics/api/v1/analyses/MGYA00414635/file/SRR1704563_FASTQ_SSU_OTU.tsv	Root-Host_associated-Human-Skin
SRR5868665	MGYA00423070	https://www.ebi.ac.uk/metagenomics/api/v1/analyses/MGYA00423070/file/SRR5868665_MERGED_FASTQ_SSU_OTU.tsv	Root-Host_associated-Human-Skin
SRR1950771	MGYA00376938	https://www.ebi.ac.uk/metagenomics/api/v1/analyses/MGYA00376938/file/SRR1950771_MERGED_FASTQ_SSU_OTU.tsv	Root-Host_associated-Human-Skin

SRR995599	MGYA00025209	https://www.ebi.ac.uk/metagenomics/api/v1/analyses/MGYA00025209/file/SRR995599_FASTQ_otu.tsv	Root-Host_associated-Human-Skin
ERR443702	MGYA00424269	https://www.ebi.ac.uk/metagenomics/api/v1/analyses/MGYA00424269/file/ERR443702_FASTQ_SSU_OTU.tsv	Root-Host_associated-Human-Skin
ERR2538520	MGYA00381527	https://www.ebi.ac.uk/metagenomics/api/v1/analyses/MGYA00381527/file/ERR2538520_FASTQ_SSU_OTU.tsv	Root-Host_associated-Human-Skin
SRR5869444	MGYA00421641	https://www.ebi.ac.uk/metagenomics/api/v1/analyses/MGYA00421641/file/SRR5869444_MERGED_FASTQ_SSU_OTU.tsv	Root-Host_associated-Human-Skin
SRR1631661	MGYA00023146	https://www.ebi.ac.uk/metagenomics/api/v1/analyses/MGYA00023146/file/SRR1631661_MERGED_FASTQ_otu.tsv	Root-Host_associated-Human-Skin
SRR920195	MGYA00023266	https://www.ebi.ac.uk/metagenomics/api/v1/analyses/MGYA00023266/file/SRR920195_FASTQ_otu.tsv	Root-Host_associated-Human-Skin
ERR451340	MGYA00412982	https://www.ebi.ac.uk/metagenomics/api/v1/analyses/MGYA00412982/file/ERR451340_FASTQ_SSU_OTU.tsv	Root-Host_associated-Human-Skin
SRR921072	MGYA00022631	https://www.ebi.ac.uk/metagenomics/api/v1/analyses/MGYA00022631/file/SRR921072_FASTQ_otu.tsv	Root-Host_associated-Human-Skin
SRR1633134	MGYA00024877	https://www.ebi.ac.uk/metagenomics/api/v1/analyses/MGYA00024877/file/SRR1633134_MERGED_FASTQ_otu.tsv	Root-Host_associated-Human-Skin
SRR995129	MGYA00023926	https://www.ebi.ac.uk/metagenomics/api/v1/analyses/MGYA00023926/file/SRR995129_FASTQ_otu.tsv	Root-Host_associated-Human-Skin
ERR1760139	MGYA00140577	https://www.ebi.ac.uk/metagenomics/api/v1/analyses/MGYA00140577/file/ERR1760139_MERGED_FASTQ_SSU_OTU.tsv	Root-Host_associated-Human-Skin
ERR1960675	MGYA00130713	https://www.ebi.ac.uk/metagenomics/api/v1/analyses/MGYA00130713/file/ERR1960675_MERGED_FASTQ_otu.tsv	Root-Host_associated-Human-Skin

ERR443415	MGYA00424313	https://www.ebi.ac.uk/metagenomics/api/v1/analyses/MGYA00424313/file/ERR443415_FASTQ_SSU_OTU.tsv	Root-Host_associated-Human-Skin
SRR1622922	MGYA00023796	https://www.ebi.ac.uk/metagenomics/api/v1/analyses/MGYA00023796/file/SRR1622922_MERGED_FASTQ_otu.tsv	Root-Host_associated-Human-Skin
SRR1632811	MGYA00022593	https://www.ebi.ac.uk/metagenomics/api/v1/analyses/MGYA00022593/file/SRR1632811_MERGED_FASTQ_otu.tsv	Root-Host_associated-Human-Skin
SRR1632463	MGYA00023130	https://www.ebi.ac.uk/metagenomics/api/v1/analyses/MGYA00023130/file/SRR1632463_MERGED_FASTQ_otu.tsv	Root-Host_associated-Human-Skin
SRR920957	MGYA00022784	https://www.ebi.ac.uk/metagenomics/api/v1/analyses/MGYA00022784/file/SRR920957_FASTQ_otu.tsv	Root-Host_associated-Human-Skin
SRR1923934	MGYA00028261	https://www.ebi.ac.uk/metagenomics/api/v1/analyses/MGYA00028261/file/SRR1923934_FASTQ_otu.tsv	Root-Host_associated-Human-Skin
SRR995548	MGYA00022748	https://www.ebi.ac.uk/metagenomics/api/v1/analyses/MGYA00022748/file/SRR995548_FASTQ_otu.tsv	Root-Host_associated-Human-Skin
SRR5869753	MGYA00416969	https://www.ebi.ac.uk/metagenomics/api/v1/analyses/MGYA00416969/file/SRR5869753_MERGED_FASTQ_SSU_OTU.tsv	Root-Host_associated-Human-Skin
ERR450887	MGYA00416707	https://www.ebi.ac.uk/metagenomics/api/v1/analyses/MGYA00416707/file/ERR450887_FASTQ_SSU_OTU.tsv	Root-Host_associated-Human-Skin
ERR1960751	MGYA00130817	https://www.ebi.ac.uk/metagenomics/api/v1/analyses/MGYA00130817/file/ERR1960751_MERGED_FASTQ_otu.tsv	Root-Host_associated-Human-Skin
SRR1924008	MGYA00028238	https://www.ebi.ac.uk/metagenomics/api/v1/analyses/MGYA00028238/file/SRR1924008_FASTQ_otu.tsv	Root-Host_associated-Human-Skin
SRR6757625	MGYA00380742	https://www.ebi.ac.uk/metagenomics/api/v1/analyses/MGYA00380742/file/SRR6757625_MERGED_FASTQ_SSU_OTU.tsv	Root-Host_associated-Human-Skin

SRR996118	MGYA00025179	https://www.ebi.ac.uk/metagenomics/api/v1/analyses/MGYA00025179/file/SRR996118_FASTQ_otu.tsv	Root-Host_associated-Human-Skin
ERR2538372	MGYA00381379	https://www.ebi.ac.uk/metagenomics/api/v1/analyses/MGYA00381379/file/ERR2538372_FASTQ_SSU_OTU.tsv	Root-Host_associated-Human-Skin
ERR443150	MGYA00413694	https://www.ebi.ac.uk/metagenomics/api/v1/analyses/MGYA00413694/file/ERR443150_FASTQ_SSU_OTU.tsv	Root-Host_associated-Human-Skin
ERR451173	MGYA00415164	https://www.ebi.ac.uk/metagenomics/api/v1/analyses/MGYA00415164/file/ERR451173_FASTQ_SSU_OTU.tsv	Root-Host_associated-Human-Skin
SRR2005652	MGYA00381311	https://www.ebi.ac.uk/metagenomics/api/v1/analyses/MGYA00381311/file/SRR2005652_MERGED_FASTQ_SSU_OTU.tsv	Root-Host_associated-Human-Skin
SRR996579	MGYA00022412	https://www.ebi.ac.uk/metagenomics/api/v1/analyses/MGYA00022412/file/SRR996579_FASTQ_otu.tsv	Root-Host_associated-Human-Skin
SRR1920905	MGYA00028384	https://www.ebi.ac.uk/metagenomics/api/v1/analyses/MGYA00028384/file/SRR1920905_FASTQ_otu.tsv	Root-Host_associated-Human-Skin
SRR5868790	MGYA00420774	https://www.ebi.ac.uk/metagenomics/api/v1/analyses/MGYA00420774/file/SRR5868790_MERGED_FASTQ_SSU_OTU.tsv	Root-Host_associated-Human-Skin
SRR1631452	MGYA00025119	https://www.ebi.ac.uk/metagenomics/api/v1/analyses/MGYA00025119/file/SRR1631452_MERGED_FASTQ_otu.tsv	Root-Host_associated-Human-Skin
SRR1704447	MGYA00428850	https://www.ebi.ac.uk/metagenomics/api/v1/analyses/MGYA00428850/file/SRR1704447_FASTQ_SSU_OTU.tsv	Root-Host_associated-Human-Skin
SRR5868741	MGYA00417118	https://www.ebi.ac.uk/metagenomics/api/v1/analyses/MGYA00417118/file/SRR5868741_MERGED_FASTQ_SSU_OTU.tsv	Root-Host_associated-Human-Skin
SRR1631483	MGYA00024935	https://www.ebi.ac.uk/metagenomics/api/v1/analyses/MGYA00024935/file/SRR1631483_MERGED_FASTQ_otu.tsv	Root-Host_associated-Human-Skin

SRR1922554	MGYA00028242	https://www.ebi.ac.uk/metagenomics/api/v1/analyses/MGYA00028242/file/SRR1922554_FASTQ_otu.tsv	Root-Host_associated-Human-Skin
SRR5869912	MGYA00421159	https://www.ebi.ac.uk/metagenomics/api/v1/analyses/MGYA00421159/file/SRR5869912_MERGED_FASTQ_SSU_OTU.tsv	Root-Host_associated-Human-Skin
ERR2538404	MGYA00381411	https://www.ebi.ac.uk/metagenomics/api/v1/analyses/MGYA00381411/file/ERR2538404_FASTQ_SSU_OTU.tsv	Root-Host_associated-Human-Skin
SRR1921020	MGYA00028247	https://www.ebi.ac.uk/metagenomics/api/v1/analyses/MGYA00028247/file/SRR1921020_FASTQ_otu.tsv	Root-Host_associated-Human-Skin
ERR1701223	MGYA00084317	https://www.ebi.ac.uk/metagenomics/api/v1/analyses/MGYA00084317/file/ERR1701223_FASTQ_otu.tsv	Root-Host_associated-Human-Skin
ERR443247	MGYA00420135	https://www.ebi.ac.uk/metagenomics/api/v1/analyses/MGYA00420135/file/ERR443247_FASTQ_SSU_OTU.tsv	Root-Host_associated-Human-Skin
ERR450856	MGYA00416336	https://www.ebi.ac.uk/metagenomics/api/v1/analyses/MGYA00416336/file/ERR450856_FASTQ_SSU_OTU.tsv	Root-Host_associated-Human-Skin
ERR1960780	MGYA00130864	https://www.ebi.ac.uk/metagenomics/api/v1/analyses/MGYA00130864/file/ERR1960780_MERGED_FASTQ_otu.tsv	Root-Host_associated-Human-Skin
SRR1633010	MGYA00023954	https://www.ebi.ac.uk/metagenomics/api/v1/analyses/MGYA00023954/file/SRR1633010_MERGED_FASTQ_otu.tsv	Root-Host_associated-Human-Skin
ERR442036	MGYA00385205	https://www.ebi.ac.uk/metagenomics/api/v1/analyses/MGYA00385205/file/ERR442036_FASTQ_SSU_OTU.tsv	Root-Host_associated-Human-Skin
SRR920588	MGYA00024246	https://www.ebi.ac.uk/metagenomics/api/v1/analyses/MGYA00024246/file/SRR920588_FASTQ_otu.tsv	Root-Host_associated-Human-Skin
SRR994870	MGYA00025265	https://www.ebi.ac.uk/metagenomics/api/v1/analyses/MGYA00025265/file/SRR994870_FASTQ_otu.tsv	Root-Host_associated-Human-Skin

SRR5870232	MGYA00415296	https://www.ebi.ac.uk/metagenomics/api/v1/analyses/MGYA00415296/file/SRR5870232_MERGED_FASTQ_SSU_OTU.tsv	Root-Host_associated-Human-Skin
SRR919554	MGYA00024497	https://www.ebi.ac.uk/metagenomics/api/v1/analyses/MGYA00024497/file/SRR919554_FASTQ_otu.tsv	Root-Host_associated-Human-Skin
SRR919926	MGYA00025195	https://www.ebi.ac.uk/metagenomics/api/v1/analyses/MGYA00025195/file/SRR919926_FASTQ_otu.tsv	Root-Host_associated-Human-Skin
SRR1633148	MGYA00024400	https://www.ebi.ac.uk/metagenomics/api/v1/analyses/MGYA00024400/file/SRR1633148_MERGED_FASTQ_otu.tsv	Root-Host_associated-Human-Skin
SRR995623	MGYA00025219	https://www.ebi.ac.uk/metagenomics/api/v1/analyses/MGYA00025219/file/SRR995623_FASTQ_otu.tsv	Root-Host_associated-Human-Skin
SRR1624863	MGYA00024471	https://www.ebi.ac.uk/metagenomics/api/v1/analyses/MGYA00024471/file/SRR1624863_MERGED_FASTQ_otu.tsv	Root-Host_associated-Human-Skin
SRR5869438	MGYA00415338	https://www.ebi.ac.uk/metagenomics/api/v1/analyses/MGYA00415338/file/SRR5869438_MERGED_FASTQ_SSU_OTU.tsv	Root-Host_associated-Human-Skin
SRR920138	MGYA00023586	https://www.ebi.ac.uk/metagenomics/api/v1/analyses/MGYA00023586/file/SRR920138_FASTQ_otu.tsv	Root-Host_associated-Human-Skin
SRR5870125	MGYA00416416	https://www.ebi.ac.uk/metagenomics/api/v1/analyses/MGYA00416416/file/SRR5870125_MERGED_FASTQ_SSU_OTU.tsv	Root-Host_associated-Human-Skin
SRR994715	MGYA00023645	https://www.ebi.ac.uk/metagenomics/api/v1/analyses/MGYA00023645/file/SRR994715_FASTQ_otu.tsv	Root-Host_associated-Human-Skin
ERR443469	MGYA00424300	https://www.ebi.ac.uk/metagenomics/api/v1/analyses/MGYA00424300/file/ERR443469_FASTQ_SSU_OTU.tsv	Root-Host_associated-Human-Skin
SRR996853	MGYA00023217	https://www.ebi.ac.uk/metagenomics/api/v1/analyses/MGYA00023217/file/SRR996853_FASTQ_otu.tsv	Root-Host_associated-Human-Skin

ERR443008	MGYA00416329	https://www.ebi.ac.uk/metagenomics/api/v1/analyses/MGYA00416329/file/ERR443008_FASTQ_SSU_OTU.tsv	Root-Host_associated-Human-Skin
SRR995946	MGYA00024279	https://www.ebi.ac.uk/metagenomics/api/v1/analyses/MGYA00024279/file/SRR995946_FASTQ_otu.tsv	Root-Host_associated-Human-Skin
SRR995293	MGYA00023593	https://www.ebi.ac.uk/metagenomics/api/v1/analyses/MGYA00023593/file/SRR995293_FASTQ_otu.tsv	Root-Host_associated-Human-Skin
SRR1923948	MGYA00028316	https://www.ebi.ac.uk/metagenomics/api/v1/analyses/MGYA00028316/file/SRR1923948_FASTQ_otu.tsv	Root-Host_associated-Human-Skin
ERR1960723	MGYA00130845	https://www.ebi.ac.uk/metagenomics/api/v1/analyses/MGYA00130845/file/ERR1960723_MERGED_FASTQ_otu.tsv	Root-Host_associated-Human-Skin
ERR1701280	MGYA00084282	https://www.ebi.ac.uk/metagenomics/api/v1/analyses/MGYA00084282/file/ERR1701280_FASTQ_otu.tsv	Root-Host_associated-Human-Skin
ERR451560	MGYA00415520	https://www.ebi.ac.uk/metagenomics/api/v1/analyses/MGYA00415520/file/ERR451560_FASTQ_SSU_OTU.tsv	Root-Host_associated-Human-Skin
SRR1622874	MGYA00024179	https://www.ebi.ac.uk/metagenomics/api/v1/analyses/MGYA00024179/file/SRR1622874_MERGED_FASTQ_otu.tsv	Root-Host_associated-Human-Skin
SRR1631041	MGYA00025008	https://www.ebi.ac.uk/metagenomics/api/v1/analyses/MGYA00025008/file/SRR1631041_MERGED_FASTQ_otu.tsv	Root-Host_associated-Human-Skin
SRR1704722	MGYA00421132	https://www.ebi.ac.uk/metagenomics/api/v1/analyses/MGYA00421132/file/SRR1704722_FASTQ_SSU_OTU.tsv	Root-Host_associated-Human-Skin
SRR5868856	MGYA00427119	https://www.ebi.ac.uk/metagenomics/api/v1/analyses/MGYA00427119/file/SRR5868856_MERGED_FASTQ_SSU_OTU.tsv	Root-Host_associated-Human-Skin
SRR6757657	MGYA00380772	https://www.ebi.ac.uk/metagenomics/api/v1/analyses/MGYA00380772/file/SRR6757657_MERGED_FASTQ_SSU_OTU.tsv	Root-Host_associated-Human-Skin

SRR1923862	MGYA00028319	https://www.ebi.ac.uk/metagenomics/api/v1/analyses/MGYA00028319/file/SRR1923862_FASTQ_otu.tsv	Root-Host_associated-Human-Skin
ERR451101	MGYA00413360	https://www.ebi.ac.uk/metagenomics/api/v1/analyses/MGYA00413360/file/ERR451101_FASTQ_SSU_OTU.tsv	Root-Host_associated-Human-Skin
ERR443685	MGYA00414255	https://www.ebi.ac.uk/metagenomics/api/v1/analyses/MGYA00414255/file/ERR443685_FASTQ_SSU_OTU.tsv	Root-Host_associated-Human-Skin
ERR443122	MGYA00420150	https://www.ebi.ac.uk/metagenomics/api/v1/analyses/MGYA00420150/file/ERR443122_FASTQ_SSU_OTU.tsv	Root-Host_associated-Human-Skin
SRR1623180	MGYA00025170	https://www.ebi.ac.uk/metagenomics/api/v1/analyses/MGYA00025170/file/SRR1623180_MERGED_FASTQ_otu.tsv	Root-Host_associated-Human-Skin
SRR1631387	MGYA00024084	https://www.ebi.ac.uk/metagenomics/api/v1/analyses/MGYA00024084/file/SRR1631387_MERGED_FASTQ_otu.tsv	Root-Host_associated-Human-Skin
SRR996979	MGYA00022639	https://www.ebi.ac.uk/metagenomics/api/v1/analyses/MGYA00022639/file/SRR996979_FASTQ_otu.tsv	Root-Host_associated-Human-Skin
SRR994528	MGYA00024054	https://www.ebi.ac.uk/metagenomics/api/v1/analyses/MGYA00024054/file/SRR994528_FASTQ_otu.tsv	Root-Host_associated-Human-Skin
SRR1632996	MGYA00022265	https://www.ebi.ac.uk/metagenomics/api/v1/analyses/MGYA00022265/file/SRR1632996_MERGED_FASTQ_otu.tsv	Root-Host_associated-Human-Skin
SRR995368	MGYA00022911	https://www.ebi.ac.uk/metagenomics/api/v1/analyses/MGYA00022911/file/SRR995368_FASTQ_otu.tsv	Root-Host_associated-Human-Skin
SRR6757686	MGYA00380799	https://www.ebi.ac.uk/metagenomics/api/v1/analyses/MGYA00380799/file/SRR6757686_MERGED_FASTQ_SSU_OTU.tsv	Root-Host_associated-Human-Skin
ERR2538476	MGYA00381483	https://www.ebi.ac.uk/metagenomics/api/v1/analyses/MGYA00381483/file/ERR2538476_FASTQ_SSU_OTU.tsv	Root-Host_associated-Human-Skin

SRR921124	MGYA00025188	https://www.ebi.ac.uk/metagenomics/api/v1/analyses/MGYA00025188/file/SRR921124_FASTQ_otu.tsv	Root-Host_associated-Human-Skin
ERR451216	MGYA00413683	https://www.ebi.ac.uk/metagenomics/api/v1/analyses/MGYA00413683/file/ERR451216_FASTQ_SSU_OTU.tsv	Root-Host_associated-Human-Skin
ERR1701251	MGYA00084320	https://www.ebi.ac.uk/metagenomics/api/v1/analyses/MGYA00084320/file/ERR1701251_FASTQ_otu.tsv	Root-Host_associated-Human-Skin
SRR994470	MGYA00022251	https://www.ebi.ac.uk/metagenomics/api/v1/analyses/MGYA00022251/file/SRR994470_FASTQ_otu.tsv	Root-Host_associated-Human-Skin
ERR442044	MGYA00385256	https://www.ebi.ac.uk/metagenomics/api/v1/analyses/MGYA00385256/file/ERR442044_FASTQ_SSU_OTU.tsv	Root-Host_associated-Human-Skin
SRR995597	MGYA00025203	https://www.ebi.ac.uk/metagenomics/api/v1/analyses/MGYA00025203/file/SRR995597_FASTQ_otu.tsv	Root-Host_associated-Human-Skin
ERR451088	MGYA00416306	https://www.ebi.ac.uk/metagenomics/api/v1/analyses/MGYA00416306/file/ERR451088_FASTQ_SSU_OTU.tsv	Root-Host_associated-Human-Skin
SRR996325	MGYA00022948	https://www.ebi.ac.uk/metagenomics/api/v1/analyses/MGYA00022948/file/SRR996325_FASTQ_otu.tsv	Root-Host_associated-Human-Skin
ERR1759929	MGYA00140539	https://www.ebi.ac.uk/metagenomics/api/v1/analyses/MGYA00140539/file/ERR1759929_MERGED_FASTQ_SSU_OTU.tsv	Root-Host_associated-Human-Skin
SRR996744	MGYA00023611	https://www.ebi.ac.uk/metagenomics/api/v1/analyses/MGYA00023611/file/SRR996744_FASTQ_otu.tsv	Root-Host_associated-Human-Skin
SRR5869838	MGYA00421172	https://www.ebi.ac.uk/metagenomics/api/v1/analyses/MGYA00421172/file/SRR5869838_MERGED_FASTQ_SSU_OTU.tsv	Root-Host_associated-Human-Skin
ERR441993	MGYA00385227	https://www.ebi.ac.uk/metagenomics/api/v1/analyses/MGYA00385227/file/ERR441993_FASTQ_SSU_OTU.tsv	Root-Host_associated-Human-Skin

SRR919631	MGYA00023764	https://www.ebi.ac.uk/metagenomics/api/v1/analyses/MGYA00023764/file/SRR919631_FASTQ_otu.tsv	Root-Host_associated-Human-Skin
ERR1760137	MGYA00140370	https://www.ebi.ac.uk/metagenomics/api/v1/analyses/MGYA00140370/file/ERR1760137_MERGED_FASTQ_SSU_OTU.tsv	Root-Host_associated-Human-Skin
SRR995127	MGYA00023924	https://www.ebi.ac.uk/metagenomics/api/v1/analyses/MGYA00023924/file/SRR995127_FASTQ_otu.tsv	Root-Host_associated-Human-Skin
SRR995546	MGYA00022744	https://www.ebi.ac.uk/metagenomics/api/v1/analyses/MGYA00022744/file/SRR995546_FASTQ_otu.tsv	Root-Host_associated-Human-Skin
SRR5870291	MGYA00415819	https://www.ebi.ac.uk/metagenomics/api/v1/analyses/MGYA00415819/file/SRR5870291_MERGED_FASTQ_SSU_OTU.tsv	Root-Host_associated-Human-Skin
SRR920959	MGYA00022790	https://www.ebi.ac.uk/metagenomics/api/v1/analyses/MGYA00022790/file/SRR920959_FASTQ_otu.tsv	Root-Host_associated-Human-Skin
SRR996116	MGYA00025177	https://www.ebi.ac.uk/metagenomics/api/v1/analyses/MGYA00025177/file/SRR996116_FASTQ_otu.tsv	Root-Host_associated-Human-Skin
SRR1924006	MGYA00028225	https://www.ebi.ac.uk/metagenomics/api/v1/analyses/MGYA00028225/file/SRR1924006_FASTQ_otu.tsv	Root-Host_associated-Human-Skin
SRR995003	MGYA00024144	https://www.ebi.ac.uk/metagenomics/api/v1/analyses/MGYA00024144/file/SRR995003_FASTQ_otu.tsv	Root-Host_associated-Human-Skin
ERR1760013	MGYA00140575	https://www.ebi.ac.uk/metagenomics/api/v1/analyses/MGYA00140575/file/ERR1760013_MERGED_FASTQ_SSU_OTU.tsv	Root-Host_associated-Human-Skin
SRR1704498	MGYA00428841	https://www.ebi.ac.uk/metagenomics/api/v1/analyses/MGYA00428841/file/SRR1704498_FASTQ_SSU_OTU.tsv	Root-Host_associated-Human-Skin
SRR996905	MGYA00025323	https://www.ebi.ac.uk/metagenomics/api/v1/analyses/MGYA00025323/file/SRR996905_FASTQ_otu.tsv	Root-Host_associated-Human-Skin

SRR996577	MGYA00022418	https://www.ebi.ac.uk/metagenomics/api/v1/analyses/MGYA00022418/file/SRR996577_FASTQ_otu.tsv	Root-Host_associated-Human-Skin
SRR994585	MGYA00022927	https://www.ebi.ac.uk/metagenomics/api/v1/analyses/MGYA00022927/file/SRR994585_FASTQ_otu.tsv	Root-Host_associated-Human-Skin
SRR996201	MGYA00024173	https://www.ebi.ac.uk/metagenomics/api/v1/analyses/MGYA00024173/file/SRR996201_FASTQ_otu.tsv	Root-Host_associated-Human-Skin
SRR994692	MGYA00023877	https://www.ebi.ac.uk/metagenomics/api/v1/analyses/MGYA00023877/file/SRR994692_FASTQ_otu.tsv	Root-Host_associated-Human-Skin
ERR450858	MGYA00423322	https://www.ebi.ac.uk/metagenomics/api/v1/analyses/MGYA00423322/file/ERR450858_FASTQ_SSU_OTU.tsv	Root-Host_associated-Human-Skin
SRR996660	MGYA00024778	https://www.ebi.ac.uk/metagenomics/api/v1/analyses/MGYA00024778/file/SRR996660_FASTQ_otu.tsv	Root-Host_associated-Human-Skin
SRR1950703	MGYA00376870	https://www.ebi.ac.uk/metagenomics/api/v1/analyses/MGYA00376870/file/SRR1950703_MERGED_FASTQ_SSU_OTU.tsv	Root-Host_associated-Human-Skin
SRR919928	MGYA00025191	https://www.ebi.ac.uk/metagenomics/api/v1/analyses/MGYA00025191/file/SRR919928_FASTQ_otu.tsv	Root-Host_associated-Human-Skin
SRR5868617	MGYA00421666	https://www.ebi.ac.uk/metagenomics/api/v1/analyses/MGYA00421666/file/SRR5868617_MERGED_FASTQ_SSU_OTU.tsv	Root-Host_associated-Human-Skin
SRR1631272	MGYA00024731	https://www.ebi.ac.uk/metagenomics/api/v1/analyses/MGYA00024731/file/SRR1631272_MERGED_FASTQ_otu.tsv	Root-Host_associated-Human-Skin
ERR1759955	MGYA00140542	https://www.ebi.ac.uk/metagenomics/api/v1/analyses/MGYA00140542/file/ERR1759955_MERGED_FASTQ_SSU_OTU.tsv	Root-Host_associated-Human-Skin
SRR920586	MGYA00024248	https://www.ebi.ac.uk/metagenomics/api/v1/analyses/MGYA00024248/file/SRR920586_FASTQ_otu.tsv	Root-Host_associated-Human-Skin

SRR920221	MGYA00023290	https://www.ebi.ac.uk/metagenomics/api/v1/analyses/MGYA00023290/file/SRR920221_FASTQ_otu.tsv	Root-Host_associated-Human-Skin
ERR442038	MGYA00385204	https://www.ebi.ac.uk/metagenomics/api/v1/analyses/MGYA00385204/file/ERR442038_FASTQ_SSU_OTU.tsv	Root-Host_associated-Human-Skin
SRR1704170	MGYA00413113	https://www.ebi.ac.uk/metagenomics/api/v1/analyses/MGYA00413113/file/SRR1704170_FASTQ_SSU_OTU.tsv	Root-Host_associated-Human-Skin
SRR1633146	MGYA00024783	https://www.ebi.ac.uk/metagenomics/api/v1/analyses/MGYA00024783/file/SRR1633146_MERGED_FASTQ_otu.tsv	Root-Host_associated-Human-Skin
ERR451332	MGYA00413352	https://www.ebi.ac.uk/metagenomics/api/v1/analyses/MGYA00413352/file/ERR451332_FASTQ_SSU_OTU.tsv	Root-Host_associated-Human-Skin
ERR450900	MGYA00423889	https://www.ebi.ac.uk/metagenomics/api/v1/analyses/MGYA00423889/file/ERR450900_FASTQ_SSU_OTU.tsv	Root-Host_associated-Human-Skin
ERR443311	MGYA00412626	https://www.ebi.ac.uk/metagenomics/api/v1/analyses/MGYA00412626/file/ERR443311_FASTQ_SSU_OTU.tsv	Root-Host_associated-Human-Skin
ERR443467	MGYA00413343	https://www.ebi.ac.uk/metagenomics/api/v1/analyses/MGYA00413343/file/ERR443467_FASTQ_SSU_OTU.tsv	Root-Host_associated-Human-Skin
SRR920136	MGYA00024318	https://www.ebi.ac.uk/metagenomics/api/v1/analyses/MGYA00024318/file/SRR920136_FASTQ_otu.tsv	Root-Host_associated-Human-Skin
SRR920691	MGYA00024841	https://www.ebi.ac.uk/metagenomics/api/v1/analyses/MGYA00024841/file/SRR920691_FASTQ_otu.tsv	Root-Host_associated-Human-Skin
ERR451444	MGYA00416254	https://www.ebi.ac.uk/metagenomics/api/v1/analyses/MGYA00416254/file/ERR451444_FASTQ_SSU_OTU.tsv	Root-Host_associated-Human-Skin
SRR5869340	MGYA00415353	https://www.ebi.ac.uk/metagenomics/api/v1/analyses/MGYA00415353/file/SRR5869340_MERGED_FASTQ_SSU_OTU.tsv	Root-Host_associated-Human-Skin

SRR995948	MGYA00024277	https://www.ebi.ac.uk/metagenomics/api/v1/analyses/MGYA00024277/file/SRR995948_FASTQ_otu.tsv	Root-Host_associated-Human-Skin
ERR451025	MGYA00422376	https://www.ebi.ac.uk/metagenomics/api/v1/analyses/MGYA00422376/file/ERR451025_FASTQ_SSU_OTU.tsv	Root-Host_associated-Human-Skin
SRR1632411	MGYA00023335	https://www.ebi.ac.uk/metagenomics/api/v1/analyses/MGYA00023335/file/SRR1632411_MERGED_FASTQ_otu.tsv	Root-Host_associated-Human-Skin
ERR1759984	MGYA00140448	https://www.ebi.ac.uk/metagenomics/api/v1/analyses/MGYA00140448/file/ERR1759984_MERGED_FASTQ_SSU_OTU.tsv	Root-Host_associated-Human-Skin
SRR1704267	MGYA00428896	https://www.ebi.ac.uk/metagenomics/api/v1/analyses/MGYA00428896/file/SRR1704267_FASTQ_SSU_OTU.tsv	Root-Host_associated-Human-Skin
SRR2005704	MGYA00381336	https://www.ebi.ac.uk/metagenomics/api/v1/analyses/MGYA00381336/file/SRR2005704_MERGED_FASTQ_SSU_OTU.tsv	Root-Host_associated-Human-Skin
SRR996111	MGYA00025172	https://www.ebi.ac.uk/metagenomics/api/v1/analyses/MGYA00025172/file/SRR996111_FASTQ_otu.tsv	Root-Host_associated-Human-Skin
SRR995004	MGYA00024151	https://www.ebi.ac.uk/metagenomics/api/v1/analyses/MGYA00024151/file/SRR995004_FASTQ_otu.tsv	Root-Host_associated-Human-Skin
ERR1760014	MGYA00140558	https://www.ebi.ac.uk/metagenomics/api/v1/analyses/MGYA00140558/file/ERR1760014_MERGED_FASTQ_SSU_OTU.tsv	Root-Host_associated-Human-Skin
ERR1960758	MGYA00130732	https://www.ebi.ac.uk/metagenomics/api/v1/analyses/MGYA00130732/file/ERR1960758_MERGED_FASTQ_otu.tsv	Root-Host_associated-Human-Skin
ERR443538	MGYA00413337	https://www.ebi.ac.uk/metagenomics/api/v1/analyses/MGYA00413337/file/ERR443538_FASTQ_SSU_OTU.tsv	Root-Host_associated-Human-Skin
SRR996570	MGYA00022415	https://www.ebi.ac.uk/metagenomics/api/v1/analyses/MGYA00022415/file/SRR996570_FASTQ_otu.tsv	Root-Host_associated-Human-Skin

SRR994582	MGYA00022926	https://www.ebi.ac.uk/metagenomics/api/v1/analyses/MGYA00022926/file/SRR994582_FASTQ_otu.tsv	Root-Host_associated-Human-Skin
SRR1923819	MGYA00028404	https://www.ebi.ac.uk/metagenomics/api/v1/analyses/MGYA00028404/file/SRR1923819_FASTQ_otu.tsv	Root-Host_associated-Human-Skin
SRR996206	MGYA00024170	https://www.ebi.ac.uk/metagenomics/api/v1/analyses/MGYA00024170/file/SRR996206_FASTQ_otu.tsv	Root-Host_associated-Human-Skin
SRR5869108	MGYA00426238	https://www.ebi.ac.uk/metagenomics/api/v1/analyses/MGYA00426238/file/SRR5869108_MERGED_FASTQ_SSU_OTU.tsv	Root-Host_associated-Human-Skin
ERR1960789	MGYA00130876	https://www.ebi.ac.uk/metagenomics/api/v1/analyses/MGYA00130876/file/ERR1960789_MERGED_FASTQ_otu.tsv	Root-Host_associated-Human-Skin
SRR994695	MGYA00023878	https://www.ebi.ac.uk/metagenomics/api/v1/analyses/MGYA00023878/file/SRR994695_FASTQ_otu.tsv	Root-Host_associated-Human-Skin
SRR1633019	MGYA00023961	https://www.ebi.ac.uk/metagenomics/api/v1/analyses/MGYA00023961/file/SRR1633019_MERGED_FASTQ_otu.tsv	Root-Host_associated-Human-Skin
SRR996667	MGYA00024773	https://www.ebi.ac.uk/metagenomics/api/v1/analyses/MGYA00024773/file/SRR996667_FASTQ_otu.tsv	Root-Host_associated-Human-Skin
ERR1759952	MGYA00140599	https://www.ebi.ac.uk/metagenomics/api/v1/analyses/MGYA00140599/file/ERR1759952_MERGED_FASTQ_SSU_OTU.tsv	Root-Host_associated-Human-Skin
SRR5868610	MGYA00428107	https://www.ebi.ac.uk/metagenomics/api/v1/analyses/MGYA00428107/file/SRR5868610_MERGED_FASTQ_SSU_OTU.tsv	Root-Host_associated-Human-Skin
SRR1631275	MGYA00024736	https://www.ebi.ac.uk/metagenomics/api/v1/analyses/MGYA00024736/file/SRR1631275_MERGED_FASTQ_otu.tsv	Root-Host_associated-Human-Skin
SRR1950704	MGYA00376871	https://www.ebi.ac.uk/metagenomics/api/v1/analyses/MGYA00376871/file/SRR1950704_MERGED_FASTQ_SSU_OTU.tsv	Root-Host_associated-Human-Skin

SRR920226	MGYA00023285	https://www.ebi.ac.uk/metagenomics/api/v1/analyses/MGYA00023285/file/SRR920226_FASTQ_otu.tsv	Root-Host_associated-Human-Skin
SRR994879	MGYA00025272	https://www.ebi.ac.uk/metagenomics/api/v1/analyses/MGYA00025272/file/SRR994879_FASTQ_otu.tsv	Root-Host_associated-Human-Skin
SRR920581	MGYA00024255	https://www.ebi.ac.uk/metagenomics/api/v1/analyses/MGYA00024255/file/SRR920581_FASTQ_otu.tsv	Root-Host_associated-Human-Skin
SRR1633141	MGYA00022607	https://www.ebi.ac.uk/metagenomics/api/v1/analyses/MGYA00022607/file/SRR1633141_MERGED_FASTQ_otu.tsv	Root-Host_associated-Human-Skin
SRR920696	MGYA00024834	https://www.ebi.ac.uk/metagenomics/api/v1/analyses/MGYA00024834/file/SRR920696_FASTQ_otu.tsv	Root-Host_associated-Human-Skin
ERR443460	MGYA00419490	https://www.ebi.ac.uk/metagenomics/api/v1/analyses/MGYA00419490/file/ERR443460_FASTQ_SSU_OTU.tsv	Root-Host_associated-Human-Skin
SRR920131	MGYA00024323	https://www.ebi.ac.uk/metagenomics/api/v1/analyses/MGYA00024323/file/SRR920131_FASTQ_otu.tsv	Root-Host_associated-Human-Skin
SRR996049	MGYA00023065	https://www.ebi.ac.uk/metagenomics/api/v1/analyses/MGYA00023065/file/SRR996049_FASTQ_otu.tsv	Root-Host_associated-Human-Skin
SRR1632416	MGYA00023811	https://www.ebi.ac.uk/metagenomics/api/v1/analyses/MGYA00023811/file/SRR1632416_MERGED_FASTQ_otu.tsv	Root-Host_associated-Human-Skin
SRR1923941	MGYA00028230	https://www.ebi.ac.uk/metagenomics/api/v1/analyses/MGYA00028230/file/SRR1923941_FASTQ_otu.tsv	Root-Host_associated-Human-Skin
SRR5869081	MGYA00418122	https://www.ebi.ac.uk/metagenomics/api/v1/analyses/MGYA00418122/file/SRR5869081_MERGED_FASTQ_SSU_OTU.tsv	Root-Host_associated-Human-Skin
ERR1759983	MGYA00140464	https://www.ebi.ac.uk/metagenomics/api/v1/analyses/MGYA00140464/file/ERR1759983_MERGED_FASTQ_SSU_OTU.tsv	Root-Host_associated-Human-Skin

ERR1760068	MGYA00140383	https://www.ebi.ac.uk/metagenomics/api/v1/analyses/MGYA00140383/file/ERR1760068_MERGED_FASTQ_SSU_OTU.tsv	Root-Host_associated-Human-Skin
ERR443544	MGYA00416252	https://www.ebi.ac.uk/metagenomics/api/v1/analyses/MGYA00416252/file/ERR443544_FASTQ_SSU_OTU.tsv	Root-Host_associated-Human-Skin
ERR1701287	MGYA00084292	https://www.ebi.ac.uk/metagenomics/api/v1/analyses/MGYA00084292/file/ERR1701287_FASTQ_otu.tsv	Root-Host_associated-Human-Skin
SRR994638	MGYA00022497	https://www.ebi.ac.uk/metagenomics/api/v1/analyses/MGYA00022497/file/SRR994638_FASTQ_otu.tsv	Root-Host_associated-Human-Skin
ERR1960724	MGYA00130829	https://www.ebi.ac.uk/metagenomics/api/v1/analyses/MGYA00130829/file/ERR1960724_MERGED_FASTQ_otu.tsv	Root-Host_associated-Human-Skin
SRR1704725	MGYA00426143	https://www.ebi.ac.uk/metagenomics/api/v1/analyses/MGYA00426143/file/SRR1704725_FASTQ_SSU_OTU.tsv	Root-Host_associated-Human-Skin
SRR1631046	MGYA00025013	https://www.ebi.ac.uk/metagenomics/api/v1/analyses/MGYA00025013/file/SRR1631046_MERGED_FASTQ_otu.tsv	Root-Host_associated-Human-Skin
SRR6757650	MGYA00380765	https://www.ebi.ac.uk/metagenomics/api/v1/analyses/MGYA00380765/file/SRR6757650_MERGED_FASTQ_SSU_OTU.tsv	Root-Host_associated-Human-Skin
SRR5868851	MGYA00414883	https://www.ebi.ac.uk/metagenomics/api/v1/analyses/MGYA00414883/file/SRR5868851_MERGED_FASTQ_SSU_OTU.tsv	Root-Host_associated-Human-Skin
SRR1622873	MGYA00024184	https://www.ebi.ac.uk/metagenomics/api/v1/analyses/MGYA00024184/file/SRR1622873_MERGED_FASTQ_otu.tsv	Root-Host_associated-Human-Skin
SRR5869263	MGYA00418765	https://www.ebi.ac.uk/metagenomics/api/v1/analyses/MGYA00418765/file/SRR5869263_MERGED_FASTQ_SSU_OTU.tsv	Root-Host_associated-Human-Skin
ERR451106	MGYA00422030	https://www.ebi.ac.uk/metagenomics/api/v1/analyses/MGYA00422030/file/ERR451106_FASTQ_SSU_OTU.tsv	Root-Host_associated-Human-Skin

SRR995419	MGYA00024923	https://www.ebi.ac.uk/metagenomics/api/v1/analyses/MGYA00024923/file/SRR995419_FASTQ_otu.tsv	Root-Host_associated-Human-Skin
SRR1923865	MGYA00028322	https://www.ebi.ac.uk/metagenomics/api/v1/analyses/MGYA00028322/file/SRR1923865_FASTQ_otu.tsv	Root-Host_associated-Human-Skin
SRR1631380	MGYA00024091	https://www.ebi.ac.uk/metagenomics/api/v1/analyses/MGYA00024091/file/SRR1631380_MERGED_FASTQ_otu.tsv	Root-Host_associated-Human-Skin
SRR1704344	MGYA00419168	https://www.ebi.ac.uk/metagenomics/api/v1/analyses/MGYA00419168/file/SRR1704344_FASTQ_SSU_OTU.tsv	Root-Host_associated-Human-Skin
SRR1623187	MGYA00023677	https://www.ebi.ac.uk/metagenomics/api/v1/analyses/MGYA00023677/file/SRR1623187_MERGED_FASTQ_otu.tsv	Root-Host_associated-Human-Skin
SRR1704432	MGYA00428853	https://www.ebi.ac.uk/metagenomics/api/v1/analyses/MGYA00428853/file/SRR1704432_FASTQ_SSU_OTU.tsv	Root-Host_associated-Human-Skin
ERR443653	MGYA00415515	https://www.ebi.ac.uk/metagenomics/api/v1/analyses/MGYA00415515/file/ERR443653_FASTQ_SSU_OTU.tsv	Root-Host_associated-Human-Skin
SRR1632991	MGYA00022270	https://www.ebi.ac.uk/metagenomics/api/v1/analyses/MGYA00022270/file/SRR1632991_MERGED_FASTQ_otu.tsv	Root-Host_associated-Human-Skin
ERR2538471	MGYA00381478	https://www.ebi.ac.uk/metagenomics/api/v1/analyses/MGYA00381478/file/ERR2538471_FASTQ_SSU_OTU.tsv	Root-Host_associated-Human-Skin
SRR6757681	MGYA00380794	https://www.ebi.ac.uk/metagenomics/api/v1/analyses/MGYA00380794/file/SRR6757681_MERGED_FASTQ_SSU_OTU.tsv	Root-Host_associated-Human-Skin
ERR1701256	MGYA00084278	https://www.ebi.ac.uk/metagenomics/api/v1/analyses/MGYA00084278/file/ERR1701256_FASTQ_otu.tsv	Root-Host_associated-Human-Skin
ERR451211	MGYA00426068	https://www.ebi.ac.uk/metagenomics/api/v1/analyses/MGYA00426068/file/ERR451211_FASTQ_SSU_OTU.tsv	Root-Host_associated-Human-Skin

SRR921123	MGYA00025181	https://www.ebi.ac.uk/metagenomics/api/v1/analyses/MGYA00025181/file/SRR921123_FASTQ_otu.tsv	Root-Host_associated-Human-Skin
SRR995590	MGYA00025206	https://www.ebi.ac.uk/metagenomics/api/v1/analyses/MGYA00025206/file/SRR995590_FASTQ_otu.tsv	Root-Host_associated-Human-Skin
ERR442043	MGYA00385240	https://www.ebi.ac.uk/metagenomics/api/v1/analyses/MGYA00385240/file/ERR442043_FASTQ_SSU_OTU.tsv	Root-Host_associated-Human-Skin
SRR994477	MGYA00022248	https://www.ebi.ac.uk/metagenomics/api/v1/analyses/MGYA00022248/file/SRR994477_FASTQ_otu.tsv	Root-Host_associated-Human-Skin
SRR994805	MGYA00023023	https://www.ebi.ac.uk/metagenomics/api/v1/analyses/MGYA00023023/file/SRR994805_FASTQ_otu.tsv	Root-Host_associated-Human-Skin
SRR1950778	MGYA00376945	https://www.ebi.ac.uk/metagenomics/api/v1/analyses/MGYA00376945/file/SRR1950778_MERGED_FASTQ_SSU_OTU.tsv	Root-Host_associated-Human-Skin
SRR996322	MGYA00022943	https://www.ebi.ac.uk/metagenomics/api/v1/analyses/MGYA00022943/file/SRR996322_FASTQ_otu.tsv	Root-Host_associated-Human-Skin
SRR5870181	MGYA00426201	https://www.ebi.ac.uk/metagenomics/api/v1/analyses/MGYA00426201/file/SRR5870181_MERGED_FASTQ_SSU_OTU.tsv	Root-Host_associated-Human-Skin
ERR451349	MGYA00426026	https://www.ebi.ac.uk/metagenomics/api/v1/analyses/MGYA00426026/file/ERR451349_FASTQ_SSU_OTU.tsv	Root-Host_associated-Human-Skin
SRR996743	MGYA00023616	https://www.ebi.ac.uk/metagenomics/api/v1/analyses/MGYA00023616/file/SRR996743_FASTQ_otu.tsv	Root-Host_associated-Human-Skin
SRR1631668	MGYA00023154	https://www.ebi.ac.uk/metagenomics/api/v1/analyses/MGYA00023154/file/SRR1631668_MERGED_FASTQ_otu.tsv	Root-Host_associated-Human-Skin
ERR441994	MGYA00385167	https://www.ebi.ac.uk/metagenomics/api/v1/analyses/MGYA00385167/file/ERR441994_FASTQ_SSU_OTU.tsv	Root-Host_associated-Human-Skin

SRR919636	MGYA00023769	https://www.ebi.ac.uk/metagenomics/api/v1/analyses/MGYA00023769/file/SRR919636_FASTQ_otu.tsv	Root-Host_associated-Human-Skin
ERR1760130	MGYA00140601	https://www.ebi.ac.uk/metagenomics/api/v1/analyses/MGYA00140601/file/ERR1760130_MERGED_FASTQ_SSU_OTU.tsv	Root-Host_associated-Human-Skin
SRR995120	MGYA00023917	https://www.ebi.ac.uk/metagenomics/api/v1/analyses/MGYA00023917/file/SRR995120_FASTQ_otu.tsv	Root-Host_associated-Human-Skin
ERR442989	MGYA00422381	https://www.ebi.ac.uk/metagenomics/api/v1/analyses/MGYA00422381/file/ERR442989_FASTQ_SSU_OTU.tsv	Root-Host_associated-Human-Skin
SRR5870150	MGYA00415833	https://www.ebi.ac.uk/metagenomics/api/v1/analyses/MGYA00415833/file/SRR5870150_MERGED_FASTQ_SSU_OTU.tsv	Root-Host_associated-Human-Skin
SRR1632818	MGYA00022598	https://www.ebi.ac.uk/metagenomics/api/v1/analyses/MGYA00022598/file/SRR1632818_MERGED_FASTQ_otu.tsv	Root-Host_associated-Human-Skin
SRR995541	MGYA00022743	https://www.ebi.ac.uk/metagenomics/api/v1/analyses/MGYA00022743/file/SRR995541_FASTQ_otu.tsv	Root-Host_associated-Human-Skin
ERR2538438	MGYA00381445	https://www.ebi.ac.uk/metagenomics/api/v1/analyses/MGYA00381445/file/ERR2538438_FASTQ_SSU_OTU.tsv	Root-Host_associated-Human-Skin
SRR997012	MGYA00023414	https://www.ebi.ac.uk/metagenomics/api/v1/analyses/MGYA00023414/file/SRR997012_FASTQ_otu.tsv	Root-Host_associated-Human-Skin
SRR995326	MGYA00022479	https://www.ebi.ac.uk/metagenomics/api/v1/analyses/MGYA00022479/file/SRR995326_FASTQ_otu.tsv	Root-Host_associated-Human-Skin
SRR1922568	MGYA00028314	https://www.ebi.ac.uk/metagenomics/api/v1/analyses/MGYA00028314/file/SRR1922568_FASTQ_otu.tsv	Root-Host_associated-Human-Skin
ERR1960808	MGYA00130862	https://www.ebi.ac.uk/metagenomics/api/v1/analyses/MGYA00130862/file/ERR1960808_MERGED_FASTQ_otu.tsv	Root-Host_associated-Human-Skin

SRR994914	MGYA00024746	https://www.ebi.ac.uk/metagenomics/api/v1/analyses/MGYA00024746/file/SRR994914_FASTQ_otu.tsv	Root-Host_associated-Human-Skin
SRR994566	MGYA00024435	https://www.ebi.ac.uk/metagenomics/api/v1/analyses/MGYA00024435/file/SRR994566_FASTQ_otu.tsv	Root-Host_associated-Human-Skin
SRR996594	MGYA00023989	https://www.ebi.ac.uk/metagenomics/api/v1/analyses/MGYA00023989/file/SRR996594_FASTQ_otu.tsv	Root-Host_associated-Human-Skin
SRR996937	MGYA00022227	https://www.ebi.ac.uk/metagenomics/api/v1/analyses/MGYA00022227/file/SRR996937_FASTQ_otu.tsv	Root-Host_associated-Human-Skin
SRR1924034	MGYA00028358	https://www.ebi.ac.uk/metagenomics/api/v1/analyses/MGYA00028358/file/SRR1924034_FASTQ_otu.tsv	Root-Host_associated-Human-Skin
SRR6757619	MGYA00380736	https://www.ebi.ac.uk/metagenomics/api/v1/analyses/MGYA00380736/file/SRR6757619_MERGED_FASTQ_SSU_OTU.tsv	Root-Host_associated-Human-Skin
SRR996124	MGYA00024892	https://www.ebi.ac.uk/metagenomics/api/v1/analyses/MGYA00024892/file/SRR996124_FASTQ_otu.tsv	Root-Host_associated-Human-Skin
SRR1923798	MGYA00028392	https://www.ebi.ac.uk/metagenomics/api/v1/analyses/MGYA00028392/file/SRR1923798_FASTQ_otu.tsv	Root-Host_associated-Human-Skin
ERR1760021	MGYA00140504	https://www.ebi.ac.uk/metagenomics/api/v1/analyses/MGYA00140504/file/ERR1760021_MERGED_FASTQ_SSU_OTU.tsv	Root-Host_associated-Human-Skin
SRR5870041	MGYA00420674	https://www.ebi.ac.uk/metagenomics/api/v1/analyses/MGYA00420674/file/SRR5870041_MERGED_FASTQ_SSU_OTU.tsv	Root-Host_associated-Human-Skin
SRR994671	MGYA00023638	https://www.ebi.ac.uk/metagenomics/api/v1/analyses/MGYA00023638/file/SRR994671_FASTQ_otu.tsv	Root-Host_associated-Human-Skin
SRR920917	MGYA00022360	https://www.ebi.ac.uk/metagenomics/api/v1/analyses/MGYA00022360/file/SRR920917_FASTQ_otu.tsv	Root-Host_associated-Human-Skin

ERR443034	MGYA00423309	https://www.ebi.ac.uk/metagenomics/api/v1/analyses/MGYA00423309/file/ERR443034_FASTQ_SSU_OTU.tsv	Root-Host_associated-Human-Skin
SRR1632384	MGYA00022376	https://www.ebi.ac.uk/metagenomics/api/v1/analyses/MGYA00022376/file/SRR1632384_MERGED_FASTQ_otu.tsv	Root-Host_associated-Human-Skin
SRR1632423	MGYA00023755	https://www.ebi.ac.uk/metagenomics/api/v1/analyses/MGYA00023755/file/SRR1632423_MERGED_FASTQ_otu.tsv	Root-Host_associated-Human-Skin
SRR1632851	MGYA00023053	https://www.ebi.ac.uk/metagenomics/api/v1/analyses/MGYA00023053/file/SRR1632851_MERGED_FASTQ_otu.tsv	Root-Host_associated-Human-Skin
ERR443793	MGYA00412585	https://www.ebi.ac.uk/metagenomics/api/v1/analyses/MGYA00412585/file/ERR443793_FASTQ_SSU_OTU.tsv	Root-Host_associated-Human-Skin
SRR995508	MGYA00023168	https://www.ebi.ac.uk/metagenomics/api/v1/analyses/MGYA00023168/file/SRR995508_FASTQ_otu.tsv	Root-Host_associated-Human-Skin
SRR1631536	MGYA00023580	https://www.ebi.ac.uk/metagenomics/api/v1/analyses/MGYA00023580/file/SRR1631536_MERGED_FASTQ_otu.tsv	Root-Host_associated-Human-Skin
SRR995169	MGYA00024322	https://www.ebi.ac.uk/metagenomics/api/v1/analyses/MGYA00024322/file/SRR995169_FASTQ_otu.tsv	Root-Host_associated-Human-Skin
ERR451476	MGYA00415125	https://www.ebi.ac.uk/metagenomics/api/v1/analyses/MGYA00415125/file/ERR451476_FASTQ_SSU_OTU.tsv	Root-Host_associated-Human-Skin
SRR5870119	MGYA00426209	https://www.ebi.ac.uk/metagenomics/api/v1/analyses/MGYA00426209/file/SRR5870119_MERGED_FASTQ_SSU_OTU.tsv	Root-Host_associated-Human-Skin
ERR443455	MGYA00421406	https://www.ebi.ac.uk/metagenomics/api/v1/analyses/MGYA00421406/file/ERR443455_FASTQ_SSU_OTU.tsv	Root-Host_associated-Human-Skin
ERR1960635	MGYA00130819	https://www.ebi.ac.uk/metagenomics/api/v1/analyses/MGYA00130819/file/ERR1960635_MERGED_FASTQ_otu.tsv	Root-Host_associated-Human-Skin

SRR5868940	MGYA00411930	https://www.ebi.ac.uk/metagenomics/api/v1/analyses/MGYA00411930/file/SRR5868940_MERGED_FASTQ_SSU_OTU.tsv	Root-Host_associated-Human-Skin
SRR2005421	MGYA00381291	https://www.ebi.ac.uk/metagenomics/api/v1/analyses/MGYA00381291/file/SRR2005421_MERGED_FASTQ_SSU_OTU.tsv	Root-Host_associated-Human-Skin
ERR443484	MGYA00419481	https://www.ebi.ac.uk/metagenomics/api/v1/analyses/MGYA00419481/file/ERR443484_FASTQ_SSU_OTU.tsv	Root-Host_associated-Human-Skin
SRR1633174	MGYA00025258	https://www.ebi.ac.uk/metagenomics/api/v1/analyses/MGYA00025258/file/SRR1633174_MERGED_FASTQ_otu.tsv	Root-Host_associated-Human-Skin
SRR921032	MGYA00023085	https://www.ebi.ac.uk/metagenomics/api/v1/analyses/MGYA00023085/file/SRR921032_FASTQ_otu.tsv	Root-Host_associated-Human-Skin
SRR5868625	MGYA00428092	https://www.ebi.ac.uk/metagenomics/api/v1/analyses/MGYA00428092/file/SRR5868625_MERGED_FASTQ_SSU_OTU.tsv	Root-Host_associated-Human-Skin
SRR1704284	MGYA00426983	https://www.ebi.ac.uk/metagenomics/api/v1/analyses/MGYA00426983/file/SRR1704284_FASTQ_SSU_OTU.tsv	Root-Host_associated-Human-Skin
ERR1759967	MGYA00140503	https://www.ebi.ac.uk/metagenomics/api/v1/analyses/MGYA00140503/file/ERR1759967_MERGED_FASTQ_SSU_OTU.tsv	Root-Host_associated-Human-Skin
SRR1623047	MGYA00024637	https://www.ebi.ac.uk/metagenomics/api/v1/analyses/MGYA00024637/file/SRR1623047_MERGED_FASTQ_otu.tsv	Root-Host_associated-Human-Skin
SRR1950731	MGYA00376898	https://www.ebi.ac.uk/metagenomics/api/v1/analyses/MGYA00376898/file/SRR1950731_MERGED_FASTQ_SSU_OTU.tsv	Root-Host_associated-Human-Skin
ERR450686	MGYA00425291	https://www.ebi.ac.uk/metagenomics/api/v1/analyses/MGYA00425291/file/ERR450686_FASTQ_SSU_OTU.tsv	Root-Host_associated-Human-Skin
ERR2538444	MGYA00381451	https://www.ebi.ac.uk/metagenomics/api/v1/analyses/MGYA00381451/file/ERR2538444_FASTQ_SSU_OTU.tsv	Root-Host_associated-Human-Skin

SRR996189	MGYA00022962	https://www.ebi.ac.uk/metagenomics/api/v1/analyses/MGYA00022962/file/SRR996189_FASTQ_otu.tsv	Root-Host_associated-Human-Skin
SRR1622897	MGYA00024768	https://www.ebi.ac.uk/metagenomics/api/v1/analyses/MGYA00024768/file/SRR1622897_MERGED_FASTQ_otu.tsv	Root-Host_associated-Human-Skin
ERR981249	MGYA00010240	https://www.ebi.ac.uk/metagenomics/api/v1/analyses/MGYA00010240/file/ERR981249_MERGED_FASTQ_otu.tsv	Root-Host_associated-Human-Skin
ERR1701263	MGYA00084253	https://www.ebi.ac.uk/metagenomics/api/v1/analyses/MGYA00084253/file/ERR1701263_FASTQ_otu.tsv	Root-Host_associated-Human-Skin
SRR1623163	MGYA00023486	https://www.ebi.ac.uk/metagenomics/api/v1/analyses/MGYA00023486/file/SRR1623163_MERGED_FASTQ_otu.tsv	Root-Host_associated-Human-Skin
SRR1631364	MGYA00022587	https://www.ebi.ac.uk/metagenomics/api/v1/analyses/MGYA00022587/file/SRR1631364_MERGED_FASTQ_otu.tsv	Root-Host_associated-Human-Skin
SRR1704407	MGYA00413084	https://www.ebi.ac.uk/metagenomics/api/v1/analyses/MGYA00413084/file/SRR1704407_FASTQ_SSU_OTU.tsv	Root-Host_associated-Human-Skin
SRR1630956	MGYA00024163	https://www.ebi.ac.uk/metagenomics/api/v1/analyses/MGYA00024163/file/SRR1630956_MERGED_FASTQ_otu.tsv	Root-Host_associated-Human-Skin
SRR1923881	MGYA00028342	https://www.ebi.ac.uk/metagenomics/api/v1/analyses/MGYA00028342/file/SRR1923881_FASTQ_otu.tsv	Root-Host_associated-Human-Skin
ERR443666	MGYA00422020	https://www.ebi.ac.uk/metagenomics/api/v1/analyses/MGYA00422020/file/ERR443666_FASTQ_SSU_OTU.tsv	Root-Host_associated-Human-Skin
ERR443110	MGYA00423306	https://www.ebi.ac.uk/metagenomics/api/v1/analyses/MGYA00423306/file/ERR443110_FASTQ_SSU_OTU.tsv	Root-Host_associated-Human-Skin
SRR5869637	MGYA00417004	https://www.ebi.ac.uk/metagenomics/api/v1/analyses/MGYA00417004/file/SRR5869637_MERGED_FASTQ_SSU_OTU.tsv	Root-Host_associated-Human-Skin

SRR996539	MGYA00022846	https://www.ebi.ac.uk/metagenomics/api/v1/analyses/MGYA00022846/file/SRR996539_FASTQ_otu.tsv	Root-Host_associated-Human-Skin
SRR5869190	MGYA00418084	https://www.ebi.ac.uk/metagenomics/api/v1/analyses/MGYA00418084/file/SRR5869190_MERGED_FASTQ_SSU_OTU.tsv	Root-Host_associated-Human-Skin
ERR1759892	MGYA00140516	https://www.ebi.ac.uk/metagenomics/api/v1/analyses/MGYA00140516/file/ERR1759892_MERGED_FASTQ_SSU_OTU.tsv	Root-Host_associated-Human-Skin
ERR1960711	MGYA00130824	https://www.ebi.ac.uk/metagenomics/api/v1/analyses/MGYA00130824/file/ERR1960711_MERGED_FASTQ_otu.tsv	Root-Host_associated-Human-Skin
SRR6757665	MGYA00380778	https://www.ebi.ac.uk/metagenomics/api/v1/analyses/MGYA00380778/file/SRR6757665_MERGED_FASTQ_SSU_OTU.tsv	Root-Host_associated-Human-Skin
SRR5868864	MGYA00416508	https://www.ebi.ac.uk/metagenomics/api/v1/analyses/MGYA00416508/file/SRR5868864_MERGED_FASTQ_SSU_OTU.tsv	Root-Host_associated-Human-Skin
SRR1704710	MGYA00428442	https://www.ebi.ac.uk/metagenomics/api/v1/analyses/MGYA00428442/file/SRR1704710_FASTQ_SSU_OTU.tsv	Root-Host_associated-Human-Skin
SRR5869983	MGYA00420687	https://www.ebi.ac.uk/metagenomics/api/v1/analyses/MGYA00420687/file/SRR5869983_MERGED_FASTQ_SSU_OTU.tsv	Root-Host_associated-Human-Skin
ERR2538495	MGYA00381502	https://www.ebi.ac.uk/metagenomics/api/v1/analyses/MGYA00381502/file/ERR2538495_FASTQ_SSU_OTU.tsv	Root-Host_associated-Human-Skin
SRR996813	MGYA00023632	https://www.ebi.ac.uk/metagenomics/api/v1/analyses/MGYA00023632/file/SRR996813_FASTQ_otu.tsv	Root-Host_associated-Human-Skin
SRR1923908	MGYA00028386	https://www.ebi.ac.uk/metagenomics/api/v1/analyses/MGYA00028386/file/SRR1923908_FASTQ_otu.tsv	Root-Host_associated-Human-Skin
SRR995906	MGYA00023840	https://www.ebi.ac.uk/metagenomics/api/v1/analyses/MGYA00023840/file/SRR995906_FASTQ_otu.tsv	Root-Host_associated-Human-Skin

SRR1704648	MGYA00421466	https://www.ebi.ac.uk/metagenomics/api/v1/analyses/MGYA00421466/file/SRR1704648_FASTQ_SSU_OTU.tsv	Root-Host_associated-Human-Skin
SRR996000	MGYA00023519	https://www.ebi.ac.uk/metagenomics/api/v1/analyses/MGYA00023519/file/SRR996000_FASTQ_otu.tsv	Root-Host_associated-Human-Skin
ERR1760105	MGYA00140457	https://www.ebi.ac.uk/metagenomics/api/v1/analyses/MGYA00140457/file/ERR1760105_MERGED_FASTQ_SSU_OTU.tsv	Root-Host_associated-Human-Skin
ERR1960649	MGYA00130908	https://www.ebi.ac.uk/metagenomics/api/v1/analyses/MGYA00130908/file/ERR1960649_MERGED_FASTQ_otu.tsv	Root-Host_associated-Human-Skin
SRR994755	MGYA00023246	https://www.ebi.ac.uk/metagenomics/api/v1/analyses/MGYA00023246/file/SRR994755_FASTQ_otu.tsv	Root-Host_associated-Human-Skin
SRR5870165	MGYA00426203	https://www.ebi.ac.uk/metagenomics/api/v1/analyses/MGYA00426203/file/SRR5870165_MERGED_FASTQ_SSU_OTU.tsv	Root-Host_associated-Human-Skin
SRR920178	MGYA00024717	https://www.ebi.ac.uk/metagenomics/api/v1/analyses/MGYA00024717/file/SRR920178_FASTQ_otu.tsv	Root-Host_associated-Human-Skin
ERR443429	MGYA00416274	https://www.ebi.ac.uk/metagenomics/api/v1/analyses/MGYA00416274/file/ERR443429_FASTQ_SSU_OTU.tsv	Root-Host_associated-Human-Skin
SRR1632799	MGYA00023212	https://www.ebi.ac.uk/metagenomics/api/v1/analyses/MGYA00023212/file/SRR1632799_MERGED_FASTQ_otu.tsv	Root-Host_associated-Human-Skin
ERR1960698	MGYA00130892	https://www.ebi.ac.uk/metagenomics/api/v1/analyses/MGYA00130892/file/ERR1960698_MERGED_FASTQ_otu.tsv	Root-Host_associated-Human-Skin
SRR1624823	MGYA00024886	https://www.ebi.ac.uk/metagenomics/api/v1/analyses/MGYA00024886/file/SRR1624823_MERGED_FASTQ_otu.tsv	Root-Host_associated-Human-Skin
SRR995663	MGYA00024846	https://www.ebi.ac.uk/metagenomics/api/v1/analyses/MGYA00024846/file/SRR995663_FASTQ_otu.tsv	Root-Host_associated-Human-Skin

ERR441970	MGYA00385189	https://www.ebi.ac.uk/metagenomics/api/v1/analyses/MGYA00385189/file/ERR441970_FASTQ_SSU_OTU.tsv	Root-Host_associated-Human-Skin
SRR5869478	MGYA00419664	https://www.ebi.ac.uk/metagenomics/api/v1/analyses/MGYA00419664/file/SRR5869478_MERGED_FASTQ_SSU_OTU.tsv	Root-Host_associated-Human-Skin
SRR1632329	MGYA00024024	https://www.ebi.ac.uk/metagenomics/api/v1/analyses/MGYA00024024/file/SRR1632329_MERGED_FASTQ_otu.tsv	Root-Host_associated-Human-Skin
ERR2538443	MGYA00381450	https://www.ebi.ac.uk/metagenomics/api/v1/analyses/MGYA00381450/file/ERR2538443_FASTQ_SSU_OTU.tsv	Root-Host_associated-Human-Skin
SRR1622890	MGYA00024767	https://www.ebi.ac.uk/metagenomics/api/v1/analyses/MGYA00024767/file/SRR1622890_MERGED_FASTQ_otu.tsv	Root-Host_associated-Human-Skin
SRR5869955	MGYA00429043	https://www.ebi.ac.uk/metagenomics/api/v1/analyses/MGYA00429043/file/SRR5869955_MERGED_FASTQ_SSU_OTU.tsv	Root-Host_associated-Human-Skin
ERR1701264	MGYA00084246	https://www.ebi.ac.uk/metagenomics/api/v1/analyses/MGYA00084246/file/ERR1701264_FASTQ_otu.tsv	Root-Host_associated-Human-Skin
ERR443200	MGYA00411909	https://www.ebi.ac.uk/metagenomics/api/v1/analyses/MGYA00411909/file/ERR443200_FASTQ_SSU_OTU.tsv	Root-Host_associated-Human-Skin
ERR450811	MGYA00426831	https://www.ebi.ac.uk/metagenomics/api/v1/analyses/MGYA00426831/file/ERR450811_FASTQ_SSU_OTU.tsv	Root-Host_associated-Human-Skin
SRR1630951	MGYA00024164	https://www.ebi.ac.uk/metagenomics/api/v1/analyses/MGYA00024164/file/SRR1630951_MERGED_FASTQ_otu.tsv	Root-Host_associated-Human-Skin
SRR996248	MGYA00023702	https://www.ebi.ac.uk/metagenomics/api/v1/analyses/MGYA00023702/file/SRR996248_FASTQ_otu.tsv	Root-Host_associated-Human-Skin
SRR1631363	MGYA00022584	https://www.ebi.ac.uk/metagenomics/api/v1/analyses/MGYA00022584/file/SRR1631363_MERGED_FASTQ_otu.tsv	Root-Host_associated-Human-Skin

SRR5868706	MGYA00417583	https://www.ebi.ac.uk/metagenomics/api/v1/analyses/MGYA00417583/file/SRR5868706_MERGED_FASTQ_SSU_OTU.tsv	Root-Host_associated-Human-Skin
SRR1623164	MGYA00023487	https://www.ebi.ac.uk/metagenomics/api/v1/analyses/MGYA00023487/file/SRR1623164_MERGED_FASTQ_otu.tsv	Root-Host_associated-Human-Skin
SRR1923886	MGYA00028343	https://www.ebi.ac.uk/metagenomics/api/v1/analyses/MGYA00028343/file/SRR1923886_FASTQ_otu.tsv	Root-Host_associated-Human-Skin
SRR1923857	MGYA00028291	https://www.ebi.ac.uk/metagenomics/api/v1/analyses/MGYA00028291/file/SRR1923857_FASTQ_otu.tsv	Root-Host_associated-Human-Skin
ERR443117	MGYA00413363	https://www.ebi.ac.uk/metagenomics/api/v1/analyses/MGYA00413363/file/ERR443117_FASTQ_SSU_OTU.tsv	Root-Host_associated-Human-Skin
ERR1759895	MGYA00140597	https://www.ebi.ac.uk/metagenomics/api/v1/analyses/MGYA00140597/file/ERR1759895_MERGED_FASTQ_SSU_OTU.tsv	Root-Host_associated-Human-Skin
ERR443576	MGYA00428678	https://www.ebi.ac.uk/metagenomics/api/v1/analyses/MGYA00428678/file/ERR443576_FASTQ_SSU_OTU.tsv	Root-Host_associated-Human-Skin
ERR1960716	MGYA00130822	https://www.ebi.ac.uk/metagenomics/api/v1/analyses/MGYA00130822/file/ERR1960716_MERGED_FASTQ_otu.tsv	Root-Host_associated-Human-Skin
ERR2538492	MGYA00381499	https://www.ebi.ac.uk/metagenomics/api/v1/analyses/MGYA00381499/file/ERR2538492_FASTQ_SSU_OTU.tsv	Root-Host_associated-Human-Skin
SRR5869251	MGYA00422539	https://www.ebi.ac.uk/metagenomics/api/v1/analyses/MGYA00422539/file/SRR5869251_MERGED_FASTQ_SSU_OTU.tsv	Root-Host_associated-Human-Skin
SRR6757662	MGYA00380818	https://www.ebi.ac.uk/metagenomics/api/v1/analyses/MGYA00380818/file/SRR6757662_MERGED_FASTQ_SSU_OTU.tsv	Root-Host_associated-Human-Skin
SRR1704717	MGYA00415650	https://www.ebi.ac.uk/metagenomics/api/v1/analyses/MGYA00415650/file/SRR1704717_FASTQ_SSU_OTU.tsv	Root-Host_associated-Human-Skin

SRR996814	MGYA00023627	https://www.ebi.ac.uk/metagenomics/api/v1/analyses/MGYA00023627/file/SRR996814_FASTQ_otu.tsv	Root-Host_associated-Human-Skin
SRR5869768	MGYA00415885	https://www.ebi.ac.uk/metagenomics/api/v1/analyses/MGYA00415885/file/SRR5869768_MERGED_FASTQ_SSU_OTU.tsv	Root-Host_associated-Human-Skin
SRR995901	MGYA00023839	https://www.ebi.ac.uk/metagenomics/api/v1/analyses/MGYA00023839/file/SRR995901_FASTQ_otu.tsv	Root-Host_associated-Human-Skin
SRR1622919	MGYA00025091	https://www.ebi.ac.uk/metagenomics/api/v1/analyses/MGYA00025091/file/SRR1622919_MERGED_FASTQ_otu.tsv	Root-Host_associated-Human-Skin
SRR996007	MGYA00023514	https://www.ebi.ac.uk/metagenomics/api/v1/analyses/MGYA00023514/file/SRR996007_FASTQ_otu.tsv	Root-Host_associated-Human-Skin
SRR5869309	MGYA00418753	https://www.ebi.ac.uk/metagenomics/api/v1/analyses/MGYA00418753/file/SRR5869309_MERGED_FASTQ_SSU_OTU.tsv	Root-Host_associated-Human-Skin
SRR994752	MGYA00023243	https://www.ebi.ac.uk/metagenomics/api/v1/analyses/MGYA00023243/file/SRR994752_FASTQ_otu.tsv	Root-Host_associated-Human-Skin
ERR443389	MGYA00424889	https://www.ebi.ac.uk/metagenomics/api/v1/analyses/MGYA00424889/file/ERR443389_FASTQ_SSU_OTU.tsv	Root-Host_associated-Human-Skin
ERR1760102	MGYA00140566	https://www.ebi.ac.uk/metagenomics/api/v1/analyses/MGYA00140566/file/ERR1760102_MERGED_FASTQ_SSU_OTU.tsv	Root-Host_associated-Human-Skin
SRR921098	MGYA00022422	https://www.ebi.ac.uk/metagenomics/api/v1/analyses/MGYA00022422/file/SRR921098_FASTQ_otu.tsv	Root-Host_associated-Human-Skin
SRR995664	MGYA00024845	https://www.ebi.ac.uk/metagenomics/api/v1/analyses/MGYA00024845/file/SRR995664_FASTQ_otu.tsv	Root-Host_associated-Human-Skin
SRR1624824	MGYA00024887	https://www.ebi.ac.uk/metagenomics/api/v1/analyses/MGYA00024887/file/SRR1624824_MERGED_FASTQ_otu.tsv	Root-Host_associated-Human-Skin

SRR920609	MGYA00022558	https://www.ebi.ac.uk/metagenomics/api/v1/analyses/MGYA00022558/file/SRR920609_FASTQ_otu.tsv	Root-Host_associated-Human-Skin
SRR5868598	MGYA00418806	https://www.ebi.ac.uk/metagenomics/api/v1/analyses/MGYA00418806/file/SRR5868598_MERGED_FASTQ_SSU_OTU.tsv	Root-Host_associated-Human-Skin
ERR441977	MGYA00385173	https://www.ebi.ac.uk/metagenomics/api/v1/analyses/MGYA00385173/file/ERR441977_FASTQ_SSU_OTU.tsv	Root-Host_associated-Human-Skin
SRR1632489	MGYA00022682	https://www.ebi.ac.uk/metagenomics/api/v1/analyses/MGYA00022682/file/SRR1632489_MERGED_FASTQ_otu.tsv	Root-Host_associated-Human-Skin
SRR1704558	MGYA00426923	https://www.ebi.ac.uk/metagenomics/api/v1/analyses/MGYA00426923/file/SRR1704558_FASTQ_SSU_OTU.tsv	Root-Host_associated-Human-Skin
ERR1701218	MGYA00084245	https://www.ebi.ac.uk/metagenomics/api/v1/analyses/MGYA00084245/file/ERR1701218_FASTQ_otu.tsv	Root-Host_associated-Human-Skin
SRR997015	MGYA00023413	https://www.ebi.ac.uk/metagenomics/api/v1/analyses/MGYA00023413/file/SRR997015_FASTQ_otu.tsv	Root-Host_associated-Human-Skin
ERR2538398	MGYA00381405	https://www.ebi.ac.uk/metagenomics/api/v1/analyses/MGYA00381405/file/ERR2538398_FASTQ_SSU_OTU.tsv	Root-Host_associated-Human-Skin
SRR5869929	MGYA00420703	https://www.ebi.ac.uk/metagenomics/api/v1/analyses/MGYA00420703/file/SRR5869929_MERGED_FASTQ_SSU_OTU.tsv	Root-Host_associated-Human-Skin
SRR994561	MGYA00024432	https://www.ebi.ac.uk/metagenomics/api/v1/analyses/MGYA00024432/file/SRR994561_FASTQ_otu.tsv	Root-Host_associated-Human-Skin
SRR994913	MGYA00024749	https://www.ebi.ac.uk/metagenomics/api/v1/analyses/MGYA00024749/file/SRR994913_FASTQ_otu.tsv	Root-Host_associated-Human-Skin
SRR920899	MGYA00023741	https://www.ebi.ac.uk/metagenomics/api/v1/analyses/MGYA00023741/file/SRR920899_FASTQ_otu.tsv	Root-Host_associated-Human-Skin

SRR995321	MGYA00022478	https://www.ebi.ac.uk/metagenomics/api/v1/analyses/MGYA00022478/file/SRR995321_FASTQ_otu.tsv	Root-Host_associated-Human-Skin
ERR451199	MGYA00426074	https://www.ebi.ac.uk/metagenomics/api/v1/analyses/MGYA00426074/file/ERR451199_FASTQ_SSU_OTU.tsv	Root-Host_associated-Human-Skin
SRR996593	MGYA00023996	https://www.ebi.ac.uk/metagenomics/api/v1/analyses/MGYA00023996/file/SRR996593_FASTQ_otu.tsv	Root-Host_associated-Human-Skin
SRR2005669	MGYA00381326	https://www.ebi.ac.uk/metagenomics/api/v1/analyses/MGYA00381326/file/SRR2005669_MERGED_FASTQ_SSU_OTU.tsv	Root-Host_associated-Human-Skin
SRR996930	MGYA00022224	https://www.ebi.ac.uk/metagenomics/api/v1/analyses/MGYA00022224/file/SRR996930_FASTQ_otu.tsv	Root-Host_associated-Human-Skin
SRR995457	MGYA00025308	https://www.ebi.ac.uk/metagenomics/api/v1/analyses/MGYA00025308/file/SRR995457_FASTQ_otu.tsv	Root-Host_associated-Human-Skin
ERR2538349	MGYA00381356	https://www.ebi.ac.uk/metagenomics/api/v1/analyses/MGYA00381356/file/ERR2538349_FASTQ_SSU_OTU.tsv	Root-Host_associated-Human-Skin
SRR1924033	MGYA00028360	https://www.ebi.ac.uk/metagenomics/api/v1/analyses/MGYA00028360/file/SRR1924033_FASTQ_otu.tsv	Root-Host_associated-Human-Skin
SRR1631008	MGYA00024603	https://www.ebi.ac.uk/metagenomics/api/v1/analyses/MGYA00024603/file/SRR1631008_MERGED_FASTQ_otu.tsv	Root-Host_associated-Human-Skin
SRR994676	MGYA00023641	https://www.ebi.ac.uk/metagenomics/api/v1/analyses/MGYA00023641/file/SRR994676_FASTQ_otu.tsv	Root-Host_associated-Human-Skin
ERR1760026	MGYA00140499	https://www.ebi.ac.uk/metagenomics/api/v1/analyses/MGYA00140499/file/ERR1760026_MERGED_FASTQ_SSU_OTU.tsv	Root-Host_associated-Human-Skin
ERR451529	MGYA00422356	https://www.ebi.ac.uk/metagenomics/api/v1/analyses/MGYA00422356/file/ERR451529_FASTQ_SSU_OTU.tsv	Root-Host_associated-Human-Skin

SRR1923973	MGYA00028267	https://www.ebi.ac.uk/metagenomics/api/v1/analyses/MGYA00028267/file/SRR1923973_FASTQ_otu.tsv	Root-Host_associated-Human-Skin
SRR1632856	MGYA00023046	https://www.ebi.ac.uk/metagenomics/api/v1/analyses/MGYA00023046/file/SRR1632856_MERGED_FASTQ_otu.tsv	Root-Host_associated-Human-Skin
SRR920910	MGYA00022355	https://www.ebi.ac.uk/metagenomics/api/v1/analyses/MGYA00022355/file/SRR920910_FASTQ_otu.tsv	Root-Host_associated-Human-Skin
SRR1632383	MGYA00025304	https://www.ebi.ac.uk/metagenomics/api/v1/analyses/MGYA00025304/file/SRR1632383_MERGED_FASTQ_otu.tsv	Root-Host_associated-Human-Skin
SRR1631531	MGYA00023583	https://www.ebi.ac.uk/metagenomics/api/v1/analyses/MGYA00023583/file/SRR1631531_MERGED_FASTQ_otu.tsv	Root-Host_associated-Human-Skin
ERR1960632	MGYA00130797	https://www.ebi.ac.uk/metagenomics/api/v1/analyses/MGYA00130797/file/ERR1960632_MERGED_FASTQ_otu.tsv	Root-Host_associated-Human-Skin
ERR443820	MGYA00424250	https://www.ebi.ac.uk/metagenomics/api/v1/analyses/MGYA00424250/file/ERR443820_FASTQ_SSU_OTU.tsv	Root-Host_associated-Human-Skin
ERR451471	MGYA00425766	https://www.ebi.ac.uk/metagenomics/api/v1/analyses/MGYA00425766/file/ERR451471_FASTQ_SSU_OTU.tsv	Root-Host_associated-Human-Skin
SRR2005381	MGYA00381290	https://www.ebi.ac.uk/metagenomics/api/v1/analyses/MGYA00381290/file/SRR2005381_MERGED_FASTQ_SSU_OTU.tsv	Root-Host_associated-Human-Skin
SRR921035	MGYA00023078	https://www.ebi.ac.uk/metagenomics/api/v1/analyses/MGYA00023078/file/SRR921035_FASTQ_otu.tsv	Root-Host_associated-Human-Skin
SRR1633173	MGYA00025261	https://www.ebi.ac.uk/metagenomics/api/v1/analyses/MGYA00025261/file/SRR1633173_MERGED_FASTQ_otu.tsv	Root-Host_associated-Human-Skin
ERR1551328	MGYA00069429	https://www.ebi.ac.uk/metagenomics/api/v1/analyses/MGYA00069429/file/ERR1551328_MERGED_FASTQ_otu.tsv	Root-Host_associated-Human-Skin

ERR450935	MGYA00416696	https://www.ebi.ac.uk/metagenomics/api/v1/analyses/MGYA00416696/file/ERR450935_FASTQ_SSU_OTU.tsv	Root-Host_associated-Human-Skin
SRR1950736	MGYA00376903	https://www.ebi.ac.uk/metagenomics/api/v1/analyses/MGYA00376903/file/SRR1950736_MERGED_FASTQ_SSU_OTU.tsv	Root-Host_associated-Human-Skin
ERR1759960	MGYA00140578	https://www.ebi.ac.uk/metagenomics/api/v1/analyses/MGYA00140578/file/ERR1759960_MERGED_FASTQ_SSU_OTU.tsv	Root-Host_associated-Human-Skin
SRR1704524	MGYA00417447	https://www.ebi.ac.uk/metagenomics/api/v1/analyses/MGYA00417447/file/SRR1704524_FASTQ_SSU_OTU.tsv	Root-Host_associated-Human-Skin
ERR443745	MGYA00424262	https://www.ebi.ac.uk/metagenomics/api/v1/analyses/MGYA00424262/file/ERR443745_FASTQ_SSU_OTU.tsv	Root-Host_associated-Human-Skin
SRR995279	MGYA00025067	https://www.ebi.ac.uk/metagenomics/api/v1/analyses/MGYA00025067/file/SRR995279_FASTQ_otu.tsv	Root-Host_associated-Human-Skin
ERR1760085	MGYA00140417	https://www.ebi.ac.uk/metagenomics/api/v1/analyses/MGYA00140417/file/ERR1760085_MERGED_FASTQ_SSU_OTU.tsv	Root-Host_associated-Human-Skin
SRR995095	MGYA00023392	https://www.ebi.ac.uk/metagenomics/api/v1/analyses/MGYA00023392/file/SRR995095_FASTQ_otu.tsv	Root-Host_associated-Human-Skin
SRR995732	MGYA00023103	https://www.ebi.ac.uk/metagenomics/api/v1/analyses/MGYA00023103/file/SRR995732_FASTQ_otu.tsv	Root-Host_associated-Human-Skin
SRR996180	MGYA00022961	https://www.ebi.ac.uk/metagenomics/api/v1/analyses/MGYA00022961/file/SRR996180_FASTQ_otu.tsv	Root-Host_associated-Human-Skin
SRR1923888	MGYA00028335	https://www.ebi.ac.uk/metagenomics/api/v1/analyses/MGYA00028335/file/SRR1923888_FASTQ_otu.tsv	Root-Host_associated-Human-Skin
SRR996246	MGYA00023710	https://www.ebi.ac.uk/metagenomics/api/v1/analyses/MGYA00023710/file/SRR996246_FASTQ_otu.tsv	Root-Host_associated-Human-Skin

SRR996530	MGYA00022843	https://www.ebi.ac.uk/metagenomics/api/v1/analyses/MGYA00022843/file/SRR996530_FASTQ_otu.tsv	Root-Host_associated-Human-Skin
ERR443119	MGYA00424911	https://www.ebi.ac.uk/metagenomics/api/v1/analyses/MGYA00424911/file/ERR443119_FASTQ_SSU_OTU.tsv	Root-Host_associated-Human-Skin
SRR1923859	MGYA00028288	https://www.ebi.ac.uk/metagenomics/api/v1/analyses/MGYA00028288/file/SRR1923859_FASTQ_otu.tsv	Root-Host_associated-Human-Skin
SRR995044	MGYA00023695	https://www.ebi.ac.uk/metagenomics/api/v1/analyses/MGYA00023695/file/SRR995044_FASTQ_otu.tsv	Root-Host_associated-Human-Skin
ERR1760054	MGYA00140556	https://www.ebi.ac.uk/metagenomics/api/v1/analyses/MGYA00140556/file/ERR1760054_MERGED_FASTQ_SSU_OTU.tsv	Root-Host_associated-Human-Skin
SRR5870034	MGYA00416930	https://www.ebi.ac.uk/metagenomics/api/v1/analyses/MGYA00416930/file/SRR5870034_MERGED_FASTQ_SSU_OTU.tsv	Root-Host_associated-Human-Skin
ERR1960718	MGYA00130766	https://www.ebi.ac.uk/metagenomics/api/v1/analyses/MGYA00130766/file/ERR1960718_MERGED_FASTQ_otu.tsv	Root-Host_associated-Human-Skin
SRR1621011	MGYA00024797	https://www.ebi.ac.uk/metagenomics/api/v1/analyses/MGYA00024797/file/SRR1621011_MERGED_FASTQ_otu.tsv	Root-Host_associated-Human-Skin
SRR1923901	MGYA00028327	https://www.ebi.ac.uk/metagenomics/api/v1/analyses/MGYA00028327/file/SRR1923901_FASTQ_otu.tsv	Root-Host_associated-Human-Skin
SRR5868681	MGYA00417138	https://www.ebi.ac.uk/metagenomics/api/v1/analyses/MGYA00417138/file/SRR5868681_MERGED_FASTQ_SSU_OTU.tsv	Root-Host_associated-Human-Skin
SRR921096	MGYA00022428	https://www.ebi.ac.uk/metagenomics/api/v1/analyses/MGYA00022428/file/SRR921096_FASTQ_otu.tsv	Root-Host_associated-Human-Skin
SRR1632790	MGYA00023374	https://www.ebi.ac.uk/metagenomics/api/v1/analyses/MGYA00023374/file/SRR1632790_MERGED_FASTQ_otu.tsv	Root-Host_associated-Human-Skin

SRR920171	MGYA00024710	https://www.ebi.ac.uk/metagenomics/api/v1/analyses/MGYA00024710/file/SRR920171_FASTQ_otu.tsv	Root-Host_associated-Human-Skin
ERR443387	MGYA00428731	https://www.ebi.ac.uk/metagenomics/api/v1/analyses/MGYA00428731/file/ERR443387_FASTQ_SSU_OTU.tsv	Root-Host_associated-Human-Skin
ERR1960640	MGYA00130759	https://www.ebi.ac.uk/metagenomics/api/v1/analyses/MGYA00130759/file/ERR1960640_MERGED_FASTQ_otu.tsv	Root-Host_associated-Human-Skin
SRR1631685	MGYA00024833	https://www.ebi.ac.uk/metagenomics/api/v1/analyses/MGYA00024833/file/SRR1631685_MERGED_FASTQ_otu.tsv	Root-Host_associated-Human-Skin
SRR996009	MGYA00023522	https://www.ebi.ac.uk/metagenomics/api/v1/analyses/MGYA00023522/file/SRR996009_FASTQ_otu.tsv	Root-Host_associated-Human-Skin
SRR1622917	MGYA00025081	https://www.ebi.ac.uk/metagenomics/api/v1/analyses/MGYA00025081/file/SRR1622917_MERGED_FASTQ_otu.tsv	Root-Host_associated-Human-Skin
ERR2538515	MGYA00381522	https://www.ebi.ac.uk/metagenomics/api/v1/analyses/MGYA00381522/file/ERR2538515_FASTQ_SSU_OTU.tsv	Root-Host_associated-Human-Skin
ERR441979	MGYA00385231	https://www.ebi.ac.uk/metagenomics/api/v1/analyses/MGYA00385231/file/ERR441979_FASTQ_SSU_OTU.tsv	Root-Host_associated-Human-Skin
ERR1960691	MGYA00130883	https://www.ebi.ac.uk/metagenomics/api/v1/analyses/MGYA00130883/file/ERR1960691_MERGED_FASTQ_otu.tsv	Root-Host_associated-Human-Skin
SRR920607	MGYA00022562	https://www.ebi.ac.uk/metagenomics/api/v1/analyses/MGYA00022562/file/SRR920607_FASTQ_otu.tsv	Root-Host_associated-Human-Skin
ERR443356	MGYA00413353	https://www.ebi.ac.uk/metagenomics/api/v1/analyses/MGYA00413353/file/ERR443356_FASTQ_SSU_OTU.tsv	Root-Host_associated-Human-Skin
SRR5868650	MGYA00420817	https://www.ebi.ac.uk/metagenomics/api/v1/analyses/MGYA00420817/file/SRR5868650_MERGED_FASTQ_SSU_OTU.tsv	Root-Host_associated-Human-Skin

ERR1759912	MGYA00140428	https://www.ebi.ac.uk/metagenomics/api/v1/analyses/MGYA00140428/file/ERR1759912_MERGED_FASTQ_SSU_OTU.tsv	Root-Host_associated-Human-Skin
SRR1950744	MGYA00376911	https://www.ebi.ac.uk/metagenomics/api/v1/analyses/MGYA00376911/file/SRR1950744_MERGED_FASTQ_SSU_OTU.tsv	Root-Host_associated-Human-Skin
SRR1632320	MGYA00023479	https://www.ebi.ac.uk/metagenomics/api/v1/analyses/MGYA00023479/file/SRR1632320_MERGED_FASTQ_otu.tsv	Root-Host_associated-Human-Skin
SRR920266	MGYA00023689	https://www.ebi.ac.uk/metagenomics/api/v1/analyses/MGYA00023689/file/SRR920266_FASTQ_otu.tsv	Root-Host_associated-Human-Skin
SRR1632487	MGYA00022676	https://www.ebi.ac.uk/metagenomics/api/v1/analyses/MGYA00022676/file/SRR1632487_MERGED_FASTQ_otu.tsv	Root-Host_associated-Human-Skin
ERR2538396	MGYA00381403	https://www.ebi.ac.uk/metagenomics/api/v1/analyses/MGYA00381403/file/ERR2538396_FASTQ_SSU_OTU.tsv	Root-Host_associated-Human-Skin
ERR2538431	MGYA00381438	https://www.ebi.ac.uk/metagenomics/api/v1/analyses/MGYA00381438/file/ERR2538431_FASTQ_SSU_OTU.tsv	Root-Host_associated-Human-Skin
ERR1701216	MGYA00084286	https://www.ebi.ac.uk/metagenomics/api/v1/analyses/MGYA00084286/file/ERR1701216_FASTQ_otu.tsv	Root-Host_associated-Human-Skin
SRR1704472	MGYA00419165	https://www.ebi.ac.uk/metagenomics/api/v1/analyses/MGYA00419165/file/SRR1704472_FASTQ_SSU_OTU.tsv	Root-Host_associated-Human-Skin
ERR1960801	MGYA00130791	https://www.ebi.ac.uk/metagenomics/api/v1/analyses/MGYA00130791/file/ERR1960801_MERGED_FASTQ_otu.tsv	Root-Host_associated-Human-Skin
SRR995459	MGYA00025314	https://www.ebi.ac.uk/metagenomics/api/v1/analyses/MGYA00025314/file/SRR995459_FASTQ_otu.tsv	Root-Host_associated-Human-Skin
SRR5869642	MGYA00428048	https://www.ebi.ac.uk/metagenomics/api/v1/analyses/MGYA00428048/file/SRR5869642_MERGED_FASTQ_SSU_OTU.tsv	Root-Host_associated-Human-Skin

SRR2005667	MGYA00381325	https://www.ebi.ac.uk/metagenomics/api/v1/analyses/MGYA00381325/file/SRR2005667_MERGED_FASTQ_SSU_OTU.tsv	Root-Host_associated-Human-Skin
SRR1631467	MGYA00024984	https://www.ebi.ac.uk/metagenomics/api/v1/analyses/MGYA00024984/file/SRR1631467_MERGED_FASTQ_otu.tsv	Root-Host_associated-Human-Skin
ERR1760028	MGYA00140540	https://www.ebi.ac.uk/metagenomics/api/v1/analyses/MGYA00140540/file/ERR1760028_MERGED_FASTQ_SSU_OTU.tsv	Root-Host_associated-Human-Skin
ERR1960764	MGYA00130890	https://www.ebi.ac.uk/metagenomics/api/v1/analyses/MGYA00130890/file/ERR1960764_MERGED_FASTQ_otu.tsv	Root-Host_associated-Human-Skin
ERR443504	MGYA00414266	https://www.ebi.ac.uk/metagenomics/api/v1/analyses/MGYA00414266/file/ERR443504_FASTQ_SSU_OTU.tsv	Root-Host_associated-Human-Skin
SRR994678	MGYA00023635	https://www.ebi.ac.uk/metagenomics/api/v1/analyses/MGYA00023635/file/SRR994678_FASTQ_otu.tsv	Root-Host_associated-Human-Skin
SRR1631006	MGYA00024597	https://www.ebi.ac.uk/metagenomics/api/v1/analyses/MGYA00024597/file/SRR1631006_MERGED_FASTQ_otu.tsv	Root-Host_associated-Human-Skin
SRR6757610	MGYA00380728	https://www.ebi.ac.uk/metagenomics/api/v1/analyses/MGYA00380728/file/SRR6757610_MERGED_FASTQ_SSU_OTU.tsv	Root-Host_associated-Human-Skin
SRR5869223	MGYA00425433	https://www.ebi.ac.uk/metagenomics/api/v1/analyses/MGYA00425433/file/SRR5869223_MERGED_FASTQ_SSU_OTU.tsv	Root-Host_associated-Human-Skin
SRR5869584	MGYA00418047	https://www.ebi.ac.uk/metagenomics/api/v1/analyses/MGYA00418047/file/SRR5869584_MERGED_FASTQ_SSU_OTU.tsv	Root-Host_associated-Human-Skin
SRR1622833	MGYA00023714	https://www.ebi.ac.uk/metagenomics/api/v1/analyses/MGYA00023714/file/SRR1622833_MERGED_FASTQ_otu.tsv	Root-Host_associated-Human-Skin
SRR996414	MGYA00024302	https://www.ebi.ac.uk/metagenomics/api/v1/analyses/MGYA00024302/file/SRR996414_FASTQ_otu.tsv	Root-Host_associated-Human-Skin

SRR1632858	MGYA00023044	https://www.ebi.ac.uk/metagenomics/api/v1/analyses/MGYA00023044/file/SRR1632858_MERGED_FASTQ_otu.tsv	Root-Host_associated-Human-Skin
SRR995501	MGYA00023177	https://www.ebi.ac.uk/metagenomics/api/v1/analyses/MGYA00023177/file/SRR995501_FASTQ_otu.tsv	Root-Host_associated-Human-Skin
SRR995160	MGYA00024329	https://www.ebi.ac.uk/metagenomics/api/v1/analyses/MGYA00024329/file/SRR995160_FASTQ_otu.tsv	Root-Host_associated-Human-Skin
ERR1551326	MGYA00069430	https://www.ebi.ac.uk/metagenomics/api/v1/analyses/MGYA00069430/file/ERR1551326_MERGED_FASTQ_otu.tsv	Root-Host_associated-Human-Skin
ERR442918	MGYA00416712	https://www.ebi.ac.uk/metagenomics/api/v1/analyses/MGYA00416712/file/ERR442918_FASTQ_SSU_OTU.tsv	Root-Host_associated-Human-Skin
ERR451309	MGYA00419504	https://www.ebi.ac.uk/metagenomics/api/v1/analyses/MGYA00419504/file/ERR451309_FASTQ_SSU_OTU.tsv	Root-Host_associated-Human-Skin
SRR996703	MGYA00022329	https://www.ebi.ac.uk/metagenomics/api/v1/analyses/MGYA00022329/file/SRR996703_FASTQ_otu.tsv	Root-Host_associated-Human-Skin
SRR995277	MGYA00025061	https://www.ebi.ac.uk/metagenomics/api/v1/analyses/MGYA00025061/file/SRR995277_FASTQ_otu.tsv	Root-Host_associated-Human-Skin
ERR442003	MGYA00385159	https://www.ebi.ac.uk/metagenomics/api/v1/analyses/MGYA00385159/file/ERR442003_FASTQ_SSU_OTU.tsv	Root-Host_associated-Human-Skin
SRR994845	MGYA00022614	https://www.ebi.ac.uk/metagenomics/api/v1/analyses/MGYA00022614/file/SRR994845_FASTQ_otu.tsv	Root-Host_associated-Human-Skin
SRR996362	MGYA00022503	https://www.ebi.ac.uk/metagenomics/api/v1/analyses/MGYA00022503/file/SRR996362_FASTQ_otu.tsv	Root-Host_associated-Human-Skin
SRR1950738	MGYA00376905	https://www.ebi.ac.uk/metagenomics/api/v1/analyses/MGYA00376905/file/SRR1950738_MERGED_FASTQ_SSU_OTU.tsv	Root-Host_associated-Human-Skin

ERR2538436	MGYA00381443	https://www.ebi.ac.uk/metagenomics/api/v1/analyses/MGYA00381443/file/ERR2538436_FASTQ_SSU_OTU.tsv	Root-Host_associated-Human-Skin
SRR5869920	MGYA00416439	https://www.ebi.ac.uk/metagenomics/api/v1/analyses/MGYA00416439/file/SRR5869920_MERGED_FASTQ_SSU_OTU.tsv	Root-Host_associated-Human-Skin
ERR2538391	MGYA00381398	https://www.ebi.ac.uk/metagenomics/api/v1/analyses/MGYA00381398/file/ERR2538391_FASTQ_SSU_OTU.tsv	Root-Host_associated-Human-Skin
ERR1701211	MGYA00084295	https://www.ebi.ac.uk/metagenomics/api/v1/analyses/MGYA00084295/file/ERR1701211_FASTQ_otu.tsv	Root-Host_associated-Human-Skin
ERR443275	MGYA00413684	https://www.ebi.ac.uk/metagenomics/api/v1/analyses/MGYA00413684/file/ERR443275_FASTQ_SSU_OTU.tsv	Root-Host_associated-Human-Skin
SRR5869694	MGYA00418037	https://www.ebi.ac.uk/metagenomics/api/v1/analyses/MGYA00418037/file/SRR5869694_MERGED_FASTQ_SSU_OTU.tsv	Root-Host_associated-Human-Skin
SRR1922566	MGYA00028304	https://www.ebi.ac.uk/metagenomics/api/v1/analyses/MGYA00028304/file/SRR1922566_FASTQ_otu.tsv	Root-Host_associated-Human-Skin
ERR1960806	MGYA00130900	https://www.ebi.ac.uk/metagenomics/api/v1/analyses/MGYA00130900/file/ERR1960806_MERGED_FASTQ_otu.tsv	Root-Host_associated-Human-Skin
SRR994568	MGYA00024439	https://www.ebi.ac.uk/metagenomics/api/v1/analyses/MGYA00024439/file/SRR994568_FASTQ_otu.tsv	Root-Host_associated-Human-Skin
ERR443614	MGYA00428661	https://www.ebi.ac.uk/metagenomics/api/v1/analyses/MGYA00428661/file/ERR443614_FASTQ_SSU_OTU.tsv	Root-Host_associated-Human-Skin
SRR995328	MGYA00022483	https://www.ebi.ac.uk/metagenomics/api/v1/analyses/MGYA00022483/file/SRR995328_FASTQ_otu.tsv	Root-Host_associated-Human-Skin
ERR450701	MGYA00426546	https://www.ebi.ac.uk/metagenomics/api/v1/analyses/MGYA00426546/file/ERR450701_FASTQ_SSU_OTU.tsv	Root-Host_associated-Human-Skin

SRR1704303	MGYA00421513	https://www.ebi.ac.uk/metagenomics/api/v1/analyses/MGYA00421513/file/SRR1704303_FASTQ_SSU_OTU.tsv	Root-Host_associated-Human-Skin
SRR996939	MGYA00022233	https://www.ebi.ac.uk/metagenomics/api/v1/analyses/MGYA00022233/file/SRR996939_FASTQ_otu.tsv	Root-Host_associated-Human-Skin
SRR2005660	MGYA00381318	https://www.ebi.ac.uk/metagenomics/api/v1/analyses/MGYA00381318/file/SRR2005660_MERGED_FASTQ_SSU_OTU.tsv	Root-Host_associated-Human-Skin
ERR1960763	MGYA00130840	https://www.ebi.ac.uk/metagenomics/api/v1/analyses/MGYA00130840/file/ERR1960763_MERGED_FASTQ_otu.tsv	Root-Host_associated-Human-Skin
SRR1923796	MGYA00028394	https://www.ebi.ac.uk/metagenomics/api/v1/analyses/MGYA00028394/file/SRR1923796_FASTQ_otu.tsv	Root-Host_associated-Human-Skin
SRR1622834	MGYA00023716	https://www.ebi.ac.uk/metagenomics/api/v1/analyses/MGYA00023716/file/SRR1622834_MERGED_FASTQ_otu.tsv	Root-Host_associated-Human-Skin
SRR5869583	MGYA00421615	https://www.ebi.ac.uk/metagenomics/api/v1/analyses/MGYA00421615/file/SRR5869583_MERGED_FASTQ_SSU_OTU.tsv	Root-Host_associated-Human-Skin
SRR6757617	MGYA00380735	https://www.ebi.ac.uk/metagenomics/api/v1/analyses/MGYA00380735/file/SRR6757617_MERGED_FASTQ_SSU_OTU.tsv	Root-Host_associated-Human-Skin
SRR1631538	MGYA00023575	https://www.ebi.ac.uk/metagenomics/api/v1/analyses/MGYA00023575/file/SRR1631538_MERGED_FASTQ_otu.tsv	Root-Host_associated-Human-Skin
SRR996861	MGYA00024402	https://www.ebi.ac.uk/metagenomics/api/v1/analyses/MGYA00024402/file/SRR996861_FASTQ_otu.tsv	Root-Host_associated-Human-Skin
SRR996413	MGYA00024299	https://www.ebi.ac.uk/metagenomics/api/v1/analyses/MGYA00024299/file/SRR996413_FASTQ_otu.tsv	Root-Host_associated-Human-Skin
SRR995506	MGYA00023170	https://www.ebi.ac.uk/metagenomics/api/v1/analyses/MGYA00023170/file/SRR995506_FASTQ_otu.tsv	Root-Host_associated-Human-Skin

SRR920919	MGYA00022349	https://www.ebi.ac.uk/metagenomics/api/v1/analyses/MGYA00022349/file/SRR920919_FASTQ_otu.tsv	Root-Host_associated-Human-Skin
SRR995167	MGYA00024328	https://www.ebi.ac.uk/metagenomics/api/v1/analyses/MGYA00024328/file/SRR995167_FASTQ_otu.tsv	Root-Host_associated-Human-Skin
ERR1551321	MGYA00069435	https://www.ebi.ac.uk/metagenomics/api/v1/analyses/MGYA00069435/file/ERR1551321_MERGED_FASTQ_otu.tsv	Root-Host_associated-Human-Skin
SRR996704	MGYA00022332	https://www.ebi.ac.uk/metagenomics/api/v1/analyses/MGYA00022332/file/SRR996704_FASTQ_otu.tsv	Root-Host_associated-Human-Skin
SRR994842	MGYA00022611	https://www.ebi.ac.uk/metagenomics/api/v1/analyses/MGYA00022611/file/SRR994842_FASTQ_otu.tsv	Root-Host_associated-Human-Skin
ERR442004	MGYA00385176	https://www.ebi.ac.uk/metagenomics/api/v1/analyses/MGYA00385176/file/ERR442004_FASTQ_SSU_OTU.tsv	Root-Host_associated-Human-Skin
SRR995270	MGYA00025062	https://www.ebi.ac.uk/metagenomics/api/v1/analyses/MGYA00025062/file/SRR995270_FASTQ_otu.tsv	Root-Host_associated-Human-Skin
SRR996365	MGYA00022500	https://www.ebi.ac.uk/metagenomics/api/v1/analyses/MGYA00022500/file/SRR996365_FASTQ_otu.tsv	Root-Host_associated-Human-Skin
SRR1623049	MGYA00024635	https://www.ebi.ac.uk/metagenomics/api/v1/analyses/MGYA00024635/file/SRR1623049_MERGED_FASTQ_otu.tsv	Root-Host_associated-Human-Skin
ERR1759969	MGYA00140451	https://www.ebi.ac.uk/metagenomics/api/v1/analyses/MGYA00140451/file/ERR1759969_MERGED_FASTQ_SSU_OTU.tsv	Root-Host_associated-Human-Skin
SRR995735	MGYA00023096	https://www.ebi.ac.uk/metagenomics/api/v1/analyses/MGYA00023096/file/SRR995735_FASTQ_otu.tsv	Root-Host_associated-Human-Skin
ERR1760082	MGYA00140369	https://www.ebi.ac.uk/metagenomics/api/v1/analyses/MGYA00140369/file/ERR1760082_MERGED_FASTQ_SSU_OTU.tsv	Root-Host_associated-Human-Skin

ERR981247	MGYA00010237	https://www.ebi.ac.uk/metagenomics/api/v1/analyses/MGYA00010237/file/ERR981247_MERGED_FASTQ_otu.tsv	Root-Host_associated-Human-Skin
SRR1622899	MGYA00024762	https://www.ebi.ac.uk/metagenomics/api/v1/analyses/MGYA00024762/file/SRR1622899_MERGED_FASTQ_otu.tsv	Root-Host_associated-Human-Skin
SRR996187	MGYA00022954	https://www.ebi.ac.uk/metagenomics/api/v1/analyses/MGYA00022954/file/SRR996187_FASTQ_otu.tsv	Root-Host_associated-Human-Skin
SRR996241	MGYA00023705	https://www.ebi.ac.uk/metagenomics/api/v1/analyses/MGYA00023705/file/SRR996241_FASTQ_otu.tsv	Root-Host_associated-Human-Skin
SRR1630958	MGYA00024159	https://www.ebi.ac.uk/metagenomics/api/v1/analyses/MGYA00024159/file/SRR1630958_MERGED_FASTQ_otu.tsv	Root-Host_associated-Human-Skin
SRR5869639	MGYA00415905	https://www.ebi.ac.uk/metagenomics/api/v1/analyses/MGYA00415905/file/SRR5869639_MERGED_FASTQ_SSU_OTU.tsv	Root-Host_associated-Human-Skin
SRR996537	MGYA00022838	https://www.ebi.ac.uk/metagenomics/api/v1/analyses/MGYA00022838/file/SRR996537_FASTQ_otu.tsv	Root-Host_associated-Human-Skin
SRR995043	MGYA00023696	https://www.ebi.ac.uk/metagenomics/api/v1/analyses/MGYA00023696/file/SRR995043_FASTQ_otu.tsv	Root-Host_associated-Human-Skin
ERR1760053	MGYA00140377	https://www.ebi.ac.uk/metagenomics/api/v1/analyses/MGYA00140377/file/ERR1760053_MERGED_FASTQ_SSU_OTU.tsv	Root-Host_associated-Human-Skin
SRR1923906	MGYA00028330	https://www.ebi.ac.uk/metagenomics/api/v1/analyses/MGYA00028330/file/SRR1923906_FASTQ_otu.tsv	Root-Host_associated-Human-Skin
SRR995908	MGYA00023844	https://www.ebi.ac.uk/metagenomics/api/v1/analyses/MGYA00023844/file/SRR995908_FASTQ_otu.tsv	Root-Host_associated-Human-Skin
SRR5868686	MGYA00420813	https://www.ebi.ac.uk/metagenomics/api/v1/analyses/MGYA00420813/file/SRR5868686_MERGED_FASTQ_SSU_OTU.tsv	Root-Host_associated-Human-Skin

ERR443380	MGYA00428733	https://www.ebi.ac.uk/metagenomics/api/v1/analyses/MGYA00428733/file/ERR443380_FASTQ_SSU_OTU.tsv	Root-Host_associated-Human-Skin
ERR1960647	MGYA00130744	https://www.ebi.ac.uk/metagenomics/api/v1/analyses/MGYA00130744/file/ERR1960647_MERGED_FASTQ_otu.tsv	Root-Host_associated-Human-Skin
SRR1632797	MGYA00023371	https://www.ebi.ac.uk/metagenomics/api/v1/analyses/MGYA00023371/file/SRR1632797_MERGED_FASTQ_otu.tsv	Root-Host_associated-Human-Skin
ERR443427	MGYA00426513	https://www.ebi.ac.uk/metagenomics/api/v1/analyses/MGYA00426513/file/ERR443427_FASTQ_SSU_OTU.tsv	Root-Host_associated-Human-Skin
SRR920176	MGYA00024713	https://www.ebi.ac.uk/metagenomics/api/v1/analyses/MGYA00024713/file/SRR920176_FASTQ_otu.tsv	Root-Host_associated-Human-Skin
SRR921091	MGYA00022425	https://www.ebi.ac.uk/metagenomics/api/v1/analyses/MGYA00022425/file/SRR921091_FASTQ_otu.tsv	Root-Host_associated-Human-Skin
SRR1622910	MGYA00025086	https://www.ebi.ac.uk/metagenomics/api/v1/analyses/MGYA00025086/file/SRR1622910_MERGED_FASTQ_otu.tsv	Root-Host_associated-Human-Skin
SRR1631682	MGYA00024826	https://www.ebi.ac.uk/metagenomics/api/v1/analyses/MGYA00024826/file/SRR1631682_MERGED_FASTQ_otu.tsv	Root-Host_associated-Human-Skin
ERR2538512	MGYA00381519	https://www.ebi.ac.uk/metagenomics/api/v1/analyses/MGYA00381519/file/ERR2538512_FASTQ_SSU_OTU.tsv	Root-Host_associated-Human-Skin
ERR1960696	MGYA00130793	https://www.ebi.ac.uk/metagenomics/api/v1/analyses/MGYA00130793/file/ERR1960696_MERGED_FASTQ_otu.tsv	Root-Host_associated-Human-Skin
SRR920600	MGYA00022565	https://www.ebi.ac.uk/metagenomics/api/v1/analyses/MGYA00022565/file/SRR920600_FASTQ_otu.tsv	Root-Host_associated-Human-Skin
ERR443351	MGYA00426046	https://www.ebi.ac.uk/metagenomics/api/v1/analyses/MGYA00426046/file/ERR443351_FASTQ_SSU_OTU.tsv	Root-Host_associated-Human-Skin

SRR1950743	MGYA00376910	https://www.ebi.ac.uk/metagenomics/api/v1/analyses/MGYA00376910/file/SRR1950743_MERGED_FASTQ_SSU_OTU.tsv	Root-Host_associated-Human-Skin
ERR1759915	MGYA00140425	https://www.ebi.ac.uk/metagenomics/api/v1/analyses/MGYA00140425/file/ERR1759915_MERGED_FASTQ_SSU_OTU.tsv	Root-Host_associated-Human-Skin
SRR5868657	MGYA00417147	https://www.ebi.ac.uk/metagenomics/api/v1/analyses/MGYA00417147/file/SRR5868657_MERGED_FASTQ_SSU_OTU.tsv	Root-Host_associated-Human-Skin
SRR1704551	MGYA00421477	https://www.ebi.ac.uk/metagenomics/api/v1/analyses/MGYA00421477/file/SRR1704551_FASTQ_SSU_OTU.tsv	Root-Host_associated-Human-Skin
SRR920261	MGYA00023686	https://www.ebi.ac.uk/metagenomics/api/v1/analyses/MGYA00023686/file/SRR920261_FASTQ_otu.tsv	Root-Host_associated-Human-Skin
SRR1632480	MGYA00022679	https://www.ebi.ac.uk/metagenomics/api/v1/analyses/MGYA00022679/file/SRR1632480_MERGED_FASTQ_otu.tsv	Root-Host_associated-Human-Skin
SRR1632327	MGYA00023478	https://www.ebi.ac.uk/metagenomics/api/v1/analyses/MGYA00023478/file/SRR1632327_MERGED_FASTQ_otu.tsv	Root-Host_associated-Human-Skin
ERR443097	MGYA00426781	https://www.ebi.ac.uk/metagenomics/api/v1/analyses/MGYA00426781/file/ERR443097_FASTQ_SSU_OTU.tsv	Root-Host_associated-Human-Skin
SRR995413	MGYA00024927	https://www.ebi.ac.uk/metagenomics/api/v1/analyses/MGYA00024927/file/SRR995413_FASTQ_otu.tsv	Root-Host_associated-Human-Skin
ERR443688	MGYA00428652	https://www.ebi.ac.uk/metagenomics/api/v1/analyses/MGYA00428652/file/ERR443688_FASTQ_SSU_OTU.tsv	Root-Host_associated-Human-Skin
SRR996974	MGYA00022642	https://www.ebi.ac.uk/metagenomics/api/v1/analyses/MGYA00022642/file/SRR996974_FASTQ_otu.tsv	Root-Host_associated-Human-Skin
SRR994632	MGYA00022568	https://www.ebi.ac.uk/metagenomics/api/v1/analyses/MGYA00022568/file/SRR994632_FASTQ_otu.tsv	Root-Host_associated-Human-Skin

SRR995072	MGYA00024640	https://www.ebi.ac.uk/metagenomics/api/v1/analyses/MGYA00024640/file/SRR995072_FASTQ_otu.tsv	Root-Host_associated-Human-Skin
ERR1760062	MGYA00140497	https://www.ebi.ac.uk/metagenomics/api/v1/analyses/MGYA00140497/file/ERR1760062_MERGED_FASTQ_SSU_OTU.tsv	Root-Host_associated-Human-Skin
SRR996167	MGYA00022739	https://www.ebi.ac.uk/metagenomics/api/v1/analyses/MGYA00022739/file/SRR996167_FASTQ_otu.tsv	Root-Host_associated-Human-Skin
SRR1622879	MGYA00024186	https://www.ebi.ac.uk/metagenomics/api/v1/analyses/MGYA00024186/file/SRR1622879_MERGED_FASTQ_otu.tsv	Root-Host_associated-Human-Skin
SRR997051	MGYA00022967	https://www.ebi.ac.uk/metagenomics/api/v1/analyses/MGYA00022967/file/SRR997051_FASTQ_otu.tsv	Root-Host_associated-Human-Skin
SRR921129	MGYA00025189	https://www.ebi.ac.uk/metagenomics/api/v1/analyses/MGYA00025189/file/SRR921129_FASTQ_otu.tsv	Root-Host_associated-Human-Skin
ERR450829	MGYA00422957	https://www.ebi.ac.uk/metagenomics/api/v1/analyses/MGYA00422957/file/ERR450829_FASTQ_SSU_OTU.tsv	Root-Host_associated-Human-Skin
SRR994957	MGYA00024340	https://www.ebi.ac.uk/metagenomics/api/v1/analyses/MGYA00024340/file/SRR994957_FASTQ_otu.tsv	Root-Host_associated-Human-Skin
SRR994525	MGYA00024047	https://www.ebi.ac.uk/metagenomics/api/v1/analyses/MGYA00024047/file/SRR994525_FASTQ_otu.tsv	Root-Host_associated-Human-Skin
SRR995365	MGYA00022916	https://www.ebi.ac.uk/metagenomics/api/v1/analyses/MGYA00022916/file/SRR995365_FASTQ_otu.tsv	Root-Host_associated-Human-Skin
SRR921071	MGYA00022628	https://www.ebi.ac.uk/metagenomics/api/v1/analyses/MGYA00022628/file/SRR921071_FASTQ_otu.tsv	Root-Host_associated-Human-Skin
SRR1633137	MGYA00024876	https://www.ebi.ac.uk/metagenomics/api/v1/analyses/MGYA00024876/file/SRR1633137_MERGED_FASTQ_otu.tsv	Root-Host_associated-Human-Skin

ERR450971	MGYA00426788	https://www.ebi.ac.uk/metagenomics/api/v1/analyses/MGYA00426788/file/ERR450971_FASTQ_SSU_OTU.tsv	Root-Host_associated-Human-Skin
SRR920196	MGYA00023269	https://www.ebi.ac.uk/metagenomics/api/v1/analyses/MGYA00023269/file/SRR920196_FASTQ_otu.tsv	Root-Host_associated-Human-Skin
SRR1631662	MGYA00023148	https://www.ebi.ac.uk/metagenomics/api/v1/analyses/MGYA00023148/file/SRR1631662_MERGED_FASTQ_otu.tsv	Root-Host_associated-Human-Skin
SRR996749	MGYA00023618	https://www.ebi.ac.uk/metagenomics/api/v1/analyses/MGYA00023618/file/SRR996749_FASTQ_otu.tsv	Root-Host_associated-Human-Skin
ERR2538523	MGYA00381530	https://www.ebi.ac.uk/metagenomics/api/v1/analyses/MGYA00381530/file/ERR2538523_FASTQ_SSU_OTU.tsv	Root-Host_associated-Human-Skin
ERR443701	MGYA00413315	https://www.ebi.ac.uk/metagenomics/api/v1/analyses/MGYA00413315/file/ERR443701_FASTQ_SSU_OTU.tsv	Root-Host_associated-Human-Skin
ERR442049	MGYA00385238	https://www.ebi.ac.uk/metagenomics/api/v1/analyses/MGYA00385238/file/ERR442049_FASTQ_SSU_OTU.tsv	Root-Host_associated-Human-Skin
SRR996328	MGYA00022951	https://www.ebi.ac.uk/metagenomics/api/v1/analyses/MGYA00022951/file/SRR996328_FASTQ_otu.tsv	Root-Host_associated-Human-Skin
SRR1950772	MGYA00376939	https://www.ebi.ac.uk/metagenomics/api/v1/analyses/MGYA00376939/file/SRR1950772_MERGED_FASTQ_SSU_OTU.tsv	Root-Host_associated-Human-Skin
ERR1759924	MGYA00140410	https://www.ebi.ac.uk/metagenomics/api/v1/analyses/MGYA00140410/file/ERR1759924_MERGED_FASTQ_SSU_OTU.tsv	Root-Host_associated-Human-Skin
SRR1923937	MGYA00028260	https://www.ebi.ac.uk/metagenomics/api/v1/analyses/MGYA00028260/file/SRR1923937_FASTQ_otu.tsv	Root-Host_associated-Human-Skin
SRR1632812	MGYA00022590	https://www.ebi.ac.uk/metagenomics/api/v1/analyses/MGYA00022590/file/SRR1632812_MERGED_FASTQ_otu.tsv	Root-Host_associated-Human-Skin

ERR443077	MGYA00414550	https://www.ebi.ac.uk/metagenomics/api/v1/analyses/MGYA00414550/file/ERR443077_FASTQ_SSU_OTU.tsv	Root-Host_associated-Human-Skin
SRR920954	MGYA00022783	https://www.ebi.ac.uk/metagenomics/api/v1/analyses/MGYA00022783/file/SRR920954_FASTQ_otu.tsv	Root-Host_associated-Human-Skin
SRR1622921	MGYA00022409	https://www.ebi.ac.uk/metagenomics/api/v1/analyses/MGYA00022409/file/SRR1622921_MERGED_FASTQ_otu.tsv	Root-Host_associated-Human-Skin
SRR1631114	MGYA00022865	https://www.ebi.ac.uk/metagenomics/api/v1/analyses/MGYA00022865/file/SRR1631114_MERGED_FASTQ_otu.tsv	Root-Host_associated-Human-Skin
SRR1704677	MGYA00420588	https://www.ebi.ac.uk/metagenomics/api/v1/analyses/MGYA00420588/file/SRR1704677_FASTQ_SSU_OTU.tsv	Root-Host_associated-Human-Skin
ERR1960676	MGYA00130849	https://www.ebi.ac.uk/metagenomics/api/v1/analyses/MGYA00130849/file/ERR1960676_MERGED_FASTQ_otu.tsv	Root-Host_associated-Human-Skin
ERR451392	MGYA00419152	https://www.ebi.ac.uk/metagenomics/api/v1/analyses/MGYA00419152/file/ERR451392_FASTQ_SSU_OTU.tsv	Root-Host_associated-Human-Skin
SRR1704495	MGYA00424943	https://www.ebi.ac.uk/metagenomics/api/v1/analyses/MGYA00424943/file/SRR1704495_FASTQ_SSU_OTU.tsv	Root-Host_associated-Human-Skin
SRR1631451	MGYA00025120	https://www.ebi.ac.uk/metagenomics/api/v1/analyses/MGYA00025120/file/SRR1631451_MERGED_FASTQ_otu.tsv	Root-Host_associated-Human-Skin
SRR1950687	MGYA00376854	https://www.ebi.ac.uk/metagenomics/api/v1/analyses/MGYA00376854/file/SRR1950687_MERGED_FASTQ_SSU_OTU.tsv	Root-Host_associated-Human-Skin
SRR2005651	MGYA00381310	https://www.ebi.ac.uk/metagenomics/api/v1/analyses/MGYA00381310/file/SRR2005651_MERGED_FASTQ_SSU_OTU.tsv	Root-Host_associated-Human-Skin
SRR1923813	MGYA00028401	https://www.ebi.ac.uk/metagenomics/api/v1/analyses/MGYA00028401/file/SRR1923813_FASTQ_otu.tsv	Root-Host_associated-Human-Skin

SRR994588	MGYA00022922	https://www.ebi.ac.uk/metagenomics/api/v1/analyses/MGYA00022922/file/SRR994588_FASTQ_otu.tsv	Root-Host_associated-Human-Skin
ERR2538371	MGYA00381378	https://www.ebi.ac.uk/metagenomics/api/v1/analyses/MGYA00381378/file/ERR2538371_FASTQ_SSU_OTU.tsv	Root-Host_associated-Human-Skin
SRR5869215	MGYA00415953	https://www.ebi.ac.uk/metagenomics/api/v1/analyses/MGYA00415953/file/SRR5869215_MERGED_FASTQ_SSU_OTU.tsv	Root-Host_associated-Human-Skin
SRR6757626	MGYA00380743	https://www.ebi.ac.uk/metagenomics/api/v1/analyses/MGYA00380743/file/SRR6757626_MERGED_FASTQ_SSU_OTU.tsv	Root-Host_associated-Human-Skin
SRR5868827	MGYA00427143	https://www.ebi.ac.uk/metagenomics/api/v1/analyses/MGYA00427143/file/SRR5868827_MERGED_FASTQ_SSU_OTU.tsv	Root-Host_associated-Human-Skin
ERR443532	MGYA00422360	https://www.ebi.ac.uk/metagenomics/api/v1/analyses/MGYA00422360/file/ERR443532_FASTQ_SSU_OTU.tsv	Root-Host_associated-Human-Skin
ERR443295	MGYA00412990	https://www.ebi.ac.uk/metagenomics/api/v1/analyses/MGYA00412990/file/ERR443295_FASTQ_SSU_OTU.tsv	Root-Host_associated-Human-Skin
ERR1960752	MGYA00130768	https://www.ebi.ac.uk/metagenomics/api/v1/analyses/MGYA00130768/file/ERR1960752_MERGED_FASTQ_otu.tsv	Root-Host_associated-Human-Skin
SRR1633013	MGYA00023955	https://www.ebi.ac.uk/metagenomics/api/v1/analyses/MGYA00023955/file/SRR1633013_MERGED_FASTQ_otu.tsv	Root-Host_associated-Human-Skin
ERR1701220	MGYA00084321	https://www.ebi.ac.uk/metagenomics/api/v1/analyses/MGYA00084321/file/ERR1701220_FASTQ_otu.tsv	Root-Host_associated-Human-Skin
ERR1960783	MGYA00130764	https://www.ebi.ac.uk/metagenomics/api/v1/analyses/MGYA00130764/file/ERR1960783_MERGED_FASTQ_otu.tsv	Root-Host_associated-Human-Skin
ERR443244	MGYA00426082	https://www.ebi.ac.uk/metagenomics/api/v1/analyses/MGYA00426082/file/ERR443244_FASTQ_SSU_OTU.tsv	Root-Host_associated-Human-Skin

ERR2538407	MGYA00381414	https://www.ebi.ac.uk/metagenomics/api/v1/analyses/MGYA00381414/file/ERR2538407_FASTQ_SSU_OTU.tsv	Root-Host_associated-Human-Skin
SRR5869563	MGYA00415921	https://www.ebi.ac.uk/metagenomics/api/v1/analyses/MGYA00415921/file/SRR5869563_MERGED_FASTQ_SSU_OTU.tsv	Root-Host_associated-Human-Skin
SRR1922557	MGYA00028240	https://www.ebi.ac.uk/metagenomics/api/v1/analyses/MGYA00028240/file/SRR1922557_FASTQ_otu.tsv	Root-Host_associated-Human-Skin
SRR5868742	MGYA00421196	https://www.ebi.ac.uk/metagenomics/api/v1/analyses/MGYA00421196/file/SRR5868742_MERGED_FASTQ_SSU_OTU.tsv	Root-Host_associated-Human-Skin
SRR1631480	MGYA00024932	https://www.ebi.ac.uk/metagenomics/api/v1/analyses/MGYA00024932/file/SRR1631480_MERGED_FASTQ_otu.tsv	Root-Host_associated-Human-Skin
ERR451498	MGYA00428685	https://www.ebi.ac.uk/metagenomics/api/v1/analyses/MGYA00428685/file/ERR451498_FASTQ_SSU_OTU.tsv	Root-Host_associated-Human-Skin
SRR1624860	MGYA00024468	https://www.ebi.ac.uk/metagenomics/api/v1/analyses/MGYA00024468/file/SRR1624860_MERGED_FASTQ_otu.tsv	Root-Host_associated-Human-Skin
SRR995620	MGYA00025222	https://www.ebi.ac.uk/metagenomics/api/v1/analyses/MGYA00025222/file/SRR995620_FASTQ_otu.tsv	Root-Host_associated-Human-Skin
SRR919925	MGYA00025194	https://www.ebi.ac.uk/metagenomics/api/v1/analyses/MGYA00025194/file/SRR919925_FASTQ_otu.tsv	Root-Host_associated-Human-Skin
SRR919557	MGYA00024498	https://www.ebi.ac.uk/metagenomics/api/v1/analyses/MGYA00024498/file/SRR919557_FASTQ_otu.tsv	Root-Host_associated-Human-Skin
ERR1759958	MGYA00140507	https://www.ebi.ac.uk/metagenomics/api/v1/analyses/MGYA00140507/file/ERR1759958_MERGED_FASTQ_SSU_OTU.tsv	Root-Host_associated-Human-Skin
SRR5870231	MGYA00418689	https://www.ebi.ac.uk/metagenomics/api/v1/analyses/MGYA00418689/file/SRR5870231_MERGED_FASTQ_SSU_OTU.tsv	Root-Host_associated-Human-Skin

SRR994873	MGYA00025266	https://www.ebi.ac.uk/metagenomics/api/v1/analyses/MGYA00025266/file/SRR994873_FASTQ_otu.tsv	Root-Host_associated-Human-Skin
ERR442035	MGYA00385179	https://www.ebi.ac.uk/metagenomics/api/v1/analyses/MGYA00385179/file/ERR442035_FASTQ_SSU_OTU.tsv	Root-Host_associated-Human-Skin
SRR995945	MGYA00024282	https://www.ebi.ac.uk/metagenomics/api/v1/analyses/MGYA00024282/file/SRR995945_FASTQ_otu.tsv	Root-Host_associated-Human-Skin
ERR451028	MGYA00426527	https://www.ebi.ac.uk/metagenomics/api/v1/analyses/MGYA00426527/file/ERR451028_FASTQ_SSU_OTU.tsv	Root-Host_associated-Human-Skin
SRR995290	MGYA00023594	https://www.ebi.ac.uk/metagenomics/api/v1/analyses/MGYA00023594/file/SRR995290_FASTQ_otu.tsv	Root-Host_associated-Human-Skin
ERR1759989	MGYA00140595	https://www.ebi.ac.uk/metagenomics/api/v1/analyses/MGYA00140595/file/ERR1759989_MERGED_FASTQ_SSU_OTU.tsv	Root-Host_associated-Human-Skin
SRR996850	MGYA00023216	https://www.ebi.ac.uk/metagenomics/api/v1/analyses/MGYA00023216/file/SRR996850_FASTQ_otu.tsv	Root-Host_associated-Human-Skin
SRR2005709	MGYA00381339	https://www.ebi.ac.uk/metagenomics/api/v1/analyses/MGYA00381339/file/SRR2005709_MERGED_FASTQ_SSU_OTU.tsv	Root-Host_associated-Human-Skin
ERR443818	MGYA00413636	https://www.ebi.ac.uk/metagenomics/api/v1/analyses/MGYA00413636/file/ERR443818_FASTQ_SSU_OTU.tsv	Root-Host_associated-Human-Skin
SRR994716	MGYA00023646	https://www.ebi.ac.uk/metagenomics/api/v1/analyses/MGYA00023646/file/SRR994716_FASTQ_otu.tsv	Root-Host_associated-Human-Skin
SRR5870126	MGYA00417505	https://www.ebi.ac.uk/metagenomics/api/v1/analyses/MGYA00417505/file/SRR5870126_MERGED_FASTQ_SSU_OTU.tsv	Root-Host_associated-Human-Skin
ERR451449	MGYA00424292	https://www.ebi.ac.uk/metagenomics/api/v1/analyses/MGYA00424292/file/ERR451449_FASTQ_SSU_OTU.tsv	Root-Host_associated-Human-Skin

SRR996043	MGYA00023069	https://www.ebi.ac.uk/metagenomics/api/v1/analyses/MGYA00023069/file/SRR996043_FASTQ_otu.tsv	Root-Host_associated-Human-Skin
SRR5869673	MGYA00416988	https://www.ebi.ac.uk/metagenomics/api/v1/analyses/MGYA00416988/file/SRR5869673_MERGED_FASTQ_SSU_OTU.tsv	Root-Host_associated-Human-Skin
SRR1920901	MGYA00028252	https://www.ebi.ac.uk/metagenomics/api/v1/analyses/MGYA00028252/file/SRR1920901_FASTQ_otu.tsv	Root-Host_associated-Human-Skin
SRR2005656	MGYA00381314	https://www.ebi.ac.uk/metagenomics/api/v1/analyses/MGYA00381314/file/SRR2005656_MERGED_FASTQ_SSU_OTU.tsv	Root-Host_associated-Human-Skin
SRR1631456	MGYA00025123	https://www.ebi.ac.uk/metagenomics/api/v1/analyses/MGYA00025123/file/SRR1631456_MERGED_FASTQ_otu.tsv	Root-Host_associated-Human-Skin
SRR5868794	MGYA00422579	https://www.ebi.ac.uk/metagenomics/api/v1/analyses/MGYA00422579/file/SRR5868794_MERGED_FASTQ_SSU_OTU.tsv	Root-Host_associated-Human-Skin
ERR450737	MGYA00417794	https://www.ebi.ac.uk/metagenomics/api/v1/analyses/MGYA00417794/file/ERR450737_FASTQ_SSU_OTU.tsv	Root-Host_associated-Human-Skin
SRR1704754	MGYA00428433	https://www.ebi.ac.uk/metagenomics/api/v1/analyses/MGYA00428433/file/SRR1704754_FASTQ_SSU_OTU.tsv	Root-Host_associated-Human-Skin
SRR5868820	MGYA00420757	https://www.ebi.ac.uk/metagenomics/api/v1/analyses/MGYA00420757/file/SRR5868820_MERGED_FASTQ_SSU_OTU.tsv	Root-Host_associated-Human-Skin
SRR6757621	MGYA00380738	https://www.ebi.ac.uk/metagenomics/api/v1/analyses/MGYA00380738/file/SRR6757621_MERGED_FASTQ_SSU_OTU.tsv	Root-Host_associated-Human-Skin
SRR1623230	MGYA00024121	https://www.ebi.ac.uk/metagenomics/api/v1/analyses/MGYA00024121/file/SRR1623230_MERGED_FASTQ_otu.tsv	Root-Host_associated-Human-Skin
ERR2538376	MGYA00381383	https://www.ebi.ac.uk/metagenomics/api/v1/analyses/MGYA00381383/file/ERR2538376_FASTQ_SSU_OTU.tsv	Root-Host_associated-Human-Skin

SRR5869212	MGYA00426234	https://www.ebi.ac.uk/metagenomics/api/v1/analyses/MGYA00426234/file/SRR5869212_MERGED_FASTQ_SSU_OTU.tsv	Root-Host_associated-Human-Skin
ERR1760019	MGYA00140562	https://www.ebi.ac.uk/metagenomics/api/v1/analyses/MGYA00140562/file/ERR1760019_MERGED_FASTQ_SSU_OTU.tsv	Root-Host_associated-Human-Skin
SRR995009	MGYA00024152	https://www.ebi.ac.uk/metagenomics/api/v1/analyses/MGYA00024152/file/SRR995009_FASTQ_otu.tsv	Root-Host_associated-Human-Skin
ERR443292	MGYA00423300	https://www.ebi.ac.uk/metagenomics/api/v1/analyses/MGYA00423300/file/ERR443292_FASTQ_SSU_OTU.tsv	Root-Host_associated-Human-Skin
ERR1960755	MGYA00130848	https://www.ebi.ac.uk/metagenomics/api/v1/analyses/MGYA00130848/file/ERR1960755_MERGED_FASTQ_otu.tsv	Root-Host_associated-Human-Skin
ERR450852	MGYA00426823	https://www.ebi.ac.uk/metagenomics/api/v1/analyses/MGYA00426823/file/ERR450852_FASTQ_SSU_OTU.tsv	Root-Host_associated-Human-Skin
ERR1960784	MGYA00130874	https://www.ebi.ac.uk/metagenomics/api/v1/analyses/MGYA00130874/file/ERR1960784_MERGED_FASTQ_otu.tsv	Root-Host_associated-Human-Skin
ERR443243	MGYA00416298	https://www.ebi.ac.uk/metagenomics/api/v1/analyses/MGYA00416298/file/ERR443243_FASTQ_SSU_OTU.tsv	Root-Host_associated-Human-Skin
SRR994698	MGYA00023883	https://www.ebi.ac.uk/metagenomics/api/v1/analyses/MGYA00023883/file/SRR994698_FASTQ_otu.tsv	Root-Host_associated-Human-Skin
ERR1701227	MGYA00084313	https://www.ebi.ac.uk/metagenomics/api/v1/analyses/MGYA00084313/file/ERR1701227_FASTQ_otu.tsv	Root-Host_associated-Human-Skin
ERR451260	MGYA00423299	https://www.ebi.ac.uk/metagenomics/api/v1/analyses/MGYA00423299/file/ERR451260_FASTQ_SSU_OTU.tsv	Root-Host_associated-Human-Skin
SRR1633014	MGYA00023958	https://www.ebi.ac.uk/metagenomics/api/v1/analyses/MGYA00023958/file/SRR1633014_MERGED_FASTQ_otu.tsv	Root-Host_associated-Human-Skin

SRR1923771	MGYA00028313	https://www.ebi.ac.uk/metagenomics/api/v1/analyses/MGYA00028313/file/SRR1923771_FASTQ_otu.tsv	Root-Host_associated-Human-Skin
ERR2538400	MGYA00381407	https://www.ebi.ac.uk/metagenomics/api/v1/analyses/MGYA00381407/file/ERR2538400_FASTQ_SSU_OTU.tsv	Root-Host_associated-Human-Skin
SRR5869564	MGYA00414852	https://www.ebi.ac.uk/metagenomics/api/v1/analyses/MGYA00414852/file/SRR5869564_MERGED_FASTQ_SSU_OTU.tsv	Root-Host_associated-Human-Skin
ERR443622	MGYA00412596	https://www.ebi.ac.uk/metagenomics/api/v1/analyses/MGYA00412596/file/ERR443622_FASTQ_SSU_OTU.tsv	Root-Host_associated-Human-Skin
ERR1960830	MGYA00130789	https://www.ebi.ac.uk/metagenomics/api/v1/analyses/MGYA00130789/file/ERR1960830_MERGED_FASTQ_otu.tsv	Root-Host_associated-Human-Skin
SRR1631320	MGYA00023021	https://www.ebi.ac.uk/metagenomics/api/v1/analyses/MGYA00023021/file/SRR1631320_MERGED_FASTQ_otu.tsv	Root-Host_associated-Human-Skin
SRR5869105	MGYA00416008	https://www.ebi.ac.uk/metagenomics/api/v1/analyses/MGYA00416008/file/SRR5869105_MERGED_FASTQ_SSU_OTU.tsv	Root-Host_associated-Human-Skin
ERR451338	MGYA00419499	https://www.ebi.ac.uk/metagenomics/api/v1/analyses/MGYA00419499/file/ERR451338_FASTQ_SSU_OTU.tsv	Root-Host_associated-Human-Skin
SRR995627	MGYA00025223	https://www.ebi.ac.uk/metagenomics/api/v1/analyses/MGYA00025223/file/SRR995627_FASTQ_otu.tsv	Root-Host_associated-Human-Skin
SRR1624867	MGYA00024467	https://www.ebi.ac.uk/metagenomics/api/v1/analyses/MGYA00024467/file/SRR1624867_MERGED_FASTQ_otu.tsv	Root-Host_associated-Human-Skin
SRR1631278	MGYA00024739	https://www.ebi.ac.uk/metagenomics/api/v1/analyses/MGYA00024739/file/SRR1631278_MERGED_FASTQ_otu.tsv	Root-Host_associated-Human-Skin
SRR919550	MGYA00024501	https://www.ebi.ac.uk/metagenomics/api/v1/analyses/MGYA00024501/file/SRR919550_FASTQ_otu.tsv	Root-Host_associated-Human-Skin

SRR919922	MGYA00025199	https://www.ebi.ac.uk/metagenomics/api/v1/analyses/MGYA00025199/file/SRR919922_FASTQ_otu.tsv	Root-Host_associated-Human-Skin
SRR1950709	MGYA00376876	https://www.ebi.ac.uk/metagenomics/api/v1/analyses/MGYA00376876/file/SRR1950709_MERGED_FASTQ_SSU_OTU.tsv	Root-Host_associated-Human-Skin
ERR442032	MGYA00385243	https://www.ebi.ac.uk/metagenomics/api/v1/analyses/MGYA00385243/file/ERR442032_FASTQ_SSU_OTU.tsv	Root-Host_associated-Human-Skin
SRR994874	MGYA00022910	https://www.ebi.ac.uk/metagenomics/api/v1/analyses/MGYA00022910/file/SRR994874_FASTQ_otu.tsv	Root-Host_associated-Human-Skin
SRR994406	MGYA00025140	https://www.ebi.ac.uk/metagenomics/api/v1/analyses/MGYA00025140/file/SRR994406_FASTQ_otu.tsv	Root-Host_associated-Human-Skin
SRR995297	MGYA00023597	https://www.ebi.ac.uk/metagenomics/api/v1/analyses/MGYA00023597/file/SRR995297_FASTQ_otu.tsv	Root-Host_associated-Human-Skin
SRR995942	MGYA00024283	https://www.ebi.ac.uk/metagenomics/api/v1/analyses/MGYA00024283/file/SRR995942_FASTQ_otu.tsv	Root-Host_associated-Human-Skin
SRR996857	MGYA00023221	https://www.ebi.ac.uk/metagenomics/api/v1/analyses/MGYA00023221/file/SRR996857_FASTQ_otu.tsv	Root-Host_associated-Human-Skin
ERR1760141	MGYA00140494	https://www.ebi.ac.uk/metagenomics/api/v1/analyses/MGYA00140494/file/ERR1760141_MERGED_FASTQ_SSU_OTU.tsv	Root-Host_associated-Human-Skin
SRR994711	MGYA00023649	https://www.ebi.ac.uk/metagenomics/api/v1/analyses/MGYA00023649/file/SRR994711_FASTQ_otu.tsv	Root-Host_associated-Human-Skin
SRR5868978	MGYA00426253	https://www.ebi.ac.uk/metagenomics/api/v1/analyses/MGYA00426253/file/SRR5868978_MERGED_FASTQ_SSU_OTU.tsv	Root-Host_associated-Human-Skin
SRR995414	MGYA00024928	https://www.ebi.ac.uk/metagenomics/api/v1/analyses/MGYA00024928/file/SRR995414_FASTQ_otu.tsv	Root-Host_associated-Human-Skin

SRR1923868	MGYA00028398	https://www.ebi.ac.uk/metagenomics/api/v1/analyses/MGYA00028398/file/SRR1923868_FASTQ_otu.tsv	Root-Host_associated-Human-Skin
SRR996973	MGYA00022645	https://www.ebi.ac.uk/metagenomics/api/v1/analyses/MGYA00022645/file/SRR996973_FASTQ_otu.tsv	Root-Host_associated-Human-Skin
SRR1704349	MGYA00426958	https://www.ebi.ac.uk/metagenomics/api/v1/analyses/MGYA00426958/file/SRR1704349_FASTQ_SSU_OTU.tsv	Root-Host_associated-Human-Skin
SRR995075	MGYA00024654	https://www.ebi.ac.uk/metagenomics/api/v1/analyses/MGYA00024654/file/SRR995075_FASTQ_otu.tsv	Root-Host_associated-Human-Skin
ERR1760065	MGYA00140545	https://www.ebi.ac.uk/metagenomics/api/v1/analyses/MGYA00140545/file/ERR1760065_MERGED_FASTQ_SSU_OTU.tsv	Root-Host_associated-Human-Skin
ERR443549	MGYA00428688	https://www.ebi.ac.uk/metagenomics/api/v1/analyses/MGYA00428688/file/ERR443549_FASTQ_SSU_OTU.tsv	Root-Host_associated-Human-Skin
SRR994635	MGYA00022506	https://www.ebi.ac.uk/metagenomics/api/v1/analyses/MGYA00022506/file/SRR994635_FASTQ_otu.tsv	Root-Host_associated-Human-Skin
ERR1960729	MGYA00130834	https://www.ebi.ac.uk/metagenomics/api/v1/analyses/MGYA00130834/file/ERR1960729_MERGED_FASTQ_otu.tsv	Root-Host_associated-Human-Skin
SRR996160	MGYA00023201	https://www.ebi.ac.uk/metagenomics/api/v1/analyses/MGYA00023201/file/SRR996160_FASTQ_otu.tsv	Root-Host_associated-Human-Skin
SRR997056	MGYA00022968	https://www.ebi.ac.uk/metagenomics/api/v1/analyses/MGYA00022968/file/SRR997056_FASTQ_otu.tsv	Root-Host_associated-Human-Skin
SRR995703	MGYA00023827	https://www.ebi.ac.uk/metagenomics/api/v1/analyses/MGYA00023827/file/SRR995703_FASTQ_otu.tsv	Root-Host_associated-Human-Skin
SRR5869179	MGYA00420260	https://www.ebi.ac.uk/metagenomics/api/v1/analyses/MGYA00420260/file/SRR5869179_MERGED_FASTQ_SSU_OTU.tsv	Root-Host_associated-Human-Skin

SRR995362	MGYA00022917	https://www.ebi.ac.uk/metagenomics/api/v1/analyses/MGYA00022917/file/SRR995362_FASTQ_otu.tsv	Root-Host_associated-Human-Skin
SRR994522	MGYA00024050	https://www.ebi.ac.uk/metagenomics/api/v1/analyses/MGYA00024050/file/SRR994522_FASTQ_otu.tsv	Root-Host_associated-Human-Skin
SRR994950	MGYA00024337	https://www.ebi.ac.uk/metagenomics/api/v1/analyses/MGYA00024337/file/SRR994950_FASTQ_otu.tsv	Root-Host_associated-Human-Skin
SRR920191	MGYA00023262	https://www.ebi.ac.uk/metagenomics/api/v1/analyses/MGYA00023262/file/SRR920191_FASTQ_otu.tsv	Root-Host_associated-Human-Skin
SRR921076	MGYA00022635	https://www.ebi.ac.uk/metagenomics/api/v1/analyses/MGYA00022635/file/SRR921076_FASTQ_otu.tsv	Root-Host_associated-Human-Skin
SRR5869832	MGYA00415867	https://www.ebi.ac.uk/metagenomics/api/v1/analyses/MGYA00415867/file/SRR5869832_MERGED_FASTQ_SSU_OTU.tsv	Root-Host_associated-Human-Skin
ERR2538524	MGYA00381531	https://www.ebi.ac.uk/metagenomics/api/v1/analyses/MGYA00381531/file/ERR2538524_FASTQ_SSU_OTU.tsv	Root-Host_associated-Human-Skin
SRR1631665	MGYA00023151	https://www.ebi.ac.uk/metagenomics/api/v1/analyses/MGYA00023151/file/SRR1631665_MERGED_FASTQ_otu.tsv	Root-Host_associated-Human-Skin
SRR994808	MGYA00023032	https://www.ebi.ac.uk/metagenomics/api/v1/analyses/MGYA00023032/file/SRR994808_FASTQ_otu.tsv	Root-Host_associated-Human-Skin
SRR1704567	MGYA00428831	https://www.ebi.ac.uk/metagenomics/api/v1/analyses/MGYA00428831/file/SRR1704567_FASTQ_SSU_OTU.tsv	Root-Host_associated-Human-Skin
ERR1759923	MGYA00140413	https://www.ebi.ac.uk/metagenomics/api/v1/analyses/MGYA00140413/file/ERR1759923_MERGED_FASTQ_SSU_OTU.tsv	Root-Host_associated-Human-Skin
SRR1950775	MGYA00376942	https://www.ebi.ac.uk/metagenomics/api/v1/analyses/MGYA00376942/file/SRR1950775_MERGED_FASTQ_SSU_OTU.tsv	Root-Host_associated-Human-Skin

SRR5869786	MGYA00416965	https://www.ebi.ac.uk/metagenomics/api/v1/analyses/MGYA00416965/file/SRR5869786_MERGED_FASTQ_SSU_OTU.tsv	Root-Host_associated-Human-Skin
SRR920953	MGYA00022788	https://www.ebi.ac.uk/metagenomics/api/v1/analyses/MGYA00022788/file/SRR920953_FASTQ_otu.tsv	Root-Host_associated-Human-Skin
SRR1632815	MGYA00022597	https://www.ebi.ac.uk/metagenomics/api/v1/analyses/MGYA00022597/file/SRR1632815_MERGED_FASTQ_otu.tsv	Root-Host_associated-Human-Skin
SRR1632467	MGYA00023136	https://www.ebi.ac.uk/metagenomics/api/v1/analyses/MGYA00023136/file/SRR1632467_MERGED_FASTQ_otu.tsv	Root-Host_associated-Human-Skin
SRR1631113	MGYA00022867	https://www.ebi.ac.uk/metagenomics/api/v1/analyses/MGYA00022867/file/SRR1631113_MERGED_FASTQ_otu.tsv	Root-Host_associated-Human-Skin
SRR1622926	MGYA00023792	https://www.ebi.ac.uk/metagenomics/api/v1/analyses/MGYA00023792/file/SRR1622926_MERGED_FASTQ_otu.tsv	Root-Host_associated-Human-Skin
ERR441999	MGYA00385164	https://www.ebi.ac.uk/metagenomics/api/v1/analyses/MGYA00385164/file/ERR441999_FASTQ_SSU_OTU.tsv	Root-Host_associated-Human-Skin
ERR451395	MGYA00415148	https://www.ebi.ac.uk/metagenomics/api/v1/analyses/MGYA00415148/file/ERR451395_FASTQ_SSU_OTU.tsv	Root-Host_associated-Human-Skin
ERR1960671	MGYA00130868	https://www.ebi.ac.uk/metagenomics/api/v1/analyses/MGYA00130868/file/ERR1960671_MERGED_FASTQ_otu.tsv	Root-Host_associated-Human-Skin
SRR994581	MGYA00022923	https://www.ebi.ac.uk/metagenomics/api/v1/analyses/MGYA00022923/file/SRR994581_FASTQ_otu.tsv	Root-Host_associated-Human-Skin
ERR450739	MGYA00426543	https://www.ebi.ac.uk/metagenomics/api/v1/analyses/MGYA00426543/file/ERR450739_FASTQ_SSU_OTU.tsv	Root-Host_associated-Human-Skin
SRR1631458	MGYA00025117	https://www.ebi.ac.uk/metagenomics/api/v1/analyses/MGYA00025117/file/SRR1631458_MERGED_FASTQ_otu.tsv	Root-Host_associated-Human-Skin

SRR2005658	MGYA00381316	https://www.ebi.ac.uk/metagenomics/api/v1/analyses/MGYA00381316/file/SRR2005658_MERGED_FASTQ_SSU_OTU.tsv	Root-Host_associated-Human-Skin
SRR996573	MGYA00022414	https://www.ebi.ac.uk/metagenomics/api/v1/analyses/MGYA00022414/file/SRR996573_FASTQ_otu.tsv	Root-Host_associated-Human-Skin
SRR995007	MGYA00024148	https://www.ebi.ac.uk/metagenomics/api/v1/analyses/MGYA00024148/file/SRR995007_FASTQ_otu.tsv	Root-Host_associated-Human-Skin
ERR1760017	MGYA00140361	https://www.ebi.ac.uk/metagenomics/api/v1/analyses/MGYA00140361/file/ERR1760017_MERGED_FASTQ_SSU_OTU.tsv	Root-Host_associated-Human-Skin
ERR451518	MGYA00425757	https://www.ebi.ac.uk/metagenomics/api/v1/analyses/MGYA00425757/file/ERR451518_FASTQ_SSU_OTU.tsv	Root-Host_associated-Human-Skin
ERR2538378	MGYA00381385	https://www.ebi.ac.uk/metagenomics/api/v1/analyses/MGYA00381385/file/ERR2538378_FASTQ_SSU_OTU.tsv	Root-Host_associated-Human-Skin
SRR996112	MGYA00025173	https://www.ebi.ac.uk/metagenomics/api/v1/analyses/MGYA00025173/file/SRR996112_FASTQ_otu.tsv	Root-Host_associated-Human-Skin
SRR1924002	MGYA00028287	https://www.ebi.ac.uk/metagenomics/api/v1/analyses/MGYA00028287/file/SRR1924002_FASTQ_otu.tsv	Root-Host_associated-Human-Skin
SRR5869918	MGYA00420708	https://www.ebi.ac.uk/metagenomics/api/v1/analyses/MGYA00420708/file/SRR5869918_MERGED_FASTQ_SSU_OTU.tsv	Root-Host_associated-Human-Skin
ERR1701229	MGYA00084316	https://www.ebi.ac.uk/metagenomics/api/v1/analyses/MGYA00084316/file/ERR1701229_FASTQ_otu.tsv	Root-Host_associated-Human-Skin
SRR996205	MGYA00024169	https://www.ebi.ac.uk/metagenomics/api/v1/analyses/MGYA00024169/file/SRR996205_FASTQ_otu.tsv	Root-Host_associated-Human-Skin
SRR1631489	MGYA00024917	https://www.ebi.ac.uk/metagenomics/api/v1/analyses/MGYA00024917/file/SRR1631489_MERGED_FASTQ_otu.tsv	Root-Host_associated-Human-Skin

SRR995629	MGYA00025213	https://www.ebi.ac.uk/metagenomics/api/v1/analyses/MGYA00025213/file/SRR995629_FASTQ_otu.tsv	Root-Host_associated-Human-Skin
SRR1624869	MGYA00024463	https://www.ebi.ac.uk/metagenomics/api/v1/analyses/MGYA00024463/file/SRR1624869_MERGED_FASTQ_otu.tsv	Root-Host_associated-Human-Skin
ERR451491	MGYA00428687	https://www.ebi.ac.uk/metagenomics/api/v1/analyses/MGYA00428687/file/ERR451491_FASTQ_SSU_OTU.tsv	Root-Host_associated-Human-Skin
SRR1633142	MGYA00022606	https://www.ebi.ac.uk/metagenomics/api/v1/analyses/MGYA00022606/file/SRR1633142_MERGED_FASTQ_otu.tsv	Root-Host_associated-Human-Skin
SRR994408	MGYA00025136	https://www.ebi.ac.uk/metagenomics/api/v1/analyses/MGYA00025136/file/SRR994408_FASTQ_otu.tsv	Root-Host_associated-Human-Skin
SRR920225	MGYA00023286	https://www.ebi.ac.uk/metagenomics/api/v1/analyses/MGYA00023286/file/SRR920225_FASTQ_otu.tsv	Root-Host_associated-Human-Skin
SRR5870238	MGYA00415828	https://www.ebi.ac.uk/metagenomics/api/v1/analyses/MGYA00415828/file/SRR5870238_MERGED_FASTQ_SSU_OTU.tsv	Root-Host_associated-Human-Skin
SRR920582	MGYA00024252	https://www.ebi.ac.uk/metagenomics/api/v1/analyses/MGYA00024252/file/SRR920582_FASTQ_otu.tsv	Root-Host_associated-Human-Skin
SRR1950707	MGYA00376874	https://www.ebi.ac.uk/metagenomics/api/v1/analyses/MGYA00376874/file/SRR1950707_MERGED_FASTQ_SSU_OTU.tsv	Root-Host_associated-Human-Skin
ERR1759951	MGYA00140476	https://www.ebi.ac.uk/metagenomics/api/v1/analyses/MGYA00140476/file/ERR1759951_MERGED_FASTQ_SSU_OTU.tsv	Root-Host_associated-Human-Skin
SRR1631276	MGYA00024735	https://www.ebi.ac.uk/metagenomics/api/v1/analyses/MGYA00024735/file/SRR1631276_MERGED_FASTQ_otu.tsv	Root-Host_associated-Human-Skin
SRR5868613	MGYA00417179	https://www.ebi.ac.uk/metagenomics/api/v1/analyses/MGYA00417179/file/SRR5868613_MERGED_FASTQ_SSU_OTU.tsv	Root-Host_associated-Human-Skin

ERR1759980	MGYA00140598	https://www.ebi.ac.uk/metagenomics/api/v1/analyses/MGYA00140598/file/ERR1759980_MERGED_FASTQ_SSU_OTU.tsv	Root-Host_associated-Human-Skin
SRR1704263	MGYA00425329	https://www.ebi.ac.uk/metagenomics/api/v1/analyses/MGYA00425329/file/SRR1704263_FASTQ_SSU_OTU.tsv	Root-Host_associated-Human-Skin
SRR996859	MGYA00023223	https://www.ebi.ac.uk/metagenomics/api/v1/analyses/MGYA00023223/file/SRR996859_FASTQ_otu.tsv	Root-Host_associated-Human-Skin
SRR995299	MGYA00023591	https://www.ebi.ac.uk/metagenomics/api/v1/analyses/MGYA00023591/file/SRR995299_FASTQ_otu.tsv	Root-Host_associated-Human-Skin
SRR1923942	MGYA00028229	https://www.ebi.ac.uk/metagenomics/api/v1/analyses/MGYA00028229/file/SRR1923942_FASTQ_otu.tsv	Root-Host_associated-Human-Skin
ERR451021	MGYA00416316	https://www.ebi.ac.uk/metagenomics/api/v1/analyses/MGYA00416316/file/ERR451021_FASTQ_SSU_OTU.tsv	Root-Host_associated-Human-Skin
SRR1632415	MGYA00023312	https://www.ebi.ac.uk/metagenomics/api/v1/analyses/MGYA00023312/file/SRR1632415_MERGED_FASTQ_otu.tsv	Root-Host_associated-Human-Skin
ERR443002	MGYA00416330	https://www.ebi.ac.uk/metagenomics/api/v1/analyses/MGYA00416330/file/ERR443002_FASTQ_SSU_OTU.tsv	Root-Host_associated-Human-Skin
SRR920695	MGYA00024837	https://www.ebi.ac.uk/metagenomics/api/v1/analyses/MGYA00024837/file/SRR920695_FASTQ_otu.tsv	Root-Host_associated-Human-Skin
SRR920132	MGYA00022975	https://www.ebi.ac.uk/metagenomics/api/v1/analyses/MGYA00022975/file/SRR920132_FASTQ_otu.tsv	Root-Host_associated-Human-Skin
ERR443811	MGYA00426777	https://www.ebi.ac.uk/metagenomics/api/v1/analyses/MGYA00426777/file/ERR443811_FASTQ_SSU_OTU.tsv	Root-Host_associated-Human-Skin
ERR451440	MGYA00428705	https://www.ebi.ac.uk/metagenomics/api/v1/analyses/MGYA00428705/file/ERR451440_FASTQ_SSU_OTU.tsv	Root-Host_associated-Human-Skin

SRR1631383	MGYA00024088	https://www.ebi.ac.uk/metagenomics/api/v1/analyses/MGYA00024088/file/SRR1631383_MERGED_FASTQ_otu.tsv	Root-Host_associated-Human-Skin
SRR1623184	MGYA00023678	https://www.ebi.ac.uk/metagenomics/api/v1/analyses/MGYA00023678/file/SRR1623184_MERGED_FASTQ_otu.tsv	Root-Host_associated-Human-Skin
SRR1923866	MGYA00028323	https://www.ebi.ac.uk/metagenomics/api/v1/analyses/MGYA00028323/file/SRR1923866_FASTQ_otu.tsv	Root-Host_associated-Human-Skin
ERR443126	MGYA00420149	https://www.ebi.ac.uk/metagenomics/api/v1/analyses/MGYA00420149/file/ERR443126_FASTQ_SSU_OTU.tsv	Root-Host_associated-Human-Skin
SRR1622870	MGYA00024183	https://www.ebi.ac.uk/metagenomics/api/v1/analyses/MGYA00024183/file/SRR1622870_MERGED_FASTQ_otu.tsv	Root-Host_associated-Human-Skin
SRR5868852	MGYA00416518	https://www.ebi.ac.uk/metagenomics/api/v1/analyses/MGYA00416518/file/SRR5868852_MERGED_FASTQ_SSU_OTU.tsv	Root-Host_associated-Human-Skin
SRR6757653	MGYA00380768	https://www.ebi.ac.uk/metagenomics/api/v1/analyses/MGYA00380768/file/SRR6757653_MERGED_FASTQ_SSU_OTU.tsv	Root-Host_associated-Human-Skin
SRR1631045	MGYA00025012	https://www.ebi.ac.uk/metagenomics/api/v1/analyses/MGYA00025012/file/SRR1631045_MERGED_FASTQ_otu.tsv	Root-Host_associated-Human-Skin
ERR1701284	MGYA00084315	https://www.ebi.ac.uk/metagenomics/api/v1/analyses/MGYA00084315/file/ERR1701284_FASTQ_otu.tsv	Root-Host_associated-Human-Skin
ERR1960727	MGYA00130758	https://www.ebi.ac.uk/metagenomics/api/v1/analyses/MGYA00130758/file/ERR1960727_MERGED_FASTQ_otu.tsv	Root-Host_associated-Human-Skin
ERR451564	MGYA00415115	https://www.ebi.ac.uk/metagenomics/api/v1/analyses/MGYA00415115/file/ERR451564_FASTQ_SSU_OTU.tsv	Root-Host_associated-Human-Skin
SRR921120	MGYA00025184	https://www.ebi.ac.uk/metagenomics/api/v1/analyses/MGYA00025184/file/SRR921120_FASTQ_otu.tsv	Root-Host_associated-Human-Skin

ERR443596	MGYA00421391	https://www.ebi.ac.uk/metagenomics/api/v1/analyses/MGYA00421391/file/ERR443596_FASTQ_SSU_OTU.tsv	Root-Host_associated-Human-Skin
ERR1701255	MGYA00084323	https://www.ebi.ac.uk/metagenomics/api/v1/analyses/MGYA00084323/file/ERR1701255_FASTQ_otu.tsv	Root-Host_associated-Human-Skin
SRR997058	MGYA00022973	https://www.ebi.ac.uk/metagenomics/api/v1/analyses/MGYA00022973/file/SRR997058_FASTQ_otu.tsv	Root-Host_associated-Human-Skin
SRR6757682	MGYA00380795	https://www.ebi.ac.uk/metagenomics/api/v1/analyses/MGYA00380795/file/SRR6757682_MERGED_FASTQ_SSU_OTU.tsv	Root-Host_associated-Human-Skin
ERR2538472	MGYA00381479	https://www.ebi.ac.uk/metagenomics/api/v1/analyses/MGYA00381479/file/ERR2538472_FASTQ_SSU_OTU.tsv	Root-Host_associated-Human-Skin
ERR450794	MGYA00422396	https://www.ebi.ac.uk/metagenomics/api/v1/analyses/MGYA00422396/file/ERR450794_FASTQ_SSU_OTU.tsv	Root-Host_associated-Human-Skin
SRR1632992	MGYA00022269	https://www.ebi.ac.uk/metagenomics/api/v1/analyses/MGYA00022269/file/SRR1632992_MERGED_FASTQ_otu.tsv	Root-Host_associated-Human-Skin
SRR1704431	MGYA00428854	https://www.ebi.ac.uk/metagenomics/api/v1/analyses/MGYA00428854/file/SRR1704431_FASTQ_SSU_OTU.tsv	Root-Host_associated-Human-Skin
SRR996740	MGYA00023615	https://www.ebi.ac.uk/metagenomics/api/v1/analyses/MGYA00023615/file/SRR996740_FASTQ_otu.tsv	Root-Host_associated-Human-Skin
SRR921078	MGYA00022637	https://www.ebi.ac.uk/metagenomics/api/v1/analyses/MGYA00022637/file/SRR921078_FASTQ_otu.tsv	Root-Host_associated-Human-Skin
SRR5870182	MGYA00418692	https://www.ebi.ac.uk/metagenomics/api/v1/analyses/MGYA00418692/file/SRR5870182_MERGED_FASTQ_SSU_OTU.tsv	Root-Host_associated-Human-Skin
ERR443369	MGYA00426041	https://www.ebi.ac.uk/metagenomics/api/v1/analyses/MGYA00426041/file/ERR443369_FASTQ_SSU_OTU.tsv	Root-Host_associated-Human-Skin

SRR996321	MGYA00022944	https://www.ebi.ac.uk/metagenomics/api/v1/analyses/MGYA00022944/file/SRR996321_FASTQ_otu.tsv	Root-Host_associated-Human-Skin
SRR5869788	MGYA00421181	https://www.ebi.ac.uk/metagenomics/api/v1/analyses/MGYA00421181/file/SRR5869788_MERGED_FASTQ_SSU_OTU.tsv	Root-Host_associated-Human-Skin
SRR1704569	MGYA00426919	https://www.ebi.ac.uk/metagenomics/api/v1/analyses/MGYA00426919/file/SRR1704569_FASTQ_SSU_OTU.tsv	Root-Host_associated-Human-Skin
SRR994806	MGYA00023024	https://www.ebi.ac.uk/metagenomics/api/v1/analyses/MGYA00023024/file/SRR994806_FASTQ_otu.tsv	Root-Host_associated-Human-Skin
SRR994474	MGYA00022247	https://www.ebi.ac.uk/metagenomics/api/v1/analyses/MGYA00022247/file/SRR994474_FASTQ_otu.tsv	Root-Host_associated-Human-Skin
ERR442040	MGYA00385156	https://www.ebi.ac.uk/metagenomics/api/v1/analyses/MGYA00385156/file/ERR442040_FASTQ_SSU_OTU.tsv	Root-Host_associated-Human-Skin
SRR995593	MGYA00025207	https://www.ebi.ac.uk/metagenomics/api/v1/analyses/MGYA00025207/file/SRR995593_FASTQ_otu.tsv	Root-Host_associated-Human-Skin
SRR995542	MGYA00022740	https://www.ebi.ac.uk/metagenomics/api/v1/analyses/MGYA00022740/file/SRR995542_FASTQ_otu.tsv	Root-Host_associated-Human-Skin
SRR5870295	MGYA00415818	https://www.ebi.ac.uk/metagenomics/api/v1/analyses/MGYA00415818/file/SRR5870295_MERGED_FASTQ_SSU_OTU.tsv	Root-Host_associated-Human-Skin
ERR1760133	MGYA00140486	https://www.ebi.ac.uk/metagenomics/api/v1/analyses/MGYA00140486/file/ERR1760133_MERGED_FASTQ_SSU_OTU.tsv	Root-Host_associated-Human-Skin
SRR995123	MGYA00023920	https://www.ebi.ac.uk/metagenomics/api/v1/analyses/MGYA00023920/file/SRR995123_FASTQ_otu.tsv	Root-Host_associated-Human-Skin
SRR919635	MGYA00023768	https://www.ebi.ac.uk/metagenomics/api/v1/analyses/MGYA00023768/file/SRR919635_FASTQ_otu.tsv	Root-Host_associated-Human-Skin

SRR1622928	MGYA00022402	https://www.ebi.ac.uk/metagenomics/api/v1/analyses/MGYA00022402/file/SRR1622928_MERGED_FASTQ_otu.tsv	Root-Host_associated-Human-Skin
ERR441997	MGYA00385160	https://www.ebi.ac.uk/metagenomics/api/v1/analyses/MGYA00385160/file/ERR441997_FASTQ_SSU_OTU.tsv	Root-Host_associated-Human-Skin
SRR5869606	MGYA00425423	https://www.ebi.ac.uk/metagenomics/api/v1/analyses/MGYA00425423/file/SRR5869606_MERGED_FASTQ_SSU_OTU.tsv	Root-Host_associated-Human-Skin
SRR1623183	MGYA00023673	https://www.ebi.ac.uk/metagenomics/api/v1/analyses/MGYA00023673/file/SRR1623183_MERGED_FASTQ_otu.tsv	Root-Host_associated-Human-Skin
SRR1631384	MGYA00024087	https://www.ebi.ac.uk/metagenomics/api/v1/analyses/MGYA00024087/file/SRR1631384_MERGED_FASTQ_otu.tsv	Root-Host_associated-Human-Skin
ERR443686	MGYA00415109	https://www.ebi.ac.uk/metagenomics/api/v1/analyses/MGYA00415109/file/ERR443686_FASTQ_SSU_OTU.tsv	Root-Host_associated-Human-Skin
SRR1923861	MGYA00028318	https://www.ebi.ac.uk/metagenomics/api/v1/analyses/MGYA00028318/file/SRR1923861_FASTQ_otu.tsv	Root-Host_associated-Human-Skin
SRR6757654	MGYA00380769	https://www.ebi.ac.uk/metagenomics/api/v1/analyses/MGYA00380769/file/SRR6757654_MERGED_FASTQ_SSU_OTU.tsv	Root-Host_associated-Human-Skin
SRR5868855	MGYA00420738	https://www.ebi.ac.uk/metagenomics/api/v1/analyses/MGYA00420738/file/SRR5868855_MERGED_FASTQ_SSU_OTU.tsv	Root-Host_associated-Human-Skin
SRR1704721	MGYA00426144	https://www.ebi.ac.uk/metagenomics/api/v1/analyses/MGYA00426144/file/SRR1704721_FASTQ_SSU_OTU.tsv	Root-Host_associated-Human-Skin
SRR1631042	MGYA00025009	https://www.ebi.ac.uk/metagenomics/api/v1/analyses/MGYA00025009/file/SRR1631042_MERGED_FASTQ_otu.tsv	Root-Host_associated-Human-Skin
SRR1622877	MGYA00024180	https://www.ebi.ac.uk/metagenomics/api/v1/analyses/MGYA00024180/file/SRR1622877_MERGED_FASTQ_otu.tsv	Root-Host_associated-Human-Skin

SRR996169	MGYA00022294	https://www.ebi.ac.uk/metagenomics/api/v1/analyses/MGYA00022294/file/SRR996169_FASTQ_otu.tsv	Root-Host_associated-Human-Skin
ERR451563	MGYA00424283	https://www.ebi.ac.uk/metagenomics/api/v1/analyses/MGYA00424283/file/ERR451563_FASTQ_SSU_OTU.tsv	Root-Host_associated-Human-Skin
ERR1960720	MGYA00130747	https://www.ebi.ac.uk/metagenomics/api/v1/analyses/MGYA00130747/file/ERR1960720_MERGED_FASTQ_otu.tsv	Root-Host_associated-Human-Skin
ERR1701283	MGYA00084280	https://www.ebi.ac.uk/metagenomics/api/v1/analyses/MGYA00084280/file/ERR1701283_FASTQ_otu.tsv	Root-Host_associated-Human-Skin
ERR1701252	MGYA00084263	https://www.ebi.ac.uk/metagenomics/api/v1/analyses/MGYA00084263/file/ERR1701252_FASTQ_otu.tsv	Root-Host_associated-Human-Skin
SRR921127	MGYA00025185	https://www.ebi.ac.uk/metagenomics/api/v1/analyses/MGYA00025185/file/SRR921127_FASTQ_otu.tsv	Root-Host_associated-Human-Skin
ERR2538475	MGYA00381482	https://www.ebi.ac.uk/metagenomics/api/v1/analyses/MGYA00381482/file/ERR2538475_FASTQ_SSU_OTU.tsv	Root-Host_associated-Human-Skin
SRR5869963	MGYA00423039	https://www.ebi.ac.uk/metagenomics/api/v1/analyses/MGYA00423039/file/SRR5869963_MERGED_FASTQ_SSU_OTU.tsv	Root-Host_associated-Human-Skin
SRR6757685	MGYA00380798	https://www.ebi.ac.uk/metagenomics/api/v1/analyses/MGYA00380798/file/SRR6757685_MERGED_FASTQ_SSU_OTU.tsv	Root-Host_associated-Human-Skin
SRR1632995	MGYA00022266	https://www.ebi.ac.uk/metagenomics/api/v1/analyses/MGYA00022266/file/SRR1632995_MERGED_FASTQ_otu.tsv	Root-Host_associated-Human-Skin
SRR994959	MGYA00024346	https://www.ebi.ac.uk/metagenomics/api/v1/analyses/MGYA00024346/file/SRR994959_FASTQ_otu.tsv	Root-Host_associated-Human-Skin
SRR1704436	MGYA00428852	https://www.ebi.ac.uk/metagenomics/api/v1/analyses/MGYA00428852/file/SRR1704436_FASTQ_SSU_OTU.tsv	Root-Host_associated-Human-Skin

SRR5869170	MGYA00429090	https://www.ebi.ac.uk/metagenomics/api/v1/analyses/MGYA00429090/file/SRR5869170_MERGED_FASTQ_SSU_OTU.tsv	Root-Host_associated-Human-Skin
SRR996747	MGYA00023612	https://www.ebi.ac.uk/metagenomics/api/v1/analyses/MGYA00023612/file/SRR996747_FASTQ_otu.tsv	Root-Host_associated-Human-Skin
SRR5869449	MGYA00418725	https://www.ebi.ac.uk/metagenomics/api/v1/analyses/MGYA00418725/file/SRR5869449_MERGED_FASTQ_SSU_OTU.tsv	Root-Host_associated-Human-Skin
SRR920198	MGYA00023261	https://www.ebi.ac.uk/metagenomics/api/v1/analyses/MGYA00023261/file/SRR920198_FASTQ_otu.tsv	Root-Host_associated-Human-Skin
SRR1633139	MGYA00024880	https://www.ebi.ac.uk/metagenomics/api/v1/analyses/MGYA00024880/file/SRR1633139_MERGED_FASTQ_otu.tsv	Root-Host_associated-Human-Skin
SRR5868668	MGYA00423069	https://www.ebi.ac.uk/metagenomics/api/v1/analyses/MGYA00423069/file/SRR5868668_MERGED_FASTQ_SSU_OTU.tsv	Root-Host_associated-Human-Skin
SRR996326	MGYA00022947	https://www.ebi.ac.uk/metagenomics/api/v1/analyses/MGYA00022947/file/SRR996326_FASTQ_otu.tsv	Root-Host_associated-Human-Skin
SRR995594	MGYA00025202	https://www.ebi.ac.uk/metagenomics/api/v1/analyses/MGYA00025202/file/SRR995594_FASTQ_otu.tsv	Root-Host_associated-Human-Skin
ERR442047	MGYA00385215	https://www.ebi.ac.uk/metagenomics/api/v1/analyses/MGYA00385215/file/ERR442047_FASTQ_SSU_OTU.tsv	Root-Host_associated-Human-Skin
SRR5870243	MGYA00416927	https://www.ebi.ac.uk/metagenomics/api/v1/analyses/MGYA00416927/file/SRR5870243_MERGED_FASTQ_SSU_OTU.tsv	Root-Host_associated-Human-Skin
SRR994473	MGYA00022252	https://www.ebi.ac.uk/metagenomics/api/v1/analyses/MGYA00022252/file/SRR994473_FASTQ_otu.tsv	Root-Host_associated-Human-Skin
SRR994801	MGYA00023027	https://www.ebi.ac.uk/metagenomics/api/v1/analyses/MGYA00023027/file/SRR994801_FASTQ_otu.tsv	Root-Host_associated-Human-Skin

SRR1923939	MGYA00028264	https://www.ebi.ac.uk/metagenomics/api/v1/analyses/MGYA00028264/file/SRR1923939_FASTQ_otu.tsv	Root-Host_associated-Human-Skin
SRR995545	MGYA00022747	https://www.ebi.ac.uk/metagenomics/api/v1/analyses/MGYA00022747/file/SRR995545_FASTQ_otu.tsv	Root-Host_associated-Human-Skin
ERR1760134	MGYA00140600	https://www.ebi.ac.uk/metagenomics/api/v1/analyses/MGYA00140600/file/ERR1760134_MERGED_FASTQ_SSU_OTU.tsv	Root-Host_associated-Human-Skin
SRR995124	MGYA00023921	https://www.ebi.ac.uk/metagenomics/api/v1/analyses/MGYA00023921/file/SRR995124_FASTQ_otu.tsv	Root-Host_associated-Human-Skin
ERR1960678	MGYA00130902	https://www.ebi.ac.uk/metagenomics/api/v1/analyses/MGYA00130902/file/ERR1960678_MERGED_FASTQ_otu.tsv	Root-Host_associated-Human-Skin
ERR443418	MGYA00416277	https://www.ebi.ac.uk/metagenomics/api/v1/analyses/MGYA00416277/file/ERR443418_FASTQ_SSU_OTU.tsv	Root-Host_associated-Human-Skin
SRR919632	MGYA00023765	https://www.ebi.ac.uk/metagenomics/api/v1/analyses/MGYA00023765/file/SRR919632_FASTQ_otu.tsv	Root-Host_associated-Human-Skin
ERR441990	MGYA00385211	https://www.ebi.ac.uk/metagenomics/api/v1/analyses/MGYA00385211/file/ERR441990_FASTQ_SSU_OTU.tsv	Root-Host_associated-Human-Skin
SRR994586	MGYA00022930	https://www.ebi.ac.uk/metagenomics/api/v1/analyses/MGYA00022930/file/SRR994586_FASTQ_otu.tsv	Root-Host_associated-Human-Skin
SRR996574	MGYA00022419	https://www.ebi.ac.uk/metagenomics/api/v1/analyses/MGYA00022419/file/SRR996574_FASTQ_otu.tsv	Root-Host_associated-Human-Skin
SRR1950689	MGYA00376856	https://www.ebi.ac.uk/metagenomics/api/v1/analyses/MGYA00376856/file/SRR1950689_MERGED_FASTQ_SSU_OTU.tsv	Root-Host_associated-Human-Skin
SRR996906	MGYA00025322	https://www.ebi.ac.uk/metagenomics/api/v1/analyses/MGYA00025322/file/SRR996906_FASTQ_otu.tsv	Root-Host_associated-Human-Skin

SRR995000	MGYA00024147	https://www.ebi.ac.uk/metagenomics/api/v1/analyses/MGYA00024147/file/SRR995000_FASTQ_otu.tsv	Root-Host_associated-Human-Skin
ERR1760010	MGYA00140372	https://www.ebi.ac.uk/metagenomics/api/v1/analyses/MGYA00140372/file/ERR1760010_MERGED_FASTQ_SSU_OTU.tsv	Root-Host_associated-Human-Skin
SRR5870070	MGYA00416420	https://www.ebi.ac.uk/metagenomics/api/v1/analyses/MGYA00416420/file/SRR5870070_MERGED_FASTQ_SSU_OTU.tsv	Root-Host_associated-Human-Skin
SRR6757628	MGYA00380745	https://www.ebi.ac.uk/metagenomics/api/v1/analyses/MGYA00380745/file/SRR6757628_MERGED_FASTQ_SSU_OTU.tsv	Root-Host_associated-Human-Skin
SRR5868829	MGYA00427140	https://www.ebi.ac.uk/metagenomics/api/v1/analyses/MGYA00427140/file/SRR5868829_MERGED_FASTQ_SSU_OTU.tsv	Root-Host_associated-Human-Skin
SRR996115	MGYA00025176	https://www.ebi.ac.uk/metagenomics/api/v1/analyses/MGYA00025176/file/SRR996115_FASTQ_otu.tsv	Root-Host_associated-Human-Skin
ERR2538409	MGYA00381416	https://www.ebi.ac.uk/metagenomics/api/v1/analyses/MGYA00381416/file/ERR2538409_FASTQ_SSU_OTU.tsv	Root-Host_associated-Human-Skin
SRR996663	MGYA00024777	https://www.ebi.ac.uk/metagenomics/api/v1/analyses/MGYA00024777/file/SRR996663_FASTQ_otu.tsv	Root-Host_associated-Human-Skin
SRR994691	MGYA00023874	https://www.ebi.ac.uk/metagenomics/api/v1/analyses/MGYA00023874/file/SRR994691_FASTQ_otu.tsv	Root-Host_associated-Human-Skin
SRR996202	MGYA00024174	https://www.ebi.ac.uk/metagenomics/api/v1/analyses/MGYA00024174/file/SRR996202_FASTQ_otu.tsv	Root-Host_associated-Human-Skin
SRR1922559	MGYA00028239	https://www.ebi.ac.uk/metagenomics/api/v1/analyses/MGYA00028239/file/SRR1922559_FASTQ_otu.tsv	Root-Host_associated-Human-Skin
ERR450903	MGYA00421442	https://www.ebi.ac.uk/metagenomics/api/v1/analyses/MGYA00421442/file/ERR450903_FASTQ_SSU_OTU.tsv	Root-Host_associated-Human-Skin

SRR1633145	MGYA00022603	https://www.ebi.ac.uk/metagenomics/api/v1/analyses/MGYA00022603/file/SRR1633145_MERGED_FASTQ_otu.tsv	Root-Host_associated-Human-Skin
ERR442920	MGYA00426820	https://www.ebi.ac.uk/metagenomics/api/v1/analyses/MGYA00426820/file/ERR442920_FASTQ_SSU_OTU.tsv	Root-Host_associated-Human-Skin
SRR1923994	MGYA00028333	https://www.ebi.ac.uk/metagenomics/api/v1/analyses/MGYA00028333/file/SRR1923994_FASTQ_otu.tsv	Root-Host_associated-Human-Skin
SRR920585	MGYA00024251	https://www.ebi.ac.uk/metagenomics/api/v1/analyses/MGYA00024251/file/SRR920585_FASTQ_otu.tsv	Root-Host_associated-Human-Skin
ERR443773	MGYA00415094	https://www.ebi.ac.uk/metagenomics/api/v1/analyses/MGYA00415094/file/ERR443773_FASTQ_SSU_OTU.tsv	Root-Host_associated-Human-Skin
SRR920222	MGYA00023289	https://www.ebi.ac.uk/metagenomics/api/v1/analyses/MGYA00023289/file/SRR920222_FASTQ_otu.tsv	Root-Host_associated-Human-Skin
SRR1631271	MGYA00024732	https://www.ebi.ac.uk/metagenomics/api/v1/analyses/MGYA00024732/file/SRR1631271_MERGED_FASTQ_otu.tsv	Root-Host_associated-Human-Skin
SRR5868614	MGYA00423533	https://www.ebi.ac.uk/metagenomics/api/v1/analyses/MGYA00423533/file/SRR5868614_MERGED_FASTQ_SSU_OTU.tsv	Root-Host_associated-Human-Skin
ERR1759956	MGYA00140548	https://www.ebi.ac.uk/metagenomics/api/v1/analyses/MGYA00140548/file/ERR1759956_MERGED_FASTQ_SSU_OTU.tsv	Root-Host_associated-Human-Skin
SRR1950700	MGYA00376867	https://www.ebi.ac.uk/metagenomics/api/v1/analyses/MGYA00376867/file/SRR1950700_MERGED_FASTQ_SSU_OTU.tsv	Root-Host_associated-Human-Skin
SRR919559	MGYA00024504	https://www.ebi.ac.uk/metagenomics/api/v1/analyses/MGYA00024504/file/SRR919559_FASTQ_otu.tsv	Root-Host_associated-Human-Skin
SRR2005707	MGYA00381338	https://www.ebi.ac.uk/metagenomics/api/v1/analyses/MGYA00381338/file/SRR2005707_MERGED_FASTQ_SSU_OTU.tsv	Root-Host_associated-Human-Skin

SRR5869085	MGYA00416479	https://www.ebi.ac.uk/metagenomics/api/v1/analyses/MGYA00416479/file/SRR5869085_MERGED_FASTQ_SSU_OTU.tsv	Root-Host_associated-Human-Skin
SRR5869722	MGYA00415888	https://www.ebi.ac.uk/metagenomics/api/v1/analyses/MGYA00415888/file/SRR5869722_MERGED_FASTQ_SSU_OTU.tsv	Root-Host_associated-Human-Skin
ERR1759987	MGYA00140411	https://www.ebi.ac.uk/metagenomics/api/v1/analyses/MGYA00140411/file/ERR1759987_MERGED_FASTQ_SSU_OTU.tsv	Root-Host_associated-Human-Skin
SRR1632412	MGYA00023313	https://www.ebi.ac.uk/metagenomics/api/v1/analyses/MGYA00023313/file/SRR1632412_MERGED_FASTQ_otu.tsv	Root-Host_associated-Human-Skin
SRR1632860	MGYA00023344	https://www.ebi.ac.uk/metagenomics/api/v1/analyses/MGYA00023344/file/SRR1632860_MERGED_FASTQ_otu.tsv	Root-Host_associated-Human-Skin
SRR1923945	MGYA00028226	https://www.ebi.ac.uk/metagenomics/api/v1/analyses/MGYA00028226/file/SRR1923945_FASTQ_otu.tsv	Root-Host_associated-Human-Skin
SRR994718	MGYA00025094	https://www.ebi.ac.uk/metagenomics/api/v1/analyses/MGYA00025094/file/SRR994718_FASTQ_otu.tsv	Root-Host_associated-Human-Skin
SRR920135	MGYA00024319	https://www.ebi.ac.uk/metagenomics/api/v1/analyses/MGYA00024319/file/SRR920135_FASTQ_otu.tsv	Root-Host_associated-Human-Skin
SRR920692	MGYA00024838	https://www.ebi.ac.uk/metagenomics/api/v1/analyses/MGYA00024838/file/SRR920692_FASTQ_otu.tsv	Root-Host_associated-Human-Skin
SRR1923885	MGYA00028346	https://www.ebi.ac.uk/metagenomics/api/v1/analyses/MGYA00028346/file/SRR1923885_FASTQ_otu.tsv	Root-Host_associated-Human-Skin
SRR1631360	MGYA00022583	https://www.ebi.ac.uk/metagenomics/api/v1/analyses/MGYA00022583/file/SRR1631360_MERGED_FASTQ_otu.tsv	Root-Host_associated-Human-Skin
SRR1704403	MGYA00428863	https://www.ebi.ac.uk/metagenomics/api/v1/analyses/MGYA00428863/file/SRR1704403_FASTQ_SSU_OTU.tsv	Root-Host_associated-Human-Skin

SRR1623167	MGYA00023490	https://www.ebi.ac.uk/metagenomics/api/v1/analyses/MGYA00023490/file/SRR1623167_MERGED_FASTQ_otu.tsv	Root-Host_associated-Human-Skin
SRR1630952	MGYA00024167	https://www.ebi.ac.uk/metagenomics/api/v1/analyses/MGYA00024167/file/SRR1630952_MERGED_FASTQ_otu.tsv	Root-Host_associated-Human-Skin
SRR5869145	MGYA00429129	https://www.ebi.ac.uk/metagenomics/api/v1/analyses/MGYA00429129/file/SRR5869145_MERGED_FASTQ_SSU_OTU.tsv	Root-Host_associated-Human-Skin
ERR1701267	MGYA00084251	https://www.ebi.ac.uk/metagenomics/api/v1/analyses/MGYA00084251/file/ERR1701267_FASTQ_otu.tsv	Root-Host_associated-Human-Skin
ERR450812	MGYA00413722	https://www.ebi.ac.uk/metagenomics/api/v1/analyses/MGYA00413722/file/ERR450812_FASTQ_SSU_OTU.tsv	Root-Host_associated-Human-Skin
ERR443203	MGYA00415166	https://www.ebi.ac.uk/metagenomics/api/v1/analyses/MGYA00415166/file/ERR443203_FASTQ_SSU_OTU.tsv	Root-Host_associated-Human-Skin
ERR1760088	MGYA00140487	https://www.ebi.ac.uk/metagenomics/api/v1/analyses/MGYA00140487/file/ERR1760088_MERGED_FASTQ_SSU_OTU.tsv	Root-Host_associated-Human-Skin
SRR1622893	MGYA00024764	https://www.ebi.ac.uk/metagenomics/api/v1/analyses/MGYA00024764/file/SRR1622893_MERGED_FASTQ_otu.tsv	Root-Host_associated-Human-Skin
ERR2538440	MGYA00381447	https://www.ebi.ac.uk/metagenomics/api/v1/analyses/MGYA00381447/file/ERR2538440_FASTQ_SSU_OTU.tsv	Root-Host_associated-Human-Skin
SRR6757661	MGYA00380775	https://www.ebi.ac.uk/metagenomics/api/v1/analyses/MGYA00380775/file/SRR6757661_MERGED_FASTQ_SSU_OTU.tsv	Root-Host_associated-Human-Skin
ERR2538491	MGYA00381498	https://www.ebi.ac.uk/metagenomics/api/v1/analyses/MGYA00381498/file/ERR2538491_FASTQ_SSU_OTU.tsv	Root-Host_associated-Human-Skin
SRR5869252	MGYA00419723	https://www.ebi.ac.uk/metagenomics/api/v1/analyses/MGYA00419723/file/SRR5869252_MERGED_FASTQ_SSU_OTU.tsv	Root-Host_associated-Human-Skin

ERR1760059	MGYA00140453	https://www.ebi.ac.uk/metagenomics/api/v1/analyses/MGYA00140453/file/ERR1760059_MERGED_FASTQ_SSU_OTU.tsv	Root-Host_associated-Human-Skin
SRR995049	MGYA00023700	https://www.ebi.ac.uk/metagenomics/api/v1/analyses/MGYA00023700/file/SRR995049_FASTQ_otu.tsv	Root-Host_associated-Human-Skin
ERR1960715	MGYA00130796	https://www.ebi.ac.uk/metagenomics/api/v1/analyses/MGYA00130796/file/ERR1960715_MERGED_FASTQ_otu.tsv	Root-Host_associated-Human-Skin
SRR5869633	MGYA00419209	https://www.ebi.ac.uk/metagenomics/api/v1/analyses/MGYA00419209/file/SRR5869633_MERGED_FASTQ_SSU_OTU.tsv	Root-Host_associated-Human-Skin
ERR1759896	MGYA00140479	https://www.ebi.ac.uk/metagenomics/api/v1/analyses/MGYA00140479/file/ERR1759896_MERGED_FASTQ_SSU_OTU.tsv	Root-Host_associated-Human-Skin
SRR1704375	MGYA00420183	https://www.ebi.ac.uk/metagenomics/api/v1/analyses/MGYA00420183/file/SRR1704375_FASTQ_SSU_OTU.tsv	Root-Host_associated-Human-Skin
ERR450777	MGYA00420166	https://www.ebi.ac.uk/metagenomics/api/v1/analyses/MGYA00420166/file/ERR450777_FASTQ_SSU_OTU.tsv	Root-Host_associated-Human-Skin
ERR1760101	MGYA00140423	https://www.ebi.ac.uk/metagenomics/api/v1/analyses/MGYA00140423/file/ERR1760101_MERGED_FASTQ_SSU_OTU.tsv	Root-Host_associated-Human-Skin
SRR994751	MGYA00023242	https://www.ebi.ac.uk/metagenomics/api/v1/analyses/MGYA00023242/file/SRR994751_FASTQ_otu.tsv	Root-Host_associated-Human-Skin
SRR1631688	MGYA00024824	https://www.ebi.ac.uk/metagenomics/api/v1/analyses/MGYA00024824/file/SRR1631688_MERGED_FASTQ_otu.tsv	Root-Host_associated-Human-Skin
SRR996004	MGYA00023515	https://www.ebi.ac.uk/metagenomics/api/v1/analyses/MGYA00023515/file/SRR996004_FASTQ_otu.tsv	Root-Host_associated-Human-Skin
SRR995902	MGYA00023836	https://www.ebi.ac.uk/metagenomics/api/v1/analyses/MGYA00023836/file/SRR995902_FASTQ_otu.tsv	Root-Host_associated-Human-Skin

SRR996817	MGYA00023628	https://www.ebi.ac.uk/metagenomics/api/v1/analyses/MGYA00023628/file/SRR996817_FASTQ_otu.tsv	Root-Host_associated-Human-Skin
SRR1950749	MGYA00376916	https://www.ebi.ac.uk/metagenomics/api/v1/analyses/MGYA00376916/file/SRR1950749_MERGED_FASTQ_SSU_OTU.tsv	Root-Host_associated-Human-Skin
SRR994834	MGYA00024584	https://www.ebi.ac.uk/metagenomics/api/v1/analyses/MGYA00024584/file/SRR994834_FASTQ_otu.tsv	Root-Host_associated-Human-Skin
ERR441974	MGYA00385252	https://www.ebi.ac.uk/metagenomics/api/v1/analyses/MGYA00385252/file/ERR441974_FASTQ_SSU_OTU.tsv	Root-Host_associated-Human-Skin
ERR2538518	MGYA00381525	https://www.ebi.ac.uk/metagenomics/api/v1/analyses/MGYA00381525/file/ERR2538518_FASTQ_SSU_OTU.tsv	Root-Host_associated-Human-Skin
SRR1624827	MGYA00024890	https://www.ebi.ac.uk/metagenomics/api/v1/analyses/MGYA00024890/file/SRR1624827_MERGED_FASTQ_otu.tsv	Root-Host_associated-Human-Skin
SRR995667	MGYA00024842	https://www.ebi.ac.uk/metagenomics/api/v1/analyses/MGYA00024842/file/SRR995667_FASTQ_otu.tsv	Root-Host_associated-Human-Skin
SRR996590	MGYA00023993	https://www.ebi.ac.uk/metagenomics/api/v1/analyses/MGYA00023993/file/SRR996590_FASTQ_otu.tsv	Root-Host_associated-Human-Skin
SRR995322	MGYA00022475	https://www.ebi.ac.uk/metagenomics/api/v1/analyses/MGYA00022475/file/SRR995322_FASTQ_otu.tsv	Root-Host_associated-Human-Skin
SRR994562	MGYA00024431	https://www.ebi.ac.uk/metagenomics/api/v1/analyses/MGYA00024431/file/SRR994562_FASTQ_otu.tsv	Root-Host_associated-Human-Skin
SRR5869558	MGYA00421623	https://www.ebi.ac.uk/metagenomics/api/v1/analyses/MGYA00421623/file/SRR5869558_MERGED_FASTQ_SSU_OTU.tsv	Root-Host_associated-Human-Skin
SRR997016	MGYA00023410	https://www.ebi.ac.uk/metagenomics/api/v1/analyses/MGYA00023410/file/SRR997016_FASTQ_otu.tsv	Root-Host_associated-Human-Skin

SRR1921018	MGYA00028311	https://www.ebi.ac.uk/metagenomics/api/v1/analyses/MGYA00028311/file/SRR1921018_FASTQ_otu.tsv	Root-Host_associated-Human-Skin
ERR1760025	MGYA00140496	https://www.ebi.ac.uk/metagenomics/api/v1/analyses/MGYA00140496/file/ERR1760025_MERGED_FASTQ_SSU_OTU.tsv	Root-Host_associated-Human-Skin
SRR994675	MGYA00023642	https://www.ebi.ac.uk/metagenomics/api/v1/analyses/MGYA00023642/file/SRR994675_FASTQ_otu.tsv	Root-Host_associated-Human-Skin
SRR1924030	MGYA00028268	https://www.ebi.ac.uk/metagenomics/api/v1/analyses/MGYA00028268/file/SRR1924030_FASTQ_otu.tsv	Root-Host_associated-Human-Skin
SRR996120	MGYA00024863	https://www.ebi.ac.uk/metagenomics/api/v1/analyses/MGYA00024863/file/SRR996120_FASTQ_otu.tsv	Root-Host_associated-Human-Skin
SRR995454	MGYA00025305	https://www.ebi.ac.uk/metagenomics/api/v1/analyses/MGYA00025305/file/SRR995454_FASTQ_otu.tsv	Root-Host_associated-Human-Skin
SRR996933	MGYA00022223	https://www.ebi.ac.uk/metagenomics/api/v1/analyses/MGYA00022223/file/SRR996933_FASTQ_otu.tsv	Root-Host_associated-Human-Skin
SRR1704309	MGYA00413832	https://www.ebi.ac.uk/metagenomics/api/v1/analyses/MGYA00413832/file/SRR1704309_FASTQ_SSU_OTU.tsv	Root-Host_associated-Human-Skin
SRR1704197	MGYA00428923	https://www.ebi.ac.uk/metagenomics/api/v1/analyses/MGYA00428923/file/SRR1704197_FASTQ_SSU_OTU.tsv	Root-Host_associated-Human-Skin
SRR5869376	MGYA00423477	https://www.ebi.ac.uk/metagenomics/api/v1/analyses/MGYA00423477/file/SRR5869376_MERGED_FASTQ_SSU_OTU.tsv	Root-Host_associated-Human-Skin
ERR451472	MGYA00414539	https://www.ebi.ac.uk/metagenomics/api/v1/analyses/MGYA00414539/file/ERR451472_FASTQ_SSU_OTU.tsv	Root-Host_associated-Human-Skin
ERR1960631	MGYA00130750	https://www.ebi.ac.uk/metagenomics/api/v1/analyses/MGYA00130750/file/ERR1960631_MERGED_FASTQ_otu.tsv	Root-Host_associated-Human-Skin

SRR996419	MGYA00024305	https://www.ebi.ac.uk/metagenomics/api/v1/analyses/MGYA00024305/file/SRR996419_FASTQ_otu.tsv	Root-Host_associated-Human-Skin
SRR1704251	MGYA00428453	https://www.ebi.ac.uk/metagenomics/api/v1/analyses/MGYA00428453/file/SRR1704251_FASTQ_SSU_OTU.tsv	Root-Host_associated-Human-Skin
SRR1631532	MGYA00023584	https://www.ebi.ac.uk/metagenomics/api/v1/analyses/MGYA00023584/file/SRR1631532_MERGED_FASTQ_otu.tsv	Root-Host_associated-Human-Skin
SRR1632427	MGYA00023752	https://www.ebi.ac.uk/metagenomics/api/v1/analyses/MGYA00023752/file/SRR1632427_MERGED_FASTQ_otu.tsv	Root-Host_associated-Human-Skin
SRR1632855	MGYA00023049	https://www.ebi.ac.uk/metagenomics/api/v1/analyses/MGYA00023049/file/SRR1632855_MERGED_FASTQ_otu.tsv	Root-Host_associated-Human-Skin
SRR1632380	MGYA00022372	https://www.ebi.ac.uk/metagenomics/api/v1/analyses/MGYA00022372/file/SRR1632380_MERGED_FASTQ_otu.tsv	Root-Host_associated-Human-Skin
ERR443030	MGYA00426539	https://www.ebi.ac.uk/metagenomics/api/v1/analyses/MGYA00426539/file/ERR443030_FASTQ_SSU_OTU.tsv	Root-Host_associated-Human-Skin
SRR920913	MGYA00022356	https://www.ebi.ac.uk/metagenomics/api/v1/analyses/MGYA00022356/file/SRR920913_FASTQ_otu.tsv	Root-Host_associated-Human-Skin
SRR994848	MGYA00022601	https://www.ebi.ac.uk/metagenomics/api/v1/analyses/MGYA00022601/file/SRR994848_FASTQ_otu.tsv	Root-Host_associated-Human-Skin
ERR1759963	MGYA00140509	https://www.ebi.ac.uk/metagenomics/api/v1/analyses/MGYA00140509/file/ERR1759963_MERGED_FASTQ_SSU_OTU.tsv	Root-Host_associated-Human-Skin
SRR5868621	MGYA00414893	https://www.ebi.ac.uk/metagenomics/api/v1/analyses/MGYA00414893/file/SRR5868621_MERGED_FASTQ_SSU_OTU.tsv	Root-Host_associated-Human-Skin
SRR1950735	MGYA00376902	https://www.ebi.ac.uk/metagenomics/api/v1/analyses/MGYA00376902/file/SRR1950735_MERGED_FASTQ_SSU_OTU.tsv	Root-Host_associated-Human-Skin

ERR450936	MGYA00416695	https://www.ebi.ac.uk/metagenomics/api/v1/analyses/MGYA00416695/file/ERR450936_FASTQ_SSU_OTU.tsv	Root-Host_associated-Human-Skin
SRR921036	MGYA00023081	https://www.ebi.ac.uk/metagenomics/api/v1/analyses/MGYA00023081/file/SRR921036_FASTQ_otu.tsv	Root-Host_associated-Human-Skin
SRR1633170	MGYA00025262	https://www.ebi.ac.uk/metagenomics/api/v1/analyses/MGYA00025262/file/SRR1633170_MERGED_FASTQ_otu.tsv	Root-Host_associated-Human-Skin
SRR5869400	MGYA00419691	https://www.ebi.ac.uk/metagenomics/api/v1/analyses/MGYA00419691/file/SRR5869400_MERGED_FASTQ_SSU_OTU.tsv	Root-Host_associated-Human-Skin
SRR996597	MGYA00023992	https://www.ebi.ac.uk/metagenomics/api/v1/analyses/MGYA00023992/file/SRR996597_FASTQ_otu.tsv	Root-Host_associated-Human-Skin
SRR994565	MGYA00024436	https://www.ebi.ac.uk/metagenomics/api/v1/analyses/MGYA00024436/file/SRR994565_FASTQ_otu.tsv	Root-Host_associated-Human-Skin
SRR994917	MGYA00024745	https://www.ebi.ac.uk/metagenomics/api/v1/analyses/MGYA00024745/file/SRR994917_FASTQ_otu.tsv	Root-Host_associated-Human-Skin
SRR995325	MGYA00022482	https://www.ebi.ac.uk/metagenomics/api/v1/analyses/MGYA00022482/file/SRR995325_FASTQ_otu.tsv	Root-Host_associated-Human-Skin
SRR997011	MGYA00023417	https://www.ebi.ac.uk/metagenomics/api/v1/analyses/MGYA00023417/file/SRR997011_FASTQ_otu.tsv	Root-Host_associated-Human-Skin
SRR5870093	MGYA00423032	https://www.ebi.ac.uk/metagenomics/api/v1/analyses/MGYA00423032/file/SRR5870093_MERGED_FASTQ_SSU_OTU.tsv	Root-Host_associated-Human-Skin
SRR920729	MGYA00023743	https://www.ebi.ac.uk/metagenomics/api/v1/analyses/MGYA00023743/file/SRR920729_FASTQ_otu.tsv	Root-Host_associated-Human-Skin
ERR443278	MGYA00426065	https://www.ebi.ac.uk/metagenomics/api/v1/analyses/MGYA00426065/file/ERR443278_FASTQ_SSU_OTU.tsv	Root-Host_associated-Human-Skin

SRR994672	MGYA00023637	https://www.ebi.ac.uk/metagenomics/api/v1/analyses/MGYA00023637/file/SRR994672_FASTQ_otu.tsv	Root-Host_associated-Human-Skin
ERR1760022	MGYA00140444	https://www.ebi.ac.uk/metagenomics/api/v1/analyses/MGYA00140444/file/ERR1760022_MERGED_FASTQ_SSU_OTU.tsv	Root-Host_associated-Human-Skin
SRR1622839	MGYA00023712	https://www.ebi.ac.uk/metagenomics/api/v1/analyses/MGYA00023712/file/SRR1622839_MERGED_FASTQ_otu.tsv	Root-Host_associated-Human-Skin
SRR996127	MGYA00024903	https://www.ebi.ac.uk/metagenomics/api/v1/analyses/MGYA00024903/file/SRR996127_FASTQ_otu.tsv	Root-Host_associated-Human-Skin
SRR5869229	MGYA00418076	https://www.ebi.ac.uk/metagenomics/api/v1/analyses/MGYA00418076/file/SRR5869229_MERGED_FASTQ_SSU_OTU.tsv	Root-Host_associated-Human-Skin
SRR1924037	MGYA00028357	https://www.ebi.ac.uk/metagenomics/api/v1/analyses/MGYA00028357/file/SRR1924037_FASTQ_otu.tsv	Root-Host_associated-Human-Skin
SRR996934	MGYA00022228	https://www.ebi.ac.uk/metagenomics/api/v1/analyses/MGYA00022228/file/SRR996934_FASTQ_otu.tsv	Root-Host_associated-Human-Skin
SRR1704637	MGYA00426911	https://www.ebi.ac.uk/metagenomics/api/v1/analyses/MGYA00426911/file/SRR1704637_FASTQ_SSU_OTU.tsv	Root-Host_associated-Human-Skin
ERR443824	MGYA00428372	https://www.ebi.ac.uk/metagenomics/api/v1/analyses/MGYA00428372/file/ERR443824_FASTQ_SSU_OTU.tsv	Root-Host_associated-Human-Skin
ERR443456	MGYA00415151	https://www.ebi.ac.uk/metagenomics/api/v1/analyses/MGYA00415151/file/ERR443456_FASTQ_SSU_OTU.tsv	Root-Host_associated-Human-Skin
ERR1960636	MGYA00130721	https://www.ebi.ac.uk/metagenomics/api/v1/analyses/MGYA00130721/file/ERR1960636_MERGED_FASTQ_otu.tsv	Root-Host_associated-Human-Skin
SRR1631292	MGYA00022886	https://www.ebi.ac.uk/metagenomics/api/v1/analyses/MGYA00022886/file/SRR1631292_MERGED_FASTQ_otu.tsv	Root-Host_associated-Human-Skin

SRR1631535	MGYA00023579	https://www.ebi.ac.uk/metagenomics/api/v1/analyses/MGYA00023579/file/SRR1631535_MERGED_FASTQ_otu.tsv	Root-Host_associated-Human-Skin
SRR1632387	MGYA00022375	https://www.ebi.ac.uk/metagenomics/api/v1/analyses/MGYA00022375/file/SRR1632387_MERGED_FASTQ_otu.tsv	Root-Host_associated-Human-Skin
SRR920914	MGYA00022359	https://www.ebi.ac.uk/metagenomics/api/v1/analyses/MGYA00022359/file/SRR920914_FASTQ_otu.tsv	Root-Host_associated-Human-Skin
ERR443790	MGYA00428384	https://www.ebi.ac.uk/metagenomics/api/v1/analyses/MGYA00428384/file/ERR443790_FASTQ_SSU_OTU.tsv	Root-Host_associated-Human-Skin
SRR1632852	MGYA00023050	https://www.ebi.ac.uk/metagenomics/api/v1/analyses/MGYA00023050/file/SRR1632852_MERGED_FASTQ_otu.tsv	Root-Host_associated-Human-Skin
SRR1632420	MGYA00023758	https://www.ebi.ac.uk/metagenomics/api/v1/analyses/MGYA00023758/file/SRR1632420_MERGED_FASTQ_otu.tsv	Root-Host_associated-Human-Skin
ERR442009	MGYA00385158	https://www.ebi.ac.uk/metagenomics/api/v1/analyses/MGYA00385158/file/ERR442009_FASTQ_SSU_OTU.tsv	Root-Host_associated-Human-Skin
SRR1950732	MGYA00376899	https://www.ebi.ac.uk/metagenomics/api/v1/analyses/MGYA00376899/file/SRR1950732_MERGED_FASTQ_SSU_OTU.tsv	Root-Host_associated-Human-Skin
SRR996368	MGYA00022508	https://www.ebi.ac.uk/metagenomics/api/v1/analyses/MGYA00022508/file/SRR996368_FASTQ_otu.tsv	Root-Host_associated-Human-Skin
ERR1759964	MGYA00140544	https://www.ebi.ac.uk/metagenomics/api/v1/analyses/MGYA00140544/file/ERR1759964_MERGED_FASTQ_SSU_OTU.tsv	Root-Host_associated-Human-Skin
SRR1633177	MGYA00022897	https://www.ebi.ac.uk/metagenomics/api/v1/analyses/MGYA00022897/file/SRR1633177_MERGED_FASTQ_otu.tsv	Root-Host_associated-Human-Skin
SRR921031	MGYA00023082	https://www.ebi.ac.uk/metagenomics/api/v1/analyses/MGYA00023082/file/SRR921031_FASTQ_otu.tsv	Root-Host_associated-Human-Skin

SRR1704141	MGYA00427529	https://www.ebi.ac.uk/metagenomics/api/v1/analyses/MGYA00427529/file/SRR1704141_FASTQ_SSU_OTU.tsv	Root-Host_associated-Human-Skin
SRR5869875	MGYA00412475	https://www.ebi.ac.uk/metagenomics/api/v1/analyses/MGYA00412475/file/SRR5869875_MERGED_FASTQ_SSU_OTU.tsv	Root-Host_associated-Human-Skin
SRR996709	MGYA00023166	https://www.ebi.ac.uk/metagenomics/api/v1/analyses/MGYA00023166/file/SRR996709_FASTQ_otu.tsv	Root-Host_associated-Human-Skin
SRR1923882	MGYA00028339	https://www.ebi.ac.uk/metagenomics/api/v1/analyses/MGYA00028339/file/SRR1923882_FASTQ_otu.tsv	Root-Host_associated-Human-Skin
SRR1630955	MGYA00024160	https://www.ebi.ac.uk/metagenomics/api/v1/analyses/MGYA00024160/file/SRR1630955_MERGED_FASTQ_otu.tsv	Root-Host_associated-Human-Skin
SRR5869142	MGYA00417013	https://www.ebi.ac.uk/metagenomics/api/v1/analyses/MGYA00417013/file/SRR5869142_MERGED_FASTQ_SSU_OTU.tsv	Root-Host_associated-Human-Skin
SRR1623160	MGYA00023483	https://www.ebi.ac.uk/metagenomics/api/v1/analyses/MGYA00023483/file/SRR1623160_MERGED_FASTQ_otu.tsv	Root-Host_associated-Human-Skin
SRR5868702	MGYA00417584	https://www.ebi.ac.uk/metagenomics/api/v1/analyses/MGYA00417584/file/SRR5868702_MERGED_FASTQ_SSU_OTU.tsv	Root-Host_associated-Human-Skin
SRR1631367	MGYA00022588	https://www.ebi.ac.uk/metagenomics/api/v1/analyses/MGYA00022588/file/SRR1631367_MERGED_FASTQ_otu.tsv	Root-Host_associated-Human-Skin
SRR995738	MGYA00023107	https://www.ebi.ac.uk/metagenomics/api/v1/analyses/MGYA00023107/file/SRR995738_FASTQ_otu.tsv	Root-Host_associated-Human-Skin
ERR1701260	MGYA00084271	https://www.ebi.ac.uk/metagenomics/api/v1/analyses/MGYA00084271/file/ERR1701260_FASTQ_otu.tsv	Root-Host_associated-Human-Skin
SRR5869284	MGYA00415929	https://www.ebi.ac.uk/metagenomics/api/v1/analyses/MGYA00415929/file/SRR5869284_MERGED_FASTQ_SSU_OTU.tsv	Root-Host_associated-Human-Skin

SRR5869951	MGYA00420694	https://www.ebi.ac.uk/metagenomics/api/v1/analyses/MGYA00420694/file/SRR5869951_MERGED_FASTQ_SSU_OTU.tsv	Root-Host_associated-Human-Skin
SRR1622894	MGYA00024771	https://www.ebi.ac.uk/metagenomics/api/v1/analyses/MGYA00024771/file/SRR1622894_MERGED_FASTQ_otu.tsv	Root-Host_associated-Human-Skin
ERR2538447	MGYA00381454	https://www.ebi.ac.uk/metagenomics/api/v1/analyses/MGYA00381454/file/ERR2538447_FASTQ_SSU_OTU.tsv	Root-Host_associated-Human-Skin
ERR2538496	MGYA00381503	https://www.ebi.ac.uk/metagenomics/api/v1/analyses/MGYA00381503/file/ERR2538496_FASTQ_SSU_OTU.tsv	Root-Host_associated-Human-Skin
SRR6757666	MGYA00380779	https://www.ebi.ac.uk/metagenomics/api/v1/analyses/MGYA00380779/file/SRR6757666_MERGED_FASTQ_SSU_OTU.tsv	Root-Host_associated-Human-Skin
ERR1960712	MGYA00130836	https://www.ebi.ac.uk/metagenomics/api/v1/analyses/MGYA00130836/file/ERR1960712_MERGED_FASTQ_otu.tsv	Root-Host_associated-Human-Skin
ERR451551	MGYA00419454	https://www.ebi.ac.uk/metagenomics/api/v1/analyses/MGYA00419454/file/ERR451551_FASTQ_SSU_OTU.tsv	Root-Host_associated-Human-Skin
SRR1704372	MGYA00420184	https://www.ebi.ac.uk/metagenomics/api/v1/analyses/MGYA00420184/file/SRR1704372_FASTQ_SSU_OTU.tsv	Root-Host_associated-Human-Skin
ERR1759891	MGYA00140374	https://www.ebi.ac.uk/metagenomics/api/v1/analyses/MGYA00140374/file/ERR1759891_MERGED_FASTQ_SSU_OTU.tsv	Root-Host_associated-Human-Skin
SRR1923853	MGYA00028293	https://www.ebi.ac.uk/metagenomics/api/v1/analyses/MGYA00028293/file/SRR1923853_FASTQ_otu.tsv	Root-Host_associated-Human-Skin
ERR451130	MGYA00426517	https://www.ebi.ac.uk/metagenomics/api/v1/analyses/MGYA00426517/file/ERR451130_FASTQ_SSU_OTU.tsv	Root-Host_associated-Human-Skin
ERR443113	MGYA00426526	https://www.ebi.ac.uk/metagenomics/api/v1/analyses/MGYA00426526/file/ERR443113_FASTQ_SSU_OTU.tsv	Root-Host_associated-Human-Skin

ERR450770	MGYA00426844	https://www.ebi.ac.uk/metagenomics/api/v1/analyses/MGYA00426844/file/ERR450770_FASTQ_SSU_OTU.tsv	Root-Host_associated-Human-Skin
SRR994756	MGYA00023247	https://www.ebi.ac.uk/metagenomics/api/v1/analyses/MGYA00023247/file/SRR994756_FASTQ_otu.tsv	Root-Host_associated-Human-Skin
ERR1760106	MGYA00140511	https://www.ebi.ac.uk/metagenomics/api/v1/analyses/MGYA00140511/file/ERR1760106_MERGED_FASTQ_SSU_OTU.tsv	Root-Host_associated-Human-Skin
SRR996003	MGYA00023518	https://www.ebi.ac.uk/metagenomics/api/v1/analyses/MGYA00023518/file/SRR996003_FASTQ_otu.tsv	Root-Host_associated-Human-Skin
SRR995905	MGYA00023843	https://www.ebi.ac.uk/metagenomics/api/v1/analyses/MGYA00023843/file/SRR995905_FASTQ_otu.tsv	Root-Host_associated-Human-Skin
SRR996810	MGYA00023631	https://www.ebi.ac.uk/metagenomics/api/v1/analyses/MGYA00023631/file/SRR996810_FASTQ_otu.tsv	Root-Host_associated-Human-Skin
ERR1759918	MGYA00140586	https://www.ebi.ac.uk/metagenomics/api/v1/analyses/MGYA00140586/file/ERR1759918_MERGED_FASTQ_SSU_OTU.tsv	Root-Host_associated-Human-Skin
ERR441973	MGYA00385232	https://www.ebi.ac.uk/metagenomics/api/v1/analyses/MGYA00385232/file/ERR441973_FASTQ_SSU_OTU.tsv	Root-Host_associated-Human-Skin
SRR1624820	MGYA00024883	https://www.ebi.ac.uk/metagenomics/api/v1/analyses/MGYA00024883/file/SRR1624820_MERGED_FASTQ_otu.tsv	Root-Host_associated-Human-Skin
ERR451193	MGYA00426077	https://www.ebi.ac.uk/metagenomics/api/v1/analyses/MGYA00426077/file/ERR451193_FASTQ_SSU_OTU.tsv	Root-Host_associated-Human-Skin
ERR1960805	MGYA00130717	https://www.ebi.ac.uk/metagenomics/api/v1/analyses/MGYA00130717/file/ERR1960805_MERGED_FASTQ_otu.tsv	Root-Host_associated-Human-Skin
SRR1922565	MGYA00028307	https://www.ebi.ac.uk/metagenomics/api/v1/analyses/MGYA00028307/file/SRR1922565_FASTQ_otu.tsv	Root-Host_associated-Human-Skin

ERR443617	MGYA00426506	https://www.ebi.ac.uk/metagenomics/api/v1/analyses/MGYA00426506/file/ERR443617_FASTQ_SSU_OTU.tsv	Root-Host_associated-Human-Skin
SRR994919	MGYA00024743	https://www.ebi.ac.uk/metagenomics/api/v1/analyses/MGYA00024743/file/SRR994919_FASTQ_otu.tsv	Root-Host_associated-Human-Skin
SRR996599	MGYA00023998	https://www.ebi.ac.uk/metagenomics/api/v1/analyses/MGYA00023998/file/SRR996599_FASTQ_otu.tsv	Root-Host_associated-Human-Skin
SRR5869697	MGYA00424992	https://www.ebi.ac.uk/metagenomics/api/v1/analyses/MGYA00424992/file/SRR5869697_MERGED_FASTQ_SSU_OTU.tsv	Root-Host_associated-Human-Skin
ERR1701212	MGYA00084257	https://www.ebi.ac.uk/metagenomics/api/v1/analyses/MGYA00084257/file/ERR1701212_FASTQ_otu.tsv	Root-Host_associated-Human-Skin
ERR443276	MGYA00416293	https://www.ebi.ac.uk/metagenomics/api/v1/analyses/MGYA00416293/file/ERR443276_FASTQ_SSU_OTU.tsv	Root-Host_associated-Human-Skin
ERR450867	MGYA00415188	https://www.ebi.ac.uk/metagenomics/api/v1/analyses/MGYA00415188/file/ERR450867_FASTQ_SSU_OTU.tsv	Root-Host_associated-Human-Skin
SRR920727	MGYA00023745	https://www.ebi.ac.uk/metagenomics/api/v1/analyses/MGYA00023745/file/SRR920727_FASTQ_otu.tsv	Root-Host_associated-Human-Skin
ERR451255	MGYA00414278	https://www.ebi.ac.uk/metagenomics/api/v1/analyses/MGYA00414278/file/ERR451255_FASTQ_SSU_OTU.tsv	Root-Host_associated-Human-Skin
SRR5869551	MGYA00414393	https://www.ebi.ac.uk/metagenomics/api/v1/analyses/MGYA00414393/file/SRR5869551_MERGED_FASTQ_SSU_OTU.tsv	Root-Host_associated-Human-Skin
ERR2538435	MGYA00381442	https://www.ebi.ac.uk/metagenomics/api/v1/analyses/MGYA00381442/file/ERR2538435_FASTQ_SSU_OTU.tsv	Root-Host_associated-Human-Skin
ERR2538392	MGYA00381399	https://www.ebi.ac.uk/metagenomics/api/v1/analyses/MGYA00381399/file/ERR2538392_FASTQ_SSU_OTU.tsv	Root-Host_associated-Human-Skin

SRR1924039	MGYA00028355	https://www.ebi.ac.uk/metagenomics/api/v1/analyses/MGYA00028355/file/SRR1924039_FASTQ_otu.tsv	Root-Host_associated-Human-Skin
SRR6757614	MGYA00380732	https://www.ebi.ac.uk/metagenomics/api/v1/analyses/MGYA00380732/file/SRR6757614_MERGED_FASTQ_SSU_OTU.tsv	Root-Host_associated-Human-Skin
SRR1631002	MGYA00024601	https://www.ebi.ac.uk/metagenomics/api/v1/analyses/MGYA00024601/file/SRR1631002_MERGED_FASTQ_otu.tsv	Root-Host_associated-Human-Skin
SRR1622837	MGYA00023717	https://www.ebi.ac.uk/metagenomics/api/v1/analyses/MGYA00023717/file/SRR1622837_MERGED_FASTQ_otu.tsv	Root-Host_associated-Human-Skin
SRR1923795	MGYA00028396	https://www.ebi.ac.uk/metagenomics/api/v1/analyses/MGYA00028396/file/SRR1923795_FASTQ_otu.tsv	Root-Host_associated-Human-Skin
ERR451523	MGYA00413654	https://www.ebi.ac.uk/metagenomics/api/v1/analyses/MGYA00413654/file/ERR451523_FASTQ_SSU_OTU.tsv	Root-Host_associated-Human-Skin
ERR1960760	MGYA00130889	https://www.ebi.ac.uk/metagenomics/api/v1/analyses/MGYA00130889/file/ERR1960760_MERGED_FASTQ_otu.tsv	Root-Host_associated-Human-Skin
SRR2005663	MGYA00381321	https://www.ebi.ac.uk/metagenomics/api/v1/analyses/MGYA00381321/file/SRR2005663_MERGED_FASTQ_SSU_OTU.tsv	Root-Host_associated-Human-Skin
SRR5869646	MGYA00419206	https://www.ebi.ac.uk/metagenomics/api/v1/analyses/MGYA00419206/file/SRR5869646_MERGED_FASTQ_SSU_OTU.tsv	Root-Host_associated-Human-Skin
ERR443161	MGYA00422947	https://www.ebi.ac.uk/metagenomics/api/v1/analyses/MGYA00422947/file/ERR443161_FASTQ_SSU_OTU.tsv	Root-Host_associated-Human-Skin
SRR995164	MGYA00024325	https://www.ebi.ac.uk/metagenomics/api/v1/analyses/MGYA00024325/file/SRR995164_FASTQ_otu.tsv	Root-Host_associated-Human-Skin
ERR1960638	MGYA00130731	https://www.ebi.ac.uk/metagenomics/api/v1/analyses/MGYA00130731/file/ERR1960638_MERGED_FASTQ_otu.tsv	Root-Host_associated-Human-Skin

ERR443039	MGYA00416692	https://www.ebi.ac.uk/metagenomics/api/v1/analyses/MGYA00416692/file/ERR443039_FASTQ_SSU_OTU.tsv	Root-Host_associated-Human-Skin
SRR1632389	MGYA00022369	https://www.ebi.ac.uk/metagenomics/api/v1/analyses/MGYA00022369/file/SRR1632389_MERGED_FASTQ_otu.tsv	Root-Host_associated-Human-Skin
SRR995505	MGYA00023173	https://www.ebi.ac.uk/metagenomics/api/v1/analyses/MGYA00023173/file/SRR995505_FASTQ_otu.tsv	Root-Host_associated-Human-Skin
SRR996410	MGYA00024298	https://www.ebi.ac.uk/metagenomics/api/v1/analyses/MGYA00024298/file/SRR996410_FASTQ_otu.tsv	Root-Host_associated-Human-Skin
SRR1704289	MGYA00413102	https://www.ebi.ac.uk/metagenomics/api/v1/analyses/MGYA00413102/file/SRR1704289_FASTQ_SSU_OTU.tsv	Root-Host_associated-Human-Skin
SRR996366	MGYA00022499	https://www.ebi.ac.uk/metagenomics/api/v1/analyses/MGYA00022499/file/SRR996366_FASTQ_otu.tsv	Root-Host_associated-Human-Skin
ERR442007	MGYA00385247	https://www.ebi.ac.uk/metagenomics/api/v1/analyses/MGYA00385247/file/ERR442007_FASTQ_SSU_OTU.tsv	Root-Host_associated-Human-Skin
SRR995273	MGYA00025065	https://www.ebi.ac.uk/metagenomics/api/v1/analyses/MGYA00025065/file/SRR995273_FASTQ_otu.tsv	Root-Host_associated-Human-Skin
SRR994841	MGYA00022610	https://www.ebi.ac.uk/metagenomics/api/v1/analyses/MGYA00022610/file/SRR994841_FASTQ_otu.tsv	Root-Host_associated-Human-Skin
SRR996707	MGYA00022338	https://www.ebi.ac.uk/metagenomics/api/v1/analyses/MGYA00022338/file/SRR996707_FASTQ_otu.tsv	Root-Host_associated-Human-Skin
ERR443489	MGYA00421401	https://www.ebi.ac.uk/metagenomics/api/v1/analyses/MGYA00421401/file/ERR443489_FASTQ_SSU_OTU.tsv	Root-Host_associated-Human-Skin
ERR1551322	MGYA00069436	https://www.ebi.ac.uk/metagenomics/api/v1/analyses/MGYA00069436/file/ERR1551322_MERGED_FASTQ_otu.tsv	Root-Host_associated-Human-Skin

SRR1631369	MGYA00022580	https://www.ebi.ac.uk/metagenomics/api/v1/analyses/MGYA00022580/file/SRR1631369_MERGED_FASTQ_otu.tsv	Root-Host_associated-Human-Skin
SRR996242	MGYA00023706	https://www.ebi.ac.uk/metagenomics/api/v1/analyses/MGYA00023706/file/SRR996242_FASTQ_otu.tsv	Root-Host_associated-Human-Skin
SRR996184	MGYA00022957	https://www.ebi.ac.uk/metagenomics/api/v1/analyses/MGYA00022957/file/SRR996184_FASTQ_otu.tsv	Root-Host_associated-Human-Skin
ERR2538449	MGYA00381456	https://www.ebi.ac.uk/metagenomics/api/v1/analyses/MGYA00381456/file/ERR2538449_FASTQ_SSU_OTU.tsv	Root-Host_associated-Human-Skin
SRR995736	MGYA00023099	https://www.ebi.ac.uk/metagenomics/api/v1/analyses/MGYA00023099/file/SRR995736_FASTQ_otu.tsv	Root-Host_associated-Human-Skin
ERR1760081	MGYA00140395	https://www.ebi.ac.uk/metagenomics/api/v1/analyses/MGYA00140395/file/ERR1760081_MERGED_FASTQ_SSU_OTU.tsv	Root-Host_associated-Human-Skin
SRR995040	MGYA00023699	https://www.ebi.ac.uk/metagenomics/api/v1/analyses/MGYA00023699/file/SRR995040_FASTQ_otu.tsv	Root-Host_associated-Human-Skin
ERR1760050	MGYA00140375	https://www.ebi.ac.uk/metagenomics/api/v1/analyses/MGYA00140375/file/ERR1760050_MERGED_FASTQ_SSU_OTU.tsv	Root-Host_associated-Human-Skin
SRR6757668	MGYA00380781	https://www.ebi.ac.uk/metagenomics/api/v1/analyses/MGYA00380781/file/SRR6757668_MERGED_FASTQ_SSU_OTU.tsv	Root-Host_associated-Human-Skin
ERR2538498	MGYA00381505	https://www.ebi.ac.uk/metagenomics/api/v1/analyses/MGYA00381505/file/ERR2538498_FASTQ_SSU_OTU.tsv	Root-Host_associated-Human-Skin
SRR996534	MGYA00022839	https://www.ebi.ac.uk/metagenomics/api/v1/analyses/MGYA00022839/file/SRR996534_FASTQ_otu.tsv	Root-Host_associated-Human-Skin
SRR1704645	MGYA00415652	https://www.ebi.ac.uk/metagenomics/api/v1/analyses/MGYA00415652/file/SRR1704645_FASTQ_SSU_OTU.tsv	Root-Host_associated-Human-Skin

SRR1631681	MGYA00024829	https://www.ebi.ac.uk/metagenomics/api/v1/analyses/MGYA00024829/file/SRR1631681_MERGED_FASTQ_otu.tsv	Root-Host_associated-Human-Skin
SRR1622913	MGYA00025085	https://www.ebi.ac.uk/metagenomics/api/v1/analyses/MGYA00025085/file/SRR1622913_MERGED_FASTQ_otu.tsv	Root-Host_associated-Human-Skin
SRR921092	MGYA00022424	https://www.ebi.ac.uk/metagenomics/api/v1/analyses/MGYA00022424/file/SRR921092_FASTQ_otu.tsv	Root-Host_associated-Human-Skin
ERR1760108	MGYA00140591	https://www.ebi.ac.uk/metagenomics/api/v1/analyses/MGYA00140591/file/ERR1760108_MERGED_FASTQ_SSU_OTU.tsv	Root-Host_associated-Human-Skin
ERR1960644	MGYA00130898	https://www.ebi.ac.uk/metagenomics/api/v1/analyses/MGYA00130898/file/ERR1960644_MERGED_FASTQ_otu.tsv	Root-Host_associated-Human-Skin
SRR920175	MGYA00024714	https://www.ebi.ac.uk/metagenomics/api/v1/analyses/MGYA00024714/file/SRR920175_FASTQ_otu.tsv	Root-Host_associated-Human-Skin
SRR994758	MGYA00023239	https://www.ebi.ac.uk/metagenomics/api/v1/analyses/MGYA00023239/file/SRR994758_FASTQ_otu.tsv	Root-Host_associated-Human-Skin
SRR1632794	MGYA00023370	https://www.ebi.ac.uk/metagenomics/api/v1/analyses/MGYA00023370/file/SRR1632794_MERGED_FASTQ_otu.tsv	Root-Host_associated-Human-Skin
SRR1950791	MGYA00376958	https://www.ebi.ac.uk/metagenomics/api/v1/analyses/MGYA00376958/file/SRR1950791_MERGED_FASTQ_SSU_OTU.tsv	Root-Host_associated-Human-Skin
SRR5868685	MGYA00417136	https://www.ebi.ac.uk/metagenomics/api/v1/analyses/MGYA00417136/file/SRR5868685_MERGED_FASTQ_SSU_OTU.tsv	Root-Host_associated-Human-Skin
SRR1632452	MGYA00024970	https://www.ebi.ac.uk/metagenomics/api/v1/analyses/MGYA00024970/file/SRR1632452_MERGED_FASTQ_otu.tsv	Root-Host_associated-Human-Skin
SRR1923905	MGYA00028331	https://www.ebi.ac.uk/metagenomics/api/v1/analyses/MGYA00028331/file/SRR1923905_FASTQ_otu.tsv	Root-Host_associated-Human-Skin

ERR451066	MGYA00412638	https://www.ebi.ac.uk/metagenomics/api/v1/analyses/MGYA00412638/file/ERR451066_FASTQ_SSU_OTU.tsv	Root-Host_associated-Human-Skin
SRR1632483	MGYA00022680	https://www.ebi.ac.uk/metagenomics/api/v1/analyses/MGYA00022680/file/SRR1632483_MERGED_FASTQ_otu.tsv	Root-Host_associated-Human-Skin
SRR920262	MGYA00023685	https://www.ebi.ac.uk/metagenomics/api/v1/analyses/MGYA00023685/file/SRR920262_FASTQ_otu.tsv	Root-Host_associated-Human-Skin
SRR1632324	MGYA00024009	https://www.ebi.ac.uk/metagenomics/api/v1/analyses/MGYA00024009/file/SRR1632324_MERGED_FASTQ_otu.tsv	Root-Host_associated-Human-Skin
ERR1759916	MGYA00140360	https://www.ebi.ac.uk/metagenomics/api/v1/analyses/MGYA00140360/file/ERR1759916_MERGED_FASTQ_SSU_OTU.tsv	Root-Host_associated-Human-Skin
SRR1704552	MGYA00417444	https://www.ebi.ac.uk/metagenomics/api/v1/analyses/MGYA00417444/file/SRR1704552_FASTQ_SSU_OTU.tsv	Root-Host_associated-Human-Skin
SRR5868654	MGYA00418793	https://www.ebi.ac.uk/metagenomics/api/v1/analyses/MGYA00418793/file/SRR5868654_MERGED_FASTQ_SSU_OTU.tsv	Root-Host_associated-Human-Skin
SRR1950740	MGYA00376907	https://www.ebi.ac.uk/metagenomics/api/v1/analyses/MGYA00376907/file/SRR1950740_MERGED_FASTQ_SSU_OTU.tsv	Root-Host_associated-Human-Skin
SRR920603	MGYA00022566	https://www.ebi.ac.uk/metagenomics/api/v1/analyses/MGYA00022566/file/SRR920603_FASTQ_otu.tsv	Root-Host_associated-Human-Skin
ERR1960695	MGYA00130771	https://www.ebi.ac.uk/metagenomics/api/v1/analyses/MGYA00130771/file/ERR1960695_MERGED_FASTQ_otu.tsv	Root-Host_associated-Human-Skin
ERR450943	MGYA00422952	https://www.ebi.ac.uk/metagenomics/api/v1/analyses/MGYA00422952/file/ERR450943_FASTQ_SSU_OTU.tsv	Root-Host_associated-Human-Skin
ERR442960	MGYA00422384	https://www.ebi.ac.uk/metagenomics/api/v1/analyses/MGYA00422384/file/ERR442960_FASTQ_SSU_OTU.tsv	Root-Host_associated-Human-Skin

ERR2538511	MGYA00381518	https://www.ebi.ac.uk/metagenomics/api/v1/analyses/MGYA00381518/file/ERR2538511_FASTQ_SSU_OTU.tsv	Root-Host_associated-Human-Skin
SRR1704694	MGYA00426584	https://www.ebi.ac.uk/metagenomics/api/v1/analyses/MGYA00426584/file/SRR1704694_FASTQ_SSU_OTU.tsv	Root-Host_associated-Human-Skin
SRR996245	MGYA00023709	https://www.ebi.ac.uk/metagenomics/api/v1/analyses/MGYA00023709/file/SRR996245_FASTQ_otu.tsv	Root-Host_associated-Human-Skin
SRR1623169	MGYA00023476	https://www.ebi.ac.uk/metagenomics/api/v1/analyses/MGYA00023476/file/SRR1623169_MERGED_FASTQ_otu.tsv	Root-Host_associated-Human-Skin
SRR996183	MGYA00022958	https://www.ebi.ac.uk/metagenomics/api/v1/analyses/MGYA00022958/file/SRR996183_FASTQ_otu.tsv	Root-Host_associated-Human-Skin
ERR1760086	MGYA00140605	https://www.ebi.ac.uk/metagenomics/api/v1/analyses/MGYA00140605/file/ERR1760086_MERGED_FASTQ_SSU_OTU.tsv	Root-Host_associated-Human-Skin
SRR995731	MGYA00023100	https://www.ebi.ac.uk/metagenomics/api/v1/analyses/MGYA00023100/file/SRR995731_FASTQ_otu.tsv	Root-Host_associated-Human-Skin
ERR1701269	MGYA00084258	https://www.ebi.ac.uk/metagenomics/api/v1/analyses/MGYA00084258/file/ERR1701269_FASTQ_otu.tsv	Root-Host_associated-Human-Skin
SRR995047	MGYA00023692	https://www.ebi.ac.uk/metagenomics/api/v1/analyses/MGYA00023692/file/SRR995047_FASTQ_otu.tsv	Root-Host_associated-Human-Skin
ERR1760057	MGYA00140463	https://www.ebi.ac.uk/metagenomics/api/v1/analyses/MGYA00140463/file/ERR1760057_MERGED_FASTQ_SSU_OTU.tsv	Root-Host_associated-Human-Skin
ERR450779	MGYA00426842	https://www.ebi.ac.uk/metagenomics/api/v1/analyses/MGYA00426842/file/ERR450779_FASTQ_SSU_OTU.tsv	Root-Host_associated-Human-Skin
ERR1759898	MGYA00140400	https://www.ebi.ac.uk/metagenomics/api/v1/analyses/MGYA00140400/file/ERR1759898_MERGED_FASTQ_SSU_OTU.tsv	Root-Host_associated-Human-Skin

SRR996533	MGYA00022842	https://www.ebi.ac.uk/metagenomics/api/v1/analyses/MGYA00022842/file/SRR996533_FASTQ_otu.tsv	Root-Host_associated-Human-Skin
SRR1622914	MGYA00025082	https://www.ebi.ac.uk/metagenomics/api/v1/analyses/MGYA00025082/file/SRR1622914_MERGED_FASTQ_otu.tsv	Root-Host_associated-Human-Skin
SRR5869304	MGYA00419707	https://www.ebi.ac.uk/metagenomics/api/v1/analyses/MGYA00419707/file/SRR5869304_MERGED_FASTQ_SSU_OTU.tsv	Root-Host_associated-Human-Skin
SRR1631686	MGYA00024830	https://www.ebi.ac.uk/metagenomics/api/v1/analyses/MGYA00024830/file/SRR1631686_MERGED_FASTQ_otu.tsv	Root-Host_associated-Human-Skin
SRR1704642	MGYA00417439	https://www.ebi.ac.uk/metagenomics/api/v1/analyses/MGYA00417439/file/SRR1704642_FASTQ_SSU_OTU.tsv	Root-Host_associated-Human-Skin
SRR920172	MGYA00024709	https://www.ebi.ac.uk/metagenomics/api/v1/analyses/MGYA00024709/file/SRR920172_FASTQ_otu.tsv	Root-Host_associated-Human-Skin
SRR1632793	MGYA00023235	https://www.ebi.ac.uk/metagenomics/api/v1/analyses/MGYA00023235/file/SRR1632793_MERGED_FASTQ_otu.tsv	Root-Host_associated-Human-Skin
ERR1960643	MGYA00130888	https://www.ebi.ac.uk/metagenomics/api/v1/analyses/MGYA00130888/file/ERR1960643_MERGED_FASTQ_otu.tsv	Root-Host_associated-Human-Skin
ERR451400	MGYA00416658	https://www.ebi.ac.uk/metagenomics/api/v1/analyses/MGYA00416658/file/ERR451400_FASTQ_SSU_OTU.tsv	Root-Host_associated-Human-Skin
SRR921095	MGYA00022429	https://www.ebi.ac.uk/metagenomics/api/v1/analyses/MGYA00022429/file/SRR921095_FASTQ_otu.tsv	Root-Host_associated-Human-Skin
SRR5868682	MGYA00420815	https://www.ebi.ac.uk/metagenomics/api/v1/analyses/MGYA00420815/file/SRR5868682_MERGED_FASTQ_SSU_OTU.tsv	Root-Host_associated-Human-Skin
SRR1704584	MGYA00421471	https://www.ebi.ac.uk/metagenomics/api/v1/analyses/MGYA00421471/file/SRR1704584_FASTQ_SSU_OTU.tsv	Root-Host_associated-Human-Skin

SRR1704223	MGYA00413110	https://www.ebi.ac.uk/metagenomics/api/v1/analyses/MGYA00413110/file/SRR1704223_FASTQ_SSU_OTU.tsv	Root-Host_associated-Human-Skin
SRR996819	MGYA00023624	https://www.ebi.ac.uk/metagenomics/api/v1/analyses/MGYA00023624/file/SRR996819_FASTQ_otu.tsv	Root-Host_associated-Human-Skin
SRR1621012	MGYA00024795	https://www.ebi.ac.uk/metagenomics/api/v1/analyses/MGYA00024795/file/SRR1621012_MERGED_FASTQ_otu.tsv	Root-Host_associated-Human-Skin
SRR1923902	MGYA00028326	https://www.ebi.ac.uk/metagenomics/api/v1/analyses/MGYA00028326/file/SRR1923902_FASTQ_otu.tsv	Root-Host_associated-Human-Skin
SRR1632323	MGYA00023482	https://www.ebi.ac.uk/metagenomics/api/v1/analyses/MGYA00023482/file/SRR1632323_MERGED_FASTQ_otu.tsv	Root-Host_associated-Human-Skin
SRR1632484	MGYA00022675	https://www.ebi.ac.uk/metagenomics/api/v1/analyses/MGYA00022675/file/SRR1632484_MERGED_FASTQ_otu.tsv	Root-Host_associated-Human-Skin
SRR920265	MGYA00023690	https://www.ebi.ac.uk/metagenomics/api/v1/analyses/MGYA00023690/file/SRR920265_FASTQ_otu.tsv	Root-Host_associated-Human-Skin
SRR1950747	MGYA00376914	https://www.ebi.ac.uk/metagenomics/api/v1/analyses/MGYA00376914/file/SRR1950747_MERGED_FASTQ_SSU_OTU.tsv	Root-Host_associated-Human-Skin
SRR1704555	MGYA00422430	https://www.ebi.ac.uk/metagenomics/api/v1/analyses/MGYA00422430/file/SRR1704555_FASTQ_SSU_OTU.tsv	Root-Host_associated-Human-Skin
ERR1759911	MGYA00140460	https://www.ebi.ac.uk/metagenomics/api/v1/analyses/MGYA00140460/file/ERR1759911_MERGED_FASTQ_SSU_OTU.tsv	Root-Host_associated-Human-Skin
SRR1624829	MGYA00024882	https://www.ebi.ac.uk/metagenomics/api/v1/analyses/MGYA00024882/file/SRR1624829_MERGED_FASTQ_otu.tsv	Root-Host_associated-Human-Skin
SRR920604	MGYA00022561	https://www.ebi.ac.uk/metagenomics/api/v1/analyses/MGYA00022561/file/SRR920604_FASTQ_otu.tsv	Root-Host_associated-Human-Skin

ERR1960692	MGYA00130712	https://www.ebi.ac.uk/metagenomics/api/v1/analyses/MGYA00130712/file/ERR1960692_MERGED_FASTQ_otu.tsv	Root-Host_associated-Human-Skin
SRR1704693	MGYA00415209	https://www.ebi.ac.uk/metagenomics/api/v1/analyses/MGYA00415209/file/SRR1704693_FASTQ_SSU_OTU.tsv	Root-Host_associated-Human-Skin
SRR5868595	MGYA00419734	https://www.ebi.ac.uk/metagenomics/api/v1/analyses/MGYA00419734/file/SRR5868595_MERGED_FASTQ_SSU_OTU.tsv	Root-Host_associated-Human-Skin
ERR2538516	MGYA00381523	https://www.ebi.ac.uk/metagenomics/api/v1/analyses/MGYA00381523/file/ERR2538516_FASTQ_SSU_OTU.tsv	Root-Host_associated-Human-Skin
ERR1960802	MGYA00130818	https://www.ebi.ac.uk/metagenomics/api/v1/analyses/MGYA00130818/file/ERR1960802_MERGED_FASTQ_otu.tsv	Root-Host_associated-Human-Skin
SRR5868777	MGYA00417108	https://www.ebi.ac.uk/metagenomics/api/v1/analyses/MGYA00417108/file/SRR5868777_MERGED_FASTQ_SSU_OTU.tsv	Root-Host_associated-Human-Skin
ERR443271	MGYA00415161	https://www.ebi.ac.uk/metagenomics/api/v1/analyses/MGYA00415161/file/ERR443271_FASTQ_SSU_OTU.tsv	Root-Host_associated-Human-Skin
ERR1701215	MGYA00084284	https://www.ebi.ac.uk/metagenomics/api/v1/analyses/MGYA00084284/file/ERR1701215_FASTQ_otu.tsv	Root-Host_associated-Human-Skin
SRR997018	MGYA00023408	https://www.ebi.ac.uk/metagenomics/api/v1/analyses/MGYA00023408/file/SRR997018_FASTQ_otu.tsv	Root-Host_associated-Human-Skin
ERR2538395	MGYA00381402	https://www.ebi.ac.uk/metagenomics/api/v1/analyses/MGYA00381402/file/ERR2538395_FASTQ_SSU_OTU.tsv	Root-Host_associated-Human-Skin
ERR2538432	MGYA00381439	https://www.ebi.ac.uk/metagenomics/api/v1/analyses/MGYA00381439/file/ERR2538432_FASTQ_SSU_OTU.tsv	Root-Host_associated-Human-Skin
SRR6757613	MGYA00380731	https://www.ebi.ac.uk/metagenomics/api/v1/analyses/MGYA00380731/file/SRR6757613_MERGED_FASTQ_SSU_OTU.tsv	Root-Host_associated-Human-Skin

SRR5868812	MGYA00427153	https://www.ebi.ac.uk/metagenomics/api/v1/analyses/MGYA00427153/file/SRR5868812_MERGED_FASTQ_SSU_OTU.tsv	Root-Host_associated-Human-Skin
SRR1631005	MGYA00024596	https://www.ebi.ac.uk/metagenomics/api/v1/analyses/MGYA00024596/file/SRR1631005_MERGED_FASTQ_otu.tsv	Root-Host_associated-Human-Skin
ERR1960767	MGYA00130882	https://www.ebi.ac.uk/metagenomics/api/v1/analyses/MGYA00130882/file/ERR1960767_MERGED_FASTQ_otu.tsv	Root-Host_associated-Human-Skin
ERR443507	MGYA00414540	https://www.ebi.ac.uk/metagenomics/api/v1/analyses/MGYA00414540/file/ERR443507_FASTQ_SSU_OTU.tsv	Root-Host_associated-Human-Skin
SRR1923792	MGYA00028397	https://www.ebi.ac.uk/metagenomics/api/v1/analyses/MGYA00028397/file/SRR1923792_FASTQ_otu.tsv	Root-Host_associated-Human-Skin
SRR1704307	MGYA00426977	https://www.ebi.ac.uk/metagenomics/api/v1/analyses/MGYA00426977/file/SRR1704307_FASTQ_SSU_OTU.tsv	Root-Host_associated-Human-Skin
SRR5869641	MGYA00415904	https://www.ebi.ac.uk/metagenomics/api/v1/analyses/MGYA00415904/file/SRR5869641_MERGED_FASTQ_SSU_OTU.tsv	Root-Host_associated-Human-Skin
SRR2005664	MGYA00381322	https://www.ebi.ac.uk/metagenomics/api/v1/analyses/MGYA00381322/file/SRR2005664_MERGED_FASTQ_SSU_OTU.tsv	Root-Host_associated-Human-Skin
ERR451145	MGYA00426093	https://www.ebi.ac.uk/metagenomics/api/v1/analyses/MGYA00426093/file/ERR451145_FASTQ_SSU_OTU.tsv	Root-Host_associated-Human-Skin
ERR450705	MGYA00423345	https://www.ebi.ac.uk/metagenomics/api/v1/analyses/MGYA00423345/file/ERR450705_FASTQ_SSU_OTU.tsv	Root-Host_associated-Human-Skin
SRR995163	MGYA00024332	https://www.ebi.ac.uk/metagenomics/api/v1/analyses/MGYA00024332/file/SRR995163_FASTQ_otu.tsv	Root-Host_associated-Human-Skin
SRR1704199	MGYA00425334	https://www.ebi.ac.uk/metagenomics/api/v1/analyses/MGYA00425334/file/SRR1704199_FASTQ_SSU_OTU.tsv	Root-Host_associated-Human-Skin

SRR995502	MGYA00023174	https://www.ebi.ac.uk/metagenomics/api/v1/analyses/MGYA00023174/file/SRR995502_FASTQ_otu.tsv	Root-Host_associated-Human-Skin
SRR995970	MGYA00023207	https://www.ebi.ac.uk/metagenomics/api/v1/analyses/MGYA00023207/file/SRR995970_FASTQ_otu.tsv	Root-Host_associated-Human-Skin
SRR1632429	MGYA00023759	https://www.ebi.ac.uk/metagenomics/api/v1/analyses/MGYA00023759/file/SRR1632429_MERGED_FASTQ_otu.tsv	Root-Host_associated-Human-Skin
SRR996417	MGYA00024303	https://www.ebi.ac.uk/metagenomics/api/v1/analyses/MGYA00024303/file/SRR996417_FASTQ_otu.tsv	Root-Host_associated-Human-Skin
SRR996361	MGYA00022504	https://www.ebi.ac.uk/metagenomics/api/v1/analyses/MGYA00022504/file/SRR996361_FASTQ_otu.tsv	Root-Host_associated-Human-Skin
SRR994846	MGYA00022615	https://www.ebi.ac.uk/metagenomics/api/v1/analyses/MGYA00022615/file/SRR994846_FASTQ_otu.tsv	Root-Host_associated-Human-Skin
SRR995274	MGYA00025058	https://www.ebi.ac.uk/metagenomics/api/v1/analyses/MGYA00025058/file/SRR995274_FASTQ_otu.tsv	Root-Host_associated-Human-Skin
ERR442000	MGYA00385153	https://www.ebi.ac.uk/metagenomics/api/v1/analyses/MGYA00385153/file/ERR442000_FASTQ_SSU_OTU.tsv	Root-Host_associated-Human-Skin
SRR996700	MGYA00022328	https://www.ebi.ac.uk/metagenomics/api/v1/analyses/MGYA00022328/file/SRR996700_FASTQ_otu.tsv	Root-Host_associated-Human-Skin
SRR921038	MGYA00023077	https://www.ebi.ac.uk/metagenomics/api/v1/analyses/MGYA00023077/file/SRR921038_FASTQ_otu.tsv	Root-Host_associated-Human-Skin
ERR443329	MGYA00426052	https://www.ebi.ac.uk/metagenomics/api/v1/analyses/MGYA00426052/file/ERR443329_FASTQ_SSU_OTU.tsv	Root-Host_associated-Human-Skin
ERR1551325	MGYA00069431	https://www.ebi.ac.uk/metagenomics/api/v1/analyses/MGYA00069431/file/ERR1551325_MERGED_FASTQ_otu.tsv	Root-Host_associated-Human-Skin

SRR1950756	MGYA00376923	https://www.ebi.ac.uk/metagenomics/api/v1/analyses/MGYA00376923/file/SRR1950756_MERGED_FASTQ_SSU_OTU.tsv	Root-Host_associated-Human-Skin
ERR1759900	MGYA00140465	https://www.ebi.ac.uk/metagenomics/api/v1/analyses/MGYA00140465/file/ERR1759900_MERGED_FASTQ_SSU_OTU.tsv	Root-Host_associated-Human-Skin
SRR1704544	MGYA00421478	https://www.ebi.ac.uk/metagenomics/api/v1/analyses/MGYA00421478/file/SRR1704544_FASTQ_SSU_OTU.tsv	Root-Host_associated-Human-Skin
SRR994459	MGYA00022463	https://www.ebi.ac.uk/metagenomics/api/v1/analyses/MGYA00022463/file/SRR994459_FASTQ_otu.tsv	Root-Host_associated-Human-Skin
SRR1632332	MGYA00024569	https://www.ebi.ac.uk/metagenomics/api/v1/analyses/MGYA00024569/file/SRR1632332_MERGED_FASTQ_otu.tsv	Root-Host_associated-Human-Skin
SRR1704682	MGYA00412706	https://www.ebi.ac.uk/metagenomics/api/v1/analyses/MGYA00412706/file/SRR1704682_FASTQ_SSU_OTU.tsv	Root-Host_associated-Human-Skin
ERR2538507	MGYA00381514	https://www.ebi.ac.uk/metagenomics/api/v1/analyses/MGYA00381514/file/ERR2538507_FASTQ_SSU_OTU.tsv	Root-Host_associated-Human-Skin
SRR921055	MGYA00022859	https://www.ebi.ac.uk/metagenomics/api/v1/analyses/MGYA00022859/file/SRR921055_FASTQ_otu.tsv	Root-Host_associated-Human-Skin
ERR442976	MGYA00413714	https://www.ebi.ac.uk/metagenomics/api/v1/analyses/MGYA00413714/file/ERR442976_FASTQ_SSU_OTU.tsv	Root-Host_associated-Human-Skin
ERR450955	MGYA00420160	https://www.ebi.ac.uk/metagenomics/api/v1/analyses/MGYA00420160/file/ERR450955_FASTQ_SSU_OTU.tsv	Root-Host_associated-Human-Skin
ERR1960683	MGYA00130707	https://www.ebi.ac.uk/metagenomics/api/v1/analyses/MGYA00130707/file/ERR1960683_MERGED_FASTQ_otu.tsv	Root-Host_associated-Human-Skin
ERR1960652	MGYA00130752	https://www.ebi.ac.uk/metagenomics/api/v1/analyses/MGYA00130752/file/ERR1960652_MERGED_FASTQ_otu.tsv	Root-Host_associated-Human-Skin

ERR443395	MGYA00412376	https://www.ebi.ac.uk/metagenomics/api/v1/analyses/MGYA00412376/file/ERR443395_FASTQ_SSU_OTU.tsv	Root-Host_associated-Human-Skin
ERR451411	MGYA00428716	https://www.ebi.ac.uk/metagenomics/api/v1/analyses/MGYA00428716/file/ERR451411_FASTQ_SSU_OTU.tsv	Root-Host_associated-Human-Skin
SRR919618	MGYA00023532	https://www.ebi.ac.uk/metagenomics/api/v1/analyses/MGYA00023532/file/SRR919618_FASTQ_otu.tsv	Root-Host_associated-Human-Skin
SRR1622905	MGYA00024357	https://www.ebi.ac.uk/metagenomics/api/v1/analyses/MGYA00024357/file/SRR1622905_MERGED_FASTQ_otu.tsv	Root-Host_associated-Human-Skin
SRR1923913	MGYA00028301	https://www.ebi.ac.uk/metagenomics/api/v1/analyses/MGYA00028301/file/SRR1923913_FASTQ_otu.tsv	Root-Host_associated-Human-Skin
SRR994488	MGYA00024343	https://www.ebi.ac.uk/metagenomics/api/v1/analyses/MGYA00024343/file/SRR994488_FASTQ_otu.tsv	Root-Host_associated-Human-Skin
SRR1632836	MGYA00022816	https://www.ebi.ac.uk/metagenomics/api/v1/analyses/MGYA00022816/file/SRR1632836_MERGED_FASTQ_otu.tsv	Root-Host_associated-Human-Skin
SRR920970	MGYA00023039	https://www.ebi.ac.uk/metagenomics/api/v1/analyses/MGYA00023039/file/SRR920970_FASTQ_otu.tsv	Root-Host_associated-Human-Skin
ERR443053	MGYA00416691	https://www.ebi.ac.uk/metagenomics/api/v1/analyses/MGYA00416691/file/ERR443053_FASTQ_SSU_OTU.tsv	Root-Host_associated-Human-Skin
SRR1704232	MGYA00423933	https://www.ebi.ac.uk/metagenomics/api/v1/analyses/MGYA00423933/file/SRR1704232_FASTQ_SSU_OTU.tsv	Root-Host_associated-Human-Skin
SRR1631551	MGYA00023804	https://www.ebi.ac.uk/metagenomics/api/v1/analyses/MGYA00023804/file/SRR1631551_MERGED_FASTQ_otu.tsv	Root-Host_associated-Human-Skin
SRR5868693	MGYA00416061	https://www.ebi.ac.uk/metagenomics/api/v1/analyses/MGYA00416061/file/SRR5868693_MERGED_FASTQ_SSU_OTU.tsv	Root-Host_associated-Human-Skin

SRR1950787	MGYA00376954	https://www.ebi.ac.uk/metagenomics/api/v1/analyses/MGYA00376954/file/SRR1950787_MERGED_FASTQ_SSU_OTU.tsv	Root-Host_associated-Human-Skin
SRR996143	MGYA00022524	https://www.ebi.ac.uk/metagenomics/api/v1/analyses/MGYA00022524/file/SRR996143_FASTQ_otu.tsv	Root-Host_associated-Human-Skin
SRR994616	MGYA00023019	https://www.ebi.ac.uk/metagenomics/api/v1/analyses/MGYA00023019/file/SRR994616_FASTQ_otu.tsv	Root-Host_associated-Human-Skin
ERR1760046	MGYA00140366	https://www.ebi.ac.uk/metagenomics/api/v1/analyses/MGYA00140366/file/ERR1760046_MERGED_FASTQ_SSU_OTU.tsv	Root-Host_associated-Human-Skin
ERR1759889	MGYA00140570	https://www.ebi.ac.uk/metagenomics/api/v1/analyses/MGYA00140570/file/ERR1759889_MERGED_FASTQ_SSU_OTU.tsv	Root-Host_associated-Human-Skin
SRR996285	MGYA00023305	https://www.ebi.ac.uk/metagenomics/api/v1/analyses/MGYA00023305/file/SRR996285_FASTQ_otu.tsv	Root-Host_associated-Human-Skin
SRR996950	MGYA00022874	https://www.ebi.ac.uk/metagenomics/api/v1/analyses/MGYA00022874/file/SRR996950_FASTQ_otu.tsv	Root-Host_associated-Human-Skin
SRR995390	MGYA00024761	https://www.ebi.ac.uk/metagenomics/api/v1/analyses/MGYA00024761/file/SRR995390_FASTQ_otu.tsv	Root-Host_associated-Human-Skin
SRR994501	MGYA00023815	https://www.ebi.ac.uk/metagenomics/api/v1/analyses/MGYA00023815/file/SRR994501_FASTQ_otu.tsv	Root-Host_associated-Human-Skin
SRR994973	MGYA00024553	https://www.ebi.ac.uk/metagenomics/api/v1/analyses/MGYA00024553/file/SRR994973_FASTQ_otu.tsv	Root-Host_associated-Human-Skin
SRR995341	MGYA00023123	https://www.ebi.ac.uk/metagenomics/api/v1/analyses/MGYA00023123/file/SRR995341_FASTQ_otu.tsv	Root-Host_associated-Human-Skin
SRR996981	MGYA00024536	https://www.ebi.ac.uk/metagenomics/api/v1/analyses/MGYA00024536/file/SRR996981_FASTQ_otu.tsv	Root-Host_associated-Human-Skin

ERR1760097	MGYA00140484	https://www.ebi.ac.uk/metagenomics/api/v1/analyses/MGYA00140484/file/ERR1760097_MERGED_FASTQ_SSU_OTU.tsv	Root-Host_associated-Human-Skin
SRR995087	MGYA00022720	https://www.ebi.ac.uk/metagenomics/api/v1/analyses/MGYA00022720/file/SRR995087_FASTQ_otu.tsv	Root-Host_associated-Human-Skin
ERR1701278	MGYA00084243	https://www.ebi.ac.uk/metagenomics/api/v1/analyses/MGYA00084243/file/ERR1701278_FASTQ_otu.tsv	Root-Host_associated-Human-Skin
SRR997075	MGYA00023163	https://www.ebi.ac.uk/metagenomics/api/v1/analyses/MGYA00023163/file/SRR997075_FASTQ_otu.tsv	Root-Host_associated-Human-Skin
SRR5869949	MGYA00423465	https://www.ebi.ac.uk/metagenomics/api/v1/analyses/MGYA00423465/file/SRR5869949_MERGED_FASTQ_SSU_OTU.tsv	Root-Host_associated-Human-Skin
ERR981252	MGYA00010235	https://www.ebi.ac.uk/metagenomics/api/v1/analyses/MGYA00010235/file/ERR981252_MERGED_FASTQ_otu.tsv	Root-Host_associated-Human-Skin
SRR920208	MGYA00023055	https://www.ebi.ac.uk/metagenomics/api/v1/analyses/MGYA00023055/file/SRR920208_FASTQ_otu.tsv	Root-Host_associated-Human-Skin
ERR442011	MGYA00385224	https://www.ebi.ac.uk/metagenomics/api/v1/analyses/MGYA00385224/file/ERR442011_FASTQ_SSU_OTU.tsv	Root-Host_associated-Human-Skin
SRR919573	MGYA00024722	https://www.ebi.ac.uk/metagenomics/api/v1/analyses/MGYA00024722/file/SRR919573_FASTQ_otu.tsv	Root-Host_associated-Human-Skin
SRR919901	MGYA00025031	https://www.ebi.ac.uk/metagenomics/api/v1/analyses/MGYA00025031/file/SRR919901_FASTQ_otu.tsv	Root-Host_associated-Human-Skin
SRR1704538	MGYA00428836	https://www.ebi.ac.uk/metagenomics/api/v1/analyses/MGYA00428836/file/SRR1704538_FASTQ_SSU_OTU.tsv	Root-Host_associated-Human-Skin
SRR1624844	MGYA00024295	https://www.ebi.ac.uk/metagenomics/api/v1/analyses/MGYA00024295/file/SRR1624844_MERGED_FASTQ_otu.tsv	Root-Host_associated-Human-Skin

SRR995604	MGYA00025037	https://www.ebi.ac.uk/metagenomics/api/v1/analyses/MGYA00025037/file/SRR995604_FASTQ_otu.tsv	Root-Host_associated-Human-Skin
ERR1551334	MGYA00069427	https://www.ebi.ac.uk/metagenomics/api/v1/analyses/MGYA00069427/file/ERR1551334_MERGED_FASTQ_otu.tsv	Root-Host_associated-Human-Skin
SRR920669	MGYA00022347	https://www.ebi.ac.uk/metagenomics/api/v1/analyses/MGYA00022347/file/SRR920669_FASTQ_otu.tsv	Root-Host_associated-Human-Skin
SRR996711	MGYA00022999	https://www.ebi.ac.uk/metagenomics/api/v1/analyses/MGYA00022999/file/SRR996711_FASTQ_otu.tsv	Root-Host_associated-Human-Skin
SRR996067	MGYA00023321	https://www.ebi.ac.uk/metagenomics/api/v1/analyses/MGYA00023321/file/SRR996067_FASTQ_otu.tsv	Root-Host_associated-Human-Skin
SRR1704188	MGYA00428929	https://www.ebi.ac.uk/metagenomics/api/v1/analyses/MGYA00428929/file/SRR1704188_FASTQ_SSU_OTU.tsv	Root-Host_associated-Human-Skin
SRR994732	MGYA00023891	https://www.ebi.ac.uk/metagenomics/api/v1/analyses/MGYA00023891/file/SRR994732_FASTQ_otu.tsv	Root-Host_associated-Human-Skin
SRR996874	MGYA00023437	https://www.ebi.ac.uk/metagenomics/api/v1/analyses/MGYA00023437/file/SRR996874_FASTQ_otu.tsv	Root-Host_associated-Human-Skin
SRR995961	MGYA00023851	https://www.ebi.ac.uk/metagenomics/api/v1/analyses/MGYA00023851/file/SRR995961_FASTQ_otu.tsv	Root-Host_associated-Human-Skin
SRR994886	MGYA00022184	https://www.ebi.ac.uk/metagenomics/api/v1/analyses/MGYA00022184/file/SRR994886_FASTQ_otu.tsv	Root-Host_associated-Human-Skin
ERR443516	MGYA00428703	https://www.ebi.ac.uk/metagenomics/api/v1/analyses/MGYA00428703/file/ERR443516_FASTQ_SSU_OTU.tsv	Root-Host_associated-Human-Skin
ERR1960776	MGYA00130782	https://www.ebi.ac.uk/metagenomics/api/v1/analyses/MGYA00130782/file/ERR1960776_MERGED_FASTQ_otu.tsv	Root-Host_associated-Human-Skin

SRR1923783	MGYA00028237	https://www.ebi.ac.uk/metagenomics/api/v1/analyses/MGYA00028237/file/SRR1923783_FASTQ_otu.tsv	Root-Host_associated-Human-Skin
ERR2538355	MGYA00381362	https://www.ebi.ac.uk/metagenomics/api/v1/analyses/MGYA00381362/file/ERR2538355_FASTQ_SSU_OTU.tsv	Root-Host_associated-Human-Skin
SRR996698	MGYA00022297	https://www.ebi.ac.uk/metagenomics/api/v1/analyses/MGYA00022297/file/SRR996698_FASTQ_otu.tsv	Root-Host_associated-Human-Skin
SRR1623213	MGYA00023908	https://www.ebi.ac.uk/metagenomics/api/v1/analyses/MGYA00023908/file/SRR1623213_MERGED_FASTQ_otu.tsv	Root-Host_associated-Human-Skin
SRR6757602	MGYA00380721	https://www.ebi.ac.uk/metagenomics/api/v1/analyses/MGYA00380721/file/SRR6757602_MERGED_FASTQ_SSU_OTU.tsv	Root-Host_associated-Human-Skin
SRR5868803	MGYA00417094	https://www.ebi.ac.uk/metagenomics/api/v1/analyses/MGYA00417094/file/SRR5868803_MERGED_FASTQ_SSU_OTU.tsv	Root-Host_associated-Human-Skin
ERR450714	MGYA00425286	https://www.ebi.ac.uk/metagenomics/api/v1/analyses/MGYA00425286/file/ERR450714_FASTQ_SSU_OTU.tsv	Root-Host_associated-Human-Skin
SRR919888	MGYA00024507	https://www.ebi.ac.uk/metagenomics/api/v1/analyses/MGYA00024507/file/SRR919888_FASTQ_otu.tsv	Root-Host_associated-Human-Skin
SRR5869126	MGYA00418098	https://www.ebi.ac.uk/metagenomics/api/v1/analyses/MGYA00418098/file/SRR5869126_MERGED_FASTQ_SSU_OTU.tsv	Root-Host_associated-Human-Skin
SRR996228	MGYA00023969	https://www.ebi.ac.uk/metagenomics/api/v1/analyses/MGYA00023969/file/SRR996228_FASTQ_otu.tsv	Root-Host_associated-Human-Skin
SRR1631303	MGYA00023231	https://www.ebi.ac.uk/metagenomics/api/v1/analyses/MGYA00023231/file/SRR1631303_MERGED_FASTQ_otu.tsv	Root-Host_associated-Human-Skin
SRR5868766	MGYA00426268	https://www.ebi.ac.uk/metagenomics/api/v1/analyses/MGYA00426268/file/SRR5868766_MERGED_FASTQ_SSU_OTU.tsv	Root-Host_associated-Human-Skin

ERR1960813	MGYA00130807	https://www.ebi.ac.uk/metagenomics/api/v1/analyses/MGYA00130807/file/ERR1960813_MERGED_FASTQ_otu.tsv	Root-Host_associated-Human-Skin
ERR2538423	MGYA00381430	https://www.ebi.ac.uk/metagenomics/api/v1/analyses/MGYA00381430/file/ERR2538423_FASTQ_SSU_OTU.tsv	Root-Host_associated-Human-Skin
SRR5869935	MGYA00415311	https://www.ebi.ac.uk/metagenomics/api/v1/analyses/MGYA00415311/file/SRR5869935_MERGED_FASTQ_SSU_OTU.tsv	Root-Host_associated-Human-Skin
ERR2538384	MGYA00381391	https://www.ebi.ac.uk/metagenomics/api/v1/analyses/MGYA00381391/file/ERR2538384_FASTQ_SSU_OTU.tsv	Root-Host_associated-Human-Skin
ERR451243	MGYA00424895	https://www.ebi.ac.uk/metagenomics/api/v1/analyses/MGYA00424895/file/ERR451243_FASTQ_SSU_OTU.tsv	Root-Host_associated-Human-Skin
SRR920731	MGYA00025022	https://www.ebi.ac.uk/metagenomics/api/v1/analyses/MGYA00025022/file/SRR920731_FASTQ_otu.tsv	Root-Host_associated-Human-Skin
ERR442016	MGYA00385171	https://www.ebi.ac.uk/metagenomics/api/v1/analyses/MGYA00385171/file/ERR442016_FASTQ_SSU_OTU.tsv	Root-Host_associated-Human-Skin
SRR1632349	MGYA00023663	https://www.ebi.ac.uk/metagenomics/api/v1/analyses/MGYA00023663/file/SRR1632349_MERGED_FASTQ_otu.tsv	Root-Host_associated-Human-Skin
SRR5868639	MGYA00428075	https://www.ebi.ac.uk/metagenomics/api/v1/analyses/MGYA00428075/file/SRR5868639_MERGED_FASTQ_SSU_OTU.tsv	Root-Host_associated-Human-Skin
SRR919906	MGYA00025024	https://www.ebi.ac.uk/metagenomics/api/v1/analyses/MGYA00025024/file/SRR919906_FASTQ_otu.tsv	Root-Host_associated-Human-Skin
SRR919574	MGYA00024729	https://www.ebi.ac.uk/metagenomics/api/v1/analyses/MGYA00024729/file/SRR919574_FASTQ_otu.tsv	Root-Host_associated-Human-Skin
ERR1551333	MGYA00069433	https://www.ebi.ac.uk/metagenomics/api/v1/analyses/MGYA00069433/file/ERR1551333_MERGED_FASTQ_otu.tsv	Root-Host_associated-Human-Skin

ERR443498	MGYA00427802	https://www.ebi.ac.uk/metagenomics/api/v1/analyses/MGYA00427802/file/ERR443498_FASTQ_SSU_OTU.tsv	Root-Host_associated-Human-Skin
SRR995603	MGYA00025042	https://www.ebi.ac.uk/metagenomics/api/v1/analyses/MGYA00025042/file/SRR995603_FASTQ_otu.tsv	Root-Host_associated-Human-Skin
SRR1624843	MGYA00024290	https://www.ebi.ac.uk/metagenomics/api/v1/analyses/MGYA00024290/file/SRR1624843_MERGED_FASTQ_otu.tsv	Root-Host_associated-Human-Skin
SRR1633168	MGYA00022379	https://www.ebi.ac.uk/metagenomics/api/v1/analyses/MGYA00022379/file/SRR1633168_MERGED_FASTQ_otu.tsv	Root-Host_associated-Human-Skin
SRR996060	MGYA00023314	https://www.ebi.ac.uk/metagenomics/api/v1/analyses/MGYA00023314/file/SRR996060_FASTQ_otu.tsv	Root-Host_associated-Human-Skin
SRR920118	MGYA00024133	https://www.ebi.ac.uk/metagenomics/api/v1/analyses/MGYA00024133/file/SRR920118_FASTQ_otu.tsv	Root-Host_associated-Human-Skin
SRR996873	MGYA00023438	https://www.ebi.ac.uk/metagenomics/api/v1/analyses/MGYA00023438/file/SRR996873_FASTQ_otu.tsv	Root-Host_associated-Human-Skin
SRR1704249	MGYA00413839	https://www.ebi.ac.uk/metagenomics/api/v1/analyses/MGYA00413839/file/SRR1704249_FASTQ_SSU_OTU.tsv	Root-Host_associated-Human-Skin
SRR994881	MGYA00022187	https://www.ebi.ac.uk/metagenomics/api/v1/analyses/MGYA00022187/file/SRR994881_FASTQ_otu.tsv	Root-Host_associated-Human-Skin
SRR995966	MGYA00023848	https://www.ebi.ac.uk/metagenomics/api/v1/analyses/MGYA00023848/file/SRR995966_FASTQ_otu.tsv	Root-Host_associated-Human-Skin
ERR451295	MGYA00424892	https://www.ebi.ac.uk/metagenomics/api/v1/analyses/MGYA00424892/file/ERR451295_FASTQ_SSU_OTU.tsv	Root-Host_associated-Human-Skin
ERR1960771	MGYA00130865	https://www.ebi.ac.uk/metagenomics/api/v1/analyses/MGYA00130865/file/ERR1960771_MERGED_FASTQ_otu.tsv	Root-Host_associated-Human-Skin

SRR6757605	MGYA00380724	https://www.ebi.ac.uk/metagenomics/api/v1/analyses/MGYA00380724/file/SRR6757605_MERGED_FASTQ_SSU_OTU.tsv	Root-Host_associated-Human-Skin
SRR1924028	MGYA00028375	https://www.ebi.ac.uk/metagenomics/api/v1/analyses/MGYA00028375/file/SRR1924028_FASTQ_otu.tsv	Root-Host_associated-Human-Skin
SRR1623214	MGYA00023913	https://www.ebi.ac.uk/metagenomics/api/v1/analyses/MGYA00023913/file/SRR1623214_MERGED_FASTQ_otu.tsv	Root-Host_associated-Human-Skin
ERR2538352	MGYA00381359	https://www.ebi.ac.uk/metagenomics/api/v1/analyses/MGYA00381359/file/ERR2538352_FASTQ_SSU_OTU.tsv	Root-Host_associated-Human-Skin
SRR5869591	MGYA00428052	https://www.ebi.ac.uk/metagenomics/api/v1/analyses/MGYA00428052/file/SRR5869591_MERGED_FASTQ_SSU_OTU.tsv	Root-Host_associated-Human-Skin
SRR996138	MGYA00024985	https://www.ebi.ac.uk/metagenomics/api/v1/analyses/MGYA00024985/file/SRR996138_FASTQ_otu.tsv	Root-Host_associated-Human-Skin
SRR2005672	MGYA00381329	https://www.ebi.ac.uk/metagenomics/api/v1/analyses/MGYA00381329/file/SRR2005672_MERGED_FASTQ_SSU_OTU.tsv	Root-Host_associated-Human-Skin
SRR996559	MGYA00022626	https://www.ebi.ac.uk/metagenomics/api/v1/analyses/MGYA00022626/file/SRR996559_FASTQ_otu.tsv	Root-Host_associated-Human-Skin
SRR1631304	MGYA00023230	https://www.ebi.ac.uk/metagenomics/api/v1/analyses/MGYA00023230/file/SRR1631304_MERGED_FASTQ_otu.tsv	Root-Host_associated-Human-Skin
SRR1630936	MGYA00023966	https://www.ebi.ac.uk/metagenomics/api/v1/analyses/MGYA00023966/file/SRR1630936_MERGED_FASTQ_otu.tsv	Root-Host_associated-Human-Skin
ERR443606	MGYA00425751	https://www.ebi.ac.uk/metagenomics/api/v1/analyses/MGYA00425751/file/ERR443606_FASTQ_SSU_OTU.tsv	Root-Host_associated-Human-Skin
ERR1960814	MGYA00130813	https://www.ebi.ac.uk/metagenomics/api/v1/analyses/MGYA00130813/file/ERR1960814_MERGED_FASTQ_otu.tsv	Root-Host_associated-Human-Skin

ERR2538383	MGYA00381390	https://www.ebi.ac.uk/metagenomics/api/v1/analyses/MGYA00381390/file/ERR2538383_FASTQ_SSU_OTU.tsv	Root-Host_associated-Human-Skin
ERR2538424	MGYA00381431	https://www.ebi.ac.uk/metagenomics/api/v1/analyses/MGYA00381431/file/ERR2538424_FASTQ_SSU_OTU.tsv	Root-Host_associated-Human-Skin
SRR920736	MGYA00025020	https://www.ebi.ac.uk/metagenomics/api/v1/analyses/MGYA00025020/file/SRR920736_FASTQ_otu.tsv	Root-Host_associated-Human-Skin
ERR451244	MGYA00419509	https://www.ebi.ac.uk/metagenomics/api/v1/analyses/MGYA00419509/file/ERR451244_FASTQ_SSU_OTU.tsv	Root-Host_associated-Human-Skin
SRR1633030	MGYA00024157	https://www.ebi.ac.uk/metagenomics/api/v1/analyses/MGYA00024157/file/SRR1633030_MERGED_FASTQ_otu.tsv	Root-Host_associated-Human-Skin
SRR1704543	MGYA00426924	https://www.ebi.ac.uk/metagenomics/api/v1/analyses/MGYA00426924/file/SRR1704543_FASTQ_SSU_OTU.tsv	Root-Host_associated-Human-Skin
ERR1759907	MGYA00140440	https://www.ebi.ac.uk/metagenomics/api/v1/analyses/MGYA00140440/file/ERR1759907_MERGED_FASTQ_SSU_OTU.tsv	Root-Host_associated-Human-Skin
SRR1950751	MGYA00376918	https://www.ebi.ac.uk/metagenomics/api/v1/analyses/MGYA00376918/file/SRR1950751_MERGED_FASTQ_SSU_OTU.tsv	Root-Host_associated-Human-Skin
SRR1632335	MGYA00024582	https://www.ebi.ac.uk/metagenomics/api/v1/analyses/MGYA00024582/file/SRR1632335_MERGED_FASTQ_otu.tsv	Root-Host_associated-Human-Skin
ERR443722	MGYA00419142	https://www.ebi.ac.uk/metagenomics/api/v1/analyses/MGYA00419142/file/ERR443722_FASTQ_SSU_OTU.tsv	Root-Host_associated-Human-Skin
SRR5869816	MGYA00425837	https://www.ebi.ac.uk/metagenomics/api/v1/analyses/MGYA00425837/file/SRR5869816_MERGED_FASTQ_SSU_OTU.tsv	Root-Host_associated-Human-Skin
SRR5869464	MGYA00421634	https://www.ebi.ac.uk/metagenomics/api/v1/analyses/MGYA00421634/file/SRR5869464_MERGED_FASTQ_SSU_OTU.tsv	Root-Host_associated-Human-Skin

ERR2538500	MGYA00381507	https://www.ebi.ac.uk/metagenomics/api/v1/analyses/MGYA00381507/file/ERR2538500_FASTQ_SSU_OTU.tsv	Root-Host_associated-Human-Skin
SRR1704685	MGYA00414624	https://www.ebi.ac.uk/metagenomics/api/v1/analyses/MGYA00414624/file/SRR1704685_FASTQ_SSU_OTU.tsv	Root-Host_associated-Human-Skin
SRR994798	MGYA00022824	https://www.ebi.ac.uk/metagenomics/api/v1/analyses/MGYA00022824/file/SRR994798_FASTQ_otu.tsv	Root-Host_associated-Human-Skin
ERR1960684	MGYA00130901	https://www.ebi.ac.uk/metagenomics/api/v1/analyses/MGYA00130901/file/ERR1960684_MERGED_FASTQ_otu.tsv	Root-Host_associated-Human-Skin
SRR921052	MGYA00022862	https://www.ebi.ac.uk/metagenomics/api/v1/analyses/MGYA00022862/file/SRR921052_FASTQ_otu.tsv	Root-Host_associated-Human-Skin
SRR995109	MGYA00023661	https://www.ebi.ac.uk/metagenomics/api/v1/analyses/MGYA00023661/file/SRR995109_FASTQ_otu.tsv	Root-Host_associated-Human-Skin
ERR1760119	MGYA00140382	https://www.ebi.ac.uk/metagenomics/api/v1/analyses/MGYA00140382/file/ERR1760119_MERGED_FASTQ_SSU_OTU.tsv	Root-Host_associated-Human-Skin
SRR5870179	MGYA00420652	https://www.ebi.ac.uk/metagenomics/api/v1/analyses/MGYA00420652/file/SRR5870179_MERGED_FASTQ_SSU_OTU.tsv	Root-Host_associated-Human-Skin
ERR1960655	MGYA00130805	https://www.ebi.ac.uk/metagenomics/api/v1/analyses/MGYA00130805/file/ERR1960655_MERGED_FASTQ_otu.tsv	Root-Host_associated-Human-Skin
SRR1622902	MGYA00022367	https://www.ebi.ac.uk/metagenomics/api/v1/analyses/MGYA00022367/file/SRR1622902_MERGED_FASTQ_otu.tsv	Root-Host_associated-Human-Skin
SRR920977	MGYA00023040	https://www.ebi.ac.uk/metagenomics/api/v1/analyses/MGYA00023040/file/SRR920977_FASTQ_otu.tsv	Root-Host_associated-Human-Skin
SRR1632831	MGYA00022819	https://www.ebi.ac.uk/metagenomics/api/v1/analyses/MGYA00022819/file/SRR1632831_MERGED_FASTQ_otu.tsv	Root-Host_associated-Human-Skin

ERR451077	MGYA00412385	https://www.ebi.ac.uk/metagenomics/api/v1/analyses/MGYA00412385/file/ERR451077_FASTQ_SSU_OTU.tsv	Root-Host_associated-Human-Skin
SRR995568	MGYA00022976	https://www.ebi.ac.uk/metagenomics/api/v1/analyses/MGYA00022976/file/SRR995568_FASTQ_otu.tsv	Root-Host_associated-Human-Skin
SRR1923914	MGYA00028294	https://www.ebi.ac.uk/metagenomics/api/v1/analyses/MGYA00028294/file/SRR1923914_FASTQ_otu.tsv	Root-Host_associated-Human-Skin
SRR1950780	MGYA00376947	https://www.ebi.ac.uk/metagenomics/api/v1/analyses/MGYA00376947/file/SRR1950780_MERGED_FASTQ_SSU_OTU.tsv	Root-Host_associated-Human-Skin
SRR5868694	MGYA00427606	https://www.ebi.ac.uk/metagenomics/api/v1/analyses/MGYA00427606/file/SRR5868694_MERGED_FASTQ_SSU_OTU.tsv	Root-Host_associated-Human-Skin
SRR1631556	MGYA00023797	https://www.ebi.ac.uk/metagenomics/api/v1/analyses/MGYA00023797/file/SRR1631556_MERGED_FASTQ_otu.tsv	Root-Host_associated-Human-Skin
SRR6757679	MGYA00380792	https://www.ebi.ac.uk/metagenomics/api/v1/analyses/MGYA00380792/file/SRR6757679_MERGED_FASTQ_SSU_OTU.tsv	Root-Host_associated-Human-Skin
SRR996144	MGYA00022531	https://www.ebi.ac.uk/metagenomics/api/v1/analyses/MGYA00022531/file/SRR996144_FASTQ_otu.tsv	Root-Host_associated-Human-Skin
ERR2538489	MGYA00381496	https://www.ebi.ac.uk/metagenomics/api/v1/analyses/MGYA00381496/file/ERR2538489_FASTQ_SSU_OTU.tsv	Root-Host_associated-Human-Skin
ERR1760041	MGYA00140608	https://www.ebi.ac.uk/metagenomics/api/v1/analyses/MGYA00140608/file/ERR1760041_MERGED_FASTQ_SSU_OTU.tsv	Root-Host_associated-Human-Skin
SRR994611	MGYA00023012	https://www.ebi.ac.uk/metagenomics/api/v1/analyses/MGYA00023012/file/SRR994611_FASTQ_otu.tsv	Root-Host_associated-Human-Skin
SRR996957	MGYA00022873	https://www.ebi.ac.uk/metagenomics/api/v1/analyses/MGYA00022873/file/SRR996957_FASTQ_otu.tsv	Root-Host_associated-Human-Skin

SRR996282	MGYA00023310	https://www.ebi.ac.uk/metagenomics/api/v1/analyses/MGYA00023310/file/SRR996282_FASTQ_otu.tsv	Root-Host_associated-Human-Skin
SRR995397	MGYA00024754	https://www.ebi.ac.uk/metagenomics/api/v1/analyses/MGYA00024754/file/SRR995397_FASTQ_otu.tsv	Root-Host_associated-Human-Skin
SRR995346	MGYA00023120	https://www.ebi.ac.uk/metagenomics/api/v1/analyses/MGYA00023120/file/SRR995346_FASTQ_otu.tsv	Root-Host_associated-Human-Skin
SRR994974	MGYA00024554	https://www.ebi.ac.uk/metagenomics/api/v1/analyses/MGYA00024554/file/SRR994974_FASTQ_otu.tsv	Root-Host_associated-Human-Skin
SRR994506	MGYA00023822	https://www.ebi.ac.uk/metagenomics/api/v1/analyses/MGYA00023822/file/SRR994506_FASTQ_otu.tsv	Root-Host_associated-Human-Skin
SRR996986	MGYA00024531	https://www.ebi.ac.uk/metagenomics/api/v1/analyses/MGYA00024531/file/SRR996986_FASTQ_otu.tsv	Root-Host_associated-Human-Skin
ERR1760090	MGYA00140445	https://www.ebi.ac.uk/metagenomics/api/v1/analyses/MGYA00140445/file/ERR1760090_MERGED_FASTQ_SSU_OTU.tsv	Root-Host_associated-Human-Skin
SRR995080	MGYA00022719	https://www.ebi.ac.uk/metagenomics/api/v1/analyses/MGYA00022719/file/SRR995080_FASTQ_otu.tsv	Root-Host_associated-Human-Skin
ERR2538458	MGYA00381465	https://www.ebi.ac.uk/metagenomics/api/v1/analyses/MGYA00381465/file/ERR2538458_FASTQ_SSU_OTU.tsv	Root-Host_associated-Human-Skin
SRR1631719	MGYA00024044	https://www.ebi.ac.uk/metagenomics/api/v1/analyses/MGYA00024044/file/SRR1631719_MERGED_FASTQ_otu.tsv	Root-Host_associated-Human-Skin
SRR997072	MGYA00023160	https://www.ebi.ac.uk/metagenomics/api/v1/analyses/MGYA00023160/file/SRR997072_FASTQ_otu.tsv	Root-Host_associated-Human-Skin
SRR919908	MGYA00025033	https://www.ebi.ac.uk/metagenomics/api/v1/analyses/MGYA00025033/file/SRR919908_FASTQ_otu.tsv	Root-Host_associated-Human-Skin

SRR1950723	MGYA00376890	https://www.ebi.ac.uk/metagenomics/api/v1/analyses/MGYA00376890/file/SRR1950723_MERGED_FASTQ_SSU_OTU.tsv	Root-Host_associated-Human-Skin
ERR1759975	MGYA00140505	https://www.ebi.ac.uk/metagenomics/api/v1/analyses/MGYA00140505/file/ERR1759975_MERGED_FASTQ_SSU_OTU.tsv	Root-Host_associated-Human-Skin
SRR5868637	MGYA00428079	https://www.ebi.ac.uk/metagenomics/api/v1/analyses/MGYA00428079/file/SRR5868637_MERGED_FASTQ_SSU_OTU.tsv	Root-Host_associated-Human-Skin
SRR1631252	MGYA00024543	https://www.ebi.ac.uk/metagenomics/api/v1/analyses/MGYA00024543/file/SRR1631252_MERGED_FASTQ_otu.tsv	Root-Host_associated-Human-Skin
SRR1704531	MGYA00421134	https://www.ebi.ac.uk/metagenomics/api/v1/analyses/MGYA00421134/file/SRR1704531_FASTQ_SSU_OTU.tsv	Root-Host_associated-Human-Skin
SRR920201	MGYA00023056	https://www.ebi.ac.uk/metagenomics/api/v1/analyses/MGYA00023056/file/SRR920201_FASTQ_otu.tsv	Root-Host_associated-Human-Skin
ERR443750	MGYA00414525	https://www.ebi.ac.uk/metagenomics/api/v1/analyses/MGYA00414525/file/ERR443750_FASTQ_SSU_OTU.tsv	Root-Host_associated-Human-Skin
SRR1632347	MGYA00025163	https://www.ebi.ac.uk/metagenomics/api/v1/analyses/MGYA00025163/file/SRR1632347_MERGED_FASTQ_otu.tsv	Root-Host_associated-Human-Skin
ERR442018	MGYA00384599	https://www.ebi.ac.uk/metagenomics/api/v1/analyses/MGYA00384599/file/ERR442018_FASTQ_SSU_OTU.tsv	Root-Host_associated-Human-Skin
SRR996718	MGYA00025249	https://www.ebi.ac.uk/metagenomics/api/v1/analyses/MGYA00025249/file/SRR996718_FASTQ_otu.tsv	Root-Host_associated-Human-Skin
ERR442903	MGYA00415564	https://www.ebi.ac.uk/metagenomics/api/v1/analyses/MGYA00415564/file/ERR442903_FASTQ_SSU_OTU.tsv	Root-Host_associated-Human-Skin
ERR451312	MGYA00428734	https://www.ebi.ac.uk/metagenomics/api/v1/analyses/MGYA00428734/file/ERR451312_FASTQ_SSU_OTU.tsv	Root-Host_associated-Human-Skin

SRR1633166	MGYA00022383	https://www.ebi.ac.uk/metagenomics/api/v1/analyses/MGYA00022383/file/SRR1633166_MERGED_FASTQ_otu.tsv	Root-Host_associated-Human-Skin
SRR920660	MGYA00022340	https://www.ebi.ac.uk/metagenomics/api/v1/analyses/MGYA00022340/file/SRR920660_FASTQ_otu.tsv	Root-Host_associated-Human-Skin
ERR443447	MGYA00413670	https://www.ebi.ac.uk/metagenomics/api/v1/analyses/MGYA00413670/file/ERR443447_FASTQ_SSU_OTU.tsv	Root-Host_associated-Human-Skin
ERR443835	MGYA00415481	https://www.ebi.ac.uk/metagenomics/api/v1/analyses/MGYA00415481/file/ERR443835_FASTQ_SSU_OTU.tsv	Root-Host_associated-Human-Skin
SRR920116	MGYA00024131	https://www.ebi.ac.uk/metagenomics/api/v1/analyses/MGYA00024131/file/SRR920116_FASTQ_otu.tsv	Root-Host_associated-Human-Skin
SRR1704626	MGYA00426148	https://www.ebi.ac.uk/metagenomics/api/v1/analyses/MGYA00426148/file/SRR1704626_FASTQ_SSU_OTU.tsv	Root-Host_associated-Human-Skin
SRR1704181	MGYA00428934	https://www.ebi.ac.uk/metagenomics/api/v1/analyses/MGYA00428934/file/SRR1704181_FASTQ_SSU_OTU.tsv	Root-Host_associated-Human-Skin
ERR451005	MGYA00416319	https://www.ebi.ac.uk/metagenomics/api/v1/analyses/MGYA00416319/file/ERR451005_FASTQ_SSU_OTU.tsv	Root-Host_associated-Human-Skin
SRR995968	MGYA00024460	https://www.ebi.ac.uk/metagenomics/api/v1/analyses/MGYA00024460/file/SRR995968_FASTQ_otu.tsv	Root-Host_associated-Human-Skin
ERR443781	MGYA00421087	https://www.ebi.ac.uk/metagenomics/api/v1/analyses/MGYA00421087/file/ERR443781_FASTQ_SSU_OTU.tsv	Root-Host_associated-Human-Skin
SRR920905	MGYA00024110	https://www.ebi.ac.uk/metagenomics/api/v1/analyses/MGYA00024110/file/SRR920905_FASTQ_otu.tsv	Root-Host_associated-Human-Skin
SRR1704247	MGYA00426995	https://www.ebi.ac.uk/metagenomics/api/v1/analyses/MGYA00426995/file/SRR1704247_FASTQ_SSU_OTU.tsv	Root-Host_associated-Human-Skin

SRR1631283	MGYA00022661	https://www.ebi.ac.uk/metagenomics/api/v1/analyses/MGYA00022661/file/SRR1631283_MERGED_FASTQ_otu.tsv	Root-Host_associated-Human-Skin
SRR2005724	MGYA00381353	https://www.ebi.ac.uk/metagenomics/api/v1/analyses/MGYA00381353/file/SRR2005724_MERGED_FASTQ_SSU_OTU.tsv	Root-Host_associated-Human-Skin
SRR996691	MGYA00022292	https://www.ebi.ac.uk/metagenomics/api/v1/analyses/MGYA00022292/file/SRR996691_FASTQ_otu.tsv	Root-Host_associated-Human-Skin
SRR996136	MGYA00024993	https://www.ebi.ac.uk/metagenomics/api/v1/analyses/MGYA00024993/file/SRR996136_FASTQ_otu.tsv	Root-Host_associated-Human-Skin
SRR1924026	MGYA00028377	https://www.ebi.ac.uk/metagenomics/api/v1/analyses/MGYA00028377/file/SRR1924026_FASTQ_otu.tsv	Root-Host_associated-Human-Skin
SRR995023	MGYA00023951	https://www.ebi.ac.uk/metagenomics/api/v1/analyses/MGYA00023951/file/SRR995023_FASTQ_otu.tsv	Root-Host_associated-Human-Skin
ERR1760033	MGYA00140443	https://www.ebi.ac.uk/metagenomics/api/v1/analyses/MGYA00140443/file/ERR1760033_MERGED_FASTQ_SSU_OTU.tsv	Root-Host_associated-Human-Skin
SRR5869659	MGYA00419195	https://www.ebi.ac.uk/metagenomics/api/v1/analyses/MGYA00419195/file/SRR5869659_MERGED_FASTQ_SSU_OTU.tsv	Root-Host_associated-Human-Skin
SRR919881	MGYA00024204	https://www.ebi.ac.uk/metagenomics/api/v1/analyses/MGYA00024204/file/SRR919881_FASTQ_otu.tsv	Root-Host_associated-Human-Skin
SRR996557	MGYA00022624	https://www.ebi.ac.uk/metagenomics/api/v1/analyses/MGYA00022624/file/SRR996557_FASTQ_otu.tsv	Root-Host_associated-Human-Skin
SRR996925	MGYA00025130	https://www.ebi.ac.uk/metagenomics/api/v1/analyses/MGYA00025130/file/SRR996925_FASTQ_otu.tsv	Root-Host_associated-Human-Skin
SRR995493	MGYA00024114	https://www.ebi.ac.uk/metagenomics/api/v1/analyses/MGYA00024114/file/SRR995493_FASTQ_otu.tsv	Root-Host_associated-Human-Skin

SRR919850	MGYA00022242	https://www.ebi.ac.uk/metagenomics/api/v1/analyses/MGYA00022242/file/SRR919850_FASTQ_otu.tsv	Root-Host_associated-Human-Skin
SRR5869688	MGYA00416985	https://www.ebi.ac.uk/metagenomics/api/v1/analyses/MGYA00416985/file/SRR5869688_MERGED_FASTQ_SSU_OTU.tsv	Root-Host_associated-Human-Skin
SRR996221	MGYA00023978	https://www.ebi.ac.uk/metagenomics/api/v1/analyses/MGYA00023978/file/SRR996221_FASTQ_otu.tsv	Root-Host_associated-Human-Skin
SRR1630938	MGYA00023964	https://www.ebi.ac.uk/metagenomics/api/v1/analyses/MGYA00023964/file/SRR1630938_MERGED_FASTQ_otu.tsv	Root-Host_associated-Human-Skin
SRR1704469	MGYA00423381	https://www.ebi.ac.uk/metagenomics/api/v1/analyses/MGYA00423381/file/SRR1704469_FASTQ_SSU_OTU.tsv	Root-Host_associated-Human-Skin
SRR995755	MGYA00022891	https://www.ebi.ac.uk/metagenomics/api/v1/analyses/MGYA00022891/file/SRR995755_FASTQ_otu.tsv	Root-Host_associated-Human-Skin
ERR450878	MGYA00422387	https://www.ebi.ac.uk/metagenomics/api/v1/analyses/MGYA00422387/file/ERR450878_FASTQ_SSU_OTU.tsv	Root-Host_associated-Human-Skin
ERR443269	MGYA00412991	https://www.ebi.ac.uk/metagenomics/api/v1/analyses/MGYA00412991/file/ERR443269_FASTQ_SSU_OTU.tsv	Root-Host_associated-Human-Skin
SRR994450	MGYA00022458	https://www.ebi.ac.uk/metagenomics/api/v1/analyses/MGYA00022458/file/SRR994450_FASTQ_otu.tsv	Root-Host_associated-Human-Skin
ERR442064	MGYA00385198	https://www.ebi.ac.uk/metagenomics/api/v1/analyses/MGYA00385198/file/ERR442064_FASTQ_SSU_OTU.tsv	Root-Host_associated-Human-Skin
SRR996305	MGYA00022707	https://www.ebi.ac.uk/metagenomics/api/v1/analyses/MGYA00022707/file/SRR996305_FASTQ_otu.tsv	Root-Host_associated-Human-Skin
ERR1759909	MGYA00140398	https://www.ebi.ac.uk/metagenomics/api/v1/analyses/MGYA00140398/file/ERR1759909_MERGED_FASTQ_SSU_OTU.tsv	Root-Host_associated-Human-Skin

SRR994796	MGYA00022830	https://www.ebi.ac.uk/metagenomics/api/v1/analyses/MGYA00022830/file/SRR994796_FASTQ_otu.tsv	Root-Host_associated-Human-Skin
ERR441962	MGYA00385163	https://www.ebi.ac.uk/metagenomics/api/v1/analyses/MGYA00385163/file/ERR441962_FASTQ_SSU_OTU.tsv	Root-Host_associated-Human-Skin
SRR996764	MGYA00023863	https://www.ebi.ac.uk/metagenomics/api/v1/analyses/MGYA00023863/file/SRR996764_FASTQ_otu.tsv	Root-Host_associated-Human-Skin
SRR919611	MGYA00023527	https://www.ebi.ac.uk/metagenomics/api/v1/analyses/MGYA00023527/file/SRR919611_FASTQ_otu.tsv	Root-Host_associated-Human-Skin
ERR451418	MGYA00422362	https://www.ebi.ac.uk/metagenomics/api/v1/analyses/MGYA00422362/file/ERR451418_FASTQ_SSU_OTU.tsv	Root-Host_associated-Human-Skin
ERR1760117	MGYA00140546	https://www.ebi.ac.uk/metagenomics/api/v1/analyses/MGYA00140546/file/ERR1760117_MERGED_FASTQ_SSU_OTU.tsv	Root-Host_associated-Human-Skin
SRR995107	MGYA00023669	https://www.ebi.ac.uk/metagenomics/api/v1/analyses/MGYA00023669/file/SRR995107_FASTQ_otu.tsv	Root-Host_associated-Human-Skin
SRR1631558	MGYA00023807	https://www.ebi.ac.uk/metagenomics/api/v1/analyses/MGYA00023807/file/SRR1631558_MERGED_FASTQ_otu.tsv	Root-Host_associated-Human-Skin
SRR995566	MGYA00022984	https://www.ebi.ac.uk/metagenomics/api/v1/analyses/MGYA00022984/file/SRR995566_FASTQ_otu.tsv	Root-Host_associated-Human-Skin
SRR920979	MGYA00023034	https://www.ebi.ac.uk/metagenomics/api/v1/analyses/MGYA00023034/file/SRR920979_FASTQ_otu.tsv	Root-Host_associated-Human-Skin
SRR994481	MGYA00023135	https://www.ebi.ac.uk/metagenomics/api/v1/analyses/MGYA00023135/file/SRR994481_FASTQ_otu.tsv	Root-Host_associated-Human-Skin
ERR1960703	MGYA00130869	https://www.ebi.ac.uk/metagenomics/api/v1/analyses/MGYA00130869/file/ERR1960703_MERGED_FASTQ_otu.tsv	Root-Host_associated-Human-Skin

SRR1622854	MGYA00024383	https://www.ebi.ac.uk/metagenomics/api/v1/analyses/MGYA00024383/file/SRR1622854_MERGED_FASTQ_otu.tsv	Root-Host_associated-Human-Skin
ERR2538487	MGYA00381494	https://www.ebi.ac.uk/metagenomics/api/v1/analyses/MGYA00381494/file/ERR2538487_FASTQ_SSU_OTU.tsv	Root-Host_associated-Human-Skin
SRR1704702	MGYA00428443	https://www.ebi.ac.uk/metagenomics/api/v1/analyses/MGYA00428443/file/SRR1704702_FASTQ_SSU_OTU.tsv	Root-Host_associated-Human-Skin
SRR5868876	MGYA00417059	https://www.ebi.ac.uk/metagenomics/api/v1/analyses/MGYA00417059/file/SRR5868876_MERGED_FASTQ_SSU_OTU.tsv	Root-Host_associated-Human-Skin
SRR6757677	MGYA00380790	https://www.ebi.ac.uk/metagenomics/api/v1/analyses/MGYA00380790/file/SRR6757677_MERGED_FASTQ_SSU_OTU.tsv	Root-Host_associated-Human-Skin
SRR995399	MGYA00024752	https://www.ebi.ac.uk/metagenomics/api/v1/analyses/MGYA00024752/file/SRR995399_FASTQ_otu.tsv	Root-Host_associated-Human-Skin
SRR1631400	MGYA00022431	https://www.ebi.ac.uk/metagenomics/api/v1/analyses/MGYA00022431/file/SRR1631400_MERGED_FASTQ_otu.tsv	Root-Host_associated-Human-Skin
SRR5869182	MGYA00415970	https://www.ebi.ac.uk/metagenomics/api/v1/analyses/MGYA00415970/file/SRR5869182_MERGED_FASTQ_SSU_OTU.tsv	Root-Host_associated-Human-Skin
SRR1630995	MGYA00022853	https://www.ebi.ac.uk/metagenomics/api/v1/analyses/MGYA00022853/file/SRR1630995_MERGED_FASTQ_otu.tsv	Root-Host_associated-Human-Skin
SRR5869153	MGYA00418089	https://www.ebi.ac.uk/metagenomics/api/v1/analyses/MGYA00418089/file/SRR5869153_MERGED_FASTQ_SSU_OTU.tsv	Root-Host_associated-Human-Skin
SRR996988	MGYA00024529	https://www.ebi.ac.uk/metagenomics/api/v1/analyses/MGYA00024529/file/SRR996988_FASTQ_otu.tsv	Root-Host_associated-Human-Skin
SRR994508	MGYA00023824	https://www.ebi.ac.uk/metagenomics/api/v1/analyses/MGYA00023824/file/SRR994508_FASTQ_otu.tsv	Root-Host_associated-Human-Skin

SRR995348	MGYA00023126	https://www.ebi.ac.uk/metagenomics/api/v1/analyses/MGYA00023126/file/SRR995348_FASTQ_otu.tsv	Root-Host_associated-Human-Skin
SRR1923893	MGYA00028369	https://www.ebi.ac.uk/metagenomics/api/v1/analyses/MGYA00028369/file/SRR1923893_FASTQ_otu.tsv	Root-Host_associated-Human-Skin
SRR1631717	MGYA00024038	https://www.ebi.ac.uk/metagenomics/api/v1/analyses/MGYA00024038/file/SRR1631717_MERGED_FASTQ_otu.tsv	Root-Host_associated-Human-Skin
ERR2538456	MGYA00381463	https://www.ebi.ac.uk/metagenomics/api/v1/analyses/MGYA00381463/file/ERR2538456_FASTQ_SSU_OTU.tsv	Root-Host_associated-Human-Skin
SRR5869295	MGYA00415927	https://www.ebi.ac.uk/metagenomics/api/v1/analyses/MGYA00415927/file/SRR5869295_MERGED_FASTQ_SSU_OTU.tsv	Root-Host_associated-Human-Skin
SRR921104	MGYA00025000	https://www.ebi.ac.uk/metagenomics/api/v1/analyses/MGYA00025000/file/SRR921104_FASTQ_otu.tsv	Root-Host_associated-Human-Skin
ERR1701271	MGYA00084301	https://www.ebi.ac.uk/metagenomics/api/v1/analyses/MGYA00084301/file/ERR1701271_FASTQ_otu.tsv	Root-Host_associated-Human-Skin
SRR920744	MGYA00023540	https://www.ebi.ac.uk/metagenomics/api/v1/analyses/MGYA00023540/file/SRR920744_FASTQ_otu.tsv	Root-Host_associated-Human-Skin
ERR442063	MGYA00385185	https://www.ebi.ac.uk/metagenomics/api/v1/analyses/MGYA00385185/file/ERR442063_FASTQ_SSU_OTU.tsv	Root-Host_associated-Human-Skin
SRR994457	MGYA00022459	https://www.ebi.ac.uk/metagenomics/api/v1/analyses/MGYA00022459/file/SRR994457_FASTQ_otu.tsv	Root-Host_associated-Human-Skin
SRR996302	MGYA00022714	https://www.ebi.ac.uk/metagenomics/api/v1/analyses/MGYA00022714/file/SRR996302_FASTQ_otu.tsv	Root-Host_associated-Human-Skin
SRR1950758	MGYA00376925	https://www.ebi.ac.uk/metagenomics/api/v1/analyses/MGYA00376925/file/SRR1950758_MERGED_FASTQ_SSU_OTU.tsv	Root-Host_associated-Human-Skin

SRR994791	MGYA00022833	https://www.ebi.ac.uk/metagenomics/api/v1/analyses/MGYA00022833/file/SRR994791_FASTQ_otu.tsv	Root-Host_associated-Human-Skin
ERR451369	MGYA00413349	https://www.ebi.ac.uk/metagenomics/api/v1/analyses/MGYA00413349/file/ERR451369_FASTQ_SSU_OTU.tsv	Root-Host_associated-Human-Skin
SRR996763	MGYA00023856	https://www.ebi.ac.uk/metagenomics/api/v1/analyses/MGYA00023856/file/SRR996763_FASTQ_otu.tsv	Root-Host_associated-Human-Skin
ERR441965	MGYA00385258	https://www.ebi.ac.uk/metagenomics/api/v1/analyses/MGYA00385258/file/ERR441965_FASTQ_SSU_OTU.tsv	Root-Host_associated-Human-Skin
ERR2538509	MGYA00381516	https://www.ebi.ac.uk/metagenomics/api/v1/analyses/MGYA00381516/file/ERR2538509_FASTQ_SSU_OTU.tsv	Root-Host_associated-Human-Skin
SRR919616	MGYA00023528	https://www.ebi.ac.uk/metagenomics/api/v1/analyses/MGYA00023528/file/SRR919616_FASTQ_otu.tsv	Root-Host_associated-Human-Skin
ERR1760110	MGYA00140559	https://www.ebi.ac.uk/metagenomics/api/v1/analyses/MGYA00140559/file/ERR1760110_MERGED_FASTQ_SSU_OTU.tsv	Root-Host_associated-Human-Skin
SRR995100	MGYA00023666	https://www.ebi.ac.uk/metagenomics/api/v1/analyses/MGYA00023666/file/SRR995100_FASTQ_otu.tsv	Root-Host_associated-Human-Skin
SRR1950789	MGYA00376956	https://www.ebi.ac.uk/metagenomics/api/v1/analyses/MGYA00376956/file/SRR1950789_MERGED_FASTQ_SSU_OTU.tsv	Root-Host_associated-Human-Skin
SRR1632838	MGYA00022822	https://www.ebi.ac.uk/metagenomics/api/v1/analyses/MGYA00022822/file/SRR1632838_MERGED_FASTQ_otu.tsv	Root-Host_associated-Human-Skin
SRR994486	MGYA00023145	https://www.ebi.ac.uk/metagenomics/api/v1/analyses/MGYA00023145/file/SRR994486_FASTQ_otu.tsv	Root-Host_associated-Human-Skin
SRR995561	MGYA00022979	https://www.ebi.ac.uk/metagenomics/api/v1/analyses/MGYA00022979/file/SRR995561_FASTQ_otu.tsv	Root-Host_associated-Human-Skin

ERR1760048	MGYA00140386	https://www.ebi.ac.uk/metagenomics/api/v1/analyses/MGYA00140386/file/ERR1760048_MERGED_FASTQ_SSU_OTU.tsv	Root-Host_associated-Human-Skin
ERR1960704	MGYA00130897	https://www.ebi.ac.uk/metagenomics/api/v1/analyses/MGYA00130897/file/ERR1960704_MERGED_FASTQ_otu.tsv	Root-Host_associated-Human-Skin
SRR994618	MGYA00023011	https://www.ebi.ac.uk/metagenomics/api/v1/analyses/MGYA00023011/file/SRR994618_FASTQ_otu.tsv	Root-Host_associated-Human-Skin
SRR1631066	MGYA00024806	https://www.ebi.ac.uk/metagenomics/api/v1/analyses/MGYA00024806/file/SRR1631066_MERGED_FASTQ_otu.tsv	Root-Host_associated-Human-Skin
SRR6757670	MGYA00380783	https://www.ebi.ac.uk/metagenomics/api/v1/analyses/MGYA00380783/file/SRR6757670_MERGED_FASTQ_SSU_OTU.tsv	Root-Host_associated-Human-Skin
SRR5868871	MGYA00416034	https://www.ebi.ac.uk/metagenomics/api/v1/analyses/MGYA00416034/file/SRR5868871_MERGED_FASTQ_SSU_OTU.tsv	Root-Host_associated-Human-Skin
SRR1622853	MGYA00024384	https://www.ebi.ac.uk/metagenomics/api/v1/analyses/MGYA00024384/file/SRR1622853_MERGED_FASTQ_otu.tsv	Root-Host_associated-Human-Skin
ERR2538480	MGYA00381487	https://www.ebi.ac.uk/metagenomics/api/v1/analyses/MGYA00381487/file/ERR2538480_FASTQ_SSU_OTU.tsv	Root-Host_associated-Human-Skin
ERR443105	MGYA00421095	https://www.ebi.ac.uk/metagenomics/api/v1/analyses/MGYA00421095/file/ERR443105_FASTQ_SSU_OTU.tsv	Root-Host_associated-Human-Skin
SRR1630992	MGYA00022852	https://www.ebi.ac.uk/metagenomics/api/v1/analyses/MGYA00022852/file/SRR1630992_MERGED_FASTQ_otu.tsv	Root-Host_associated-Human-Skin
ERR1759887	MGYA00140489	https://www.ebi.ac.uk/metagenomics/api/v1/analyses/MGYA00140489/file/ERR1759887_MERGED_FASTQ_SSU_OTU.tsv	Root-Host_associated-Human-Skin
SRR5868714	MGYA00420804	https://www.ebi.ac.uk/metagenomics/api/v1/analyses/MGYA00420804/file/SRR5868714_MERGED_FASTQ_SSU_OTU.tsv	Root-Host_associated-Human-Skin

SRR1704412	MGYA00428859	https://www.ebi.ac.uk/metagenomics/api/v1/analyses/MGYA00428859/file/SRR1704412_FASTQ_SSU_OTU.tsv	Root-Host_associated-Human-Skin
SRR1923894	MGYA00028372	https://www.ebi.ac.uk/metagenomics/api/v1/analyses/MGYA00028372/file/SRR1923894_FASTQ_otu.tsv	Root-Host_associated-Human-Skin
ERR443673	MGYA00424276	https://www.ebi.ac.uk/metagenomics/api/v1/analyses/MGYA00424276/file/ERR443673_FASTQ_SSU_OTU.tsv	Root-Host_associated-Human-Skin
ERR2538451	MGYA00381458	https://www.ebi.ac.uk/metagenomics/api/v1/analyses/MGYA00381458/file/ERR2538451_FASTQ_SSU_OTU.tsv	Root-Host_associated-Human-Skin
SRR1631710	MGYA00024039	https://www.ebi.ac.uk/metagenomics/api/v1/analyses/MGYA00024039/file/SRR1631710_MERGED_FASTQ_otu.tsv	Root-Host_associated-Human-Skin
SRR920743	MGYA00023539	https://www.ebi.ac.uk/metagenomics/api/v1/analyses/MGYA00023539/file/SRR920743_FASTQ_otu.tsv	Root-Host_associated-Human-Skin
ERR1701276	MGYA00084290	https://www.ebi.ac.uk/metagenomics/api/v1/analyses/MGYA00084290/file/ERR1701276_FASTQ_otu.tsv	Root-Host_associated-Human-Skin
SRR921103	MGYA00025005	https://www.ebi.ac.uk/metagenomics/api/v1/analyses/MGYA00025005/file/SRR921103_FASTQ_otu.tsv	Root-Host_associated-Human-Skin
SRR995089	MGYA00023280	https://www.ebi.ac.uk/metagenomics/api/v1/analyses/MGYA00023280/file/SRR995089_FASTQ_otu.tsv	Root-Host_associated-Human-Skin
ERR1760099	MGYA00140495	https://www.ebi.ac.uk/metagenomics/api/v1/analyses/MGYA00140495/file/ERR1760099_MERGED_FASTQ_SSU_OTU.tsv	Root-Host_associated-Human-Skin
SRR5868630	MGYA00411950	https://www.ebi.ac.uk/metagenomics/api/v1/analyses/MGYA00411950/file/SRR5868630_MERGED_FASTQ_SSU_OTU.tsv	Root-Host_associated-Human-Skin
SRR1631255	MGYA00024544	https://www.ebi.ac.uk/metagenomics/api/v1/analyses/MGYA00024544/file/SRR1631255_MERGED_FASTQ_otu.tsv	Root-Host_associated-Human-Skin

SRR1704291	MGYA00426981	https://www.ebi.ac.uk/metagenomics/api/v1/analyses/MGYA00426981/file/SRR1704291_FASTQ_SSU_OTU.tsv	Root-Host_associated-Human-Skin
ERR1759972	MGYA00140380	https://www.ebi.ac.uk/metagenomics/api/v1/analyses/MGYA00140380/file/ERR1759972_MERGED_FASTQ_SSU_OTU.tsv	Root-Host_associated-Human-Skin
SRR1950724	MGYA00376891	https://www.ebi.ac.uk/metagenomics/api/v1/analyses/MGYA00376891/file/SRR1950724_MERGED_FASTQ_SSU_OTU.tsv	Root-Host_associated-Human-Skin
SRR1632340	MGYA00025168	https://www.ebi.ac.uk/metagenomics/api/v1/analyses/MGYA00025168/file/SRR1632340_MERGED_FASTQ_otu.tsv	Root-Host_associated-Human-Skin
SRR920206	MGYA00023063	https://www.ebi.ac.uk/metagenomics/api/v1/analyses/MGYA00023063/file/SRR920206_FASTQ_otu.tsv	Root-Host_associated-Human-Skin
SRR1704157	MGYA00428946	https://www.ebi.ac.uk/metagenomics/api/v1/analyses/MGYA00428946/file/SRR1704157_FASTQ_SSU_OTU.tsv	Root-Host_associated-Human-Skin
ERR443491	MGYA00424296	https://www.ebi.ac.uk/metagenomics/api/v1/analyses/MGYA00424296/file/ERR443491_FASTQ_SSU_OTU.tsv	Root-Host_associated-Human-Skin
SRR920667	MGYA00022333	https://www.ebi.ac.uk/metagenomics/api/v1/analyses/MGYA00022333/file/SRR920667_FASTQ_otu.tsv	Root-Host_associated-Human-Skin
SRR1633161	MGYA00022384	https://www.ebi.ac.uk/metagenomics/api/v1/analyses/MGYA00022384/file/SRR1633161_MERGED_FASTQ_otu.tsv	Root-Host_associated-Human-Skin
ERR443440	MGYA00426011	https://www.ebi.ac.uk/metagenomics/api/v1/analyses/MGYA00426011/file/ERR443440_FASTQ_SSU_OTU.tsv	Root-Host_associated-Human-Skin
SRR920111	MGYA00024124	https://www.ebi.ac.uk/metagenomics/api/v1/analyses/MGYA00024124/file/SRR920111_FASTQ_otu.tsv	Root-Host_associated-Human-Skin
SRR996069	MGYA00023323	https://www.ebi.ac.uk/metagenomics/api/v1/analyses/MGYA00023323/file/SRR996069_FASTQ_otu.tsv	Root-Host_associated-Human-Skin

ERR443021	MGYA00423311	https://www.ebi.ac.uk/metagenomics/api/v1/analyses/MGYA00423311/file/ERR443021_FASTQ_SSU_OTU.tsv	Root-Host_associated-Human-Skin
SRR920902	MGYA00024094	https://www.ebi.ac.uk/metagenomics/api/v1/analyses/MGYA00024094/file/SRR920902_FASTQ_otu.tsv	Root-Host_associated-Human-Skin
SRR1923961	MGYA00028220	https://www.ebi.ac.uk/metagenomics/api/v1/analyses/MGYA00028220/file/SRR1923961_FASTQ_otu.tsv	Root-Host_associated-Human-Skin
SRR2005723	MGYA00381352	https://www.ebi.ac.uk/metagenomics/api/v1/analyses/MGYA00381352/file/SRR2005723_MERGED_FASTQ_SSU_OTU.tsv	Root-Host_associated-Human-Skin
SRR1631284	MGYA00025254	https://www.ebi.ac.uk/metagenomics/api/v1/analyses/MGYA00025254/file/SRR1631284_MERGED_FASTQ_otu.tsv	Root-Host_associated-Human-Skin
SRR1924021	MGYA00028378	https://www.ebi.ac.uk/metagenomics/api/v1/analyses/MGYA00028378/file/SRR1924021_FASTQ_otu.tsv	Root-Host_associated-Human-Skin
SRR996131	MGYA00024992	https://www.ebi.ac.uk/metagenomics/api/v1/analyses/MGYA00024992/file/SRR996131_FASTQ_otu.tsv	Root-Host_associated-Human-Skin
SRR996696	MGYA00023181	https://www.ebi.ac.uk/metagenomics/api/v1/analyses/MGYA00023181/file/SRR996696_FASTQ_otu.tsv	Root-Host_associated-Human-Skin
SRR995024	MGYA00023946	https://www.ebi.ac.uk/metagenomics/api/v1/analyses/MGYA00023946/file/SRR995024_FASTQ_otu.tsv	Root-Host_associated-Human-Skin
ERR1760034	MGYA00140535	https://www.ebi.ac.uk/metagenomics/api/v1/analyses/MGYA00140535/file/ERR1760034_MERGED_FASTQ_SSU_OTU.tsv	Root-Host_associated-Human-Skin
ERR443518	MGYA00422361	https://www.ebi.ac.uk/metagenomics/api/v1/analyses/MGYA00422361/file/ERR443518_FASTQ_SSU_OTU.tsv	Root-Host_associated-Human-Skin
ERR1960778	MGYA00130770	https://www.ebi.ac.uk/metagenomics/api/v1/analyses/MGYA00130770/file/ERR1960778_MERGED_FASTQ_otu.tsv	Root-Host_associated-Human-Skin

SRR996922	MGYA00025129	https://www.ebi.ac.uk/metagenomics/api/v1/analyses/MGYA00025129/file/SRR996922_FASTQ_otu.tsv	Root-Host_associated-Human-Skin
SRR996550	MGYA00022617	https://www.ebi.ac.uk/metagenomics/api/v1/analyses/MGYA00022617/file/SRR996550_FASTQ_otu.tsv	Root-Host_associated-Human-Skin
SRR919886	MGYA00024199	https://www.ebi.ac.uk/metagenomics/api/v1/analyses/MGYA00024199/file/SRR919886_FASTQ_otu.tsv	Root-Host_associated-Human-Skin
SRR995494	MGYA00024115	https://www.ebi.ac.uk/metagenomics/api/v1/analyses/MGYA00024115/file/SRR995494_FASTQ_otu.tsv	Root-Host_associated-Human-Skin
SRR996226	MGYA00023971	https://www.ebi.ac.uk/metagenomics/api/v1/analyses/MGYA00023971/file/SRR996226_FASTQ_otu.tsv	Root-Host_associated-Human-Skin
SRR995752	MGYA00022894	https://www.ebi.ac.uk/metagenomics/api/v1/analyses/MGYA00022894/file/SRR995752_FASTQ_otu.tsv	Root-Host_associated-Human-Skin
SRR5869549	MGYA00421625	https://www.ebi.ac.uk/metagenomics/api/v1/analyses/MGYA00421625/file/SRR5869549_MERGED_FASTQ_SSU_OTU.tsv	Root-Host_associated-Human-Skin
SRR996833	MGYA00023864	https://www.ebi.ac.uk/metagenomics/api/v1/analyses/MGYA00023864/file/SRR996833_FASTQ_otu.tsv	Root-Host_associated-Human-Skin
SRR1704209	MGYA00415672	https://www.ebi.ac.uk/metagenomics/api/v1/analyses/MGYA00415672/file/SRR1704209_FASTQ_SSU_OTU.tsv	Root-Host_associated-Human-Skin
SRR995926	MGYA00024071	https://www.ebi.ac.uk/metagenomics/api/v1/analyses/MGYA00024071/file/SRR995926_FASTQ_otu.tsv	Root-Host_associated-Human-Skin
SRR1704668	MGYA00424369	https://www.ebi.ac.uk/metagenomics/api/v1/analyses/MGYA00424369/file/SRR1704668_FASTQ_SSU_OTU.tsv	Root-Host_associated-Human-Skin
ERR441981	MGYA00385183	https://www.ebi.ac.uk/metagenomics/api/v1/analyses/MGYA00385183/file/ERR441981_FASTQ_SSU_OTU.tsv	Root-Host_associated-Human-Skin

SRR996020	MGYA00023725	https://www.ebi.ac.uk/metagenomics/api/v1/analyses/MGYA00023725/file/SRR996020_FASTQ_otu.tsv	Root-Host_associated-Human-Skin
SRR5869489	MGYA00414858	https://www.ebi.ac.uk/metagenomics/api/v1/analyses/MGYA00414858/file/SRR5869489_MERGED_FASTQ_SSU_OTU.tsv	Root-Host_associated-Human-Skin
SRR996787	MGYA00024080	https://www.ebi.ac.uk/metagenomics/api/v1/analyses/MGYA00024080/file/SRR996787_FASTQ_otu.tsv	Root-Host_associated-Human-Skin
ERR1760125	MGYA00140446	https://www.ebi.ac.uk/metagenomics/api/v1/analyses/MGYA00140446/file/ERR1760125_MERGED_FASTQ_SSU_OTU.tsv	Root-Host_associated-Human-Skin
SRR995692	MGYA00023006	https://www.ebi.ac.uk/metagenomics/api/v1/analyses/MGYA00023006/file/SRR995692_FASTQ_otu.tsv	Root-Host_associated-Human-Skin
SRR994775	MGYA00023029	https://www.ebi.ac.uk/metagenomics/api/v1/analyses/MGYA00023029/file/SRR994775_FASTQ_otu.tsv	Root-Host_associated-Human-Skin
SRR920158	MGYA00024509	https://www.ebi.ac.uk/metagenomics/api/v1/analyses/MGYA00024509/file/SRR920158_FASTQ_otu.tsv	Root-Host_associated-Human-Skin
ERR443409	MGYA00428407	https://www.ebi.ac.uk/metagenomics/api/v1/analyses/MGYA00428407/file/ERR443409_FASTQ_SSU_OTU.tsv	Root-Host_associated-Human-Skin
ERR1960669	MGYA00130887	https://www.ebi.ac.uk/metagenomics/api/v1/analyses/MGYA00130887/file/ERR1960669_MERGED_FASTQ_otu.tsv	Root-Host_associated-Human-Skin
SRR1624803	MGYA00024673	https://www.ebi.ac.uk/metagenomics/api/v1/analyses/MGYA00024673/file/SRR1624803_MERGED_FASTQ_otu.tsv	Root-Host_associated-Human-Skin
SRR995643	MGYA00024629	https://www.ebi.ac.uk/metagenomics/api/v1/analyses/MGYA00024629/file/SRR995643_FASTQ_otu.tsv	Root-Host_associated-Human-Skin
SRR5869458	MGYA00415328	https://www.ebi.ac.uk/metagenomics/api/v1/analyses/MGYA00415328/file/SRR5869458_MERGED_FASTQ_SSU_OTU.tsv	Root-Host_associated-Human-Skin

SRR997116	MGYA00024563	https://www.ebi.ac.uk/metagenomics/api/v1/analyses/MGYA00024563/file/SRR997116_FASTQ_otu.tsv	Root-Host_associated-Human-Skin
SRR995585	MGYA00022320	https://www.ebi.ac.uk/metagenomics/api/v1/analyses/MGYA00022320/file/SRR995585_FASTQ_otu.tsv	Root-Host_associated-Human-Skin
ERR442056	MGYA00385254	https://www.ebi.ac.uk/metagenomics/api/v1/analyses/MGYA00385254/file/ERR442056_FASTQ_SSU_OTU.tsv	Root-Host_associated-Human-Skin
SRR1632309	MGYA00023279	https://www.ebi.ac.uk/metagenomics/api/v1/analyses/MGYA00023279/file/SRR1632309_MERGED_FASTQ_otu.tsv	Root-Host_associated-Human-Skin
SRR5868679	MGYA00418567	https://www.ebi.ac.uk/metagenomics/api/v1/analyses/MGYA00418567/file/SRR5868679_MERGED_FASTQ_SSU_OTU.tsv	Root-Host_associated-Human-Skin
SRR996490	MGYA00023400	https://www.ebi.ac.uk/metagenomics/api/v1/analyses/MGYA00023400/file/SRR996490_FASTQ_otu.tsv	Root-Host_associated-Human-Skin
SRR5869039	MGYA00420725	https://www.ebi.ac.uk/metagenomics/api/v1/analyses/MGYA00420725/file/SRR5869039_MERGED_FASTQ_SSU_OTU.tsv	Root-Host_associated-Human-Skin
SRR919534	MGYA00024312	https://www.ebi.ac.uk/metagenomics/api/v1/analyses/MGYA00024312/file/SRR919534_FASTQ_otu.tsv	Root-Host_associated-Human-Skin
SRR919946	MGYA00024621	https://www.ebi.ac.uk/metagenomics/api/v1/analyses/MGYA00024621/file/SRR919946_FASTQ_otu.tsv	Root-Host_associated-Human-Skin
ERR2538464	MGYA00381471	https://www.ebi.ac.uk/metagenomics/api/v1/analyses/MGYA00381471/file/ERR2538464_FASTQ_SSU_OTU.tsv	Root-Host_associated-Human-Skin
SRR1631082	MGYA00023789	https://www.ebi.ac.uk/metagenomics/api/v1/analyses/MGYA00023789/file/SRR1631082_MERGED_FASTQ_otu.tsv	Root-Host_associated-Human-Skin
SRR6757694	MGYA00380807	https://www.ebi.ac.uk/metagenomics/api/v1/analyses/MGYA00380807/file/SRR6757694_MERGED_FASTQ_SSU_OTU.tsv	Root-Host_associated-Human-Skin

ERR1701243	MGYA00084312	https://www.ebi.ac.uk/metagenomics/api/v1/analyses/MGYA00084312/file/ERR1701243_FASTQ_otu.tsv	Root-Host_associated-Human-Skin
SRR921136	MGYA00022300	https://www.ebi.ac.uk/metagenomics/api/v1/analyses/MGYA00022300/file/SRR921136_FASTQ_otu.tsv	Root-Host_associated-Human-Skin
SRR1631344	MGYA00022811	https://www.ebi.ac.uk/metagenomics/api/v1/analyses/MGYA00022811/file/SRR1631344_MERGED_FASTQ_otu.tsv	Root-Host_associated-Human-Skin
SRR1630976	MGYA00024348	https://www.ebi.ac.uk/metagenomics/api/v1/analyses/MGYA00024348/file/SRR1630976_MERGED_FASTQ_otu.tsv	Root-Host_associated-Human-Skin
SRR1632984	MGYA00025156	https://www.ebi.ac.uk/metagenomics/api/v1/analyses/MGYA00025156/file/SRR1632984_MERGED_FASTQ_otu.tsv	Root-Host_associated-Human-Skin
SRR994948	MGYA00022493	https://www.ebi.ac.uk/metagenomics/api/v1/analyses/MGYA00022493/file/SRR994948_FASTQ_otu.tsv	Root-Host_associated-Human-Skin
ERR443130	MGYA00416308	https://www.ebi.ac.uk/metagenomics/api/v1/analyses/MGYA00416308/file/ERR443130_FASTQ_SSU_OTU.tsv	Root-Host_associated-Human-Skin
SRR994999	MGYA00023936	https://www.ebi.ac.uk/metagenomics/api/v1/analyses/MGYA00023936/file/SRR994999_FASTQ_otu.tsv	Root-Host_associated-Human-Skin
ERR443697	MGYA00426001	https://www.ebi.ac.uk/metagenomics/api/v1/analyses/MGYA00426001/file/ERR443697_FASTQ_SSU_OTU.tsv	Root-Host_associated-Human-Skin
SRR1923870	MGYA00028256	https://www.ebi.ac.uk/metagenomics/api/v1/analyses/MGYA00028256/file/SRR1923870_FASTQ_otu.tsv	Root-Host_associated-Human-Skin
SRR996519	MGYA00023067	https://www.ebi.ac.uk/metagenomics/api/v1/analyses/MGYA00023067/file/SRR996519_FASTQ_otu.tsv	Root-Host_associated-Human-Skin
SRR1631395	MGYA00022750	https://www.ebi.ac.uk/metagenomics/api/v1/analyses/MGYA00022750/file/SRR1631395_MERGED_FASTQ_otu.tsv	Root-Host_associated-Human-Skin

ERR451572	MGYA00426501	https://www.ebi.ac.uk/metagenomics/api/v1/analyses/MGYA00426501/file/ERR451572_FASTQ_SSU_OTU.tsv	Root-Host_associated-Human-Skin
ERR1701292	MGYA00084279	https://www.ebi.ac.uk/metagenomics/api/v1/analyses/MGYA00084279/file/ERR1701292_FASTQ_otu.tsv	Root-Host_associated-Human-Skin
ERR1960731	MGYA00130815	https://www.ebi.ac.uk/metagenomics/api/v1/analyses/MGYA00130815/file/ERR1960731_MERGED_FASTQ_otu.tsv	Root-Host_associated-Human-Skin
SRR6757645	MGYA00380760	https://www.ebi.ac.uk/metagenomics/api/v1/analyses/MGYA00380760/file/SRR6757645_MERGED_FASTQ_SSU_OTU.tsv	Root-Host_associated-Human-Skin
SRR5868844	MGYA00414884	https://www.ebi.ac.uk/metagenomics/api/v1/analyses/MGYA00414884/file/SRR5868844_MERGED_FASTQ_SSU_OTU.tsv	Root-Host_associated-Human-Skin
SRR1704730	MGYA00426574	https://www.ebi.ac.uk/metagenomics/api/v1/analyses/MGYA00426574/file/SRR1704730_FASTQ_SSU_OTU.tsv	Root-Host_associated-Human-Skin
SRR1632403	MGYA00023552	https://www.ebi.ac.uk/metagenomics/api/v1/analyses/MGYA00023552/file/SRR1632403_MERGED_FASTQ_otu.tsv	Root-Host_associated-Human-Skin
SRR920937	MGYA00022572	https://www.ebi.ac.uk/metagenomics/api/v1/analyses/MGYA00022572/file/SRR920937_FASTQ_otu.tsv	Root-Host_associated-Human-Skin
SRR995528	MGYA00023622	https://www.ebi.ac.uk/metagenomics/api/v1/analyses/MGYA00023622/file/SRR995528_FASTQ_otu.tsv	Root-Host_associated-Human-Skin
ERR451037	MGYA00428411	https://www.ebi.ac.uk/metagenomics/api/v1/analyses/MGYA00428411/file/ERR451037_FASTQ_SSU_OTU.tsv	Root-Host_associated-Human-Skin
SRR919599	MGYA00024956	https://www.ebi.ac.uk/metagenomics/api/v1/analyses/MGYA00024956/file/SRR919599_FASTQ_otu.tsv	Root-Host_associated-Human-Skin
SRR2005716	MGYA00381345	https://www.ebi.ac.uk/metagenomics/api/v1/analyses/MGYA00381345/file/SRR2005716_MERGED_FASTQ_SSU_OTU.tsv	Root-Host_associated-Human-Skin

ERR1759996	MGYA00140414	https://www.ebi.ac.uk/metagenomics/api/v1/analyses/MGYA00140414/file/ERR1759996_MERGED_FASTQ_SSU_OTU.tsv	Root-Host_associated-Human-Skin
SRR1631516	MGYA00023383	https://www.ebi.ac.uk/metagenomics/api/v1/analyses/MGYA00023383/file/SRR1631516_MERGED_FASTQ_otu.tsv	Root-Host_associated-Human-Skin
ERR451456	MGYA00413338	https://www.ebi.ac.uk/metagenomics/api/v1/analyses/MGYA00413338/file/ERR451456_FASTQ_SSU_OTU.tsv	Root-Host_associated-Human-Skin
SRR995149	MGYA00024143	https://www.ebi.ac.uk/metagenomics/api/v1/analyses/MGYA00024143/file/SRR995149_FASTQ_otu.tsv	Root-Host_associated-Human-Skin
SRR994709	MGYA00024494	https://www.ebi.ac.uk/metagenomics/api/v1/analyses/MGYA00024494/file/SRR994709_FASTQ_otu.tsv	Root-Host_associated-Human-Skin
SRR5868960	MGYA00426649	https://www.ebi.ac.uk/metagenomics/api/v1/analyses/MGYA00426649/file/SRR5868960_MERGED_FASTQ_SSU_OTU.tsv	Root-Host_associated-Human-Skin
SRR1704162	MGYA00422449	https://www.ebi.ac.uk/metagenomics/api/v1/analyses/MGYA00422449/file/SRR1704162_FASTQ_SSU_OTU.tsv	Root-Host_associated-Human-Skin
ERR450912	MGYA00413371	https://www.ebi.ac.uk/metagenomics/api/v1/analyses/MGYA00413371/file/ERR450912_FASTQ_SSU_OTU.tsv	Root-Host_associated-Human-Skin
SRR920652	MGYA00025211	https://www.ebi.ac.uk/metagenomics/api/v1/analyses/MGYA00025211/file/SRR920652_FASTQ_otu.tsv	Root-Host_associated-Human-Skin
SRR921012	MGYA00023325	https://www.ebi.ac.uk/metagenomics/api/v1/analyses/MGYA00023325/file/SRR921012_FASTQ_otu.tsv	Root-Host_associated-Human-Skin
ERR451320	MGYA00424890	https://www.ebi.ac.uk/metagenomics/api/v1/analyses/MGYA00424890/file/ERR451320_FASTQ_SSU_OTU.tsv	Root-Host_associated-Human-Skin
ERR1759947	MGYA00140407	https://www.ebi.ac.uk/metagenomics/api/v1/analyses/MGYA00140407/file/ERR1759947_MERGED_FASTQ_SSU_OTU.tsv	Root-Host_associated-Human-Skin

SRR1623067	MGYA00024413	https://www.ebi.ac.uk/metagenomics/api/v1/analyses/MGYA00024413/file/SRR1623067_MERGED_FASTQ_otu.tsv	Root-Host_associated-Human-Skin
SRR1704503	MGYA00417842	https://www.ebi.ac.uk/metagenomics/api/v1/analyses/MGYA00417842/file/SRR1704503_FASTQ_SSU_OTU.tsv	Root-Host_associated-Human-Skin
SRR5868605	MGYA00428113	https://www.ebi.ac.uk/metagenomics/api/v1/analyses/MGYA00428113/file/SRR5868605_MERGED_FASTQ_SSU_OTU.tsv	Root-Host_associated-Human-Skin
SRR5869045	MGYA00416023	https://www.ebi.ac.uk/metagenomics/api/v1/analyses/MGYA00416023/file/SRR5869045_MERGED_FASTQ_SSU_OTU.tsv	Root-Host_associated-Human-Skin
SRR1950711	MGYA00376878	https://www.ebi.ac.uk/metagenomics/api/v1/analyses/MGYA00376878/file/SRR1950711_MERGED_FASTQ_SSU_OTU.tsv	Root-Host_associated-Human-Skin
ERR443762	MGYA00415095	https://www.ebi.ac.uk/metagenomics/api/v1/analyses/MGYA00415095/file/ERR443762_FASTQ_SSU_OTU.tsv	Root-Host_associated-Human-Skin
ERR2538418	MGYA00381425	https://www.ebi.ac.uk/metagenomics/api/v1/analyses/MGYA00381425/file/ERR2538418_FASTQ_SSU_OTU.tsv	Root-Host_associated-Human-Skin
SRR997032	MGYA00023606	https://www.ebi.ac.uk/metagenomics/api/v1/analyses/MGYA00023606/file/SRR997032_FASTQ_otu.tsv	Root-Host_associated-Human-Skin
SRR995306	MGYA00022701	https://www.ebi.ac.uk/metagenomics/api/v1/analyses/MGYA00022701/file/SRR995306_FASTQ_otu.tsv	Root-Host_associated-Human-Skin
SRR994546	MGYA00024261	https://www.ebi.ac.uk/metagenomics/api/v1/analyses/MGYA00024261/file/SRR994546_FASTQ_otu.tsv	Root-Host_associated-Human-Skin
SRR994934	MGYA00024958	https://www.ebi.ac.uk/metagenomics/api/v1/analyses/MGYA00024958/file/SRR994934_FASTQ_otu.tsv	Root-Host_associated-Human-Skin
SRR1922548	MGYA00028399	https://www.ebi.ac.uk/metagenomics/api/v1/analyses/MGYA00028399/file/SRR1922548_FASTQ_otu.tsv	Root-Host_associated-Human-Skin

ERR1960828	MGYA00130841	https://www.ebi.ac.uk/metagenomics/api/v1/analyses/MGYA00130841/file/ERR1960828_MERGED_FASTQ_otu.tsv	Root-Host_associated-Human-Skin
SRR919862	MGYA00023903	https://www.ebi.ac.uk/metagenomics/api/v1/analyses/MGYA00023903/file/SRR919862_FASTQ_otu.tsv	Root-Host_associated-Human-Skin
SRR1950698	MGYA00376865	https://www.ebi.ac.uk/metagenomics/api/v1/analyses/MGYA00376865/file/SRR1950698_MERGED_FASTQ_SSU_OTU.tsv	Root-Host_associated-Human-Skin
SRR996917	MGYA00022444	https://www.ebi.ac.uk/metagenomics/api/v1/analyses/MGYA00022444/file/SRR996917_FASTQ_otu.tsv	Root-Host_associated-Human-Skin
SRR996565	MGYA00025300	https://www.ebi.ac.uk/metagenomics/api/v1/analyses/MGYA00025300/file/SRR996565_FASTQ_otu.tsv	Root-Host_associated-Human-Skin
SRR995470	MGYA00025104	https://www.ebi.ac.uk/metagenomics/api/v1/analyses/MGYA00025104/file/SRR995470_FASTQ_otu.tsv	Root-Host_associated-Human-Skin
SRR6757639	MGYA00380754	https://www.ebi.ac.uk/metagenomics/api/v1/analyses/MGYA00380754/file/SRR6757639_MERGED_FASTQ_SSU_OTU.tsv	Root-Host_associated-Human-Skin
SRR1924014	MGYA00028390	https://www.ebi.ac.uk/metagenomics/api/v1/analyses/MGYA00028390/file/SRR1924014_FASTQ_otu.tsv	Root-Host_associated-Human-Skin
SRR5868838	MGYA00422568	https://www.ebi.ac.uk/metagenomics/api/v1/analyses/MGYA00422568/file/SRR5868838_MERGED_FASTQ_SSU_OTU.tsv	Root-Host_associated-Human-Skin
ERR1760001	MGYA00140584	https://www.ebi.ac.uk/metagenomics/api/v1/analyses/MGYA00140584/file/ERR1760001_MERGED_FASTQ_SSU_OTU.tsv	Root-Host_associated-Human-Skin
SRR994651	MGYA00023448	https://www.ebi.ac.uk/metagenomics/api/v1/analyses/MGYA00023448/file/SRR994651_FASTQ_otu.tsv	Root-Host_associated-Human-Skin
SRR1923953	MGYA00028347	https://www.ebi.ac.uk/metagenomics/api/v1/analyses/MGYA00028347/file/SRR1923953_FASTQ_otu.tsv	Root-Host_associated-Human-Skin

SRR920930	MGYA00022579	https://www.ebi.ac.uk/metagenomics/api/v1/analyses/MGYA00022579/file/SRR920930_FASTQ_otu.tsv	Root-Host_associated-Human-Skin
SRR1632404	MGYA00023547	https://www.ebi.ac.uk/metagenomics/api/v1/analyses/MGYA00023547/file/SRR1632404_MERGED_FASTQ_otu.tsv	Root-Host_associated-Human-Skin
ERR1759991	MGYA00140530	https://www.ebi.ac.uk/metagenomics/api/v1/analyses/MGYA00140530/file/ERR1759991_MERGED_FASTQ_SSU_OTU.tsv	Root-Host_associated-Human-Skin
SRR1631511	MGYA00023382	https://www.ebi.ac.uk/metagenomics/api/v1/analyses/MGYA00023382/file/SRR1631511_MERGED_FASTQ_otu.tsv	Root-Host_associated-Human-Skin
ERR443472	MGYA00422026	https://www.ebi.ac.uk/metagenomics/api/v1/analyses/MGYA00422026/file/ERR443472_FASTQ_SSU_OTU.tsv	Root-Host_associated-Human-Skin
SRR5869880	MGYA00415854	https://www.ebi.ac.uk/metagenomics/api/v1/analyses/MGYA00415854/file/SRR5869880_MERGED_FASTQ_SSU_OTU.tsv	Root-Host_associated-Human-Skin
SRR1704165	MGYA00426599	https://www.ebi.ac.uk/metagenomics/api/v1/analyses/MGYA00426599/file/SRR1704165_FASTQ_SSU_OTU.tsv	Root-Host_associated-Human-Skin
SRR995638	MGYA00022337	https://www.ebi.ac.uk/metagenomics/api/v1/analyses/MGYA00022337/file/SRR995638_FASTQ_otu.tsv	Root-Host_associated-Human-Skin
SRR921015	MGYA00023330	https://www.ebi.ac.uk/metagenomics/api/v1/analyses/MGYA00023330/file/SRR921015_FASTQ_otu.tsv	Root-Host_associated-Human-Skin
SRR920655	MGYA00025218	https://www.ebi.ac.uk/metagenomics/api/v1/analyses/MGYA00025218/file/SRR920655_FASTQ_otu.tsv	Root-Host_associated-Human-Skin
SRR1950716	MGYA00376883	https://www.ebi.ac.uk/metagenomics/api/v1/analyses/MGYA00376883/file/SRR1950716_MERGED_FASTQ_SSU_OTU.tsv	Root-Host_associated-Human-Skin
SRR996899	MGYA00022802	https://www.ebi.ac.uk/metagenomics/api/v1/analyses/MGYA00022802/file/SRR996899_FASTQ_otu.tsv	Root-Host_associated-Human-Skin

SRR5868602	MGYA00428115	https://www.ebi.ac.uk/metagenomics/api/v1/analyses/MGYA00428115/file/SRR5868602_MERGED_FASTQ_SSU_OTU.tsv	Root-Host_associated-Human-Skin
ERR1759940	MGYA00140355	https://www.ebi.ac.uk/metagenomics/api/v1/analyses/MGYA00140355/file/ERR1759940_MERGED_FASTQ_SSU_OTU.tsv	Root-Host_associated-Human-Skin
SRR1623060	MGYA00024416	https://www.ebi.ac.uk/metagenomics/api/v1/analyses/MGYA00024416/file/SRR1623060_MERGED_FASTQ_otu.tsv	Root-Host_associated-Human-Skin
ERR443765	MGYA00420467	https://www.ebi.ac.uk/metagenomics/api/v1/analyses/MGYA00420467/file/ERR443765_FASTQ_SSU_OTU.tsv	Root-Host_associated-Human-Skin
SRR920234	MGYA00023387	https://www.ebi.ac.uk/metagenomics/api/v1/analyses/MGYA00023387/file/SRR920234_FASTQ_otu.tsv	Root-Host_associated-Human-Skin
ERR1701238	MGYA00084288	https://www.ebi.ac.uk/metagenomics/api/v1/analyses/MGYA00084288/file/ERR1701238_FASTQ_otu.tsv	Root-Host_associated-Human-Skin
SRR997035	MGYA00023601	https://www.ebi.ac.uk/metagenomics/api/v1/analyses/MGYA00023601/file/SRR997035_FASTQ_otu.tsv	Root-Host_associated-Human-Skin
SRR994933	MGYA00024965	https://www.ebi.ac.uk/metagenomics/api/v1/analyses/MGYA00024965/file/SRR994933_FASTQ_otu.tsv	Root-Host_associated-Human-Skin
SRR994541	MGYA00024262	https://www.ebi.ac.uk/metagenomics/api/v1/analyses/MGYA00024262/file/SRR994541_FASTQ_otu.tsv	Root-Host_associated-Human-Skin
SRR995301	MGYA00022696	https://www.ebi.ac.uk/metagenomics/api/v1/analyses/MGYA00022696/file/SRR995301_FASTQ_otu.tsv	Root-Host_associated-Human-Skin
SRR2005698	MGYA00381335	https://www.ebi.ac.uk/metagenomics/api/v1/analyses/MGYA00381335/file/SRR2005698_MERGED_FASTQ_SSU_OTU.tsv	Root-Host_associated-Human-Skin
SRR919865	MGYA00023896	https://www.ebi.ac.uk/metagenomics/api/v1/analyses/MGYA00023896/file/SRR919865_FASTQ_otu.tsv	Root-Host_associated-Human-Skin

SRR1631498	MGYA00024721	https://www.ebi.ac.uk/metagenomics/api/v1/analyses/MGYA00024721/file/SRR1631498_MERGED_FASTQ_otu.tsv	Root-Host_associated-Human-Skin
SRR2005649	MGYA00381308	https://www.ebi.ac.uk/metagenomics/api/v1/analyses/MGYA00381308/file/SRR2005649_MERGED_FASTQ_SSU_OTU.tsv	Root-Host_associated-Human-Skin
SRR996562	MGYA00025299	https://www.ebi.ac.uk/metagenomics/api/v1/analyses/MGYA00025299/file/SRR996562_FASTQ_otu.tsv	Root-Host_associated-Human-Skin
SRR996910	MGYA00022437	https://www.ebi.ac.uk/metagenomics/api/v1/analyses/MGYA00022437/file/SRR996910_FASTQ_otu.tsv	Root-Host_associated-Human-Skin
SRR995477	MGYA00025099	https://www.ebi.ac.uk/metagenomics/api/v1/analyses/MGYA00025099/file/SRR995477_FASTQ_otu.tsv	Root-Host_associated-Human-Skin
ERR451168	MGYA00412631	https://www.ebi.ac.uk/metagenomics/api/v1/analyses/MGYA00412631/file/ERR451168_FASTQ_SSU_OTU.tsv	Root-Host_associated-Human-Skin
ERR2538369	MGYA00381376	https://www.ebi.ac.uk/metagenomics/api/v1/analyses/MGYA00381376/file/ERR2538369_FASTQ_SSU_OTU.tsv	Root-Host_associated-Human-Skin
SRR1924013	MGYA00028389	https://www.ebi.ac.uk/metagenomics/api/v1/analyses/MGYA00028389/file/SRR1924013_FASTQ_otu.tsv	Root-Host_associated-Human-Skin
SRR994656	MGYA00023447	https://www.ebi.ac.uk/metagenomics/api/v1/analyses/MGYA00023447/file/SRR994656_FASTQ_otu.tsv	Root-Host_associated-Human-Skin
ERR1760006	MGYA00140371	https://www.ebi.ac.uk/metagenomics/api/v1/analyses/MGYA00140371/file/ERR1760006_MERGED_FASTQ_SSU_OTU.tsv	Root-Host_associated-Human-Skin
SRR1920448	MGYA00028280	https://www.ebi.ac.uk/metagenomics/api/v1/analyses/MGYA00028280/file/SRR1920448_FASTQ_otu.tsv	Root-Host_associated-Human-Skin
SRR996834	MGYA00023871	https://www.ebi.ac.uk/metagenomics/api/v1/analyses/MGYA00023871/file/SRR996834_FASTQ_otu.tsv	Root-Host_associated-Human-Skin

SRR995921	MGYA00024066	https://www.ebi.ac.uk/metagenomics/api/v1/analyses/MGYA00024066/file/SRR995921_FASTQ_otu.tsv	Root-Host_associated-Human-Skin
SRR996780	MGYA00024075	https://www.ebi.ac.uk/metagenomics/api/v1/analyses/MGYA00024075/file/SRR996780_FASTQ_otu.tsv	Root-Host_associated-Human-Skin
SRR996027	MGYA00023724	https://www.ebi.ac.uk/metagenomics/api/v1/analyses/MGYA00023724/file/SRR996027_FASTQ_otu.tsv	Root-Host_associated-Human-Skin
ERR441986	MGYA00385229	https://www.ebi.ac.uk/metagenomics/api/v1/analyses/MGYA00385229/file/ERR441986_FASTQ_SSU_OTU.tsv	Root-Host_associated-Human-Skin
SRR5870142	MGYA00414807	https://www.ebi.ac.uk/metagenomics/api/v1/analyses/MGYA00414807/file/SRR5870142_MERGED_FASTQ_SSU_OTU.tsv	Root-Host_associated-Human-Skin
SRR995695	MGYA00023005	https://www.ebi.ac.uk/metagenomics/api/v1/analyses/MGYA00023005/file/SRR995695_FASTQ_otu.tsv	Root-Host_associated-Human-Skin
ERR1760122	MGYA00140515	https://www.ebi.ac.uk/metagenomics/api/v1/analyses/MGYA00140515/file/ERR1760122_MERGED_FASTQ_SSU_OTU.tsv	Root-Host_associated-Human-Skin
SRR995644	MGYA00024632	https://www.ebi.ac.uk/metagenomics/api/v1/analyses/MGYA00024632/file/SRR995644_FASTQ_otu.tsv	Root-Host_associated-Human-Skin
SRR1624804	MGYA00024670	https://www.ebi.ac.uk/metagenomics/api/v1/analyses/MGYA00024670/file/SRR1624804_MERGED_FASTQ_otu.tsv	Root-Host_associated-Human-Skin
SRR920629	MGYA00022769	https://www.ebi.ac.uk/metagenomics/api/v1/analyses/MGYA00022769/file/SRR920629_FASTQ_otu.tsv	Root-Host_associated-Human-Skin
ERR450969	MGYA00412391	https://www.ebi.ac.uk/metagenomics/api/v1/analyses/MGYA00412391/file/ERR450969_FASTQ_SSU_OTU.tsv	Root-Host_associated-Human-Skin
SRR997111	MGYA00024560	https://www.ebi.ac.uk/metagenomics/api/v1/analyses/MGYA00024560/file/SRR997111_FASTQ_otu.tsv	Root-Host_associated-Human-Skin

SRR920248	MGYA00023502	https://www.ebi.ac.uk/metagenomics/api/v1/analyses/MGYA00023502/file/SRR920248_FASTQ_otu.tsv	Root-Host_associated-Human-Skin
SRR5870255	MGYA00423985	https://www.ebi.ac.uk/metagenomics/api/v1/analyses/MGYA00423985/file/SRR5870255_MERGED_FASTQ_SSU_OTU.tsv	Root-Host_associated-Human-Skin
ERR442051	MGYA00385202	https://www.ebi.ac.uk/metagenomics/api/v1/analyses/MGYA00385202/file/ERR442051_FASTQ_SSU_OTU.tsv	Root-Host_associated-Human-Skin
SRR5869799	MGYA00415879	https://www.ebi.ac.uk/metagenomics/api/v1/analyses/MGYA00415879/file/SRR5869799_MERGED_FASTQ_SSU_OTU.tsv	Root-Host_associated-Human-Skin
SRR919941	MGYA00024620	https://www.ebi.ac.uk/metagenomics/api/v1/analyses/MGYA00024620/file/SRR919941_FASTQ_otu.tsv	Root-Host_associated-Human-Skin
SRR919533	MGYA00024313	https://www.ebi.ac.uk/metagenomics/api/v1/analyses/MGYA00024313/file/SRR919533_FASTQ_otu.tsv	Root-Host_associated-Human-Skin
SRR996497	MGYA00023405	https://www.ebi.ac.uk/metagenomics/api/v1/analyses/MGYA00023405/file/SRR996497_FASTQ_otu.tsv	Root-Host_associated-Human-Skin
SRR1631085	MGYA00023784	https://www.ebi.ac.uk/metagenomics/api/v1/analyses/MGYA00023784/file/SRR1631085_MERGED_FASTQ_otu.tsv	Root-Host_associated-Human-Skin
SRR6757693	MGYA00380806	https://www.ebi.ac.uk/metagenomics/api/v1/analyses/MGYA00380806/file/SRR6757693_MERGED_FASTQ_SSU_OTU.tsv	Root-Host_associated-Human-Skin
SRR996609	MGYA00024580	https://www.ebi.ac.uk/metagenomics/api/v1/analyses/MGYA00024580/file/SRR996609_FASTQ_otu.tsv	Root-Host_associated-Human-Skin
ERR2538463	MGYA00381470	https://www.ebi.ac.uk/metagenomics/api/v1/analyses/MGYA00381470/file/ERR2538463_FASTQ_SSU_OTU.tsv	Root-Host_associated-Human-Skin
SRR921131	MGYA00022303	https://www.ebi.ac.uk/metagenomics/api/v1/analyses/MGYA00022303/file/SRR921131_FASTQ_otu.tsv	Root-Host_associated-Human-Skin

ERR451203	MGYA00426071	https://www.ebi.ac.uk/metagenomics/api/v1/analyses/MGYA00426071/file/ERR451203_FASTQ_SSU_OTU.tsv	Root-Host_associated-Human-Skin
ERR443587	MGYA00421392	https://www.ebi.ac.uk/metagenomics/api/v1/analyses/MGYA00421392/file/ERR443587_FASTQ_SSU_OTU.tsv	Root-Host_associated-Human-Skin
ERR1701244	MGYA00084332	https://www.ebi.ac.uk/metagenomics/api/v1/analyses/MGYA00084332/file/ERR1701244_FASTQ_otu.tsv	Root-Host_associated-Human-Skin
SRR996268	MGYA00023511	https://www.ebi.ac.uk/metagenomics/api/v1/analyses/MGYA00023511/file/SRR996268_FASTQ_otu.tsv	Root-Host_associated-Human-Skin
SRR1630971	MGYA00024353	https://www.ebi.ac.uk/metagenomics/api/v1/analyses/MGYA00024353/file/SRR1630971_MERGED_FASTQ_otu.tsv	Root-Host_associated-Human-Skin
SRR1704387	MGYA00413399	https://www.ebi.ac.uk/metagenomics/api/v1/analyses/MGYA00413399/file/SRR1704387_FASTQ_SSU_OTU.tsv	Root-Host_associated-Human-Skin
SRR1631343	MGYA00022804	https://www.ebi.ac.uk/metagenomics/api/v1/analyses/MGYA00022804/file/SRR1631343_MERGED_FASTQ_otu.tsv	Root-Host_associated-Human-Skin
ERR443641	MGYA00419146	https://www.ebi.ac.uk/metagenomics/api/v1/analyses/MGYA00419146/file/ERR443641_FASTQ_SSU_OTU.tsv	Root-Host_associated-Human-Skin
SRR1632983	MGYA00025155	https://www.ebi.ac.uk/metagenomics/api/v1/analyses/MGYA00025155/file/SRR1632983_MERGED_FASTQ_otu.tsv	Root-Host_associated-Human-Skin
SRR1923877	MGYA00028348	https://www.ebi.ac.uk/metagenomics/api/v1/analyses/MGYA00028348/file/SRR1923877_FASTQ_otu.tsv	Root-Host_associated-Human-Skin
ERR443690	MGYA00420473	https://www.ebi.ac.uk/metagenomics/api/v1/analyses/MGYA00420473/file/ERR443690_FASTQ_SSU_OTU.tsv	Root-Host_associated-Human-Skin
ERR450754	MGYA00413728	https://www.ebi.ac.uk/metagenomics/api/v1/analyses/MGYA00413728/file/ERR450754_FASTQ_SSU_OTU.tsv	Root-Host_associated-Human-Skin

SRR1704356	MGYA00425325	https://www.ebi.ac.uk/metagenomics/api/v1/analyses/MGYA00425325/file/SRR1704356_FASTQ_SSU_OTU.tsv	Root-Host_associated-Human-Skin
SRR1631435	MGYA00025317	https://www.ebi.ac.uk/metagenomics/api/v1/analyses/MGYA00025317/file/SRR1631435_MERGED_FASTQ_otu.tsv	Root-Host_associated-Human-Skin
SRR1631392	MGYA00022737	https://www.ebi.ac.uk/metagenomics/api/v1/analyses/MGYA00022737/file/SRR1631392_MERGED_FASTQ_otu.tsv	Root-Host_associated-Human-Skin
SRR2005635	MGYA00381295	https://www.ebi.ac.uk/metagenomics/api/v1/analyses/MGYA00381295/file/SRR2005635_MERGED_FASTQ_SSU_OTU.tsv	Root-Host_associated-Human-Skin
ERR1960736	MGYA00130772	https://www.ebi.ac.uk/metagenomics/api/v1/analyses/MGYA00130772/file/ERR1960736_MERGED_FASTQ_otu.tsv	Root-Host_associated-Human-Skin
ERR1701295	MGYA00084267	https://www.ebi.ac.uk/metagenomics/api/v1/analyses/MGYA00084267/file/ERR1701295_FASTQ_otu.tsv	Root-Host_associated-Human-Skin
SRR997098	MGYA00022518	https://www.ebi.ac.uk/metagenomics/api/v1/analyses/MGYA00022518/file/SRR997098_FASTQ_otu.tsv	Root-Host_associated-Human-Skin
SRR5868843	MGYA00425844	https://www.ebi.ac.uk/metagenomics/api/v1/analyses/MGYA00425844/file/SRR5868843_MERGED_FASTQ_SSU_OTU.tsv	Root-Host_associated-Human-Skin
SRR6757642	MGYA00380757	https://www.ebi.ac.uk/metagenomics/api/v1/analyses/MGYA00380757/file/SRR6757642_MERGED_FASTQ_SSU_OTU.tsv	Root-Host_associated-Human-Skin
SRR1704737	MGYA00413069	https://www.ebi.ac.uk/metagenomics/api/v1/analyses/MGYA00413069/file/SRR1704737_FASTQ_SSU_OTU.tsv	Root-Host_associated-Human-Skin
SRR996434	MGYA00024475	https://www.ebi.ac.uk/metagenomics/api/v1/analyses/MGYA00024475/file/SRR996434_FASTQ_otu.tsv	Root-Host_associated-Human-Skin
SRR996393	MGYA00024977	https://www.ebi.ac.uk/metagenomics/api/v1/analyses/MGYA00024977/file/SRR996393_FASTQ_otu.tsv	Root-Host_associated-Human-Skin

SRR919590	MGYA00024949	https://www.ebi.ac.uk/metagenomics/api/v1/analyses/MGYA00024949/file/SRR919590_FASTQ_otu.tsv	Root-Host_associated-Human-Skin
SRR995521	MGYA00023423	https://www.ebi.ac.uk/metagenomics/api/v1/analyses/MGYA00023423/file/SRR995521_FASTQ_otu.tsv	Root-Host_associated-Human-Skin
SRR995140	MGYA00024140	https://www.ebi.ac.uk/metagenomics/api/v1/analyses/MGYA00024140/file/SRR995140_FASTQ_otu.tsv	Root-Host_associated-Human-Skin
SRR5870130	MGYA00424473	https://www.ebi.ac.uk/metagenomics/api/v1/analyses/MGYA00424473/file/SRR5870130_MERGED_FASTQ_SSU_OTU.tsv	Root-Host_associated-Human-Skin
SRR994700	MGYA00024483	https://www.ebi.ac.uk/metagenomics/api/v1/analyses/MGYA00024483/file/SRR994700_FASTQ_otu.tsv	Root-Host_associated-Human-Skin
SRR995636	MGYA00022343	https://www.ebi.ac.uk/metagenomics/api/v1/analyses/MGYA00022343/file/SRR995636_FASTQ_otu.tsv	Root-Host_associated-Human-Skin
SRR996084	MGYA00024362	https://www.ebi.ac.uk/metagenomics/api/v1/analyses/MGYA00024362/file/SRR996084_FASTQ_otu.tsv	Root-Host_associated-Human-Skin
SRR996723	MGYA00023430	https://www.ebi.ac.uk/metagenomics/api/v1/analyses/MGYA00023430/file/SRR996723_FASTQ_otu.tsv	Root-Host_associated-Human-Skin
ERR442023	MGYA00385217	https://www.ebi.ac.uk/metagenomics/api/v1/analyses/MGYA00385217/file/ERR442023_FASTQ_SSU_OTU.tsv	Root-Host_associated-Human-Skin
SRR995982	MGYA00022933	https://www.ebi.ac.uk/metagenomics/api/v1/analyses/MGYA00022933/file/SRR995982_FASTQ_otu.tsv	Root-Host_associated-Human-Skin
SRR994865	MGYA00022395	https://www.ebi.ac.uk/metagenomics/api/v1/analyses/MGYA00022395/file/SRR994865_FASTQ_otu.tsv	Root-Host_associated-Human-Skin
SRR996897	MGYA00022796	https://www.ebi.ac.uk/metagenomics/api/v1/analyses/MGYA00022796/file/SRR996897_FASTQ_otu.tsv	Root-Host_associated-Human-Skin

SRR1950718	MGYA00376885	https://www.ebi.ac.uk/metagenomics/api/v1/analyses/MGYA00376885/file/SRR1950718_MERGED_FASTQ_SSU_OTU.tsv	Root-Host_associated-Human-Skin
SRR996342	MGYA00022279	https://www.ebi.ac.uk/metagenomics/api/v1/analyses/MGYA00022279/file/SRR996342_FASTQ_otu.tsv	Root-Host_associated-Human-Skin
SRR1920899	MGYA00028266	https://www.ebi.ac.uk/metagenomics/api/v1/analyses/MGYA00028266/file/SRR1920899_FASTQ_otu.tsv	Root-Host_associated-Human-Skin
SRR5869907	MGYA00416442	https://www.ebi.ac.uk/metagenomics/api/v1/analyses/MGYA00416442/file/SRR5869907_MERGED_FASTQ_SSU_OTU.tsv	Root-Host_associated-Human-Skin
ERR2538411	MGYA00381418	https://www.ebi.ac.uk/metagenomics/api/v1/analyses/MGYA00381418/file/ERR2538411_FASTQ_SSU_OTU.tsv	Root-Host_associated-Human-Skin
ERR1701236	MGYA00084255	https://www.ebi.ac.uk/metagenomics/api/v1/analyses/MGYA00084255/file/ERR1701236_FASTQ_otu.tsv	Root-Host_associated-Human-Skin
ERR1960795	MGYA00130827	https://www.ebi.ac.uk/metagenomics/api/v1/analyses/MGYA00130827/file/ERR1960795_MERGED_FASTQ_otu.tsv	Root-Host_associated-Human-Skin
ERR443252	MGYA00425770	https://www.ebi.ac.uk/metagenomics/api/v1/analyses/MGYA00425770/file/ERR443252_FASTQ_SSU_OTU.tsv	Root-Host_associated-Human-Skin
SRR921143	MGYA00024587	https://www.ebi.ac.uk/metagenomics/api/v1/analyses/MGYA00024587/file/SRR921143_FASTQ_otu.tsv	Root-Host_associated-Human-Skin
SRR1704452	MGYA00426931	https://www.ebi.ac.uk/metagenomics/api/v1/analyses/MGYA00426931/file/SRR1704452_FASTQ_SSU_OTU.tsv	Root-Host_associated-Human-Skin
SRR5869114	MGYA00416004	https://www.ebi.ac.uk/metagenomics/api/v1/analyses/MGYA00416004/file/SRR5869114_MERGED_FASTQ_SSU_OTU.tsv	Root-Host_associated-Human-Skin
ERR1960821	MGYA00130761	https://www.ebi.ac.uk/metagenomics/api/v1/analyses/MGYA00130761/file/ERR1960821_MERGED_FASTQ_otu.tsv	Root-Host_associated-Human-Skin

SRR1620023	MGYA00024921	https://www.ebi.ac.uk/metagenomics/api/v1/analyses/MGYA00024921/file/SRR1620023_MERGED_FASTQ_otu.tsv	Root-Host_associated-Human-Skin
ERR443633	MGYA00413326	https://www.ebi.ac.uk/metagenomics/api/v1/analyses/MGYA00413326/file/ERR443633_FASTQ_SSU_OTU.tsv	Root-Host_associated-Human-Skin
ERR450726	MGYA00427433	https://www.ebi.ac.uk/metagenomics/api/v1/analyses/MGYA00427433/file/ERR450726_FASTQ_SSU_OTU.tsv	Root-Host_associated-Human-Skin
SRR995479	MGYA00025097	https://www.ebi.ac.uk/metagenomics/api/v1/analyses/MGYA00025097/file/SRR995479_FASTQ_otu.tsv	Root-Host_associated-Human-Skin
SRR1923805	MGYA00028383	https://www.ebi.ac.uk/metagenomics/api/v1/analyses/MGYA00028383/file/SRR1923805_FASTQ_otu.tsv	Root-Host_associated-Human-Skin
SRR1950691	MGYA00376858	https://www.ebi.ac.uk/metagenomics/api/v1/analyses/MGYA00376858/file/SRR1950691_MERGED_FASTQ_SSU_OTU.tsv	Root-Host_associated-Human-Skin
SRR2005647	MGYA00381306	https://www.ebi.ac.uk/metagenomics/api/v1/analyses/MGYA00381306/file/SRR2005647_MERGED_FASTQ_SSU_OTU.tsv	Root-Host_associated-Human-Skin
SRR1704324	MGYA00416345	https://www.ebi.ac.uk/metagenomics/api/v1/analyses/MGYA00416345/file/SRR1704324_FASTQ_SSU_OTU.tsv	Root-Host_associated-Human-Skin
ERR1760008	MGYA00140510	https://www.ebi.ac.uk/metagenomics/api/v1/analyses/MGYA00140510/file/ERR1760008_MERGED_FASTQ_SSU_OTU.tsv	Root-Host_associated-Human-Skin
ERR451507	MGYA00425761	https://www.ebi.ac.uk/metagenomics/api/v1/analyses/MGYA00425761/file/ERR451507_FASTQ_SSU_OTU.tsv	Root-Host_associated-Human-Skin
SRR994658	MGYA00023453	https://www.ebi.ac.uk/metagenomics/api/v1/analyses/MGYA00023453/file/SRR994658_FASTQ_otu.tsv	Root-Host_associated-Human-Skin
SRR5870068	MGYA00420666	https://www.ebi.ac.uk/metagenomics/api/v1/analyses/MGYA00420666/file/SRR5870068_MERGED_FASTQ_SSU_OTU.tsv	Root-Host_associated-Human-Skin

ERR443283	MGYA00412628	https://www.ebi.ac.uk/metagenomics/api/v1/analyses/MGYA00412628/file/ERR443283_FASTQ_SSU_OTU.tsv	Root-Host_associated-Human-Skin
ERR1960744	MGYA00130867	https://www.ebi.ac.uk/metagenomics/api/v1/analyses/MGYA00130867/file/ERR1960744_MERGED_FASTQ_otu.tsv	Root-Host_associated-Human-Skin
SRR1704745	MGYA00412420	https://www.ebi.ac.uk/metagenomics/api/v1/analyses/MGYA00412420/file/SRR1704745_FASTQ_SSU_OTU.tsv	Root-Host_associated-Human-Skin
SRR5868831	MGYA00420285	https://www.ebi.ac.uk/metagenomics/api/v1/analyses/MGYA00420285/file/SRR5868831_MERGED_FASTQ_SSU_OTU.tsv	Root-Host_associated-Human-Skin
SRR6757630	MGYA00380747	https://www.ebi.ac.uk/metagenomics/api/v1/analyses/MGYA00380747/file/SRR6757630_MERGED_FASTQ_SSU_OTU.tsv	Root-Host_associated-Human-Skin
SRR1622813	MGYA00023979	https://www.ebi.ac.uk/metagenomics/api/v1/analyses/MGYA00023979/file/SRR1622813_MERGED_FASTQ_otu.tsv	Root-Host_associated-Human-Skin
ERR2538367	MGYA00381374	https://www.ebi.ac.uk/metagenomics/api/v1/analyses/MGYA00381374/file/ERR2538367_FASTQ_SSU_OTU.tsv	Root-Host_associated-Human-Skin
SRR1632476	MGYA00022490	https://www.ebi.ac.uk/metagenomics/api/v1/analyses/MGYA00022490/file/SRR1632476_MERGED_FASTQ_otu.tsv	Root-Host_associated-Human-Skin
SRR1923921	MGYA00028271	https://www.ebi.ac.uk/metagenomics/api/v1/analyses/MGYA00028271/file/SRR1923921_FASTQ_otu.tsv	Root-Host_associated-Human-Skin
SRR1920446	MGYA00028276	https://www.ebi.ac.uk/metagenomics/api/v1/analyses/MGYA00028276/file/SRR1920446_FASTQ_otu.tsv	Root-Host_associated-Human-Skin
ERR451384	MGYA00424307	https://www.ebi.ac.uk/metagenomics/api/v1/analyses/MGYA00424307/file/ERR451384_FASTQ_SSU_OTU.tsv	Root-Host_associated-Human-Skin
ERR1960660	MGYA00130847	https://www.ebi.ac.uk/metagenomics/api/v1/analyses/MGYA00130847/file/ERR1960660_MERGED_FASTQ_otu.tsv	Root-Host_associated-Human-Skin

ERR443400	MGYA00423295	https://www.ebi.ac.uk/metagenomics/api/v1/analyses/MGYA00423295/file/ERR443400_FASTQ_SSU_OTU.tsv	Root-Host_associated-Human-Skin
SRR920151	MGYA00024514	https://www.ebi.ac.uk/metagenomics/api/v1/analyses/MGYA00024514/file/SRR920151_FASTQ_otu.tsv	Root-Host_associated-Human-Skin
SRR5869480	MGYA00419663	https://www.ebi.ac.uk/metagenomics/api/v1/analyses/MGYA00419663/file/SRR5869480_MERGED_FASTQ_SSU_OTU.tsv	Root-Host_associated-Human-Skin
SRR1622937	MGYA00025293	https://www.ebi.ac.uk/metagenomics/api/v1/analyses/MGYA00025293/file/SRR1622937_MERGED_FASTQ_otu.tsv	Root-Host_associated-Human-Skin
SRR996029	MGYA00023720	https://www.ebi.ac.uk/metagenomics/api/v1/analyses/MGYA00023720/file/SRR996029_FASTQ_otu.tsv	Root-Host_associated-Human-Skin
ERR441988	MGYA00385154	https://www.ebi.ac.uk/metagenomics/api/v1/analyses/MGYA00385154/file/ERR441988_FASTQ_SSU_OTU.tsv	Root-Host_associated-Human-Skin
SRR5869823	MGYA00415870	https://www.ebi.ac.uk/metagenomics/api/v1/analyses/MGYA00415870/file/SRR5869823_MERGED_FASTQ_SSU_OTU.tsv	Root-Host_associated-Human-Skin
SRR5869451	MGYA00420717	https://www.ebi.ac.uk/metagenomics/api/v1/analyses/MGYA00420717/file/SRR5869451_MERGED_FASTQ_SSU_OTU.tsv	Root-Host_associated-Human-Skin
ERR441959	MGYA00385251	https://www.ebi.ac.uk/metagenomics/api/v1/analyses/MGYA00385251/file/ERR441959_FASTQ_SSU_OTU.tsv	Root-Host_associated-Human-Skin
SRR1631674	MGYA00023831	https://www.ebi.ac.uk/metagenomics/api/v1/analyses/MGYA00023831/file/SRR1631674_MERGED_FASTQ_otu.tsv	Root-Host_associated-Human-Skin
SRR920627	MGYA00022765	https://www.ebi.ac.uk/metagenomics/api/v1/analyses/MGYA00022765/file/SRR920627_FASTQ_otu.tsv	Root-Host_associated-Human-Skin
ERR1759932	MGYA00140441	https://www.ebi.ac.uk/metagenomics/api/v1/analyses/MGYA00140441/file/ERR1759932_MERGED_FASTQ_SSU_OTU.tsv	Root-Host_associated-Human-Skin

SRR1704576	MGYA00412711	https://www.ebi.ac.uk/metagenomics/api/v1/analyses/MGYA00412711/file/SRR1704576_FASTQ_SSU_OTU.tsv	Root-Host_associated-Human-Skin
SRR1950764	MGYA00376931	https://www.ebi.ac.uk/metagenomics/api/v1/analyses/MGYA00376931/file/SRR1950764_MERGED_FASTQ_SSU_OTU.tsv	Root-Host_associated-Human-Skin
SRR996499	MGYA00023397	https://www.ebi.ac.uk/metagenomics/api/v1/analyses/MGYA00023397/file/SRR996499_FASTQ_otu.tsv	Root-Host_associated-Human-Skin
SRR920246	MGYA00023494	https://www.ebi.ac.uk/metagenomics/api/v1/analyses/MGYA00023494/file/SRR920246_FASTQ_otu.tsv	Root-Host_associated-Human-Skin
ERR443717	MGYA00413644	https://www.ebi.ac.uk/metagenomics/api/v1/analyses/MGYA00413644/file/ERR443717_FASTQ_SSU_OTU.tsv	Root-Host_associated-Human-Skin
SRR920993	MGYA00023251	https://www.ebi.ac.uk/metagenomics/api/v1/analyses/MGYA00023251/file/SRR920993_FASTQ_otu.tsv	Root-Host_associated-Human-Skin
SRR1632300	MGYA00023272	https://www.ebi.ac.uk/metagenomics/api/v1/analyses/MGYA00023272/file/SRR1632300_MERGED_FASTQ_otu.tsv	Root-Host_associated-Human-Skin
SRR995712	MGYA00023354	https://www.ebi.ac.uk/metagenomics/api/v1/analyses/MGYA00023354/file/SRR995712_FASTQ_otu.tsv	Root-Host_associated-Human-Skin
SRR996607	MGYA00024578	https://www.ebi.ac.uk/metagenomics/api/v1/analyses/MGYA00024578/file/SRR996607_FASTQ_otu.tsv	Root-Host_associated-Human-Skin
SRR5869509	MGYA00417538	https://www.ebi.ac.uk/metagenomics/api/v1/analyses/MGYA00417538/file/SRR5869509_MERGED_FASTQ_SSU_OTU.tsv	Root-Host_associated-Human-Skin
SRR1704389	MGYA00414648	https://www.ebi.ac.uk/metagenomics/api/v1/analyses/MGYA00414648/file/SRR1704389_FASTQ_SSU_OTU.tsv	Root-Host_associated-Human-Skin
SRR996266	MGYA00023507	https://www.ebi.ac.uk/metagenomics/api/v1/analyses/MGYA00023507/file/SRR996266_FASTQ_otu.tsv	Root-Host_associated-Human-Skin

SRR996510	MGYA00023072	https://www.ebi.ac.uk/metagenomics/api/v1/analyses/MGYA00023072/file/SRR996510_FASTQ_otu.tsv	Root-Host_associated-Human-Skin
SRR994990	MGYA00023933	https://www.ebi.ac.uk/metagenomics/api/v1/analyses/MGYA00023933/file/SRR994990_FASTQ_otu.tsv	Root-Host_associated-Human-Skin
SRR1923879	MGYA00028361	https://www.ebi.ac.uk/metagenomics/api/v1/analyses/MGYA00028361/file/SRR1923879_FASTQ_otu.tsv	Root-Host_associated-Human-Skin
SRR997096	MGYA00022509	https://www.ebi.ac.uk/metagenomics/api/v1/analyses/MGYA00022509/file/SRR997096_FASTQ_otu.tsv	Root-Host_associated-Human-Skin
SRR995064	MGYA00024060	https://www.ebi.ac.uk/metagenomics/api/v1/analyses/MGYA00024060/file/SRR995064_FASTQ_otu.tsv	Root-Host_associated-Human-Skin
ERR1760074	MGYA00140409	https://www.ebi.ac.uk/metagenomics/api/v1/analyses/MGYA00140409/file/ERR1760074_MERGED_FASTQ_SSU_OTU.tsv	Root-Host_associated-Human-Skin
ERR1960738	MGYA00130773	https://www.ebi.ac.uk/metagenomics/api/v1/analyses/MGYA00130773/file/ERR1960738_MERGED_FASTQ_otu.tsv	Root-Host_associated-Human-Skin
SRR995928	MGYA00024063	https://www.ebi.ac.uk/metagenomics/api/v1/analyses/MGYA00024063/file/SRR995928_FASTQ_otu.tsv	Root-Host_associated-Human-Skin
ERR451045	MGYA00420483	https://www.ebi.ac.uk/metagenomics/api/v1/analyses/MGYA00420483/file/ERR451045_FASTQ_SSU_OTU.tsv	Root-Host_associated-Human-Skin
SRR1923926	MGYA00028278	https://www.ebi.ac.uk/metagenomics/api/v1/analyses/MGYA00028278/file/SRR1923926_FASTQ_otu.tsv	Root-Host_associated-Human-Skin
SRR1632471	MGYA00022495	https://www.ebi.ac.uk/metagenomics/api/v1/analyses/MGYA00022495/file/SRR1632471_MERGED_FASTQ_otu.tsv	Root-Host_associated-Human-Skin
SRR5869741	MGYA00416972	https://www.ebi.ac.uk/metagenomics/api/v1/analyses/MGYA00416972/file/SRR5869741_MERGED_FASTQ_SSU_OTU.tsv	Root-Host_associated-Human-Skin

ERR1960667	MGYA00130800	https://www.ebi.ac.uk/metagenomics/api/v1/analyses/MGYA00130800/file/ERR1960667_MERGED_FASTQ_otu.tsv	Root-Host_associated-Human-Skin
ERR451424	MGYA00428712	https://www.ebi.ac.uk/metagenomics/api/v1/analyses/MGYA00428712/file/ERR451424_FASTQ_SSU_OTU.tsv	Root-Host_associated-Human-Skin
SRR1622930	MGYA00025286	https://www.ebi.ac.uk/metagenomics/api/v1/analyses/MGYA00025286/file/SRR1622930_MERGED_FASTQ_otu.tsv	Root-Host_associated-Human-Skin
SRR996789	MGYA00024082	https://www.ebi.ac.uk/metagenomics/api/v1/analyses/MGYA00024082/file/SRR996789_FASTQ_otu.tsv	Root-Host_associated-Human-Skin
SRR1704666	MGYA00412708	https://www.ebi.ac.uk/metagenomics/api/v1/analyses/MGYA00412708/file/SRR1704666_FASTQ_SSU_OTU.tsv	Root-Host_associated-Human-Skin
SRR1631673	MGYA00023832	https://www.ebi.ac.uk/metagenomics/api/v1/analyses/MGYA00023832/file/SRR1631673_MERGED_FASTQ_otu.tsv	Root-Host_associated-Human-Skin
SRR997118	MGYA00024567	https://www.ebi.ac.uk/metagenomics/api/v1/analyses/MGYA00024567/file/SRR997118_FASTQ_otu.tsv	Root-Host_associated-Human-Skin
ERR451352	MGYA00428721	https://www.ebi.ac.uk/metagenomics/api/v1/analyses/MGYA00428721/file/ERR451352_FASTQ_SSU_OTU.tsv	Root-Host_associated-Human-Skin
ERR450960	MGYA00416324	https://www.ebi.ac.uk/metagenomics/api/v1/analyses/MGYA00416324/file/ERR450960_FASTQ_SSU_OTU.tsv	Root-Host_associated-Human-Skin
SRR920620	MGYA00022764	https://www.ebi.ac.uk/metagenomics/api/v1/analyses/MGYA00022764/file/SRR920620_FASTQ_otu.tsv	Root-Host_associated-Human-Skin
ERR443371	MGYA00428737	https://www.ebi.ac.uk/metagenomics/api/v1/analyses/MGYA00428737/file/ERR443371_FASTQ_SSU_OTU.tsv	Root-Host_associated-Human-Skin
SRR5869037	MGYA00428529	https://www.ebi.ac.uk/metagenomics/api/v1/analyses/MGYA00428529/file/SRR5869037_MERGED_FASTQ_SSU_OTU.tsv	Root-Host_associated-Human-Skin

SRR1950763	MGYA00376930	https://www.ebi.ac.uk/metagenomics/api/v1/analyses/MGYA00376930/file/SRR1950763_MERGED_FASTQ_SSU_OTU.tsv	Root-Host_associated-Human-Skin
SRR919948	MGYA00024615	https://www.ebi.ac.uk/metagenomics/api/v1/analyses/MGYA00024615/file/SRR919948_FASTQ_otu.tsv	Root-Host_associated-Human-Skin
SRR1704571	MGYA00419541	https://www.ebi.ac.uk/metagenomics/api/v1/analyses/MGYA00419541/file/SRR1704571_FASTQ_SSU_OTU.tsv	Root-Host_associated-Human-Skin
ERR1759935	MGYA00140525	https://www.ebi.ac.uk/metagenomics/api/v1/analyses/MGYA00140525/file/ERR1759935_MERGED_FASTQ_SSU_OTU.tsv	Root-Host_associated-Human-Skin
SRR920994	MGYA00023254	https://www.ebi.ac.uk/metagenomics/api/v1/analyses/MGYA00023254/file/SRR920994_FASTQ_otu.tsv	Root-Host_associated-Human-Skin
SRR1632307	MGYA00023275	https://www.ebi.ac.uk/metagenomics/api/v1/analyses/MGYA00023275/file/SRR1632307_MERGED_FASTQ_otu.tsv	Root-Host_associated-Human-Skin
SRR920241	MGYA00023495	https://www.ebi.ac.uk/metagenomics/api/v1/analyses/MGYA00023495/file/SRR920241_FASTQ_otu.tsv	Root-Host_associated-Human-Skin
ERR442058	MGYA00385253	https://www.ebi.ac.uk/metagenomics/api/v1/analyses/MGYA00385253/file/ERR442058_FASTQ_SSU_OTU.tsv	Root-Host_associated-Human-Skin
SRR995715	MGYA00023359	https://www.ebi.ac.uk/metagenomics/api/v1/analyses/MGYA00023359/file/SRR995715_FASTQ_otu.tsv	Root-Host_associated-Human-Skin
SRR921138	MGYA00022310	https://www.ebi.ac.uk/metagenomics/api/v1/analyses/MGYA00022310/file/SRR921138_FASTQ_otu.tsv	Root-Host_associated-Human-Skin
ERR443229	MGYA00412997	https://www.ebi.ac.uk/metagenomics/api/v1/analyses/MGYA00412997/file/ERR443229_FASTQ_SSU_OTU.tsv	Root-Host_associated-Human-Skin
SRR996600	MGYA00024571	https://www.ebi.ac.uk/metagenomics/api/v1/analyses/MGYA00024571/file/SRR996600_FASTQ_otu.tsv	Root-Host_associated-Human-Skin

SRR994946	MGYA00022489	https://www.ebi.ac.uk/metagenomics/api/v1/analyses/MGYA00022489/file/SRR994946_FASTQ_otu.tsv	Root-Host_associated-Human-Skin
SRR1630978	MGYA00024356	https://www.ebi.ac.uk/metagenomics/api/v1/analyses/MGYA00024356/file/SRR1630978_MERGED_FASTQ_otu.tsv	Root-Host_associated-Human-Skin
SRR996261	MGYA00023506	https://www.ebi.ac.uk/metagenomics/api/v1/analyses/MGYA00023506/file/SRR996261_FASTQ_otu.tsv	Root-Host_associated-Human-Skin
ERR443699	MGYA00415107	https://www.ebi.ac.uk/metagenomics/api/v1/analyses/MGYA00415107/file/ERR443699_FASTQ_SSU_OTU.tsv	Root-Host_associated-Human-Skin
SRR994997	MGYA00023928	https://www.ebi.ac.uk/metagenomics/api/v1/analyses/MGYA00023928/file/SRR994997_FASTQ_otu.tsv	Root-Host_associated-Human-Skin
SRR997091	MGYA00022516	https://www.ebi.ac.uk/metagenomics/api/v1/analyses/MGYA00022516/file/SRR997091_FASTQ_otu.tsv	Root-Host_associated-Human-Skin
SRR995063	MGYA00024057	https://www.ebi.ac.uk/metagenomics/api/v1/analyses/MGYA00024057/file/SRR995063_FASTQ_otu.tsv	Root-Host_associated-Human-Skin
ERR1760073	MGYA00140439	https://www.ebi.ac.uk/metagenomics/api/v1/analyses/MGYA00140439/file/ERR1760073_MERGED_FASTQ_SSU_OTU.tsv	Root-Host_associated-Human-Skin
SRR1631518	MGYA00023377	https://www.ebi.ac.uk/metagenomics/api/v1/analyses/MGYA00023377/file/SRR1631518_MERGED_FASTQ_otu.tsv	Root-Host_associated-Human-Skin
ERR1759998	MGYA00140356	https://www.ebi.ac.uk/metagenomics/api/v1/analyses/MGYA00140356/file/ERR1759998_MERGED_FASTQ_SSU_OTU.tsv	Root-Host_associated-Human-Skin
SRR996394	MGYA00024974	https://www.ebi.ac.uk/metagenomics/api/v1/analyses/MGYA00024974/file/SRR996394_FASTQ_otu.tsv	Root-Host_associated-Human-Skin
SRR919597	MGYA00024954	https://www.ebi.ac.uk/metagenomics/api/v1/analyses/MGYA00024954/file/SRR919597_FASTQ_otu.tsv	Root-Host_associated-Human-Skin

SRR996433	MGYA00024476	https://www.ebi.ac.uk/metagenomics/api/v1/analyses/MGYA00024476/file/SRR996433_FASTQ_otu.tsv	Root-Host_associated-Human-Skin
SRR2005718	MGYA00381347	https://www.ebi.ac.uk/metagenomics/api/v1/analyses/MGYA00381347/file/SRR2005718_MERGED_FASTQ_SSU_OTU.tsv	Root-Host_associated-Human-Skin
SRR995526	MGYA00023420	https://www.ebi.ac.uk/metagenomics/api/v1/analyses/MGYA00023420/file/SRR995526_FASTQ_otu.tsv	Root-Host_associated-Human-Skin
SRR920939	MGYA00022570	https://www.ebi.ac.uk/metagenomics/api/v1/analyses/MGYA00022570/file/SRR920939_FASTQ_otu.tsv	Root-Host_associated-Human-Skin
SRR2005379	MGYA00381288	https://www.ebi.ac.uk/metagenomics/api/v1/analyses/MGYA00381288/file/SRR2005379_MERGED_FASTQ_SSU_OTU.tsv	Root-Host_associated-Human-Skin
SRR995147	MGYA00024135	https://www.ebi.ac.uk/metagenomics/api/v1/analyses/MGYA00024135/file/SRR995147_FASTQ_otu.tsv	Root-Host_associated-Human-Skin
ERR451458	MGYA00415130	https://www.ebi.ac.uk/metagenomics/api/v1/analyses/MGYA00415130/file/ERR451458_FASTQ_SSU_OTU.tsv	Root-Host_associated-Human-Skin
SRR995631	MGYA00022346	https://www.ebi.ac.uk/metagenomics/api/v1/analyses/MGYA00022346/file/SRR995631_FASTQ_otu.tsv	Root-Host_associated-Human-Skin
SRR996724	MGYA00023429	https://www.ebi.ac.uk/metagenomics/api/v1/analyses/MGYA00023429/file/SRR996724_FASTQ_otu.tsv	Root-Host_associated-Human-Skin
SRR996083	MGYA00024365	https://www.ebi.ac.uk/metagenomics/api/v1/analyses/MGYA00024365/file/SRR996083_FASTQ_otu.tsv	Root-Host_associated-Human-Skin
SRR994862	MGYA00022388	https://www.ebi.ac.uk/metagenomics/api/v1/analyses/MGYA00022388/file/SRR994862_FASTQ_otu.tsv	Root-Host_associated-Human-Skin
SRR995985	MGYA00022940	https://www.ebi.ac.uk/metagenomics/api/v1/analyses/MGYA00022940/file/SRR995985_FASTQ_otu.tsv	Root-Host_associated-Human-Skin

ERR442024	MGYA00385208	https://www.ebi.ac.uk/metagenomics/api/v1/analyses/MGYA00385208/file/ERR442024_FASTQ_SSU_OTU.tsv	Root-Host_associated-Human-Skin
SRR996345	MGYA00022280	https://www.ebi.ac.uk/metagenomics/api/v1/analyses/MGYA00022280/file/SRR996345_FASTQ_otu.tsv	Root-Host_associated-Human-Skin
SRR996890	MGYA00022799	https://www.ebi.ac.uk/metagenomics/api/v1/analyses/MGYA00022799/file/SRR996890_FASTQ_otu.tsv	Root-Host_associated-Human-Skin
SRR1623069	MGYA00024419	https://www.ebi.ac.uk/metagenomics/api/v1/analyses/MGYA00024419/file/SRR1623069_MERGED_FASTQ_otu.tsv	Root-Host_associated-Human-Skin
ERR1759949	MGYA00140408	https://www.ebi.ac.uk/metagenomics/api/v1/analyses/MGYA00140408/file/ERR1759949_MERGED_FASTQ_SSU_OTU.tsv	Root-Host_associated-Human-Skin
SRR5869900	MGYA00423469	https://www.ebi.ac.uk/metagenomics/api/v1/analyses/MGYA00423469/file/SRR5869900_MERGED_FASTQ_SSU_OTU.tsv	Root-Host_associated-Human-Skin
SRR5869572	MGYA00418049	https://www.ebi.ac.uk/metagenomics/api/v1/analyses/MGYA00418049/file/SRR5869572_MERGED_FASTQ_SSU_OTU.tsv	Root-Host_associated-Human-Skin
ERR2538416	MGYA00381423	https://www.ebi.ac.uk/metagenomics/api/v1/analyses/MGYA00381423/file/ERR2538416_FASTQ_SSU_OTU.tsv	Root-Host_associated-Human-Skin
SRR921144	MGYA00024590	https://www.ebi.ac.uk/metagenomics/api/v1/analyses/MGYA00024590/file/SRR921144_FASTQ_otu.tsv	Root-Host_associated-Human-Skin
SRR1923767	MGYA00028337	https://www.ebi.ac.uk/metagenomics/api/v1/analyses/MGYA00028337/file/SRR1923767_FASTQ_otu.tsv	Root-Host_associated-Human-Skin
ERR1960792	MGYA00130843	https://www.ebi.ac.uk/metagenomics/api/v1/analyses/MGYA00130843/file/ERR1960792_MERGED_FASTQ_otu.tsv	Root-Host_associated-Human-Skin
ERR450844	MGYA00422047	https://www.ebi.ac.uk/metagenomics/api/v1/analyses/MGYA00422047/file/ERR450844_FASTQ_SSU_OTU.tsv	Root-Host_associated-Human-Skin

ERR443255	MGYA00416296	https://www.ebi.ac.uk/metagenomics/api/v1/analyses/MGYA00416296/file/ERR443255_FASTQ_SSU_OTU.tsv	Root-Host_associated-Human-Skin
ERR1701231	MGYA00084324	https://www.ebi.ac.uk/metagenomics/api/v1/analyses/MGYA00084324/file/ERR1701231_FASTQ_otu.tsv	Root-Host_associated-Human-Skin
SRR5869113	MGYA00418104	https://www.ebi.ac.uk/metagenomics/api/v1/analyses/MGYA00418104/file/SRR5869113_MERGED_FASTQ_SSU_OTU.tsv	Root-Host_associated-Human-Skin
SRR994548	MGYA00024257	https://www.ebi.ac.uk/metagenomics/api/v1/analyses/MGYA00024257/file/SRR994548_FASTQ_otu.tsv	Root-Host_associated-Human-Skin
SRR1922546	MGYA00028409	https://www.ebi.ac.uk/metagenomics/api/v1/analyses/MGYA00028409/file/SRR1922546_FASTQ_otu.tsv	Root-Host_associated-Human-Skin
ERR1960826	MGYA00130909	https://www.ebi.ac.uk/metagenomics/api/v1/analyses/MGYA00130909/file/ERR1960826_MERGED_FASTQ_otu.tsv	Root-Host_associated-Human-Skin
SRR995308	MGYA00022693	https://www.ebi.ac.uk/metagenomics/api/v1/analyses/MGYA00022693/file/SRR995308_FASTQ_otu.tsv	Root-Host_associated-Human-Skin
ERR450721	MGYA00423895	https://www.ebi.ac.uk/metagenomics/api/v1/analyses/MGYA00423895/file/ERR450721_FASTQ_SSU_OTU.tsv	Root-Host_associated-Human-Skin
SRR1704323	MGYA00415217	https://www.ebi.ac.uk/metagenomics/api/v1/analyses/MGYA00415217/file/SRR1704323_FASTQ_SSU_OTU.tsv	Root-Host_associated-Human-Skin
SRR5868782	MGYA00420782	https://www.ebi.ac.uk/metagenomics/api/v1/analyses/MGYA00420782/file/SRR5868782_MERGED_FASTQ_SSU_OTU.tsv	Root-Host_associated-Human-Skin
SRR5869665	MGYA00415897	https://www.ebi.ac.uk/metagenomics/api/v1/analyses/MGYA00415897/file/SRR5869665_MERGED_FASTQ_SSU_OTU.tsv	Root-Host_associated-Human-Skin
SRR996919	MGYA00022436	https://www.ebi.ac.uk/metagenomics/api/v1/analyses/MGYA00022436/file/SRR996919_FASTQ_otu.tsv	Root-Host_associated-Human-Skin

SRR1950696	MGYA00376863	https://www.ebi.ac.uk/metagenomics/api/v1/analyses/MGYA00376863/file/SRR1950696_MERGED_FASTQ_SSU_OTU.tsv	Root-Host_associated-Human-Skin
SRR2005640	MGYA00381300	https://www.ebi.ac.uk/metagenomics/api/v1/analyses/MGYA00381300/file/SRR2005640_MERGED_FASTQ_SSU_OTU.tsv	Root-Host_associated-Human-Skin
ERR1960743	MGYA00130708	https://www.ebi.ac.uk/metagenomics/api/v1/analyses/MGYA00130708/file/ERR1960743_MERGED_FASTQ_otu.tsv	Root-Host_associated-Human-Skin
ERR451500	MGYA00428684	https://www.ebi.ac.uk/metagenomics/api/v1/analyses/MGYA00428684/file/ERR451500_FASTQ_SSU_OTU.tsv	Root-Host_associated-Human-Skin
SRR5869204	MGYA00415960	https://www.ebi.ac.uk/metagenomics/api/v1/analyses/MGYA00415960/file/SRR5869204_MERGED_FASTQ_SSU_OTU.tsv	Root-Host_associated-Human-Skin
ERR2538360	MGYA00381367	https://www.ebi.ac.uk/metagenomics/api/v1/analyses/MGYA00381367/file/ERR2538360_FASTQ_SSU_OTU.tsv	Root-Host_associated-Human-Skin
SRR1622814	MGYA00023986	https://www.ebi.ac.uk/metagenomics/api/v1/analyses/MGYA00023986/file/SRR1622814_MERGED_FASTQ_otu.tsv	Root-Host_associated-Human-Skin
SRR1704742	MGYA00425790	https://www.ebi.ac.uk/metagenomics/api/v1/analyses/MGYA00425790/file/SRR1704742_FASTQ_SSU_OTU.tsv	Root-Host_associated-Human-Skin
SRR6757637	MGYA00380752	https://www.ebi.ac.uk/metagenomics/api/v1/analyses/MGYA00380752/file/SRR6757637_MERGED_FASTQ_SSU_OTU.tsv	Root-Host_associated-Human-Skin
SRR5868836	MGYA00416047	https://www.ebi.ac.uk/metagenomics/api/v1/analyses/MGYA00416047/file/SRR5868836_MERGED_FASTQ_SSU_OTU.tsv	Root-Host_associated-Human-Skin
SRR996244	MGYA00023708	https://www.ebi.ac.uk/metagenomics/api/v1/analyses/MGYA00023708/file/SRR996244_FASTQ_otu.tsv	Root-Host_associated-Human-Skin
SRR1623168	MGYA00023475	https://www.ebi.ac.uk/metagenomics/api/v1/analyses/MGYA00023475/file/SRR1623168_MERGED_FASTQ_otu.tsv	Root-Host_associated-Human-Skin

SRR995730	MGYA00023101	https://www.ebi.ac.uk/metagenomics/api/v1/analyses/MGYA00023101/file/SRR995730_FASTQ_otu.tsv	Root-Host_associated-Human-Skin
ERR1760087	MGYA00140587	https://www.ebi.ac.uk/metagenomics/api/v1/analyses/MGYA00140587/file/ERR1760087_MERGED_FASTQ_SSU_OTU.tsv	Root-Host_associated-Human-Skin
ERR1701268	MGYA00084299	https://www.ebi.ac.uk/metagenomics/api/v1/analyses/MGYA00084299/file/ERR1701268_FASTQ_otu.tsv	Root-Host_associated-Human-Skin
SRR5869959	MGYA00415845	https://www.ebi.ac.uk/metagenomics/api/v1/analyses/MGYA00415845/file/SRR5869959_MERGED_FASTQ_SSU_OTU.tsv	Root-Host_associated-Human-Skin
SRR996182	MGYA00022959	https://www.ebi.ac.uk/metagenomics/api/v1/analyses/MGYA00022959/file/SRR996182_FASTQ_otu.tsv	Root-Host_associated-Human-Skin
ERR1760056	MGYA00140430	https://www.ebi.ac.uk/metagenomics/api/v1/analyses/MGYA00140430/file/ERR1760056_MERGED_FASTQ_SSU_OTU.tsv	Root-Host_associated-Human-Skin
SRR995046	MGYA00023693	https://www.ebi.ac.uk/metagenomics/api/v1/analyses/MGYA00023693/file/SRR995046_FASTQ_otu.tsv	Root-Host_associated-Human-Skin
ERR1759899	MGYA00140450	https://www.ebi.ac.uk/metagenomics/api/v1/analyses/MGYA00140450/file/ERR1759899_MERGED_FASTQ_SSU_OTU.tsv	Root-Host_associated-Human-Skin
SRR996532	MGYA00022841	https://www.ebi.ac.uk/metagenomics/api/v1/analyses/MGYA00022841/file/SRR996532_FASTQ_otu.tsv	Root-Host_associated-Human-Skin
ERR450778	MGYA00416726	https://www.ebi.ac.uk/metagenomics/api/v1/analyses/MGYA00416726/file/ERR450778_FASTQ_SSU_OTU.tsv	Root-Host_associated-Human-Skin
ERR1960642	MGYA00130906	https://www.ebi.ac.uk/metagenomics/api/v1/analyses/MGYA00130906/file/ERR1960642_MERGED_FASTQ_otu.tsv	Root-Host_associated-Human-Skin
SRR1632792	MGYA00023236	https://www.ebi.ac.uk/metagenomics/api/v1/analyses/MGYA00023236/file/SRR1632792_MERGED_FASTQ_otu.tsv	Root-Host_associated-Human-Skin

SRR920173	MGYA00024708	https://www.ebi.ac.uk/metagenomics/api/v1/analyses/MGYA00024708/file/SRR920173_FASTQ_otu.tsv	Root-Host_associated-Human-Skin
SRR921094	MGYA00022430	https://www.ebi.ac.uk/metagenomics/api/v1/analyses/MGYA00022430/file/SRR921094_FASTQ_otu.tsv	Root-Host_associated-Human-Skin
SRR1622915	MGYA00025083	https://www.ebi.ac.uk/metagenomics/api/v1/analyses/MGYA00025083/file/SRR1622915_MERGED_FASTQ_otu.tsv	Root-Host_associated-Human-Skin
SRR1631687	MGYA00024831	https://www.ebi.ac.uk/metagenomics/api/v1/analyses/MGYA00024831/file/SRR1631687_MERGED_FASTQ_otu.tsv	Root-Host_associated-Human-Skin
SRR1923903	MGYA00028325	https://www.ebi.ac.uk/metagenomics/api/v1/analyses/MGYA00028325/file/SRR1923903_FASTQ_otu.tsv	Root-Host_associated-Human-Skin
ERR451060	MGYA00416684	https://www.ebi.ac.uk/metagenomics/api/v1/analyses/MGYA00416684/file/ERR451060_FASTQ_SSU_OTU.tsv	Root-Host_associated-Human-Skin
ERR443043	MGYA00416321	https://www.ebi.ac.uk/metagenomics/api/v1/analyses/MGYA00416321/file/ERR443043_FASTQ_SSU_OTU.tsv	Root-Host_associated-Human-Skin
SRR1704585	MGYA00413393	https://www.ebi.ac.uk/metagenomics/api/v1/analyses/MGYA00413393/file/SRR1704585_FASTQ_SSU_OTU.tsv	Root-Host_associated-Human-Skin
SRR5868683	MGYA00423501	https://www.ebi.ac.uk/metagenomics/api/v1/analyses/MGYA00423501/file/SRR5868683_MERGED_FASTQ_SSU_OTU.tsv	Root-Host_associated-Human-Skin
SRR996818	MGYA00023625	https://www.ebi.ac.uk/metagenomics/api/v1/analyses/MGYA00023625/file/SRR996818_FASTQ_otu.tsv	Root-Host_associated-Human-Skin
SRR1950746	MGYA00376913	https://www.ebi.ac.uk/metagenomics/api/v1/analyses/MGYA00376913/file/SRR1950746_MERGED_FASTQ_SSU_OTU.tsv	Root-Host_associated-Human-Skin
ERR1759910	MGYA00140412	https://www.ebi.ac.uk/metagenomics/api/v1/analyses/MGYA00140412/file/ERR1759910_MERGED_FASTQ_SSU_OTU.tsv	Root-Host_associated-Human-Skin

SRR5868652	MGYA00428068	https://www.ebi.ac.uk/metagenomics/api/v1/analyses/MGYA00428068/file/SRR5868652_MERGED_FASTQ_SSU_OTU.tsv	Root-Host_associated-Human-Skin
SRR920264	MGYA00023691	https://www.ebi.ac.uk/metagenomics/api/v1/analyses/MGYA00023691/file/SRR920264_FASTQ_otu.tsv	Root-Host_associated-Human-Skin
ERR443735	MGYA00412587	https://www.ebi.ac.uk/metagenomics/api/v1/analyses/MGYA00412587/file/ERR443735_FASTQ_SSU_OTU.tsv	Root-Host_associated-Human-Skin
SRR1632485	MGYA00022674	https://www.ebi.ac.uk/metagenomics/api/v1/analyses/MGYA00022674/file/SRR1632485_MERGED_FASTQ_otu.tsv	Root-Host_associated-Human-Skin
SRR1632322	MGYA00023481	https://www.ebi.ac.uk/metagenomics/api/v1/analyses/MGYA00023481/file/SRR1632322_MERGED_FASTQ_otu.tsv	Root-Host_associated-Human-Skin
SRR5869801	MGYA00425841	https://www.ebi.ac.uk/metagenomics/api/v1/analyses/MGYA00425841/file/SRR5869801_MERGED_FASTQ_SSU_OTU.tsv	Root-Host_associated-Human-Skin
ERR2538517	MGYA00381524	https://www.ebi.ac.uk/metagenomics/api/v1/analyses/MGYA00381524/file/ERR2538517_FASTQ_SSU_OTU.tsv	Root-Host_associated-Human-Skin
SRR1624828	MGYA00024881	https://www.ebi.ac.uk/metagenomics/api/v1/analyses/MGYA00024881/file/SRR1624828_MERGED_FASTQ_otu.tsv	Root-Host_associated-Human-Skin
ERR1960693	MGYA00130737	https://www.ebi.ac.uk/metagenomics/api/v1/analyses/MGYA00130737/file/ERR1960693_MERGED_FASTQ_otu.tsv	Root-Host_associated-Human-Skin
SRR920605	MGYA00022560	https://www.ebi.ac.uk/metagenomics/api/v1/analyses/MGYA00022560/file/SRR920605_FASTQ_otu.tsv	Root-Host_associated-Human-Skin
SRR919849	MGYA00024099	https://www.ebi.ac.uk/metagenomics/api/v1/analyses/MGYA00024099/file/SRR919849_FASTQ_otu.tsv	Root-Host_associated-Human-Skin
SRR1922563	MGYA00028308	https://www.ebi.ac.uk/metagenomics/api/v1/analyses/MGYA00028308/file/SRR1922563_FASTQ_otu.tsv	Root-Host_associated-Human-Skin

ERR1960803	MGYA00130804	https://www.ebi.ac.uk/metagenomics/api/v1/analyses/MGYA00130804/file/ERR1960803_MERGED_FASTQ_otu.tsv	Root-Host_associated-Human-Skin
ERR443611	MGYA00414533	https://www.ebi.ac.uk/metagenomics/api/v1/analyses/MGYA00414533/file/ERR443611_FASTQ_SSU_OTU.tsv	Root-Host_associated-Human-Skin
SRR997019	MGYA00023409	https://www.ebi.ac.uk/metagenomics/api/v1/analyses/MGYA00023409/file/SRR997019_FASTQ_otu.tsv	Root-Host_associated-Human-Skin
ERR2538433	MGYA00381440	https://www.ebi.ac.uk/metagenomics/api/v1/analyses/MGYA00381440/file/ERR2538433_FASTQ_SSU_OTU.tsv	Root-Host_associated-Human-Skin
SRR5869925	MGYA00413456	https://www.ebi.ac.uk/metagenomics/api/v1/analyses/MGYA00413456/file/SRR5869925_MERGED_FASTQ_SSU_OTU.tsv	Root-Host_associated-Human-Skin
ERR2538394	MGYA00381401	https://www.ebi.ac.uk/metagenomics/api/v1/analyses/MGYA00381401/file/ERR2538394_FASTQ_SSU_OTU.tsv	Root-Host_associated-Human-Skin
ERR1701214	MGYA00084281	https://www.ebi.ac.uk/metagenomics/api/v1/analyses/MGYA00084281/file/ERR1701214_FASTQ_otu.tsv	Root-Host_associated-Human-Skin
ERR450861	MGYA00417790	https://www.ebi.ac.uk/metagenomics/api/v1/analyses/MGYA00417790/file/ERR450861_FASTQ_SSU_OTU.tsv	Root-Host_associated-Human-Skin
ERR1960766	MGYA00130894	https://www.ebi.ac.uk/metagenomics/api/v1/analyses/MGYA00130894/file/ERR1960766_MERGED_FASTQ_otu.tsv	Root-Host_associated-Human-Skin
ERR451525	MGYA00424885	https://www.ebi.ac.uk/metagenomics/api/v1/analyses/MGYA00424885/file/ERR451525_FASTQ_SSU_OTU.tsv	Root-Host_associated-Human-Skin
ERR451282	MGYA00428745	https://www.ebi.ac.uk/metagenomics/api/v1/analyses/MGYA00428745/file/ERR451282_FASTQ_SSU_OTU.tsv	Root-Host_associated-Human-Skin
SRR5869586	MGYA00414846	https://www.ebi.ac.uk/metagenomics/api/v1/analyses/MGYA00414846/file/SRR5869586_MERGED_FASTQ_SSU_OTU.tsv	Root-Host_associated-Human-Skin

SRR1631004	MGYA00024595	https://www.ebi.ac.uk/metagenomics/api/v1/analyses/MGYA00024595/file/SRR1631004_MERGED_FASTQ_otu.tsv	Root-Host_associated-Human-Skin
SRR6757612	MGYA00380730	https://www.ebi.ac.uk/metagenomics/api/v1/analyses/MGYA00380730/file/SRR6757612_MERGED_FASTQ_SSU_OTU.tsv	Root-Host_associated-Human-Skin
SRR5868813	MGYA00417088	https://www.ebi.ac.uk/metagenomics/api/v1/analyses/MGYA00417088/file/SRR5868813_MERGED_FASTQ_SSU_OTU.tsv	Root-Host_associated-Human-Skin
ERR443167	MGYA00420479	https://www.ebi.ac.uk/metagenomics/api/v1/analyses/MGYA00420479/file/ERR443167_FASTQ_SSU_OTU.tsv	Root-Host_associated-Human-Skin
ERR450704	MGYA00425287	https://www.ebi.ac.uk/metagenomics/api/v1/analyses/MGYA00425287/file/ERR450704_FASTQ_SSU_OTU.tsv	Root-Host_associated-Human-Skin
SRR2005665	MGYA00381323	https://www.ebi.ac.uk/metagenomics/api/v1/analyses/MGYA00381323/file/SRR2005665_MERGED_FASTQ_SSU_OTU.tsv	Root-Host_associated-Human-Skin
SRR1704198	MGYA00415673	https://www.ebi.ac.uk/metagenomics/api/v1/analyses/MGYA00415673/file/SRR1704198_FASTQ_SSU_OTU.tsv	Root-Host_associated-Human-Skin
SRR995162	MGYA00024331	https://www.ebi.ac.uk/metagenomics/api/v1/analyses/MGYA00024331/file/SRR995162_FASTQ_otu.tsv	Root-Host_associated-Human-Skin
SRR996416	MGYA00024304	https://www.ebi.ac.uk/metagenomics/api/v1/analyses/MGYA00024304/file/SRR996416_FASTQ_otu.tsv	Root-Host_associated-Human-Skin
SRR995503	MGYA00023175	https://www.ebi.ac.uk/metagenomics/api/v1/analyses/MGYA00023175/file/SRR995503_FASTQ_otu.tsv	Root-Host_associated-Human-Skin
ERR443798	MGYA00428380	https://www.ebi.ac.uk/metagenomics/api/v1/analyses/MGYA00428380/file/ERR443798_FASTQ_SSU_OTU.tsv	Root-Host_associated-Human-Skin
SRR1632428	MGYA00023760	https://www.ebi.ac.uk/metagenomics/api/v1/analyses/MGYA00023760/file/SRR1632428_MERGED_FASTQ_otu.tsv	Root-Host_associated-Human-Skin

SRR994847	MGYA00022616	https://www.ebi.ac.uk/metagenomics/api/v1/analyses/MGYA00022616/file/SRR994847_FASTQ_otu.tsv	Root-Host_associated-Human-Skin
SRR995275	MGYA00025059	https://www.ebi.ac.uk/metagenomics/api/v1/analyses/MGYA00025059/file/SRR995275_FASTQ_otu.tsv	Root-Host_associated-Human-Skin
SRR996360	MGYA00022505	https://www.ebi.ac.uk/metagenomics/api/v1/analyses/MGYA00022505/file/SRR996360_FASTQ_otu.tsv	Root-Host_associated-Human-Skin
SRR921039	MGYA00023076	https://www.ebi.ac.uk/metagenomics/api/v1/analyses/MGYA00023076/file/SRR921039_FASTQ_otu.tsv	Root-Host_associated-Human-Skin
ERR1551324	MGYA00069425	https://www.ebi.ac.uk/metagenomics/api/v1/analyses/MGYA00069425/file/ERR1551324_MERGED_FASTQ_otu.tsv	Root-Host_associated-Human-Skin
SRR996701	MGYA00022327	https://www.ebi.ac.uk/metagenomics/api/v1/analyses/MGYA00022327/file/SRR996701_FASTQ_otu.tsv	Root-Host_associated-Human-Skin
SRR996598	MGYA00023997	https://www.ebi.ac.uk/metagenomics/api/v1/analyses/MGYA00023997/file/SRR996598_FASTQ_otu.tsv	Root-Host_associated-Human-Skin
SRR994918	MGYA00024742	https://www.ebi.ac.uk/metagenomics/api/v1/analyses/MGYA00024742/file/SRR994918_FASTQ_otu.tsv	Root-Host_associated-Human-Skin
ERR1960804	MGYA00130749	https://www.ebi.ac.uk/metagenomics/api/v1/analyses/MGYA00130749/file/ERR1960804_MERGED_FASTQ_otu.tsv	Root-Host_associated-Human-Skin
SRR1922564	MGYA00028306	https://www.ebi.ac.uk/metagenomics/api/v1/analyses/MGYA00028306/file/SRR1922564_FASTQ_otu.tsv	Root-Host_associated-Human-Skin
ERR2538393	MGYA00381400	https://www.ebi.ac.uk/metagenomics/api/v1/analyses/MGYA00381400/file/ERR2538393_FASTQ_SSU_OTU.tsv	Root-Host_associated-Human-Skin
ERR2538434	MGYA00381441	https://www.ebi.ac.uk/metagenomics/api/v1/analyses/MGYA00381441/file/ERR2538434_FASTQ_SSU_OTU.tsv	Root-Host_associated-Human-Skin

ERR1701213	MGYA00084303	https://www.ebi.ac.uk/metagenomics/api/v1/analyses/MGYA00084303/file/ERR1701213_FASTQ_otu.tsv	Root-Host_associated-Human-Skin
ERR1960761	MGYA00130776	https://www.ebi.ac.uk/metagenomics/api/v1/analyses/MGYA00130776/file/ERR1960761_MERGED_FASTQ_otu.tsv	Root-Host_associated-Human-Skin
ERR443501	MGYA00415135	https://www.ebi.ac.uk/metagenomics/api/v1/analyses/MGYA00415135/file/ERR443501_FASTQ_SSU_OTU.tsv	Root-Host_associated-Human-Skin
SRR1631003	MGYA00024602	https://www.ebi.ac.uk/metagenomics/api/v1/analyses/MGYA00024602/file/SRR1631003_MERGED_FASTQ_otu.tsv	Root-Host_associated-Human-Skin
SRR6757615	MGYA00380733	https://www.ebi.ac.uk/metagenomics/api/v1/analyses/MGYA00380733/file/SRR6757615_MERGED_FASTQ_SSU_OTU.tsv	Root-Host_associated-Human-Skin
SRR1924038	MGYA00028236	https://www.ebi.ac.uk/metagenomics/api/v1/analyses/MGYA00028236/file/SRR1924038_FASTQ_otu.tsv	Root-Host_associated-Human-Skin
SRR1622836	MGYA00023718	https://www.ebi.ac.uk/metagenomics/api/v1/analyses/MGYA00023718/file/SRR1622836_MERGED_FASTQ_otu.tsv	Root-Host_associated-Human-Skin
ERR451143	MGYA00420140	https://www.ebi.ac.uk/metagenomics/api/v1/analyses/MGYA00420140/file/ERR451143_FASTQ_SSU_OTU.tsv	Root-Host_associated-Human-Skin
SRR5869647	MGYA00415901	https://www.ebi.ac.uk/metagenomics/api/v1/analyses/MGYA00415901/file/SRR5869647_MERGED_FASTQ_SSU_OTU.tsv	Root-Host_associated-Human-Skin
SRR2005662	MGYA00381320	https://www.ebi.ac.uk/metagenomics/api/v1/analyses/MGYA00381320/file/SRR2005662_MERGED_FASTQ_SSU_OTU.tsv	Root-Host_associated-Human-Skin
SRR995165	MGYA00024326	https://www.ebi.ac.uk/metagenomics/api/v1/analyses/MGYA00024326/file/SRR995165_FASTQ_otu.tsv	Root-Host_associated-Human-Skin
ERR1960639	MGYA00130826	https://www.ebi.ac.uk/metagenomics/api/v1/analyses/MGYA00130826/file/ERR1960639_MERGED_FASTQ_otu.tsv	Root-Host_associated-Human-Skin

ERR443459	MGYA00419491	https://www.ebi.ac.uk/metagenomics/api/v1/analyses/MGYA00419491/file/ERR443459_FASTQ_SSU_OTU.tsv	Root-Host_associated-Human-Skin
SRR996411	MGYA00024297	https://www.ebi.ac.uk/metagenomics/api/v1/analyses/MGYA00024297/file/SRR996411_FASTQ_otu.tsv	Root-Host_associated-Human-Skin
SRR1632388	MGYA00022368	https://www.ebi.ac.uk/metagenomics/api/v1/analyses/MGYA00022368/file/SRR1632388_MERGED_FASTQ_otu.tsv	Root-Host_associated-Human-Skin
SRR1923978	MGYA00028255	https://www.ebi.ac.uk/metagenomics/api/v1/analyses/MGYA00028255/file/SRR1923978_FASTQ_otu.tsv	Root-Host_associated-Human-Skin
SRR995504	MGYA00023172	https://www.ebi.ac.uk/metagenomics/api/v1/analyses/MGYA00023172/file/SRR995504_FASTQ_otu.tsv	Root-Host_associated-Human-Skin
SRR995272	MGYA00025064	https://www.ebi.ac.uk/metagenomics/api/v1/analyses/MGYA00025064/file/SRR995272_FASTQ_otu.tsv	Root-Host_associated-Human-Skin
ERR442006	MGYA00385165	https://www.ebi.ac.uk/metagenomics/api/v1/analyses/MGYA00385165/file/ERR442006_FASTQ_SSU_OTU.tsv	Root-Host_associated-Human-Skin
SRR994840	MGYA00022609	https://www.ebi.ac.uk/metagenomics/api/v1/analyses/MGYA00022609/file/SRR994840_FASTQ_otu.tsv	Root-Host_associated-Human-Skin
SRR996367	MGYA00022498	https://www.ebi.ac.uk/metagenomics/api/v1/analyses/MGYA00022498/file/SRR996367_FASTQ_otu.tsv	Root-Host_associated-Human-Skin
ERR1551323	MGYA00069434	https://www.ebi.ac.uk/metagenomics/api/v1/analyses/MGYA00069434/file/ERR1551323_MERGED_FASTQ_otu.tsv	Root-Host_associated-Human-Skin
SRR996706	MGYA00022434	https://www.ebi.ac.uk/metagenomics/api/v1/analyses/MGYA00022434/file/SRR996706_FASTQ_otu.tsv	Root-Host_associated-Human-Skin
SRR1631368	MGYA00022581	https://www.ebi.ac.uk/metagenomics/api/v1/analyses/MGYA00022581/file/SRR1631368_MERGED_FASTQ_otu.tsv	Root-Host_associated-Human-Skin

SRR996243	MGYA00023707	https://www.ebi.ac.uk/metagenomics/api/v1/analyses/MGYA00023707/file/SRR996243_FASTQ_otu.tsv	Root-Host_associated-Human-Skin
ERR1760080	MGYA00140603	https://www.ebi.ac.uk/metagenomics/api/v1/analyses/MGYA00140603/file/ERR1760080_MERGED_FASTQ_SSU_OTU.tsv	Root-Host_associated-Human-Skin
SRR995737	MGYA00023098	https://www.ebi.ac.uk/metagenomics/api/v1/analyses/MGYA00023098/file/SRR995737_FASTQ_otu.tsv	Root-Host_associated-Human-Skin
ERR981245	MGYA00010238	https://www.ebi.ac.uk/metagenomics/api/v1/analyses/MGYA00010238/file/ERR981245_MERGED_FASTQ_otu.tsv	Root-Host_associated-Human-Skin
ERR2538448	MGYA00381455	https://www.ebi.ac.uk/metagenomics/api/v1/analyses/MGYA00381455/file/ERR2538448_FASTQ_SSU_OTU.tsv	Root-Host_associated-Human-Skin
SRR996185	MGYA00022956	https://www.ebi.ac.uk/metagenomics/api/v1/analyses/MGYA00022956/file/SRR996185_FASTQ_otu.tsv	Root-Host_associated-Human-Skin
SRR5868868	MGYA00416505	https://www.ebi.ac.uk/metagenomics/api/v1/analyses/MGYA00416505/file/SRR5868868_MERGED_FASTQ_SSU_OTU.tsv	Root-Host_associated-Human-Skin
SRR6757669	MGYA00380782	https://www.ebi.ac.uk/metagenomics/api/v1/analyses/MGYA00380782/file/SRR6757669_MERGED_FASTQ_SSU_OTU.tsv	Root-Host_associated-Human-Skin
ERR2538499	MGYA00381506	https://www.ebi.ac.uk/metagenomics/api/v1/analyses/MGYA00381506/file/ERR2538499_FASTQ_SSU_OTU.tsv	Root-Host_associated-Human-Skin
ERR1760051	MGYA00140415	https://www.ebi.ac.uk/metagenomics/api/v1/analyses/MGYA00140415/file/ERR1760051_MERGED_FASTQ_SSU_OTU.tsv	Root-Host_associated-Human-Skin
SRR995041	MGYA00023698	https://www.ebi.ac.uk/metagenomics/api/v1/analyses/MGYA00023698/file/SRR995041_FASTQ_otu.tsv	Root-Host_associated-Human-Skin
SRR996535	MGYA00022840	https://www.ebi.ac.uk/metagenomics/api/v1/analyses/MGYA00022840/file/SRR996535_FASTQ_otu.tsv	Root-Host_associated-Human-Skin

ERR1760109	MGYA00140363	https://www.ebi.ac.uk/metagenomics/api/v1/analyses/MGYA00140363/file/ERR1760109_MERGED_FASTQ_SSU_OTU.tsv	Root-Host_associated-Human-Skin
SRR921093	MGYA00022423	https://www.ebi.ac.uk/metagenomics/api/v1/analyses/MGYA00022423/file/SRR921093_FASTQ_otu.tsv	Root-Host_associated-Human-Skin
SRR1632795	MGYA00023369	https://www.ebi.ac.uk/metagenomics/api/v1/analyses/MGYA00023369/file/SRR1632795_MERGED_FASTQ_otu.tsv	Root-Host_associated-Human-Skin
ERR443425	MGYA00412980	https://www.ebi.ac.uk/metagenomics/api/v1/analyses/MGYA00412980/file/ERR443425_FASTQ_SSU_OTU.tsv	Root-Host_associated-Human-Skin
SRR920174	MGYA00024715	https://www.ebi.ac.uk/metagenomics/api/v1/analyses/MGYA00024715/file/SRR920174_FASTQ_otu.tsv	Root-Host_associated-Human-Skin
SRR994759	MGYA00023240	https://www.ebi.ac.uk/metagenomics/api/v1/analyses/MGYA00023240/file/SRR994759_FASTQ_otu.tsv	Root-Host_associated-Human-Skin
ERR443382	MGYA00416283	https://www.ebi.ac.uk/metagenomics/api/v1/analyses/MGYA00416283/file/ERR443382_FASTQ_SSU_OTU.tsv	Root-Host_associated-Human-Skin
ERR1960645	MGYA00130711	https://www.ebi.ac.uk/metagenomics/api/v1/analyses/MGYA00130711/file/ERR1960645_MERGED_FASTQ_otu.tsv	Root-Host_associated-Human-Skin
SRR1631680	MGYA00024828	https://www.ebi.ac.uk/metagenomics/api/v1/analyses/MGYA00024828/file/SRR1631680_MERGED_FASTQ_otu.tsv	Root-Host_associated-Human-Skin
ERR451067	MGYA00421094	https://www.ebi.ac.uk/metagenomics/api/v1/analyses/MGYA00421094/file/ERR451067_FASTQ_SSU_OTU.tsv	Root-Host_associated-Human-Skin
SRR1923904	MGYA00028332	https://www.ebi.ac.uk/metagenomics/api/v1/analyses/MGYA00028332/file/SRR1923904_FASTQ_otu.tsv	Root-Host_associated-Human-Skin
SRR1950790	MGYA00376957	https://www.ebi.ac.uk/metagenomics/api/v1/analyses/MGYA00376957/file/SRR1950790_MERGED_FASTQ_SSU_OTU.tsv	Root-Host_associated-Human-Skin

SRR5868655	MGYA00427623	https://www.ebi.ac.uk/metagenomics/api/v1/analyses/MGYA00427623/file/SRR5868655_MERGED_FASTQ_SSU_OTU.tsv	Root-Host_associated-Human-Skin
ERR1759917	MGYA00140419	https://www.ebi.ac.uk/metagenomics/api/v1/analyses/MGYA00140419/file/ERR1759917_MERGED_FASTQ_SSU_OTU.tsv	Root-Host_associated-Human-Skin
SRR1950741	MGYA00376908	https://www.ebi.ac.uk/metagenomics/api/v1/analyses/MGYA00376908/file/SRR1950741_MERGED_FASTQ_SSU_OTU.tsv	Root-Host_associated-Human-Skin
SRR1632325	MGYA00024010	https://www.ebi.ac.uk/metagenomics/api/v1/analyses/MGYA00024010/file/SRR1632325_MERGED_FASTQ_otu.tsv	Root-Host_associated-Human-Skin
SRR920263	MGYA00023684	https://www.ebi.ac.uk/metagenomics/api/v1/analyses/MGYA00023684/file/SRR920263_FASTQ_otu.tsv	Root-Host_associated-Human-Skin
SRR1632482	MGYA00022681	https://www.ebi.ac.uk/metagenomics/api/v1/analyses/MGYA00022681/file/SRR1632482_MERGED_FASTQ_otu.tsv	Root-Host_associated-Human-Skin
ERR2538510	MGYA00381517	https://www.ebi.ac.uk/metagenomics/api/v1/analyses/MGYA00381517/file/ERR2538510_FASTQ_SSU_OTU.tsv	Root-Host_associated-Human-Skin
SRR1704695	MGYA00413071	https://www.ebi.ac.uk/metagenomics/api/v1/analyses/MGYA00413071/file/SRR1704695_FASTQ_SSU_OTU.tsv	Root-Host_associated-Human-Skin
ERR1960694	MGYA00130710	https://www.ebi.ac.uk/metagenomics/api/v1/analyses/MGYA00130710/file/ERR1960694_MERGED_FASTQ_otu.tsv	Root-Host_associated-Human-Skin
SRR920602	MGYA00022567	https://www.ebi.ac.uk/metagenomics/api/v1/analyses/MGYA00022567/file/SRR920602_FASTQ_otu.tsv	Root-Host_associated-Human-Skin
SRR994564	MGYA00024437	https://www.ebi.ac.uk/metagenomics/api/v1/analyses/MGYA00024437/file/SRR994564_FASTQ_otu.tsv	Root-Host_associated-Human-Skin
SRR994916	MGYA00024744	https://www.ebi.ac.uk/metagenomics/api/v1/analyses/MGYA00024744/file/SRR994916_FASTQ_otu.tsv	Root-Host_associated-Human-Skin

SRR995324	MGYA00022481	https://www.ebi.ac.uk/metagenomics/api/v1/analyses/MGYA00022481/file/SRR995324_FASTQ_otu.tsv	Root-Host_associated-Human-Skin
SRR996596	MGYA00023991	https://www.ebi.ac.uk/metagenomics/api/v1/analyses/MGYA00023991/file/SRR996596_FASTQ_otu.tsv	Root-Host_associated-Human-Skin
ERR450868	MGYA00426818	https://www.ebi.ac.uk/metagenomics/api/v1/analyses/MGYA00426818/file/ERR450868_FASTQ_SSU_OTU.tsv	Root-Host_associated-Human-Skin
SRR920728	MGYA00023742	https://www.ebi.ac.uk/metagenomics/api/v1/analyses/MGYA00023742/file/SRR920728_FASTQ_otu.tsv	Root-Host_associated-Human-Skin
SRR997010	MGYA00023416	https://www.ebi.ac.uk/metagenomics/api/v1/analyses/MGYA00023416/file/SRR997010_FASTQ_otu.tsv	Root-Host_associated-Human-Skin
SRR996126	MGYA00024915	https://www.ebi.ac.uk/metagenomics/api/v1/analyses/MGYA00024915/file/SRR996126_FASTQ_otu.tsv	Root-Host_associated-Human-Skin
SRR1622838	MGYA00023713	https://www.ebi.ac.uk/metagenomics/api/v1/analyses/MGYA00023713/file/SRR1622838_MERGED_FASTQ_otu.tsv	Root-Host_associated-Human-Skin
SRR1924036	MGYA00028356	https://www.ebi.ac.uk/metagenomics/api/v1/analyses/MGYA00028356/file/SRR1924036_FASTQ_otu.tsv	Root-Host_associated-Human-Skin
SRR994673	MGYA00023636	https://www.ebi.ac.uk/metagenomics/api/v1/analyses/MGYA00023636/file/SRR994673_FASTQ_otu.tsv	Root-Host_associated-Human-Skin
ERR1760023	MGYA00140358	https://www.ebi.ac.uk/metagenomics/api/v1/analyses/MGYA00140358/file/ERR1760023_MERGED_FASTQ_SSU_OTU.tsv	Root-Host_associated-Human-Skin
SRR996935	MGYA00022229	https://www.ebi.ac.uk/metagenomics/api/v1/analyses/MGYA00022229/file/SRR996935_FASTQ_otu.tsv	Root-Host_associated-Human-Skin
ERR1960637	MGYA00130866	https://www.ebi.ac.uk/metagenomics/api/v1/analyses/MGYA00130866/file/ERR1960637_MERGED_FASTQ_otu.tsv	Root-Host_associated-Human-Skin

ERR443457	MGYA00416661	https://www.ebi.ac.uk/metagenomics/api/v1/analyses/MGYA00416661/file/ERR443457_FASTQ_SSU_OTU.tsv	Root-Host_associated-Human-Skin
SRR1632853	MGYA00023051	https://www.ebi.ac.uk/metagenomics/api/v1/analyses/MGYA00023051/file/SRR1632853_MERGED_FASTQ_otu.tsv	Root-Host_associated-Human-Skin
SRR1632421	MGYA00023757	https://www.ebi.ac.uk/metagenomics/api/v1/analyses/MGYA00023757/file/SRR1632421_MERGED_FASTQ_otu.tsv	Root-Host_associated-Human-Skin
ERR443791	MGYA00415488	https://www.ebi.ac.uk/metagenomics/api/v1/analyses/MGYA00415488/file/ERR443791_FASTQ_SSU_OTU.tsv	Root-Host_associated-Human-Skin
SRR920915	MGYA00022358	https://www.ebi.ac.uk/metagenomics/api/v1/analyses/MGYA00022358/file/SRR920915_FASTQ_otu.tsv	Root-Host_associated-Human-Skin
ERR443036	MGYA00414553	https://www.ebi.ac.uk/metagenomics/api/v1/analyses/MGYA00414553/file/ERR443036_FASTQ_SSU_OTU.tsv	Root-Host_associated-Human-Skin
SRR1632386	MGYA00022374	https://www.ebi.ac.uk/metagenomics/api/v1/analyses/MGYA00022374/file/SRR1632386_MERGED_FASTQ_otu.tsv	Root-Host_associated-Human-Skin
SRR1631534	MGYA00023578	https://www.ebi.ac.uk/metagenomics/api/v1/analyses/MGYA00023578/file/SRR1631534_MERGED_FASTQ_otu.tsv	Root-Host_associated-Human-Skin
SRR1704257	MGYA00421519	https://www.ebi.ac.uk/metagenomics/api/v1/analyses/MGYA00421519/file/SRR1704257_FASTQ_SSU_OTU.tsv	Root-Host_associated-Human-Skin
SRR996369	MGYA00022507	https://www.ebi.ac.uk/metagenomics/api/v1/analyses/MGYA00022507/file/SRR996369_FASTQ_otu.tsv	Root-Host_associated-Human-Skin
SRR1950733	MGYA00376900	https://www.ebi.ac.uk/metagenomics/api/v1/analyses/MGYA00376900/file/SRR1950733_MERGED_FASTQ_SSU_OTU.tsv	Root-Host_associated-Human-Skin
ERR1759965	MGYA00140508	https://www.ebi.ac.uk/metagenomics/api/v1/analyses/MGYA00140508/file/ERR1759965_MERGED_FASTQ_SSU_OTU.tsv	Root-Host_associated-Human-Skin

SRR1623045	MGYA00024639	https://www.ebi.ac.uk/metagenomics/api/v1/analyses/MGYA00024639/file/SRR1623045_MERGED_FASTQ_otu.tsv	Root-Host_associated-Human-Skin
ERR442008	MGYA00385220	https://www.ebi.ac.uk/metagenomics/api/v1/analyses/MGYA00385220/file/ERR442008_FASTQ_SSU_OTU.tsv	Root-Host_associated-Human-Skin
SRR1704140	MGYA00413843	https://www.ebi.ac.uk/metagenomics/api/v1/analyses/MGYA00413843/file/SRR1704140_FASTQ_SSU_OTU.tsv	Root-Host_associated-Human-Skin
SRR996708	MGYA00023167	https://www.ebi.ac.uk/metagenomics/api/v1/analyses/MGYA00023167/file/SRR996708_FASTQ_otu.tsv	Root-Host_associated-Human-Skin
SRR921030	MGYA00023083	https://www.ebi.ac.uk/metagenomics/api/v1/analyses/MGYA00023083/file/SRR921030_FASTQ_otu.tsv	Root-Host_associated-Human-Skin
ERR442913	MGYA00412651	https://www.ebi.ac.uk/metagenomics/api/v1/analyses/MGYA00412651/file/ERR442913_FASTQ_SSU_OTU.tsv	Root-Host_associated-Human-Skin
SRR1633176	MGYA00025256	https://www.ebi.ac.uk/metagenomics/api/v1/analyses/MGYA00025256/file/SRR1633176_MERGED_FASTQ_otu.tsv	Root-Host_associated-Human-Skin
ERR443321	MGYA00428751	https://www.ebi.ac.uk/metagenomics/api/v1/analyses/MGYA00428751/file/ERR443321_FASTQ_SSU_OTU.tsv	Root-Host_associated-Human-Skin
SRR1630954	MGYA00024161	https://www.ebi.ac.uk/metagenomics/api/v1/analyses/MGYA00024161/file/SRR1630954_MERGED_FASTQ_otu.tsv	Root-Host_associated-Human-Skin
SRR1631366	MGYA00022589	https://www.ebi.ac.uk/metagenomics/api/v1/analyses/MGYA00022589/file/SRR1631366_MERGED_FASTQ_otu.tsv	Root-Host_associated-Human-Skin
SRR1623161	MGYA00023484	https://www.ebi.ac.uk/metagenomics/api/v1/analyses/MGYA00023484/file/SRR1623161_MERGED_FASTQ_otu.tsv	Root-Host_associated-Human-Skin
SRR1923883	MGYA00028340	https://www.ebi.ac.uk/metagenomics/api/v1/analyses/MGYA00028340/file/SRR1923883_FASTQ_otu.tsv	Root-Host_associated-Human-Skin

ERR2538446	MGYA00381453	https://www.ebi.ac.uk/metagenomics/api/v1/analyses/MGYA00381453/file/ERR2538446_FASTQ_SSU_OTU.tsv	Root-Host_associated-Human-Skin
SRR1622895	MGYA00024770	https://www.ebi.ac.uk/metagenomics/api/v1/analyses/MGYA00024770/file/SRR1622895_MERGED_FASTQ_otu.tsv	Root-Host_associated-Human-Skin
SRR5869950	MGYA00420695	https://www.ebi.ac.uk/metagenomics/api/v1/analyses/MGYA00420695/file/SRR5869950_MERGED_FASTQ_SSU_OTU.tsv	Root-Host_associated-Human-Skin
SRR5869285	MGYA00415361	https://www.ebi.ac.uk/metagenomics/api/v1/analyses/MGYA00415361/file/SRR5869285_MERGED_FASTQ_SSU_OTU.tsv	Root-Host_associated-Human-Skin
ERR451226	MGYA00428758	https://www.ebi.ac.uk/metagenomics/api/v1/analyses/MGYA00428758/file/ERR451226_FASTQ_SSU_OTU.tsv	Root-Host_associated-Human-Skin
SRR995739	MGYA00023106	https://www.ebi.ac.uk/metagenomics/api/v1/analyses/MGYA00023106/file/SRR995739_FASTQ_otu.tsv	Root-Host_associated-Human-Skin
ERR1701261	MGYA00084265	https://www.ebi.ac.uk/metagenomics/api/v1/analyses/MGYA00084265/file/ERR1701261_FASTQ_otu.tsv	Root-Host_associated-Human-Skin
ERR1960713	MGYA00130811	https://www.ebi.ac.uk/metagenomics/api/v1/analyses/MGYA00130811/file/ERR1960713_MERGED_FASTQ_otu.tsv	Root-Host_associated-Human-Skin
ERR2538497	MGYA00381504	https://www.ebi.ac.uk/metagenomics/api/v1/analyses/MGYA00381504/file/ERR2538497_FASTQ_SSU_OTU.tsv	Root-Host_associated-Human-Skin
SRR5869254	MGYA00415940	https://www.ebi.ac.uk/metagenomics/api/v1/analyses/MGYA00415940/file/SRR5869254_MERGED_FASTQ_SSU_OTU.tsv	Root-Host_associated-Human-Skin
SRR6757667	MGYA00380780	https://www.ebi.ac.uk/metagenomics/api/v1/analyses/MGYA00380780/file/SRR6757667_MERGED_FASTQ_SSU_OTU.tsv	Root-Host_associated-Human-Skin
SRR1704712	MGYA00419535	https://www.ebi.ac.uk/metagenomics/api/v1/analyses/MGYA00419535/file/SRR1704712_FASTQ_SSU_OTU.tsv	Root-Host_associated-Human-Skin

ERR450771	MGYA00426843	https://www.ebi.ac.uk/metagenomics/api/v1/analyses/MGYA00426843/file/ERR450771_FASTQ_SSU_OTU.tsv	Root-Host_associated-Human-Skin
SRR1704373	MGYA00426951	https://www.ebi.ac.uk/metagenomics/api/v1/analyses/MGYA00426951/file/SRR1704373_FASTQ_SSU_OTU.tsv	Root-Host_associated-Human-Skin
ERR1759890	MGYA00140424	https://www.ebi.ac.uk/metagenomics/api/v1/analyses/MGYA00140424/file/ERR1759890_MERGED_FASTQ_SSU_OTU.tsv	Root-Host_associated-Human-Skin
SRR5869192	MGYA00429075	https://www.ebi.ac.uk/metagenomics/api/v1/analyses/MGYA00429075/file/SRR5869192_MERGED_FASTQ_SSU_OTU.tsv	Root-Host_associated-Human-Skin
SRR996002	MGYA00023517	https://www.ebi.ac.uk/metagenomics/api/v1/analyses/MGYA00023517/file/SRR996002_FASTQ_otu.tsv	Root-Host_associated-Human-Skin
SRR994757	MGYA00023248	https://www.ebi.ac.uk/metagenomics/api/v1/analyses/MGYA00023248/file/SRR994757_FASTQ_otu.tsv	Root-Host_associated-Human-Skin
ERR451408	MGYA00419485	https://www.ebi.ac.uk/metagenomics/api/v1/analyses/MGYA00419485/file/ERR451408_FASTQ_SSU_OTU.tsv	Root-Host_associated-Human-Skin
ERR1760107	MGYA00140399	https://www.ebi.ac.uk/metagenomics/api/v1/analyses/MGYA00140399/file/ERR1760107_MERGED_FASTQ_SSU_OTU.tsv	Root-Host_associated-Human-Skin
SRR996811	MGYA00023630	https://www.ebi.ac.uk/metagenomics/api/v1/analyses/MGYA00023630/file/SRR996811_FASTQ_otu.tsv	Root-Host_associated-Human-Skin
SRR995904	MGYA00023842	https://www.ebi.ac.uk/metagenomics/api/v1/analyses/MGYA00023842/file/SRR995904_FASTQ_otu.tsv	Root-Host_associated-Human-Skin
ERR1759919	MGYA00140462	https://www.ebi.ac.uk/metagenomics/api/v1/analyses/MGYA00140462/file/ERR1759919_MERGED_FASTQ_SSU_OTU.tsv	Root-Host_associated-Human-Skin
SRR1624821	MGYA00024884	https://www.ebi.ac.uk/metagenomics/api/v1/analyses/MGYA00024884/file/SRR1624821_MERGED_FASTQ_otu.tsv	Root-Host_associated-Human-Skin

ERR441972	MGYA00385233	https://www.ebi.ac.uk/metagenomics/api/v1/analyses/MGYA00385233/file/ERR441972_FASTQ_SSU_OTU.tsv	Root-Host_associated-Human-Skin
SRR1623166	MGYA00023489	https://www.ebi.ac.uk/metagenomics/api/v1/analyses/MGYA00023489/file/SRR1623166_MERGED_FASTQ_otu.tsv	Root-Host_associated-Human-Skin
SRR1631361	MGYA00022582	https://www.ebi.ac.uk/metagenomics/api/v1/analyses/MGYA00022582/file/SRR1631361_MERGED_FASTQ_otu.tsv	Root-Host_associated-Human-Skin
SRR1704402	MGYA00419545	https://www.ebi.ac.uk/metagenomics/api/v1/analyses/MGYA00419545/file/SRR1704402_FASTQ_SSU_OTU.tsv	Root-Host_associated-Human-Skin
SRR5868704	MGYA00423061	https://www.ebi.ac.uk/metagenomics/api/v1/analyses/MGYA00423061/file/SRR5868704_MERGED_FASTQ_SSU_OTU.tsv	Root-Host_associated-Human-Skin
SRR5869144	MGYA00425434	https://www.ebi.ac.uk/metagenomics/api/v1/analyses/MGYA00425434/file/SRR5869144_MERGED_FASTQ_SSU_OTU.tsv	Root-Host_associated-Human-Skin
SRR1630953	MGYA00024166	https://www.ebi.ac.uk/metagenomics/api/v1/analyses/MGYA00024166/file/SRR1630953_MERGED_FASTQ_otu.tsv	Root-Host_associated-Human-Skin
SRR1923884	MGYA00028345	https://www.ebi.ac.uk/metagenomics/api/v1/analyses/MGYA00028345/file/SRR1923884_FASTQ_otu.tsv	Root-Host_associated-Human-Skin
SRR1922511	MGYA00028281	https://www.ebi.ac.uk/metagenomics/api/v1/analyses/MGYA00028281/file/SRR1922511_FASTQ_otu.tsv	Root-Host_associated-Human-Skin
ERR2538441	MGYA00381448	https://www.ebi.ac.uk/metagenomics/api/v1/analyses/MGYA00381448/file/ERR2538441_FASTQ_SSU_OTU.tsv	Root-Host_associated-Human-Skin
SRR5869957	MGYA00423040	https://www.ebi.ac.uk/metagenomics/api/v1/analyses/MGYA00423040/file/SRR5869957_MERGED_FASTQ_SSU_OTU.tsv	Root-Host_associated-Human-Skin
SRR1622892	MGYA00024765	https://www.ebi.ac.uk/metagenomics/api/v1/analyses/MGYA00024765/file/SRR1622892_MERGED_FASTQ_otu.tsv	Root-Host_associated-Human-Skin

ERR1701266	MGYA00084283	https://www.ebi.ac.uk/metagenomics/api/v1/analyses/MGYA00084283/file/ERR1701266_FASTQ_otu.tsv	Root-Host_associated-Human-Skin
ERR1760089	MGYA00140529	https://www.ebi.ac.uk/metagenomics/api/v1/analyses/MGYA00140529/file/ERR1760089_MERGED_FASTQ_SSU_OTU.tsv	Root-Host_associated-Human-Skin
SRR995099	MGYA00023388	https://www.ebi.ac.uk/metagenomics/api/v1/analyses/MGYA00023388/file/SRR995099_FASTQ_otu.tsv	Root-Host_associated-Human-Skin
SRR995048	MGYA00023701	https://www.ebi.ac.uk/metagenomics/api/v1/analyses/MGYA00023701/file/SRR995048_FASTQ_otu.tsv	Root-Host_associated-Human-Skin
ERR1760058	MGYA00140569	https://www.ebi.ac.uk/metagenomics/api/v1/analyses/MGYA00140569/file/ERR1760058_MERGED_FASTQ_SSU_OTU.tsv	Root-Host_associated-Human-Skin
ERR1960714	MGYA00130787	https://www.ebi.ac.uk/metagenomics/api/v1/analyses/MGYA00130787/file/ERR1960714_MERGED_FASTQ_otu.tsv	Root-Host_associated-Human-Skin
SRR5870038	MGYA00420675	https://www.ebi.ac.uk/metagenomics/api/v1/analyses/MGYA00420675/file/SRR5870038_MERGED_FASTQ_SSU_OTU.tsv	Root-Host_associated-Human-Skin
SRR6757660	MGYA00380774	https://www.ebi.ac.uk/metagenomics/api/v1/analyses/MGYA00380774/file/SRR6757660_MERGED_FASTQ_SSU_OTU.tsv	Root-Host_associated-Human-Skin
SRR5868861	MGYA00416038	https://www.ebi.ac.uk/metagenomics/api/v1/analyses/MGYA00416038/file/SRR5868861_MERGED_FASTQ_SSU_OTU.tsv	Root-Host_associated-Human-Skin
ERR2538490	MGYA00381497	https://www.ebi.ac.uk/metagenomics/api/v1/analyses/MGYA00381497/file/ERR2538490_FASTQ_SSU_OTU.tsv	Root-Host_associated-Human-Skin
SRR1923855	MGYA00028290	https://www.ebi.ac.uk/metagenomics/api/v1/analyses/MGYA00028290/file/SRR1923855_FASTQ_otu.tsv	Root-Host_associated-Human-Skin
ERR1759897	MGYA00140551	https://www.ebi.ac.uk/metagenomics/api/v1/analyses/MGYA00140551/file/ERR1759897_MERGED_FASTQ_SSU_OTU.tsv	Root-Host_associated-Human-Skin

SRR1704374	MGYA00428877	https://www.ebi.ac.uk/metagenomics/api/v1/analyses/MGYA00428877/file/SRR1704374_FASTQ_SSU_OTU.tsv	Root-Host_associated-Human-Skin
SRR1631689	MGYA00024825	https://www.ebi.ac.uk/metagenomics/api/v1/analyses/MGYA00024825/file/SRR1631689_MERGED_FASTQ_otu.tsv	Root-Host_associated-Human-Skin
SRR996005	MGYA00023516	https://www.ebi.ac.uk/metagenomics/api/v1/analyses/MGYA00023516/file/SRR996005_FASTQ_otu.tsv	Root-Host_associated-Human-Skin
ERR1760100	MGYA00140493	https://www.ebi.ac.uk/metagenomics/api/v1/analyses/MGYA00140493/file/ERR1760100_MERGED_FASTQ_SSU_OTU.tsv	Root-Host_associated-Human-Skin
SRR994750	MGYA00023241	https://www.ebi.ac.uk/metagenomics/api/v1/analyses/MGYA00023241/file/SRR994750_FASTQ_otu.tsv	Root-Host_associated-Human-Skin
SRR996816	MGYA00023629	https://www.ebi.ac.uk/metagenomics/api/v1/analyses/MGYA00023629/file/SRR996816_FASTQ_otu.tsv	Root-Host_associated-Human-Skin
SRR995903	MGYA00023837	https://www.ebi.ac.uk/metagenomics/api/v1/analyses/MGYA00023837/file/SRR995903_FASTQ_otu.tsv	Root-Host_associated-Human-Skin
SRR5870277	MGYA00426189	https://www.ebi.ac.uk/metagenomics/api/v1/analyses/MGYA00426189/file/SRR5870277_MERGED_FASTQ_SSU_OTU.tsv	Root-Host_associated-Human-Skin
SRR994835	MGYA00024588	https://www.ebi.ac.uk/metagenomics/api/v1/analyses/MGYA00024588/file/SRR994835_FASTQ_otu.tsv	Root-Host_associated-Human-Skin
SRR1950748	MGYA00376915	https://www.ebi.ac.uk/metagenomics/api/v1/analyses/MGYA00376915/file/SRR1950748_MERGED_FASTQ_SSU_OTU.tsv	Root-Host_associated-Human-Skin
SRR1624826	MGYA00024889	https://www.ebi.ac.uk/metagenomics/api/v1/analyses/MGYA00024889/file/SRR1624826_MERGED_FASTQ_otu.tsv	Root-Host_associated-Human-Skin
ERR451379	MGYA00412615	https://www.ebi.ac.uk/metagenomics/api/v1/analyses/MGYA00412615/file/ERR451379_FASTQ_SSU_OTU.tsv	Root-Host_associated-Human-Skin

SRR995666	MGYA00024843	https://www.ebi.ac.uk/metagenomics/api/v1/analyses/MGYA00024843/file/SRR995666_FASTQ_otu.tsv	Root-Host_associated-Human-Skin
ERR441975	MGYA00385184	https://www.ebi.ac.uk/metagenomics/api/v1/analyses/MGYA00385184/file/ERR441975_FASTQ_SSU_OTU.tsv	Root-Host_associated-Human-Skin
ERR2538519	MGYA00381526	https://www.ebi.ac.uk/metagenomics/api/v1/analyses/MGYA00381526/file/ERR2538519_FASTQ_SSU_OTU.tsv	Root-Host_associated-Human-Skin
SRR995323	MGYA00022476	https://www.ebi.ac.uk/metagenomics/api/v1/analyses/MGYA00022476/file/SRR995323_FASTQ_otu.tsv	Root-Host_associated-Human-Skin
SRR994563	MGYA00024430	https://www.ebi.ac.uk/metagenomics/api/v1/analyses/MGYA00024430/file/SRR994563_FASTQ_otu.tsv	Root-Host_associated-Human-Skin
SRR5868778	MGYA00423487	https://www.ebi.ac.uk/metagenomics/api/v1/analyses/MGYA00423487/file/SRR5868778_MERGED_FASTQ_SSU_OTU.tsv	Root-Host_associated-Human-Skin
SRR996591	MGYA00023994	https://www.ebi.ac.uk/metagenomics/api/v1/analyses/MGYA00023994/file/SRR996591_FASTQ_otu.tsv	Root-Host_associated-Human-Skin
SRR1921019	MGYA00028310	https://www.ebi.ac.uk/metagenomics/api/v1/analyses/MGYA00028310/file/SRR1921019_FASTQ_otu.tsv	Root-Host_associated-Human-Skin
SRR997017	MGYA00023411	https://www.ebi.ac.uk/metagenomics/api/v1/analyses/MGYA00023411/file/SRR997017_FASTQ_otu.tsv	Root-Host_associated-Human-Skin
SRR1924031	MGYA00028270	https://www.ebi.ac.uk/metagenomics/api/v1/analyses/MGYA00028270/file/SRR1924031_FASTQ_otu.tsv	Root-Host_associated-Human-Skin
SRR996121	MGYA00024862	https://www.ebi.ac.uk/metagenomics/api/v1/analyses/MGYA00024862/file/SRR996121_FASTQ_otu.tsv	Root-Host_associated-Human-Skin
ERR1760024	MGYA00140501	https://www.ebi.ac.uk/metagenomics/api/v1/analyses/MGYA00140501/file/ERR1760024_MERGED_FASTQ_SSU_OTU.tsv	Root-Host_associated-Human-Skin

SRR5870044	MGYA00426220	https://www.ebi.ac.uk/metagenomics/api/v1/analyses/MGYA00426220/file/SRR5870044_MERGED_FASTQ_SSU_OTU.tsv	Root-Host_associated-Human-Skin
SRR994674	MGYA00023643	https://www.ebi.ac.uk/metagenomics/api/v1/analyses/MGYA00023643/file/SRR994674_FASTQ_otu.tsv	Root-Host_associated-Human-Skin
ERR1960768	MGYA00130873	https://www.ebi.ac.uk/metagenomics/api/v1/analyses/MGYA00130873/file/ERR1960768_MERGED_FASTQ_otu.tsv	Root-Host_associated-Human-Skin
SRR996932	MGYA00022222	https://www.ebi.ac.uk/metagenomics/api/v1/analyses/MGYA00022222/file/SRR996932_FASTQ_otu.tsv	Root-Host_associated-Human-Skin
SRR995455	MGYA00025306	https://www.ebi.ac.uk/metagenomics/api/v1/analyses/MGYA00025306/file/SRR995455_FASTQ_otu.tsv	Root-Host_associated-Human-Skin
ERR443450	MGYA00416269	https://www.ebi.ac.uk/metagenomics/api/v1/analyses/MGYA00416269/file/ERR443450_FASTQ_SSU_OTU.tsv	Root-Host_associated-Human-Skin
ERR1960630	MGYA00130739	https://www.ebi.ac.uk/metagenomics/api/v1/analyses/MGYA00130739/file/ERR1960630_MERGED_FASTQ_otu.tsv	Root-Host_associated-Human-Skin
SRR1704631	MGYA00421467	https://www.ebi.ac.uk/metagenomics/api/v1/analyses/MGYA00421467/file/SRR1704631_FASTQ_SSU_OTU.tsv	Root-Host_associated-Human-Skin
SRR920912	MGYA00022357	https://www.ebi.ac.uk/metagenomics/api/v1/analyses/MGYA00022357/file/SRR920912_FASTQ_otu.tsv	Root-Host_associated-Human-Skin
SRR1632381	MGYA00022373	https://www.ebi.ac.uk/metagenomics/api/v1/analyses/MGYA00022373/file/SRR1632381_MERGED_FASTQ_otu.tsv	Root-Host_associated-Human-Skin
SRR1632426	MGYA00023753	https://www.ebi.ac.uk/metagenomics/api/v1/analyses/MGYA00023753/file/SRR1632426_MERGED_FASTQ_otu.tsv	Root-Host_associated-Human-Skin
SRR1632854	MGYA00023048	https://www.ebi.ac.uk/metagenomics/api/v1/analyses/MGYA00023048/file/SRR1632854_MERGED_FASTQ_otu.tsv	Root-Host_associated-Human-Skin

SRR996418	MGYA00024306	https://www.ebi.ac.uk/metagenomics/api/v1/analyses/MGYA00024306/file/SRR996418_FASTQ_otu.tsv	Root-Host_associated-Human-Skin
SRR1704250	MGYA00422442	https://www.ebi.ac.uk/metagenomics/api/v1/analyses/MGYA00422442/file/SRR1704250_FASTQ_SSU_OTU.tsv	Root-Host_associated-Human-Skin
SRR1631533	MGYA00023585	https://www.ebi.ac.uk/metagenomics/api/v1/analyses/MGYA00023585/file/SRR1631533_MERGED_FASTQ_otu.tsv	Root-Host_associated-Human-Skin
SRR5868620	MGYA00428096	https://www.ebi.ac.uk/metagenomics/api/v1/analyses/MGYA00428096/file/SRR5868620_MERGED_FASTQ_SSU_OTU.tsv	Root-Host_associated-Human-Skin
ERR1759962	MGYA00140602	https://www.ebi.ac.uk/metagenomics/api/v1/analyses/MGYA00140602/file/ERR1759962_MERGED_FASTQ_SSU_OTU.tsv	Root-Host_associated-Human-Skin
SRR1950734	MGYA00376901	https://www.ebi.ac.uk/metagenomics/api/v1/analyses/MGYA00376901/file/SRR1950734_MERGED_FASTQ_SSU_OTU.tsv	Root-Host_associated-Human-Skin
SRR5869060	MGYA00416021	https://www.ebi.ac.uk/metagenomics/api/v1/analyses/MGYA00416021/file/SRR5869060_MERGED_FASTQ_SSU_OTU.tsv	Root-Host_associated-Human-Skin
SRR994849	MGYA00022602	https://www.ebi.ac.uk/metagenomics/api/v1/analyses/MGYA00022602/file/SRR994849_FASTQ_otu.tsv	Root-Host_associated-Human-Skin
SRR5869401	MGYA00419689	https://www.ebi.ac.uk/metagenomics/api/v1/analyses/MGYA00419689/file/SRR5869401_MERGED_FASTQ_SSU_OTU.tsv	Root-Host_associated-Human-Skin
ERR443481	MGYA00414541	https://www.ebi.ac.uk/metagenomics/api/v1/analyses/MGYA00414541/file/ERR443481_FASTQ_SSU_OTU.tsv	Root-Host_associated-Human-Skin
SRR921037	MGYA00023080	https://www.ebi.ac.uk/metagenomics/api/v1/analyses/MGYA00023080/file/SRR921037_FASTQ_otu.tsv	Root-Host_associated-Human-Skin
ERR442914	MGYA00415189	https://www.ebi.ac.uk/metagenomics/api/v1/analyses/MGYA00415189/file/ERR442914_FASTQ_SSU_OTU.tsv	Root-Host_associated-Human-Skin

ERR451103	MGYA00420480	https://www.ebi.ac.uk/metagenomics/api/v1/analyses/MGYA00420480/file/ERR451103_FASTQ_SSU_OTU.tsv	Root-Host_associated-Human-Skin
SRR1923860	MGYA00028317	https://www.ebi.ac.uk/metagenomics/api/v1/analyses/MGYA00028317/file/SRR1923860_FASTQ_otu.tsv	Root-Host_associated-Human-Skin
SRR1631385	MGYA00024086	https://www.ebi.ac.uk/metagenomics/api/v1/analyses/MGYA00024086/file/SRR1631385_MERGED_FASTQ_otu.tsv	Root-Host_associated-Human-Skin
ERR451562	MGYA00415116	https://www.ebi.ac.uk/metagenomics/api/v1/analyses/MGYA00415116/file/ERR451562_FASTQ_SSU_OTU.tsv	Root-Host_associated-Human-Skin
ERR443541	MGYA00413336	https://www.ebi.ac.uk/metagenomics/api/v1/analyses/MGYA00413336/file/ERR443541_FASTQ_SSU_OTU.tsv	Root-Host_associated-Human-Skin
ERR1701282	MGYA00084239	https://www.ebi.ac.uk/metagenomics/api/v1/analyses/MGYA00084239/file/ERR1701282_FASTQ_otu.tsv	Root-Host_associated-Human-Skin
ERR1960721	MGYA00130880	https://www.ebi.ac.uk/metagenomics/api/v1/analyses/MGYA00130880/file/ERR1960721_MERGED_FASTQ_otu.tsv	Root-Host_associated-Human-Skin
SRR1704720	MGYA00425793	https://www.ebi.ac.uk/metagenomics/api/v1/analyses/MGYA00425793/file/SRR1704720_FASTQ_SSU_OTU.tsv	Root-Host_associated-Human-Skin
SRR1631043	MGYA00025010	https://www.ebi.ac.uk/metagenomics/api/v1/analyses/MGYA00025010/file/SRR1631043_MERGED_FASTQ_otu.tsv	Root-Host_associated-Human-Skin
SRR6757655	MGYA00380770	https://www.ebi.ac.uk/metagenomics/api/v1/analyses/MGYA00380770/file/SRR6757655_MERGED_FASTQ_SSU_OTU.tsv	Root-Host_associated-Human-Skin
SRR996168	MGYA00022295	https://www.ebi.ac.uk/metagenomics/api/v1/analyses/MGYA00022295/file/SRR996168_FASTQ_otu.tsv	Root-Host_associated-Human-Skin
SRR1622876	MGYA00024181	https://www.ebi.ac.uk/metagenomics/api/v1/analyses/MGYA00024181/file/SRR1622876_MERGED_FASTQ_otu.tsv	Root-Host_associated-Human-Skin

SRR5869510	MGYA00419655	https://www.ebi.ac.uk/metagenomics/api/v1/analyses/MGYA00419655/file/SRR5869510_MERGED_FASTQ_SSU_OTU.tsv	Root-Host_associated-Human-Skin
ERR2538474	MGYA00381481	https://www.ebi.ac.uk/metagenomics/api/v1/analyses/MGYA00381481/file/ERR2538474_FASTQ_SSU_OTU.tsv	Root-Host_associated-Human-Skin
SRR6757684	MGYA00380797	https://www.ebi.ac.uk/metagenomics/api/v1/analyses/MGYA00380797/file/SRR6757684_MERGED_FASTQ_SSU_OTU.tsv	Root-Host_associated-Human-Skin
ERR1701253	MGYA00084300	https://www.ebi.ac.uk/metagenomics/api/v1/analyses/MGYA00084300/file/ERR1701253_FASTQ_otu.tsv	Root-Host_associated-Human-Skin
ERR443590	MGYA00425755	https://www.ebi.ac.uk/metagenomics/api/v1/analyses/MGYA00425755/file/ERR443590_FASTQ_SSU_OTU.tsv	Root-Host_associated-Human-Skin
ERR451214	MGYA00420132	https://www.ebi.ac.uk/metagenomics/api/v1/analyses/MGYA00420132/file/ERR451214_FASTQ_SSU_OTU.tsv	Root-Host_associated-Human-Skin
SRR921126	MGYA00025186	https://www.ebi.ac.uk/metagenomics/api/v1/analyses/MGYA00025186/file/SRR921126_FASTQ_otu.tsv	Root-Host_associated-Human-Skin
SRR1704390	MGYA00428868	https://www.ebi.ac.uk/metagenomics/api/v1/analyses/MGYA00428868/file/SRR1704390_FASTQ_SSU_OTU.tsv	Root-Host_associated-Human-Skin
SRR994958	MGYA00024345	https://www.ebi.ac.uk/metagenomics/api/v1/analyses/MGYA00024345/file/SRR994958_FASTQ_otu.tsv	Root-Host_associated-Human-Skin
SRR1632994	MGYA00022267	https://www.ebi.ac.uk/metagenomics/api/v1/analyses/MGYA00022267/file/SRR1632994_MERGED_FASTQ_otu.tsv	Root-Host_associated-Human-Skin
SRR5870184	MGYA00425419	https://www.ebi.ac.uk/metagenomics/api/v1/analyses/MGYA00425419/file/SRR5870184_MERGED_FASTQ_SSU_OTU.tsv	Root-Host_associated-Human-Skin
SRR920199	MGYA00023260	https://www.ebi.ac.uk/metagenomics/api/v1/analyses/MGYA00023260/file/SRR920199_FASTQ_otu.tsv	Root-Host_associated-Human-Skin

SRR1633138	MGYA00024879	https://www.ebi.ac.uk/metagenomics/api/v1/analyses/MGYA00024879/file/SRR1633138_MERGED_FASTQ_otu.tsv	Root-Host_associated-Human-Skin
SRR5869448	MGYA00419672	https://www.ebi.ac.uk/metagenomics/api/v1/analyses/MGYA00419672/file/SRR5869448_MERGED_FASTQ_SSU_OTU.tsv	Root-Host_associated-Human-Skin
SRR996746	MGYA00023613	https://www.ebi.ac.uk/metagenomics/api/v1/analyses/MGYA00023613/file/SRR996746_FASTQ_otu.tsv	Root-Host_associated-Human-Skin
SRR995595	MGYA00025201	https://www.ebi.ac.uk/metagenomics/api/v1/analyses/MGYA00025201/file/SRR995595_FASTQ_otu.tsv	Root-Host_associated-Human-Skin
ERR442046	MGYA00385239	https://www.ebi.ac.uk/metagenomics/api/v1/analyses/MGYA00385239/file/ERR442046_FASTQ_SSU_OTU.tsv	Root-Host_associated-Human-Skin
SRR994472	MGYA00022253	https://www.ebi.ac.uk/metagenomics/api/v1/analyses/MGYA00022253/file/SRR994472_FASTQ_otu.tsv	Root-Host_associated-Human-Skin
SRR994800	MGYA00023026	https://www.ebi.ac.uk/metagenomics/api/v1/analyses/MGYA00023026/file/SRR994800_FASTQ_otu.tsv	Root-Host_associated-Human-Skin
SRR996327	MGYA00022946	https://www.ebi.ac.uk/metagenomics/api/v1/analyses/MGYA00022946/file/SRR996327_FASTQ_otu.tsv	Root-Host_associated-Human-Skin
SRR995544	MGYA00022746	https://www.ebi.ac.uk/metagenomics/api/v1/analyses/MGYA00022746/file/SRR995544_FASTQ_otu.tsv	Root-Host_associated-Human-Skin
SRR1923938	MGYA00028263	https://www.ebi.ac.uk/metagenomics/api/v1/analyses/MGYA00028263/file/SRR1923938_FASTQ_otu.tsv	Root-Host_associated-Human-Skin
ERR441991	MGYA00385161	https://www.ebi.ac.uk/metagenomics/api/v1/analyses/MGYA00385161/file/ERR441991_FASTQ_SSU_OTU.tsv	Root-Host_associated-Human-Skin
SRR919633	MGYA00023766	https://www.ebi.ac.uk/metagenomics/api/v1/analyses/MGYA00023766/file/SRR919633_FASTQ_otu.tsv	Root-Host_associated-Human-Skin

SRR995125	MGYA00023922	https://www.ebi.ac.uk/metagenomics/api/v1/analyses/MGYA00023922/file/SRR995125_FASTQ_otu.tsv	Root-Host_associated-Human-Skin
ERR1760135	MGYA00140531	https://www.ebi.ac.uk/metagenomics/api/v1/analyses/MGYA00140531/file/ERR1760135_MERGED_FASTQ_SSU_OTU.tsv	Root-Host_associated-Human-Skin
ERR1960679	MGYA00130905	https://www.ebi.ac.uk/metagenomics/api/v1/analyses/MGYA00130905/file/ERR1960679_MERGED_FASTQ_otu.tsv	Root-Host_associated-Human-Skin
SRR996575	MGYA00022420	https://www.ebi.ac.uk/metagenomics/api/v1/analyses/MGYA00022420/file/SRR996575_FASTQ_otu.tsv	Root-Host_associated-Human-Skin
SRR996907	MGYA00025321	https://www.ebi.ac.uk/metagenomics/api/v1/analyses/MGYA00025321/file/SRR996907_FASTQ_otu.tsv	Root-Host_associated-Human-Skin
SRR1950688	MGYA00376855	https://www.ebi.ac.uk/metagenomics/api/v1/analyses/MGYA00376855/file/SRR1950688_MERGED_FASTQ_SSU_OTU.tsv	Root-Host_associated-Human-Skin
SRR994587	MGYA00022929	https://www.ebi.ac.uk/metagenomics/api/v1/analyses/MGYA00022929/file/SRR994587_FASTQ_otu.tsv	Root-Host_associated-Human-Skin
SRR6757629	MGYA00380746	https://www.ebi.ac.uk/metagenomics/api/v1/analyses/MGYA00380746/file/SRR6757629_MERGED_FASTQ_SSU_OTU.tsv	Root-Host_associated-Human-Skin
SRR1924004	MGYA00028285	https://www.ebi.ac.uk/metagenomics/api/v1/analyses/MGYA00028285/file/SRR1924004_FASTQ_otu.tsv	Root-Host_associated-Human-Skin
SRR5868828	MGYA00420286	https://www.ebi.ac.uk/metagenomics/api/v1/analyses/MGYA00420286/file/SRR5868828_MERGED_FASTQ_SSU_OTU.tsv	Root-Host_associated-Human-Skin
SRR996114	MGYA00025175	https://www.ebi.ac.uk/metagenomics/api/v1/analyses/MGYA00025175/file/SRR996114_FASTQ_otu.tsv	Root-Host_associated-Human-Skin
ERR1760011	MGYA00140391	https://www.ebi.ac.uk/metagenomics/api/v1/analyses/MGYA00140391/file/ERR1760011_MERGED_FASTQ_SSU_OTU.tsv	Root-Host_associated-Human-Skin

SRR995001	MGYA00024146	https://www.ebi.ac.uk/metagenomics/api/v1/analyses/MGYA00024146/file/SRR995001_FASTQ_otu.tsv	Root-Host_associated-Human-Skin
SRR994690	MGYA00023875	https://www.ebi.ac.uk/metagenomics/api/v1/analyses/MGYA00023875/file/SRR994690_FASTQ_otu.tsv	Root-Host_associated-Human-Skin
SRR1923779	MGYA00028223	https://www.ebi.ac.uk/metagenomics/api/v1/analyses/MGYA00028223/file/SRR1923779_FASTQ_otu.tsv	Root-Host_associated-Human-Skin
SRR996662	MGYA00024776	https://www.ebi.ac.uk/metagenomics/api/v1/analyses/MGYA00024776/file/SRR996662_FASTQ_otu.tsv	Root-Host_associated-Human-Skin
ERR2538408	MGYA00381415	https://www.ebi.ac.uk/metagenomics/api/v1/analyses/MGYA00381415/file/ERR2538408_FASTQ_SSU_OTU.tsv	Root-Host_associated-Human-Skin
SRR997022	MGYA00024266	https://www.ebi.ac.uk/metagenomics/api/v1/analyses/MGYA00024266/file/SRR997022_FASTQ_otu.tsv	Root-Host_associated-Human-Skin
SRR996203	MGYA00024175	https://www.ebi.ac.uk/metagenomics/api/v1/analyses/MGYA00024175/file/SRR996203_FASTQ_otu.tsv	Root-Host_associated-Human-Skin
SRR5869846	MGYA00412476	https://www.ebi.ac.uk/metagenomics/api/v1/analyses/MGYA00412476/file/SRR5869846_MERGED_FASTQ_SSU_OTU.tsv	Root-Host_associated-Human-Skin
SRR5869434	MGYA00415341	https://www.ebi.ac.uk/metagenomics/api/v1/analyses/MGYA00415341/file/SRR5869434_MERGED_FASTQ_SSU_OTU.tsv	Root-Host_associated-Human-Skin
ERR442921	MGYA00416710	https://www.ebi.ac.uk/metagenomics/api/v1/analyses/MGYA00416710/file/ERR442921_FASTQ_SSU_OTU.tsv	Root-Host_associated-Human-Skin
SRR1633144	MGYA00022604	https://www.ebi.ac.uk/metagenomics/api/v1/analyses/MGYA00022604/file/SRR1633144_MERGED_FASTQ_otu.tsv	Root-Host_associated-Human-Skin
ERR1759957	MGYA00140420	https://www.ebi.ac.uk/metagenomics/api/v1/analyses/MGYA00140420/file/ERR1759957_MERGED_FASTQ_SSU_OTU.tsv	Root-Host_associated-Human-Skin

SRR5868615	MGYA00421668	https://www.ebi.ac.uk/metagenomics/api/v1/analyses/MGYA00421668/file/SRR5868615_MERGED_FASTQ_SSU_OTU.tsv	Root-Host_associated-Human-Skin
SRR1631270	MGYA00024733	https://www.ebi.ac.uk/metagenomics/api/v1/analyses/MGYA00024733/file/SRR1631270_MERGED_FASTQ_otu.tsv	Root-Host_associated-Human-Skin
SRR919558	MGYA00024505	https://www.ebi.ac.uk/metagenomics/api/v1/analyses/MGYA00024505/file/SRR919558_FASTQ_otu.tsv	Root-Host_associated-Human-Skin
SRR1950701	MGYA00376868	https://www.ebi.ac.uk/metagenomics/api/v1/analyses/MGYA00376868/file/SRR1950701_MERGED_FASTQ_SSU_OTU.tsv	Root-Host_associated-Human-Skin
SRR920223	MGYA00023288	https://www.ebi.ac.uk/metagenomics/api/v1/analyses/MGYA00023288/file/SRR920223_FASTQ_otu.tsv	Root-Host_associated-Human-Skin
ERR443772	MGYA00424258	https://www.ebi.ac.uk/metagenomics/api/v1/analyses/MGYA00424258/file/ERR443772_FASTQ_SSU_OTU.tsv	Root-Host_associated-Human-Skin
SRR920584	MGYA00024250	https://www.ebi.ac.uk/metagenomics/api/v1/analyses/MGYA00024250/file/SRR920584_FASTQ_otu.tsv	Root-Host_associated-Human-Skin
SRR1632413	MGYA00023825	https://www.ebi.ac.uk/metagenomics/api/v1/analyses/MGYA00023825/file/SRR1632413_MERGED_FASTQ_otu.tsv	Root-Host_associated-Human-Skin
SRR1632861	MGYA00023343	https://www.ebi.ac.uk/metagenomics/api/v1/analyses/MGYA00023343/file/SRR1632861_MERGED_FASTQ_otu.tsv	Root-Host_associated-Human-Skin
SRR1923944	MGYA00028227	https://www.ebi.ac.uk/metagenomics/api/v1/analyses/MGYA00028227/file/SRR1923944_FASTQ_otu.tsv	Root-Host_associated-Human-Skin
ERR451027	MGYA00421432	https://www.ebi.ac.uk/metagenomics/api/v1/analyses/MGYA00421432/file/ERR451027_FASTQ_SSU_OTU.tsv	Root-Host_associated-Human-Skin
SRR5869084	MGYA00417037	https://www.ebi.ac.uk/metagenomics/api/v1/analyses/MGYA00417037/file/SRR5869084_MERGED_FASTQ_SSU_OTU.tsv	Root-Host_associated-Human-Skin

SRR2005706	MGYA00381337	https://www.ebi.ac.uk/metagenomics/api/v1/analyses/MGYA00381337/file/SRR2005706_MERGED_FASTQ_SSU_OTU.tsv	Root-Host_associated-Human-Skin
ERR1759986	MGYA00140404	https://www.ebi.ac.uk/metagenomics/api/v1/analyses/MGYA00140404/file/ERR1759986_MERGED_FASTQ_SSU_OTU.tsv	Root-Host_associated-Human-Skin
SRR920693	MGYA00024839	https://www.ebi.ac.uk/metagenomics/api/v1/analyses/MGYA00024839/file/SRR920693_FASTQ_otu.tsv	Root-Host_associated-Human-Skin
SRR994719	MGYA00025095	https://www.ebi.ac.uk/metagenomics/api/v1/analyses/MGYA00025095/file/SRR994719_FASTQ_otu.tsv	Root-Host_associated-Human-Skin
SRR920134	MGYA00024320	https://www.ebi.ac.uk/metagenomics/api/v1/analyses/MGYA00024320/file/SRR920134_FASTQ_otu.tsv	Root-Host_associated-Human-Skin
SRR1631459	MGYA00025116	https://www.ebi.ac.uk/metagenomics/api/v1/analyses/MGYA00025116/file/SRR1631459_MERGED_FASTQ_otu.tsv	Root-Host_associated-Human-Skin
SRR996572	MGYA00022413	https://www.ebi.ac.uk/metagenomics/api/v1/analyses/MGYA00022413/file/SRR996572_FASTQ_otu.tsv	Root-Host_associated-Human-Skin
SRR2005659	MGYA00381317	https://www.ebi.ac.uk/metagenomics/api/v1/analyses/MGYA00381317/file/SRR2005659_MERGED_FASTQ_SSU_OTU.tsv	Root-Host_associated-Human-Skin
ERR450738	MGYA00415580	https://www.ebi.ac.uk/metagenomics/api/v1/analyses/MGYA00415580/file/ERR450738_FASTQ_SSU_OTU.tsv	Root-Host_associated-Human-Skin
SRR994580	MGYA00022924	https://www.ebi.ac.uk/metagenomics/api/v1/analyses/MGYA00022924/file/SRR994580_FASTQ_otu.tsv	Root-Host_associated-Human-Skin
SRR996113	MGYA00025174	https://www.ebi.ac.uk/metagenomics/api/v1/analyses/MGYA00025174/file/SRR996113_FASTQ_otu.tsv	Root-Host_associated-Human-Skin
ERR2538379	MGYA00381386	https://www.ebi.ac.uk/metagenomics/api/v1/analyses/MGYA00381386/file/ERR2538379_FASTQ_SSU_OTU.tsv	Root-Host_associated-Human-Skin

ERR1760016	MGYA00140472	https://www.ebi.ac.uk/metagenomics/api/v1/analyses/MGYA00140472/file/ERR1760016_MERGED_FASTQ_SSU_OTU.tsv	Root-Host_associated-Human-Skin
SRR995006	MGYA00024149	https://www.ebi.ac.uk/metagenomics/api/v1/analyses/MGYA00024149/file/SRR995006_FASTQ_otu.tsv	Root-Host_associated-Human-Skin
SRR994697	MGYA00023880	https://www.ebi.ac.uk/metagenomics/api/v1/analyses/MGYA00023880/file/SRR994697_FASTQ_otu.tsv	Root-Host_associated-Human-Skin
ERR1701228	MGYA00084270	https://www.ebi.ac.uk/metagenomics/api/v1/analyses/MGYA00084270/file/ERR1701228_FASTQ_otu.tsv	Root-Host_associated-Human-Skin
SRR996665	MGYA00024775	https://www.ebi.ac.uk/metagenomics/api/v1/analyses/MGYA00024775/file/SRR996665_FASTQ_otu.tsv	Root-Host_associated-Human-Skin
SRR996204	MGYA00024168	https://www.ebi.ac.uk/metagenomics/api/v1/analyses/MGYA00024168/file/SRR996204_FASTQ_otu.tsv	Root-Host_associated-Human-Skin
SRR1631488	MGYA00024916	https://www.ebi.ac.uk/metagenomics/api/v1/analyses/MGYA00024916/file/SRR1631488_MERGED_FASTQ_otu.tsv	Root-Host_associated-Human-Skin
SRR1633143	MGYA00022605	https://www.ebi.ac.uk/metagenomics/api/v1/analyses/MGYA00022605/file/SRR1633143_MERGED_FASTQ_otu.tsv	Root-Host_associated-Human-Skin
ERR451337	MGYA00418479	https://www.ebi.ac.uk/metagenomics/api/v1/analyses/MGYA00418479/file/ERR451337_FASTQ_SSU_OTU.tsv	Root-Host_associated-Human-Skin
SRR995628	MGYA00022716	https://www.ebi.ac.uk/metagenomics/api/v1/analyses/MGYA00022716/file/SRR995628_FASTQ_otu.tsv	Root-Host_associated-Human-Skin
SRR1624868	MGYA00024462	https://www.ebi.ac.uk/metagenomics/api/v1/analyses/MGYA00024462/file/SRR1624868_MERGED_FASTQ_otu.tsv	Root-Host_associated-Human-Skin
ERR443314	MGYA00422365	https://www.ebi.ac.uk/metagenomics/api/v1/analyses/MGYA00422365/file/ERR443314_FASTQ_SSU_OTU.tsv	Root-Host_associated-Human-Skin

SRR1950706	MGYA00376873	https://www.ebi.ac.uk/metagenomics/api/v1/analyses/MGYA00376873/file/SRR1950706_MERGED_FASTQ_SSU_OTU.tsv	Root-Host_associated-Human-Skin
SRR5868612	MGYA00428104	https://www.ebi.ac.uk/metagenomics/api/v1/analyses/MGYA00428104/file/SRR5868612_MERGED_FASTQ_SSU_OTU.tsv	Root-Host_associated-Human-Skin
SRR1631277	MGYA00024734	https://www.ebi.ac.uk/metagenomics/api/v1/analyses/MGYA00024734/file/SRR1631277_MERGED_FASTQ_otu.tsv	Root-Host_associated-Human-Skin
SRR1704514	MGYA00428445	https://www.ebi.ac.uk/metagenomics/api/v1/analyses/MGYA00428445/file/SRR1704514_FASTQ_SSU_OTU.tsv	Root-Host_associated-Human-Skin
ERR1759950	MGYA00140362	https://www.ebi.ac.uk/metagenomics/api/v1/analyses/MGYA00140362/file/ERR1759950_MERGED_FASTQ_SSU_OTU.tsv	Root-Host_associated-Human-Skin
SRR920583	MGYA00024253	https://www.ebi.ac.uk/metagenomics/api/v1/analyses/MGYA00024253/file/SRR920583_FASTQ_otu.tsv	Root-Host_associated-Human-Skin
SRR994409	MGYA00025137	https://www.ebi.ac.uk/metagenomics/api/v1/analyses/MGYA00025137/file/SRR994409_FASTQ_otu.tsv	Root-Host_associated-Human-Skin
SRR920224	MGYA00023287	https://www.ebi.ac.uk/metagenomics/api/v1/analyses/MGYA00023287/file/SRR920224_FASTQ_otu.tsv	Root-Host_associated-Human-Skin
ERR451020	MGYA00421097	https://www.ebi.ac.uk/metagenomics/api/v1/analyses/MGYA00421097/file/ERR451020_FASTQ_SSU_OTU.tsv	Root-Host_associated-Human-Skin
SRR995298	MGYA00023590	https://www.ebi.ac.uk/metagenomics/api/v1/analyses/MGYA00023590/file/SRR995298_FASTQ_otu.tsv	Root-Host_associated-Human-Skin
SRR1923943	MGYA00028228	https://www.ebi.ac.uk/metagenomics/api/v1/analyses/MGYA00028228/file/SRR1923943_FASTQ_otu.tsv	Root-Host_associated-Human-Skin
SRR1632414	MGYA00023814	https://www.ebi.ac.uk/metagenomics/api/v1/analyses/MGYA00023814/file/SRR1632414_MERGED_FASTQ_otu.tsv	Root-Host_associated-Human-Skin

ERR1759981	MGYA00140437	https://www.ebi.ac.uk/metagenomics/api/v1/analyses/MGYA00140437/file/ERR1759981_MERGED_FASTQ_SSU_OTU.tsv	Root-Host_associated-Human-Skin
SRR1704262	MGYA00428898	https://www.ebi.ac.uk/metagenomics/api/v1/analyses/MGYA00428898/file/SRR1704262_FASTQ_SSU_OTU.tsv	Root-Host_associated-Human-Skin
SRR996858	MGYA00023224	https://www.ebi.ac.uk/metagenomics/api/v1/analyses/MGYA00023224/file/SRR996858_FASTQ_otu.tsv	Root-Host_associated-Human-Skin
SRR5869083	MGYA00417038	https://www.ebi.ac.uk/metagenomics/api/v1/analyses/MGYA00417038/file/SRR5869083_MERGED_FASTQ_SSU_OTU.tsv	Root-Host_associated-Human-Skin
ERR443462	MGYA00419151	https://www.ebi.ac.uk/metagenomics/api/v1/analyses/MGYA00419151/file/ERR443462_FASTQ_SSU_OTU.tsv	Root-Host_associated-Human-Skin
SRR920133	MGYA00022972	https://www.ebi.ac.uk/metagenomics/api/v1/analyses/MGYA00022972/file/SRR920133_FASTQ_otu.tsv	Root-Host_associated-Human-Skin
SRR920694	MGYA00024836	https://www.ebi.ac.uk/metagenomics/api/v1/analyses/MGYA00024836/file/SRR920694_FASTQ_otu.tsv	Root-Host_associated-Human-Skin
ERR451441	MGYA00421399	https://www.ebi.ac.uk/metagenomics/api/v1/analyses/MGYA00421399/file/ERR451441_FASTQ_SSU_OTU.tsv	Root-Host_associated-Human-Skin
SRR5869890	MGYA00416950	https://www.ebi.ac.uk/metagenomics/api/v1/analyses/MGYA00416950/file/SRR5869890_MERGED_FASTQ_SSU_OTU.tsv	Root-Host_associated-Human-Skin
SRR1923867	MGYA00028324	https://www.ebi.ac.uk/metagenomics/api/v1/analyses/MGYA00028324/file/SRR1923867_FASTQ_otu.tsv	Root-Host_associated-Human-Skin
ERR443680	MGYA00425737	https://www.ebi.ac.uk/metagenomics/api/v1/analyses/MGYA00425737/file/ERR443680_FASTQ_SSU_OTU.tsv	Root-Host_associated-Human-Skin
SRR1623185	MGYA00023679	https://www.ebi.ac.uk/metagenomics/api/v1/analyses/MGYA00023679/file/SRR1623185_MERGED_FASTQ_otu.tsv	Root-Host_associated-Human-Skin

SRR1631382	MGYA00024089	https://www.ebi.ac.uk/metagenomics/api/v1/analyses/MGYA00024089/file/SRR1631382_MERGED_FASTQ_otu.tsv	Root-Host_associated-Human-Skin
ERR1960726	MGYA00130858	https://www.ebi.ac.uk/metagenomics/api/v1/analyses/MGYA00130858/file/ERR1960726_MERGED_FASTQ_otu.tsv	Root-Host_associated-Human-Skin
ERR1701285	MGYA00084291	https://www.ebi.ac.uk/metagenomics/api/v1/analyses/MGYA00084291/file/ERR1701285_FASTQ_otu.tsv	Root-Host_associated-Human-Skin
ERR451565	MGYA00419450	https://www.ebi.ac.uk/metagenomics/api/v1/analyses/MGYA00419450/file/ERR451565_FASTQ_SSU_OTU.tsv	Root-Host_associated-Human-Skin
SRR1622871	MGYA00024182	https://www.ebi.ac.uk/metagenomics/api/v1/analyses/MGYA00024182/file/SRR1622871_MERGED_FASTQ_otu.tsv	Root-Host_associated-Human-Skin
SRR1631044	MGYA00025011	https://www.ebi.ac.uk/metagenomics/api/v1/analyses/MGYA00025011/file/SRR1631044_MERGED_FASTQ_otu.tsv	Root-Host_associated-Human-Skin
SRR5868853	MGYA00420740	https://www.ebi.ac.uk/metagenomics/api/v1/analyses/MGYA00420740/file/SRR5868853_MERGED_FASTQ_SSU_OTU.tsv	Root-Host_associated-Human-Skin
SRR6757652	MGYA00380767	https://www.ebi.ac.uk/metagenomics/api/v1/analyses/MGYA00380767/file/SRR6757652_MERGED_FASTQ_SSU_OTU.tsv	Root-Host_associated-Human-Skin
SRR997059	MGYA00022974	https://www.ebi.ac.uk/metagenomics/api/v1/analyses/MGYA00022974/file/SRR997059_FASTQ_otu.tsv	Root-Host_associated-Human-Skin
SRR6757683	MGYA00380796	https://www.ebi.ac.uk/metagenomics/api/v1/analyses/MGYA00380796/file/SRR6757683_MERGED_FASTQ_SSU_OTU.tsv	Root-Host_associated-Human-Skin
ERR2538473	MGYA00381480	https://www.ebi.ac.uk/metagenomics/api/v1/analyses/MGYA00381480/file/ERR2538473_FASTQ_SSU_OTU.tsv	Root-Host_associated-Human-Skin
SRR921121	MGYA00025183	https://www.ebi.ac.uk/metagenomics/api/v1/analyses/MGYA00025183/file/SRR921121_FASTQ_otu.tsv	Root-Host_associated-Human-Skin

ERR450821	MGYA00426829	https://www.ebi.ac.uk/metagenomics/api/v1/analyses/MGYA00426829/file/ERR450821_FASTQ_SSU_OTU.tsv	Root-Host_associated-Human-Skin
ERR1701254	MGYA00084262	https://www.ebi.ac.uk/metagenomics/api/v1/analyses/MGYA00084262/file/ERR1701254_FASTQ_otu.tsv	Root-Host_associated-Human-Skin
SRR5868736	MGYA00417578	https://www.ebi.ac.uk/metagenomics/api/v1/analyses/MGYA00417578/file/SRR5868736_MERGED_FASTQ_SSU_OTU.tsv	Root-Host_associated-Human-Skin
SRR1632993	MGYA00022268	https://www.ebi.ac.uk/metagenomics/api/v1/analyses/MGYA00022268/file/SRR1632993_MERGED_FASTQ_otu.tsv	Root-Host_associated-Human-Skin
SRR921079	MGYA00022636	https://www.ebi.ac.uk/metagenomics/api/v1/analyses/MGYA00022636/file/SRR921079_FASTQ_otu.tsv	Root-Host_associated-Human-Skin
SRR920639	MGYA00022953	https://www.ebi.ac.uk/metagenomics/api/v1/analyses/MGYA00022953/file/SRR920639_FASTQ_otu.tsv	Root-Host_associated-Human-Skin
SRR996741	MGYA00023614	https://www.ebi.ac.uk/metagenomics/api/v1/analyses/MGYA00023614/file/SRR996741_FASTQ_otu.tsv	Root-Host_associated-Human-Skin
SRR994807	MGYA00023025	https://www.ebi.ac.uk/metagenomics/api/v1/analyses/MGYA00023025/file/SRR994807_FASTQ_otu.tsv	Root-Host_associated-Human-Skin
SRR994475	MGYA00022246	https://www.ebi.ac.uk/metagenomics/api/v1/analyses/MGYA00022246/file/SRR994475_FASTQ_otu.tsv	Root-Host_associated-Human-Skin
ERR442041	MGYA00385203	https://www.ebi.ac.uk/metagenomics/api/v1/analyses/MGYA00385203/file/ERR442041_FASTQ_SSU_OTU.tsv	Root-Host_associated-Human-Skin
SRR995592	MGYA00025208	https://www.ebi.ac.uk/metagenomics/api/v1/analyses/MGYA00025208/file/SRR995592_FASTQ_otu.tsv	Root-Host_associated-Human-Skin
SRR996320	MGYA00022945	https://www.ebi.ac.uk/metagenomics/api/v1/analyses/MGYA00022945/file/SRR996320_FASTQ_otu.tsv	Root-Host_associated-Human-Skin

SRR995543	MGYA00022741	https://www.ebi.ac.uk/metagenomics/api/v1/analyses/MGYA00022741/file/SRR995543_FASTQ_otu.tsv	Root-Host_associated-Human-Skin
SRR1622929	MGYA00022401	https://www.ebi.ac.uk/metagenomics/api/v1/analyses/MGYA00022401/file/SRR1622929_MERGED_FASTQ_otu.tsv	Root-Host_associated-Human-Skin
ERR441996	MGYA00385210	https://www.ebi.ac.uk/metagenomics/api/v1/analyses/MGYA00385210/file/ERR441996_FASTQ_SSU_OTU.tsv	Root-Host_associated-Human-Skin
SRR919634	MGYA00023767	https://www.ebi.ac.uk/metagenomics/api/v1/analyses/MGYA00023767/file/SRR919634_FASTQ_otu.tsv	Root-Host_associated-Human-Skin
SRR995122	MGYA00023919	https://www.ebi.ac.uk/metagenomics/api/v1/analyses/MGYA00023919/file/SRR995122_FASTQ_otu.tsv	Root-Host_associated-Human-Skin
ERR1760132	MGYA00140482	https://www.ebi.ac.uk/metagenomics/api/v1/analyses/MGYA00140482/file/ERR1760132_MERGED_FASTQ_SSU_OTU.tsv	Root-Host_associated-Human-Skin
SRR1923815	MGYA00028402	https://www.ebi.ac.uk/metagenomics/api/v1/analyses/MGYA00028402/file/SRR1923815_FASTQ_otu.tsv	Root-Host_associated-Human-Skin
SRR2005657	MGYA00381315	https://www.ebi.ac.uk/metagenomics/api/v1/analyses/MGYA00381315/file/SRR2005657_MERGED_FASTQ_SSU_OTU.tsv	Root-Host_associated-Human-Skin
SRR1920900	MGYA00028251	https://www.ebi.ac.uk/metagenomics/api/v1/analyses/MGYA00028251/file/SRR1920900_FASTQ_otu.tsv	Root-Host_associated-Human-Skin
SRR5869672	MGYA00419192	https://www.ebi.ac.uk/metagenomics/api/v1/analyses/MGYA00419192/file/SRR5869672_MERGED_FASTQ_SSU_OTU.tsv	Root-Host_associated-Human-Skin
SRR5868795	MGYA00420769	https://www.ebi.ac.uk/metagenomics/api/v1/analyses/MGYA00420769/file/SRR5868795_MERGED_FASTQ_SSU_OTU.tsv	Root-Host_associated-Human-Skin
SRR1631457	MGYA00025122	https://www.ebi.ac.uk/metagenomics/api/v1/analyses/MGYA00025122/file/SRR1631457_MERGED_FASTQ_otu.tsv	Root-Host_associated-Human-Skin

SRR995008	MGYA00024153	https://www.ebi.ac.uk/metagenomics/api/v1/analyses/MGYA00024153/file/SRR995008_FASTQ_otu.tsv	Root-Host_associated-Human-Skin
ERR1760018	MGYA00140469	https://www.ebi.ac.uk/metagenomics/api/v1/analyses/MGYA00140469/file/ERR1760018_MERGED_FASTQ_SSU_OTU.tsv	Root-Host_associated-Human-Skin
ERR1960754	MGYA00130904	https://www.ebi.ac.uk/metagenomics/api/v1/analyses/MGYA00130904/file/ERR1960754_MERGED_FASTQ_otu.tsv	Root-Host_associated-Human-Skin
SRR1623231	MGYA00024122	https://www.ebi.ac.uk/metagenomics/api/v1/analyses/MGYA00024122/file/SRR1623231_MERGED_FASTQ_otu.tsv	Root-Host_associated-Human-Skin
SRR5868821	MGYA00427150	https://www.ebi.ac.uk/metagenomics/api/v1/analyses/MGYA00427150/file/SRR5868821_MERGED_FASTQ_SSU_OTU.tsv	Root-Host_associated-Human-Skin
SRR6757620	MGYA00380737	https://www.ebi.ac.uk/metagenomics/api/v1/analyses/MGYA00380737/file/SRR6757620_MERGED_FASTQ_SSU_OTU.tsv	Root-Host_associated-Human-Skin
ERR2538377	MGYA00381384	https://www.ebi.ac.uk/metagenomics/api/v1/analyses/MGYA00381384/file/ERR2538377_FASTQ_SSU_OTU.tsv	Root-Host_associated-Human-Skin
SRR5869917	MGYA00413457	https://www.ebi.ac.uk/metagenomics/api/v1/analyses/MGYA00413457/file/SRR5869917_MERGED_FASTQ_SSU_OTU.tsv	Root-Host_associated-Human-Skin
ERR2538401	MGYA00381408	https://www.ebi.ac.uk/metagenomics/api/v1/analyses/MGYA00381408/file/ERR2538401_FASTQ_SSU_OTU.tsv	Root-Host_associated-Human-Skin
SRR994699	MGYA00023882	https://www.ebi.ac.uk/metagenomics/api/v1/analyses/MGYA00023882/file/SRR994699_FASTQ_otu.tsv	Root-Host_associated-Human-Skin
ERR1701226	MGYA00084272	https://www.ebi.ac.uk/metagenomics/api/v1/analyses/MGYA00084272/file/ERR1701226_FASTQ_otu.tsv	Root-Host_associated-Human-Skin
ERR1960785	MGYA00130746	https://www.ebi.ac.uk/metagenomics/api/v1/analyses/MGYA00130746/file/ERR1960785_MERGED_FASTQ_otu.tsv	Root-Host_associated-Human-Skin

SRR1923770	MGYA00028312	https://www.ebi.ac.uk/metagenomics/api/v1/analyses/MGYA00028312/file/SRR1923770_FASTQ_otu.tsv	Root-Host_associated-Human-Skin
SRR1633015	MGYA00023957	https://www.ebi.ac.uk/metagenomics/api/v1/analyses/MGYA00023957/file/SRR1633015_MERGED_FASTQ_otu.tsv	Root-Host_associated-Human-Skin
ERR451261	MGYA00426055	https://www.ebi.ac.uk/metagenomics/api/v1/analyses/MGYA00426055/file/ERR451261_FASTQ_SSU_OTU.tsv	Root-Host_associated-Human-Skin
SRR1631321	MGYA00023020	https://www.ebi.ac.uk/metagenomics/api/v1/analyses/MGYA00023020/file/SRR1631321_MERGED_FASTQ_otu.tsv	Root-Host_associated-Human-Skin
SRR5869104	MGYA00424490	https://www.ebi.ac.uk/metagenomics/api/v1/analyses/MGYA00424490/file/SRR5869104_MERGED_FASTQ_SSU_OTU.tsv	Root-Host_associated-Human-Skin
ERR443184	MGYA00420478	https://www.ebi.ac.uk/metagenomics/api/v1/analyses/MGYA00420478/file/ERR443184_FASTQ_SSU_OTU.tsv	Root-Host_associated-Human-Skin
ERR1960831	MGYA00130814	https://www.ebi.ac.uk/metagenomics/api/v1/analyses/MGYA00130814/file/ERR1960831_MERGED_FASTQ_otu.tsv	Root-Host_associated-Human-Skin
SRR1922551	MGYA00028244	https://www.ebi.ac.uk/metagenomics/api/v1/analyses/MGYA00028244/file/SRR1922551_FASTQ_otu.tsv	Root-Host_associated-Human-Skin
SRR995626	MGYA00025224	https://www.ebi.ac.uk/metagenomics/api/v1/analyses/MGYA00025224/file/SRR995626_FASTQ_otu.tsv	Root-Host_associated-Human-Skin
SRR1624866	MGYA00024466	https://www.ebi.ac.uk/metagenomics/api/v1/analyses/MGYA00024466/file/SRR1624866_MERGED_FASTQ_otu.tsv	Root-Host_associated-Human-Skin
ERR442033	MGYA00385242	https://www.ebi.ac.uk/metagenomics/api/v1/analyses/MGYA00385242/file/ERR442033_FASTQ_SSU_OTU.tsv	Root-Host_associated-Human-Skin
SRR994875	MGYA00025268	https://www.ebi.ac.uk/metagenomics/api/v1/analyses/MGYA00025268/file/SRR994875_FASTQ_otu.tsv	Root-Host_associated-Human-Skin

SRR994407	MGYA00025141	https://www.ebi.ac.uk/metagenomics/api/v1/analyses/MGYA00025141/file/SRR994407_FASTQ_otu.tsv	Root-Host_associated-Human-Skin
SRR1631279	MGYA00024738	https://www.ebi.ac.uk/metagenomics/api/v1/analyses/MGYA00024738/file/SRR1631279_MERGED_FASTQ_otu.tsv	Root-Host_associated-Human-Skin
SRR1950708	MGYA00376875	https://www.ebi.ac.uk/metagenomics/api/v1/analyses/MGYA00376875/file/SRR1950708_MERGED_FASTQ_SSU_OTU.tsv	Root-Host_associated-Human-Skin
SRR919551	MGYA00024202	https://www.ebi.ac.uk/metagenomics/api/v1/analyses/MGYA00024202/file/SRR919551_FASTQ_otu.tsv	Root-Host_associated-Human-Skin
SRR919923	MGYA00025200	https://www.ebi.ac.uk/metagenomics/api/v1/analyses/MGYA00025200/file/SRR919923_FASTQ_otu.tsv	Root-Host_associated-Human-Skin
SRR996856	MGYA00023222	https://www.ebi.ac.uk/metagenomics/api/v1/analyses/MGYA00023222/file/SRR996856_FASTQ_otu.tsv	Root-Host_associated-Human-Skin
SRR995943	MGYA00024284	https://www.ebi.ac.uk/metagenomics/api/v1/analyses/MGYA00024284/file/SRR995943_FASTQ_otu.tsv	Root-Host_associated-Human-Skin
SRR995296	MGYA00023596	https://www.ebi.ac.uk/metagenomics/api/v1/analyses/MGYA00023596/file/SRR995296_FASTQ_otu.tsv	Root-Host_associated-Human-Skin
ERR1760140	MGYA00140389	https://www.ebi.ac.uk/metagenomics/api/v1/analyses/MGYA00140389/file/ERR1760140_MERGED_FASTQ_SSU_OTU.tsv	Root-Host_associated-Human-Skin
SRR994710	MGYA00023648	https://www.ebi.ac.uk/metagenomics/api/v1/analyses/MGYA00023648/file/SRR994710_FASTQ_otu.tsv	Root-Host_associated-Human-Skin
SRR996972	MGYA00022644	https://www.ebi.ac.uk/metagenomics/api/v1/analyses/MGYA00022644/file/SRR996972_FASTQ_otu.tsv	Root-Host_associated-Human-Skin
ERR443129	MGYA00426780	https://www.ebi.ac.uk/metagenomics/api/v1/analyses/MGYA00426780/file/ERR443129_FASTQ_SSU_OTU.tsv	Root-Host_associated-Human-Skin

SRR1923869	MGYA00028221	https://www.ebi.ac.uk/metagenomics/api/v1/analyses/MGYA00028221/file/SRR1923869_FASTQ_otu.tsv	Root-Host_associated-Human-Skin
SRR995415	MGYA00024929	https://www.ebi.ac.uk/metagenomics/api/v1/analyses/MGYA00024929/file/SRR995415_FASTQ_otu.tsv	Root-Host_associated-Human-Skin
SRR1704729	MGYA00428439	https://www.ebi.ac.uk/metagenomics/api/v1/analyses/MGYA00428439/file/SRR1704729_FASTQ_SSU_OTU.tsv	Root-Host_associated-Human-Skin
SRR996161	MGYA00023198	https://www.ebi.ac.uk/metagenomics/api/v1/analyses/MGYA00023198/file/SRR996161_FASTQ_otu.tsv	Root-Host_associated-Human-Skin
ERR1760064	MGYA00140522	https://www.ebi.ac.uk/metagenomics/api/v1/analyses/MGYA00140522/file/ERR1760064_MERGED_FASTQ_SSU_OTU.tsv	Root-Host_associated-Human-Skin
ERR1960728	MGYA00130851	https://www.ebi.ac.uk/metagenomics/api/v1/analyses/MGYA00130851/file/ERR1960728_MERGED_FASTQ_otu.tsv	Root-Host_associated-Human-Skin
SRR997057	MGYA00022969	https://www.ebi.ac.uk/metagenomics/api/v1/analyses/MGYA00022969/file/SRR997057_FASTQ_otu.tsv	Root-Host_associated-Human-Skin
SRR995363	MGYA00022918	https://www.ebi.ac.uk/metagenomics/api/v1/analyses/MGYA00022918/file/SRR995363_FASTQ_otu.tsv	Root-Host_associated-Human-Skin
SRR994523	MGYA00024049	https://www.ebi.ac.uk/metagenomics/api/v1/analyses/MGYA00024049/file/SRR994523_FASTQ_otu.tsv	Root-Host_associated-Human-Skin
SRR994951	MGYA00024338	https://www.ebi.ac.uk/metagenomics/api/v1/analyses/MGYA00024338/file/SRR994951_FASTQ_otu.tsv	Root-Host_associated-Human-Skin
SRR1704399	MGYA00414647	https://www.ebi.ac.uk/metagenomics/api/v1/analyses/MGYA00414647/file/SRR1704399_FASTQ_SSU_OTU.tsv	Root-Host_associated-Human-Skin
SRR5869178	MGYA00429085	https://www.ebi.ac.uk/metagenomics/api/v1/analyses/MGYA00429085/file/SRR5869178_MERGED_FASTQ_SSU_OTU.tsv	Root-Host_associated-Human-Skin

ERR2538525	MGYA00381532	https://www.ebi.ac.uk/metagenomics/api/v1/analyses/MGYA00381532/file/ERR2538525_FASTQ_SSU_OTU.tsv	Root-Host_associated-Human-Skin
SRR5869441	MGYA00425429	https://www.ebi.ac.uk/metagenomics/api/v1/analyses/MGYA00425429/file/SRR5869441_MERGED_FASTQ_SSU_OTU.tsv	Root-Host_associated-Human-Skin
SRR5869833	MGYA00421173	https://www.ebi.ac.uk/metagenomics/api/v1/analyses/MGYA00421173/file/SRR5869833_MERGED_FASTQ_SSU_OTU.tsv	Root-Host_associated-Human-Skin
SRR1631664	MGYA00023150	https://www.ebi.ac.uk/metagenomics/api/v1/analyses/MGYA00023150/file/SRR1631664_MERGED_FASTQ_otu.tsv	Root-Host_associated-Human-Skin
SRR920190	MGYA00023263	https://www.ebi.ac.uk/metagenomics/api/v1/analyses/MGYA00023263/file/SRR920190_FASTQ_otu.tsv	Root-Host_associated-Human-Skin
ERR451345	MGYA00424314	https://www.ebi.ac.uk/metagenomics/api/v1/analyses/MGYA00424314/file/ERR451345_FASTQ_SSU_OTU.tsv	Root-Host_associated-Human-Skin
SRR921077	MGYA00022634	https://www.ebi.ac.uk/metagenomics/api/v1/analyses/MGYA00022634/file/SRR921077_FASTQ_otu.tsv	Root-Host_associated-Human-Skin
ERR442954	MGYA00423319	https://www.ebi.ac.uk/metagenomics/api/v1/analyses/MGYA00423319/file/ERR442954_FASTQ_SSU_OTU.tsv	Root-Host_associated-Human-Skin
ERR1759922	MGYA00140467	https://www.ebi.ac.uk/metagenomics/api/v1/analyses/MGYA00140467/file/ERR1759922_MERGED_FASTQ_SSU_OTU.tsv	Root-Host_associated-Human-Skin
SRR5868660	MGYA00417144	https://www.ebi.ac.uk/metagenomics/api/v1/analyses/MGYA00417144/file/SRR5868660_MERGED_FASTQ_SSU_OTU.tsv	Root-Host_associated-Human-Skin
SRR1950774	MGYA00376941	https://www.ebi.ac.uk/metagenomics/api/v1/analyses/MGYA00376941/file/SRR1950774_MERGED_FASTQ_SSU_OTU.tsv	Root-Host_associated-Human-Skin
SRR994809	MGYA00023033	https://www.ebi.ac.uk/metagenomics/api/v1/analyses/MGYA00023033/file/SRR994809_FASTQ_otu.tsv	Root-Host_associated-Human-Skin

SRR1632814	MGYA00022596	https://www.ebi.ac.uk/metagenomics/api/v1/analyses/MGYA00022596/file/SRR1632814_MERGED_FASTQ_otu.tsv	Root-Host_associated-Human-Skin
SRR1632466	MGYA00023138	https://www.ebi.ac.uk/metagenomics/api/v1/analyses/MGYA00023138/file/SRR1632466_MERGED_FASTQ_otu.tsv	Root-Host_associated-Human-Skin
SRR920952	MGYA00022789	https://www.ebi.ac.uk/metagenomics/api/v1/analyses/MGYA00022789/file/SRR920952_FASTQ_otu.tsv	Root-Host_associated-Human-Skin
ERR451052	MGYA00415173	https://www.ebi.ac.uk/metagenomics/api/v1/analyses/MGYA00415173/file/ERR451052_FASTQ_SSU_OTU.tsv	Root-Host_associated-Human-Skin
SRR1704210	MGYA00426151	https://www.ebi.ac.uk/metagenomics/api/v1/analyses/MGYA00426151/file/SRR1704210_FASTQ_SSU_OTU.tsv	Root-Host_associated-Human-Skin
ERR442985	MGYA00416700	https://www.ebi.ac.uk/metagenomics/api/v1/analyses/MGYA00416700/file/ERR442985_FASTQ_SSU_OTU.tsv	Root-Host_associated-Human-Skin
ERR451394	MGYA00416265	https://www.ebi.ac.uk/metagenomics/api/v1/analyses/MGYA00416265/file/ERR451394_FASTQ_SSU_OTU.tsv	Root-Host_associated-Human-Skin
ERR451433	MGYA00416255	https://www.ebi.ac.uk/metagenomics/api/v1/analyses/MGYA00416255/file/ERR451433_FASTQ_SSU_OTU.tsv	Root-Host_associated-Human-Skin
ERR1960670	MGYA00130722	https://www.ebi.ac.uk/metagenomics/api/v1/analyses/MGYA00130722/file/ERR1960670_MERGED_FASTQ_otu.tsv	Root-Host_associated-Human-Skin
ERR443410	MGYA00413674	https://www.ebi.ac.uk/metagenomics/api/v1/analyses/MGYA00413674/file/ERR443410_FASTQ_SSU_OTU.tsv	Root-Host_associated-Human-Skin
SRR1631112	MGYA00022866	https://www.ebi.ac.uk/metagenomics/api/v1/analyses/MGYA00022866/file/SRR1631112_MERGED_FASTQ_otu.tsv	Root-Host_associated-Human-Skin
SRR1622927	MGYA00023791	https://www.ebi.ac.uk/metagenomics/api/v1/analyses/MGYA00023791/file/SRR1622927_MERGED_FASTQ_otu.tsv	Root-Host_associated-Human-Skin

ERR441998	MGYA00385248	https://www.ebi.ac.uk/metagenomics/api/v1/analyses/MGYA00385248/file/ERR441998_FASTQ_SSU_OTU.tsv	Root-Host_associated-Human-Skin
SRR996975	MGYA00022643	https://www.ebi.ac.uk/metagenomics/api/v1/analyses/MGYA00022643/file/SRR996975_FASTQ_otu.tsv	Root-Host_associated-Human-Skin
SRR995412	MGYA00024926	https://www.ebi.ac.uk/metagenomics/api/v1/analyses/MGYA00024926/file/SRR995412_FASTQ_otu.tsv	Root-Host_associated-Human-Skin
SRR1622878	MGYA00024187	https://www.ebi.ac.uk/metagenomics/api/v1/analyses/MGYA00024187/file/SRR1622878_MERGED_FASTQ_otu.tsv	Root-Host_associated-Human-Skin
SRR996166	MGYA00024398	https://www.ebi.ac.uk/metagenomics/api/v1/analyses/MGYA00024398/file/SRR996166_FASTQ_otu.tsv	Root-Host_associated-Human-Skin
SRR5870003	MGYA00416428	https://www.ebi.ac.uk/metagenomics/api/v1/analyses/MGYA00416428/file/SRR5870003_MERGED_FASTQ_SSU_OTU.tsv	Root-Host_associated-Human-Skin
SRR994633	MGYA00022517	https://www.ebi.ac.uk/metagenomics/api/v1/analyses/MGYA00022517/file/SRR994633_FASTQ_otu.tsv	Root-Host_associated-Human-Skin
ERR1760063	MGYA00140543	https://www.ebi.ac.uk/metagenomics/api/v1/analyses/MGYA00140543/file/ERR1760063_MERGED_FASTQ_SSU_OTU.tsv	Root-Host_associated-Human-Skin
SRR995073	MGYA00024641	https://www.ebi.ac.uk/metagenomics/api/v1/analyses/MGYA00024641/file/SRR995073_FASTQ_otu.tsv	Root-Host_associated-Human-Skin
SRR921128	MGYA00025190	https://www.ebi.ac.uk/metagenomics/api/v1/analyses/MGYA00025190/file/SRR921128_FASTQ_otu.tsv	Root-Host_associated-Human-Skin
ERR450828	MGYA00413718	https://www.ebi.ac.uk/metagenomics/api/v1/analyses/MGYA00413718/file/ERR450828_FASTQ_SSU_OTU.tsv	Root-Host_associated-Human-Skin
SRR997050	MGYA00022966	https://www.ebi.ac.uk/metagenomics/api/v1/analyses/MGYA00022966/file/SRR997050_FASTQ_otu.tsv	Root-Host_associated-Human-Skin

SRR994956	MGYA00024339	https://www.ebi.ac.uk/metagenomics/api/v1/analyses/MGYA00024339/file/SRR994956_FASTQ_otu.tsv	Root-Host_associated-Human-Skin
SRR994524	MGYA00024048	https://www.ebi.ac.uk/metagenomics/api/v1/analyses/MGYA00024048/file/SRR994524_FASTQ_otu.tsv	Root-Host_associated-Human-Skin
SRR995364	MGYA00022915	https://www.ebi.ac.uk/metagenomics/api/v1/analyses/MGYA00022915/file/SRR995364_FASTQ_otu.tsv	Root-Host_associated-Human-Skin
SRR1631663	MGYA00023149	https://www.ebi.ac.uk/metagenomics/api/v1/analyses/MGYA00023149/file/SRR1631663_MERGED_FASTQ_otu.tsv	Root-Host_associated-Human-Skin
ERR2538522	MGYA00381529	https://www.ebi.ac.uk/metagenomics/api/v1/analyses/MGYA00381529/file/ERR2538522_FASTQ_SSU_OTU.tsv	Root-Host_associated-Human-Skin
SRR5869446	MGYA00421640	https://www.ebi.ac.uk/metagenomics/api/v1/analyses/MGYA00421640/file/SRR5869446_MERGED_FASTQ_SSU_OTU.tsv	Root-Host_associated-Human-Skin
SRR996748	MGYA00023619	https://www.ebi.ac.uk/metagenomics/api/v1/analyses/MGYA00023619/file/SRR996748_FASTQ_otu.tsv	Root-Host_associated-Human-Skin
SRR1633136	MGYA00024875	https://www.ebi.ac.uk/metagenomics/api/v1/analyses/MGYA00024875/file/SRR1633136_MERGED_FASTQ_otu.tsv	Root-Host_associated-Human-Skin
SRR921070	MGYA00022629	https://www.ebi.ac.uk/metagenomics/api/v1/analyses/MGYA00022629/file/SRR921070_FASTQ_otu.tsv	Root-Host_associated-Human-Skin
ERR451342	MGYA00426027	https://www.ebi.ac.uk/metagenomics/api/v1/analyses/MGYA00426027/file/ERR451342_FASTQ_SSU_OTU.tsv	Root-Host_associated-Human-Skin
SRR920197	MGYA00023268	https://www.ebi.ac.uk/metagenomics/api/v1/analyses/MGYA00023268/file/SRR920197_FASTQ_otu.tsv	Root-Host_associated-Human-Skin
SRR1950773	MGYA00376940	https://www.ebi.ac.uk/metagenomics/api/v1/analyses/MGYA00376940/file/SRR1950773_MERGED_FASTQ_SSU_OTU.tsv	Root-Host_associated-Human-Skin

SRR996329	MGYA00022950	https://www.ebi.ac.uk/metagenomics/api/v1/analyses/MGYA00022950/file/SRR996329_FASTQ_otu.tsv	Root-Host_associated-Human-Skin
SRR1704561	MGYA00423378	https://www.ebi.ac.uk/metagenomics/api/v1/analyses/MGYA00423378/file/SRR1704561_FASTQ_SSU_OTU.tsv	Root-Host_associated-Human-Skin
SRR5868667	MGYA00421219	https://www.ebi.ac.uk/metagenomics/api/v1/analyses/MGYA00421219/file/SRR5868667_MERGED_FASTQ_SSU_OTU.tsv	Root-Host_associated-Human-Skin
ERR1759925	MGYA00140456	https://www.ebi.ac.uk/metagenomics/api/v1/analyses/MGYA00140456/file/ERR1759925_MERGED_FASTQ_SSU_OTU.tsv	Root-Host_associated-Human-Skin
ERR451084	MGYA00426520	https://www.ebi.ac.uk/metagenomics/api/v1/analyses/MGYA00426520/file/ERR451084_FASTQ_SSU_OTU.tsv	Root-Host_associated-Human-Skin
ERR442048	MGYA00385214	https://www.ebi.ac.uk/metagenomics/api/v1/analyses/MGYA00385214/file/ERR442048_FASTQ_SSU_OTU.tsv	Root-Host_associated-Human-Skin
SRR1923936	MGYA00028259	https://www.ebi.ac.uk/metagenomics/api/v1/analyses/MGYA00028259/file/SRR1923936_FASTQ_otu.tsv	Root-Host_associated-Human-Skin
SRR920955	MGYA00022782	https://www.ebi.ac.uk/metagenomics/api/v1/analyses/MGYA00022782/file/SRR920955_FASTQ_otu.tsv	Root-Host_associated-Human-Skin
SRR1632813	MGYA00022591	https://www.ebi.ac.uk/metagenomics/api/v1/analyses/MGYA00022591/file/SRR1632813_MERGED_FASTQ_otu.tsv	Root-Host_associated-Human-Skin
SRR1704217	MGYA00427520	https://www.ebi.ac.uk/metagenomics/api/v1/analyses/MGYA00427520/file/SRR1704217_FASTQ_SSU_OTU.tsv	Root-Host_associated-Human-Skin
ERR443417	MGYA00428402	https://www.ebi.ac.uk/metagenomics/api/v1/analyses/MGYA00428402/file/ERR443417_FASTQ_SSU_OTU.tsv	Root-Host_associated-Human-Skin
ERR1960677	MGYA00130718	https://www.ebi.ac.uk/metagenomics/api/v1/analyses/MGYA00130718/file/ERR1960677_MERGED_FASTQ_otu.tsv	Root-Host_associated-Human-Skin

ERR451393	MGYA00416660	https://www.ebi.ac.uk/metagenomics/api/v1/analyses/MGYA00416660/file/ERR451393_FASTQ_SSU_OTU.tsv	Root-Host_associated-Human-Skin
SRR1622920	MGYA00022410	https://www.ebi.ac.uk/metagenomics/api/v1/analyses/MGYA00022410/file/SRR1622920_MERGED_FASTQ_otu.tsv	Root-Host_associated-Human-Skin
SRR5869330	MGYA00417563	https://www.ebi.ac.uk/metagenomics/api/v1/analyses/MGYA00417563/file/SRR5869330_MERGED_FASTQ_SSU_OTU.tsv	Root-Host_associated-Human-Skin
SRR1704676	MGYA00417438	https://www.ebi.ac.uk/metagenomics/api/v1/analyses/MGYA00417438/file/SRR1704676_FASTQ_SSU_OTU.tsv	Root-Host_associated-Human-Skin
ERR451171	MGYA00422027	https://www.ebi.ac.uk/metagenomics/api/v1/analyses/MGYA00422027/file/ERR451171_FASTQ_SSU_OTU.tsv	Root-Host_associated-Human-Skin
SRR994589	MGYA00022921	https://www.ebi.ac.uk/metagenomics/api/v1/analyses/MGYA00022921/file/SRR994589_FASTQ_otu.tsv	Root-Host_associated-Human-Skin
SRR1631450	MGYA00025121	https://www.ebi.ac.uk/metagenomics/api/v1/analyses/MGYA00025121/file/SRR1631450_MERGED_FASTQ_otu.tsv	Root-Host_associated-Human-Skin
SRR1704333	MGYA00417848	https://www.ebi.ac.uk/metagenomics/api/v1/analyses/MGYA00417848/file/SRR1704333_FASTQ_SSU_OTU.tsv	Root-Host_associated-Human-Skin
SRR2005650	MGYA00381309	https://www.ebi.ac.uk/metagenomics/api/v1/analyses/MGYA00381309/file/SRR2005650_MERGED_FASTQ_SSU_OTU.tsv	Root-Host_associated-Human-Skin
SRR1950686	MGYA00376853	https://www.ebi.ac.uk/metagenomics/api/v1/analyses/MGYA00376853/file/SRR1950686_MERGED_FASTQ_SSU_OTU.tsv	Root-Host_associated-Human-Skin
ERR1960753	MGYA00130735	https://www.ebi.ac.uk/metagenomics/api/v1/analyses/MGYA00130735/file/ERR1960753_MERGED_FASTQ_otu.tsv	Root-Host_associated-Human-Skin
ERR2538370	MGYA00381377	https://www.ebi.ac.uk/metagenomics/api/v1/analyses/MGYA00381377/file/ERR2538370_FASTQ_SSU_OTU.tsv	Root-Host_associated-Human-Skin

SRR6757627	MGYA00380744	https://www.ebi.ac.uk/metagenomics/api/v1/analyses/MGYA00380744/file/SRR6757627_MERGED_FASTQ_SSU_OTU.tsv	Root-Host_associated-Human-Skin
SRR5868826	MGYA00420287	https://www.ebi.ac.uk/metagenomics/api/v1/analyses/MGYA00420287/file/SRR5868826_MERGED_FASTQ_SSU_OTU.tsv	Root-Host_associated-Human-Skin
SRR1704752	MGYA00417435	https://www.ebi.ac.uk/metagenomics/api/v1/analyses/MGYA00417435/file/SRR1704752_FASTQ_SSU_OTU.tsv	Root-Host_associated-Human-Skin
ERR2538406	MGYA00381413	https://www.ebi.ac.uk/metagenomics/api/v1/analyses/MGYA00381413/file/ERR2538406_FASTQ_SSU_OTU.tsv	Root-Host_associated-Human-Skin
SRR1923777	MGYA00028246	https://www.ebi.ac.uk/metagenomics/api/v1/analyses/MGYA00028246/file/SRR1923777_FASTQ_otu.tsv	Root-Host_associated-Human-Skin
SRR1633012	MGYA00023956	https://www.ebi.ac.uk/metagenomics/api/v1/analyses/MGYA00023956/file/SRR1633012_MERGED_FASTQ_otu.tsv	Root-Host_associated-Human-Skin
ERR443245	MGYA00426081	https://www.ebi.ac.uk/metagenomics/api/v1/analyses/MGYA00426081/file/ERR443245_FASTQ_SSU_OTU.tsv	Root-Host_associated-Human-Skin
ERR1960782	MGYA00130756	https://www.ebi.ac.uk/metagenomics/api/v1/analyses/MGYA00130756/file/ERR1960782_MERGED_FASTQ_otu.tsv	Root-Host_associated-Human-Skin
ERR1701221	MGYA00084314	https://www.ebi.ac.uk/metagenomics/api/v1/analyses/MGYA00084314/file/ERR1701221_FASTQ_otu.tsv	Root-Host_associated-Human-Skin
SRR1631481	MGYA00024933	https://www.ebi.ac.uk/metagenomics/api/v1/analyses/MGYA00024933/file/SRR1631481_MERGED_FASTQ_otu.tsv	Root-Host_associated-Human-Skin
SRR1624861	MGYA00024469	https://www.ebi.ac.uk/metagenomics/api/v1/analyses/MGYA00024469/file/SRR1624861_MERGED_FASTQ_otu.tsv	Root-Host_associated-Human-Skin
SRR995621	MGYA00022724	https://www.ebi.ac.uk/metagenomics/api/v1/analyses/MGYA00022724/file/SRR995621_FASTQ_otu.tsv	Root-Host_associated-Human-Skin

SRR994872	MGYA00025267	https://www.ebi.ac.uk/metagenomics/api/v1/analyses/MGYA00025267/file/SRR994872_FASTQ_otu.tsv	Root-Host_associated-Human-Skin
ERR442034	MGYA00385221	https://www.ebi.ac.uk/metagenomics/api/v1/analyses/MGYA00385221/file/ERR442034_FASTQ_SSU_OTU.tsv	Root-Host_associated-Human-Skin
SRR919924	MGYA00025193	https://www.ebi.ac.uk/metagenomics/api/v1/analyses/MGYA00025193/file/SRR919924_FASTQ_otu.tsv	Root-Host_associated-Human-Skin
SRR919556	MGYA00024201	https://www.ebi.ac.uk/metagenomics/api/v1/analyses/MGYA00024201/file/SRR919556_FASTQ_otu.tsv	Root-Host_associated-Human-Skin
ERR1759959	MGYA00140387	https://www.ebi.ac.uk/metagenomics/api/v1/analyses/MGYA00140387/file/ERR1759959_MERGED_FASTQ_SSU_OTU.tsv	Root-Host_associated-Human-Skin
ERR1759988	MGYA00140396	https://www.ebi.ac.uk/metagenomics/api/v1/analyses/MGYA00140396/file/ERR1759988_MERGED_FASTQ_SSU_OTU.tsv	Root-Host_associated-Human-Skin
SRR996851	MGYA00023215	https://www.ebi.ac.uk/metagenomics/api/v1/analyses/MGYA00023215/file/SRR996851_FASTQ_otu.tsv	Root-Host_associated-Human-Skin
SRR995291	MGYA00023595	https://www.ebi.ac.uk/metagenomics/api/v1/analyses/MGYA00023595/file/SRR995291_FASTQ_otu.tsv	Root-Host_associated-Human-Skin
SRR995944	MGYA00024281	https://www.ebi.ac.uk/metagenomics/api/v1/analyses/MGYA00024281/file/SRR995944_FASTQ_otu.tsv	Root-Host_associated-Human-Skin
SRR2005369	MGYA00381283	https://www.ebi.ac.uk/metagenomics/api/v1/analyses/MGYA00381283/file/SRR2005369_MERGED_FASTQ_SSU_OTU.tsv	Root-Host_associated-Human-Skin
SRR994717	MGYA00023647	https://www.ebi.ac.uk/metagenomics/api/v1/analyses/MGYA00023647/file/SRR994717_FASTQ_otu.tsv	Root-Host_associated-Human-Skin
ERR443819	MGYA00413305	https://www.ebi.ac.uk/metagenomics/api/v1/analyses/MGYA00413305/file/ERR443819_FASTQ_SSU_OTU.tsv	Root-Host_associated-Human-Skin

SRR995929	MGYA00024064	https://www.ebi.ac.uk/metagenomics/api/v1/analyses/MGYA00024064/file/SRR995929_FASTQ_otu.tsv	Root-Host_associated-Human-Skin
SRR1632470	MGYA00022494	https://www.ebi.ac.uk/metagenomics/api/v1/analyses/MGYA00022494/file/SRR1632470_MERGED_FASTQ_otu.tsv	Root-Host_associated-Human-Skin
SRR996788	MGYA00024083	https://www.ebi.ac.uk/metagenomics/api/v1/analyses/MGYA00024083/file/SRR996788_FASTQ_otu.tsv	Root-Host_associated-Human-Skin
SRR1622931	MGYA00025287	https://www.ebi.ac.uk/metagenomics/api/v1/analyses/MGYA00025287/file/SRR1622931_MERGED_FASTQ_otu.tsv	Root-Host_associated-Human-Skin
ERR1960666	MGYA00130839	https://www.ebi.ac.uk/metagenomics/api/v1/analyses/MGYA00130839/file/ERR1960666_MERGED_FASTQ_otu.tsv	Root-Host_associated-Human-Skin
SRR920157	MGYA00024512	https://www.ebi.ac.uk/metagenomics/api/v1/analyses/MGYA00024512/file/SRR920157_FASTQ_otu.tsv	Root-Host_associated-Human-Skin
ERR442993	MGYA00420162	https://www.ebi.ac.uk/metagenomics/api/v1/analyses/MGYA00420162/file/ERR442993_FASTQ_SSU_OTU.tsv	Root-Host_associated-Human-Skin
ERR451382	MGYA00421407	https://www.ebi.ac.uk/metagenomics/api/v1/analyses/MGYA00421407/file/ERR451382_FASTQ_SSU_OTU.tsv	Root-Host_associated-Human-Skin
ERR451425	MGYA00428711	https://www.ebi.ac.uk/metagenomics/api/v1/analyses/MGYA00428711/file/ERR451425_FASTQ_SSU_OTU.tsv	Root-Host_associated-Human-Skin
ERR451353	MGYA00426023	https://www.ebi.ac.uk/metagenomics/api/v1/analyses/MGYA00426023/file/ERR451353_FASTQ_SSU_OTU.tsv	Root-Host_associated-Human-Skin
ERR450961	MGYA00421436	https://www.ebi.ac.uk/metagenomics/api/v1/analyses/MGYA00421436/file/ERR450961_FASTQ_SSU_OTU.tsv	Root-Host_associated-Human-Skin
SRR920621	MGYA00022763	https://www.ebi.ac.uk/metagenomics/api/v1/analyses/MGYA00022763/file/SRR920621_FASTQ_otu.tsv	Root-Host_associated-Human-Skin

SRR997119	MGYA00024566	https://www.ebi.ac.uk/metagenomics/api/v1/analyses/MGYA00024566/file/SRR997119_FASTQ_otu.tsv	Root-Host_associated-Human-Skin
SRR1631672	MGYA00023833	https://www.ebi.ac.uk/metagenomics/api/v1/analyses/MGYA00023833/file/SRR1631672_MERGED_FASTQ_otu.tsv	Root-Host_associated-Human-Skin
SRR5869457	MGYA00418060	https://www.ebi.ac.uk/metagenomics/api/v1/analyses/MGYA00418060/file/SRR5869457_MERGED_FASTQ_SSU_OTU.tsv	Root-Host_associated-Human-Skin
SRR5869825	MGYA00416461	https://www.ebi.ac.uk/metagenomics/api/v1/analyses/MGYA00416461/file/SRR5869825_MERGED_FASTQ_SSU_OTU.tsv	Root-Host_associated-Human-Skin
ERR443711	MGYA00412592	https://www.ebi.ac.uk/metagenomics/api/v1/analyses/MGYA00412592/file/ERR443711_FASTQ_SSU_OTU.tsv	Root-Host_associated-Human-Skin
SRR920240	MGYA00023496	https://www.ebi.ac.uk/metagenomics/api/v1/analyses/MGYA00023496/file/SRR920240_FASTQ_otu.tsv	Root-Host_associated-Human-Skin
SRR1632306	MGYA00023274	https://www.ebi.ac.uk/metagenomics/api/v1/analyses/MGYA00023274/file/SRR1632306_MERGED_FASTQ_otu.tsv	Root-Host_associated-Human-Skin
SRR920995	MGYA00023253	https://www.ebi.ac.uk/metagenomics/api/v1/analyses/MGYA00023253/file/SRR920995_FASTQ_otu.tsv	Root-Host_associated-Human-Skin
ERR442059	MGYA00385188	https://www.ebi.ac.uk/metagenomics/api/v1/analyses/MGYA00385188/file/ERR442059_FASTQ_SSU_OTU.tsv	Root-Host_associated-Human-Skin
SRR1950762	MGYA00376929	https://www.ebi.ac.uk/metagenomics/api/v1/analyses/MGYA00376929/file/SRR1950762_MERGED_FASTQ_SSU_OTU.tsv	Root-Host_associated-Human-Skin
SRR5869036	MGYA00413975	https://www.ebi.ac.uk/metagenomics/api/v1/analyses/MGYA00413975/file/SRR5869036_MERGED_FASTQ_SSU_OTU.tsv	Root-Host_associated-Human-Skin
ERR1759934	MGYA00140481	https://www.ebi.ac.uk/metagenomics/api/v1/analyses/MGYA00140481/file/ERR1759934_MERGED_FASTQ_SSU_OTU.tsv	Root-Host_associated-Human-Skin

SRR1704570	MGYA00426918	https://www.ebi.ac.uk/metagenomics/api/v1/analyses/MGYA00426918/file/SRR1704570_FASTQ_SSU_OTU.tsv	Root-Host_associated-Human-Skin
SRR996601	MGYA00024572	https://www.ebi.ac.uk/metagenomics/api/v1/analyses/MGYA00024572/file/SRR996601_FASTQ_otu.tsv	Root-Host_associated-Human-Skin
SRR995714	MGYA00023360	https://www.ebi.ac.uk/metagenomics/api/v1/analyses/MGYA00023360/file/SRR995714_FASTQ_otu.tsv	Root-Host_associated-Human-Skin
SRR921139	MGYA00023238	https://www.ebi.ac.uk/metagenomics/api/v1/analyses/MGYA00023238/file/SRR921139_FASTQ_otu.tsv	Root-Host_associated-Human-Skin
ERR443228	MGYA00416300	https://www.ebi.ac.uk/metagenomics/api/v1/analyses/MGYA00416300/file/ERR443228_FASTQ_SSU_OTU.tsv	Root-Host_associated-Human-Skin
ERR450839	MGYA00412397	https://www.ebi.ac.uk/metagenomics/api/v1/analyses/MGYA00412397/file/ERR450839_FASTQ_SSU_OTU.tsv	Root-Host_associated-Human-Skin
SRR996260	MGYA00023505	https://www.ebi.ac.uk/metagenomics/api/v1/analyses/MGYA00023505/file/SRR996260_FASTQ_otu.tsv	Root-Host_associated-Human-Skin
SRR1630979	MGYA00024355	https://www.ebi.ac.uk/metagenomics/api/v1/analyses/MGYA00024355/file/SRR1630979_MERGED_FASTQ_otu.tsv	Root-Host_associated-Human-Skin
ERR443649	MGYA00419448	https://www.ebi.ac.uk/metagenomics/api/v1/analyses/MGYA00419448/file/ERR443649_FASTQ_SSU_OTU.tsv	Root-Host_associated-Human-Skin
SRR994947	MGYA00022487	https://www.ebi.ac.uk/metagenomics/api/v1/analyses/MGYA00022487/file/SRR994947_FASTQ_otu.tsv	Root-Host_associated-Human-Skin
SRR994996	MGYA00023927	https://www.ebi.ac.uk/metagenomics/api/v1/analyses/MGYA00023927/file/SRR994996_FASTQ_otu.tsv	Root-Host_associated-Human-Skin
SRR5870012	MGYA00428518	https://www.ebi.ac.uk/metagenomics/api/v1/analyses/MGYA00428518/file/SRR5870012_MERGED_FASTQ_SSU_OTU.tsv	Root-Host_associated-Human-Skin

ERR1760072	MGYA00140442	https://www.ebi.ac.uk/metagenomics/api/v1/analyses/MGYA00140442/file/ERR1760072_MERGED_FASTQ_SSU_OTU.tsv	Root-Host_associated-Human-Skin
SRR995062	MGYA00024058	https://www.ebi.ac.uk/metagenomics/api/v1/analyses/MGYA00024058/file/SRR995062_FASTQ_otu.tsv	Root-Host_associated-Human-Skin
SRR997090	MGYA00022515	https://www.ebi.ac.uk/metagenomics/api/v1/analyses/MGYA00022515/file/SRR997090_FASTQ_otu.tsv	Root-Host_associated-Human-Skin
SRR995527	MGYA00023421	https://www.ebi.ac.uk/metagenomics/api/v1/analyses/MGYA00023421/file/SRR995527_FASTQ_otu.tsv	Root-Host_associated-Human-Skin
SRR920938	MGYA00022571	https://www.ebi.ac.uk/metagenomics/api/v1/analyses/MGYA00022571/file/SRR920938_FASTQ_otu.tsv	Root-Host_associated-Human-Skin
ERR1759999	MGYA00140520	https://www.ebi.ac.uk/metagenomics/api/v1/analyses/MGYA00140520/file/ERR1759999_MERGED_FASTQ_SSU_OTU.tsv	Root-Host_associated-Human-Skin
SRR1631519	MGYA00023378	https://www.ebi.ac.uk/metagenomics/api/v1/analyses/MGYA00023378/file/SRR1631519_MERGED_FASTQ_otu.tsv	Root-Host_associated-Human-Skin
SRR2005719	MGYA00381348	https://www.ebi.ac.uk/metagenomics/api/v1/analyses/MGYA00381348/file/SRR2005719_MERGED_FASTQ_SSU_OTU.tsv	Root-Host_associated-Human-Skin
SRR996432	MGYA00024477	https://www.ebi.ac.uk/metagenomics/api/v1/analyses/MGYA00024477/file/SRR996432_FASTQ_otu.tsv	Root-Host_associated-Human-Skin
SRR919596	MGYA00024955	https://www.ebi.ac.uk/metagenomics/api/v1/analyses/MGYA00024955/file/SRR919596_FASTQ_otu.tsv	Root-Host_associated-Human-Skin
SRR996395	MGYA00024975	https://www.ebi.ac.uk/metagenomics/api/v1/analyses/MGYA00024975/file/SRR996395_FASTQ_otu.tsv	Root-Host_associated-Human-Skin
SRR995146	MGYA00024134	https://www.ebi.ac.uk/metagenomics/api/v1/analyses/MGYA00024134/file/SRR995146_FASTQ_otu.tsv	Root-Host_associated-Human-Skin

SRR2005378	MGYA00381287	https://www.ebi.ac.uk/metagenomics/api/v1/analyses/MGYA00381287/file/SRR2005378_MERGED_FASTQ_SSU_OTU.tsv	Root-Host_associated-Human-Skin
SRR5869888	MGYA00421163	https://www.ebi.ac.uk/metagenomics/api/v1/analyses/MGYA00421163/file/SRR5869888_MERGED_FASTQ_SSU_OTU.tsv	Root-Host_associated-Human-Skin
SRR996082	MGYA00024364	https://www.ebi.ac.uk/metagenomics/api/v1/analyses/MGYA00024364/file/SRR996082_FASTQ_otu.tsv	Root-Host_associated-Human-Skin
SRR996725	MGYA00023428	https://www.ebi.ac.uk/metagenomics/api/v1/analyses/MGYA00023428/file/SRR996725_FASTQ_otu.tsv	Root-Host_associated-Human-Skin
SRR995630	MGYA00022345	https://www.ebi.ac.uk/metagenomics/api/v1/analyses/MGYA00022345/file/SRR995630_FASTQ_otu.tsv	Root-Host_associated-Human-Skin
SRR996891	MGYA00022798	https://www.ebi.ac.uk/metagenomics/api/v1/analyses/MGYA00022798/file/SRR996891_FASTQ_otu.tsv	Root-Host_associated-Human-Skin
SRR996344	MGYA00022281	https://www.ebi.ac.uk/metagenomics/api/v1/analyses/MGYA00022281/file/SRR996344_FASTQ_otu.tsv	Root-Host_associated-Human-Skin
ERR1759948	MGYA00140555	https://www.ebi.ac.uk/metagenomics/api/v1/analyses/MGYA00140555/file/ERR1759948_MERGED_FASTQ_SSU_OTU.tsv	Root-Host_associated-Human-Skin
SRR1623068	MGYA00024420	https://www.ebi.ac.uk/metagenomics/api/v1/analyses/MGYA00024420/file/SRR1623068_MERGED_FASTQ_otu.tsv	Root-Host_associated-Human-Skin
SRR994863	MGYA00022389	https://www.ebi.ac.uk/metagenomics/api/v1/analyses/MGYA00022389/file/SRR994863_FASTQ_otu.tsv	Root-Host_associated-Human-Skin
SRR995984	MGYA00022939	https://www.ebi.ac.uk/metagenomics/api/v1/analyses/MGYA00022939/file/SRR995984_FASTQ_otu.tsv	Root-Host_associated-Human-Skin
ERR442025	MGYA00385244	https://www.ebi.ac.uk/metagenomics/api/v1/analyses/MGYA00385244/file/ERR442025_FASTQ_SSU_OTU.tsv	Root-Host_associated-Human-Skin

ERR451277	MGYA00426048	https://www.ebi.ac.uk/metagenomics/api/v1/analyses/MGYA00426048/file/ERR451277_FASTQ_SSU_OTU.tsv	Root-Host_associated-Human-Skin
SRR921145	MGYA00024589	https://www.ebi.ac.uk/metagenomics/api/v1/analyses/MGYA00024589/file/SRR921145_FASTQ_otu.tsv	Root-Host_associated-Human-Skin
ERR1701230	MGYA00084306	https://www.ebi.ac.uk/metagenomics/api/v1/analyses/MGYA00084306/file/ERR1701230_FASTQ_otu.tsv	Root-Host_associated-Human-Skin
ERR1960793	MGYA00130725	https://www.ebi.ac.uk/metagenomics/api/v1/analyses/MGYA00130725/file/ERR1960793_MERGED_FASTQ_otu.tsv	Root-Host_associated-Human-Skin
ERR450845	MGYA00414562	https://www.ebi.ac.uk/metagenomics/api/v1/analyses/MGYA00414562/file/ERR450845_FASTQ_SSU_OTU.tsv	Root-Host_associated-Human-Skin
ERR2538417	MGYA00381424	https://www.ebi.ac.uk/metagenomics/api/v1/analyses/MGYA00381424/file/ERR2538417_FASTQ_SSU_OTU.tsv	Root-Host_associated-Human-Skin
ERR443192	MGYA00421423	https://www.ebi.ac.uk/metagenomics/api/v1/analyses/MGYA00421423/file/ERR443192_FASTQ_SSU_OTU.tsv	Root-Host_associated-Human-Skin
SRR1922547	MGYA00028410	https://www.ebi.ac.uk/metagenomics/api/v1/analyses/MGYA00028410/file/SRR1922547_FASTQ_otu.tsv	Root-Host_associated-Human-Skin
ERR1960827	MGYA00130835	https://www.ebi.ac.uk/metagenomics/api/v1/analyses/MGYA00130835/file/ERR1960827_MERGED_FASTQ_otu.tsv	Root-Host_associated-Human-Skin
SRR994549	MGYA00024256	https://www.ebi.ac.uk/metagenomics/api/v1/analyses/MGYA00024256/file/SRR994549_FASTQ_otu.tsv	Root-Host_associated-Human-Skin
SRR995309	MGYA00022694	https://www.ebi.ac.uk/metagenomics/api/v1/analyses/MGYA00022694/file/SRR995309_FASTQ_otu.tsv	Root-Host_associated-Human-Skin
SRR1950697	MGYA00376864	https://www.ebi.ac.uk/metagenomics/api/v1/analyses/MGYA00376864/file/SRR1950697_MERGED_FASTQ_SSU_OTU.tsv	Root-Host_associated-Human-Skin

SRR996918	MGYA00022435	https://www.ebi.ac.uk/metagenomics/api/v1/analyses/MGYA00022435/file/SRR996918_FASTQ_otu.tsv	Root-Host_associated-Human-Skin
SRR1622815	MGYA00023985	https://www.ebi.ac.uk/metagenomics/api/v1/analyses/MGYA00023985/file/SRR1622815_MERGED_FASTQ_otu.tsv	Root-Host_associated-Human-Skin
ERR2538361	MGYA00381368	https://www.ebi.ac.uk/metagenomics/api/v1/analyses/MGYA00381368/file/ERR2538361_FASTQ_SSU_OTU.tsv	Root-Host_associated-Human-Skin
SRR5869205	MGYA00419225	https://www.ebi.ac.uk/metagenomics/api/v1/analyses/MGYA00419225/file/SRR5869205_MERGED_FASTQ_SSU_OTU.tsv	Root-Host_associated-Human-Skin
SRR6757636	MGYA00380816	https://www.ebi.ac.uk/metagenomics/api/v1/analyses/MGYA00380816/file/SRR6757636_MERGED_FASTQ_SSU_OTU.tsv	Root-Host_associated-Human-Skin
SRR5868837	MGYA00418548	https://www.ebi.ac.uk/metagenomics/api/v1/analyses/MGYA00418548/file/SRR5868837_MERGED_FASTQ_SSU_OTU.tsv	Root-Host_associated-Human-Skin
SRR1704743	MGYA00415649	https://www.ebi.ac.uk/metagenomics/api/v1/analyses/MGYA00415649/file/SRR1704743_FASTQ_SSU_OTU.tsv	Root-Host_associated-Human-Skin
ERR1960742	MGYA00130846	https://www.ebi.ac.uk/metagenomics/api/v1/analyses/MGYA00130846/file/ERR1960742_MERGED_FASTQ_otu.tsv	Root-Host_associated-Human-Skin
ERR450894	MGYA00413372	https://www.ebi.ac.uk/metagenomics/api/v1/analyses/MGYA00413372/file/ERR450894_FASTQ_SSU_OTU.tsv	Root-Host_associated-Human-Skin
SRR995520	MGYA00023422	https://www.ebi.ac.uk/metagenomics/api/v1/analyses/MGYA00023422/file/SRR995520_FASTQ_otu.tsv	Root-Host_associated-Human-Skin
SRR919591	MGYA00024948	https://www.ebi.ac.uk/metagenomics/api/v1/analyses/MGYA00024948/file/SRR919591_FASTQ_otu.tsv	Root-Host_associated-Human-Skin
SRR996392	MGYA00024976	https://www.ebi.ac.uk/metagenomics/api/v1/analyses/MGYA00024976/file/SRR996392_FASTQ_otu.tsv	Root-Host_associated-Human-Skin

SRR996435	MGYA00024474	https://www.ebi.ac.uk/metagenomics/api/v1/analyses/MGYA00024474/file/SRR996435_FASTQ_otu.tsv	Root-Host_associated-Human-Skin
SRR995141	MGYA00024141	https://www.ebi.ac.uk/metagenomics/api/v1/analyses/MGYA00024141/file/SRR995141_FASTQ_otu.tsv	Root-Host_associated-Human-Skin
SRR5870131	MGYA00420656	https://www.ebi.ac.uk/metagenomics/api/v1/analyses/MGYA00420656/file/SRR5870131_MERGED_FASTQ_SSU_OTU.tsv	Root-Host_associated-Human-Skin
SRR994701	MGYA00024482	https://www.ebi.ac.uk/metagenomics/api/v1/analyses/MGYA00024482/file/SRR994701_FASTQ_otu.tsv	Root-Host_associated-Human-Skin
SRR996722	MGYA00023431	https://www.ebi.ac.uk/metagenomics/api/v1/analyses/MGYA00023431/file/SRR996722_FASTQ_otu.tsv	Root-Host_associated-Human-Skin
SRR996085	MGYA00024363	https://www.ebi.ac.uk/metagenomics/api/v1/analyses/MGYA00024363/file/SRR996085_FASTQ_otu.tsv	Root-Host_associated-Human-Skin
SRR995637	MGYA00022344	https://www.ebi.ac.uk/metagenomics/api/v1/analyses/MGYA00022344/file/SRR995637_FASTQ_otu.tsv	Root-Host_associated-Human-Skin
SRR996343	MGYA00022278	https://www.ebi.ac.uk/metagenomics/api/v1/analyses/MGYA00022278/file/SRR996343_FASTQ_otu.tsv	Root-Host_associated-Human-Skin
SRR1950719	MGYA00376886	https://www.ebi.ac.uk/metagenomics/api/v1/analyses/MGYA00376886/file/SRR1950719_MERGED_FASTQ_SSU_OTU.tsv	Root-Host_associated-Human-Skin
SRR996896	MGYA00022797	https://www.ebi.ac.uk/metagenomics/api/v1/analyses/MGYA00022797/file/SRR996896_FASTQ_otu.tsv	Root-Host_associated-Human-Skin
ERR442022	MGYA00385157	https://www.ebi.ac.uk/metagenomics/api/v1/analyses/MGYA00385157/file/ERR442022_FASTQ_SSU_OTU.tsv	Root-Host_associated-Human-Skin
SRR995983	MGYA00022934	https://www.ebi.ac.uk/metagenomics/api/v1/analyses/MGYA00022934/file/SRR995983_FASTQ_otu.tsv	Root-Host_associated-Human-Skin

SRR994864	MGYA00022394	https://www.ebi.ac.uk/metagenomics/api/v1/analyses/MGYA00022394/file/SRR994864_FASTQ_otu.tsv	Root-Host_associated-Human-Skin
SRR920702	MGYA00024017	https://www.ebi.ac.uk/metagenomics/api/v1/analyses/MGYA00024017/file/SRR920702_FASTQ_otu.tsv	Root-Host_associated-Human-Skin
ERR1960794	MGYA00130748	https://www.ebi.ac.uk/metagenomics/api/v1/analyses/MGYA00130748/file/ERR1960794_MERGED_FASTQ_otu.tsv	Root-Host_associated-Human-Skin
ERR1701237	MGYA00084238	https://www.ebi.ac.uk/metagenomics/api/v1/analyses/MGYA00084238/file/ERR1701237_FASTQ_otu.tsv	Root-Host_associated-Human-Skin
SRR921142	MGYA00024794	https://www.ebi.ac.uk/metagenomics/api/v1/analyses/MGYA00024794/file/SRR921142_FASTQ_otu.tsv	Root-Host_associated-Human-Skin
ERR2538410	MGYA00381417	https://www.ebi.ac.uk/metagenomics/api/v1/analyses/MGYA00381417/file/ERR2538410_FASTQ_SSU_OTU.tsv	Root-Host_associated-Human-Skin
SRR1620022	MGYA00024920	https://www.ebi.ac.uk/metagenomics/api/v1/analyses/MGYA00024920/file/SRR1620022_MERGED_FASTQ_otu.tsv	Root-Host_associated-Human-Skin
ERR1960820	MGYA00130833	https://www.ebi.ac.uk/metagenomics/api/v1/analyses/MGYA00130833/file/ERR1960820_MERGED_FASTQ_otu.tsv	Root-Host_associated-Human-Skin
SRR2005697	MGYA00381334	https://www.ebi.ac.uk/metagenomics/api/v1/analyses/MGYA00381334/file/SRR2005697_MERGED_FASTQ_SSU_OTU.tsv	Root-Host_associated-Human-Skin
SRR2005646	MGYA00381305	https://www.ebi.ac.uk/metagenomics/api/v1/analyses/MGYA00381305/file/SRR2005646_MERGED_FASTQ_SSU_OTU.tsv	Root-Host_associated-Human-Skin
SRR1950690	MGYA00376857	https://www.ebi.ac.uk/metagenomics/api/v1/analyses/MGYA00376857/file/SRR1950690_MERGED_FASTQ_SSU_OTU.tsv	Root-Host_associated-Human-Skin
SRR1704325	MGYA00426972	https://www.ebi.ac.uk/metagenomics/api/v1/analyses/MGYA00426972/file/SRR1704325_FASTQ_SSU_OTU.tsv	Root-Host_associated-Human-Skin

SRR5868784	MGYA00423047	https://www.ebi.ac.uk/metagenomics/api/v1/analyses/MGYA00423047/file/SRR5868784_MERGED_FASTQ_SSU_OTU.tsv	Root-Host_associated-Human-Skin
SRR1923804	MGYA00028382	https://www.ebi.ac.uk/metagenomics/api/v1/analyses/MGYA00028382/file/SRR1923804_FASTQ_otu.tsv	Root-Host_associated-Human-Skin
SRR995478	MGYA00025096	https://www.ebi.ac.uk/metagenomics/api/v1/analyses/MGYA00025096/file/SRR995478_FASTQ_otu.tsv	Root-Host_associated-Human-Skin
SRR6757631	MGYA00380748	https://www.ebi.ac.uk/metagenomics/api/v1/analyses/MGYA00380748/file/SRR6757631_MERGED_FASTQ_SSU_OTU.tsv	Root-Host_associated-Human-Skin
SRR1704744	MGYA00419534	https://www.ebi.ac.uk/metagenomics/api/v1/analyses/MGYA00419534/file/SRR1704744_FASTQ_SSU_OTU.tsv	Root-Host_associated-Human-Skin
ERR2538366	MGYA00381373	https://www.ebi.ac.uk/metagenomics/api/v1/analyses/MGYA00381373/file/ERR2538366_FASTQ_SSU_OTU.tsv	Root-Host_associated-Human-Skin
SRR1622812	MGYA00023980	https://www.ebi.ac.uk/metagenomics/api/v1/analyses/MGYA00023980/file/SRR1622812_MERGED_FASTQ_otu.tsv	Root-Host_associated-Human-Skin
ERR451506	MGYA00415120	https://www.ebi.ac.uk/metagenomics/api/v1/analyses/MGYA00415120/file/ERR451506_FASTQ_SSU_OTU.tsv	Root-Host_associated-Human-Skin
ERR1760009	MGYA00140549	https://www.ebi.ac.uk/metagenomics/api/v1/analyses/MGYA00140549/file/ERR1760009_MERGED_FASTQ_SSU_OTU.tsv	Root-Host_associated-Human-Skin
ERR450893	MGYA00422043	https://www.ebi.ac.uk/metagenomics/api/v1/analyses/MGYA00422043/file/ERR450893_FASTQ_SSU_OTU.tsv	Root-Host_associated-Human-Skin
ERR1960745	MGYA00130820	https://www.ebi.ac.uk/metagenomics/api/v1/analyses/MGYA00130820/file/ERR1960745_MERGED_FASTQ_otu.tsv	Root-Host_associated-Human-Skin
SRR994659	MGYA00023452	https://www.ebi.ac.uk/metagenomics/api/v1/analyses/MGYA00023452/file/SRR994659_FASTQ_otu.tsv	Root-Host_associated-Human-Skin

ERR443525	MGYA00412603	https://www.ebi.ac.uk/metagenomics/api/v1/analyses/MGYA00412603/file/ERR443525_FASTQ_SSU_OTU.tsv	Root-Host_associated-Human-Skin
SRR1920447	MGYA00028275	https://www.ebi.ac.uk/metagenomics/api/v1/analyses/MGYA00028275/file/SRR1920447_FASTQ_otu.tsv	Root-Host_associated-Human-Skin
SRR1632477	MGYA00022492	https://www.ebi.ac.uk/metagenomics/api/v1/analyses/MGYA00022492/file/SRR1632477_MERGED_FASTQ_otu.tsv	Root-Host_associated-Human-Skin
SRR1923920	MGYA00028272	https://www.ebi.ac.uk/metagenomics/api/v1/analyses/MGYA00028272/file/SRR1923920_FASTQ_otu.tsv	Root-Host_associated-Human-Skin
SRR996028	MGYA00023719	https://www.ebi.ac.uk/metagenomics/api/v1/analyses/MGYA00023719/file/SRR996028_FASTQ_otu.tsv	Root-Host_associated-Human-Skin
SRR5869481	MGYA00417541	https://www.ebi.ac.uk/metagenomics/api/v1/analyses/MGYA00417541/file/SRR5869481_MERGED_FASTQ_SSU_OTU.tsv	Root-Host_associated-Human-Skin
SRR1622936	MGYA00025292	https://www.ebi.ac.uk/metagenomics/api/v1/analyses/MGYA00025292/file/SRR1622936_MERGED_FASTQ_otu.tsv	Root-Host_associated-Human-Skin
ERR441989	MGYA00385168	https://www.ebi.ac.uk/metagenomics/api/v1/analyses/MGYA00385168/file/ERR441989_FASTQ_SSU_OTU.tsv	Root-Host_associated-Human-Skin
SRR5869326	MGYA00418746	https://www.ebi.ac.uk/metagenomics/api/v1/analyses/MGYA00418746/file/SRR5869326_MERGED_FASTQ_SSU_OTU.tsv	Root-Host_associated-Human-Skin
SRR920150	MGYA00024515	https://www.ebi.ac.uk/metagenomics/api/v1/analyses/MGYA00024515/file/SRR920150_FASTQ_otu.tsv	Root-Host_associated-Human-Skin
ERR1960661	MGYA00130871	https://www.ebi.ac.uk/metagenomics/api/v1/analyses/MGYA00130871/file/ERR1960661_MERGED_FASTQ_otu.tsv	Root-Host_associated-Human-Skin
ERR443377	MGYA00420128	https://www.ebi.ac.uk/metagenomics/api/v1/analyses/MGYA00420128/file/ERR443377_FASTQ_SSU_OTU.tsv	Root-Host_associated-Human-Skin

SRR920626	MGYA00022766	https://www.ebi.ac.uk/metagenomics/api/v1/analyses/MGYA00022766/file/SRR920626_FASTQ_otu.tsv	Root-Host_associated-Human-Skin
ERR451354	MGYA00426021	https://www.ebi.ac.uk/metagenomics/api/v1/analyses/MGYA00426021/file/ERR451354_FASTQ_SSU_OTU.tsv	Root-Host_associated-Human-Skin
SRR5869450	MGYA00421639	https://www.ebi.ac.uk/metagenomics/api/v1/analyses/MGYA00421639/file/SRR5869450_MERGED_FASTQ_SSU_OTU.tsv	Root-Host_associated-Human-Skin
ERR441958	MGYA00385212	https://www.ebi.ac.uk/metagenomics/api/v1/analyses/MGYA00385212/file/ERR441958_FASTQ_SSU_OTU.tsv	Root-Host_associated-Human-Skin
SRR1631675	MGYA00023830	https://www.ebi.ac.uk/metagenomics/api/v1/analyses/MGYA00023830/file/SRR1631675_MERGED_FASTQ_otu.tsv	Root-Host_associated-Human-Skin
SRR1632301	MGYA00023273	https://www.ebi.ac.uk/metagenomics/api/v1/analyses/MGYA00023273/file/SRR1632301_MERGED_FASTQ_otu.tsv	Root-Host_associated-Human-Skin
SRR920992	MGYA00023252	https://www.ebi.ac.uk/metagenomics/api/v1/analyses/MGYA00023252/file/SRR920992_FASTQ_otu.tsv	Root-Host_associated-Human-Skin
SRR920247	MGYA00023493	https://www.ebi.ac.uk/metagenomics/api/v1/analyses/MGYA00023493/file/SRR920247_FASTQ_otu.tsv	Root-Host_associated-Human-Skin
SRR1704577	MGYA00424937	https://www.ebi.ac.uk/metagenomics/api/v1/analyses/MGYA00424937/file/SRR1704577_FASTQ_SSU_OTU.tsv	Root-Host_associated-Human-Skin
ERR1759933	MGYA00140427	https://www.ebi.ac.uk/metagenomics/api/v1/analyses/MGYA00140427/file/ERR1759933_MERGED_FASTQ_SSU_OTU.tsv	Root-Host_associated-Human-Skin
SRR1950765	MGYA00376932	https://www.ebi.ac.uk/metagenomics/api/v1/analyses/MGYA00376932/file/SRR1950765_MERGED_FASTQ_SSU_OTU.tsv	Root-Host_associated-Human-Skin
SRR996498	MGYA00023398	https://www.ebi.ac.uk/metagenomics/api/v1/analyses/MGYA00023398/file/SRR996498_FASTQ_otu.tsv	Root-Host_associated-Human-Skin

SRR5869031	MGYA00426252	https://www.ebi.ac.uk/metagenomics/api/v1/analyses/MGYA00426252/file/SRR5869031_MERGED_FASTQ_SSU_OTU.tsv	Root-Host_associated-Human-Skin
SRR5869796	MGYA00416467	https://www.ebi.ac.uk/metagenomics/api/v1/analyses/MGYA00416467/file/SRR5869796_MERGED_FASTQ_SSU_OTU.tsv	Root-Host_associated-Human-Skin
SRR5869508	MGYA00419656	https://www.ebi.ac.uk/metagenomics/api/v1/analyses/MGYA00419656/file/SRR5869508_MERGED_FASTQ_SSU_OTU.tsv	Root-Host_associated-Human-Skin
SRR996606	MGYA00024577	https://www.ebi.ac.uk/metagenomics/api/v1/analyses/MGYA00024577/file/SRR996606_FASTQ_otu.tsv	Root-Host_associated-Human-Skin
SRR995713	MGYA00023353	https://www.ebi.ac.uk/metagenomics/api/v1/analyses/MGYA00023353/file/SRR995713_FASTQ_otu.tsv	Root-Host_associated-Human-Skin
SRR1704388	MGYA00426946	https://www.ebi.ac.uk/metagenomics/api/v1/analyses/MGYA00426946/file/SRR1704388_FASTQ_SSU_OTU.tsv	Root-Host_associated-Human-Skin
SRR996267	MGYA00023508	https://www.ebi.ac.uk/metagenomics/api/v1/analyses/MGYA00023508/file/SRR996267_FASTQ_otu.tsv	Root-Host_associated-Human-Skin
SRR994991	MGYA00023934	https://www.ebi.ac.uk/metagenomics/api/v1/analyses/MGYA00023934/file/SRR994991_FASTQ_otu.tsv	Root-Host_associated-Human-Skin
SRR1923878	MGYA00028362	https://www.ebi.ac.uk/metagenomics/api/v1/analyses/MGYA00028362/file/SRR1923878_FASTQ_otu.tsv	Root-Host_associated-Human-Skin
SRR996511	MGYA00023073	https://www.ebi.ac.uk/metagenomics/api/v1/analyses/MGYA00023073/file/SRR996511_FASTQ_otu.tsv	Root-Host_associated-Human-Skin
ERR1760075	MGYA00140405	https://www.ebi.ac.uk/metagenomics/api/v1/analyses/MGYA00140405/file/ERR1760075_MERGED_FASTQ_SSU_OTU.tsv	Root-Host_associated-Human-Skin
SRR995065	MGYA00024059	https://www.ebi.ac.uk/metagenomics/api/v1/analyses/MGYA00024059/file/SRR995065_FASTQ_otu.tsv	Root-Host_associated-Human-Skin

SRR5870015	MGYA00418717	https://www.ebi.ac.uk/metagenomics/api/v1/analyses/MGYA00418717/file/SRR5870015_MERGED_FASTQ_SSU_OTU.tsv	Root-Host_associated-Human-Skin
ERR443559	MGYA00422024	https://www.ebi.ac.uk/metagenomics/api/v1/analyses/MGYA00422024/file/ERR443559_FASTQ_SSU_OTU.tsv	Root-Host_associated-Human-Skin
ERR1960739	MGYA00130860	https://www.ebi.ac.uk/metagenomics/api/v1/analyses/MGYA00130860/file/ERR1960739_MERGED_FASTQ_otu.tsv	Root-Host_associated-Human-Skin
SRR997097	MGYA00022510	https://www.ebi.ac.uk/metagenomics/api/v1/analyses/MGYA00022510/file/SRR997097_FASTQ_otu.tsv	Root-Host_associated-Human-Skin
SRR1704738	MGYA00426906	https://www.ebi.ac.uk/metagenomics/api/v1/analyses/MGYA00426906/file/SRR1704738_FASTQ_SSU_OTU.tsv	Root-Host_associated-Human-Skin
SRR1631510	MGYA00023381	https://www.ebi.ac.uk/metagenomics/api/v1/analyses/MGYA00023381/file/SRR1631510_MERGED_FASTQ_otu.tsv	Root-Host_associated-Human-Skin
ERR1759990	MGYA00140534	https://www.ebi.ac.uk/metagenomics/api/v1/analyses/MGYA00140534/file/ERR1759990_MERGED_FASTQ_SSU_OTU.tsv	Root-Host_associated-Human-Skin
SRR2005710	MGYA00381340	https://www.ebi.ac.uk/metagenomics/api/v1/analyses/MGYA00381340/file/SRR2005710_MERGED_FASTQ_SSU_OTU.tsv	Root-Host_associated-Human-Skin
SRR1632405	MGYA00023546	https://www.ebi.ac.uk/metagenomics/api/v1/analyses/MGYA00023546/file/SRR1632405_MERGED_FASTQ_otu.tsv	Root-Host_associated-Human-Skin
SRR920931	MGYA00022578	https://www.ebi.ac.uk/metagenomics/api/v1/analyses/MGYA00022578/file/SRR920931_FASTQ_otu.tsv	Root-Host_associated-Human-Skin
ERR451450	MGYA00415536	https://www.ebi.ac.uk/metagenomics/api/v1/analyses/MGYA00415536/file/ERR451450_FASTQ_SSU_OTU.tsv	Root-Host_associated-Human-Skin
SRR995639	MGYA00023394	https://www.ebi.ac.uk/metagenomics/api/v1/analyses/MGYA00023394/file/SRR995639_FASTQ_otu.tsv	Root-Host_associated-Human-Skin

SRR921014	MGYA00023331	https://www.ebi.ac.uk/metagenomics/api/v1/analyses/MGYA00023331/file/SRR921014_FASTQ_otu.tsv	Root-Host_associated-Human-Skin
SRR920654	MGYA00025217	https://www.ebi.ac.uk/metagenomics/api/v1/analyses/MGYA00025217/file/SRR920654_FASTQ_otu.tsv	Root-Host_associated-Human-Skin
SRR996898	MGYA00022803	https://www.ebi.ac.uk/metagenomics/api/v1/analyses/MGYA00022803/file/SRR996898_FASTQ_otu.tsv	Root-Host_associated-Human-Skin
SRR1950717	MGYA00376884	https://www.ebi.ac.uk/metagenomics/api/v1/analyses/MGYA00376884/file/SRR1950717_MERGED_FASTQ_SSU_OTU.tsv	Root-Host_associated-Human-Skin
SRR5869043	MGYA00416024	https://www.ebi.ac.uk/metagenomics/api/v1/analyses/MGYA00416024/file/SRR5869043_MERGED_FASTQ_SSU_OTU.tsv	Root-Host_associated-Human-Skin
SRR1623061	MGYA00024415	https://www.ebi.ac.uk/metagenomics/api/v1/analyses/MGYA00024415/file/SRR1623061_MERGED_FASTQ_otu.tsv	Root-Host_associated-Human-Skin
ERR1759941	MGYA00140610	https://www.ebi.ac.uk/metagenomics/api/v1/analyses/MGYA00140610/file/ERR1759941_MERGED_FASTQ_SSU_OTU.tsv	Root-Host_associated-Human-Skin
SRR5868603	MGYA00417186	https://www.ebi.ac.uk/metagenomics/api/v1/analyses/MGYA00417186/file/SRR5868603_MERGED_FASTQ_SSU_OTU.tsv	Root-Host_associated-Human-Skin
SRR997034	MGYA00023600	https://www.ebi.ac.uk/metagenomics/api/v1/analyses/MGYA00023600/file/SRR997034_FASTQ_otu.tsv	Root-Host_associated-Human-Skin
SRR5869908	MGYA00417517	https://www.ebi.ac.uk/metagenomics/api/v1/analyses/MGYA00417517/file/SRR5869908_MERGED_FASTQ_SSU_OTU.tsv	Root-Host_associated-Human-Skin
SRR994686	MGYA00024657	https://www.ebi.ac.uk/metagenomics/api/v1/analyses/MGYA00024657/file/SRR994686_FASTQ_otu.tsv	Root-Host_associated-Human-Skin
ERR1701239	MGYA00084294	https://www.ebi.ac.uk/metagenomics/api/v1/analyses/MGYA00084294/file/ERR1701239_FASTQ_otu.tsv	Root-Host_associated-Human-Skin

SRR919864	MGYA00023897	https://www.ebi.ac.uk/metagenomics/api/v1/analyses/MGYA00023897/file/SRR919864_FASTQ_otu.tsv	Root-Host_associated-Human-Skin
SRR1631499	MGYA00024720	https://www.ebi.ac.uk/metagenomics/api/v1/analyses/MGYA00024720/file/SRR1631499_MERGED_FASTQ_otu.tsv	Root-Host_associated-Human-Skin
SRR994540	MGYA00024263	https://www.ebi.ac.uk/metagenomics/api/v1/analyses/MGYA00024263/file/SRR994540_FASTQ_otu.tsv	Root-Host_associated-Human-Skin
SRR995300	MGYA00022695	https://www.ebi.ac.uk/metagenomics/api/v1/analyses/MGYA00022695/file/SRR995300_FASTQ_otu.tsv	Root-Host_associated-Human-Skin
ERR450729	MGYA00420497	https://www.ebi.ac.uk/metagenomics/api/v1/analyses/MGYA00420497/file/ERR450729_FASTQ_SSU_OTU.tsv	Root-Host_associated-Human-Skin
SRR996563	MGYA00025298	https://www.ebi.ac.uk/metagenomics/api/v1/analyses/MGYA00025298/file/SRR996563_FASTQ_otu.tsv	Root-Host_associated-Human-Skin
SRR996911	MGYA00022438	https://www.ebi.ac.uk/metagenomics/api/v1/analyses/MGYA00022438/file/SRR996911_FASTQ_otu.tsv	Root-Host_associated-Human-Skin
SRR2005648	MGYA00381307	https://www.ebi.ac.uk/metagenomics/api/v1/analyses/MGYA00381307/file/SRR2005648_MERGED_FASTQ_SSU_OTU.tsv	Root-Host_associated-Human-Skin
SRR994657	MGYA00023446	https://www.ebi.ac.uk/metagenomics/api/v1/analyses/MGYA00023446/file/SRR994657_FASTQ_otu.tsv	Root-Host_associated-Human-Skin
SRR5870067	MGYA00418706	https://www.ebi.ac.uk/metagenomics/api/v1/analyses/MGYA00418706/file/SRR5870067_MERGED_FASTQ_SSU_OTU.tsv	Root-Host_associated-Human-Skin
ERR1760007	MGYA00140594	https://www.ebi.ac.uk/metagenomics/api/v1/analyses/MGYA00140594/file/ERR1760007_MERGED_FASTQ_SSU_OTU.tsv	Root-Host_associated-Human-Skin
ERR2538368	MGYA00381375	https://www.ebi.ac.uk/metagenomics/api/v1/analyses/MGYA00381375/file/ERR2538368_FASTQ_SSU_OTU.tsv	Root-Host_associated-Human-Skin

SRR995920	MGYA00024065	https://www.ebi.ac.uk/metagenomics/api/v1/analyses/MGYA00024065/file/SRR995920_FASTQ_otu.tsv	Root-Host_associated-Human-Skin
SRR996835	MGYA00023870	https://www.ebi.ac.uk/metagenomics/api/v1/analyses/MGYA00023870/file/SRR996835_FASTQ_otu.tsv	Root-Host_associated-Human-Skin
SRR1920449	MGYA00028279	https://www.ebi.ac.uk/metagenomics/api/v1/analyses/MGYA00028279/file/SRR1920449_FASTQ_otu.tsv	Root-Host_associated-Human-Skin
ERR1760123	MGYA00140438	https://www.ebi.ac.uk/metagenomics/api/v1/analyses/MGYA00140438/file/ERR1760123_MERGED_FASTQ_SSU_OTU.tsv	Root-Host_associated-Human-Skin
SRR995694	MGYA00023004	https://www.ebi.ac.uk/metagenomics/api/v1/analyses/MGYA00023004/file/SRR995694_FASTQ_otu.tsv	Root-Host_associated-Human-Skin
SRR1622938	MGYA00025285	https://www.ebi.ac.uk/metagenomics/api/v1/analyses/MGYA00025285/file/SRR1622938_MERGED_FASTQ_otu.tsv	Root-Host_associated-Human-Skin
SRR996026	MGYA00023723	https://www.ebi.ac.uk/metagenomics/api/v1/analyses/MGYA00023723/file/SRR996026_FASTQ_otu.tsv	Root-Host_associated-Human-Skin
ERR441987	MGYA00385193	https://www.ebi.ac.uk/metagenomics/api/v1/analyses/MGYA00385193/file/ERR441987_FASTQ_SSU_OTU.tsv	Root-Host_associated-Human-Skin
SRR996781	MGYA00024074	https://www.ebi.ac.uk/metagenomics/api/v1/analyses/MGYA00024074/file/SRR996781_FASTQ_otu.tsv	Root-Host_associated-Human-Skin
SRR997110	MGYA00024561	https://www.ebi.ac.uk/metagenomics/api/v1/analyses/MGYA00024561/file/SRR997110_FASTQ_otu.tsv	Root-Host_associated-Human-Skin
SRR995645	MGYA00024631	https://www.ebi.ac.uk/metagenomics/api/v1/analyses/MGYA00024631/file/SRR995645_FASTQ_otu.tsv	Root-Host_associated-Human-Skin
SRR1624805	MGYA00024671	https://www.ebi.ac.uk/metagenomics/api/v1/analyses/MGYA00024671/file/SRR1624805_MERGED_FASTQ_otu.tsv	Root-Host_associated-Human-Skin

SRR920628	MGYA00022770	https://www.ebi.ac.uk/metagenomics/api/v1/analyses/MGYA00022770/file/SRR920628_FASTQ_otu.tsv	Root-Host_associated-Human-Skin
ERR450968	MGYA00420159	https://www.ebi.ac.uk/metagenomics/api/v1/analyses/MGYA00420159/file/ERR450968_FASTQ_SSU_OTU.tsv	Root-Host_associated-Human-Skin
SRR996496	MGYA00023406	https://www.ebi.ac.uk/metagenomics/api/v1/analyses/MGYA00023406/file/SRR996496_FASTQ_otu.tsv	Root-Host_associated-Human-Skin
SRR919940	MGYA00024619	https://www.ebi.ac.uk/metagenomics/api/v1/analyses/MGYA00024619/file/SRR919940_FASTQ_otu.tsv	Root-Host_associated-Human-Skin
SRR919532	MGYA00024314	https://www.ebi.ac.uk/metagenomics/api/v1/analyses/MGYA00024314/file/SRR919532_FASTQ_otu.tsv	Root-Host_associated-Human-Skin
SRR920249	MGYA00023501	https://www.ebi.ac.uk/metagenomics/api/v1/analyses/MGYA00023501/file/SRR920249_FASTQ_otu.tsv	Root-Host_associated-Human-Skin
ERR442050	MGYA00385174	https://www.ebi.ac.uk/metagenomics/api/v1/analyses/MGYA00385174/file/ERR442050_FASTQ_SSU_OTU.tsv	Root-Host_associated-Human-Skin
SRR921130	MGYA00022302	https://www.ebi.ac.uk/metagenomics/api/v1/analyses/MGYA00022302/file/SRR921130_FASTQ_otu.tsv	Root-Host_associated-Human-Skin
ERR451202	MGYA00424899	https://www.ebi.ac.uk/metagenomics/api/v1/analyses/MGYA00424899/file/ERR451202_FASTQ_SSU_OTU.tsv	Root-Host_associated-Human-Skin
ERR1701245	MGYA00084250	https://www.ebi.ac.uk/metagenomics/api/v1/analyses/MGYA00084250/file/ERR1701245_FASTQ_otu.tsv	Root-Host_associated-Human-Skin
ERR450830	MGYA00412654	https://www.ebi.ac.uk/metagenomics/api/v1/analyses/MGYA00412654/file/ERR450830_FASTQ_SSU_OTU.tsv	Root-Host_associated-Human-Skin
SRR6757692	MGYA00380805	https://www.ebi.ac.uk/metagenomics/api/v1/analyses/MGYA00380805/file/SRR6757692_MERGED_FASTQ_SSU_OTU.tsv	Root-Host_associated-Human-Skin

SRR1631084	MGYA00023783	https://www.ebi.ac.uk/metagenomics/api/v1/analyses/MGYA00023783/file/SRR1631084_MERGED_FASTQ_otu.tsv	Root-Host_associated-Human-Skin
ERR2538462	MGYA00381469	https://www.ebi.ac.uk/metagenomics/api/v1/analyses/MGYA00381469/file/ERR2538462_FASTQ_SSU_OTU.tsv	Root-Host_associated-Human-Skin
SRR996608	MGYA00024579	https://www.ebi.ac.uk/metagenomics/api/v1/analyses/MGYA00024579/file/SRR996608_FASTQ_otu.tsv	Root-Host_associated-Human-Skin
SRR1632982	MGYA00025154	https://www.ebi.ac.uk/metagenomics/api/v1/analyses/MGYA00025154/file/SRR1632982_MERGED_FASTQ_otu.tsv	Root-Host_associated-Human-Skin
ERR443640	MGYA00421383	https://www.ebi.ac.uk/metagenomics/api/v1/analyses/MGYA00421383/file/ERR443640_FASTQ_SSU_OTU.tsv	Root-Host_associated-Human-Skin
SRR1630970	MGYA00024354	https://www.ebi.ac.uk/metagenomics/api/v1/analyses/MGYA00024354/file/SRR1630970_MERGED_FASTQ_otu.tsv	Root-Host_associated-Human-Skin
SRR996269	MGYA00023512	https://www.ebi.ac.uk/metagenomics/api/v1/analyses/MGYA00023512/file/SRR996269_FASTQ_otu.tsv	Root-Host_associated-Human-Skin
SRR5868727	MGYA00421201	https://www.ebi.ac.uk/metagenomics/api/v1/analyses/MGYA00421201/file/SRR5868727_MERGED_FASTQ_SSU_OTU.tsv	Root-Host_associated-Human-Skin
SRR1631342	MGYA00022805	https://www.ebi.ac.uk/metagenomics/api/v1/analyses/MGYA00022805/file/SRR1631342_MERGED_FASTQ_otu.tsv	Root-Host_associated-Human-Skin
SRR1704386	MGYA00428870	https://www.ebi.ac.uk/metagenomics/api/v1/analyses/MGYA00428870/file/SRR1704386_FASTQ_SSU_OTU.tsv	Root-Host_associated-Human-Skin
SRR1631393	MGYA00022738	https://www.ebi.ac.uk/metagenomics/api/v1/analyses/MGYA00022738/file/SRR1631393_MERGED_FASTQ_otu.tsv	Root-Host_associated-Human-Skin
SRR1631434	MGYA00025318	https://www.ebi.ac.uk/metagenomics/api/v1/analyses/MGYA00025318/file/SRR1631434_MERGED_FASTQ_otu.tsv	Root-Host_associated-Human-Skin

SRR1923876	MGYA00028349	https://www.ebi.ac.uk/metagenomics/api/v1/analyses/MGYA00028349/file/SRR1923876_FASTQ_otu.tsv	Root-Host_associated-Human-Skin
ERR443136	MGYA00420148	https://www.ebi.ac.uk/metagenomics/api/v1/analyses/MGYA00420148/file/ERR443136_FASTQ_SSU_OTU.tsv	Root-Host_associated-Human-Skin
ERR450755	MGYA00412661	https://www.ebi.ac.uk/metagenomics/api/v1/analyses/MGYA00412661/file/ERR450755_FASTQ_SSU_OTU.tsv	Root-Host_associated-Human-Skin
SRR5868842	MGYA00420747	https://www.ebi.ac.uk/metagenomics/api/v1/analyses/MGYA00420747/file/SRR5868842_MERGED_FASTQ_SSU_OTU.tsv	Root-Host_associated-Human-Skin
SRR997099	MGYA00022519	https://www.ebi.ac.uk/metagenomics/api/v1/analyses/MGYA00022519/file/SRR997099_FASTQ_otu.tsv	Root-Host_associated-Human-Skin
SRR6757643	MGYA00380758	https://www.ebi.ac.uk/metagenomics/api/v1/analyses/MGYA00380758/file/SRR6757643_MERGED_FASTQ_SSU_OTU.tsv	Root-Host_associated-Human-Skin
ERR443557	MGYA00426007	https://www.ebi.ac.uk/metagenomics/api/v1/analyses/MGYA00426007/file/ERR443557_FASTQ_SSU_OTU.tsv	Root-Host_associated-Human-Skin
ERR1701294	MGYA00084241	https://www.ebi.ac.uk/metagenomics/api/v1/analyses/MGYA00084241/file/ERR1701294_FASTQ_otu.tsv	Root-Host_associated-Human-Skin
ERR1960737	MGYA00130741	https://www.ebi.ac.uk/metagenomics/api/v1/analyses/MGYA00130741/file/ERR1960737_MERGED_FASTQ_otu.tsv	Root-Host_associated-Human-Skin
SRR5870282	MGYA00416925	https://www.ebi.ac.uk/metagenomics/api/v1/analyses/MGYA00416925/file/SRR5870282_MERGED_FASTQ_SSU_OTU.tsv	Root-Host_associated-Human-Skin
SRR995927	MGYA00024072	https://www.ebi.ac.uk/metagenomics/api/v1/analyses/MGYA00024072/file/SRR995927_FASTQ_otu.tsv	Root-Host_associated-Human-Skin
SRR996832	MGYA00023865	https://www.ebi.ac.uk/metagenomics/api/v1/analyses/MGYA00023865/file/SRR996832_FASTQ_otu.tsv	Root-Host_associated-Human-Skin

SRR1704208	MGYA00417864	https://www.ebi.ac.uk/metagenomics/api/v1/analyses/MGYA00417864/file/SRR1704208_FASTQ_SSU_OTU.tsv	Root-Host_associated-Human-Skin
SRR995693	MGYA00023007	https://www.ebi.ac.uk/metagenomics/api/v1/analyses/MGYA00023007/file/SRR995693_FASTQ_otu.tsv	Root-Host_associated-Human-Skin
ERR1760124	MGYA00140490	https://www.ebi.ac.uk/metagenomics/api/v1/analyses/MGYA00140490/file/ERR1760124_MERGED_FASTQ_SSU_OTU.tsv	Root-Host_associated-Human-Skin
ERR1960668	MGYA00130754	https://www.ebi.ac.uk/metagenomics/api/v1/analyses/MGYA00130754/file/ERR1960668_MERGED_FASTQ_otu.tsv	Root-Host_associated-Human-Skin
ERR443408	MGYA00415156	https://www.ebi.ac.uk/metagenomics/api/v1/analyses/MGYA00415156/file/ERR443408_FASTQ_SSU_OTU.tsv	Root-Host_associated-Human-Skin
SRR994774	MGYA00023028	https://www.ebi.ac.uk/metagenomics/api/v1/analyses/MGYA00023028/file/SRR994774_FASTQ_otu.tsv	Root-Host_associated-Human-Skin
SRR920159	MGYA00024508	https://www.ebi.ac.uk/metagenomics/api/v1/analyses/MGYA00024508/file/SRR920159_FASTQ_otu.tsv	Root-Host_associated-Human-Skin
SRR996786	MGYA00024081	https://www.ebi.ac.uk/metagenomics/api/v1/analyses/MGYA00024081/file/SRR996786_FASTQ_otu.tsv	Root-Host_associated-Human-Skin
ERR441980	MGYA00385187	https://www.ebi.ac.uk/metagenomics/api/v1/analyses/MGYA00385187/file/ERR441980_FASTQ_SSU_OTU.tsv	Root-Host_associated-Human-Skin
SRR996021	MGYA00023726	https://www.ebi.ac.uk/metagenomics/api/v1/analyses/MGYA00023726/file/SRR996021_FASTQ_otu.tsv	Root-Host_associated-Human-Skin
SRR997117	MGYA00024562	https://www.ebi.ac.uk/metagenomics/api/v1/analyses/MGYA00024562/file/SRR997117_FASTQ_otu.tsv	Root-Host_associated-Human-Skin
SRR1624802	MGYA00024672	https://www.ebi.ac.uk/metagenomics/api/v1/analyses/MGYA00024672/file/SRR1624802_MERGED_FASTQ_otu.tsv	Root-Host_associated-Human-Skin

SRR995642	MGYA00024630	https://www.ebi.ac.uk/metagenomics/api/v1/analyses/MGYA00024630/file/SRR995642_FASTQ_otu.tsv	Root-Host_associated-Human-Skin
SRR919535	MGYA00024311	https://www.ebi.ac.uk/metagenomics/api/v1/analyses/MGYA00024311/file/SRR919535_FASTQ_otu.tsv	Root-Host_associated-Human-Skin
SRR919947	MGYA00024622	https://www.ebi.ac.uk/metagenomics/api/v1/analyses/MGYA00024622/file/SRR919947_FASTQ_otu.tsv	Root-Host_associated-Human-Skin
SRR996491	MGYA00023399	https://www.ebi.ac.uk/metagenomics/api/v1/analyses/MGYA00023399/file/SRR996491_FASTQ_otu.tsv	Root-Host_associated-Human-Skin
SRR995584	MGYA00022319	https://www.ebi.ac.uk/metagenomics/api/v1/analyses/MGYA00022319/file/SRR995584_FASTQ_otu.tsv	Root-Host_associated-Human-Skin
ERR442057	MGYA00385200	https://www.ebi.ac.uk/metagenomics/api/v1/analyses/MGYA00385200/file/ERR442057_FASTQ_SSU_OTU.tsv	Root-Host_associated-Human-Skin
SRR1632308	MGYA00023278	https://www.ebi.ac.uk/metagenomics/api/v1/analyses/MGYA00023278/file/SRR1632308_MERGED_FASTQ_otu.tsv	Root-Host_associated-Human-Skin
ERR1701242	MGYA00084327	https://www.ebi.ac.uk/metagenomics/api/v1/analyses/MGYA00084327/file/ERR1701242_FASTQ_otu.tsv	Root-Host_associated-Human-Skin
SRR921137	MGYA00023218	https://www.ebi.ac.uk/metagenomics/api/v1/analyses/MGYA00023218/file/SRR921137_FASTQ_otu.tsv	Root-Host_associated-Human-Skin
ERR2538465	MGYA00381472	https://www.ebi.ac.uk/metagenomics/api/v1/analyses/MGYA00381472/file/ERR2538465_FASTQ_SSU_OTU.tsv	Root-Host_associated-Human-Skin
SRR6757695	MGYA00380808	https://www.ebi.ac.uk/metagenomics/api/v1/analyses/MGYA00380808/file/SRR6757695_MERGED_FASTQ_SSU_OTU.tsv	Root-Host_associated-Human-Skin
SRR1631083	MGYA00023790	https://www.ebi.ac.uk/metagenomics/api/v1/analyses/MGYA00023790/file/SRR1631083_MERGED_FASTQ_otu.tsv	Root-Host_associated-Human-Skin

SRR994949	MGYA00022491	https://www.ebi.ac.uk/metagenomics/api/v1/analyses/MGYA00022491/file/SRR994949_FASTQ_otu.tsv	Root-Host_associated-Human-Skin
SRR1632985	MGYA00025157	https://www.ebi.ac.uk/metagenomics/api/v1/analyses/MGYA00025157/file/SRR1632985_MERGED_FASTQ_otu.tsv	Root-Host_associated-Human-Skin
ERR443647	MGYA00421381	https://www.ebi.ac.uk/metagenomics/api/v1/analyses/MGYA00421381/file/ERR443647_FASTQ_SSU_OTU.tsv	Root-Host_associated-Human-Skin
SRR1704381	MGYA00420181	https://www.ebi.ac.uk/metagenomics/api/v1/analyses/MGYA00420181/file/SRR1704381_FASTQ_SSU_OTU.tsv	Root-Host_associated-Human-Skin
SRR5868720	MGYA00417126	https://www.ebi.ac.uk/metagenomics/api/v1/analyses/MGYA00417126/file/SRR5868720_MERGED_FASTQ_SSU_OTU.tsv	Root-Host_associated-Human-Skin
SRR1631345	MGYA00022810	https://www.ebi.ac.uk/metagenomics/api/v1/analyses/MGYA00022810/file/SRR1631345_MERGED_FASTQ_otu.tsv	Root-Host_associated-Human-Skin
SRR1704426	MGYA00424945	https://www.ebi.ac.uk/metagenomics/api/v1/analyses/MGYA00424945/file/SRR1704426_FASTQ_SSU_OTU.tsv	Root-Host_associated-Human-Skin
SRR1630977	MGYA00024347	https://www.ebi.ac.uk/metagenomics/api/v1/analyses/MGYA00024347/file/SRR1630977_MERGED_FASTQ_otu.tsv	Root-Host_associated-Human-Skin
SRR5869160	MGYA00429100	https://www.ebi.ac.uk/metagenomics/api/v1/analyses/MGYA00429100/file/SRR5869160_MERGED_FASTQ_SSU_OTU.tsv	Root-Host_associated-Human-Skin
SRR996518	MGYA00023066	https://www.ebi.ac.uk/metagenomics/api/v1/analyses/MGYA00023066/file/SRR996518_FASTQ_otu.tsv	Root-Host_associated-Human-Skin
SRR1704350	MGYA00421503	https://www.ebi.ac.uk/metagenomics/api/v1/analyses/MGYA00421503/file/SRR1704350_FASTQ_SSU_OTU.tsv	Root-Host_associated-Human-Skin
ERR443696	MGYA00424272	https://www.ebi.ac.uk/metagenomics/api/v1/analyses/MGYA00424272/file/ERR443696_FASTQ_SSU_OTU.tsv	Root-Host_associated-Human-Skin

SRR994998	MGYA00023935	https://www.ebi.ac.uk/metagenomics/api/v1/analyses/MGYA00023935/file/SRR994998_FASTQ_otu.tsv	Root-Host_associated-Human-Skin
SRR1923871	MGYA00028354	https://www.ebi.ac.uk/metagenomics/api/v1/analyses/MGYA00028354/file/SRR1923871_FASTQ_otu.tsv	Root-Host_associated-Human-Skin
SRR1704731	MGYA00425792	https://www.ebi.ac.uk/metagenomics/api/v1/analyses/MGYA00425792/file/SRR1704731_FASTQ_SSU_OTU.tsv	Root-Host_associated-Human-Skin
SRR6757644	MGYA00380759	https://www.ebi.ac.uk/metagenomics/api/v1/analyses/MGYA00380759/file/SRR6757644_MERGED_FASTQ_SSU_OTU.tsv	Root-Host_associated-Human-Skin
SRR5868845	MGYA00426662	https://www.ebi.ac.uk/metagenomics/api/v1/analyses/MGYA00426662/file/SRR5868845_MERGED_FASTQ_SSU_OTU.tsv	Root-Host_associated-Human-Skin
ERR1960730	MGYA00130723	https://www.ebi.ac.uk/metagenomics/api/v1/analyses/MGYA00130723/file/ERR1960730_MERGED_FASTQ_otu.tsv	Root-Host_associated-Human-Skin
ERR1701293	MGYA00084276	https://www.ebi.ac.uk/metagenomics/api/v1/analyses/MGYA00084276/file/ERR1701293_FASTQ_otu.tsv	Root-Host_associated-Human-Skin
SRR5869095	MGYA00416013	https://www.ebi.ac.uk/metagenomics/api/v1/analyses/MGYA00416013/file/SRR5869095_MERGED_FASTQ_SSU_OTU.tsv	Root-Host_associated-Human-Skin
SRR2005717	MGYA00381346	https://www.ebi.ac.uk/metagenomics/api/v1/analyses/MGYA00381346/file/SRR2005717_MERGED_FASTQ_SSU_OTU.tsv	Root-Host_associated-Human-Skin
SRR919598	MGYA00024957	https://www.ebi.ac.uk/metagenomics/api/v1/analyses/MGYA00024957/file/SRR919598_FASTQ_otu.tsv	Root-Host_associated-Human-Skin
SRR1631517	MGYA00023384	https://www.ebi.ac.uk/metagenomics/api/v1/analyses/MGYA00023384/file/SRR1631517_MERGED_FASTQ_otu.tsv	Root-Host_associated-Human-Skin
ERR1759997	MGYA00140431	https://www.ebi.ac.uk/metagenomics/api/v1/analyses/MGYA00140431/file/ERR1759997_MERGED_FASTQ_SSU_OTU.tsv	Root-Host_associated-Human-Skin

ERR443015	MGYA00426794	https://www.ebi.ac.uk/metagenomics/api/v1/analyses/MGYA00426794/file/ERR443015_FASTQ_SSU_OTU.tsv	Root-Host_associated-Human-Skin
SRR920936	MGYA00022573	https://www.ebi.ac.uk/metagenomics/api/v1/analyses/MGYA00022573/file/SRR920936_FASTQ_otu.tsv	Root-Host_associated-Human-Skin
SRR1632402	MGYA00023553	https://www.ebi.ac.uk/metagenomics/api/v1/analyses/MGYA00023553/file/SRR1632402_MERGED_FASTQ_otu.tsv	Root-Host_associated-Human-Skin
SRR995529	MGYA00023623	https://www.ebi.ac.uk/metagenomics/api/v1/analyses/MGYA00023623/file/SRR995529_FASTQ_otu.tsv	Root-Host_associated-Human-Skin
SRR1923955	MGYA00028391	https://www.ebi.ac.uk/metagenomics/api/v1/analyses/MGYA00028391/file/SRR1923955_FASTQ_otu.tsv	Root-Host_associated-Human-Skin
SRR2005376	MGYA00381286	https://www.ebi.ac.uk/metagenomics/api/v1/analyses/MGYA00381286/file/SRR2005376_MERGED_FASTQ_SSU_OTU.tsv	Root-Host_associated-Human-Skin
SRR995148	MGYA00024142	https://www.ebi.ac.uk/metagenomics/api/v1/analyses/MGYA00024142/file/SRR995148_FASTQ_otu.tsv	Root-Host_associated-Human-Skin
SRR994708	MGYA00024495	https://www.ebi.ac.uk/metagenomics/api/v1/analyses/MGYA00024495/file/SRR994708_FASTQ_otu.tsv	Root-Host_associated-Human-Skin
SRR920653	MGYA00025212	https://www.ebi.ac.uk/metagenomics/api/v1/analyses/MGYA00025212/file/SRR920653_FASTQ_otu.tsv	Root-Host_associated-Human-Skin
SRR921013	MGYA00023324	https://www.ebi.ac.uk/metagenomics/api/v1/analyses/MGYA00023324/file/SRR921013_FASTQ_otu.tsv	Root-Host_associated-Human-Skin
ERR443763	MGYA00414524	https://www.ebi.ac.uk/metagenomics/api/v1/analyses/MGYA00414524/file/ERR443763_FASTQ_SSU_OTU.tsv	Root-Host_associated-Human-Skin
SRR5868604	MGYA00420291	https://www.ebi.ac.uk/metagenomics/api/v1/analyses/MGYA00420291/file/SRR5868604_MERGED_FASTQ_SSU_OTU.tsv	Root-Host_associated-Human-Skin

SRR1704502	MGYA00414639	https://www.ebi.ac.uk/metagenomics/api/v1/analyses/MGYA00414639/file/SRR1704502_FASTQ_SSU_OTU.tsv	Root-Host_associated-Human-Skin
SRR1631261	MGYA00024983	https://www.ebi.ac.uk/metagenomics/api/v1/analyses/MGYA00024983/file/SRR1631261_MERGED_FASTQ_otu.tsv	Root-Host_associated-Human-Skin
SRR1623066	MGYA00024414	https://www.ebi.ac.uk/metagenomics/api/v1/analyses/MGYA00024414/file/SRR1623066_MERGED_FASTQ_otu.tsv	Root-Host_associated-Human-Skin
ERR1759946	MGYA00140565	https://www.ebi.ac.uk/metagenomics/api/v1/analyses/MGYA00140565/file/ERR1759946_MERGED_FASTQ_SSU_OTU.tsv	Root-Host_associated-Human-Skin
SRR1950710	MGYA00376877	https://www.ebi.ac.uk/metagenomics/api/v1/analyses/MGYA00376877/file/SRR1950710_MERGED_FASTQ_SSU_OTU.tsv	Root-Host_associated-Human-Skin
ERR2538419	MGYA00381426	https://www.ebi.ac.uk/metagenomics/api/v1/analyses/MGYA00381426/file/ERR2538419_FASTQ_SSU_OTU.tsv	Root-Host_associated-Human-Skin
SRR997033	MGYA00023607	https://www.ebi.ac.uk/metagenomics/api/v1/analyses/MGYA00023607/file/SRR997033_FASTQ_otu.tsv	Root-Host_associated-Human-Skin
SRR1923768	MGYA00028385	https://www.ebi.ac.uk/metagenomics/api/v1/analyses/MGYA00028385/file/SRR1923768_FASTQ_otu.tsv	Root-Host_associated-Human-Skin
SRR919863	MGYA00023902	https://www.ebi.ac.uk/metagenomics/api/v1/analyses/MGYA00023902/file/SRR919863_FASTQ_otu.tsv	Root-Host_associated-Human-Skin
SRR995307	MGYA00022702	https://www.ebi.ac.uk/metagenomics/api/v1/analyses/MGYA00022702/file/SRR995307_FASTQ_otu.tsv	Root-Host_associated-Human-Skin
SRR1922549	MGYA00028400	https://www.ebi.ac.uk/metagenomics/api/v1/analyses/MGYA00028400/file/SRR1922549_FASTQ_otu.tsv	Root-Host_associated-Human-Skin
ERR1960829	MGYA00130816	https://www.ebi.ac.uk/metagenomics/api/v1/analyses/MGYA00130816/file/ERR1960829_MERGED_FASTQ_otu.tsv	Root-Host_associated-Human-Skin

SRR994547	MGYA00024260	https://www.ebi.ac.uk/metagenomics/api/v1/analyses/MGYA00024260/file/SRR994547_FASTQ_otu.tsv	Root-Host_associated-Human-Skin
SRR994935	MGYA00024959	https://www.ebi.ac.uk/metagenomics/api/v1/analyses/MGYA00024959/file/SRR994935_FASTQ_otu.tsv	Root-Host_associated-Human-Skin
SRR995471	MGYA00025105	https://www.ebi.ac.uk/metagenomics/api/v1/analyses/MGYA00025105/file/SRR995471_FASTQ_otu.tsv	Root-Host_associated-Human-Skin
SRR996916	MGYA00022443	https://www.ebi.ac.uk/metagenomics/api/v1/analyses/MGYA00022443/file/SRR996916_FASTQ_otu.tsv	Root-Host_associated-Human-Skin
SRR1950699	MGYA00376866	https://www.ebi.ac.uk/metagenomics/api/v1/analyses/MGYA00376866/file/SRR1950699_MERGED_FASTQ_SSU_OTU.tsv	Root-Host_associated-Human-Skin
SRR996564	MGYA00025301	https://www.ebi.ac.uk/metagenomics/api/v1/analyses/MGYA00025301/file/SRR996564_FASTQ_otu.tsv	Root-Host_associated-Human-Skin
ERR1760000	MGYA00140596	https://www.ebi.ac.uk/metagenomics/api/v1/analyses/MGYA00140596/file/ERR1760000_MERGED_FASTQ_SSU_OTU.tsv	Root-Host_associated-Human-Skin
SRR994650	MGYA00023449	https://www.ebi.ac.uk/metagenomics/api/v1/analyses/MGYA00023449/file/SRR994650_FASTQ_otu.tsv	Root-Host_associated-Human-Skin
SRR6757638	MGYA00380753	https://www.ebi.ac.uk/metagenomics/api/v1/analyses/MGYA00380753/file/SRR6757638_MERGED_FASTQ_SSU_OTU.tsv	Root-Host_associated-Human-Skin
SRR1950759	MGYA00376926	https://www.ebi.ac.uk/metagenomics/api/v1/analyses/MGYA00376926/file/SRR1950759_MERGED_FASTQ_SSU_OTU.tsv	Root-Host_associated-Human-Skin
SRR996303	MGYA00022713	https://www.ebi.ac.uk/metagenomics/api/v1/analyses/MGYA00022713/file/SRR996303_FASTQ_otu.tsv	Root-Host_associated-Human-Skin
ERR442062	MGYA00385218	https://www.ebi.ac.uk/metagenomics/api/v1/analyses/MGYA00385218/file/ERR442062_FASTQ_SSU_OTU.tsv	Root-Host_associated-Human-Skin

SRR994456	MGYA00022460	https://www.ebi.ac.uk/metagenomics/api/v1/analyses/MGYA00022460/file/SRR994456_FASTQ_otu.tsv	Root-Host_associated-Human-Skin
ERR441964	MGYA00385235	https://www.ebi.ac.uk/metagenomics/api/v1/analyses/MGYA00385235/file/ERR441964_FASTQ_SSU_OTU.tsv	Root-Host_associated-Human-Skin
ERR2538508	MGYA00381515	https://www.ebi.ac.uk/metagenomics/api/v1/analyses/MGYA00381515/file/ERR2538508_FASTQ_SSU_OTU.tsv	Root-Host_associated-Human-Skin
SRR996762	MGYA00023857	https://www.ebi.ac.uk/metagenomics/api/v1/analyses/MGYA00023857/file/SRR996762_FASTQ_otu.tsv	Root-Host_associated-Human-Skin
SRR994790	MGYA00022832	https://www.ebi.ac.uk/metagenomics/api/v1/analyses/MGYA00022832/file/SRR994790_FASTQ_otu.tsv	Root-Host_associated-Human-Skin
SRR995101	MGYA00023667	https://www.ebi.ac.uk/metagenomics/api/v1/analyses/MGYA00023667/file/SRR995101_FASTQ_otu.tsv	Root-Host_associated-Human-Skin
ERR1760111	MGYA00140581	https://www.ebi.ac.uk/metagenomics/api/v1/analyses/MGYA00140581/file/ERR1760111_MERGED_FASTQ_SSU_OTU.tsv	Root-Host_associated-Human-Skin
SRR919617	MGYA00023529	https://www.ebi.ac.uk/metagenomics/api/v1/analyses/MGYA00023529/file/SRR919617_FASTQ_otu.tsv	Root-Host_associated-Human-Skin
SRR2005498	MGYA00381293	https://www.ebi.ac.uk/metagenomics/api/v1/analyses/MGYA00381293/file/SRR2005498_MERGED_FASTQ_SSU_OTU.tsv	Root-Host_associated-Human-Skin
SRR994487	MGYA00023147	https://www.ebi.ac.uk/metagenomics/api/v1/analyses/MGYA00023147/file/SRR994487_FASTQ_otu.tsv	Root-Host_associated-Human-Skin
SRR1632839	MGYA00022823	https://www.ebi.ac.uk/metagenomics/api/v1/analyses/MGYA00022823/file/SRR1632839_MERGED_FASTQ_otu.tsv	Root-Host_associated-Human-Skin
SRR995560	MGYA00022978	https://www.ebi.ac.uk/metagenomics/api/v1/analyses/MGYA00022978/file/SRR995560_FASTQ_otu.tsv	Root-Host_associated-Human-Skin

SRR1950788	MGYA00376955	https://www.ebi.ac.uk/metagenomics/api/v1/analyses/MGYA00376955/file/SRR1950788_MERGED_FASTQ_SSU_OTU.tsv	Root-Host_associated-Human-Skin
SRR6757671	MGYA00380784	https://www.ebi.ac.uk/metagenomics/api/v1/analyses/MGYA00380784/file/SRR6757671_MERGED_FASTQ_SSU_OTU.tsv	Root-Host_associated-Human-Skin
SRR5868870	MGYA00416503	https://www.ebi.ac.uk/metagenomics/api/v1/analyses/MGYA00416503/file/SRR5868870_MERGED_FASTQ_SSU_OTU.tsv	Root-Host_associated-Human-Skin
SRR1631067	MGYA00024807	https://www.ebi.ac.uk/metagenomics/api/v1/analyses/MGYA00024807/file/SRR1631067_MERGED_FASTQ_otu.tsv	Root-Host_associated-Human-Skin
ERR2538481	MGYA00381488	https://www.ebi.ac.uk/metagenomics/api/v1/analyses/MGYA00381488/file/ERR2538481_FASTQ_SSU_OTU.tsv	Root-Host_associated-Human-Skin
SRR1622852	MGYA00024385	https://www.ebi.ac.uk/metagenomics/api/v1/analyses/MGYA00024385/file/SRR1622852_MERGED_FASTQ_otu.tsv	Root-Host_associated-Human-Skin
ERR1760049	MGYA00140432	https://www.ebi.ac.uk/metagenomics/api/v1/analyses/MGYA00140432/file/ERR1760049_MERGED_FASTQ_SSU_OTU.tsv	Root-Host_associated-Human-Skin
SRR5870029	MGYA00420678	https://www.ebi.ac.uk/metagenomics/api/v1/analyses/MGYA00420678/file/SRR5870029_MERGED_FASTQ_SSU_OTU.tsv	Root-Host_associated-Human-Skin
SRR994619	MGYA00023010	https://www.ebi.ac.uk/metagenomics/api/v1/analyses/MGYA00023010/file/SRR994619_FASTQ_otu.tsv	Root-Host_associated-Human-Skin
ERR443565	MGYA00421395	https://www.ebi.ac.uk/metagenomics/api/v1/analyses/MGYA00421395/file/ERR443565_FASTQ_SSU_OTU.tsv	Root-Host_associated-Human-Skin
ERR1960705	MGYA00130729	https://www.ebi.ac.uk/metagenomics/api/v1/analyses/MGYA00130729/file/ERR1960705_MERGED_FASTQ_otu.tsv	Root-Host_associated-Human-Skin
SRR1630993	MGYA00022851	https://www.ebi.ac.uk/metagenomics/api/v1/analyses/MGYA00022851/file/SRR1630993_MERGED_FASTQ_otu.tsv	Root-Host_associated-Human-Skin

ERR1759886	MGYA00140547	https://www.ebi.ac.uk/metagenomics/api/v1/analyses/MGYA00140547/file/ERR1759886_MERGED_FASTQ_SSU_OTU.tsv	Root-Host_associated-Human-Skin
ERR450767	MGYA00415573	https://www.ebi.ac.uk/metagenomics/api/v1/analyses/MGYA00415573/file/ERR450767_FASTQ_SSU_OTU.tsv	Root-Host_associated-Human-Skin
SRR1923895	MGYA00028371	https://www.ebi.ac.uk/metagenomics/api/v1/analyses/MGYA00028371/file/SRR1923895_FASTQ_otu.tsv	Root-Host_associated-Human-Skin
ERR443672	MGYA00421380	https://www.ebi.ac.uk/metagenomics/api/v1/analyses/MGYA00421380/file/ERR443672_FASTQ_SSU_OTU.tsv	Root-Host_associated-Human-Skin
ERR1701277	MGYA00084309	https://www.ebi.ac.uk/metagenomics/api/v1/analyses/MGYA00084309/file/ERR1701277_FASTQ_otu.tsv	Root-Host_associated-Human-Skin
SRR920742	MGYA00023538	https://www.ebi.ac.uk/metagenomics/api/v1/analyses/MGYA00023538/file/SRR920742_FASTQ_otu.tsv	Root-Host_associated-Human-Skin
ERR1760098	MGYA00140473	https://www.ebi.ac.uk/metagenomics/api/v1/analyses/MGYA00140473/file/ERR1760098_MERGED_FASTQ_SSU_OTU.tsv	Root-Host_associated-Human-Skin
SRR995088	MGYA00023281	https://www.ebi.ac.uk/metagenomics/api/v1/analyses/MGYA00023281/file/SRR995088_FASTQ_otu.tsv	Root-Host_associated-Human-Skin
SRR921102	MGYA00025006	https://www.ebi.ac.uk/metagenomics/api/v1/analyses/MGYA00025006/file/SRR921102_FASTQ_otu.tsv	Root-Host_associated-Human-Skin
SRR5869946	MGYA00414819	https://www.ebi.ac.uk/metagenomics/api/v1/analyses/MGYA00414819/file/SRR5869946_MERGED_FASTQ_SSU_OTU.tsv	Root-Host_associated-Human-Skin
ERR2538450	MGYA00381457	https://www.ebi.ac.uk/metagenomics/api/v1/analyses/MGYA00381457/file/ERR2538450_FASTQ_SSU_OTU.tsv	Root-Host_associated-Human-Skin
SRR5869293	MGYA00419710	https://www.ebi.ac.uk/metagenomics/api/v1/analyses/MGYA00419710/file/SRR5869293_MERGED_FASTQ_SSU_OTU.tsv	Root-Host_associated-Human-Skin

SRR1631711	MGYA00024040	https://www.ebi.ac.uk/metagenomics/api/v1/analyses/MGYA00024040/file/SRR1631711_MERGED_FASTQ_otu.tsv	Root-Host_associated-Human-Skin
ERR443756	MGYA00422352	https://www.ebi.ac.uk/metagenomics/api/v1/analyses/MGYA00422352/file/ERR443756_FASTQ_SSU_OTU.tsv	Root-Host_associated-Human-Skin
SRR920207	MGYA00023062	https://www.ebi.ac.uk/metagenomics/api/v1/analyses/MGYA00023062/file/SRR920207_FASTQ_otu.tsv	Root-Host_associated-Human-Skin
SRR1632341	MGYA00025169	https://www.ebi.ac.uk/metagenomics/api/v1/analyses/MGYA00025169/file/SRR1632341_MERGED_FASTQ_otu.tsv	Root-Host_associated-Human-Skin
SRR1704290	MGYA00428887	https://www.ebi.ac.uk/metagenomics/api/v1/analyses/MGYA00428887/file/SRR1704290_FASTQ_SSU_OTU.tsv	Root-Host_associated-Human-Skin
ERR1759973	MGYA00140483	https://www.ebi.ac.uk/metagenomics/api/v1/analyses/MGYA00140483/file/ERR1759973_MERGED_FASTQ_SSU_OTU.tsv	Root-Host_associated-Human-Skin
SRR1631254	MGYA00024545	https://www.ebi.ac.uk/metagenomics/api/v1/analyses/MGYA00024545/file/SRR1631254_MERGED_FASTQ_otu.tsv	Root-Host_associated-Human-Skin
SRR1950725	MGYA00376892	https://www.ebi.ac.uk/metagenomics/api/v1/analyses/MGYA00376892/file/SRR1950725_MERGED_FASTQ_SSU_OTU.tsv	Root-Host_associated-Human-Skin
ERR443337	MGYA00428748	https://www.ebi.ac.uk/metagenomics/api/v1/analyses/MGYA00428748/file/ERR443337_FASTQ_SSU_OTU.tsv	Root-Host_associated-Human-Skin
SRR920666	MGYA00022334	https://www.ebi.ac.uk/metagenomics/api/v1/analyses/MGYA00022334/file/SRR920666_FASTQ_otu.tsv	Root-Host_associated-Human-Skin
ERR451314	MGYA00419503	https://www.ebi.ac.uk/metagenomics/api/v1/analyses/MGYA00419503/file/ERR451314_FASTQ_SSU_OTU.tsv	Root-Host_associated-Human-Skin
SRR1633160	MGYA00022385	https://www.ebi.ac.uk/metagenomics/api/v1/analyses/MGYA00022385/file/SRR1633160_MERGED_FASTQ_otu.tsv	Root-Host_associated-Human-Skin

SRR1704156	MGYA00425338	https://www.ebi.ac.uk/metagenomics/api/v1/analyses/MGYA00425338/file/SRR1704156_FASTQ_SSU_OTU.tsv	Root-Host_associated-Human-Skin
SRR1704620	MGYA00421468	https://www.ebi.ac.uk/metagenomics/api/v1/analyses/MGYA00421468/file/SRR1704620_FASTQ_SSU_OTU.tsv	Root-Host_associated-Human-Skin
SRR996068	MGYA00023322	https://www.ebi.ac.uk/metagenomics/api/v1/analyses/MGYA00023322/file/SRR996068_FASTQ_otu.tsv	Root-Host_associated-Human-Skin
ERR443833	MGYA00428370	https://www.ebi.ac.uk/metagenomics/api/v1/analyses/MGYA00428370/file/ERR443833_FASTQ_SSU_OTU.tsv	Root-Host_associated-Human-Skin
ERR443441	MGYA00412371	https://www.ebi.ac.uk/metagenomics/api/v1/analyses/MGYA00412371/file/ERR443441_FASTQ_SSU_OTU.tsv	Root-Host_associated-Human-Skin
SRR5869707	MGYA00421589	https://www.ebi.ac.uk/metagenomics/api/v1/analyses/MGYA00421589/file/SRR5869707_MERGED_FASTQ_SSU_OTU.tsv	Root-Host_associated-Human-Skin
SRR2005722	MGYA00381351	https://www.ebi.ac.uk/metagenomics/api/v1/analyses/MGYA00381351/file/SRR2005722_MERGED_FASTQ_SSU_OTU.tsv	Root-Host_associated-Human-Skin
SRR1631285	MGYA00025255	https://www.ebi.ac.uk/metagenomics/api/v1/analyses/MGYA00025255/file/SRR1631285_MERGED_FASTQ_otu.tsv	Root-Host_associated-Human-Skin
SRR1923960	MGYA00028379	https://www.ebi.ac.uk/metagenomics/api/v1/analyses/MGYA00028379/file/SRR1923960_FASTQ_otu.tsv	Root-Host_associated-Human-Skin
ERR1760035	MGYA00140402	https://www.ebi.ac.uk/metagenomics/api/v1/analyses/MGYA00140402/file/ERR1760035_MERGED_FASTQ_SSU_OTU.tsv	Root-Host_associated-Human-Skin
SRR995025	MGYA00023945	https://www.ebi.ac.uk/metagenomics/api/v1/analyses/MGYA00023945/file/SRR995025_FASTQ_otu.tsv	Root-Host_associated-Human-Skin
ERR1960779	MGYA00130879	https://www.ebi.ac.uk/metagenomics/api/v1/analyses/MGYA00130879/file/ERR1960779_MERGED_FASTQ_otu.tsv	Root-Host_associated-Human-Skin

SRR3217221	MGYA00412250	https://www.ebi.ac.uk/metagenomics/api/v1/analyses/MGYA00412250/file/SRR3217221_FASTQ_SSU_OTU.tsv	Root-Host_associated-Human-Skin
SRR996697	MGYA00023178	https://www.ebi.ac.uk/metagenomics/api/v1/analyses/MGYA00023178/file/SRR996697_FASTQ_otu.tsv	Root-Host_associated-Human-Skin
SRR996130	MGYA00024991	https://www.ebi.ac.uk/metagenomics/api/v1/analyses/MGYA00024991/file/SRR996130_FASTQ_otu.tsv	Root-Host_associated-Human-Skin
SRR919887	MGYA00024198	https://www.ebi.ac.uk/metagenomics/api/v1/analyses/MGYA00024198/file/SRR919887_FASTQ_otu.tsv	Root-Host_associated-Human-Skin
SRR996923	MGYA00025128	https://www.ebi.ac.uk/metagenomics/api/v1/analyses/MGYA00025128/file/SRR996923_FASTQ_otu.tsv	Root-Host_associated-Human-Skin
SRR996551	MGYA00022618	https://www.ebi.ac.uk/metagenomics/api/v1/analyses/MGYA00022618/file/SRR996551_FASTQ_otu.tsv	Root-Host_associated-Human-Skin
SRR1704319	MGYA00426974	https://www.ebi.ac.uk/metagenomics/api/v1/analyses/MGYA00426974/file/SRR1704319_FASTQ_SSU_OTU.tsv	Root-Host_associated-Human-Skin
SRR996227	MGYA00023972	https://www.ebi.ac.uk/metagenomics/api/v1/analyses/MGYA00023972/file/SRR996227_FASTQ_otu.tsv	Root-Host_associated-Human-Skin
SRR995495	MGYA00024116	https://www.ebi.ac.uk/metagenomics/api/v1/analyses/MGYA00024116/file/SRR995495_FASTQ_otu.tsv	Root-Host_associated-Human-Skin
SRR997006	MGYA00022952	https://www.ebi.ac.uk/metagenomics/api/v1/analyses/MGYA00022952/file/SRR997006_FASTQ_otu.tsv	Root-Host_associated-Human-Skin
SRR995753	MGYA00022893	https://www.ebi.ac.uk/metagenomics/api/v1/analyses/MGYA00022893/file/SRR995753_FASTQ_otu.tsv	Root-Host_associated-Human-Skin
SRR1632346	MGYA00025162	https://www.ebi.ac.uk/metagenomics/api/v1/analyses/MGYA00025162/file/SRR1632346_MERGED_FASTQ_otu.tsv	Root-Host_associated-Human-Skin

SRR920200	MGYA00023057	https://www.ebi.ac.uk/metagenomics/api/v1/analyses/MGYA00023057/file/SRR920200_FASTQ_otu.tsv	Root-Host_associated-Human-Skin
ERR442019	MGYA00385222	https://www.ebi.ac.uk/metagenomics/api/v1/analyses/MGYA00385222/file/ERR442019_FASTQ_SSU_OTU.tsv	Root-Host_associated-Human-Skin
SRR1950722	MGYA00376889	https://www.ebi.ac.uk/metagenomics/api/v1/analyses/MGYA00376889/file/SRR1950722_MERGED_FASTQ_SSU_OTU.tsv	Root-Host_associated-Human-Skin
SRR919909	MGYA00025034	https://www.ebi.ac.uk/metagenomics/api/v1/analyses/MGYA00025034/file/SRR919909_FASTQ_otu.tsv	Root-Host_associated-Human-Skin
SRR1631253	MGYA00024542	https://www.ebi.ac.uk/metagenomics/api/v1/analyses/MGYA00024542/file/SRR1631253_MERGED_FASTQ_otu.tsv	Root-Host_associated-Human-Skin
SRR5868636	MGYA00428081	https://www.ebi.ac.uk/metagenomics/api/v1/analyses/MGYA00428081/file/SRR5868636_MERGED_FASTQ_SSU_OTU.tsv	Root-Host_associated-Human-Skin
ERR1759974	MGYA00140589	https://www.ebi.ac.uk/metagenomics/api/v1/analyses/MGYA00140589/file/ERR1759974_MERGED_FASTQ_SSU_OTU.tsv	Root-Host_associated-Human-Skin
SRR1633167	MGYA00022382	https://www.ebi.ac.uk/metagenomics/api/v1/analyses/MGYA00022382/file/SRR1633167_MERGED_FASTQ_otu.tsv	Root-Host_associated-Human-Skin
ERR442902	MGYA00416339	https://www.ebi.ac.uk/metagenomics/api/v1/analyses/MGYA00416339/file/ERR442902_FASTQ_SSU_OTU.tsv	Root-Host_associated-Human-Skin
SRR920661	MGYA00022339	https://www.ebi.ac.uk/metagenomics/api/v1/analyses/MGYA00022339/file/SRR920661_FASTQ_otu.tsv	Root-Host_associated-Human-Skin
SRR996719	MGYA00022986	https://www.ebi.ac.uk/metagenomics/api/v1/analyses/MGYA00022986/file/SRR996719_FASTQ_otu.tsv	Root-Host_associated-Human-Skin
SRR920117	MGYA00024130	https://www.ebi.ac.uk/metagenomics/api/v1/analyses/MGYA00024130/file/SRR920117_FASTQ_otu.tsv	Root-Host_associated-Human-Skin

ERR443446	MGYA00419155	https://www.ebi.ac.uk/metagenomics/api/v1/analyses/MGYA00419155/file/ERR443446_FASTQ_SSU_OTU.tsv	Root-Host_associated-Human-Skin
SRR2005725	MGYA00381354	https://www.ebi.ac.uk/metagenomics/api/v1/analyses/MGYA00381354/file/SRR2005725_MERGED_FASTQ_SSU_OTU.tsv	Root-Host_associated-Human-Skin
SRR995969	MGYA00024461	https://www.ebi.ac.uk/metagenomics/api/v1/analyses/MGYA00024461/file/SRR995969_FASTQ_otu.tsv	Root-Host_associated-Human-Skin
SRR920904	MGYA00024109	https://www.ebi.ac.uk/metagenomics/api/v1/analyses/MGYA00024109/file/SRR920904_FASTQ_otu.tsv	Root-Host_associated-Human-Skin
ERR1760032	MGYA00140532	https://www.ebi.ac.uk/metagenomics/api/v1/analyses/MGYA00140532/file/ERR1760032_MERGED_FASTQ_SSU_OTU.tsv	Root-Host_associated-Human-Skin
SRR995022	MGYA00023952	https://www.ebi.ac.uk/metagenomics/api/v1/analyses/MGYA00023952/file/SRR995022_FASTQ_otu.tsv	Root-Host_associated-Human-Skin
SRR996137	MGYA00024994	https://www.ebi.ac.uk/metagenomics/api/v1/analyses/MGYA00024994/file/SRR996137_FASTQ_otu.tsv	Root-Host_associated-Human-Skin
SRR996690	MGYA00023205	https://www.ebi.ac.uk/metagenomics/api/v1/analyses/MGYA00023205/file/SRR996690_FASTQ_otu.tsv	Root-Host_associated-Human-Skin
SRR996556	MGYA00022623	https://www.ebi.ac.uk/metagenomics/api/v1/analyses/MGYA00022623/file/SRR996556_FASTQ_otu.tsv	Root-Host_associated-Human-Skin
SRR996924	MGYA00025131	https://www.ebi.ac.uk/metagenomics/api/v1/analyses/MGYA00025131/file/SRR996924_FASTQ_otu.tsv	Root-Host_associated-Human-Skin
SRR919880	MGYA00024205	https://www.ebi.ac.uk/metagenomics/api/v1/analyses/MGYA00024205/file/SRR919880_FASTQ_otu.tsv	Root-Host_associated-Human-Skin
SRR5869658	MGYA00422105	https://www.ebi.ac.uk/metagenomics/api/v1/analyses/MGYA00422105/file/SRR5869658_MERGED_FASTQ_SSU_OTU.tsv	Root-Host_associated-Human-Skin

SRR1630939	MGYA00023963	https://www.ebi.ac.uk/metagenomics/api/v1/analyses/MGYA00023963/file/SRR1630939_MERGED_FASTQ_otu.tsv	Root-Host_associated-Human-Skin
SRR996220	MGYA00023977	https://www.ebi.ac.uk/metagenomics/api/v1/analyses/MGYA00023977/file/SRR996220_FASTQ_otu.tsv	Root-Host_associated-Human-Skin
SRR5869689	MGYA00415891	https://www.ebi.ac.uk/metagenomics/api/v1/analyses/MGYA00415891/file/SRR5869689_MERGED_FASTQ_SSU_OTU.tsv	Root-Host_associated-Human-Skin
ERR443609	MGYA00414534	https://www.ebi.ac.uk/metagenomics/api/v1/analyses/MGYA00414534/file/ERR443609_FASTQ_SSU_OTU.tsv	Root-Host_associated-Human-Skin
SRR995492	MGYA00024113	https://www.ebi.ac.uk/metagenomics/api/v1/analyses/MGYA00024113/file/SRR995492_FASTQ_otu.tsv	Root-Host_associated-Human-Skin
SRR995754	MGYA00022892	https://www.ebi.ac.uk/metagenomics/api/v1/analyses/MGYA00022892/file/SRR995754_FASTQ_otu.tsv	Root-Host_associated-Human-Skin
SRR996304	MGYA00022708	https://www.ebi.ac.uk/metagenomics/api/v1/analyses/MGYA00022708/file/SRR996304_FASTQ_otu.tsv	Root-Host_associated-Human-Skin
ERR1759908	MGYA00140585	https://www.ebi.ac.uk/metagenomics/api/v1/analyses/MGYA00140585/file/ERR1759908_MERGED_FASTQ_SSU_OTU.tsv	Root-Host_associated-Human-Skin
SRR994451	MGYA00022457	https://www.ebi.ac.uk/metagenomics/api/v1/analyses/MGYA00022457/file/SRR994451_FASTQ_otu.tsv	Root-Host_associated-Human-Skin
ERR442065	MGYA00385197	https://www.ebi.ac.uk/metagenomics/api/v1/analyses/MGYA00385197/file/ERR442065_FASTQ_SSU_OTU.tsv	Root-Host_associated-Human-Skin
SRR996765	MGYA00023862	https://www.ebi.ac.uk/metagenomics/api/v1/analyses/MGYA00023862/file/SRR996765_FASTQ_otu.tsv	Root-Host_associated-Human-Skin
ERR441963	MGYA00385236	https://www.ebi.ac.uk/metagenomics/api/v1/analyses/MGYA00385236/file/ERR441963_FASTQ_SSU_OTU.tsv	Root-Host_associated-Human-Skin

SRR994797	MGYA00022831	https://www.ebi.ac.uk/metagenomics/api/v1/analyses/MGYA00022831/file/SRR994797_FASTQ_otu.tsv	Root-Host_associated-Human-Skin
SRR995106	MGYA00023668	https://www.ebi.ac.uk/metagenomics/api/v1/analyses/MGYA00023668/file/SRR995106_FASTQ_otu.tsv	Root-Host_associated-Human-Skin
ERR1760116	MGYA00140538	https://www.ebi.ac.uk/metagenomics/api/v1/analyses/MGYA00140538/file/ERR1760116_MERGED_FASTQ_SSU_OTU.tsv	Root-Host_associated-Human-Skin
ERR451419	MGYA00414268	https://www.ebi.ac.uk/metagenomics/api/v1/analyses/MGYA00414268/file/ERR451419_FASTQ_SSU_OTU.tsv	Root-Host_associated-Human-Skin
SRR919610	MGYA00023526	https://www.ebi.ac.uk/metagenomics/api/v1/analyses/MGYA00023526/file/SRR919610_FASTQ_otu.tsv	Root-Host_associated-Human-Skin
SRR995567	MGYA00022985	https://www.ebi.ac.uk/metagenomics/api/v1/analyses/MGYA00022985/file/SRR995567_FASTQ_otu.tsv	Root-Host_associated-Human-Skin
SRR994480	MGYA00023133	https://www.ebi.ac.uk/metagenomics/api/v1/analyses/MGYA00023133/file/SRR994480_FASTQ_otu.tsv	Root-Host_associated-Human-Skin
SRR920978	MGYA00023035	https://www.ebi.ac.uk/metagenomics/api/v1/analyses/MGYA00023035/file/SRR920978_FASTQ_otu.tsv	Root-Host_associated-Human-Skin
ERR2538486	MGYA00381493	https://www.ebi.ac.uk/metagenomics/api/v1/analyses/MGYA00381493/file/ERR2538486_FASTQ_SSU_OTU.tsv	Root-Host_associated-Human-Skin
SRR1622855	MGYA00024382	https://www.ebi.ac.uk/metagenomics/api/v1/analyses/MGYA00024382/file/SRR1622855_MERGED_FASTQ_otu.tsv	Root-Host_associated-Human-Skin
SRR6757676	MGYA00380789	https://www.ebi.ac.uk/metagenomics/api/v1/analyses/MGYA00380789/file/SRR6757676_MERGED_FASTQ_SSU_OTU.tsv	Root-Host_associated-Human-Skin
SRR1631060	MGYA00024812	https://www.ebi.ac.uk/metagenomics/api/v1/analyses/MGYA00024812/file/SRR1631060_MERGED_FASTQ_otu.tsv	Root-Host_associated-Human-Skin

SRR1704703	MGYA00424367	https://www.ebi.ac.uk/metagenomics/api/v1/analyses/MGYA00424367/file/SRR1704703_FASTQ_SSU_OTU.tsv	Root-Host_associated-Human-Skin
ERR1960702	MGYA00130745	https://www.ebi.ac.uk/metagenomics/api/v1/analyses/MGYA00130745/file/ERR1960702_MERGED_FASTQ_otu.tsv	Root-Host_associated-Human-Skin
ERR451541	MGYA00428664	https://www.ebi.ac.uk/metagenomics/api/v1/analyses/MGYA00428664/file/ERR451541_FASTQ_SSU_OTU.tsv	Root-Host_associated-Human-Skin
SRR1631401	MGYA00022432	https://www.ebi.ac.uk/metagenomics/api/v1/analyses/MGYA00022432/file/SRR1631401_MERGED_FASTQ_otu.tsv	Root-Host_associated-Human-Skin
SRR1630994	MGYA00022854	https://www.ebi.ac.uk/metagenomics/api/v1/analyses/MGYA00022854/file/SRR1630994_MERGED_FASTQ_otu.tsv	Root-Host_associated-Human-Skin
SRR995398	MGYA00024753	https://www.ebi.ac.uk/metagenomics/api/v1/analyses/MGYA00024753/file/SRR995398_FASTQ_otu.tsv	Root-Host_associated-Human-Skin
ERR443103	MGYA00413701	https://www.ebi.ac.uk/metagenomics/api/v1/analyses/MGYA00413701/file/ERR443103_FASTQ_SSU_OTU.tsv	Root-Host_associated-Human-Skin
SRR994509	MGYA00023823	https://www.ebi.ac.uk/metagenomics/api/v1/analyses/MGYA00023823/file/SRR994509_FASTQ_otu.tsv	Root-Host_associated-Human-Skin
SRR995349	MGYA00023127	https://www.ebi.ac.uk/metagenomics/api/v1/analyses/MGYA00023127/file/SRR995349_FASTQ_otu.tsv	Root-Host_associated-Human-Skin
SRR1923892	MGYA00028370	https://www.ebi.ac.uk/metagenomics/api/v1/analyses/MGYA00028370/file/SRR1923892_FASTQ_otu.tsv	Root-Host_associated-Human-Skin
SRR996989	MGYA00024528	https://www.ebi.ac.uk/metagenomics/api/v1/analyses/MGYA00024528/file/SRR996989_FASTQ_otu.tsv	Root-Host_associated-Human-Skin
SRR5869152	MGYA00417009	https://www.ebi.ac.uk/metagenomics/api/v1/analyses/MGYA00417009/file/SRR5869152_MERGED_FASTQ_SSU_OTU.tsv	Root-Host_associated-Human-Skin

SRR5868712	MGYA00423494	https://www.ebi.ac.uk/metagenomics/api/v1/analyses/MGYA00423494/file/SRR5868712_MERGED_FASTQ_SSU_OTU.tsv	Root-Host_associated-Human-Skin
SRR921105	MGYA00024999	https://www.ebi.ac.uk/metagenomics/api/v1/analyses/MGYA00024999/file/SRR921105_FASTQ_otu.tsv	Root-Host_associated-Human-Skin
SRR920745	MGYA00023541	https://www.ebi.ac.uk/metagenomics/api/v1/analyses/MGYA00023541/file/SRR920745_FASTQ_otu.tsv	Root-Host_associated-Human-Skin
ERR1701270	MGYA00084242	https://www.ebi.ac.uk/metagenomics/api/v1/analyses/MGYA00084242/file/ERR1701270_FASTQ_otu.tsv	Root-Host_associated-Human-Skin
SRR1631716	MGYA00024037	https://www.ebi.ac.uk/metagenomics/api/v1/analyses/MGYA00024037/file/SRR1631716_MERGED_FASTQ_otu.tsv	Root-Host_associated-Human-Skin
SRR5869294	MGYA00418756	https://www.ebi.ac.uk/metagenomics/api/v1/analyses/MGYA00418756/file/SRR5869294_MERGED_FASTQ_SSU_OTU.tsv	Root-Host_associated-Human-Skin
SRR5869941	MGYA00427108	https://www.ebi.ac.uk/metagenomics/api/v1/analyses/MGYA00427108/file/SRR5869941_MERGED_FASTQ_SSU_OTU.tsv	Root-Host_associated-Human-Skin
ERR2538457	MGYA00381464	https://www.ebi.ac.uk/metagenomics/api/v1/analyses/MGYA00381464/file/ERR2538457_FASTQ_SSU_OTU.tsv	Root-Host_associated-Human-Skin
SRR5869078	MGYA00416486	https://www.ebi.ac.uk/metagenomics/api/v1/analyses/MGYA00416486/file/SRR5869078_MERGED_FASTQ_SSU_OTU.tsv	Root-Host_associated-Human-Skin
SRR919907	MGYA00025025	https://www.ebi.ac.uk/metagenomics/api/v1/analyses/MGYA00025025/file/SRR919907_FASTQ_otu.tsv	Root-Host_associated-Human-Skin
SRR919575	MGYA00024728	https://www.ebi.ac.uk/metagenomics/api/v1/analyses/MGYA00024728/file/SRR919575_FASTQ_otu.tsv	Root-Host_associated-Human-Skin
ERR442017	MGYA00385223	https://www.ebi.ac.uk/metagenomics/api/v1/analyses/MGYA00385223/file/ERR442017_FASTQ_SSU_OTU.tsv	Root-Host_associated-Human-Skin

SRR1632348	MGYA00023662	https://www.ebi.ac.uk/metagenomics/api/v1/analyses/MGYA00023662/file/SRR1632348_MERGED_FASTQ_otu.tsv	Root-Host_associated-Human-Skin
ERR1551332	MGYA00069423	https://www.ebi.ac.uk/metagenomics/api/v1/analyses/MGYA00069423/file/ERR1551332_MERGED_FASTQ_otu.tsv	Root-Host_associated-Human-Skin
SRR1633169	MGYA00022378	https://www.ebi.ac.uk/metagenomics/api/v1/analyses/MGYA00022378/file/SRR1633169_MERGED_FASTQ_otu.tsv	Root-Host_associated-Human-Skin
SRR995602	MGYA00025043	https://www.ebi.ac.uk/metagenomics/api/v1/analyses/MGYA00025043/file/SRR995602_FASTQ_otu.tsv	Root-Host_associated-Human-Skin
SRR1624842	MGYA00024289	https://www.ebi.ac.uk/metagenomics/api/v1/analyses/MGYA00024289/file/SRR1624842_MERGED_FASTQ_otu.tsv	Root-Host_associated-Human-Skin
SRR920119	MGYA00024132	https://www.ebi.ac.uk/metagenomics/api/v1/analyses/MGYA00024132/file/SRR920119_FASTQ_otu.tsv	Root-Host_associated-Human-Skin
SRR996061	MGYA00023315	https://www.ebi.ac.uk/metagenomics/api/v1/analyses/MGYA00023315/file/SRR996061_FASTQ_otu.tsv	Root-Host_associated-Human-Skin
SRR994880	MGYA00022186	https://www.ebi.ac.uk/metagenomics/api/v1/analyses/MGYA00022186/file/SRR994880_FASTQ_otu.tsv	Root-Host_associated-Human-Skin
SRR995967	MGYA00023849	https://www.ebi.ac.uk/metagenomics/api/v1/analyses/MGYA00023849/file/SRR995967_FASTQ_otu.tsv	Root-Host_associated-Human-Skin
SRR996872	MGYA00023439	https://www.ebi.ac.uk/metagenomics/api/v1/analyses/MGYA00023439/file/SRR996872_FASTQ_otu.tsv	Root-Host_associated-Human-Skin
SRR1623215	MGYA00023914	https://www.ebi.ac.uk/metagenomics/api/v1/analyses/MGYA00023914/file/SRR1623215_MERGED_FASTQ_otu.tsv	Root-Host_associated-Human-Skin
SRR6757604	MGYA00380723	https://www.ebi.ac.uk/metagenomics/api/v1/analyses/MGYA00380723/file/SRR6757604_MERGED_FASTQ_SSU_OTU.tsv	Root-Host_associated-Human-Skin

SRR996139	MGYA00024986	https://www.ebi.ac.uk/metagenomics/api/v1/analyses/MGYA00024986/file/SRR996139_FASTQ_otu.tsv	Root-Host_associated-Human-Skin
ERR2538353	MGYA00381360	https://www.ebi.ac.uk/metagenomics/api/v1/analyses/MGYA00381360/file/ERR2538353_FASTQ_SSU_OTU.tsv	Root-Host_associated-Human-Skin
SRR1923785	MGYA00028232	https://www.ebi.ac.uk/metagenomics/api/v1/analyses/MGYA00028232/file/SRR1923785_FASTQ_otu.tsv	Root-Host_associated-Human-Skin
ERR451533	MGYA00428669	https://www.ebi.ac.uk/metagenomics/api/v1/analyses/MGYA00428669/file/ERR451533_FASTQ_SSU_OTU.tsv	Root-Host_associated-Human-Skin
ERR451294	MGYA00426044	https://www.ebi.ac.uk/metagenomics/api/v1/analyses/MGYA00426044/file/ERR451294_FASTQ_SSU_OTU.tsv	Root-Host_associated-Human-Skin
SRR3217228	MGYA00412170	https://www.ebi.ac.uk/metagenomics/api/v1/analyses/MGYA00412170/file/SRR3217228_FASTQ_SSU_OTU.tsv	Root-Host_associated-Human-Skin
ERR1960770	MGYA00130709	https://www.ebi.ac.uk/metagenomics/api/v1/analyses/MGYA00130709/file/ERR1960770_MERGED_FASTQ_otu.tsv	Root-Host_associated-Human-Skin
SRR996558	MGYA00022625	https://www.ebi.ac.uk/metagenomics/api/v1/analyses/MGYA00022625/file/SRR996558_FASTQ_otu.tsv	Root-Host_associated-Human-Skin
SRR2005673	MGYA00381330	https://www.ebi.ac.uk/metagenomics/api/v1/analyses/MGYA00381330/file/SRR2005673_MERGED_FASTQ_SSU_OTU.tsv	Root-Host_associated-Human-Skin
SRR5869656	MGYA00416997	https://www.ebi.ac.uk/metagenomics/api/v1/analyses/MGYA00416997/file/SRR5869656_MERGED_FASTQ_SSU_OTU.tsv	Root-Host_associated-Human-Skin
ERR443171	MGYA00412634	https://www.ebi.ac.uk/metagenomics/api/v1/analyses/MGYA00412634/file/ERR443171_FASTQ_SSU_OTU.tsv	Root-Host_associated-Human-Skin
ERR1960815	MGYA00130850	https://www.ebi.ac.uk/metagenomics/api/v1/analyses/MGYA00130850/file/ERR1960815_MERGED_FASTQ_otu.tsv	Root-Host_associated-Human-Skin

ERR443607	MGYA00415523	https://www.ebi.ac.uk/metagenomics/api/v1/analyses/MGYA00415523/file/ERR443607_FASTQ_SSU_OTU.tsv	Root-Host_associated-Human-Skin
SRR1704466	MGYA00413396	https://www.ebi.ac.uk/metagenomics/api/v1/analyses/MGYA00413396/file/SRR1704466_FASTQ_SSU_OTU.tsv	Root-Host_associated-Human-Skin
SRR1631305	MGYA00023229	https://www.ebi.ac.uk/metagenomics/api/v1/analyses/MGYA00023229/file/SRR1631305_MERGED_FASTQ_otu.tsv	Root-Host_associated-Human-Skin
SRR1630937	MGYA00023965	https://www.ebi.ac.uk/metagenomics/api/v1/analyses/MGYA00023965/file/SRR1630937_MERGED_FASTQ_otu.tsv	Root-Host_associated-Human-Skin
SRR5869687	MGYA00428043	https://www.ebi.ac.uk/metagenomics/api/v1/analyses/MGYA00428043/file/SRR5869687_MERGED_FASTQ_SSU_OTU.tsv	Root-Host_associated-Human-Skin
SRR920737	MGYA00025019	https://www.ebi.ac.uk/metagenomics/api/v1/analyses/MGYA00025019/file/SRR920737_FASTQ_otu.tsv	Root-Host_associated-Human-Skin
SRR1633031	MGYA00024156	https://www.ebi.ac.uk/metagenomics/api/v1/analyses/MGYA00024156/file/SRR1633031_MERGED_FASTQ_otu.tsv	Root-Host_associated-Human-Skin
ERR2538425	MGYA00381432	https://www.ebi.ac.uk/metagenomics/api/v1/analyses/MGYA00381432/file/ERR2538425_FASTQ_SSU_OTU.tsv	Root-Host_associated-Human-Skin
ERR2538382	MGYA00381389	https://www.ebi.ac.uk/metagenomics/api/v1/analyses/MGYA00381389/file/ERR2538382_FASTQ_SSU_OTU.tsv	Root-Host_associated-Human-Skin
ERR443723	MGYA00414528	https://www.ebi.ac.uk/metagenomics/api/v1/analyses/MGYA00414528/file/ERR443723_FASTQ_SSU_OTU.tsv	Root-Host_associated-Human-Skin
ERR443084	MGYA00415553	https://www.ebi.ac.uk/metagenomics/api/v1/analyses/MGYA00415553/file/ERR443084_FASTQ_SSU_OTU.tsv	Root-Host_associated-Human-Skin
SRR1632334	MGYA00024583	https://www.ebi.ac.uk/metagenomics/api/v1/analyses/MGYA00024583/file/SRR1632334_MERGED_FASTQ_otu.tsv	Root-Host_associated-Human-Skin

ERR1759906	MGYA00140436	https://www.ebi.ac.uk/metagenomics/api/v1/analyses/MGYA00140436/file/ERR1759906_MERGED_FASTQ_SSU_OTU.tsv	Root-Host_associated-Human-Skin
SRR5868644	MGYA00421226	https://www.ebi.ac.uk/metagenomics/api/v1/analyses/MGYA00421226/file/SRR5868644_MERGED_FASTQ_SSU_OTU.tsv	Root-Host_associated-Human-Skin
SRR1950750	MGYA00376917	https://www.ebi.ac.uk/metagenomics/api/v1/analyses/MGYA00376917/file/SRR1950750_MERGED_FASTQ_SSU_OTU.tsv	Root-Host_associated-Human-Skin
ERR1960685	MGYA00130863	https://www.ebi.ac.uk/metagenomics/api/v1/analyses/MGYA00130863/file/ERR1960685_MERGED_FASTQ_otu.tsv	Root-Host_associated-Human-Skin
ERR450953	MGYA00416327	https://www.ebi.ac.uk/metagenomics/api/v1/analyses/MGYA00416327/file/ERR450953_FASTQ_SSU_OTU.tsv	Root-Host_associated-Human-Skin
ERR443342	MGYA00421417	https://www.ebi.ac.uk/metagenomics/api/v1/analyses/MGYA00421417/file/ERR443342_FASTQ_SSU_OTU.tsv	Root-Host_associated-Human-Skin
SRR994799	MGYA00022825	https://www.ebi.ac.uk/metagenomics/api/v1/analyses/MGYA00022825/file/SRR994799_FASTQ_otu.tsv	Root-Host_associated-Human-Skin
ERR442970	MGYA00426808	https://www.ebi.ac.uk/metagenomics/api/v1/analyses/MGYA00426808/file/ERR442970_FASTQ_SSU_OTU.tsv	Root-Host_associated-Human-Skin
SRR921053	MGYA00022861	https://www.ebi.ac.uk/metagenomics/api/v1/analyses/MGYA00022861/file/SRR921053_FASTQ_otu.tsv	Root-Host_associated-Human-Skin
ERR2538501	MGYA00381508	https://www.ebi.ac.uk/metagenomics/api/v1/analyses/MGYA00381508/file/ERR2538501_FASTQ_SSU_OTU.tsv	Root-Host_associated-Human-Skin
SRR1622903	MGYA00022366	https://www.ebi.ac.uk/metagenomics/api/v1/analyses/MGYA00022366/file/SRR1622903_MERGED_FASTQ_otu.tsv	Root-Host_associated-Human-Skin
ERR1760118	MGYA00140576	https://www.ebi.ac.uk/metagenomics/api/v1/analyses/MGYA00140576/file/ERR1760118_MERGED_FASTQ_SSU_OTU.tsv	Root-Host_associated-Human-Skin

SRR995108	MGYA00023660	https://www.ebi.ac.uk/metagenomics/api/v1/analyses/MGYA00023660/file/SRR995108_FASTQ_otu.tsv	Root-Host_associated-Human-Skin
ERR1960654	MGYA00130872	https://www.ebi.ac.uk/metagenomics/api/v1/analyses/MGYA00130872/file/ERR1960654_MERGED_FASTQ_otu.tsv	Root-Host_associated-Human-Skin
ERR443434	MGYA00413673	https://www.ebi.ac.uk/metagenomics/api/v1/analyses/MGYA00413673/file/ERR443434_FASTQ_SSU_OTU.tsv	Root-Host_associated-Human-Skin
SRR1950781	MGYA00376948	https://www.ebi.ac.uk/metagenomics/api/v1/analyses/MGYA00376948/file/SRR1950781_MERGED_FASTQ_SSU_OTU.tsv	Root-Host_associated-Human-Skin
SRR1631557	MGYA00023798	https://www.ebi.ac.uk/metagenomics/api/v1/analyses/MGYA00023798/file/SRR1631557_MERGED_FASTQ_otu.tsv	Root-Host_associated-Human-Skin
SRR1704234	MGYA00413109	https://www.ebi.ac.uk/metagenomics/api/v1/analyses/MGYA00413109/file/SRR1704234_FASTQ_SSU_OTU.tsv	Root-Host_associated-Human-Skin
SRR1632830	MGYA00022818	https://www.ebi.ac.uk/metagenomics/api/v1/analyses/MGYA00022818/file/SRR1632830_MERGED_FASTQ_otu.tsv	Root-Host_associated-Human-Skin
SRR920976	MGYA00023041	https://www.ebi.ac.uk/metagenomics/api/v1/analyses/MGYA00023041/file/SRR920976_FASTQ_otu.tsv	Root-Host_associated-Human-Skin
SRR1923915	MGYA00028295	https://www.ebi.ac.uk/metagenomics/api/v1/analyses/MGYA00028295/file/SRR1923915_FASTQ_otu.tsv	Root-Host_associated-Human-Skin
SRR995569	MGYA00022977	https://www.ebi.ac.uk/metagenomics/api/v1/analyses/MGYA00022977/file/SRR995569_FASTQ_otu.tsv	Root-Host_associated-Human-Skin
ERR1760040	MGYA00140579	https://www.ebi.ac.uk/metagenomics/api/v1/analyses/MGYA00140579/file/ERR1760040_MERGED_FASTQ_SSU_OTU.tsv	Root-Host_associated-Human-Skin
SRR994610	MGYA00023013	https://www.ebi.ac.uk/metagenomics/api/v1/analyses/MGYA00023013/file/SRR994610_FASTQ_otu.tsv	Root-Host_associated-Human-Skin

SRR5870020	MGYA00416426	https://www.ebi.ac.uk/metagenomics/api/v1/analyses/MGYA00416426/file/SRR5870020_MERGED_FASTQ_SSU_OTU.tsv	Root-Host_associated-Human-Skin
SRR6757678	MGYA00380791	https://www.ebi.ac.uk/metagenomics/api/v1/analyses/MGYA00380791/file/SRR6757678_MERGED_FASTQ_SSU_OTU.tsv	Root-Host_associated-Human-Skin
ERR2538488	MGYA00381495	https://www.ebi.ac.uk/metagenomics/api/v1/analyses/MGYA00381495/file/ERR2538488_FASTQ_SSU_OTU.tsv	Root-Host_associated-Human-Skin
SRR996145	MGYA00022530	https://www.ebi.ac.uk/metagenomics/api/v1/analyses/MGYA00022530/file/SRR996145_FASTQ_otu.tsv	Root-Host_associated-Human-Skin
SRR995396	MGYA00024755	https://www.ebi.ac.uk/metagenomics/api/v1/analyses/MGYA00024755/file/SRR995396_FASTQ_otu.tsv	Root-Host_associated-Human-Skin
SRR996283	MGYA00023311	https://www.ebi.ac.uk/metagenomics/api/v1/analyses/MGYA00023311/file/SRR996283_FASTQ_otu.tsv	Root-Host_associated-Human-Skin
SRR996956	MGYA00022872	https://www.ebi.ac.uk/metagenomics/api/v1/analyses/MGYA00022872/file/SRR996956_FASTQ_otu.tsv	Root-Host_associated-Human-Skin
SRR996987	MGYA00024530	https://www.ebi.ac.uk/metagenomics/api/v1/analyses/MGYA00024530/file/SRR996987_FASTQ_otu.tsv	Root-Host_associated-Human-Skin
SRR995347	MGYA00023121	https://www.ebi.ac.uk/metagenomics/api/v1/analyses/MGYA00023121/file/SRR995347_FASTQ_otu.tsv	Root-Host_associated-Human-Skin
SRR994975	MGYA00024555	https://www.ebi.ac.uk/metagenomics/api/v1/analyses/MGYA00024555/file/SRR994975_FASTQ_otu.tsv	Root-Host_associated-Human-Skin
SRR994507	MGYA00023821	https://www.ebi.ac.uk/metagenomics/api/v1/analyses/MGYA00023821/file/SRR994507_FASTQ_otu.tsv	Root-Host_associated-Human-Skin
ERR981254	MGYA00010241	https://www.ebi.ac.uk/metagenomics/api/v1/analyses/MGYA00010241/file/ERR981254_MERGED_FASTQ_otu.tsv	Root-Host_associated-Human-Skin

ERR2538459	MGYA00381466	https://www.ebi.ac.uk/metagenomics/api/v1/analyses/MGYA00381466/file/ERR2538459_FASTQ_SSU_OTU.tsv	Root-Host_associated-Human-Skin
SRR997073	MGYA00023161	https://www.ebi.ac.uk/metagenomics/api/v1/analyses/MGYA00023161/file/SRR997073_FASTQ_otu.tsv	Root-Host_associated-Human-Skin
SRR1631718	MGYA00024043	https://www.ebi.ac.uk/metagenomics/api/v1/analyses/MGYA00024043/file/SRR1631718_MERGED_FASTQ_otu.tsv	Root-Host_associated-Human-Skin
SRR995081	MGYA00022718	https://www.ebi.ac.uk/metagenomics/api/v1/analyses/MGYA00022718/file/SRR995081_FASTQ_otu.tsv	Root-Host_associated-Human-Skin
ERR1760091	MGYA00140492	https://www.ebi.ac.uk/metagenomics/api/v1/analyses/MGYA00140492/file/ERR1760091_MERGED_FASTQ_SSU_OTU.tsv	Root-Host_associated-Human-Skin
ERR443083	MGYA00420486	https://www.ebi.ac.uk/metagenomics/api/v1/analyses/MGYA00420486/file/ERR443083_FASTQ_SSU_OTU.tsv	Root-Host_associated-Human-Skin
SRR1632333	MGYA00024568	https://www.ebi.ac.uk/metagenomics/api/v1/analyses/MGYA00024568/file/SRR1632333_MERGED_FASTQ_otu.tsv	Root-Host_associated-Human-Skin
SRR994458	MGYA00022464	https://www.ebi.ac.uk/metagenomics/api/v1/analyses/MGYA00022464/file/SRR994458_FASTQ_otu.tsv	Root-Host_associated-Human-Skin
SRR1950757	MGYA00376924	https://www.ebi.ac.uk/metagenomics/api/v1/analyses/MGYA00376924/file/SRR1950757_MERGED_FASTQ_SSU_OTU.tsv	Root-Host_associated-Human-Skin
SRR5868643	MGYA00423519	https://www.ebi.ac.uk/metagenomics/api/v1/analyses/MGYA00423519/file/SRR5868643_MERGED_FASTQ_SSU_OTU.tsv	Root-Host_associated-Human-Skin
SRR1704545	MGYA00425322	https://www.ebi.ac.uk/metagenomics/api/v1/analyses/MGYA00425322/file/SRR1704545_FASTQ_SSU_OTU.tsv	Root-Host_associated-Human-Skin
ERR1759901	MGYA00140592	https://www.ebi.ac.uk/metagenomics/api/v1/analyses/MGYA00140592/file/ERR1759901_MERGED_FASTQ_SSU_OTU.tsv	Root-Host_associated-Human-Skin

ERR442977	MGYA00420165	https://www.ebi.ac.uk/metagenomics/api/v1/analyses/MGYA00420165/file/ERR442977_FASTQ_SSU_OTU.tsv	Root-Host_associated-Human-Skin
SRR921054	MGYA00022860	https://www.ebi.ac.uk/metagenomics/api/v1/analyses/MGYA00022860/file/SRR921054_FASTQ_otu.tsv	Root-Host_associated-Human-Skin
ERR1960682	MGYA00130779	https://www.ebi.ac.uk/metagenomics/api/v1/analyses/MGYA00130779/file/ERR1960682_MERGED_FASTQ_otu.tsv	Root-Host_associated-Human-Skin
SRR1704683	MGYA00420587	https://www.ebi.ac.uk/metagenomics/api/v1/analyses/MGYA00420587/file/SRR1704683_FASTQ_SSU_OTU.tsv	Root-Host_associated-Human-Skin
ERR2538506	MGYA00381513	https://www.ebi.ac.uk/metagenomics/api/v1/analyses/MGYA00381513/file/ERR2538506_FASTQ_SSU_OTU.tsv	Root-Host_associated-Human-Skin
SRR1622904	MGYA00022353	https://www.ebi.ac.uk/metagenomics/api/v1/analyses/MGYA00022353/file/SRR1622904_MERGED_FASTQ_otu.tsv	Root-Host_associated-Human-Skin
SRR919619	MGYA00023533	https://www.ebi.ac.uk/metagenomics/api/v1/analyses/MGYA00023533/file/SRR919619_FASTQ_otu.tsv	Root-Host_associated-Human-Skin
ERR443433	MGYA00412978	https://www.ebi.ac.uk/metagenomics/api/v1/analyses/MGYA00412978/file/ERR443433_FASTQ_SSU_OTU.tsv	Root-Host_associated-Human-Skin
ERR1960653	MGYA00130786	https://www.ebi.ac.uk/metagenomics/api/v1/analyses/MGYA00130786/file/ERR1960653_MERGED_FASTQ_otu.tsv	Root-Host_associated-Human-Skin
SRR5868692	MGYA00420810	https://www.ebi.ac.uk/metagenomics/api/v1/analyses/MGYA00420810/file/SRR5868692_MERGED_FASTQ_SSU_OTU.tsv	Root-Host_associated-Human-Skin
SRR1631550	MGYA00023803	https://www.ebi.ac.uk/metagenomics/api/v1/analyses/MGYA00023803/file/SRR1631550_MERGED_FASTQ_otu.tsv	Root-Host_associated-Human-Skin
SRR1950786	MGYA00376953	https://www.ebi.ac.uk/metagenomics/api/v1/analyses/MGYA00376953/file/SRR1950786_MERGED_FASTQ_SSU_OTU.tsv	Root-Host_associated-Human-Skin

SRR1923912	MGYA00028300	https://www.ebi.ac.uk/metagenomics/api/v1/analyses/MGYA00028300/file/SRR1923912_FASTQ_otu.tsv	Root-Host_associated-Human-Skin
SRR920971	MGYA00023038	https://www.ebi.ac.uk/metagenomics/api/v1/analyses/MGYA00023038/file/SRR920971_FASTQ_otu.tsv	Root-Host_associated-Human-Skin
SRR1632837	MGYA00022817	https://www.ebi.ac.uk/metagenomics/api/v1/analyses/MGYA00022817/file/SRR1632837_MERGED_FASTQ_otu.tsv	Root-Host_associated-Human-Skin
SRR994489	MGYA00024344	https://www.ebi.ac.uk/metagenomics/api/v1/analyses/MGYA00024344/file/SRR994489_FASTQ_otu.tsv	Root-Host_associated-Human-Skin
SRR994617	MGYA00023018	https://www.ebi.ac.uk/metagenomics/api/v1/analyses/MGYA00023018/file/SRR994617_FASTQ_otu.tsv	Root-Host_associated-Human-Skin
ERR451548	MGYA00425750	https://www.ebi.ac.uk/metagenomics/api/v1/analyses/MGYA00425750/file/ERR451548_FASTQ_SSU_OTU.tsv	Root-Host_associated-Human-Skin
ERR1760047	MGYA00140588	https://www.ebi.ac.uk/metagenomics/api/v1/analyses/MGYA00140588/file/ERR1760047_MERGED_FASTQ_SSU_OTU.tsv	Root-Host_associated-Human-Skin
SRR996142	MGYA00022525	https://www.ebi.ac.uk/metagenomics/api/v1/analyses/MGYA00022525/file/SRR996142_FASTQ_otu.tsv	Root-Host_associated-Human-Skin
ERR451129	MGYA00416303	https://www.ebi.ac.uk/metagenomics/api/v1/analyses/MGYA00416303/file/ERR451129_FASTQ_SSU_OTU.tsv	Root-Host_associated-Human-Skin
SRR995391	MGYA00024760	https://www.ebi.ac.uk/metagenomics/api/v1/analyses/MGYA00024760/file/SRR995391_FASTQ_otu.tsv	Root-Host_associated-Human-Skin
ERR450769	MGYA00416731	https://www.ebi.ac.uk/metagenomics/api/v1/analyses/MGYA00416731/file/ERR450769_FASTQ_SSU_OTU.tsv	Root-Host_associated-Human-Skin
ERR1759888	MGYA00140488	https://www.ebi.ac.uk/metagenomics/api/v1/analyses/MGYA00140488/file/ERR1759888_MERGED_FASTQ_SSU_OTU.tsv	Root-Host_associated-Human-Skin

SRR996951	MGYA00022875	https://www.ebi.ac.uk/metagenomics/api/v1/analyses/MGYA00022875/file/SRR996951_FASTQ_otu.tsv	Root-Host_associated-Human-Skin
SRR996284	MGYA00023304	https://www.ebi.ac.uk/metagenomics/api/v1/analyses/MGYA00023304/file/SRR996284_FASTQ_otu.tsv	Root-Host_associated-Human-Skin
SRR996980	MGYA00024537	https://www.ebi.ac.uk/metagenomics/api/v1/analyses/MGYA00024537/file/SRR996980_FASTQ_otu.tsv	Root-Host_associated-Human-Skin
SRR994500	MGYA00023816	https://www.ebi.ac.uk/metagenomics/api/v1/analyses/MGYA00023816/file/SRR994500_FASTQ_otu.tsv	Root-Host_associated-Human-Skin
SRR994972	MGYA00024552	https://www.ebi.ac.uk/metagenomics/api/v1/analyses/MGYA00024552/file/SRR994972_FASTQ_otu.tsv	Root-Host_associated-Human-Skin
SRR995340	MGYA00023122	https://www.ebi.ac.uk/metagenomics/api/v1/analyses/MGYA00023122/file/SRR995340_FASTQ_otu.tsv	Root-Host_associated-Human-Skin
SRR997074	MGYA00023162	https://www.ebi.ac.uk/metagenomics/api/v1/analyses/MGYA00023162/file/SRR997074_FASTQ_otu.tsv	Root-Host_associated-Human-Skin
ERR981253	MGYA00010234	https://www.ebi.ac.uk/metagenomics/api/v1/analyses/MGYA00010234/file/ERR981253_MERGED_FASTQ_otu.tsv	Root-Host_associated-Human-Skin
SRR995086	MGYA00022721	https://www.ebi.ac.uk/metagenomics/api/v1/analyses/MGYA00022721/file/SRR995086_FASTQ_otu.tsv	Root-Host_associated-Human-Skin
ERR1760096	MGYA00140475	https://www.ebi.ac.uk/metagenomics/api/v1/analyses/MGYA00140475/file/ERR1760096_MERGED_FASTQ_SSU_OTU.tsv	Root-Host_associated-Human-Skin
ERR1701279	MGYA00084289	https://www.ebi.ac.uk/metagenomics/api/v1/analyses/MGYA00084289/file/ERR1701279_FASTQ_otu.tsv	Root-Host_associated-Human-Skin
SRR919572	MGYA00024723	https://www.ebi.ac.uk/metagenomics/api/v1/analyses/MGYA00024723/file/SRR919572_FASTQ_otu.tsv	Root-Host_associated-Human-Skin

SRR919900	MGYA00025030	https://www.ebi.ac.uk/metagenomics/api/v1/analyses/MGYA00025030/file/SRR919900_FASTQ_otu.tsv	Root-Host_associated-Human-Skin
SRR920209	MGYA00023054	https://www.ebi.ac.uk/metagenomics/api/v1/analyses/MGYA00023054/file/SRR920209_FASTQ_otu.tsv	Root-Host_associated-Human-Skin
SRR994424	MGYA00024964	https://www.ebi.ac.uk/metagenomics/api/v1/analyses/MGYA00024964/file/SRR994424_FASTQ_otu.tsv	Root-Host_associated-Human-Skin
ERR443758	MGYA00415493	https://www.ebi.ac.uk/metagenomics/api/v1/analyses/MGYA00415493/file/ERR443758_FASTQ_SSU_OTU.tsv	Root-Host_associated-Human-Skin
ERR442010	MGYA00385225	https://www.ebi.ac.uk/metagenomics/api/v1/analyses/MGYA00385225/file/ERR442010_FASTQ_SSU_OTU.tsv	Root-Host_associated-Human-Skin
SRR996710	MGYA00022996	https://www.ebi.ac.uk/metagenomics/api/v1/analyses/MGYA00022996/file/SRR996710_FASTQ_otu.tsv	Root-Host_associated-Human-Skin
SRR1624845	MGYA00024296	https://www.ebi.ac.uk/metagenomics/api/v1/analyses/MGYA00024296/file/SRR1624845_MERGED_FASTQ_otu.tsv	Root-Host_associated-Human-Skin
SRR995605	MGYA00025036	https://www.ebi.ac.uk/metagenomics/api/v1/analyses/MGYA00025036/file/SRR995605_FASTQ_otu.tsv	Root-Host_associated-Human-Skin
SRR920668	MGYA00022348	https://www.ebi.ac.uk/metagenomics/api/v1/analyses/MGYA00022348/file/SRR920668_FASTQ_otu.tsv	Root-Host_associated-Human-Skin
SRR994733	MGYA00023892	https://www.ebi.ac.uk/metagenomics/api/v1/analyses/MGYA00023892/file/SRR994733_FASTQ_otu.tsv	Root-Host_associated-Human-Skin
SRR996066	MGYA00023320	https://www.ebi.ac.uk/metagenomics/api/v1/analyses/MGYA00023320/file/SRR996066_FASTQ_otu.tsv	Root-Host_associated-Human-Skin
SRR995960	MGYA00023850	https://www.ebi.ac.uk/metagenomics/api/v1/analyses/MGYA00023850/file/SRR995960_FASTQ_otu.tsv	Root-Host_associated-Human-Skin

ERR443789	MGYA00422347	https://www.ebi.ac.uk/metagenomics/api/v1/analyses/MGYA00422347/file/ERR443789_FASTQ_SSU_OTU.tsv	Root-Host_associated-Human-Skin
SRR994887	MGYA00022185	https://www.ebi.ac.uk/metagenomics/api/v1/analyses/MGYA00022185/file/SRR994887_FASTQ_otu.tsv	Root-Host_associated-Human-Skin
SRR5869709	MGYA00414830	https://www.ebi.ac.uk/metagenomics/api/v1/analyses/MGYA00414830/file/SRR5869709_MERGED_FASTQ_SSU_OTU.tsv	Root-Host_associated-Human-Skin
SRR996875	MGYA00023436	https://www.ebi.ac.uk/metagenomics/api/v1/analyses/MGYA00023436/file/SRR996875_FASTQ_otu.tsv	Root-Host_associated-Human-Skin
SRR996699	MGYA00022296	https://www.ebi.ac.uk/metagenomics/api/v1/analyses/MGYA00022296/file/SRR996699_FASTQ_otu.tsv	Root-Host_associated-Human-Skin
ERR2538354	MGYA00381361	https://www.ebi.ac.uk/metagenomics/api/v1/analyses/MGYA00381361/file/ERR2538354_FASTQ_SSU_OTU.tsv	Root-Host_associated-Human-Skin
SRR6757603	MGYA00380722	https://www.ebi.ac.uk/metagenomics/api/v1/analyses/MGYA00380722/file/SRR6757603_MERGED_FASTQ_SSU_OTU.tsv	Root-Host_associated-Human-Skin
SRR5868802	MGYA00423485	https://www.ebi.ac.uk/metagenomics/api/v1/analyses/MGYA00423485/file/SRR5868802_MERGED_FASTQ_SSU_OTU.tsv	Root-Host_associated-Human-Skin
SRR1623212	MGYA00023907	https://www.ebi.ac.uk/metagenomics/api/v1/analyses/MGYA00023907/file/SRR1623212_MERGED_FASTQ_otu.tsv	Root-Host_associated-Human-Skin
ERR1960777	MGYA00130716	https://www.ebi.ac.uk/metagenomics/api/v1/analyses/MGYA00130716/file/ERR1960777_MERGED_FASTQ_otu.tsv	Root-Host_associated-Human-Skin
ERR451293	MGYA00416287	https://www.ebi.ac.uk/metagenomics/api/v1/analyses/MGYA00416287/file/ERR451293_FASTQ_SSU_OTU.tsv	Root-Host_associated-Human-Skin
ERR451534	MGYA00421390	https://www.ebi.ac.uk/metagenomics/api/v1/analyses/MGYA00421390/file/ERR451534_FASTQ_SSU_OTU.tsv	Root-Host_associated-Human-Skin

SRR5869651	MGYA00419202	https://www.ebi.ac.uk/metagenomics/api/v1/analyses/MGYA00419202/file/SRR5869651_MERGED_FASTQ_SSU_OTU.tsv	Root-Host_associated-Human-Skin
SRR919889	MGYA00024506	https://www.ebi.ac.uk/metagenomics/api/v1/analyses/MGYA00024506/file/SRR919889_FASTQ_otu.tsv	Root-Host_associated-Human-Skin
SRR2005674	MGYA00381331	https://www.ebi.ac.uk/metagenomics/api/v1/analyses/MGYA00381331/file/SRR2005674_MERGED_FASTQ_SSU_OTU.tsv	Root-Host_associated-Human-Skin
ERR443176	MGYA00411911	https://www.ebi.ac.uk/metagenomics/api/v1/analyses/MGYA00411911/file/ERR443176_FASTQ_SSU_OTU.tsv	Root-Host_associated-Human-Skin
ERR443600	MGYA00413653	https://www.ebi.ac.uk/metagenomics/api/v1/analyses/MGYA00413653/file/ERR443600_FASTQ_SSU_OTU.tsv	Root-Host_associated-Human-Skin
ERR1960812	MGYA00130854	https://www.ebi.ac.uk/metagenomics/api/v1/analyses/MGYA00130854/file/ERR1960812_MERGED_FASTQ_otu.tsv	Root-Host_associated-Human-Skin
SRR996229	MGYA00023970	https://www.ebi.ac.uk/metagenomics/api/v1/analyses/MGYA00023970/file/SRR996229_FASTQ_otu.tsv	Root-Host_associated-Human-Skin
SRR5868767	MGYA00417112	https://www.ebi.ac.uk/metagenomics/api/v1/analyses/MGYA00417112/file/SRR5868767_MERGED_FASTQ_SSU_OTU.tsv	Root-Host_associated-Human-Skin
SRR1631302	MGYA00023232	https://www.ebi.ac.uk/metagenomics/api/v1/analyses/MGYA00023232/file/SRR1631302_MERGED_FASTQ_otu.tsv	Root-Host_associated-Human-Skin
SRR920730	MGYA00025023	https://www.ebi.ac.uk/metagenomics/api/v1/analyses/MGYA00025023/file/SRR920730_FASTQ_otu.tsv	Root-Host_associated-Human-Skin
ERR443261	MGYA00428760	https://www.ebi.ac.uk/metagenomics/api/v1/analyses/MGYA00428760/file/ERR443261_FASTQ_SSU_OTU.tsv	Root-Host_associated-Human-Skin
SRR997008	MGYA00022941	https://www.ebi.ac.uk/metagenomics/api/v1/analyses/MGYA00022941/file/SRR997008_FASTQ_otu.tsv	Root-Host_associated-Human-Skin

ERR2538385	MGYA00381392	https://www.ebi.ac.uk/metagenomics/api/v1/analyses/MGYA00381392/file/ERR2538385_FASTQ_SSU_OTU.tsv	Root-Host_associated-Human-Skin
ERR2538422	MGYA00381429	https://www.ebi.ac.uk/metagenomics/api/v1/analyses/MGYA00381429/file/ERR2538422_FASTQ_SSU_OTU.tsv	Root-Host_associated-Human-Skin
SRR3589563	MGYA00193343	https://www.ebi.ac.uk/metagenomics/api/v1/analyses/MGYA00193343/file/SRR3589563_MERGED_FASTQ_SSU_OTU.tsv	Root-Environmental-Aquatic-Marine-Intertidal_zone-Sediment
SRR3589518	MGYA00193348	https://www.ebi.ac.uk/metagenomics/api/v1/analyses/MGYA00193348/file/SRR3589518_MERGED_FASTQ_SSU_OTU.tsv	Root-Environmental-Aquatic-Marine-Intertidal_zone-Sediment
SRR3589564	MGYA00193318	https://www.ebi.ac.uk/metagenomics/api/v1/analyses/MGYA00193318/file/SRR3589564_MERGED_FASTQ_SSU_OTU.tsv	Root-Environmental-Aquatic-Marine-Intertidal_zone-Sediment
SRR389088	MGYA00142375	https://www.ebi.ac.uk/metagenomics/api/v1/analyses/MGYA00142375/file/SRR389088_FASTQ_SSU_OTU.tsv	Root-Environmental-Aquatic-Marine-Intertidal_zone-Sediment
SRR3589516	MGYA00193326	https://www.ebi.ac.uk/metagenomics/api/v1/analyses/MGYA00193326/file/SRR3589516_MERGED_FASTQ_SSU_OTU.tsv	Root-Environmental-Aquatic-Marine-Intertidal_zone-Sediment
SRR3589589	MGYA00193303	https://www.ebi.ac.uk/metagenomics/api/v1/analyses/MGYA00193303/file/SRR3589589_MERGED_FASTQ_SSU_OTU.tsv	Root-Environmental-Aquatic-Marine-Intertidal_zone-Sediment
SRR3589558	MGYA00193350	https://www.ebi.ac.uk/metagenomics/api/v1/analyses/MGYA00193350/file/SRR3589558_MERGED_FASTQ_SSU_OTU.tsv	Root-Environmental-Aquatic-Marine-Intertidal_zone-Sediment
SRR3589524	MGYA00193322	https://www.ebi.ac.uk/metagenomics/api/v1/analyses/MGYA00193322/file/SRR3589524_MERGED_FASTQ_SSU_OTU.tsv	Root-Environmental-Aquatic-Marine-Intertidal_zone-Sediment
SRR3589523	MGYA00193305	https://www.ebi.ac.uk/metagenomics/api/v1/analyses/MGYA00193305/file/SRR3589523_MERGED_FASTQ_SSU_OTU.tsv	Root-Environmental-Aquatic-Marine-Intertidal_zone-Sediment
SRR7535349	MGYA00363293	https://www.ebi.ac.uk/metagenomics/api/v1/analyses/MGYA00363293/file/SRR7535349_FASTQ_SSU_OTU.tsv	Root-Environmental-Aquatic-Marine-Intertidal_zone-Sediment

SRR7535347	MGYA00363291	https://www.ebi.ac.uk/metagenomics/api/v1/analyses/MGYA00363291/file/SRR7535347_FASTQ_SSU_OTU.tsv	Root-Environmental-Aquatic-Marine-Intertidal_zone-Sediment
SRR1611593	MGYA00214209	https://www.ebi.ac.uk/metagenomics/api/v1/analyses/MGYA00214209/file/SRR1611593_FASTQ_SSU_OTU.tsv	Root-Environmental-Aquatic-Marine-Intertidal_zone-Sediment
SRR3589580	MGYA00193287	https://www.ebi.ac.uk/metagenomics/api/v1/analyses/MGYA00193287/file/SRR3589580_MERGED_FASTQ_SSU_OTU.tsv	Root-Environmental-Aquatic-Marine-Intertidal_zone-Sediment
SRR3589551	MGYA00193352	https://www.ebi.ac.uk/metagenomics/api/v1/analyses/MGYA00193352/file/SRR3589551_MERGED_FASTQ_SSU_OTU.tsv	Root-Environmental-Aquatic-Marine-Intertidal_zone-Sediment
SRR3589587	MGYA00193314	https://www.ebi.ac.uk/metagenomics/api/v1/analyses/MGYA00193314/file/SRR3589587_MERGED_FASTQ_SSU_OTU.tsv	Root-Environmental-Aquatic-Marine-Intertidal_zone-Sediment
SRR3589556	MGYA00193268	https://www.ebi.ac.uk/metagenomics/api/v1/analyses/MGYA00193268/file/SRR3589556_MERGED_FASTQ_SSU_OTU.tsv	Root-Environmental-Aquatic-Marine-Intertidal_zone-Sediment
SRR3589547	MGYA00193298	https://www.ebi.ac.uk/metagenomics/api/v1/analyses/MGYA00193298/file/SRR3589547_MERGED_FASTQ_SSU_OTU.tsv	Root-Environmental-Aquatic-Marine-Intertidal_zone-Sediment
SRR3589596	MGYA00193336	https://www.ebi.ac.uk/metagenomics/api/v1/analyses/MGYA00193336/file/SRR3589596_MERGED_FASTQ_SSU_OTU.tsv	Root-Environmental-Aquatic-Marine-Intertidal_zone-Sediment
SRR7535351	MGYA00363295	https://www.ebi.ac.uk/metagenomics/api/v1/analyses/MGYA00363295/file/SRR7535351_FASTQ_SSU_OTU.tsv	Root-Environmental-Aquatic-Marine-Intertidal_zone-Sediment
SRR7535356	MGYA00363300	https://www.ebi.ac.uk/metagenomics/api/v1/analyses/MGYA00363300/file/SRR7535356_FASTQ_SSU_OTU.tsv	Root-Environmental-Aquatic-Marine-Intertidal_zone-Sediment
SRR3589540	MGYA00193359	https://www.ebi.ac.uk/metagenomics/api/v1/analyses/MGYA00193359/file/SRR3589540_MERGED_FASTQ_SSU_OTU.tsv	Root-Environmental-Aquatic-Marine-Intertidal_zone-Sediment
SRR3589591	MGYA00193356	https://www.ebi.ac.uk/metagenomics/api/v1/analyses/MGYA00193356/file/SRR3589591_MERGED_FASTQ_SSU_OTU.tsv	Root-Environmental-Aquatic-Marine-Intertidal_zone-Sediment

SRR3589532	MGYA00193332	https://www.ebi.ac.uk/metagenomics/api/v1/analyses/MGYA00193332/file/SRR3589532_MERGED_FASTQ_SSU_OTU.tsv	Root-Environmental-Aquatic-Marine-Intertidal_zone-Sediment
SRR3589625	MGYA00193328	https://www.ebi.ac.uk/metagenomics/api/v1/analyses/MGYA00193328/file/SRR3589625_MERGED_FASTQ_SSU_OTU.tsv	Root-Environmental-Aquatic-Marine-Intertidal_zone-Sediment
SRR3589549	MGYA00193327	https://www.ebi.ac.uk/metagenomics/api/v1/analyses/MGYA00193327/file/SRR3589549_MERGED_FASTQ_SSU_OTU.tsv	Root-Environmental-Aquatic-Marine-Intertidal_zone-Sediment
SRR3589598	MGYA00193363	https://www.ebi.ac.uk/metagenomics/api/v1/analyses/MGYA00193363/file/SRR3589598_MERGED_FASTQ_SSU_OTU.tsv	Root-Environmental-Aquatic-Marine-Intertidal_zone-Sediment
SRR3589535	MGYA00193279	https://www.ebi.ac.uk/metagenomics/api/v1/analyses/MGYA00193279/file/SRR3589535_MERGED_FASTQ_SSU_OTU.tsv	Root-Environmental-Aquatic-Marine-Intertidal_zone-Sediment
SRR3589622	MGYA00193346	https://www.ebi.ac.uk/metagenomics/api/v1/analyses/MGYA00193346/file/SRR3589622_MERGED_FASTQ_SSU_OTU.tsv	Root-Environmental-Aquatic-Marine-Intertidal_zone-Sediment
SRR1611604	MGYA00214211	https://www.ebi.ac.uk/metagenomics/api/v1/analyses/MGYA00214211/file/SRR1611604_FASTQ_SSU_OTU.tsv	Root-Environmental-Aquatic-Marine-Intertidal_zone-Sediment
SRR830622	MGYA00142361	https://www.ebi.ac.uk/metagenomics/api/v1/analyses/MGYA00142361/file/SRR830622_MERGED_FASTQ_SSU_OTU.tsv	Root-Environmental-Aquatic-Marine-Intertidal_zone-Sediment
SRR3589617	MGYA00193325	https://www.ebi.ac.uk/metagenomics/api/v1/analyses/MGYA00193325/file/SRR3589617_MERGED_FASTQ_SSU_OTU.tsv	Root-Environmental-Aquatic-Marine-Intertidal_zone-Sediment
SRR830625	MGYA00142363	https://www.ebi.ac.uk/metagenomics/api/v1/analyses/MGYA00142363/file/SRR830625_MERGED_FASTQ_SSU_OTU.tsv	Root-Environmental-Aquatic-Marine-Intertidal_zone-Sediment
SRR3589610	MGYA00193320	https://www.ebi.ac.uk/metagenomics/api/v1/analyses/MGYA00193320/file/SRR3589610_MERGED_FASTQ_SSU_OTU.tsv	Root-Environmental-Aquatic-Marine-Intertidal_zone-Sediment
SRR1611603	MGYA00214207	https://www.ebi.ac.uk/metagenomics/api/v1/analyses/MGYA00214207/file/SRR1611603_FASTQ_SSU_OTU.tsv	Root-Environmental-Aquatic-Marine-Intertidal_zone-Sediment

SRR023193	MGYA00357571	https://www.ebi.ac.uk/metagenomics/api/v1/analyses/MGYA00357571/file/SRR023193_FASTQ_SSU_OTU.tsv	Root-Environmental-Aquatic-Marine-Intertidal_zone-Sediment
SRR3589575	MGYA00193271	https://www.ebi.ac.uk/metagenomics/api/v1/analyses/MGYA00193271/file/SRR3589575_MERGED_FASTQ_SSU_OTU.tsv	Root-Environmental-Aquatic-Marine-Intertidal_zone-Sediment
SRR3589572	MGYA00193289	https://www.ebi.ac.uk/metagenomics/api/v1/analyses/MGYA00193289/file/SRR3589572_MERGED_FASTQ_SSU_OTU.tsv	Root-Environmental-Aquatic-Marine-Intertidal_zone-Sediment
SRR3589619	MGYA00193361	https://www.ebi.ac.uk/metagenomics/api/v1/analyses/MGYA00193361/file/SRR3589619_MERGED_FASTQ_SSU_OTU.tsv	Root-Environmental-Aquatic-Marine-Intertidal_zone-Sediment
SRR3589586	MGYA00193293	https://www.ebi.ac.uk/metagenomics/api/v1/analyses/MGYA00193293/file/SRR3589586_MERGED_FASTQ_SSU_OTU.tsv	Root-Environmental-Aquatic-Marine-Intertidal_zone-Sediment
SRR3589557	MGYA00193292	https://www.ebi.ac.uk/metagenomics/api/v1/analyses/MGYA00193292/file/SRR3589557_MERGED_FASTQ_SSU_OTU.tsv	Root-Environmental-Aquatic-Marine-Intertidal_zone-Sediment
SRR1611595	MGYA00214198	https://www.ebi.ac.uk/metagenomics/api/v1/analyses/MGYA00214198/file/SRR1611595_FASTQ_SSU_OTU.tsv	Root-Environmental-Aquatic-Marine-Intertidal_zone-Sediment
SRR1611592	MGYA00214202	https://www.ebi.ac.uk/metagenomics/api/v1/analyses/MGYA00214202/file/SRR1611592_FASTQ_SSU_OTU.tsv	Root-Environmental-Aquatic-Marine-Intertidal_zone-Sediment
SRR3589581	MGYA00193367	https://www.ebi.ac.uk/metagenomics/api/v1/analyses/MGYA00193367/file/SRR3589581_MERGED_FASTQ_SSU_OTU.tsv	Root-Environmental-Aquatic-Marine-Intertidal_zone-Sediment
SRR3589550	MGYA00193370	https://www.ebi.ac.uk/metagenomics/api/v1/analyses/MGYA00193370/file/SRR3589550_MERGED_FASTQ_SSU_OTU.tsv	Root-Environmental-Aquatic-Marine-Intertidal_zone-Sediment
SRR3589522	MGYA00193307	https://www.ebi.ac.uk/metagenomics/api/v1/analyses/MGYA00193307/file/SRR3589522_MERGED_FASTQ_SSU_OTU.tsv	Root-Environmental-Aquatic-Marine-Intertidal_zone-Sediment
SRR7535348	MGYA00363292	https://www.ebi.ac.uk/metagenomics/api/v1/analyses/MGYA00363292/file/SRR7535348_FASTQ_SSU_OTU.tsv	Root-Environmental-Aquatic-Marine-Intertidal_zone-Sediment

SRR3589588	MGYA00193308	https://www.ebi.ac.uk/metagenomics/api/v1/analyses/MGYA00193308/file/SRR3589588_MERGED_FASTQ_SSU_OTU.tsv	Root-Environmental-Aquatic-Marine-Intertidal_zone-Sediment
SRR3589559	MGYA00193321	https://www.ebi.ac.uk/metagenomics/api/v1/analyses/MGYA00193321/file/SRR3589559_MERGED_FASTQ_SSU_OTU.tsv	Root-Environmental-Aquatic-Marine-Intertidal_zone-Sediment
SRR3589525	MGYA00193316	https://www.ebi.ac.uk/metagenomics/api/v1/analyses/MGYA00193316/file/SRR3589525_MERGED_FASTQ_SSU_OTU.tsv	Root-Environmental-Aquatic-Marine-Intertidal_zone-Sediment
SRR3589517	MGYA00193273	https://www.ebi.ac.uk/metagenomics/api/v1/analyses/MGYA00193273/file/SRR3589517_MERGED_FASTQ_SSU_OTU.tsv	Root-Environmental-Aquatic-Marine-Intertidal_zone-Sediment
SRR389087	MGYA00142376	https://www.ebi.ac.uk/metagenomics/api/v1/analyses/MGYA00142376/file/SRR389087_FASTQ_SSU_OTU.tsv	Root-Environmental-Aquatic-Marine-Intertidal_zone-Sediment
SRR3589519	MGYA00193366	https://www.ebi.ac.uk/metagenomics/api/v1/analyses/MGYA00193366/file/SRR3589519_MERGED_FASTQ_SSU_OTU.tsv	Root-Environmental-Aquatic-Marine-Intertidal_zone-Sediment
SRR3589565	MGYA00193369	https://www.ebi.ac.uk/metagenomics/api/v1/analyses/MGYA00193369/file/SRR3589565_MERGED_FASTQ_SSU_OTU.tsv	Root-Environmental-Aquatic-Marine-Intertidal_zone-Sediment
SRR3589562	MGYA00193300	https://www.ebi.ac.uk/metagenomics/api/v1/analyses/MGYA00193300/file/SRR3589562_MERGED_FASTQ_SSU_OTU.tsv	Root-Environmental-Aquatic-Marine-Intertidal_zone-Sediment
SRR3589573	MGYA00193355	https://www.ebi.ac.uk/metagenomics/api/v1/analyses/MGYA00193355/file/SRR3589573_MERGED_FASTQ_SSU_OTU.tsv	Root-Environmental-Aquatic-Marine-Intertidal_zone-Sediment
SRR3589618	MGYA00193338	https://www.ebi.ac.uk/metagenomics/api/v1/analyses/MGYA00193338/file/SRR3589618_MERGED_FASTQ_SSU_OTU.tsv	Root-Environmental-Aquatic-Marine-Intertidal_zone-Sediment
SRR3589574	MGYA00193315	https://www.ebi.ac.uk/metagenomics/api/v1/analyses/MGYA00193315/file/SRR3589574_MERGED_FASTQ_SSU_OTU.tsv	Root-Environmental-Aquatic-Marine-Intertidal_zone-Sediment
SRR830624	MGYA00142362	https://www.ebi.ac.uk/metagenomics/api/v1/analyses/MGYA00142362/file/SRR830624_MERGED_FASTQ_SSU_OTU.tsv	Root-Environmental-Aquatic-Marine-Intertidal_zone-Sediment

SRR3589611	MGYA00193337	https://www.ebi.ac.uk/metagenomics/api/v1/analyses/MGYA00193337/file/SRR3589611_MERGED_FASTQ_SSU_OTU.tsv	Root-Environmental-Aquatic-Marine-Intertidal_zone-Sediment
SRR1611602	MGYA00214204	https://www.ebi.ac.uk/metagenomics/api/v1/analyses/MGYA00214204/file/SRR1611602_FASTQ_SSU_OTU.tsv	Root-Environmental-Aquatic-Marine-Intertidal_zone-Sediment
SRR1611605	MGYA00214203	https://www.ebi.ac.uk/metagenomics/api/v1/analyses/MGYA00214203/file/SRR1611605_FASTQ_SSU_OTU.tsv	Root-Environmental-Aquatic-Marine-Intertidal_zone-Sediment
SRR3589616	MGYA00193324	https://www.ebi.ac.uk/metagenomics/api/v1/analyses/MGYA00193324/file/SRR3589616_MERGED_FASTQ_SSU_OTU.tsv	Root-Environmental-Aquatic-Marine-Intertidal_zone-Sediment
SRR3589548	MGYA00193270	https://www.ebi.ac.uk/metagenomics/api/v1/analyses/MGYA00193270/file/SRR3589548_MERGED_FASTQ_SSU_OTU.tsv	Root-Environmental-Aquatic-Marine-Intertidal_zone-Sediment
SRR3589599	MGYA00193282	https://www.ebi.ac.uk/metagenomics/api/v1/analyses/MGYA00193282/file/SRR3589599_MERGED_FASTQ_SSU_OTU.tsv	Root-Environmental-Aquatic-Marine-Intertidal_zone-Sediment
SRR3589534	MGYA00193275	https://www.ebi.ac.uk/metagenomics/api/v1/analyses/MGYA00193275/file/SRR3589534_MERGED_FASTQ_SSU_OTU.tsv	Root-Environmental-Aquatic-Marine-Intertidal_zone-Sediment
SRR3589623	MGYA00193313	https://www.ebi.ac.uk/metagenomics/api/v1/analyses/MGYA00193313/file/SRR3589623_MERGED_FASTQ_SSU_OTU.tsv	Root-Environmental-Aquatic-Marine-Intertidal_zone-Sediment
SRR3589533	MGYA00193360	https://www.ebi.ac.uk/metagenomics/api/v1/analyses/MGYA00193360/file/SRR3589533_MERGED_FASTQ_SSU_OTU.tsv	Root-Environmental-Aquatic-Marine-Intertidal_zone-Sediment
SRR3589624	MGYA00193295	https://www.ebi.ac.uk/metagenomics/api/v1/analyses/MGYA00193295/file/SRR3589624_MERGED_FASTQ_SSU_OTU.tsv	Root-Environmental-Aquatic-Marine-Intertidal_zone-Sediment
SRR3589541	MGYA00193301	https://www.ebi.ac.uk/metagenomics/api/v1/analyses/MGYA00193301/file/SRR3589541_MERGED_FASTQ_SSU_OTU.tsv	Root-Environmental-Aquatic-Marine-Intertidal_zone-Sediment
SRR3589590	MGYA00193347	https://www.ebi.ac.uk/metagenomics/api/v1/analyses/MGYA00193347/file/SRR3589590_MERGED_FASTQ_SSU_OTU.tsv	Root-Environmental-Aquatic-Marine-Intertidal_zone-Sediment

SRR3589546	MGYA00193280	https://www.ebi.ac.uk/metagenomics/api/v1/analyses/MGYA00193280/file/SRR3589546_MERGED_FASTQ_SSU_OTU.tsv	Root-Environmental-Aquatic-Marine-Intertidal_zone-Sediment
SRR3589597	MGYA00193342	https://www.ebi.ac.uk/metagenomics/api/v1/analyses/MGYA00193342/file/SRR3589597_MERGED_FASTQ_SSU_OTU.tsv	Root-Environmental-Aquatic-Marine-Intertidal_zone-Sediment
SRR7535350	MGYA00363294	https://www.ebi.ac.uk/metagenomics/api/v1/analyses/MGYA00363294/file/SRR7535350_FASTQ_SSU_OTU.tsv	Root-Environmental-Aquatic-Marine-Intertidal_zone-Sediment
SRR3589577	MGYA00193302	https://www.ebi.ac.uk/metagenomics/api/v1/analyses/MGYA00193302/file/SRR3589577_MERGED_FASTQ_SSU_OTU.tsv	Root-Environmental-Aquatic-Marine-Intertidal_zone-Sediment
SRR3589570	MGYA00193284	https://www.ebi.ac.uk/metagenomics/api/v1/analyses/MGYA00193284/file/SRR3589570_MERGED_FASTQ_SSU_OTU.tsv	Root-Environmental-Aquatic-Marine-Intertidal_zone-Sediment
SRR1611606	MGYA00214210	https://www.ebi.ac.uk/metagenomics/api/v1/analyses/MGYA00214210/file/SRR1611606_FASTQ_SSU_OTU.tsv	Root-Environmental-Aquatic-Marine-Intertidal_zone-Sediment
SRR3589615	MGYA00193319	https://www.ebi.ac.uk/metagenomics/api/v1/analyses/MGYA00193319/file/SRR3589615_MERGED_FASTQ_SSU_OTU.tsv	Root-Environmental-Aquatic-Marine-Intertidal_zone-Sediment
SRR3589612	MGYA00193291	https://www.ebi.ac.uk/metagenomics/api/v1/analyses/MGYA00193291/file/SRR3589612_MERGED_FASTQ_SSU_OTU.tsv	Root-Environmental-Aquatic-Marine-Intertidal_zone-Sediment
SRR1611601	MGYA00214213	https://www.ebi.ac.uk/metagenomics/api/v1/analyses/MGYA00214213/file/SRR1611601_FASTQ_SSU_OTU.tsv	Root-Environmental-Aquatic-Marine-Intertidal_zone-Sediment
SRR3589579	MGYA00193362	https://www.ebi.ac.uk/metagenomics/api/v1/analyses/MGYA00193362/file/SRR3589579_MERGED_FASTQ_SSU_OTU.tsv	Root-Environmental-Aquatic-Marine-Intertidal_zone-Sediment
SRR3589530	MGYA00193323	https://www.ebi.ac.uk/metagenomics/api/v1/analyses/MGYA00193323/file/SRR3589530_MERGED_FASTQ_SSU_OTU.tsv	Root-Environmental-Aquatic-Marine-Intertidal_zone-Sediment
SRR3589627	MGYA00193351	https://www.ebi.ac.uk/metagenomics/api/v1/analyses/MGYA00193351/file/SRR3589627_MERGED_FASTQ_SSU_OTU.tsv	Root-Environmental-Aquatic-Marine-Intertidal_zone-Sediment

SRR3589537	MGYA00193312	https://www.ebi.ac.uk/metagenomics/api/v1/analyses/MGYA00193312/file/SRR3589537_MERGED_FASTQ_SSU_OTU.tsv	Root-Environmental-Aquatic-Marine-Intertidal_zone-Sediment
SRR3589620	MGYA00193339	https://www.ebi.ac.uk/metagenomics/api/v1/analyses/MGYA00193339/file/SRR3589620_MERGED_FASTQ_SSU_OTU.tsv	Root-Environmental-Aquatic-Marine-Intertidal_zone-Sediment
SRR3589545	MGYA00193278	https://www.ebi.ac.uk/metagenomics/api/v1/analyses/MGYA00193278/file/SRR3589545_MERGED_FASTQ_SSU_OTU.tsv	Root-Environmental-Aquatic-Marine-Intertidal_zone-Sediment
SRR3589594	MGYA00193330	https://www.ebi.ac.uk/metagenomics/api/v1/analyses/MGYA00193330/file/SRR3589594_MERGED_FASTQ_SSU_OTU.tsv	Root-Environmental-Aquatic-Marine-Intertidal_zone-Sediment
SRR7535353	MGYA00363297	https://www.ebi.ac.uk/metagenomics/api/v1/analyses/MGYA00363297/file/SRR7535353_FASTQ_SSU_OTU.tsv	Root-Environmental-Aquatic-Marine-Intertidal_zone-Sediment
SRR3589539	MGYA00193294	https://www.ebi.ac.uk/metagenomics/api/v1/analyses/MGYA00193294/file/SRR3589539_MERGED_FASTQ_SSU_OTU.tsv	Root-Environmental-Aquatic-Marine-Intertidal_zone-Sediment
SRR7535354	MGYA00363298	https://www.ebi.ac.uk/metagenomics/api/v1/analyses/MGYA00363298/file/SRR7535354_FASTQ_SSU_OTU.tsv	Root-Environmental-Aquatic-Marine-Intertidal_zone-Sediment
SRR3589629	MGYA00193340	https://www.ebi.ac.uk/metagenomics/api/v1/analyses/MGYA00193340/file/SRR3589629_MERGED_FASTQ_SSU_OTU.tsv	Root-Environmental-Aquatic-Marine-Intertidal_zone-Sediment
SRR3589542	MGYA00193281	https://www.ebi.ac.uk/metagenomics/api/v1/analyses/MGYA00193281/file/SRR3589542_MERGED_FASTQ_SSU_OTU.tsv	Root-Environmental-Aquatic-Marine-Intertidal_zone-Sediment
SRR3589593	MGYA00193365	https://www.ebi.ac.uk/metagenomics/api/v1/analyses/MGYA00193365/file/SRR3589593_MERGED_FASTQ_SSU_OTU.tsv	Root-Environmental-Aquatic-Marine-Intertidal_zone-Sediment
SRR1611591	MGYA00214196	https://www.ebi.ac.uk/metagenomics/api/v1/analyses/MGYA00214196/file/SRR1611591_FASTQ_SSU_OTU.tsv	Root-Environmental-Aquatic-Marine-Intertidal_zone-Sediment
SRR3589582	MGYA00193349	https://www.ebi.ac.uk/metagenomics/api/v1/analyses/MGYA00193349/file/SRR3589582_MERGED_FASTQ_SSU_OTU.tsv	Root-Environmental-Aquatic-Marine-Intertidal_zone-Sediment

SRR3589553	MGYA00193341	https://www.ebi.ac.uk/metagenomics/api/v1/analyses/MGYA00193341/file/SRR3589553_MERGED_FASTQ_SSU_OTU.tsv	Root-Environmental-Aquatic-Marine-Intertidal_zone-Sediment
SRR3589585	MGYA00193354	https://www.ebi.ac.uk/metagenomics/api/v1/analyses/MGYA00193354/file/SRR3589585_MERGED_FASTQ_SSU_OTU.tsv	Root-Environmental-Aquatic-Marine-Intertidal_zone-Sediment
SRR3589554	MGYA00193286	https://www.ebi.ac.uk/metagenomics/api/v1/analyses/MGYA00193286/file/SRR3589554_MERGED_FASTQ_SSU_OTU.tsv	Root-Environmental-Aquatic-Marine-Intertidal_zone-Sediment
SRR3589528	MGYA00193334	https://www.ebi.ac.uk/metagenomics/api/v1/analyses/MGYA00193334/file/SRR3589528_MERGED_FASTQ_SSU_OTU.tsv	Root-Environmental-Aquatic-Marine-Intertidal_zone-Sediment
SRR1611596	MGYA00214212	https://www.ebi.ac.uk/metagenomics/api/v1/analyses/MGYA00214212/file/SRR1611596_FASTQ_SSU_OTU.tsv	Root-Environmental-Aquatic-Marine-Intertidal_zone-Sediment
SRR3589526	MGYA00193357	https://www.ebi.ac.uk/metagenomics/api/v1/analyses/MGYA00193357/file/SRR3589526_MERGED_FASTQ_SSU_OTU.tsv	Root-Environmental-Aquatic-Marine-Intertidal_zone-Sediment
SRR1611598	MGYA00214199	https://www.ebi.ac.uk/metagenomics/api/v1/analyses/MGYA00214199/file/SRR1611598_FASTQ_SSU_OTU.tsv	Root-Environmental-Aquatic-Marine-Intertidal_zone-Sediment
SRR3589521	MGYA00193304	https://www.ebi.ac.uk/metagenomics/api/v1/analyses/MGYA00193304/file/SRR3589521_MERGED_FASTQ_SSU_OTU.tsv	Root-Environmental-Aquatic-Marine-Intertidal_zone-Sediment
SRR3589514	MGYA00193272	https://www.ebi.ac.uk/metagenomics/api/v1/analyses/MGYA00193272/file/SRR3589514_MERGED_FASTQ_SSU_OTU.tsv	Root-Environmental-Aquatic-Marine-Intertidal_zone-Sediment
SRR3589568	MGYA00193277	https://www.ebi.ac.uk/metagenomics/api/v1/analyses/MGYA00193277/file/SRR3589568_MERGED_FASTQ_SSU_OTU.tsv	Root-Environmental-Aquatic-Marine-Intertidal_zone-Sediment
SRR1611610	MGYA00214208	https://www.ebi.ac.uk/metagenomics/api/v1/analyses/MGYA00214208/file/SRR1611610_FASTQ_SSU_OTU.tsv	Root-Environmental-Aquatic-Marine-Intertidal_zone-Sediment
SRR3589561	MGYA00193335	https://www.ebi.ac.uk/metagenomics/api/v1/analyses/MGYA00193335/file/SRR3589561_MERGED_FASTQ_SSU_OTU.tsv	Root-Environmental-Aquatic-Marine-Intertidal_zone-Sediment

SRR3589566	MGYA00193276	https://www.ebi.ac.uk/metagenomics/api/v1/analyses/MGYA00193276/file/SRR3589566_MERGED_FASTQ_SSU_OTU.tsv	Root-Environmental-Aquatic-Marine-Intertidal_zone-Sediment
SRR7535355	MGYA00363299	https://www.ebi.ac.uk/metagenomics/api/v1/analyses/MGYA00363299/file/SRR7535355_FASTQ_SSU_OTU.tsv	Root-Environmental-Aquatic-Marine-Intertidal_zone-Sediment
SRR3589628	MGYA00193285	https://www.ebi.ac.uk/metagenomics/api/v1/analyses/MGYA00193285/file/SRR3589628_MERGED_FASTQ_SSU_OTU.tsv	Root-Environmental-Aquatic-Marine-Intertidal_zone-Sediment
SRR3589543	MGYA00193329	https://www.ebi.ac.uk/metagenomics/api/v1/analyses/MGYA00193329/file/SRR3589543_MERGED_FASTQ_SSU_OTU.tsv	Root-Environmental-Aquatic-Marine-Intertidal_zone-Sediment
SRR3589592	MGYA00193267	https://www.ebi.ac.uk/metagenomics/api/v1/analyses/MGYA00193267/file/SRR3589592_MERGED_FASTQ_SSU_OTU.tsv	Root-Environmental-Aquatic-Marine-Intertidal_zone-Sediment
SRR3589544	MGYA00193299	https://www.ebi.ac.uk/metagenomics/api/v1/analyses/MGYA00193299/file/SRR3589544_MERGED_FASTQ_SSU_OTU.tsv	Root-Environmental-Aquatic-Marine-Intertidal_zone-Sediment
SRR3589595	MGYA00193288	https://www.ebi.ac.uk/metagenomics/api/v1/analyses/MGYA00193288/file/SRR3589595_MERGED_FASTQ_SSU_OTU.tsv	Root-Environmental-Aquatic-Marine-Intertidal_zone-Sediment
SRR7535352	MGYA00363296	https://www.ebi.ac.uk/metagenomics/api/v1/analyses/MGYA00363296/file/SRR7535352_FASTQ_SSU_OTU.tsv	Root-Environmental-Aquatic-Marine-Intertidal_zone-Sediment
SRR3589538	MGYA00193310	https://www.ebi.ac.uk/metagenomics/api/v1/analyses/MGYA00193310/file/SRR3589538_MERGED_FASTQ_SSU_OTU.tsv	Root-Environmental-Aquatic-Marine-Intertidal_zone-Sediment
SRR3589536	MGYA00193274	https://www.ebi.ac.uk/metagenomics/api/v1/analyses/MGYA00193274/file/SRR3589536_MERGED_FASTQ_SSU_OTU.tsv	Root-Environmental-Aquatic-Marine-Intertidal_zone-Sediment
SRR3589621	MGYA00193353	https://www.ebi.ac.uk/metagenomics/api/v1/analyses/MGYA00193353/file/SRR3589621_MERGED_FASTQ_SSU_OTU.tsv	Root-Environmental-Aquatic-Marine-Intertidal_zone-Sediment
SRR3589531	MGYA00193358	https://www.ebi.ac.uk/metagenomics/api/v1/analyses/MGYA00193358/file/SRR3589531_MERGED_FASTQ_SSU_OTU.tsv	Root-Environmental-Aquatic-Marine-Intertidal_zone-Sediment

SRR3589626	MGYA00193364	https://www.ebi.ac.uk/metagenomics/api/v1/analyses/MGYA00193364/file/SRR3589626_MERGED_FASTQ_SSU_OTU.tsv	Root-Environmental-Aquatic-Marine-Intertidal_zone-Sediment
SRR3589613	MGYA00193311	https://www.ebi.ac.uk/metagenomics/api/v1/analyses/MGYA00193311/file/SRR3589613_MERGED_FASTQ_SSU_OTU.tsv	Root-Environmental-Aquatic-Marine-Intertidal_zone-Sediment
SRR3589578	MGYA00193368	https://www.ebi.ac.uk/metagenomics/api/v1/analyses/MGYA00193368/file/SRR3589578_MERGED_FASTQ_SSU_OTU.tsv	Root-Environmental-Aquatic-Marine-Intertidal_zone-Sediment
SRR1611607	MGYA00214197	https://www.ebi.ac.uk/metagenomics/api/v1/analyses/MGYA00214197/file/SRR1611607_FASTQ_SSU_OTU.tsv	Root-Environmental-Aquatic-Marine-Intertidal_zone-Sediment
SRR3589614	MGYA00193283	https://www.ebi.ac.uk/metagenomics/api/v1/analyses/MGYA00193283/file/SRR3589614_MERGED_FASTQ_SSU_OTU.tsv	Root-Environmental-Aquatic-Marine-Intertidal_zone-Sediment
SRR1611609	MGYA00214205	https://www.ebi.ac.uk/metagenomics/api/v1/analyses/MGYA00214205/file/SRR1611609_FASTQ_SSU_OTU.tsv	Root-Environmental-Aquatic-Marine-Intertidal_zone-Sediment
SRR3589571	MGYA00193269	https://www.ebi.ac.uk/metagenomics/api/v1/analyses/MGYA00193269/file/SRR3589571_MERGED_FASTQ_SSU_OTU.tsv	Root-Environmental-Aquatic-Marine-Intertidal_zone-Sediment
SRR3589576	MGYA00193333	https://www.ebi.ac.uk/metagenomics/api/v1/analyses/MGYA00193333/file/SRR3589576_MERGED_FASTQ_SSU_OTU.tsv	Root-Environmental-Aquatic-Marine-Intertidal_zone-Sediment
SRR3589567	MGYA00193345	https://www.ebi.ac.uk/metagenomics/api/v1/analyses/MGYA00193345/file/SRR3589567_MERGED_FASTQ_SSU_OTU.tsv	Root-Environmental-Aquatic-Marine-Intertidal_zone-Sediment
SRR3589560	MGYA00193371	https://www.ebi.ac.uk/metagenomics/api/v1/analyses/MGYA00193371/file/SRR3589560_MERGED_FASTQ_SSU_OTU.tsv	Root-Environmental-Aquatic-Marine-Intertidal_zone-Sediment
SRR3589515	MGYA00193306	https://www.ebi.ac.uk/metagenomics/api/v1/analyses/MGYA00193306/file/SRR3589515_MERGED_FASTQ_SSU_OTU.tsv	Root-Environmental-Aquatic-Marine-Intertidal_zone-Sediment
SRR3589569	MGYA00193296	https://www.ebi.ac.uk/metagenomics/api/v1/analyses/MGYA00193296/file/SRR3589569_MERGED_FASTQ_SSU_OTU.tsv	Root-Environmental-Aquatic-Marine-Intertidal_zone-Sediment

SRR1611611	MGYA00214201	https://www.ebi.ac.uk/metagenomics/api/v1/analyses/MGYA00214201/file/SRR1611611_FASTQ_SSU_OTU.tsv	Root-Environmental-Aquatic-Marine-Intertidal_zone-Sediment
SRR3589520	MGYA00193344	https://www.ebi.ac.uk/metagenomics/api/v1/analyses/MGYA00193344/file/SRR3589520_MERGED_FASTQ_SSU_OTU.tsv	Root-Environmental-Aquatic-Marine-Intertidal_zone-Sediment
SRR3589527	MGYA00193317	https://www.ebi.ac.uk/metagenomics/api/v1/analyses/MGYA00193317/file/SRR3589527_MERGED_FASTQ_SSU_OTU.tsv	Root-Environmental-Aquatic-Marine-Intertidal_zone-Sediment
SRR1611599	MGYA00214206	https://www.ebi.ac.uk/metagenomics/api/v1/analyses/MGYA00214206/file/SRR1611599_FASTQ_SSU_OTU.tsv	Root-Environmental-Aquatic-Marine-Intertidal_zone-Sediment
SRR3589584	MGYA00193290	https://www.ebi.ac.uk/metagenomics/api/v1/analyses/MGYA00193290/file/SRR3589584_MERGED_FASTQ_SSU_OTU.tsv	Root-Environmental-Aquatic-Marine-Intertidal_zone-Sediment
SRR3589555	MGYA00193372	https://www.ebi.ac.uk/metagenomics/api/v1/analyses/MGYA00193372/file/SRR3589555_MERGED_FASTQ_SSU_OTU.tsv	Root-Environmental-Aquatic-Marine-Intertidal_zone-Sediment
SRR3589529	MGYA00193297	https://www.ebi.ac.uk/metagenomics/api/v1/analyses/MGYA00193297/file/SRR3589529_MERGED_FASTQ_SSU_OTU.tsv	Root-Environmental-Aquatic-Marine-Intertidal_zone-Sediment
SRR1611597	MGYA00214200	https://www.ebi.ac.uk/metagenomics/api/v1/analyses/MGYA00214200/file/SRR1611597_FASTQ_SSU_OTU.tsv	Root-Environmental-Aquatic-Marine-Intertidal_zone-Sediment
SRR3589583	MGYA00193309	https://www.ebi.ac.uk/metagenomics/api/v1/analyses/MGYA00193309/file/SRR3589583_MERGED_FASTQ_SSU_OTU.tsv	Root-Environmental-Aquatic-Marine-Intertidal_zone-Sediment
SRR3589552	MGYA00193331	https://www.ebi.ac.uk/metagenomics/api/v1/analyses/MGYA00193331/file/SRR3589552_MERGED_FASTQ_SSU_OTU.tsv	Root-Environmental-Aquatic-Marine-Intertidal_zone-Sediment
SRR5465810	MGYA00207275	https://www.ebi.ac.uk/metagenomics/api/v1/analyses/MGYA00207275/file/SRR5465810_MERGED_FASTQ_SSU_OTU.tsv	Root-Environmental-Aquatic-Marine-Oceanic-Aphotic_zone
SRR3884365	MGYA00211419	https://www.ebi.ac.uk/metagenomics/api/v1/analyses/MGYA00211419/file/SRR3884365_MERGED_FASTQ_SSU_OTU.tsv	Root-Environmental-Aquatic-Marine-Oceanic-Aphotic_zone

SRR5465798	MGYA00207264	https://www.ebi.ac.uk/metagenomics/api/v1/analyses/MGYA00207264/file/SRR5465798_MERGED_FASTQ_SSU_OTU.tsv	Root-Environmental-Aquatic-Marine-Oceanic-Aphotic_zone
SRR3884381	MGYA00211410	https://www.ebi.ac.uk/metagenomics/api/v1/analyses/MGYA00211410/file/SRR3884381_MERGED_FASTQ_SSU_OTU.tsv	Root-Environmental-Aquatic-Marine-Oceanic-Aphotic_zone
SRR5465791	MGYA00207252	https://www.ebi.ac.uk/metagenomics/api/v1/analyses/MGYA00207252/file/SRR5465791_MERGED_FASTQ_SSU_OTU.tsv	Root-Environmental-Aquatic-Marine-Oceanic-Aphotic_zone
SRR3884386	MGYA00211427	https://www.ebi.ac.uk/metagenomics/api/v1/analyses/MGYA00211427/file/SRR3884386_MERGED_FASTQ_SSU_OTU.tsv	Root-Environmental-Aquatic-Marine-Oceanic-Aphotic_zone
SRR5465796	MGYA00207285	https://www.ebi.ac.uk/metagenomics/api/v1/analyses/MGYA00207285/file/SRR5465796_MERGED_FASTQ_SSU_OTU.tsv	Root-Environmental-Aquatic-Marine-Oceanic-Aphotic_zone
SRR5465787	MGYA00207270	https://www.ebi.ac.uk/metagenomics/api/v1/analyses/MGYA00207270/file/SRR5465787_MERGED_FASTQ_SSU_OTU.tsv	Root-Environmental-Aquatic-Marine-Oceanic-Aphotic_zone
SRR5465780	MGYA00207280	https://www.ebi.ac.uk/metagenomics/api/v1/analyses/MGYA00207280/file/SRR5465780_MERGED_FASTQ_SSU_OTU.tsv	Root-Environmental-Aquatic-Marine-Oceanic-Aphotic_zone
SRR5465789	MGYA00207265	https://www.ebi.ac.uk/metagenomics/api/v1/analyses/MGYA00207265/file/SRR5465789_MERGED_FASTQ_SSU_OTU.tsv	Root-Environmental-Aquatic-Marine-Oceanic-Aphotic_zone
SRR5465808	MGYA00207266	https://www.ebi.ac.uk/metagenomics/api/v1/analyses/MGYA00207266/file/SRR5465808_MERGED_FASTQ_SSU_OTU.tsv	Root-Environmental-Aquatic-Marine-Oceanic-Aphotic_zone
SRR3884374	MGYA00211414	https://www.ebi.ac.uk/metagenomics/api/v1/analyses/MGYA00211414/file/SRR3884374_MERGED_FASTQ_SSU_OTU.tsv	Root-Environmental-Aquatic-Marine-Oceanic-Aphotic_zone
SRR5465801	MGYA00207269	https://www.ebi.ac.uk/metagenomics/api/v1/analyses/MGYA00207269/file/SRR5465801_MERGED_FASTQ_SSU_OTU.tsv	Root-Environmental-Aquatic-Marine-Oceanic-Aphotic_zone
SRR3884373	MGYA00211430	https://www.ebi.ac.uk/metagenomics/api/v1/analyses/MGYA00211430/file/SRR3884373_MERGED_FASTQ_SSU_OTU.tsv	Root-Environmental-Aquatic-Marine-Oceanic-Aphotic_zone

SRR5465806	MGYA00207268	https://www.ebi.ac.uk/metagenomics/api/v1/analyses/MGYA00207268/file/SRR5465806_MERGED_FASTQ_SSU_OTU.tsv	Root-Environmental-Aquatic-Marine-Oceanic-Aphotic_zone
SRR5465797	MGYA00207286	https://www.ebi.ac.uk/metagenomics/api/v1/analyses/MGYA00207286/file/SRR5465797_MERGED_FASTQ_SSU_OTU.tsv	Root-Environmental-Aquatic-Marine-Oceanic-Aphotic_zone
SRR3884387	MGYA00211417	https://www.ebi.ac.uk/metagenomics/api/v1/analyses/MGYA00211417/file/SRR3884387_MERGED_FASTQ_SSU_OTU.tsv	Root-Environmental-Aquatic-Marine-Oceanic-Aphotic_zone
SRR5465790	MGYA00207254	https://www.ebi.ac.uk/metagenomics/api/v1/analyses/MGYA00207254/file/SRR5465790_MERGED_FASTQ_SSU_OTU.tsv	Root-Environmental-Aquatic-Marine-Oceanic-Aphotic_zone
SRR3884380	MGYA00211425	https://www.ebi.ac.uk/metagenomics/api/v1/analyses/MGYA00211425/file/SRR3884380_MERGED_FASTQ_SSU_OTU.tsv	Root-Environmental-Aquatic-Marine-Oceanic-Aphotic_zone
SRR5465799	MGYA00207283	https://www.ebi.ac.uk/metagenomics/api/v1/analyses/MGYA00207283/file/SRR5465799_MERGED_FASTQ_SSU_OTU.tsv	Root-Environmental-Aquatic-Marine-Oceanic-Aphotic_zone
SRR5465811	MGYA00207279	https://www.ebi.ac.uk/metagenomics/api/v1/analyses/MGYA00207279/file/SRR5465811_MERGED_FASTQ_SSU_OTU.tsv	Root-Environmental-Aquatic-Marine-Oceanic-Aphotic_zone
SRR3884364	MGYA00211422	https://www.ebi.ac.uk/metagenomics/api/v1/analyses/MGYA00211422/file/SRR3884364_MERGED_FASTQ_SSU_OTU.tsv	Root-Environmental-Aquatic-Marine-Oceanic-Aphotic_zone
SRR5465816	MGYA00207256	https://www.ebi.ac.uk/metagenomics/api/v1/analyses/MGYA00207256/file/SRR5465816_MERGED_FASTQ_SSU_OTU.tsv	Root-Environmental-Aquatic-Marine-Oceanic-Aphotic_zone
SRR3884372	MGYA00211424	https://www.ebi.ac.uk/metagenomics/api/v1/analyses/MGYA00211424/file/SRR3884372_MERGED_FASTQ_SSU_OTU.tsv	Root-Environmental-Aquatic-Marine-Oceanic-Aphotic_zone
SRR5465807	MGYA00207262	https://www.ebi.ac.uk/metagenomics/api/v1/analyses/MGYA00207262/file/SRR5465807_MERGED_FASTQ_SSU_OTU.tsv	Root-Environmental-Aquatic-Marine-Oceanic-Aphotic_zone
SRR3884375	MGYA00211418	https://www.ebi.ac.uk/metagenomics/api/v1/analyses/MGYA00211418/file/SRR3884375_MERGED_FASTQ_SSU_OTU.tsv	Root-Environmental-Aquatic-Marine-Oceanic-Aphotic_zone

SRR5465800	MGYA00207263	https://www.ebi.ac.uk/metagenomics/api/v1/analyses/MGYA00207263/file/SRR5465800_MERGED_FASTQ_SSU_OTU.tsv	Root-Environmental-Aquatic-Marine-Oceanic-Aphotic_zone
SRR5465809	MGYA00207261	https://www.ebi.ac.uk/metagenomics/api/v1/analyses/MGYA00207261/file/SRR5465809_MERGED_FASTQ_SSU_OTU.tsv	Root-Environmental-Aquatic-Marine-Oceanic-Aphotic_zone
SRR5465788	MGYA00207267	https://www.ebi.ac.uk/metagenomics/api/v1/analyses/MGYA00207267/file/SRR5465788_MERGED_FASTQ_SSU_OTU.tsv	Root-Environmental-Aquatic-Marine-Oceanic-Aphotic_zone
SRR5465781	MGYA00207282	https://www.ebi.ac.uk/metagenomics/api/v1/analyses/MGYA00207282/file/SRR5465781_MERGED_FASTQ_SSU_OTU.tsv	Root-Environmental-Aquatic-Marine-Oceanic-Aphotic_zone
SRR5465786	MGYA00207272	https://www.ebi.ac.uk/metagenomics/api/v1/analyses/MGYA00207272/file/SRR5465786_MERGED_FASTQ_SSU_OTU.tsv	Root-Environmental-Aquatic-Marine-Oceanic-Aphotic_zone
SRR3884376	MGYA00211415	https://www.ebi.ac.uk/metagenomics/api/v1/analyses/MGYA00211415/file/SRR3884376_MERGED_FASTQ_SSU_OTU.tsv	Root-Environmental-Aquatic-Marine-Oceanic-Aphotic_zone
SRR5465803	MGYA00207278	https://www.ebi.ac.uk/metagenomics/api/v1/analyses/MGYA00207278/file/SRR5465803_MERGED_FASTQ_SSU_OTU.tsv	Root-Environmental-Aquatic-Marine-Oceanic-Aphotic_zone
SRR3884371	MGYA00211421	https://www.ebi.ac.uk/metagenomics/api/v1/analyses/MGYA00211421/file/SRR3884371_MERGED_FASTQ_SSU_OTU.tsv	Root-Environmental-Aquatic-Marine-Oceanic-Aphotic_zone
SRR5465804	MGYA00207274	https://www.ebi.ac.uk/metagenomics/api/v1/analyses/MGYA00207274/file/SRR5465804_MERGED_FASTQ_SSU_OTU.tsv	Root-Environmental-Aquatic-Marine-Oceanic-Aphotic_zone
SRR3884378	MGYA00211409	https://www.ebi.ac.uk/metagenomics/api/v1/analyses/MGYA00211409/file/SRR3884378_MERGED_FASTQ_SSU_OTU.tsv	Root-Environmental-Aquatic-Marine-Oceanic-Aphotic_zone
SRR5465785	MGYA00207271	https://www.ebi.ac.uk/metagenomics/api/v1/analyses/MGYA00207271/file/SRR5465785_MERGED_FASTQ_SSU_OTU.tsv	Root-Environmental-Aquatic-Marine-Oceanic-Aphotic_zone
SRR5465782	MGYA00207276	https://www.ebi.ac.uk/metagenomics/api/v1/analyses/MGYA00207276/file/SRR5465782_MERGED_FASTQ_SSU_OTU.tsv	Root-Environmental-Aquatic-Marine-Oceanic-Aphotic_zone

SRR3884383	MGYA00211420	https://www.ebi.ac.uk/metagenomics/api/v1/analyses/MGYA00211420/file/SRR3884383_MERGED_FASTQ_SSU_OTU.tsv	Root-Environmental-Aquatic-Marine-Oceanic-Aphotic_zone
SRR5465793	MGYA00207284	https://www.ebi.ac.uk/metagenomics/api/v1/analyses/MGYA00207284/file/SRR5465793_MERGED_FASTQ_SSU_OTU.tsv	Root-Environmental-Aquatic-Marine-Oceanic-Aphotic_zone
SRR3884384	MGYA00211429	https://www.ebi.ac.uk/metagenomics/api/v1/analyses/MGYA00211429/file/SRR3884384_MERGED_FASTQ_SSU_OTU.tsv	Root-Environmental-Aquatic-Marine-Oceanic-Aphotic_zone
SRR5465794	MGYA00207273	https://www.ebi.ac.uk/metagenomics/api/v1/analyses/MGYA00207273/file/SRR5465794_MERGED_FASTQ_SSU_OTU.tsv	Root-Environmental-Aquatic-Marine-Oceanic-Aphotic_zone
SRR3884369	MGYA00211423	https://www.ebi.ac.uk/metagenomics/api/v1/analyses/MGYA00211423/file/SRR3884369_MERGED_FASTQ_SSU_OTU.tsv	Root-Environmental-Aquatic-Marine-Oceanic-Aphotic_zone
SRR5465779	MGYA00207281	https://www.ebi.ac.uk/metagenomics/api/v1/analyses/MGYA00207281/file/SRR5465779_MERGED_FASTQ_SSU_OTU.tsv	Root-Environmental-Aquatic-Marine-Oceanic-Aphotic_zone
SRR5465815	MGYA00207258	https://www.ebi.ac.uk/metagenomics/api/v1/analyses/MGYA00207258/file/SRR5465815_MERGED_FASTQ_SSU_OTU.tsv	Root-Environmental-Aquatic-Marine-Oceanic-Aphotic_zone
SRR5465812	MGYA00207248	https://www.ebi.ac.uk/metagenomics/api/v1/analyses/MGYA00207248/file/SRR5465812_MERGED_FASTQ_SSU_OTU.tsv	Root-Environmental-Aquatic-Marine-Oceanic-Aphotic_zone
SRR5465783	MGYA00207255	https://www.ebi.ac.uk/metagenomics/api/v1/analyses/MGYA00207255/file/SRR5465783_MERGED_FASTQ_SSU_OTU.tsv	Root-Environmental-Aquatic-Marine-Oceanic-Aphotic_zone
SRR5465784	MGYA00207250	https://www.ebi.ac.uk/metagenomics/api/v1/analyses/MGYA00207250/file/SRR5465784_MERGED_FASTQ_SSU_OTU.tsv	Root-Environmental-Aquatic-Marine-Oceanic-Aphotic_zone
SRR3884379	MGYA00211431	https://www.ebi.ac.uk/metagenomics/api/v1/analyses/MGYA00211431/file/SRR3884379_MERGED_FASTQ_SSU_OTU.tsv	Root-Environmental-Aquatic-Marine-Oceanic-Aphotic_zone
SRR3884370	MGYA00211428	https://www.ebi.ac.uk/metagenomics/api/v1/analyses/MGYA00211428/file/SRR3884370_MERGED_FASTQ_SSU_OTU.tsv	Root-Environmental-Aquatic-Marine-Oceanic-Aphotic_zone

SRR5465805	MGYA00207251	https://www.ebi.ac.uk/metagenomics/api/v1/analyses/MGYA00207251/file/SRR5465805_MERGED_FASTQ_SSU_OTU.tsv	Root-Environmental-Aquatic-Marine-Oceanic-Aphotic_zone
SRR3884377	MGYA00211412	https://www.ebi.ac.uk/metagenomics/api/v1/analyses/MGYA00211412/file/SRR3884377_MERGED_FASTQ_SSU_OTU.tsv	Root-Environmental-Aquatic-Marine-Oceanic-Aphotic_zone
SRR5465802	MGYA00207260	https://www.ebi.ac.uk/metagenomics/api/v1/analyses/MGYA00207260/file/SRR5465802_MERGED_FASTQ_SSU_OTU.tsv	Root-Environmental-Aquatic-Marine-Oceanic-Aphotic_zone
SRR5465813	MGYA00207277	https://www.ebi.ac.uk/metagenomics/api/v1/analyses/MGYA00207277/file/SRR5465813_MERGED_FASTQ_SSU_OTU.tsv	Root-Environmental-Aquatic-Marine-Oceanic-Aphotic_zone
SRR3884366	MGYA00211411	https://www.ebi.ac.uk/metagenomics/api/v1/analyses/MGYA00211411/file/SRR3884366_MERGED_FASTQ_SSU_OTU.tsv	Root-Environmental-Aquatic-Marine-Oceanic-Aphotic_zone
SRR5465814	MGYA00207257	https://www.ebi.ac.uk/metagenomics/api/v1/analyses/MGYA00207257/file/SRR5465814_MERGED_FASTQ_SSU_OTU.tsv	Root-Environmental-Aquatic-Marine-Oceanic-Aphotic_zone
SRR5465778	MGYA00207259	https://www.ebi.ac.uk/metagenomics/api/v1/analyses/MGYA00207259/file/SRR5465778_MERGED_FASTQ_SSU_OTU.tsv	Root-Environmental-Aquatic-Marine-Oceanic-Aphotic_zone
SRR3884368	MGYA00211413	https://www.ebi.ac.uk/metagenomics/api/v1/analyses/MGYA00211413/file/SRR3884368_MERGED_FASTQ_SSU_OTU.tsv	Root-Environmental-Aquatic-Marine-Oceanic-Aphotic_zone
SRR5465795	MGYA00207253	https://www.ebi.ac.uk/metagenomics/api/v1/analyses/MGYA00207253/file/SRR5465795_MERGED_FASTQ_SSU_OTU.tsv	Root-Environmental-Aquatic-Marine-Oceanic-Aphotic_zone
SRR3884385	MGYA00211426	https://www.ebi.ac.uk/metagenomics/api/v1/analyses/MGYA00211426/file/SRR3884385_MERGED_FASTQ_SSU_OTU.tsv	Root-Environmental-Aquatic-Marine-Oceanic-Aphotic_zone
SRR5465792	MGYA00207249	https://www.ebi.ac.uk/metagenomics/api/v1/analyses/MGYA00207249/file/SRR5465792_MERGED_FASTQ_SSU_OTU.tsv	Root-Environmental-Aquatic-Marine-Oceanic-Aphotic_zone
SRR3884382	MGYA00211416	https://www.ebi.ac.uk/metagenomics/api/v1/analyses/MGYA00211416/file/SRR3884382_MERGED_FASTQ_SSU_OTU.tsv	Root-Environmental-Aquatic-Marine-Oceanic-Aphotic_zone

SRR1773438	MGYA00044363	https://www.ebi.ac.uk/metagenomics/api/v1/analyses/MGYA00044363/file/SRR1773438_FASTQ_otu.tsv	Root-Environmental-Terrestrial-Soil-Crop
SRR1525752	MGYA00150683	https://www.ebi.ac.uk/metagenomics/api/v1/analyses/MGYA00150683/file/SRR1525752_MERGED_FASTQ_SSU_OTU.tsv	Root-Host_associated-Human-Digestive_system-Oral-Periodontal_pockets
SRR1525755	MGYA00150689	https://www.ebi.ac.uk/metagenomics/api/v1/analyses/MGYA00150689/file/SRR1525755_MERGED_FASTQ_SSU_OTU.tsv	Root-Host_associated-Human-Digestive_system-Oral-Periodontal_pockets
SRR1525727	MGYA00150687	https://www.ebi.ac.uk/metagenomics/api/v1/analyses/MGYA00150687/file/SRR1525727_MERGED_FASTQ_SSU_OTU.tsv	Root-Host_associated-Human-Digestive_system-Oral-Periodontal_pockets
SRR1525720	MGYA00150686	https://www.ebi.ac.uk/metagenomics/api/v1/analyses/MGYA00150686/file/SRR1525720_MERGED_FASTQ_SSU_OTU.tsv	Root-Host_associated-Human-Digestive_system-Oral-Periodontal_pockets
SRR1525721	MGYA00150685	https://www.ebi.ac.uk/metagenomics/api/v1/analyses/MGYA00150685/file/SRR1525721_MERGED_FASTQ_SSU_OTU.tsv	Root-Host_associated-Human-Digestive_system-Oral-Periodontal_pockets
SRR1525754	MGYA00150692	https://www.ebi.ac.uk/metagenomics/api/v1/analyses/MGYA00150692/file/SRR1525754_MERGED_FASTQ_SSU_OTU.tsv	Root-Host_associated-Human-Digestive_system-Oral-Periodontal_pockets
SRR1525753	MGYA00150690	https://www.ebi.ac.uk/metagenomics/api/v1/analyses/MGYA00150690/file/SRR1525753_MERGED_FASTQ_SSU_OTU.tsv	Root-Host_associated-Human-Digestive_system-Oral-Periodontal_pockets
SRR1525750	MGYA00150688	https://www.ebi.ac.uk/metagenomics/api/v1/analyses/MGYA00150688/file/SRR1525750_MERGED_FASTQ_SSU_OTU.tsv	Root-Host_associated-Human-Digestive_system-Oral-Periodontal_pockets
SRR1525751	MGYA00150684	https://www.ebi.ac.uk/metagenomics/api/v1/analyses/MGYA00150684/file/SRR1525751_MERGED_FASTQ_SSU_OTU.tsv	Root-Host_associated-Human-Digestive_system-Oral-Periodontal_pockets
SRR1525724	MGYA00150691	https://www.ebi.ac.uk/metagenomics/api/v1/analyses/MGYA00150691/file/SRR1525724_MERGED_FASTQ_SSU_OTU.tsv	Root-Host_associated-Human-Digestive_system-Oral-Periodontal_pockets
ERR2278753	MGYA00147093	https://www.ebi.ac.uk/metagenomics/api/v1/analyses/MGYA00147093/file/ERR2278753_FASTQ_SSU_OTU.tsv	Root-Host_associated-Human-Circulatory_system

ERR2278752	MGYA00147094	https://www.ebi.ac.uk/metagenomics/api/v1/analyses/MGYA00147094/file/ERR2278752_FASTQ_SSU_OTU.tsv	Root-Host_associated-Human-Circulatory_system
SRR5003158	MGYA00414365	https://www.ebi.ac.uk/metagenomics/api/v1/analyses/MGYA00414365/file/SRR5003158_MERGED_FASTQ_SSU_OTU.tsv	Root-Environmental-Aquatic-Freshwater-Groundwater-Biofilm
SRR5003009	MGYA00414367	https://www.ebi.ac.uk/metagenomics/api/v1/analyses/MGYA00414367/file/SRR5003009_MERGED_FASTQ_SSU_OTU.tsv	Root-Environmental-Aquatic-Freshwater-Groundwater-Biofilm
SRR5002909	MGYA00413927	https://www.ebi.ac.uk/metagenomics/api/v1/analyses/MGYA00413927/file/SRR5002909_MERGED_FASTQ_SSU_OTU.tsv	Root-Environmental-Aquatic-Freshwater-Groundwater-Biofilm
SRR5003174	MGYA00414363	https://www.ebi.ac.uk/metagenomics/api/v1/analyses/MGYA00414363/file/SRR5003174_MERGED_FASTQ_SSU_OTU.tsv	Root-Environmental-Aquatic-Freshwater-Groundwater-Biofilm
SRR5003173	MGYA00414364	https://www.ebi.ac.uk/metagenomics/api/v1/analyses/MGYA00414364/file/SRR5003173_MERGED_FASTQ_SSU_OTU.tsv	Root-Environmental-Aquatic-Freshwater-Groundwater-Biofilm
SRR5002924	MGYA00413925	https://www.ebi.ac.uk/metagenomics/api/v1/analyses/MGYA00413925/file/SRR5002924_MERGED_FASTQ_SSU_OTU.tsv	Root-Environmental-Aquatic-Freshwater-Groundwater-Biofilm
SRR5002969	MGYA00414370	https://www.ebi.ac.uk/metagenomics/api/v1/analyses/MGYA00414370/file/SRR5002969_MERGED_FASTQ_SSU_OTU.tsv	Root-Environmental-Aquatic-Freshwater-Groundwater-Biofilm
SRR5002992	MGYA00412782	https://www.ebi.ac.uk/metagenomics/api/v1/analyses/MGYA00412782/file/SRR5002992_MERGED_FASTQ_SSU_OTU.tsv	Root-Environmental-Aquatic-Freshwater-Groundwater-Biofilm
SRR5003078	MGYA00412454	https://www.ebi.ac.uk/metagenomics/api/v1/analyses/MGYA00412454/file/SRR5003078_MERGED_FASTQ_SSU_OTU.tsv	Root-Environmental-Aquatic-Freshwater-Groundwater-Biofilm
SRR5002913	MGYA00414378	https://www.ebi.ac.uk/metagenomics/api/v1/analyses/MGYA00414378/file/SRR5002913_MERGED_FASTQ_SSU_OTU.tsv	Root-Environmental-Aquatic-Freshwater-Groundwater-Biofilm
SRR5003178	MGYA00414360	https://www.ebi.ac.uk/metagenomics/api/v1/analyses/MGYA00414360/file/SRR5003178_MERGED_FASTQ_SSU_OTU.tsv	Root-Environmental-Aquatic-Freshwater-Groundwater-Biofilm

ERR1582455	MGYA00352692	https://www.ebi.ac.uk/metagenomics/api/v1/analyses/MGYA00352692/file/ERR1582455_FASTQ_SSU_OTU.tsv	Root-Environmental-Aquatic-Freshwater-Lake
ERR1582827	MGYA00353064	https://www.ebi.ac.uk/metagenomics/api/v1/analyses/MGYA00353064/file/ERR1582827_FASTQ_SSU_OTU.tsv	Root-Environmental-Aquatic-Freshwater-Lake
ERR1583215	MGYA00353452	https://www.ebi.ac.uk/metagenomics/api/v1/analyses/MGYA00353452/file/ERR1583215_FASTQ_SSU_OTU.tsv	Root-Environmental-Aquatic-Freshwater-Lake
ERR1897619	MGYA00351564	https://www.ebi.ac.uk/metagenomics/api/v1/analyses/MGYA00351564/file/ERR1897619_FASTQ_SSU_OTU.tsv	Root-Environmental-Aquatic-Freshwater-Lake
ERR1843313	MGYA00102752	https://www.ebi.ac.uk/metagenomics/api/v1/analyses/MGYA00102752/file/ERR1843313_FASTQ_otu.tsv	Root-Environmental-Aquatic-Freshwater-Lake
ERR1582034	MGYA00352271	https://www.ebi.ac.uk/metagenomics/api/v1/analyses/MGYA00352271/file/ERR1582034_FASTQ_SSU_OTU.tsv	Root-Environmental-Aquatic-Freshwater-Lake
ERR1314354	MGYA00095154	https://www.ebi.ac.uk/metagenomics/api/v1/analyses/MGYA00095154/file/ERR1314354_MERGED_FASTQ_otu.tsv	Root-Environmental-Aquatic-Freshwater-Lake
ERR1582793	MGYA00353030	https://www.ebi.ac.uk/metagenomics/api/v1/analyses/MGYA00353030/file/ERR1582793_FASTQ_SSU_OTU.tsv	Root-Environmental-Aquatic-Freshwater-Lake
ERR1547093	MGYA00320804	https://www.ebi.ac.uk/metagenomics/api/v1/analyses/MGYA00320804/file/ERR1547093_FASTQ_SSU_OTU.tsv	Root-Environmental-Aquatic-Freshwater-Lake
ERR1897278	MGYA00351223	https://www.ebi.ac.uk/metagenomics/api/v1/analyses/MGYA00351223/file/ERR1897278_FASTQ_SSU_OTU.tsv	Root-Environmental-Aquatic-Freshwater-Lake
ERR1547042	MGYA00320753	https://www.ebi.ac.uk/metagenomics/api/v1/analyses/MGYA00320753/file/ERR1547042_FASTQ_SSU_OTU.tsv	Root-Environmental-Aquatic-Freshwater-Lake
ERR1582742	MGYA00352979	https://www.ebi.ac.uk/metagenomics/api/v1/analyses/MGYA00352979/file/ERR1582742_FASTQ_SSU_OTU.tsv	Root-Environmental-Aquatic-Freshwater-Lake

ERR1314385	MGYA00095186	https://www.ebi.ac.uk/metagenomics/api/v1/analyses/MGYA00095186/file/ERR1314385_MERGED_FASTQ_otu.tsv	Root-Environmental-Aquatic-Freshwater-Lake
ERR1583102	MGYA00353339	https://www.ebi.ac.uk/metagenomics/api/v1/analyses/MGYA00353339/file/ERR1583102_FASTQ_SSU_OTU.tsv	Root-Environmental-Aquatic-Freshwater-Lake
ERR1582484	MGYA00352721	https://www.ebi.ac.uk/metagenomics/api/v1/analyses/MGYA00352721/file/ERR1582484_FASTQ_SSU_OTU.tsv	Root-Environmental-Aquatic-Freshwater-Lake
ERR1582323	MGYA00352560	https://www.ebi.ac.uk/metagenomics/api/v1/analyses/MGYA00352560/file/ERR1582323_FASTQ_SSU_OTU.tsv	Root-Environmental-Aquatic-Freshwater-Lake
ERR1897456	MGYA00351401	https://www.ebi.ac.uk/metagenomics/api/v1/analyses/MGYA00351401/file/ERR1897456_FASTQ_SSU_OTU.tsv	Root-Environmental-Aquatic-Freshwater-Lake
ERR1743325	MGYA00087172	https://www.ebi.ac.uk/metagenomics/api/v1/analyses/MGYA00087172/file/ERR1743325_MERGED_FASTQ_otu.tsv	Root-Environmental-Aquatic-Freshwater-Lake
ERR1896677	MGYA00350622	https://www.ebi.ac.uk/metagenomics/api/v1/analyses/MGYA00350622/file/ERR1896677_FASTQ_SSU_OTU.tsv	Root-Environmental-Aquatic-Freshwater-Lake
ERR1897037	MGYA00350982	https://www.ebi.ac.uk/metagenomics/api/v1/analyses/MGYA00350982/file/ERR1897037_FASTQ_SSU_OTU.tsv	Root-Environmental-Aquatic-Freshwater-Lake
ERR1897741	MGYA00351686	https://www.ebi.ac.uk/metagenomics/api/v1/analyses/MGYA00351686/file/ERR1897741_FASTQ_SSU_OTU.tsv	Root-Environmental-Aquatic-Freshwater-Lake
SRR2443005	MGYA00359009	https://www.ebi.ac.uk/metagenomics/api/v1/analyses/MGYA00359009/file/SRR2443005_MERGED_FASTQ_SSU_OTU.tsv	Root-Environmental-Aquatic-Freshwater-Lake
ERR1896912	MGYA00350857	https://www.ebi.ac.uk/metagenomics/api/v1/analyses/MGYA00350857/file/ERR1896912_FASTQ_SSU_OTU.tsv	Root-Environmental-Aquatic-Freshwater-Lake
ERR1897487	MGYA00351432	https://www.ebi.ac.uk/metagenomics/api/v1/analyses/MGYA00351432/file/ERR1897487_FASTQ_SSU_OTU.tsv	Root-Environmental-Aquatic-Freshwater-Lake

ERR1897320	MGYA00351265	https://www.ebi.ac.uk/metagenomics/api/v1/analyses/MGYA00351265/file/ERR1897320_FASTQ_SSU_OTU.tsv	Root-Environmental-Aquatic-Freshwater-Lake
ERR1897665	MGYA00351610	https://www.ebi.ac.uk/metagenomics/api/v1/analyses/MGYA00351610/file/ERR1897665_FASTQ_SSU_OTU.tsv	Root-Environmental-Aquatic-Freshwater-Lake
ERR1535949	MGYA00320240	https://www.ebi.ac.uk/metagenomics/api/v1/analyses/MGYA00320240/file/ERR1535949_FASTQ_SSU_OTU.tsv	Root-Environmental-Aquatic-Freshwater-Lake
ERR1896782	MGYA00350727	https://www.ebi.ac.uk/metagenomics/api/v1/analyses/MGYA00350727/file/ERR1896782_FASTQ_SSU_OTU.tsv	Root-Environmental-Aquatic-Freshwater-Lake
ERR658177	MGYA00103508	https://www.ebi.ac.uk/metagenomics/api/v1/analyses/MGYA00103508/file/ERR658177_FASTQ_otu.tsv	Root-Environmental-Aquatic-Freshwater-Lake
ERR1582429	MGYA00352666	https://www.ebi.ac.uk/metagenomics/api/v1/analyses/MGYA00352666/file/ERR1582429_FASTQ_SSU_OTU.tsv	Root-Environmental-Aquatic-Freshwater-Lake
ERR1583269	MGYA00353506	https://www.ebi.ac.uk/metagenomics/api/v1/analyses/MGYA00353506/file/ERR1583269_FASTQ_SSU_OTU.tsv	Root-Environmental-Aquatic-Freshwater-Lake
ERR1896836	MGYA00350781	https://www.ebi.ac.uk/metagenomics/api/v1/analyses/MGYA00350781/file/ERR1896836_FASTQ_SSU_OTU.tsv	Root-Environmental-Aquatic-Freshwater-Lake
ERR1897204	MGYA00351149	https://www.ebi.ac.uk/metagenomics/api/v1/analyses/MGYA00351149/file/ERR1897204_FASTQ_SSU_OTU.tsv	Root-Environmental-Aquatic-Freshwater-Lake
ERR1582048	MGYA00352285	https://www.ebi.ac.uk/metagenomics/api/v1/analyses/MGYA00352285/file/ERR1582048_FASTQ_SSU_OTU.tsv	Root-Environmental-Aquatic-Freshwater-Lake
ERR1582099	MGYA00352336	https://www.ebi.ac.uk/metagenomics/api/v1/analyses/MGYA00352336/file/ERR1582099_FASTQ_SSU_OTU.tsv	Root-Environmental-Aquatic-Freshwater-Lake
SRR7081369	MGYA00425937	https://www.ebi.ac.uk/metagenomics/api/v1/analyses/MGYA00425937/file/SRR7081369_MERGED_FASTQ_SSU_OTU.tsv	Root-Environmental-Aquatic-Freshwater-Lake

ERR1897572	MGYA00351517	https://www.ebi.ac.uk/metagenomics/api/v1/analyses/MGYA00351517/file/ERR1897572_FASTQ_SSU_OTU.tsv	Root-Environmental-Aquatic-Freshwater-Lake
ERR1896753	MGYA00350698	https://www.ebi.ac.uk/metagenomics/api/v1/analyses/MGYA00350698/file/ERR1896753_FASTQ_SSU_OTU.tsv	Root-Environmental-Aquatic-Freshwater-Lake
ERR1897113	MGYA00351058	https://www.ebi.ac.uk/metagenomics/api/v1/analyses/MGYA00351058/file/ERR1897113_FASTQ_SSU_OTU.tsv	Root-Environmental-Aquatic-Freshwater-Lake
ERR1743359	MGYA00087179	https://www.ebi.ac.uk/metagenomics/api/v1/analyses/MGYA00087179/file/ERR1743359_MERGED_FASTQ_otu.tsv	Root-Environmental-Aquatic-Freshwater-Lake
ERR1582666	MGYA00352903	https://www.ebi.ac.uk/metagenomics/api/v1/analyses/MGYA00352903/file/ERR1582666_FASTQ_SSU_OTU.tsv	Root-Environmental-Aquatic-Freshwater-Lake
ERR1583026	MGYA00353263	https://www.ebi.ac.uk/metagenomics/api/v1/analyses/MGYA00353263/file/ERR1583026_FASTQ_SSU_OTU.tsv	Root-Environmental-Aquatic-Freshwater-Lake
ERR1583447	MGYA00353684	https://www.ebi.ac.uk/metagenomics/api/v1/analyses/MGYA00353684/file/ERR1583447_FASTQ_SSU_OTU.tsv	Root-Environmental-Aquatic-Freshwater-Lake
ERR1582207	MGYA00352444	https://www.ebi.ac.uk/metagenomics/api/v1/analyses/MGYA00352444/file/ERR1582207_FASTQ_SSU_OTU.tsv	Root-Environmental-Aquatic-Freshwater-Lake
ERR1582903	MGYA00353140	https://www.ebi.ac.uk/metagenomics/api/v1/analyses/MGYA00353140/file/ERR1582903_FASTQ_SSU_OTU.tsv	Root-Environmental-Aquatic-Freshwater-Lake
ERR1582571	MGYA00352808	https://www.ebi.ac.uk/metagenomics/api/v1/analyses/MGYA00352808/file/ERR1582571_FASTQ_SSU_OTU.tsv	Root-Environmental-Aquatic-Freshwater-Lake
ERR1583496	MGYA00353733	https://www.ebi.ac.uk/metagenomics/api/v1/analyses/MGYA00353733/file/ERR1583496_FASTQ_SSU_OTU.tsv	Root-Environmental-Aquatic-Freshwater-Lake
ERR1583331	MGYA00353568	https://www.ebi.ac.uk/metagenomics/api/v1/analyses/MGYA00353568/file/ERR1583331_FASTQ_SSU_OTU.tsv	Root-Environmental-Aquatic-Freshwater-Lake

ERR1582110	MGYA00352347	https://www.ebi.ac.uk/metagenomics/api/v1/analyses/MGYA00352347/file/ERR1582110_FASTQ_SSU_OTU.tsv	Root-Environmental-Aquatic-Freshwater-Lake
ERR1743388	MGYA00087184	https://www.ebi.ac.uk/metagenomics/api/v1/analyses/MGYA00087184/file/ERR1743388_MERGED_FASTQ_otu.tsv	Root-Environmental-Aquatic-Freshwater-Lake
ERR589329	MGYA00103412	https://www.ebi.ac.uk/metagenomics/api/v1/analyses/MGYA00103412/file/ERR589329_FASTQ_otu.tsv	Root-Environmental-Aquatic-Freshwater-Lake
ERR1896785	MGYA00350730	https://www.ebi.ac.uk/metagenomics/api/v1/analyses/MGYA00350730/file/ERR1896785_FASTQ_SSU_OTU.tsv	Root-Environmental-Aquatic-Freshwater-Lake
ERR1897662	MGYA00351607	https://www.ebi.ac.uk/metagenomics/api/v1/analyses/MGYA00351607/file/ERR1897662_FASTQ_SSU_OTU.tsv	Root-Environmental-Aquatic-Freshwater-Lake
ERR1333519	MGYA00107594	https://www.ebi.ac.uk/metagenomics/api/v1/analyses/MGYA00107594/file/ERR1333519_FASTQ_otu.tsv	Root-Environmental-Aquatic-Freshwater-Lake
ERR1582389	MGYA00352626	https://www.ebi.ac.uk/metagenomics/api/v1/analyses/MGYA00352626/file/ERR1582389_FASTQ_SSU_OTU.tsv	Root-Environmental-Aquatic-Freshwater-Lake
ERR1897203	MGYA00351148	https://www.ebi.ac.uk/metagenomics/api/v1/analyses/MGYA00351148/file/ERR1897203_FASTQ_SSU_OTU.tsv	Root-Environmental-Aquatic-Freshwater-Lake
ERR1896831	MGYA00350776	https://www.ebi.ac.uk/metagenomics/api/v1/analyses/MGYA00350776/file/ERR1896831_FASTQ_SSU_OTU.tsv	Root-Environmental-Aquatic-Freshwater-Lake
ERR1824222	MGYA00099975	https://www.ebi.ac.uk/metagenomics/api/v1/analyses/MGYA00099975/file/ERR1824222_FASTQ_otu.tsv	Root-Environmental-Aquatic-Freshwater-Lake
ERR1583179	MGYA00353416	https://www.ebi.ac.uk/metagenomics/api/v1/analyses/MGYA00353416/file/ERR1583179_FASTQ_SSU_OTU.tsv	Root-Environmental-Aquatic-Freshwater-Lake
ERR1582739	MGYA00352976	https://www.ebi.ac.uk/metagenomics/api/v1/analyses/MGYA00352976/file/ERR1582739_FASTQ_SSU_OTU.tsv	Root-Environmental-Aquatic-Freshwater-Lake

ERR1547039	MGYA00320750	https://www.ebi.ac.uk/metagenomics/api/v1/analyses/MGYA00320750/file/ERR1547039_FASTQ_SSU_OTU.tsv	Root-Environmental-Aquatic-Freshwater-Lake
ERR1897575	MGYA00351520	https://www.ebi.ac.uk/metagenomics/api/v1/analyses/MGYA00351520/file/ERR1897575_FASTQ_SSU_OTU.tsv	Root-Environmental-Aquatic-Freshwater-Lake
ERR1582358	MGYA00352595	https://www.ebi.ac.uk/metagenomics/api/v1/analyses/MGYA00352595/file/ERR1582358_FASTQ_SSU_OTU.tsv	Root-Environmental-Aquatic-Freshwater-Lake
ERR1583518	MGYA00353755	https://www.ebi.ac.uk/metagenomics/api/v1/analyses/MGYA00353755/file/ERR1583518_FASTQ_SSU_OTU.tsv	Root-Environmental-Aquatic-Freshwater-Lake
ERR1897114	MGYA00351059	https://www.ebi.ac.uk/metagenomics/api/v1/analyses/MGYA00351059/file/ERR1897114_FASTQ_SSU_OTU.tsv	Root-Environmental-Aquatic-Freshwater-Lake
ERR1896754	MGYA00350699	https://www.ebi.ac.uk/metagenomics/api/v1/analyses/MGYA00350699/file/ERR1896754_FASTQ_SSU_OTU.tsv	Root-Environmental-Aquatic-Freshwater-Lake
SRR7081236	MGYA00425695	https://www.ebi.ac.uk/metagenomics/api/v1/analyses/MGYA00425695/file/SRR7081236_MERGED_FASTQ_SSU_OTU.tsv	Root-Environmental-Aquatic-Freshwater-Lake
ERR1583021	MGYA00353258	https://www.ebi.ac.uk/metagenomics/api/v1/analyses/MGYA00353258/file/ERR1583021_FASTQ_SSU_OTU.tsv	Root-Environmental-Aquatic-Freshwater-Lake
ERR1582661	MGYA00352898	https://www.ebi.ac.uk/metagenomics/api/v1/analyses/MGYA00352898/file/ERR1582661_FASTQ_SSU_OTU.tsv	Root-Environmental-Aquatic-Freshwater-Lake
ERR1582200	MGYA00352437	https://www.ebi.ac.uk/metagenomics/api/v1/analyses/MGYA00352437/file/ERR1582200_FASTQ_SSU_OTU.tsv	Root-Environmental-Aquatic-Freshwater-Lake
ERR1583440	MGYA00353677	https://www.ebi.ac.uk/metagenomics/api/v1/analyses/MGYA00353677/file/ERR1583440_FASTQ_SSU_OTU.tsv	Root-Environmental-Aquatic-Freshwater-Lake
ERR1583336	MGYA00353573	https://www.ebi.ac.uk/metagenomics/api/v1/analyses/MGYA00353573/file/ERR1583336_FASTQ_SSU_OTU.tsv	Root-Environmental-Aquatic-Freshwater-Lake

ERR1583491	MGYA00353728	https://www.ebi.ac.uk/metagenomics/api/v1/analyses/MGYA00353728/file/ERR1583491_FASTQ_SSU_OTU.tsv	Root-Environmental-Aquatic-Freshwater-Lake
ERR1582576	MGYA00352813	https://www.ebi.ac.uk/metagenomics/api/v1/analyses/MGYA00352813/file/ERR1582576_FASTQ_SSU_OTU.tsv	Root-Environmental-Aquatic-Freshwater-Lake
ERR1582904	MGYA00353141	https://www.ebi.ac.uk/metagenomics/api/v1/analyses/MGYA00353141/file/ERR1582904_FASTQ_SSU_OTU.tsv	Root-Environmental-Aquatic-Freshwater-Lake
ERR1582117	MGYA00352354	https://www.ebi.ac.uk/metagenomics/api/v1/analyses/MGYA00352354/file/ERR1582117_FASTQ_SSU_OTU.tsv	Root-Environmental-Aquatic-Freshwater-Lake
ERR1896969	MGYA00350914	https://www.ebi.ac.uk/metagenomics/api/v1/analyses/MGYA00350914/file/ERR1896969_FASTQ_SSU_OTU.tsv	Root-Environmental-Aquatic-Freshwater-Lake
ERR1583212	MGYA00353449	https://www.ebi.ac.uk/metagenomics/api/v1/analyses/MGYA00353449/file/ERR1583212_FASTQ_SSU_OTU.tsv	Root-Environmental-Aquatic-Freshwater-Lake
ERR1582820	MGYA00353057	https://www.ebi.ac.uk/metagenomics/api/v1/analyses/MGYA00353057/file/ERR1582820_FASTQ_SSU_OTU.tsv	Root-Environmental-Aquatic-Freshwater-Lake
ERR1582452	MGYA00352689	https://www.ebi.ac.uk/metagenomics/api/v1/analyses/MGYA00352689/file/ERR1582452_FASTQ_SSU_OTU.tsv	Root-Environmental-Aquatic-Freshwater-Lake
ERR1314353	MGYA00095162	https://www.ebi.ac.uk/metagenomics/api/v1/analyses/MGYA00095162/file/ERR1314353_MERGED_FASTQ_otu.tsv	Root-Environmental-Aquatic-Freshwater-Lake
ERR1582794	MGYA00353031	https://www.ebi.ac.uk/metagenomics/api/v1/analyses/MGYA00353031/file/ERR1582794_FASTQ_SSU_OTU.tsv	Root-Environmental-Aquatic-Freshwater-Lake
ERR1582033	MGYA00352270	https://www.ebi.ac.uk/metagenomics/api/v1/analyses/MGYA00352270/file/ERR1582033_FASTQ_SSU_OTU.tsv	Root-Environmental-Aquatic-Freshwater-Lake
ERR1547094	MGYA00320805	https://www.ebi.ac.uk/metagenomics/api/v1/analyses/MGYA00320805/file/ERR1547094_FASTQ_SSU_OTU.tsv	Root-Environmental-Aquatic-Freshwater-Lake

ERR1547045	MGYA00320756	https://www.ebi.ac.uk/metagenomics/api/v1/analyses/MGYA00320756/file/ERR1547045_FASTQ_SSU_OTU.tsv	Root-Environmental-Aquatic-Freshwater-Lake
ERR1897509	MGYA00351454	https://www.ebi.ac.uk/metagenomics/api/v1/analyses/MGYA00351454/file/ERR1897509_FASTQ_SSU_OTU.tsv	Root-Environmental-Aquatic-Freshwater-Lake
ERR1583105	MGYA00353342	https://www.ebi.ac.uk/metagenomics/api/v1/analyses/MGYA00353342/file/ERR1583105_FASTQ_SSU_OTU.tsv	Root-Environmental-Aquatic-Freshwater-Lake
ERR1582745	MGYA00352982	https://www.ebi.ac.uk/metagenomics/api/v1/analyses/MGYA00352982/file/ERR1582745_FASTQ_SSU_OTU.tsv	Root-Environmental-Aquatic-Freshwater-Lake
ERR1314382	MGYA00095211	https://www.ebi.ac.uk/metagenomics/api/v1/analyses/MGYA00095211/file/ERR1314382_MERGED_FASTQ_otu.tsv	Root-Environmental-Aquatic-Freshwater-Lake
ERR1897168	MGYA00351113	https://www.ebi.ac.uk/metagenomics/api/v1/analyses/MGYA00351113/file/ERR1897168_FASTQ_SSU_OTU.tsv	Root-Environmental-Aquatic-Freshwater-Lake
ERR1896728	MGYA00350673	https://www.ebi.ac.uk/metagenomics/api/v1/analyses/MGYA00350673/file/ERR1896728_FASTQ_SSU_OTU.tsv	Root-Environmental-Aquatic-Freshwater-Lake
ERR1582324	MGYA00352561	https://www.ebi.ac.uk/metagenomics/api/v1/analyses/MGYA00352561/file/ERR1582324_FASTQ_SSU_OTU.tsv	Root-Environmental-Aquatic-Freshwater-Lake
ERR1582483	MGYA00352720	https://www.ebi.ac.uk/metagenomics/api/v1/analyses/MGYA00352720/file/ERR1582483_FASTQ_SSU_OTU.tsv	Root-Environmental-Aquatic-Freshwater-Lake
ERR1743322	MGYA00087128	https://www.ebi.ac.uk/metagenomics/api/v1/analyses/MGYA00087128/file/ERR1743322_MERGED_FASTQ_otu.tsv	Root-Environmental-Aquatic-Freshwater-Lake
ERR1897451	MGYA00351396	https://www.ebi.ac.uk/metagenomics/api/v1/analyses/MGYA00351396/file/ERR1897451_FASTQ_SSU_OTU.tsv	Root-Environmental-Aquatic-Freshwater-Lake
ERR1897030	MGYA00350975	https://www.ebi.ac.uk/metagenomics/api/v1/analyses/MGYA00350975/file/ERR1897030_FASTQ_SSU_OTU.tsv	Root-Environmental-Aquatic-Freshwater-Lake

ERR1896670	MGYA00350615	https://www.ebi.ac.uk/metagenomics/api/v1/analyses/MGYA00350615/file/ERR1896670_FASTQ_SSU_OTU.tsv	Root-Environmental-Aquatic-Freshwater-Lake
ERR1897746	MGYA00351691	https://www.ebi.ac.uk/metagenomics/api/v1/analyses/MGYA00351691/file/ERR1897746_FASTQ_SSU_OTU.tsv	Root-Environmental-Aquatic-Freshwater-Lake
SRR2443002	MGYA00359006	https://www.ebi.ac.uk/metagenomics/api/v1/analyses/MGYA00359006/file/SRR2443002_MERGED_FASTQ_SSU_OTU.tsv	Root-Environmental-Aquatic-Freshwater-Lake
ERR1582978	MGYA00353215	https://www.ebi.ac.uk/metagenomics/api/v1/analyses/MGYA00353215/file/ERR1582978_FASTQ_SSU_OTU.tsv	Root-Environmental-Aquatic-Freshwater-Lake
ERR1897327	MGYA00351272	https://www.ebi.ac.uk/metagenomics/api/v1/analyses/MGYA00351272/file/ERR1897327_FASTQ_SSU_OTU.tsv	Root-Environmental-Aquatic-Freshwater-Lake
ERR1897480	MGYA00351425	https://www.ebi.ac.uk/metagenomics/api/v1/analyses/MGYA00351425/file/ERR1897480_FASTQ_SSU_OTU.tsv	Root-Environmental-Aquatic-Freshwater-Lake
ERR1896915	MGYA00350860	https://www.ebi.ac.uk/metagenomics/api/v1/analyses/MGYA00350860/file/ERR1896915_FASTQ_SSU_OTU.tsv	Root-Environmental-Aquatic-Freshwater-Lake
ERR1582852	MGYA00353089	https://www.ebi.ac.uk/metagenomics/api/v1/analyses/MGYA00353089/file/ERR1582852_FASTQ_SSU_OTU.tsv	Root-Environmental-Aquatic-Freshwater-Lake
ERR1582420	MGYA00352657	https://www.ebi.ac.uk/metagenomics/api/v1/analyses/MGYA00352657/file/ERR1582420_FASTQ_SSU_OTU.tsv	Root-Environmental-Aquatic-Freshwater-Lake
ERR1582387	MGYA00352624	https://www.ebi.ac.uk/metagenomics/api/v1/analyses/MGYA00352624/file/ERR1582387_FASTQ_SSU_OTU.tsv	Root-Environmental-Aquatic-Freshwater-Lake
ERR1583260	MGYA00353497	https://www.ebi.ac.uk/metagenomics/api/v1/analyses/MGYA00353497/file/ERR1583260_FASTQ_SSU_OTU.tsv	Root-Environmental-Aquatic-Freshwater-Lake
ERR1333517	MGYA00107590	https://www.ebi.ac.uk/metagenomics/api/v1/analyses/MGYA00107590/file/ERR1333517_FASTQ_otu.tsv	Root-Environmental-Aquatic-Freshwater-Lake

ERR1582041	MGYA00352278	https://www.ebi.ac.uk/metagenomics/api/v1/analyses/MGYA00352278/file/ERR1582041_FASTQ_SSU_OTU.tsv	Root-Environmental-Aquatic-Freshwater-Lake
ERR1547037	MGYA00320748	https://www.ebi.ac.uk/metagenomics/api/v1/analyses/MGYA00320748/file/ERR1547037_FASTQ_SSU_OTU.tsv	Root-Environmental-Aquatic-Freshwater-Lake
ERR1582090	MGYA00352327	https://www.ebi.ac.uk/metagenomics/api/v1/analyses/MGYA00352327/file/ERR1582090_FASTQ_SSU_OTU.tsv	Root-Environmental-Aquatic-Freshwater-Lake
ERR1582737	MGYA00352974	https://www.ebi.ac.uk/metagenomics/api/v1/analyses/MGYA00352974/file/ERR1582737_FASTQ_SSU_OTU.tsv	Root-Environmental-Aquatic-Freshwater-Lake
ERR1583177	MGYA00353414	https://www.ebi.ac.uk/metagenomics/api/v1/analyses/MGYA00353414/file/ERR1583177_FASTQ_SSU_OTU.tsv	Root-Environmental-Aquatic-Freshwater-Lake
ERR1535991	MGYA00320282	https://www.ebi.ac.uk/metagenomics/api/v1/analyses/MGYA00320282/file/ERR1535991_FASTQ_SSU_OTU.tsv	Root-Environmental-Aquatic-Freshwater-Lake
ERR1583516	MGYA00353753	https://www.ebi.ac.uk/metagenomics/api/v1/analyses/MGYA00353753/file/ERR1583516_FASTQ_SSU_OTU.tsv	Root-Environmental-Aquatic-Freshwater-Lake
ERR1582883	MGYA00353120	https://www.ebi.ac.uk/metagenomics/api/v1/analyses/MGYA00353120/file/ERR1582883_FASTQ_SSU_OTU.tsv	Root-Environmental-Aquatic-Freshwater-Lake
ERR1429273	MGYA00107626	https://www.ebi.ac.uk/metagenomics/api/v1/analyses/MGYA00107626/file/ERR1429273_FASTQ_otu.tsv	Root-Environmental-Aquatic-Freshwater-Lake
ERR1582356	MGYA00352593	https://www.ebi.ac.uk/metagenomics/api/v1/analyses/MGYA00352593/file/ERR1582356_FASTQ_SSU_OTU.tsv	Root-Environmental-Aquatic-Freshwater-Lake
ERR1897423	MGYA00351368	https://www.ebi.ac.uk/metagenomics/api/v1/analyses/MGYA00351368/file/ERR1897423_FASTQ_SSU_OTU.tsv	Root-Environmental-Aquatic-Freshwater-Lake
ERR1897384	MGYA00351329	https://www.ebi.ac.uk/metagenomics/api/v1/analyses/MGYA00351329/file/ERR1897384_FASTQ_SSU_OTU.tsv	Root-Environmental-Aquatic-Freshwater-Lake

ERR1743350	MGYA00087163	https://www.ebi.ac.uk/metagenomics/api/v1/analyses/MGYA00087163/file/ERR1743350_MERGED_FASTQ_otu.tsv	Root-Environmental-Aquatic-Freshwater-Lake
SRR2442955	MGYA00358959	https://www.ebi.ac.uk/metagenomics/api/v1/analyses/MGYA00358959/file/SRR2442955_MERGED_FASTQ_SSU_OTU.tsv	Root-Environmental-Aquatic-Freshwater-Lake
ERR1897042	MGYA00350987	https://www.ebi.ac.uk/metagenomics/api/v1/analyses/MGYA00350987/file/ERR1897042_FASTQ_SSU_OTU.tsv	Root-Environmental-Aquatic-Freshwater-Lake
ERR1897093	MGYA00351038	https://www.ebi.ac.uk/metagenomics/api/v1/analyses/MGYA00351038/file/ERR1897093_FASTQ_SSU_OTU.tsv	Root-Environmental-Aquatic-Freshwater-Lake
ERR1897734	MGYA00351679	https://www.ebi.ac.uk/metagenomics/api/v1/analyses/MGYA00351679/file/ERR1897734_FASTQ_SSU_OTU.tsv	Root-Environmental-Aquatic-Freshwater-Lake
ERR1582578	MGYA00352815	https://www.ebi.ac.uk/metagenomics/api/v1/analyses/MGYA00352815/file/ERR1582578_FASTQ_SSU_OTU.tsv	Root-Environmental-Aquatic-Freshwater-Lake
ERR1583338	MGYA00353575	https://www.ebi.ac.uk/metagenomics/api/v1/analyses/MGYA00353575/file/ERR1583338_FASTQ_SSU_OTU.tsv	Root-Environmental-Aquatic-Freshwater-Lake
ERR1743381	MGYA00087134	https://www.ebi.ac.uk/metagenomics/api/v1/analyses/MGYA00087134/file/ERR1743381_MERGED_FASTQ_otu.tsv	Root-Environmental-Aquatic-Freshwater-Lake
SRR2442984	MGYA00358988	https://www.ebi.ac.uk/metagenomics/api/v1/analyses/MGYA00358988/file/SRR2442984_MERGED_FASTQ_SSU_OTU.tsv	Root-Environmental-Aquatic-Freshwater-Lake
ERR1896967	MGYA00350912	https://www.ebi.ac.uk/metagenomics/api/v1/analyses/MGYA00350912/file/ERR1896967_FASTQ_SSU_OTU.tsv	Root-Environmental-Aquatic-Freshwater-Lake
ERR589320	MGYA00103413	https://www.ebi.ac.uk/metagenomics/api/v1/analyses/MGYA00103413/file/ERR589320_FASTQ_otu.tsv	Root-Environmental-Aquatic-Freshwater-Lake
ERR1897355	MGYA00351300	https://www.ebi.ac.uk/metagenomics/api/v1/analyses/MGYA00351300/file/ERR1897355_FASTQ_SSU_OTU.tsv	Root-Environmental-Aquatic-Freshwater-Lake

ERR1582119	MGYA00352356	https://www.ebi.ac.uk/metagenomics/api/v1/analyses/MGYA00352356/file/ERR1582119_FASTQ_SSU_OTU.tsv	Root-Environmental-Aquatic-Freshwater-Lake
ERR1897610	MGYA00351555	https://www.ebi.ac.uk/metagenomics/api/v1/analyses/MGYA00351555/file/ERR1897610_FASTQ_SSU_OTU.tsv	Root-Environmental-Aquatic-Freshwater-Lake
ERR1896843	MGYA00350788	https://www.ebi.ac.uk/metagenomics/api/v1/analyses/MGYA00350788/file/ERR1896843_FASTQ_SSU_OTU.tsv	Root-Environmental-Aquatic-Freshwater-Lake
ERR1897271	MGYA00351216	https://www.ebi.ac.uk/metagenomics/api/v1/analyses/MGYA00351216/file/ERR1897271_FASTQ_SSU_OTU.tsv	Root-Environmental-Aquatic-Freshwater-Lake
ERR658215	MGYA00103485	https://www.ebi.ac.uk/metagenomics/api/v1/analyses/MGYA00103485/file/ERR658215_FASTQ_otu.tsv	Root-Environmental-Aquatic-Freshwater-Lake
ERR1897507	MGYA00351452	https://www.ebi.ac.uk/metagenomics/api/v1/analyses/MGYA00351452/file/ERR1897507_FASTQ_SSU_OTU.tsv	Root-Environmental-Aquatic-Freshwater-Lake
ERR1896892	MGYA00350837	https://www.ebi.ac.uk/metagenomics/api/v1/analyses/MGYA00350837/file/ERR1896892_FASTQ_SSU_OTU.tsv	Root-Environmental-Aquatic-Freshwater-Lake
ERR1743274	MGYA00087165	https://www.ebi.ac.uk/metagenomics/api/v1/analyses/MGYA00087165/file/ERR1743274_MERGED_FASTQ_otu.tsv	Root-Environmental-Aquatic-Freshwater-Lake
ERR1896726	MGYA00350671	https://www.ebi.ac.uk/metagenomics/api/v1/analyses/MGYA00350671/file/ERR1896726_FASTQ_SSU_OTU.tsv	Root-Environmental-Aquatic-Freshwater-Lake
ERR1897166	MGYA00351111	https://www.ebi.ac.uk/metagenomics/api/v1/analyses/MGYA00351111/file/ERR1897166_FASTQ_SSU_OTU.tsv	Root-Environmental-Aquatic-Freshwater-Lake
ERR1547113	MGYA00320824	https://www.ebi.ac.uk/metagenomics/api/v1/analyses/MGYA00320824/file/ERR1547113_FASTQ_SSU_OTU.tsv	Root-Environmental-Aquatic-Freshwater-Lake
ERR1582613	MGYA00352850	https://www.ebi.ac.uk/metagenomics/api/v1/analyses/MGYA00352850/file/ERR1582613_FASTQ_SSU_OTU.tsv	Root-Environmental-Aquatic-Freshwater-Lake

ERR1583053	MGYA00353290	https://www.ebi.ac.uk/metagenomics/api/v1/analyses/MGYA00353290/file/ERR1583053_FASTQ_SSU_OTU.tsv	Root-Environmental-Aquatic-Freshwater-Lake
ERR1583432	MGYA00353669	https://www.ebi.ac.uk/metagenomics/api/v1/analyses/MGYA00353669/file/ERR1583432_FASTQ_SSU_OTU.tsv	Root-Environmental-Aquatic-Freshwater-Lake
ERR1583395	MGYA00353632	https://www.ebi.ac.uk/metagenomics/api/v1/analyses/MGYA00353632/file/ERR1583395_FASTQ_SSU_OTU.tsv	Root-Environmental-Aquatic-Freshwater-Lake
ERR1582272	MGYA00352509	https://www.ebi.ac.uk/metagenomics/api/v1/analyses/MGYA00352509/file/ERR1582272_FASTQ_SSU_OTU.tsv	Root-Environmental-Aquatic-Freshwater-Lake
ERR1582504	MGYA00352741	https://www.ebi.ac.uk/metagenomics/api/v1/analyses/MGYA00352741/file/ERR1582504_FASTQ_SSU_OTU.tsv	Root-Environmental-Aquatic-Freshwater-Lake
ERR1582976	MGYA00353213	https://www.ebi.ac.uk/metagenomics/api/v1/analyses/MGYA00353213/file/ERR1582976_FASTQ_SSU_OTU.tsv	Root-Environmental-Aquatic-Freshwater-Lake
ERR1583344	MGYA00353581	https://www.ebi.ac.uk/metagenomics/api/v1/analyses/MGYA00353581/file/ERR1583344_FASTQ_SSU_OTU.tsv	Root-Environmental-Aquatic-Freshwater-Lake
ERR1897748	MGYA00351693	https://www.ebi.ac.uk/metagenomics/api/v1/analyses/MGYA00351693/file/ERR1897748_FASTQ_SSU_OTU.tsv	Root-Environmental-Aquatic-Freshwater-Lake
SRR7081153	MGYA00413196	https://www.ebi.ac.uk/metagenomics/api/v1/analyses/MGYA00413196/file/SRR7081153_MERGED_FASTQ_SSU_OTU.tsv	Root-Environmental-Aquatic-Freshwater-Lake
ERR1583082	MGYA00353319	https://www.ebi.ac.uk/metagenomics/api/v1/analyses/MGYA00353319/file/ERR1583082_FASTQ_SSU_OTU.tsv	Root-Environmental-Aquatic-Freshwater-Lake
ERR1582165	MGYA00352402	https://www.ebi.ac.uk/metagenomics/api/v1/analyses/MGYA00352402/file/ERR1582165_FASTQ_SSU_OTU.tsv	Root-Environmental-Aquatic-Freshwater-Lake
ERR711863	MGYA00098277	https://www.ebi.ac.uk/metagenomics/api/v1/analyses/MGYA00098277/file/ERR711863_MERGED_FASTQ_otu.tsv	Root-Environmental-Aquatic-Freshwater-Lake

ERR1897329	MGYA00351274	https://www.ebi.ac.uk/metagenomics/api/v1/analyses/MGYA00351274/file/ERR1897329_FASTQ_SSU_OTU.tsv	Root-Environmental-Aquatic-Freshwater-Lake
ERR1897617	MGYA00351562	https://www.ebi.ac.uk/metagenomics/api/v1/analyses/MGYA00351562/file/ERR1897617_FASTQ_SSU_OTU.tsv	Root-Environmental-Aquatic-Freshwater-Lake
ERR1582829	MGYA00353066	https://www.ebi.ac.uk/metagenomics/api/v1/analyses/MGYA00353066/file/ERR1582829_FASTQ_SSU_OTU.tsv	Root-Environmental-Aquatic-Freshwater-Lake
ERR1897276	MGYA00351221	https://www.ebi.ac.uk/metagenomics/api/v1/analyses/MGYA00351221/file/ERR1897276_FASTQ_SSU_OTU.tsv	Root-Environmental-Aquatic-Freshwater-Lake
ERR1896844	MGYA00350789	https://www.ebi.ac.uk/metagenomics/api/v1/analyses/MGYA00350789/file/ERR1896844_FASTQ_SSU_OTU.tsv	Root-Environmental-Aquatic-Freshwater-Lake
ERR658212	MGYA00103489	https://www.ebi.ac.uk/metagenomics/api/v1/analyses/MGYA00103489/file/ERR658212_FASTQ_otu.tsv	Root-Environmental-Aquatic-Freshwater-Lake
ERR1896895	MGYA00350840	https://www.ebi.ac.uk/metagenomics/api/v1/analyses/MGYA00350840/file/ERR1896895_FASTQ_SSU_OTU.tsv	Root-Environmental-Aquatic-Freshwater-Lake
ERR1897500	MGYA00351445	https://www.ebi.ac.uk/metagenomics/api/v1/analyses/MGYA00351445/file/ERR1897500_FASTQ_SSU_OTU.tsv	Root-Environmental-Aquatic-Freshwater-Lake
ERR1897161	MGYA00351106	https://www.ebi.ac.uk/metagenomics/api/v1/analyses/MGYA00351106/file/ERR1897161_FASTQ_SSU_OTU.tsv	Root-Environmental-Aquatic-Freshwater-Lake
ERR1896721	MGYA00350666	https://www.ebi.ac.uk/metagenomics/api/v1/analyses/MGYA00350666/file/ERR1896721_FASTQ_SSU_OTU.tsv	Root-Environmental-Aquatic-Freshwater-Lake
ERR1547114	MGYA00320825	https://www.ebi.ac.uk/metagenomics/api/v1/analyses/MGYA00320825/file/ERR1547114_FASTQ_SSU_OTU.tsv	Root-Environmental-Aquatic-Freshwater-Lake
ERR1897458	MGYA00351403	https://www.ebi.ac.uk/metagenomics/api/v1/analyses/MGYA00351403/file/ERR1897458_FASTQ_SSU_OTU.tsv	Root-Environmental-Aquatic-Freshwater-Lake

ERR1583054	MGYA00353291	https://www.ebi.ac.uk/metagenomics/api/v1/analyses/MGYA00353291/file/ERR1583054_FASTQ_SSU_OTU.tsv	Root-Environmental-Aquatic-Freshwater-Lake
ERR1582614	MGYA00352851	https://www.ebi.ac.uk/metagenomics/api/v1/analyses/MGYA00352851/file/ERR1582614_FASTQ_SSU_OTU.tsv	Root-Environmental-Aquatic-Freshwater-Lake
ERR1897039	MGYA00350984	https://www.ebi.ac.uk/metagenomics/api/v1/analyses/MGYA00350984/file/ERR1897039_FASTQ_SSU_OTU.tsv	Root-Environmental-Aquatic-Freshwater-Lake
ERR1896679	MGYA00350624	https://www.ebi.ac.uk/metagenomics/api/v1/analyses/MGYA00350624/file/ERR1896679_FASTQ_SSU_OTU.tsv	Root-Environmental-Aquatic-Freshwater-Lake
ERR1582275	MGYA00352512	https://www.ebi.ac.uk/metagenomics/api/v1/analyses/MGYA00352512/file/ERR1582275_FASTQ_SSU_OTU.tsv	Root-Environmental-Aquatic-Freshwater-Lake
ERR1583392	MGYA00353629	https://www.ebi.ac.uk/metagenomics/api/v1/analyses/MGYA00353629/file/ERR1583392_FASTQ_SSU_OTU.tsv	Root-Environmental-Aquatic-Freshwater-Lake
ERR1583435	MGYA00353672	https://www.ebi.ac.uk/metagenomics/api/v1/analyses/MGYA00353672/file/ERR1583435_FASTQ_SSU_OTU.tsv	Root-Environmental-Aquatic-Freshwater-Lake
ERR1583343	MGYA00353580	https://www.ebi.ac.uk/metagenomics/api/v1/analyses/MGYA00353580/file/ERR1583343_FASTQ_SSU_OTU.tsv	Root-Environmental-Aquatic-Freshwater-Lake
ERR1582971	MGYA00353208	https://www.ebi.ac.uk/metagenomics/api/v1/analyses/MGYA00353208/file/ERR1582971_FASTQ_SSU_OTU.tsv	Root-Environmental-Aquatic-Freshwater-Lake
ERR1582503	MGYA00352740	https://www.ebi.ac.uk/metagenomics/api/v1/analyses/MGYA00352740/file/ERR1582503_FASTQ_SSU_OTU.tsv	Root-Environmental-Aquatic-Freshwater-Lake
SRR7081154	MGYA00413195	https://www.ebi.ac.uk/metagenomics/api/v1/analyses/MGYA00413195/file/SRR7081154_MERGED_FASTQ_SSU_OTU.tsv	Root-Environmental-Aquatic-Freshwater-Lake
ERR1582162	MGYA00352399	https://www.ebi.ac.uk/metagenomics/api/v1/analyses/MGYA00352399/file/ERR1582162_FASTQ_SSU_OTU.tsv	Root-Environmental-Aquatic-Freshwater-Lake

ERR1583085	MGYA00353322	https://www.ebi.ac.uk/metagenomics/api/v1/analyses/MGYA00353322/file/ERR1583085_FASTQ_SSU_OTU.tsv	Root-Environmental-Aquatic-Freshwater-Lake
ERR1897489	MGYA00351434	https://www.ebi.ac.uk/metagenomics/api/v1/analyses/MGYA00351434/file/ERR1897489_FASTQ_SSU_OTU.tsv	Root-Environmental-Aquatic-Freshwater-Lake
ERR1333510	MGYA00107608	https://www.ebi.ac.uk/metagenomics/api/v1/analyses/MGYA00107608/file/ERR1333510_FASTQ_otu.tsv	Root-Environmental-Aquatic-Freshwater-Lake
ERR1583267	MGYA00353504	https://www.ebi.ac.uk/metagenomics/api/v1/analyses/MGYA00353504/file/ERR1583267_FASTQ_SSU_OTU.tsv	Root-Environmental-Aquatic-Freshwater-Lake
ERR1582380	MGYA00352617	https://www.ebi.ac.uk/metagenomics/api/v1/analyses/MGYA00352617/file/ERR1582380_FASTQ_SSU_OTU.tsv	Root-Environmental-Aquatic-Freshwater-Lake
ERR1582427	MGYA00352664	https://www.ebi.ac.uk/metagenomics/api/v1/analyses/MGYA00352664/file/ERR1582427_FASTQ_SSU_OTU.tsv	Root-Environmental-Aquatic-Freshwater-Lake
ERR1582855	MGYA00353092	https://www.ebi.ac.uk/metagenomics/api/v1/analyses/MGYA00353092/file/ERR1582855_FASTQ_SSU_OTU.tsv	Root-Environmental-Aquatic-Freshwater-Lake
ERR658179	MGYA00103511	https://www.ebi.ac.uk/metagenomics/api/v1/analyses/MGYA00103511/file/ERR658179_FASTQ_otu.tsv	Root-Environmental-Aquatic-Freshwater-Lake
ERR1535947	MGYA00320238	https://www.ebi.ac.uk/metagenomics/api/v1/analyses/MGYA00320238/file/ERR1535947_FASTQ_SSU_OTU.tsv	Root-Environmental-Aquatic-Freshwater-Lake
ERR1582046	MGYA00352283	https://www.ebi.ac.uk/metagenomics/api/v1/analyses/MGYA00352283/file/ERR1582046_FASTQ_SSU_OTU.tsv	Root-Environmental-Aquatic-Freshwater-Lake
ERR1896838	MGYA00350783	https://www.ebi.ac.uk/metagenomics/api/v1/analyses/MGYA00350783/file/ERR1896838_FASTQ_SSU_OTU.tsv	Root-Environmental-Aquatic-Freshwater-Lake
SRR7081367	MGYA00423210	https://www.ebi.ac.uk/metagenomics/api/v1/analyses/MGYA00423210/file/SRR7081367_MERGED_FASTQ_SSU_OTU.tsv	Root-Environmental-Aquatic-Freshwater-Lake

ERR1583170	MGYA00353407	https://www.ebi.ac.uk/metagenomics/api/v1/analyses/MGYA00353407/file/ERR1583170_FASTQ_SSU_OTU.tsv	Root-Environmental-Aquatic-Freshwater-Lake
ERR1582730	MGYA00352967	https://www.ebi.ac.uk/metagenomics/api/v1/analyses/MGYA00352967/file/ERR1582730_FASTQ_SSU_OTU.tsv	Root-Environmental-Aquatic-Freshwater-Lake
ERR1582097	MGYA00352334	https://www.ebi.ac.uk/metagenomics/api/v1/analyses/MGYA00352334/file/ERR1582097_FASTQ_SSU_OTU.tsv	Root-Environmental-Aquatic-Freshwater-Lake
ERR1582351	MGYA00352588	https://www.ebi.ac.uk/metagenomics/api/v1/analyses/MGYA00352588/file/ERR1582351_FASTQ_SSU_OTU.tsv	Root-Environmental-Aquatic-Freshwater-Lake
ERR1429274	MGYA00107632	https://www.ebi.ac.uk/metagenomics/api/v1/analyses/MGYA00107632/file/ERR1429274_FASTQ_otu.tsv	Root-Environmental-Aquatic-Freshwater-Lake
ERR1582884	MGYA00353121	https://www.ebi.ac.uk/metagenomics/api/v1/analyses/MGYA00353121/file/ERR1582884_FASTQ_SSU_OTU.tsv	Root-Environmental-Aquatic-Freshwater-Lake
ERR1583511	MGYA00353748	https://www.ebi.ac.uk/metagenomics/api/v1/analyses/MGYA00353748/file/ERR1583511_FASTQ_SSU_OTU.tsv	Root-Environmental-Aquatic-Freshwater-Lake
ERR1583028	MGYA00353265	https://www.ebi.ac.uk/metagenomics/api/v1/analyses/MGYA00353265/file/ERR1583028_FASTQ_SSU_OTU.tsv	Root-Environmental-Aquatic-Freshwater-Lake
ERR1582668	MGYA00352905	https://www.ebi.ac.uk/metagenomics/api/v1/analyses/MGYA00352905/file/ERR1582668_FASTQ_SSU_OTU.tsv	Root-Environmental-Aquatic-Freshwater-Lake
SRR2442952	MGYA00358956	https://www.ebi.ac.uk/metagenomics/api/v1/analyses/MGYA00358956/file/SRR2442952_MERGED_FASTQ_SSU_OTU.tsv	Root-Environmental-Aquatic-Freshwater-Lake
ERR1743357	MGYA00087205	https://www.ebi.ac.uk/metagenomics/api/v1/analyses/MGYA00087205/file/ERR1743357_MERGED_FASTQ_otu.tsv	Root-Environmental-Aquatic-Freshwater-Lake
ERR1897424	MGYA00351369	https://www.ebi.ac.uk/metagenomics/api/v1/analyses/MGYA00351369/file/ERR1897424_FASTQ_SSU_OTU.tsv	Root-Environmental-Aquatic-Freshwater-Lake

ERR1582209	MGYA00352446	https://www.ebi.ac.uk/metagenomics/api/v1/analyses/MGYA00352446/file/ERR1582209_FASTQ_SSU_OTU.tsv	Root-Environmental-Aquatic-Freshwater-Lake
ERR1333499	MGYA00107600	https://www.ebi.ac.uk/metagenomics/api/v1/analyses/MGYA00107600/file/ERR1333499_FASTQ_otu.tsv	Root-Environmental-Aquatic-Freshwater-Lake
ERR1583449	MGYA00353686	https://www.ebi.ac.uk/metagenomics/api/v1/analyses/MGYA00353686/file/ERR1583449_FASTQ_SSU_OTU.tsv	Root-Environmental-Aquatic-Freshwater-Lake
ERR1897045	MGYA00350990	https://www.ebi.ac.uk/metagenomics/api/v1/analyses/MGYA00350990/file/ERR1897045_FASTQ_SSU_OTU.tsv	Root-Environmental-Aquatic-Freshwater-Lake
ERR1897733	MGYA00351678	https://www.ebi.ac.uk/metagenomics/api/v1/analyses/MGYA00351678/file/ERR1897733_FASTQ_SSU_OTU.tsv	Root-Environmental-Aquatic-Freshwater-Lake
ERR1897094	MGYA00351039	https://www.ebi.ac.uk/metagenomics/api/v1/analyses/MGYA00351039/file/ERR1897094_FASTQ_SSU_OTU.tsv	Root-Environmental-Aquatic-Freshwater-Lake
ERR1583498	MGYA00353735	https://www.ebi.ac.uk/metagenomics/api/v1/analyses/MGYA00353735/file/ERR1583498_FASTQ_SSU_OTU.tsv	Root-Environmental-Aquatic-Freshwater-Lake
ERR1897352	MGYA00351297	https://www.ebi.ac.uk/metagenomics/api/v1/analyses/MGYA00351297/file/ERR1897352_FASTQ_SSU_OTU.tsv	Root-Environmental-Aquatic-Freshwater-Lake
ERR589327	MGYA00103425	https://www.ebi.ac.uk/metagenomics/api/v1/analyses/MGYA00103425/file/ERR589327_FASTQ_otu.tsv	Root-Environmental-Aquatic-Freshwater-Lake
ERR1896960	MGYA00350905	https://www.ebi.ac.uk/metagenomics/api/v1/analyses/MGYA00350905/file/ERR1896960_FASTQ_SSU_OTU.tsv	Root-Environmental-Aquatic-Freshwater-Lake
SRR2442983	MGYA00358987	https://www.ebi.ac.uk/metagenomics/api/v1/analyses/MGYA00358987/file/SRR2442983_MERGED_FASTQ_SSU_OTU.tsv	Root-Environmental-Aquatic-Freshwater-Lake
ERR1743386	MGYA00087175	https://www.ebi.ac.uk/metagenomics/api/v1/analyses/MGYA00087175/file/ERR1743386_MERGED_FASTQ_otu.tsv	Root-Environmental-Aquatic-Freshwater-Lake

ERR1582318	MGYA00352555	https://www.ebi.ac.uk/metagenomics/api/v1/analyses/MGYA00352555/file/ERR1582318_FASTQ_SSU_OTU.tsv	Root-Environmental-Aquatic-Freshwater-Lake
ERR1897154	MGYA00351099	https://www.ebi.ac.uk/metagenomics/api/v1/analyses/MGYA00351099/file/ERR1897154_FASTQ_SSU_OTU.tsv	Root-Environmental-Aquatic-Freshwater-Lake
ERR1896714	MGYA00350659	https://www.ebi.ac.uk/metagenomics/api/v1/analyses/MGYA00350659/file/ERR1896714_FASTQ_SSU_OTU.tsv	Root-Environmental-Aquatic-Freshwater-Lake
ERR1583139	MGYA00353376	https://www.ebi.ac.uk/metagenomics/api/v1/analyses/MGYA00353376/file/ERR1583139_FASTQ_SSU_OTU.tsv	Root-Environmental-Aquatic-Freshwater-Lake
ERR1582779	MGYA00353016	https://www.ebi.ac.uk/metagenomics/api/v1/analyses/MGYA00353016/file/ERR1582779_FASTQ_SSU_OTU.tsv	Root-Environmental-Aquatic-Freshwater-Lake
ERR1547079	MGYA00320790	https://www.ebi.ac.uk/metagenomics/api/v1/analyses/MGYA00320790/file/ERR1547079_FASTQ_SSU_OTU.tsv	Root-Environmental-Aquatic-Freshwater-Lake
ERR1897292	MGYA00351237	https://www.ebi.ac.uk/metagenomics/api/v1/analyses/MGYA00351237/file/ERR1897292_FASTQ_SSU_OTU.tsv	Root-Environmental-Aquatic-Freshwater-Lake
ERR1897535	MGYA00351480	https://www.ebi.ac.uk/metagenomics/api/v1/analyses/MGYA00351480/file/ERR1897535_FASTQ_SSU_OTU.tsv	Root-Environmental-Aquatic-Freshwater-Lake
ERR1897243	MGYA00351188	https://www.ebi.ac.uk/metagenomics/api/v1/analyses/MGYA00351188/file/ERR1897243_FASTQ_SSU_OTU.tsv	Root-Environmental-Aquatic-Freshwater-Lake
ERR1896871	MGYA00350816	https://www.ebi.ac.uk/metagenomics/api/v1/analyses/MGYA00350816/file/ERR1896871_FASTQ_SSU_OTU.tsv	Root-Environmental-Aquatic-Freshwater-Lake
ERR1743297	MGYA00087119	https://www.ebi.ac.uk/metagenomics/api/v1/analyses/MGYA00087119/file/ERR1743297_MERGED_FASTQ_otu.tsv	Root-Environmental-Aquatic-Freshwater-Lake
ERR1897622	MGYA00351567	https://www.ebi.ac.uk/metagenomics/api/v1/analyses/MGYA00351567/file/ERR1897622_FASTQ_SSU_OTU.tsv	Root-Environmental-Aquatic-Freshwater-Lake

ERR1897185	MGYA00351130	https://www.ebi.ac.uk/metagenomics/api/v1/analyses/MGYA00351130/file/ERR1897185_FASTQ_SSU_OTU.tsv	Root-Environmental-Aquatic-Freshwater-Lake
ERR1582157	MGYA00352394	https://www.ebi.ac.uk/metagenomics/api/v1/analyses/MGYA00352394/file/ERR1582157_FASTQ_SSU_OTU.tsv	Root-Environmental-Aquatic-Freshwater-Lake
ERR1896929	MGYA00350874	https://www.ebi.ac.uk/metagenomics/api/v1/analyses/MGYA00350874/file/ERR1896929_FASTQ_SSU_OTU.tsv	Root-Environmental-Aquatic-Freshwater-Lake
ERR1583376	MGYA00353613	https://www.ebi.ac.uk/metagenomics/api/v1/analyses/MGYA00353613/file/ERR1583376_FASTQ_SSU_OTU.tsv	Root-Environmental-Aquatic-Freshwater-Lake
ERR1582291	MGYA00352528	https://www.ebi.ac.uk/metagenomics/api/v1/analyses/MGYA00352528/file/ERR1582291_FASTQ_SSU_OTU.tsv	Root-Environmental-Aquatic-Freshwater-Lake
ERR1582944	MGYA00353181	https://www.ebi.ac.uk/metagenomics/api/v1/analyses/MGYA00353181/file/ERR1582944_FASTQ_SSU_OTU.tsv	Root-Environmental-Aquatic-Freshwater-Lake
ERR1582536	MGYA00352773	https://www.ebi.ac.uk/metagenomics/api/v1/analyses/MGYA00352773/file/ERR1582536_FASTQ_SSU_OTU.tsv	Root-Environmental-Aquatic-Freshwater-Lake
SRR7081161	MGYA00426466	https://www.ebi.ac.uk/metagenomics/api/v1/analyses/MGYA00426466/file/SRR7081161_MERGED_FASTQ_SSU_OTU.tsv	Root-Environmental-Aquatic-Freshwater-Lake
ERR1582240	MGYA00352477	https://www.ebi.ac.uk/metagenomics/api/v1/analyses/MGYA00352477/file/ERR1582240_FASTQ_SSU_OTU.tsv	Root-Environmental-Aquatic-Freshwater-Lake
ERR430933	MGYA00103260	https://www.ebi.ac.uk/metagenomics/api/v1/analyses/MGYA00103260/file/ERR430933_FASTQ_otu.tsv	Root-Environmental-Aquatic-Freshwater-Lake
ERR1582995	MGYA00353232	https://www.ebi.ac.uk/metagenomics/api/v1/analyses/MGYA00353232/file/ERR1582995_FASTQ_SSU_OTU.tsv	Root-Environmental-Aquatic-Freshwater-Lake
ERR1583400	MGYA00353637	https://www.ebi.ac.uk/metagenomics/api/v1/analyses/MGYA00353637/file/ERR1583400_FASTQ_SSU_OTU.tsv	Root-Environmental-Aquatic-Freshwater-Lake

ERR1547121	MGYA00320832	https://www.ebi.ac.uk/metagenomics/api/v1/analyses/MGYA00320832/file/ERR1547121_FASTQ_SSU_OTU.tsv	Root-Environmental-Aquatic-Freshwater-Lake
ERR1583061	MGYA00353298	https://www.ebi.ac.uk/metagenomics/api/v1/analyses/MGYA00353298/file/ERR1583061_FASTQ_SSU_OTU.tsv	Root-Environmental-Aquatic-Freshwater-Lake
ERR1582621	MGYA00352858	https://www.ebi.ac.uk/metagenomics/api/v1/analyses/MGYA00352858/file/ERR1582621_FASTQ_SSU_OTU.tsv	Root-Environmental-Aquatic-Freshwater-Lake
ERR1582186	MGYA00352423	https://www.ebi.ac.uk/metagenomics/api/v1/analyses/MGYA00352423/file/ERR1582186_FASTQ_SSU_OTU.tsv	Root-Environmental-Aquatic-Freshwater-Lake
SRR7081094	MGYA00413207	https://www.ebi.ac.uk/metagenomics/api/v1/analyses/MGYA00413207/file/SRR7081094_MERGED_FASTQ_SSU_OTU.tsv	Root-Environmental-Aquatic-Freshwater-Lake
ERR1897128	MGYA00351073	https://www.ebi.ac.uk/metagenomics/api/v1/analyses/MGYA00351073/file/ERR1897128_FASTQ_SSU_OTU.tsv	Root-Environmental-Aquatic-Freshwater-Lake
ERR1896768	MGYA00350713	https://www.ebi.ac.uk/metagenomics/api/v1/analyses/MGYA00350713/file/ERR1896768_FASTQ_SSU_OTU.tsv	Root-Environmental-Aquatic-Freshwater-Lake
ERR1429241	MGYA00107646	https://www.ebi.ac.uk/metagenomics/api/v1/analyses/MGYA00107646/file/ERR1429241_FASTQ_otu.tsv	Root-Environmental-Aquatic-Freshwater-Lake
ERR1582364	MGYA00352601	https://www.ebi.ac.uk/metagenomics/api/v1/analyses/MGYA00352601/file/ERR1582364_FASTQ_SSU_OTU.tsv	Root-Environmental-Aquatic-Freshwater-Lake
ERR1583283	MGYA00353520	https://www.ebi.ac.uk/metagenomics/api/v1/analyses/MGYA00353520/file/ERR1583283_FASTQ_SSU_OTU.tsv	Root-Environmental-Aquatic-Freshwater-Lake
ERR1897549	MGYA00351494	https://www.ebi.ac.uk/metagenomics/api/v1/analyses/MGYA00351494/file/ERR1897549_FASTQ_SSU_OTU.tsv	Root-Environmental-Aquatic-Freshwater-Lake
SRR7081352	MGYA00423823	https://www.ebi.ac.uk/metagenomics/api/v1/analyses/MGYA00423823/file/SRR7081352_MERGED_FASTQ_SSU_OTU.tsv	Root-Environmental-Aquatic-Freshwater-Lake

ERR1583145	MGYA00353382	https://www.ebi.ac.uk/metagenomics/api/v1/analyses/MGYA00353382/file/ERR1583145_FASTQ_SSU_OTU.tsv	Root-Environmental-Aquatic-Freshwater-Lake
ERR1582705	MGYA00352942	https://www.ebi.ac.uk/metagenomics/api/v1/analyses/MGYA00352942/file/ERR1582705_FASTQ_SSU_OTU.tsv	Root-Environmental-Aquatic-Freshwater-Lake
ERR1582073	MGYA00352310	https://www.ebi.ac.uk/metagenomics/api/v1/analyses/MGYA00352310/file/ERR1582073_FASTQ_SSU_OTU.tsv	Root-Environmental-Aquatic-Freshwater-Lake
ERR1583194	MGYA00353431	https://www.ebi.ac.uk/metagenomics/api/v1/analyses/MGYA00353431/file/ERR1583194_FASTQ_SSU_OTU.tsv	Root-Environmental-Aquatic-Freshwater-Lake
ERR1897598	MGYA00351543	https://www.ebi.ac.uk/metagenomics/api/v1/analyses/MGYA00351543/file/ERR1897598_FASTQ_SSU_OTU.tsv	Root-Environmental-Aquatic-Freshwater-Lake
SRR7081383	MGYA00423189	https://www.ebi.ac.uk/metagenomics/api/v1/analyses/MGYA00423189/file/SRR7081383_MERGED_FASTQ_SSU_OTU.tsv	Root-Environmental-Aquatic-Freshwater-Lake
ERR1583252	MGYA00353489	https://www.ebi.ac.uk/metagenomics/api/v1/analyses/MGYA00353489/file/ERR1583252_FASTQ_SSU_OTU.tsv	Root-Environmental-Aquatic-Freshwater-Lake
ERR1333525	MGYA00107612	https://www.ebi.ac.uk/metagenomics/api/v1/analyses/MGYA00107612/file/ERR1333525_FASTQ_otu.tsv	Root-Environmental-Aquatic-Freshwater-Lake
ERR1582412	MGYA00352649	https://www.ebi.ac.uk/metagenomics/api/v1/analyses/MGYA00352649/file/ERR1582412_FASTQ_SSU_OTU.tsv	Root-Environmental-Aquatic-Freshwater-Lake
ERR1582860	MGYA00353097	https://www.ebi.ac.uk/metagenomics/api/v1/analyses/MGYA00353097/file/ERR1582860_FASTQ_SSU_OTU.tsv	Root-Environmental-Aquatic-Freshwater-Lake
ERR1535972	MGYA00320263	https://www.ebi.ac.uk/metagenomics/api/v1/analyses/MGYA00320263/file/ERR1535972_FASTQ_SSU_OTU.tsv	Root-Environmental-Aquatic-Freshwater-Lake
ERR1742285	MGYA00311586	https://www.ebi.ac.uk/metagenomics/api/v1/analyses/MGYA00311586/file/ERR1742285_FASTQ_SSU_OTU.tsv	Root-Environmental-Aquatic-Freshwater-Lake

ERR589312	MGYA00103420	https://www.ebi.ac.uk/metagenomics/api/v1/analyses/MGYA00103420/file/ERR589312_FASTQ_otu.tsv	Root-Environmental-Aquatic-Freshwater-Lake
ERR1897367	MGYA00351312	https://www.ebi.ac.uk/metagenomics/api/v1/analyses/MGYA00351312/file/ERR1897367_FASTQ_SSU_OTU.tsv	Root-Environmental-Aquatic-Freshwater-Lake
ERR1896955	MGYA00350900	https://www.ebi.ac.uk/metagenomics/api/v1/analyses/MGYA00350900/file/ERR1896955_FASTQ_SSU_OTU.tsv	Root-Environmental-Aquatic-Freshwater-Lake
ERR1897706	MGYA00351651	https://www.ebi.ac.uk/metagenomics/api/v1/analyses/MGYA00351651/file/ERR1897706_FASTQ_SSU_OTU.tsv	Root-Environmental-Aquatic-Freshwater-Lake
SRR2443042	MGYA00359046	https://www.ebi.ac.uk/metagenomics/api/v1/analyses/MGYA00359046/file/SRR2443042_MERGED_FASTQ_SSU_OTU.tsv	Root-Environmental-Aquatic-Freshwater-Lake
ERR1582938	MGYA00353175	https://www.ebi.ac.uk/metagenomics/api/v1/analyses/MGYA00353175/file/ERR1582938_FASTQ_SSU_OTU.tsv	Root-Environmental-Aquatic-Freshwater-Lake
ERR1897070	MGYA00351015	https://www.ebi.ac.uk/metagenomics/api/v1/analyses/MGYA00351015/file/ERR1897070_FASTQ_SSU_OTU.tsv	Root-Environmental-Aquatic-Freshwater-Lake
SRR2442967	MGYA00358971	https://www.ebi.ac.uk/metagenomics/api/v1/analyses/MGYA00358971/file/SRR2442967_MERGED_FASTQ_SSU_OTU.tsv	Root-Environmental-Aquatic-Freshwater-Lake
ERR1743362	MGYA00087139	https://www.ebi.ac.uk/metagenomics/api/v1/analyses/MGYA00087139/file/ERR1743362_MERGED_FASTQ_otu.tsv	Root-Environmental-Aquatic-Freshwater-Lake
ERR1896984	MGYA00350929	https://www.ebi.ac.uk/metagenomics/api/v1/analyses/MGYA00350929/file/ERR1896984_FASTQ_SSU_OTU.tsv	Root-Environmental-Aquatic-Freshwater-Lake
ERR1897411	MGYA00351356	https://www.ebi.ac.uk/metagenomics/api/v1/analyses/MGYA00351356/file/ERR1897411_FASTQ_SSU_OTU.tsv	Root-Environmental-Aquatic-Freshwater-Lake
ERR1897688	MGYA00351633	https://www.ebi.ac.uk/metagenomics/api/v1/analyses/MGYA00351633/file/ERR1897688_FASTQ_SSU_OTU.tsv	Root-Environmental-Aquatic-Freshwater-Lake

SRR7081093	MGYA00414125	https://www.ebi.ac.uk/metagenomics/api/v1/analyses/MGYA00414125/file/SRR7081093_MERGED_FASTQ_SSU_OTU.tsv	Root-Environmental-Aquatic-Freshwater-Lake
ERR1583284	MGYA00353521	https://www.ebi.ac.uk/metagenomics/api/v1/analyses/MGYA00353521/file/ERR1583284_FASTQ_SSU_OTU.tsv	Root-Environmental-Aquatic-Freshwater-Lake
ERR1582363	MGYA00352600	https://www.ebi.ac.uk/metagenomics/api/v1/analyses/MGYA00352600/file/ERR1582363_FASTQ_SSU_OTU.tsv	Root-Environmental-Aquatic-Freshwater-Lake
ERR1429246	MGYA00107679	https://www.ebi.ac.uk/metagenomics/api/v1/analyses/MGYA00107679/file/ERR1429246_FASTQ_otu.tsv	Root-Environmental-Aquatic-Freshwater-Lake
ERR1582702	MGYA00352939	https://www.ebi.ac.uk/metagenomics/api/v1/analyses/MGYA00352939/file/ERR1582702_FASTQ_SSU_OTU.tsv	Root-Environmental-Aquatic-Freshwater-Lake
ERR1583142	MGYA00353379	https://www.ebi.ac.uk/metagenomics/api/v1/analyses/MGYA00353379/file/ERR1583142_FASTQ_SSU_OTU.tsv	Root-Environmental-Aquatic-Freshwater-Lake
ERR1583193	MGYA00353430	https://www.ebi.ac.uk/metagenomics/api/v1/analyses/MGYA00353430/file/ERR1583193_FASTQ_SSU_OTU.tsv	Root-Environmental-Aquatic-Freshwater-Lake
ERR1582074	MGYA00352311	https://www.ebi.ac.uk/metagenomics/api/v1/analyses/MGYA00352311/file/ERR1582074_FASTQ_SSU_OTU.tsv	Root-Environmental-Aquatic-Freshwater-Lake
ERR1897238	MGYA00351183	https://www.ebi.ac.uk/metagenomics/api/v1/analyses/MGYA00351183/file/ERR1897238_FASTQ_SSU_OTU.tsv	Root-Environmental-Aquatic-Freshwater-Lake
ERR1582867	MGYA00353104	https://www.ebi.ac.uk/metagenomics/api/v1/analyses/MGYA00353104/file/ERR1582867_FASTQ_SSU_OTU.tsv	Root-Environmental-Aquatic-Freshwater-Lake
ERR1582415	MGYA00352652	https://www.ebi.ac.uk/metagenomics/api/v1/analyses/MGYA00352652/file/ERR1582415_FASTQ_SSU_OTU.tsv	Root-Environmental-Aquatic-Freshwater-Lake
ERR1333522	MGYA00107611	https://www.ebi.ac.uk/metagenomics/api/v1/analyses/MGYA00107611/file/ERR1333522_FASTQ_otu.tsv	Root-Environmental-Aquatic-Freshwater-Lake

ERR1583255	MGYA00353492	https://www.ebi.ac.uk/metagenomics/api/v1/analyses/MGYA00353492/file/ERR1583255_FASTQ_SSU_OTU.tsv	Root-Environmental-Aquatic-Freshwater-Lake
ERR1897659	MGYA00351604	https://www.ebi.ac.uk/metagenomics/api/v1/analyses/MGYA00351604/file/ERR1897659_FASTQ_SSU_OTU.tsv	Root-Environmental-Aquatic-Freshwater-Lake
ERR1742282	MGYA00311583	https://www.ebi.ac.uk/metagenomics/api/v1/analyses/MGYA00311583/file/ERR1742282_FASTQ_SSU_OTU.tsv	Root-Environmental-Aquatic-Freshwater-Lake
ERR1535975	MGYA00320266	https://www.ebi.ac.uk/metagenomics/api/v1/analyses/MGYA00320266/file/ERR1535975_FASTQ_SSU_OTU.tsv	Root-Environmental-Aquatic-Freshwater-Lake
ERR1896952	MGYA00350897	https://www.ebi.ac.uk/metagenomics/api/v1/analyses/MGYA00350897/file/ERR1896952_FASTQ_SSU_OTU.tsv	Root-Environmental-Aquatic-Freshwater-Lake
ERR1897360	MGYA00351305	https://www.ebi.ac.uk/metagenomics/api/v1/analyses/MGYA00351305/file/ERR1897360_FASTQ_SSU_OTU.tsv	Root-Environmental-Aquatic-Freshwater-Lake
ERR589315	MGYA00103415	https://www.ebi.ac.uk/metagenomics/api/v1/analyses/MGYA00103415/file/ERR589315_FASTQ_otu.tsv	Root-Environmental-Aquatic-Freshwater-Lake
ERR1897701	MGYA00351646	https://www.ebi.ac.uk/metagenomics/api/v1/analyses/MGYA00351646/file/ERR1897701_FASTQ_SSU_OTU.tsv	Root-Environmental-Aquatic-Freshwater-Lake
SRR2443045	MGYA00359049	https://www.ebi.ac.uk/metagenomics/api/v1/analyses/MGYA00359049/file/SRR2443045_MERGED_FASTQ_SSU_OTU.tsv	Root-Environmental-Aquatic-Freshwater-Lake
ERR1896637	MGYA00350582	https://www.ebi.ac.uk/metagenomics/api/v1/analyses/MGYA00350582/file/ERR1896637_FASTQ_SSU_OTU.tsv	Root-Environmental-Aquatic-Freshwater-Lake
ERR1897077	MGYA00351022	https://www.ebi.ac.uk/metagenomics/api/v1/analyses/MGYA00351022/file/ERR1897077_FASTQ_SSU_OTU.tsv	Root-Environmental-Aquatic-Freshwater-Lake
ERR1896983	MGYA00350928	https://www.ebi.ac.uk/metagenomics/api/v1/analyses/MGYA00350928/file/ERR1896983_FASTQ_SSU_OTU.tsv	Root-Environmental-Aquatic-Freshwater-Lake

ERR1743365	MGYA00087152	https://www.ebi.ac.uk/metagenomics/api/v1/analyses/MGYA00087152/file/ERR1743365_MERGED_FASTQ_otu.tsv	Root-Environmental-Aquatic-Freshwater-Lake
SRR2442960	MGYA00358964	https://www.ebi.ac.uk/metagenomics/api/v1/analyses/MGYA00358964/file/SRR2442960_MERGED_FASTQ_SSU_OTU.tsv	Root-Environmental-Aquatic-Freshwater-Lake
ERR1990503	MGYA00128221	https://www.ebi.ac.uk/metagenomics/api/v1/analyses/MGYA00128221/file/ERR1990503_MERGED_FASTQ_otu.tsv	Root-Environmental-Aquatic-Freshwater-Lake
ERR1896713	MGYA00350658	https://www.ebi.ac.uk/metagenomics/api/v1/analyses/MGYA00350658/file/ERR1896713_FASTQ_SSU_OTU.tsv	Root-Environmental-Aquatic-Freshwater-Lake
ERR1897153	MGYA00351098	https://www.ebi.ac.uk/metagenomics/api/v1/analyses/MGYA00351098/file/ERR1897153_FASTQ_SSU_OTU.tsv	Root-Environmental-Aquatic-Freshwater-Lake
ERR1897532	MGYA00351477	https://www.ebi.ac.uk/metagenomics/api/v1/analyses/MGYA00351477/file/ERR1897532_FASTQ_SSU_OTU.tsv	Root-Environmental-Aquatic-Freshwater-Lake
SRR7081329	MGYA00424198	https://www.ebi.ac.uk/metagenomics/api/v1/analyses/MGYA00424198/file/SRR7081329_MERGED_FASTQ_SSU_OTU.tsv	Root-Environmental-Aquatic-Freshwater-Lake
ERR1897295	MGYA00351240	https://www.ebi.ac.uk/metagenomics/api/v1/analyses/MGYA00351240/file/ERR1897295_FASTQ_SSU_OTU.tsv	Root-Environmental-Aquatic-Freshwater-Lake
ERR2868527	MGYA00377409	https://www.ebi.ac.uk/metagenomics/api/v1/analyses/MGYA00377409/file/ERR2868527_FASTQ_SSU_OTU.tsv	Root-Environmental-Aquatic-Freshwater-Lake
ERR1743290	MGYA00087154	https://www.ebi.ac.uk/metagenomics/api/v1/analyses/MGYA00087154/file/ERR1743290_MERGED_FASTQ_otu.tsv	Root-Environmental-Aquatic-Freshwater-Lake
ERR1896876	MGYA00350821	https://www.ebi.ac.uk/metagenomics/api/v1/analyses/MGYA00350821/file/ERR1896876_FASTQ_SSU_OTU.tsv	Root-Environmental-Aquatic-Freshwater-Lake
ERR1897244	MGYA00351189	https://www.ebi.ac.uk/metagenomics/api/v1/analyses/MGYA00351189/file/ERR1897244_FASTQ_SSU_OTU.tsv	Root-Environmental-Aquatic-Freshwater-Lake

ERR389729	MGYA00103376	https://www.ebi.ac.uk/metagenomics/api/v1/analyses/MGYA00103376/file/ERR389729_FASTQ_otu.tsv	Root-Environmental-Aquatic-Freshwater-Lake
ERR1314368	MGYA00095159	https://www.ebi.ac.uk/metagenomics/api/v1/analyses/MGYA00095159/file/ERR1314368_MERGED_FASTQ_otu.tsv	Root-Environmental-Aquatic-Freshwater-Lake
ERR1897182	MGYA00351127	https://www.ebi.ac.uk/metagenomics/api/v1/analyses/MGYA00351127/file/ERR1897182_FASTQ_SSU_OTU.tsv	Root-Environmental-Aquatic-Freshwater-Lake
ERR1897625	MGYA00351570	https://www.ebi.ac.uk/metagenomics/api/v1/analyses/MGYA00351570/file/ERR1897625_FASTQ_SSU_OTU.tsv	Root-Environmental-Aquatic-Freshwater-Lake
ERR1582469	MGYA00352706	https://www.ebi.ac.uk/metagenomics/api/v1/analyses/MGYA00352706/file/ERR1582469_FASTQ_SSU_OTU.tsv	Root-Environmental-Aquatic-Freshwater-Lake
ERR1583229	MGYA00353466	https://www.ebi.ac.uk/metagenomics/api/v1/analyses/MGYA00353466/file/ERR1583229_FASTQ_SSU_OTU.tsv	Root-Environmental-Aquatic-Freshwater-Lake
ERR1582150	MGYA00352387	https://www.ebi.ac.uk/metagenomics/api/v1/analyses/MGYA00352387/file/ERR1582150_FASTQ_SSU_OTU.tsv	Root-Environmental-Aquatic-Freshwater-Lake
ERR1582531	MGYA00352768	https://www.ebi.ac.uk/metagenomics/api/v1/analyses/MGYA00352768/file/ERR1582531_FASTQ_SSU_OTU.tsv	Root-Environmental-Aquatic-Freshwater-Lake
ERR1582943	MGYA00353180	https://www.ebi.ac.uk/metagenomics/api/v1/analyses/MGYA00353180/file/ERR1582943_FASTQ_SSU_OTU.tsv	Root-Environmental-Aquatic-Freshwater-Lake
ERR1582296	MGYA00352533	https://www.ebi.ac.uk/metagenomics/api/v1/analyses/MGYA00352533/file/ERR1582296_FASTQ_SSU_OTU.tsv	Root-Environmental-Aquatic-Freshwater-Lake
ERR1583371	MGYA00353608	https://www.ebi.ac.uk/metagenomics/api/v1/analyses/MGYA00353608/file/ERR1583371_FASTQ_SSU_OTU.tsv	Root-Environmental-Aquatic-Freshwater-Lake
SRR7081166	MGYA00425957	https://www.ebi.ac.uk/metagenomics/api/v1/analyses/MGYA00425957/file/SRR7081166_MERGED_FASTQ_SSU_OTU.tsv	Root-Environmental-Aquatic-Freshwater-Lake

SRR2443039	MGYA00359043	https://www.ebi.ac.uk/metagenomics/api/v1/analyses/MGYA00359043/file/SRR2443039_MERGED_FASTQ_SSU_OTU.tsv	Root-Environmental-Aquatic-Freshwater-Lake
ERR1583407	MGYA00353644	https://www.ebi.ac.uk/metagenomics/api/v1/analyses/MGYA00353644/file/ERR1583407_FASTQ_SSU_OTU.tsv	Root-Environmental-Aquatic-Freshwater-Lake
ERR430934	MGYA00103254	https://www.ebi.ac.uk/metagenomics/api/v1/analyses/MGYA00103254/file/ERR430934_FASTQ_otu.tsv	Root-Environmental-Aquatic-Freshwater-Lake
ERR1582992	MGYA00353229	https://www.ebi.ac.uk/metagenomics/api/v1/analyses/MGYA00353229/file/ERR1582992_FASTQ_SSU_OTU.tsv	Root-Environmental-Aquatic-Freshwater-Lake
ERR1582247	MGYA00352484	https://www.ebi.ac.uk/metagenomics/api/v1/analyses/MGYA00352484/file/ERR1582247_FASTQ_SSU_OTU.tsv	Root-Environmental-Aquatic-Freshwater-Lake
SRR7081271	MGYA00413189	https://www.ebi.ac.uk/metagenomics/api/v1/analyses/MGYA00413189/file/SRR7081271_MERGED_FASTQ_SSU_OTU.tsv	Root-Environmental-Aquatic-Freshwater-Lake
ERR1743319	MGYA00087220	https://www.ebi.ac.uk/metagenomics/api/v1/analyses/MGYA00087220/file/ERR1743319_MERGED_FASTQ_otu.tsv	Root-Environmental-Aquatic-Freshwater-Lake
ERR1547126	MGYA00320837	https://www.ebi.ac.uk/metagenomics/api/v1/analyses/MGYA00320837/file/ERR1547126_FASTQ_SSU_OTU.tsv	Root-Environmental-Aquatic-Freshwater-Lake
ERR1582181	MGYA00352418	https://www.ebi.ac.uk/metagenomics/api/v1/analyses/MGYA00352418/file/ERR1582181_FASTQ_SSU_OTU.tsv	Root-Environmental-Aquatic-Freshwater-Lake
ERR1582626	MGYA00352863	https://www.ebi.ac.uk/metagenomics/api/v1/analyses/MGYA00352863/file/ERR1582626_FASTQ_SSU_OTU.tsv	Root-Environmental-Aquatic-Freshwater-Lake
ERR1583066	MGYA00353303	https://www.ebi.ac.uk/metagenomics/api/v1/analyses/MGYA00353303/file/ERR1583066_FASTQ_SSU_OTU.tsv	Root-Environmental-Aquatic-Freshwater-Lake
ERR1429248	MGYA00107662	https://www.ebi.ac.uk/metagenomics/api/v1/analyses/MGYA00107662/file/ERR1429248_FASTQ_otu.tsv	Root-Environmental-Aquatic-Freshwater-Lake

ERR658194	MGYA00103470	https://www.ebi.ac.uk/metagenomics/api/v1/analyses/MGYA00103470/file/ERR658194_FASTQ_otu.tsv	Root-Environmental-Aquatic-Freshwater-Lake
ERR1897686	MGYA00351631	https://www.ebi.ac.uk/metagenomics/api/v1/analyses/MGYA00351631/file/ERR1897686_FASTQ_SSU_OTU.tsv	Root-Environmental-Aquatic-Freshwater-Lake
ERR1897121	MGYA00351066	https://www.ebi.ac.uk/metagenomics/api/v1/analyses/MGYA00351066/file/ERR1897121_FASTQ_SSU_OTU.tsv	Root-Environmental-Aquatic-Freshwater-Lake
ERR1896761	MGYA00350706	https://www.ebi.ac.uk/metagenomics/api/v1/analyses/MGYA00350706/file/ERR1896761_FASTQ_SSU_OTU.tsv	Root-Environmental-Aquatic-Freshwater-Lake
ERR1437517	MGYA00107955	https://www.ebi.ac.uk/metagenomics/api/v1/analyses/MGYA00107955/file/ERR1437517_FASTQ_otu.tsv	Root-Environmental-Aquatic-Freshwater-Lake
ERR589292	MGYA00103444	https://www.ebi.ac.uk/metagenomics/api/v1/analyses/MGYA00103444/file/ERR589292_FASTQ_otu.tsv	Root-Environmental-Aquatic-Freshwater-Lake
ERR1897540	MGYA00351485	https://www.ebi.ac.uk/metagenomics/api/v1/analyses/MGYA00351485/file/ERR1897540_FASTQ_SSU_OTU.tsv	Root-Environmental-Aquatic-Freshwater-Lake
ERR1897236	MGYA00351181	https://www.ebi.ac.uk/metagenomics/api/v1/analyses/MGYA00351181/file/ERR1897236_FASTQ_SSU_OTU.tsv	Root-Environmental-Aquatic-Freshwater-Lake
ERR1897591	MGYA00351536	https://www.ebi.ac.uk/metagenomics/api/v1/analyses/MGYA00351536/file/ERR1897591_FASTQ_SSU_OTU.tsv	Root-Environmental-Aquatic-Freshwater-Lake
ERR1896804	MGYA00350749	https://www.ebi.ac.uk/metagenomics/api/v1/analyses/MGYA00350749/file/ERR1896804_FASTQ_SSU_OTU.tsv	Root-Environmental-Aquatic-Freshwater-Lake
ERR1897657	MGYA00351602	https://www.ebi.ac.uk/metagenomics/api/v1/analyses/MGYA00351602/file/ERR1897657_FASTQ_SSU_OTU.tsv	Root-Environmental-Aquatic-Freshwater-Lake
ERR1582869	MGYA00353106	https://www.ebi.ac.uk/metagenomics/api/v1/analyses/MGYA00353106/file/ERR1582869_FASTQ_SSU_OTU.tsv	Root-Environmental-Aquatic-Freshwater-Lake

ERR1582685	MGYA00352922	https://www.ebi.ac.uk/metagenomics/api/v1/analyses/MGYA00352922/file/ERR1582685_FASTQ_SSU_OTU.tsv	Root-Environmental-Aquatic-Freshwater-Lake
ERR1582122	MGYA00352359	https://www.ebi.ac.uk/metagenomics/api/v1/analyses/MGYA00352359/file/ERR1582122_FASTQ_SSU_OTU.tsv	Root-Environmental-Aquatic-Freshwater-Lake
ERR1583303	MGYA00353540	https://www.ebi.ac.uk/metagenomics/api/v1/analyses/MGYA00353540/file/ERR1583303_FASTQ_SSU_OTU.tsv	Root-Environmental-Aquatic-Freshwater-Lake
ERR1582543	MGYA00352780	https://www.ebi.ac.uk/metagenomics/api/v1/analyses/MGYA00352780/file/ERR1582543_FASTQ_SSU_OTU.tsv	Root-Environmental-Aquatic-Freshwater-Lake
ERR1582931	MGYA00353168	https://www.ebi.ac.uk/metagenomics/api/v1/analyses/MGYA00353168/file/ERR1582931_FASTQ_SSU_OTU.tsv	Root-Environmental-Aquatic-Freshwater-Lake
ERR1897079	MGYA00351024	https://www.ebi.ac.uk/metagenomics/api/v1/analyses/MGYA00351024/file/ERR1897079_FASTQ_SSU_OTU.tsv	Root-Environmental-Aquatic-Freshwater-Lake
ERR1896639	MGYA00350584	https://www.ebi.ac.uk/metagenomics/api/v1/analyses/MGYA00350584/file/ERR1896639_FASTQ_SSU_OTU.tsv	Root-Environmental-Aquatic-Freshwater-Lake
ERR1582235	MGYA00352472	https://www.ebi.ac.uk/metagenomics/api/v1/analyses/MGYA00352472/file/ERR1582235_FASTQ_SSU_OTU.tsv	Root-Environmental-Aquatic-Freshwater-Lake
ERR1582592	MGYA00352829	https://www.ebi.ac.uk/metagenomics/api/v1/analyses/MGYA00352829/file/ERR1582592_FASTQ_SSU_OTU.tsv	Root-Environmental-Aquatic-Freshwater-Lake
ERR1583475	MGYA00353712	https://www.ebi.ac.uk/metagenomics/api/v1/analyses/MGYA00353712/file/ERR1583475_FASTQ_SSU_OTU.tsv	Root-Environmental-Aquatic-Freshwater-Lake
SRR7081203	MGYA00425947	https://www.ebi.ac.uk/metagenomics/api/v1/analyses/MGYA00425947/file/SRR7081203_MERGED_FASTQ_SSU_OTU.tsv	Root-Environmental-Aquatic-Freshwater-Lake
ERR1897418	MGYA00351363	https://www.ebi.ac.uk/metagenomics/api/v1/analyses/MGYA00351363/file/ERR1897418_FASTQ_SSU_OTU.tsv	Root-Environmental-Aquatic-Freshwater-Lake

ERR1583014	MGYA00353251	https://www.ebi.ac.uk/metagenomics/api/v1/analyses/MGYA00353251/file/ERR1583014_FASTQ_SSU_OTU.tsv	Root-Environmental-Aquatic-Freshwater-Lake
ERR1582654	MGYA00352891	https://www.ebi.ac.uk/metagenomics/api/v1/analyses/MGYA00352891/file/ERR1582654_FASTQ_SSU_OTU.tsv	Root-Environmental-Aquatic-Freshwater-Lake
ERR1429234	MGYA00107653	https://www.ebi.ac.uk/metagenomics/api/v1/analyses/MGYA00107653/file/ERR1429234_FASTQ_otu.tsv	Root-Environmental-Aquatic-Freshwater-Lake
ERR1582311	MGYA00352548	https://www.ebi.ac.uk/metagenomics/api/v1/analyses/MGYA00352548/file/ERR1582311_FASTQ_SSU_OTU.tsv	Root-Environmental-Aquatic-Freshwater-Lake
ERR1547070	MGYA00320781	https://www.ebi.ac.uk/metagenomics/api/v1/analyses/MGYA00320781/file/ERR1547070_FASTQ_SSU_OTU.tsv	Root-Environmental-Aquatic-Freshwater-Lake
ERR1583130	MGYA00353367	https://www.ebi.ac.uk/metagenomics/api/v1/analyses/MGYA00353367/file/ERR1583130_FASTQ_SSU_OTU.tsv	Root-Environmental-Aquatic-Freshwater-Lake
ERR1582770	MGYA00353007	https://www.ebi.ac.uk/metagenomics/api/v1/analyses/MGYA00353007/file/ERR1582770_FASTQ_SSU_OTU.tsv	Root-Environmental-Aquatic-Freshwater-Lake
ERR1314366	MGYA00095203	https://www.ebi.ac.uk/metagenomics/api/v1/analyses/MGYA00095203/file/ERR1314366_MERGED_FASTQ_otu.tsv	Root-Environmental-Aquatic-Freshwater-Lake
ERR389727	MGYA00103372	https://www.ebi.ac.uk/metagenomics/api/v1/analyses/MGYA00103372/file/ERR389727_FASTQ_otu.tsv	Root-Environmental-Aquatic-Freshwater-Lake
ERR1896878	MGYA00350823	https://www.ebi.ac.uk/metagenomics/api/v1/analyses/MGYA00350823/file/ERR1896878_FASTQ_SSU_OTU.tsv	Root-Environmental-Aquatic-Freshwater-Lake
ERR1583227	MGYA00353464	https://www.ebi.ac.uk/metagenomics/api/v1/analyses/MGYA00353464/file/ERR1583227_FASTQ_SSU_OTU.tsv	Root-Environmental-Aquatic-Freshwater-Lake
ERR1582815	MGYA00353052	https://www.ebi.ac.uk/metagenomics/api/v1/analyses/MGYA00353052/file/ERR1582815_FASTQ_SSU_OTU.tsv	Root-Environmental-Aquatic-Freshwater-Lake

ERR1582467	MGYA00352704	https://www.ebi.ac.uk/metagenomics/api/v1/analyses/MGYA00352704/file/ERR1582467_FASTQ_SSU_OTU.tsv	Root-Environmental-Aquatic-Freshwater-Lake
ERR1897312	MGYA00351257	https://www.ebi.ac.uk/metagenomics/api/v1/analyses/MGYA00351257/file/ERR1897312_FASTQ_SSU_OTU.tsv	Root-Environmental-Aquatic-Freshwater-Lake
ERR1896920	MGYA00350865	https://www.ebi.ac.uk/metagenomics/api/v1/analyses/MGYA00350865/file/ERR1896920_FASTQ_SSU_OTU.tsv	Root-Environmental-Aquatic-Freshwater-Lake
ERR1896694	MGYA00350639	https://www.ebi.ac.uk/metagenomics/api/v1/analyses/MGYA00350639/file/ERR1896694_FASTQ_SSU_OTU.tsv	Root-Environmental-Aquatic-Freshwater-Lake
SRR2443037	MGYA00359041	https://www.ebi.ac.uk/metagenomics/api/v1/analyses/MGYA00359041/file/SRR2443037_MERGED_FASTQ_SSU_OTU.tsv	Root-Environmental-Aquatic-Freshwater-Lake
ERR1897773	MGYA00351718	https://www.ebi.ac.uk/metagenomics/api/v1/analyses/MGYA00351718/file/ERR1897773_FASTQ_SSU_OTU.tsv	Root-Environmental-Aquatic-Freshwater-Lake
ERR1990484	MGYA00128223	https://www.ebi.ac.uk/metagenomics/api/v1/analyses/MGYA00128223/file/ERR1990484_MERGED_FASTQ_otu.tsv	Root-Environmental-Aquatic-Freshwater-Lake
ERR1582298	MGYA00352535	https://www.ebi.ac.uk/metagenomics/api/v1/analyses/MGYA00352535/file/ERR1582298_FASTQ_SSU_OTU.tsv	Root-Environmental-Aquatic-Freshwater-Lake
ERR1582249	MGYA00352486	https://www.ebi.ac.uk/metagenomics/api/v1/analyses/MGYA00352486/file/ERR1582249_FASTQ_SSU_OTU.tsv	Root-Environmental-Aquatic-Freshwater-Lake
ERR1583409	MGYA00353646	https://www.ebi.ac.uk/metagenomics/api/v1/analyses/MGYA00353646/file/ERR1583409_FASTQ_SSU_OTU.tsv	Root-Environmental-Aquatic-Freshwater-Lake
ERR1897005	MGYA00350950	https://www.ebi.ac.uk/metagenomics/api/v1/analyses/MGYA00350950/file/ERR1897005_FASTQ_SSU_OTU.tsv	Root-Environmental-Aquatic-Freshwater-Lake
ERR1896645	MGYA00350590	https://www.ebi.ac.uk/metagenomics/api/v1/analyses/MGYA00350590/file/ERR1896645_FASTQ_SSU_OTU.tsv	Root-Environmental-Aquatic-Freshwater-Lake

ERR1583068	MGYA00353305	https://www.ebi.ac.uk/metagenomics/api/v1/analyses/MGYA00353305/file/ERR1583068_FASTQ_SSU_OTU.tsv	Root-Environmental-Aquatic-Freshwater-Lake
ERR1582628	MGYA00352865	https://www.ebi.ac.uk/metagenomics/api/v1/analyses/MGYA00352865/file/ERR1582628_FASTQ_SSU_OTU.tsv	Root-Environmental-Aquatic-Freshwater-Lake
ERR1743317	MGYA00087168	https://www.ebi.ac.uk/metagenomics/api/v1/analyses/MGYA00087168/file/ERR1743317_MERGED_FASTQ_otu.tsv	Root-Environmental-Aquatic-Freshwater-Lake
ERR1897464	MGYA00351409	https://www.ebi.ac.uk/metagenomics/api/v1/analyses/MGYA00351409/file/ERR1897464_FASTQ_SSU_OTU.tsv	Root-Environmental-Aquatic-Freshwater-Lake
ERR1582316	MGYA00352553	https://www.ebi.ac.uk/metagenomics/api/v1/analyses/MGYA00352553/file/ERR1582316_FASTQ_SSU_OTU.tsv	Root-Environmental-Aquatic-Freshwater-Lake
ERR1429233	MGYA00107665	https://www.ebi.ac.uk/metagenomics/api/v1/analyses/MGYA00107665/file/ERR1429233_FASTQ_otu.tsv	Root-Environmental-Aquatic-Freshwater-Lake
ERR1547077	MGYA00320788	https://www.ebi.ac.uk/metagenomics/api/v1/analyses/MGYA00320788/file/ERR1547077_FASTQ_SSU_OTU.tsv	Root-Environmental-Aquatic-Freshwater-Lake
ERR1582777	MGYA00353014	https://www.ebi.ac.uk/metagenomics/api/v1/analyses/MGYA00353014/file/ERR1582777_FASTQ_SSU_OTU.tsv	Root-Environmental-Aquatic-Freshwater-Lake
ERR1583137	MGYA00353374	https://www.ebi.ac.uk/metagenomics/api/v1/analyses/MGYA00353374/file/ERR1583137_FASTQ_SSU_OTU.tsv	Root-Environmental-Aquatic-Freshwater-Lake
ERR1314361	MGYA00095175	https://www.ebi.ac.uk/metagenomics/api/v1/analyses/MGYA00095175/file/ERR1314361_MERGED_FASTQ_otu.tsv	Root-Environmental-Aquatic-Freshwater-Lake
ERR1743299	MGYA00087213	https://www.ebi.ac.uk/metagenomics/api/v1/analyses/MGYA00087213/file/ERR1743299_MERGED_FASTQ_otu.tsv	Root-Environmental-Aquatic-Freshwater-Lake
ERR1582460	MGYA00352697	https://www.ebi.ac.uk/metagenomics/api/v1/analyses/MGYA00352697/file/ERR1582460_FASTQ_SSU_OTU.tsv	Root-Environmental-Aquatic-Freshwater-Lake

ERR1582812	MGYA00353049	https://www.ebi.ac.uk/metagenomics/api/v1/analyses/MGYA00353049/file/ERR1582812_FASTQ_SSU_OTU.tsv	Root-Environmental-Aquatic-Freshwater-Lake
ERR1583220	MGYA00353457	https://www.ebi.ac.uk/metagenomics/api/v1/analyses/MGYA00353457/file/ERR1583220_FASTQ_SSU_OTU.tsv	Root-Environmental-Aquatic-Freshwater-Lake
ERR1896927	MGYA00350872	https://www.ebi.ac.uk/metagenomics/api/v1/analyses/MGYA00350872/file/ERR1896927_FASTQ_SSU_OTU.tsv	Root-Environmental-Aquatic-Freshwater-Lake
ERR1897315	MGYA00351260	https://www.ebi.ac.uk/metagenomics/api/v1/analyses/MGYA00351260/file/ERR1897315_FASTQ_SSU_OTU.tsv	Root-Environmental-Aquatic-Freshwater-Lake
ERR1582159	MGYA00352396	https://www.ebi.ac.uk/metagenomics/api/v1/analyses/MGYA00352396/file/ERR1582159_FASTQ_SSU_OTU.tsv	Root-Environmental-Aquatic-Freshwater-Lake
SRR2443030	MGYA00359034	https://www.ebi.ac.uk/metagenomics/api/v1/analyses/MGYA00359034/file/SRR2443030_MERGED_FASTQ_SSU_OTU.tsv	Root-Environmental-Aquatic-Freshwater-Lake
ERR1897774	MGYA00351719	https://www.ebi.ac.uk/metagenomics/api/v1/analyses/MGYA00351719/file/ERR1897774_FASTQ_SSU_OTU.tsv	Root-Environmental-Aquatic-Freshwater-Lake
ERR1896693	MGYA00350638	https://www.ebi.ac.uk/metagenomics/api/v1/analyses/MGYA00350638/file/ERR1896693_FASTQ_SSU_OTU.tsv	Root-Environmental-Aquatic-Freshwater-Lake
ERR1582538	MGYA00352775	https://www.ebi.ac.uk/metagenomics/api/v1/analyses/MGYA00352775/file/ERR1582538_FASTQ_SSU_OTU.tsv	Root-Environmental-Aquatic-Freshwater-Lake
ERR1990483	MGYA00128199	https://www.ebi.ac.uk/metagenomics/api/v1/analyses/MGYA00128199/file/ERR1990483_MERGED_FASTQ_otu.tsv	Root-Environmental-Aquatic-Freshwater-Lake
ERR1583378	MGYA00353615	https://www.ebi.ac.uk/metagenomics/api/v1/analyses/MGYA00353615/file/ERR1583378_FASTQ_SSU_OTU.tsv	Root-Environmental-Aquatic-Freshwater-Lake
ERR1896642	MGYA00350587	https://www.ebi.ac.uk/metagenomics/api/v1/analyses/MGYA00350587/file/ERR1896642_FASTQ_SSU_OTU.tsv	Root-Environmental-Aquatic-Freshwater-Lake

ERR1897002	MGYA00350947	https://www.ebi.ac.uk/metagenomics/api/v1/analyses/MGYA00350947/file/ERR1897002_FASTQ_SSU_OTU.tsv	Root-Environmental-Aquatic-Freshwater-Lake
ERR1582188	MGYA00352425	https://www.ebi.ac.uk/metagenomics/api/v1/analyses/MGYA00352425/file/ERR1582188_FASTQ_SSU_OTU.tsv	Root-Environmental-Aquatic-Freshwater-Lake
ERR1897463	MGYA00351408	https://www.ebi.ac.uk/metagenomics/api/v1/analyses/MGYA00351408/file/ERR1897463_FASTQ_SSU_OTU.tsv	Root-Environmental-Aquatic-Freshwater-Lake
ERR1743310	MGYA00087198	https://www.ebi.ac.uk/metagenomics/api/v1/analyses/MGYA00087198/file/ERR1743310_MERGED_FASTQ_otu.tsv	Root-Environmental-Aquatic-Freshwater-Lake
ERR658193	MGYA00103507	https://www.ebi.ac.uk/metagenomics/api/v1/analyses/MGYA00103507/file/ERR658193_FASTQ_otu.tsv	Root-Environmental-Aquatic-Freshwater-Lake
ERR1896766	MGYA00350711	https://www.ebi.ac.uk/metagenomics/api/v1/analyses/MGYA00350711/file/ERR1896766_FASTQ_SSU_OTU.tsv	Root-Environmental-Aquatic-Freshwater-Lake
ERR1897126	MGYA00351071	https://www.ebi.ac.uk/metagenomics/api/v1/analyses/MGYA00351071/file/ERR1897126_FASTQ_SSU_OTU.tsv	Root-Environmental-Aquatic-Freshwater-Lake
ERR1897681	MGYA00351626	https://www.ebi.ac.uk/metagenomics/api/v1/analyses/MGYA00351626/file/ERR1897681_FASTQ_SSU_OTU.tsv	Root-Environmental-Aquatic-Freshwater-Lake
ERR1897547	MGYA00351492	https://www.ebi.ac.uk/metagenomics/api/v1/analyses/MGYA00351492/file/ERR1897547_FASTQ_SSU_OTU.tsv	Root-Environmental-Aquatic-Freshwater-Lake
ERR589295	MGYA00103439	https://www.ebi.ac.uk/metagenomics/api/v1/analyses/MGYA00103439/file/ERR589295_FASTQ_otu.tsv	Root-Environmental-Aquatic-Freshwater-Lake
ERR1896803	MGYA00350748	https://www.ebi.ac.uk/metagenomics/api/v1/analyses/MGYA00350748/file/ERR1896803_FASTQ_SSU_OTU.tsv	Root-Environmental-Aquatic-Freshwater-Lake
ERR1897596	MGYA00351541	https://www.ebi.ac.uk/metagenomics/api/v1/analyses/MGYA00351541/file/ERR1897596_FASTQ_SSU_OTU.tsv	Root-Environmental-Aquatic-Freshwater-Lake

ERR1897231	MGYA00351176	https://www.ebi.ac.uk/metagenomics/api/v1/analyses/MGYA00351176/file/ERR1897231_FASTQ_SSU_OTU.tsv	Root-Environmental-Aquatic-Freshwater-Lake
ERR1897650	MGYA00351595	https://www.ebi.ac.uk/metagenomics/api/v1/analyses/MGYA00351595/file/ERR1897650_FASTQ_SSU_OTU.tsv	Root-Environmental-Aquatic-Freshwater-Lake
ERR1582125	MGYA00352362	https://www.ebi.ac.uk/metagenomics/api/v1/analyses/MGYA00352362/file/ERR1582125_FASTQ_SSU_OTU.tsv	Root-Environmental-Aquatic-Freshwater-Lake
ERR1582682	MGYA00352919	https://www.ebi.ac.uk/metagenomics/api/v1/analyses/MGYA00352919/file/ERR1582682_FASTQ_SSU_OTU.tsv	Root-Environmental-Aquatic-Freshwater-Lake
ERR1897369	MGYA00351314	https://www.ebi.ac.uk/metagenomics/api/v1/analyses/MGYA00351314/file/ERR1897369_FASTQ_SSU_OTU.tsv	Root-Environmental-Aquatic-Freshwater-Lake
ERR1582936	MGYA00353173	https://www.ebi.ac.uk/metagenomics/api/v1/analyses/MGYA00353173/file/ERR1582936_FASTQ_SSU_OTU.tsv	Root-Environmental-Aquatic-Freshwater-Lake
ERR1582544	MGYA00352781	https://www.ebi.ac.uk/metagenomics/api/v1/analyses/MGYA00352781/file/ERR1582544_FASTQ_SSU_OTU.tsv	Root-Environmental-Aquatic-Freshwater-Lake
ERR1583304	MGYA00353541	https://www.ebi.ac.uk/metagenomics/api/v1/analyses/MGYA00353541/file/ERR1583304_FASTQ_SSU_OTU.tsv	Root-Environmental-Aquatic-Freshwater-Lake
ERR1897708	MGYA00351653	https://www.ebi.ac.uk/metagenomics/api/v1/analyses/MGYA00351653/file/ERR1897708_FASTQ_SSU_OTU.tsv	Root-Environmental-Aquatic-Freshwater-Lake
ERR1583472	MGYA00353709	https://www.ebi.ac.uk/metagenomics/api/v1/analyses/MGYA00353709/file/ERR1583472_FASTQ_SSU_OTU.tsv	Root-Environmental-Aquatic-Freshwater-Lake
ERR1582595	MGYA00352832	https://www.ebi.ac.uk/metagenomics/api/v1/analyses/MGYA00352832/file/ERR1582595_FASTQ_SSU_OTU.tsv	Root-Environmental-Aquatic-Freshwater-Lake
ERR1582232	MGYA00352469	https://www.ebi.ac.uk/metagenomics/api/v1/analyses/MGYA00352469/file/ERR1582232_FASTQ_SSU_OTU.tsv	Root-Environmental-Aquatic-Freshwater-Lake

SRR2442969	MGYA00358973	https://www.ebi.ac.uk/metagenomics/api/v1/analyses/MGYA00358973/file/SRR2442969_MERGED_FASTQ_SSU_OTU.tsv	Root-Environmental-Aquatic-Freshwater-Lake
ERR1582653	MGYA00352890	https://www.ebi.ac.uk/metagenomics/api/v1/analyses/MGYA00352890/file/ERR1582653_FASTQ_SSU_OTU.tsv	Root-Environmental-Aquatic-Freshwater-Lake
ERR1583013	MGYA00353250	https://www.ebi.ac.uk/metagenomics/api/v1/analyses/MGYA00353250/file/ERR1583013_FASTQ_SSU_OTU.tsv	Root-Environmental-Aquatic-Freshwater-Lake
ERR1896653	MGYA00350598	https://www.ebi.ac.uk/metagenomics/api/v1/analyses/MGYA00350598/file/ERR1896653_FASTQ_SSU_OTU.tsv	Root-Environmental-Aquatic-Freshwater-Lake
ERR1897013	MGYA00350958	https://www.ebi.ac.uk/metagenomics/api/v1/analyses/MGYA00350958/file/ERR1897013_FASTQ_SSU_OTU.tsv	Root-Environmental-Aquatic-Freshwater-Lake
ERR1897472	MGYA00351417	https://www.ebi.ac.uk/metagenomics/api/v1/analyses/MGYA00351417/file/ERR1897472_FASTQ_SSU_OTU.tsv	Root-Environmental-Aquatic-Freshwater-Lake
ERR1743301	MGYA00087113	https://www.ebi.ac.uk/metagenomics/api/v1/analyses/MGYA00087113/file/ERR1743301_MERGED_FASTQ_otu.tsv	Root-Environmental-Aquatic-Freshwater-Lake
ERR1582199	MGYA00352436	https://www.ebi.ac.uk/metagenomics/api/v1/analyses/MGYA00352436/file/ERR1582199_FASTQ_SSU_OTU.tsv	Root-Environmental-Aquatic-Freshwater-Lake
ERR1582148	MGYA00352385	https://www.ebi.ac.uk/metagenomics/api/v1/analyses/MGYA00352385/file/ERR1582148_FASTQ_SSU_OTU.tsv	Root-Environmental-Aquatic-Freshwater-Lake
ERR1896936	MGYA00350881	https://www.ebi.ac.uk/metagenomics/api/v1/analyses/MGYA00350881/file/ERR1896936_FASTQ_SSU_OTU.tsv	Root-Environmental-Aquatic-Freshwater-Lake
ERR1897304	MGYA00351249	https://www.ebi.ac.uk/metagenomics/api/v1/analyses/MGYA00351249/file/ERR1897304_FASTQ_SSU_OTU.tsv	Root-Environmental-Aquatic-Freshwater-Lake
ERR1582529	MGYA00352766	https://www.ebi.ac.uk/metagenomics/api/v1/analyses/MGYA00352766/file/ERR1582529_FASTQ_SSU_OTU.tsv	Root-Environmental-Aquatic-Freshwater-Lake

ERR1990492	MGYA00128218	https://www.ebi.ac.uk/metagenomics/api/v1/analyses/MGYA00128218/file/ERR1990492_MERGED_FASTQ_otu.tsv	Root-Environmental-Aquatic-Freshwater-Lake
ERR1583369	MGYA00353606	https://www.ebi.ac.uk/metagenomics/api/v1/analyses/MGYA00353606/file/ERR1583369_FASTQ_SSU_OTU.tsv	Root-Environmental-Aquatic-Freshwater-Lake
SRR2443021	MGYA00359025	https://www.ebi.ac.uk/metagenomics/api/v1/analyses/MGYA00359025/file/SRR2443021_MERGED_FASTQ_SSU_OTU.tsv	Root-Environmental-Aquatic-Freshwater-Lake
ERR1897765	MGYA00351710	https://www.ebi.ac.uk/metagenomics/api/v1/analyses/MGYA00351710/file/ERR1897765_FASTQ_SSU_OTU.tsv	Root-Environmental-Aquatic-Freshwater-Lake
ERR1896682	MGYA00350627	https://www.ebi.ac.uk/metagenomics/api/v1/analyses/MGYA00350627/file/ERR1896682_FASTQ_SSU_OTU.tsv	Root-Environmental-Aquatic-Freshwater-Lake
ERR1743288	MGYA00087210	https://www.ebi.ac.uk/metagenomics/api/v1/analyses/MGYA00087210/file/ERR1743288_MERGED_FASTQ_otu.tsv	Root-Environmental-Aquatic-Freshwater-Lake
ERR1314370	MGYA00095219	https://www.ebi.ac.uk/metagenomics/api/v1/analyses/MGYA00095219/file/ERR1314370_MERGED_FASTQ_otu.tsv	Root-Environmental-Aquatic-Freshwater-Lake
ERR1582803	MGYA00353040	https://www.ebi.ac.uk/metagenomics/api/v1/analyses/MGYA00353040/file/ERR1582803_FASTQ_SSU_OTU.tsv	Root-Environmental-Aquatic-Freshwater-Lake
ERR1582471	MGYA00352708	https://www.ebi.ac.uk/metagenomics/api/v1/analyses/MGYA00352708/file/ERR1582471_FASTQ_SSU_OTU.tsv	Root-Environmental-Aquatic-Freshwater-Lake
ERR1583231	MGYA00353468	https://www.ebi.ac.uk/metagenomics/api/v1/analyses/MGYA00353468/file/ERR1583231_FASTQ_SSU_OTU.tsv	Root-Environmental-Aquatic-Freshwater-Lake
ERR1582307	MGYA00352544	https://www.ebi.ac.uk/metagenomics/api/v1/analyses/MGYA00352544/file/ERR1582307_FASTQ_SSU_OTU.tsv	Root-Environmental-Aquatic-Freshwater-Lake
ERR1429222	MGYA00107631	https://www.ebi.ac.uk/metagenomics/api/v1/analyses/MGYA00107631/file/ERR1429222_FASTQ_otu.tsv	Root-Environmental-Aquatic-Freshwater-Lake

ERR1314406	MGYA00095185	https://www.ebi.ac.uk/metagenomics/api/v1/analyses/MGYA00095185/file/ERR1314406_MERGED_FASTQ_otu.tsv	Root-Environmental-Aquatic-Freshwater-Lake
ERR1582766	MGYA00353003	https://www.ebi.ac.uk/metagenomics/api/v1/analyses/MGYA00353003/file/ERR1582766_FASTQ_SSU_OTU.tsv	Root-Environmental-Aquatic-Freshwater-Lake
ERR1583126	MGYA00353363	https://www.ebi.ac.uk/metagenomics/api/v1/analyses/MGYA00353363/file/ERR1583126_FASTQ_SSU_OTU.tsv	Root-Environmental-Aquatic-Freshwater-Lake
SRR7081331	MGYA00424195	https://www.ebi.ac.uk/metagenomics/api/v1/analyses/MGYA00424195/file/SRR7081331_MERGED_FASTQ_SSU_OTU.tsv	Root-Environmental-Aquatic-Freshwater-Lake
ERR1547066	MGYA00320777	https://www.ebi.ac.uk/metagenomics/api/v1/analyses/MGYA00320777/file/ERR1547066_FASTQ_SSU_OTU.tsv	Root-Environmental-Aquatic-Freshwater-Lake
ERR1583463	MGYA00353700	https://www.ebi.ac.uk/metagenomics/api/v1/analyses/MGYA00353700/file/ERR1583463_FASTQ_SSU_OTU.tsv	Root-Environmental-Aquatic-Freshwater-Lake
ERR1582223	MGYA00352460	https://www.ebi.ac.uk/metagenomics/api/v1/analyses/MGYA00352460/file/ERR1582223_FASTQ_SSU_OTU.tsv	Root-Environmental-Aquatic-Freshwater-Lake
ERR1582584	MGYA00352821	https://www.ebi.ac.uk/metagenomics/api/v1/analyses/MGYA00352821/file/ERR1582584_FASTQ_SSU_OTU.tsv	Root-Environmental-Aquatic-Freshwater-Lake
ERR1582642	MGYA00352879	https://www.ebi.ac.uk/metagenomics/api/v1/analyses/MGYA00352879/file/ERR1582642_FASTQ_SSU_OTU.tsv	Root-Environmental-Aquatic-Freshwater-Lake
ERR1583002	MGYA00353239	https://www.ebi.ac.uk/metagenomics/api/v1/analyses/MGYA00353239/file/ERR1583002_FASTQ_SSU_OTU.tsv	Root-Environmental-Aquatic-Freshwater-Lake
SRR2442978	MGYA00358982	https://www.ebi.ac.uk/metagenomics/api/v1/analyses/MGYA00358982/file/SRR2442978_MERGED_FASTQ_SSU_OTU.tsv	Root-Environmental-Aquatic-Freshwater-Lake
ERR1897378	MGYA00351323	https://www.ebi.ac.uk/metagenomics/api/v1/analyses/MGYA00351323/file/ERR1897378_FASTQ_SSU_OTU.tsv	Root-Environmental-Aquatic-Freshwater-Lake

ERR1582693	MGYA00352930	https://www.ebi.ac.uk/metagenomics/api/v1/analyses/MGYA00352930/file/ERR1582693_FASTQ_SSU_OTU.tsv	Root-Environmental-Aquatic-Freshwater-Lake
ERR1582134	MGYA00352371	https://www.ebi.ac.uk/metagenomics/api/v1/analyses/MGYA00352371/file/ERR1582134_FASTQ_SSU_OTU.tsv	Root-Environmental-Aquatic-Freshwater-Lake
ERR1897719	MGYA00351664	https://www.ebi.ac.uk/metagenomics/api/v1/analyses/MGYA00351664/file/ERR1897719_FASTQ_SSU_OTU.tsv	Root-Environmental-Aquatic-Freshwater-Lake
ERR1582555	MGYA00352792	https://www.ebi.ac.uk/metagenomics/api/v1/analyses/MGYA00352792/file/ERR1582555_FASTQ_SSU_OTU.tsv	Root-Environmental-Aquatic-Freshwater-Lake
ERR1582927	MGYA00353164	https://www.ebi.ac.uk/metagenomics/api/v1/analyses/MGYA00353164/file/ERR1582927_FASTQ_SSU_OTU.tsv	Root-Environmental-Aquatic-Freshwater-Lake
ERR1583315	MGYA00353552	https://www.ebi.ac.uk/metagenomics/api/v1/analyses/MGYA00353552/file/ERR1583315_FASTQ_SSU_OTU.tsv	Root-Environmental-Aquatic-Freshwater-Lake
ERR1896812	MGYA00350757	https://www.ebi.ac.uk/metagenomics/api/v1/analyses/MGYA00350757/file/ERR1896812_FASTQ_SSU_OTU.tsv	Root-Environmental-Aquatic-Freshwater-Lake
ERR1897220	MGYA00351165	https://www.ebi.ac.uk/metagenomics/api/v1/analyses/MGYA00351165/file/ERR1897220_FASTQ_SSU_OTU.tsv	Root-Environmental-Aquatic-Freshwater-Lake
ERR1897587	MGYA00351532	https://www.ebi.ac.uk/metagenomics/api/v1/analyses/MGYA00351532/file/ERR1897587_FASTQ_SSU_OTU.tsv	Root-Environmental-Aquatic-Freshwater-Lake
ERR1897641	MGYA00351586	https://www.ebi.ac.uk/metagenomics/api/v1/analyses/MGYA00351586/file/ERR1897641_FASTQ_SSU_OTU.tsv	Root-Environmental-Aquatic-Freshwater-Lake
ERR1896777	MGYA00350722	https://www.ebi.ac.uk/metagenomics/api/v1/analyses/MGYA00350722/file/ERR1896777_FASTQ_SSU_OTU.tsv	Root-Environmental-Aquatic-Freshwater-Lake
ERR1897690	MGYA00351635	https://www.ebi.ac.uk/metagenomics/api/v1/analyses/MGYA00351635/file/ERR1897690_FASTQ_SSU_OTU.tsv	Root-Environmental-Aquatic-Freshwater-Lake

ERR1897137	MGYA00351082	https://www.ebi.ac.uk/metagenomics/api/v1/analyses/MGYA00351082/file/ERR1897137_FASTQ_SSU_OTU.tsv	Root-Environmental-Aquatic-Freshwater-Lake
ERR658182	MGYA00103496	https://www.ebi.ac.uk/metagenomics/api/v1/analyses/MGYA00103496/file/ERR658182_FASTQ_otu.tsv	Root-Environmental-Aquatic-Freshwater-Lake
ERR1897556	MGYA00351501	https://www.ebi.ac.uk/metagenomics/api/v1/analyses/MGYA00351501/file/ERR1897556_FASTQ_SSU_OTU.tsv	Root-Environmental-Aquatic-Freshwater-Lake
ERR1582583	MGYA00352820	https://www.ebi.ac.uk/metagenomics/api/v1/analyses/MGYA00352820/file/ERR1582583_FASTQ_SSU_OTU.tsv	Root-Environmental-Aquatic-Freshwater-Lake
ERR1582224	MGYA00352461	https://www.ebi.ac.uk/metagenomics/api/v1/analyses/MGYA00352461/file/ERR1582224_FASTQ_SSU_OTU.tsv	Root-Environmental-Aquatic-Freshwater-Lake
ERR1583464	MGYA00353701	https://www.ebi.ac.uk/metagenomics/api/v1/analyses/MGYA00353701/file/ERR1583464_FASTQ_SSU_OTU.tsv	Root-Environmental-Aquatic-Freshwater-Lake
ERR1897068	MGYA00351013	https://www.ebi.ac.uk/metagenomics/api/v1/analyses/MGYA00351013/file/ERR1897068_FASTQ_SSU_OTU.tsv	Root-Environmental-Aquatic-Freshwater-Lake
ERR1583005	MGYA00353242	https://www.ebi.ac.uk/metagenomics/api/v1/analyses/MGYA00353242/file/ERR1583005_FASTQ_SSU_OTU.tsv	Root-Environmental-Aquatic-Freshwater-Lake
ERR1582645	MGYA00352882	https://www.ebi.ac.uk/metagenomics/api/v1/analyses/MGYA00352882/file/ERR1582645_FASTQ_SSU_OTU.tsv	Root-Environmental-Aquatic-Freshwater-Lake
ERR1897409	MGYA00351354	https://www.ebi.ac.uk/metagenomics/api/v1/analyses/MGYA00351354/file/ERR1897409_FASTQ_SSU_OTU.tsv	Root-Environmental-Aquatic-Freshwater-Lake
SRR7081212	MGYA00425710	https://www.ebi.ac.uk/metagenomics/api/v1/analyses/MGYA00425710/file/SRR7081212_MERGED_FASTQ_SSU_OTU.tsv	Root-Environmental-Aquatic-Freshwater-Lake
ERR1582133	MGYA00352370	https://www.ebi.ac.uk/metagenomics/api/v1/analyses/MGYA00352370/file/ERR1582133_FASTQ_SSU_OTU.tsv	Root-Environmental-Aquatic-Freshwater-Lake

ERR1582694	MGYA00352931	https://www.ebi.ac.uk/metagenomics/api/v1/analyses/MGYA00352931/file/ERR1582694_FASTQ_SSU_OTU.tsv	Root-Environmental-Aquatic-Freshwater-Lake
ERR1583312	MGYA00353549	https://www.ebi.ac.uk/metagenomics/api/v1/analyses/MGYA00353549/file/ERR1583312_FASTQ_SSU_OTU.tsv	Root-Environmental-Aquatic-Freshwater-Lake
ERR1582920	MGYA00353157	https://www.ebi.ac.uk/metagenomics/api/v1/analyses/MGYA00353157/file/ERR1582920_FASTQ_SSU_OTU.tsv	Root-Environmental-Aquatic-Freshwater-Lake
ERR1582552	MGYA00352789	https://www.ebi.ac.uk/metagenomics/api/v1/analyses/MGYA00352789/file/ERR1582552_FASTQ_SSU_OTU.tsv	Root-Environmental-Aquatic-Freshwater-Lake
ERR1897580	MGYA00351525	https://www.ebi.ac.uk/metagenomics/api/v1/analyses/MGYA00351525/file/ERR1897580_FASTQ_SSU_OTU.tsv	Root-Environmental-Aquatic-Freshwater-Lake
ERR1897227	MGYA00351172	https://www.ebi.ac.uk/metagenomics/api/v1/analyses/MGYA00351172/file/ERR1897227_FASTQ_SSU_OTU.tsv	Root-Environmental-Aquatic-Freshwater-Lake
ERR1896815	MGYA00350760	https://www.ebi.ac.uk/metagenomics/api/v1/analyses/MGYA00350760/file/ERR1896815_FASTQ_SSU_OTU.tsv	Root-Environmental-Aquatic-Freshwater-Lake
ERR1582878	MGYA00353115	https://www.ebi.ac.uk/metagenomics/api/v1/analyses/MGYA00353115/file/ERR1582878_FASTQ_SSU_OTU.tsv	Root-Environmental-Aquatic-Freshwater-Lake
ERR1897646	MGYA00351591	https://www.ebi.ac.uk/metagenomics/api/v1/analyses/MGYA00351591/file/ERR1897646_FASTQ_SSU_OTU.tsv	Root-Environmental-Aquatic-Freshwater-Lake
ERR1897130	MGYA00351075	https://www.ebi.ac.uk/metagenomics/api/v1/analyses/MGYA00351075/file/ERR1897130_FASTQ_SSU_OTU.tsv	Root-Environmental-Aquatic-Freshwater-Lake
ERR1897697	MGYA00351642	https://www.ebi.ac.uk/metagenomics/api/v1/analyses/MGYA00351642/file/ERR1897697_FASTQ_SSU_OTU.tsv	Root-Environmental-Aquatic-Freshwater-Lake
ERR1896770	MGYA00350715	https://www.ebi.ac.uk/metagenomics/api/v1/analyses/MGYA00350715/file/ERR1896770_FASTQ_SSU_OTU.tsv	Root-Environmental-Aquatic-Freshwater-Lake

ERR658185	MGYA00103494	https://www.ebi.ac.uk/metagenomics/api/v1/analyses/MGYA00103494/file/ERR658185_FASTQ_otu.tsv	Root-Environmental-Aquatic-Freshwater-Lake
ERR1429259	MGYA00107625	https://www.ebi.ac.uk/metagenomics/api/v1/analyses/MGYA00107625/file/ERR1429259_FASTQ_otu.tsv	Root-Environmental-Aquatic-Freshwater-Lake
ERR1897551	MGYA00351496	https://www.ebi.ac.uk/metagenomics/api/v1/analyses/MGYA00351496/file/ERR1897551_FASTQ_SSU_OTU.tsv	Root-Environmental-Aquatic-Freshwater-Lake
ERR1897014	MGYA00350959	https://www.ebi.ac.uk/metagenomics/api/v1/analyses/MGYA00350959/file/ERR1897014_FASTQ_SSU_OTU.tsv	Root-Environmental-Aquatic-Freshwater-Lake
ERR1896654	MGYA00350599	https://www.ebi.ac.uk/metagenomics/api/v1/analyses/MGYA00350599/file/ERR1896654_FASTQ_SSU_OTU.tsv	Root-Environmental-Aquatic-Freshwater-Lake
ERR1582258	MGYA00352495	https://www.ebi.ac.uk/metagenomics/api/v1/analyses/MGYA00352495/file/ERR1582258_FASTQ_SSU_OTU.tsv	Root-Environmental-Aquatic-Freshwater-Lake
ERR1583418	MGYA00353655	https://www.ebi.ac.uk/metagenomics/api/v1/analyses/MGYA00353655/file/ERR1583418_FASTQ_SSU_OTU.tsv	Root-Environmental-Aquatic-Freshwater-Lake
ERR1743306	MGYA00087106	https://www.ebi.ac.uk/metagenomics/api/v1/analyses/MGYA00087106/file/ERR1743306_MERGED_FASTQ_otu.tsv	Root-Environmental-Aquatic-Freshwater-Lake
ERR1897475	MGYA00351420	https://www.ebi.ac.uk/metagenomics/api/v1/analyses/MGYA00351420/file/ERR1897475_FASTQ_SSU_OTU.tsv	Root-Environmental-Aquatic-Freshwater-Lake
SRR3986665	MGYA00095520	https://www.ebi.ac.uk/metagenomics/api/v1/analyses/MGYA00095520/file/SRR3986665_MERGED_FASTQ_otu.tsv	Root-Environmental-Aquatic-Freshwater-Lake
ERR1583079	MGYA00353316	https://www.ebi.ac.uk/metagenomics/api/v1/analyses/MGYA00353316/file/ERR1583079_FASTQ_SSU_OTU.tsv	Root-Environmental-Aquatic-Freshwater-Lake
ERR1582639	MGYA00352876	https://www.ebi.ac.uk/metagenomics/api/v1/analyses/MGYA00352876/file/ERR1582639_FASTQ_SSU_OTU.tsv	Root-Environmental-Aquatic-Freshwater-Lake

ERR1897303	MGYA00351248	https://www.ebi.ac.uk/metagenomics/api/v1/analyses/MGYA00351248/file/ERR1897303_FASTQ_SSU_OTU.tsv	Root-Environmental-Aquatic-Freshwater-Lake
ERR1896931	MGYA00350876	https://www.ebi.ac.uk/metagenomics/api/v1/analyses/MGYA00350876/file/ERR1896931_FASTQ_SSU_OTU.tsv	Root-Environmental-Aquatic-Freshwater-Lake
ERR1582289	MGYA00352526	https://www.ebi.ac.uk/metagenomics/api/v1/analyses/MGYA00352526/file/ERR1582289_FASTQ_SSU_OTU.tsv	Root-Environmental-Aquatic-Freshwater-Lake
ERR1990495	MGYA00128222	https://www.ebi.ac.uk/metagenomics/api/v1/analyses/MGYA00128222/file/ERR1990495_MERGED_FASTQ_otu.tsv	Root-Environmental-Aquatic-Freshwater-Lake
ERR1896685	MGYA00350630	https://www.ebi.ac.uk/metagenomics/api/v1/analyses/MGYA00350630/file/ERR1896685_FASTQ_SSU_OTU.tsv	Root-Environmental-Aquatic-Freshwater-Lake
SRR2443026	MGYA00359030	https://www.ebi.ac.uk/metagenomics/api/v1/analyses/MGYA00359030/file/SRR2443026_MERGED_FASTQ_SSU_OTU.tsv	Root-Environmental-Aquatic-Freshwater-Lake
ERR1897762	MGYA00351707	https://www.ebi.ac.uk/metagenomics/api/v1/analyses/MGYA00351707/file/ERR1897762_FASTQ_SSU_OTU.tsv	Root-Environmental-Aquatic-Freshwater-Lake
ERR1896869	MGYA00350814	https://www.ebi.ac.uk/metagenomics/api/v1/analyses/MGYA00350814/file/ERR1896869_FASTQ_SSU_OTU.tsv	Root-Environmental-Aquatic-Freshwater-Lake
ERR1314377	MGYA00095168	https://www.ebi.ac.uk/metagenomics/api/v1/analyses/MGYA00095168/file/ERR1314377_MERGED_FASTQ_otu.tsv	Root-Environmental-Aquatic-Freshwater-Lake
ERR1582017	MGYA00352254	https://www.ebi.ac.uk/metagenomics/api/v1/analyses/MGYA00352254/file/ERR1582017_FASTQ_SSU_OTU.tsv	Root-Environmental-Aquatic-Freshwater-Lake
SRR7081021	MGYA00427380	https://www.ebi.ac.uk/metagenomics/api/v1/analyses/MGYA00427380/file/SRR7081021_MERGED_FASTQ_SSU_OTU.tsv	Root-Environmental-Aquatic-Freshwater-Lake
ERR1583236	MGYA00353473	https://www.ebi.ac.uk/metagenomics/api/v1/analyses/MGYA00353473/file/ERR1583236_FASTQ_SSU_OTU.tsv	Root-Environmental-Aquatic-Freshwater-Lake

ERR1582476	MGYA00352713	https://www.ebi.ac.uk/metagenomics/api/v1/analyses/MGYA00352713/file/ERR1582476_FASTQ_SSU_OTU.tsv	Root-Environmental-Aquatic-Freshwater-Lake
ERR1582804	MGYA00353041	https://www.ebi.ac.uk/metagenomics/api/v1/analyses/MGYA00353041/file/ERR1582804_FASTQ_SSU_OTU.tsv	Root-Environmental-Aquatic-Freshwater-Lake
ERR1429225	MGYA00107652	https://www.ebi.ac.uk/metagenomics/api/v1/analyses/MGYA00107652/file/ERR1429225_FASTQ_otu.tsv	Root-Environmental-Aquatic-Freshwater-Lake
ERR1582300	MGYA00352537	https://www.ebi.ac.uk/metagenomics/api/v1/analyses/MGYA00352537/file/ERR1582300_FASTQ_SSU_OTU.tsv	Root-Environmental-Aquatic-Freshwater-Lake
ERR1583121	MGYA00353358	https://www.ebi.ac.uk/metagenomics/api/v1/analyses/MGYA00353358/file/ERR1583121_FASTQ_SSU_OTU.tsv	Root-Environmental-Aquatic-Freshwater-Lake
ERR1582761	MGYA00352998	https://www.ebi.ac.uk/metagenomics/api/v1/analyses/MGYA00352998/file/ERR1582761_FASTQ_SSU_OTU.tsv	Root-Environmental-Aquatic-Freshwater-Lake
ERR1314401	MGYA00095223	https://www.ebi.ac.uk/metagenomics/api/v1/analyses/MGYA00095223/file/ERR1314401_MERGED_FASTQ_otu.tsv	Root-Environmental-Aquatic-Freshwater-Lake
ERR1547061	MGYA00320772	https://www.ebi.ac.uk/metagenomics/api/v1/analyses/MGYA00320772/file/ERR1547061_FASTQ_SSU_OTU.tsv	Root-Environmental-Aquatic-Freshwater-Lake
ERR1897066	MGYA00351011	https://www.ebi.ac.uk/metagenomics/api/v1/analyses/MGYA00351011/file/ERR1897066_FASTQ_SSU_OTU.tsv	Root-Environmental-Aquatic-Freshwater-Lake
ERR1897407	MGYA00351352	https://www.ebi.ac.uk/metagenomics/api/v1/analyses/MGYA00351352/file/ERR1897407_FASTQ_SSU_OTU.tsv	Root-Environmental-Aquatic-Freshwater-Lake
SRR2442971	MGYA00358975	https://www.ebi.ac.uk/metagenomics/api/v1/analyses/MGYA00358975/file/SRR2442971_MERGED_FASTQ_SSU_OTU.tsv	Root-Environmental-Aquatic-Freshwater-Lake
ERR1896992	MGYA00350937	https://www.ebi.ac.uk/metagenomics/api/v1/analyses/MGYA00350937/file/ERR1896992_FASTQ_SSU_OTU.tsv	Root-Environmental-Aquatic-Freshwater-Lake

ERR1896943	MGYA00350888	https://www.ebi.ac.uk/metagenomics/api/v1/analyses/MGYA00350888/file/ERR1896943_FASTQ_SSU_OTU.tsv	Root-Environmental-Aquatic-Freshwater-Lake
ERR589304	MGYA00103454	https://www.ebi.ac.uk/metagenomics/api/v1/analyses/MGYA00103454/file/ERR589304_FASTQ_otu.tsv	Root-Environmental-Aquatic-Freshwater-Lake
ERR1897371	MGYA00351316	https://www.ebi.ac.uk/metagenomics/api/v1/analyses/MGYA00351316/file/ERR1897371_FASTQ_SSU_OTU.tsv	Root-Environmental-Aquatic-Freshwater-Lake
ERR1897710	MGYA00351655	https://www.ebi.ac.uk/metagenomics/api/v1/analyses/MGYA00351655/file/ERR1897710_FASTQ_SSU_OTU.tsv	Root-Environmental-Aquatic-Freshwater-Lake
ERR1897229	MGYA00351174	https://www.ebi.ac.uk/metagenomics/api/v1/analyses/MGYA00351174/file/ERR1897229_FASTQ_SSU_OTU.tsv	Root-Environmental-Aquatic-Freshwater-Lake
ERR1583182	MGYA00353419	https://www.ebi.ac.uk/metagenomics/api/v1/analyses/MGYA00353419/file/ERR1583182_FASTQ_SSU_OTU.tsv	Root-Environmental-Aquatic-Freshwater-Lake
ERR1582065	MGYA00352302	https://www.ebi.ac.uk/metagenomics/api/v1/analyses/MGYA00352302/file/ERR1582065_FASTQ_SSU_OTU.tsv	Root-Environmental-Aquatic-Freshwater-Lake
SRR7081053	MGYA00426731	https://www.ebi.ac.uk/metagenomics/api/v1/analyses/MGYA00426731/file/SRR7081053_MERGED_FASTQ_SSU_OTU.tsv	Root-Environmental-Aquatic-Freshwater-Lake
ERR1897648	MGYA00351593	https://www.ebi.ac.uk/metagenomics/api/v1/analyses/MGYA00351593/file/ERR1897648_FASTQ_SSU_OTU.tsv	Root-Environmental-Aquatic-Freshwater-Lake
ERR1535964	MGYA00320255	https://www.ebi.ac.uk/metagenomics/api/v1/analyses/MGYA00320255/file/ERR1535964_FASTQ_SSU_OTU.tsv	Root-Environmental-Aquatic-Freshwater-Lake
ERR1742293	MGYA00311594	https://www.ebi.ac.uk/metagenomics/api/v1/analyses/MGYA00311594/file/ERR1742293_FASTQ_SSU_OTU.tsv	Root-Environmental-Aquatic-Freshwater-Lake
ERR1582404	MGYA00352641	https://www.ebi.ac.uk/metagenomics/api/v1/analyses/MGYA00352641/file/ERR1582404_FASTQ_SSU_OTU.tsv	Root-Environmental-Aquatic-Freshwater-Lake

ERR1582876	MGYA00353113	https://www.ebi.ac.uk/metagenomics/api/v1/analyses/MGYA00353113/file/ERR1582876_FASTQ_SSU_OTU.tsv	Root-Environmental-Aquatic-Freshwater-Lake
ERR1583244	MGYA00353481	https://www.ebi.ac.uk/metagenomics/api/v1/analyses/MGYA00353481/file/ERR1583244_FASTQ_SSU_OTU.tsv	Root-Environmental-Aquatic-Freshwater-Lake
ERR1583295	MGYA00353532	https://www.ebi.ac.uk/metagenomics/api/v1/analyses/MGYA00353532/file/ERR1583295_FASTQ_SSU_OTU.tsv	Root-Environmental-Aquatic-Freshwater-Lake
ERR1582372	MGYA00352609	https://www.ebi.ac.uk/metagenomics/api/v1/analyses/MGYA00352609/file/ERR1582372_FASTQ_SSU_OTU.tsv	Root-Environmental-Aquatic-Freshwater-Lake
ERR1429257	MGYA00107676	https://www.ebi.ac.uk/metagenomics/api/v1/analyses/MGYA00107676/file/ERR1429257_FASTQ_otu.tsv	Root-Environmental-Aquatic-Freshwater-Lake
ERR1897699	MGYA00351644	https://www.ebi.ac.uk/metagenomics/api/v1/analyses/MGYA00351644/file/ERR1897699_FASTQ_SSU_OTU.tsv	Root-Environmental-Aquatic-Freshwater-Lake
ERR1582713	MGYA00352950	https://www.ebi.ac.uk/metagenomics/api/v1/analyses/MGYA00352950/file/ERR1582713_FASTQ_SSU_OTU.tsv	Root-Environmental-Aquatic-Freshwater-Lake
ERR1583153	MGYA00353390	https://www.ebi.ac.uk/metagenomics/api/v1/analyses/MGYA00353390/file/ERR1583153_FASTQ_SSU_OTU.tsv	Root-Environmental-Aquatic-Freshwater-Lake
ERR1583416	MGYA00353653	https://www.ebi.ac.uk/metagenomics/api/v1/analyses/MGYA00353653/file/ERR1583416_FASTQ_SSU_OTU.tsv	Root-Environmental-Aquatic-Freshwater-Lake
ERR1582256	MGYA00352493	https://www.ebi.ac.uk/metagenomics/api/v1/analyses/MGYA00352493/file/ERR1582256_FASTQ_SSU_OTU.tsv	Root-Environmental-Aquatic-Freshwater-Lake
ERR430925	MGYA00103261	https://www.ebi.ac.uk/metagenomics/api/v1/analyses/MGYA00103261/file/ERR430925_FASTQ_otu.tsv	Root-Environmental-Aquatic-Freshwater-Lake
ERR1582983	MGYA00353220	https://www.ebi.ac.uk/metagenomics/api/v1/analyses/MGYA00353220/file/ERR1582983_FASTQ_SSU_OTU.tsv	Root-Environmental-Aquatic-Freshwater-Lake

ERR1582637	MGYA00352874	https://www.ebi.ac.uk/metagenomics/api/v1/analyses/MGYA00352874/file/ERR1582637_FASTQ_SSU_OTU.tsv	Root-Environmental-Aquatic-Freshwater-Lake
ERR1582190	MGYA00352427	https://www.ebi.ac.uk/metagenomics/api/v1/analyses/MGYA00352427/file/ERR1582190_FASTQ_SSU_OTU.tsv	Root-Environmental-Aquatic-Freshwater-Lake
ERR1583077	MGYA00353314	https://www.ebi.ac.uk/metagenomics/api/v1/analyses/MGYA00353314/file/ERR1583077_FASTQ_SSU_OTU.tsv	Root-Environmental-Aquatic-Freshwater-Lake
ERR1743308	MGYA00087186	https://www.ebi.ac.uk/metagenomics/api/v1/analyses/MGYA00087186/file/ERR1743308_MERGED_FASTQ_otu.tsv	Root-Environmental-Aquatic-Freshwater-Lake
ERR1582141	MGYA00352378	https://www.ebi.ac.uk/metagenomics/api/v1/analyses/MGYA00352378/file/ERR1582141_FASTQ_SSU_OTU.tsv	Root-Environmental-Aquatic-Freshwater-Lake
SRR2443028	MGYA00359032	https://www.ebi.ac.uk/metagenomics/api/v1/analyses/MGYA00359032/file/SRR2443028_MERGED_FASTQ_SSU_OTU.tsv	Root-Environmental-Aquatic-Freshwater-Lake
ERR1582287	MGYA00352524	https://www.ebi.ac.uk/metagenomics/api/v1/analyses/MGYA00352524/file/ERR1582287_FASTQ_SSU_OTU.tsv	Root-Environmental-Aquatic-Freshwater-Lake
ERR1582952	MGYA00353189	https://www.ebi.ac.uk/metagenomics/api/v1/analyses/MGYA00353189/file/ERR1582952_FASTQ_SSU_OTU.tsv	Root-Environmental-Aquatic-Freshwater-Lake
ERR1582520	MGYA00352757	https://www.ebi.ac.uk/metagenomics/api/v1/analyses/MGYA00352757/file/ERR1582520_FASTQ_SSU_OTU.tsv	Root-Environmental-Aquatic-Freshwater-Lake
ERR1583360	MGYA00353597	https://www.ebi.ac.uk/metagenomics/api/v1/analyses/MGYA00353597/file/ERR1583360_FASTQ_SSU_OTU.tsv	Root-Environmental-Aquatic-Freshwater-Lake
ERR1314379	MGYA00095160	https://www.ebi.ac.uk/metagenomics/api/v1/analyses/MGYA00095160/file/ERR1314379_MERGED_FASTQ_otu.tsv	Root-Environmental-Aquatic-Freshwater-Lake
ERR1582019	MGYA00352256	https://www.ebi.ac.uk/metagenomics/api/v1/analyses/MGYA00352256/file/ERR1582019_FASTQ_SSU_OTU.tsv	Root-Environmental-Aquatic-Freshwater-Lake

ERR1896867	MGYA00350812	https://www.ebi.ac.uk/metagenomics/api/v1/analyses/MGYA00350812/file/ERR1896867_FASTQ_SSU_OTU.tsv	Root-Environmental-Aquatic-Freshwater-Lake
ERR1743281	MGYA00087188	https://www.ebi.ac.uk/metagenomics/api/v1/analyses/MGYA00087188/file/ERR1743281_MERGED_FASTQ_otu.tsv	Root-Environmental-Aquatic-Freshwater-Lake
ERR1897255	MGYA00351200	https://www.ebi.ac.uk/metagenomics/api/v1/analyses/MGYA00351200/file/ERR1897255_FASTQ_SSU_OTU.tsv	Root-Environmental-Aquatic-Freshwater-Lake
ERR1582478	MGYA00352715	https://www.ebi.ac.uk/metagenomics/api/v1/analyses/MGYA00352715/file/ERR1582478_FASTQ_SSU_OTU.tsv	Root-Environmental-Aquatic-Freshwater-Lake
ERR1583238	MGYA00353475	https://www.ebi.ac.uk/metagenomics/api/v1/analyses/MGYA00353475/file/ERR1583238_FASTQ_SSU_OTU.tsv	Root-Environmental-Aquatic-Freshwater-Lake
ERR1897634	MGYA00351579	https://www.ebi.ac.uk/metagenomics/api/v1/analyses/MGYA00351579/file/ERR1897634_FASTQ_SSU_OTU.tsv	Root-Environmental-Aquatic-Freshwater-Lake
ERR1897193	MGYA00351138	https://www.ebi.ac.uk/metagenomics/api/v1/analyses/MGYA00351138/file/ERR1897193_FASTQ_SSU_OTU.tsv	Root-Environmental-Aquatic-Freshwater-Lake
ERR1896702	MGYA00350647	https://www.ebi.ac.uk/metagenomics/api/v1/analyses/MGYA00350647/file/ERR1896702_FASTQ_SSU_OTU.tsv	Root-Environmental-Aquatic-Freshwater-Lake
ERR1897142	MGYA00351087	https://www.ebi.ac.uk/metagenomics/api/v1/analyses/MGYA00351087/file/ERR1897142_FASTQ_SSU_OTU.tsv	Root-Environmental-Aquatic-Freshwater-Lake
ERR1897284	MGYA00351229	https://www.ebi.ac.uk/metagenomics/api/v1/analyses/MGYA00351229/file/ERR1897284_FASTQ_SSU_OTU.tsv	Root-Environmental-Aquatic-Freshwater-Lake
ERR1897523	MGYA00351468	https://www.ebi.ac.uk/metagenomics/api/v1/analyses/MGYA00351468/file/ERR1897523_FASTQ_SSU_OTU.tsv	Root-Environmental-Aquatic-Freshwater-Lake
ERR430922	MGYA00103249	https://www.ebi.ac.uk/metagenomics/api/v1/analyses/MGYA00103249/file/ERR430922_FASTQ_otu.tsv	Root-Environmental-Aquatic-Freshwater-Lake

ERR1582984	MGYA00353221	https://www.ebi.ac.uk/metagenomics/api/v1/analyses/MGYA00353221/file/ERR1582984_FASTQ_SSU_OTU.tsv	Root-Environmental-Aquatic-Freshwater-Lake
ERR1582251	MGYA00352488	https://www.ebi.ac.uk/metagenomics/api/v1/analyses/MGYA00352488/file/ERR1582251_FASTQ_SSU_OTU.tsv	Root-Environmental-Aquatic-Freshwater-Lake
ERR1583411	MGYA00353648	https://www.ebi.ac.uk/metagenomics/api/v1/analyses/MGYA00353648/file/ERR1583411_FASTQ_SSU_OTU.tsv	Root-Environmental-Aquatic-Freshwater-Lake
ERR1583070	MGYA00353307	https://www.ebi.ac.uk/metagenomics/api/v1/analyses/MGYA00353307/file/ERR1583070_FASTQ_SSU_OTU.tsv	Root-Environmental-Aquatic-Freshwater-Lake
ERR1582197	MGYA00352434	https://www.ebi.ac.uk/metagenomics/api/v1/analyses/MGYA00352434/file/ERR1582197_FASTQ_SSU_OTU.tsv	Root-Environmental-Aquatic-Freshwater-Lake
ERR1582630	MGYA00352867	https://www.ebi.ac.uk/metagenomics/api/v1/analyses/MGYA00352867/file/ERR1582630_FASTQ_SSU_OTU.tsv	Root-Environmental-Aquatic-Freshwater-Lake
ERR1896938	MGYA00350883	https://www.ebi.ac.uk/metagenomics/api/v1/analyses/MGYA00350883/file/ERR1896938_FASTQ_SSU_OTU.tsv	Root-Environmental-Aquatic-Freshwater-Lake
ERR1582146	MGYA00352383	https://www.ebi.ac.uk/metagenomics/api/v1/analyses/MGYA00352383/file/ERR1582146_FASTQ_SSU_OTU.tsv	Root-Environmental-Aquatic-Freshwater-Lake
SRR7081170	MGYA00425955	https://www.ebi.ac.uk/metagenomics/api/v1/analyses/MGYA00425955/file/SRR7081170_MERGED_FASTQ_SSU_OTU.tsv	Root-Environmental-Aquatic-Freshwater-Lake
ERR1583367	MGYA00353604	https://www.ebi.ac.uk/metagenomics/api/v1/analyses/MGYA00353604/file/ERR1583367_FASTQ_SSU_OTU.tsv	Root-Environmental-Aquatic-Freshwater-Lake
ERR1582527	MGYA00352764	https://www.ebi.ac.uk/metagenomics/api/v1/analyses/MGYA00352764/file/ERR1582527_FASTQ_SSU_OTU.tsv	Root-Environmental-Aquatic-Freshwater-Lake
ERR1582955	MGYA00353192	https://www.ebi.ac.uk/metagenomics/api/v1/analyses/MGYA00353192/file/ERR1582955_FASTQ_SSU_OTU.tsv	Root-Environmental-Aquatic-Freshwater-Lake

ERR1582280	MGYA00352517	https://www.ebi.ac.uk/metagenomics/api/v1/analyses/MGYA00352517/file/ERR1582280_FASTQ_SSU_OTU.tsv	Root-Environmental-Aquatic-Freshwater-Lake
ERR1897252	MGYA00351197	https://www.ebi.ac.uk/metagenomics/api/v1/analyses/MGYA00351197/file/ERR1897252_FASTQ_SSU_OTU.tsv	Root-Environmental-Aquatic-Freshwater-Lake
ERR1743286	MGYA00087120	https://www.ebi.ac.uk/metagenomics/api/v1/analyses/MGYA00087120/file/ERR1743286_MERGED_FASTQ_otu.tsv	Root-Environmental-Aquatic-Freshwater-Lake
ERR1896860	MGYA00350805	https://www.ebi.ac.uk/metagenomics/api/v1/analyses/MGYA00350805/file/ERR1896860_FASTQ_SSU_OTU.tsv	Root-Environmental-Aquatic-Freshwater-Lake
ERR1897194	MGYA00351139	https://www.ebi.ac.uk/metagenomics/api/v1/analyses/MGYA00351139/file/ERR1897194_FASTQ_SSU_OTU.tsv	Root-Environmental-Aquatic-Freshwater-Lake
ERR1897633	MGYA00351578	https://www.ebi.ac.uk/metagenomics/api/v1/analyses/MGYA00351578/file/ERR1897633_FASTQ_SSU_OTU.tsv	Root-Environmental-Aquatic-Freshwater-Lake
ERR1897145	MGYA00351090	https://www.ebi.ac.uk/metagenomics/api/v1/analyses/MGYA00351090/file/ERR1897145_FASTQ_SSU_OTU.tsv	Root-Environmental-Aquatic-Freshwater-Lake
ERR1896705	MGYA00350650	https://www.ebi.ac.uk/metagenomics/api/v1/analyses/MGYA00350650/file/ERR1896705_FASTQ_SSU_OTU.tsv	Root-Environmental-Aquatic-Freshwater-Lake
ERR1582309	MGYA00352546	https://www.ebi.ac.uk/metagenomics/api/v1/analyses/MGYA00352546/file/ERR1582309_FASTQ_SSU_OTU.tsv	Root-Environmental-Aquatic-Freshwater-Lake
ERR1547068	MGYA00320779	https://www.ebi.ac.uk/metagenomics/api/v1/analyses/MGYA00320779/file/ERR1547068_FASTQ_SSU_OTU.tsv	Root-Environmental-Aquatic-Freshwater-Lake
ERR1897524	MGYA00351469	https://www.ebi.ac.uk/metagenomics/api/v1/analyses/MGYA00351469/file/ERR1897524_FASTQ_SSU_OTU.tsv	Root-Environmental-Aquatic-Freshwater-Lake
ERR1897283	MGYA00351228	https://www.ebi.ac.uk/metagenomics/api/v1/analyses/MGYA00351228/file/ERR1897283_FASTQ_SSU_OTU.tsv	Root-Environmental-Aquatic-Freshwater-Lake

ERR1583128	MGYA00353365	https://www.ebi.ac.uk/metagenomics/api/v1/analyses/MGYA00353365/file/ERR1583128_FASTQ_SSU_OTU.tsv	Root-Environmental-Aquatic-Freshwater-Lake
ERR1314408	MGYA00095217	https://www.ebi.ac.uk/metagenomics/api/v1/analyses/MGYA00095217/file/ERR1314408_MERGED_FASTQ_otu.tsv	Root-Environmental-Aquatic-Freshwater-Lake
ERR1582768	MGYA00353005	https://www.ebi.ac.uk/metagenomics/api/v1/analyses/MGYA00353005/file/ERR1582768_FASTQ_SSU_OTU.tsv	Root-Environmental-Aquatic-Freshwater-Lake
ERR1897061	MGYA00351006	https://www.ebi.ac.uk/metagenomics/api/v1/analyses/MGYA00351006/file/ERR1897061_FASTQ_SSU_OTU.tsv	Root-Environmental-Aquatic-Freshwater-Lake
ERR1896995	MGYA00350940	https://www.ebi.ac.uk/metagenomics/api/v1/analyses/MGYA00350940/file/ERR1896995_FASTQ_SSU_OTU.tsv	Root-Environmental-Aquatic-Freshwater-Lake
SRR2442976	MGYA00358980	https://www.ebi.ac.uk/metagenomics/api/v1/analyses/MGYA00358980/file/SRR2442976_MERGED_FASTQ_SSU_OTU.tsv	Root-Environmental-Aquatic-Freshwater-Lake
ERR1743373	MGYA00087122	https://www.ebi.ac.uk/metagenomics/api/v1/analyses/MGYA00087122/file/ERR1743373_MERGED_FASTQ_otu.tsv	Root-Environmental-Aquatic-Freshwater-Lake
ERR1897400	MGYA00351345	https://www.ebi.ac.uk/metagenomics/api/v1/analyses/MGYA00351345/file/ERR1897400_FASTQ_SSU_OTU.tsv	Root-Environmental-Aquatic-Freshwater-Lake
ERR1897376	MGYA00351321	https://www.ebi.ac.uk/metagenomics/api/v1/analyses/MGYA00351321/file/ERR1897376_FASTQ_SSU_OTU.tsv	Root-Environmental-Aquatic-Freshwater-Lake
ERR589303	MGYA00103410	https://www.ebi.ac.uk/metagenomics/api/v1/analyses/MGYA00103410/file/ERR589303_FASTQ_otu.tsv	Root-Environmental-Aquatic-Freshwater-Lake
ERR1896944	MGYA00350889	https://www.ebi.ac.uk/metagenomics/api/v1/analyses/MGYA00350889/file/ERR1896944_FASTQ_SSU_OTU.tsv	Root-Environmental-Aquatic-Freshwater-Lake
ERR1582929	MGYA00353166	https://www.ebi.ac.uk/metagenomics/api/v1/analyses/MGYA00353166/file/ERR1582929_FASTQ_SSU_OTU.tsv	Root-Environmental-Aquatic-Freshwater-Lake

ERR1897717	MGYA00351662	https://www.ebi.ac.uk/metagenomics/api/v1/analyses/MGYA00351662/file/ERR1897717_FASTQ_SSU_OTU.tsv	Root-Environmental-Aquatic-Freshwater-Lake
ERR1897589	MGYA00351534	https://www.ebi.ac.uk/metagenomics/api/v1/analyses/MGYA00351534/file/ERR1897589_FASTQ_SSU_OTU.tsv	Root-Environmental-Aquatic-Freshwater-Lake
ERR1582062	MGYA00352299	https://www.ebi.ac.uk/metagenomics/api/v1/analyses/MGYA00352299/file/ERR1582062_FASTQ_SSU_OTU.tsv	Root-Environmental-Aquatic-Freshwater-Lake
ERR1583185	MGYA00353422	https://www.ebi.ac.uk/metagenomics/api/v1/analyses/MGYA00353422/file/ERR1583185_FASTQ_SSU_OTU.tsv	Root-Environmental-Aquatic-Freshwater-Lake
ERR1742294	MGYA00311595	https://www.ebi.ac.uk/metagenomics/api/v1/analyses/MGYA00311595/file/ERR1742294_FASTQ_SSU_OTU.tsv	Root-Environmental-Aquatic-Freshwater-Lake
ERR1535963	MGYA00320254	https://www.ebi.ac.uk/metagenomics/api/v1/analyses/MGYA00320254/file/ERR1535963_FASTQ_SSU_OTU.tsv	Root-Environmental-Aquatic-Freshwater-Lake
ERR1583243	MGYA00353480	https://www.ebi.ac.uk/metagenomics/api/v1/analyses/MGYA00353480/file/ERR1583243_FASTQ_SSU_OTU.tsv	Root-Environmental-Aquatic-Freshwater-Lake
ERR1429281	MGYA00107658	https://www.ebi.ac.uk/metagenomics/api/v1/analyses/MGYA00107658/file/ERR1429281_FASTQ_otu.tsv	Root-Environmental-Aquatic-Freshwater-Lake
ERR1582871	MGYA00353108	https://www.ebi.ac.uk/metagenomics/api/v1/analyses/MGYA00353108/file/ERR1582871_FASTQ_SSU_OTU.tsv	Root-Environmental-Aquatic-Freshwater-Lake
ERR1582403	MGYA00352640	https://www.ebi.ac.uk/metagenomics/api/v1/analyses/MGYA00352640/file/ERR1582403_FASTQ_SSU_OTU.tsv	Root-Environmental-Aquatic-Freshwater-Lake
ERR1429250	MGYA00107639	https://www.ebi.ac.uk/metagenomics/api/v1/analyses/MGYA00107639/file/ERR1429250_FASTQ_otu.tsv	Root-Environmental-Aquatic-Freshwater-Lake
ERR1582375	MGYA00352612	https://www.ebi.ac.uk/metagenomics/api/v1/analyses/MGYA00352612/file/ERR1582375_FASTQ_SSU_OTU.tsv	Root-Environmental-Aquatic-Freshwater-Lake

ERR1583292	MGYA00353529	https://www.ebi.ac.uk/metagenomics/api/v1/analyses/MGYA00353529/file/ERR1583292_FASTQ_SSU_OTU.tsv	Root-Environmental-Aquatic-Freshwater-Lake
ERR1897139	MGYA00351084	https://www.ebi.ac.uk/metagenomics/api/v1/analyses/MGYA00351084/file/ERR1897139_FASTQ_SSU_OTU.tsv	Root-Environmental-Aquatic-Freshwater-Lake
ERR1896779	MGYA00350724	https://www.ebi.ac.uk/metagenomics/api/v1/analyses/MGYA00350724/file/ERR1896779_FASTQ_SSU_OTU.tsv	Root-Environmental-Aquatic-Freshwater-Lake
ERR1583154	MGYA00353391	https://www.ebi.ac.uk/metagenomics/api/v1/analyses/MGYA00353391/file/ERR1583154_FASTQ_SSU_OTU.tsv	Root-Environmental-Aquatic-Freshwater-Lake
ERR1582714	MGYA00352951	https://www.ebi.ac.uk/metagenomics/api/v1/analyses/MGYA00352951/file/ERR1582714_FASTQ_SSU_OTU.tsv	Root-Environmental-Aquatic-Freshwater-Lake
ERR1897558	MGYA00351503	https://www.ebi.ac.uk/metagenomics/api/v1/analyses/MGYA00351503/file/ERR1897558_FASTQ_SSU_OTU.tsv	Root-Environmental-Aquatic-Freshwater-Lake
ERR1583352	MGYA00353589	https://www.ebi.ac.uk/metagenomics/api/v1/analyses/MGYA00353589/file/ERR1583352_FASTQ_SSU_OTU.tsv	Root-Environmental-Aquatic-Freshwater-Lake
ERR1582512	MGYA00352749	https://www.ebi.ac.uk/metagenomics/api/v1/analyses/MGYA00352749/file/ERR1582512_FASTQ_SSU_OTU.tsv	Root-Environmental-Aquatic-Freshwater-Lake
ERR1582960	MGYA00353197	https://www.ebi.ac.uk/metagenomics/api/v1/analyses/MGYA00353197/file/ERR1582960_FASTQ_SSU_OTU.tsv	Root-Environmental-Aquatic-Freshwater-Lake
ERR1897498	MGYA00351443	https://www.ebi.ac.uk/metagenomics/api/v1/analyses/MGYA00351443/file/ERR1897498_FASTQ_SSU_OTU.tsv	Root-Environmental-Aquatic-Freshwater-Lake
ERR1582173	MGYA00352410	https://www.ebi.ac.uk/metagenomics/api/v1/analyses/MGYA00352410/file/ERR1582173_FASTQ_SSU_OTU.tsv	Root-Environmental-Aquatic-Freshwater-Lake
ERR1583094	MGYA00353331	https://www.ebi.ac.uk/metagenomics/api/v1/analyses/MGYA00353331/file/ERR1583094_FASTQ_SSU_OTU.tsv	Root-Environmental-Aquatic-Freshwater-Lake

ERR1583045	MGYA00353282	https://www.ebi.ac.uk/metagenomics/api/v1/analyses/MGYA00353282/file/ERR1583045_FASTQ_SSU_OTU.tsv	Root-Environmental-Aquatic-Freshwater-Lake
ERR1582605	MGYA00352842	https://www.ebi.ac.uk/metagenomics/api/v1/analyses/MGYA00352842/file/ERR1582605_FASTQ_SSU_OTU.tsv	Root-Environmental-Aquatic-Freshwater-Lake
ERR1547105	MGYA00320816	https://www.ebi.ac.uk/metagenomics/api/v1/analyses/MGYA00320816/file/ERR1547105_FASTQ_SSU_OTU.tsv	Root-Environmental-Aquatic-Freshwater-Lake
ERR1897449	MGYA00351394	https://www.ebi.ac.uk/metagenomics/api/v1/analyses/MGYA00351394/file/ERR1897449_FASTQ_SSU_OTU.tsv	Root-Environmental-Aquatic-Freshwater-Lake
ERR1582264	MGYA00352501	https://www.ebi.ac.uk/metagenomics/api/v1/analyses/MGYA00352501/file/ERR1582264_FASTQ_SSU_OTU.tsv	Root-Environmental-Aquatic-Freshwater-Lake
ERR1583424	MGYA00353661	https://www.ebi.ac.uk/metagenomics/api/v1/analyses/MGYA00353661/file/ERR1583424_FASTQ_SSU_OTU.tsv	Root-Environmental-Aquatic-Freshwater-Lake
ERR1583383	MGYA00353620	https://www.ebi.ac.uk/metagenomics/api/v1/analyses/MGYA00353620/file/ERR1583383_FASTQ_SSU_OTU.tsv	Root-Environmental-Aquatic-Freshwater-Lake
ERR1897028	MGYA00350973	https://www.ebi.ac.uk/metagenomics/api/v1/analyses/MGYA00350973/file/ERR1897028_FASTQ_SSU_OTU.tsv	Root-Environmental-Aquatic-Freshwater-Lake
ERR1896668	MGYA00350613	https://www.ebi.ac.uk/metagenomics/api/v1/analyses/MGYA00350613/file/ERR1896668_FASTQ_SSU_OTU.tsv	Root-Environmental-Aquatic-Freshwater-Lake
ERR1896884	MGYA00350829	https://www.ebi.ac.uk/metagenomics/api/v1/analyses/MGYA00350829/file/ERR1896884_FASTQ_SSU_OTU.tsv	Root-Environmental-Aquatic-Freshwater-Lake
ERR1897511	MGYA00351456	https://www.ebi.ac.uk/metagenomics/api/v1/analyses/MGYA00351456/file/ERR1897511_FASTQ_SSU_OTU.tsv	Root-Environmental-Aquatic-Freshwater-Lake
ERR658203	MGYA00103500	https://www.ebi.ac.uk/metagenomics/api/v1/analyses/MGYA00103500/file/ERR658203_FASTQ_otu.tsv	Root-Environmental-Aquatic-Freshwater-Lake

ERR1897170	MGYA00351115	https://www.ebi.ac.uk/metagenomics/api/v1/analyses/MGYA00351115/file/ERR1897170_FASTQ_SSU_OTU.tsv	Root-Environmental-Aquatic-Freshwater-Lake
ERR1896730	MGYA00350675	https://www.ebi.ac.uk/metagenomics/api/v1/analyses/MGYA00350675/file/ERR1896730_FASTQ_SSU_OTU.tsv	Root-Environmental-Aquatic-Freshwater-Lake
ERR1582838	MGYA00353075	https://www.ebi.ac.uk/metagenomics/api/v1/analyses/MGYA00353075/file/ERR1582838_FASTQ_SSU_OTU.tsv	Root-Environmental-Aquatic-Freshwater-Lake
ERR1897606	MGYA00351551	https://www.ebi.ac.uk/metagenomics/api/v1/analyses/MGYA00351551/file/ERR1897606_FASTQ_SSU_OTU.tsv	Root-Environmental-Aquatic-Freshwater-Lake
ERR1897267	MGYA00351212	https://www.ebi.ac.uk/metagenomics/api/v1/analyses/MGYA00351212/file/ERR1897267_FASTQ_SSU_OTU.tsv	Root-Environmental-Aquatic-Freshwater-Lake
ERR1896855	MGYA00350800	https://www.ebi.ac.uk/metagenomics/api/v1/analyses/MGYA00350800/file/ERR1896855_FASTQ_SSU_OTU.tsv	Root-Environmental-Aquatic-Freshwater-Lake
ERR1583489	MGYA00353726	https://www.ebi.ac.uk/metagenomics/api/v1/analyses/MGYA00353726/file/ERR1583489_FASTQ_SSU_OTU.tsv	Root-Environmental-Aquatic-Freshwater-Lake
ERR1897085	MGYA00351030	https://www.ebi.ac.uk/metagenomics/api/v1/analyses/MGYA00351030/file/ERR1897085_FASTQ_SSU_OTU.tsv	Root-Environmental-Aquatic-Freshwater-Lake
ERR1897722	MGYA00351667	https://www.ebi.ac.uk/metagenomics/api/v1/analyses/MGYA00351667/file/ERR1897722_FASTQ_SSU_OTU.tsv	Root-Environmental-Aquatic-Freshwater-Lake
ERR589336	MGYA00103433	https://www.ebi.ac.uk/metagenomics/api/v1/analyses/MGYA00103433/file/ERR589336_FASTQ_otu.tsv	Root-Environmental-Aquatic-Freshwater-Lake
ERR1897343	MGYA00351288	https://www.ebi.ac.uk/metagenomics/api/v1/analyses/MGYA00351288/file/ERR1897343_FASTQ_SSU_OTU.tsv	Root-Environmental-Aquatic-Freshwater-Lake
SRR2442992	MGYA00358996	https://www.ebi.ac.uk/metagenomics/api/v1/analyses/MGYA00358996/file/SRR2442992_MERGED_FASTQ_SSU_OTU.tsv	Root-Environmental-Aquatic-Freshwater-Lake

ERR1896971	MGYA00350916	https://www.ebi.ac.uk/metagenomics/api/v1/analyses/MGYA00350916/file/ERR1896971_FASTQ_SSU_OTU.tsv	Root-Environmental-Aquatic-Freshwater-Lake
ERR1743346	MGYA00087153	https://www.ebi.ac.uk/metagenomics/api/v1/analyses/MGYA00087153/file/ERR1743346_MERGED_FASTQ_otu.tsv	Root-Environmental-Aquatic-Freshwater-Lake
ERR1897435	MGYA00351380	https://www.ebi.ac.uk/metagenomics/api/v1/analyses/MGYA00351380/file/ERR1897435_FASTQ_SSU_OTU.tsv	Root-Environmental-Aquatic-Freshwater-Lake
ERR1897392	MGYA00351337	https://www.ebi.ac.uk/metagenomics/api/v1/analyses/MGYA00351337/file/ERR1897392_FASTQ_SSU_OTU.tsv	Root-Environmental-Aquatic-Freshwater-Lake
ERR1583039	MGYA00353276	https://www.ebi.ac.uk/metagenomics/api/v1/analyses/MGYA00353276/file/ERR1583039_FASTQ_SSU_OTU.tsv	Root-Environmental-Aquatic-Freshwater-Lake
ERR1582679	MGYA00352916	https://www.ebi.ac.uk/metagenomics/api/v1/analyses/MGYA00352916/file/ERR1582679_FASTQ_SSU_OTU.tsv	Root-Environmental-Aquatic-Freshwater-Lake
ERR1897054	MGYA00350999	https://www.ebi.ac.uk/metagenomics/api/v1/analyses/MGYA00350999/file/ERR1897054_FASTQ_SSU_OTU.tsv	Root-Environmental-Aquatic-Freshwater-Lake
ERR1582218	MGYA00352455	https://www.ebi.ac.uk/metagenomics/api/v1/analyses/MGYA00352455/file/ERR1582218_FASTQ_SSU_OTU.tsv	Root-Environmental-Aquatic-Freshwater-Lake
ERR1583458	MGYA00353695	https://www.ebi.ac.uk/metagenomics/api/v1/analyses/MGYA00353695/file/ERR1583458_FASTQ_SSU_OTU.tsv	Root-Environmental-Aquatic-Freshwater-Lake
ERR1583161	MGYA00353398	https://www.ebi.ac.uk/metagenomics/api/v1/analyses/MGYA00353398/file/ERR1583161_FASTQ_SSU_OTU.tsv	Root-Environmental-Aquatic-Freshwater-Lake
ERR1582086	MGYA00352323	https://www.ebi.ac.uk/metagenomics/api/v1/analyses/MGYA00352323/file/ERR1582086_FASTQ_SSU_OTU.tsv	Root-Environmental-Aquatic-Freshwater-Lake
ERR1582721	MGYA00352958	https://www.ebi.ac.uk/metagenomics/api/v1/analyses/MGYA00352958/file/ERR1582721_FASTQ_SSU_OTU.tsv	Root-Environmental-Aquatic-Freshwater-Lake

ERR1582895	MGYA00353132	https://www.ebi.ac.uk/metagenomics/api/v1/analyses/MGYA00353132/file/ERR1582895_FASTQ_SSU_OTU.tsv	Root-Environmental-Aquatic-Freshwater-Lake
ERR1582340	MGYA00352577	https://www.ebi.ac.uk/metagenomics/api/v1/analyses/MGYA00352577/file/ERR1582340_FASTQ_SSU_OTU.tsv	Root-Environmental-Aquatic-Freshwater-Lake
ERR1429265	MGYA00107638	https://www.ebi.ac.uk/metagenomics/api/v1/analyses/MGYA00107638/file/ERR1429265_FASTQ_otu.tsv	Root-Environmental-Aquatic-Freshwater-Lake
ERR1583500	MGYA00353737	https://www.ebi.ac.uk/metagenomics/api/v1/analyses/MGYA00353737/file/ERR1583500_FASTQ_SSU_OTU.tsv	Root-Environmental-Aquatic-Freshwater-Lake
ERR1535987	MGYA00320278	https://www.ebi.ac.uk/metagenomics/api/v1/analyses/MGYA00320278/file/ERR1535987_FASTQ_SSU_OTU.tsv	Root-Environmental-Aquatic-Freshwater-Lake
ERR1535956	MGYA00320247	https://www.ebi.ac.uk/metagenomics/api/v1/analyses/MGYA00320247/file/ERR1535956_FASTQ_SSU_OTU.tsv	Root-Environmental-Aquatic-Freshwater-Lake
ERR1583276	MGYA00353513	https://www.ebi.ac.uk/metagenomics/api/v1/analyses/MGYA00353513/file/ERR1583276_FASTQ_SSU_OTU.tsv	Root-Environmental-Aquatic-Freshwater-Lake
ERR1333501	MGYA00107588	https://www.ebi.ac.uk/metagenomics/api/v1/analyses/MGYA00107588/file/ERR1333501_FASTQ_otu.tsv	Root-Environmental-Aquatic-Freshwater-Lake
ERR1582844	MGYA00353081	https://www.ebi.ac.uk/metagenomics/api/v1/analyses/MGYA00353081/file/ERR1582844_FASTQ_SSU_OTU.tsv	Root-Environmental-Aquatic-Freshwater-Lake
ERR1582436	MGYA00352673	https://www.ebi.ac.uk/metagenomics/api/v1/analyses/MGYA00352673/file/ERR1582436_FASTQ_SSU_OTU.tsv	Root-Environmental-Aquatic-Freshwater-Lake
ERR1582391	MGYA00352628	https://www.ebi.ac.uk/metagenomics/api/v1/analyses/MGYA00352628/file/ERR1582391_FASTQ_SSU_OTU.tsv	Root-Environmental-Aquatic-Freshwater-Lake
ERR1896829	MGYA00350774	https://www.ebi.ac.uk/metagenomics/api/v1/analyses/MGYA00350774/file/ERR1896829_FASTQ_SSU_OTU.tsv	Root-Environmental-Aquatic-Freshwater-Lake

ERR1582057	MGYA00352294	https://www.ebi.ac.uk/metagenomics/api/v1/analyses/MGYA00352294/file/ERR1582057_FASTQ_SSU_OTU.tsv	Root-Environmental-Aquatic-Freshwater-Lake
ERR1314337	MGYA00095193	https://www.ebi.ac.uk/metagenomics/api/v1/analyses/MGYA00095193/file/ERR1314337_MERGED_FASTQ_otu.tsv	Root-Environmental-Aquatic-Freshwater-Lake
ERR1582569	MGYA00352806	https://www.ebi.ac.uk/metagenomics/api/v1/analyses/MGYA00352806/file/ERR1582569_FASTQ_SSU_OTU.tsv	Root-Environmental-Aquatic-Freshwater-Lake
ERR1583329	MGYA00353566	https://www.ebi.ac.uk/metagenomics/api/v1/analyses/MGYA00353566/file/ERR1583329_FASTQ_SSU_OTU.tsv	Root-Environmental-Aquatic-Freshwater-Lake
ERR1897725	MGYA00351670	https://www.ebi.ac.uk/metagenomics/api/v1/analyses/MGYA00351670/file/ERR1897725_FASTQ_SSU_OTU.tsv	Root-Environmental-Aquatic-Freshwater-Lake
ERR1897082	MGYA00351027	https://www.ebi.ac.uk/metagenomics/api/v1/analyses/MGYA00351027/file/ERR1897082_FASTQ_SSU_OTU.tsv	Root-Environmental-Aquatic-Freshwater-Lake
ERR1582108	MGYA00352345	https://www.ebi.ac.uk/metagenomics/api/v1/analyses/MGYA00352345/file/ERR1582108_FASTQ_SSU_OTU.tsv	Root-Environmental-Aquatic-Freshwater-Lake
ERR1896976	MGYA00350921	https://www.ebi.ac.uk/metagenomics/api/v1/analyses/MGYA00350921/file/ERR1896976_FASTQ_SSU_OTU.tsv	Root-Environmental-Aquatic-Freshwater-Lake
ERR1743390	MGYA00087221	https://www.ebi.ac.uk/metagenomics/api/v1/analyses/MGYA00087221/file/ERR1743390_MERGED_FASTQ_otu.tsv	Root-Environmental-Aquatic-Freshwater-Lake
SRR2442995	MGYA00358999	https://www.ebi.ac.uk/metagenomics/api/v1/analyses/MGYA00358999/file/SRR2442995_MERGED_FASTQ_SSU_OTU.tsv	Root-Environmental-Aquatic-Freshwater-Lake
ERR1897344	MGYA00351289	https://www.ebi.ac.uk/metagenomics/api/v1/analyses/MGYA00351289/file/ERR1897344_FASTQ_SSU_OTU.tsv	Root-Environmental-Aquatic-Freshwater-Lake
ERR589331	MGYA00103437	https://www.ebi.ac.uk/metagenomics/api/v1/analyses/MGYA00103437/file/ERR589331_FASTQ_otu.tsv	Root-Environmental-Aquatic-Freshwater-Lake

ERR1897395	MGYA00351340	https://www.ebi.ac.uk/metagenomics/api/v1/analyses/MGYA00351340/file/ERR1897395_FASTQ_SSU_OTU.tsv	Root-Environmental-Aquatic-Freshwater-Lake
SRR7081229	MGYA00425698	https://www.ebi.ac.uk/metagenomics/api/v1/analyses/MGYA00425698/file/SRR7081229_MERGED_FASTQ_SSU_OTU.tsv	Root-Environmental-Aquatic-Freshwater-Lake
ERR1897432	MGYA00351377	https://www.ebi.ac.uk/metagenomics/api/v1/analyses/MGYA00351377/file/ERR1897432_FASTQ_SSU_OTU.tsv	Root-Environmental-Aquatic-Freshwater-Lake
ERR1743341	MGYA00087112	https://www.ebi.ac.uk/metagenomics/api/v1/analyses/MGYA00087112/file/ERR1743341_MERGED_FASTQ_otu.tsv	Root-Environmental-Aquatic-Freshwater-Lake
ERR1897053	MGYA00350998	https://www.ebi.ac.uk/metagenomics/api/v1/analyses/MGYA00350998/file/ERR1897053_FASTQ_SSU_OTU.tsv	Root-Environmental-Aquatic-Freshwater-Lake
ERR1582726	MGYA00352963	https://www.ebi.ac.uk/metagenomics/api/v1/analyses/MGYA00352963/file/ERR1582726_FASTQ_SSU_OTU.tsv	Root-Environmental-Aquatic-Freshwater-Lake
ERR1582081	MGYA00352318	https://www.ebi.ac.uk/metagenomics/api/v1/analyses/MGYA00352318/file/ERR1582081_FASTQ_SSU_OTU.tsv	Root-Environmental-Aquatic-Freshwater-Lake
ERR1583166	MGYA00353403	https://www.ebi.ac.uk/metagenomics/api/v1/analyses/MGYA00353403/file/ERR1583166_FASTQ_SSU_OTU.tsv	Root-Environmental-Aquatic-Freshwater-Lake
ERR1583507	MGYA00353744	https://www.ebi.ac.uk/metagenomics/api/v1/analyses/MGYA00353744/file/ERR1583507_FASTQ_SSU_OTU.tsv	Root-Environmental-Aquatic-Freshwater-Lake
ERR1429262	MGYA00107659	https://www.ebi.ac.uk/metagenomics/api/v1/analyses/MGYA00107659/file/ERR1429262_FASTQ_otu.tsv	Root-Environmental-Aquatic-Freshwater-Lake
ERR1582347	MGYA00352584	https://www.ebi.ac.uk/metagenomics/api/v1/analyses/MGYA00352584/file/ERR1582347_FASTQ_SSU_OTU.tsv	Root-Environmental-Aquatic-Freshwater-Lake
ERR1582892	MGYA00353129	https://www.ebi.ac.uk/metagenomics/api/v1/analyses/MGYA00353129/file/ERR1582892_FASTQ_SSU_OTU.tsv	Root-Environmental-Aquatic-Freshwater-Lake

ERR1535980	MGYA00320271	https://www.ebi.ac.uk/metagenomics/api/v1/analyses/MGYA00320271/file/ERR1535980_FASTQ_SSU_OTU.tsv	Root-Environmental-Aquatic-Freshwater-Lake
ERR1535951	MGYA00320242	https://www.ebi.ac.uk/metagenomics/api/v1/analyses/MGYA00320242/file/ERR1535951_FASTQ_SSU_OTU.tsv	Root-Environmental-Aquatic-Freshwater-Lake
ERR1582396	MGYA00352633	https://www.ebi.ac.uk/metagenomics/api/v1/analyses/MGYA00352633/file/ERR1582396_FASTQ_SSU_OTU.tsv	Root-Environmental-Aquatic-Freshwater-Lake
ERR1582431	MGYA00352668	https://www.ebi.ac.uk/metagenomics/api/v1/analyses/MGYA00352668/file/ERR1582431_FASTQ_SSU_OTU.tsv	Root-Environmental-Aquatic-Freshwater-Lake
ERR1582843	MGYA00353080	https://www.ebi.ac.uk/metagenomics/api/v1/analyses/MGYA00353080/file/ERR1582843_FASTQ_SSU_OTU.tsv	Root-Environmental-Aquatic-Freshwater-Lake
ERR1333506	MGYA00107598	https://www.ebi.ac.uk/metagenomics/api/v1/analyses/MGYA00107598/file/ERR1333506_FASTQ_otu.tsv	Root-Environmental-Aquatic-Freshwater-Lake
ERR1583271	MGYA00353508	https://www.ebi.ac.uk/metagenomics/api/v1/analyses/MGYA00353508/file/ERR1583271_FASTQ_SSU_OTU.tsv	Root-Environmental-Aquatic-Freshwater-Lake
SRR7081407	MGYA00425683	https://www.ebi.ac.uk/metagenomics/api/v1/analyses/MGYA00425683/file/SRR7081407_MERGED_FASTQ_SSU_OTU.tsv	Root-Environmental-Aquatic-Freshwater-Lake
ERR1314330	MGYA00095213	https://www.ebi.ac.uk/metagenomics/api/v1/analyses/MGYA00095213/file/ERR1314330_MERGED_FASTQ_otu.tsv	Root-Environmental-Aquatic-Freshwater-Lake
ERR1582050	MGYA00352287	https://www.ebi.ac.uk/metagenomics/api/v1/analyses/MGYA00352287/file/ERR1582050_FASTQ_SSU_OTU.tsv	Root-Environmental-Aquatic-Freshwater-Lake
ERR1897759	MGYA00351704	https://www.ebi.ac.uk/metagenomics/api/v1/analyses/MGYA00351704/file/ERR1897759_FASTQ_SSU_OTU.tsv	Root-Environmental-Aquatic-Freshwater-Lake
ERR1582967	MGYA00353204	https://www.ebi.ac.uk/metagenomics/api/v1/analyses/MGYA00353204/file/ERR1582967_FASTQ_SSU_OTU.tsv	Root-Environmental-Aquatic-Freshwater-Lake

ERR1582515	MGYA00352752	https://www.ebi.ac.uk/metagenomics/api/v1/analyses/MGYA00352752/file/ERR1582515_FASTQ_SSU_OTU.tsv	Root-Environmental-Aquatic-Freshwater-Lake
ERR1583355	MGYA00353592	https://www.ebi.ac.uk/metagenomics/api/v1/analyses/MGYA00353592/file/ERR1583355_FASTQ_SSU_OTU.tsv	Root-Environmental-Aquatic-Freshwater-Lake
ERR1897338	MGYA00351283	https://www.ebi.ac.uk/metagenomics/api/v1/analyses/MGYA00351283/file/ERR1897338_FASTQ_SSU_OTU.tsv	Root-Environmental-Aquatic-Freshwater-Lake
ERR1583093	MGYA00353330	https://www.ebi.ac.uk/metagenomics/api/v1/analyses/MGYA00353330/file/ERR1583093_FASTQ_SSU_OTU.tsv	Root-Environmental-Aquatic-Freshwater-Lake
ERR1582174	MGYA00352411	https://www.ebi.ac.uk/metagenomics/api/v1/analyses/MGYA00352411/file/ERR1582174_FASTQ_SSU_OTU.tsv	Root-Environmental-Aquatic-Freshwater-Lake
ERR1582602	MGYA00352839	https://www.ebi.ac.uk/metagenomics/api/v1/analyses/MGYA00352839/file/ERR1582602_FASTQ_SSU_OTU.tsv	Root-Environmental-Aquatic-Freshwater-Lake
ERR1583042	MGYA00353279	https://www.ebi.ac.uk/metagenomics/api/v1/analyses/MGYA00353279/file/ERR1583042_FASTQ_SSU_OTU.tsv	Root-Environmental-Aquatic-Freshwater-Lake
ERR1547102	MGYA00320813	https://www.ebi.ac.uk/metagenomics/api/v1/analyses/MGYA00320813/file/ERR1547102_FASTQ_SSU_OTU.tsv	Root-Environmental-Aquatic-Freshwater-Lake
ERR1583384	MGYA00353621	https://www.ebi.ac.uk/metagenomics/api/v1/analyses/MGYA00353621/file/ERR1583384_FASTQ_SSU_OTU.tsv	Root-Environmental-Aquatic-Freshwater-Lake
ERR1583423	MGYA00353660	https://www.ebi.ac.uk/metagenomics/api/v1/analyses/MGYA00353660/file/ERR1583423_FASTQ_SSU_OTU.tsv	Root-Environmental-Aquatic-Freshwater-Lake
ERR1582263	MGYA00352500	https://www.ebi.ac.uk/metagenomics/api/v1/analyses/MGYA00352500/file/ERR1582263_FASTQ_SSU_OTU.tsv	Root-Environmental-Aquatic-Freshwater-Lake
ERR1897516	MGYA00351461	https://www.ebi.ac.uk/metagenomics/api/v1/analyses/MGYA00351461/file/ERR1897516_FASTQ_SSU_OTU.tsv	Root-Environmental-Aquatic-Freshwater-Lake

ERR1896883	MGYA00350828	https://www.ebi.ac.uk/metagenomics/api/v1/analyses/MGYA00350828/file/ERR1896883_FASTQ_SSU_OTU.tsv	Root-Environmental-Aquatic-Freshwater-Lake
ERR658204	MGYA00103498	https://www.ebi.ac.uk/metagenomics/api/v1/analyses/MGYA00103498/file/ERR658204_FASTQ_otu.tsv	Root-Environmental-Aquatic-Freshwater-Lake
ERR1896737	MGYA00350682	https://www.ebi.ac.uk/metagenomics/api/v1/analyses/MGYA00350682/file/ERR1896737_FASTQ_SSU_OTU.tsv	Root-Environmental-Aquatic-Freshwater-Lake
ERR1897177	MGYA00351122	https://www.ebi.ac.uk/metagenomics/api/v1/analyses/MGYA00351122/file/ERR1897177_FASTQ_SSU_OTU.tsv	Root-Environmental-Aquatic-Freshwater-Lake
ERR1897601	MGYA00351546	https://www.ebi.ac.uk/metagenomics/api/v1/analyses/MGYA00351546/file/ERR1897601_FASTQ_SSU_OTU.tsv	Root-Environmental-Aquatic-Freshwater-Lake
ERR1896852	MGYA00350797	https://www.ebi.ac.uk/metagenomics/api/v1/analyses/MGYA00350797/file/ERR1896852_FASTQ_SSU_OTU.tsv	Root-Environmental-Aquatic-Freshwater-Lake
ERR1897260	MGYA00351205	https://www.ebi.ac.uk/metagenomics/api/v1/analyses/MGYA00351205/file/ERR1897260_FASTQ_SSU_OTU.tsv	Root-Environmental-Aquatic-Freshwater-Lake
ERR1583480	MGYA00353717	https://www.ebi.ac.uk/metagenomics/api/v1/analyses/MGYA00353717/file/ERR1583480_FASTQ_SSU_OTU.tsv	Root-Environmental-Aquatic-Freshwater-Lake
ERR1583327	MGYA00353564	https://www.ebi.ac.uk/metagenomics/api/v1/analyses/MGYA00353564/file/ERR1583327_FASTQ_SSU_OTU.tsv	Root-Environmental-Aquatic-Freshwater-Lake
ERR1582915	MGYA00353152	https://www.ebi.ac.uk/metagenomics/api/v1/analyses/MGYA00353152/file/ERR1582915_FASTQ_SSU_OTU.tsv	Root-Environmental-Aquatic-Freshwater-Lake
ERR1582567	MGYA00352804	https://www.ebi.ac.uk/metagenomics/api/v1/analyses/MGYA00352804/file/ERR1582567_FASTQ_SSU_OTU.tsv	Root-Environmental-Aquatic-Freshwater-Lake
ERR1896978	MGYA00350923	https://www.ebi.ac.uk/metagenomics/api/v1/analyses/MGYA00350923/file/ERR1896978_FASTQ_SSU_OTU.tsv	Root-Environmental-Aquatic-Freshwater-Lake

ERR1582106	MGYA00352343	https://www.ebi.ac.uk/metagenomics/api/v1/analyses/MGYA00352343/file/ERR1582106_FASTQ_SSU_OTU.tsv	Root-Environmental-Aquatic-Freshwater-Lake
ERR1583030	MGYA00353267	https://www.ebi.ac.uk/metagenomics/api/v1/analyses/MGYA00353267/file/ERR1583030_FASTQ_SSU_OTU.tsv	Root-Environmental-Aquatic-Freshwater-Lake
ERR1582670	MGYA00352907	https://www.ebi.ac.uk/metagenomics/api/v1/analyses/MGYA00352907/file/ERR1582670_FASTQ_SSU_OTU.tsv	Root-Environmental-Aquatic-Freshwater-Lake
SRR7081227	MGYA00425701	https://www.ebi.ac.uk/metagenomics/api/v1/analyses/MGYA00425701/file/SRR7081227_MERGED_FASTQ_SSU_OTU.tsv	Root-Environmental-Aquatic-Freshwater-Lake
ERR1582211	MGYA00352448	https://www.ebi.ac.uk/metagenomics/api/v1/analyses/MGYA00352448/file/ERR1582211_FASTQ_SSU_OTU.tsv	Root-Environmental-Aquatic-Freshwater-Lake
ERR1583451	MGYA00353688	https://www.ebi.ac.uk/metagenomics/api/v1/analyses/MGYA00353688/file/ERR1583451_FASTQ_SSU_OTU.tsv	Root-Environmental-Aquatic-Freshwater-Lake
ERR1897564	MGYA00351509	https://www.ebi.ac.uk/metagenomics/api/v1/analyses/MGYA00351509/file/ERR1897564_FASTQ_SSU_OTU.tsv	Root-Environmental-Aquatic-Freshwater-Lake
ERR1583168	MGYA00353405	https://www.ebi.ac.uk/metagenomics/api/v1/analyses/MGYA00353405/file/ERR1583168_FASTQ_SSU_OTU.tsv	Root-Environmental-Aquatic-Freshwater-Lake
ERR1582728	MGYA00352965	https://www.ebi.ac.uk/metagenomics/api/v1/analyses/MGYA00352965/file/ERR1582728_FASTQ_SSU_OTU.tsv	Root-Environmental-Aquatic-Freshwater-Lake
ERR1897105	MGYA00351050	https://www.ebi.ac.uk/metagenomics/api/v1/analyses/MGYA00351050/file/ERR1897105_FASTQ_SSU_OTU.tsv	Root-Environmental-Aquatic-Freshwater-Lake
ERR1742279	MGYA00311580	https://www.ebi.ac.uk/metagenomics/api/v1/analyses/MGYA00311580/file/ERR1742279_FASTQ_SSU_OTU.tsv	Root-Environmental-Aquatic-Freshwater-Lake
ERR1896745	MGYA00350690	https://www.ebi.ac.uk/metagenomics/api/v1/analyses/MGYA00350690/file/ERR1896745_FASTQ_SSU_OTU.tsv	Root-Environmental-Aquatic-Freshwater-Lake

ERR1582349	MGYA00352586	https://www.ebi.ac.uk/metagenomics/api/v1/analyses/MGYA00352586/file/ERR1582349_FASTQ_SSU_OTU.tsv	Root-Environmental-Aquatic-Freshwater-Lake
ERR1583509	MGYA00353746	https://www.ebi.ac.uk/metagenomics/api/v1/analyses/MGYA00353746/file/ERR1583509_FASTQ_SSU_OTU.tsv	Root-Environmental-Aquatic-Freshwater-Lake
ERR1333508	MGYA00107592	https://www.ebi.ac.uk/metagenomics/api/v1/analyses/MGYA00107592/file/ERR1333508_FASTQ_otu.tsv	Root-Environmental-Aquatic-Freshwater-Lake
ERR1582398	MGYA00352635	https://www.ebi.ac.uk/metagenomics/api/v1/analyses/MGYA00352635/file/ERR1582398_FASTQ_SSU_OTU.tsv	Root-Environmental-Aquatic-Freshwater-Lake
ERR1896794	MGYA00350739	https://www.ebi.ac.uk/metagenomics/api/v1/analyses/MGYA00350739/file/ERR1896794_FASTQ_SSU_OTU.tsv	Root-Environmental-Aquatic-Freshwater-Lake
ERR1897673	MGYA00351618	https://www.ebi.ac.uk/metagenomics/api/v1/analyses/MGYA00351618/file/ERR1897673_FASTQ_SSU_OTU.tsv	Root-Environmental-Aquatic-Freshwater-Lake
ERR1897212	MGYA00351157	https://www.ebi.ac.uk/metagenomics/api/v1/analyses/MGYA00351157/file/ERR1897212_FASTQ_SSU_OTU.tsv	Root-Environmental-Aquatic-Freshwater-Lake
ERR1896820	MGYA00350765	https://www.ebi.ac.uk/metagenomics/api/v1/analyses/MGYA00350765/file/ERR1896820_FASTQ_SSU_OTU.tsv	Root-Environmental-Aquatic-Freshwater-Lake
ERR1582969	MGYA00353206	https://www.ebi.ac.uk/metagenomics/api/v1/analyses/MGYA00353206/file/ERR1582969_FASTQ_SSU_OTU.tsv	Root-Environmental-Aquatic-Freshwater-Lake
ERR1897757	MGYA00351702	https://www.ebi.ac.uk/metagenomics/api/v1/analyses/MGYA00351702/file/ERR1897757_FASTQ_SSU_OTU.tsv	Root-Environmental-Aquatic-Freshwater-Lake
SRR2443013	MGYA00359017	https://www.ebi.ac.uk/metagenomics/api/v1/analyses/MGYA00359017/file/SRR2443013_MERGED_FASTQ_SSU_OTU.tsv	Root-Environmental-Aquatic-Freshwater-Lake
ERR1897491	MGYA00351436	https://www.ebi.ac.uk/metagenomics/api/v1/analyses/MGYA00351436/file/ERR1897491_FASTQ_SSU_OTU.tsv	Root-Environmental-Aquatic-Freshwater-Lake

ERR589343	MGYA00103451	https://www.ebi.ac.uk/metagenomics/api/v1/analyses/MGYA00103451/file/ERR589343_FASTQ_otu.tsv	Root-Environmental-Aquatic-Freshwater-Lake
ERR1897336	MGYA00351281	https://www.ebi.ac.uk/metagenomics/api/v1/analyses/MGYA00351281/file/ERR1897336_FASTQ_SSU_OTU.tsv	Root-Environmental-Aquatic-Freshwater-Lake
ERR1896904	MGYA00350849	https://www.ebi.ac.uk/metagenomics/api/v1/analyses/MGYA00350849/file/ERR1896904_FASTQ_SSU_OTU.tsv	Root-Environmental-Aquatic-Freshwater-Lake
ERR1743333	MGYA00087156	https://www.ebi.ac.uk/metagenomics/api/v1/analyses/MGYA00087156/file/ERR1743333_MERGED_FASTQ_otu.tsv	Root-Environmental-Aquatic-Freshwater-Lake
ERR1897440	MGYA00351385	https://www.ebi.ac.uk/metagenomics/api/v1/analyses/MGYA00351385/file/ERR1897440_FASTQ_SSU_OTU.tsv	Root-Environmental-Aquatic-Freshwater-Lake
ERR1897021	MGYA00350966	https://www.ebi.ac.uk/metagenomics/api/v1/analyses/MGYA00350966/file/ERR1897021_FASTQ_SSU_OTU.tsv	Root-Environmental-Aquatic-Freshwater-Lake
ERR1896661	MGYA00350606	https://www.ebi.ac.uk/metagenomics/api/v1/analyses/MGYA00350606/file/ERR1896661_FASTQ_SSU_OTU.tsv	Root-Environmental-Aquatic-Freshwater-Lake
ERR1583114	MGYA00353351	https://www.ebi.ac.uk/metagenomics/api/v1/analyses/MGYA00353351/file/ERR1583114_FASTQ_SSU_OTU.tsv	Root-Environmental-Aquatic-Freshwater-Lake
ERR1314393	MGYA00095200	https://www.ebi.ac.uk/metagenomics/api/v1/analyses/MGYA00095200/file/ERR1314393_MERGED_FASTQ_otu.tsv	Root-Environmental-Aquatic-Freshwater-Lake
ERR1582754	MGYA00352991	https://www.ebi.ac.uk/metagenomics/api/v1/analyses/MGYA00352991/file/ERR1582754_FASTQ_SSU_OTU.tsv	Root-Environmental-Aquatic-Freshwater-Lake
ERR1547054	MGYA00320765	https://www.ebi.ac.uk/metagenomics/api/v1/analyses/MGYA00320765/file/ERR1547054_FASTQ_SSU_OTU.tsv	Root-Environmental-Aquatic-Freshwater-Lake
ERR1897518	MGYA00351463	https://www.ebi.ac.uk/metagenomics/api/v1/analyses/MGYA00351463/file/ERR1897518_FASTQ_SSU_OTU.tsv	Root-Environmental-Aquatic-Freshwater-Lake

ERR1582492	MGYA00352729	https://www.ebi.ac.uk/metagenomics/api/v1/analyses/MGYA00352729/file/ERR1582492_FASTQ_SSU_OTU.tsv	Root-Environmental-Aquatic-Freshwater-Lake
ERR1582335	MGYA00352572	https://www.ebi.ac.uk/metagenomics/api/v1/analyses/MGYA00352572/file/ERR1582335_FASTQ_SSU_OTU.tsv	Root-Environmental-Aquatic-Freshwater-Lake
ERR1897179	MGYA00351124	https://www.ebi.ac.uk/metagenomics/api/v1/analyses/MGYA00351124/file/ERR1897179_FASTQ_SSU_OTU.tsv	Root-Environmental-Aquatic-Freshwater-Lake
ERR1896739	MGYA00350684	https://www.ebi.ac.uk/metagenomics/api/v1/analyses/MGYA00350684/file/ERR1896739_FASTQ_SSU_OTU.tsv	Root-Environmental-Aquatic-Freshwater-Lake
ERR1583203	MGYA00353440	https://www.ebi.ac.uk/metagenomics/api/v1/analyses/MGYA00353440/file/ERR1583203_FASTQ_SSU_OTU.tsv	Root-Environmental-Aquatic-Freshwater-Lake
ERR1582443	MGYA00352680	https://www.ebi.ac.uk/metagenomics/api/v1/analyses/MGYA00352680/file/ERR1582443_FASTQ_SSU_OTU.tsv	Root-Environmental-Aquatic-Freshwater-Lake
ERR1582831	MGYA00353068	https://www.ebi.ac.uk/metagenomics/api/v1/analyses/MGYA00353068/file/ERR1582831_FASTQ_SSU_OTU.tsv	Root-Environmental-Aquatic-Freshwater-Lake
ERR1547085	MGYA00320796	https://www.ebi.ac.uk/metagenomics/api/v1/analyses/MGYA00320796/file/ERR1547085_FASTQ_SSU_OTU.tsv	Root-Environmental-Aquatic-Freshwater-Lake
ERR1582022	MGYA00352259	https://www.ebi.ac.uk/metagenomics/api/v1/analyses/MGYA00352259/file/ERR1582022_FASTQ_SSU_OTU.tsv	Root-Environmental-Aquatic-Freshwater-Lake
ERR1582785	MGYA00353022	https://www.ebi.ac.uk/metagenomics/api/v1/analyses/MGYA00353022/file/ERR1582785_FASTQ_SSU_OTU.tsv	Root-Environmental-Aquatic-Freshwater-Lake
ERR1314342	MGYA00095188	https://www.ebi.ac.uk/metagenomics/api/v1/analyses/MGYA00095188/file/ERR1314342_MERGED_FASTQ_otu.tsv	Root-Environmental-Aquatic-Freshwater-Lake
ERR1897750	MGYA00351695	https://www.ebi.ac.uk/metagenomics/api/v1/analyses/MGYA00351695/file/ERR1897750_FASTQ_SSU_OTU.tsv	Root-Environmental-Aquatic-Freshwater-Lake

SRR2443014	MGYA00359018	https://www.ebi.ac.uk/metagenomics/api/v1/analyses/MGYA00359018/file/SRR2443014_MERGED_FASTQ_SSU_OTU.tsv	Root-Environmental-Aquatic-Freshwater-Lake
ERR1896903	MGYA00350848	https://www.ebi.ac.uk/metagenomics/api/v1/analyses/MGYA00350848/file/ERR1896903_FASTQ_SSU_OTU.tsv	Root-Environmental-Aquatic-Freshwater-Lake
ERR1897331	MGYA00351276	https://www.ebi.ac.uk/metagenomics/api/v1/analyses/MGYA00351276/file/ERR1897331_FASTQ_SSU_OTU.tsv	Root-Environmental-Aquatic-Freshwater-Lake
ERR589344	MGYA00103450	https://www.ebi.ac.uk/metagenomics/api/v1/analyses/MGYA00103450/file/ERR589344_FASTQ_otu.tsv	Root-Environmental-Aquatic-Freshwater-Lake
ERR1897496	MGYA00351441	https://www.ebi.ac.uk/metagenomics/api/v1/analyses/MGYA00351441/file/ERR1897496_FASTQ_SSU_OTU.tsv	Root-Environmental-Aquatic-Freshwater-Lake
ERR1897447	MGYA00351392	https://www.ebi.ac.uk/metagenomics/api/v1/analyses/MGYA00351392/file/ERR1897447_FASTQ_SSU_OTU.tsv	Root-Environmental-Aquatic-Freshwater-Lake
ERR1743334	MGYA00087178	https://www.ebi.ac.uk/metagenomics/api/v1/analyses/MGYA00087178/file/ERR1743334_MERGED_FASTQ_otu.tsv	Root-Environmental-Aquatic-Freshwater-Lake
ERR1896666	MGYA00350611	https://www.ebi.ac.uk/metagenomics/api/v1/analyses/MGYA00350611/file/ERR1896666_FASTQ_SSU_OTU.tsv	Root-Environmental-Aquatic-Freshwater-Lake
ERR1897026	MGYA00350971	https://www.ebi.ac.uk/metagenomics/api/v1/analyses/MGYA00350971/file/ERR1897026_FASTQ_SSU_OTU.tsv	Root-Environmental-Aquatic-Freshwater-Lake
ERR430919	MGYA00103259	https://www.ebi.ac.uk/metagenomics/api/v1/analyses/MGYA00103259/file/ERR430919_FASTQ_otu.tsv	Root-Environmental-Aquatic-Freshwater-Lake
ERR1314394	MGYA00095155	https://www.ebi.ac.uk/metagenomics/api/v1/analyses/MGYA00095155/file/ERR1314394_MERGED_FASTQ_otu.tsv	Root-Environmental-Aquatic-Freshwater-Lake
ERR1582753	MGYA00352990	https://www.ebi.ac.uk/metagenomics/api/v1/analyses/MGYA00352990/file/ERR1582753_FASTQ_SSU_OTU.tsv	Root-Environmental-Aquatic-Freshwater-Lake

ERR1583113	MGYA00353350	https://www.ebi.ac.uk/metagenomics/api/v1/analyses/MGYA00353350/file/ERR1583113_FASTQ_SSU_OTU.tsv	Root-Environmental-Aquatic-Freshwater-Lake
ERR1547053	MGYA00320764	https://www.ebi.ac.uk/metagenomics/api/v1/analyses/MGYA00320764/file/ERR1547053_FASTQ_SSU_OTU.tsv	Root-Environmental-Aquatic-Freshwater-Lake
ERR1582332	MGYA00352569	https://www.ebi.ac.uk/metagenomics/api/v1/analyses/MGYA00352569/file/ERR1582332_FASTQ_SSU_OTU.tsv	Root-Environmental-Aquatic-Freshwater-Lake
ERR1582495	MGYA00352732	https://www.ebi.ac.uk/metagenomics/api/v1/analyses/MGYA00352732/file/ERR1582495_FASTQ_SSU_OTU.tsv	Root-Environmental-Aquatic-Freshwater-Lake
ERR1897608	MGYA00351553	https://www.ebi.ac.uk/metagenomics/api/v1/analyses/MGYA00351553/file/ERR1897608_FASTQ_SSU_OTU.tsv	Root-Environmental-Aquatic-Freshwater-Lake
ERR1582836	MGYA00353073	https://www.ebi.ac.uk/metagenomics/api/v1/analyses/MGYA00353073/file/ERR1582836_FASTQ_SSU_OTU.tsv	Root-Environmental-Aquatic-Freshwater-Lake
ERR1582444	MGYA00352681	https://www.ebi.ac.uk/metagenomics/api/v1/analyses/MGYA00352681/file/ERR1582444_FASTQ_SSU_OTU.tsv	Root-Environmental-Aquatic-Freshwater-Lake
ERR1583204	MGYA00353441	https://www.ebi.ac.uk/metagenomics/api/v1/analyses/MGYA00353441/file/ERR1583204_FASTQ_SSU_OTU.tsv	Root-Environmental-Aquatic-Freshwater-Lake
ERR1547082	MGYA00320793	https://www.ebi.ac.uk/metagenomics/api/v1/analyses/MGYA00320793/file/ERR1547082_FASTQ_SSU_OTU.tsv	Root-Environmental-Aquatic-Freshwater-Lake
ERR1897269	MGYA00351214	https://www.ebi.ac.uk/metagenomics/api/v1/analyses/MGYA00351214/file/ERR1897269_FASTQ_SSU_OTU.tsv	Root-Environmental-Aquatic-Freshwater-Lake
ERR1582782	MGYA00353019	https://www.ebi.ac.uk/metagenomics/api/v1/analyses/MGYA00353019/file/ERR1582782_FASTQ_SSU_OTU.tsv	Root-Environmental-Aquatic-Freshwater-Lake
ERR1314345	MGYA00095214	https://www.ebi.ac.uk/metagenomics/api/v1/analyses/MGYA00095214/file/ERR1314345_MERGED_FASTQ_otu.tsv	Root-Environmental-Aquatic-Freshwater-Lake

ERR1582025	MGYA00352262	https://www.ebi.ac.uk/metagenomics/api/v1/analyses/MGYA00352262/file/ERR1582025_FASTQ_SSU_OTU.tsv	Root-Environmental-Aquatic-Freshwater-Lake
ERR1582560	MGYA00352797	https://www.ebi.ac.uk/metagenomics/api/v1/analyses/MGYA00352797/file/ERR1582560_FASTQ_SSU_OTU.tsv	Root-Environmental-Aquatic-Freshwater-Lake
ERR1582912	MGYA00353149	https://www.ebi.ac.uk/metagenomics/api/v1/analyses/MGYA00353149/file/ERR1582912_FASTQ_SSU_OTU.tsv	Root-Environmental-Aquatic-Freshwater-Lake
ERR1583320	MGYA00353557	https://www.ebi.ac.uk/metagenomics/api/v1/analyses/MGYA00353557/file/ERR1583320_FASTQ_SSU_OTU.tsv	Root-Environmental-Aquatic-Freshwater-Lake
ERR1583487	MGYA00353724	https://www.ebi.ac.uk/metagenomics/api/v1/analyses/MGYA00353724/file/ERR1583487_FASTQ_SSU_OTU.tsv	Root-Environmental-Aquatic-Freshwater-Lake
ERR589338	MGYA00103461	https://www.ebi.ac.uk/metagenomics/api/v1/analyses/MGYA00103461/file/ERR589338_FASTQ_otu.tsv	Root-Environmental-Aquatic-Freshwater-Lake
ERR1582101	MGYA00352338	https://www.ebi.ac.uk/metagenomics/api/v1/analyses/MGYA00352338/file/ERR1582101_FASTQ_SSU_OTU.tsv	Root-Environmental-Aquatic-Freshwater-Lake
ERR1582677	MGYA00352914	https://www.ebi.ac.uk/metagenomics/api/v1/analyses/MGYA00352914/file/ERR1582677_FASTQ_SSU_OTU.tsv	Root-Environmental-Aquatic-Freshwater-Lake
ERR1583037	MGYA00353274	https://www.ebi.ac.uk/metagenomics/api/v1/analyses/MGYA00353274/file/ERR1583037_FASTQ_SSU_OTU.tsv	Root-Environmental-Aquatic-Freshwater-Lake
ERR1743348	MGYA00087203	https://www.ebi.ac.uk/metagenomics/api/v1/analyses/MGYA00087203/file/ERR1743348_MERGED_FASTQ_otu.tsv	Root-Environmental-Aquatic-Freshwater-Lake
ERR1583456	MGYA00353693	https://www.ebi.ac.uk/metagenomics/api/v1/analyses/MGYA00353693/file/ERR1583456_FASTQ_SSU_OTU.tsv	Root-Environmental-Aquatic-Freshwater-Lake
ERR1582216	MGYA00352453	https://www.ebi.ac.uk/metagenomics/api/v1/analyses/MGYA00352453/file/ERR1582216_FASTQ_SSU_OTU.tsv	Root-Environmental-Aquatic-Freshwater-Lake

ERR1897563	MGYA00351508	https://www.ebi.ac.uk/metagenomics/api/v1/analyses/MGYA00351508/file/ERR1897563_FASTQ_SSU_OTU.tsv	Root-Environmental-Aquatic-Freshwater-Lake
SRR7081378	MGYA00423199	https://www.ebi.ac.uk/metagenomics/api/v1/analyses/MGYA00423199/file/SRR7081378_MERGED_FASTQ_SSU_OTU.tsv	Root-Environmental-Aquatic-Freshwater-Lake
ERR1582088	MGYA00352325	https://www.ebi.ac.uk/metagenomics/api/v1/analyses/MGYA00352325/file/ERR1582088_FASTQ_SSU_OTU.tsv	Root-Environmental-Aquatic-Freshwater-Lake
ERR1896742	MGYA00350687	https://www.ebi.ac.uk/metagenomics/api/v1/analyses/MGYA00350687/file/ERR1896742_FASTQ_SSU_OTU.tsv	Root-Environmental-Aquatic-Freshwater-Lake
ERR1535989	MGYA00320280	https://www.ebi.ac.uk/metagenomics/api/v1/analyses/MGYA00320280/file/ERR1535989_FASTQ_SSU_OTU.tsv	Root-Environmental-Aquatic-Freshwater-Lake
ERR1897102	MGYA00351047	https://www.ebi.ac.uk/metagenomics/api/v1/analyses/MGYA00351047/file/ERR1897102_FASTQ_SSU_OTU.tsv	Root-Environmental-Aquatic-Freshwater-Lake
ERR1582438	MGYA00352675	https://www.ebi.ac.uk/metagenomics/api/v1/analyses/MGYA00352675/file/ERR1582438_FASTQ_SSU_OTU.tsv	Root-Environmental-Aquatic-Freshwater-Lake
ERR1583278	MGYA00353515	https://www.ebi.ac.uk/metagenomics/api/v1/analyses/MGYA00353515/file/ERR1583278_FASTQ_SSU_OTU.tsv	Root-Environmental-Aquatic-Freshwater-Lake
ERR1897674	MGYA00351619	https://www.ebi.ac.uk/metagenomics/api/v1/analyses/MGYA00351619/file/ERR1897674_FASTQ_SSU_OTU.tsv	Root-Environmental-Aquatic-Freshwater-Lake
ERR1535958	MGYA00320249	https://www.ebi.ac.uk/metagenomics/api/v1/analyses/MGYA00320249/file/ERR1535958_FASTQ_SSU_OTU.tsv	Root-Environmental-Aquatic-Freshwater-Lake
ERR1896793	MGYA00350738	https://www.ebi.ac.uk/metagenomics/api/v1/analyses/MGYA00350738/file/ERR1896793_FASTQ_SSU_OTU.tsv	Root-Environmental-Aquatic-Freshwater-Lake
ERR1582059	MGYA00352296	https://www.ebi.ac.uk/metagenomics/api/v1/analyses/MGYA00352296/file/ERR1582059_FASTQ_SSU_OTU.tsv	Root-Environmental-Aquatic-Freshwater-Lake

ERR1314339	MGYA00095207	https://www.ebi.ac.uk/metagenomics/api/v1/analyses/MGYA00095207/file/ERR1314339_MERGED_FASTQ_otu.tsv	Root-Environmental-Aquatic-Freshwater-Lake
ERR1896827	MGYA00350772	https://www.ebi.ac.uk/metagenomics/api/v1/analyses/MGYA00350772/file/ERR1896827_FASTQ_SSU_OTU.tsv	Root-Environmental-Aquatic-Freshwater-Lake
ERR1897215	MGYA00351160	https://www.ebi.ac.uk/metagenomics/api/v1/analyses/MGYA00351160/file/ERR1897215_FASTQ_SSU_OTU.tsv	Root-Environmental-Aquatic-Freshwater-Lake
ERR1429232	MGYA00107677	https://www.ebi.ac.uk/metagenomics/api/v1/analyses/MGYA00107677/file/ERR1429232_FASTQ_otu.tsv	Root-Environmental-Aquatic-Freshwater-Lake
ERR1582317	MGYA00352554	https://www.ebi.ac.uk/metagenomics/api/v1/analyses/MGYA00352554/file/ERR1582317_FASTQ_SSU_OTU.tsv	Root-Environmental-Aquatic-Freshwater-Lake
ERR1582776	MGYA00353013	https://www.ebi.ac.uk/metagenomics/api/v1/analyses/MGYA00353013/file/ERR1582776_FASTQ_SSU_OTU.tsv	Root-Environmental-Aquatic-Freshwater-Lake
ERR1583136	MGYA00353373	https://www.ebi.ac.uk/metagenomics/api/v1/analyses/MGYA00353373/file/ERR1583136_FASTQ_SSU_OTU.tsv	Root-Environmental-Aquatic-Freshwater-Lake
SRR7081321	MGYA00424206	https://www.ebi.ac.uk/metagenomics/api/v1/analyses/MGYA00424206/file/SRR7081321_MERGED_FASTQ_SSU_OTU.tsv	Root-Environmental-Aquatic-Freshwater-Lake
ERR1547076	MGYA00320787	https://www.ebi.ac.uk/metagenomics/api/v1/analyses/MGYA00320787/file/ERR1547076_FASTQ_SSU_OTU.tsv	Root-Environmental-Aquatic-Freshwater-Lake
ERR1743298	MGYA00087147	https://www.ebi.ac.uk/metagenomics/api/v1/analyses/MGYA00087147/file/ERR1743298_MERGED_FASTQ_otu.tsv	Root-Environmental-Aquatic-Freshwater-Lake
ERR1314360	MGYA00095215	https://www.ebi.ac.uk/metagenomics/api/v1/analyses/MGYA00095215/file/ERR1314360_MERGED_FASTQ_otu.tsv	Root-Environmental-Aquatic-Freshwater-Lake
ERR1582461	MGYA00352698	https://www.ebi.ac.uk/metagenomics/api/v1/analyses/MGYA00352698/file/ERR1582461_FASTQ_SSU_OTU.tsv	Root-Environmental-Aquatic-Freshwater-Lake

ERR1582813	MGYA00353050	https://www.ebi.ac.uk/metagenomics/api/v1/analyses/MGYA00353050/file/ERR1582813_FASTQ_SSU_OTU.tsv	Root-Environmental-Aquatic-Freshwater-Lake
ERR1583221	MGYA00353458	https://www.ebi.ac.uk/metagenomics/api/v1/analyses/MGYA00353458/file/ERR1583221_FASTQ_SSU_OTU.tsv	Root-Environmental-Aquatic-Freshwater-Lake
ERR1582158	MGYA00352395	https://www.ebi.ac.uk/metagenomics/api/v1/analyses/MGYA00352395/file/ERR1582158_FASTQ_SSU_OTU.tsv	Root-Environmental-Aquatic-Freshwater-Lake
ERR1896926	MGYA00350871	https://www.ebi.ac.uk/metagenomics/api/v1/analyses/MGYA00350871/file/ERR1896926_FASTQ_SSU_OTU.tsv	Root-Environmental-Aquatic-Freshwater-Lake
ERR1897314	MGYA00351259	https://www.ebi.ac.uk/metagenomics/api/v1/analyses/MGYA00351259/file/ERR1897314_FASTQ_SSU_OTU.tsv	Root-Environmental-Aquatic-Freshwater-Lake
ERR1990482	MGYA00128216	https://www.ebi.ac.uk/metagenomics/api/v1/analyses/MGYA00128216/file/ERR1990482_MERGED_FASTQ_otu.tsv	Root-Environmental-Aquatic-Freshwater-Lake
ERR1582539	MGYA00352776	https://www.ebi.ac.uk/metagenomics/api/v1/analyses/MGYA00352776/file/ERR1582539_FASTQ_SSU_OTU.tsv	Root-Environmental-Aquatic-Freshwater-Lake
ERR1583379	MGYA00353616	https://www.ebi.ac.uk/metagenomics/api/v1/analyses/MGYA00353616/file/ERR1583379_FASTQ_SSU_OTU.tsv	Root-Environmental-Aquatic-Freshwater-Lake
ERR1897775	MGYA00351720	https://www.ebi.ac.uk/metagenomics/api/v1/analyses/MGYA00351720/file/ERR1897775_FASTQ_SSU_OTU.tsv	Root-Environmental-Aquatic-Freshwater-Lake
SRR2443031	MGYA00359035	https://www.ebi.ac.uk/metagenomics/api/v1/analyses/MGYA00359035/file/SRR2443031_MERGED_FASTQ_SSU_OTU.tsv	Root-Environmental-Aquatic-Freshwater-Lake
ERR1896692	MGYA00350637	https://www.ebi.ac.uk/metagenomics/api/v1/analyses/MGYA00350637/file/ERR1896692_FASTQ_SSU_OTU.tsv	Root-Environmental-Aquatic-Freshwater-Lake
ERR1896643	MGYA00350588	https://www.ebi.ac.uk/metagenomics/api/v1/analyses/MGYA00350588/file/ERR1896643_FASTQ_SSU_OTU.tsv	Root-Environmental-Aquatic-Freshwater-Lake

ERR1897003	MGYA00350948	https://www.ebi.ac.uk/metagenomics/api/v1/analyses/MGYA00350948/file/ERR1897003_FASTQ_SSU_OTU.tsv	Root-Environmental-Aquatic-Freshwater-Lake
ERR1897462	MGYA00351407	https://www.ebi.ac.uk/metagenomics/api/v1/analyses/MGYA00351407/file/ERR1897462_FASTQ_SSU_OTU.tsv	Root-Environmental-Aquatic-Freshwater-Lake
ERR1743311	MGYA00087117	https://www.ebi.ac.uk/metagenomics/api/v1/analyses/MGYA00087117/file/ERR1743311_MERGED_FASTQ_otu.tsv	Root-Environmental-Aquatic-Freshwater-Lake
ERR1582189	MGYA00352426	https://www.ebi.ac.uk/metagenomics/api/v1/analyses/MGYA00352426/file/ERR1582189_FASTQ_SSU_OTU.tsv	Root-Environmental-Aquatic-Freshwater-Lake
ERR1896767	MGYA00350712	https://www.ebi.ac.uk/metagenomics/api/v1/analyses/MGYA00350712/file/ERR1896767_FASTQ_SSU_OTU.tsv	Root-Environmental-Aquatic-Freshwater-Lake
ERR1897680	MGYA00351625	https://www.ebi.ac.uk/metagenomics/api/v1/analyses/MGYA00351625/file/ERR1897680_FASTQ_SSU_OTU.tsv	Root-Environmental-Aquatic-Freshwater-Lake
ERR1897127	MGYA00351072	https://www.ebi.ac.uk/metagenomics/api/v1/analyses/MGYA00351072/file/ERR1897127_FASTQ_SSU_OTU.tsv	Root-Environmental-Aquatic-Freshwater-Lake
ERR658192	MGYA00103467	https://www.ebi.ac.uk/metagenomics/api/v1/analyses/MGYA00103467/file/ERR658192_FASTQ_otu.tsv	Root-Environmental-Aquatic-Freshwater-Lake
ERR1897546	MGYA00351491	https://www.ebi.ac.uk/metagenomics/api/v1/analyses/MGYA00351491/file/ERR1897546_FASTQ_SSU_OTU.tsv	Root-Environmental-Aquatic-Freshwater-Lake
ERR589294	MGYA00103452	https://www.ebi.ac.uk/metagenomics/api/v1/analyses/MGYA00103452/file/ERR589294_FASTQ_otu.tsv	Root-Environmental-Aquatic-Freshwater-Lake
ERR1896802	MGYA00350747	https://www.ebi.ac.uk/metagenomics/api/v1/analyses/MGYA00350747/file/ERR1896802_FASTQ_SSU_OTU.tsv	Root-Environmental-Aquatic-Freshwater-Lake
ERR1897230	MGYA00351175	https://www.ebi.ac.uk/metagenomics/api/v1/analyses/MGYA00351175/file/ERR1897230_FASTQ_SSU_OTU.tsv	Root-Environmental-Aquatic-Freshwater-Lake

ERR1897597	MGYA00351542	https://www.ebi.ac.uk/metagenomics/api/v1/analyses/MGYA00351542/file/ERR1897597_FASTQ_SSU_OTU.tsv	Root-Environmental-Aquatic-Freshwater-Lake
ERR1897651	MGYA00351596	https://www.ebi.ac.uk/metagenomics/api/v1/analyses/MGYA00351596/file/ERR1897651_FASTQ_SSU_OTU.tsv	Root-Environmental-Aquatic-Freshwater-Lake
ERR1897368	MGYA00351313	https://www.ebi.ac.uk/metagenomics/api/v1/analyses/MGYA00351313/file/ERR1897368_FASTQ_SSU_OTU.tsv	Root-Environmental-Aquatic-Freshwater-Lake
ERR1582683	MGYA00352920	https://www.ebi.ac.uk/metagenomics/api/v1/analyses/MGYA00352920/file/ERR1582683_FASTQ_SSU_OTU.tsv	Root-Environmental-Aquatic-Freshwater-Lake
ERR1582124	MGYA00352361	https://www.ebi.ac.uk/metagenomics/api/v1/analyses/MGYA00352361/file/ERR1582124_FASTQ_SSU_OTU.tsv	Root-Environmental-Aquatic-Freshwater-Lake
ERR1897709	MGYA00351654	https://www.ebi.ac.uk/metagenomics/api/v1/analyses/MGYA00351654/file/ERR1897709_FASTQ_SSU_OTU.tsv	Root-Environmental-Aquatic-Freshwater-Lake
ERR1582937	MGYA00353174	https://www.ebi.ac.uk/metagenomics/api/v1/analyses/MGYA00353174/file/ERR1582937_FASTQ_SSU_OTU.tsv	Root-Environmental-Aquatic-Freshwater-Lake
ERR1582545	MGYA00352782	https://www.ebi.ac.uk/metagenomics/api/v1/analyses/MGYA00352782/file/ERR1582545_FASTQ_SSU_OTU.tsv	Root-Environmental-Aquatic-Freshwater-Lake
ERR1583305	MGYA00353542	https://www.ebi.ac.uk/metagenomics/api/v1/analyses/MGYA00353542/file/ERR1583305_FASTQ_SSU_OTU.tsv	Root-Environmental-Aquatic-Freshwater-Lake
ERR1583473	MGYA00353710	https://www.ebi.ac.uk/metagenomics/api/v1/analyses/MGYA00353710/file/ERR1583473_FASTQ_SSU_OTU.tsv	Root-Environmental-Aquatic-Freshwater-Lake
ERR1582233	MGYA00352470	https://www.ebi.ac.uk/metagenomics/api/v1/analyses/MGYA00352470/file/ERR1582233_FASTQ_SSU_OTU.tsv	Root-Environmental-Aquatic-Freshwater-Lake
ERR1582594	MGYA00352831	https://www.ebi.ac.uk/metagenomics/api/v1/analyses/MGYA00352831/file/ERR1582594_FASTQ_SSU_OTU.tsv	Root-Environmental-Aquatic-Freshwater-Lake

ERR1582652	MGYA00352889	https://www.ebi.ac.uk/metagenomics/api/v1/analyses/MGYA00352889/file/ERR1582652_FASTQ_SSU_OTU.tsv	Root-Environmental-Aquatic-Freshwater-Lake
ERR1583012	MGYA00353249	https://www.ebi.ac.uk/metagenomics/api/v1/analyses/MGYA00353249/file/ERR1583012_FASTQ_SSU_OTU.tsv	Root-Environmental-Aquatic-Freshwater-Lake
SRR2442968	MGYA00358972	https://www.ebi.ac.uk/metagenomics/api/v1/analyses/MGYA00358972/file/SRR2442968_MERGED_FASTQ_SSU_OTU.tsv	Root-Environmental-Aquatic-Freshwater-Lake
ERR1897120	MGYA00351065	https://www.ebi.ac.uk/metagenomics/api/v1/analyses/MGYA00351065/file/ERR1897120_FASTQ_SSU_OTU.tsv	Root-Environmental-Aquatic-Freshwater-Lake
ERR1897687	MGYA00351632	https://www.ebi.ac.uk/metagenomics/api/v1/analyses/MGYA00351632/file/ERR1897687_FASTQ_SSU_OTU.tsv	Root-Environmental-Aquatic-Freshwater-Lake
ERR1896760	MGYA00350705	https://www.ebi.ac.uk/metagenomics/api/v1/analyses/MGYA00350705/file/ERR1896760_FASTQ_SSU_OTU.tsv	Root-Environmental-Aquatic-Freshwater-Lake
ERR658195	MGYA00103482	https://www.ebi.ac.uk/metagenomics/api/v1/analyses/MGYA00103482/file/ERR658195_FASTQ_otu.tsv	Root-Environmental-Aquatic-Freshwater-Lake
ERR1429249	MGYA00107642	https://www.ebi.ac.uk/metagenomics/api/v1/analyses/MGYA00107642/file/ERR1429249_FASTQ_otu.tsv	Root-Environmental-Aquatic-Freshwater-Lake
ERR589293	MGYA00103462	https://www.ebi.ac.uk/metagenomics/api/v1/analyses/MGYA00103462/file/ERR589293_FASTQ_otu.tsv	Root-Environmental-Aquatic-Freshwater-Lake
ERR1897541	MGYA00351486	https://www.ebi.ac.uk/metagenomics/api/v1/analyses/MGYA00351486/file/ERR1897541_FASTQ_SSU_OTU.tsv	Root-Environmental-Aquatic-Freshwater-Lake
ERR1437516	MGYA00107951	https://www.ebi.ac.uk/metagenomics/api/v1/analyses/MGYA00107951/file/ERR1437516_FASTQ_otu.tsv	Root-Environmental-Aquatic-Freshwater-Lake
ERR1897590	MGYA00351535	https://www.ebi.ac.uk/metagenomics/api/v1/analyses/MGYA00351535/file/ERR1897590_FASTQ_SSU_OTU.tsv	Root-Environmental-Aquatic-Freshwater-Lake

ERR1897237	MGYA00351182	https://www.ebi.ac.uk/metagenomics/api/v1/analyses/MGYA00351182/file/ERR1897237_FASTQ_SSU_OTU.tsv	Root-Environmental-Aquatic-Freshwater-Lake
ERR1896805	MGYA00350750	https://www.ebi.ac.uk/metagenomics/api/v1/analyses/MGYA00350750/file/ERR1896805_FASTQ_SSU_OTU.tsv	Root-Environmental-Aquatic-Freshwater-Lake
ERR1582868	MGYA00353105	https://www.ebi.ac.uk/metagenomics/api/v1/analyses/MGYA00353105/file/ERR1582868_FASTQ_SSU_OTU.tsv	Root-Environmental-Aquatic-Freshwater-Lake
ERR1897656	MGYA00351601	https://www.ebi.ac.uk/metagenomics/api/v1/analyses/MGYA00351601/file/ERR1897656_FASTQ_SSU_OTU.tsv	Root-Environmental-Aquatic-Freshwater-Lake
ERR1582123	MGYA00352360	https://www.ebi.ac.uk/metagenomics/api/v1/analyses/MGYA00352360/file/ERR1582123_FASTQ_SSU_OTU.tsv	Root-Environmental-Aquatic-Freshwater-Lake
ERR1582684	MGYA00352921	https://www.ebi.ac.uk/metagenomics/api/v1/analyses/MGYA00352921/file/ERR1582684_FASTQ_SSU_OTU.tsv	Root-Environmental-Aquatic-Freshwater-Lake
ERR1583302	MGYA00353539	https://www.ebi.ac.uk/metagenomics/api/v1/analyses/MGYA00353539/file/ERR1583302_FASTQ_SSU_OTU.tsv	Root-Environmental-Aquatic-Freshwater-Lake
ERR1582542	MGYA00352779	https://www.ebi.ac.uk/metagenomics/api/v1/analyses/MGYA00352779/file/ERR1582542_FASTQ_SSU_OTU.tsv	Root-Environmental-Aquatic-Freshwater-Lake
ERR1582930	MGYA00353167	https://www.ebi.ac.uk/metagenomics/api/v1/analyses/MGYA00353167/file/ERR1582930_FASTQ_SSU_OTU.tsv	Root-Environmental-Aquatic-Freshwater-Lake
ERR1582593	MGYA00352830	https://www.ebi.ac.uk/metagenomics/api/v1/analyses/MGYA00352830/file/ERR1582593_FASTQ_SSU_OTU.tsv	Root-Environmental-Aquatic-Freshwater-Lake
ERR1582234	MGYA00352471	https://www.ebi.ac.uk/metagenomics/api/v1/analyses/MGYA00352471/file/ERR1582234_FASTQ_SSU_OTU.tsv	Root-Environmental-Aquatic-Freshwater-Lake
ERR1583474	MGYA00353711	https://www.ebi.ac.uk/metagenomics/api/v1/analyses/MGYA00353711/file/ERR1583474_FASTQ_SSU_OTU.tsv	Root-Environmental-Aquatic-Freshwater-Lake

ERR1897078	MGYA00351023	https://www.ebi.ac.uk/metagenomics/api/v1/analyses/MGYA00351023/file/ERR1897078_FASTQ_SSU_OTU.tsv	Root-Environmental-Aquatic-Freshwater-Lake
ERR1896638	MGYA00350583	https://www.ebi.ac.uk/metagenomics/api/v1/analyses/MGYA00350583/file/ERR1896638_FASTQ_SSU_OTU.tsv	Root-Environmental-Aquatic-Freshwater-Lake
ERR1583015	MGYA00353252	https://www.ebi.ac.uk/metagenomics/api/v1/analyses/MGYA00353252/file/ERR1583015_FASTQ_SSU_OTU.tsv	Root-Environmental-Aquatic-Freshwater-Lake
ERR1582655	MGYA00352892	https://www.ebi.ac.uk/metagenomics/api/v1/analyses/MGYA00352892/file/ERR1582655_FASTQ_SSU_OTU.tsv	Root-Environmental-Aquatic-Freshwater-Lake
ERR1897419	MGYA00351364	https://www.ebi.ac.uk/metagenomics/api/v1/analyses/MGYA00351364/file/ERR1897419_FASTQ_SSU_OTU.tsv	Root-Environmental-Aquatic-Freshwater-Lake
ERR1582310	MGYA00352547	https://www.ebi.ac.uk/metagenomics/api/v1/analyses/MGYA00352547/file/ERR1582310_FASTQ_SSU_OTU.tsv	Root-Environmental-Aquatic-Freshwater-Lake
ERR1429235	MGYA00107655	https://www.ebi.ac.uk/metagenomics/api/v1/analyses/MGYA00107655/file/ERR1429235_FASTQ_otu.tsv	Root-Environmental-Aquatic-Freshwater-Lake
ERR1583131	MGYA00353368	https://www.ebi.ac.uk/metagenomics/api/v1/analyses/MGYA00353368/file/ERR1583131_FASTQ_SSU_OTU.tsv	Root-Environmental-Aquatic-Freshwater-Lake
ERR1582771	MGYA00353008	https://www.ebi.ac.uk/metagenomics/api/v1/analyses/MGYA00353008/file/ERR1582771_FASTQ_SSU_OTU.tsv	Root-Environmental-Aquatic-Freshwater-Lake
ERR1547071	MGYA00320782	https://www.ebi.ac.uk/metagenomics/api/v1/analyses/MGYA00320782/file/ERR1547071_FASTQ_SSU_OTU.tsv	Root-Environmental-Aquatic-Freshwater-Lake
SRR7081326	MGYA00424811	https://www.ebi.ac.uk/metagenomics/api/v1/analyses/MGYA00424811/file/SRR7081326_MERGED_FASTQ_SSU_OTU.tsv	Root-Environmental-Aquatic-Freshwater-Lake
ERR1896879	MGYA00350824	https://www.ebi.ac.uk/metagenomics/api/v1/analyses/MGYA00350824/file/ERR1896879_FASTQ_SSU_OTU.tsv	Root-Environmental-Aquatic-Freshwater-Lake

ERR1314367	MGYA00095189	https://www.ebi.ac.uk/metagenomics/api/v1/analyses/MGYA00095189/file/ERR1314367_MERGED_FASTQ_otu.tsv	Root-Environmental-Aquatic-Freshwater-Lake
ERR389726	MGYA00103371	https://www.ebi.ac.uk/metagenomics/api/v1/analyses/MGYA00103371/file/ERR389726_FASTQ_otu.tsv	Root-Environmental-Aquatic-Freshwater-Lake
SRR7081031	MGYA00427364	https://www.ebi.ac.uk/metagenomics/api/v1/analyses/MGYA00427364/file/SRR7081031_MERGED_FASTQ_SSU_OTU.tsv	Root-Environmental-Aquatic-Freshwater-Lake
ERR1583226	MGYA00353463	https://www.ebi.ac.uk/metagenomics/api/v1/analyses/MGYA00353463/file/ERR1583226_FASTQ_SSU_OTU.tsv	Root-Environmental-Aquatic-Freshwater-Lake
ERR1582814	MGYA00353051	https://www.ebi.ac.uk/metagenomics/api/v1/analyses/MGYA00353051/file/ERR1582814_FASTQ_SSU_OTU.tsv	Root-Environmental-Aquatic-Freshwater-Lake
ERR1582466	MGYA00352703	https://www.ebi.ac.uk/metagenomics/api/v1/analyses/MGYA00352703/file/ERR1582466_FASTQ_SSU_OTU.tsv	Root-Environmental-Aquatic-Freshwater-Lake
ERR1897313	MGYA00351258	https://www.ebi.ac.uk/metagenomics/api/v1/analyses/MGYA00351258/file/ERR1897313_FASTQ_SSU_OTU.tsv	Root-Environmental-Aquatic-Freshwater-Lake
ERR1896921	MGYA00350866	https://www.ebi.ac.uk/metagenomics/api/v1/analyses/MGYA00350866/file/ERR1896921_FASTQ_SSU_OTU.tsv	Root-Environmental-Aquatic-Freshwater-Lake
ERR1582299	MGYA00352536	https://www.ebi.ac.uk/metagenomics/api/v1/analyses/MGYA00352536/file/ERR1582299_FASTQ_SSU_OTU.tsv	Root-Environmental-Aquatic-Freshwater-Lake
SRR4029366	MGYA00095526	https://www.ebi.ac.uk/metagenomics/api/v1/analyses/MGYA00095526/file/SRR4029366_MERGED_FASTQ_otu.tsv	Root-Environmental-Aquatic-Freshwater-Lake
ERR1990485	MGYA00128217	https://www.ebi.ac.uk/metagenomics/api/v1/analyses/MGYA00128217/file/ERR1990485_MERGED_FASTQ_otu.tsv	Root-Environmental-Aquatic-Freshwater-Lake
ERR1896695	MGYA00350640	https://www.ebi.ac.uk/metagenomics/api/v1/analyses/MGYA00350640/file/ERR1896695_FASTQ_SSU_OTU.tsv	Root-Environmental-Aquatic-Freshwater-Lake

ERR1897772	MGYA00351717	https://www.ebi.ac.uk/metagenomics/api/v1/analyses/MGYA00351717/file/ERR1897772_FASTQ_SSU_OTU.tsv	Root-Environmental-Aquatic-Freshwater-Lake
SRR2443036	MGYA00359040	https://www.ebi.ac.uk/metagenomics/api/v1/analyses/MGYA00359040/file/SRR2443036_MERGED_FASTQ_SSU_OTU.tsv	Root-Environmental-Aquatic-Freshwater-Lake
SRR7081169	MGYA00426459	https://www.ebi.ac.uk/metagenomics/api/v1/analyses/MGYA00426459/file/SRR7081169_MERGED_FASTQ_SSU_OTU.tsv	Root-Environmental-Aquatic-Freshwater-Lake
ERR1897004	MGYA00350949	https://www.ebi.ac.uk/metagenomics/api/v1/analyses/MGYA00350949/file/ERR1897004_FASTQ_SSU_OTU.tsv	Root-Environmental-Aquatic-Freshwater-Lake
ERR1896644	MGYA00350589	https://www.ebi.ac.uk/metagenomics/api/v1/analyses/MGYA00350589/file/ERR1896644_FASTQ_SSU_OTU.tsv	Root-Environmental-Aquatic-Freshwater-Lake
ERR1582248	MGYA00352485	https://www.ebi.ac.uk/metagenomics/api/v1/analyses/MGYA00352485/file/ERR1582248_FASTQ_SSU_OTU.tsv	Root-Environmental-Aquatic-Freshwater-Lake
ERR1583408	MGYA00353645	https://www.ebi.ac.uk/metagenomics/api/v1/analyses/MGYA00353645/file/ERR1583408_FASTQ_SSU_OTU.tsv	Root-Environmental-Aquatic-Freshwater-Lake
ERR1743316	MGYA00087127	https://www.ebi.ac.uk/metagenomics/api/v1/analyses/MGYA00087127/file/ERR1743316_MERGED_FASTQ_otu.tsv	Root-Environmental-Aquatic-Freshwater-Lake
ERR1897465	MGYA00351410	https://www.ebi.ac.uk/metagenomics/api/v1/analyses/MGYA00351410/file/ERR1897465_FASTQ_SSU_OTU.tsv	Root-Environmental-Aquatic-Freshwater-Lake
ERR1583069	MGYA00353306	https://www.ebi.ac.uk/metagenomics/api/v1/analyses/MGYA00353306/file/ERR1583069_FASTQ_SSU_OTU.tsv	Root-Environmental-Aquatic-Freshwater-Lake
ERR1582629	MGYA00352866	https://www.ebi.ac.uk/metagenomics/api/v1/analyses/MGYA00352866/file/ERR1582629_FASTQ_SSU_OTU.tsv	Root-Environmental-Aquatic-Freshwater-Lake
ERR1583285	MGYA00353522	https://www.ebi.ac.uk/metagenomics/api/v1/analyses/MGYA00353522/file/ERR1583285_FASTQ_SSU_OTU.tsv	Root-Environmental-Aquatic-Freshwater-Lake

ERR1429247	MGYA00107641	https://www.ebi.ac.uk/metagenomics/api/v1/analyses/MGYA00107641/file/ERR1429247_FASTQ_otu.tsv	Root-Environmental-Aquatic-Freshwater-Lake
ERR1582362	MGYA00352599	https://www.ebi.ac.uk/metagenomics/api/v1/analyses/MGYA00352599/file/ERR1582362_FASTQ_SSU_OTU.tsv	Root-Environmental-Aquatic-Freshwater-Lake
ERR1897689	MGYA00351634	https://www.ebi.ac.uk/metagenomics/api/v1/analyses/MGYA00351634/file/ERR1897689_FASTQ_SSU_OTU.tsv	Root-Environmental-Aquatic-Freshwater-Lake
ERR1437518	MGYA00107949	https://www.ebi.ac.uk/metagenomics/api/v1/analyses/MGYA00107949/file/ERR1437518_FASTQ_otu.tsv	Root-Environmental-Aquatic-Freshwater-Lake
ERR1582703	MGYA00352940	https://www.ebi.ac.uk/metagenomics/api/v1/analyses/MGYA00352940/file/ERR1582703_FASTQ_SSU_OTU.tsv	Root-Environmental-Aquatic-Freshwater-Lake
ERR1583143	MGYA00353380	https://www.ebi.ac.uk/metagenomics/api/v1/analyses/MGYA00353380/file/ERR1583143_FASTQ_SSU_OTU.tsv	Root-Environmental-Aquatic-Freshwater-Lake
ERR1897239	MGYA00351184	https://www.ebi.ac.uk/metagenomics/api/v1/analyses/MGYA00351184/file/ERR1897239_FASTQ_SSU_OTU.tsv	Root-Environmental-Aquatic-Freshwater-Lake
ERR1583192	MGYA00353429	https://www.ebi.ac.uk/metagenomics/api/v1/analyses/MGYA00353429/file/ERR1583192_FASTQ_SSU_OTU.tsv	Root-Environmental-Aquatic-Freshwater-Lake
ERR1582075	MGYA00352312	https://www.ebi.ac.uk/metagenomics/api/v1/analyses/MGYA00352312/file/ERR1582075_FASTQ_SSU_OTU.tsv	Root-Environmental-Aquatic-Freshwater-Lake
ERR1897658	MGYA00351603	https://www.ebi.ac.uk/metagenomics/api/v1/analyses/MGYA00351603/file/ERR1897658_FASTQ_SSU_OTU.tsv	Root-Environmental-Aquatic-Freshwater-Lake
ERR1535974	MGYA00320265	https://www.ebi.ac.uk/metagenomics/api/v1/analyses/MGYA00320265/file/ERR1535974_FASTQ_SSU_OTU.tsv	Root-Environmental-Aquatic-Freshwater-Lake
ERR1742283	MGYA00311584	https://www.ebi.ac.uk/metagenomics/api/v1/analyses/MGYA00311584/file/ERR1742283_FASTQ_SSU_OTU.tsv	Root-Environmental-Aquatic-Freshwater-Lake

ERR1582866	MGYA00353103	https://www.ebi.ac.uk/metagenomics/api/v1/analyses/MGYA00353103/file/ERR1582866_FASTQ_SSU_OTU.tsv	Root-Environmental-Aquatic-Freshwater-Lake
ERR1582414	MGYA00352651	https://www.ebi.ac.uk/metagenomics/api/v1/analyses/MGYA00352651/file/ERR1582414_FASTQ_SSU_OTU.tsv	Root-Environmental-Aquatic-Freshwater-Lake
ERR1583254	MGYA00353491	https://www.ebi.ac.uk/metagenomics/api/v1/analyses/MGYA00353491/file/ERR1583254_FASTQ_SSU_OTU.tsv	Root-Environmental-Aquatic-Freshwater-Lake
ERR1333523	MGYA00107606	https://www.ebi.ac.uk/metagenomics/api/v1/analyses/MGYA00107606/file/ERR1333523_FASTQ_otu.tsv	Root-Environmental-Aquatic-Freshwater-Lake
ERR1896953	MGYA00350898	https://www.ebi.ac.uk/metagenomics/api/v1/analyses/MGYA00350898/file/ERR1896953_FASTQ_SSU_OTU.tsv	Root-Environmental-Aquatic-Freshwater-Lake
ERR589314	MGYA00103442	https://www.ebi.ac.uk/metagenomics/api/v1/analyses/MGYA00103442/file/ERR589314_FASTQ_otu.tsv	Root-Environmental-Aquatic-Freshwater-Lake
ERR1897361	MGYA00351306	https://www.ebi.ac.uk/metagenomics/api/v1/analyses/MGYA00351306/file/ERR1897361_FASTQ_SSU_OTU.tsv	Root-Environmental-Aquatic-Freshwater-Lake
SRR2443044	MGYA00359048	https://www.ebi.ac.uk/metagenomics/api/v1/analyses/MGYA00359048/file/SRR2443044_MERGED_FASTQ_SSU_OTU.tsv	Root-Environmental-Aquatic-Freshwater-Lake
ERR1897700	MGYA00351645	https://www.ebi.ac.uk/metagenomics/api/v1/analyses/MGYA00351645/file/ERR1897700_FASTQ_SSU_OTU.tsv	Root-Environmental-Aquatic-Freshwater-Lake
ERR1896636	MGYA00350581	https://www.ebi.ac.uk/metagenomics/api/v1/analyses/MGYA00350581/file/ERR1896636_FASTQ_SSU_OTU.tsv	Root-Environmental-Aquatic-Freshwater-Lake
ERR1897076	MGYA00351021	https://www.ebi.ac.uk/metagenomics/api/v1/analyses/MGYA00351021/file/ERR1897076_FASTQ_SSU_OTU.tsv	Root-Environmental-Aquatic-Freshwater-Lake
ERR1897417	MGYA00351362	https://www.ebi.ac.uk/metagenomics/api/v1/analyses/MGYA00351362/file/ERR1897417_FASTQ_SSU_OTU.tsv	Root-Environmental-Aquatic-Freshwater-Lake

ERR1743364	MGYA00087170	https://www.ebi.ac.uk/metagenomics/api/v1/analyses/MGYA00087170/file/ERR1743364_MERGED_FASTQ_otu.tsv	Root-Environmental-Aquatic-Freshwater-Lake
SRR2442961	MGYA00358965	https://www.ebi.ac.uk/metagenomics/api/v1/analyses/MGYA00358965/file/SRR2442961_MERGED_FASTQ_SSU_OTU.tsv	Root-Environmental-Aquatic-Freshwater-Lake
ERR1896982	MGYA00350927	https://www.ebi.ac.uk/metagenomics/api/v1/analyses/MGYA00350927/file/ERR1896982_FASTQ_SSU_OTU.tsv	Root-Environmental-Aquatic-Freshwater-Lake
ERR1896712	MGYA00350657	https://www.ebi.ac.uk/metagenomics/api/v1/analyses/MGYA00350657/file/ERR1896712_FASTQ_SSU_OTU.tsv	Root-Environmental-Aquatic-Freshwater-Lake
ERR1897152	MGYA00351097	https://www.ebi.ac.uk/metagenomics/api/v1/analyses/MGYA00351097/file/ERR1897152_FASTQ_SSU_OTU.tsv	Root-Environmental-Aquatic-Freshwater-Lake
ERR1990502	MGYA00128215	https://www.ebi.ac.uk/metagenomics/api/v1/analyses/MGYA00128215/file/ERR1990502_MERGED_FASTQ_otu.tsv	Root-Environmental-Aquatic-Freshwater-Lake
SRR7081328	MGYA00424201	https://www.ebi.ac.uk/metagenomics/api/v1/analyses/MGYA00424201/file/SRR7081328_MERGED_FASTQ_SSU_OTU.tsv	Root-Environmental-Aquatic-Freshwater-Lake
ERR1897294	MGYA00351239	https://www.ebi.ac.uk/metagenomics/api/v1/analyses/MGYA00351239/file/ERR1897294_FASTQ_SSU_OTU.tsv	Root-Environmental-Aquatic-Freshwater-Lake
ERR1897533	MGYA00351478	https://www.ebi.ac.uk/metagenomics/api/v1/analyses/MGYA00351478/file/ERR1897533_FASTQ_SSU_OTU.tsv	Root-Environmental-Aquatic-Freshwater-Lake
ERR2868526	MGYA00377408	https://www.ebi.ac.uk/metagenomics/api/v1/analyses/MGYA00377408/file/ERR2868526_FASTQ_SSU_OTU.tsv	Root-Environmental-Aquatic-Freshwater-Lake
ERR389728	MGYA00103378	https://www.ebi.ac.uk/metagenomics/api/v1/analyses/MGYA00103378/file/ERR389728_FASTQ_otu.tsv	Root-Environmental-Aquatic-Freshwater-Lake
ERR1314369	MGYA00095218	https://www.ebi.ac.uk/metagenomics/api/v1/analyses/MGYA00095218/file/ERR1314369_MERGED_FASTQ_otu.tsv	Root-Environmental-Aquatic-Freshwater-Lake

ERR1896877	MGYA00350822	https://www.ebi.ac.uk/metagenomics/api/v1/analyses/MGYA00350822/file/ERR1896877_FASTQ_SSU_OTU.tsv	Root-Environmental-Aquatic-Freshwater-Lake
ERR1743291	MGYA00087107	https://www.ebi.ac.uk/metagenomics/api/v1/analyses/MGYA00087107/file/ERR1743291_MERGED_FASTQ_otu.tsv	Root-Environmental-Aquatic-Freshwater-Lake
ERR1897245	MGYA00351190	https://www.ebi.ac.uk/metagenomics/api/v1/analyses/MGYA00351190/file/ERR1897245_FASTQ_SSU_OTU.tsv	Root-Environmental-Aquatic-Freshwater-Lake
ERR1582468	MGYA00352705	https://www.ebi.ac.uk/metagenomics/api/v1/analyses/MGYA00352705/file/ERR1582468_FASTQ_SSU_OTU.tsv	Root-Environmental-Aquatic-Freshwater-Lake
ERR1583228	MGYA00353465	https://www.ebi.ac.uk/metagenomics/api/v1/analyses/MGYA00353465/file/ERR1583228_FASTQ_SSU_OTU.tsv	Root-Environmental-Aquatic-Freshwater-Lake
ERR1897624	MGYA00351569	https://www.ebi.ac.uk/metagenomics/api/v1/analyses/MGYA00351569/file/ERR1897624_FASTQ_SSU_OTU.tsv	Root-Environmental-Aquatic-Freshwater-Lake
ERR1897183	MGYA00351128	https://www.ebi.ac.uk/metagenomics/api/v1/analyses/MGYA00351128/file/ERR1897183_FASTQ_SSU_OTU.tsv	Root-Environmental-Aquatic-Freshwater-Lake
ERR1582151	MGYA00352388	https://www.ebi.ac.uk/metagenomics/api/v1/analyses/MGYA00352388/file/ERR1582151_FASTQ_SSU_OTU.tsv	Root-Environmental-Aquatic-Freshwater-Lake
SRR2443038	MGYA00359042	https://www.ebi.ac.uk/metagenomics/api/v1/analyses/MGYA00359042/file/SRR2443038_MERGED_FASTQ_SSU_OTU.tsv	Root-Environmental-Aquatic-Freshwater-Lake
ERR1582297	MGYA00352534	https://www.ebi.ac.uk/metagenomics/api/v1/analyses/MGYA00352534/file/ERR1582297_FASTQ_SSU_OTU.tsv	Root-Environmental-Aquatic-Freshwater-Lake
ERR1582530	MGYA00352767	https://www.ebi.ac.uk/metagenomics/api/v1/analyses/MGYA00352767/file/ERR1582530_FASTQ_SSU_OTU.tsv	Root-Environmental-Aquatic-Freshwater-Lake
ERR1582942	MGYA00353179	https://www.ebi.ac.uk/metagenomics/api/v1/analyses/MGYA00353179/file/ERR1582942_FASTQ_SSU_OTU.tsv	Root-Environmental-Aquatic-Freshwater-Lake

ERR1583370	MGYA00353607	https://www.ebi.ac.uk/metagenomics/api/v1/analyses/MGYA00353607/file/ERR1583370_FASTQ_SSU_OTU.tsv	Root-Environmental-Aquatic-Freshwater-Lake
ERR1583406	MGYA00353643	https://www.ebi.ac.uk/metagenomics/api/v1/analyses/MGYA00353643/file/ERR1583406_FASTQ_SSU_OTU.tsv	Root-Environmental-Aquatic-Freshwater-Lake
ERR1582246	MGYA00352483	https://www.ebi.ac.uk/metagenomics/api/v1/analyses/MGYA00352483/file/ERR1582246_FASTQ_SSU_OTU.tsv	Root-Environmental-Aquatic-Freshwater-Lake
ERR1582993	MGYA00353230	https://www.ebi.ac.uk/metagenomics/api/v1/analyses/MGYA00353230/file/ERR1582993_FASTQ_SSU_OTU.tsv	Root-Environmental-Aquatic-Freshwater-Lake
ERR430935	MGYA00103246	https://www.ebi.ac.uk/metagenomics/api/v1/analyses/MGYA00103246/file/ERR430935_FASTQ_otu.tsv	Root-Environmental-Aquatic-Freshwater-Lake
ERR1582627	MGYA00352864	https://www.ebi.ac.uk/metagenomics/api/v1/analyses/MGYA00352864/file/ERR1582627_FASTQ_SSU_OTU.tsv	Root-Environmental-Aquatic-Freshwater-Lake
ERR1582180	MGYA00352417	https://www.ebi.ac.uk/metagenomics/api/v1/analyses/MGYA00352417/file/ERR1582180_FASTQ_SSU_OTU.tsv	Root-Environmental-Aquatic-Freshwater-Lake
ERR1583067	MGYA00353304	https://www.ebi.ac.uk/metagenomics/api/v1/analyses/MGYA00353304/file/ERR1583067_FASTQ_SSU_OTU.tsv	Root-Environmental-Aquatic-Freshwater-Lake
ERR1743318	MGYA00087149	https://www.ebi.ac.uk/metagenomics/api/v1/analyses/MGYA00087149/file/ERR1743318_MERGED_FASTQ_otu.tsv	Root-Environmental-Aquatic-Freshwater-Lake
ERR1547127	MGYA00320838	https://www.ebi.ac.uk/metagenomics/api/v1/analyses/MGYA00320838/file/ERR1547127_FASTQ_SSU_OTU.tsv	Root-Environmental-Aquatic-Freshwater-Lake
ERR1897155	MGYA00351100	https://www.ebi.ac.uk/metagenomics/api/v1/analyses/MGYA00351100/file/ERR1897155_FASTQ_SSU_OTU.tsv	Root-Environmental-Aquatic-Freshwater-Lake
ERR1896715	MGYA00350660	https://www.ebi.ac.uk/metagenomics/api/v1/analyses/MGYA00350660/file/ERR1896715_FASTQ_SSU_OTU.tsv	Root-Environmental-Aquatic-Freshwater-Lake

ERR1582319	MGYA00352556	https://www.ebi.ac.uk/metagenomics/api/v1/analyses/MGYA00352556/file/ERR1582319_FASTQ_SSU_OTU.tsv	Root-Environmental-Aquatic-Freshwater-Lake
ERR1547078	MGYA00320789	https://www.ebi.ac.uk/metagenomics/api/v1/analyses/MGYA00320789/file/ERR1547078_FASTQ_SSU_OTU.tsv	Root-Environmental-Aquatic-Freshwater-Lake
ERR1897534	MGYA00351479	https://www.ebi.ac.uk/metagenomics/api/v1/analyses/MGYA00351479/file/ERR1897534_FASTQ_SSU_OTU.tsv	Root-Environmental-Aquatic-Freshwater-Lake
ERR1897293	MGYA00351238	https://www.ebi.ac.uk/metagenomics/api/v1/analyses/MGYA00351238/file/ERR1897293_FASTQ_SSU_OTU.tsv	Root-Environmental-Aquatic-Freshwater-Lake
ERR1583138	MGYA00353375	https://www.ebi.ac.uk/metagenomics/api/v1/analyses/MGYA00353375/file/ERR1583138_FASTQ_SSU_OTU.tsv	Root-Environmental-Aquatic-Freshwater-Lake
ERR1582778	MGYA00353015	https://www.ebi.ac.uk/metagenomics/api/v1/analyses/MGYA00353015/file/ERR1582778_FASTQ_SSU_OTU.tsv	Root-Environmental-Aquatic-Freshwater-Lake
ERR1897242	MGYA00351187	https://www.ebi.ac.uk/metagenomics/api/v1/analyses/MGYA00351187/file/ERR1897242_FASTQ_SSU_OTU.tsv	Root-Environmental-Aquatic-Freshwater-Lake
ERR1743296	MGYA00087108	https://www.ebi.ac.uk/metagenomics/api/v1/analyses/MGYA00087108/file/ERR1743296_MERGED_FASTQ_otu.tsv	Root-Environmental-Aquatic-Freshwater-Lake
ERR1896870	MGYA00350815	https://www.ebi.ac.uk/metagenomics/api/v1/analyses/MGYA00350815/file/ERR1896870_FASTQ_SSU_OTU.tsv	Root-Environmental-Aquatic-Freshwater-Lake
ERR1897184	MGYA00351129	https://www.ebi.ac.uk/metagenomics/api/v1/analyses/MGYA00351129/file/ERR1897184_FASTQ_SSU_OTU.tsv	Root-Environmental-Aquatic-Freshwater-Lake
ERR1897623	MGYA00351568	https://www.ebi.ac.uk/metagenomics/api/v1/analyses/MGYA00351568/file/ERR1897623_FASTQ_SSU_OTU.tsv	Root-Environmental-Aquatic-Freshwater-Lake
ERR1896928	MGYA00350873	https://www.ebi.ac.uk/metagenomics/api/v1/analyses/MGYA00350873/file/ERR1896928_FASTQ_SSU_OTU.tsv	Root-Environmental-Aquatic-Freshwater-Lake

ERR1582156	MGYA00352393	https://www.ebi.ac.uk/metagenomics/api/v1/analyses/MGYA00352393/file/ERR1582156_FASTQ_SSU_OTU.tsv	Root-Environmental-Aquatic-Freshwater-Lake
ERR1583377	MGYA00353614	https://www.ebi.ac.uk/metagenomics/api/v1/analyses/MGYA00353614/file/ERR1583377_FASTQ_SSU_OTU.tsv	Root-Environmental-Aquatic-Freshwater-Lake
ERR1582945	MGYA00353182	https://www.ebi.ac.uk/metagenomics/api/v1/analyses/MGYA00353182/file/ERR1582945_FASTQ_SSU_OTU.tsv	Root-Environmental-Aquatic-Freshwater-Lake
ERR1582537	MGYA00352774	https://www.ebi.ac.uk/metagenomics/api/v1/analyses/MGYA00352774/file/ERR1582537_FASTQ_SSU_OTU.tsv	Root-Environmental-Aquatic-Freshwater-Lake
ERR1582290	MGYA00352527	https://www.ebi.ac.uk/metagenomics/api/v1/analyses/MGYA00352527/file/ERR1582290_FASTQ_SSU_OTU.tsv	Root-Environmental-Aquatic-Freshwater-Lake
ERR1582994	MGYA00353231	https://www.ebi.ac.uk/metagenomics/api/v1/analyses/MGYA00353231/file/ERR1582994_FASTQ_SSU_OTU.tsv	Root-Environmental-Aquatic-Freshwater-Lake
ERR430932	MGYA00103258	https://www.ebi.ac.uk/metagenomics/api/v1/analyses/MGYA00103258/file/ERR430932_FASTQ_otu.tsv	Root-Environmental-Aquatic-Freshwater-Lake
ERR1582241	MGYA00352478	https://www.ebi.ac.uk/metagenomics/api/v1/analyses/MGYA00352478/file/ERR1582241_FASTQ_SSU_OTU.tsv	Root-Environmental-Aquatic-Freshwater-Lake
ERR1583401	MGYA00353638	https://www.ebi.ac.uk/metagenomics/api/v1/analyses/MGYA00353638/file/ERR1583401_FASTQ_SSU_OTU.tsv	Root-Environmental-Aquatic-Freshwater-Lake
ERR1583060	MGYA00353297	https://www.ebi.ac.uk/metagenomics/api/v1/analyses/MGYA00353297/file/ERR1583060_FASTQ_SSU_OTU.tsv	Root-Environmental-Aquatic-Freshwater-Lake
ERR1582187	MGYA00352424	https://www.ebi.ac.uk/metagenomics/api/v1/analyses/MGYA00352424/file/ERR1582187_FASTQ_SSU_OTU.tsv	Root-Environmental-Aquatic-Freshwater-Lake
ERR1582620	MGYA00352857	https://www.ebi.ac.uk/metagenomics/api/v1/analyses/MGYA00352857/file/ERR1582620_FASTQ_SSU_OTU.tsv	Root-Environmental-Aquatic-Freshwater-Lake

ERR1547120	MGYA00320831	https://www.ebi.ac.uk/metagenomics/api/v1/analyses/MGYA00320831/file/ERR1547120_FASTQ_SSU_OTU.tsv	Root-Environmental-Aquatic-Freshwater-Lake
ERR1582365	MGYA00352602	https://www.ebi.ac.uk/metagenomics/api/v1/analyses/MGYA00352602/file/ERR1582365_FASTQ_SSU_OTU.tsv	Root-Environmental-Aquatic-Freshwater-Lake
ERR1429240	MGYA00107622	https://www.ebi.ac.uk/metagenomics/api/v1/analyses/MGYA00107622/file/ERR1429240_FASTQ_otu.tsv	Root-Environmental-Aquatic-Freshwater-Lake
ERR1583282	MGYA00353519	https://www.ebi.ac.uk/metagenomics/api/v1/analyses/MGYA00353519/file/ERR1583282_FASTQ_SSU_OTU.tsv	Root-Environmental-Aquatic-Freshwater-Lake
ERR1897129	MGYA00351074	https://www.ebi.ac.uk/metagenomics/api/v1/analyses/MGYA00351074/file/ERR1897129_FASTQ_SSU_OTU.tsv	Root-Environmental-Aquatic-Freshwater-Lake
ERR1896769	MGYA00350714	https://www.ebi.ac.uk/metagenomics/api/v1/analyses/MGYA00350714/file/ERR1896769_FASTQ_SSU_OTU.tsv	Root-Environmental-Aquatic-Freshwater-Lake
ERR1583144	MGYA00353381	https://www.ebi.ac.uk/metagenomics/api/v1/analyses/MGYA00353381/file/ERR1583144_FASTQ_SSU_OTU.tsv	Root-Environmental-Aquatic-Freshwater-Lake
ERR1582704	MGYA00352941	https://www.ebi.ac.uk/metagenomics/api/v1/analyses/MGYA00352941/file/ERR1582704_FASTQ_SSU_OTU.tsv	Root-Environmental-Aquatic-Freshwater-Lake
ERR1897548	MGYA00351493	https://www.ebi.ac.uk/metagenomics/api/v1/analyses/MGYA00351493/file/ERR1897548_FASTQ_SSU_OTU.tsv	Root-Environmental-Aquatic-Freshwater-Lake
ERR1897599	MGYA00351544	https://www.ebi.ac.uk/metagenomics/api/v1/analyses/MGYA00351544/file/ERR1897599_FASTQ_SSU_OTU.tsv	Root-Environmental-Aquatic-Freshwater-Lake
ERR1582072	MGYA00352309	https://www.ebi.ac.uk/metagenomics/api/v1/analyses/MGYA00352309/file/ERR1582072_FASTQ_SSU_OTU.tsv	Root-Environmental-Aquatic-Freshwater-Lake
ERR1583195	MGYA00353432	https://www.ebi.ac.uk/metagenomics/api/v1/analyses/MGYA00353432/file/ERR1583195_FASTQ_SSU_OTU.tsv	Root-Environmental-Aquatic-Freshwater-Lake

ERR1742284	MGYA00311585	https://www.ebi.ac.uk/metagenomics/api/v1/analyses/MGYA00311585/file/ERR1742284_FASTQ_SSU_OTU.tsv	Root-Environmental-Aquatic-Freshwater-Lake
ERR1535973	MGYA00320264	https://www.ebi.ac.uk/metagenomics/api/v1/analyses/MGYA00320264/file/ERR1535973_FASTQ_SSU_OTU.tsv	Root-Environmental-Aquatic-Freshwater-Lake
ERR1333524	MGYA00107591	https://www.ebi.ac.uk/metagenomics/api/v1/analyses/MGYA00107591/file/ERR1333524_FASTQ_otu.tsv	Root-Environmental-Aquatic-Freshwater-Lake
ERR1583253	MGYA00353490	https://www.ebi.ac.uk/metagenomics/api/v1/analyses/MGYA00353490/file/ERR1583253_FASTQ_SSU_OTU.tsv	Root-Environmental-Aquatic-Freshwater-Lake
ERR1582413	MGYA00352650	https://www.ebi.ac.uk/metagenomics/api/v1/analyses/MGYA00352650/file/ERR1582413_FASTQ_SSU_OTU.tsv	Root-Environmental-Aquatic-Freshwater-Lake
ERR1582861	MGYA00353098	https://www.ebi.ac.uk/metagenomics/api/v1/analyses/MGYA00353098/file/ERR1582861_FASTQ_SSU_OTU.tsv	Root-Environmental-Aquatic-Freshwater-Lake
ERR1897366	MGYA00351311	https://www.ebi.ac.uk/metagenomics/api/v1/analyses/MGYA00351311/file/ERR1897366_FASTQ_SSU_OTU.tsv	Root-Environmental-Aquatic-Freshwater-Lake
ERR589313	MGYA00103416	https://www.ebi.ac.uk/metagenomics/api/v1/analyses/MGYA00103416/file/ERR589313_FASTQ_otu.tsv	Root-Environmental-Aquatic-Freshwater-Lake
ERR1896954	MGYA00350899	https://www.ebi.ac.uk/metagenomics/api/v1/analyses/MGYA00350899/file/ERR1896954_FASTQ_SSU_OTU.tsv	Root-Environmental-Aquatic-Freshwater-Lake
ERR1582939	MGYA00353176	https://www.ebi.ac.uk/metagenomics/api/v1/analyses/MGYA00353176/file/ERR1582939_FASTQ_SSU_OTU.tsv	Root-Environmental-Aquatic-Freshwater-Lake
SRR2443043	MGYA00359047	https://www.ebi.ac.uk/metagenomics/api/v1/analyses/MGYA00359047/file/SRR2443043_MERGED_FASTQ_SSU_OTU.tsv	Root-Environmental-Aquatic-Freshwater-Lake
ERR1897707	MGYA00351652	https://www.ebi.ac.uk/metagenomics/api/v1/analyses/MGYA00351652/file/ERR1897707_FASTQ_SSU_OTU.tsv	Root-Environmental-Aquatic-Freshwater-Lake

ERR1897071	MGYA00351016	https://www.ebi.ac.uk/metagenomics/api/v1/analyses/MGYA00351016/file/ERR1897071_FASTQ_SSU_OTU.tsv	Root-Environmental-Aquatic-Freshwater-Lake
ERR1896985	MGYA00350930	https://www.ebi.ac.uk/metagenomics/api/v1/analyses/MGYA00350930/file/ERR1896985_FASTQ_SSU_OTU.tsv	Root-Environmental-Aquatic-Freshwater-Lake
SRR2442966	MGYA00358970	https://www.ebi.ac.uk/metagenomics/api/v1/analyses/MGYA00358970/file/SRR2442966_MERGED_FASTQ_SSU_OTU.tsv	Root-Environmental-Aquatic-Freshwater-Lake
ERR1743363	MGYA00087215	https://www.ebi.ac.uk/metagenomics/api/v1/analyses/MGYA00087215/file/ERR1743363_MERGED_FASTQ_otu.tsv	Root-Environmental-Aquatic-Freshwater-Lake
ERR1897410	MGYA00351355	https://www.ebi.ac.uk/metagenomics/api/v1/analyses/MGYA00351355/file/ERR1897410_FASTQ_SSU_OTU.tsv	Root-Environmental-Aquatic-Freshwater-Lake
ERR1582828	MGYA00353065	https://www.ebi.ac.uk/metagenomics/api/v1/analyses/MGYA00353065/file/ERR1582828_FASTQ_SSU_OTU.tsv	Root-Environmental-Aquatic-Freshwater-Lake
ERR1897616	MGYA00351561	https://www.ebi.ac.uk/metagenomics/api/v1/analyses/MGYA00351561/file/ERR1897616_FASTQ_SSU_OTU.tsv	Root-Environmental-Aquatic-Freshwater-Lake
ERR1897277	MGYA00351222	https://www.ebi.ac.uk/metagenomics/api/v1/analyses/MGYA00351222/file/ERR1897277_FASTQ_SSU_OTU.tsv	Root-Environmental-Aquatic-Freshwater-Lake
ERR1896845	MGYA00350790	https://www.ebi.ac.uk/metagenomics/api/v1/analyses/MGYA00350790/file/ERR1896845_FASTQ_SSU_OTU.tsv	Root-Environmental-Aquatic-Freshwater-Lake
ERR1896894	MGYA00350839	https://www.ebi.ac.uk/metagenomics/api/v1/analyses/MGYA00350839/file/ERR1896894_FASTQ_SSU_OTU.tsv	Root-Environmental-Aquatic-Freshwater-Lake
ERR1897501	MGYA00351446	https://www.ebi.ac.uk/metagenomics/api/v1/analyses/MGYA00351446/file/ERR1897501_FASTQ_SSU_OTU.tsv	Root-Environmental-Aquatic-Freshwater-Lake
ERR658213	MGYA00103499	https://www.ebi.ac.uk/metagenomics/api/v1/analyses/MGYA00103499/file/ERR658213_FASTQ_otu.tsv	Root-Environmental-Aquatic-Freshwater-Lake

ERR1897160	MGYA00351105	https://www.ebi.ac.uk/metagenomics/api/v1/analyses/MGYA00351105/file/ERR1897160_FASTQ_SSU_OTU.tsv	Root-Environmental-Aquatic-Freshwater-Lake
ERR1896720	MGYA00350665	https://www.ebi.ac.uk/metagenomics/api/v1/analyses/MGYA00350665/file/ERR1896720_FASTQ_SSU_OTU.tsv	Root-Environmental-Aquatic-Freshwater-Lake
ERR1583055	MGYA00353292	https://www.ebi.ac.uk/metagenomics/api/v1/analyses/MGYA00353292/file/ERR1583055_FASTQ_SSU_OTU.tsv	Root-Environmental-Aquatic-Freshwater-Lake
ERR1582615	MGYA00352852	https://www.ebi.ac.uk/metagenomics/api/v1/analyses/MGYA00352852/file/ERR1582615_FASTQ_SSU_OTU.tsv	Root-Environmental-Aquatic-Freshwater-Lake
ERR1547115	MGYA00320826	https://www.ebi.ac.uk/metagenomics/api/v1/analyses/MGYA00320826/file/ERR1547115_FASTQ_SSU_OTU.tsv	Root-Environmental-Aquatic-Freshwater-Lake
ERR1897459	MGYA00351404	https://www.ebi.ac.uk/metagenomics/api/v1/analyses/MGYA00351404/file/ERR1897459_FASTQ_SSU_OTU.tsv	Root-Environmental-Aquatic-Freshwater-Lake
ERR1582274	MGYA00352511	https://www.ebi.ac.uk/metagenomics/api/v1/analyses/MGYA00352511/file/ERR1582274_FASTQ_SSU_OTU.tsv	Root-Environmental-Aquatic-Freshwater-Lake
ERR1583434	MGYA00353671	https://www.ebi.ac.uk/metagenomics/api/v1/analyses/MGYA00353671/file/ERR1583434_FASTQ_SSU_OTU.tsv	Root-Environmental-Aquatic-Freshwater-Lake
ERR1583393	MGYA00353630	https://www.ebi.ac.uk/metagenomics/api/v1/analyses/MGYA00353630/file/ERR1583393_FASTQ_SSU_OTU.tsv	Root-Environmental-Aquatic-Freshwater-Lake
ERR1897038	MGYA00350983	https://www.ebi.ac.uk/metagenomics/api/v1/analyses/MGYA00350983/file/ERR1897038_FASTQ_SSU_OTU.tsv	Root-Environmental-Aquatic-Freshwater-Lake
ERR1896678	MGYA00350623	https://www.ebi.ac.uk/metagenomics/api/v1/analyses/MGYA00350623/file/ERR1896678_FASTQ_SSU_OTU.tsv	Root-Environmental-Aquatic-Freshwater-Lake
SRR7081155	MGYA00413194	https://www.ebi.ac.uk/metagenomics/api/v1/analyses/MGYA00413194/file/SRR7081155_MERGED_FASTQ_SSU_OTU.tsv	Root-Environmental-Aquatic-Freshwater-Lake

ERR1583342	MGYA00353579	https://www.ebi.ac.uk/metagenomics/api/v1/analyses/MGYA00353579/file/ERR1583342_FASTQ_SSU_OTU.tsv	Root-Environmental-Aquatic-Freshwater-Lake
ERR1582970	MGYA00353207	https://www.ebi.ac.uk/metagenomics/api/v1/analyses/MGYA00353207/file/ERR1582970_FASTQ_SSU_OTU.tsv	Root-Environmental-Aquatic-Freshwater-Lake
ERR1582502	MGYA00352739	https://www.ebi.ac.uk/metagenomics/api/v1/analyses/MGYA00352739/file/ERR1582502_FASTQ_SSU_OTU.tsv	Root-Environmental-Aquatic-Freshwater-Lake
ERR1897488	MGYA00351433	https://www.ebi.ac.uk/metagenomics/api/v1/analyses/MGYA00351433/file/ERR1897488_FASTQ_SSU_OTU.tsv	Root-Environmental-Aquatic-Freshwater-Lake
ERR1582163	MGYA00352400	https://www.ebi.ac.uk/metagenomics/api/v1/analyses/MGYA00352400/file/ERR1582163_FASTQ_SSU_OTU.tsv	Root-Environmental-Aquatic-Freshwater-Lake
ERR1583084	MGYA00353321	https://www.ebi.ac.uk/metagenomics/api/v1/analyses/MGYA00353321/file/ERR1583084_FASTQ_SSU_OTU.tsv	Root-Environmental-Aquatic-Freshwater-Lake
ERR1535946	MGYA00320237	https://www.ebi.ac.uk/metagenomics/api/v1/analyses/MGYA00320237/file/ERR1535946_FASTQ_SSU_OTU.tsv	Root-Environmental-Aquatic-Freshwater-Lake
ERR1583266	MGYA00353503	https://www.ebi.ac.uk/metagenomics/api/v1/analyses/MGYA00353503/file/ERR1583266_FASTQ_SSU_OTU.tsv	Root-Environmental-Aquatic-Freshwater-Lake
ERR1333511	MGYA00107589	https://www.ebi.ac.uk/metagenomics/api/v1/analyses/MGYA00107589/file/ERR1333511_FASTQ_otu.tsv	Root-Environmental-Aquatic-Freshwater-Lake
ERR1582426	MGYA00352663	https://www.ebi.ac.uk/metagenomics/api/v1/analyses/MGYA00352663/file/ERR1582426_FASTQ_SSU_OTU.tsv	Root-Environmental-Aquatic-Freshwater-Lake
ERR658178	MGYA00103484	https://www.ebi.ac.uk/metagenomics/api/v1/analyses/MGYA00103484/file/ERR658178_FASTQ_otu.tsv	Root-Environmental-Aquatic-Freshwater-Lake
ERR1582854	MGYA00353091	https://www.ebi.ac.uk/metagenomics/api/v1/analyses/MGYA00353091/file/ERR1582854_FASTQ_SSU_OTU.tsv	Root-Environmental-Aquatic-Freshwater-Lake

ERR1582381	MGYA00352618	https://www.ebi.ac.uk/metagenomics/api/v1/analyses/MGYA00352618/file/ERR1582381_FASTQ_SSU_OTU.tsv	Root-Environmental-Aquatic-Freshwater-Lake
ERR1896839	MGYA00350784	https://www.ebi.ac.uk/metagenomics/api/v1/analyses/MGYA00350784/file/ERR1896839_FASTQ_SSU_OTU.tsv	Root-Environmental-Aquatic-Freshwater-Lake
ERR1582047	MGYA00352284	https://www.ebi.ac.uk/metagenomics/api/v1/analyses/MGYA00352284/file/ERR1582047_FASTQ_SSU_OTU.tsv	Root-Environmental-Aquatic-Freshwater-Lake
ERR1583171	MGYA00353408	https://www.ebi.ac.uk/metagenomics/api/v1/analyses/MGYA00353408/file/ERR1583171_FASTQ_SSU_OTU.tsv	Root-Environmental-Aquatic-Freshwater-Lake
ERR1582096	MGYA00352333	https://www.ebi.ac.uk/metagenomics/api/v1/analyses/MGYA00352333/file/ERR1582096_FASTQ_SSU_OTU.tsv	Root-Environmental-Aquatic-Freshwater-Lake
ERR1582731	MGYA00352968	https://www.ebi.ac.uk/metagenomics/api/v1/analyses/MGYA00352968/file/ERR1582731_FASTQ_SSU_OTU.tsv	Root-Environmental-Aquatic-Freshwater-Lake
SRR7081366	MGYA00423212	https://www.ebi.ac.uk/metagenomics/api/v1/analyses/MGYA00423212/file/SRR7081366_MERGED_FASTQ_SSU_OTU.tsv	Root-Environmental-Aquatic-Freshwater-Lake
ERR1582885	MGYA00353122	https://www.ebi.ac.uk/metagenomics/api/v1/analyses/MGYA00353122/file/ERR1582885_FASTQ_SSU_OTU.tsv	Root-Environmental-Aquatic-Freshwater-Lake
ERR1429275	MGYA00107654	https://www.ebi.ac.uk/metagenomics/api/v1/analyses/MGYA00107654/file/ERR1429275_FASTQ_otu.tsv	Root-Environmental-Aquatic-Freshwater-Lake
ERR1582350	MGYA00352587	https://www.ebi.ac.uk/metagenomics/api/v1/analyses/MGYA00352587/file/ERR1582350_FASTQ_SSU_OTU.tsv	Root-Environmental-Aquatic-Freshwater-Lake
ERR1583510	MGYA00353747	https://www.ebi.ac.uk/metagenomics/api/v1/analyses/MGYA00353747/file/ERR1583510_FASTQ_SSU_OTU.tsv	Root-Environmental-Aquatic-Freshwater-Lake
SRR2442953	MGYA00358957	https://www.ebi.ac.uk/metagenomics/api/v1/analyses/MGYA00358957/file/SRR2442953_MERGED_FASTQ_SSU_OTU.tsv	Root-Environmental-Aquatic-Freshwater-Lake

ERR1743356	MGYA00087126	https://www.ebi.ac.uk/metagenomics/api/v1/analyses/MGYA00087126/file/ERR1743356_MERGED_FASTQ_otu.tsv	Root-Environmental-Aquatic-Freshwater-Lake
ERR1897425	MGYA00351370	https://www.ebi.ac.uk/metagenomics/api/v1/analyses/MGYA00351370/file/ERR1897425_FASTQ_SSU_OTU.tsv	Root-Environmental-Aquatic-Freshwater-Lake
ERR1897382	MGYA00351327	https://www.ebi.ac.uk/metagenomics/api/v1/analyses/MGYA00351327/file/ERR1897382_FASTQ_SSU_OTU.tsv	Root-Environmental-Aquatic-Freshwater-Lake
ERR1583029	MGYA00353266	https://www.ebi.ac.uk/metagenomics/api/v1/analyses/MGYA00353266/file/ERR1583029_FASTQ_SSU_OTU.tsv	Root-Environmental-Aquatic-Freshwater-Lake
ERR1582669	MGYA00352906	https://www.ebi.ac.uk/metagenomics/api/v1/analyses/MGYA00352906/file/ERR1582669_FASTQ_SSU_OTU.tsv	Root-Environmental-Aquatic-Freshwater-Lake
ERR1897044	MGYA00350989	https://www.ebi.ac.uk/metagenomics/api/v1/analyses/MGYA00350989/file/ERR1897044_FASTQ_SSU_OTU.tsv	Root-Environmental-Aquatic-Freshwater-Lake
ERR1582208	MGYA00352445	https://www.ebi.ac.uk/metagenomics/api/v1/analyses/MGYA00352445/file/ERR1582208_FASTQ_SSU_OTU.tsv	Root-Environmental-Aquatic-Freshwater-Lake
ERR1583448	MGYA00353685	https://www.ebi.ac.uk/metagenomics/api/v1/analyses/MGYA00353685/file/ERR1583448_FASTQ_SSU_OTU.tsv	Root-Environmental-Aquatic-Freshwater-Lake
ERR1583499	MGYA00353736	https://www.ebi.ac.uk/metagenomics/api/v1/analyses/MGYA00353736/file/ERR1583499_FASTQ_SSU_OTU.tsv	Root-Environmental-Aquatic-Freshwater-Lake
ERR1897732	MGYA00351677	https://www.ebi.ac.uk/metagenomics/api/v1/analyses/MGYA00351677/file/ERR1897732_FASTQ_SSU_OTU.tsv	Root-Environmental-Aquatic-Freshwater-Lake
ERR589326	MGYA00103407	https://www.ebi.ac.uk/metagenomics/api/v1/analyses/MGYA00103407/file/ERR589326_FASTQ_otu.tsv	Root-Environmental-Aquatic-Freshwater-Lake
ERR1897353	MGYA00351298	https://www.ebi.ac.uk/metagenomics/api/v1/analyses/MGYA00351298/file/ERR1897353_FASTQ_SSU_OTU.tsv	Root-Environmental-Aquatic-Freshwater-Lake

SRR2442982	MGYA00358986	https://www.ebi.ac.uk/metagenomics/api/v1/analyses/MGYA00358986/file/SRR2442982_MERGED_FASTQ_SSU_OTU.tsv	Root-Environmental-Aquatic-Freshwater-Lake
ERR1743387	MGYA00087214	https://www.ebi.ac.uk/metagenomics/api/v1/analyses/MGYA00087214/file/ERR1743387_MERGED_FASTQ_otu.tsv	Root-Environmental-Aquatic-Freshwater-Lake
ERR1896961	MGYA00350906	https://www.ebi.ac.uk/metagenomics/api/v1/analyses/MGYA00350906/file/ERR1896961_FASTQ_SSU_OTU.tsv	Root-Environmental-Aquatic-Freshwater-Lake
ERR1582386	MGYA00352623	https://www.ebi.ac.uk/metagenomics/api/v1/analyses/MGYA00352623/file/ERR1582386_FASTQ_SSU_OTU.tsv	Root-Environmental-Aquatic-Freshwater-Lake
ERR1582853	MGYA00353090	https://www.ebi.ac.uk/metagenomics/api/v1/analyses/MGYA00353090/file/ERR1582853_FASTQ_SSU_OTU.tsv	Root-Environmental-Aquatic-Freshwater-Lake
ERR1582421	MGYA00352658	https://www.ebi.ac.uk/metagenomics/api/v1/analyses/MGYA00352658/file/ERR1582421_FASTQ_SSU_OTU.tsv	Root-Environmental-Aquatic-Freshwater-Lake
ERR1333516	MGYA00107610	https://www.ebi.ac.uk/metagenomics/api/v1/analyses/MGYA00107610/file/ERR1333516_FASTQ_otu.tsv	Root-Environmental-Aquatic-Freshwater-Lake
ERR1583261	MGYA00353498	https://www.ebi.ac.uk/metagenomics/api/v1/analyses/MGYA00353498/file/ERR1583261_FASTQ_SSU_OTU.tsv	Root-Environmental-Aquatic-Freshwater-Lake
ERR1582040	MGYA00352277	https://www.ebi.ac.uk/metagenomics/api/v1/analyses/MGYA00352277/file/ERR1582040_FASTQ_SSU_OTU.tsv	Root-Environmental-Aquatic-Freshwater-Lake
ERR1582736	MGYA00352973	https://www.ebi.ac.uk/metagenomics/api/v1/analyses/MGYA00352973/file/ERR1582736_FASTQ_SSU_OTU.tsv	Root-Environmental-Aquatic-Freshwater-Lake
ERR1582091	MGYA00352328	https://www.ebi.ac.uk/metagenomics/api/v1/analyses/MGYA00352328/file/ERR1582091_FASTQ_SSU_OTU.tsv	Root-Environmental-Aquatic-Freshwater-Lake
ERR1583176	MGYA00353413	https://www.ebi.ac.uk/metagenomics/api/v1/analyses/MGYA00353413/file/ERR1583176_FASTQ_SSU_OTU.tsv	Root-Environmental-Aquatic-Freshwater-Lake

SRR7081361	MGYA00426444	https://www.ebi.ac.uk/metagenomics/api/v1/analyses/MGYA00426444/file/SRR7081361_MERGED_FASTQ_SSU_OTU.tsv	Root-Environmental-Aquatic-Freshwater-Lake
ERR1547036	MGYA00320747	https://www.ebi.ac.uk/metagenomics/api/v1/analyses/MGYA00320747/file/ERR1547036_FASTQ_SSU_OTU.tsv	Root-Environmental-Aquatic-Freshwater-Lake
ERR1583517	MGYA00353754	https://www.ebi.ac.uk/metagenomics/api/v1/analyses/MGYA00353754/file/ERR1583517_FASTQ_SSU_OTU.tsv	Root-Environmental-Aquatic-Freshwater-Lake
ERR1582357	MGYA00352594	https://www.ebi.ac.uk/metagenomics/api/v1/analyses/MGYA00352594/file/ERR1582357_FASTQ_SSU_OTU.tsv	Root-Environmental-Aquatic-Freshwater-Lake
ERR1429272	MGYA00107634	https://www.ebi.ac.uk/metagenomics/api/v1/analyses/MGYA00107634/file/ERR1429272_FASTQ_otu.tsv	Root-Environmental-Aquatic-Freshwater-Lake
ERR1582882	MGYA00353119	https://www.ebi.ac.uk/metagenomics/api/v1/analyses/MGYA00353119/file/ERR1582882_FASTQ_SSU_OTU.tsv	Root-Environmental-Aquatic-Freshwater-Lake
ERR1535990	MGYA00320281	https://www.ebi.ac.uk/metagenomics/api/v1/analyses/MGYA00320281/file/ERR1535990_FASTQ_SSU_OTU.tsv	Root-Environmental-Aquatic-Freshwater-Lake
ERR1897385	MGYA00351330	https://www.ebi.ac.uk/metagenomics/api/v1/analyses/MGYA00351330/file/ERR1897385_FASTQ_SSU_OTU.tsv	Root-Environmental-Aquatic-Freshwater-Lake
SRR7081239	MGYA00425692	https://www.ebi.ac.uk/metagenomics/api/v1/analyses/MGYA00425692/file/SRR7081239_MERGED_FASTQ_SSU_OTU.tsv	Root-Environmental-Aquatic-Freshwater-Lake
ERR1897422	MGYA00351367	https://www.ebi.ac.uk/metagenomics/api/v1/analyses/MGYA00351367/file/ERR1897422_FASTQ_SSU_OTU.tsv	Root-Environmental-Aquatic-Freshwater-Lake
ERR1743351	MGYA00087158	https://www.ebi.ac.uk/metagenomics/api/v1/analyses/MGYA00087158/file/ERR1743351_MERGED_FASTQ_otu.tsv	Root-Environmental-Aquatic-Freshwater-Lake
SRR2442954	MGYA00358958	https://www.ebi.ac.uk/metagenomics/api/v1/analyses/MGYA00358958/file/SRR2442954_MERGED_FASTQ_SSU_OTU.tsv	Root-Environmental-Aquatic-Freshwater-Lake

SRR330054	MGYA00356817	https://www.ebi.ac.uk/metagenomics/api/v1/analyses/MGYA00356817/file/SRR330054_FASTQ_SSU_OTU.tsv	Root-Environmental-Aquatic-Freshwater-Lake
ERR1897043	MGYA00350988	https://www.ebi.ac.uk/metagenomics/api/v1/analyses/MGYA00350988/file/ERR1897043_FASTQ_SSU_OTU.tsv	Root-Environmental-Aquatic-Freshwater-Lake
ERR1582579	MGYA00352816	https://www.ebi.ac.uk/metagenomics/api/v1/analyses/MGYA00352816/file/ERR1582579_FASTQ_SSU_OTU.tsv	Root-Environmental-Aquatic-Freshwater-Lake
ERR1583339	MGYA00353576	https://www.ebi.ac.uk/metagenomics/api/v1/analyses/MGYA00353576/file/ERR1583339_FASTQ_SSU_OTU.tsv	Root-Environmental-Aquatic-Freshwater-Lake
ERR1897735	MGYA00351680	https://www.ebi.ac.uk/metagenomics/api/v1/analyses/MGYA00351680/file/ERR1897735_FASTQ_SSU_OTU.tsv	Root-Environmental-Aquatic-Freshwater-Lake
ERR1897092	MGYA00351037	https://www.ebi.ac.uk/metagenomics/api/v1/analyses/MGYA00351037/file/ERR1897092_FASTQ_SSU_OTU.tsv	Root-Environmental-Aquatic-Freshwater-Lake
ERR1582118	MGYA00352355	https://www.ebi.ac.uk/metagenomics/api/v1/analyses/MGYA00352355/file/ERR1582118_FASTQ_SSU_OTU.tsv	Root-Environmental-Aquatic-Freshwater-Lake
ERR1896966	MGYA00350911	https://www.ebi.ac.uk/metagenomics/api/v1/analyses/MGYA00350911/file/ERR1896966_FASTQ_SSU_OTU.tsv	Root-Environmental-Aquatic-Freshwater-Lake
ERR1743380	MGYA00087219	https://www.ebi.ac.uk/metagenomics/api/v1/analyses/MGYA00087219/file/ERR1743380_MERGED_FASTQ_otu.tsv	Root-Environmental-Aquatic-Freshwater-Lake
SRR2442985	MGYA00358989	https://www.ebi.ac.uk/metagenomics/api/v1/analyses/MGYA00358989/file/SRR2442985_MERGED_FASTQ_SSU_OTU.tsv	Root-Environmental-Aquatic-Freshwater-Lake
ERR1897354	MGYA00351299	https://www.ebi.ac.uk/metagenomics/api/v1/analyses/MGYA00351299/file/ERR1897354_FASTQ_SSU_OTU.tsv	Root-Environmental-Aquatic-Freshwater-Lake
ERR589321	MGYA00103429	https://www.ebi.ac.uk/metagenomics/api/v1/analyses/MGYA00103429/file/ERR589321_FASTQ_otu.tsv	Root-Environmental-Aquatic-Freshwater-Lake

ERR1897611	MGYA00351556	https://www.ebi.ac.uk/metagenomics/api/v1/analyses/MGYA00351556/file/ERR1897611_FASTQ_SSU_OTU.tsv	Root-Environmental-Aquatic-Freshwater-Lake
ERR1896842	MGYA00350787	https://www.ebi.ac.uk/metagenomics/api/v1/analyses/MGYA00350787/file/ERR1896842_FASTQ_SSU_OTU.tsv	Root-Environmental-Aquatic-Freshwater-Lake
ERR1897270	MGYA00351215	https://www.ebi.ac.uk/metagenomics/api/v1/analyses/MGYA00351215/file/ERR1897270_FASTQ_SSU_OTU.tsv	Root-Environmental-Aquatic-Freshwater-Lake
ERR1897506	MGYA00351451	https://www.ebi.ac.uk/metagenomics/api/v1/analyses/MGYA00351451/file/ERR1897506_FASTQ_SSU_OTU.tsv	Root-Environmental-Aquatic-Freshwater-Lake
ERR1743275	MGYA00087135	https://www.ebi.ac.uk/metagenomics/api/v1/analyses/MGYA00087135/file/ERR1743275_MERGED_FASTQ_otu.tsv	Root-Environmental-Aquatic-Freshwater-Lake
ERR1896893	MGYA00350838	https://www.ebi.ac.uk/metagenomics/api/v1/analyses/MGYA00350838/file/ERR1896893_FASTQ_SSU_OTU.tsv	Root-Environmental-Aquatic-Freshwater-Lake
ERR658214	MGYA00103472	https://www.ebi.ac.uk/metagenomics/api/v1/analyses/MGYA00103472/file/ERR658214_FASTQ_otu.tsv	Root-Environmental-Aquatic-Freshwater-Lake
ERR1896727	MGYA00350672	https://www.ebi.ac.uk/metagenomics/api/v1/analyses/MGYA00350672/file/ERR1896727_FASTQ_SSU_OTU.tsv	Root-Environmental-Aquatic-Freshwater-Lake
ERR1897167	MGYA00351112	https://www.ebi.ac.uk/metagenomics/api/v1/analyses/MGYA00351112/file/ERR1897167_FASTQ_SSU_OTU.tsv	Root-Environmental-Aquatic-Freshwater-Lake
ERR1582612	MGYA00352849	https://www.ebi.ac.uk/metagenomics/api/v1/analyses/MGYA00352849/file/ERR1582612_FASTQ_SSU_OTU.tsv	Root-Environmental-Aquatic-Freshwater-Lake
ERR1583052	MGYA00353289	https://www.ebi.ac.uk/metagenomics/api/v1/analyses/MGYA00353289/file/ERR1583052_FASTQ_SSU_OTU.tsv	Root-Environmental-Aquatic-Freshwater-Lake
SRR7081245	MGYA00425689	https://www.ebi.ac.uk/metagenomics/api/v1/analyses/MGYA00425689/file/SRR7081245_MERGED_FASTQ_SSU_OTU.tsv	Root-Environmental-Aquatic-Freshwater-Lake

ERR1547112	MGYA00320823	https://www.ebi.ac.uk/metagenomics/api/v1/analyses/MGYA00320823/file/ERR1547112_FASTQ_SSU_OTU.tsv	Root-Environmental-Aquatic-Freshwater-Lake
ERR1583394	MGYA00353631	https://www.ebi.ac.uk/metagenomics/api/v1/analyses/MGYA00353631/file/ERR1583394_FASTQ_SSU_OTU.tsv	Root-Environmental-Aquatic-Freshwater-Lake
ERR1583433	MGYA00353670	https://www.ebi.ac.uk/metagenomics/api/v1/analyses/MGYA00353670/file/ERR1583433_FASTQ_SSU_OTU.tsv	Root-Environmental-Aquatic-Freshwater-Lake
ERR1582273	MGYA00352510	https://www.ebi.ac.uk/metagenomics/api/v1/analyses/MGYA00352510/file/ERR1582273_FASTQ_SSU_OTU.tsv	Root-Environmental-Aquatic-Freshwater-Lake
ERR1897749	MGYA00351694	https://www.ebi.ac.uk/metagenomics/api/v1/analyses/MGYA00351694/file/ERR1897749_FASTQ_SSU_OTU.tsv	Root-Environmental-Aquatic-Freshwater-Lake
ERR1582505	MGYA00352742	https://www.ebi.ac.uk/metagenomics/api/v1/analyses/MGYA00352742/file/ERR1582505_FASTQ_SSU_OTU.tsv	Root-Environmental-Aquatic-Freshwater-Lake
ERR1582977	MGYA00353214	https://www.ebi.ac.uk/metagenomics/api/v1/analyses/MGYA00353214/file/ERR1582977_FASTQ_SSU_OTU.tsv	Root-Environmental-Aquatic-Freshwater-Lake
ERR1583345	MGYA00353582	https://www.ebi.ac.uk/metagenomics/api/v1/analyses/MGYA00353582/file/ERR1583345_FASTQ_SSU_OTU.tsv	Root-Environmental-Aquatic-Freshwater-Lake
SRR3987482	MGYA00095523	https://www.ebi.ac.uk/metagenomics/api/v1/analyses/MGYA00095523/file/SRR3987482_MERGED_FASTQ_otu.tsv	Root-Environmental-Aquatic-Freshwater-Lake
ERR1897328	MGYA00351273	https://www.ebi.ac.uk/metagenomics/api/v1/analyses/MGYA00351273/file/ERR1897328_FASTQ_SSU_OTU.tsv	Root-Environmental-Aquatic-Freshwater-Lake
ERR1583083	MGYA00353320	https://www.ebi.ac.uk/metagenomics/api/v1/analyses/MGYA00353320/file/ERR1583083_FASTQ_SSU_OTU.tsv	Root-Environmental-Aquatic-Freshwater-Lake
ERR1582164	MGYA00352401	https://www.ebi.ac.uk/metagenomics/api/v1/analyses/MGYA00352401/file/ERR1582164_FASTQ_SSU_OTU.tsv	Root-Environmental-Aquatic-Freshwater-Lake

ERR1333518	MGYA00107596	https://www.ebi.ac.uk/metagenomics/api/v1/analyses/MGYA00107596/file/ERR1333518_FASTQ_otu.tsv	Root-Environmental-Aquatic-Freshwater-Lake
ERR1582388	MGYA00352625	https://www.ebi.ac.uk/metagenomics/api/v1/analyses/MGYA00352625/file/ERR1582388_FASTQ_SSU_OTU.tsv	Root-Environmental-Aquatic-Freshwater-Lake
ERR1896784	MGYA00350729	https://www.ebi.ac.uk/metagenomics/api/v1/analyses/MGYA00350729/file/ERR1896784_FASTQ_SSU_OTU.tsv	Root-Environmental-Aquatic-Freshwater-Lake
ERR1897663	MGYA00351608	https://www.ebi.ac.uk/metagenomics/api/v1/analyses/MGYA00351608/file/ERR1897663_FASTQ_SSU_OTU.tsv	Root-Environmental-Aquatic-Freshwater-Lake
ERR1897202	MGYA00351147	https://www.ebi.ac.uk/metagenomics/api/v1/analyses/MGYA00351147/file/ERR1897202_FASTQ_SSU_OTU.tsv	Root-Environmental-Aquatic-Freshwater-Lake
ERR1896830	MGYA00350775	https://www.ebi.ac.uk/metagenomics/api/v1/analyses/MGYA00350775/file/ERR1896830_FASTQ_SSU_OTU.tsv	Root-Environmental-Aquatic-Freshwater-Lake
ERR1547038	MGYA00320749	https://www.ebi.ac.uk/metagenomics/api/v1/analyses/MGYA00320749/file/ERR1547038_FASTQ_SSU_OTU.tsv	Root-Environmental-Aquatic-Freshwater-Lake
ERR1897574	MGYA00351519	https://www.ebi.ac.uk/metagenomics/api/v1/analyses/MGYA00351519/file/ERR1897574_FASTQ_SSU_OTU.tsv	Root-Environmental-Aquatic-Freshwater-Lake
ERR1583178	MGYA00353415	https://www.ebi.ac.uk/metagenomics/api/v1/analyses/MGYA00353415/file/ERR1583178_FASTQ_SSU_OTU.tsv	Root-Environmental-Aquatic-Freshwater-Lake
ERR1582738	MGYA00352975	https://www.ebi.ac.uk/metagenomics/api/v1/analyses/MGYA00352975/file/ERR1582738_FASTQ_SSU_OTU.tsv	Root-Environmental-Aquatic-Freshwater-Lake
ERR1897115	MGYA00351060	https://www.ebi.ac.uk/metagenomics/api/v1/analyses/MGYA00351060/file/ERR1897115_FASTQ_SSU_OTU.tsv	Root-Environmental-Aquatic-Freshwater-Lake
ERR1896755	MGYA00350700	https://www.ebi.ac.uk/metagenomics/api/v1/analyses/MGYA00350700/file/ERR1896755_FASTQ_SSU_OTU.tsv	Root-Environmental-Aquatic-Freshwater-Lake

ERR1582359	MGYA00352596	https://www.ebi.ac.uk/metagenomics/api/v1/analyses/MGYA00352596/file/ERR1582359_FASTQ_SSU_OTU.tsv	Root-Environmental-Aquatic-Freshwater-Lake
ERR1583519	MGYA00353756	https://www.ebi.ac.uk/metagenomics/api/v1/analyses/MGYA00353756/file/ERR1583519_FASTQ_SSU_OTU.tsv	Root-Environmental-Aquatic-Freshwater-Lake
ERR1583020	MGYA00353257	https://www.ebi.ac.uk/metagenomics/api/v1/analyses/MGYA00353257/file/ERR1583020_FASTQ_SSU_OTU.tsv	Root-Environmental-Aquatic-Freshwater-Lake
ERR1582660	MGYA00352897	https://www.ebi.ac.uk/metagenomics/api/v1/analyses/MGYA00352897/file/ERR1582660_FASTQ_SSU_OTU.tsv	Root-Environmental-Aquatic-Freshwater-Lake
ERR1582201	MGYA00352438	https://www.ebi.ac.uk/metagenomics/api/v1/analyses/MGYA00352438/file/ERR1582201_FASTQ_SSU_OTU.tsv	Root-Environmental-Aquatic-Freshwater-Lake
ERR1583441	MGYA00353678	https://www.ebi.ac.uk/metagenomics/api/v1/analyses/MGYA00353678/file/ERR1583441_FASTQ_SSU_OTU.tsv	Root-Environmental-Aquatic-Freshwater-Lake
ERR1583490	MGYA00353727	https://www.ebi.ac.uk/metagenomics/api/v1/analyses/MGYA00353727/file/ERR1583490_FASTQ_SSU_OTU.tsv	Root-Environmental-Aquatic-Freshwater-Lake
ERR1583337	MGYA00353574	https://www.ebi.ac.uk/metagenomics/api/v1/analyses/MGYA00353574/file/ERR1583337_FASTQ_SSU_OTU.tsv	Root-Environmental-Aquatic-Freshwater-Lake
ERR1582577	MGYA00352814	https://www.ebi.ac.uk/metagenomics/api/v1/analyses/MGYA00352814/file/ERR1582577_FASTQ_SSU_OTU.tsv	Root-Environmental-Aquatic-Freshwater-Lake
ERR1582905	MGYA00353142	https://www.ebi.ac.uk/metagenomics/api/v1/analyses/MGYA00353142/file/ERR1582905_FASTQ_SSU_OTU.tsv	Root-Environmental-Aquatic-Freshwater-Lake
ERR1896968	MGYA00350913	https://www.ebi.ac.uk/metagenomics/api/v1/analyses/MGYA00350913/file/ERR1896968_FASTQ_SSU_OTU.tsv	Root-Environmental-Aquatic-Freshwater-Lake
ERR1582116	MGYA00352353	https://www.ebi.ac.uk/metagenomics/api/v1/analyses/MGYA00352353/file/ERR1582116_FASTQ_SSU_OTU.tsv	Root-Environmental-Aquatic-Freshwater-Lake

ERR1583213	MGYA00353450	https://www.ebi.ac.uk/metagenomics/api/v1/analyses/MGYA00353450/file/ERR1583213_FASTQ_SSU_OTU.tsv	Root-Environmental-Aquatic-Freshwater-Lake
ERR1582821	MGYA00353058	https://www.ebi.ac.uk/metagenomics/api/v1/analyses/MGYA00353058/file/ERR1582821_FASTQ_SSU_OTU.tsv	Root-Environmental-Aquatic-Freshwater-Lake
ERR1582453	MGYA00352690	https://www.ebi.ac.uk/metagenomics/api/v1/analyses/MGYA00352690/file/ERR1582453_FASTQ_SSU_OTU.tsv	Root-Environmental-Aquatic-Freshwater-Lake
ERR1547095	MGYA00320806	https://www.ebi.ac.uk/metagenomics/api/v1/analyses/MGYA00320806/file/ERR1547095_FASTQ_SSU_OTU.tsv	Root-Environmental-Aquatic-Freshwater-Lake
ERR1582032	MGYA00352269	https://www.ebi.ac.uk/metagenomics/api/v1/analyses/MGYA00352269/file/ERR1582032_FASTQ_SSU_OTU.tsv	Root-Environmental-Aquatic-Freshwater-Lake
ERR1582795	MGYA00353032	https://www.ebi.ac.uk/metagenomics/api/v1/analyses/MGYA00353032/file/ERR1582795_FASTQ_SSU_OTU.tsv	Root-Environmental-Aquatic-Freshwater-Lake
ERR1314352	MGYA00095229	https://www.ebi.ac.uk/metagenomics/api/v1/analyses/MGYA00095229/file/ERR1314352_MERGED_FASTQ_otu.tsv	Root-Environmental-Aquatic-Freshwater-Lake
ERR1583104	MGYA00353341	https://www.ebi.ac.uk/metagenomics/api/v1/analyses/MGYA00353341/file/ERR1583104_FASTQ_SSU_OTU.tsv	Root-Environmental-Aquatic-Freshwater-Lake
ERR1314383	MGYA00095169	https://www.ebi.ac.uk/metagenomics/api/v1/analyses/MGYA00095169/file/ERR1314383_MERGED_FASTQ_otu.tsv	Root-Environmental-Aquatic-Freshwater-Lake
ERR1582744	MGYA00352981	https://www.ebi.ac.uk/metagenomics/api/v1/analyses/MGYA00352981/file/ERR1582744_FASTQ_SSU_OTU.tsv	Root-Environmental-Aquatic-Freshwater-Lake
ERR1547044	MGYA00320755	https://www.ebi.ac.uk/metagenomics/api/v1/analyses/MGYA00320755/file/ERR1547044_FASTQ_SSU_OTU.tsv	Root-Environmental-Aquatic-Freshwater-Lake
ERR1897508	MGYA00351453	https://www.ebi.ac.uk/metagenomics/api/v1/analyses/MGYA00351453/file/ERR1897508_FASTQ_SSU_OTU.tsv	Root-Environmental-Aquatic-Freshwater-Lake

ERR1582482	MGYA00352719	https://www.ebi.ac.uk/metagenomics/api/v1/analyses/MGYA00352719/file/ERR1582482_FASTQ_SSU_OTU.tsv	Root-Environmental-Aquatic-Freshwater-Lake
ERR1582325	MGYA00352562	https://www.ebi.ac.uk/metagenomics/api/v1/analyses/MGYA00352562/file/ERR1582325_FASTQ_SSU_OTU.tsv	Root-Environmental-Aquatic-Freshwater-Lake
ERR1897169	MGYA00351114	https://www.ebi.ac.uk/metagenomics/api/v1/analyses/MGYA00351114/file/ERR1897169_FASTQ_SSU_OTU.tsv	Root-Environmental-Aquatic-Freshwater-Lake
ERR1896729	MGYA00350674	https://www.ebi.ac.uk/metagenomics/api/v1/analyses/MGYA00350674/file/ERR1896729_FASTQ_SSU_OTU.tsv	Root-Environmental-Aquatic-Freshwater-Lake
ERR1743323	MGYA00087208	https://www.ebi.ac.uk/metagenomics/api/v1/analyses/MGYA00087208/file/ERR1743323_MERGED_FASTQ_otu.tsv	Root-Environmental-Aquatic-Freshwater-Lake
ERR1897450	MGYA00351395	https://www.ebi.ac.uk/metagenomics/api/v1/analyses/MGYA00351395/file/ERR1897450_FASTQ_SSU_OTU.tsv	Root-Environmental-Aquatic-Freshwater-Lake
ERR1897031	MGYA00350976	https://www.ebi.ac.uk/metagenomics/api/v1/analyses/MGYA00350976/file/ERR1897031_FASTQ_SSU_OTU.tsv	Root-Environmental-Aquatic-Freshwater-Lake
ERR1896671	MGYA00350616	https://www.ebi.ac.uk/metagenomics/api/v1/analyses/MGYA00350616/file/ERR1896671_FASTQ_SSU_OTU.tsv	Root-Environmental-Aquatic-Freshwater-Lake
ERR1582979	MGYA00353216	https://www.ebi.ac.uk/metagenomics/api/v1/analyses/MGYA00353216/file/ERR1582979_FASTQ_SSU_OTU.tsv	Root-Environmental-Aquatic-Freshwater-Lake
SRR2443003	MGYA00359007	https://www.ebi.ac.uk/metagenomics/api/v1/analyses/MGYA00359007/file/SRR2443003_MERGED_FASTQ_SSU_OTU.tsv	Root-Environmental-Aquatic-Freshwater-Lake
ERR1897747	MGYA00351692	https://www.ebi.ac.uk/metagenomics/api/v1/analyses/MGYA00351692/file/ERR1897747_FASTQ_SSU_OTU.tsv	Root-Environmental-Aquatic-Freshwater-Lake
ERR1897481	MGYA00351426	https://www.ebi.ac.uk/metagenomics/api/v1/analyses/MGYA00351426/file/ERR1897481_FASTQ_SSU_OTU.tsv	Root-Environmental-Aquatic-Freshwater-Lake

ERR1897326	MGYA00351271	https://www.ebi.ac.uk/metagenomics/api/v1/analyses/MGYA00351271/file/ERR1897326_FASTQ_SSU_OTU.tsv	Root-Environmental-Aquatic-Freshwater-Lake
ERR1896914	MGYA00350859	https://www.ebi.ac.uk/metagenomics/api/v1/analyses/MGYA00350859/file/ERR1896914_FASTQ_SSU_OTU.tsv	Root-Environmental-Aquatic-Freshwater-Lake
ERR1897618	MGYA00351563	https://www.ebi.ac.uk/metagenomics/api/v1/analyses/MGYA00351563/file/ERR1897618_FASTQ_SSU_OTU.tsv	Root-Environmental-Aquatic-Freshwater-Lake
ERR1582454	MGYA00352691	https://www.ebi.ac.uk/metagenomics/api/v1/analyses/MGYA00352691/file/ERR1582454_FASTQ_SSU_OTU.tsv	Root-Environmental-Aquatic-Freshwater-Lake
ERR1582826	MGYA00353063	https://www.ebi.ac.uk/metagenomics/api/v1/analyses/MGYA00353063/file/ERR1582826_FASTQ_SSU_OTU.tsv	Root-Environmental-Aquatic-Freshwater-Lake
ERR1583214	MGYA00353451	https://www.ebi.ac.uk/metagenomics/api/v1/analyses/MGYA00353451/file/ERR1583214_FASTQ_SSU_OTU.tsv	Root-Environmental-Aquatic-Freshwater-Lake
ERR1547092	MGYA00320803	https://www.ebi.ac.uk/metagenomics/api/v1/analyses/MGYA00320803/file/ERR1547092_FASTQ_SSU_OTU.tsv	Root-Environmental-Aquatic-Freshwater-Lake
ERR1897279	MGYA00351224	https://www.ebi.ac.uk/metagenomics/api/v1/analyses/MGYA00351224/file/ERR1897279_FASTQ_SSU_OTU.tsv	Root-Environmental-Aquatic-Freshwater-Lake
ERR1843312	MGYA00102753	https://www.ebi.ac.uk/metagenomics/api/v1/analyses/MGYA00102753/file/ERR1843312_FASTQ_otu.tsv	Root-Environmental-Aquatic-Freshwater-Lake
ERR1582792	MGYA00353029	https://www.ebi.ac.uk/metagenomics/api/v1/analyses/MGYA00353029/file/ERR1582792_FASTQ_SSU_OTU.tsv	Root-Environmental-Aquatic-Freshwater-Lake
ERR1314355	MGYA00095166	https://www.ebi.ac.uk/metagenomics/api/v1/analyses/MGYA00095166/file/ERR1314355_MERGED_FASTQ_otu.tsv	Root-Environmental-Aquatic-Freshwater-Lake
ERR1582035	MGYA00352272	https://www.ebi.ac.uk/metagenomics/api/v1/analyses/MGYA00352272/file/ERR1582035_FASTQ_SSU_OTU.tsv	Root-Environmental-Aquatic-Freshwater-Lake

ERR1314384	MGYA00095149	https://www.ebi.ac.uk/metagenomics/api/v1/analyses/MGYA00095149/file/ERR1314384_MERGED_FASTQ_otu.tsv	Root-Environmental-Aquatic-Freshwater-Lake
ERR1582743	MGYA00352980	https://www.ebi.ac.uk/metagenomics/api/v1/analyses/MGYA00352980/file/ERR1582743_FASTQ_SSU_OTU.tsv	Root-Environmental-Aquatic-Freshwater-Lake
ERR1583103	MGYA00353340	https://www.ebi.ac.uk/metagenomics/api/v1/analyses/MGYA00353340/file/ERR1583103_FASTQ_SSU_OTU.tsv	Root-Environmental-Aquatic-Freshwater-Lake
ERR1547043	MGYA00320754	https://www.ebi.ac.uk/metagenomics/api/v1/analyses/MGYA00320754/file/ERR1547043_FASTQ_SSU_OTU.tsv	Root-Environmental-Aquatic-Freshwater-Lake
ERR1582322	MGYA00352559	https://www.ebi.ac.uk/metagenomics/api/v1/analyses/MGYA00352559/file/ERR1582322_FASTQ_SSU_OTU.tsv	Root-Environmental-Aquatic-Freshwater-Lake
ERR1582485	MGYA00352722	https://www.ebi.ac.uk/metagenomics/api/v1/analyses/MGYA00352722/file/ERR1582485_FASTQ_SSU_OTU.tsv	Root-Environmental-Aquatic-Freshwater-Lake
ERR1897457	MGYA00351402	https://www.ebi.ac.uk/metagenomics/api/v1/analyses/MGYA00351402/file/ERR1897457_FASTQ_SSU_OTU.tsv	Root-Environmental-Aquatic-Freshwater-Lake
ERR1743324	MGYA00087173	https://www.ebi.ac.uk/metagenomics/api/v1/analyses/MGYA00087173/file/ERR1743324_MERGED_FASTQ_otu.tsv	Root-Environmental-Aquatic-Freshwater-Lake
ERR1896676	MGYA00350621	https://www.ebi.ac.uk/metagenomics/api/v1/analyses/MGYA00350621/file/ERR1896676_FASTQ_SSU_OTU.tsv	Root-Environmental-Aquatic-Freshwater-Lake
ERR1897036	MGYA00350981	https://www.ebi.ac.uk/metagenomics/api/v1/analyses/MGYA00350981/file/ERR1897036_FASTQ_SSU_OTU.tsv	Root-Environmental-Aquatic-Freshwater-Lake
SRR2443004	MGYA00359008	https://www.ebi.ac.uk/metagenomics/api/v1/analyses/MGYA00359008/file/SRR2443004_MERGED_FASTQ_SSU_OTU.tsv	Root-Environmental-Aquatic-Freshwater-Lake
ERR1897740	MGYA00351685	https://www.ebi.ac.uk/metagenomics/api/v1/analyses/MGYA00351685/file/ERR1897740_FASTQ_SSU_OTU.tsv	Root-Environmental-Aquatic-Freshwater-Lake

ERR1896913	MGYA00350858	https://www.ebi.ac.uk/metagenomics/api/v1/analyses/MGYA00350858/file/ERR1896913_FASTQ_SSU_OTU.tsv	Root-Environmental-Aquatic-Freshwater-Lake
ERR1897321	MGYA00351266	https://www.ebi.ac.uk/metagenomics/api/v1/analyses/MGYA00351266/file/ERR1897321_FASTQ_SSU_OTU.tsv	Root-Environmental-Aquatic-Freshwater-Lake
ERR1897486	MGYA00351431	https://www.ebi.ac.uk/metagenomics/api/v1/analyses/MGYA00351431/file/ERR1897486_FASTQ_SSU_OTU.tsv	Root-Environmental-Aquatic-Freshwater-Lake
ERR658176	MGYA00103477	https://www.ebi.ac.uk/metagenomics/api/v1/analyses/MGYA00103477/file/ERR658176_FASTQ_otu.tsv	Root-Environmental-Aquatic-Freshwater-Lake
ERR1582428	MGYA00352665	https://www.ebi.ac.uk/metagenomics/api/v1/analyses/MGYA00352665/file/ERR1582428_FASTQ_SSU_OTU.tsv	Root-Environmental-Aquatic-Freshwater-Lake
ERR1583268	MGYA00353505	https://www.ebi.ac.uk/metagenomics/api/v1/analyses/MGYA00353505/file/ERR1583268_FASTQ_SSU_OTU.tsv	Root-Environmental-Aquatic-Freshwater-Lake
ERR1897664	MGYA00351609	https://www.ebi.ac.uk/metagenomics/api/v1/analyses/MGYA00351609/file/ERR1897664_FASTQ_SSU_OTU.tsv	Root-Environmental-Aquatic-Freshwater-Lake
ERR1896783	MGYA00350728	https://www.ebi.ac.uk/metagenomics/api/v1/analyses/MGYA00350728/file/ERR1896783_FASTQ_SSU_OTU.tsv	Root-Environmental-Aquatic-Freshwater-Lake
ERR1535948	MGYA00320239	https://www.ebi.ac.uk/metagenomics/api/v1/analyses/MGYA00320239/file/ERR1535948_FASTQ_SSU_OTU.tsv	Root-Environmental-Aquatic-Freshwater-Lake
ERR1582049	MGYA00352286	https://www.ebi.ac.uk/metagenomics/api/v1/analyses/MGYA00352286/file/ERR1582049_FASTQ_SSU_OTU.tsv	Root-Environmental-Aquatic-Freshwater-Lake
ERR1314329	MGYA00095222	https://www.ebi.ac.uk/metagenomics/api/v1/analyses/MGYA00095222/file/ERR1314329_MERGED_FASTQ_otu.tsv	Root-Environmental-Aquatic-Freshwater-Lake
ERR1896837	MGYA00350782	https://www.ebi.ac.uk/metagenomics/api/v1/analyses/MGYA00350782/file/ERR1896837_FASTQ_SSU_OTU.tsv	Root-Environmental-Aquatic-Freshwater-Lake

ERR1897205	MGYA00351150	https://www.ebi.ac.uk/metagenomics/api/v1/analyses/MGYA00351150/file/ERR1897205_FASTQ_SSU_OTU.tsv	Root-Environmental-Aquatic-Freshwater-Lake
ERR1897573	MGYA00351518	https://www.ebi.ac.uk/metagenomics/api/v1/analyses/MGYA00351518/file/ERR1897573_FASTQ_SSU_OTU.tsv	Root-Environmental-Aquatic-Freshwater-Lake
ERR1582098	MGYA00352335	https://www.ebi.ac.uk/metagenomics/api/v1/analyses/MGYA00352335/file/ERR1582098_FASTQ_SSU_OTU.tsv	Root-Environmental-Aquatic-Freshwater-Lake
ERR1896752	MGYA00350697	https://www.ebi.ac.uk/metagenomics/api/v1/analyses/MGYA00350697/file/ERR1896752_FASTQ_SSU_OTU.tsv	Root-Environmental-Aquatic-Freshwater-Lake
ERR1897112	MGYA00351057	https://www.ebi.ac.uk/metagenomics/api/v1/analyses/MGYA00351057/file/ERR1897112_FASTQ_SSU_OTU.tsv	Root-Environmental-Aquatic-Freshwater-Lake
ERR1582667	MGYA00352904	https://www.ebi.ac.uk/metagenomics/api/v1/analyses/MGYA00352904/file/ERR1582667_FASTQ_SSU_OTU.tsv	Root-Environmental-Aquatic-Freshwater-Lake
ERR1583027	MGYA00353264	https://www.ebi.ac.uk/metagenomics/api/v1/analyses/MGYA00353264/file/ERR1583027_FASTQ_SSU_OTU.tsv	Root-Environmental-Aquatic-Freshwater-Lake
SRR7081230	MGYA00425156	https://www.ebi.ac.uk/metagenomics/api/v1/analyses/MGYA00425156/file/SRR7081230_MERGED_FASTQ_SSU_OTU.tsv	Root-Environmental-Aquatic-Freshwater-Lake
ERR1743358	MGYA00087191	https://www.ebi.ac.uk/metagenomics/api/v1/analyses/MGYA00087191/file/ERR1743358_MERGED_FASTQ_otu.tsv	Root-Environmental-Aquatic-Freshwater-Lake
ERR1583446	MGYA00353683	https://www.ebi.ac.uk/metagenomics/api/v1/analyses/MGYA00353683/file/ERR1583446_FASTQ_SSU_OTU.tsv	Root-Environmental-Aquatic-Freshwater-Lake
ERR1582206	MGYA00352443	https://www.ebi.ac.uk/metagenomics/api/v1/analyses/MGYA00352443/file/ERR1582206_FASTQ_SSU_OTU.tsv	Root-Environmental-Aquatic-Freshwater-Lake
ERR1582902	MGYA00353139	https://www.ebi.ac.uk/metagenomics/api/v1/analyses/MGYA00353139/file/ERR1582902_FASTQ_SSU_OTU.tsv	Root-Environmental-Aquatic-Freshwater-Lake

ERR1582570	MGYA00352807	https://www.ebi.ac.uk/metagenomics/api/v1/analyses/MGYA00352807/file/ERR1582570_FASTQ_SSU_OTU.tsv	Root-Environmental-Aquatic-Freshwater-Lake
ERR1583330	MGYA00353567	https://www.ebi.ac.uk/metagenomics/api/v1/analyses/MGYA00353567/file/ERR1583330_FASTQ_SSU_OTU.tsv	Root-Environmental-Aquatic-Freshwater-Lake
ERR1583497	MGYA00353734	https://www.ebi.ac.uk/metagenomics/api/v1/analyses/MGYA00353734/file/ERR1583497_FASTQ_SSU_OTU.tsv	Root-Environmental-Aquatic-Freshwater-Lake
ERR1743389	MGYA00087202	https://www.ebi.ac.uk/metagenomics/api/v1/analyses/MGYA00087202/file/ERR1743389_MERGED_FASTQ_otu.tsv	Root-Environmental-Aquatic-Freshwater-Lake
ERR589328	MGYA00103427	https://www.ebi.ac.uk/metagenomics/api/v1/analyses/MGYA00103427/file/ERR589328_FASTQ_otu.tsv	Root-Environmental-Aquatic-Freshwater-Lake
ERR1582111	MGYA00352348	https://www.ebi.ac.uk/metagenomics/api/v1/analyses/MGYA00352348/file/ERR1582111_FASTQ_SSU_OTU.tsv	Root-Environmental-Aquatic-Freshwater-Lake
SRR2443015	MGYA00359019	https://www.ebi.ac.uk/metagenomics/api/v1/analyses/MGYA00359019/file/SRR2443015_MERGED_FASTQ_SSU_OTU.tsv	Root-Environmental-Aquatic-Freshwater-Lake
ERR1897751	MGYA00351696	https://www.ebi.ac.uk/metagenomics/api/v1/analyses/MGYA00351696/file/ERR1897751_FASTQ_SSU_OTU.tsv	Root-Environmental-Aquatic-Freshwater-Lake
ERR1896902	MGYA00350847	https://www.ebi.ac.uk/metagenomics/api/v1/analyses/MGYA00350847/file/ERR1896902_FASTQ_SSU_OTU.tsv	Root-Environmental-Aquatic-Freshwater-Lake
ERR589345	MGYA00103463	https://www.ebi.ac.uk/metagenomics/api/v1/analyses/MGYA00103463/file/ERR589345_FASTQ_otu.tsv	Root-Environmental-Aquatic-Freshwater-Lake
ERR1897497	MGYA00351442	https://www.ebi.ac.uk/metagenomics/api/v1/analyses/MGYA00351442/file/ERR1897497_FASTQ_SSU_OTU.tsv	Root-Environmental-Aquatic-Freshwater-Lake
ERR1897330	MGYA00351275	https://www.ebi.ac.uk/metagenomics/api/v1/analyses/MGYA00351275/file/ERR1897330_FASTQ_SSU_OTU.tsv	Root-Environmental-Aquatic-Freshwater-Lake

ERR1897446	MGYA00351391	https://www.ebi.ac.uk/metagenomics/api/v1/analyses/MGYA00351391/file/ERR1897446_FASTQ_SSU_OTU.tsv	Root-Environmental-Aquatic-Freshwater-Lake
ERR1743335	MGYA00087169	https://www.ebi.ac.uk/metagenomics/api/v1/analyses/MGYA00087169/file/ERR1743335_MERGED_FASTQ_otu.tsv	Root-Environmental-Aquatic-Freshwater-Lake
ERR1896667	MGYA00350612	https://www.ebi.ac.uk/metagenomics/api/v1/analyses/MGYA00350612/file/ERR1896667_FASTQ_SSU_OTU.tsv	Root-Environmental-Aquatic-Freshwater-Lake
ERR1897027	MGYA00350972	https://www.ebi.ac.uk/metagenomics/api/v1/analyses/MGYA00350972/file/ERR1897027_FASTQ_SSU_OTU.tsv	Root-Environmental-Aquatic-Freshwater-Lake
SRR7081305	MGYA00424840	https://www.ebi.ac.uk/metagenomics/api/v1/analyses/MGYA00424840/file/SRR7081305_MERGED_FASTQ_SSU_OTU.tsv	Root-Environmental-Aquatic-Freshwater-Lake
ERR1547052	MGYA00320763	https://www.ebi.ac.uk/metagenomics/api/v1/analyses/MGYA00320763/file/ERR1547052_FASTQ_SSU_OTU.tsv	Root-Environmental-Aquatic-Freshwater-Lake
ERR1582752	MGYA00352989	https://www.ebi.ac.uk/metagenomics/api/v1/analyses/MGYA00352989/file/ERR1582752_FASTQ_SSU_OTU.tsv	Root-Environmental-Aquatic-Freshwater-Lake
ERR1314395	MGYA00095196	https://www.ebi.ac.uk/metagenomics/api/v1/analyses/MGYA00095196/file/ERR1314395_MERGED_FASTQ_otu.tsv	Root-Environmental-Aquatic-Freshwater-Lake
ERR1583112	MGYA00353349	https://www.ebi.ac.uk/metagenomics/api/v1/analyses/MGYA00353349/file/ERR1583112_FASTQ_SSU_OTU.tsv	Root-Environmental-Aquatic-Freshwater-Lake
ERR1582494	MGYA00352731	https://www.ebi.ac.uk/metagenomics/api/v1/analyses/MGYA00352731/file/ERR1582494_FASTQ_SSU_OTU.tsv	Root-Environmental-Aquatic-Freshwater-Lake
ERR1582333	MGYA00352570	https://www.ebi.ac.uk/metagenomics/api/v1/analyses/MGYA00352570/file/ERR1582333_FASTQ_SSU_OTU.tsv	Root-Environmental-Aquatic-Freshwater-Lake
ERR1582837	MGYA00353074	https://www.ebi.ac.uk/metagenomics/api/v1/analyses/MGYA00353074/file/ERR1582837_FASTQ_SSU_OTU.tsv	Root-Environmental-Aquatic-Freshwater-Lake

ERR1582445	MGYA00352682	https://www.ebi.ac.uk/metagenomics/api/v1/analyses/MGYA00352682/file/ERR1582445_FASTQ_SSU_OTU.tsv	Root-Environmental-Aquatic-Freshwater-Lake
ERR1583205	MGYA00353442	https://www.ebi.ac.uk/metagenomics/api/v1/analyses/MGYA00353442/file/ERR1583205_FASTQ_SSU_OTU.tsv	Root-Environmental-Aquatic-Freshwater-Lake
ERR1897609	MGYA00351554	https://www.ebi.ac.uk/metagenomics/api/v1/analyses/MGYA00351554/file/ERR1897609_FASTQ_SSU_OTU.tsv	Root-Environmental-Aquatic-Freshwater-Lake
ERR1582024	MGYA00352261	https://www.ebi.ac.uk/metagenomics/api/v1/analyses/MGYA00352261/file/ERR1582024_FASTQ_SSU_OTU.tsv	Root-Environmental-Aquatic-Freshwater-Lake
ERR1314344	MGYA00095165	https://www.ebi.ac.uk/metagenomics/api/v1/analyses/MGYA00095165/file/ERR1314344_MERGED_FASTQ_otu.tsv	Root-Environmental-Aquatic-Freshwater-Lake
ERR1582783	MGYA00353020	https://www.ebi.ac.uk/metagenomics/api/v1/analyses/MGYA00353020/file/ERR1582783_FASTQ_SSU_OTU.tsv	Root-Environmental-Aquatic-Freshwater-Lake
ERR1547083	MGYA00320794	https://www.ebi.ac.uk/metagenomics/api/v1/analyses/MGYA00320794/file/ERR1547083_FASTQ_SSU_OTU.tsv	Root-Environmental-Aquatic-Freshwater-Lake
ERR1897268	MGYA00351213	https://www.ebi.ac.uk/metagenomics/api/v1/analyses/MGYA00351213/file/ERR1897268_FASTQ_SSU_OTU.tsv	Root-Environmental-Aquatic-Freshwater-Lake
ERR1582561	MGYA00352798	https://www.ebi.ac.uk/metagenomics/api/v1/analyses/MGYA00352798/file/ERR1582561_FASTQ_SSU_OTU.tsv	Root-Environmental-Aquatic-Freshwater-Lake
ERR1582913	MGYA00353150	https://www.ebi.ac.uk/metagenomics/api/v1/analyses/MGYA00353150/file/ERR1582913_FASTQ_SSU_OTU.tsv	Root-Environmental-Aquatic-Freshwater-Lake
ERR1583486	MGYA00353723	https://www.ebi.ac.uk/metagenomics/api/v1/analyses/MGYA00353723/file/ERR1583486_FASTQ_SSU_OTU.tsv	Root-Environmental-Aquatic-Freshwater-Lake
ERR1583321	MGYA00353558	https://www.ebi.ac.uk/metagenomics/api/v1/analyses/MGYA00353558/file/ERR1583321_FASTQ_SSU_OTU.tsv	Root-Environmental-Aquatic-Freshwater-Lake

ERR1582100	MGYA00352337	https://www.ebi.ac.uk/metagenomics/api/v1/analyses/MGYA00352337/file/ERR1582100_FASTQ_SSU_OTU.tsv	Root-Environmental-Aquatic-Freshwater-Lake
ERR589339	MGYA00103422	https://www.ebi.ac.uk/metagenomics/api/v1/analyses/MGYA00103422/file/ERR589339_FASTQ_otu.tsv	Root-Environmental-Aquatic-Freshwater-Lake
ERR1743349	MGYA00087199	https://www.ebi.ac.uk/metagenomics/api/v1/analyses/MGYA00087199/file/ERR1743349_MERGED_FASTQ_otu.tsv	Root-Environmental-Aquatic-Freshwater-Lake
ERR1582676	MGYA00352913	https://www.ebi.ac.uk/metagenomics/api/v1/analyses/MGYA00352913/file/ERR1582676_FASTQ_SSU_OTU.tsv	Root-Environmental-Aquatic-Freshwater-Lake
ERR1583036	MGYA00353273	https://www.ebi.ac.uk/metagenomics/api/v1/analyses/MGYA00353273/file/ERR1583036_FASTQ_SSU_OTU.tsv	Root-Environmental-Aquatic-Freshwater-Lake
ERR1583457	MGYA00353694	https://www.ebi.ac.uk/metagenomics/api/v1/analyses/MGYA00353694/file/ERR1583457_FASTQ_SSU_OTU.tsv	Root-Environmental-Aquatic-Freshwater-Lake
ERR1582217	MGYA00352454	https://www.ebi.ac.uk/metagenomics/api/v1/analyses/MGYA00352454/file/ERR1582217_FASTQ_SSU_OTU.tsv	Root-Environmental-Aquatic-Freshwater-Lake
ERR1582089	MGYA00352326	https://www.ebi.ac.uk/metagenomics/api/v1/analyses/MGYA00352326/file/ERR1582089_FASTQ_SSU_OTU.tsv	Root-Environmental-Aquatic-Freshwater-Lake
ERR1897562	MGYA00351507	https://www.ebi.ac.uk/metagenomics/api/v1/analyses/MGYA00351507/file/ERR1897562_FASTQ_SSU_OTU.tsv	Root-Environmental-Aquatic-Freshwater-Lake
ERR1535988	MGYA00320279	https://www.ebi.ac.uk/metagenomics/api/v1/analyses/MGYA00320279/file/ERR1535988_FASTQ_SSU_OTU.tsv	Root-Environmental-Aquatic-Freshwater-Lake
ERR1896743	MGYA00350688	https://www.ebi.ac.uk/metagenomics/api/v1/analyses/MGYA00350688/file/ERR1896743_FASTQ_SSU_OTU.tsv	Root-Environmental-Aquatic-Freshwater-Lake
ERR1897103	MGYA00351048	https://www.ebi.ac.uk/metagenomics/api/v1/analyses/MGYA00351048/file/ERR1897103_FASTQ_SSU_OTU.tsv	Root-Environmental-Aquatic-Freshwater-Lake

ERR1897675	MGYA00351620	https://www.ebi.ac.uk/metagenomics/api/v1/analyses/MGYA00351620/file/ERR1897675_FASTQ_SSU_OTU.tsv	Root-Environmental-Aquatic-Freshwater-Lake
ERR1896792	MGYA00350737	https://www.ebi.ac.uk/metagenomics/api/v1/analyses/MGYA00350737/file/ERR1896792_FASTQ_SSU_OTU.tsv	Root-Environmental-Aquatic-Freshwater-Lake
ERR1535959	MGYA00320250	https://www.ebi.ac.uk/metagenomics/api/v1/analyses/MGYA00320250/file/ERR1535959_FASTQ_SSU_OTU.tsv	Root-Environmental-Aquatic-Freshwater-Lake
ERR1582439	MGYA00352676	https://www.ebi.ac.uk/metagenomics/api/v1/analyses/MGYA00352676/file/ERR1582439_FASTQ_SSU_OTU.tsv	Root-Environmental-Aquatic-Freshwater-Lake
ERR1583279	MGYA00353516	https://www.ebi.ac.uk/metagenomics/api/v1/analyses/MGYA00353516/file/ERR1583279_FASTQ_SSU_OTU.tsv	Root-Environmental-Aquatic-Freshwater-Lake
ERR1896826	MGYA00350771	https://www.ebi.ac.uk/metagenomics/api/v1/analyses/MGYA00350771/file/ERR1896826_FASTQ_SSU_OTU.tsv	Root-Environmental-Aquatic-Freshwater-Lake
ERR1897214	MGYA00351159	https://www.ebi.ac.uk/metagenomics/api/v1/analyses/MGYA00351159/file/ERR1897214_FASTQ_SSU_OTU.tsv	Root-Environmental-Aquatic-Freshwater-Lake
ERR1314338	MGYA00095202	https://www.ebi.ac.uk/metagenomics/api/v1/analyses/MGYA00095202/file/ERR1314338_MERGED_FASTQ_otu.tsv	Root-Environmental-Aquatic-Freshwater-Lake
ERR1582058	MGYA00352295	https://www.ebi.ac.uk/metagenomics/api/v1/analyses/MGYA00352295/file/ERR1582058_FASTQ_SSU_OTU.tsv	Root-Environmental-Aquatic-Freshwater-Lake
ERR1583326	MGYA00353563	https://www.ebi.ac.uk/metagenomics/api/v1/analyses/MGYA00353563/file/ERR1583326_FASTQ_SSU_OTU.tsv	Root-Environmental-Aquatic-Freshwater-Lake
ERR1583481	MGYA00353718	https://www.ebi.ac.uk/metagenomics/api/v1/analyses/MGYA00353718/file/ERR1583481_FASTQ_SSU_OTU.tsv	Root-Environmental-Aquatic-Freshwater-Lake
ERR1582914	MGYA00353151	https://www.ebi.ac.uk/metagenomics/api/v1/analyses/MGYA00353151/file/ERR1582914_FASTQ_SSU_OTU.tsv	Root-Environmental-Aquatic-Freshwater-Lake

ERR1582566	MGYA00352803	https://www.ebi.ac.uk/metagenomics/api/v1/analyses/MGYA00352803/file/ERR1582566_FASTQ_SSU_OTU.tsv	Root-Environmental-Aquatic-Freshwater-Lake
ERR1582107	MGYA00352344	https://www.ebi.ac.uk/metagenomics/api/v1/analyses/MGYA00352344/file/ERR1582107_FASTQ_SSU_OTU.tsv	Root-Environmental-Aquatic-Freshwater-Lake
ERR1896979	MGYA00350924	https://www.ebi.ac.uk/metagenomics/api/v1/analyses/MGYA00350924/file/ERR1896979_FASTQ_SSU_OTU.tsv	Root-Environmental-Aquatic-Freshwater-Lake
ERR1583031	MGYA00353268	https://www.ebi.ac.uk/metagenomics/api/v1/analyses/MGYA00353268/file/ERR1583031_FASTQ_SSU_OTU.tsv	Root-Environmental-Aquatic-Freshwater-Lake
ERR1582671	MGYA00352908	https://www.ebi.ac.uk/metagenomics/api/v1/analyses/MGYA00352908/file/ERR1582671_FASTQ_SSU_OTU.tsv	Root-Environmental-Aquatic-Freshwater-Lake
ERR1582210	MGYA00352447	https://www.ebi.ac.uk/metagenomics/api/v1/analyses/MGYA00352447/file/ERR1582210_FASTQ_SSU_OTU.tsv	Root-Environmental-Aquatic-Freshwater-Lake
ERR1583450	MGYA00353687	https://www.ebi.ac.uk/metagenomics/api/v1/analyses/MGYA00353687/file/ERR1583450_FASTQ_SSU_OTU.tsv	Root-Environmental-Aquatic-Freshwater-Lake
ERR1583169	MGYA00353406	https://www.ebi.ac.uk/metagenomics/api/v1/analyses/MGYA00353406/file/ERR1583169_FASTQ_SSU_OTU.tsv	Root-Environmental-Aquatic-Freshwater-Lake
ERR1582729	MGYA00352966	https://www.ebi.ac.uk/metagenomics/api/v1/analyses/MGYA00352966/file/ERR1582729_FASTQ_SSU_OTU.tsv	Root-Environmental-Aquatic-Freshwater-Lake
ERR1897565	MGYA00351510	https://www.ebi.ac.uk/metagenomics/api/v1/analyses/MGYA00351510/file/ERR1897565_FASTQ_SSU_OTU.tsv	Root-Environmental-Aquatic-Freshwater-Lake
ERR1582348	MGYA00352585	https://www.ebi.ac.uk/metagenomics/api/v1/analyses/MGYA00352585/file/ERR1582348_FASTQ_SSU_OTU.tsv	Root-Environmental-Aquatic-Freshwater-Lake
ERR1583508	MGYA00353745	https://www.ebi.ac.uk/metagenomics/api/v1/analyses/MGYA00353745/file/ERR1583508_FASTQ_SSU_OTU.tsv	Root-Environmental-Aquatic-Freshwater-Lake

ERR1897104	MGYA00351049	https://www.ebi.ac.uk/metagenomics/api/v1/analyses/MGYA00351049/file/ERR1897104_FASTQ_SSU_OTU.tsv	Root-Environmental-Aquatic-Freshwater-Lake
ERR1896744	MGYA00350689	https://www.ebi.ac.uk/metagenomics/api/v1/analyses/MGYA00350689/file/ERR1896744_FASTQ_SSU_OTU.tsv	Root-Environmental-Aquatic-Freshwater-Lake
ERR1742278	MGYA00311579	https://www.ebi.ac.uk/metagenomics/api/v1/analyses/MGYA00311579/file/ERR1742278_FASTQ_SSU_OTU.tsv	Root-Environmental-Aquatic-Freshwater-Lake
ERR1896795	MGYA00350740	https://www.ebi.ac.uk/metagenomics/api/v1/analyses/MGYA00350740/file/ERR1896795_FASTQ_SSU_OTU.tsv	Root-Environmental-Aquatic-Freshwater-Lake
ERR1897672	MGYA00351617	https://www.ebi.ac.uk/metagenomics/api/v1/analyses/MGYA00351617/file/ERR1897672_FASTQ_SSU_OTU.tsv	Root-Environmental-Aquatic-Freshwater-Lake
ERR1333509	MGYA00107595	https://www.ebi.ac.uk/metagenomics/api/v1/analyses/MGYA00107595/file/ERR1333509_FASTQ_otu.tsv	Root-Environmental-Aquatic-Freshwater-Lake
ERR1582399	MGYA00352636	https://www.ebi.ac.uk/metagenomics/api/v1/analyses/MGYA00352636/file/ERR1582399_FASTQ_SSU_OTU.tsv	Root-Environmental-Aquatic-Freshwater-Lake
ERR1897213	MGYA00351158	https://www.ebi.ac.uk/metagenomics/api/v1/analyses/MGYA00351158/file/ERR1897213_FASTQ_SSU_OTU.tsv	Root-Environmental-Aquatic-Freshwater-Lake
ERR1896821	MGYA00350766	https://www.ebi.ac.uk/metagenomics/api/v1/analyses/MGYA00350766/file/ERR1896821_FASTQ_SSU_OTU.tsv	Root-Environmental-Aquatic-Freshwater-Lake
SRR2443012	MGYA00359016	https://www.ebi.ac.uk/metagenomics/api/v1/analyses/MGYA00359016/file/SRR2443012_MERGED_FASTQ_SSU_OTU.tsv	Root-Environmental-Aquatic-Freshwater-Lake
ERR1897756	MGYA00351701	https://www.ebi.ac.uk/metagenomics/api/v1/analyses/MGYA00351701/file/ERR1897756_FASTQ_SSU_OTU.tsv	Root-Environmental-Aquatic-Freshwater-Lake
ERR1582968	MGYA00353205	https://www.ebi.ac.uk/metagenomics/api/v1/analyses/MGYA00353205/file/ERR1582968_FASTQ_SSU_OTU.tsv	Root-Environmental-Aquatic-Freshwater-Lake

ERR1897337	MGYA00351282	https://www.ebi.ac.uk/metagenomics/api/v1/analyses/MGYA00351282/file/ERR1897337_FASTQ_SSU_OTU.tsv	Root-Environmental-Aquatic-Freshwater-Lake
ERR1897490	MGYA00351435	https://www.ebi.ac.uk/metagenomics/api/v1/analyses/MGYA00351435/file/ERR1897490_FASTQ_SSU_OTU.tsv	Root-Environmental-Aquatic-Freshwater-Lake
ERR589342	MGYA00103417	https://www.ebi.ac.uk/metagenomics/api/v1/analyses/MGYA00103417/file/ERR589342_FASTQ_otu.tsv	Root-Environmental-Aquatic-Freshwater-Lake
ERR1896905	MGYA00350850	https://www.ebi.ac.uk/metagenomics/api/v1/analyses/MGYA00350850/file/ERR1896905_FASTQ_SSU_OTU.tsv	Root-Environmental-Aquatic-Freshwater-Lake
ERR1743332	MGYA00087143	https://www.ebi.ac.uk/metagenomics/api/v1/analyses/MGYA00087143/file/ERR1743332_MERGED_FASTQ_otu.tsv	Root-Environmental-Aquatic-Freshwater-Lake
ERR1897441	MGYA00351386	https://www.ebi.ac.uk/metagenomics/api/v1/analyses/MGYA00351386/file/ERR1897441_FASTQ_SSU_OTU.tsv	Root-Environmental-Aquatic-Freshwater-Lake
ERR1897020	MGYA00350965	https://www.ebi.ac.uk/metagenomics/api/v1/analyses/MGYA00350965/file/ERR1897020_FASTQ_SSU_OTU.tsv	Root-Environmental-Aquatic-Freshwater-Lake
ERR1896660	MGYA00350605	https://www.ebi.ac.uk/metagenomics/api/v1/analyses/MGYA00350605/file/ERR1896660_FASTQ_SSU_OTU.tsv	Root-Environmental-Aquatic-Freshwater-Lake
ERR1547055	MGYA00320766	https://www.ebi.ac.uk/metagenomics/api/v1/analyses/MGYA00320766/file/ERR1547055_FASTQ_SSU_OTU.tsv	Root-Environmental-Aquatic-Freshwater-Lake
SRR7081302	MGYA00421988	https://www.ebi.ac.uk/metagenomics/api/v1/analyses/MGYA00421988/file/SRR7081302_MERGED_FASTQ_SSU_OTU.tsv	Root-Environmental-Aquatic-Freshwater-Lake
ERR1897519	MGYA00351464	https://www.ebi.ac.uk/metagenomics/api/v1/analyses/MGYA00351464/file/ERR1897519_FASTQ_SSU_OTU.tsv	Root-Environmental-Aquatic-Freshwater-Lake
ERR1583115	MGYA00353352	https://www.ebi.ac.uk/metagenomics/api/v1/analyses/MGYA00353352/file/ERR1583115_FASTQ_SSU_OTU.tsv	Root-Environmental-Aquatic-Freshwater-Lake

ERR1582755	MGYA00352992	https://www.ebi.ac.uk/metagenomics/api/v1/analyses/MGYA00352992/file/ERR1582755_FASTQ_SSU_OTU.tsv	Root-Environmental-Aquatic-Freshwater-Lake
ERR1314392	MGYA00095176	https://www.ebi.ac.uk/metagenomics/api/v1/analyses/MGYA00095176/file/ERR1314392_MERGED_FASTQ_otu.tsv	Root-Environmental-Aquatic-Freshwater-Lake
ERR1897178	MGYA00351123	https://www.ebi.ac.uk/metagenomics/api/v1/analyses/MGYA00351123/file/ERR1897178_FASTQ_SSU_OTU.tsv	Root-Environmental-Aquatic-Freshwater-Lake
ERR1896738	MGYA00350683	https://www.ebi.ac.uk/metagenomics/api/v1/analyses/MGYA00350683/file/ERR1896738_FASTQ_SSU_OTU.tsv	Root-Environmental-Aquatic-Freshwater-Lake
ERR1582334	MGYA00352571	https://www.ebi.ac.uk/metagenomics/api/v1/analyses/MGYA00352571/file/ERR1582334_FASTQ_SSU_OTU.tsv	Root-Environmental-Aquatic-Freshwater-Lake
ERR1582493	MGYA00352730	https://www.ebi.ac.uk/metagenomics/api/v1/analyses/MGYA00352730/file/ERR1582493_FASTQ_SSU_OTU.tsv	Root-Environmental-Aquatic-Freshwater-Lake
ERR1583202	MGYA00353439	https://www.ebi.ac.uk/metagenomics/api/v1/analyses/MGYA00353439/file/ERR1583202_FASTQ_SSU_OTU.tsv	Root-Environmental-Aquatic-Freshwater-Lake
ERR1582442	MGYA00352679	https://www.ebi.ac.uk/metagenomics/api/v1/analyses/MGYA00352679/file/ERR1582442_FASTQ_SSU_OTU.tsv	Root-Environmental-Aquatic-Freshwater-Lake
ERR1582830	MGYA00353067	https://www.ebi.ac.uk/metagenomics/api/v1/analyses/MGYA00353067/file/ERR1582830_FASTQ_SSU_OTU.tsv	Root-Environmental-Aquatic-Freshwater-Lake
ERR1314343	MGYA00095182	https://www.ebi.ac.uk/metagenomics/api/v1/analyses/MGYA00095182/file/ERR1314343_MERGED_FASTQ_otu.tsv	Root-Environmental-Aquatic-Freshwater-Lake
ERR1582784	MGYA00353021	https://www.ebi.ac.uk/metagenomics/api/v1/analyses/MGYA00353021/file/ERR1582784_FASTQ_SSU_OTU.tsv	Root-Environmental-Aquatic-Freshwater-Lake
ERR1582023	MGYA00352260	https://www.ebi.ac.uk/metagenomics/api/v1/analyses/MGYA00352260/file/ERR1582023_FASTQ_SSU_OTU.tsv	Root-Environmental-Aquatic-Freshwater-Lake

ERR1547084	MGYA00320795	https://www.ebi.ac.uk/metagenomics/api/v1/analyses/MGYA00320795/file/ERR1547084_FASTQ_SSU_OTU.tsv	Root-Environmental-Aquatic-Freshwater-Lake
ERR1897083	MGYA00351028	https://www.ebi.ac.uk/metagenomics/api/v1/analyses/MGYA00351028/file/ERR1897083_FASTQ_SSU_OTU.tsv	Root-Environmental-Aquatic-Freshwater-Lake
ERR1897724	MGYA00351669	https://www.ebi.ac.uk/metagenomics/api/v1/analyses/MGYA00351669/file/ERR1897724_FASTQ_SSU_OTU.tsv	Root-Environmental-Aquatic-Freshwater-Lake
ERR1582568	MGYA00352805	https://www.ebi.ac.uk/metagenomics/api/v1/analyses/MGYA00352805/file/ERR1582568_FASTQ_SSU_OTU.tsv	Root-Environmental-Aquatic-Freshwater-Lake
ERR1583328	MGYA00353565	https://www.ebi.ac.uk/metagenomics/api/v1/analyses/MGYA00353565/file/ERR1583328_FASTQ_SSU_OTU.tsv	Root-Environmental-Aquatic-Freshwater-Lake
ERR1743391	MGYA00087193	https://www.ebi.ac.uk/metagenomics/api/v1/analyses/MGYA00087193/file/ERR1743391_MERGED_FASTQ_otu.tsv	Root-Environmental-Aquatic-Freshwater-Lake
SRR2442994	MGYA00358998	https://www.ebi.ac.uk/metagenomics/api/v1/analyses/MGYA00358998/file/SRR2442994_MERGED_FASTQ_SSU_OTU.tsv	Root-Environmental-Aquatic-Freshwater-Lake
ERR1896977	MGYA00350922	https://www.ebi.ac.uk/metagenomics/api/v1/analyses/MGYA00350922/file/ERR1896977_FASTQ_SSU_OTU.tsv	Root-Environmental-Aquatic-Freshwater-Lake
ERR589330	MGYA00103428	https://www.ebi.ac.uk/metagenomics/api/v1/analyses/MGYA00103428/file/ERR589330_FASTQ_otu.tsv	Root-Environmental-Aquatic-Freshwater-Lake
ERR1897345	MGYA00351290	https://www.ebi.ac.uk/metagenomics/api/v1/analyses/MGYA00351290/file/ERR1897345_FASTQ_SSU_OTU.tsv	Root-Environmental-Aquatic-Freshwater-Lake
ERR1582109	MGYA00352346	https://www.ebi.ac.uk/metagenomics/api/v1/analyses/MGYA00352346/file/ERR1582109_FASTQ_SSU_OTU.tsv	Root-Environmental-Aquatic-Freshwater-Lake
ERR1897433	MGYA00351378	https://www.ebi.ac.uk/metagenomics/api/v1/analyses/MGYA00351378/file/ERR1897433_FASTQ_SSU_OTU.tsv	Root-Environmental-Aquatic-Freshwater-Lake

ERR1897394	MGYA00351339	https://www.ebi.ac.uk/metagenomics/api/v1/analyses/MGYA00351339/file/ERR1897394_FASTQ_SSU_OTU.tsv	Root-Environmental-Aquatic-Freshwater-Lake
ERR1743340	MGYA00087167	https://www.ebi.ac.uk/metagenomics/api/v1/analyses/MGYA00087167/file/ERR1743340_MERGED_FASTQ_otu.tsv	Root-Environmental-Aquatic-Freshwater-Lake
ERR1897052	MGYA00350997	https://www.ebi.ac.uk/metagenomics/api/v1/analyses/MGYA00350997/file/ERR1897052_FASTQ_SSU_OTU.tsv	Root-Environmental-Aquatic-Freshwater-Lake
ERR1582080	MGYA00352317	https://www.ebi.ac.uk/metagenomics/api/v1/analyses/MGYA00352317/file/ERR1582080_FASTQ_SSU_OTU.tsv	Root-Environmental-Aquatic-Freshwater-Lake
ERR1582727	MGYA00352964	https://www.ebi.ac.uk/metagenomics/api/v1/analyses/MGYA00352964/file/ERR1582727_FASTQ_SSU_OTU.tsv	Root-Environmental-Aquatic-Freshwater-Lake
ERR1583167	MGYA00353404	https://www.ebi.ac.uk/metagenomics/api/v1/analyses/MGYA00353404/file/ERR1583167_FASTQ_SSU_OTU.tsv	Root-Environmental-Aquatic-Freshwater-Lake
ERR1535981	MGYA00320272	https://www.ebi.ac.uk/metagenomics/api/v1/analyses/MGYA00320272/file/ERR1535981_FASTQ_SSU_OTU.tsv	Root-Environmental-Aquatic-Freshwater-Lake
ERR1583506	MGYA00353743	https://www.ebi.ac.uk/metagenomics/api/v1/analyses/MGYA00353743/file/ERR1583506_FASTQ_SSU_OTU.tsv	Root-Environmental-Aquatic-Freshwater-Lake
ERR1582893	MGYA00353130	https://www.ebi.ac.uk/metagenomics/api/v1/analyses/MGYA00353130/file/ERR1582893_FASTQ_SSU_OTU.tsv	Root-Environmental-Aquatic-Freshwater-Lake
ERR1582346	MGYA00352583	https://www.ebi.ac.uk/metagenomics/api/v1/analyses/MGYA00352583/file/ERR1582346_FASTQ_SSU_OTU.tsv	Root-Environmental-Aquatic-Freshwater-Lake
ERR1429263	MGYA00107635	https://www.ebi.ac.uk/metagenomics/api/v1/analyses/MGYA00107635/file/ERR1429263_FASTQ_otu.tsv	Root-Environmental-Aquatic-Freshwater-Lake
ERR1582430	MGYA00352667	https://www.ebi.ac.uk/metagenomics/api/v1/analyses/MGYA00352667/file/ERR1582430_FASTQ_SSU_OTU.tsv	Root-Environmental-Aquatic-Freshwater-Lake

ERR1582842	MGYA00353079	https://www.ebi.ac.uk/metagenomics/api/v1/analyses/MGYA00353079/file/ERR1582842_FASTQ_SSU_OTU.tsv	Root-Environmental-Aquatic-Freshwater-Lake
ERR1582397	MGYA00352634	https://www.ebi.ac.uk/metagenomics/api/v1/analyses/MGYA00352634/file/ERR1582397_FASTQ_SSU_OTU.tsv	Root-Environmental-Aquatic-Freshwater-Lake
ERR1583270	MGYA00353507	https://www.ebi.ac.uk/metagenomics/api/v1/analyses/MGYA00353507/file/ERR1583270_FASTQ_SSU_OTU.tsv	Root-Environmental-Aquatic-Freshwater-Lake
ERR1333507	MGYA00107602	https://www.ebi.ac.uk/metagenomics/api/v1/analyses/MGYA00107602/file/ERR1333507_FASTQ_otu.tsv	Root-Environmental-Aquatic-Freshwater-Lake
ERR1535950	MGYA00320241	https://www.ebi.ac.uk/metagenomics/api/v1/analyses/MGYA00320241/file/ERR1535950_FASTQ_SSU_OTU.tsv	Root-Environmental-Aquatic-Freshwater-Lake
ERR1582051	MGYA00352288	https://www.ebi.ac.uk/metagenomics/api/v1/analyses/MGYA00352288/file/ERR1582051_FASTQ_SSU_OTU.tsv	Root-Environmental-Aquatic-Freshwater-Lake
ERR1314331	MGYA00095191	https://www.ebi.ac.uk/metagenomics/api/v1/analyses/MGYA00095191/file/ERR1314331_MERGED_FASTQ_otu.tsv	Root-Environmental-Aquatic-Freshwater-Lake
ERR1582966	MGYA00353203	https://www.ebi.ac.uk/metagenomics/api/v1/analyses/MGYA00353203/file/ERR1582966_FASTQ_SSU_OTU.tsv	Root-Environmental-Aquatic-Freshwater-Lake
ERR1582514	MGYA00352751	https://www.ebi.ac.uk/metagenomics/api/v1/analyses/MGYA00352751/file/ERR1582514_FASTQ_SSU_OTU.tsv	Root-Environmental-Aquatic-Freshwater-Lake
ERR1583354	MGYA00353591	https://www.ebi.ac.uk/metagenomics/api/v1/analyses/MGYA00353591/file/ERR1583354_FASTQ_SSU_OTU.tsv	Root-Environmental-Aquatic-Freshwater-Lake
ERR1897758	MGYA00351703	https://www.ebi.ac.uk/metagenomics/api/v1/analyses/MGYA00351703/file/ERR1897758_FASTQ_SSU_OTU.tsv	Root-Environmental-Aquatic-Freshwater-Lake
ERR1583092	MGYA00353329	https://www.ebi.ac.uk/metagenomics/api/v1/analyses/MGYA00353329/file/ERR1583092_FASTQ_SSU_OTU.tsv	Root-Environmental-Aquatic-Freshwater-Lake

ERR1582175	MGYA00352412	https://www.ebi.ac.uk/metagenomics/api/v1/analyses/MGYA00352412/file/ERR1582175_FASTQ_SSU_OTU.tsv	Root-Environmental-Aquatic-Freshwater-Lake
ERR1897339	MGYA00351284	https://www.ebi.ac.uk/metagenomics/api/v1/analyses/MGYA00351284/file/ERR1897339_FASTQ_SSU_OTU.tsv	Root-Environmental-Aquatic-Freshwater-Lake
ERR1547103	MGYA00320814	https://www.ebi.ac.uk/metagenomics/api/v1/analyses/MGYA00320814/file/ERR1547103_FASTQ_SSU_OTU.tsv	Root-Environmental-Aquatic-Freshwater-Lake
ERR1582603	MGYA00352840	https://www.ebi.ac.uk/metagenomics/api/v1/analyses/MGYA00352840/file/ERR1582603_FASTQ_SSU_OTU.tsv	Root-Environmental-Aquatic-Freshwater-Lake
ERR1583043	MGYA00353280	https://www.ebi.ac.uk/metagenomics/api/v1/analyses/MGYA00353280/file/ERR1583043_FASTQ_SSU_OTU.tsv	Root-Environmental-Aquatic-Freshwater-Lake
ERR1583422	MGYA00353659	https://www.ebi.ac.uk/metagenomics/api/v1/analyses/MGYA00353659/file/ERR1583422_FASTQ_SSU_OTU.tsv	Root-Environmental-Aquatic-Freshwater-Lake
ERR1583385	MGYA00353622	https://www.ebi.ac.uk/metagenomics/api/v1/analyses/MGYA00353622/file/ERR1583385_FASTQ_SSU_OTU.tsv	Root-Environmental-Aquatic-Freshwater-Lake
ERR1582262	MGYA00352499	https://www.ebi.ac.uk/metagenomics/api/v1/analyses/MGYA00352499/file/ERR1582262_FASTQ_SSU_OTU.tsv	Root-Environmental-Aquatic-Freshwater-Lake
ERR658205	MGYA00103476	https://www.ebi.ac.uk/metagenomics/api/v1/analyses/MGYA00103476/file/ERR658205_FASTQ_otu.tsv	Root-Environmental-Aquatic-Freshwater-Lake
ERR1897517	MGYA00351462	https://www.ebi.ac.uk/metagenomics/api/v1/analyses/MGYA00351462/file/ERR1897517_FASTQ_SSU_OTU.tsv	Root-Environmental-Aquatic-Freshwater-Lake
ERR1896882	MGYA00350827	https://www.ebi.ac.uk/metagenomics/api/v1/analyses/MGYA00350827/file/ERR1896882_FASTQ_SSU_OTU.tsv	Root-Environmental-Aquatic-Freshwater-Lake
ERR1896736	MGYA00350681	https://www.ebi.ac.uk/metagenomics/api/v1/analyses/MGYA00350681/file/ERR1896736_FASTQ_SSU_OTU.tsv	Root-Environmental-Aquatic-Freshwater-Lake

ERR1897176	MGYA00351121	https://www.ebi.ac.uk/metagenomics/api/v1/analyses/MGYA00351121/file/ERR1897176_FASTQ_SSU_OTU.tsv	Root-Environmental-Aquatic-Freshwater-Lake
ERR1897600	MGYA00351545	https://www.ebi.ac.uk/metagenomics/api/v1/analyses/MGYA00351545/file/ERR1897600_FASTQ_SSU_OTU.tsv	Root-Environmental-Aquatic-Freshwater-Lake
ERR1896853	MGYA00350798	https://www.ebi.ac.uk/metagenomics/api/v1/analyses/MGYA00350798/file/ERR1896853_FASTQ_SSU_OTU.tsv	Root-Environmental-Aquatic-Freshwater-Lake
ERR1897261	MGYA00351206	https://www.ebi.ac.uk/metagenomics/api/v1/analyses/MGYA00351206/file/ERR1897261_FASTQ_SSU_OTU.tsv	Root-Environmental-Aquatic-Freshwater-Lake
ERR1583353	MGYA00353590	https://www.ebi.ac.uk/metagenomics/api/v1/analyses/MGYA00353590/file/ERR1583353_FASTQ_SSU_OTU.tsv	Root-Environmental-Aquatic-Freshwater-Lake
ERR1582513	MGYA00352750	https://www.ebi.ac.uk/metagenomics/api/v1/analyses/MGYA00352750/file/ERR1582513_FASTQ_SSU_OTU.tsv	Root-Environmental-Aquatic-Freshwater-Lake
ERR1582961	MGYA00353198	https://www.ebi.ac.uk/metagenomics/api/v1/analyses/MGYA00353198/file/ERR1582961_FASTQ_SSU_OTU.tsv	Root-Environmental-Aquatic-Freshwater-Lake
ERR1582172	MGYA00352409	https://www.ebi.ac.uk/metagenomics/api/v1/analyses/MGYA00352409/file/ERR1582172_FASTQ_SSU_OTU.tsv	Root-Environmental-Aquatic-Freshwater-Lake
ERR1583095	MGYA00353332	https://www.ebi.ac.uk/metagenomics/api/v1/analyses/MGYA00353332/file/ERR1583095_FASTQ_SSU_OTU.tsv	Root-Environmental-Aquatic-Freshwater-Lake
ERR1897499	MGYA00351444	https://www.ebi.ac.uk/metagenomics/api/v1/analyses/MGYA00351444/file/ERR1897499_FASTQ_SSU_OTU.tsv	Root-Environmental-Aquatic-Freshwater-Lake
ERR1547104	MGYA00320815	https://www.ebi.ac.uk/metagenomics/api/v1/analyses/MGYA00320815/file/ERR1547104_FASTQ_SSU_OTU.tsv	Root-Environmental-Aquatic-Freshwater-Lake
ERR1897448	MGYA00351393	https://www.ebi.ac.uk/metagenomics/api/v1/analyses/MGYA00351393/file/ERR1897448_FASTQ_SSU_OTU.tsv	Root-Environmental-Aquatic-Freshwater-Lake

ERR1583044	MGYA00353281	https://www.ebi.ac.uk/metagenomics/api/v1/analyses/MGYA00353281/file/ERR1583044_FASTQ_SSU_OTU.tsv	Root-Environmental-Aquatic-Freshwater-Lake
ERR1582604	MGYA00352841	https://www.ebi.ac.uk/metagenomics/api/v1/analyses/MGYA00352841/file/ERR1582604_FASTQ_SSU_OTU.tsv	Root-Environmental-Aquatic-Freshwater-Lake
ERR1897029	MGYA00350974	https://www.ebi.ac.uk/metagenomics/api/v1/analyses/MGYA00350974/file/ERR1897029_FASTQ_SSU_OTU.tsv	Root-Environmental-Aquatic-Freshwater-Lake
SRR7081195	MGYA00425950	https://www.ebi.ac.uk/metagenomics/api/v1/analyses/MGYA00425950/file/SRR7081195_MERGED_FASTQ_SSU_OTU.tsv	Root-Environmental-Aquatic-Freshwater-Lake
ERR1896669	MGYA00350614	https://www.ebi.ac.uk/metagenomics/api/v1/analyses/MGYA00350614/file/ERR1896669_FASTQ_SSU_OTU.tsv	Root-Environmental-Aquatic-Freshwater-Lake
ERR1990479	MGYA00128212	https://www.ebi.ac.uk/metagenomics/api/v1/analyses/MGYA00128212/file/ERR1990479_MERGED_FASTQ_otu.tsv	Root-Environmental-Aquatic-Freshwater-Lake
ERR1582265	MGYA00352502	https://www.ebi.ac.uk/metagenomics/api/v1/analyses/MGYA00352502/file/ERR1582265_FASTQ_SSU_OTU.tsv	Root-Environmental-Aquatic-Freshwater-Lake
ERR1583382	MGYA00353619	https://www.ebi.ac.uk/metagenomics/api/v1/analyses/MGYA00353619/file/ERR1583382_FASTQ_SSU_OTU.tsv	Root-Environmental-Aquatic-Freshwater-Lake
ERR1583425	MGYA00353662	https://www.ebi.ac.uk/metagenomics/api/v1/analyses/MGYA00353662/file/ERR1583425_FASTQ_SSU_OTU.tsv	Root-Environmental-Aquatic-Freshwater-Lake
ERR658202	MGYA00103503	https://www.ebi.ac.uk/metagenomics/api/v1/analyses/MGYA00103503/file/ERR658202_FASTQ_otu.tsv	Root-Environmental-Aquatic-Freshwater-Lake
ERR1896885	MGYA00350830	https://www.ebi.ac.uk/metagenomics/api/v1/analyses/MGYA00350830/file/ERR1896885_FASTQ_SSU_OTU.tsv	Root-Environmental-Aquatic-Freshwater-Lake
ERR1897510	MGYA00351455	https://www.ebi.ac.uk/metagenomics/api/v1/analyses/MGYA00351455/file/ERR1897510_FASTQ_SSU_OTU.tsv	Root-Environmental-Aquatic-Freshwater-Lake

SRR3960573	MGYA00095519	https://www.ebi.ac.uk/metagenomics/api/v1/analyses/MGYA00095519/file/SRR3960573_MERGED_FASTQ_otu.tsv	Root-Environmental-Aquatic-Freshwater-Lake
ERR1897171	MGYA00351116	https://www.ebi.ac.uk/metagenomics/api/v1/analyses/MGYA00351116/file/ERR1897171_FASTQ_SSU_OTU.tsv	Root-Environmental-Aquatic-Freshwater-Lake
ERR1896731	MGYA00350676	https://www.ebi.ac.uk/metagenomics/api/v1/analyses/MGYA00350676/file/ERR1896731_FASTQ_SSU_OTU.tsv	Root-Environmental-Aquatic-Freshwater-Lake
ERR1897607	MGYA00351552	https://www.ebi.ac.uk/metagenomics/api/v1/analyses/MGYA00351552/file/ERR1897607_FASTQ_SSU_OTU.tsv	Root-Environmental-Aquatic-Freshwater-Lake
ERR1582839	MGYA00353076	https://www.ebi.ac.uk/metagenomics/api/v1/analyses/MGYA00353076/file/ERR1582839_FASTQ_SSU_OTU.tsv	Root-Environmental-Aquatic-Freshwater-Lake
ERR1897266	MGYA00351211	https://www.ebi.ac.uk/metagenomics/api/v1/analyses/MGYA00351211/file/ERR1897266_FASTQ_SSU_OTU.tsv	Root-Environmental-Aquatic-Freshwater-Lake
ERR1896854	MGYA00350799	https://www.ebi.ac.uk/metagenomics/api/v1/analyses/MGYA00350799/file/ERR1896854_FASTQ_SSU_OTU.tsv	Root-Environmental-Aquatic-Freshwater-Lake
ERR1897723	MGYA00351668	https://www.ebi.ac.uk/metagenomics/api/v1/analyses/MGYA00351668/file/ERR1897723_FASTQ_SSU_OTU.tsv	Root-Environmental-Aquatic-Freshwater-Lake
ERR1897084	MGYA00351029	https://www.ebi.ac.uk/metagenomics/api/v1/analyses/MGYA00351029/file/ERR1897084_FASTQ_SSU_OTU.tsv	Root-Environmental-Aquatic-Freshwater-Lake
ERR1583488	MGYA00353725	https://www.ebi.ac.uk/metagenomics/api/v1/analyses/MGYA00353725/file/ERR1583488_FASTQ_SSU_OTU.tsv	Root-Environmental-Aquatic-Freshwater-Lake
ERR1897342	MGYA00351287	https://www.ebi.ac.uk/metagenomics/api/v1/analyses/MGYA00351287/file/ERR1897342_FASTQ_SSU_OTU.tsv	Root-Environmental-Aquatic-Freshwater-Lake
ERR589337	MGYA00103419	https://www.ebi.ac.uk/metagenomics/api/v1/analyses/MGYA00103419/file/ERR589337_FASTQ_otu.tsv	Root-Environmental-Aquatic-Freshwater-Lake

ERR1896970	MGYA00350915	https://www.ebi.ac.uk/metagenomics/api/v1/analyses/MGYA00350915/file/ERR1896970_FASTQ_SSU_OTU.tsv	Root-Environmental-Aquatic-Freshwater-Lake
SRR2442993	MGYA00358997	https://www.ebi.ac.uk/metagenomics/api/v1/analyses/MGYA00358997/file/SRR2442993_MERGED_FASTQ_SSU_OTU.tsv	Root-Environmental-Aquatic-Freshwater-Lake
ERR1583038	MGYA00353275	https://www.ebi.ac.uk/metagenomics/api/v1/analyses/MGYA00353275/file/ERR1583038_FASTQ_SSU_OTU.tsv	Root-Environmental-Aquatic-Freshwater-Lake
ERR1582678	MGYA00352915	https://www.ebi.ac.uk/metagenomics/api/v1/analyses/MGYA00352915/file/ERR1582678_FASTQ_SSU_OTU.tsv	Root-Environmental-Aquatic-Freshwater-Lake
ERR1743347	MGYA00087216	https://www.ebi.ac.uk/metagenomics/api/v1/analyses/MGYA00087216/file/ERR1743347_MERGED_FASTQ_otu.tsv	Root-Environmental-Aquatic-Freshwater-Lake
ERR1897393	MGYA00351338	https://www.ebi.ac.uk/metagenomics/api/v1/analyses/MGYA00351338/file/ERR1897393_FASTQ_SSU_OTU.tsv	Root-Environmental-Aquatic-Freshwater-Lake
ERR1897434	MGYA00351379	https://www.ebi.ac.uk/metagenomics/api/v1/analyses/MGYA00351379/file/ERR1897434_FASTQ_SSU_OTU.tsv	Root-Environmental-Aquatic-Freshwater-Lake
ERR1582219	MGYA00352456	https://www.ebi.ac.uk/metagenomics/api/v1/analyses/MGYA00352456/file/ERR1582219_FASTQ_SSU_OTU.tsv	Root-Environmental-Aquatic-Freshwater-Lake
ERR1583459	MGYA00353696	https://www.ebi.ac.uk/metagenomics/api/v1/analyses/MGYA00353696/file/ERR1583459_FASTQ_SSU_OTU.tsv	Root-Environmental-Aquatic-Freshwater-Lake
ERR1897055	MGYA00351000	https://www.ebi.ac.uk/metagenomics/api/v1/analyses/MGYA00351000/file/ERR1897055_FASTQ_SSU_OTU.tsv	Root-Environmental-Aquatic-Freshwater-Lake
SRR7081377	MGYA00414115	https://www.ebi.ac.uk/metagenomics/api/v1/analyses/MGYA00414115/file/SRR7081377_MERGED_FASTQ_SSU_OTU.tsv	Root-Environmental-Aquatic-Freshwater-Lake
ERR1583160	MGYA00353397	https://www.ebi.ac.uk/metagenomics/api/v1/analyses/MGYA00353397/file/ERR1583160_FASTQ_SSU_OTU.tsv	Root-Environmental-Aquatic-Freshwater-Lake

ERR1582720	MGYA00352957	https://www.ebi.ac.uk/metagenomics/api/v1/analyses/MGYA00352957/file/ERR1582720_FASTQ_SSU_OTU.tsv	Root-Environmental-Aquatic-Freshwater-Lake
ERR1582087	MGYA00352324	https://www.ebi.ac.uk/metagenomics/api/v1/analyses/MGYA00352324/file/ERR1582087_FASTQ_SSU_OTU.tsv	Root-Environmental-Aquatic-Freshwater-Lake
ERR1535986	MGYA00320277	https://www.ebi.ac.uk/metagenomics/api/v1/analyses/MGYA00320277/file/ERR1535986_FASTQ_SSU_OTU.tsv	Root-Environmental-Aquatic-Freshwater-Lake
ERR1429264	MGYA00107657	https://www.ebi.ac.uk/metagenomics/api/v1/analyses/MGYA00107657/file/ERR1429264_FASTQ_otu.tsv	Root-Environmental-Aquatic-Freshwater-Lake
ERR1582341	MGYA00352578	https://www.ebi.ac.uk/metagenomics/api/v1/analyses/MGYA00352578/file/ERR1582341_FASTQ_SSU_OTU.tsv	Root-Environmental-Aquatic-Freshwater-Lake
ERR1582894	MGYA00353131	https://www.ebi.ac.uk/metagenomics/api/v1/analyses/MGYA00353131/file/ERR1582894_FASTQ_SSU_OTU.tsv	Root-Environmental-Aquatic-Freshwater-Lake
ERR1583501	MGYA00353738	https://www.ebi.ac.uk/metagenomics/api/v1/analyses/MGYA00353738/file/ERR1583501_FASTQ_SSU_OTU.tsv	Root-Environmental-Aquatic-Freshwater-Lake
ERR1333500	MGYA00107603	https://www.ebi.ac.uk/metagenomics/api/v1/analyses/MGYA00107603/file/ERR1333500_FASTQ_otu.tsv	Root-Environmental-Aquatic-Freshwater-Lake
ERR1583277	MGYA00353514	https://www.ebi.ac.uk/metagenomics/api/v1/analyses/MGYA00353514/file/ERR1583277_FASTQ_SSU_OTU.tsv	Root-Environmental-Aquatic-Freshwater-Lake
ERR1582390	MGYA00352627	https://www.ebi.ac.uk/metagenomics/api/v1/analyses/MGYA00352627/file/ERR1582390_FASTQ_SSU_OTU.tsv	Root-Environmental-Aquatic-Freshwater-Lake
ERR1582845	MGYA00353082	https://www.ebi.ac.uk/metagenomics/api/v1/analyses/MGYA00353082/file/ERR1582845_FASTQ_SSU_OTU.tsv	Root-Environmental-Aquatic-Freshwater-Lake
ERR1582437	MGYA00352674	https://www.ebi.ac.uk/metagenomics/api/v1/analyses/MGYA00352674/file/ERR1582437_FASTQ_SSU_OTU.tsv	Root-Environmental-Aquatic-Freshwater-Lake

ERR1535957	MGYA00320248	https://www.ebi.ac.uk/metagenomics/api/v1/analyses/MGYA00320248/file/ERR1535957_FASTQ_SSU_OTU.tsv	Root-Environmental-Aquatic-Freshwater-Lake
ERR1314336	MGYA00095177	https://www.ebi.ac.uk/metagenomics/api/v1/analyses/MGYA00095177/file/ERR1314336_MERGED_FASTQ_otu.tsv	Root-Environmental-Aquatic-Freshwater-Lake
ERR1582056	MGYA00352293	https://www.ebi.ac.uk/metagenomics/api/v1/analyses/MGYA00352293/file/ERR1582056_FASTQ_SSU_OTU.tsv	Root-Environmental-Aquatic-Freshwater-Lake
ERR1896828	MGYA00350773	https://www.ebi.ac.uk/metagenomics/api/v1/analyses/MGYA00350773/file/ERR1896828_FASTQ_SSU_OTU.tsv	Root-Environmental-Aquatic-Freshwater-Lake
ERR1582250	MGYA00352487	https://www.ebi.ac.uk/metagenomics/api/v1/analyses/MGYA00352487/file/ERR1582250_FASTQ_SSU_OTU.tsv	Root-Environmental-Aquatic-Freshwater-Lake
ERR1582985	MGYA00353222	https://www.ebi.ac.uk/metagenomics/api/v1/analyses/MGYA00353222/file/ERR1582985_FASTQ_SSU_OTU.tsv	Root-Environmental-Aquatic-Freshwater-Lake
ERR430923	MGYA00103255	https://www.ebi.ac.uk/metagenomics/api/v1/analyses/MGYA00103255/file/ERR430923_FASTQ_otu.tsv	Root-Environmental-Aquatic-Freshwater-Lake
ERR1583410	MGYA00353647	https://www.ebi.ac.uk/metagenomics/api/v1/analyses/MGYA00353647/file/ERR1583410_FASTQ_SSU_OTU.tsv	Root-Environmental-Aquatic-Freshwater-Lake
ERR1583071	MGYA00353308	https://www.ebi.ac.uk/metagenomics/api/v1/analyses/MGYA00353308/file/ERR1583071_FASTQ_SSU_OTU.tsv	Root-Environmental-Aquatic-Freshwater-Lake
ERR1582631	MGYA00352868	https://www.ebi.ac.uk/metagenomics/api/v1/analyses/MGYA00352868/file/ERR1582631_FASTQ_SSU_OTU.tsv	Root-Environmental-Aquatic-Freshwater-Lake
ERR1582196	MGYA00352433	https://www.ebi.ac.uk/metagenomics/api/v1/analyses/MGYA00352433/file/ERR1582196_FASTQ_SSU_OTU.tsv	Root-Environmental-Aquatic-Freshwater-Lake
ERR1582147	MGYA00352384	https://www.ebi.ac.uk/metagenomics/api/v1/analyses/MGYA00352384/file/ERR1582147_FASTQ_SSU_OTU.tsv	Root-Environmental-Aquatic-Freshwater-Lake

ERR1896939	MGYA00350884	https://www.ebi.ac.uk/metagenomics/api/v1/analyses/MGYA00350884/file/ERR1896939_FASTQ_SSU_OTU.tsv	Root-Environmental-Aquatic-Freshwater-Lake
ERR1583366	MGYA00353603	https://www.ebi.ac.uk/metagenomics/api/v1/analyses/MGYA00353603/file/ERR1583366_FASTQ_SSU_OTU.tsv	Root-Environmental-Aquatic-Freshwater-Lake
ERR1582281	MGYA00352518	https://www.ebi.ac.uk/metagenomics/api/v1/analyses/MGYA00352518/file/ERR1582281_FASTQ_SSU_OTU.tsv	Root-Environmental-Aquatic-Freshwater-Lake
ERR1582526	MGYA00352763	https://www.ebi.ac.uk/metagenomics/api/v1/analyses/MGYA00352763/file/ERR1582526_FASTQ_SSU_OTU.tsv	Root-Environmental-Aquatic-Freshwater-Lake
ERR1582954	MGYA00353191	https://www.ebi.ac.uk/metagenomics/api/v1/analyses/MGYA00353191/file/ERR1582954_FASTQ_SSU_OTU.tsv	Root-Environmental-Aquatic-Freshwater-Lake
ERR1897253	MGYA00351198	https://www.ebi.ac.uk/metagenomics/api/v1/analyses/MGYA00351198/file/ERR1897253_FASTQ_SSU_OTU.tsv	Root-Environmental-Aquatic-Freshwater-Lake
ERR1896861	MGYA00350806	https://www.ebi.ac.uk/metagenomics/api/v1/analyses/MGYA00350806/file/ERR1896861_FASTQ_SSU_OTU.tsv	Root-Environmental-Aquatic-Freshwater-Lake
ERR1743287	MGYA00087180	https://www.ebi.ac.uk/metagenomics/api/v1/analyses/MGYA00087180/file/ERR1743287_MERGED_FASTQ_otu.tsv	Root-Environmental-Aquatic-Freshwater-Lake
ERR1897632	MGYA00351577	https://www.ebi.ac.uk/metagenomics/api/v1/analyses/MGYA00351577/file/ERR1897632_FASTQ_SSU_OTU.tsv	Root-Environmental-Aquatic-Freshwater-Lake
ERR1897195	MGYA00351140	https://www.ebi.ac.uk/metagenomics/api/v1/analyses/MGYA00351140/file/ERR1897195_FASTQ_SSU_OTU.tsv	Root-Environmental-Aquatic-Freshwater-Lake
ERR1582308	MGYA00352545	https://www.ebi.ac.uk/metagenomics/api/v1/analyses/MGYA00352545/file/ERR1582308_FASTQ_SSU_OTU.tsv	Root-Environmental-Aquatic-Freshwater-Lake
ERR1897144	MGYA00351089	https://www.ebi.ac.uk/metagenomics/api/v1/analyses/MGYA00351089/file/ERR1897144_FASTQ_SSU_OTU.tsv	Root-Environmental-Aquatic-Freshwater-Lake

ERR1896704	MGYA00350649	https://www.ebi.ac.uk/metagenomics/api/v1/analyses/MGYA00350649/file/ERR1896704_FASTQ_SSU_OTU.tsv	Root-Environmental-Aquatic-Freshwater-Lake
ERR1583129	MGYA00353366	https://www.ebi.ac.uk/metagenomics/api/v1/analyses/MGYA00353366/file/ERR1583129_FASTQ_SSU_OTU.tsv	Root-Environmental-Aquatic-Freshwater-Lake
ERR1582769	MGYA00353006	https://www.ebi.ac.uk/metagenomics/api/v1/analyses/MGYA00353006/file/ERR1582769_FASTQ_SSU_OTU.tsv	Root-Environmental-Aquatic-Freshwater-Lake
ERR1314409	MGYA00095180	https://www.ebi.ac.uk/metagenomics/api/v1/analyses/MGYA00095180/file/ERR1314409_MERGED_FASTQ_otu.tsv	Root-Environmental-Aquatic-Freshwater-Lake
ERR1547069	MGYA00320780	https://www.ebi.ac.uk/metagenomics/api/v1/analyses/MGYA00320780/file/ERR1547069_FASTQ_SSU_OTU.tsv	Root-Environmental-Aquatic-Freshwater-Lake
ERR1897282	MGYA00351227	https://www.ebi.ac.uk/metagenomics/api/v1/analyses/MGYA00351227/file/ERR1897282_FASTQ_SSU_OTU.tsv	Root-Environmental-Aquatic-Freshwater-Lake
ERR1897525	MGYA00351470	https://www.ebi.ac.uk/metagenomics/api/v1/analyses/MGYA00351470/file/ERR1897525_FASTQ_SSU_OTU.tsv	Root-Environmental-Aquatic-Freshwater-Lake
ERR1897060	MGYA00351005	https://www.ebi.ac.uk/metagenomics/api/v1/analyses/MGYA00351005/file/ERR1897060_FASTQ_SSU_OTU.tsv	Root-Environmental-Aquatic-Freshwater-Lake
SRR2442977	MGYA00358981	https://www.ebi.ac.uk/metagenomics/api/v1/analyses/MGYA00358981/file/SRR2442977_MERGED_FASTQ_SSU_OTU.tsv	Root-Environmental-Aquatic-Freshwater-Lake
ERR1743372	MGYA00087105	https://www.ebi.ac.uk/metagenomics/api/v1/analyses/MGYA00087105/file/ERR1743372_MERGED_FASTQ_otu.tsv	Root-Environmental-Aquatic-Freshwater-Lake
ERR1896994	MGYA00350939	https://www.ebi.ac.uk/metagenomics/api/v1/analyses/MGYA00350939/file/ERR1896994_FASTQ_SSU_OTU.tsv	Root-Environmental-Aquatic-Freshwater-Lake
ERR1897401	MGYA00351346	https://www.ebi.ac.uk/metagenomics/api/v1/analyses/MGYA00351346/file/ERR1897401_FASTQ_SSU_OTU.tsv	Root-Environmental-Aquatic-Freshwater-Lake

ERR589302	MGYA00103423	https://www.ebi.ac.uk/metagenomics/api/v1/analyses/MGYA00103423/file/ERR589302_FASTQ_otu.tsv	Root-Environmental-Aquatic-Freshwater-Lake
ERR1897377	MGYA00351322	https://www.ebi.ac.uk/metagenomics/api/v1/analyses/MGYA00351322/file/ERR1897377_FASTQ_SSU_OTU.tsv	Root-Environmental-Aquatic-Freshwater-Lake
ERR1896945	MGYA00350890	https://www.ebi.ac.uk/metagenomics/api/v1/analyses/MGYA00350890/file/ERR1896945_FASTQ_SSU_OTU.tsv	Root-Environmental-Aquatic-Freshwater-Lake
ERR1897716	MGYA00351661	https://www.ebi.ac.uk/metagenomics/api/v1/analyses/MGYA00351661/file/ERR1897716_FASTQ_SSU_OTU.tsv	Root-Environmental-Aquatic-Freshwater-Lake
ERR1582928	MGYA00353165	https://www.ebi.ac.uk/metagenomics/api/v1/analyses/MGYA00353165/file/ERR1582928_FASTQ_SSU_OTU.tsv	Root-Environmental-Aquatic-Freshwater-Lake
ERR1582063	MGYA00352300	https://www.ebi.ac.uk/metagenomics/api/v1/analyses/MGYA00352300/file/ERR1582063_FASTQ_SSU_OTU.tsv	Root-Environmental-Aquatic-Freshwater-Lake
ERR1583184	MGYA00353421	https://www.ebi.ac.uk/metagenomics/api/v1/analyses/MGYA00353421/file/ERR1583184_FASTQ_SSU_OTU.tsv	Root-Environmental-Aquatic-Freshwater-Lake
ERR1897588	MGYA00351533	https://www.ebi.ac.uk/metagenomics/api/v1/analyses/MGYA00351533/file/ERR1897588_FASTQ_SSU_OTU.tsv	Root-Environmental-Aquatic-Freshwater-Lake
ERR1583242	MGYA00353479	https://www.ebi.ac.uk/metagenomics/api/v1/analyses/MGYA00353479/file/ERR1583242_FASTQ_SSU_OTU.tsv	Root-Environmental-Aquatic-Freshwater-Lake
ERR1582870	MGYA00353107	https://www.ebi.ac.uk/metagenomics/api/v1/analyses/MGYA00353107/file/ERR1582870_FASTQ_SSU_OTU.tsv	Root-Environmental-Aquatic-Freshwater-Lake
ERR1582402	MGYA00352639	https://www.ebi.ac.uk/metagenomics/api/v1/analyses/MGYA00352639/file/ERR1582402_FASTQ_SSU_OTU.tsv	Root-Environmental-Aquatic-Freshwater-Lake
ERR1429280	MGYA00107674	https://www.ebi.ac.uk/metagenomics/api/v1/analyses/MGYA00107674/file/ERR1429280_FASTQ_otu.tsv	Root-Environmental-Aquatic-Freshwater-Lake

ERR1535962	MGYA00320253	https://www.ebi.ac.uk/metagenomics/api/v1/analyses/MGYA00320253/file/ERR1535962_FASTQ_SSU_OTU.tsv	Root-Environmental-Aquatic-Freshwater-Lake
ERR1742295	MGYA00311596	https://www.ebi.ac.uk/metagenomics/api/v1/analyses/MGYA00311596/file/ERR1742295_FASTQ_SSU_OTU.tsv	Root-Environmental-Aquatic-Freshwater-Lake
ERR1897138	MGYA00351083	https://www.ebi.ac.uk/metagenomics/api/v1/analyses/MGYA00351083/file/ERR1897138_FASTQ_SSU_OTU.tsv	Root-Environmental-Aquatic-Freshwater-Lake
ERR1896778	MGYA00350723	https://www.ebi.ac.uk/metagenomics/api/v1/analyses/MGYA00350723/file/ERR1896778_FASTQ_SSU_OTU.tsv	Root-Environmental-Aquatic-Freshwater-Lake
ERR1582374	MGYA00352611	https://www.ebi.ac.uk/metagenomics/api/v1/analyses/MGYA00352611/file/ERR1582374_FASTQ_SSU_OTU.tsv	Root-Environmental-Aquatic-Freshwater-Lake
ERR1429251	MGYA00107660	https://www.ebi.ac.uk/metagenomics/api/v1/analyses/MGYA00107660/file/ERR1429251_FASTQ_otu.tsv	Root-Environmental-Aquatic-Freshwater-Lake
ERR1583293	MGYA00353530	https://www.ebi.ac.uk/metagenomics/api/v1/analyses/MGYA00353530/file/ERR1583293_FASTQ_SSU_OTU.tsv	Root-Environmental-Aquatic-Freshwater-Lake
ERR1897559	MGYA00351504	https://www.ebi.ac.uk/metagenomics/api/v1/analyses/MGYA00351504/file/ERR1897559_FASTQ_SSU_OTU.tsv	Root-Environmental-Aquatic-Freshwater-Lake
ERR1583155	MGYA00353392	https://www.ebi.ac.uk/metagenomics/api/v1/analyses/MGYA00353392/file/ERR1583155_FASTQ_SSU_OTU.tsv	Root-Environmental-Aquatic-Freshwater-Lake
ERR1582715	MGYA00352952	https://www.ebi.ac.uk/metagenomics/api/v1/analyses/MGYA00352952/file/ERR1582715_FASTQ_SSU_OTU.tsv	Root-Environmental-Aquatic-Freshwater-Lake
ERR1897067	MGYA00351012	https://www.ebi.ac.uk/metagenomics/api/v1/analyses/MGYA00351012/file/ERR1897067_FASTQ_SSU_OTU.tsv	Root-Environmental-Aquatic-Freshwater-Lake
ERR1897406	MGYA00351351	https://www.ebi.ac.uk/metagenomics/api/v1/analyses/MGYA00351351/file/ERR1897406_FASTQ_SSU_OTU.tsv	Root-Environmental-Aquatic-Freshwater-Lake

ERR1896993	MGYA00350938	https://www.ebi.ac.uk/metagenomics/api/v1/analyses/MGYA00350938/file/ERR1896993_FASTQ_SSU_OTU.tsv	Root-Environmental-Aquatic-Freshwater-Lake
ERR1743375	MGYA00087114	https://www.ebi.ac.uk/metagenomics/api/v1/analyses/MGYA00087114/file/ERR1743375_MERGED_FASTQ_otu.tsv	Root-Environmental-Aquatic-Freshwater-Lake
SRR2442970	MGYA00358974	https://www.ebi.ac.uk/metagenomics/api/v1/analyses/MGYA00358974/file/SRR2442970_MERGED_FASTQ_SSU_OTU.tsv	Root-Environmental-Aquatic-Freshwater-Lake
ERR1896942	MGYA00350887	https://www.ebi.ac.uk/metagenomics/api/v1/analyses/MGYA00350887/file/ERR1896942_FASTQ_SSU_OTU.tsv	Root-Environmental-Aquatic-Freshwater-Lake
ERR1897370	MGYA00351315	https://www.ebi.ac.uk/metagenomics/api/v1/analyses/MGYA00351315/file/ERR1897370_FASTQ_SSU_OTU.tsv	Root-Environmental-Aquatic-Freshwater-Lake
ERR589305	MGYA00103431	https://www.ebi.ac.uk/metagenomics/api/v1/analyses/MGYA00103431/file/ERR589305_FASTQ_otu.tsv	Root-Environmental-Aquatic-Freshwater-Lake
ERR1897711	MGYA00351656	https://www.ebi.ac.uk/metagenomics/api/v1/analyses/MGYA00351656/file/ERR1897711_FASTQ_SSU_OTU.tsv	Root-Environmental-Aquatic-Freshwater-Lake
ERR1583183	MGYA00353420	https://www.ebi.ac.uk/metagenomics/api/v1/analyses/MGYA00353420/file/ERR1583183_FASTQ_SSU_OTU.tsv	Root-Environmental-Aquatic-Freshwater-Lake
ERR1582064	MGYA00352301	https://www.ebi.ac.uk/metagenomics/api/v1/analyses/MGYA00352301/file/ERR1582064_FASTQ_SSU_OTU.tsv	Root-Environmental-Aquatic-Freshwater-Lake
SRR7081394	MGYA00422888	https://www.ebi.ac.uk/metagenomics/api/v1/analyses/MGYA00422888/file/SRR7081394_MERGED_FASTQ_SSU_OTU.tsv	Root-Environmental-Aquatic-Freshwater-Lake
ERR1897228	MGYA00351173	https://www.ebi.ac.uk/metagenomics/api/v1/analyses/MGYA00351173/file/ERR1897228_FASTQ_SSU_OTU.tsv	Root-Environmental-Aquatic-Freshwater-Lake
ERR1582405	MGYA00352642	https://www.ebi.ac.uk/metagenomics/api/v1/analyses/MGYA00352642/file/ERR1582405_FASTQ_SSU_OTU.tsv	Root-Environmental-Aquatic-Freshwater-Lake

ERR1582877	MGYA00353114	https://www.ebi.ac.uk/metagenomics/api/v1/analyses/MGYA00353114/file/ERR1582877_FASTQ_SSU_OTU.tsv	Root-Environmental-Aquatic-Freshwater-Lake
ERR1583245	MGYA00353482	https://www.ebi.ac.uk/metagenomics/api/v1/analyses/MGYA00353482/file/ERR1583245_FASTQ_SSU_OTU.tsv	Root-Environmental-Aquatic-Freshwater-Lake
ERR1897649	MGYA00351594	https://www.ebi.ac.uk/metagenomics/api/v1/analyses/MGYA00351594/file/ERR1897649_FASTQ_SSU_OTU.tsv	Root-Environmental-Aquatic-Freshwater-Lake
ERR1742292	MGYA00311593	https://www.ebi.ac.uk/metagenomics/api/v1/analyses/MGYA00311593/file/ERR1742292_FASTQ_SSU_OTU.tsv	Root-Environmental-Aquatic-Freshwater-Lake
ERR1535965	MGYA00320256	https://www.ebi.ac.uk/metagenomics/api/v1/analyses/MGYA00320256/file/ERR1535965_FASTQ_SSU_OTU.tsv	Root-Environmental-Aquatic-Freshwater-Lake
ERR1897698	MGYA00351643	https://www.ebi.ac.uk/metagenomics/api/v1/analyses/MGYA00351643/file/ERR1897698_FASTQ_SSU_OTU.tsv	Root-Environmental-Aquatic-Freshwater-Lake
ERR1583294	MGYA00353531	https://www.ebi.ac.uk/metagenomics/api/v1/analyses/MGYA00353531/file/ERR1583294_FASTQ_SSU_OTU.tsv	Root-Environmental-Aquatic-Freshwater-Lake
ERR1429256	MGYA00107633	https://www.ebi.ac.uk/metagenomics/api/v1/analyses/MGYA00107633/file/ERR1429256_FASTQ_otu.tsv	Root-Environmental-Aquatic-Freshwater-Lake
ERR1582373	MGYA00352610	https://www.ebi.ac.uk/metagenomics/api/v1/analyses/MGYA00352610/file/ERR1582373_FASTQ_SSU_OTU.tsv	Root-Environmental-Aquatic-Freshwater-Lake
ERR1582712	MGYA00352949	https://www.ebi.ac.uk/metagenomics/api/v1/analyses/MGYA00352949/file/ERR1582712_FASTQ_SSU_OTU.tsv	Root-Environmental-Aquatic-Freshwater-Lake
ERR1583152	MGYA00353389	https://www.ebi.ac.uk/metagenomics/api/v1/analyses/MGYA00353389/file/ERR1583152_FASTQ_SSU_OTU.tsv	Root-Environmental-Aquatic-Freshwater-Lake
ERR1583417	MGYA00353654	https://www.ebi.ac.uk/metagenomics/api/v1/analyses/MGYA00353654/file/ERR1583417_FASTQ_SSU_OTU.tsv	Root-Environmental-Aquatic-Freshwater-Lake

ERR1582982	MGYA00353219	https://www.ebi.ac.uk/metagenomics/api/v1/analyses/MGYA00353219/file/ERR1582982_FASTQ_SSU_OTU.tsv	Root-Environmental-Aquatic-Freshwater-Lake
ERR430924	MGYA00103248	https://www.ebi.ac.uk/metagenomics/api/v1/analyses/MGYA00103248/file/ERR430924_FASTQ_otu.tsv	Root-Environmental-Aquatic-Freshwater-Lake
ERR1582257	MGYA00352494	https://www.ebi.ac.uk/metagenomics/api/v1/analyses/MGYA00352494/file/ERR1582257_FASTQ_SSU_OTU.tsv	Root-Environmental-Aquatic-Freshwater-Lake
ERR1743309	MGYA00087207	https://www.ebi.ac.uk/metagenomics/api/v1/analyses/MGYA00087207/file/ERR1743309_MERGED_FASTQ_otu.tsv	Root-Environmental-Aquatic-Freshwater-Lake
ERR1582191	MGYA00352428	https://www.ebi.ac.uk/metagenomics/api/v1/analyses/MGYA00352428/file/ERR1582191_FASTQ_SSU_OTU.tsv	Root-Environmental-Aquatic-Freshwater-Lake
ERR1582636	MGYA00352873	https://www.ebi.ac.uk/metagenomics/api/v1/analyses/MGYA00352873/file/ERR1582636_FASTQ_SSU_OTU.tsv	Root-Environmental-Aquatic-Freshwater-Lake
ERR1583076	MGYA00353313	https://www.ebi.ac.uk/metagenomics/api/v1/analyses/MGYA00353313/file/ERR1583076_FASTQ_SSU_OTU.tsv	Root-Environmental-Aquatic-Freshwater-Lake
ERR1582140	MGYA00352377	https://www.ebi.ac.uk/metagenomics/api/v1/analyses/MGYA00352377/file/ERR1582140_FASTQ_SSU_OTU.tsv	Root-Environmental-Aquatic-Freshwater-Lake
ERR1582953	MGYA00353190	https://www.ebi.ac.uk/metagenomics/api/v1/analyses/MGYA00353190/file/ERR1582953_FASTQ_SSU_OTU.tsv	Root-Environmental-Aquatic-Freshwater-Lake
ERR1582521	MGYA00352758	https://www.ebi.ac.uk/metagenomics/api/v1/analyses/MGYA00352758/file/ERR1582521_FASTQ_SSU_OTU.tsv	Root-Environmental-Aquatic-Freshwater-Lake
ERR1582286	MGYA00352523	https://www.ebi.ac.uk/metagenomics/api/v1/analyses/MGYA00352523/file/ERR1582286_FASTQ_SSU_OTU.tsv	Root-Environmental-Aquatic-Freshwater-Lake
ERR1583361	MGYA00353598	https://www.ebi.ac.uk/metagenomics/api/v1/analyses/MGYA00353598/file/ERR1583361_FASTQ_SSU_OTU.tsv	Root-Environmental-Aquatic-Freshwater-Lake

SRR2443029	MGYA00359033	https://www.ebi.ac.uk/metagenomics/api/v1/analyses/MGYA00359033/file/SRR2443029_MERGED_FASTQ_SSU_OTU.tsv	Root-Environmental-Aquatic-Freshwater-Lake
ERR1743280	MGYA00087129	https://www.ebi.ac.uk/metagenomics/api/v1/analyses/MGYA00087129/file/ERR1743280_MERGED_FASTQ_otu.tsv	Root-Environmental-Aquatic-Freshwater-Lake
ERR1896866	MGYA00350811	https://www.ebi.ac.uk/metagenomics/api/v1/analyses/MGYA00350811/file/ERR1896866_FASTQ_SSU_OTU.tsv	Root-Environmental-Aquatic-Freshwater-Lake
ERR1897254	MGYA00351199	https://www.ebi.ac.uk/metagenomics/api/v1/analyses/MGYA00351199/file/ERR1897254_FASTQ_SSU_OTU.tsv	Root-Environmental-Aquatic-Freshwater-Lake
ERR1582018	MGYA00352255	https://www.ebi.ac.uk/metagenomics/api/v1/analyses/MGYA00352255/file/ERR1582018_FASTQ_SSU_OTU.tsv	Root-Environmental-Aquatic-Freshwater-Lake
ERR1314378	MGYA00095228	https://www.ebi.ac.uk/metagenomics/api/v1/analyses/MGYA00095228/file/ERR1314378_MERGED_FASTQ_otu.tsv	Root-Environmental-Aquatic-Freshwater-Lake
ERR1897192	MGYA00351137	https://www.ebi.ac.uk/metagenomics/api/v1/analyses/MGYA00351137/file/ERR1897192_FASTQ_SSU_OTU.tsv	Root-Environmental-Aquatic-Freshwater-Lake
ERR1897635	MGYA00351580	https://www.ebi.ac.uk/metagenomics/api/v1/analyses/MGYA00351580/file/ERR1897635_FASTQ_SSU_OTU.tsv	Root-Environmental-Aquatic-Freshwater-Lake
ERR1582479	MGYA00352716	https://www.ebi.ac.uk/metagenomics/api/v1/analyses/MGYA00352716/file/ERR1582479_FASTQ_SSU_OTU.tsv	Root-Environmental-Aquatic-Freshwater-Lake
ERR1583239	MGYA00353476	https://www.ebi.ac.uk/metagenomics/api/v1/analyses/MGYA00353476/file/ERR1583239_FASTQ_SSU_OTU.tsv	Root-Environmental-Aquatic-Freshwater-Lake
ERR1896703	MGYA00350648	https://www.ebi.ac.uk/metagenomics/api/v1/analyses/MGYA00350648/file/ERR1896703_FASTQ_SSU_OTU.tsv	Root-Environmental-Aquatic-Freshwater-Lake
ERR1897143	MGYA00351088	https://www.ebi.ac.uk/metagenomics/api/v1/analyses/MGYA00351088/file/ERR1897143_FASTQ_SSU_OTU.tsv	Root-Environmental-Aquatic-Freshwater-Lake

ERR1554596	MGYA00100724	https://www.ebi.ac.uk/metagenomics/api/v1/analyses/MGYA00100724/file/ERR1554596_MERGED_FASTQ_otu.tsv	Root-Environmental-Aquatic-Freshwater-Lake
ERR1897522	MGYA00351467	https://www.ebi.ac.uk/metagenomics/api/v1/analyses/MGYA00351467/file/ERR1897522_FASTQ_SSU_OTU.tsv	Root-Environmental-Aquatic-Freshwater-Lake
ERR1897285	MGYA00351230	https://www.ebi.ac.uk/metagenomics/api/v1/analyses/MGYA00351230/file/ERR1897285_FASTQ_SSU_OTU.tsv	Root-Environmental-Aquatic-Freshwater-Lake
ERR1897069	MGYA00351014	https://www.ebi.ac.uk/metagenomics/api/v1/analyses/MGYA00351014/file/ERR1897069_FASTQ_SSU_OTU.tsv	Root-Environmental-Aquatic-Freshwater-Lake
ERR1582225	MGYA00352462	https://www.ebi.ac.uk/metagenomics/api/v1/analyses/MGYA00352462/file/ERR1582225_FASTQ_SSU_OTU.tsv	Root-Environmental-Aquatic-Freshwater-Lake
ERR1582582	MGYA00352819	https://www.ebi.ac.uk/metagenomics/api/v1/analyses/MGYA00352819/file/ERR1582582_FASTQ_SSU_OTU.tsv	Root-Environmental-Aquatic-Freshwater-Lake
ERR1583465	MGYA00353702	https://www.ebi.ac.uk/metagenomics/api/v1/analyses/MGYA00353702/file/ERR1583465_FASTQ_SSU_OTU.tsv	Root-Environmental-Aquatic-Freshwater-Lake
SRR7081213	MGYA00425709	https://www.ebi.ac.uk/metagenomics/api/v1/analyses/MGYA00425709/file/SRR7081213_MERGED_FASTQ_SSU_OTU.tsv	Root-Environmental-Aquatic-Freshwater-Lake
ERR1897408	MGYA00351353	https://www.ebi.ac.uk/metagenomics/api/v1/analyses/MGYA00351353/file/ERR1897408_FASTQ_SSU_OTU.tsv	Root-Environmental-Aquatic-Freshwater-Lake
ERR1583004	MGYA00353241	https://www.ebi.ac.uk/metagenomics/api/v1/analyses/MGYA00353241/file/ERR1583004_FASTQ_SSU_OTU.tsv	Root-Environmental-Aquatic-Freshwater-Lake
ERR1582644	MGYA00352881	https://www.ebi.ac.uk/metagenomics/api/v1/analyses/MGYA00352881/file/ERR1582644_FASTQ_SSU_OTU.tsv	Root-Environmental-Aquatic-Freshwater-Lake
ERR1582695	MGYA00352932	https://www.ebi.ac.uk/metagenomics/api/v1/analyses/MGYA00352932/file/ERR1582695_FASTQ_SSU_OTU.tsv	Root-Environmental-Aquatic-Freshwater-Lake

ERR1582132	MGYA00352369	https://www.ebi.ac.uk/metagenomics/api/v1/analyses/MGYA00352369/file/ERR1582132_FASTQ_SSU_OTU.tsv	Root-Environmental-Aquatic-Freshwater-Lake
ERR1583313	MGYA00353550	https://www.ebi.ac.uk/metagenomics/api/v1/analyses/MGYA00353550/file/ERR1583313_FASTQ_SSU_OTU.tsv	Root-Environmental-Aquatic-Freshwater-Lake
ERR1582921	MGYA00353158	https://www.ebi.ac.uk/metagenomics/api/v1/analyses/MGYA00353158/file/ERR1582921_FASTQ_SSU_OTU.tsv	Root-Environmental-Aquatic-Freshwater-Lake
ERR1582553	MGYA00352790	https://www.ebi.ac.uk/metagenomics/api/v1/analyses/MGYA00352790/file/ERR1582553_FASTQ_SSU_OTU.tsv	Root-Environmental-Aquatic-Freshwater-Lake
ERR1897226	MGYA00351171	https://www.ebi.ac.uk/metagenomics/api/v1/analyses/MGYA00351171/file/ERR1897226_FASTQ_SSU_OTU.tsv	Root-Environmental-Aquatic-Freshwater-Lake
ERR1897581	MGYA00351526	https://www.ebi.ac.uk/metagenomics/api/v1/analyses/MGYA00351526/file/ERR1897581_FASTQ_SSU_OTU.tsv	Root-Environmental-Aquatic-Freshwater-Lake
ERR1896814	MGYA00350759	https://www.ebi.ac.uk/metagenomics/api/v1/analyses/MGYA00350759/file/ERR1896814_FASTQ_SSU_OTU.tsv	Root-Environmental-Aquatic-Freshwater-Lake
ERR1897647	MGYA00351592	https://www.ebi.ac.uk/metagenomics/api/v1/analyses/MGYA00351592/file/ERR1897647_FASTQ_SSU_OTU.tsv	Root-Environmental-Aquatic-Freshwater-Lake
ERR1582879	MGYA00353116	https://www.ebi.ac.uk/metagenomics/api/v1/analyses/MGYA00353116/file/ERR1582879_FASTQ_SSU_OTU.tsv	Root-Environmental-Aquatic-Freshwater-Lake
ERR1429258	MGYA00107680	https://www.ebi.ac.uk/metagenomics/api/v1/analyses/MGYA00107680/file/ERR1429258_FASTQ_otu.tsv	Root-Environmental-Aquatic-Freshwater-Lake
ERR658184	MGYA00103505	https://www.ebi.ac.uk/metagenomics/api/v1/analyses/MGYA00103505/file/ERR658184_FASTQ_otu.tsv	Root-Environmental-Aquatic-Freshwater-Lake
ERR1897696	MGYA00351641	https://www.ebi.ac.uk/metagenomics/api/v1/analyses/MGYA00351641/file/ERR1897696_FASTQ_SSU_OTU.tsv	Root-Environmental-Aquatic-Freshwater-Lake

ERR1897131	MGYA00351076	https://www.ebi.ac.uk/metagenomics/api/v1/analyses/MGYA00351076/file/ERR1897131_FASTQ_SSU_OTU.tsv	Root-Environmental-Aquatic-Freshwater-Lake
ERR1896771	MGYA00350716	https://www.ebi.ac.uk/metagenomics/api/v1/analyses/MGYA00350716/file/ERR1896771_FASTQ_SSU_OTU.tsv	Root-Environmental-Aquatic-Freshwater-Lake
ERR1897550	MGYA00351495	https://www.ebi.ac.uk/metagenomics/api/v1/analyses/MGYA00351495/file/ERR1897550_FASTQ_SSU_OTU.tsv	Root-Environmental-Aquatic-Freshwater-Lake
ERR1582259	MGYA00352496	https://www.ebi.ac.uk/metagenomics/api/v1/analyses/MGYA00352496/file/ERR1582259_FASTQ_SSU_OTU.tsv	Root-Environmental-Aquatic-Freshwater-Lake
ERR1583419	MGYA00353656	https://www.ebi.ac.uk/metagenomics/api/v1/analyses/MGYA00353656/file/ERR1583419_FASTQ_SSU_OTU.tsv	Root-Environmental-Aquatic-Freshwater-Lake
ERR1897015	MGYA00350960	https://www.ebi.ac.uk/metagenomics/api/v1/analyses/MGYA00350960/file/ERR1897015_FASTQ_SSU_OTU.tsv	Root-Environmental-Aquatic-Freshwater-Lake
ERR1896655	MGYA00350600	https://www.ebi.ac.uk/metagenomics/api/v1/analyses/MGYA00350600/file/ERR1896655_FASTQ_SSU_OTU.tsv	Root-Environmental-Aquatic-Freshwater-Lake
ERR1583078	MGYA00353315	https://www.ebi.ac.uk/metagenomics/api/v1/analyses/MGYA00353315/file/ERR1583078_FASTQ_SSU_OTU.tsv	Root-Environmental-Aquatic-Freshwater-Lake
ERR1582638	MGYA00352875	https://www.ebi.ac.uk/metagenomics/api/v1/analyses/MGYA00352875/file/ERR1582638_FASTQ_SSU_OTU.tsv	Root-Environmental-Aquatic-Freshwater-Lake
ERR1743307	MGYA00087145	https://www.ebi.ac.uk/metagenomics/api/v1/analyses/MGYA00087145/file/ERR1743307_MERGED_FASTQ_otu.tsv	Root-Environmental-Aquatic-Freshwater-Lake
ERR1897474	MGYA00351419	https://www.ebi.ac.uk/metagenomics/api/v1/analyses/MGYA00351419/file/ERR1897474_FASTQ_SSU_OTU.tsv	Root-Environmental-Aquatic-Freshwater-Lake
ERR1897302	MGYA00351247	https://www.ebi.ac.uk/metagenomics/api/v1/analyses/MGYA00351247/file/ERR1897302_FASTQ_SSU_OTU.tsv	Root-Environmental-Aquatic-Freshwater-Lake

ERR1896930	MGYA00350875	https://www.ebi.ac.uk/metagenomics/api/v1/analyses/MGYA00350875/file/ERR1896930_FASTQ_SSU_OTU.tsv	Root-Environmental-Aquatic-Freshwater-Lake
ERR1896684	MGYA00350629	https://www.ebi.ac.uk/metagenomics/api/v1/analyses/MGYA00350629/file/ERR1896684_FASTQ_SSU_OTU.tsv	Root-Environmental-Aquatic-Freshwater-Lake
ERR1897763	MGYA00351708	https://www.ebi.ac.uk/metagenomics/api/v1/analyses/MGYA00351708/file/ERR1897763_FASTQ_SSU_OTU.tsv	Root-Environmental-Aquatic-Freshwater-Lake
SRR2443027	MGYA00359031	https://www.ebi.ac.uk/metagenomics/api/v1/analyses/MGYA00359031/file/SRR2443027_MERGED_FASTQ_SSU_OTU.tsv	Root-Environmental-Aquatic-Freshwater-Lake
ERR1990494	MGYA00128208	https://www.ebi.ac.uk/metagenomics/api/v1/analyses/MGYA00128208/file/ERR1990494_MERGED_FASTQ_otu.tsv	Root-Environmental-Aquatic-Freshwater-Lake
ERR1582288	MGYA00352525	https://www.ebi.ac.uk/metagenomics/api/v1/analyses/MGYA00352525/file/ERR1582288_FASTQ_SSU_OTU.tsv	Root-Environmental-Aquatic-Freshwater-Lake
ERR1582016	MGYA00352253	https://www.ebi.ac.uk/metagenomics/api/v1/analyses/MGYA00352253/file/ERR1582016_FASTQ_SSU_OTU.tsv	Root-Environmental-Aquatic-Freshwater-Lake
ERR1314376	MGYA00095224	https://www.ebi.ac.uk/metagenomics/api/v1/analyses/MGYA00095224/file/ERR1314376_MERGED_FASTQ_otu.tsv	Root-Environmental-Aquatic-Freshwater-Lake
ERR1896868	MGYA00350813	https://www.ebi.ac.uk/metagenomics/api/v1/analyses/MGYA00350813/file/ERR1896868_FASTQ_SSU_OTU.tsv	Root-Environmental-Aquatic-Freshwater-Lake
ERR1583237	MGYA00353474	https://www.ebi.ac.uk/metagenomics/api/v1/analyses/MGYA00353474/file/ERR1583237_FASTQ_SSU_OTU.tsv	Root-Environmental-Aquatic-Freshwater-Lake
ERR1582477	MGYA00352714	https://www.ebi.ac.uk/metagenomics/api/v1/analyses/MGYA00352714/file/ERR1582477_FASTQ_SSU_OTU.tsv	Root-Environmental-Aquatic-Freshwater-Lake
ERR1582805	MGYA00353042	https://www.ebi.ac.uk/metagenomics/api/v1/analyses/MGYA00353042/file/ERR1582805_FASTQ_SSU_OTU.tsv	Root-Environmental-Aquatic-Freshwater-Lake

SRR4029112	MGYA00095524	https://www.ebi.ac.uk/metagenomics/api/v1/analyses/MGYA00095524/file/SRR4029112_MERGED_FASTQ_otu.tsv	Root-Environmental-Aquatic-Freshwater-Lake
ERR1582301	MGYA00352538	https://www.ebi.ac.uk/metagenomics/api/v1/analyses/MGYA00352538/file/ERR1582301_FASTQ_SSU_OTU.tsv	Root-Environmental-Aquatic-Freshwater-Lake
ERR1429224	MGYA00107678	https://www.ebi.ac.uk/metagenomics/api/v1/analyses/MGYA00107678/file/ERR1429224_FASTQ_otu.tsv	Root-Environmental-Aquatic-Freshwater-Lake
ERR1547060	MGYA00320771	https://www.ebi.ac.uk/metagenomics/api/v1/analyses/MGYA00320771/file/ERR1547060_FASTQ_SSU_OTU.tsv	Root-Environmental-Aquatic-Freshwater-Lake
SRR7081337	MGYA00423842	https://www.ebi.ac.uk/metagenomics/api/v1/analyses/MGYA00423842/file/SRR7081337_MERGED_FASTQ_SSU_OTU.tsv	Root-Environmental-Aquatic-Freshwater-Lake
ERR1583120	MGYA00353357	https://www.ebi.ac.uk/metagenomics/api/v1/analyses/MGYA00353357/file/ERR1583120_FASTQ_SSU_OTU.tsv	Root-Environmental-Aquatic-Freshwater-Lake
ERR1314400	MGYA00095192	https://www.ebi.ac.uk/metagenomics/api/v1/analyses/MGYA00095192/file/ERR1314400_MERGED_FASTQ_otu.tsv	Root-Environmental-Aquatic-Freshwater-Lake
ERR1582760	MGYA00352997	https://www.ebi.ac.uk/metagenomics/api/v1/analyses/MGYA00352997/file/ERR1582760_FASTQ_SSU_OTU.tsv	Root-Environmental-Aquatic-Freshwater-Lake
ERR1896652	MGYA00350597	https://www.ebi.ac.uk/metagenomics/api/v1/analyses/MGYA00350597/file/ERR1896652_FASTQ_SSU_OTU.tsv	Root-Environmental-Aquatic-Freshwater-Lake
ERR1897012	MGYA00350957	https://www.ebi.ac.uk/metagenomics/api/v1/analyses/MGYA00350957/file/ERR1897012_FASTQ_SSU_OTU.tsv	Root-Environmental-Aquatic-Freshwater-Lake
ERR1582198	MGYA00352435	https://www.ebi.ac.uk/metagenomics/api/v1/analyses/MGYA00352435/file/ERR1582198_FASTQ_SSU_OTU.tsv	Root-Environmental-Aquatic-Freshwater-Lake
ERR1897473	MGYA00351418	https://www.ebi.ac.uk/metagenomics/api/v1/analyses/MGYA00351418/file/ERR1897473_FASTQ_SSU_OTU.tsv	Root-Environmental-Aquatic-Freshwater-Lake

ERR1743300	MGYA00087196	https://www.ebi.ac.uk/metagenomics/api/v1/analyses/MGYA00087196/file/ERR1743300_MERGED_FASTQ_otu.tsv	Root-Environmental-Aquatic-Freshwater-Lake
ERR1896937	MGYA00350882	https://www.ebi.ac.uk/metagenomics/api/v1/analyses/MGYA00350882/file/ERR1896937_FASTQ_SSU_OTU.tsv	Root-Environmental-Aquatic-Freshwater-Lake
ERR1897305	MGYA00351250	https://www.ebi.ac.uk/metagenomics/api/v1/analyses/MGYA00351250/file/ERR1897305_FASTQ_SSU_OTU.tsv	Root-Environmental-Aquatic-Freshwater-Lake
ERR1582149	MGYA00352386	https://www.ebi.ac.uk/metagenomics/api/v1/analyses/MGYA00352386/file/ERR1582149_FASTQ_SSU_OTU.tsv	Root-Environmental-Aquatic-Freshwater-Lake
ERR1897764	MGYA00351709	https://www.ebi.ac.uk/metagenomics/api/v1/analyses/MGYA00351709/file/ERR1897764_FASTQ_SSU_OTU.tsv	Root-Environmental-Aquatic-Freshwater-Lake
SRR2443020	MGYA00359024	https://www.ebi.ac.uk/metagenomics/api/v1/analyses/MGYA00359024/file/SRR2443020_MERGED_FASTQ_SSU_OTU.tsv	Root-Environmental-Aquatic-Freshwater-Lake
ERR1896683	MGYA00350628	https://www.ebi.ac.uk/metagenomics/api/v1/analyses/MGYA00350628/file/ERR1896683_FASTQ_SSU_OTU.tsv	Root-Environmental-Aquatic-Freshwater-Lake
ERR1990493	MGYA00128213	https://www.ebi.ac.uk/metagenomics/api/v1/analyses/MGYA00128213/file/ERR1990493_MERGED_FASTQ_otu.tsv	Root-Environmental-Aquatic-Freshwater-Lake
ERR1582528	MGYA00352765	https://www.ebi.ac.uk/metagenomics/api/v1/analyses/MGYA00352765/file/ERR1582528_FASTQ_SSU_OTU.tsv	Root-Environmental-Aquatic-Freshwater-Lake
ERR1583368	MGYA00353605	https://www.ebi.ac.uk/metagenomics/api/v1/analyses/MGYA00353605/file/ERR1583368_FASTQ_SSU_OTU.tsv	Root-Environmental-Aquatic-Freshwater-Lake
ERR1314371	MGYA00095199	https://www.ebi.ac.uk/metagenomics/api/v1/analyses/MGYA00095199/file/ERR1314371_MERGED_FASTQ_otu.tsv	Root-Environmental-Aquatic-Freshwater-Lake
ERR1743289	MGYA00087185	https://www.ebi.ac.uk/metagenomics/api/v1/analyses/MGYA00087185/file/ERR1743289_MERGED_FASTQ_otu.tsv	Root-Environmental-Aquatic-Freshwater-Lake

ERR1582802	MGYA00353039	https://www.ebi.ac.uk/metagenomics/api/v1/analyses/MGYA00353039/file/ERR1582802_FASTQ_SSU_OTU.tsv	Root-Environmental-Aquatic-Freshwater-Lake
ERR1582470	MGYA00352707	https://www.ebi.ac.uk/metagenomics/api/v1/analyses/MGYA00352707/file/ERR1582470_FASTQ_SSU_OTU.tsv	Root-Environmental-Aquatic-Freshwater-Lake
ERR1583230	MGYA00353467	https://www.ebi.ac.uk/metagenomics/api/v1/analyses/MGYA00353467/file/ERR1583230_FASTQ_SSU_OTU.tsv	Root-Environmental-Aquatic-Freshwater-Lake
SRR7081027	MGYA00427371	https://www.ebi.ac.uk/metagenomics/api/v1/analyses/MGYA00427371/file/SRR7081027_MERGED_FASTQ_SSU_OTU.tsv	Root-Environmental-Aquatic-Freshwater-Lake
ERR1429223	MGYA00107627	https://www.ebi.ac.uk/metagenomics/api/v1/analyses/MGYA00107627/file/ERR1429223_FASTQ_otu.tsv	Root-Environmental-Aquatic-Freshwater-Lake
ERR1582306	MGYA00352543	https://www.ebi.ac.uk/metagenomics/api/v1/analyses/MGYA00352543/file/ERR1582306_FASTQ_SSU_OTU.tsv	Root-Environmental-Aquatic-Freshwater-Lake
SRR7081330	MGYA00415419	https://www.ebi.ac.uk/metagenomics/api/v1/analyses/MGYA00415419/file/SRR7081330_MERGED_FASTQ_SSU_OTU.tsv	Root-Environmental-Aquatic-Freshwater-Lake
ERR1547067	MGYA00320778	https://www.ebi.ac.uk/metagenomics/api/v1/analyses/MGYA00320778/file/ERR1547067_FASTQ_SSU_OTU.tsv	Root-Environmental-Aquatic-Freshwater-Lake
ERR1582767	MGYA00353004	https://www.ebi.ac.uk/metagenomics/api/v1/analyses/MGYA00353004/file/ERR1582767_FASTQ_SSU_OTU.tsv	Root-Environmental-Aquatic-Freshwater-Lake
ERR1314407	MGYA00095205	https://www.ebi.ac.uk/metagenomics/api/v1/analyses/MGYA00095205/file/ERR1314407_MERGED_FASTQ_otu.tsv	Root-Environmental-Aquatic-Freshwater-Lake
ERR1583127	MGYA00353364	https://www.ebi.ac.uk/metagenomics/api/v1/analyses/MGYA00353364/file/ERR1583127_FASTQ_SSU_OTU.tsv	Root-Environmental-Aquatic-Freshwater-Lake
ERR1583462	MGYA00353699	https://www.ebi.ac.uk/metagenomics/api/v1/analyses/MGYA00353699/file/ERR1583462_FASTQ_SSU_OTU.tsv	Root-Environmental-Aquatic-Freshwater-Lake

ERR1582585	MGYA00352822	https://www.ebi.ac.uk/metagenomics/api/v1/analyses/MGYA00352822/file/ERR1582585_FASTQ_SSU_OTU.tsv	Root-Environmental-Aquatic-Freshwater-Lake
ERR1582222	MGYA00352459	https://www.ebi.ac.uk/metagenomics/api/v1/analyses/MGYA00352459/file/ERR1582222_FASTQ_SSU_OTU.tsv	Root-Environmental-Aquatic-Freshwater-Lake
SRR7081214	MGYA00425707	https://www.ebi.ac.uk/metagenomics/api/v1/analyses/MGYA00425707/file/SRR7081214_MERGED_FASTQ_SSU_OTU.tsv	Root-Environmental-Aquatic-Freshwater-Lake
SRR2442979	MGYA00358983	https://www.ebi.ac.uk/metagenomics/api/v1/analyses/MGYA00358983/file/SRR2442979_MERGED_FASTQ_SSU_OTU.tsv	Root-Environmental-Aquatic-Freshwater-Lake
ERR1582643	MGYA00352880	https://www.ebi.ac.uk/metagenomics/api/v1/analyses/MGYA00352880/file/ERR1582643_FASTQ_SSU_OTU.tsv	Root-Environmental-Aquatic-Freshwater-Lake
ERR1583003	MGYA00353240	https://www.ebi.ac.uk/metagenomics/api/v1/analyses/MGYA00353240/file/ERR1583003_FASTQ_SSU_OTU.tsv	Root-Environmental-Aquatic-Freshwater-Lake
ERR1582135	MGYA00352372	https://www.ebi.ac.uk/metagenomics/api/v1/analyses/MGYA00352372/file/ERR1582135_FASTQ_SSU_OTU.tsv	Root-Environmental-Aquatic-Freshwater-Lake
ERR1582692	MGYA00352929	https://www.ebi.ac.uk/metagenomics/api/v1/analyses/MGYA00352929/file/ERR1582692_FASTQ_SSU_OTU.tsv	Root-Environmental-Aquatic-Freshwater-Lake
ERR1897379	MGYA00351324	https://www.ebi.ac.uk/metagenomics/api/v1/analyses/MGYA00351324/file/ERR1897379_FASTQ_SSU_OTU.tsv	Root-Environmental-Aquatic-Freshwater-Lake
ERR1582554	MGYA00352791	https://www.ebi.ac.uk/metagenomics/api/v1/analyses/MGYA00352791/file/ERR1582554_FASTQ_SSU_OTU.tsv	Root-Environmental-Aquatic-Freshwater-Lake
ERR1582926	MGYA00353163	https://www.ebi.ac.uk/metagenomics/api/v1/analyses/MGYA00353163/file/ERR1582926_FASTQ_SSU_OTU.tsv	Root-Environmental-Aquatic-Freshwater-Lake
ERR1583314	MGYA00353551	https://www.ebi.ac.uk/metagenomics/api/v1/analyses/MGYA00353551/file/ERR1583314_FASTQ_SSU_OTU.tsv	Root-Environmental-Aquatic-Freshwater-Lake

ERR1897718	MGYA00351663	https://www.ebi.ac.uk/metagenomics/api/v1/analyses/MGYA00351663/file/ERR1897718_FASTQ_SSU_OTU.tsv	Root-Environmental-Aquatic-Freshwater-Lake
ERR1896813	MGYA00350758	https://www.ebi.ac.uk/metagenomics/api/v1/analyses/MGYA00350758/file/ERR1896813_FASTQ_SSU_OTU.tsv	Root-Environmental-Aquatic-Freshwater-Lake
ERR1897586	MGYA00351531	https://www.ebi.ac.uk/metagenomics/api/v1/analyses/MGYA00351531/file/ERR1897586_FASTQ_SSU_OTU.tsv	Root-Environmental-Aquatic-Freshwater-Lake
ERR1897221	MGYA00351166	https://www.ebi.ac.uk/metagenomics/api/v1/analyses/MGYA00351166/file/ERR1897221_FASTQ_SSU_OTU.tsv	Root-Environmental-Aquatic-Freshwater-Lake
ERR1897640	MGYA00351585	https://www.ebi.ac.uk/metagenomics/api/v1/analyses/MGYA00351585/file/ERR1897640_FASTQ_SSU_OTU.tsv	Root-Environmental-Aquatic-Freshwater-Lake
ERR658183	MGYA00103501	https://www.ebi.ac.uk/metagenomics/api/v1/analyses/MGYA00103501/file/ERR658183_FASTQ_otu.tsv	Root-Environmental-Aquatic-Freshwater-Lake
ERR1896776	MGYA00350721	https://www.ebi.ac.uk/metagenomics/api/v1/analyses/MGYA00350721/file/ERR1896776_FASTQ_SSU_OTU.tsv	Root-Environmental-Aquatic-Freshwater-Lake
ERR1897136	MGYA00351081	https://www.ebi.ac.uk/metagenomics/api/v1/analyses/MGYA00351081/file/ERR1897136_FASTQ_SSU_OTU.tsv	Root-Environmental-Aquatic-Freshwater-Lake
ERR1897691	MGYA00351636	https://www.ebi.ac.uk/metagenomics/api/v1/analyses/MGYA00351636/file/ERR1897691_FASTQ_SSU_OTU.tsv	Root-Environmental-Aquatic-Freshwater-Lake
ERR1897557	MGYA00351502	https://www.ebi.ac.uk/metagenomics/api/v1/analyses/MGYA00351502/file/ERR1897557_FASTQ_SSU_OTU.tsv	Root-Environmental-Aquatic-Freshwater-Lake
SRR2442999	MGYA00359003	https://www.ebi.ac.uk/metagenomics/api/v1/analyses/MGYA00359003/file/SRR2442999_MERGED_FASTQ_SSU_OTU.tsv	Root-Environmental-Aquatic-Freshwater-Lake
ERR1897348	MGYA00351293	https://www.ebi.ac.uk/metagenomics/api/v1/analyses/MGYA00351293/file/ERR1897348_FASTQ_SSU_OTU.tsv	Root-Environmental-Aquatic-Freshwater-Lake

ERR1582104	MGYA00352341	https://www.ebi.ac.uk/metagenomics/api/v1/analyses/MGYA00352341/file/ERR1582104_FASTQ_SSU_OTU.tsv	Root-Environmental-Aquatic-Freshwater-Lake
ERR1897729	MGYA00351674	https://www.ebi.ac.uk/metagenomics/api/v1/analyses/MGYA00351674/file/ERR1897729_FASTQ_SSU_OTU.tsv	Root-Environmental-Aquatic-Freshwater-Lake
ERR1582565	MGYA00352802	https://www.ebi.ac.uk/metagenomics/api/v1/analyses/MGYA00352802/file/ERR1582565_FASTQ_SSU_OTU.tsv	Root-Environmental-Aquatic-Freshwater-Lake
ERR1582917	MGYA00353154	https://www.ebi.ac.uk/metagenomics/api/v1/analyses/MGYA00353154/file/ERR1582917_FASTQ_SSU_OTU.tsv	Root-Environmental-Aquatic-Freshwater-Lake
ERR1583325	MGYA00353562	https://www.ebi.ac.uk/metagenomics/api/v1/analyses/MGYA00353562/file/ERR1583325_FASTQ_SSU_OTU.tsv	Root-Environmental-Aquatic-Freshwater-Lake
ERR1583482	MGYA00353719	https://www.ebi.ac.uk/metagenomics/api/v1/analyses/MGYA00353719/file/ERR1583482_FASTQ_SSU_OTU.tsv	Root-Environmental-Aquatic-Freshwater-Lake
ERR1583453	MGYA00353690	https://www.ebi.ac.uk/metagenomics/api/v1/analyses/MGYA00353690/file/ERR1583453_FASTQ_SSU_OTU.tsv	Root-Environmental-Aquatic-Freshwater-Lake
ERR1582213	MGYA00352450	https://www.ebi.ac.uk/metagenomics/api/v1/analyses/MGYA00352450/file/ERR1582213_FASTQ_SSU_OTU.tsv	Root-Environmental-Aquatic-Freshwater-Lake
ERR1582672	MGYA00352909	https://www.ebi.ac.uk/metagenomics/api/v1/analyses/MGYA00352909/file/ERR1582672_FASTQ_SSU_OTU.tsv	Root-Environmental-Aquatic-Freshwater-Lake
ERR1583032	MGYA00353269	https://www.ebi.ac.uk/metagenomics/api/v1/analyses/MGYA00353269/file/ERR1583032_FASTQ_SSU_OTU.tsv	Root-Environmental-Aquatic-Freshwater-Lake
ERR1897399	MGYA00351344	https://www.ebi.ac.uk/metagenomics/api/v1/analyses/MGYA00351344/file/ERR1897399_FASTQ_SSU_OTU.tsv	Root-Environmental-Aquatic-Freshwater-Lake
ERR1896747	MGYA00350692	https://www.ebi.ac.uk/metagenomics/api/v1/analyses/MGYA00350692/file/ERR1896747_FASTQ_SSU_OTU.tsv	Root-Environmental-Aquatic-Freshwater-Lake

ERR1897107	MGYA00351052	https://www.ebi.ac.uk/metagenomics/api/v1/analyses/MGYA00351052/file/ERR1897107_FASTQ_SSU_OTU.tsv	Root-Environmental-Aquatic-Freshwater-Lake
ERR1897566	MGYA00351511	https://www.ebi.ac.uk/metagenomics/api/v1/analyses/MGYA00351511/file/ERR1897566_FASTQ_SSU_OTU.tsv	Root-Environmental-Aquatic-Freshwater-Lake
ERR1896822	MGYA00350767	https://www.ebi.ac.uk/metagenomics/api/v1/analyses/MGYA00350767/file/ERR1896822_FASTQ_SSU_OTU.tsv	Root-Environmental-Aquatic-Freshwater-Lake
ERR1897210	MGYA00351155	https://www.ebi.ac.uk/metagenomics/api/v1/analyses/MGYA00351155/file/ERR1897210_FASTQ_SSU_OTU.tsv	Root-Environmental-Aquatic-Freshwater-Lake
SRR4029120	MGYA00095525	https://www.ebi.ac.uk/metagenomics/api/v1/analyses/MGYA00095525/file/SRR4029120_MERGED_FASTQ_otu.tsv	Root-Environmental-Aquatic-Freshwater-Lake
ERR1897671	MGYA00351616	https://www.ebi.ac.uk/metagenomics/api/v1/analyses/MGYA00351616/file/ERR1897671_FASTQ_SSU_OTU.tsv	Root-Environmental-Aquatic-Freshwater-Lake
ERR1896796	MGYA00350741	https://www.ebi.ac.uk/metagenomics/api/v1/analyses/MGYA00350741/file/ERR1896796_FASTQ_SSU_OTU.tsv	Root-Environmental-Aquatic-Freshwater-Lake
ERR1582178	MGYA00352415	https://www.ebi.ac.uk/metagenomics/api/v1/analyses/MGYA00352415/file/ERR1582178_FASTQ_SSU_OTU.tsv	Root-Environmental-Aquatic-Freshwater-Lake
ERR1896906	MGYA00350851	https://www.ebi.ac.uk/metagenomics/api/v1/analyses/MGYA00350851/file/ERR1896906_FASTQ_SSU_OTU.tsv	Root-Environmental-Aquatic-Freshwater-Lake
ERR1897334	MGYA00351279	https://www.ebi.ac.uk/metagenomics/api/v1/analyses/MGYA00351279/file/ERR1897334_FASTQ_SSU_OTU.tsv	Root-Environmental-Aquatic-Freshwater-Lake
ERR589341	MGYA00103448	https://www.ebi.ac.uk/metagenomics/api/v1/analyses/MGYA00103448/file/ERR589341_FASTQ_otu.tsv	Root-Environmental-Aquatic-Freshwater-Lake
ERR1897493	MGYA00351438	https://www.ebi.ac.uk/metagenomics/api/v1/analyses/MGYA00351438/file/ERR1897493_FASTQ_SSU_OTU.tsv	Root-Environmental-Aquatic-Freshwater-Lake

ERR1582519	MGYA00352756	https://www.ebi.ac.uk/metagenomics/api/v1/analyses/MGYA00352756/file/ERR1582519_FASTQ_SSU_OTU.tsv	Root-Environmental-Aquatic-Freshwater-Lake
ERR1583359	MGYA00353596	https://www.ebi.ac.uk/metagenomics/api/v1/analyses/MGYA00353596/file/ERR1583359_FASTQ_SSU_OTU.tsv	Root-Environmental-Aquatic-Freshwater-Lake
ERR1897755	MGYA00351700	https://www.ebi.ac.uk/metagenomics/api/v1/analyses/MGYA00351700/file/ERR1897755_FASTQ_SSU_OTU.tsv	Root-Environmental-Aquatic-Freshwater-Lake
SRR2443011	MGYA00359015	https://www.ebi.ac.uk/metagenomics/api/v1/analyses/MGYA00359015/file/SRR2443011_MERGED_FASTQ_SSU_OTU.tsv	Root-Environmental-Aquatic-Freshwater-Lake
ERR1896663	MGYA00350608	https://www.ebi.ac.uk/metagenomics/api/v1/analyses/MGYA00350608/file/ERR1896663_FASTQ_SSU_OTU.tsv	Root-Environmental-Aquatic-Freshwater-Lake
ERR1897023	MGYA00350968	https://www.ebi.ac.uk/metagenomics/api/v1/analyses/MGYA00350968/file/ERR1897023_FASTQ_SSU_OTU.tsv	Root-Environmental-Aquatic-Freshwater-Lake
ERR1583388	MGYA00353625	https://www.ebi.ac.uk/metagenomics/api/v1/analyses/MGYA00353625/file/ERR1583388_FASTQ_SSU_OTU.tsv	Root-Environmental-Aquatic-Freshwater-Lake
ERR1897442	MGYA00351387	https://www.ebi.ac.uk/metagenomics/api/v1/analyses/MGYA00351387/file/ERR1897442_FASTQ_SSU_OTU.tsv	Root-Environmental-Aquatic-Freshwater-Lake
ERR1743331	MGYA00087125	https://www.ebi.ac.uk/metagenomics/api/v1/analyses/MGYA00087125/file/ERR1743331_MERGED_FASTQ_otu.tsv	Root-Environmental-Aquatic-Freshwater-Lake
ERR1582337	MGYA00352574	https://www.ebi.ac.uk/metagenomics/api/v1/analyses/MGYA00352574/file/ERR1582337_FASTQ_SSU_OTU.tsv	Root-Environmental-Aquatic-Freshwater-Lake
ERR1582490	MGYA00352727	https://www.ebi.ac.uk/metagenomics/api/v1/analyses/MGYA00352727/file/ERR1582490_FASTQ_SSU_OTU.tsv	Root-Environmental-Aquatic-Freshwater-Lake
ERR1314391	MGYA00095158	https://www.ebi.ac.uk/metagenomics/api/v1/analyses/MGYA00095158/file/ERR1314391_MERGED_FASTQ_otu.tsv	Root-Environmental-Aquatic-Freshwater-Lake

ERR658208	MGYA00103490	https://www.ebi.ac.uk/metagenomics/api/v1/analyses/MGYA00103490/file/ERR658208_FASTQ_otu.tsv	Root-Environmental-Aquatic-Freshwater-Lake
ERR1582756	MGYA00352993	https://www.ebi.ac.uk/metagenomics/api/v1/analyses/MGYA00352993/file/ERR1582756_FASTQ_SSU_OTU.tsv	Root-Environmental-Aquatic-Freshwater-Lake
ERR1583116	MGYA00353353	https://www.ebi.ac.uk/metagenomics/api/v1/analyses/MGYA00353353/file/ERR1583116_FASTQ_SSU_OTU.tsv	Root-Environmental-Aquatic-Freshwater-Lake
ERR1547056	MGYA00320767	https://www.ebi.ac.uk/metagenomics/api/v1/analyses/MGYA00320767/file/ERR1547056_FASTQ_SSU_OTU.tsv	Root-Environmental-Aquatic-Freshwater-Lake
ERR1547087	MGYA00320798	https://www.ebi.ac.uk/metagenomics/api/v1/analyses/MGYA00320798/file/ERR1547087_FASTQ_SSU_OTU.tsv	Root-Environmental-Aquatic-Freshwater-Lake
ERR1582787	MGYA00353024	https://www.ebi.ac.uk/metagenomics/api/v1/analyses/MGYA00353024/file/ERR1582787_FASTQ_SSU_OTU.tsv	Root-Environmental-Aquatic-Freshwater-Lake
ERR1314340	MGYA00095209	https://www.ebi.ac.uk/metagenomics/api/v1/analyses/MGYA00095209/file/ERR1314340_MERGED_FASTQ_otu.tsv	Root-Environmental-Aquatic-Freshwater-Lake
ERR1582020	MGYA00352257	https://www.ebi.ac.uk/metagenomics/api/v1/analyses/MGYA00352257/file/ERR1582020_FASTQ_SSU_OTU.tsv	Root-Environmental-Aquatic-Freshwater-Lake
ERR1582833	MGYA00353070	https://www.ebi.ac.uk/metagenomics/api/v1/analyses/MGYA00353070/file/ERR1582833_FASTQ_SSU_OTU.tsv	Root-Environmental-Aquatic-Freshwater-Lake
ERR1582441	MGYA00352678	https://www.ebi.ac.uk/metagenomics/api/v1/analyses/MGYA00352678/file/ERR1582441_FASTQ_SSU_OTU.tsv	Root-Environmental-Aquatic-Freshwater-Lake
ERR1583201	MGYA00353438	https://www.ebi.ac.uk/metagenomics/api/v1/analyses/MGYA00353438/file/ERR1583201_FASTQ_SSU_OTU.tsv	Root-Environmental-Aquatic-Freshwater-Lake
ERR1583098	MGYA00353335	https://www.ebi.ac.uk/metagenomics/api/v1/analyses/MGYA00353335/file/ERR1583098_FASTQ_SSU_OTU.tsv	Root-Environmental-Aquatic-Freshwater-Lake

ERR1897494	MGYA00351439	https://www.ebi.ac.uk/metagenomics/api/v1/analyses/MGYA00351439/file/ERR1897494_FASTQ_SSU_OTU.tsv	Root-Environmental-Aquatic-Freshwater-Lake
ERR1897333	MGYA00351278	https://www.ebi.ac.uk/metagenomics/api/v1/analyses/MGYA00351278/file/ERR1897333_FASTQ_SSU_OTU.tsv	Root-Environmental-Aquatic-Freshwater-Lake
ERR1896901	MGYA00350846	https://www.ebi.ac.uk/metagenomics/api/v1/analyses/MGYA00350846/file/ERR1896901_FASTQ_SSU_OTU.tsv	Root-Environmental-Aquatic-Freshwater-Lake
SRR7081149	MGYA00413203	https://www.ebi.ac.uk/metagenomics/api/v1/analyses/MGYA00413203/file/SRR7081149_MERGED_FASTQ_SSU_OTU.tsv	Root-Environmental-Aquatic-Freshwater-Lake
ERR1897752	MGYA00351697	https://www.ebi.ac.uk/metagenomics/api/v1/analyses/MGYA00351697/file/ERR1897752_FASTQ_SSU_OTU.tsv	Root-Environmental-Aquatic-Freshwater-Lake
SRR2443016	MGYA00359020	https://www.ebi.ac.uk/metagenomics/api/v1/analyses/MGYA00359020/file/SRR2443016_MERGED_FASTQ_SSU_OTU.tsv	Root-Environmental-Aquatic-Freshwater-Lake
ERR1897024	MGYA00350969	https://www.ebi.ac.uk/metagenomics/api/v1/analyses/MGYA00350969/file/ERR1897024_FASTQ_SSU_OTU.tsv	Root-Environmental-Aquatic-Freshwater-Lake
ERR1896664	MGYA00350609	https://www.ebi.ac.uk/metagenomics/api/v1/analyses/MGYA00350609/file/ERR1896664_FASTQ_SSU_OTU.tsv	Root-Environmental-Aquatic-Freshwater-Lake
ERR1582268	MGYA00352505	https://www.ebi.ac.uk/metagenomics/api/v1/analyses/MGYA00352505/file/ERR1582268_FASTQ_SSU_OTU.tsv	Root-Environmental-Aquatic-Freshwater-Lake
ERR1583428	MGYA00353665	https://www.ebi.ac.uk/metagenomics/api/v1/analyses/MGYA00353665/file/ERR1583428_FASTQ_SSU_OTU.tsv	Root-Environmental-Aquatic-Freshwater-Lake
ERR1743336	MGYA00087197	https://www.ebi.ac.uk/metagenomics/api/v1/analyses/MGYA00087197/file/ERR1743336_MERGED_FASTQ_otu.tsv	Root-Environmental-Aquatic-Freshwater-Lake
ERR1547109	MGYA00320820	https://www.ebi.ac.uk/metagenomics/api/v1/analyses/MGYA00320820/file/ERR1547109_FASTQ_SSU_OTU.tsv	Root-Environmental-Aquatic-Freshwater-Lake

ERR1897445	MGYA00351390	https://www.ebi.ac.uk/metagenomics/api/v1/analyses/MGYA00351390/file/ERR1897445_FASTQ_SSU_OTU.tsv	Root-Environmental-Aquatic-Freshwater-Lake
ERR1583049	MGYA00353286	https://www.ebi.ac.uk/metagenomics/api/v1/analyses/MGYA00353286/file/ERR1583049_FASTQ_SSU_OTU.tsv	Root-Environmental-Aquatic-Freshwater-Lake
ERR1582609	MGYA00352846	https://www.ebi.ac.uk/metagenomics/api/v1/analyses/MGYA00352846/file/ERR1582609_FASTQ_SSU_OTU.tsv	Root-Environmental-Aquatic-Freshwater-Lake
ERR1582497	MGYA00352734	https://www.ebi.ac.uk/metagenomics/api/v1/analyses/MGYA00352734/file/ERR1582497_FASTQ_SSU_OTU.tsv	Root-Environmental-Aquatic-Freshwater-Lake
ERR1582330	MGYA00352567	https://www.ebi.ac.uk/metagenomics/api/v1/analyses/MGYA00352567/file/ERR1582330_FASTQ_SSU_OTU.tsv	Root-Environmental-Aquatic-Freshwater-Lake
ERR1583111	MGYA00353348	https://www.ebi.ac.uk/metagenomics/api/v1/analyses/MGYA00353348/file/ERR1583111_FASTQ_SSU_OTU.tsv	Root-Environmental-Aquatic-Freshwater-Lake
ERR1314396	MGYA00095153	https://www.ebi.ac.uk/metagenomics/api/v1/analyses/MGYA00095153/file/ERR1314396_MERGED_FASTQ_otu.tsv	Root-Environmental-Aquatic-Freshwater-Lake
ERR1582751	MGYA00352988	https://www.ebi.ac.uk/metagenomics/api/v1/analyses/MGYA00352988/file/ERR1582751_FASTQ_SSU_OTU.tsv	Root-Environmental-Aquatic-Freshwater-Lake
ERR1547051	MGYA00320762	https://www.ebi.ac.uk/metagenomics/api/v1/analyses/MGYA00320762/file/ERR1547051_FASTQ_SSU_OTU.tsv	Root-Environmental-Aquatic-Freshwater-Lake
ERR1896888	MGYA00350833	https://www.ebi.ac.uk/metagenomics/api/v1/analyses/MGYA00350833/file/ERR1896888_FASTQ_SSU_OTU.tsv	Root-Environmental-Aquatic-Freshwater-Lake
ERR1547080	MGYA00320791	https://www.ebi.ac.uk/metagenomics/api/v1/analyses/MGYA00320791/file/ERR1547080_FASTQ_SSU_OTU.tsv	Root-Environmental-Aquatic-Freshwater-Lake
ERR1896859	MGYA00350804	https://www.ebi.ac.uk/metagenomics/api/v1/analyses/MGYA00350804/file/ERR1896859_FASTQ_SSU_OTU.tsv	Root-Environmental-Aquatic-Freshwater-Lake

ERR1582027	MGYA00352264	https://www.ebi.ac.uk/metagenomics/api/v1/analyses/MGYA00352264/file/ERR1582027_FASTQ_SSU_OTU.tsv	Root-Environmental-Aquatic-Freshwater-Lake
ERR1582780	MGYA00353017	https://www.ebi.ac.uk/metagenomics/api/v1/analyses/MGYA00353017/file/ERR1582780_FASTQ_SSU_OTU.tsv	Root-Environmental-Aquatic-Freshwater-Lake
ERR1314347	MGYA00095208	https://www.ebi.ac.uk/metagenomics/api/v1/analyses/MGYA00095208/file/ERR1314347_MERGED_FASTQ_otu.tsv	Root-Environmental-Aquatic-Freshwater-Lake
ERR1583206	MGYA00353443	https://www.ebi.ac.uk/metagenomics/api/v1/analyses/MGYA00353443/file/ERR1583206_FASTQ_SSU_OTU.tsv	Root-Environmental-Aquatic-Freshwater-Lake
ERR1582446	MGYA00352683	https://www.ebi.ac.uk/metagenomics/api/v1/analyses/MGYA00352683/file/ERR1582446_FASTQ_SSU_OTU.tsv	Root-Environmental-Aquatic-Freshwater-Lake
ERR1582834	MGYA00353071	https://www.ebi.ac.uk/metagenomics/api/v1/analyses/MGYA00353071/file/ERR1582834_FASTQ_SSU_OTU.tsv	Root-Environmental-Aquatic-Freshwater-Lake
ERR1582103	MGYA00352340	https://www.ebi.ac.uk/metagenomics/api/v1/analyses/MGYA00352340/file/ERR1582103_FASTQ_SSU_OTU.tsv	Root-Environmental-Aquatic-Freshwater-Lake
ERR1897089	MGYA00351034	https://www.ebi.ac.uk/metagenomics/api/v1/analyses/MGYA00351034/file/ERR1897089_FASTQ_SSU_OTU.tsv	Root-Environmental-Aquatic-Freshwater-Lake
ERR1583485	MGYA00353722	https://www.ebi.ac.uk/metagenomics/api/v1/analyses/MGYA00353722/file/ERR1583485_FASTQ_SSU_OTU.tsv	Root-Environmental-Aquatic-Freshwater-Lake
ERR1583322	MGYA00353559	https://www.ebi.ac.uk/metagenomics/api/v1/analyses/MGYA00353559/file/ERR1583322_FASTQ_SSU_OTU.tsv	Root-Environmental-Aquatic-Freshwater-Lake
ERR1582910	MGYA00353147	https://www.ebi.ac.uk/metagenomics/api/v1/analyses/MGYA00353147/file/ERR1582910_FASTQ_SSU_OTU.tsv	Root-Environmental-Aquatic-Freshwater-Lake
ERR1582562	MGYA00352799	https://www.ebi.ac.uk/metagenomics/api/v1/analyses/MGYA00352799/file/ERR1582562_FASTQ_SSU_OTU.tsv	Root-Environmental-Aquatic-Freshwater-Lake

ERR1582214	MGYA00352451	https://www.ebi.ac.uk/metagenomics/api/v1/analyses/MGYA00352451/file/ERR1582214_FASTQ_SSU_OTU.tsv	Root-Environmental-Aquatic-Freshwater-Lake
ERR1583454	MGYA00353691	https://www.ebi.ac.uk/metagenomics/api/v1/analyses/MGYA00353691/file/ERR1583454_FASTQ_SSU_OTU.tsv	Root-Environmental-Aquatic-Freshwater-Lake
ERR1897058	MGYA00351003	https://www.ebi.ac.uk/metagenomics/api/v1/analyses/MGYA00351003/file/ERR1897058_FASTQ_SSU_OTU.tsv	Root-Environmental-Aquatic-Freshwater-Lake
ERR1583035	MGYA00353272	https://www.ebi.ac.uk/metagenomics/api/v1/analyses/MGYA00353272/file/ERR1583035_FASTQ_SSU_OTU.tsv	Root-Environmental-Aquatic-Freshwater-Lake
ERR1582675	MGYA00352912	https://www.ebi.ac.uk/metagenomics/api/v1/analyses/MGYA00352912/file/ERR1582675_FASTQ_SSU_OTU.tsv	Root-Environmental-Aquatic-Freshwater-Lake
ERR1897100	MGYA00351045	https://www.ebi.ac.uk/metagenomics/api/v1/analyses/MGYA00351045/file/ERR1897100_FASTQ_SSU_OTU.tsv	Root-Environmental-Aquatic-Freshwater-Lake
ERR1896740	MGYA00350685	https://www.ebi.ac.uk/metagenomics/api/v1/analyses/MGYA00350685/file/ERR1896740_FASTQ_SSU_OTU.tsv	Root-Environmental-Aquatic-Freshwater-Lake
ERR1429269	MGYA00107644	https://www.ebi.ac.uk/metagenomics/api/v1/analyses/MGYA00107644/file/ERR1429269_FASTQ_otu.tsv	Root-Environmental-Aquatic-Freshwater-Lake
ERR1582899	MGYA00353136	https://www.ebi.ac.uk/metagenomics/api/v1/analyses/MGYA00353136/file/ERR1582899_FASTQ_SSU_OTU.tsv	Root-Environmental-Aquatic-Freshwater-Lake
ERR1897561	MGYA00351506	https://www.ebi.ac.uk/metagenomics/api/v1/analyses/MGYA00351506/file/ERR1897561_FASTQ_SSU_OTU.tsv	Root-Environmental-Aquatic-Freshwater-Lake
ERR1897217	MGYA00351162	https://www.ebi.ac.uk/metagenomics/api/v1/analyses/MGYA00351162/file/ERR1897217_FASTQ_SSU_OTU.tsv	Root-Environmental-Aquatic-Freshwater-Lake
ERR1896825	MGYA00350770	https://www.ebi.ac.uk/metagenomics/api/v1/analyses/MGYA00350770/file/ERR1896825_FASTQ_SSU_OTU.tsv	Root-Environmental-Aquatic-Freshwater-Lake

ERR1582848	MGYA00353085	https://www.ebi.ac.uk/metagenomics/api/v1/analyses/MGYA00353085/file/ERR1582848_FASTQ_SSU_OTU.tsv	Root-Environmental-Aquatic-Freshwater-Lake
ERR1896791	MGYA00350736	https://www.ebi.ac.uk/metagenomics/api/v1/analyses/MGYA00350736/file/ERR1896791_FASTQ_SSU_OTU.tsv	Root-Environmental-Aquatic-Freshwater-Lake
ERR1897676	MGYA00351621	https://www.ebi.ac.uk/metagenomics/api/v1/analyses/MGYA00351621/file/ERR1897676_FASTQ_SSU_OTU.tsv	Root-Environmental-Aquatic-Freshwater-Lake
ERR1583096	MGYA00353333	https://www.ebi.ac.uk/metagenomics/api/v1/analyses/MGYA00353333/file/ERR1583096_FASTQ_SSU_OTU.tsv	Root-Environmental-Aquatic-Freshwater-Lake
ERR1582171	MGYA00352408	https://www.ebi.ac.uk/metagenomics/api/v1/analyses/MGYA00352408/file/ERR1582171_FASTQ_SSU_OTU.tsv	Root-Environmental-Aquatic-Freshwater-Lake
SRR2443018	MGYA00359022	https://www.ebi.ac.uk/metagenomics/api/v1/analyses/MGYA00359022/file/SRR2443018_MERGED_FASTQ_SSU_OTU.tsv	Root-Environmental-Aquatic-Freshwater-Lake
ERR1582962	MGYA00353199	https://www.ebi.ac.uk/metagenomics/api/v1/analyses/MGYA00353199/file/ERR1582962_FASTQ_SSU_OTU.tsv	Root-Environmental-Aquatic-Freshwater-Lake
ERR1582510	MGYA00352747	https://www.ebi.ac.uk/metagenomics/api/v1/analyses/MGYA00352747/file/ERR1582510_FASTQ_SSU_OTU.tsv	Root-Environmental-Aquatic-Freshwater-Lake
ERR1583350	MGYA00353587	https://www.ebi.ac.uk/metagenomics/api/v1/analyses/MGYA00353587/file/ERR1583350_FASTQ_SSU_OTU.tsv	Root-Environmental-Aquatic-Freshwater-Lake
ERR1583381	MGYA00353618	https://www.ebi.ac.uk/metagenomics/api/v1/analyses/MGYA00353618/file/ERR1583381_FASTQ_SSU_OTU.tsv	Root-Environmental-Aquatic-Freshwater-Lake
ERR1583426	MGYA00353663	https://www.ebi.ac.uk/metagenomics/api/v1/analyses/MGYA00353663/file/ERR1583426_FASTQ_SSU_OTU.tsv	Root-Environmental-Aquatic-Freshwater-Lake
ERR1582266	MGYA00352503	https://www.ebi.ac.uk/metagenomics/api/v1/analyses/MGYA00352503/file/ERR1582266_FASTQ_SSU_OTU.tsv	Root-Environmental-Aquatic-Freshwater-Lake

ERR1582607	MGYA00352844	https://www.ebi.ac.uk/metagenomics/api/v1/analyses/MGYA00352844/file/ERR1582607_FASTQ_SSU_OTU.tsv	Root-Environmental-Aquatic-Freshwater-Lake
ERR1583047	MGYA00353284	https://www.ebi.ac.uk/metagenomics/api/v1/analyses/MGYA00353284/file/ERR1583047_FASTQ_SSU_OTU.tsv	Root-Environmental-Aquatic-Freshwater-Lake
ERR1743338	MGYA00087171	https://www.ebi.ac.uk/metagenomics/api/v1/analyses/MGYA00087171/file/ERR1743338_MERGED_FASTQ_otu.tsv	Root-Environmental-Aquatic-Freshwater-Lake
ERR1547107	MGYA00320818	https://www.ebi.ac.uk/metagenomics/api/v1/analyses/MGYA00320818/file/ERR1547107_FASTQ_SSU_OTU.tsv	Root-Environmental-Aquatic-Freshwater-Lake
ERR1896732	MGYA00350677	https://www.ebi.ac.uk/metagenomics/api/v1/analyses/MGYA00350677/file/ERR1896732_FASTQ_SSU_OTU.tsv	Root-Environmental-Aquatic-Freshwater-Lake
ERR1897172	MGYA00351117	https://www.ebi.ac.uk/metagenomics/api/v1/analyses/MGYA00351117/file/ERR1897172_FASTQ_SSU_OTU.tsv	Root-Environmental-Aquatic-Freshwater-Lake
ERR1582499	MGYA00352736	https://www.ebi.ac.uk/metagenomics/api/v1/analyses/MGYA00352736/file/ERR1582499_FASTQ_SSU_OTU.tsv	Root-Environmental-Aquatic-Freshwater-Lake
ERR1897513	MGYA00351458	https://www.ebi.ac.uk/metagenomics/api/v1/analyses/MGYA00351458/file/ERR1897513_FASTQ_SSU_OTU.tsv	Root-Environmental-Aquatic-Freshwater-Lake
ERR1896886	MGYA00350831	https://www.ebi.ac.uk/metagenomics/api/v1/analyses/MGYA00350831/file/ERR1896886_FASTQ_SSU_OTU.tsv	Root-Environmental-Aquatic-Freshwater-Lake
ERR658201	MGYA00103487	https://www.ebi.ac.uk/metagenomics/api/v1/analyses/MGYA00103487/file/ERR658201_FASTQ_otu.tsv	Root-Environmental-Aquatic-Freshwater-Lake
ERR1314398	MGYA00095163	https://www.ebi.ac.uk/metagenomics/api/v1/analyses/MGYA00095163/file/ERR1314398_MERGED_FASTQ_otu.tsv	Root-Environmental-Aquatic-Freshwater-Lake
ERR1582029	MGYA00352266	https://www.ebi.ac.uk/metagenomics/api/v1/analyses/MGYA00352266/file/ERR1582029_FASTQ_SSU_OTU.tsv	Root-Environmental-Aquatic-Freshwater-Lake

ERR1314349	MGYA00095201	https://www.ebi.ac.uk/metagenomics/api/v1/analyses/MGYA00095201/file/ERR1314349_MERGED_FASTQ_otu.tsv	Root-Environmental-Aquatic-Freshwater-Lake
ERR1896857	MGYA00350802	https://www.ebi.ac.uk/metagenomics/api/v1/analyses/MGYA00350802/file/ERR1896857_FASTQ_SSU_OTU.tsv	Root-Environmental-Aquatic-Freshwater-Lake
ERR1897265	MGYA00351210	https://www.ebi.ac.uk/metagenomics/api/v1/analyses/MGYA00351210/file/ERR1897265_FASTQ_SSU_OTU.tsv	Root-Environmental-Aquatic-Freshwater-Lake
ERR1582448	MGYA00352685	https://www.ebi.ac.uk/metagenomics/api/v1/analyses/MGYA00352685/file/ERR1582448_FASTQ_SSU_OTU.tsv	Root-Environmental-Aquatic-Freshwater-Lake
ERR1583208	MGYA00353445	https://www.ebi.ac.uk/metagenomics/api/v1/analyses/MGYA00353445/file/ERR1583208_FASTQ_SSU_OTU.tsv	Root-Environmental-Aquatic-Freshwater-Lake
ERR1897604	MGYA00351549	https://www.ebi.ac.uk/metagenomics/api/v1/analyses/MGYA00351549/file/ERR1897604_FASTQ_SSU_OTU.tsv	Root-Environmental-Aquatic-Freshwater-Lake
ERR1896973	MGYA00350918	https://www.ebi.ac.uk/metagenomics/api/v1/analyses/MGYA00350918/file/ERR1896973_FASTQ_SSU_OTU.tsv	Root-Environmental-Aquatic-Freshwater-Lake
SRR2442990	MGYA00358994	https://www.ebi.ac.uk/metagenomics/api/v1/analyses/MGYA00358994/file/SRR2442990_MERGED_FASTQ_SSU_OTU.tsv	Root-Environmental-Aquatic-Freshwater-Lake
ERR1897341	MGYA00351286	https://www.ebi.ac.uk/metagenomics/api/v1/analyses/MGYA00351286/file/ERR1897341_FASTQ_SSU_OTU.tsv	Root-Environmental-Aquatic-Freshwater-Lake
ERR589334	MGYA00103459	https://www.ebi.ac.uk/metagenomics/api/v1/analyses/MGYA00103459/file/ERR589334_FASTQ_otu.tsv	Root-Environmental-Aquatic-Freshwater-Lake
ERR1897720	MGYA00351665	https://www.ebi.ac.uk/metagenomics/api/v1/analyses/MGYA00351665/file/ERR1897720_FASTQ_SSU_OTU.tsv	Root-Environmental-Aquatic-Freshwater-Lake
ERR1897087	MGYA00351032	https://www.ebi.ac.uk/metagenomics/api/v1/analyses/MGYA00351032/file/ERR1897087_FASTQ_SSU_OTU.tsv	Root-Environmental-Aquatic-Freshwater-Lake

ERR1897056	MGYA00351001	https://www.ebi.ac.uk/metagenomics/api/v1/analyses/MGYA00351001/file/ERR1897056_FASTQ_SSU_OTU.tsv	Root-Environmental-Aquatic-Freshwater-Lake
ERR1897390	MGYA00351335	https://www.ebi.ac.uk/metagenomics/api/v1/analyses/MGYA00351335/file/ERR1897390_FASTQ_SSU_OTU.tsv	Root-Environmental-Aquatic-Freshwater-Lake
ERR1897437	MGYA00351382	https://www.ebi.ac.uk/metagenomics/api/v1/analyses/MGYA00351382/file/ERR1897437_FASTQ_SSU_OTU.tsv	Root-Environmental-Aquatic-Freshwater-Lake
ERR1743344	MGYA00087176	https://www.ebi.ac.uk/metagenomics/api/v1/analyses/MGYA00087176/file/ERR1743344_MERGED_FASTQ_otu.tsv	Root-Environmental-Aquatic-Freshwater-Lake
ERR1583502	MGYA00353739	https://www.ebi.ac.uk/metagenomics/api/v1/analyses/MGYA00353739/file/ERR1583502_FASTQ_SSU_OTU.tsv	Root-Environmental-Aquatic-Freshwater-Lake
ERR1429267	MGYA00107623	https://www.ebi.ac.uk/metagenomics/api/v1/analyses/MGYA00107623/file/ERR1429267_FASTQ_otu.tsv	Root-Environmental-Aquatic-Freshwater-Lake
ERR1582342	MGYA00352579	https://www.ebi.ac.uk/metagenomics/api/v1/analyses/MGYA00352579/file/ERR1582342_FASTQ_SSU_OTU.tsv	Root-Environmental-Aquatic-Freshwater-Lake
ERR1582897	MGYA00353134	https://www.ebi.ac.uk/metagenomics/api/v1/analyses/MGYA00353134/file/ERR1582897_FASTQ_SSU_OTU.tsv	Root-Environmental-Aquatic-Freshwater-Lake
ERR1535985	MGYA00320276	https://www.ebi.ac.uk/metagenomics/api/v1/analyses/MGYA00320276/file/ERR1535985_FASTQ_SSU_OTU.tsv	Root-Environmental-Aquatic-Freshwater-Lake
ERR1582723	MGYA00352960	https://www.ebi.ac.uk/metagenomics/api/v1/analyses/MGYA00352960/file/ERR1582723_FASTQ_SSU_OTU.tsv	Root-Environmental-Aquatic-Freshwater-Lake
ERR1582084	MGYA00352321	https://www.ebi.ac.uk/metagenomics/api/v1/analyses/MGYA00352321/file/ERR1582084_FASTQ_SSU_OTU.tsv	Root-Environmental-Aquatic-Freshwater-Lake
ERR1583163	MGYA00353400	https://www.ebi.ac.uk/metagenomics/api/v1/analyses/MGYA00353400/file/ERR1583163_FASTQ_SSU_OTU.tsv	Root-Environmental-Aquatic-Freshwater-Lake

ERR1897219	MGYA00351164	https://www.ebi.ac.uk/metagenomics/api/v1/analyses/MGYA00351164/file/ERR1897219_FASTQ_SSU_OTU.tsv	Root-Environmental-Aquatic-Freshwater-Lake
ERR1314335	MGYA00095150	https://www.ebi.ac.uk/metagenomics/api/v1/analyses/MGYA00095150/file/ERR1314335_MERGED_FASTQ_otu.tsv	Root-Environmental-Aquatic-Freshwater-Lake
ERR1582055	MGYA00352292	https://www.ebi.ac.uk/metagenomics/api/v1/analyses/MGYA00352292/file/ERR1582055_FASTQ_SSU_OTU.tsv	Root-Environmental-Aquatic-Freshwater-Lake
ERR1897678	MGYA00351623	https://www.ebi.ac.uk/metagenomics/api/v1/analyses/MGYA00351623/file/ERR1897678_FASTQ_SSU_OTU.tsv	Root-Environmental-Aquatic-Freshwater-Lake
ERR1535954	MGYA00320245	https://www.ebi.ac.uk/metagenomics/api/v1/analyses/MGYA00320245/file/ERR1535954_FASTQ_SSU_OTU.tsv	Root-Environmental-Aquatic-Freshwater-Lake
ERR1582393	MGYA00352630	https://www.ebi.ac.uk/metagenomics/api/v1/analyses/MGYA00352630/file/ERR1582393_FASTQ_SSU_OTU.tsv	Root-Environmental-Aquatic-Freshwater-Lake
ERR1582434	MGYA00352671	https://www.ebi.ac.uk/metagenomics/api/v1/analyses/MGYA00352671/file/ERR1582434_FASTQ_SSU_OTU.tsv	Root-Environmental-Aquatic-Freshwater-Lake
ERR1582846	MGYA00353083	https://www.ebi.ac.uk/metagenomics/api/v1/analyses/MGYA00353083/file/ERR1582846_FASTQ_SSU_OTU.tsv	Root-Environmental-Aquatic-Freshwater-Lake
ERR1333503	MGYA00107586	https://www.ebi.ac.uk/metagenomics/api/v1/analyses/MGYA00107586/file/ERR1333503_FASTQ_otu.tsv	Root-Environmental-Aquatic-Freshwater-Lake
ERR1583274	MGYA00353511	https://www.ebi.ac.uk/metagenomics/api/v1/analyses/MGYA00353511/file/ERR1583274_FASTQ_SSU_OTU.tsv	Root-Environmental-Aquatic-Freshwater-Lake
ERR589333	MGYA00103458	https://www.ebi.ac.uk/metagenomics/api/v1/analyses/MGYA00103458/file/ERR589333_FASTQ_otu.tsv	Root-Environmental-Aquatic-Freshwater-Lake
ERR1897346	MGYA00351291	https://www.ebi.ac.uk/metagenomics/api/v1/analyses/MGYA00351291/file/ERR1897346_FASTQ_SSU_OTU.tsv	Root-Environmental-Aquatic-Freshwater-Lake

SRR2442997	MGYA00359001	https://www.ebi.ac.uk/metagenomics/api/v1/analyses/MGYA00359001/file/SRR2442997_MERGED_FASTQ_SSU_OTU.tsv	Root-Environmental-Aquatic-Freshwater-Lake
ERR1743392	MGYA00087162	https://www.ebi.ac.uk/metagenomics/api/v1/analyses/MGYA00087162/file/ERR1743392_MERGED_FASTQ_otu.tsv	Root-Environmental-Aquatic-Freshwater-Lake
ERR1896974	MGYA00350919	https://www.ebi.ac.uk/metagenomics/api/v1/analyses/MGYA00350919/file/ERR1896974_FASTQ_SSU_OTU.tsv	Root-Environmental-Aquatic-Freshwater-Lake
ERR1582919	MGYA00353156	https://www.ebi.ac.uk/metagenomics/api/v1/analyses/MGYA00353156/file/ERR1582919_FASTQ_SSU_OTU.tsv	Root-Environmental-Aquatic-Freshwater-Lake
ERR1897080	MGYA00351025	https://www.ebi.ac.uk/metagenomics/api/v1/analyses/MGYA00351025/file/ERR1897080_FASTQ_SSU_OTU.tsv	Root-Environmental-Aquatic-Freshwater-Lake
ERR1897727	MGYA00351672	https://www.ebi.ac.uk/metagenomics/api/v1/analyses/MGYA00351672/file/ERR1897727_FASTQ_SSU_OTU.tsv	Root-Environmental-Aquatic-Freshwater-Lake
ERR1897051	MGYA00350996	https://www.ebi.ac.uk/metagenomics/api/v1/analyses/MGYA00350996/file/ERR1897051_FASTQ_SSU_OTU.tsv	Root-Environmental-Aquatic-Freshwater-Lake
SRR3987489	MGYA00095528	https://www.ebi.ac.uk/metagenomics/api/v1/analyses/MGYA00095528/file/SRR3987489_MERGED_FASTQ_otu.tsv	Root-Environmental-Aquatic-Freshwater-Lake
ERR1743343	MGYA00087201	https://www.ebi.ac.uk/metagenomics/api/v1/analyses/MGYA00087201/file/ERR1743343_MERGED_FASTQ_otu.tsv	Root-Environmental-Aquatic-Freshwater-Lake
ERR1897430	MGYA00351375	https://www.ebi.ac.uk/metagenomics/api/v1/analyses/MGYA00351375/file/ERR1897430_FASTQ_SSU_OTU.tsv	Root-Environmental-Aquatic-Freshwater-Lake
ERR1897397	MGYA00351342	https://www.ebi.ac.uk/metagenomics/api/v1/analyses/MGYA00351342/file/ERR1897397_FASTQ_SSU_OTU.tsv	Root-Environmental-Aquatic-Freshwater-Lake
ERR1582890	MGYA00353127	https://www.ebi.ac.uk/metagenomics/api/v1/analyses/MGYA00353127/file/ERR1582890_FASTQ_SSU_OTU.tsv	Root-Environmental-Aquatic-Freshwater-Lake

ERR1582345	MGYA00352582	https://www.ebi.ac.uk/metagenomics/api/v1/analyses/MGYA00352582/file/ERR1582345_FASTQ_SSU_OTU.tsv	Root-Environmental-Aquatic-Freshwater-Lake
ERR1429260	MGYA00107647	https://www.ebi.ac.uk/metagenomics/api/v1/analyses/MGYA00107647/file/ERR1429260_FASTQ_otu.tsv	Root-Environmental-Aquatic-Freshwater-Lake
ERR1583505	MGYA00353742	https://www.ebi.ac.uk/metagenomics/api/v1/analyses/MGYA00353742/file/ERR1583505_FASTQ_SSU_OTU.tsv	Root-Environmental-Aquatic-Freshwater-Lake
ERR1897109	MGYA00351054	https://www.ebi.ac.uk/metagenomics/api/v1/analyses/MGYA00351054/file/ERR1897109_FASTQ_SSU_OTU.tsv	Root-Environmental-Aquatic-Freshwater-Lake
ERR1535982	MGYA00320273	https://www.ebi.ac.uk/metagenomics/api/v1/analyses/MGYA00320273/file/ERR1535982_FASTQ_SSU_OTU.tsv	Root-Environmental-Aquatic-Freshwater-Lake
ERR1896749	MGYA00350694	https://www.ebi.ac.uk/metagenomics/api/v1/analyses/MGYA00350694/file/ERR1896749_FASTQ_SSU_OTU.tsv	Root-Environmental-Aquatic-Freshwater-Lake
ERR1583164	MGYA00353401	https://www.ebi.ac.uk/metagenomics/api/v1/analyses/MGYA00353401/file/ERR1583164_FASTQ_SSU_OTU.tsv	Root-Environmental-Aquatic-Freshwater-Lake
ERR1582083	MGYA00352320	https://www.ebi.ac.uk/metagenomics/api/v1/analyses/MGYA00352320/file/ERR1582083_FASTQ_SSU_OTU.tsv	Root-Environmental-Aquatic-Freshwater-Lake
ERR1582724	MGYA00352961	https://www.ebi.ac.uk/metagenomics/api/v1/analyses/MGYA00352961/file/ERR1582724_FASTQ_SSU_OTU.tsv	Root-Environmental-Aquatic-Freshwater-Lake
ERR1897568	MGYA00351513	https://www.ebi.ac.uk/metagenomics/api/v1/analyses/MGYA00351513/file/ERR1897568_FASTQ_SSU_OTU.tsv	Root-Environmental-Aquatic-Freshwater-Lake
ERR1582052	MGYA00352289	https://www.ebi.ac.uk/metagenomics/api/v1/analyses/MGYA00352289/file/ERR1582052_FASTQ_SSU_OTU.tsv	Root-Environmental-Aquatic-Freshwater-Lake
ERR1314332	MGYA00095172	https://www.ebi.ac.uk/metagenomics/api/v1/analyses/MGYA00095172/file/ERR1314332_MERGED_FASTQ_otu.tsv	Root-Environmental-Aquatic-Freshwater-Lake

ERR1896798	MGYA00350743	https://www.ebi.ac.uk/metagenomics/api/v1/analyses/MGYA00350743/file/ERR1896798_FASTQ_SSU_OTU.tsv	Root-Environmental-Aquatic-Freshwater-Lake
ERR1535953	MGYA00320244	https://www.ebi.ac.uk/metagenomics/api/v1/analyses/MGYA00320244/file/ERR1535953_FASTQ_SSU_OTU.tsv	Root-Environmental-Aquatic-Freshwater-Lake
ERR1583273	MGYA00353510	https://www.ebi.ac.uk/metagenomics/api/v1/analyses/MGYA00353510/file/ERR1583273_FASTQ_SSU_OTU.tsv	Root-Environmental-Aquatic-Freshwater-Lake
ERR1333504	MGYA00107599	https://www.ebi.ac.uk/metagenomics/api/v1/analyses/MGYA00107599/file/ERR1333504_FASTQ_otu.tsv	Root-Environmental-Aquatic-Freshwater-Lake
ERR1582841	MGYA00353078	https://www.ebi.ac.uk/metagenomics/api/v1/analyses/MGYA00353078/file/ERR1582841_FASTQ_SSU_OTU.tsv	Root-Environmental-Aquatic-Freshwater-Lake
ERR1582433	MGYA00352670	https://www.ebi.ac.uk/metagenomics/api/v1/analyses/MGYA00352670/file/ERR1582433_FASTQ_SSU_OTU.tsv	Root-Environmental-Aquatic-Freshwater-Lake
ERR1582394	MGYA00352631	https://www.ebi.ac.uk/metagenomics/api/v1/analyses/MGYA00352631/file/ERR1582394_FASTQ_SSU_OTU.tsv	Root-Environmental-Aquatic-Freshwater-Lake
ERR1896908	MGYA00350853	https://www.ebi.ac.uk/metagenomics/api/v1/analyses/MGYA00350853/file/ERR1896908_FASTQ_SSU_OTU.tsv	Root-Environmental-Aquatic-Freshwater-Lake
ERR1582176	MGYA00352413	https://www.ebi.ac.uk/metagenomics/api/v1/analyses/MGYA00352413/file/ERR1582176_FASTQ_SSU_OTU.tsv	Root-Environmental-Aquatic-Freshwater-Lake
ERR1583091	MGYA00353328	https://www.ebi.ac.uk/metagenomics/api/v1/analyses/MGYA00353328/file/ERR1583091_FASTQ_SSU_OTU.tsv	Root-Environmental-Aquatic-Freshwater-Lake
ERR1583357	MGYA00353594	https://www.ebi.ac.uk/metagenomics/api/v1/analyses/MGYA00353594/file/ERR1583357_FASTQ_SSU_OTU.tsv	Root-Environmental-Aquatic-Freshwater-Lake
ERR1582517	MGYA00352754	https://www.ebi.ac.uk/metagenomics/api/v1/analyses/MGYA00352754/file/ERR1582517_FASTQ_SSU_OTU.tsv	Root-Environmental-Aquatic-Freshwater-Lake

ERR1582965	MGYA00353202	https://www.ebi.ac.uk/metagenomics/api/v1/analyses/MGYA00353202/file/ERR1582965_FASTQ_SSU_OTU.tsv	Root-Environmental-Aquatic-Freshwater-Lake
ERR1582261	MGYA00352498	https://www.ebi.ac.uk/metagenomics/api/v1/analyses/MGYA00352498/file/ERR1582261_FASTQ_SSU_OTU.tsv	Root-Environmental-Aquatic-Freshwater-Lake
ERR1583421	MGYA00353658	https://www.ebi.ac.uk/metagenomics/api/v1/analyses/MGYA00353658/file/ERR1583421_FASTQ_SSU_OTU.tsv	Root-Environmental-Aquatic-Freshwater-Lake
ERR1583386	MGYA00353623	https://www.ebi.ac.uk/metagenomics/api/v1/analyses/MGYA00353623/file/ERR1583386_FASTQ_SSU_OTU.tsv	Root-Environmental-Aquatic-Freshwater-Lake
ERR1583040	MGYA00353277	https://www.ebi.ac.uk/metagenomics/api/v1/analyses/MGYA00353277/file/ERR1583040_FASTQ_SSU_OTU.tsv	Root-Environmental-Aquatic-Freshwater-Lake
ERR1582600	MGYA00352837	https://www.ebi.ac.uk/metagenomics/api/v1/analyses/MGYA00352837/file/ERR1582600_FASTQ_SSU_OTU.tsv	Root-Environmental-Aquatic-Freshwater-Lake
ERR1547100	MGYA00320811	https://www.ebi.ac.uk/metagenomics/api/v1/analyses/MGYA00320811/file/ERR1547100_FASTQ_SSU_OTU.tsv	Root-Environmental-Aquatic-Freshwater-Lake
ERR1897175	MGYA00351120	https://www.ebi.ac.uk/metagenomics/api/v1/analyses/MGYA00351120/file/ERR1897175_FASTQ_SSU_OTU.tsv	Root-Environmental-Aquatic-Freshwater-Lake
ERR1896735	MGYA00350680	https://www.ebi.ac.uk/metagenomics/api/v1/analyses/MGYA00350680/file/ERR1896735_FASTQ_SSU_OTU.tsv	Root-Environmental-Aquatic-Freshwater-Lake
ERR1582339	MGYA00352576	https://www.ebi.ac.uk/metagenomics/api/v1/analyses/MGYA00352576/file/ERR1582339_FASTQ_SSU_OTU.tsv	Root-Environmental-Aquatic-Freshwater-Lake
ERR1896881	MGYA00350826	https://www.ebi.ac.uk/metagenomics/api/v1/analyses/MGYA00350826/file/ERR1896881_FASTQ_SSU_OTU.tsv	Root-Environmental-Aquatic-Freshwater-Lake
ERR1547058	MGYA00320769	https://www.ebi.ac.uk/metagenomics/api/v1/analyses/MGYA00320769/file/ERR1547058_FASTQ_SSU_OTU.tsv	Root-Environmental-Aquatic-Freshwater-Lake

ERR1897514	MGYA00351459	https://www.ebi.ac.uk/metagenomics/api/v1/analyses/MGYA00351459/file/ERR1897514_FASTQ_SSU_OTU.tsv	Root-Environmental-Aquatic-Freshwater-Lake
ERR1583118	MGYA00353355	https://www.ebi.ac.uk/metagenomics/api/v1/analyses/MGYA00353355/file/ERR1583118_FASTQ_SSU_OTU.tsv	Root-Environmental-Aquatic-Freshwater-Lake
ERR1582758	MGYA00352995	https://www.ebi.ac.uk/metagenomics/api/v1/analyses/MGYA00352995/file/ERR1582758_FASTQ_SSU_OTU.tsv	Root-Environmental-Aquatic-Freshwater-Lake
ERR658206	MGYA00103497	https://www.ebi.ac.uk/metagenomics/api/v1/analyses/MGYA00103497/file/ERR658206_FASTQ_otu.tsv	Root-Environmental-Aquatic-Freshwater-Lake
ERR1582789	MGYA00353026	https://www.ebi.ac.uk/metagenomics/api/v1/analyses/MGYA00353026/file/ERR1582789_FASTQ_SSU_OTU.tsv	Root-Environmental-Aquatic-Freshwater-Lake
ERR1897262	MGYA00351207	https://www.ebi.ac.uk/metagenomics/api/v1/analyses/MGYA00351207/file/ERR1897262_FASTQ_SSU_OTU.tsv	Root-Environmental-Aquatic-Freshwater-Lake
ERR1896850	MGYA00350795	https://www.ebi.ac.uk/metagenomics/api/v1/analyses/MGYA00350795/file/ERR1896850_FASTQ_SSU_OTU.tsv	Root-Environmental-Aquatic-Freshwater-Lake
ERR1547089	MGYA00320800	https://www.ebi.ac.uk/metagenomics/api/v1/analyses/MGYA00320800/file/ERR1547089_FASTQ_SSU_OTU.tsv	Root-Environmental-Aquatic-Freshwater-Lake
ERR1897603	MGYA00351548	https://www.ebi.ac.uk/metagenomics/api/v1/analyses/MGYA00351548/file/ERR1897603_FASTQ_SSU_OTU.tsv	Root-Environmental-Aquatic-Freshwater-Lake
ERR1896990	MGYA00350935	https://www.ebi.ac.uk/metagenomics/api/v1/analyses/MGYA00350935/file/ERR1896990_FASTQ_SSU_OTU.tsv	Root-Environmental-Aquatic-Freshwater-Lake
SRR2442973	MGYA00358977	https://www.ebi.ac.uk/metagenomics/api/v1/analyses/MGYA00358977/file/SRR2442973_MERGED_FASTQ_SSU_OTU.tsv	Root-Environmental-Aquatic-Freshwater-Lake
ERR1743376	MGYA00087116	https://www.ebi.ac.uk/metagenomics/api/v1/analyses/MGYA00087116/file/ERR1743376_MERGED_FASTQ_otu.tsv	Root-Environmental-Aquatic-Freshwater-Lake

ERR1897405	MGYA00351350	https://www.ebi.ac.uk/metagenomics/api/v1/analyses/MGYA00351350/file/ERR1897405_FASTQ_SSU_OTU.tsv	Root-Environmental-Aquatic-Freshwater-Lake
ERR1583009	MGYA00353246	https://www.ebi.ac.uk/metagenomics/api/v1/analyses/MGYA00353246/file/ERR1583009_FASTQ_SSU_OTU.tsv	Root-Environmental-Aquatic-Freshwater-Lake
ERR1582649	MGYA00352886	https://www.ebi.ac.uk/metagenomics/api/v1/analyses/MGYA00352886/file/ERR1582649_FASTQ_SSU_OTU.tsv	Root-Environmental-Aquatic-Freshwater-Lake
ERR1897064	MGYA00351009	https://www.ebi.ac.uk/metagenomics/api/v1/analyses/MGYA00351009/file/ERR1897064_FASTQ_SSU_OTU.tsv	Root-Environmental-Aquatic-Freshwater-Lake
ERR1582228	MGYA00352465	https://www.ebi.ac.uk/metagenomics/api/v1/analyses/MGYA00352465/file/ERR1582228_FASTQ_SSU_OTU.tsv	Root-Environmental-Aquatic-Freshwater-Lake
ERR1583468	MGYA00353705	https://www.ebi.ac.uk/metagenomics/api/v1/analyses/MGYA00353705/file/ERR1583468_FASTQ_SSU_OTU.tsv	Root-Environmental-Aquatic-Freshwater-Lake
ERR1897712	MGYA00351657	https://www.ebi.ac.uk/metagenomics/api/v1/analyses/MGYA00351657/file/ERR1897712_FASTQ_SSU_OTU.tsv	Root-Environmental-Aquatic-Freshwater-Lake
ERR1582698	MGYA00352935	https://www.ebi.ac.uk/metagenomics/api/v1/analyses/MGYA00352935/file/ERR1582698_FASTQ_SSU_OTU.tsv	Root-Environmental-Aquatic-Freshwater-Lake
ERR1897373	MGYA00351318	https://www.ebi.ac.uk/metagenomics/api/v1/analyses/MGYA00351318/file/ERR1897373_FASTQ_SSU_OTU.tsv	Root-Environmental-Aquatic-Freshwater-Lake
ERR589306	MGYA00103464	https://www.ebi.ac.uk/metagenomics/api/v1/analyses/MGYA00103464/file/ERR589306_FASTQ_otu.tsv	Root-Environmental-Aquatic-Freshwater-Lake
ERR1896941	MGYA00350886	https://www.ebi.ac.uk/metagenomics/api/v1/analyses/MGYA00350886/file/ERR1896941_FASTQ_SSU_OTU.tsv	Root-Environmental-Aquatic-Freshwater-Lake
ERR1742291	MGYA00311592	https://www.ebi.ac.uk/metagenomics/api/v1/analyses/MGYA00311592/file/ERR1742291_FASTQ_SSU_OTU.tsv	Root-Environmental-Aquatic-Freshwater-Lake

ERR1535966	MGYA00320257	https://www.ebi.ac.uk/metagenomics/api/v1/analyses/MGYA00320257/file/ERR1535966_FASTQ_SSU_OTU.tsv	Root-Environmental-Aquatic-Freshwater-Lake
ERR1583246	MGYA00353483	https://www.ebi.ac.uk/metagenomics/api/v1/analyses/MGYA00353483/file/ERR1583246_FASTQ_SSU_OTU.tsv	Root-Environmental-Aquatic-Freshwater-Lake
ERR1582874	MGYA00353111	https://www.ebi.ac.uk/metagenomics/api/v1/analyses/MGYA00353111/file/ERR1582874_FASTQ_SSU_OTU.tsv	Root-Environmental-Aquatic-Freshwater-Lake
ERR1582406	MGYA00352643	https://www.ebi.ac.uk/metagenomics/api/v1/analyses/MGYA00352643/file/ERR1582406_FASTQ_SSU_OTU.tsv	Root-Environmental-Aquatic-Freshwater-Lake
ERR1896819	MGYA00350764	https://www.ebi.ac.uk/metagenomics/api/v1/analyses/MGYA00350764/file/ERR1896819_FASTQ_SSU_OTU.tsv	Root-Environmental-Aquatic-Freshwater-Lake
ERR1582067	MGYA00352304	https://www.ebi.ac.uk/metagenomics/api/v1/analyses/MGYA00352304/file/ERR1582067_FASTQ_SSU_OTU.tsv	Root-Environmental-Aquatic-Freshwater-Lake
ERR1583180	MGYA00353417	https://www.ebi.ac.uk/metagenomics/api/v1/analyses/MGYA00353417/file/ERR1583180_FASTQ_SSU_OTU.tsv	Root-Environmental-Aquatic-Freshwater-Lake
ERR1583151	MGYA00353388	https://www.ebi.ac.uk/metagenomics/api/v1/analyses/MGYA00353388/file/ERR1583151_FASTQ_SSU_OTU.tsv	Root-Environmental-Aquatic-Freshwater-Lake
ERR1582711	MGYA00352948	https://www.ebi.ac.uk/metagenomics/api/v1/analyses/MGYA00352948/file/ERR1582711_FASTQ_SSU_OTU.tsv	Root-Environmental-Aquatic-Freshwater-Lake
ERR1429255	MGYA00107661	https://www.ebi.ac.uk/metagenomics/api/v1/analyses/MGYA00107661/file/ERR1429255_FASTQ_otu.tsv	Root-Environmental-Aquatic-Freshwater-Lake
ERR1582370	MGYA00352607	https://www.ebi.ac.uk/metagenomics/api/v1/analyses/MGYA00352607/file/ERR1582370_FASTQ_SSU_OTU.tsv	Root-Environmental-Aquatic-Freshwater-Lake
ERR658189	MGYA00103479	https://www.ebi.ac.uk/metagenomics/api/v1/analyses/MGYA00103479/file/ERR658189_FASTQ_otu.tsv	Root-Environmental-Aquatic-Freshwater-Lake

ERR1583297	MGYA00353534	https://www.ebi.ac.uk/metagenomics/api/v1/analyses/MGYA00353534/file/ERR1583297_FASTQ_SSU_OTU.tsv	Root-Environmental-Aquatic-Freshwater-Lake
ERR1583075	MGYA00353312	https://www.ebi.ac.uk/metagenomics/api/v1/analyses/MGYA00353312/file/ERR1583075_FASTQ_SSU_OTU.tsv	Root-Environmental-Aquatic-Freshwater-Lake
ERR1582192	MGYA00352429	https://www.ebi.ac.uk/metagenomics/api/v1/analyses/MGYA00352429/file/ERR1582192_FASTQ_SSU_OTU.tsv	Root-Environmental-Aquatic-Freshwater-Lake
ERR1582635	MGYA00352872	https://www.ebi.ac.uk/metagenomics/api/v1/analyses/MGYA00352872/file/ERR1582635_FASTQ_SSU_OTU.tsv	Root-Environmental-Aquatic-Freshwater-Lake
ERR1897479	MGYA00351424	https://www.ebi.ac.uk/metagenomics/api/v1/analyses/MGYA00351424/file/ERR1897479_FASTQ_SSU_OTU.tsv	Root-Environmental-Aquatic-Freshwater-Lake
ERR430927	MGYA00103257	https://www.ebi.ac.uk/metagenomics/api/v1/analyses/MGYA00103257/file/ERR430927_FASTQ_otu.tsv	Root-Environmental-Aquatic-Freshwater-Lake
ERR1582981	MGYA00353218	https://www.ebi.ac.uk/metagenomics/api/v1/analyses/MGYA00353218/file/ERR1582981_FASTQ_SSU_OTU.tsv	Root-Environmental-Aquatic-Freshwater-Lake
ERR1582254	MGYA00352491	https://www.ebi.ac.uk/metagenomics/api/v1/analyses/MGYA00352491/file/ERR1582254_FASTQ_SSU_OTU.tsv	Root-Environmental-Aquatic-Freshwater-Lake
ERR1583414	MGYA00353651	https://www.ebi.ac.uk/metagenomics/api/v1/analyses/MGYA00353651/file/ERR1583414_FASTQ_SSU_OTU.tsv	Root-Environmental-Aquatic-Freshwater-Lake
ERR1897018	MGYA00350963	https://www.ebi.ac.uk/metagenomics/api/v1/analyses/MGYA00350963/file/ERR1897018_FASTQ_SSU_OTU.tsv	Root-Environmental-Aquatic-Freshwater-Lake
ERR1896658	MGYA00350603	https://www.ebi.ac.uk/metagenomics/api/v1/analyses/MGYA00350603/file/ERR1896658_FASTQ_SSU_OTU.tsv	Root-Environmental-Aquatic-Freshwater-Lake
ERR1896689	MGYA00350634	https://www.ebi.ac.uk/metagenomics/api/v1/analyses/MGYA00350634/file/ERR1896689_FASTQ_SSU_OTU.tsv	Root-Environmental-Aquatic-Freshwater-Lake

ERR1583362	MGYA00353599	https://www.ebi.ac.uk/metagenomics/api/v1/analyses/MGYA00353599/file/ERR1583362_FASTQ_SSU_OTU.tsv	Root-Environmental-Aquatic-Freshwater-Lake
ERR1990499	MGYA00128206	https://www.ebi.ac.uk/metagenomics/api/v1/analyses/MGYA00128206/file/ERR1990499_MERGED_FASTQ_otu.tsv	Root-Environmental-Aquatic-Freshwater-Lake
ERR1582522	MGYA00352759	https://www.ebi.ac.uk/metagenomics/api/v1/analyses/MGYA00352759/file/ERR1582522_FASTQ_SSU_OTU.tsv	Root-Environmental-Aquatic-Freshwater-Lake
ERR1582950	MGYA00353187	https://www.ebi.ac.uk/metagenomics/api/v1/analyses/MGYA00353187/file/ERR1582950_FASTQ_SSU_OTU.tsv	Root-Environmental-Aquatic-Freshwater-Lake
ERR1582285	MGYA00352522	https://www.ebi.ac.uk/metagenomics/api/v1/analyses/MGYA00352522/file/ERR1582285_FASTQ_SSU_OTU.tsv	Root-Environmental-Aquatic-Freshwater-Lake
ERR1582143	MGYA00352380	https://www.ebi.ac.uk/metagenomics/api/v1/analyses/MGYA00352380/file/ERR1582143_FASTQ_SSU_OTU.tsv	Root-Environmental-Aquatic-Freshwater-Lake
ERR1582808	MGYA00353045	https://www.ebi.ac.uk/metagenomics/api/v1/analyses/MGYA00353045/file/ERR1582808_FASTQ_SSU_OTU.tsv	Root-Environmental-Aquatic-Freshwater-Lake
ERR1897191	MGYA00351136	https://www.ebi.ac.uk/metagenomics/api/v1/analyses/MGYA00351136/file/ERR1897191_FASTQ_SSU_OTU.tsv	Root-Environmental-Aquatic-Freshwater-Lake
ERR1897636	MGYA00351581	https://www.ebi.ac.uk/metagenomics/api/v1/analyses/MGYA00351581/file/ERR1897636_FASTQ_SSU_OTU.tsv	Root-Environmental-Aquatic-Freshwater-Lake
ERR1897257	MGYA00351202	https://www.ebi.ac.uk/metagenomics/api/v1/analyses/MGYA00351202/file/ERR1897257_FASTQ_SSU_OTU.tsv	Root-Environmental-Aquatic-Freshwater-Lake
ERR1743283	MGYA00087195	https://www.ebi.ac.uk/metagenomics/api/v1/analyses/MGYA00087195/file/ERR1743283_MERGED_FASTQ_otu.tsv	Root-Environmental-Aquatic-Freshwater-Lake
ERR1896865	MGYA00350810	https://www.ebi.ac.uk/metagenomics/api/v1/analyses/MGYA00350810/file/ERR1896865_FASTQ_SSU_OTU.tsv	Root-Environmental-Aquatic-Freshwater-Lake

ERR1897521	MGYA00351466	https://www.ebi.ac.uk/metagenomics/api/v1/analyses/MGYA00351466/file/ERR1897521_FASTQ_SSU_OTU.tsv	Root-Environmental-Aquatic-Freshwater-Lake
ERR1897286	MGYA00351231	https://www.ebi.ac.uk/metagenomics/api/v1/analyses/MGYA00351231/file/ERR1897286_FASTQ_SSU_OTU.tsv	Root-Environmental-Aquatic-Freshwater-Lake
ERR1897140	MGYA00351085	https://www.ebi.ac.uk/metagenomics/api/v1/analyses/MGYA00351085/file/ERR1897140_FASTQ_SSU_OTU.tsv	Root-Environmental-Aquatic-Freshwater-Lake
ERR1896700	MGYA00350645	https://www.ebi.ac.uk/metagenomics/api/v1/analyses/MGYA00350645/file/ERR1896700_FASTQ_SSU_OTU.tsv	Root-Environmental-Aquatic-Freshwater-Lake
ERR1429229	MGYA00107670	https://www.ebi.ac.uk/metagenomics/api/v1/analyses/MGYA00107670/file/ERR1429229_FASTQ_otu.tsv	Root-Environmental-Aquatic-Freshwater-Lake
ERR1582632	MGYA00352869	https://www.ebi.ac.uk/metagenomics/api/v1/analyses/MGYA00352869/file/ERR1582632_FASTQ_SSU_OTU.tsv	Root-Environmental-Aquatic-Freshwater-Lake
ERR1582195	MGYA00352432	https://www.ebi.ac.uk/metagenomics/api/v1/analyses/MGYA00352432/file/ERR1582195_FASTQ_SSU_OTU.tsv	Root-Environmental-Aquatic-Freshwater-Lake
ERR1583072	MGYA00353309	https://www.ebi.ac.uk/metagenomics/api/v1/analyses/MGYA00353309/file/ERR1583072_FASTQ_SSU_OTU.tsv	Root-Environmental-Aquatic-Freshwater-Lake
ERR1583413	MGYA00353650	https://www.ebi.ac.uk/metagenomics/api/v1/analyses/MGYA00353650/file/ERR1583413_FASTQ_SSU_OTU.tsv	Root-Environmental-Aquatic-Freshwater-Lake
ERR1582253	MGYA00352490	https://www.ebi.ac.uk/metagenomics/api/v1/analyses/MGYA00352490/file/ERR1582253_FASTQ_SSU_OTU.tsv	Root-Environmental-Aquatic-Freshwater-Lake
ERR430920	MGYA00103247	https://www.ebi.ac.uk/metagenomics/api/v1/analyses/MGYA00103247/file/ERR430920_FASTQ_otu.tsv	Root-Environmental-Aquatic-Freshwater-Lake
ERR1582986	MGYA00353223	https://www.ebi.ac.uk/metagenomics/api/v1/analyses/MGYA00353223/file/ERR1582986_FASTQ_SSU_OTU.tsv	Root-Environmental-Aquatic-Freshwater-Lake

ERR1897769	MGYA00351714	https://www.ebi.ac.uk/metagenomics/api/v1/analyses/MGYA00351714/file/ERR1897769_FASTQ_SSU_OTU.tsv	Root-Environmental-Aquatic-Freshwater-Lake
ERR1582282	MGYA00352519	https://www.ebi.ac.uk/metagenomics/api/v1/analyses/MGYA00352519/file/ERR1582282_FASTQ_SSU_OTU.tsv	Root-Environmental-Aquatic-Freshwater-Lake
ERR1582957	MGYA00353194	https://www.ebi.ac.uk/metagenomics/api/v1/analyses/MGYA00353194/file/ERR1582957_FASTQ_SSU_OTU.tsv	Root-Environmental-Aquatic-Freshwater-Lake
ERR1582525	MGYA00352762	https://www.ebi.ac.uk/metagenomics/api/v1/analyses/MGYA00352762/file/ERR1582525_FASTQ_SSU_OTU.tsv	Root-Environmental-Aquatic-Freshwater-Lake
ERR1750013	MGYA00089085	https://www.ebi.ac.uk/metagenomics/api/v1/analyses/MGYA00089085/file/ERR1750013_FASTQ_otu.tsv	Root-Environmental-Aquatic-Freshwater-Lake
ERR1583365	MGYA00353602	https://www.ebi.ac.uk/metagenomics/api/v1/analyses/MGYA00353602/file/ERR1583365_FASTQ_SSU_OTU.tsv	Root-Environmental-Aquatic-Freshwater-Lake
ERR1897308	MGYA00351253	https://www.ebi.ac.uk/metagenomics/api/v1/analyses/MGYA00351253/file/ERR1897308_FASTQ_SSU_OTU.tsv	Root-Environmental-Aquatic-Freshwater-Lake
ERR1582144	MGYA00352381	https://www.ebi.ac.uk/metagenomics/api/v1/analyses/MGYA00352381/file/ERR1582144_FASTQ_SSU_OTU.tsv	Root-Environmental-Aquatic-Freshwater-Lake
ERR1897631	MGYA00351576	https://www.ebi.ac.uk/metagenomics/api/v1/analyses/MGYA00351576/file/ERR1897631_FASTQ_SSU_OTU.tsv	Root-Environmental-Aquatic-Freshwater-Lake
ERR1897196	MGYA00351141	https://www.ebi.ac.uk/metagenomics/api/v1/analyses/MGYA00351141/file/ERR1897196_FASTQ_SSU_OTU.tsv	Root-Environmental-Aquatic-Freshwater-Lake
ERR1896862	MGYA00350807	https://www.ebi.ac.uk/metagenomics/api/v1/analyses/MGYA00350807/file/ERR1896862_FASTQ_SSU_OTU.tsv	Root-Environmental-Aquatic-Freshwater-Lake
ERR1743284	MGYA00087137	https://www.ebi.ac.uk/metagenomics/api/v1/analyses/MGYA00087137/file/ERR1743284_MERGED_FASTQ_otu.tsv	Root-Environmental-Aquatic-Freshwater-Lake

ERR1897250	MGYA00351195	https://www.ebi.ac.uk/metagenomics/api/v1/analyses/MGYA00351195/file/ERR1897250_FASTQ_SSU_OTU.tsv	Root-Environmental-Aquatic-Freshwater-Lake
ERR1897281	MGYA00351226	https://www.ebi.ac.uk/metagenomics/api/v1/analyses/MGYA00351226/file/ERR1897281_FASTQ_SSU_OTU.tsv	Root-Environmental-Aquatic-Freshwater-Lake
ERR1897526	MGYA00351471	https://www.ebi.ac.uk/metagenomics/api/v1/analyses/MGYA00351471/file/ERR1897526_FASTQ_SSU_OTU.tsv	Root-Environmental-Aquatic-Freshwater-Lake
ERR1554592	MGYA00100725	https://www.ebi.ac.uk/metagenomics/api/v1/analyses/MGYA00100725/file/ERR1554592_MERGED_FASTQ_otu.tsv	Root-Environmental-Aquatic-Freshwater-Lake
ERR1896707	MGYA00350652	https://www.ebi.ac.uk/metagenomics/api/v1/analyses/MGYA00350652/file/ERR1896707_FASTQ_SSU_OTU.tsv	Root-Environmental-Aquatic-Freshwater-Lake
ERR1897147	MGYA00351092	https://www.ebi.ac.uk/metagenomics/api/v1/analyses/MGYA00351092/file/ERR1897147_FASTQ_SSU_OTU.tsv	Root-Environmental-Aquatic-Freshwater-Lake
ERR1897402	MGYA00351347	https://www.ebi.ac.uk/metagenomics/api/v1/analyses/MGYA00351347/file/ERR1897402_FASTQ_SSU_OTU.tsv	Root-Environmental-Aquatic-Freshwater-Lake
ERR1743371	MGYA00087182	https://www.ebi.ac.uk/metagenomics/api/v1/analyses/MGYA00087182/file/ERR1743371_MERGED_FASTQ_otu.tsv	Root-Environmental-Aquatic-Freshwater-Lake
SRR2442974	MGYA00358978	https://www.ebi.ac.uk/metagenomics/api/v1/analyses/MGYA00358978/file/SRR2442974_MERGED_FASTQ_SSU_OTU.tsv	Root-Environmental-Aquatic-Freshwater-Lake
ERR1896997	MGYA00350942	https://www.ebi.ac.uk/metagenomics/api/v1/analyses/MGYA00350942/file/ERR1896997_FASTQ_SSU_OTU.tsv	Root-Environmental-Aquatic-Freshwater-Lake
ERR1897063	MGYA00351008	https://www.ebi.ac.uk/metagenomics/api/v1/analyses/MGYA00351008/file/ERR1897063_FASTQ_SSU_OTU.tsv	Root-Environmental-Aquatic-Freshwater-Lake
ERR1582588	MGYA00352825	https://www.ebi.ac.uk/metagenomics/api/v1/analyses/MGYA00352825/file/ERR1582588_FASTQ_SSU_OTU.tsv	Root-Environmental-Aquatic-Freshwater-Lake

ERR1582559	MGYA00352796	https://www.ebi.ac.uk/metagenomics/api/v1/analyses/MGYA00352796/file/ERR1582559_FASTQ_SSU_OTU.tsv	Root-Environmental-Aquatic-Freshwater-Lake
ERR1583319	MGYA00353556	https://www.ebi.ac.uk/metagenomics/api/v1/analyses/MGYA00353556/file/ERR1583319_FASTQ_SSU_OTU.tsv	Root-Environmental-Aquatic-Freshwater-Lake
ERR1897715	MGYA00351660	https://www.ebi.ac.uk/metagenomics/api/v1/analyses/MGYA00351660/file/ERR1897715_FASTQ_SSU_OTU.tsv	Root-Environmental-Aquatic-Freshwater-Lake
ERR1582138	MGYA00352375	https://www.ebi.ac.uk/metagenomics/api/v1/analyses/MGYA00352375/file/ERR1582138_FASTQ_SSU_OTU.tsv	Root-Environmental-Aquatic-Freshwater-Lake
ERR1896946	MGYA00350891	https://www.ebi.ac.uk/metagenomics/api/v1/analyses/MGYA00350891/file/ERR1896946_FASTQ_SSU_OTU.tsv	Root-Environmental-Aquatic-Freshwater-Lake
ERR589301	MGYA00103435	https://www.ebi.ac.uk/metagenomics/api/v1/analyses/MGYA00103435/file/ERR589301_FASTQ_otu.tsv	Root-Environmental-Aquatic-Freshwater-Lake
ERR1897374	MGYA00351319	https://www.ebi.ac.uk/metagenomics/api/v1/analyses/MGYA00351319/file/ERR1897374_FASTQ_SSU_OTU.tsv	Root-Environmental-Aquatic-Freshwater-Lake
ERR1535961	MGYA00320252	https://www.ebi.ac.uk/metagenomics/api/v1/analyses/MGYA00320252/file/ERR1535961_FASTQ_SSU_OTU.tsv	Root-Environmental-Aquatic-Freshwater-Lake
ERR1742296	MGYA00311597	https://www.ebi.ac.uk/metagenomics/api/v1/analyses/MGYA00311597/file/ERR1742296_FASTQ_SSU_OTU.tsv	Root-Environmental-Aquatic-Freshwater-Lake
ERR1582401	MGYA00352638	https://www.ebi.ac.uk/metagenomics/api/v1/analyses/MGYA00352638/file/ERR1582401_FASTQ_SSU_OTU.tsv	Root-Environmental-Aquatic-Freshwater-Lake
ERR1582873	MGYA00353110	https://www.ebi.ac.uk/metagenomics/api/v1/analyses/MGYA00353110/file/ERR1582873_FASTQ_SSU_OTU.tsv	Root-Environmental-Aquatic-Freshwater-Lake
ERR1583241	MGYA00353478	https://www.ebi.ac.uk/metagenomics/api/v1/analyses/MGYA00353478/file/ERR1583241_FASTQ_SSU_OTU.tsv	Root-Environmental-Aquatic-Freshwater-Lake

SRR7081390	MGYA00414438	https://www.ebi.ac.uk/metagenomics/api/v1/analyses/MGYA00414438/file/SRR7081390_MERGED_FASTQ_SSU_OTU.tsv	Root-Environmental-Aquatic-Freshwater-Lake
ERR1583187	MGYA00353424	https://www.ebi.ac.uk/metagenomics/api/v1/analyses/MGYA00353424/file/ERR1583187_FASTQ_SSU_OTU.tsv	Root-Environmental-Aquatic-Freshwater-Lake
ERR1582060	MGYA00352297	https://www.ebi.ac.uk/metagenomics/api/v1/analyses/MGYA00352297/file/ERR1582060_FASTQ_SSU_OTU.tsv	Root-Environmental-Aquatic-Freshwater-Lake
ERR1582716	MGYA00352953	https://www.ebi.ac.uk/metagenomics/api/v1/analyses/MGYA00352953/file/ERR1582716_FASTQ_SSU_OTU.tsv	Root-Environmental-Aquatic-Freshwater-Lake
ERR1583156	MGYA00353393	https://www.ebi.ac.uk/metagenomics/api/v1/analyses/MGYA00353393/file/ERR1583156_FASTQ_SSU_OTU.tsv	Root-Environmental-Aquatic-Freshwater-Lake
ERR589288	MGYA00103414	https://www.ebi.ac.uk/metagenomics/api/v1/analyses/MGYA00103414/file/ERR589288_FASTQ_otu.tsv	Root-Environmental-Aquatic-Freshwater-Lake
SRR7081341	MGYA00413549	https://www.ebi.ac.uk/metagenomics/api/v1/analyses/MGYA00413549/file/SRR7081341_MERGED_FASTQ_SSU_OTU.tsv	Root-Environmental-Aquatic-Freshwater-Lake
ERR1583290	MGYA00353527	https://www.ebi.ac.uk/metagenomics/api/v1/analyses/MGYA00353527/file/ERR1583290_FASTQ_SSU_OTU.tsv	Root-Environmental-Aquatic-Freshwater-Lake
ERR504688	MGYA00103199	https://www.ebi.ac.uk/metagenomics/api/v1/analyses/MGYA00103199/file/ERR504688_FASTQ_otu.tsv	Root-Environmental-Aquatic-Freshwater-Lake
ERR1582377	MGYA00352614	https://www.ebi.ac.uk/metagenomics/api/v1/analyses/MGYA00352614/file/ERR1582377_FASTQ_SSU_OTU.tsv	Root-Environmental-Aquatic-Freshwater-Lake
ERR1429252	MGYA00107669	https://www.ebi.ac.uk/metagenomics/api/v1/analyses/MGYA00107669/file/ERR1429252_FASTQ_otu.tsv	Root-Environmental-Aquatic-Freshwater-Lake
ERR1743303	MGYA00087166	https://www.ebi.ac.uk/metagenomics/api/v1/analyses/MGYA00087166/file/ERR1743303_MERGED_FASTQ_otu.tsv	Root-Environmental-Aquatic-Freshwater-Lake

ERR1897470	MGYA00351415	https://www.ebi.ac.uk/metagenomics/api/v1/analyses/MGYA00351415/file/ERR1897470_FASTQ_SSU_OTU.tsv	Root-Environmental-Aquatic-Freshwater-Lake
ERR1897011	MGYA00350956	https://www.ebi.ac.uk/metagenomics/api/v1/analyses/MGYA00350956/file/ERR1897011_FASTQ_SSU_OTU.tsv	Root-Environmental-Aquatic-Freshwater-Lake
ERR1896651	MGYA00350596	https://www.ebi.ac.uk/metagenomics/api/v1/analyses/MGYA00350596/file/ERR1896651_FASTQ_SSU_OTU.tsv	Root-Environmental-Aquatic-Freshwater-Lake
ERR1582988	MGYA00353225	https://www.ebi.ac.uk/metagenomics/api/v1/analyses/MGYA00353225/file/ERR1582988_FASTQ_SSU_OTU.tsv	Root-Environmental-Aquatic-Freshwater-Lake
ERR1990490	MGYA00128210	https://www.ebi.ac.uk/metagenomics/api/v1/analyses/MGYA00128210/file/ERR1990490_MERGED_FASTQ_otu.tsv	Root-Environmental-Aquatic-Freshwater-Lake
ERR1582959	MGYA00353196	https://www.ebi.ac.uk/metagenomics/api/v1/analyses/MGYA00353196/file/ERR1582959_FASTQ_SSU_OTU.tsv	Root-Environmental-Aquatic-Freshwater-Lake
ERR1896680	MGYA00350625	https://www.ebi.ac.uk/metagenomics/api/v1/analyses/MGYA00350625/file/ERR1896680_FASTQ_SSU_OTU.tsv	Root-Environmental-Aquatic-Freshwater-Lake
SRR2443023	MGYA00359027	https://www.ebi.ac.uk/metagenomics/api/v1/analyses/MGYA00359027/file/SRR2443023_MERGED_FASTQ_SSU_OTU.tsv	Root-Environmental-Aquatic-Freshwater-Lake
ERR1897767	MGYA00351712	https://www.ebi.ac.uk/metagenomics/api/v1/analyses/MGYA00351712/file/ERR1897767_FASTQ_SSU_OTU.tsv	Root-Environmental-Aquatic-Freshwater-Lake
SRR3987089	MGYA00095521	https://www.ebi.ac.uk/metagenomics/api/v1/analyses/MGYA00095521/file/SRR3987089_MERGED_FASTQ_otu.tsv	Root-Environmental-Aquatic-Freshwater-Lake
ERR1897306	MGYA00351251	https://www.ebi.ac.uk/metagenomics/api/v1/analyses/MGYA00351251/file/ERR1897306_FASTQ_SSU_OTU.tsv	Root-Environmental-Aquatic-Freshwater-Lake
ERR1896934	MGYA00350879	https://www.ebi.ac.uk/metagenomics/api/v1/analyses/MGYA00350879/file/ERR1896934_FASTQ_SSU_OTU.tsv	Root-Environmental-Aquatic-Freshwater-Lake

ERR1897198	MGYA00351143	https://www.ebi.ac.uk/metagenomics/api/v1/analyses/MGYA00351143/file/ERR1897198_FASTQ_SSU_OTU.tsv	Root-Environmental-Aquatic-Freshwater-Lake
SRR7081024	MGYA00427373	https://www.ebi.ac.uk/metagenomics/api/v1/analyses/MGYA00427373/file/SRR7081024_MERGED_FASTQ_SSU_OTU.tsv	Root-Environmental-Aquatic-Freshwater-Lake
ERR1583233	MGYA00353470	https://www.ebi.ac.uk/metagenomics/api/v1/analyses/MGYA00353470/file/ERR1583233_FASTQ_SSU_OTU.tsv	Root-Environmental-Aquatic-Freshwater-Lake
ERR1582473	MGYA00352710	https://www.ebi.ac.uk/metagenomics/api/v1/analyses/MGYA00352710/file/ERR1582473_FASTQ_SSU_OTU.tsv	Root-Environmental-Aquatic-Freshwater-Lake
ERR1582801	MGYA00353038	https://www.ebi.ac.uk/metagenomics/api/v1/analyses/MGYA00353038/file/ERR1582801_FASTQ_SSU_OTU.tsv	Root-Environmental-Aquatic-Freshwater-Lake
ERR1314372	MGYA00095157	https://www.ebi.ac.uk/metagenomics/api/v1/analyses/MGYA00095157/file/ERR1314372_MERGED_FASTQ_otu.tsv	Root-Environmental-Aquatic-Freshwater-Lake
ERR1583124	MGYA00353361	https://www.ebi.ac.uk/metagenomics/api/v1/analyses/MGYA00353361/file/ERR1583124_FASTQ_SSU_OTU.tsv	Root-Environmental-Aquatic-Freshwater-Lake
ERR1582764	MGYA00353001	https://www.ebi.ac.uk/metagenomics/api/v1/analyses/MGYA00353001/file/ERR1582764_FASTQ_SSU_OTU.tsv	Root-Environmental-Aquatic-Freshwater-Lake
ERR1314404	MGYA00095170	https://www.ebi.ac.uk/metagenomics/api/v1/analyses/MGYA00095170/file/ERR1314404_MERGED_FASTQ_otu.tsv	Root-Environmental-Aquatic-Freshwater-Lake
ERR1547064	MGYA00320775	https://www.ebi.ac.uk/metagenomics/api/v1/analyses/MGYA00320775/file/ERR1547064_FASTQ_SSU_OTU.tsv	Root-Environmental-Aquatic-Freshwater-Lake
SRR7081333	MGYA00424193	https://www.ebi.ac.uk/metagenomics/api/v1/analyses/MGYA00424193/file/SRR7081333_MERGED_FASTQ_SSU_OTU.tsv	Root-Environmental-Aquatic-Freshwater-Lake
ERR1897528	MGYA00351473	https://www.ebi.ac.uk/metagenomics/api/v1/analyses/MGYA00351473/file/ERR1897528_FASTQ_SSU_OTU.tsv	Root-Environmental-Aquatic-Freshwater-Lake

ERR1582305	MGYA00352542	https://www.ebi.ac.uk/metagenomics/api/v1/analyses/MGYA00352542/file/ERR1582305_FASTQ_SSU_OTU.tsv	Root-Environmental-Aquatic-Freshwater-Lake
ERR1897149	MGYA00351094	https://www.ebi.ac.uk/metagenomics/api/v1/analyses/MGYA00351094/file/ERR1897149_FASTQ_SSU_OTU.tsv	Root-Environmental-Aquatic-Freshwater-Lake
ERR1896709	MGYA00350654	https://www.ebi.ac.uk/metagenomics/api/v1/analyses/MGYA00350654/file/ERR1896709_FASTQ_SSU_OTU.tsv	Root-Environmental-Aquatic-Freshwater-Lake
ERR1583000	MGYA00353237	https://www.ebi.ac.uk/metagenomics/api/v1/analyses/MGYA00353237/file/ERR1583000_FASTQ_SSU_OTU.tsv	Root-Environmental-Aquatic-Freshwater-Lake
ERR1582640	MGYA00352877	https://www.ebi.ac.uk/metagenomics/api/v1/analyses/MGYA00352877/file/ERR1582640_FASTQ_SSU_OTU.tsv	Root-Environmental-Aquatic-Freshwater-Lake
ERR1896999	MGYA00350944	https://www.ebi.ac.uk/metagenomics/api/v1/analyses/MGYA00350944/file/ERR1896999_FASTQ_SSU_OTU.tsv	Root-Environmental-Aquatic-Freshwater-Lake
ERR1582586	MGYA00352823	https://www.ebi.ac.uk/metagenomics/api/v1/analyses/MGYA00352823/file/ERR1582586_FASTQ_SSU_OTU.tsv	Root-Environmental-Aquatic-Freshwater-Lake
ERR1582221	MGYA00352458	https://www.ebi.ac.uk/metagenomics/api/v1/analyses/MGYA00352458/file/ERR1582221_FASTQ_SSU_OTU.tsv	Root-Environmental-Aquatic-Freshwater-Lake
ERR1583461	MGYA00353698	https://www.ebi.ac.uk/metagenomics/api/v1/analyses/MGYA00353698/file/ERR1583461_FASTQ_SSU_OTU.tsv	Root-Environmental-Aquatic-Freshwater-Lake
ERR1583317	MGYA00353554	https://www.ebi.ac.uk/metagenomics/api/v1/analyses/MGYA00353554/file/ERR1583317_FASTQ_SSU_OTU.tsv	Root-Environmental-Aquatic-Freshwater-Lake
ERR1582925	MGYA00353162	https://www.ebi.ac.uk/metagenomics/api/v1/analyses/MGYA00353162/file/ERR1582925_FASTQ_SSU_OTU.tsv	Root-Environmental-Aquatic-Freshwater-Lake
ERR1582557	MGYA00352794	https://www.ebi.ac.uk/metagenomics/api/v1/analyses/MGYA00352794/file/ERR1582557_FASTQ_SSU_OTU.tsv	Root-Environmental-Aquatic-Freshwater-Lake

ERR1896948	MGYA00350893	https://www.ebi.ac.uk/metagenomics/api/v1/analyses/MGYA00350893/file/ERR1896948_FASTQ_SSU_OTU.tsv	Root-Environmental-Aquatic-Freshwater-Lake
ERR1582136	MGYA00352373	https://www.ebi.ac.uk/metagenomics/api/v1/analyses/MGYA00352373/file/ERR1582136_FASTQ_SSU_OTU.tsv	Root-Environmental-Aquatic-Freshwater-Lake
ERR1582691	MGYA00352928	https://www.ebi.ac.uk/metagenomics/api/v1/analyses/MGYA00352928/file/ERR1582691_FASTQ_SSU_OTU.tsv	Root-Environmental-Aquatic-Freshwater-Lake
ERR1742298	MGYA00311599	https://www.ebi.ac.uk/metagenomics/api/v1/analyses/MGYA00311599/file/ERR1742298_FASTQ_SSU_OTU.tsv	Root-Environmental-Aquatic-Freshwater-Lake
ERR1897643	MGYA00351588	https://www.ebi.ac.uk/metagenomics/api/v1/analyses/MGYA00351588/file/ERR1897643_FASTQ_SSU_OTU.tsv	Root-Environmental-Aquatic-Freshwater-Lake
ERR1583189	MGYA00353426	https://www.ebi.ac.uk/metagenomics/api/v1/analyses/MGYA00353426/file/ERR1583189_FASTQ_SSU_OTU.tsv	Root-Environmental-Aquatic-Freshwater-Lake
ERR1897585	MGYA00351530	https://www.ebi.ac.uk/metagenomics/api/v1/analyses/MGYA00351530/file/ERR1897585_FASTQ_SSU_OTU.tsv	Root-Environmental-Aquatic-Freshwater-Lake
ERR1897222	MGYA00351167	https://www.ebi.ac.uk/metagenomics/api/v1/analyses/MGYA00351167/file/ERR1897222_FASTQ_SSU_OTU.tsv	Root-Environmental-Aquatic-Freshwater-Lake
ERR1896810	MGYA00350755	https://www.ebi.ac.uk/metagenomics/api/v1/analyses/MGYA00350755/file/ERR1896810_FASTQ_SSU_OTU.tsv	Root-Environmental-Aquatic-Freshwater-Lake
ERR589286	MGYA00103443	https://www.ebi.ac.uk/metagenomics/api/v1/analyses/MGYA00103443/file/ERR589286_FASTQ_otu.tsv	Root-Environmental-Aquatic-Freshwater-Lake
ERR1897554	MGYA00351499	https://www.ebi.ac.uk/metagenomics/api/v1/analyses/MGYA00351499/file/ERR1897554_FASTQ_SSU_OTU.tsv	Root-Environmental-Aquatic-Freshwater-Lake
ERR1583158	MGYA00353395	https://www.ebi.ac.uk/metagenomics/api/v1/analyses/MGYA00353395/file/ERR1583158_FASTQ_SSU_OTU.tsv	Root-Environmental-Aquatic-Freshwater-Lake

ERR1582718	MGYA00352955	https://www.ebi.ac.uk/metagenomics/api/v1/analyses/MGYA00352955/file/ERR1582718_FASTQ_SSU_OTU.tsv	Root-Environmental-Aquatic-Freshwater-Lake
ERR1897135	MGYA00351080	https://www.ebi.ac.uk/metagenomics/api/v1/analyses/MGYA00351080/file/ERR1897135_FASTQ_SSU_OTU.tsv	Root-Environmental-Aquatic-Freshwater-Lake
ERR1897692	MGYA00351637	https://www.ebi.ac.uk/metagenomics/api/v1/analyses/MGYA00351637/file/ERR1897692_FASTQ_SSU_OTU.tsv	Root-Environmental-Aquatic-Freshwater-Lake
ERR1896775	MGYA00350720	https://www.ebi.ac.uk/metagenomics/api/v1/analyses/MGYA00350720/file/ERR1896775_FASTQ_SSU_OTU.tsv	Root-Environmental-Aquatic-Freshwater-Lake
ERR658180	MGYA00103506	https://www.ebi.ac.uk/metagenomics/api/v1/analyses/MGYA00103506/file/ERR658180_FASTQ_otu.tsv	Root-Environmental-Aquatic-Freshwater-Lake
ERR1582379	MGYA00352616	https://www.ebi.ac.uk/metagenomics/api/v1/analyses/MGYA00352616/file/ERR1582379_FASTQ_SSU_OTU.tsv	Root-Environmental-Aquatic-Freshwater-Lake
ERR1582647	MGYA00352884	https://www.ebi.ac.uk/metagenomics/api/v1/analyses/MGYA00352884/file/ERR1582647_FASTQ_SSU_OTU.tsv	Root-Environmental-Aquatic-Freshwater-Lake
ERR1583007	MGYA00353244	https://www.ebi.ac.uk/metagenomics/api/v1/analyses/MGYA00353244/file/ERR1583007_FASTQ_SSU_OTU.tsv	Root-Environmental-Aquatic-Freshwater-Lake
SRR7081210	MGYA00425712	https://www.ebi.ac.uk/metagenomics/api/v1/analyses/MGYA00425712/file/SRR7081210_MERGED_FASTQ_SSU_OTU.tsv	Root-Environmental-Aquatic-Freshwater-Lake
ERR1743378	MGYA00087110	https://www.ebi.ac.uk/metagenomics/api/v1/analyses/MGYA00087110/file/ERR1743378_MERGED_FASTQ_otu.tsv	Root-Environmental-Aquatic-Freshwater-Lake
ERR1583466	MGYA00353703	https://www.ebi.ac.uk/metagenomics/api/v1/analyses/MGYA00353703/file/ERR1583466_FASTQ_SSU_OTU.tsv	Root-Environmental-Aquatic-Freshwater-Lake
ERR1582226	MGYA00352463	https://www.ebi.ac.uk/metagenomics/api/v1/analyses/MGYA00352463/file/ERR1582226_FASTQ_SSU_OTU.tsv	Root-Environmental-Aquatic-Freshwater-Lake

ERR1582581	MGYA00352818	https://www.ebi.ac.uk/metagenomics/api/v1/analyses/MGYA00352818/file/ERR1582581_FASTQ_SSU_OTU.tsv	Root-Environmental-Aquatic-Freshwater-Lake
ERR1582550	MGYA00352787	https://www.ebi.ac.uk/metagenomics/api/v1/analyses/MGYA00352787/file/ERR1582550_FASTQ_SSU_OTU.tsv	Root-Environmental-Aquatic-Freshwater-Lake
ERR1582922	MGYA00353159	https://www.ebi.ac.uk/metagenomics/api/v1/analyses/MGYA00353159/file/ERR1582922_FASTQ_SSU_OTU.tsv	Root-Environmental-Aquatic-Freshwater-Lake
ERR1583310	MGYA00353547	https://www.ebi.ac.uk/metagenomics/api/v1/analyses/MGYA00353547/file/ERR1583310_FASTQ_SSU_OTU.tsv	Root-Environmental-Aquatic-Freshwater-Lake
ERR589308	MGYA00103441	https://www.ebi.ac.uk/metagenomics/api/v1/analyses/MGYA00103441/file/ERR589308_FASTQ_otu.tsv	Root-Environmental-Aquatic-Freshwater-Lake
ERR1582696	MGYA00352933	https://www.ebi.ac.uk/metagenomics/api/v1/analyses/MGYA00352933/file/ERR1582696_FASTQ_SSU_OTU.tsv	Root-Environmental-Aquatic-Freshwater-Lake
ERR1582131	MGYA00352368	https://www.ebi.ac.uk/metagenomics/api/v1/analyses/MGYA00352368/file/ERR1582131_FASTQ_SSU_OTU.tsv	Root-Environmental-Aquatic-Freshwater-Lake
ERR1582408	MGYA00352645	https://www.ebi.ac.uk/metagenomics/api/v1/analyses/MGYA00352645/file/ERR1582408_FASTQ_SSU_OTU.tsv	Root-Environmental-Aquatic-Freshwater-Lake
ERR1583248	MGYA00353485	https://www.ebi.ac.uk/metagenomics/api/v1/analyses/MGYA00353485/file/ERR1583248_FASTQ_SSU_OTU.tsv	Root-Environmental-Aquatic-Freshwater-Lake
ERR1897644	MGYA00351589	https://www.ebi.ac.uk/metagenomics/api/v1/analyses/MGYA00351589/file/ERR1897644_FASTQ_SSU_OTU.tsv	Root-Environmental-Aquatic-Freshwater-Lake
ERR1535968	MGYA00320259	https://www.ebi.ac.uk/metagenomics/api/v1/analyses/MGYA00320259/file/ERR1535968_FASTQ_SSU_OTU.tsv	Root-Environmental-Aquatic-Freshwater-Lake
ERR1582069	MGYA00352306	https://www.ebi.ac.uk/metagenomics/api/v1/analyses/MGYA00352306/file/ERR1582069_FASTQ_SSU_OTU.tsv	Root-Environmental-Aquatic-Freshwater-Lake

ERR1896817	MGYA00350762	https://www.ebi.ac.uk/metagenomics/api/v1/analyses/MGYA00350762/file/ERR1896817_FASTQ_SSU_OTU.tsv	Root-Environmental-Aquatic-Freshwater-Lake
ERR1897225	MGYA00351170	https://www.ebi.ac.uk/metagenomics/api/v1/analyses/MGYA00351170/file/ERR1897225_FASTQ_SSU_OTU.tsv	Root-Environmental-Aquatic-Freshwater-Lake
ERR1897582	MGYA00351527	https://www.ebi.ac.uk/metagenomics/api/v1/analyses/MGYA00351527/file/ERR1897582_FASTQ_SSU_OTU.tsv	Root-Environmental-Aquatic-Freshwater-Lake
ERR1897553	MGYA00351498	https://www.ebi.ac.uk/metagenomics/api/v1/analyses/MGYA00351498/file/ERR1897553_FASTQ_SSU_OTU.tsv	Root-Environmental-Aquatic-Freshwater-Lake
ERR1896772	MGYA00350717	https://www.ebi.ac.uk/metagenomics/api/v1/analyses/MGYA00350717/file/ERR1896772_FASTQ_SSU_OTU.tsv	Root-Environmental-Aquatic-Freshwater-Lake
ERR1897695	MGYA00351640	https://www.ebi.ac.uk/metagenomics/api/v1/analyses/MGYA00351640/file/ERR1897695_FASTQ_SSU_OTU.tsv	Root-Environmental-Aquatic-Freshwater-Lake
ERR1897132	MGYA00351077	https://www.ebi.ac.uk/metagenomics/api/v1/analyses/MGYA00351077/file/ERR1897132_FASTQ_SSU_OTU.tsv	Root-Environmental-Aquatic-Freshwater-Lake
ERR1583299	MGYA00353536	https://www.ebi.ac.uk/metagenomics/api/v1/analyses/MGYA00353536/file/ERR1583299_FASTQ_SSU_OTU.tsv	Root-Environmental-Aquatic-Freshwater-Lake
ERR658187	MGYA00103493	https://www.ebi.ac.uk/metagenomics/api/v1/analyses/MGYA00103493/file/ERR658187_FASTQ_otu.tsv	Root-Environmental-Aquatic-Freshwater-Lake
ERR1897477	MGYA00351422	https://www.ebi.ac.uk/metagenomics/api/v1/analyses/MGYA00351422/file/ERR1897477_FASTQ_SSU_OTU.tsv	Root-Environmental-Aquatic-Freshwater-Lake
ERR1743304	MGYA00087103	https://www.ebi.ac.uk/metagenomics/api/v1/analyses/MGYA00087103/file/ERR1743304_MERGED_FASTQ_otu.tsv	Root-Environmental-Aquatic-Freshwater-Lake
ERR1896656	MGYA00350601	https://www.ebi.ac.uk/metagenomics/api/v1/analyses/MGYA00350601/file/ERR1896656_FASTQ_SSU_OTU.tsv	Root-Environmental-Aquatic-Freshwater-Lake

ERR1897016	MGYA00350961	https://www.ebi.ac.uk/metagenomics/api/v1/analyses/MGYA00350961/file/ERR1897016_FASTQ_SSU_OTU.tsv	Root-Environmental-Aquatic-Freshwater-Lake
ERR430929	MGYA00103251	https://www.ebi.ac.uk/metagenomics/api/v1/analyses/MGYA00103251/file/ERR430929_FASTQ_otu.tsv	Root-Environmental-Aquatic-Freshwater-Lake
ERR1990497	MGYA00128214	https://www.ebi.ac.uk/metagenomics/api/v1/analyses/MGYA00128214/file/ERR1990497_MERGED_FASTQ_otu.tsv	Root-Environmental-Aquatic-Freshwater-Lake
SRR2443024	MGYA00359028	https://www.ebi.ac.uk/metagenomics/api/v1/analyses/MGYA00359028/file/SRR2443024_MERGED_FASTQ_SSU_OTU.tsv	Root-Environmental-Aquatic-Freshwater-Lake
ERR1897760	MGYA00351705	https://www.ebi.ac.uk/metagenomics/api/v1/analyses/MGYA00351705/file/ERR1897760_FASTQ_SSU_OTU.tsv	Root-Environmental-Aquatic-Freshwater-Lake
ERR1896687	MGYA00350632	https://www.ebi.ac.uk/metagenomics/api/v1/analyses/MGYA00350632/file/ERR1896687_FASTQ_SSU_OTU.tsv	Root-Environmental-Aquatic-Freshwater-Lake
ERR1896933	MGYA00350878	https://www.ebi.ac.uk/metagenomics/api/v1/analyses/MGYA00350878/file/ERR1896933_FASTQ_SSU_OTU.tsv	Root-Environmental-Aquatic-Freshwater-Lake
ERR1897301	MGYA00351246	https://www.ebi.ac.uk/metagenomics/api/v1/analyses/MGYA00351246/file/ERR1897301_FASTQ_SSU_OTU.tsv	Root-Environmental-Aquatic-Freshwater-Lake
ERR1897638	MGYA00351583	https://www.ebi.ac.uk/metagenomics/api/v1/analyses/MGYA00351583/file/ERR1897638_FASTQ_SSU_OTU.tsv	Root-Environmental-Aquatic-Freshwater-Lake
ERR1582806	MGYA00353043	https://www.ebi.ac.uk/metagenomics/api/v1/analyses/MGYA00353043/file/ERR1582806_FASTQ_SSU_OTU.tsv	Root-Environmental-Aquatic-Freshwater-Lake
ERR1582474	MGYA00352711	https://www.ebi.ac.uk/metagenomics/api/v1/analyses/MGYA00352711/file/ERR1582474_FASTQ_SSU_OTU.tsv	Root-Environmental-Aquatic-Freshwater-Lake
ERR1583234	MGYA00353471	https://www.ebi.ac.uk/metagenomics/api/v1/analyses/MGYA00353471/file/ERR1583234_FASTQ_SSU_OTU.tsv	Root-Environmental-Aquatic-Freshwater-Lake

ERR1897259	MGYA00351204	https://www.ebi.ac.uk/metagenomics/api/v1/analyses/MGYA00351204/file/ERR1897259_FASTQ_SSU_OTU.tsv	Root-Environmental-Aquatic-Freshwater-Lake
ERR1314375	MGYA00095184	https://www.ebi.ac.uk/metagenomics/api/v1/analyses/MGYA00095184/file/ERR1314375_MERGED_FASTQ_otu.tsv	Root-Environmental-Aquatic-Freshwater-Lake
ERR1314403	MGYA00095221	https://www.ebi.ac.uk/metagenomics/api/v1/analyses/MGYA00095221/file/ERR1314403_MERGED_FASTQ_otu.tsv	Root-Environmental-Aquatic-Freshwater-Lake
ERR1582763	MGYA00353000	https://www.ebi.ac.uk/metagenomics/api/v1/analyses/MGYA00353000/file/ERR1582763_FASTQ_SSU_OTU.tsv	Root-Environmental-Aquatic-Freshwater-Lake
ERR1583123	MGYA00353360	https://www.ebi.ac.uk/metagenomics/api/v1/analyses/MGYA00353360/file/ERR1583123_FASTQ_SSU_OTU.tsv	Root-Environmental-Aquatic-Freshwater-Lake
ERR1897288	MGYA00351233	https://www.ebi.ac.uk/metagenomics/api/v1/analyses/MGYA00351233/file/ERR1897288_FASTQ_SSU_OTU.tsv	Root-Environmental-Aquatic-Freshwater-Lake
ERR1547063	MGYA00320774	https://www.ebi.ac.uk/metagenomics/api/v1/analyses/MGYA00320774/file/ERR1547063_FASTQ_SSU_OTU.tsv	Root-Environmental-Aquatic-Freshwater-Lake
ERR1582302	MGYA00352539	https://www.ebi.ac.uk/metagenomics/api/v1/analyses/MGYA00352539/file/ERR1582302_FASTQ_SSU_OTU.tsv	Root-Environmental-Aquatic-Freshwater-Lake
ERR1429227	MGYA00107673	https://www.ebi.ac.uk/metagenomics/api/v1/analyses/MGYA00107673/file/ERR1429227_FASTQ_otu.tsv	Root-Environmental-Aquatic-Freshwater-Lake
ERR1437515	MGYA00107965	https://www.ebi.ac.uk/metagenomics/api/v1/analyses/MGYA00107965/file/ERR1437515_FASTQ_otu.tsv	Root-Environmental-Aquatic-Freshwater-Lake
ERR1897542	MGYA00351487	https://www.ebi.ac.uk/metagenomics/api/v1/analyses/MGYA00351487/file/ERR1897542_FASTQ_SSU_OTU.tsv	Root-Environmental-Aquatic-Freshwater-Lake
ERR589290	MGYA00103426	https://www.ebi.ac.uk/metagenomics/api/v1/analyses/MGYA00103426/file/ERR589290_FASTQ_otu.tsv	Root-Environmental-Aquatic-Freshwater-Lake

SRR7081359	MGYA00426445	https://www.ebi.ac.uk/metagenomics/api/v1/analyses/MGYA00426445/file/SRR7081359_MERGED_FASTQ_SSU_OTU.tsv	Root-Environmental-Aquatic-Freshwater-Lake
ERR1583288	MGYA00353525	https://www.ebi.ac.uk/metagenomics/api/v1/analyses/MGYA00353525/file/ERR1583288_FASTQ_SSU_OTU.tsv	Root-Environmental-Aquatic-Freshwater-Lake
ERR658196	MGYA00103495	https://www.ebi.ac.uk/metagenomics/api/v1/analyses/MGYA00103495/file/ERR658196_FASTQ_otu.tsv	Root-Environmental-Aquatic-Freshwater-Lake
ERR1896763	MGYA00350708	https://www.ebi.ac.uk/metagenomics/api/v1/analyses/MGYA00350708/file/ERR1896763_FASTQ_SSU_OTU.tsv	Root-Environmental-Aquatic-Freshwater-Lake
ERR1897123	MGYA00351068	https://www.ebi.ac.uk/metagenomics/api/v1/analyses/MGYA00351068/file/ERR1897123_FASTQ_SSU_OTU.tsv	Root-Environmental-Aquatic-Freshwater-Lake
ERR1897684	MGYA00351629	https://www.ebi.ac.uk/metagenomics/api/v1/analyses/MGYA00351629/file/ERR1897684_FASTQ_SSU_OTU.tsv	Root-Environmental-Aquatic-Freshwater-Lake
ERR1897655	MGYA00351600	https://www.ebi.ac.uk/metagenomics/api/v1/analyses/MGYA00351600/file/ERR1897655_FASTQ_SSU_OTU.tsv	Root-Environmental-Aquatic-Freshwater-Lake
ERR1535979	MGYA00320270	https://www.ebi.ac.uk/metagenomics/api/v1/analyses/MGYA00320270/file/ERR1535979_FASTQ_SSU_OTU.tsv	Root-Environmental-Aquatic-Freshwater-Lake
ERR1582419	MGYA00352656	https://www.ebi.ac.uk/metagenomics/api/v1/analyses/MGYA00352656/file/ERR1582419_FASTQ_SSU_OTU.tsv	Root-Environmental-Aquatic-Freshwater-Lake
ERR1583259	MGYA00353496	https://www.ebi.ac.uk/metagenomics/api/v1/analyses/MGYA00353496/file/ERR1583259_FASTQ_SSU_OTU.tsv	Root-Environmental-Aquatic-Freshwater-Lake
ERR1896806	MGYA00350751	https://www.ebi.ac.uk/metagenomics/api/v1/analyses/MGYA00350751/file/ERR1896806_FASTQ_SSU_OTU.tsv	Root-Environmental-Aquatic-Freshwater-Lake
ERR1897593	MGYA00351538	https://www.ebi.ac.uk/metagenomics/api/v1/analyses/MGYA00351538/file/ERR1897593_FASTQ_SSU_OTU.tsv	Root-Environmental-Aquatic-Freshwater-Lake

ERR1897234	MGYA00351179	https://www.ebi.ac.uk/metagenomics/api/v1/analyses/MGYA00351179/file/ERR1897234_FASTQ_SSU_OTU.tsv	Root-Environmental-Aquatic-Freshwater-Lake
ERR1582078	MGYA00352315	https://www.ebi.ac.uk/metagenomics/api/v1/analyses/MGYA00352315/file/ERR1582078_FASTQ_SSU_OTU.tsv	Root-Environmental-Aquatic-Freshwater-Lake
ERR1582933	MGYA00353170	https://www.ebi.ac.uk/metagenomics/api/v1/analyses/MGYA00353170/file/ERR1582933_FASTQ_SSU_OTU.tsv	Root-Environmental-Aquatic-Freshwater-Lake
ERR1582541	MGYA00352778	https://www.ebi.ac.uk/metagenomics/api/v1/analyses/MGYA00352778/file/ERR1582541_FASTQ_SSU_OTU.tsv	Root-Environmental-Aquatic-Freshwater-Lake
ERR1583301	MGYA00353538	https://www.ebi.ac.uk/metagenomics/api/v1/analyses/MGYA00353538/file/ERR1583301_FASTQ_SSU_OTU.tsv	Root-Environmental-Aquatic-Freshwater-Lake
ERR1582120	MGYA00352357	https://www.ebi.ac.uk/metagenomics/api/v1/analyses/MGYA00352357/file/ERR1582120_FASTQ_SSU_OTU.tsv	Root-Environmental-Aquatic-Freshwater-Lake
ERR1582687	MGYA00352924	https://www.ebi.ac.uk/metagenomics/api/v1/analyses/MGYA00352924/file/ERR1582687_FASTQ_SSU_OTU.tsv	Root-Environmental-Aquatic-Freshwater-Lake
ERR589319	MGYA00103445	https://www.ebi.ac.uk/metagenomics/api/v1/analyses/MGYA00103445/file/ERR589319_FASTQ_otu.tsv	Root-Environmental-Aquatic-Freshwater-Lake
ERR1743369	MGYA00087211	https://www.ebi.ac.uk/metagenomics/api/v1/analyses/MGYA00087211/file/ERR1743369_MERGED_FASTQ_otu.tsv	Root-Environmental-Aquatic-Freshwater-Lake
ERR1582656	MGYA00352893	https://www.ebi.ac.uk/metagenomics/api/v1/analyses/MGYA00352893/file/ERR1582656_FASTQ_SSU_OTU.tsv	Root-Environmental-Aquatic-Freshwater-Lake
ERR1583016	MGYA00353253	https://www.ebi.ac.uk/metagenomics/api/v1/analyses/MGYA00353253/file/ERR1583016_FASTQ_SSU_OTU.tsv	Root-Environmental-Aquatic-Freshwater-Lake
ERR1583477	MGYA00353714	https://www.ebi.ac.uk/metagenomics/api/v1/analyses/MGYA00353714/file/ERR1583477_FASTQ_SSU_OTU.tsv	Root-Environmental-Aquatic-Freshwater-Lake

ERR1582590	MGYA00352827	https://www.ebi.ac.uk/metagenomics/api/v1/analyses/MGYA00352827/file/ERR1582590_FASTQ_SSU_OTU.tsv	Root-Environmental-Aquatic-Freshwater-Lake
ERR1582237	MGYA00352474	https://www.ebi.ac.uk/metagenomics/api/v1/analyses/MGYA00352474/file/ERR1582237_FASTQ_SSU_OTU.tsv	Root-Environmental-Aquatic-Freshwater-Lake
ERR1897299	MGYA00351244	https://www.ebi.ac.uk/metagenomics/api/v1/analyses/MGYA00351244/file/ERR1897299_FASTQ_SSU_OTU.tsv	Root-Environmental-Aquatic-Freshwater-Lake
SRR7081325	MGYA00424812	https://www.ebi.ac.uk/metagenomics/api/v1/analyses/MGYA00424812/file/SRR7081325_MERGED_FASTQ_SSU_OTU.tsv	Root-Environmental-Aquatic-Freshwater-Lake
ERR1547072	MGYA00320783	https://www.ebi.ac.uk/metagenomics/api/v1/analyses/MGYA00320783/file/ERR1547072_FASTQ_SSU_OTU.tsv	Root-Environmental-Aquatic-Freshwater-Lake
ERR1582772	MGYA00353009	https://www.ebi.ac.uk/metagenomics/api/v1/analyses/MGYA00353009/file/ERR1582772_FASTQ_SSU_OTU.tsv	Root-Environmental-Aquatic-Freshwater-Lake
ERR1583132	MGYA00353369	https://www.ebi.ac.uk/metagenomics/api/v1/analyses/MGYA00353369/file/ERR1583132_FASTQ_SSU_OTU.tsv	Root-Environmental-Aquatic-Freshwater-Lake
ERR1582313	MGYA00352550	https://www.ebi.ac.uk/metagenomics/api/v1/analyses/MGYA00352550/file/ERR1582313_FASTQ_SSU_OTU.tsv	Root-Environmental-Aquatic-Freshwater-Lake
ERR1429236	MGYA00107666	https://www.ebi.ac.uk/metagenomics/api/v1/analyses/MGYA00107666/file/ERR1429236_FASTQ_otu.tsv	Root-Environmental-Aquatic-Freshwater-Lake
ERR1582465	MGYA00352702	https://www.ebi.ac.uk/metagenomics/api/v1/analyses/MGYA00352702/file/ERR1582465_FASTQ_SSU_OTU.tsv	Root-Environmental-Aquatic-Freshwater-Lake
ERR1582817	MGYA00353054	https://www.ebi.ac.uk/metagenomics/api/v1/analyses/MGYA00353054/file/ERR1582817_FASTQ_SSU_OTU.tsv	Root-Environmental-Aquatic-Freshwater-Lake
ERR1583225	MGYA00353462	https://www.ebi.ac.uk/metagenomics/api/v1/analyses/MGYA00353462/file/ERR1583225_FASTQ_SSU_OTU.tsv	Root-Environmental-Aquatic-Freshwater-Lake

ERR1897629	MGYA00351574	https://www.ebi.ac.uk/metagenomics/api/v1/analyses/MGYA00351574/file/ERR1897629_FASTQ_SSU_OTU.tsv	Root-Environmental-Aquatic-Freshwater-Lake
ERR389725	MGYA00103377	https://www.ebi.ac.uk/metagenomics/api/v1/analyses/MGYA00103377/file/ERR389725_FASTQ_otu.tsv	Root-Environmental-Aquatic-Freshwater-Lake
ERR1314364	MGYA00095174	https://www.ebi.ac.uk/metagenomics/api/v1/analyses/MGYA00095174/file/ERR1314364_MERGED_FASTQ_otu.tsv	Root-Environmental-Aquatic-Freshwater-Lake
ERR1897248	MGYA00351193	https://www.ebi.ac.uk/metagenomics/api/v1/analyses/MGYA00351193/file/ERR1897248_FASTQ_SSU_OTU.tsv	Root-Environmental-Aquatic-Freshwater-Lake
SRR2443035	MGYA00359039	https://www.ebi.ac.uk/metagenomics/api/v1/analyses/MGYA00359039/file/SRR2443035_MERGED_FASTQ_SSU_OTU.tsv	Root-Environmental-Aquatic-Freshwater-Lake
ERR1897771	MGYA00351716	https://www.ebi.ac.uk/metagenomics/api/v1/analyses/MGYA00351716/file/ERR1897771_FASTQ_SSU_OTU.tsv	Root-Environmental-Aquatic-Freshwater-Lake
ERR1896696	MGYA00350641	https://www.ebi.ac.uk/metagenomics/api/v1/analyses/MGYA00350641/file/ERR1896696_FASTQ_SSU_OTU.tsv	Root-Environmental-Aquatic-Freshwater-Lake
ERR1990486	MGYA00128202	https://www.ebi.ac.uk/metagenomics/api/v1/analyses/MGYA00128202/file/ERR1990486_MERGED_FASTQ_otu.tsv	Root-Environmental-Aquatic-Freshwater-Lake
ERR1896922	MGYA00350867	https://www.ebi.ac.uk/metagenomics/api/v1/analyses/MGYA00350867/file/ERR1896922_FASTQ_SSU_OTU.tsv	Root-Environmental-Aquatic-Freshwater-Lake
ERR1897310	MGYA00351255	https://www.ebi.ac.uk/metagenomics/api/v1/analyses/MGYA00351255/file/ERR1897310_FASTQ_SSU_OTU.tsv	Root-Environmental-Aquatic-Freshwater-Lake
ERR1897466	MGYA00351411	https://www.ebi.ac.uk/metagenomics/api/v1/analyses/MGYA00351411/file/ERR1897466_FASTQ_SSU_OTU.tsv	Root-Environmental-Aquatic-Freshwater-Lake
ERR1743315	MGYA00087146	https://www.ebi.ac.uk/metagenomics/api/v1/analyses/MGYA00087146/file/ERR1743315_MERGED_FASTQ_otu.tsv	Root-Environmental-Aquatic-Freshwater-Lake

ERR1896647	MGYA00350592	https://www.ebi.ac.uk/metagenomics/api/v1/analyses/MGYA00350592/file/ERR1896647_FASTQ_SSU_OTU.tsv	Root-Environmental-Aquatic-Freshwater-Lake
ERR1897007	MGYA00350952	https://www.ebi.ac.uk/metagenomics/api/v1/analyses/MGYA00350952/file/ERR1897007_FASTQ_SSU_OTU.tsv	Root-Environmental-Aquatic-Freshwater-Lake
ERR1547075	MGYA00320786	https://www.ebi.ac.uk/metagenomics/api/v1/analyses/MGYA00320786/file/ERR1547075_FASTQ_SSU_OTU.tsv	Root-Environmental-Aquatic-Freshwater-Lake
ERR1897539	MGYA00351484	https://www.ebi.ac.uk/metagenomics/api/v1/analyses/MGYA00351484/file/ERR1897539_FASTQ_SSU_OTU.tsv	Root-Environmental-Aquatic-Freshwater-Lake
SRR7081322	MGYA00424818	https://www.ebi.ac.uk/metagenomics/api/v1/analyses/MGYA00424818/file/SRR7081322_MERGED_FASTQ_SSU_OTU.tsv	Root-Environmental-Aquatic-Freshwater-Lake
ERR1583135	MGYA00353372	https://www.ebi.ac.uk/metagenomics/api/v1/analyses/MGYA00353372/file/ERR1583135_FASTQ_SSU_OTU.tsv	Root-Environmental-Aquatic-Freshwater-Lake
ERR1582775	MGYA00353012	https://www.ebi.ac.uk/metagenomics/api/v1/analyses/MGYA00353012/file/ERR1582775_FASTQ_SSU_OTU.tsv	Root-Environmental-Aquatic-Freshwater-Lake
ERR1897158	MGYA00351103	https://www.ebi.ac.uk/metagenomics/api/v1/analyses/MGYA00351103/file/ERR1897158_FASTQ_SSU_OTU.tsv	Root-Environmental-Aquatic-Freshwater-Lake
ERR1896718	MGYA00350663	https://www.ebi.ac.uk/metagenomics/api/v1/analyses/MGYA00350663/file/ERR1896718_FASTQ_SSU_OTU.tsv	Root-Environmental-Aquatic-Freshwater-Lake
ERR1429231	MGYA00107636	https://www.ebi.ac.uk/metagenomics/api/v1/analyses/MGYA00107636/file/ERR1429231_FASTQ_otu.tsv	Root-Environmental-Aquatic-Freshwater-Lake
ERR1582314	MGYA00352551	https://www.ebi.ac.uk/metagenomics/api/v1/analyses/MGYA00352551/file/ERR1582314_FASTQ_SSU_OTU.tsv	Root-Environmental-Aquatic-Freshwater-Lake
ERR1583222	MGYA00353459	https://www.ebi.ac.uk/metagenomics/api/v1/analyses/MGYA00353459/file/ERR1583222_FASTQ_SSU_OTU.tsv	Root-Environmental-Aquatic-Freshwater-Lake

ERR1582810	MGYA00353047	https://www.ebi.ac.uk/metagenomics/api/v1/analyses/MGYA00353047/file/ERR1582810_FASTQ_SSU_OTU.tsv	Root-Environmental-Aquatic-Freshwater-Lake
ERR1582462	MGYA00352699	https://www.ebi.ac.uk/metagenomics/api/v1/analyses/MGYA00352699/file/ERR1582462_FASTQ_SSU_OTU.tsv	Root-Environmental-Aquatic-Freshwater-Lake
ERR1897189	MGYA00351134	https://www.ebi.ac.uk/metagenomics/api/v1/analyses/MGYA00351134/file/ERR1897189_FASTQ_SSU_OTU.tsv	Root-Environmental-Aquatic-Freshwater-Lake
ERR1314363	MGYA00095178	https://www.ebi.ac.uk/metagenomics/api/v1/analyses/MGYA00095178/file/ERR1314363_MERGED_FASTQ_otu.tsv	Root-Environmental-Aquatic-Freshwater-Lake
ERR389722	MGYA00103373	https://www.ebi.ac.uk/metagenomics/api/v1/analyses/MGYA00103373/file/ERR389722_FASTQ_otu.tsv	Root-Environmental-Aquatic-Freshwater-Lake
ERR1896691	MGYA00350636	https://www.ebi.ac.uk/metagenomics/api/v1/analyses/MGYA00350636/file/ERR1896691_FASTQ_SSU_OTU.tsv	Root-Environmental-Aquatic-Freshwater-Lake
SRR2443032	MGYA00359036	https://www.ebi.ac.uk/metagenomics/api/v1/analyses/MGYA00359036/file/SRR2443032_MERGED_FASTQ_SSU_OTU.tsv	Root-Environmental-Aquatic-Freshwater-Lake
ERR1897776	MGYA00351721	https://www.ebi.ac.uk/metagenomics/api/v1/analyses/MGYA00351721/file/ERR1897776_FASTQ_SSU_OTU.tsv	Root-Environmental-Aquatic-Freshwater-Lake
ERR1582948	MGYA00353185	https://www.ebi.ac.uk/metagenomics/api/v1/analyses/MGYA00353185/file/ERR1582948_FASTQ_SSU_OTU.tsv	Root-Environmental-Aquatic-Freshwater-Lake
ERR1990481	MGYA00128203	https://www.ebi.ac.uk/metagenomics/api/v1/analyses/MGYA00128203/file/ERR1990481_MERGED_FASTQ_otu.tsv	Root-Environmental-Aquatic-Freshwater-Lake
ERR1897317	MGYA00351262	https://www.ebi.ac.uk/metagenomics/api/v1/analyses/MGYA00351262/file/ERR1897317_FASTQ_SSU_OTU.tsv	Root-Environmental-Aquatic-Freshwater-Lake
ERR1896925	MGYA00350870	https://www.ebi.ac.uk/metagenomics/api/v1/analyses/MGYA00350870/file/ERR1896925_FASTQ_SSU_OTU.tsv	Root-Environmental-Aquatic-Freshwater-Lake

ERR1743312	MGYA00087183	https://www.ebi.ac.uk/metagenomics/api/v1/analyses/MGYA00087183/file/ERR1743312_MERGED_FASTQ_otu.tsv	Root-Environmental-Aquatic-Freshwater-Lake
ERR1897461	MGYA00351406	https://www.ebi.ac.uk/metagenomics/api/v1/analyses/MGYA00351406/file/ERR1897461_FASTQ_SSU_OTU.tsv	Root-Environmental-Aquatic-Freshwater-Lake
ERR1582999	MGYA00353236	https://www.ebi.ac.uk/metagenomics/api/v1/analyses/MGYA00353236/file/ERR1582999_FASTQ_SSU_OTU.tsv	Root-Environmental-Aquatic-Freshwater-Lake
ERR1897000	MGYA00350945	https://www.ebi.ac.uk/metagenomics/api/v1/analyses/MGYA00350945/file/ERR1897000_FASTQ_SSU_OTU.tsv	Root-Environmental-Aquatic-Freshwater-Lake
ERR1896640	MGYA00350585	https://www.ebi.ac.uk/metagenomics/api/v1/analyses/MGYA00350585/file/ERR1896640_FASTQ_SSU_OTU.tsv	Root-Environmental-Aquatic-Freshwater-Lake
ERR1583149	MGYA00353386	https://www.ebi.ac.uk/metagenomics/api/v1/analyses/MGYA00353386/file/ERR1583149_FASTQ_SSU_OTU.tsv	Root-Environmental-Aquatic-Freshwater-Lake
ERR1582709	MGYA00352946	https://www.ebi.ac.uk/metagenomics/api/v1/analyses/MGYA00352946/file/ERR1582709_FASTQ_SSU_OTU.tsv	Root-Environmental-Aquatic-Freshwater-Lake
ERR1437512	MGYA00107952	https://www.ebi.ac.uk/metagenomics/api/v1/analyses/MGYA00107952/file/ERR1437512_FASTQ_otu.tsv	Root-Environmental-Aquatic-Freshwater-Lake
ERR589297	MGYA00103455	https://www.ebi.ac.uk/metagenomics/api/v1/analyses/MGYA00103455/file/ERR589297_FASTQ_otu.tsv	Root-Environmental-Aquatic-Freshwater-Lake
ERR1897545	MGYA00351490	https://www.ebi.ac.uk/metagenomics/api/v1/analyses/MGYA00351490/file/ERR1897545_FASTQ_SSU_OTU.tsv	Root-Environmental-Aquatic-Freshwater-Lake
ERR1582368	MGYA00352605	https://www.ebi.ac.uk/metagenomics/api/v1/analyses/MGYA00352605/file/ERR1582368_FASTQ_SSU_OTU.tsv	Root-Environmental-Aquatic-Freshwater-Lake
ERR658191	MGYA00103486	https://www.ebi.ac.uk/metagenomics/api/v1/analyses/MGYA00103486/file/ERR658191_FASTQ_otu.tsv	Root-Environmental-Aquatic-Freshwater-Lake

ERR1897683	MGYA00351628	https://www.ebi.ac.uk/metagenomics/api/v1/analyses/MGYA00351628/file/ERR1897683_FASTQ_SSU_OTU.tsv	Root-Environmental-Aquatic-Freshwater-Lake
ERR1897124	MGYA00351069	https://www.ebi.ac.uk/metagenomics/api/v1/analyses/MGYA00351069/file/ERR1897124_FASTQ_SSU_OTU.tsv	Root-Environmental-Aquatic-Freshwater-Lake
ERR1896764	MGYA00350709	https://www.ebi.ac.uk/metagenomics/api/v1/analyses/MGYA00350709/file/ERR1896764_FASTQ_SSU_OTU.tsv	Root-Environmental-Aquatic-Freshwater-Lake
ERR1742289	MGYA00311590	https://www.ebi.ac.uk/metagenomics/api/v1/analyses/MGYA00311590/file/ERR1742289_FASTQ_SSU_OTU.tsv	Root-Environmental-Aquatic-Freshwater-Lake
ERR1897652	MGYA00351597	https://www.ebi.ac.uk/metagenomics/api/v1/analyses/MGYA00351597/file/ERR1897652_FASTQ_SSU_OTU.tsv	Root-Environmental-Aquatic-Freshwater-Lake
ERR1897233	MGYA00351178	https://www.ebi.ac.uk/metagenomics/api/v1/analyses/MGYA00351178/file/ERR1897233_FASTQ_SSU_OTU.tsv	Root-Environmental-Aquatic-Freshwater-Lake
ERR1897594	MGYA00351539	https://www.ebi.ac.uk/metagenomics/api/v1/analyses/MGYA00351539/file/ERR1897594_FASTQ_SSU_OTU.tsv	Root-Environmental-Aquatic-Freshwater-Lake
ERR1896801	MGYA00350746	https://www.ebi.ac.uk/metagenomics/api/v1/analyses/MGYA00350746/file/ERR1896801_FASTQ_SSU_OTU.tsv	Root-Environmental-Aquatic-Freshwater-Lake
ERR1583198	MGYA00353435	https://www.ebi.ac.uk/metagenomics/api/v1/analyses/MGYA00353435/file/ERR1583198_FASTQ_SSU_OTU.tsv	Root-Environmental-Aquatic-Freshwater-Lake
ERR1583306	MGYA00353543	https://www.ebi.ac.uk/metagenomics/api/v1/analyses/MGYA00353543/file/ERR1583306_FASTQ_SSU_OTU.tsv	Root-Environmental-Aquatic-Freshwater-Lake
ERR1582546	MGYA00352783	https://www.ebi.ac.uk/metagenomics/api/v1/analyses/MGYA00352783/file/ERR1582546_FASTQ_SSU_OTU.tsv	Root-Environmental-Aquatic-Freshwater-Lake
ERR1582934	MGYA00353171	https://www.ebi.ac.uk/metagenomics/api/v1/analyses/MGYA00353171/file/ERR1582934_FASTQ_SSU_OTU.tsv	Root-Environmental-Aquatic-Freshwater-Lake

ERR1582680	MGYA00352917	https://www.ebi.ac.uk/metagenomics/api/v1/analyses/MGYA00352917/file/ERR1582680_FASTQ_SSU_OTU.tsv	Root-Environmental-Aquatic-Freshwater-Lake
ERR1582127	MGYA00352364	https://www.ebi.ac.uk/metagenomics/api/v1/analyses/MGYA00352364/file/ERR1582127_FASTQ_SSU_OTU.tsv	Root-Environmental-Aquatic-Freshwater-Lake
ERR1896959	MGYA00350904	https://www.ebi.ac.uk/metagenomics/api/v1/analyses/MGYA00350904/file/ERR1896959_FASTQ_SSU_OTU.tsv	Root-Environmental-Aquatic-Freshwater-Lake
ERR1896988	MGYA00350933	https://www.ebi.ac.uk/metagenomics/api/v1/analyses/MGYA00350933/file/ERR1896988_FASTQ_SSU_OTU.tsv	Root-Environmental-Aquatic-Freshwater-Lake
SRR7081206	MGYA00413190	https://www.ebi.ac.uk/metagenomics/api/v1/analyses/MGYA00413190/file/SRR7081206_MERGED_FASTQ_SSU_OTU.tsv	Root-Environmental-Aquatic-Freshwater-Lake
ERR1583011	MGYA00353248	https://www.ebi.ac.uk/metagenomics/api/v1/analyses/MGYA00353248/file/ERR1583011_FASTQ_SSU_OTU.tsv	Root-Environmental-Aquatic-Freshwater-Lake
ERR1582651	MGYA00352888	https://www.ebi.ac.uk/metagenomics/api/v1/analyses/MGYA00352888/file/ERR1582651_FASTQ_SSU_OTU.tsv	Root-Environmental-Aquatic-Freshwater-Lake
ERR1582230	MGYA00352467	https://www.ebi.ac.uk/metagenomics/api/v1/analyses/MGYA00352467/file/ERR1582230_FASTQ_SSU_OTU.tsv	Root-Environmental-Aquatic-Freshwater-Lake
ERR1582597	MGYA00352834	https://www.ebi.ac.uk/metagenomics/api/v1/analyses/MGYA00352834/file/ERR1582597_FASTQ_SSU_OTU.tsv	Root-Environmental-Aquatic-Freshwater-Lake
ERR1583470	MGYA00353707	https://www.ebi.ac.uk/metagenomics/api/v1/analyses/MGYA00353707/file/ERR1583470_FASTQ_SSU_OTU.tsv	Root-Environmental-Aquatic-Freshwater-Lake
ERR1897537	MGYA00351482	https://www.ebi.ac.uk/metagenomics/api/v1/analyses/MGYA00351482/file/ERR1897537_FASTQ_SSU_OTU.tsv	Root-Environmental-Aquatic-Freshwater-Lake
ERR1897290	MGYA00351235	https://www.ebi.ac.uk/metagenomics/api/v1/analyses/MGYA00351235/file/ERR1897290_FASTQ_SSU_OTU.tsv	Root-Environmental-Aquatic-Freshwater-Lake

ERR1896716	MGYA00350661	https://www.ebi.ac.uk/metagenomics/api/v1/analyses/MGYA00350661/file/ERR1896716_FASTQ_SSU_OTU.tsv	Root-Environmental-Aquatic-Freshwater-Lake
ERR1897156	MGYA00351101	https://www.ebi.ac.uk/metagenomics/api/v1/analyses/MGYA00351101/file/ERR1897156_FASTQ_SSU_OTU.tsv	Root-Environmental-Aquatic-Freshwater-Lake
ERR1897187	MGYA00351132	https://www.ebi.ac.uk/metagenomics/api/v1/analyses/MGYA00351132/file/ERR1897187_FASTQ_SSU_OTU.tsv	Root-Environmental-Aquatic-Freshwater-Lake
ERR1897620	MGYA00351565	https://www.ebi.ac.uk/metagenomics/api/v1/analyses/MGYA00351565/file/ERR1897620_FASTQ_SSU_OTU.tsv	Root-Environmental-Aquatic-Freshwater-Lake
ERR1743295	MGYA00087123	https://www.ebi.ac.uk/metagenomics/api/v1/analyses/MGYA00087123/file/ERR1743295_MERGED_FASTQ_otu.tsv	Root-Environmental-Aquatic-Freshwater-Lake
ERR1896873	MGYA00350818	https://www.ebi.ac.uk/metagenomics/api/v1/analyses/MGYA00350818/file/ERR1896873_FASTQ_SSU_OTU.tsv	Root-Environmental-Aquatic-Freshwater-Lake
ERR1897241	MGYA00351186	https://www.ebi.ac.uk/metagenomics/api/v1/analyses/MGYA00351186/file/ERR1897241_FASTQ_SSU_OTU.tsv	Root-Environmental-Aquatic-Freshwater-Lake
ERR1582534	MGYA00352771	https://www.ebi.ac.uk/metagenomics/api/v1/analyses/MGYA00352771/file/ERR1582534_FASTQ_SSU_OTU.tsv	Root-Environmental-Aquatic-Freshwater-Lake
ERR1582946	MGYA00353183	https://www.ebi.ac.uk/metagenomics/api/v1/analyses/MGYA00353183/file/ERR1582946_FASTQ_SSU_OTU.tsv	Root-Environmental-Aquatic-Freshwater-Lake
ERR1582293	MGYA00352530	https://www.ebi.ac.uk/metagenomics/api/v1/analyses/MGYA00352530/file/ERR1582293_FASTQ_SSU_OTU.tsv	Root-Environmental-Aquatic-Freshwater-Lake
ERR1583374	MGYA00353611	https://www.ebi.ac.uk/metagenomics/api/v1/analyses/MGYA00353611/file/ERR1583374_FASTQ_SSU_OTU.tsv	Root-Environmental-Aquatic-Freshwater-Lake
ERR1582155	MGYA00352392	https://www.ebi.ac.uk/metagenomics/api/v1/analyses/MGYA00352392/file/ERR1582155_FASTQ_SSU_OTU.tsv	Root-Environmental-Aquatic-Freshwater-Lake

ERR1897319	MGYA00351264	https://www.ebi.ac.uk/metagenomics/api/v1/analyses/MGYA00351264/file/ERR1897319_FASTQ_SSU_OTU.tsv	Root-Environmental-Aquatic-Freshwater-Lake
ERR1547123	MGYA00320834	https://www.ebi.ac.uk/metagenomics/api/v1/analyses/MGYA00320834/file/ERR1547123_FASTQ_SSU_OTU.tsv	Root-Environmental-Aquatic-Freshwater-Lake
ERR1582184	MGYA00352421	https://www.ebi.ac.uk/metagenomics/api/v1/analyses/MGYA00352421/file/ERR1582184_FASTQ_SSU_OTU.tsv	Root-Environmental-Aquatic-Freshwater-Lake
ERR1582623	MGYA00352860	https://www.ebi.ac.uk/metagenomics/api/v1/analyses/MGYA00352860/file/ERR1582623_FASTQ_SSU_OTU.tsv	Root-Environmental-Aquatic-Freshwater-Lake
ERR1583063	MGYA00353300	https://www.ebi.ac.uk/metagenomics/api/v1/analyses/MGYA00353300/file/ERR1583063_FASTQ_SSU_OTU.tsv	Root-Environmental-Aquatic-Freshwater-Lake
ERR1583402	MGYA00353639	https://www.ebi.ac.uk/metagenomics/api/v1/analyses/MGYA00353639/file/ERR1583402_FASTQ_SSU_OTU.tsv	Root-Environmental-Aquatic-Freshwater-Lake
ERR430931	MGYA00103250	https://www.ebi.ac.uk/metagenomics/api/v1/analyses/MGYA00103250/file/ERR430931_FASTQ_otu.tsv	Root-Environmental-Aquatic-Freshwater-Lake
ERR1582997	MGYA00353234	https://www.ebi.ac.uk/metagenomics/api/v1/analyses/MGYA00353234/file/ERR1582997_FASTQ_SSU_OTU.tsv	Root-Environmental-Aquatic-Freshwater-Lake
ERR1582242	MGYA00352479	https://www.ebi.ac.uk/metagenomics/api/v1/analyses/MGYA00352479/file/ERR1582242_FASTQ_SSU_OTU.tsv	Root-Environmental-Aquatic-Freshwater-Lake
ERR589299	MGYA00103456	https://www.ebi.ac.uk/metagenomics/api/v1/analyses/MGYA00103456/file/ERR589299_FASTQ_otu.tsv	Root-Environmental-Aquatic-Freshwater-Lake
ERR1582707	MGYA00352944	https://www.ebi.ac.uk/metagenomics/api/v1/analyses/MGYA00352944/file/ERR1582707_FASTQ_SSU_OTU.tsv	Root-Environmental-Aquatic-Freshwater-Lake
ERR1583147	MGYA00353384	https://www.ebi.ac.uk/metagenomics/api/v1/analyses/MGYA00353384/file/ERR1583147_FASTQ_SSU_OTU.tsv	Root-Environmental-Aquatic-Freshwater-Lake

ERR1583281	MGYA00353518	https://www.ebi.ac.uk/metagenomics/api/v1/analyses/MGYA00353518/file/ERR1583281_FASTQ_SSU_OTU.tsv	Root-Environmental-Aquatic-Freshwater-Lake
ERR1582366	MGYA00352603	https://www.ebi.ac.uk/metagenomics/api/v1/analyses/MGYA00352603/file/ERR1582366_FASTQ_SSU_OTU.tsv	Root-Environmental-Aquatic-Freshwater-Lake
ERR1429243	MGYA00107667	https://www.ebi.ac.uk/metagenomics/api/v1/analyses/MGYA00107667/file/ERR1429243_FASTQ_otu.tsv	Root-Environmental-Aquatic-Freshwater-Lake
ERR1582862	MGYA00353099	https://www.ebi.ac.uk/metagenomics/api/v1/analyses/MGYA00353099/file/ERR1582862_FASTQ_SSU_OTU.tsv	Root-Environmental-Aquatic-Freshwater-Lake
ERR1582410	MGYA00352647	https://www.ebi.ac.uk/metagenomics/api/v1/analyses/MGYA00352647/file/ERR1582410_FASTQ_SSU_OTU.tsv	Root-Environmental-Aquatic-Freshwater-Lake
ERR1583250	MGYA00353487	https://www.ebi.ac.uk/metagenomics/api/v1/analyses/MGYA00353487/file/ERR1583250_FASTQ_SSU_OTU.tsv	Root-Environmental-Aquatic-Freshwater-Lake
ERR1742287	MGYA00311588	https://www.ebi.ac.uk/metagenomics/api/v1/analyses/MGYA00311588/file/ERR1742287_FASTQ_SSU_OTU.tsv	Root-Environmental-Aquatic-Freshwater-Lake
ERR1535970	MGYA00320261	https://www.ebi.ac.uk/metagenomics/api/v1/analyses/MGYA00320261/file/ERR1535970_FASTQ_SSU_OTU.tsv	Root-Environmental-Aquatic-Freshwater-Lake
ERR1583196	MGYA00353433	https://www.ebi.ac.uk/metagenomics/api/v1/analyses/MGYA00353433/file/ERR1583196_FASTQ_SSU_OTU.tsv	Root-Environmental-Aquatic-Freshwater-Lake
ERR1582071	MGYA00352308	https://www.ebi.ac.uk/metagenomics/api/v1/analyses/MGYA00352308/file/ERR1582071_FASTQ_SSU_OTU.tsv	Root-Environmental-Aquatic-Freshwater-Lake
SRR7081381	MGYA00423195	https://www.ebi.ac.uk/metagenomics/api/v1/analyses/MGYA00423195/file/SRR7081381_MERGED_FASTQ_SSU_OTU.tsv	Root-Environmental-Aquatic-Freshwater-Lake
ERR1897704	MGYA00351649	https://www.ebi.ac.uk/metagenomics/api/v1/analyses/MGYA00351649/file/ERR1897704_FASTQ_SSU_OTU.tsv	Root-Environmental-Aquatic-Freshwater-Lake

SRR2443040	MGYA00359044	https://www.ebi.ac.uk/metagenomics/api/v1/analyses/MGYA00359044/file/SRR2443040_MERGED_FASTQ_SSU_OTU.tsv	Root-Environmental-Aquatic-Freshwater-Lake
ERR1582548	MGYA00352785	https://www.ebi.ac.uk/metagenomics/api/v1/analyses/MGYA00352785/file/ERR1582548_FASTQ_SSU_OTU.tsv	Root-Environmental-Aquatic-Freshwater-Lake
ERR1583308	MGYA00353545	https://www.ebi.ac.uk/metagenomics/api/v1/analyses/MGYA00353545/file/ERR1583308_FASTQ_SSU_OTU.tsv	Root-Environmental-Aquatic-Freshwater-Lake
ERR1896957	MGYA00350902	https://www.ebi.ac.uk/metagenomics/api/v1/analyses/MGYA00350902/file/ERR1896957_FASTQ_SSU_OTU.tsv	Root-Environmental-Aquatic-Freshwater-Lake
ERR1897365	MGYA00351310	https://www.ebi.ac.uk/metagenomics/api/v1/analyses/MGYA00351310/file/ERR1897365_FASTQ_SSU_OTU.tsv	Root-Environmental-Aquatic-Freshwater-Lake
ERR589310	MGYA00103440	https://www.ebi.ac.uk/metagenomics/api/v1/analyses/MGYA00103440/file/ERR589310_FASTQ_otu.tsv	Root-Environmental-Aquatic-Freshwater-Lake
ERR1582129	MGYA00352366	https://www.ebi.ac.uk/metagenomics/api/v1/analyses/MGYA00352366/file/ERR1582129_FASTQ_SSU_OTU.tsv	Root-Environmental-Aquatic-Freshwater-Lake
ERR1897413	MGYA00351358	https://www.ebi.ac.uk/metagenomics/api/v1/analyses/MGYA00351358/file/ERR1897413_FASTQ_SSU_OTU.tsv	Root-Environmental-Aquatic-Freshwater-Lake
ERR1896986	MGYA00350931	https://www.ebi.ac.uk/metagenomics/api/v1/analyses/MGYA00350931/file/ERR1896986_FASTQ_SSU_OTU.tsv	Root-Environmental-Aquatic-Freshwater-Lake
ERR1743360	MGYA00087200	https://www.ebi.ac.uk/metagenomics/api/v1/analyses/MGYA00087200/file/ERR1743360_MERGED_FASTQ_otu.tsv	Root-Environmental-Aquatic-Freshwater-Lake
SRR2442965	MGYA00358969	https://www.ebi.ac.uk/metagenomics/api/v1/analyses/MGYA00358969/file/SRR2442965_MERGED_FASTQ_SSU_OTU.tsv	Root-Environmental-Aquatic-Freshwater-Lake
ERR1582599	MGYA00352836	https://www.ebi.ac.uk/metagenomics/api/v1/analyses/MGYA00352836/file/ERR1582599_FASTQ_SSU_OTU.tsv	Root-Environmental-Aquatic-Freshwater-Lake

ERR1897072	MGYA00351017	https://www.ebi.ac.uk/metagenomics/api/v1/analyses/MGYA00351017/file/ERR1897072_FASTQ_SSU_OTU.tsv	Root-Environmental-Aquatic-Freshwater-Lake
ERR1583140	MGYA00353377	https://www.ebi.ac.uk/metagenomics/api/v1/analyses/MGYA00353377/file/ERR1583140_FASTQ_SSU_OTU.tsv	Root-Environmental-Aquatic-Freshwater-Lake
ERR1582700	MGYA00352937	https://www.ebi.ac.uk/metagenomics/api/v1/analyses/MGYA00352937/file/ERR1582700_FASTQ_SSU_OTU.tsv	Root-Environmental-Aquatic-Freshwater-Lake
ERR658198	MGYA00103510	https://www.ebi.ac.uk/metagenomics/api/v1/analyses/MGYA00103510/file/ERR658198_FASTQ_otu.tsv	Root-Environmental-Aquatic-Freshwater-Lake
ERR1429244	MGYA00107651	https://www.ebi.ac.uk/metagenomics/api/v1/analyses/MGYA00107651/file/ERR1429244_FASTQ_otu.tsv	Root-Environmental-Aquatic-Freshwater-Lake
ERR1582361	MGYA00352598	https://www.ebi.ac.uk/metagenomics/api/v1/analyses/MGYA00352598/file/ERR1582361_FASTQ_SSU_OTU.tsv	Root-Environmental-Aquatic-Freshwater-Lake
ERR1583286	MGYA00353523	https://www.ebi.ac.uk/metagenomics/api/v1/analyses/MGYA00353523/file/ERR1583286_FASTQ_SSU_OTU.tsv	Root-Environmental-Aquatic-Freshwater-Lake
ERR1583257	MGYA00353494	https://www.ebi.ac.uk/metagenomics/api/v1/analyses/MGYA00353494/file/ERR1583257_FASTQ_SSU_OTU.tsv	Root-Environmental-Aquatic-Freshwater-Lake
ERR1333520	MGYA00107587	https://www.ebi.ac.uk/metagenomics/api/v1/analyses/MGYA00107587/file/ERR1333520_FASTQ_otu.tsv	Root-Environmental-Aquatic-Freshwater-Lake
ERR1582417	MGYA00352654	https://www.ebi.ac.uk/metagenomics/api/v1/analyses/MGYA00352654/file/ERR1582417_FASTQ_SSU_OTU.tsv	Root-Environmental-Aquatic-Freshwater-Lake
ERR1582865	MGYA00353102	https://www.ebi.ac.uk/metagenomics/api/v1/analyses/MGYA00353102/file/ERR1582865_FASTQ_SSU_OTU.tsv	Root-Environmental-Aquatic-Freshwater-Lake
ERR1535977	MGYA00320268	https://www.ebi.ac.uk/metagenomics/api/v1/analyses/MGYA00320268/file/ERR1535977_FASTQ_SSU_OTU.tsv	Root-Environmental-Aquatic-Freshwater-Lake

ERR1742280	MGYA00311581	https://www.ebi.ac.uk/metagenomics/api/v1/analyses/MGYA00311581/file/ERR1742280_FASTQ_SSU_OTU.tsv	Root-Environmental-Aquatic-Freshwater-Lake
SRR7081040	MGYA00427355	https://www.ebi.ac.uk/metagenomics/api/v1/analyses/MGYA00427355/file/SRR7081040_MERGED_FASTQ_SSU_OTU.tsv	Root-Environmental-Aquatic-Freshwater-Lake
ERR1582076	MGYA00352313	https://www.ebi.ac.uk/metagenomics/api/v1/analyses/MGYA00352313/file/ERR1582076_FASTQ_SSU_OTU.tsv	Root-Environmental-Aquatic-Freshwater-Lake
ERR1583191	MGYA00353428	https://www.ebi.ac.uk/metagenomics/api/v1/analyses/MGYA00353428/file/ERR1583191_FASTQ_SSU_OTU.tsv	Root-Environmental-Aquatic-Freshwater-Lake
ERR1896808	MGYA00350753	https://www.ebi.ac.uk/metagenomics/api/v1/analyses/MGYA00350753/file/ERR1896808_FASTQ_SSU_OTU.tsv	Root-Environmental-Aquatic-Freshwater-Lake
ERR1897703	MGYA00351648	https://www.ebi.ac.uk/metagenomics/api/v1/analyses/MGYA00351648/file/ERR1897703_FASTQ_SSU_OTU.tsv	Root-Environmental-Aquatic-Freshwater-Lake
SRR2443047	MGYA00359051	https://www.ebi.ac.uk/metagenomics/api/v1/analyses/MGYA00359051/file/SRR2443047_MERGED_FASTQ_SSU_OTU.tsv	Root-Environmental-Aquatic-Freshwater-Lake
ERR589317	MGYA00103424	https://www.ebi.ac.uk/metagenomics/api/v1/analyses/MGYA00103424/file/ERR589317_FASTQ_otu.tsv	Root-Environmental-Aquatic-Freshwater-Lake
ERR1897362	MGYA00351307	https://www.ebi.ac.uk/metagenomics/api/v1/analyses/MGYA00351307/file/ERR1897362_FASTQ_SSU_OTU.tsv	Root-Environmental-Aquatic-Freshwater-Lake
ERR1896950	MGYA00350895	https://www.ebi.ac.uk/metagenomics/api/v1/analyses/MGYA00350895/file/ERR1896950_FASTQ_SSU_OTU.tsv	Root-Environmental-Aquatic-Freshwater-Lake
ERR1582689	MGYA00352926	https://www.ebi.ac.uk/metagenomics/api/v1/analyses/MGYA00352926/file/ERR1582689_FASTQ_SSU_OTU.tsv	Root-Environmental-Aquatic-Freshwater-Lake
ERR1583018	MGYA00353255	https://www.ebi.ac.uk/metagenomics/api/v1/analyses/MGYA00353255/file/ERR1583018_FASTQ_SSU_OTU.tsv	Root-Environmental-Aquatic-Freshwater-Lake

ERR1582658	MGYA00352895	https://www.ebi.ac.uk/metagenomics/api/v1/analyses/MGYA00352895/file/ERR1582658_FASTQ_SSU_OTU.tsv	Root-Environmental-Aquatic-Freshwater-Lake
SRR2442962	MGYA00358966	https://www.ebi.ac.uk/metagenomics/api/v1/analyses/MGYA00358966/file/SRR2442962_MERGED_FASTQ_SSU_OTU.tsv	Root-Environmental-Aquatic-Freshwater-Lake
ERR1743367	MGYA00087192	https://www.ebi.ac.uk/metagenomics/api/v1/analyses/MGYA00087192/file/ERR1743367_MERGED_FASTQ_otu.tsv	Root-Environmental-Aquatic-Freshwater-Lake
ERR1896981	MGYA00350926	https://www.ebi.ac.uk/metagenomics/api/v1/analyses/MGYA00350926/file/ERR1896981_FASTQ_SSU_OTU.tsv	Root-Environmental-Aquatic-Freshwater-Lake
ERR1897414	MGYA00351359	https://www.ebi.ac.uk/metagenomics/api/v1/analyses/MGYA00351359/file/ERR1897414_FASTQ_SSU_OTU.tsv	Root-Environmental-Aquatic-Freshwater-Lake
ERR1582239	MGYA00352476	https://www.ebi.ac.uk/metagenomics/api/v1/analyses/MGYA00352476/file/ERR1582239_FASTQ_SSU_OTU.tsv	Root-Environmental-Aquatic-Freshwater-Lake
ERR1583479	MGYA00353716	https://www.ebi.ac.uk/metagenomics/api/v1/analyses/MGYA00353716/file/ERR1583479_FASTQ_SSU_OTU.tsv	Root-Environmental-Aquatic-Freshwater-Lake
ERR1897075	MGYA00351020	https://www.ebi.ac.uk/metagenomics/api/v1/analyses/MGYA00351020/file/ERR1897075_FASTQ_SSU_OTU.tsv	Root-Environmental-Aquatic-Freshwater-Lake
ERR1896635	MGYA00350580	https://www.ebi.ac.uk/metagenomics/api/v1/analyses/MGYA00350580/file/ERR1896635_FASTQ_SSU_OTU.tsv	Root-Environmental-Aquatic-Freshwater-Lake
ERR2868525	MGYA00377407	https://www.ebi.ac.uk/metagenomics/api/v1/analyses/MGYA00377407/file/ERR2868525_FASTQ_SSU_OTU.tsv	Root-Environmental-Aquatic-Freshwater-Lake
ERR1897297	MGYA00351242	https://www.ebi.ac.uk/metagenomics/api/v1/analyses/MGYA00351242/file/ERR1897297_FASTQ_SSU_OTU.tsv	Root-Environmental-Aquatic-Freshwater-Lake
ERR1897530	MGYA00351475	https://www.ebi.ac.uk/metagenomics/api/v1/analyses/MGYA00351475/file/ERR1897530_FASTQ_SSU_OTU.tsv	Root-Environmental-Aquatic-Freshwater-Lake

ERR1429238	MGYA00107664	https://www.ebi.ac.uk/metagenomics/api/v1/analyses/MGYA00107664/file/ERR1429238_FASTQ_otu.tsv	Root-Environmental-Aquatic-Freshwater-Lake
ERR1990501	MGYA00128211	https://www.ebi.ac.uk/metagenomics/api/v1/analyses/MGYA00128211/file/ERR1990501_MERGED_FASTQ_otu.tsv	Root-Environmental-Aquatic-Freshwater-Lake
ERR1897151	MGYA00351096	https://www.ebi.ac.uk/metagenomics/api/v1/analyses/MGYA00351096/file/ERR1897151_FASTQ_SSU_OTU.tsv	Root-Environmental-Aquatic-Freshwater-Lake
ERR1896711	MGYA00350656	https://www.ebi.ac.uk/metagenomics/api/v1/analyses/MGYA00350656/file/ERR1896711_FASTQ_SSU_OTU.tsv	Root-Environmental-Aquatic-Freshwater-Lake
ERR1897627	MGYA00351572	https://www.ebi.ac.uk/metagenomics/api/v1/analyses/MGYA00351572/file/ERR1897627_FASTQ_SSU_OTU.tsv	Root-Environmental-Aquatic-Freshwater-Lake
ERR1897180	MGYA00351125	https://www.ebi.ac.uk/metagenomics/api/v1/analyses/MGYA00351125/file/ERR1897180_FASTQ_SSU_OTU.tsv	Root-Environmental-Aquatic-Freshwater-Lake
ERR1582819	MGYA00353056	https://www.ebi.ac.uk/metagenomics/api/v1/analyses/MGYA00353056/file/ERR1582819_FASTQ_SSU_OTU.tsv	Root-Environmental-Aquatic-Freshwater-Lake
ERR1897246	MGYA00351191	https://www.ebi.ac.uk/metagenomics/api/v1/analyses/MGYA00351191/file/ERR1897246_FASTQ_SSU_OTU.tsv	Root-Environmental-Aquatic-Freshwater-Lake
ERR1896874	MGYA00350819	https://www.ebi.ac.uk/metagenomics/api/v1/analyses/MGYA00350819/file/ERR1896874_FASTQ_SSU_OTU.tsv	Root-Environmental-Aquatic-Freshwater-Lake
ERR1743292	MGYA00087161	https://www.ebi.ac.uk/metagenomics/api/v1/analyses/MGYA00087161/file/ERR1743292_MERGED_FASTQ_otu.tsv	Root-Environmental-Aquatic-Freshwater-Lake
ERR1583373	MGYA00353610	https://www.ebi.ac.uk/metagenomics/api/v1/analyses/MGYA00353610/file/ERR1583373_FASTQ_SSU_OTU.tsv	Root-Environmental-Aquatic-Freshwater-Lake
ERR1582294	MGYA00352531	https://www.ebi.ac.uk/metagenomics/api/v1/analyses/MGYA00352531/file/ERR1582294_FASTQ_SSU_OTU.tsv	Root-Environmental-Aquatic-Freshwater-Lake

ERR1582941	MGYA00353178	https://www.ebi.ac.uk/metagenomics/api/v1/analyses/MGYA00353178/file/ERR1582941_FASTQ_SSU_OTU.tsv	Root-Environmental-Aquatic-Freshwater-Lake
ERR1990488	MGYA00128200	https://www.ebi.ac.uk/metagenomics/api/v1/analyses/MGYA00128200/file/ERR1990488_MERGED_FASTQ_otu.tsv	Root-Environmental-Aquatic-Freshwater-Lake
ERR1582533	MGYA00352770	https://www.ebi.ac.uk/metagenomics/api/v1/analyses/MGYA00352770/file/ERR1582533_FASTQ_SSU_OTU.tsv	Root-Environmental-Aquatic-Freshwater-Lake
ERR1896698	MGYA00350643	https://www.ebi.ac.uk/metagenomics/api/v1/analyses/MGYA00350643/file/ERR1896698_FASTQ_SSU_OTU.tsv	Root-Environmental-Aquatic-Freshwater-Lake
ERR1582152	MGYA00352389	https://www.ebi.ac.uk/metagenomics/api/v1/analyses/MGYA00352389/file/ERR1582152_FASTQ_SSU_OTU.tsv	Root-Environmental-Aquatic-Freshwater-Lake
ERR1547124	MGYA00320835	https://www.ebi.ac.uk/metagenomics/api/v1/analyses/MGYA00320835/file/ERR1547124_FASTQ_SSU_OTU.tsv	Root-Environmental-Aquatic-Freshwater-Lake
SRR7081273	MGYA00425153	https://www.ebi.ac.uk/metagenomics/api/v1/analyses/MGYA00425153/file/SRR7081273_MERGED_FASTQ_SSU_OTU.tsv	Root-Environmental-Aquatic-Freshwater-Lake
ERR1897468	MGYA00351413	https://www.ebi.ac.uk/metagenomics/api/v1/analyses/MGYA00351413/file/ERR1897468_FASTQ_SSU_OTU.tsv	Root-Environmental-Aquatic-Freshwater-Lake
ERR1583064	MGYA00353301	https://www.ebi.ac.uk/metagenomics/api/v1/analyses/MGYA00353301/file/ERR1583064_FASTQ_SSU_OTU.tsv	Root-Environmental-Aquatic-Freshwater-Lake
ERR1582624	MGYA00352861	https://www.ebi.ac.uk/metagenomics/api/v1/analyses/MGYA00352861/file/ERR1582624_FASTQ_SSU_OTU.tsv	Root-Environmental-Aquatic-Freshwater-Lake
ERR1582183	MGYA00352420	https://www.ebi.ac.uk/metagenomics/api/v1/analyses/MGYA00352420/file/ERR1582183_FASTQ_SSU_OTU.tsv	Root-Environmental-Aquatic-Freshwater-Lake
ERR1897009	MGYA00350954	https://www.ebi.ac.uk/metagenomics/api/v1/analyses/MGYA00350954/file/ERR1897009_FASTQ_SSU_OTU.tsv	Root-Environmental-Aquatic-Freshwater-Lake

ERR1896649	MGYA00350594	https://www.ebi.ac.uk/metagenomics/api/v1/analyses/MGYA00350594/file/ERR1896649_FASTQ_SSU_OTU.tsv	Root-Environmental-Aquatic-Freshwater-Lake
ERR1582245	MGYA00352482	https://www.ebi.ac.uk/metagenomics/api/v1/analyses/MGYA00352482/file/ERR1582245_FASTQ_SSU_OTU.tsv	Root-Environmental-Aquatic-Freshwater-Lake
ERR430936	MGYA00103253	https://www.ebi.ac.uk/metagenomics/api/v1/analyses/MGYA00103253/file/ERR430936_FASTQ_otu.tsv	Root-Environmental-Aquatic-Freshwater-Lake
ERR1582990	MGYA00353227	https://www.ebi.ac.uk/metagenomics/api/v1/analyses/MGYA00353227/file/ERR1582990_FASTQ_SSU_OTU.tsv	Root-Environmental-Aquatic-Freshwater-Lake
ERR1583405	MGYA00353642	https://www.ebi.ac.uk/metagenomics/api/v1/analyses/MGYA00353642/file/ERR1583405_FASTQ_SSU_OTU.tsv	Root-Environmental-Aquatic-Freshwater-Lake
ERR1582043	MGYA00352280	https://www.ebi.ac.uk/metagenomics/api/v1/analyses/MGYA00352280/file/ERR1582043_FASTQ_SSU_OTU.tsv	Root-Environmental-Aquatic-Freshwater-Lake
ERR1333515	MGYA00107605	https://www.ebi.ac.uk/metagenomics/api/v1/analyses/MGYA00107605/file/ERR1333515_FASTQ_otu.tsv	Root-Environmental-Aquatic-Freshwater-Lake
ERR1583262	MGYA00353499	https://www.ebi.ac.uk/metagenomics/api/v1/analyses/MGYA00353499/file/ERR1583262_FASTQ_SSU_OTU.tsv	Root-Environmental-Aquatic-Freshwater-Lake
ERR1582385	MGYA00352622	https://www.ebi.ac.uk/metagenomics/api/v1/analyses/MGYA00352622/file/ERR1582385_FASTQ_SSU_OTU.tsv	Root-Environmental-Aquatic-Freshwater-Lake
ERR1582422	MGYA00352659	https://www.ebi.ac.uk/metagenomics/api/v1/analyses/MGYA00352659/file/ERR1582422_FASTQ_SSU_OTU.tsv	Root-Environmental-Aquatic-Freshwater-Lake
ERR1582850	MGYA00353087	https://www.ebi.ac.uk/metagenomics/api/v1/analyses/MGYA00353087/file/ERR1582850_FASTQ_SSU_OTU.tsv	Root-Environmental-Aquatic-Freshwater-Lake
ERR1896789	MGYA00350734	https://www.ebi.ac.uk/metagenomics/api/v1/analyses/MGYA00350734/file/ERR1896789_FASTQ_SSU_OTU.tsv	Root-Environmental-Aquatic-Freshwater-Lake

ERR1897118	MGYA00351063	https://www.ebi.ac.uk/metagenomics/api/v1/analyses/MGYA00351063/file/ERR1897118_FASTQ_SSU_OTU.tsv	Root-Environmental-Aquatic-Freshwater-Lake
ERR1535993	MGYA00320284	https://www.ebi.ac.uk/metagenomics/api/v1/analyses/MGYA00320284/file/ERR1535993_FASTQ_SSU_OTU.tsv	Root-Environmental-Aquatic-Freshwater-Lake
ERR1896758	MGYA00350703	https://www.ebi.ac.uk/metagenomics/api/v1/analyses/MGYA00350703/file/ERR1896758_FASTQ_SSU_OTU.tsv	Root-Environmental-Aquatic-Freshwater-Lake
ERR1582354	MGYA00352591	https://www.ebi.ac.uk/metagenomics/api/v1/analyses/MGYA00352591/file/ERR1582354_FASTQ_SSU_OTU.tsv	Root-Environmental-Aquatic-Freshwater-Lake
ERR1429271	MGYA00107624	https://www.ebi.ac.uk/metagenomics/api/v1/analyses/MGYA00107624/file/ERR1429271_FASTQ_otu.tsv	Root-Environmental-Aquatic-Freshwater-Lake
ERR1582881	MGYA00353118	https://www.ebi.ac.uk/metagenomics/api/v1/analyses/MGYA00353118/file/ERR1582881_FASTQ_SSU_OTU.tsv	Root-Environmental-Aquatic-Freshwater-Lake
ERR1583514	MGYA00353751	https://www.ebi.ac.uk/metagenomics/api/v1/analyses/MGYA00353751/file/ERR1583514_FASTQ_SSU_OTU.tsv	Root-Environmental-Aquatic-Freshwater-Lake
ERR1547035	MGYA00320746	https://www.ebi.ac.uk/metagenomics/api/v1/analyses/MGYA00320746/file/ERR1547035_FASTQ_SSU_OTU.tsv	Root-Environmental-Aquatic-Freshwater-Lake
SRR7081362	MGYA00423812	https://www.ebi.ac.uk/metagenomics/api/v1/analyses/MGYA00423812/file/SRR7081362_MERGED_FASTQ_SSU_OTU.tsv	Root-Environmental-Aquatic-Freshwater-Lake
ERR1897579	MGYA00351524	https://www.ebi.ac.uk/metagenomics/api/v1/analyses/MGYA00351524/file/ERR1897579_FASTQ_SSU_OTU.tsv	Root-Environmental-Aquatic-Freshwater-Lake
ERR1583175	MGYA00353412	https://www.ebi.ac.uk/metagenomics/api/v1/analyses/MGYA00353412/file/ERR1583175_FASTQ_SSU_OTU.tsv	Root-Environmental-Aquatic-Freshwater-Lake
ERR1582735	MGYA00352972	https://www.ebi.ac.uk/metagenomics/api/v1/analyses/MGYA00352972/file/ERR1582735_FASTQ_SSU_OTU.tsv	Root-Environmental-Aquatic-Freshwater-Lake

ERR1582092	MGYA00352329	https://www.ebi.ac.uk/metagenomics/api/v1/analyses/MGYA00352329/file/ERR1582092_FASTQ_SSU_OTU.tsv	Root-Environmental-Aquatic-Freshwater-Lake
ERR1897040	MGYA00350985	https://www.ebi.ac.uk/metagenomics/api/v1/analyses/MGYA00350985/file/ERR1897040_FASTQ_SSU_OTU.tsv	Root-Environmental-Aquatic-Freshwater-Lake
SRR2442957	MGYA00358961	https://www.ebi.ac.uk/metagenomics/api/v1/analyses/MGYA00358961/file/SRR2442957_MERGED_FASTQ_SSU_OTU.tsv	Root-Environmental-Aquatic-Freshwater-Lake
ERR1743352	MGYA00087102	https://www.ebi.ac.uk/metagenomics/api/v1/analyses/MGYA00087102/file/ERR1743352_MERGED_FASTQ_otu.tsv	Root-Environmental-Aquatic-Freshwater-Lake
ERR1897386	MGYA00351331	https://www.ebi.ac.uk/metagenomics/api/v1/analyses/MGYA00351331/file/ERR1897386_FASTQ_SSU_OTU.tsv	Root-Environmental-Aquatic-Freshwater-Lake
ERR1897421	MGYA00351366	https://www.ebi.ac.uk/metagenomics/api/v1/analyses/MGYA00351366/file/ERR1897421_FASTQ_SSU_OTU.tsv	Root-Environmental-Aquatic-Freshwater-Lake
ERR1897357	MGYA00351302	https://www.ebi.ac.uk/metagenomics/api/v1/analyses/MGYA00351302/file/ERR1897357_FASTQ_SSU_OTU.tsv	Root-Environmental-Aquatic-Freshwater-Lake
ERR589322	MGYA00103430	https://www.ebi.ac.uk/metagenomics/api/v1/analyses/MGYA00103430/file/ERR589322_FASTQ_otu.tsv	Root-Environmental-Aquatic-Freshwater-Lake
ERR1896965	MGYA00350910	https://www.ebi.ac.uk/metagenomics/api/v1/analyses/MGYA00350910/file/ERR1896965_FASTQ_SSU_OTU.tsv	Root-Environmental-Aquatic-Freshwater-Lake
SRR2442986	MGYA00358990	https://www.ebi.ac.uk/metagenomics/api/v1/analyses/MGYA00358990/file/SRR2442986_MERGED_FASTQ_SSU_OTU.tsv	Root-Environmental-Aquatic-Freshwater-Lake
ERR1743383	MGYA00087189	https://www.ebi.ac.uk/metagenomics/api/v1/analyses/MGYA00087189/file/ERR1743383_MERGED_FASTQ_otu.tsv	Root-Environmental-Aquatic-Freshwater-Lake
ERR1897736	MGYA00351681	https://www.ebi.ac.uk/metagenomics/api/v1/analyses/MGYA00351681/file/ERR1897736_FASTQ_SSU_OTU.tsv	Root-Environmental-Aquatic-Freshwater-Lake

ERR1897091	MGYA00351036	https://www.ebi.ac.uk/metagenomics/api/v1/analyses/MGYA00351036/file/ERR1897091_FASTQ_SSU_OTU.tsv	Root-Environmental-Aquatic-Freshwater-Lake
ERR1582908	MGYA00353145	https://www.ebi.ac.uk/metagenomics/api/v1/analyses/MGYA00353145/file/ERR1582908_FASTQ_SSU_OTU.tsv	Root-Environmental-Aquatic-Freshwater-Lake
ERR1897273	MGYA00351218	https://www.ebi.ac.uk/metagenomics/api/v1/analyses/MGYA00351218/file/ERR1897273_FASTQ_SSU_OTU.tsv	Root-Environmental-Aquatic-Freshwater-Lake
ERR1547098	MGYA00320809	https://www.ebi.ac.uk/metagenomics/api/v1/analyses/MGYA00320809/file/ERR1547098_FASTQ_SSU_OTU.tsv	Root-Environmental-Aquatic-Freshwater-Lake
ERR1896841	MGYA00350786	https://www.ebi.ac.uk/metagenomics/api/v1/analyses/MGYA00350786/file/ERR1896841_FASTQ_SSU_OTU.tsv	Root-Environmental-Aquatic-Freshwater-Lake
ERR1582798	MGYA00353035	https://www.ebi.ac.uk/metagenomics/api/v1/analyses/MGYA00353035/file/ERR1582798_FASTQ_SSU_OTU.tsv	Root-Environmental-Aquatic-Freshwater-Lake
ERR1897612	MGYA00351557	https://www.ebi.ac.uk/metagenomics/api/v1/analyses/MGYA00351557/file/ERR1897612_FASTQ_SSU_OTU.tsv	Root-Environmental-Aquatic-Freshwater-Lake
ERR1582328	MGYA00352565	https://www.ebi.ac.uk/metagenomics/api/v1/analyses/MGYA00352565/file/ERR1582328_FASTQ_SSU_OTU.tsv	Root-Environmental-Aquatic-Freshwater-Lake
ERR1897164	MGYA00351109	https://www.ebi.ac.uk/metagenomics/api/v1/analyses/MGYA00351109/file/ERR1897164_FASTQ_SSU_OTU.tsv	Root-Environmental-Aquatic-Freshwater-Lake
ERR1896724	MGYA00350669	https://www.ebi.ac.uk/metagenomics/api/v1/analyses/MGYA00350669/file/ERR1896724_FASTQ_SSU_OTU.tsv	Root-Environmental-Aquatic-Freshwater-Lake
ERR1583109	MGYA00353346	https://www.ebi.ac.uk/metagenomics/api/v1/analyses/MGYA00353346/file/ERR1583109_FASTQ_SSU_OTU.tsv	Root-Environmental-Aquatic-Freshwater-Lake
ERR658217	MGYA00103483	https://www.ebi.ac.uk/metagenomics/api/v1/analyses/MGYA00103483/file/ERR658217_FASTQ_otu.tsv	Root-Environmental-Aquatic-Freshwater-Lake

ERR1582749	MGYA00352986	https://www.ebi.ac.uk/metagenomics/api/v1/analyses/MGYA00352986/file/ERR1582749_FASTQ_SSU_OTU.tsv	Root-Environmental-Aquatic-Freshwater-Lake
ERR1743276	MGYA00087157	https://www.ebi.ac.uk/metagenomics/api/v1/analyses/MGYA00087157/file/ERR1743276_MERGED_FASTQ_otu.tsv	Root-Environmental-Aquatic-Freshwater-Lake
ERR1547049	MGYA00320760	https://www.ebi.ac.uk/metagenomics/api/v1/analyses/MGYA00320760/file/ERR1547049_FASTQ_SSU_OTU.tsv	Root-Environmental-Aquatic-Freshwater-Lake
ERR1896890	MGYA00350835	https://www.ebi.ac.uk/metagenomics/api/v1/analyses/MGYA00350835/file/ERR1896890_FASTQ_SSU_OTU.tsv	Root-Environmental-Aquatic-Freshwater-Lake
ERR1897505	MGYA00351450	https://www.ebi.ac.uk/metagenomics/api/v1/analyses/MGYA00351450/file/ERR1897505_FASTQ_SSU_OTU.tsv	Root-Environmental-Aquatic-Freshwater-Lake
ERR1582270	MGYA00352507	https://www.ebi.ac.uk/metagenomics/api/v1/analyses/MGYA00352507/file/ERR1582270_FASTQ_SSU_OTU.tsv	Root-Environmental-Aquatic-Freshwater-Lake
ERR1583397	MGYA00353634	https://www.ebi.ac.uk/metagenomics/api/v1/analyses/MGYA00353634/file/ERR1583397_FASTQ_SSU_OTU.tsv	Root-Environmental-Aquatic-Freshwater-Lake
ERR1583430	MGYA00353667	https://www.ebi.ac.uk/metagenomics/api/v1/analyses/MGYA00353667/file/ERR1583430_FASTQ_SSU_OTU.tsv	Root-Environmental-Aquatic-Freshwater-Lake
ERR1547111	MGYA00320822	https://www.ebi.ac.uk/metagenomics/api/v1/analyses/MGYA00320822/file/ERR1547111_FASTQ_SSU_OTU.tsv	Root-Environmental-Aquatic-Freshwater-Lake
SRR7081246	MGYA00425688	https://www.ebi.ac.uk/metagenomics/api/v1/analyses/MGYA00425688/file/SRR7081246_MERGED_FASTQ_SSU_OTU.tsv	Root-Environmental-Aquatic-Freshwater-Lake
ERR1583051	MGYA00353288	https://www.ebi.ac.uk/metagenomics/api/v1/analyses/MGYA00353288/file/ERR1583051_FASTQ_SSU_OTU.tsv	Root-Environmental-Aquatic-Freshwater-Lake
ERR1582611	MGYA00352848	https://www.ebi.ac.uk/metagenomics/api/v1/analyses/MGYA00352848/file/ERR1582611_FASTQ_SSU_OTU.tsv	Root-Environmental-Aquatic-Freshwater-Lake

ERR1582167	MGYA00352404	https://www.ebi.ac.uk/metagenomics/api/v1/analyses/MGYA00352404/file/ERR1582167_FASTQ_SSU_OTU.tsv	Root-Environmental-Aquatic-Freshwater-Lake
ERR1583080	MGYA00353317	https://www.ebi.ac.uk/metagenomics/api/v1/analyses/MGYA00353317/file/ERR1583080_FASTQ_SSU_OTU.tsv	Root-Environmental-Aquatic-Freshwater-Lake
ERR1896919	MGYA00350864	https://www.ebi.ac.uk/metagenomics/api/v1/analyses/MGYA00350864/file/ERR1896919_FASTQ_SSU_OTU.tsv	Root-Environmental-Aquatic-Freshwater-Lake
ERR1583346	MGYA00353583	https://www.ebi.ac.uk/metagenomics/api/v1/analyses/MGYA00353583/file/ERR1583346_FASTQ_SSU_OTU.tsv	Root-Environmental-Aquatic-Freshwater-Lake
ERR1582974	MGYA00353211	https://www.ebi.ac.uk/metagenomics/api/v1/analyses/MGYA00353211/file/ERR1582974_FASTQ_SSU_OTU.tsv	Root-Environmental-Aquatic-Freshwater-Lake
ERR1582506	MGYA00352743	https://www.ebi.ac.uk/metagenomics/api/v1/analyses/MGYA00352743/file/ERR1582506_FASTQ_SSU_OTU.tsv	Root-Environmental-Aquatic-Freshwater-Lake
SRR7081151	MGYA00413199	https://www.ebi.ac.uk/metagenomics/api/v1/analyses/MGYA00413199/file/SRR7081151_MERGED_FASTQ_SSU_OTU.tsv	Root-Environmental-Aquatic-Freshwater-Lake
ERR1896846	MGYA00350791	https://www.ebi.ac.uk/metagenomics/api/v1/analyses/MGYA00350791/file/ERR1896846_FASTQ_SSU_OTU.tsv	Root-Environmental-Aquatic-Freshwater-Lake
ERR1897274	MGYA00351219	https://www.ebi.ac.uk/metagenomics/api/v1/analyses/MGYA00351219/file/ERR1897274_FASTQ_SSU_OTU.tsv	Root-Environmental-Aquatic-Freshwater-Lake
ERR1314358	MGYA00095161	https://www.ebi.ac.uk/metagenomics/api/v1/analyses/MGYA00095161/file/ERR1314358_MERGED_FASTQ_otu.tsv	Root-Environmental-Aquatic-Freshwater-Lake
ERR1582038	MGYA00352275	https://www.ebi.ac.uk/metagenomics/api/v1/analyses/MGYA00352275/file/ERR1582038_FASTQ_SSU_OTU.tsv	Root-Environmental-Aquatic-Freshwater-Lake
ERR1897615	MGYA00351560	https://www.ebi.ac.uk/metagenomics/api/v1/analyses/MGYA00351560/file/ERR1897615_FASTQ_SSU_OTU.tsv	Root-Environmental-Aquatic-Freshwater-Lake

ERR1582459	MGYA00352696	https://www.ebi.ac.uk/metagenomics/api/v1/analyses/MGYA00352696/file/ERR1582459_FASTQ_SSU_OTU.tsv	Root-Environmental-Aquatic-Freshwater-Lake
ERR1583219	MGYA00353456	https://www.ebi.ac.uk/metagenomics/api/v1/analyses/MGYA00353456/file/ERR1583219_FASTQ_SSU_OTU.tsv	Root-Environmental-Aquatic-Freshwater-Lake
ERR1582488	MGYA00352725	https://www.ebi.ac.uk/metagenomics/api/v1/analyses/MGYA00352725/file/ERR1582488_FASTQ_SSU_OTU.tsv	Root-Environmental-Aquatic-Freshwater-Lake
ERR1896723	MGYA00350668	https://www.ebi.ac.uk/metagenomics/api/v1/analyses/MGYA00350668/file/ERR1896723_FASTQ_SSU_OTU.tsv	Root-Environmental-Aquatic-Freshwater-Lake
ERR1897163	MGYA00351108	https://www.ebi.ac.uk/metagenomics/api/v1/analyses/MGYA00351108/file/ERR1897163_FASTQ_SSU_OTU.tsv	Root-Environmental-Aquatic-Freshwater-Lake
ERR1314389	MGYA00095206	https://www.ebi.ac.uk/metagenomics/api/v1/analyses/MGYA00095206/file/ERR1314389_MERGED_FASTQ_otu.tsv	Root-Environmental-Aquatic-Freshwater-Lake
ERR658210	MGYA00103475	https://www.ebi.ac.uk/metagenomics/api/v1/analyses/MGYA00103475/file/ERR658210_FASTQ_otu.tsv	Root-Environmental-Aquatic-Freshwater-Lake
SRR7081319	MGYA00424821	https://www.ebi.ac.uk/metagenomics/api/v1/analyses/MGYA00424821/file/SRR7081319_MERGED_FASTQ_SSU_OTU.tsv	Root-Environmental-Aquatic-Freshwater-Lake
ERR1897502	MGYA00351447	https://www.ebi.ac.uk/metagenomics/api/v1/analyses/MGYA00351447/file/ERR1897502_FASTQ_SSU_OTU.tsv	Root-Environmental-Aquatic-Freshwater-Lake
ERR1896897	MGYA00350842	https://www.ebi.ac.uk/metagenomics/api/v1/analyses/MGYA00350842/file/ERR1896897_FASTQ_SSU_OTU.tsv	Root-Environmental-Aquatic-Freshwater-Lake
ERR1583437	MGYA00353674	https://www.ebi.ac.uk/metagenomics/api/v1/analyses/MGYA00353674/file/ERR1583437_FASTQ_SSU_OTU.tsv	Root-Environmental-Aquatic-Freshwater-Lake
ERR1583390	MGYA00353627	https://www.ebi.ac.uk/metagenomics/api/v1/analyses/MGYA00353627/file/ERR1583390_FASTQ_SSU_OTU.tsv	Root-Environmental-Aquatic-Freshwater-Lake

ERR1582277	MGYA00352514	https://www.ebi.ac.uk/metagenomics/api/v1/analyses/MGYA00352514/file/ERR1582277_FASTQ_SSU_OTU.tsv	Root-Environmental-Aquatic-Freshwater-Lake
SRR7081241	MGYA00414121	https://www.ebi.ac.uk/metagenomics/api/v1/analyses/MGYA00414121/file/SRR7081241_MERGED_FASTQ_SSU_OTU.tsv	Root-Environmental-Aquatic-Freshwater-Lake
ERR1743329	MGYA00087148	https://www.ebi.ac.uk/metagenomics/api/v1/analyses/MGYA00087148/file/ERR1743329_MERGED_FASTQ_otu.tsv	Root-Environmental-Aquatic-Freshwater-Lake
ERR1547116	MGYA00320827	https://www.ebi.ac.uk/metagenomics/api/v1/analyses/MGYA00320827/file/ERR1547116_FASTQ_SSU_OTU.tsv	Root-Environmental-Aquatic-Freshwater-Lake
ERR1582616	MGYA00352853	https://www.ebi.ac.uk/metagenomics/api/v1/analyses/MGYA00352853/file/ERR1582616_FASTQ_SSU_OTU.tsv	Root-Environmental-Aquatic-Freshwater-Lake
ERR1583056	MGYA00353293	https://www.ebi.ac.uk/metagenomics/api/v1/analyses/MGYA00353293/file/ERR1583056_FASTQ_SSU_OTU.tsv	Root-Environmental-Aquatic-Freshwater-Lake
ERR1583087	MGYA00353324	https://www.ebi.ac.uk/metagenomics/api/v1/analyses/MGYA00353324/file/ERR1583087_FASTQ_SSU_OTU.tsv	Root-Environmental-Aquatic-Freshwater-Lake
ERR1582160	MGYA00352397	https://www.ebi.ac.uk/metagenomics/api/v1/analyses/MGYA00352397/file/ERR1582160_FASTQ_SSU_OTU.tsv	Root-Environmental-Aquatic-Freshwater-Lake
ERR1582501	MGYA00352738	https://www.ebi.ac.uk/metagenomics/api/v1/analyses/MGYA00352738/file/ERR1582501_FASTQ_SSU_OTU.tsv	Root-Environmental-Aquatic-Freshwater-Lake
ERR1582973	MGYA00353210	https://www.ebi.ac.uk/metagenomics/api/v1/analyses/MGYA00353210/file/ERR1582973_FASTQ_SSU_OTU.tsv	Root-Environmental-Aquatic-Freshwater-Lake
ERR1583341	MGYA00353578	https://www.ebi.ac.uk/metagenomics/api/v1/analyses/MGYA00353578/file/ERR1583341_FASTQ_SSU_OTU.tsv	Root-Environmental-Aquatic-Freshwater-Lake
SRR2443009	MGYA00359013	https://www.ebi.ac.uk/metagenomics/api/v1/analyses/MGYA00359013/file/SRR2443009_MERGED_FASTQ_SSU_OTU.tsv	Root-Environmental-Aquatic-Freshwater-Lake

ERR1582044	MGYA00352281	https://www.ebi.ac.uk/metagenomics/api/v1/analyses/MGYA00352281/file/ERR1582044_FASTQ_SSU_OTU.tsv	Root-Environmental-Aquatic-Freshwater-Lake
SRR7081413	MGYA00414113	https://www.ebi.ac.uk/metagenomics/api/v1/analyses/MGYA00414113/file/SRR7081413_MERGED_FASTQ_SSU_OTU.tsv	Root-Environmental-Aquatic-Freshwater-Lake
ERR1897208	MGYA00351153	https://www.ebi.ac.uk/metagenomics/api/v1/analyses/MGYA00351153/file/ERR1897208_FASTQ_SSU_OTU.tsv	Root-Environmental-Aquatic-Freshwater-Lake
ERR1582857	MGYA00353094	https://www.ebi.ac.uk/metagenomics/api/v1/analyses/MGYA00353094/file/ERR1582857_FASTQ_SSU_OTU.tsv	Root-Environmental-Aquatic-Freshwater-Lake
ERR1582425	MGYA00352662	https://www.ebi.ac.uk/metagenomics/api/v1/analyses/MGYA00352662/file/ERR1582425_FASTQ_SSU_OTU.tsv	Root-Environmental-Aquatic-Freshwater-Lake
ERR1582382	MGYA00352619	https://www.ebi.ac.uk/metagenomics/api/v1/analyses/MGYA00352619/file/ERR1582382_FASTQ_SSU_OTU.tsv	Root-Environmental-Aquatic-Freshwater-Lake
ERR1583265	MGYA00353502	https://www.ebi.ac.uk/metagenomics/api/v1/analyses/MGYA00353502/file/ERR1583265_FASTQ_SSU_OTU.tsv	Root-Environmental-Aquatic-Freshwater-Lake
ERR1333512	MGYA00107604	https://www.ebi.ac.uk/metagenomics/api/v1/analyses/MGYA00107604/file/ERR1333512_FASTQ_otu.tsv	Root-Environmental-Aquatic-Freshwater-Lake
SRR7081072	MGYA00426726	https://www.ebi.ac.uk/metagenomics/api/v1/analyses/MGYA00426726/file/SRR7081072_MERGED_FASTQ_SSU_OTU.tsv	Root-Environmental-Aquatic-Freshwater-Lake
ERR1897669	MGYA00351614	https://www.ebi.ac.uk/metagenomics/api/v1/analyses/MGYA00351614/file/ERR1897669_FASTQ_SSU_OTU.tsv	Root-Environmental-Aquatic-Freshwater-Lake
ERR1535994	MGYA00320285	https://www.ebi.ac.uk/metagenomics/api/v1/analyses/MGYA00320285/file/ERR1535994_FASTQ_SSU_OTU.tsv	Root-Environmental-Aquatic-Freshwater-Lake
ERR1583513	MGYA00353750	https://www.ebi.ac.uk/metagenomics/api/v1/analyses/MGYA00353750/file/ERR1583513_FASTQ_SSU_OTU.tsv	Root-Environmental-Aquatic-Freshwater-Lake

ERR1582886	MGYA00353123	https://www.ebi.ac.uk/metagenomics/api/v1/analyses/MGYA00353123/file/ERR1582886_FASTQ_SSU_OTU.tsv	Root-Environmental-Aquatic-Freshwater-Lake
ERR1429276	MGYA00107628	https://www.ebi.ac.uk/metagenomics/api/v1/analyses/MGYA00107628/file/ERR1429276_FASTQ_otu.tsv	Root-Environmental-Aquatic-Freshwater-Lake
ERR1582353	MGYA00352590	https://www.ebi.ac.uk/metagenomics/api/v1/analyses/MGYA00352590/file/ERR1582353_FASTQ_SSU_OTU.tsv	Root-Environmental-Aquatic-Freshwater-Lake
SRR7081365	MGYA00423213	https://www.ebi.ac.uk/metagenomics/api/v1/analyses/MGYA00423213/file/SRR7081365_MERGED_FASTQ_SSU_OTU.tsv	Root-Environmental-Aquatic-Freshwater-Lake
ERR1547032	MGYA00320743	https://www.ebi.ac.uk/metagenomics/api/v1/analyses/MGYA00320743/file/ERR1547032_FASTQ_SSU_OTU.tsv	Root-Environmental-Aquatic-Freshwater-Lake
ERR1582095	MGYA00352332	https://www.ebi.ac.uk/metagenomics/api/v1/analyses/MGYA00352332/file/ERR1582095_FASTQ_SSU_OTU.tsv	Root-Environmental-Aquatic-Freshwater-Lake
ERR1582732	MGYA00352969	https://www.ebi.ac.uk/metagenomics/api/v1/analyses/MGYA00352969/file/ERR1582732_FASTQ_SSU_OTU.tsv	Root-Environmental-Aquatic-Freshwater-Lake
ERR1583172	MGYA00353409	https://www.ebi.ac.uk/metagenomics/api/v1/analyses/MGYA00353409/file/ERR1583172_FASTQ_SSU_OTU.tsv	Root-Environmental-Aquatic-Freshwater-Lake
ERR1897047	MGYA00350992	https://www.ebi.ac.uk/metagenomics/api/v1/analyses/MGYA00350992/file/ERR1897047_FASTQ_SSU_OTU.tsv	Root-Environmental-Aquatic-Freshwater-Lake
ERR1897426	MGYA00351371	https://www.ebi.ac.uk/metagenomics/api/v1/analyses/MGYA00351371/file/ERR1897426_FASTQ_SSU_OTU.tsv	Root-Environmental-Aquatic-Freshwater-Lake
ERR1897381	MGYA00351326	https://www.ebi.ac.uk/metagenomics/api/v1/analyses/MGYA00351326/file/ERR1897381_FASTQ_SSU_OTU.tsv	Root-Environmental-Aquatic-Freshwater-Lake
ERR1743355	MGYA00087206	https://www.ebi.ac.uk/metagenomics/api/v1/analyses/MGYA00087206/file/ERR1743355_MERGED_FASTQ_otu.tsv	Root-Environmental-Aquatic-Freshwater-Lake

SRR2442950	MGYA00358954	https://www.ebi.ac.uk/metagenomics/api/v1/analyses/MGYA00358954/file/SRR2442950_MERGED_FASTQ_SSU_OTU.tsv	Root-Environmental-Aquatic-Freshwater-Lake
ERR1743384	MGYA00087109	https://www.ebi.ac.uk/metagenomics/api/v1/analyses/MGYA00087109/file/ERR1743384_MERGED_FASTQ_otu.tsv	Root-Environmental-Aquatic-Freshwater-Lake
SRR2442981	MGYA00358985	https://www.ebi.ac.uk/metagenomics/api/v1/analyses/MGYA00358985/file/SRR2442981_MERGED_FASTQ_SSU_OTU.tsv	Root-Environmental-Aquatic-Freshwater-Lake
ERR1896962	MGYA00350907	https://www.ebi.ac.uk/metagenomics/api/v1/analyses/MGYA00350907/file/ERR1896962_FASTQ_SSU_OTU.tsv	Root-Environmental-Aquatic-Freshwater-Lake
ERR589325	MGYA00103447	https://www.ebi.ac.uk/metagenomics/api/v1/analyses/MGYA00103447/file/ERR589325_FASTQ_otu.tsv	Root-Environmental-Aquatic-Freshwater-Lake
ERR1897350	MGYA00351295	https://www.ebi.ac.uk/metagenomics/api/v1/analyses/MGYA00351295/file/ERR1897350_FASTQ_SSU_OTU.tsv	Root-Environmental-Aquatic-Freshwater-Lake
ERR1897096	MGYA00351041	https://www.ebi.ac.uk/metagenomics/api/v1/analyses/MGYA00351041/file/ERR1897096_FASTQ_SSU_OTU.tsv	Root-Environmental-Aquatic-Freshwater-Lake
ERR1897731	MGYA00351676	https://www.ebi.ac.uk/metagenomics/api/v1/analyses/MGYA00351676/file/ERR1897731_FASTQ_SSU_OTU.tsv	Root-Environmental-Aquatic-Freshwater-Lake
ERR1314356	MGYA00095197	https://www.ebi.ac.uk/metagenomics/api/v1/analyses/MGYA00095197/file/ERR1314356_MERGED_FASTQ_otu.tsv	Root-Environmental-Aquatic-Freshwater-Lake
ERR1582791	MGYA00353028	https://www.ebi.ac.uk/metagenomics/api/v1/analyses/MGYA00353028/file/ERR1582791_FASTQ_SSU_OTU.tsv	Root-Environmental-Aquatic-Freshwater-Lake
ERR1582036	MGYA00352273	https://www.ebi.ac.uk/metagenomics/api/v1/analyses/MGYA00352273/file/ERR1582036_FASTQ_SSU_OTU.tsv	Root-Environmental-Aquatic-Freshwater-Lake
ERR1896848	MGYA00350793	https://www.ebi.ac.uk/metagenomics/api/v1/analyses/MGYA00350793/file/ERR1896848_FASTQ_SSU_OTU.tsv	Root-Environmental-Aquatic-Freshwater-Lake

ERR1547091	MGYA00320802	https://www.ebi.ac.uk/metagenomics/api/v1/analyses/MGYA00320802/file/ERR1547091_FASTQ_SSU_OTU.tsv	Root-Environmental-Aquatic-Freshwater-Lake
ERR1583217	MGYA00353454	https://www.ebi.ac.uk/metagenomics/api/v1/analyses/MGYA00353454/file/ERR1583217_FASTQ_SSU_OTU.tsv	Root-Environmental-Aquatic-Freshwater-Lake
ERR1582825	MGYA00353062	https://www.ebi.ac.uk/metagenomics/api/v1/analyses/MGYA00353062/file/ERR1582825_FASTQ_SSU_OTU.tsv	Root-Environmental-Aquatic-Freshwater-Lake
ERR1582457	MGYA00352694	https://www.ebi.ac.uk/metagenomics/api/v1/analyses/MGYA00352694/file/ERR1582457_FASTQ_SSU_OTU.tsv	Root-Environmental-Aquatic-Freshwater-Lake
ERR1582321	MGYA00352558	https://www.ebi.ac.uk/metagenomics/api/v1/analyses/MGYA00352558/file/ERR1582321_FASTQ_SSU_OTU.tsv	Root-Environmental-Aquatic-Freshwater-Lake
ERR1582486	MGYA00352723	https://www.ebi.ac.uk/metagenomics/api/v1/analyses/MGYA00352723/file/ERR1582486_FASTQ_SSU_OTU.tsv	Root-Environmental-Aquatic-Freshwater-Lake
ERR1896899	MGYA00350844	https://www.ebi.ac.uk/metagenomics/api/v1/analyses/MGYA00350844/file/ERR1896899_FASTQ_SSU_OTU.tsv	Root-Environmental-Aquatic-Freshwater-Lake
ERR1547040	MGYA00320751	https://www.ebi.ac.uk/metagenomics/api/v1/analyses/MGYA00320751/file/ERR1547040_FASTQ_SSU_OTU.tsv	Root-Environmental-Aquatic-Freshwater-Lake
SRR7081317	MGYA00424829	https://www.ebi.ac.uk/metagenomics/api/v1/analyses/MGYA00424829/file/SRR7081317_MERGED_FASTQ_SSU_OTU.tsv	Root-Environmental-Aquatic-Freshwater-Lake
ERR1583100	MGYA00353337	https://www.ebi.ac.uk/metagenomics/api/v1/analyses/MGYA00353337/file/ERR1583100_FASTQ_SSU_OTU.tsv	Root-Environmental-Aquatic-Freshwater-Lake
ERR1582740	MGYA00352977	https://www.ebi.ac.uk/metagenomics/api/v1/analyses/MGYA00352977/file/ERR1582740_FASTQ_SSU_OTU.tsv	Root-Environmental-Aquatic-Freshwater-Lake
ERR1314387	MGYA00095187	https://www.ebi.ac.uk/metagenomics/api/v1/analyses/MGYA00095187/file/ERR1314387_MERGED_FASTQ_otu.tsv	Root-Environmental-Aquatic-Freshwater-Lake

ERR1582279	MGYA00352516	https://www.ebi.ac.uk/metagenomics/api/v1/analyses/MGYA00352516/file/ERR1582279_FASTQ_SSU_OTU.tsv	Root-Environmental-Aquatic-Freshwater-Lake
ERR1583439	MGYA00353676	https://www.ebi.ac.uk/metagenomics/api/v1/analyses/MGYA00353676/file/ERR1583439_FASTQ_SSU_OTU.tsv	Root-Environmental-Aquatic-Freshwater-Lake
ERR1897035	MGYA00350980	https://www.ebi.ac.uk/metagenomics/api/v1/analyses/MGYA00350980/file/ERR1897035_FASTQ_SSU_OTU.tsv	Root-Environmental-Aquatic-Freshwater-Lake
ERR1896675	MGYA00350620	https://www.ebi.ac.uk/metagenomics/api/v1/analyses/MGYA00350620/file/ERR1896675_FASTQ_SSU_OTU.tsv	Root-Environmental-Aquatic-Freshwater-Lake
ERR1583058	MGYA00353295	https://www.ebi.ac.uk/metagenomics/api/v1/analyses/MGYA00353295/file/ERR1583058_FASTQ_SSU_OTU.tsv	Root-Environmental-Aquatic-Freshwater-Lake
ERR1582618	MGYA00352855	https://www.ebi.ac.uk/metagenomics/api/v1/analyses/MGYA00352855/file/ERR1582618_FASTQ_SSU_OTU.tsv	Root-Environmental-Aquatic-Freshwater-Lake
ERR1743327	MGYA00087160	https://www.ebi.ac.uk/metagenomics/api/v1/analyses/MGYA00087160/file/ERR1743327_MERGED_FASTQ_otu.tsv	Root-Environmental-Aquatic-Freshwater-Lake
ERR1547118	MGYA00320829	https://www.ebi.ac.uk/metagenomics/api/v1/analyses/MGYA00320829/file/ERR1547118_FASTQ_SSU_OTU.tsv	Root-Environmental-Aquatic-Freshwater-Lake
ERR1897454	MGYA00351399	https://www.ebi.ac.uk/metagenomics/api/v1/analyses/MGYA00351399/file/ERR1897454_FASTQ_SSU_OTU.tsv	Root-Environmental-Aquatic-Freshwater-Lake
ERR1897322	MGYA00351267	https://www.ebi.ac.uk/metagenomics/api/v1/analyses/MGYA00351267/file/ERR1897322_FASTQ_SSU_OTU.tsv	Root-Environmental-Aquatic-Freshwater-Lake
ERR1897485	MGYA00351430	https://www.ebi.ac.uk/metagenomics/api/v1/analyses/MGYA00351430/file/ERR1897485_FASTQ_SSU_OTU.tsv	Root-Environmental-Aquatic-Freshwater-Lake
ERR1896910	MGYA00350855	https://www.ebi.ac.uk/metagenomics/api/v1/analyses/MGYA00350855/file/ERR1896910_FASTQ_SSU_OTU.tsv	Root-Environmental-Aquatic-Freshwater-Lake

SRR3987091	MGYA00095522	https://www.ebi.ac.uk/metagenomics/api/v1/analyses/MGYA00095522/file/SRR3987091_MERGED_FASTQ_otu.tsv	Root-Environmental-Aquatic-Freshwater-Lake
ERR1583089	MGYA00353326	https://www.ebi.ac.uk/metagenomics/api/v1/analyses/MGYA00353326/file/ERR1583089_FASTQ_SSU_OTU.tsv	Root-Environmental-Aquatic-Freshwater-Lake
ERR1897743	MGYA00351688	https://www.ebi.ac.uk/metagenomics/api/v1/analyses/MGYA00351688/file/ERR1897743_FASTQ_SSU_OTU.tsv	Root-Environmental-Aquatic-Freshwater-Lake
SRR2443007	MGYA00359011	https://www.ebi.ac.uk/metagenomics/api/v1/analyses/MGYA00359011/file/SRR2443007_MERGED_FASTQ_SSU_OTU.tsv	Root-Environmental-Aquatic-Freshwater-Lake
ERR1897206	MGYA00351151	https://www.ebi.ac.uk/metagenomics/api/v1/analyses/MGYA00351151/file/ERR1897206_FASTQ_SSU_OTU.tsv	Root-Environmental-Aquatic-Freshwater-Lake
ERR1896834	MGYA00350779	https://www.ebi.ac.uk/metagenomics/api/v1/analyses/MGYA00350779/file/ERR1896834_FASTQ_SSU_OTU.tsv	Root-Environmental-Aquatic-Freshwater-Lake
ERR1896780	MGYA00350725	https://www.ebi.ac.uk/metagenomics/api/v1/analyses/MGYA00350725/file/ERR1896780_FASTQ_SSU_OTU.tsv	Root-Environmental-Aquatic-Freshwater-Lake
ERR1897667	MGYA00351612	https://www.ebi.ac.uk/metagenomics/api/v1/analyses/MGYA00351612/file/ERR1897667_FASTQ_SSU_OTU.tsv	Root-Environmental-Aquatic-Freshwater-Lake
ERR1582859	MGYA00353096	https://www.ebi.ac.uk/metagenomics/api/v1/analyses/MGYA00353096/file/ERR1582859_FASTQ_SSU_OTU.tsv	Root-Environmental-Aquatic-Freshwater-Lake
ERR658175	MGYA00103502	https://www.ebi.ac.uk/metagenomics/api/v1/analyses/MGYA00103502/file/ERR658175_FASTQ_otu.tsv	Root-Environmental-Aquatic-Freshwater-Lake
ERR1582888	MGYA00353125	https://www.ebi.ac.uk/metagenomics/api/v1/analyses/MGYA00353125/file/ERR1582888_FASTQ_SSU_OTU.tsv	Root-Environmental-Aquatic-Freshwater-Lake
ERR1429278	MGYA00107672	https://www.ebi.ac.uk/metagenomics/api/v1/analyses/MGYA00107672/file/ERR1429278_FASTQ_otu.tsv	Root-Environmental-Aquatic-Freshwater-Lake

ERR1897111	MGYA00351056	https://www.ebi.ac.uk/metagenomics/api/v1/analyses/MGYA00351056/file/ERR1897111_FASTQ_SSU_OTU.tsv	Root-Environmental-Aquatic-Freshwater-Lake
ERR1896751	MGYA00350696	https://www.ebi.ac.uk/metagenomics/api/v1/analyses/MGYA00350696/file/ERR1896751_FASTQ_SSU_OTU.tsv	Root-Environmental-Aquatic-Freshwater-Lake
ERR1897570	MGYA00351515	https://www.ebi.ac.uk/metagenomics/api/v1/analyses/MGYA00351515/file/ERR1897570_FASTQ_SSU_OTU.tsv	Root-Environmental-Aquatic-Freshwater-Lake
ERR1897049	MGYA00350994	https://www.ebi.ac.uk/metagenomics/api/v1/analyses/MGYA00350994/file/ERR1897049_FASTQ_SSU_OTU.tsv	Root-Environmental-Aquatic-Freshwater-Lake
ERR1582205	MGYA00352442	https://www.ebi.ac.uk/metagenomics/api/v1/analyses/MGYA00352442/file/ERR1582205_FASTQ_SSU_OTU.tsv	Root-Environmental-Aquatic-Freshwater-Lake
ERR1583445	MGYA00353682	https://www.ebi.ac.uk/metagenomics/api/v1/analyses/MGYA00353682/file/ERR1583445_FASTQ_SSU_OTU.tsv	Root-Environmental-Aquatic-Freshwater-Lake
ERR1897428	MGYA00351373	https://www.ebi.ac.uk/metagenomics/api/v1/analyses/MGYA00351373/file/ERR1897428_FASTQ_SSU_OTU.tsv	Root-Environmental-Aquatic-Freshwater-Lake
ERR1583024	MGYA00353261	https://www.ebi.ac.uk/metagenomics/api/v1/analyses/MGYA00353261/file/ERR1583024_FASTQ_SSU_OTU.tsv	Root-Environmental-Aquatic-Freshwater-Lake
ERR1582664	MGYA00352901	https://www.ebi.ac.uk/metagenomics/api/v1/analyses/MGYA00352901/file/ERR1582664_FASTQ_SSU_OTU.tsv	Root-Environmental-Aquatic-Freshwater-Lake
ERR1582112	MGYA00352349	https://www.ebi.ac.uk/metagenomics/api/v1/analyses/MGYA00352349/file/ERR1582112_FASTQ_SSU_OTU.tsv	Root-Environmental-Aquatic-Freshwater-Lake
ERR1583333	MGYA00353570	https://www.ebi.ac.uk/metagenomics/api/v1/analyses/MGYA00353570/file/ERR1583333_FASTQ_SSU_OTU.tsv	Root-Environmental-Aquatic-Freshwater-Lake
ERR1583494	MGYA00353731	https://www.ebi.ac.uk/metagenomics/api/v1/analyses/MGYA00353731/file/ERR1583494_FASTQ_SSU_OTU.tsv	Root-Environmental-Aquatic-Freshwater-Lake

ERR1582573	MGYA00352810	https://www.ebi.ac.uk/metagenomics/api/v1/analyses/MGYA00352810/file/ERR1582573_FASTQ_SSU_OTU.tsv	Root-Environmental-Aquatic-Freshwater-Lake
ERR1582901	MGYA00353138	https://www.ebi.ac.uk/metagenomics/api/v1/analyses/MGYA00353138/file/ERR1582901_FASTQ_SSU_OTU.tsv	Root-Environmental-Aquatic-Freshwater-Lake
ERR1897098	MGYA00351043	https://www.ebi.ac.uk/metagenomics/api/v1/analyses/MGYA00351043/file/ERR1897098_FASTQ_SSU_OTU.tsv	Root-Environmental-Aquatic-Freshwater-Lake
ERR1896833	MGYA00350778	https://www.ebi.ac.uk/metagenomics/api/v1/analyses/MGYA00350778/file/ERR1896833_FASTQ_SSU_OTU.tsv	Root-Environmental-Aquatic-Freshwater-Lake
ERR1897201	MGYA00351146	https://www.ebi.ac.uk/metagenomics/api/v1/analyses/MGYA00351146/file/ERR1897201_FASTQ_SSU_OTU.tsv	Root-Environmental-Aquatic-Freshwater-Lake
ERR1897660	MGYA00351605	https://www.ebi.ac.uk/metagenomics/api/v1/analyses/MGYA00351605/file/ERR1897660_FASTQ_SSU_OTU.tsv	Root-Environmental-Aquatic-Freshwater-Lake
ERR1896787	MGYA00350732	https://www.ebi.ac.uk/metagenomics/api/v1/analyses/MGYA00350732/file/ERR1896787_FASTQ_SSU_OTU.tsv	Root-Environmental-Aquatic-Freshwater-Lake
ERR1896756	MGYA00350701	https://www.ebi.ac.uk/metagenomics/api/v1/analyses/MGYA00350701/file/ERR1896756_FASTQ_SSU_OTU.tsv	Root-Environmental-Aquatic-Freshwater-Lake
ERR1897116	MGYA00351061	https://www.ebi.ac.uk/metagenomics/api/v1/analyses/MGYA00351061/file/ERR1897116_FASTQ_SSU_OTU.tsv	Root-Environmental-Aquatic-Freshwater-Lake
ERR1437520	MGYA00107954	https://www.ebi.ac.uk/metagenomics/api/v1/analyses/MGYA00107954/file/ERR1437520_FASTQ_otu.tsv	Root-Environmental-Aquatic-Freshwater-Lake
ERR1897577	MGYA00351522	https://www.ebi.ac.uk/metagenomics/api/v1/analyses/MGYA00351522/file/ERR1897577_FASTQ_SSU_OTU.tsv	Root-Environmental-Aquatic-Freshwater-Lake
ERR1583442	MGYA00353679	https://www.ebi.ac.uk/metagenomics/api/v1/analyses/MGYA00353679/file/ERR1583442_FASTQ_SSU_OTU.tsv	Root-Environmental-Aquatic-Freshwater-Lake

ERR1582202	MGYA00352439	https://www.ebi.ac.uk/metagenomics/api/v1/analyses/MGYA00352439/file/ERR1582202_FASTQ_SSU_OTU.tsv	Root-Environmental-Aquatic-Freshwater-Lake
ERR1897388	MGYA00351333	https://www.ebi.ac.uk/metagenomics/api/v1/analyses/MGYA00351333/file/ERR1897388_FASTQ_SSU_OTU.tsv	Root-Environmental-Aquatic-Freshwater-Lake
SRR2442959	MGYA00358963	https://www.ebi.ac.uk/metagenomics/api/v1/analyses/MGYA00358963/file/SRR2442959_MERGED_FASTQ_SSU_OTU.tsv	Root-Environmental-Aquatic-Freshwater-Lake
ERR1582663	MGYA00352900	https://www.ebi.ac.uk/metagenomics/api/v1/analyses/MGYA00352900/file/ERR1582663_FASTQ_SSU_OTU.tsv	Root-Environmental-Aquatic-Freshwater-Lake
ERR1583023	MGYA00353260	https://www.ebi.ac.uk/metagenomics/api/v1/analyses/MGYA00353260/file/ERR1583023_FASTQ_SSU_OTU.tsv	Root-Environmental-Aquatic-Freshwater-Lake
ERR1582115	MGYA00352352	https://www.ebi.ac.uk/metagenomics/api/v1/analyses/MGYA00352352/file/ERR1582115_FASTQ_SSU_OTU.tsv	Root-Environmental-Aquatic-Freshwater-Lake
SRR2442988	MGYA00358992	https://www.ebi.ac.uk/metagenomics/api/v1/analyses/MGYA00358992/file/SRR2442988_MERGED_FASTQ_SSU_OTU.tsv	Root-Environmental-Aquatic-Freshwater-Lake
ERR1897359	MGYA00351304	https://www.ebi.ac.uk/metagenomics/api/v1/analyses/MGYA00351304/file/ERR1897359_FASTQ_SSU_OTU.tsv	Root-Environmental-Aquatic-Freshwater-Lake
ERR1582906	MGYA00353143	https://www.ebi.ac.uk/metagenomics/api/v1/analyses/MGYA00353143/file/ERR1582906_FASTQ_SSU_OTU.tsv	Root-Environmental-Aquatic-Freshwater-Lake
ERR1582574	MGYA00352811	https://www.ebi.ac.uk/metagenomics/api/v1/analyses/MGYA00352811/file/ERR1582574_FASTQ_SSU_OTU.tsv	Root-Environmental-Aquatic-Freshwater-Lake
ERR1583493	MGYA00353730	https://www.ebi.ac.uk/metagenomics/api/v1/analyses/MGYA00353730/file/ERR1583493_FASTQ_SSU_OTU.tsv	Root-Environmental-Aquatic-Freshwater-Lake
ERR1583334	MGYA00353571	https://www.ebi.ac.uk/metagenomics/api/v1/analyses/MGYA00353571/file/ERR1583334_FASTQ_SSU_OTU.tsv	Root-Environmental-Aquatic-Freshwater-Lake

ERR1897738	MGYA00351683	https://www.ebi.ac.uk/metagenomics/api/v1/analyses/MGYA00351683/file/ERR1897738_FASTQ_SSU_OTU.tsv	Root-Environmental-Aquatic-Freshwater-Lake
ERR1582031	MGYA00352268	https://www.ebi.ac.uk/metagenomics/api/v1/analyses/MGYA00352268/file/ERR1582031_FASTQ_SSU_OTU.tsv	Root-Environmental-Aquatic-Freshwater-Lake
ERR1314351	MGYA00095164	https://www.ebi.ac.uk/metagenomics/api/v1/analyses/MGYA00095164/file/ERR1314351_MERGED_FASTQ_otu.tsv	Root-Environmental-Aquatic-Freshwater-Lake
ERR1582796	MGYA00353033	https://www.ebi.ac.uk/metagenomics/api/v1/analyses/MGYA00353033/file/ERR1582796_FASTQ_SSU_OTU.tsv	Root-Environmental-Aquatic-Freshwater-Lake
ERR1547096	MGYA00320807	https://www.ebi.ac.uk/metagenomics/api/v1/analyses/MGYA00320807/file/ERR1547096_FASTQ_SSU_OTU.tsv	Root-Environmental-Aquatic-Freshwater-Lake
ERR1582450	MGYA00352687	https://www.ebi.ac.uk/metagenomics/api/v1/analyses/MGYA00352687/file/ERR1582450_FASTQ_SSU_OTU.tsv	Root-Environmental-Aquatic-Freshwater-Lake
ERR1582822	MGYA00353059	https://www.ebi.ac.uk/metagenomics/api/v1/analyses/MGYA00353059/file/ERR1582822_FASTQ_SSU_OTU.tsv	Root-Environmental-Aquatic-Freshwater-Lake
ERR1583210	MGYA00353447	https://www.ebi.ac.uk/metagenomics/api/v1/analyses/MGYA00353447/file/ERR1583210_FASTQ_SSU_OTU.tsv	Root-Environmental-Aquatic-Freshwater-Lake
ERR1582481	MGYA00352718	https://www.ebi.ac.uk/metagenomics/api/v1/analyses/MGYA00352718/file/ERR1582481_FASTQ_SSU_OTU.tsv	Root-Environmental-Aquatic-Freshwater-Lake
ERR1582326	MGYA00352563	https://www.ebi.ac.uk/metagenomics/api/v1/analyses/MGYA00352563/file/ERR1582326_FASTQ_SSU_OTU.tsv	Root-Environmental-Aquatic-Freshwater-Lake
ERR1743278	MGYA00087131	https://www.ebi.ac.uk/metagenomics/api/v1/analyses/MGYA00087131/file/ERR1743278_MERGED_FASTQ_otu.tsv	Root-Environmental-Aquatic-Freshwater-Lake
ERR1547047	MGYA00320758	https://www.ebi.ac.uk/metagenomics/api/v1/analyses/MGYA00320758/file/ERR1547047_FASTQ_SSU_OTU.tsv	Root-Environmental-Aquatic-Freshwater-Lake

ERR1582747	MGYA00352984	https://www.ebi.ac.uk/metagenomics/api/v1/analyses/MGYA00352984/file/ERR1582747_FASTQ_SSU_OTU.tsv	Root-Environmental-Aquatic-Freshwater-Lake
ERR1314380	MGYA00095204	https://www.ebi.ac.uk/metagenomics/api/v1/analyses/MGYA00095204/file/ERR1314380_MERGED_FASTQ_otu.tsv	Root-Environmental-Aquatic-Freshwater-Lake
ERR1583107	MGYA00353344	https://www.ebi.ac.uk/metagenomics/api/v1/analyses/MGYA00353344/file/ERR1583107_FASTQ_SSU_OTU.tsv	Root-Environmental-Aquatic-Freshwater-Lake
ERR1583399	MGYA00353636	https://www.ebi.ac.uk/metagenomics/api/v1/analyses/MGYA00353636/file/ERR1583399_FASTQ_SSU_OTU.tsv	Root-Environmental-Aquatic-Freshwater-Lake
ERR1896672	MGYA00350617	https://www.ebi.ac.uk/metagenomics/api/v1/analyses/MGYA00350617/file/ERR1896672_FASTQ_SSU_OTU.tsv	Root-Environmental-Aquatic-Freshwater-Lake
ERR1897032	MGYA00350977	https://www.ebi.ac.uk/metagenomics/api/v1/analyses/MGYA00350977/file/ERR1897032_FASTQ_SSU_OTU.tsv	Root-Environmental-Aquatic-Freshwater-Lake
ERR1897453	MGYA00351398	https://www.ebi.ac.uk/metagenomics/api/v1/analyses/MGYA00351398/file/ERR1897453_FASTQ_SSU_OTU.tsv	Root-Environmental-Aquatic-Freshwater-Lake
ERR1743320	MGYA00087151	https://www.ebi.ac.uk/metagenomics/api/v1/analyses/MGYA00087151/file/ERR1743320_MERGED_FASTQ_otu.tsv	Root-Environmental-Aquatic-Freshwater-Lake
ERR1896917	MGYA00350862	https://www.ebi.ac.uk/metagenomics/api/v1/analyses/MGYA00350862/file/ERR1896917_FASTQ_SSU_OTU.tsv	Root-Environmental-Aquatic-Freshwater-Lake
ERR1897482	MGYA00351427	https://www.ebi.ac.uk/metagenomics/api/v1/analyses/MGYA00351427/file/ERR1897482_FASTQ_SSU_OTU.tsv	Root-Environmental-Aquatic-Freshwater-Lake
ERR1897325	MGYA00351270	https://www.ebi.ac.uk/metagenomics/api/v1/analyses/MGYA00351270/file/ERR1897325_FASTQ_SSU_OTU.tsv	Root-Environmental-Aquatic-Freshwater-Lake
ERR1582169	MGYA00352406	https://www.ebi.ac.uk/metagenomics/api/v1/analyses/MGYA00352406/file/ERR1582169_FASTQ_SSU_OTU.tsv	Root-Environmental-Aquatic-Freshwater-Lake

ERR1897744	MGYA00351689	https://www.ebi.ac.uk/metagenomics/api/v1/analyses/MGYA00351689/file/ERR1897744_FASTQ_SSU_OTU.tsv	Root-Environmental-Aquatic-Freshwater-Lake
SRR2443000	MGYA00359004	https://www.ebi.ac.uk/metagenomics/api/v1/analyses/MGYA00359004/file/SRR2443000_MERGED_FASTQ_SSU_OTU.tsv	Root-Environmental-Aquatic-Freshwater-Lake
ERR1582508	MGYA00352745	https://www.ebi.ac.uk/metagenomics/api/v1/analyses/MGYA00352745/file/ERR1582508_FASTQ_SSU_OTU.tsv	Root-Environmental-Aquatic-Freshwater-Lake
ERR1583348	MGYA00353585	https://www.ebi.ac.uk/metagenomics/api/v1/analyses/MGYA00353585/file/ERR1583348_FASTQ_SSU_OTU.tsv	Root-Environmental-Aquatic-Freshwater-Lake
SRR7081211	MGYA00425711	https://www.ebi.ac.uk/metagenomics/api/v1/analyses/MGYA00425711/file/SRR7081211_MERGED_FASTQ_SSU_OTU.tsv	Root-Environmental-Aquatic-Freshwater-Lake
ERR1743379	MGYA00087142	https://www.ebi.ac.uk/metagenomics/api/v1/analyses/MGYA00087142/file/ERR1743379_MERGED_FASTQ_otu.tsv	Root-Environmental-Aquatic-Freshwater-Lake
ERR1582646	MGYA00352883	https://www.ebi.ac.uk/metagenomics/api/v1/analyses/MGYA00352883/file/ERR1582646_FASTQ_SSU_OTU.tsv	Root-Environmental-Aquatic-Freshwater-Lake
ERR1583006	MGYA00353243	https://www.ebi.ac.uk/metagenomics/api/v1/analyses/MGYA00353243/file/ERR1583006_FASTQ_SSU_OTU.tsv	Root-Environmental-Aquatic-Freshwater-Lake
ERR1583467	MGYA00353704	https://www.ebi.ac.uk/metagenomics/api/v1/analyses/MGYA00353704/file/ERR1583467_FASTQ_SSU_OTU.tsv	Root-Environmental-Aquatic-Freshwater-Lake
ERR1582580	MGYA00352817	https://www.ebi.ac.uk/metagenomics/api/v1/analyses/MGYA00352817/file/ERR1582580_FASTQ_SSU_OTU.tsv	Root-Environmental-Aquatic-Freshwater-Lake
ERR1582227	MGYA00352464	https://www.ebi.ac.uk/metagenomics/api/v1/analyses/MGYA00352464/file/ERR1582227_FASTQ_SSU_OTU.tsv	Root-Environmental-Aquatic-Freshwater-Lake
ERR1582551	MGYA00352788	https://www.ebi.ac.uk/metagenomics/api/v1/analyses/MGYA00352788/file/ERR1582551_FASTQ_SSU_OTU.tsv	Root-Environmental-Aquatic-Freshwater-Lake

ERR1582923	MGYA00353160	https://www.ebi.ac.uk/metagenomics/api/v1/analyses/MGYA00353160/file/ERR1582923_FASTQ_SSU_OTU.tsv	Root-Environmental-Aquatic-Freshwater-Lake
ERR1583311	MGYA00353548	https://www.ebi.ac.uk/metagenomics/api/v1/analyses/MGYA00353548/file/ERR1583311_FASTQ_SSU_OTU.tsv	Root-Environmental-Aquatic-Freshwater-Lake
ERR1582130	MGYA00352367	https://www.ebi.ac.uk/metagenomics/api/v1/analyses/MGYA00352367/file/ERR1582130_FASTQ_SSU_OTU.tsv	Root-Environmental-Aquatic-Freshwater-Lake
ERR1582697	MGYA00352934	https://www.ebi.ac.uk/metagenomics/api/v1/analyses/MGYA00352934/file/ERR1582697_FASTQ_SSU_OTU.tsv	Root-Environmental-Aquatic-Freshwater-Lake
ERR589309	MGYA00103446	https://www.ebi.ac.uk/metagenomics/api/v1/analyses/MGYA00103446/file/ERR589309_FASTQ_otu.tsv	Root-Environmental-Aquatic-Freshwater-Lake
ERR1897645	MGYA00351590	https://www.ebi.ac.uk/metagenomics/api/v1/analyses/MGYA00351590/file/ERR1897645_FASTQ_SSU_OTU.tsv	Root-Environmental-Aquatic-Freshwater-Lake
ERR1535969	MGYA00320260	https://www.ebi.ac.uk/metagenomics/api/v1/analyses/MGYA00320260/file/ERR1535969_FASTQ_SSU_OTU.tsv	Root-Environmental-Aquatic-Freshwater-Lake
ERR1582409	MGYA00352646	https://www.ebi.ac.uk/metagenomics/api/v1/analyses/MGYA00352646/file/ERR1582409_FASTQ_SSU_OTU.tsv	Root-Environmental-Aquatic-Freshwater-Lake
ERR1583249	MGYA00353486	https://www.ebi.ac.uk/metagenomics/api/v1/analyses/MGYA00353486/file/ERR1583249_FASTQ_SSU_OTU.tsv	Root-Environmental-Aquatic-Freshwater-Lake
ERR1896816	MGYA00350761	https://www.ebi.ac.uk/metagenomics/api/v1/analyses/MGYA00350761/file/ERR1896816_FASTQ_SSU_OTU.tsv	Root-Environmental-Aquatic-Freshwater-Lake
ERR1897583	MGYA00351528	https://www.ebi.ac.uk/metagenomics/api/v1/analyses/MGYA00351528/file/ERR1897583_FASTQ_SSU_OTU.tsv	Root-Environmental-Aquatic-Freshwater-Lake
ERR1897224	MGYA00351169	https://www.ebi.ac.uk/metagenomics/api/v1/analyses/MGYA00351169/file/ERR1897224_FASTQ_SSU_OTU.tsv	Root-Environmental-Aquatic-Freshwater-Lake

ERR1582068	MGYA00352305	https://www.ebi.ac.uk/metagenomics/api/v1/analyses/MGYA00352305/file/ERR1582068_FASTQ_SSU_OTU.tsv	Root-Environmental-Aquatic-Freshwater-Lake
ERR1897552	MGYA00351497	https://www.ebi.ac.uk/metagenomics/api/v1/analyses/MGYA00351497/file/ERR1897552_FASTQ_SSU_OTU.tsv	Root-Environmental-Aquatic-Freshwater-Lake
SRR7081349	MGYA00427681	https://www.ebi.ac.uk/metagenomics/api/v1/analyses/MGYA00427681/file/SRR7081349_MERGED_FASTQ_SSU_OTU.tsv	Root-Environmental-Aquatic-Freshwater-Lake
ERR1583298	MGYA00353535	https://www.ebi.ac.uk/metagenomics/api/v1/analyses/MGYA00353535/file/ERR1583298_FASTQ_SSU_OTU.tsv	Root-Environmental-Aquatic-Freshwater-Lake
ERR658186	MGYA00103471	https://www.ebi.ac.uk/metagenomics/api/v1/analyses/MGYA00103471/file/ERR658186_FASTQ_otu.tsv	Root-Environmental-Aquatic-Freshwater-Lake
ERR1896773	MGYA00350718	https://www.ebi.ac.uk/metagenomics/api/v1/analyses/MGYA00350718/file/ERR1896773_FASTQ_SSU_OTU.tsv	Root-Environmental-Aquatic-Freshwater-Lake
ERR1897133	MGYA00351078	https://www.ebi.ac.uk/metagenomics/api/v1/analyses/MGYA00351078/file/ERR1897133_FASTQ_SSU_OTU.tsv	Root-Environmental-Aquatic-Freshwater-Lake
ERR1897694	MGYA00351639	https://www.ebi.ac.uk/metagenomics/api/v1/analyses/MGYA00351639/file/ERR1897694_FASTQ_SSU_OTU.tsv	Root-Environmental-Aquatic-Freshwater-Lake
ERR1897476	MGYA00351421	https://www.ebi.ac.uk/metagenomics/api/v1/analyses/MGYA00351421/file/ERR1897476_FASTQ_SSU_OTU.tsv	Root-Environmental-Aquatic-Freshwater-Lake
ERR1743305	MGYA00087136	https://www.ebi.ac.uk/metagenomics/api/v1/analyses/MGYA00087136/file/ERR1743305_MERGED_FASTQ_otu.tsv	Root-Environmental-Aquatic-Freshwater-Lake
ERR430928	MGYA00103256	https://www.ebi.ac.uk/metagenomics/api/v1/analyses/MGYA00103256/file/ERR430928_FASTQ_otu.tsv	Root-Environmental-Aquatic-Freshwater-Lake
ERR1896657	MGYA00350602	https://www.ebi.ac.uk/metagenomics/api/v1/analyses/MGYA00350602/file/ERR1896657_FASTQ_SSU_OTU.tsv	Root-Environmental-Aquatic-Freshwater-Lake

ERR1897017	MGYA00350962	https://www.ebi.ac.uk/metagenomics/api/v1/analyses/MGYA00350962/file/ERR1897017_FASTQ_SSU_OTU.tsv	Root-Environmental-Aquatic-Freshwater-Lake
ERR1897761	MGYA00351706	https://www.ebi.ac.uk/metagenomics/api/v1/analyses/MGYA00351706/file/ERR1897761_FASTQ_SSU_OTU.tsv	Root-Environmental-Aquatic-Freshwater-Lake
SRR2443025	MGYA00359029	https://www.ebi.ac.uk/metagenomics/api/v1/analyses/MGYA00359029/file/SRR2443025_MERGED_FASTQ_SSU_OTU.tsv	Root-Environmental-Aquatic-Freshwater-Lake
ERR1896686	MGYA00350631	https://www.ebi.ac.uk/metagenomics/api/v1/analyses/MGYA00350631/file/ERR1896686_FASTQ_SSU_OTU.tsv	Root-Environmental-Aquatic-Freshwater-Lake
ERR1990496	MGYA00128209	https://www.ebi.ac.uk/metagenomics/api/v1/analyses/MGYA00128209/file/ERR1990496_MERGED_FASTQ_otu.tsv	Root-Environmental-Aquatic-Freshwater-Lake
ERR1896932	MGYA00350877	https://www.ebi.ac.uk/metagenomics/api/v1/analyses/MGYA00350877/file/ERR1896932_FASTQ_SSU_OTU.tsv	Root-Environmental-Aquatic-Freshwater-Lake
ERR1897300	MGYA00351245	https://www.ebi.ac.uk/metagenomics/api/v1/analyses/MGYA00351245/file/ERR1897300_FASTQ_SSU_OTU.tsv	Root-Environmental-Aquatic-Freshwater-Lake
ERR1582807	MGYA00353044	https://www.ebi.ac.uk/metagenomics/api/v1/analyses/MGYA00353044/file/ERR1582807_FASTQ_SSU_OTU.tsv	Root-Environmental-Aquatic-Freshwater-Lake
ERR1582475	MGYA00352712	https://www.ebi.ac.uk/metagenomics/api/v1/analyses/MGYA00352712/file/ERR1582475_FASTQ_SSU_OTU.tsv	Root-Environmental-Aquatic-Freshwater-Lake
ERR1583235	MGYA00353472	https://www.ebi.ac.uk/metagenomics/api/v1/analyses/MGYA00353472/file/ERR1583235_FASTQ_SSU_OTU.tsv	Root-Environmental-Aquatic-Freshwater-Lake
ERR1897639	MGYA00351584	https://www.ebi.ac.uk/metagenomics/api/v1/analyses/MGYA00351584/file/ERR1897639_FASTQ_SSU_OTU.tsv	Root-Environmental-Aquatic-Freshwater-Lake
SRR7081022	MGYA00413218	https://www.ebi.ac.uk/metagenomics/api/v1/analyses/MGYA00413218/file/SRR7081022_MERGED_FASTQ_SSU_OTU.tsv	Root-Environmental-Aquatic-Freshwater-Lake

ERR1314374	MGYA00095210	https://www.ebi.ac.uk/metagenomics/api/v1/analyses/MGYA00095210/file/ERR1314374_MERGED_FASTQ_otu.tsv	Root-Environmental-Aquatic-Freshwater-Lake
ERR1897258	MGYA00351203	https://www.ebi.ac.uk/metagenomics/api/v1/analyses/MGYA00351203/file/ERR1897258_FASTQ_SSU_OTU.tsv	Root-Environmental-Aquatic-Freshwater-Lake
ERR1897289	MGYA00351234	https://www.ebi.ac.uk/metagenomics/api/v1/analyses/MGYA00351234/file/ERR1897289_FASTQ_SSU_OTU.tsv	Root-Environmental-Aquatic-Freshwater-Lake
SRR7081335	MGYA00424190	https://www.ebi.ac.uk/metagenomics/api/v1/analyses/MGYA00424190/file/SRR7081335_MERGED_FASTQ_SSU_OTU.tsv	Root-Environmental-Aquatic-Freshwater-Lake
ERR1547062	MGYA00320773	https://www.ebi.ac.uk/metagenomics/api/v1/analyses/MGYA00320773/file/ERR1547062_FASTQ_SSU_OTU.tsv	Root-Environmental-Aquatic-Freshwater-Lake
ERR1582762	MGYA00352999	https://www.ebi.ac.uk/metagenomics/api/v1/analyses/MGYA00352999/file/ERR1582762_FASTQ_SSU_OTU.tsv	Root-Environmental-Aquatic-Freshwater-Lake
ERR1314402	MGYA00095179	https://www.ebi.ac.uk/metagenomics/api/v1/analyses/MGYA00095179/file/ERR1314402_MERGED_FASTQ_otu.tsv	Root-Environmental-Aquatic-Freshwater-Lake
ERR1583122	MGYA00353359	https://www.ebi.ac.uk/metagenomics/api/v1/analyses/MGYA00353359/file/ERR1583122_FASTQ_SSU_OTU.tsv	Root-Environmental-Aquatic-Freshwater-Lake
ERR1429226	MGYA00107645	https://www.ebi.ac.uk/metagenomics/api/v1/analyses/MGYA00107645/file/ERR1429226_FASTQ_otu.tsv	Root-Environmental-Aquatic-Freshwater-Lake
ERR1582303	MGYA00352540	https://www.ebi.ac.uk/metagenomics/api/v1/analyses/MGYA00352540/file/ERR1582303_FASTQ_SSU_OTU.tsv	Root-Environmental-Aquatic-Freshwater-Lake
ERR1743302	MGYA00087174	https://www.ebi.ac.uk/metagenomics/api/v1/analyses/MGYA00087174/file/ERR1743302_MERGED_FASTQ_otu.tsv	Root-Environmental-Aquatic-Freshwater-Lake
ERR1897471	MGYA00351416	https://www.ebi.ac.uk/metagenomics/api/v1/analyses/MGYA00351416/file/ERR1897471_FASTQ_SSU_OTU.tsv	Root-Environmental-Aquatic-Freshwater-Lake

ERR1582989	MGYA00353226	https://www.ebi.ac.uk/metagenomics/api/v1/analyses/MGYA00353226/file/ERR1582989_FASTQ_SSU_OTU.tsv	Root-Environmental-Aquatic-Freshwater-Lake
ERR1897010	MGYA00350955	https://www.ebi.ac.uk/metagenomics/api/v1/analyses/MGYA00350955/file/ERR1897010_FASTQ_SSU_OTU.tsv	Root-Environmental-Aquatic-Freshwater-Lake
ERR1896650	MGYA00350595	https://www.ebi.ac.uk/metagenomics/api/v1/analyses/MGYA00350595/file/ERR1896650_FASTQ_SSU_OTU.tsv	Root-Environmental-Aquatic-Freshwater-Lake
ERR1896681	MGYA00350626	https://www.ebi.ac.uk/metagenomics/api/v1/analyses/MGYA00350626/file/ERR1896681_FASTQ_SSU_OTU.tsv	Root-Environmental-Aquatic-Freshwater-Lake
ERR1897766	MGYA00351711	https://www.ebi.ac.uk/metagenomics/api/v1/analyses/MGYA00351711/file/ERR1897766_FASTQ_SSU_OTU.tsv	Root-Environmental-Aquatic-Freshwater-Lake
SRR2443022	MGYA00359026	https://www.ebi.ac.uk/metagenomics/api/v1/analyses/MGYA00359026/file/SRR2443022_MERGED_FASTQ_SSU_OTU.tsv	Root-Environmental-Aquatic-Freshwater-Lake
ERR1990491	MGYA00128205	https://www.ebi.ac.uk/metagenomics/api/v1/analyses/MGYA00128205/file/ERR1990491_MERGED_FASTQ_otu.tsv	Root-Environmental-Aquatic-Freshwater-Lake
ERR1582958	MGYA00353195	https://www.ebi.ac.uk/metagenomics/api/v1/analyses/MGYA00353195/file/ERR1582958_FASTQ_SSU_OTU.tsv	Root-Environmental-Aquatic-Freshwater-Lake
ERR1897307	MGYA00351252	https://www.ebi.ac.uk/metagenomics/api/v1/analyses/MGYA00351252/file/ERR1897307_FASTQ_SSU_OTU.tsv	Root-Environmental-Aquatic-Freshwater-Lake
ERR1896935	MGYA00350880	https://www.ebi.ac.uk/metagenomics/api/v1/analyses/MGYA00350880/file/ERR1896935_FASTQ_SSU_OTU.tsv	Root-Environmental-Aquatic-Freshwater-Lake
ERR1583232	MGYA00353469	https://www.ebi.ac.uk/metagenomics/api/v1/analyses/MGYA00353469/file/ERR1583232_FASTQ_SSU_OTU.tsv	Root-Environmental-Aquatic-Freshwater-Lake
ERR1582472	MGYA00352709	https://www.ebi.ac.uk/metagenomics/api/v1/analyses/MGYA00352709/file/ERR1582472_FASTQ_SSU_OTU.tsv	Root-Environmental-Aquatic-Freshwater-Lake

ERR1582800	MGYA00353037	https://www.ebi.ac.uk/metagenomics/api/v1/analyses/MGYA00353037/file/ERR1582800_FASTQ_SSU_OTU.tsv	Root-Environmental-Aquatic-Freshwater-Lake
ERR1897199	MGYA00351144	https://www.ebi.ac.uk/metagenomics/api/v1/analyses/MGYA00351144/file/ERR1897199_FASTQ_SSU_OTU.tsv	Root-Environmental-Aquatic-Freshwater-Lake
ERR1314373	MGYA00095226	https://www.ebi.ac.uk/metagenomics/api/v1/analyses/MGYA00095226/file/ERR1314373_MERGED_FASTQ_otu.tsv	Root-Environmental-Aquatic-Freshwater-Lake
ERR1547065	MGYA00320776	https://www.ebi.ac.uk/metagenomics/api/v1/analyses/MGYA00320776/file/ERR1547065_FASTQ_SSU_OTU.tsv	Root-Environmental-Aquatic-Freshwater-Lake
ERR1897529	MGYA00351474	https://www.ebi.ac.uk/metagenomics/api/v1/analyses/MGYA00351474/file/ERR1897529_FASTQ_SSU_OTU.tsv	Root-Environmental-Aquatic-Freshwater-Lake
SRR7081332	MGYA00423844	https://www.ebi.ac.uk/metagenomics/api/v1/analyses/MGYA00423844/file/SRR7081332_MERGED_FASTQ_SSU_OTU.tsv	Root-Environmental-Aquatic-Freshwater-Lake
ERR1583125	MGYA00353362	https://www.ebi.ac.uk/metagenomics/api/v1/analyses/MGYA00353362/file/ERR1583125_FASTQ_SSU_OTU.tsv	Root-Environmental-Aquatic-Freshwater-Lake
ERR1314405	MGYA00095173	https://www.ebi.ac.uk/metagenomics/api/v1/analyses/MGYA00095173/file/ERR1314405_MERGED_FASTQ_otu.tsv	Root-Environmental-Aquatic-Freshwater-Lake
ERR1582765	MGYA00353002	https://www.ebi.ac.uk/metagenomics/api/v1/analyses/MGYA00353002/file/ERR1582765_FASTQ_SSU_OTU.tsv	Root-Environmental-Aquatic-Freshwater-Lake
ERR1897148	MGYA00351093	https://www.ebi.ac.uk/metagenomics/api/v1/analyses/MGYA00351093/file/ERR1897148_FASTQ_SSU_OTU.tsv	Root-Environmental-Aquatic-Freshwater-Lake
ERR1896708	MGYA00350653	https://www.ebi.ac.uk/metagenomics/api/v1/analyses/MGYA00350653/file/ERR1896708_FASTQ_SSU_OTU.tsv	Root-Environmental-Aquatic-Freshwater-Lake
ERR1582304	MGYA00352541	https://www.ebi.ac.uk/metagenomics/api/v1/analyses/MGYA00352541/file/ERR1582304_FASTQ_SSU_OTU.tsv	Root-Environmental-Aquatic-Freshwater-Lake

ERR1896998	MGYA00350943	https://www.ebi.ac.uk/metagenomics/api/v1/analyses/MGYA00350943/file/ERR1896998_FASTQ_SSU_OTU.tsv	Root-Environmental-Aquatic-Freshwater-Lake
ERR1583001	MGYA00353238	https://www.ebi.ac.uk/metagenomics/api/v1/analyses/MGYA00353238/file/ERR1583001_FASTQ_SSU_OTU.tsv	Root-Environmental-Aquatic-Freshwater-Lake
ERR1582641	MGYA00352878	https://www.ebi.ac.uk/metagenomics/api/v1/analyses/MGYA00352878/file/ERR1582641_FASTQ_SSU_OTU.tsv	Root-Environmental-Aquatic-Freshwater-Lake
ERR1582220	MGYA00352457	https://www.ebi.ac.uk/metagenomics/api/v1/analyses/MGYA00352457/file/ERR1582220_FASTQ_SSU_OTU.tsv	Root-Environmental-Aquatic-Freshwater-Lake
ERR1582587	MGYA00352824	https://www.ebi.ac.uk/metagenomics/api/v1/analyses/MGYA00352824/file/ERR1582587_FASTQ_SSU_OTU.tsv	Root-Environmental-Aquatic-Freshwater-Lake
ERR1583460	MGYA00353697	https://www.ebi.ac.uk/metagenomics/api/v1/analyses/MGYA00353697/file/ERR1583460_FASTQ_SSU_OTU.tsv	Root-Environmental-Aquatic-Freshwater-Lake
ERR1583316	MGYA00353553	https://www.ebi.ac.uk/metagenomics/api/v1/analyses/MGYA00353553/file/ERR1583316_FASTQ_SSU_OTU.tsv	Root-Environmental-Aquatic-Freshwater-Lake
ERR1582924	MGYA00353161	https://www.ebi.ac.uk/metagenomics/api/v1/analyses/MGYA00353161/file/ERR1582924_FASTQ_SSU_OTU.tsv	Root-Environmental-Aquatic-Freshwater-Lake
ERR1582556	MGYA00352793	https://www.ebi.ac.uk/metagenomics/api/v1/analyses/MGYA00352793/file/ERR1582556_FASTQ_SSU_OTU.tsv	Root-Environmental-Aquatic-Freshwater-Lake
ERR1582690	MGYA00352927	https://www.ebi.ac.uk/metagenomics/api/v1/analyses/MGYA00352927/file/ERR1582690_FASTQ_SSU_OTU.tsv	Root-Environmental-Aquatic-Freshwater-Lake
ERR1582137	MGYA00352374	https://www.ebi.ac.uk/metagenomics/api/v1/analyses/MGYA00352374/file/ERR1582137_FASTQ_SSU_OTU.tsv	Root-Environmental-Aquatic-Freshwater-Lake
ERR1896949	MGYA00350894	https://www.ebi.ac.uk/metagenomics/api/v1/analyses/MGYA00350894/file/ERR1896949_FASTQ_SSU_OTU.tsv	Root-Environmental-Aquatic-Freshwater-Lake

ERR1897642	MGYA00351587	https://www.ebi.ac.uk/metagenomics/api/v1/analyses/MGYA00351587/file/ERR1897642_FASTQ_SSU_OTU.tsv	Root-Environmental-Aquatic-Freshwater-Lake
ERR1897223	MGYA00351168	https://www.ebi.ac.uk/metagenomics/api/v1/analyses/MGYA00351168/file/ERR1897223_FASTQ_SSU_OTU.tsv	Root-Environmental-Aquatic-Freshwater-Lake
ERR1897584	MGYA00351529	https://www.ebi.ac.uk/metagenomics/api/v1/analyses/MGYA00351529/file/ERR1897584_FASTQ_SSU_OTU.tsv	Root-Environmental-Aquatic-Freshwater-Lake
ERR1896811	MGYA00350756	https://www.ebi.ac.uk/metagenomics/api/v1/analyses/MGYA00350756/file/ERR1896811_FASTQ_SSU_OTU.tsv	Root-Environmental-Aquatic-Freshwater-Lake
ERR1583188	MGYA00353425	https://www.ebi.ac.uk/metagenomics/api/v1/analyses/MGYA00353425/file/ERR1583188_FASTQ_SSU_OTU.tsv	Root-Environmental-Aquatic-Freshwater-Lake
ERR1583159	MGYA00353396	https://www.ebi.ac.uk/metagenomics/api/v1/analyses/MGYA00353396/file/ERR1583159_FASTQ_SSU_OTU.tsv	Root-Environmental-Aquatic-Freshwater-Lake
ERR1582719	MGYA00352956	https://www.ebi.ac.uk/metagenomics/api/v1/analyses/MGYA00352956/file/ERR1582719_FASTQ_SSU_OTU.tsv	Root-Environmental-Aquatic-Freshwater-Lake
ERR589287	MGYA00103405	https://www.ebi.ac.uk/metagenomics/api/v1/analyses/MGYA00103405/file/ERR589287_FASTQ_otu.tsv	Root-Environmental-Aquatic-Freshwater-Lake
ERR1897555	MGYA00351500	https://www.ebi.ac.uk/metagenomics/api/v1/analyses/MGYA00351500/file/ERR1897555_FASTQ_SSU_OTU.tsv	Root-Environmental-Aquatic-Freshwater-Lake
ERR1582378	MGYA00352615	https://www.ebi.ac.uk/metagenomics/api/v1/analyses/MGYA00352615/file/ERR1582378_FASTQ_SSU_OTU.tsv	Root-Environmental-Aquatic-Freshwater-Lake
ERR658181	MGYA00103469	https://www.ebi.ac.uk/metagenomics/api/v1/analyses/MGYA00103469/file/ERR658181_FASTQ_otu.tsv	Root-Environmental-Aquatic-Freshwater-Lake
ERR1897693	MGYA00351638	https://www.ebi.ac.uk/metagenomics/api/v1/analyses/MGYA00351638/file/ERR1897693_FASTQ_SSU_OTU.tsv	Root-Environmental-Aquatic-Freshwater-Lake

ERR1897134	MGYA00351079	https://www.ebi.ac.uk/metagenomics/api/v1/analyses/MGYA00351079/file/ERR1897134_FASTQ_SSU_OTU.tsv	Root-Environmental-Aquatic-Freshwater-Lake
ERR1896774	MGYA00350719	https://www.ebi.ac.uk/metagenomics/api/v1/analyses/MGYA00350719/file/ERR1896774_FASTQ_SSU_OTU.tsv	Root-Environmental-Aquatic-Freshwater-Lake
ERR1582194	MGYA00352431	https://www.ebi.ac.uk/metagenomics/api/v1/analyses/MGYA00352431/file/ERR1582194_FASTQ_SSU_OTU.tsv	Root-Environmental-Aquatic-Freshwater-Lake
ERR1582633	MGYA00352870	https://www.ebi.ac.uk/metagenomics/api/v1/analyses/MGYA00352870/file/ERR1582633_FASTQ_SSU_OTU.tsv	Root-Environmental-Aquatic-Freshwater-Lake
ERR1583073	MGYA00353310	https://www.ebi.ac.uk/metagenomics/api/v1/analyses/MGYA00353310/file/ERR1583073_FASTQ_SSU_OTU.tsv	Root-Environmental-Aquatic-Freshwater-Lake
ERR1583412	MGYA00353649	https://www.ebi.ac.uk/metagenomics/api/v1/analyses/MGYA00353649/file/ERR1583412_FASTQ_SSU_OTU.tsv	Root-Environmental-Aquatic-Freshwater-Lake
ERR1582987	MGYA00353224	https://www.ebi.ac.uk/metagenomics/api/v1/analyses/MGYA00353224/file/ERR1582987_FASTQ_SSU_OTU.tsv	Root-Environmental-Aquatic-Freshwater-Lake
ERR430921	MGYA00103263	https://www.ebi.ac.uk/metagenomics/api/v1/analyses/MGYA00103263/file/ERR430921_FASTQ_otu.tsv	Root-Environmental-Aquatic-Freshwater-Lake
ERR1582252	MGYA00352489	https://www.ebi.ac.uk/metagenomics/api/v1/analyses/MGYA00352489/file/ERR1582252_FASTQ_SSU_OTU.tsv	Root-Environmental-Aquatic-Freshwater-Lake
ERR1582956	MGYA00353193	https://www.ebi.ac.uk/metagenomics/api/v1/analyses/MGYA00353193/file/ERR1582956_FASTQ_SSU_OTU.tsv	Root-Environmental-Aquatic-Freshwater-Lake
ERR1582524	MGYA00352761	https://www.ebi.ac.uk/metagenomics/api/v1/analyses/MGYA00352761/file/ERR1582524_FASTQ_SSU_OTU.tsv	Root-Environmental-Aquatic-Freshwater-Lake
ERR1582283	MGYA00352520	https://www.ebi.ac.uk/metagenomics/api/v1/analyses/MGYA00352520/file/ERR1582283_FASTQ_SSU_OTU.tsv	Root-Environmental-Aquatic-Freshwater-Lake

ERR1583364	MGYA00353601	https://www.ebi.ac.uk/metagenomics/api/v1/analyses/MGYA00353601/file/ERR1583364_FASTQ_SSU_OTU.tsv	Root-Environmental-Aquatic-Freshwater-Lake
ERR1897768	MGYA00351713	https://www.ebi.ac.uk/metagenomics/api/v1/analyses/MGYA00351713/file/ERR1897768_FASTQ_SSU_OTU.tsv	Root-Environmental-Aquatic-Freshwater-Lake
ERR1582145	MGYA00352382	https://www.ebi.ac.uk/metagenomics/api/v1/analyses/MGYA00352382/file/ERR1582145_FASTQ_SSU_OTU.tsv	Root-Environmental-Aquatic-Freshwater-Lake
ERR1897309	MGYA00351254	https://www.ebi.ac.uk/metagenomics/api/v1/analyses/MGYA00351254/file/ERR1897309_FASTQ_SSU_OTU.tsv	Root-Environmental-Aquatic-Freshwater-Lake
ERR1897197	MGYA00351142	https://www.ebi.ac.uk/metagenomics/api/v1/analyses/MGYA00351142/file/ERR1897197_FASTQ_SSU_OTU.tsv	Root-Environmental-Aquatic-Freshwater-Lake
ERR1897630	MGYA00351575	https://www.ebi.ac.uk/metagenomics/api/v1/analyses/MGYA00351575/file/ERR1897630_FASTQ_SSU_OTU.tsv	Root-Environmental-Aquatic-Freshwater-Lake
ERR1743285	MGYA00087209	https://www.ebi.ac.uk/metagenomics/api/v1/analyses/MGYA00087209/file/ERR1743285_MERGED_FASTQ_otu.tsv	Root-Environmental-Aquatic-Freshwater-Lake
ERR1896863	MGYA00350808	https://www.ebi.ac.uk/metagenomics/api/v1/analyses/MGYA00350808/file/ERR1896863_FASTQ_SSU_OTU.tsv	Root-Environmental-Aquatic-Freshwater-Lake
ERR1897251	MGYA00351196	https://www.ebi.ac.uk/metagenomics/api/v1/analyses/MGYA00351196/file/ERR1897251_FASTQ_SSU_OTU.tsv	Root-Environmental-Aquatic-Freshwater-Lake
ERR1554593	MGYA00100723	https://www.ebi.ac.uk/metagenomics/api/v1/analyses/MGYA00100723/file/ERR1554593_MERGED_FASTQ_otu.tsv	Root-Environmental-Aquatic-Freshwater-Lake
ERR1897527	MGYA00351472	https://www.ebi.ac.uk/metagenomics/api/v1/analyses/MGYA00351472/file/ERR1897527_FASTQ_SSU_OTU.tsv	Root-Environmental-Aquatic-Freshwater-Lake
ERR1897280	MGYA00351225	https://www.ebi.ac.uk/metagenomics/api/v1/analyses/MGYA00351225/file/ERR1897280_FASTQ_SSU_OTU.tsv	Root-Environmental-Aquatic-Freshwater-Lake

ERR1896706	MGYA00350651	https://www.ebi.ac.uk/metagenomics/api/v1/analyses/MGYA00350651/file/ERR1896706_FASTQ_SSU_OTU.tsv	Root-Environmental-Aquatic-Freshwater-Lake
ERR1897146	MGYA00351091	https://www.ebi.ac.uk/metagenomics/api/v1/analyses/MGYA00351091/file/ERR1897146_FASTQ_SSU_OTU.tsv	Root-Environmental-Aquatic-Freshwater-Lake
ERR1897403	MGYA00351348	https://www.ebi.ac.uk/metagenomics/api/v1/analyses/MGYA00351348/file/ERR1897403_FASTQ_SSU_OTU.tsv	Root-Environmental-Aquatic-Freshwater-Lake
ERR1896996	MGYA00350941	https://www.ebi.ac.uk/metagenomics/api/v1/analyses/MGYA00350941/file/ERR1896996_FASTQ_SSU_OTU.tsv	Root-Environmental-Aquatic-Freshwater-Lake
ERR1743370	MGYA00087133	https://www.ebi.ac.uk/metagenomics/api/v1/analyses/MGYA00087133/file/ERR1743370_MERGED_FASTQ_otu.tsv	Root-Environmental-Aquatic-Freshwater-Lake
SRR2442975	MGYA00358979	https://www.ebi.ac.uk/metagenomics/api/v1/analyses/MGYA00358979/file/SRR2442975_MERGED_FASTQ_SSU_OTU.tsv	Root-Environmental-Aquatic-Freshwater-Lake
ERR1582589	MGYA00352826	https://www.ebi.ac.uk/metagenomics/api/v1/analyses/MGYA00352826/file/ERR1582589_FASTQ_SSU_OTU.tsv	Root-Environmental-Aquatic-Freshwater-Lake
ERR1897062	MGYA00351007	https://www.ebi.ac.uk/metagenomics/api/v1/analyses/MGYA00351007/file/ERR1897062_FASTQ_SSU_OTU.tsv	Root-Environmental-Aquatic-Freshwater-Lake
ERR1897714	MGYA00351659	https://www.ebi.ac.uk/metagenomics/api/v1/analyses/MGYA00351659/file/ERR1897714_FASTQ_SSU_OTU.tsv	Root-Environmental-Aquatic-Freshwater-Lake
ERR1582558	MGYA00352795	https://www.ebi.ac.uk/metagenomics/api/v1/analyses/MGYA00352795/file/ERR1582558_FASTQ_SSU_OTU.tsv	Root-Environmental-Aquatic-Freshwater-Lake
ERR1583318	MGYA00353555	https://www.ebi.ac.uk/metagenomics/api/v1/analyses/MGYA00353555/file/ERR1583318_FASTQ_SSU_OTU.tsv	Root-Environmental-Aquatic-Freshwater-Lake
ERR1896947	MGYA00350892	https://www.ebi.ac.uk/metagenomics/api/v1/analyses/MGYA00350892/file/ERR1896947_FASTQ_SSU_OTU.tsv	Root-Environmental-Aquatic-Freshwater-Lake

ERR1897375	MGYA00351320	https://www.ebi.ac.uk/metagenomics/api/v1/analyses/MGYA00351320/file/ERR1897375_FASTQ_SSU_OTU.tsv	Root-Environmental-Aquatic-Freshwater-Lake
ERR589300	MGYA00103406	https://www.ebi.ac.uk/metagenomics/api/v1/analyses/MGYA00103406/file/ERR589300_FASTQ_otu.tsv	Root-Environmental-Aquatic-Freshwater-Lake
ERR1582139	MGYA00352376	https://www.ebi.ac.uk/metagenomics/api/v1/analyses/MGYA00352376/file/ERR1582139_FASTQ_SSU_OTU.tsv	Root-Environmental-Aquatic-Freshwater-Lake
ERR1582400	MGYA00352637	https://www.ebi.ac.uk/metagenomics/api/v1/analyses/MGYA00352637/file/ERR1582400_FASTQ_SSU_OTU.tsv	Root-Environmental-Aquatic-Freshwater-Lake
ERR1582872	MGYA00353109	https://www.ebi.ac.uk/metagenomics/api/v1/analyses/MGYA00353109/file/ERR1582872_FASTQ_SSU_OTU.tsv	Root-Environmental-Aquatic-Freshwater-Lake
ERR1583240	MGYA00353477	https://www.ebi.ac.uk/metagenomics/api/v1/analyses/MGYA00353477/file/ERR1583240_FASTQ_SSU_OTU.tsv	Root-Environmental-Aquatic-Freshwater-Lake
ERR1742297	MGYA00311598	https://www.ebi.ac.uk/metagenomics/api/v1/analyses/MGYA00311598/file/ERR1742297_FASTQ_SSU_OTU.tsv	Root-Environmental-Aquatic-Freshwater-Lake
ERR1535960	MGYA00320251	https://www.ebi.ac.uk/metagenomics/api/v1/analyses/MGYA00320251/file/ERR1535960_FASTQ_SSU_OTU.tsv	Root-Environmental-Aquatic-Freshwater-Lake
ERR1583186	MGYA00353423	https://www.ebi.ac.uk/metagenomics/api/v1/analyses/MGYA00353423/file/ERR1583186_FASTQ_SSU_OTU.tsv	Root-Environmental-Aquatic-Freshwater-Lake
ERR1582061	MGYA00352298	https://www.ebi.ac.uk/metagenomics/api/v1/analyses/MGYA00352298/file/ERR1582061_FASTQ_SSU_OTU.tsv	Root-Environmental-Aquatic-Freshwater-Lake
ERR589289	MGYA00103457	https://www.ebi.ac.uk/metagenomics/api/v1/analyses/MGYA00103457/file/ERR589289_FASTQ_otu.tsv	Root-Environmental-Aquatic-Freshwater-Lake
ERR1582717	MGYA00352954	https://www.ebi.ac.uk/metagenomics/api/v1/analyses/MGYA00352954/file/ERR1582717_FASTQ_SSU_OTU.tsv	Root-Environmental-Aquatic-Freshwater-Lake

ERR1583157	MGYA00353394	https://www.ebi.ac.uk/metagenomics/api/v1/analyses/MGYA00353394/file/ERR1583157_FASTQ_SSU_OTU.tsv	Root-Environmental-Aquatic-Freshwater-Lake
ERR1583291	MGYA00353528	https://www.ebi.ac.uk/metagenomics/api/v1/analyses/MGYA00353528/file/ERR1583291_FASTQ_SSU_OTU.tsv	Root-Environmental-Aquatic-Freshwater-Lake
ERR1429253	MGYA00107629	https://www.ebi.ac.uk/metagenomics/api/v1/analyses/MGYA00107629/file/ERR1429253_FASTQ_otu.tsv	Root-Environmental-Aquatic-Freshwater-Lake
ERR1582376	MGYA00352613	https://www.ebi.ac.uk/metagenomics/api/v1/analyses/MGYA00352613/file/ERR1582376_FASTQ_SSU_OTU.tsv	Root-Environmental-Aquatic-Freshwater-Lake
ERR1583008	MGYA00353245	https://www.ebi.ac.uk/metagenomics/api/v1/analyses/MGYA00353245/file/ERR1583008_FASTQ_SSU_OTU.tsv	Root-Environmental-Aquatic-Freshwater-Lake
ERR1582648	MGYA00352885	https://www.ebi.ac.uk/metagenomics/api/v1/analyses/MGYA00352885/file/ERR1582648_FASTQ_SSU_OTU.tsv	Root-Environmental-Aquatic-Freshwater-Lake
SRR2442972	MGYA00358976	https://www.ebi.ac.uk/metagenomics/api/v1/analyses/MGYA00358976/file/SRR2442972_MERGED_FASTQ_SSU_OTU.tsv	Root-Environmental-Aquatic-Freshwater-Lake
ERR1743377	MGYA00087187	https://www.ebi.ac.uk/metagenomics/api/v1/analyses/MGYA00087187/file/ERR1743377_MERGED_FASTQ_otu.tsv	Root-Environmental-Aquatic-Freshwater-Lake
ERR1896991	MGYA00350936	https://www.ebi.ac.uk/metagenomics/api/v1/analyses/MGYA00350936/file/ERR1896991_FASTQ_SSU_OTU.tsv	Root-Environmental-Aquatic-Freshwater-Lake
ERR1897404	MGYA00351349	https://www.ebi.ac.uk/metagenomics/api/v1/analyses/MGYA00351349/file/ERR1897404_FASTQ_SSU_OTU.tsv	Root-Environmental-Aquatic-Freshwater-Lake
ERR1582229	MGYA00352466	https://www.ebi.ac.uk/metagenomics/api/v1/analyses/MGYA00352466/file/ERR1582229_FASTQ_SSU_OTU.tsv	Root-Environmental-Aquatic-Freshwater-Lake
ERR1583469	MGYA00353706	https://www.ebi.ac.uk/metagenomics/api/v1/analyses/MGYA00353706/file/ERR1583469_FASTQ_SSU_OTU.tsv	Root-Environmental-Aquatic-Freshwater-Lake

ERR1897065	MGYA00351010	https://www.ebi.ac.uk/metagenomics/api/v1/analyses/MGYA00351010/file/ERR1897065_FASTQ_SSU_OTU.tsv	Root-Environmental-Aquatic-Freshwater-Lake
ERR1897713	MGYA00351658	https://www.ebi.ac.uk/metagenomics/api/v1/analyses/MGYA00351658/file/ERR1897713_FASTQ_SSU_OTU.tsv	Root-Environmental-Aquatic-Freshwater-Lake
ERR589307	MGYA00103436	https://www.ebi.ac.uk/metagenomics/api/v1/analyses/MGYA00103436/file/ERR589307_FASTQ_otu.tsv	Root-Environmental-Aquatic-Freshwater-Lake
ERR1897372	MGYA00351317	https://www.ebi.ac.uk/metagenomics/api/v1/analyses/MGYA00351317/file/ERR1897372_FASTQ_SSU_OTU.tsv	Root-Environmental-Aquatic-Freshwater-Lake
ERR1896940	MGYA00350885	https://www.ebi.ac.uk/metagenomics/api/v1/analyses/MGYA00350885/file/ERR1896940_FASTQ_SSU_OTU.tsv	Root-Environmental-Aquatic-Freshwater-Lake
ERR1582699	MGYA00352936	https://www.ebi.ac.uk/metagenomics/api/v1/analyses/MGYA00352936/file/ERR1582699_FASTQ_SSU_OTU.tsv	Root-Environmental-Aquatic-Freshwater-Lake
ERR1583247	MGYA00353484	https://www.ebi.ac.uk/metagenomics/api/v1/analyses/MGYA00353484/file/ERR1583247_FASTQ_SSU_OTU.tsv	Root-Environmental-Aquatic-Freshwater-Lake
ERR1582875	MGYA00353112	https://www.ebi.ac.uk/metagenomics/api/v1/analyses/MGYA00353112/file/ERR1582875_FASTQ_SSU_OTU.tsv	Root-Environmental-Aquatic-Freshwater-Lake
ERR1582407	MGYA00352644	https://www.ebi.ac.uk/metagenomics/api/v1/analyses/MGYA00352644/file/ERR1582407_FASTQ_SSU_OTU.tsv	Root-Environmental-Aquatic-Freshwater-Lake
ERR1535967	MGYA00320258	https://www.ebi.ac.uk/metagenomics/api/v1/analyses/MGYA00320258/file/ERR1535967_FASTQ_SSU_OTU.tsv	Root-Environmental-Aquatic-Freshwater-Lake
ERR1742290	MGYA00311591	https://www.ebi.ac.uk/metagenomics/api/v1/analyses/MGYA00311591/file/ERR1742290_FASTQ_SSU_OTU.tsv	Root-Environmental-Aquatic-Freshwater-Lake
ERR1582066	MGYA00352303	https://www.ebi.ac.uk/metagenomics/api/v1/analyses/MGYA00352303/file/ERR1582066_FASTQ_SSU_OTU.tsv	Root-Environmental-Aquatic-Freshwater-Lake

ERR1583181	MGYA00353418	https://www.ebi.ac.uk/metagenomics/api/v1/analyses/MGYA00353418/file/ERR1583181_FASTQ_SSU_OTU.tsv	Root-Environmental-Aquatic-Freshwater-Lake
ERR1896818	MGYA00350763	https://www.ebi.ac.uk/metagenomics/api/v1/analyses/MGYA00350763/file/ERR1896818_FASTQ_SSU_OTU.tsv	Root-Environmental-Aquatic-Freshwater-Lake
ERR1583150	MGYA00353387	https://www.ebi.ac.uk/metagenomics/api/v1/analyses/MGYA00353387/file/ERR1583150_FASTQ_SSU_OTU.tsv	Root-Environmental-Aquatic-Freshwater-Lake
ERR1582710	MGYA00352947	https://www.ebi.ac.uk/metagenomics/api/v1/analyses/MGYA00352947/file/ERR1582710_FASTQ_SSU_OTU.tsv	Root-Environmental-Aquatic-Freshwater-Lake
ERR658188	MGYA00103504	https://www.ebi.ac.uk/metagenomics/api/v1/analyses/MGYA00103504/file/ERR658188_FASTQ_otu.tsv	Root-Environmental-Aquatic-Freshwater-Lake
ERR1582371	MGYA00352608	https://www.ebi.ac.uk/metagenomics/api/v1/analyses/MGYA00352608/file/ERR1582371_FASTQ_SSU_OTU.tsv	Root-Environmental-Aquatic-Freshwater-Lake
ERR1429254	MGYA00107640	https://www.ebi.ac.uk/metagenomics/api/v1/analyses/MGYA00107640/file/ERR1429254_FASTQ_otu.tsv	Root-Environmental-Aquatic-Freshwater-Lake
ERR1583296	MGYA00353533	https://www.ebi.ac.uk/metagenomics/api/v1/analyses/MGYA00353533/file/ERR1583296_FASTQ_SSU_OTU.tsv	Root-Environmental-Aquatic-Freshwater-Lake
ERR1897478	MGYA00351423	https://www.ebi.ac.uk/metagenomics/api/v1/analyses/MGYA00351423/file/ERR1897478_FASTQ_SSU_OTU.tsv	Root-Environmental-Aquatic-Freshwater-Lake
ERR1583074	MGYA00353311	https://www.ebi.ac.uk/metagenomics/api/v1/analyses/MGYA00353311/file/ERR1583074_FASTQ_SSU_OTU.tsv	Root-Environmental-Aquatic-Freshwater-Lake
ERR1582634	MGYA00352871	https://www.ebi.ac.uk/metagenomics/api/v1/analyses/MGYA00352871/file/ERR1582634_FASTQ_SSU_OTU.tsv	Root-Environmental-Aquatic-Freshwater-Lake
ERR1582193	MGYA00352430	https://www.ebi.ac.uk/metagenomics/api/v1/analyses/MGYA00352430/file/ERR1582193_FASTQ_SSU_OTU.tsv	Root-Environmental-Aquatic-Freshwater-Lake

ERR1897019	MGYA00350964	https://www.ebi.ac.uk/metagenomics/api/v1/analyses/MGYA00350964/file/ERR1897019_FASTQ_SSU_OTU.tsv	Root-Environmental-Aquatic-Freshwater-Lake
ERR1896659	MGYA00350604	https://www.ebi.ac.uk/metagenomics/api/v1/analyses/MGYA00350604/file/ERR1896659_FASTQ_SSU_OTU.tsv	Root-Environmental-Aquatic-Freshwater-Lake
ERR1582255	MGYA00352492	https://www.ebi.ac.uk/metagenomics/api/v1/analyses/MGYA00352492/file/ERR1582255_FASTQ_SSU_OTU.tsv	Root-Environmental-Aquatic-Freshwater-Lake
ERR1582980	MGYA00353217	https://www.ebi.ac.uk/metagenomics/api/v1/analyses/MGYA00353217/file/ERR1582980_FASTQ_SSU_OTU.tsv	Root-Environmental-Aquatic-Freshwater-Lake
ERR430926	MGYA00103262	https://www.ebi.ac.uk/metagenomics/api/v1/analyses/MGYA00103262/file/ERR430926_FASTQ_otu.tsv	Root-Environmental-Aquatic-Freshwater-Lake
ERR1583415	MGYA00353652	https://www.ebi.ac.uk/metagenomics/api/v1/analyses/MGYA00353652/file/ERR1583415_FASTQ_SSU_OTU.tsv	Root-Environmental-Aquatic-Freshwater-Lake
ERR1583363	MGYA00353600	https://www.ebi.ac.uk/metagenomics/api/v1/analyses/MGYA00353600/file/ERR1583363_FASTQ_SSU_OTU.tsv	Root-Environmental-Aquatic-Freshwater-Lake
ERR1582284	MGYA00352521	https://www.ebi.ac.uk/metagenomics/api/v1/analyses/MGYA00352521/file/ERR1582284_FASTQ_SSU_OTU.tsv	Root-Environmental-Aquatic-Freshwater-Lake
ERR1582523	MGYA00352760	https://www.ebi.ac.uk/metagenomics/api/v1/analyses/MGYA00352760/file/ERR1582523_FASTQ_SSU_OTU.tsv	Root-Environmental-Aquatic-Freshwater-Lake
ERR1990498	MGYA00128220	https://www.ebi.ac.uk/metagenomics/api/v1/analyses/MGYA00128220/file/ERR1990498_MERGED_FASTQ_otu.tsv	Root-Environmental-Aquatic-Freshwater-Lake
ERR1582951	MGYA00353188	https://www.ebi.ac.uk/metagenomics/api/v1/analyses/MGYA00353188/file/ERR1582951_FASTQ_SSU_OTU.tsv	Root-Environmental-Aquatic-Freshwater-Lake
ERR1896688	MGYA00350633	https://www.ebi.ac.uk/metagenomics/api/v1/analyses/MGYA00350633/file/ERR1896688_FASTQ_SSU_OTU.tsv	Root-Environmental-Aquatic-Freshwater-Lake

ERR1582142	MGYA00352379	https://www.ebi.ac.uk/metagenomics/api/v1/analyses/MGYA00352379/file/ERR1582142_FASTQ_SSU_OTU.tsv	Root-Environmental-Aquatic-Freshwater-Lake
ERR1897637	MGYA00351582	https://www.ebi.ac.uk/metagenomics/api/v1/analyses/MGYA00351582/file/ERR1897637_FASTQ_SSU_OTU.tsv	Root-Environmental-Aquatic-Freshwater-Lake
ERR1897190	MGYA00351135	https://www.ebi.ac.uk/metagenomics/api/v1/analyses/MGYA00351135/file/ERR1897190_FASTQ_SSU_OTU.tsv	Root-Environmental-Aquatic-Freshwater-Lake
ERR1582809	MGYA00353046	https://www.ebi.ac.uk/metagenomics/api/v1/analyses/MGYA00353046/file/ERR1582809_FASTQ_SSU_OTU.tsv	Root-Environmental-Aquatic-Freshwater-Lake
ERR1897256	MGYA00351201	https://www.ebi.ac.uk/metagenomics/api/v1/analyses/MGYA00351201/file/ERR1897256_FASTQ_SSU_OTU.tsv	Root-Environmental-Aquatic-Freshwater-Lake
ERR1896864	MGYA00350809	https://www.ebi.ac.uk/metagenomics/api/v1/analyses/MGYA00350809/file/ERR1896864_FASTQ_SSU_OTU.tsv	Root-Environmental-Aquatic-Freshwater-Lake
ERR1743282	MGYA00087218	https://www.ebi.ac.uk/metagenomics/api/v1/analyses/MGYA00087218/file/ERR1743282_MERGED_FASTQ_otu.tsv	Root-Environmental-Aquatic-Freshwater-Lake
ERR1554594	MGYA00100722	https://www.ebi.ac.uk/metagenomics/api/v1/analyses/MGYA00100722/file/ERR1554594_MERGED_FASTQ_otu.tsv	Root-Environmental-Aquatic-Freshwater-Lake
ERR1897287	MGYA00351232	https://www.ebi.ac.uk/metagenomics/api/v1/analyses/MGYA00351232/file/ERR1897287_FASTQ_SSU_OTU.tsv	Root-Environmental-Aquatic-Freshwater-Lake
ERR1897520	MGYA00351465	https://www.ebi.ac.uk/metagenomics/api/v1/analyses/MGYA00351465/file/ERR1897520_FASTQ_SSU_OTU.tsv	Root-Environmental-Aquatic-Freshwater-Lake
ERR1429228	MGYA00107637	https://www.ebi.ac.uk/metagenomics/api/v1/analyses/MGYA00107637/file/ERR1429228_FASTQ_otu.tsv	Root-Environmental-Aquatic-Freshwater-Lake
ERR1897141	MGYA00351086	https://www.ebi.ac.uk/metagenomics/api/v1/analyses/MGYA00351086/file/ERR1897141_FASTQ_SSU_OTU.tsv	Root-Environmental-Aquatic-Freshwater-Lake

ERR1896701	MGYA00350646	https://www.ebi.ac.uk/metagenomics/api/v1/analyses/MGYA00350646/file/ERR1896701_FASTQ_SSU_OTU.tsv	Root-Environmental-Aquatic-Freshwater-Lake
ERR1897347	MGYA00351292	https://www.ebi.ac.uk/metagenomics/api/v1/analyses/MGYA00351292/file/ERR1897347_FASTQ_SSU_OTU.tsv	Root-Environmental-Aquatic-Freshwater-Lake
ERR589332	MGYA00103411	https://www.ebi.ac.uk/metagenomics/api/v1/analyses/MGYA00103411/file/ERR589332_FASTQ_otu.tsv	Root-Environmental-Aquatic-Freshwater-Lake
ERR1896975	MGYA00350920	https://www.ebi.ac.uk/metagenomics/api/v1/analyses/MGYA00350920/file/ERR1896975_FASTQ_SSU_OTU.tsv	Root-Environmental-Aquatic-Freshwater-Lake
SRR2442996	MGYA00359000	https://www.ebi.ac.uk/metagenomics/api/v1/analyses/MGYA00359000/file/SRR2442996_MERGED_FASTQ_SSU_OTU.tsv	Root-Environmental-Aquatic-Freshwater-Lake
ERR1743393	MGYA00087111	https://www.ebi.ac.uk/metagenomics/api/v1/analyses/MGYA00087111/file/ERR1743393_MERGED_FASTQ_otu.tsv	Root-Environmental-Aquatic-Freshwater-Lake
ERR1897726	MGYA00351671	https://www.ebi.ac.uk/metagenomics/api/v1/analyses/MGYA00351671/file/ERR1897726_FASTQ_SSU_OTU.tsv	Root-Environmental-Aquatic-Freshwater-Lake
ERR1897081	MGYA00351026	https://www.ebi.ac.uk/metagenomics/api/v1/analyses/MGYA00351026/file/ERR1897081_FASTQ_SSU_OTU.tsv	Root-Environmental-Aquatic-Freshwater-Lake
ERR1582918	MGYA00353155	https://www.ebi.ac.uk/metagenomics/api/v1/analyses/MGYA00353155/file/ERR1582918_FASTQ_SSU_OTU.tsv	Root-Environmental-Aquatic-Freshwater-Lake
ERR1897050	MGYA00350995	https://www.ebi.ac.uk/metagenomics/api/v1/analyses/MGYA00350995/file/ERR1897050_FASTQ_SSU_OTU.tsv	Root-Environmental-Aquatic-Freshwater-Lake
ERR1743342	MGYA00087130	https://www.ebi.ac.uk/metagenomics/api/v1/analyses/MGYA00087130/file/ERR1743342_MERGED_FASTQ_otu.tsv	Root-Environmental-Aquatic-Freshwater-Lake
ERR1897396	MGYA00351341	https://www.ebi.ac.uk/metagenomics/api/v1/analyses/MGYA00351341/file/ERR1897396_FASTQ_SSU_OTU.tsv	Root-Environmental-Aquatic-Freshwater-Lake

ERR1897431	MGYA00351376	https://www.ebi.ac.uk/metagenomics/api/v1/analyses/MGYA00351376/file/ERR1897431_FASTQ_SSU_OTU.tsv	Root-Environmental-Aquatic-Freshwater-Lake
ERR1897108	MGYA00351053	https://www.ebi.ac.uk/metagenomics/api/v1/analyses/MGYA00351053/file/ERR1897108_FASTQ_SSU_OTU.tsv	Root-Environmental-Aquatic-Freshwater-Lake
ERR1896748	MGYA00350693	https://www.ebi.ac.uk/metagenomics/api/v1/analyses/MGYA00350693/file/ERR1896748_FASTQ_SSU_OTU.tsv	Root-Environmental-Aquatic-Freshwater-Lake
ERR1535983	MGYA00320274	https://www.ebi.ac.uk/metagenomics/api/v1/analyses/MGYA00320274/file/ERR1535983_FASTQ_SSU_OTU.tsv	Root-Environmental-Aquatic-Freshwater-Lake
ERR1429261	MGYA00107656	https://www.ebi.ac.uk/metagenomics/api/v1/analyses/MGYA00107656/file/ERR1429261_FASTQ_otu.tsv	Root-Environmental-Aquatic-Freshwater-Lake
ERR1582344	MGYA00352581	https://www.ebi.ac.uk/metagenomics/api/v1/analyses/MGYA00352581/file/ERR1582344_FASTQ_SSU_OTU.tsv	Root-Environmental-Aquatic-Freshwater-Lake
ERR1582891	MGYA00353128	https://www.ebi.ac.uk/metagenomics/api/v1/analyses/MGYA00353128/file/ERR1582891_FASTQ_SSU_OTU.tsv	Root-Environmental-Aquatic-Freshwater-Lake
ERR1583504	MGYA00353741	https://www.ebi.ac.uk/metagenomics/api/v1/analyses/MGYA00353741/file/ERR1583504_FASTQ_SSU_OTU.tsv	Root-Environmental-Aquatic-Freshwater-Lake
ERR1897569	MGYA00351514	https://www.ebi.ac.uk/metagenomics/api/v1/analyses/MGYA00351514/file/ERR1897569_FASTQ_SSU_OTU.tsv	Root-Environmental-Aquatic-Freshwater-Lake
ERR1583165	MGYA00353402	https://www.ebi.ac.uk/metagenomics/api/v1/analyses/MGYA00353402/file/ERR1583165_FASTQ_SSU_OTU.tsv	Root-Environmental-Aquatic-Freshwater-Lake
ERR1582725	MGYA00352962	https://www.ebi.ac.uk/metagenomics/api/v1/analyses/MGYA00352962/file/ERR1582725_FASTQ_SSU_OTU.tsv	Root-Environmental-Aquatic-Freshwater-Lake
ERR1582082	MGYA00352319	https://www.ebi.ac.uk/metagenomics/api/v1/analyses/MGYA00352319/file/ERR1582082_FASTQ_SSU_OTU.tsv	Root-Environmental-Aquatic-Freshwater-Lake

ERR1314333	MGYA00095220	https://www.ebi.ac.uk/metagenomics/api/v1/analyses/MGYA00095220/file/ERR1314333_MERGED_FASTQ_otu.tsv	Root-Environmental-Aquatic-Freshwater-Lake
ERR1582053	MGYA00352290	https://www.ebi.ac.uk/metagenomics/api/v1/analyses/MGYA00352290/file/ERR1582053_FASTQ_SSU_OTU.tsv	Root-Environmental-Aquatic-Freshwater-Lake
ERR1333505	MGYA00107601	https://www.ebi.ac.uk/metagenomics/api/v1/analyses/MGYA00107601/file/ERR1333505_FASTQ_otu.tsv	Root-Environmental-Aquatic-Freshwater-Lake
ERR1583272	MGYA00353509	https://www.ebi.ac.uk/metagenomics/api/v1/analyses/MGYA00353509/file/ERR1583272_FASTQ_SSU_OTU.tsv	Root-Environmental-Aquatic-Freshwater-Lake
ERR1582395	MGYA00352632	https://www.ebi.ac.uk/metagenomics/api/v1/analyses/MGYA00352632/file/ERR1582395_FASTQ_SSU_OTU.tsv	Root-Environmental-Aquatic-Freshwater-Lake
ERR1582840	MGYA00353077	https://www.ebi.ac.uk/metagenomics/api/v1/analyses/MGYA00353077/file/ERR1582840_FASTQ_SSU_OTU.tsv	Root-Environmental-Aquatic-Freshwater-Lake
ERR1582432	MGYA00352669	https://www.ebi.ac.uk/metagenomics/api/v1/analyses/MGYA00352669/file/ERR1582432_FASTQ_SSU_OTU.tsv	Root-Environmental-Aquatic-Freshwater-Lake
ERR1535952	MGYA00320243	https://www.ebi.ac.uk/metagenomics/api/v1/analyses/MGYA00320243/file/ERR1535952_FASTQ_SSU_OTU.tsv	Root-Environmental-Aquatic-Freshwater-Lake
ERR1896799	MGYA00350744	https://www.ebi.ac.uk/metagenomics/api/v1/analyses/MGYA00350744/file/ERR1896799_FASTQ_SSU_OTU.tsv	Root-Environmental-Aquatic-Freshwater-Lake
ERR1582177	MGYA00352414	https://www.ebi.ac.uk/metagenomics/api/v1/analyses/MGYA00352414/file/ERR1582177_FASTQ_SSU_OTU.tsv	Root-Environmental-Aquatic-Freshwater-Lake
ERR1583090	MGYA00353327	https://www.ebi.ac.uk/metagenomics/api/v1/analyses/MGYA00353327/file/ERR1583090_FASTQ_SSU_OTU.tsv	Root-Environmental-Aquatic-Freshwater-Lake
ERR1896909	MGYA00350854	https://www.ebi.ac.uk/metagenomics/api/v1/analyses/MGYA00350854/file/ERR1896909_FASTQ_SSU_OTU.tsv	Root-Environmental-Aquatic-Freshwater-Lake

ERR1583356	MGYA00353593	https://www.ebi.ac.uk/metagenomics/api/v1/analyses/MGYA00353593/file/ERR1583356_FASTQ_SSU_OTU.tsv	Root-Environmental-Aquatic-Freshwater-Lake
ERR1582516	MGYA00352753	https://www.ebi.ac.uk/metagenomics/api/v1/analyses/MGYA00352753/file/ERR1582516_FASTQ_SSU_OTU.tsv	Root-Environmental-Aquatic-Freshwater-Lake
ERR1582964	MGYA00353201	https://www.ebi.ac.uk/metagenomics/api/v1/analyses/MGYA00353201/file/ERR1582964_FASTQ_SSU_OTU.tsv	Root-Environmental-Aquatic-Freshwater-Lake
SRR7081190	MGYA00425954	https://www.ebi.ac.uk/metagenomics/api/v1/analyses/MGYA00425954/file/SRR7081190_MERGED_FASTQ_SSU_OTU.tsv	Root-Environmental-Aquatic-Freshwater-Lake
ERR1582260	MGYA00352497	https://www.ebi.ac.uk/metagenomics/api/v1/analyses/MGYA00352497/file/ERR1582260_FASTQ_SSU_OTU.tsv	Root-Environmental-Aquatic-Freshwater-Lake
ERR1583387	MGYA00353624	https://www.ebi.ac.uk/metagenomics/api/v1/analyses/MGYA00353624/file/ERR1583387_FASTQ_SSU_OTU.tsv	Root-Environmental-Aquatic-Freshwater-Lake
ERR1583420	MGYA00353657	https://www.ebi.ac.uk/metagenomics/api/v1/analyses/MGYA00353657/file/ERR1583420_FASTQ_SSU_OTU.tsv	Root-Environmental-Aquatic-Freshwater-Lake
ERR1547101	MGYA00320812	https://www.ebi.ac.uk/metagenomics/api/v1/analyses/MGYA00320812/file/ERR1547101_FASTQ_SSU_OTU.tsv	Root-Environmental-Aquatic-Freshwater-Lake
SRR7081256	MGYA00425155	https://www.ebi.ac.uk/metagenomics/api/v1/analyses/MGYA00425155/file/SRR7081256_MERGED_FASTQ_SSU_OTU.tsv	Root-Environmental-Aquatic-Freshwater-Lake
ERR1583041	MGYA00353278	https://www.ebi.ac.uk/metagenomics/api/v1/analyses/MGYA00353278/file/ERR1583041_FASTQ_SSU_OTU.tsv	Root-Environmental-Aquatic-Freshwater-Lake
ERR1582601	MGYA00352838	https://www.ebi.ac.uk/metagenomics/api/v1/analyses/MGYA00352838/file/ERR1582601_FASTQ_SSU_OTU.tsv	Root-Environmental-Aquatic-Freshwater-Lake
ERR1582338	MGYA00352575	https://www.ebi.ac.uk/metagenomics/api/v1/analyses/MGYA00352575/file/ERR1582338_FASTQ_SSU_OTU.tsv	Root-Environmental-Aquatic-Freshwater-Lake

ERR1897174	MGYA00351119	https://www.ebi.ac.uk/metagenomics/api/v1/analyses/MGYA00351119/file/ERR1897174_FASTQ_SSU_OTU.tsv	Root-Environmental-Aquatic-Freshwater-Lake
ERR1896734	MGYA00350679	https://www.ebi.ac.uk/metagenomics/api/v1/analyses/MGYA00350679/file/ERR1896734_FASTQ_SSU_OTU.tsv	Root-Environmental-Aquatic-Freshwater-Lake
ERR1583119	MGYA00353356	https://www.ebi.ac.uk/metagenomics/api/v1/analyses/MGYA00353356/file/ERR1583119_FASTQ_SSU_OTU.tsv	Root-Environmental-Aquatic-Freshwater-Lake
ERR1582759	MGYA00352996	https://www.ebi.ac.uk/metagenomics/api/v1/analyses/MGYA00352996/file/ERR1582759_FASTQ_SSU_OTU.tsv	Root-Environmental-Aquatic-Freshwater-Lake
ERR658207	MGYA00103492	https://www.ebi.ac.uk/metagenomics/api/v1/analyses/MGYA00103492/file/ERR658207_FASTQ_otu.tsv	Root-Environmental-Aquatic-Freshwater-Lake
ERR1547059	MGYA00320770	https://www.ebi.ac.uk/metagenomics/api/v1/analyses/MGYA00320770/file/ERR1547059_FASTQ_SSU_OTU.tsv	Root-Environmental-Aquatic-Freshwater-Lake
ERR1896880	MGYA00350825	https://www.ebi.ac.uk/metagenomics/api/v1/analyses/MGYA00350825/file/ERR1896880_FASTQ_SSU_OTU.tsv	Root-Environmental-Aquatic-Freshwater-Lake
ERR1897515	MGYA00351460	https://www.ebi.ac.uk/metagenomics/api/v1/analyses/MGYA00351460/file/ERR1897515_FASTQ_SSU_OTU.tsv	Root-Environmental-Aquatic-Freshwater-Lake
ERR1897263	MGYA00351208	https://www.ebi.ac.uk/metagenomics/api/v1/analyses/MGYA00351208/file/ERR1897263_FASTQ_SSU_OTU.tsv	Root-Environmental-Aquatic-Freshwater-Lake
ERR1547088	MGYA00320799	https://www.ebi.ac.uk/metagenomics/api/v1/analyses/MGYA00320799/file/ERR1547088_FASTQ_SSU_OTU.tsv	Root-Environmental-Aquatic-Freshwater-Lake
ERR1896851	MGYA00350796	https://www.ebi.ac.uk/metagenomics/api/v1/analyses/MGYA00350796/file/ERR1896851_FASTQ_SSU_OTU.tsv	Root-Environmental-Aquatic-Freshwater-Lake
ERR1582788	MGYA00353025	https://www.ebi.ac.uk/metagenomics/api/v1/analyses/MGYA00353025/file/ERR1582788_FASTQ_SSU_OTU.tsv	Root-Environmental-Aquatic-Freshwater-Lake

ERR1897602	MGYA00351547	https://www.ebi.ac.uk/metagenomics/api/v1/analyses/MGYA00351547/file/ERR1897602_FASTQ_SSU_OTU.tsv	Root-Environmental-Aquatic-Freshwater-Lake
ERR1583097	MGYA00353334	https://www.ebi.ac.uk/metagenomics/api/v1/analyses/MGYA00353334/file/ERR1583097_FASTQ_SSU_OTU.tsv	Root-Environmental-Aquatic-Freshwater-Lake
ERR1582170	MGYA00352407	https://www.ebi.ac.uk/metagenomics/api/v1/analyses/MGYA00352407/file/ERR1582170_FASTQ_SSU_OTU.tsv	Root-Environmental-Aquatic-Freshwater-Lake
ERR1582963	MGYA00353200	https://www.ebi.ac.uk/metagenomics/api/v1/analyses/MGYA00353200/file/ERR1582963_FASTQ_SSU_OTU.tsv	Root-Environmental-Aquatic-Freshwater-Lake
ERR1582511	MGYA00352748	https://www.ebi.ac.uk/metagenomics/api/v1/analyses/MGYA00352748/file/ERR1582511_FASTQ_SSU_OTU.tsv	Root-Environmental-Aquatic-Freshwater-Lake
ERR1583351	MGYA00353588	https://www.ebi.ac.uk/metagenomics/api/v1/analyses/MGYA00353588/file/ERR1583351_FASTQ_SSU_OTU.tsv	Root-Environmental-Aquatic-Freshwater-Lake
SRR2443019	MGYA00359023	https://www.ebi.ac.uk/metagenomics/api/v1/analyses/MGYA00359023/file/SRR2443019_MERGED_FASTQ_SSU_OTU.tsv	Root-Environmental-Aquatic-Freshwater-Lake
ERR1583427	MGYA00353664	https://www.ebi.ac.uk/metagenomics/api/v1/analyses/MGYA00353664/file/ERR1583427_FASTQ_SSU_OTU.tsv	Root-Environmental-Aquatic-Freshwater-Lake
ERR1583380	MGYA00353617	https://www.ebi.ac.uk/metagenomics/api/v1/analyses/MGYA00353617/file/ERR1583380_FASTQ_SSU_OTU.tsv	Root-Environmental-Aquatic-Freshwater-Lake
ERR1582267	MGYA00352504	https://www.ebi.ac.uk/metagenomics/api/v1/analyses/MGYA00352504/file/ERR1582267_FASTQ_SSU_OTU.tsv	Root-Environmental-Aquatic-Freshwater-Lake
ERR1743339	MGYA00087190	https://www.ebi.ac.uk/metagenomics/api/v1/analyses/MGYA00087190/file/ERR1743339_MERGED_FASTQ_otu.tsv	Root-Environmental-Aquatic-Freshwater-Lake
ERR1547106	MGYA00320817	https://www.ebi.ac.uk/metagenomics/api/v1/analyses/MGYA00320817/file/ERR1547106_FASTQ_SSU_OTU.tsv	Root-Environmental-Aquatic-Freshwater-Lake

ERR1582606	MGYA00352843	https://www.ebi.ac.uk/metagenomics/api/v1/analyses/MGYA00352843/file/ERR1582606_FASTQ_SSU_OTU.tsv	Root-Environmental-Aquatic-Freshwater-Lake
ERR1583046	MGYA00353283	https://www.ebi.ac.uk/metagenomics/api/v1/analyses/MGYA00353283/file/ERR1583046_FASTQ_SSU_OTU.tsv	Root-Environmental-Aquatic-Freshwater-Lake
ERR1582498	MGYA00352735	https://www.ebi.ac.uk/metagenomics/api/v1/analyses/MGYA00352735/file/ERR1582498_FASTQ_SSU_OTU.tsv	Root-Environmental-Aquatic-Freshwater-Lake
ERR1896733	MGYA00350678	https://www.ebi.ac.uk/metagenomics/api/v1/analyses/MGYA00350678/file/ERR1896733_FASTQ_SSU_OTU.tsv	Root-Environmental-Aquatic-Freshwater-Lake
ERR1897173	MGYA00351118	https://www.ebi.ac.uk/metagenomics/api/v1/analyses/MGYA00351118/file/ERR1897173_FASTQ_SSU_OTU.tsv	Root-Environmental-Aquatic-Freshwater-Lake
ERR1314399	MGYA00095198	https://www.ebi.ac.uk/metagenomics/api/v1/analyses/MGYA00095198/file/ERR1314399_MERGED_FASTQ_otu.tsv	Root-Environmental-Aquatic-Freshwater-Lake
ERR658200	MGYA00103474	https://www.ebi.ac.uk/metagenomics/api/v1/analyses/MGYA00103474/file/ERR658200_FASTQ_otu.tsv	Root-Environmental-Aquatic-Freshwater-Lake
SRR7081309	MGYA00413188	https://www.ebi.ac.uk/metagenomics/api/v1/analyses/MGYA00413188/file/SRR7081309_MERGED_FASTQ_SSU_OTU.tsv	Root-Environmental-Aquatic-Freshwater-Lake
ERR1897512	MGYA00351457	https://www.ebi.ac.uk/metagenomics/api/v1/analyses/MGYA00351457/file/ERR1897512_FASTQ_SSU_OTU.tsv	Root-Environmental-Aquatic-Freshwater-Lake
ERR1896887	MGYA00350832	https://www.ebi.ac.uk/metagenomics/api/v1/analyses/MGYA00350832/file/ERR1896887_FASTQ_SSU_OTU.tsv	Root-Environmental-Aquatic-Freshwater-Lake
ERR1896856	MGYA00350801	https://www.ebi.ac.uk/metagenomics/api/v1/analyses/MGYA00350801/file/ERR1896856_FASTQ_SSU_OTU.tsv	Root-Environmental-Aquatic-Freshwater-Lake
ERR1897264	MGYA00351209	https://www.ebi.ac.uk/metagenomics/api/v1/analyses/MGYA00351209/file/ERR1897264_FASTQ_SSU_OTU.tsv	Root-Environmental-Aquatic-Freshwater-Lake

ERR1314348	MGYA00095195	https://www.ebi.ac.uk/metagenomics/api/v1/analyses/MGYA00095195/file/ERR1314348_MERGED_FASTQ_otu.tsv	Root-Environmental-Aquatic-Freshwater-Lake
ERR1582028	MGYA00352265	https://www.ebi.ac.uk/metagenomics/api/v1/analyses/MGYA00352265/file/ERR1582028_FASTQ_SSU_OTU.tsv	Root-Environmental-Aquatic-Freshwater-Lake
ERR1897605	MGYA00351550	https://www.ebi.ac.uk/metagenomics/api/v1/analyses/MGYA00351550/file/ERR1897605_FASTQ_SSU_OTU.tsv	Root-Environmental-Aquatic-Freshwater-Lake
ERR1582449	MGYA00352686	https://www.ebi.ac.uk/metagenomics/api/v1/analyses/MGYA00352686/file/ERR1582449_FASTQ_SSU_OTU.tsv	Root-Environmental-Aquatic-Freshwater-Lake
ERR1583209	MGYA00353446	https://www.ebi.ac.uk/metagenomics/api/v1/analyses/MGYA00353446/file/ERR1583209_FASTQ_SSU_OTU.tsv	Root-Environmental-Aquatic-Freshwater-Lake
SRR2442991	MGYA00358995	https://www.ebi.ac.uk/metagenomics/api/v1/analyses/MGYA00358995/file/SRR2442991_MERGED_FASTQ_SSU_OTU.tsv	Root-Environmental-Aquatic-Freshwater-Lake
ERR1896972	MGYA00350917	https://www.ebi.ac.uk/metagenomics/api/v1/analyses/MGYA00350917/file/ERR1896972_FASTQ_SSU_OTU.tsv	Root-Environmental-Aquatic-Freshwater-Lake
ERR589335	MGYA00103449	https://www.ebi.ac.uk/metagenomics/api/v1/analyses/MGYA00103449/file/ERR589335_FASTQ_otu.tsv	Root-Environmental-Aquatic-Freshwater-Lake
ERR1897340	MGYA00351285	https://www.ebi.ac.uk/metagenomics/api/v1/analyses/MGYA00351285/file/ERR1897340_FASTQ_SSU_OTU.tsv	Root-Environmental-Aquatic-Freshwater-Lake
ERR1897086	MGYA00351031	https://www.ebi.ac.uk/metagenomics/api/v1/analyses/MGYA00351031/file/ERR1897086_FASTQ_SSU_OTU.tsv	Root-Environmental-Aquatic-Freshwater-Lake
ERR1897721	MGYA00351666	https://www.ebi.ac.uk/metagenomics/api/v1/analyses/MGYA00351666/file/ERR1897721_FASTQ_SSU_OTU.tsv	Root-Environmental-Aquatic-Freshwater-Lake
ERR1897057	MGYA00351002	https://www.ebi.ac.uk/metagenomics/api/v1/analyses/MGYA00351002/file/ERR1897057_FASTQ_SSU_OTU.tsv	Root-Environmental-Aquatic-Freshwater-Lake

ERR1897436	MGYA00351381	https://www.ebi.ac.uk/metagenomics/api/v1/analyses/MGYA00351381/file/ERR1897436_FASTQ_SSU_OTU.tsv	Root-Environmental-Aquatic-Freshwater-Lake
ERR1897391	MGYA00351336	https://www.ebi.ac.uk/metagenomics/api/v1/analyses/MGYA00351336/file/ERR1897391_FASTQ_SSU_OTU.tsv	Root-Environmental-Aquatic-Freshwater-Lake
ERR1743345	MGYA00087212	https://www.ebi.ac.uk/metagenomics/api/v1/analyses/MGYA00087212/file/ERR1743345_MERGED_FASTQ_otu.tsv	Root-Environmental-Aquatic-Freshwater-Lake
ERR1535984	MGYA00320275	https://www.ebi.ac.uk/metagenomics/api/v1/analyses/MGYA00320275/file/ERR1535984_FASTQ_SSU_OTU.tsv	Root-Environmental-Aquatic-Freshwater-Lake
ERR1583503	MGYA00353740	https://www.ebi.ac.uk/metagenomics/api/v1/analyses/MGYA00353740/file/ERR1583503_FASTQ_SSU_OTU.tsv	Root-Environmental-Aquatic-Freshwater-Lake
ERR1582896	MGYA00353133	https://www.ebi.ac.uk/metagenomics/api/v1/analyses/MGYA00353133/file/ERR1582896_FASTQ_SSU_OTU.tsv	Root-Environmental-Aquatic-Freshwater-Lake
ERR1582343	MGYA00352580	https://www.ebi.ac.uk/metagenomics/api/v1/analyses/MGYA00352580/file/ERR1582343_FASTQ_SSU_OTU.tsv	Root-Environmental-Aquatic-Freshwater-Lake
ERR1429266	MGYA00107671	https://www.ebi.ac.uk/metagenomics/api/v1/analyses/MGYA00107671/file/ERR1429266_FASTQ_otu.tsv	Root-Environmental-Aquatic-Freshwater-Lake
SRR7081375	MGYA00423203	https://www.ebi.ac.uk/metagenomics/api/v1/analyses/MGYA00423203/file/SRR7081375_MERGED_FASTQ_SSU_OTU.tsv	Root-Environmental-Aquatic-Freshwater-Lake
ERR1582085	MGYA00352322	https://www.ebi.ac.uk/metagenomics/api/v1/analyses/MGYA00352322/file/ERR1582085_FASTQ_SSU_OTU.tsv	Root-Environmental-Aquatic-Freshwater-Lake
ERR1582722	MGYA00352959	https://www.ebi.ac.uk/metagenomics/api/v1/analyses/MGYA00352959/file/ERR1582722_FASTQ_SSU_OTU.tsv	Root-Environmental-Aquatic-Freshwater-Lake
ERR1583162	MGYA00353399	https://www.ebi.ac.uk/metagenomics/api/v1/analyses/MGYA00353399/file/ERR1583162_FASTQ_SSU_OTU.tsv	Root-Environmental-Aquatic-Freshwater-Lake

ERR1582054	MGYA00352291	https://www.ebi.ac.uk/metagenomics/api/v1/analyses/MGYA00352291/file/ERR1582054_FASTQ_SSU_OTU.tsv	Root-Environmental-Aquatic-Freshwater-Lake
ERR1314334	MGYA00095216	https://www.ebi.ac.uk/metagenomics/api/v1/analyses/MGYA00095216/file/ERR1314334_MERGED_FASTQ_otu.tsv	Root-Environmental-Aquatic-Freshwater-Lake
ERR1897218	MGYA00351163	https://www.ebi.ac.uk/metagenomics/api/v1/analyses/MGYA00351163/file/ERR1897218_FASTQ_SSU_OTU.tsv	Root-Environmental-Aquatic-Freshwater-Lake
ERR1582435	MGYA00352672	https://www.ebi.ac.uk/metagenomics/api/v1/analyses/MGYA00352672/file/ERR1582435_FASTQ_SSU_OTU.tsv	Root-Environmental-Aquatic-Freshwater-Lake
ERR1582847	MGYA00353084	https://www.ebi.ac.uk/metagenomics/api/v1/analyses/MGYA00353084/file/ERR1582847_FASTQ_SSU_OTU.tsv	Root-Environmental-Aquatic-Freshwater-Lake
ERR1582392	MGYA00352629	https://www.ebi.ac.uk/metagenomics/api/v1/analyses/MGYA00352629/file/ERR1582392_FASTQ_SSU_OTU.tsv	Root-Environmental-Aquatic-Freshwater-Lake
ERR1583275	MGYA00353512	https://www.ebi.ac.uk/metagenomics/api/v1/analyses/MGYA00353512/file/ERR1583275_FASTQ_SSU_OTU.tsv	Root-Environmental-Aquatic-Freshwater-Lake
ERR1333502	MGYA00107593	https://www.ebi.ac.uk/metagenomics/api/v1/analyses/MGYA00107593/file/ERR1333502_FASTQ_otu.tsv	Root-Environmental-Aquatic-Freshwater-Lake
ERR1897679	MGYA00351624	https://www.ebi.ac.uk/metagenomics/api/v1/analyses/MGYA00351624/file/ERR1897679_FASTQ_SSU_OTU.tsv	Root-Environmental-Aquatic-Freshwater-Lake
ERR1535955	MGYA00320246	https://www.ebi.ac.uk/metagenomics/api/v1/analyses/MGYA00320246/file/ERR1535955_FASTQ_SSU_OTU.tsv	Root-Environmental-Aquatic-Freshwater-Lake
ERR1897332	MGYA00351277	https://www.ebi.ac.uk/metagenomics/api/v1/analyses/MGYA00351277/file/ERR1897332_FASTQ_SSU_OTU.tsv	Root-Environmental-Aquatic-Freshwater-Lake
ERR1897495	MGYA00351440	https://www.ebi.ac.uk/metagenomics/api/v1/analyses/MGYA00351440/file/ERR1897495_FASTQ_SSU_OTU.tsv	Root-Environmental-Aquatic-Freshwater-Lake

ERR1896900	MGYA00350845	https://www.ebi.ac.uk/metagenomics/api/v1/analyses/MGYA00350845/file/ERR1896900_FASTQ_SSU_OTU.tsv	Root-Environmental-Aquatic-Freshwater-Lake
ERR1583099	MGYA00353336	https://www.ebi.ac.uk/metagenomics/api/v1/analyses/MGYA00353336/file/ERR1583099_FASTQ_SSU_OTU.tsv	Root-Environmental-Aquatic-Freshwater-Lake
SRR2443017	MGYA00359021	https://www.ebi.ac.uk/metagenomics/api/v1/analyses/MGYA00359021/file/SRR2443017_MERGED_FASTQ_SSU_OTU.tsv	Root-Environmental-Aquatic-Freshwater-Lake
ERR1897753	MGYA00351698	https://www.ebi.ac.uk/metagenomics/api/v1/analyses/MGYA00351698/file/ERR1897753_FASTQ_SSU_OTU.tsv	Root-Environmental-Aquatic-Freshwater-Lake
ERR1582269	MGYA00352506	https://www.ebi.ac.uk/metagenomics/api/v1/analyses/MGYA00352506/file/ERR1582269_FASTQ_SSU_OTU.tsv	Root-Environmental-Aquatic-Freshwater-Lake
ERR1583429	MGYA00353666	https://www.ebi.ac.uk/metagenomics/api/v1/analyses/MGYA00353666/file/ERR1583429_FASTQ_SSU_OTU.tsv	Root-Environmental-Aquatic-Freshwater-Lake
SRR7081199	MGYA00426725	https://www.ebi.ac.uk/metagenomics/api/v1/analyses/MGYA00426725/file/SRR7081199_MERGED_FASTQ_SSU_OTU.tsv	Root-Environmental-Aquatic-Freshwater-Lake
ERR1897025	MGYA00350970	https://www.ebi.ac.uk/metagenomics/api/v1/analyses/MGYA00350970/file/ERR1897025_FASTQ_SSU_OTU.tsv	Root-Environmental-Aquatic-Freshwater-Lake
ERR1896665	MGYA00350610	https://www.ebi.ac.uk/metagenomics/api/v1/analyses/MGYA00350610/file/ERR1896665_FASTQ_SSU_OTU.tsv	Root-Environmental-Aquatic-Freshwater-Lake
ERR1583048	MGYA00353285	https://www.ebi.ac.uk/metagenomics/api/v1/analyses/MGYA00353285/file/ERR1583048_FASTQ_SSU_OTU.tsv	Root-Environmental-Aquatic-Freshwater-Lake
ERR1582608	MGYA00352845	https://www.ebi.ac.uk/metagenomics/api/v1/analyses/MGYA00352845/file/ERR1582608_FASTQ_SSU_OTU.tsv	Root-Environmental-Aquatic-Freshwater-Lake
ERR1743337	MGYA00087155	https://www.ebi.ac.uk/metagenomics/api/v1/analyses/MGYA00087155/file/ERR1743337_MERGED_FASTQ_otu.tsv	Root-Environmental-Aquatic-Freshwater-Lake

ERR1547108	MGYA00320819	https://www.ebi.ac.uk/metagenomics/api/v1/analyses/MGYA00320819/file/ERR1547108_FASTQ_SSU_OTU.tsv	Root-Environmental-Aquatic-Freshwater-Lake
ERR1897444	MGYA00351389	https://www.ebi.ac.uk/metagenomics/api/v1/analyses/MGYA00351389/file/ERR1897444_FASTQ_SSU_OTU.tsv	Root-Environmental-Aquatic-Freshwater-Lake
ERR1582331	MGYA00352568	https://www.ebi.ac.uk/metagenomics/api/v1/analyses/MGYA00352568/file/ERR1582331_FASTQ_SSU_OTU.tsv	Root-Environmental-Aquatic-Freshwater-Lake
ERR1582496	MGYA00352733	https://www.ebi.ac.uk/metagenomics/api/v1/analyses/MGYA00352733/file/ERR1582496_FASTQ_SSU_OTU.tsv	Root-Environmental-Aquatic-Freshwater-Lake
ERR1896889	MGYA00350834	https://www.ebi.ac.uk/metagenomics/api/v1/analyses/MGYA00350834/file/ERR1896889_FASTQ_SSU_OTU.tsv	Root-Environmental-Aquatic-Freshwater-Lake
ERR1547050	MGYA00320761	https://www.ebi.ac.uk/metagenomics/api/v1/analyses/MGYA00320761/file/ERR1547050_FASTQ_SSU_OTU.tsv	Root-Environmental-Aquatic-Freshwater-Lake
ERR1583110	MGYA00353347	https://www.ebi.ac.uk/metagenomics/api/v1/analyses/MGYA00353347/file/ERR1583110_FASTQ_SSU_OTU.tsv	Root-Environmental-Aquatic-Freshwater-Lake
ERR1582750	MGYA00352987	https://www.ebi.ac.uk/metagenomics/api/v1/analyses/MGYA00352987/file/ERR1582750_FASTQ_SSU_OTU.tsv	Root-Environmental-Aquatic-Freshwater-Lake
ERR1314397	MGYA00095156	https://www.ebi.ac.uk/metagenomics/api/v1/analyses/MGYA00095156/file/ERR1314397_MERGED_FASTQ_otu.tsv	Root-Environmental-Aquatic-Freshwater-Lake
ERR1314346	MGYA00095167	https://www.ebi.ac.uk/metagenomics/api/v1/analyses/MGYA00095167/file/ERR1314346_MERGED_FASTQ_otu.tsv	Root-Environmental-Aquatic-Freshwater-Lake
ERR1582781	MGYA00353018	https://www.ebi.ac.uk/metagenomics/api/v1/analyses/MGYA00353018/file/ERR1582781_FASTQ_SSU_OTU.tsv	Root-Environmental-Aquatic-Freshwater-Lake
ERR1582026	MGYA00352263	https://www.ebi.ac.uk/metagenomics/api/v1/analyses/MGYA00352263/file/ERR1582026_FASTQ_SSU_OTU.tsv	Root-Environmental-Aquatic-Freshwater-Lake

ERR1896858	MGYA00350803	https://www.ebi.ac.uk/metagenomics/api/v1/analyses/MGYA00350803/file/ERR1896858_FASTQ_SSU_OTU.tsv	Root-Environmental-Aquatic-Freshwater-Lake
ERR1547081	MGYA00320792	https://www.ebi.ac.uk/metagenomics/api/v1/analyses/MGYA00320792/file/ERR1547081_FASTQ_SSU_OTU.tsv	Root-Environmental-Aquatic-Freshwater-Lake
ERR1583207	MGYA00353444	https://www.ebi.ac.uk/metagenomics/api/v1/analyses/MGYA00353444/file/ERR1583207_FASTQ_SSU_OTU.tsv	Root-Environmental-Aquatic-Freshwater-Lake
ERR1582447	MGYA00352684	https://www.ebi.ac.uk/metagenomics/api/v1/analyses/MGYA00352684/file/ERR1582447_FASTQ_SSU_OTU.tsv	Root-Environmental-Aquatic-Freshwater-Lake
ERR1582835	MGYA00353072	https://www.ebi.ac.uk/metagenomics/api/v1/analyses/MGYA00353072/file/ERR1582835_FASTQ_SSU_OTU.tsv	Root-Environmental-Aquatic-Freshwater-Lake
ERR1582102	MGYA00352339	https://www.ebi.ac.uk/metagenomics/api/v1/analyses/MGYA00352339/file/ERR1582102_FASTQ_SSU_OTU.tsv	Root-Environmental-Aquatic-Freshwater-Lake
ERR1583323	MGYA00353560	https://www.ebi.ac.uk/metagenomics/api/v1/analyses/MGYA00353560/file/ERR1583323_FASTQ_SSU_OTU.tsv	Root-Environmental-Aquatic-Freshwater-Lake
ERR1583484	MGYA00353721	https://www.ebi.ac.uk/metagenomics/api/v1/analyses/MGYA00353721/file/ERR1583484_FASTQ_SSU_OTU.tsv	Root-Environmental-Aquatic-Freshwater-Lake
ERR1582911	MGYA00353148	https://www.ebi.ac.uk/metagenomics/api/v1/analyses/MGYA00353148/file/ERR1582911_FASTQ_SSU_OTU.tsv	Root-Environmental-Aquatic-Freshwater-Lake
ERR1582563	MGYA00352800	https://www.ebi.ac.uk/metagenomics/api/v1/analyses/MGYA00352800/file/ERR1582563_FASTQ_SSU_OTU.tsv	Root-Environmental-Aquatic-Freshwater-Lake
ERR1897088	MGYA00351033	https://www.ebi.ac.uk/metagenomics/api/v1/analyses/MGYA00351033/file/ERR1897088_FASTQ_SSU_OTU.tsv	Root-Environmental-Aquatic-Freshwater-Lake
ERR1897059	MGYA00351004	https://www.ebi.ac.uk/metagenomics/api/v1/analyses/MGYA00351004/file/ERR1897059_FASTQ_SSU_OTU.tsv	Root-Environmental-Aquatic-Freshwater-Lake

ERR1582215	MGYA00352452	https://www.ebi.ac.uk/metagenomics/api/v1/analyses/MGYA00352452/file/ERR1582215_FASTQ_SSU_OTU.tsv	Root-Environmental-Aquatic-Freshwater-Lake
ERR1583455	MGYA00353692	https://www.ebi.ac.uk/metagenomics/api/v1/analyses/MGYA00353692/file/ERR1583455_FASTQ_SSU_OTU.tsv	Root-Environmental-Aquatic-Freshwater-Lake
ERR1897438	MGYA00351383	https://www.ebi.ac.uk/metagenomics/api/v1/analyses/MGYA00351383/file/ERR1897438_FASTQ_SSU_OTU.tsv	Root-Environmental-Aquatic-Freshwater-Lake
ERR1583034	MGYA00353271	https://www.ebi.ac.uk/metagenomics/api/v1/analyses/MGYA00353271/file/ERR1583034_FASTQ_SSU_OTU.tsv	Root-Environmental-Aquatic-Freshwater-Lake
ERR1582674	MGYA00352911	https://www.ebi.ac.uk/metagenomics/api/v1/analyses/MGYA00352911/file/ERR1582674_FASTQ_SSU_OTU.tsv	Root-Environmental-Aquatic-Freshwater-Lake
ERR1582898	MGYA00353135	https://www.ebi.ac.uk/metagenomics/api/v1/analyses/MGYA00353135/file/ERR1582898_FASTQ_SSU_OTU.tsv	Root-Environmental-Aquatic-Freshwater-Lake
ERR1429268	MGYA00107650	https://www.ebi.ac.uk/metagenomics/api/v1/analyses/MGYA00107650/file/ERR1429268_FASTQ_otu.tsv	Root-Environmental-Aquatic-Freshwater-Lake
ERR1897101	MGYA00351046	https://www.ebi.ac.uk/metagenomics/api/v1/analyses/MGYA00351046/file/ERR1897101_FASTQ_SSU_OTU.tsv	Root-Environmental-Aquatic-Freshwater-Lake
ERR1896741	MGYA00350686	https://www.ebi.ac.uk/metagenomics/api/v1/analyses/MGYA00350686/file/ERR1896741_FASTQ_SSU_OTU.tsv	Root-Environmental-Aquatic-Freshwater-Lake
ERR1897560	MGYA00351505	https://www.ebi.ac.uk/metagenomics/api/v1/analyses/MGYA00351505/file/ERR1897560_FASTQ_SSU_OTU.tsv	Root-Environmental-Aquatic-Freshwater-Lake
ERR1897216	MGYA00351161	https://www.ebi.ac.uk/metagenomics/api/v1/analyses/MGYA00351161/file/ERR1897216_FASTQ_SSU_OTU.tsv	Root-Environmental-Aquatic-Freshwater-Lake
ERR1896824	MGYA00350769	https://www.ebi.ac.uk/metagenomics/api/v1/analyses/MGYA00350769/file/ERR1896824_FASTQ_SSU_OTU.tsv	Root-Environmental-Aquatic-Freshwater-Lake

ERR1896790	MGYA00350735	https://www.ebi.ac.uk/metagenomics/api/v1/analyses/MGYA00350735/file/ERR1896790_FASTQ_SSU_OTU.tsv	Root-Environmental-Aquatic-Freshwater-Lake
ERR1897677	MGYA00351622	https://www.ebi.ac.uk/metagenomics/api/v1/analyses/MGYA00351622/file/ERR1897677_FASTQ_SSU_OTU.tsv	Root-Environmental-Aquatic-Freshwater-Lake
ERR1582849	MGYA00353086	https://www.ebi.ac.uk/metagenomics/api/v1/analyses/MGYA00353086/file/ERR1582849_FASTQ_SSU_OTU.tsv	Root-Environmental-Aquatic-Freshwater-Lake
ERR1582105	MGYA00352342	https://www.ebi.ac.uk/metagenomics/api/v1/analyses/MGYA00352342/file/ERR1582105_FASTQ_SSU_OTU.tsv	Root-Environmental-Aquatic-Freshwater-Lake
SRR2442998	MGYA00359002	https://www.ebi.ac.uk/metagenomics/api/v1/analyses/MGYA00359002/file/SRR2442998_MERGED_FASTQ_SSU_OTU.tsv	Root-Environmental-Aquatic-Freshwater-Lake
ERR1897349	MGYA00351294	https://www.ebi.ac.uk/metagenomics/api/v1/analyses/MGYA00351294/file/ERR1897349_FASTQ_SSU_OTU.tsv	Root-Environmental-Aquatic-Freshwater-Lake
ERR1582564	MGYA00352801	https://www.ebi.ac.uk/metagenomics/api/v1/analyses/MGYA00352801/file/ERR1582564_FASTQ_SSU_OTU.tsv	Root-Environmental-Aquatic-Freshwater-Lake
ERR1582916	MGYA00353153	https://www.ebi.ac.uk/metagenomics/api/v1/analyses/MGYA00353153/file/ERR1582916_FASTQ_SSU_OTU.tsv	Root-Environmental-Aquatic-Freshwater-Lake
ERR1583483	MGYA00353720	https://www.ebi.ac.uk/metagenomics/api/v1/analyses/MGYA00353720/file/ERR1583483_FASTQ_SSU_OTU.tsv	Root-Environmental-Aquatic-Freshwater-Lake
ERR1583324	MGYA00353561	https://www.ebi.ac.uk/metagenomics/api/v1/analyses/MGYA00353561/file/ERR1583324_FASTQ_SSU_OTU.tsv	Root-Environmental-Aquatic-Freshwater-Lake
ERR1897728	MGYA00351673	https://www.ebi.ac.uk/metagenomics/api/v1/analyses/MGYA00351673/file/ERR1897728_FASTQ_SSU_OTU.tsv	Root-Environmental-Aquatic-Freshwater-Lake
ERR1583452	MGYA00353689	https://www.ebi.ac.uk/metagenomics/api/v1/analyses/MGYA00353689/file/ERR1583452_FASTQ_SSU_OTU.tsv	Root-Environmental-Aquatic-Freshwater-Lake

ERR1582212	MGYA00352449	https://www.ebi.ac.uk/metagenomics/api/v1/analyses/MGYA00352449/file/ERR1582212_FASTQ_SSU_OTU.tsv	Root-Environmental-Aquatic-Freshwater-Lake
ERR1897398	MGYA00351343	https://www.ebi.ac.uk/metagenomics/api/v1/analyses/MGYA00351343/file/ERR1897398_FASTQ_SSU_OTU.tsv	Root-Environmental-Aquatic-Freshwater-Lake
ERR1582673	MGYA00352910	https://www.ebi.ac.uk/metagenomics/api/v1/analyses/MGYA00352910/file/ERR1582673_FASTQ_SSU_OTU.tsv	Root-Environmental-Aquatic-Freshwater-Lake
ERR1583033	MGYA00353270	https://www.ebi.ac.uk/metagenomics/api/v1/analyses/MGYA00353270/file/ERR1583033_FASTQ_SSU_OTU.tsv	Root-Environmental-Aquatic-Freshwater-Lake
ERR1896746	MGYA00350691	https://www.ebi.ac.uk/metagenomics/api/v1/analyses/MGYA00350691/file/ERR1896746_FASTQ_SSU_OTU.tsv	Root-Environmental-Aquatic-Freshwater-Lake
ERR1897106	MGYA00351051	https://www.ebi.ac.uk/metagenomics/api/v1/analyses/MGYA00351051/file/ERR1897106_FASTQ_SSU_OTU.tsv	Root-Environmental-Aquatic-Freshwater-Lake
ERR1897567	MGYA00351512	https://www.ebi.ac.uk/metagenomics/api/v1/analyses/MGYA00351512/file/ERR1897567_FASTQ_SSU_OTU.tsv	Root-Environmental-Aquatic-Freshwater-Lake
ERR1896823	MGYA00350768	https://www.ebi.ac.uk/metagenomics/api/v1/analyses/MGYA00350768/file/ERR1896823_FASTQ_SSU_OTU.tsv	Root-Environmental-Aquatic-Freshwater-Lake
ERR1897211	MGYA00351156	https://www.ebi.ac.uk/metagenomics/api/v1/analyses/MGYA00351156/file/ERR1897211_FASTQ_SSU_OTU.tsv	Root-Environmental-Aquatic-Freshwater-Lake
ERR1897670	MGYA00351615	https://www.ebi.ac.uk/metagenomics/api/v1/analyses/MGYA00351615/file/ERR1897670_FASTQ_SSU_OTU.tsv	Root-Environmental-Aquatic-Freshwater-Lake
ERR1896797	MGYA00350742	https://www.ebi.ac.uk/metagenomics/api/v1/analyses/MGYA00350742/file/ERR1896797_FASTQ_SSU_OTU.tsv	Root-Environmental-Aquatic-Freshwater-Lake
ERR1896907	MGYA00350852	https://www.ebi.ac.uk/metagenomics/api/v1/analyses/MGYA00350852/file/ERR1896907_FASTQ_SSU_OTU.tsv	Root-Environmental-Aquatic-Freshwater-Lake

ERR589340	MGYA00103453	https://www.ebi.ac.uk/metagenomics/api/v1/analyses/MGYA00103453/file/ERR589340_FASTQ_otu.tsv	Root-Environmental-Aquatic-Freshwater-Lake
ERR1897492	MGYA00351437	https://www.ebi.ac.uk/metagenomics/api/v1/analyses/MGYA00351437/file/ERR1897492_FASTQ_SSU_OTU.tsv	Root-Environmental-Aquatic-Freshwater-Lake
ERR1897335	MGYA00351280	https://www.ebi.ac.uk/metagenomics/api/v1/analyses/MGYA00351280/file/ERR1897335_FASTQ_SSU_OTU.tsv	Root-Environmental-Aquatic-Freshwater-Lake
ERR1582179	MGYA00352416	https://www.ebi.ac.uk/metagenomics/api/v1/analyses/MGYA00352416/file/ERR1582179_FASTQ_SSU_OTU.tsv	Root-Environmental-Aquatic-Freshwater-Lake
SRR2443010	MGYA00359014	https://www.ebi.ac.uk/metagenomics/api/v1/analyses/MGYA00359014/file/SRR2443010_MERGED_FASTQ_SSU_OTU.tsv	Root-Environmental-Aquatic-Freshwater-Lake
ERR1897754	MGYA00351699	https://www.ebi.ac.uk/metagenomics/api/v1/analyses/MGYA00351699/file/ERR1897754_FASTQ_SSU_OTU.tsv	Root-Environmental-Aquatic-Freshwater-Lake
ERR1582518	MGYA00352755	https://www.ebi.ac.uk/metagenomics/api/v1/analyses/MGYA00352755/file/ERR1582518_FASTQ_SSU_OTU.tsv	Root-Environmental-Aquatic-Freshwater-Lake
ERR1583358	MGYA00353595	https://www.ebi.ac.uk/metagenomics/api/v1/analyses/MGYA00353595/file/ERR1583358_FASTQ_SSU_OTU.tsv	Root-Environmental-Aquatic-Freshwater-Lake
ERR1583389	MGYA00353626	https://www.ebi.ac.uk/metagenomics/api/v1/analyses/MGYA00353626/file/ERR1583389_FASTQ_SSU_OTU.tsv	Root-Environmental-Aquatic-Freshwater-Lake
ERR1896662	MGYA00350607	https://www.ebi.ac.uk/metagenomics/api/v1/analyses/MGYA00350607/file/ERR1896662_FASTQ_SSU_OTU.tsv	Root-Environmental-Aquatic-Freshwater-Lake
ERR1897022	MGYA00350967	https://www.ebi.ac.uk/metagenomics/api/v1/analyses/MGYA00350967/file/ERR1897022_FASTQ_SSU_OTU.tsv	Root-Environmental-Aquatic-Freshwater-Lake
ERR1897443	MGYA00351388	https://www.ebi.ac.uk/metagenomics/api/v1/analyses/MGYA00351388/file/ERR1897443_FASTQ_SSU_OTU.tsv	Root-Environmental-Aquatic-Freshwater-Lake

SRR7081258	MGYA00425154	https://www.ebi.ac.uk/metagenomics/api/v1/analyses/MGYA00425154/file/SRR7081258_MERGED_FASTQ_SSU_OTU.tsv	Root-Environmental-Aquatic-Freshwater-Lake
ERR1743330	MGYA00087181	https://www.ebi.ac.uk/metagenomics/api/v1/analyses/MGYA00087181/file/ERR1743330_MERGED_FASTQ_otu.tsv	Root-Environmental-Aquatic-Freshwater-Lake
ERR1582491	MGYA00352728	https://www.ebi.ac.uk/metagenomics/api/v1/analyses/MGYA00352728/file/ERR1582491_FASTQ_SSU_OTU.tsv	Root-Environmental-Aquatic-Freshwater-Lake
ERR1582336	MGYA00352573	https://www.ebi.ac.uk/metagenomics/api/v1/analyses/MGYA00352573/file/ERR1582336_FASTQ_SSU_OTU.tsv	Root-Environmental-Aquatic-Freshwater-Lake
ERR1547057	MGYA00320768	https://www.ebi.ac.uk/metagenomics/api/v1/analyses/MGYA00320768/file/ERR1547057_FASTQ_SSU_OTU.tsv	Root-Environmental-Aquatic-Freshwater-Lake
ERR658209	MGYA00103488	https://www.ebi.ac.uk/metagenomics/api/v1/analyses/MGYA00103488/file/ERR658209_FASTQ_otu.tsv	Root-Environmental-Aquatic-Freshwater-Lake
ERR1582757	MGYA00352994	https://www.ebi.ac.uk/metagenomics/api/v1/analyses/MGYA00352994/file/ERR1582757_FASTQ_SSU_OTU.tsv	Root-Environmental-Aquatic-Freshwater-Lake
ERR1314390	MGYA00095225	https://www.ebi.ac.uk/metagenomics/api/v1/analyses/MGYA00095225/file/ERR1314390_MERGED_FASTQ_otu.tsv	Root-Environmental-Aquatic-Freshwater-Lake
ERR1583117	MGYA00353354	https://www.ebi.ac.uk/metagenomics/api/v1/analyses/MGYA00353354/file/ERR1583117_FASTQ_SSU_OTU.tsv	Root-Environmental-Aquatic-Freshwater-Lake
ERR1582021	MGYA00352258	https://www.ebi.ac.uk/metagenomics/api/v1/analyses/MGYA00352258/file/ERR1582021_FASTQ_SSU_OTU.tsv	Root-Environmental-Aquatic-Freshwater-Lake
ERR1314341	MGYA00095181	https://www.ebi.ac.uk/metagenomics/api/v1/analyses/MGYA00095181/file/ERR1314341_MERGED_FASTQ_otu.tsv	Root-Environmental-Aquatic-Freshwater-Lake
ERR1582786	MGYA00353023	https://www.ebi.ac.uk/metagenomics/api/v1/analyses/MGYA00353023/file/ERR1582786_FASTQ_SSU_OTU.tsv	Root-Environmental-Aquatic-Freshwater-Lake

ERR1547086	MGYA00320797	https://www.ebi.ac.uk/metagenomics/api/v1/analyses/MGYA00320797/file/ERR1547086_FASTQ_SSU_OTU.tsv	Root-Environmental-Aquatic-Freshwater-Lake
ERR1582832	MGYA00353069	https://www.ebi.ac.uk/metagenomics/api/v1/analyses/MGYA00353069/file/ERR1582832_FASTQ_SSU_OTU.tsv	Root-Environmental-Aquatic-Freshwater-Lake
ERR1582440	MGYA00352677	https://www.ebi.ac.uk/metagenomics/api/v1/analyses/MGYA00352677/file/ERR1582440_FASTQ_SSU_OTU.tsv	Root-Environmental-Aquatic-Freshwater-Lake
ERR1583200	MGYA00353437	https://www.ebi.ac.uk/metagenomics/api/v1/analyses/MGYA00353437/file/ERR1583200_FASTQ_SSU_OTU.tsv	Root-Environmental-Aquatic-Freshwater-Lake
ERR1896832	MGYA00350777	https://www.ebi.ac.uk/metagenomics/api/v1/analyses/MGYA00350777/file/ERR1896832_FASTQ_SSU_OTU.tsv	Root-Environmental-Aquatic-Freshwater-Lake
ERR1897200	MGYA00351145	https://www.ebi.ac.uk/metagenomics/api/v1/analyses/MGYA00351145/file/ERR1897200_FASTQ_SSU_OTU.tsv	Root-Environmental-Aquatic-Freshwater-Lake
ERR658173	MGYA00103481	https://www.ebi.ac.uk/metagenomics/api/v1/analyses/MGYA00103481/file/ERR658173_FASTQ_otu.tsv	Root-Environmental-Aquatic-Freshwater-Lake
ERR1897661	MGYA00351606	https://www.ebi.ac.uk/metagenomics/api/v1/analyses/MGYA00351606/file/ERR1897661_FASTQ_SSU_OTU.tsv	Root-Environmental-Aquatic-Freshwater-Lake
ERR1896786	MGYA00350731	https://www.ebi.ac.uk/metagenomics/api/v1/analyses/MGYA00350731/file/ERR1896786_FASTQ_SSU_OTU.tsv	Root-Environmental-Aquatic-Freshwater-Lake
ERR1896757	MGYA00350702	https://www.ebi.ac.uk/metagenomics/api/v1/analyses/MGYA00350702/file/ERR1896757_FASTQ_SSU_OTU.tsv	Root-Environmental-Aquatic-Freshwater-Lake
ERR1897117	MGYA00351062	https://www.ebi.ac.uk/metagenomics/api/v1/analyses/MGYA00351062/file/ERR1897117_FASTQ_SSU_OTU.tsv	Root-Environmental-Aquatic-Freshwater-Lake
ERR1897576	MGYA00351521	https://www.ebi.ac.uk/metagenomics/api/v1/analyses/MGYA00351521/file/ERR1897576_FASTQ_SSU_OTU.tsv	Root-Environmental-Aquatic-Freshwater-Lake

ERR1437521	MGYA00107969	https://www.ebi.ac.uk/metagenomics/api/v1/analyses/MGYA00107969/file/ERR1437521_FASTQ_otu.tsv	Root-Environmental-Aquatic-Freshwater-Lake
ERR1583443	MGYA00353680	https://www.ebi.ac.uk/metagenomics/api/v1/analyses/MGYA00353680/file/ERR1583443_FASTQ_SSU_OTU.tsv	Root-Environmental-Aquatic-Freshwater-Lake
ERR1582203	MGYA00352440	https://www.ebi.ac.uk/metagenomics/api/v1/analyses/MGYA00352440/file/ERR1582203_FASTQ_SSU_OTU.tsv	Root-Environmental-Aquatic-Freshwater-Lake
ERR1582662	MGYA00352899	https://www.ebi.ac.uk/metagenomics/api/v1/analyses/MGYA00352899/file/ERR1582662_FASTQ_SSU_OTU.tsv	Root-Environmental-Aquatic-Freshwater-Lake
ERR1583022	MGYA00353259	https://www.ebi.ac.uk/metagenomics/api/v1/analyses/MGYA00353259/file/ERR1583022_FASTQ_SSU_OTU.tsv	Root-Environmental-Aquatic-Freshwater-Lake
SRR7081235	MGYA00425696	https://www.ebi.ac.uk/metagenomics/api/v1/analyses/MGYA00425696/file/SRR7081235_MERGED_FASTQ_SSU_OTU.tsv	Root-Environmental-Aquatic-Freshwater-Lake
ERR1897389	MGYA00351334	https://www.ebi.ac.uk/metagenomics/api/v1/analyses/MGYA00351334/file/ERR1897389_FASTQ_SSU_OTU.tsv	Root-Environmental-Aquatic-Freshwater-Lake
SRR2442958	MGYA00358962	https://www.ebi.ac.uk/metagenomics/api/v1/analyses/MGYA00358962/file/SRR2442958_MERGED_FASTQ_SSU_OTU.tsv	Root-Environmental-Aquatic-Freshwater-Lake
SRR2442989	MGYA00358993	https://www.ebi.ac.uk/metagenomics/api/v1/analyses/MGYA00358993/file/SRR2442989_MERGED_FASTQ_SSU_OTU.tsv	Root-Environmental-Aquatic-Freshwater-Lake
ERR1897358	MGYA00351303	https://www.ebi.ac.uk/metagenomics/api/v1/analyses/MGYA00351303/file/ERR1897358_FASTQ_SSU_OTU.tsv	Root-Environmental-Aquatic-Freshwater-Lake
ERR1582114	MGYA00352351	https://www.ebi.ac.uk/metagenomics/api/v1/analyses/MGYA00352351/file/ERR1582114_FASTQ_SSU_OTU.tsv	Root-Environmental-Aquatic-Freshwater-Lake
ERR1897739	MGYA00351684	https://www.ebi.ac.uk/metagenomics/api/v1/analyses/MGYA00351684/file/ERR1897739_FASTQ_SSU_OTU.tsv	Root-Environmental-Aquatic-Freshwater-Lake

ERR1582907	MGYA00353144	https://www.ebi.ac.uk/metagenomics/api/v1/analyses/MGYA00353144/file/ERR1582907_FASTQ_SSU_OTU.tsv	Root-Environmental-Aquatic-Freshwater-Lake
ERR1582575	MGYA00352812	https://www.ebi.ac.uk/metagenomics/api/v1/analyses/MGYA00352812/file/ERR1582575_FASTQ_SSU_OTU.tsv	Root-Environmental-Aquatic-Freshwater-Lake
ERR1583335	MGYA00353572	https://www.ebi.ac.uk/metagenomics/api/v1/analyses/MGYA00353572/file/ERR1583335_FASTQ_SSU_OTU.tsv	Root-Environmental-Aquatic-Freshwater-Lake
ERR1583492	MGYA00353729	https://www.ebi.ac.uk/metagenomics/api/v1/analyses/MGYA00353729/file/ERR1583492_FASTQ_SSU_OTU.tsv	Root-Environmental-Aquatic-Freshwater-Lake
ERR1547097	MGYA00320808	https://www.ebi.ac.uk/metagenomics/api/v1/analyses/MGYA00320808/file/ERR1547097_FASTQ_SSU_OTU.tsv	Root-Environmental-Aquatic-Freshwater-Lake
ERR1582797	MGYA00353034	https://www.ebi.ac.uk/metagenomics/api/v1/analyses/MGYA00353034/file/ERR1582797_FASTQ_SSU_OTU.tsv	Root-Environmental-Aquatic-Freshwater-Lake
ERR1314350	MGYA00095171	https://www.ebi.ac.uk/metagenomics/api/v1/analyses/MGYA00095171/file/ERR1314350_MERGED_FASTQ_otu.tsv	Root-Environmental-Aquatic-Freshwater-Lake
ERR1582030	MGYA00352267	https://www.ebi.ac.uk/metagenomics/api/v1/analyses/MGYA00352267/file/ERR1582030_FASTQ_SSU_OTU.tsv	Root-Environmental-Aquatic-Freshwater-Lake
ERR1582451	MGYA00352688	https://www.ebi.ac.uk/metagenomics/api/v1/analyses/MGYA00352688/file/ERR1582451_FASTQ_SSU_OTU.tsv	Root-Environmental-Aquatic-Freshwater-Lake
ERR1582823	MGYA00353060	https://www.ebi.ac.uk/metagenomics/api/v1/analyses/MGYA00353060/file/ERR1582823_FASTQ_SSU_OTU.tsv	Root-Environmental-Aquatic-Freshwater-Lake
ERR1583211	MGYA00353448	https://www.ebi.ac.uk/metagenomics/api/v1/analyses/MGYA00353448/file/ERR1583211_FASTQ_SSU_OTU.tsv	Root-Environmental-Aquatic-Freshwater-Lake
ERR1582327	MGYA00352564	https://www.ebi.ac.uk/metagenomics/api/v1/analyses/MGYA00352564/file/ERR1582327_FASTQ_SSU_OTU.tsv	Root-Environmental-Aquatic-Freshwater-Lake

ERR1582480	MGYA00352717	https://www.ebi.ac.uk/metagenomics/api/v1/analyses/MGYA00352717/file/ERR1582480_FASTQ_SSU_OTU.tsv	Root-Environmental-Aquatic-Freshwater-Lake
ERR1314381	MGYA00095190	https://www.ebi.ac.uk/metagenomics/api/v1/analyses/MGYA00095190/file/ERR1314381_MERGED_FASTQ_otu.tsv	Root-Environmental-Aquatic-Freshwater-Lake
ERR1582746	MGYA00352983	https://www.ebi.ac.uk/metagenomics/api/v1/analyses/MGYA00352983/file/ERR1582746_FASTQ_SSU_OTU.tsv	Root-Environmental-Aquatic-Freshwater-Lake
ERR1583106	MGYA00353343	https://www.ebi.ac.uk/metagenomics/api/v1/analyses/MGYA00353343/file/ERR1583106_FASTQ_SSU_OTU.tsv	Root-Environmental-Aquatic-Freshwater-Lake
ERR1743279	MGYA00087177	https://www.ebi.ac.uk/metagenomics/api/v1/analyses/MGYA00087177/file/ERR1743279_MERGED_FASTQ_otu.tsv	Root-Environmental-Aquatic-Freshwater-Lake
ERR1547046	MGYA00320757	https://www.ebi.ac.uk/metagenomics/api/v1/analyses/MGYA00320757/file/ERR1547046_FASTQ_SSU_OTU.tsv	Root-Environmental-Aquatic-Freshwater-Lake
ERR1896673	MGYA00350618	https://www.ebi.ac.uk/metagenomics/api/v1/analyses/MGYA00350618/file/ERR1896673_FASTQ_SSU_OTU.tsv	Root-Environmental-Aquatic-Freshwater-Lake
ERR1897033	MGYA00350978	https://www.ebi.ac.uk/metagenomics/api/v1/analyses/MGYA00350978/file/ERR1897033_FASTQ_SSU_OTU.tsv	Root-Environmental-Aquatic-Freshwater-Lake
ERR1583398	MGYA00353635	https://www.ebi.ac.uk/metagenomics/api/v1/analyses/MGYA00353635/file/ERR1583398_FASTQ_SSU_OTU.tsv	Root-Environmental-Aquatic-Freshwater-Lake
ERR1897452	MGYA00351397	https://www.ebi.ac.uk/metagenomics/api/v1/analyses/MGYA00351397/file/ERR1897452_FASTQ_SSU_OTU.tsv	Root-Environmental-Aquatic-Freshwater-Lake
ERR1743321	MGYA00087121	https://www.ebi.ac.uk/metagenomics/api/v1/analyses/MGYA00087121/file/ERR1743321_MERGED_FASTQ_otu.tsv	Root-Environmental-Aquatic-Freshwater-Lake
ERR1582168	MGYA00352405	https://www.ebi.ac.uk/metagenomics/api/v1/analyses/MGYA00352405/file/ERR1582168_FASTQ_SSU_OTU.tsv	Root-Environmental-Aquatic-Freshwater-Lake

ERR1896916	MGYA00350861	https://www.ebi.ac.uk/metagenomics/api/v1/analyses/MGYA00350861/file/ERR1896916_FASTQ_SSU_OTU.tsv	Root-Environmental-Aquatic-Freshwater-Lake
ERR1897324	MGYA00351269	https://www.ebi.ac.uk/metagenomics/api/v1/analyses/MGYA00351269/file/ERR1897324_FASTQ_SSU_OTU.tsv	Root-Environmental-Aquatic-Freshwater-Lake
ERR1897483	MGYA00351428	https://www.ebi.ac.uk/metagenomics/api/v1/analyses/MGYA00351428/file/ERR1897483_FASTQ_SSU_OTU.tsv	Root-Environmental-Aquatic-Freshwater-Lake
ERR1582509	MGYA00352746	https://www.ebi.ac.uk/metagenomics/api/v1/analyses/MGYA00352746/file/ERR1582509_FASTQ_SSU_OTU.tsv	Root-Environmental-Aquatic-Freshwater-Lake
ERR1583349	MGYA00353586	https://www.ebi.ac.uk/metagenomics/api/v1/analyses/MGYA00353586/file/ERR1583349_FASTQ_SSU_OTU.tsv	Root-Environmental-Aquatic-Freshwater-Lake
SRR2443001	MGYA00359005	https://www.ebi.ac.uk/metagenomics/api/v1/analyses/MGYA00359005/file/SRR2443001_MERGED_FASTQ_SSU_OTU.tsv	Root-Environmental-Aquatic-Freshwater-Lake
ERR1897745	MGYA00351690	https://www.ebi.ac.uk/metagenomics/api/v1/analyses/MGYA00351690/file/ERR1897745_FASTQ_SSU_OTU.tsv	Root-Environmental-Aquatic-Freshwater-Lake
ERR1547090	MGYA00320801	https://www.ebi.ac.uk/metagenomics/api/v1/analyses/MGYA00320801/file/ERR1547090_FASTQ_SSU_OTU.tsv	Root-Environmental-Aquatic-Freshwater-Lake
ERR1896849	MGYA00350794	https://www.ebi.ac.uk/metagenomics/api/v1/analyses/MGYA00350794/file/ERR1896849_FASTQ_SSU_OTU.tsv	Root-Environmental-Aquatic-Freshwater-Lake
ERR1582037	MGYA00352274	https://www.ebi.ac.uk/metagenomics/api/v1/analyses/MGYA00352274/file/ERR1582037_FASTQ_SSU_OTU.tsv	Root-Environmental-Aquatic-Freshwater-Lake
ERR1582790	MGYA00353027	https://www.ebi.ac.uk/metagenomics/api/v1/analyses/MGYA00353027/file/ERR1582790_FASTQ_SSU_OTU.tsv	Root-Environmental-Aquatic-Freshwater-Lake
ERR1314357	MGYA00095227	https://www.ebi.ac.uk/metagenomics/api/v1/analyses/MGYA00095227/file/ERR1314357_MERGED_FASTQ_otu.tsv	Root-Environmental-Aquatic-Freshwater-Lake

ERR1583216	MGYA00353453	https://www.ebi.ac.uk/metagenomics/api/v1/analyses/MGYA00353453/file/ERR1583216_FASTQ_SSU_OTU.tsv	Root-Environmental-Aquatic-Freshwater-Lake
ERR1582824	MGYA00353061	https://www.ebi.ac.uk/metagenomics/api/v1/analyses/MGYA00353061/file/ERR1582824_FASTQ_SSU_OTU.tsv	Root-Environmental-Aquatic-Freshwater-Lake
ERR1582456	MGYA00352693	https://www.ebi.ac.uk/metagenomics/api/v1/analyses/MGYA00352693/file/ERR1582456_FASTQ_SSU_OTU.tsv	Root-Environmental-Aquatic-Freshwater-Lake
ERR1582487	MGYA00352724	https://www.ebi.ac.uk/metagenomics/api/v1/analyses/MGYA00352724/file/ERR1582487_FASTQ_SSU_OTU.tsv	Root-Environmental-Aquatic-Freshwater-Lake
ERR1582320	MGYA00352557	https://www.ebi.ac.uk/metagenomics/api/v1/analyses/MGYA00352557/file/ERR1582320_FASTQ_SSU_OTU.tsv	Root-Environmental-Aquatic-Freshwater-Lake
ERR1583101	MGYA00353338	https://www.ebi.ac.uk/metagenomics/api/v1/analyses/MGYA00353338/file/ERR1583101_FASTQ_SSU_OTU.tsv	Root-Environmental-Aquatic-Freshwater-Lake
ERR1314386	MGYA00095194	https://www.ebi.ac.uk/metagenomics/api/v1/analyses/MGYA00095194/file/ERR1314386_MERGED_FASTQ_otu.tsv	Root-Environmental-Aquatic-Freshwater-Lake
ERR1582741	MGYA00352978	https://www.ebi.ac.uk/metagenomics/api/v1/analyses/MGYA00352978/file/ERR1582741_FASTQ_SSU_OTU.tsv	Root-Environmental-Aquatic-Freshwater-Lake
ERR1547041	MGYA00320752	https://www.ebi.ac.uk/metagenomics/api/v1/analyses/MGYA00320752/file/ERR1547041_FASTQ_SSU_OTU.tsv	Root-Environmental-Aquatic-Freshwater-Lake
ERR1896898	MGYA00350843	https://www.ebi.ac.uk/metagenomics/api/v1/analyses/MGYA00350843/file/ERR1896898_FASTQ_SSU_OTU.tsv	Root-Environmental-Aquatic-Freshwater-Lake
SRR7081316	MGYA00424831	https://www.ebi.ac.uk/metagenomics/api/v1/analyses/MGYA00424831/file/SRR7081316_MERGED_FASTQ_SSU_OTU.tsv	Root-Environmental-Aquatic-Freshwater-Lake
ERR1897034	MGYA00350979	https://www.ebi.ac.uk/metagenomics/api/v1/analyses/MGYA00350979/file/ERR1897034_FASTQ_SSU_OTU.tsv	Root-Environmental-Aquatic-Freshwater-Lake

ERR1896674	MGYA00350619	https://www.ebi.ac.uk/metagenomics/api/v1/analyses/MGYA00350619/file/ERR1896674_FASTQ_SSU_OTU.tsv	Root-Environmental-Aquatic-Freshwater-Lake
ERR1582278	MGYA00352515	https://www.ebi.ac.uk/metagenomics/api/v1/analyses/MGYA00352515/file/ERR1582278_FASTQ_SSU_OTU.tsv	Root-Environmental-Aquatic-Freshwater-Lake
ERR1583438	MGYA00353675	https://www.ebi.ac.uk/metagenomics/api/v1/analyses/MGYA00353675/file/ERR1583438_FASTQ_SSU_OTU.tsv	Root-Environmental-Aquatic-Freshwater-Lake
ERR1743326	MGYA00087217	https://www.ebi.ac.uk/metagenomics/api/v1/analyses/MGYA00087217/file/ERR1743326_MERGED_FASTQ_otu.tsv	Root-Environmental-Aquatic-Freshwater-Lake
ERR1547119	MGYA00320830	https://www.ebi.ac.uk/metagenomics/api/v1/analyses/MGYA00320830/file/ERR1547119_FASTQ_SSU_OTU.tsv	Root-Environmental-Aquatic-Freshwater-Lake
ERR1897455	MGYA00351400	https://www.ebi.ac.uk/metagenomics/api/v1/analyses/MGYA00351400/file/ERR1897455_FASTQ_SSU_OTU.tsv	Root-Environmental-Aquatic-Freshwater-Lake
ERR1583059	MGYA00353296	https://www.ebi.ac.uk/metagenomics/api/v1/analyses/MGYA00353296/file/ERR1583059_FASTQ_SSU_OTU.tsv	Root-Environmental-Aquatic-Freshwater-Lake
ERR1582619	MGYA00352856	https://www.ebi.ac.uk/metagenomics/api/v1/analyses/MGYA00352856/file/ERR1582619_FASTQ_SSU_OTU.tsv	Root-Environmental-Aquatic-Freshwater-Lake
ERR1583088	MGYA00353325	https://www.ebi.ac.uk/metagenomics/api/v1/analyses/MGYA00353325/file/ERR1583088_FASTQ_SSU_OTU.tsv	Root-Environmental-Aquatic-Freshwater-Lake
ERR1897484	MGYA00351429	https://www.ebi.ac.uk/metagenomics/api/v1/analyses/MGYA00351429/file/ERR1897484_FASTQ_SSU_OTU.tsv	Root-Environmental-Aquatic-Freshwater-Lake
ERR1897323	MGYA00351268	https://www.ebi.ac.uk/metagenomics/api/v1/analyses/MGYA00351268/file/ERR1897323_FASTQ_SSU_OTU.tsv	Root-Environmental-Aquatic-Freshwater-Lake
ERR1896911	MGYA00350856	https://www.ebi.ac.uk/metagenomics/api/v1/analyses/MGYA00350856/file/ERR1896911_FASTQ_SSU_OTU.tsv	Root-Environmental-Aquatic-Freshwater-Lake

SRR2443006	MGYA00359010	https://www.ebi.ac.uk/metagenomics/api/v1/analyses/MGYA00359010/file/SRR2443006_MERGED_FASTQ_SSU_OTU.tsv	Root-Environmental-Aquatic-Freshwater-Lake
ERR1897742	MGYA00351687	https://www.ebi.ac.uk/metagenomics/api/v1/analyses/MGYA00351687/file/ERR1897742_FASTQ_SSU_OTU.tsv	Root-Environmental-Aquatic-Freshwater-Lake
ERR1897207	MGYA00351152	https://www.ebi.ac.uk/metagenomics/api/v1/analyses/MGYA00351152/file/ERR1897207_FASTQ_SSU_OTU.tsv	Root-Environmental-Aquatic-Freshwater-Lake
ERR1896835	MGYA00350780	https://www.ebi.ac.uk/metagenomics/api/v1/analyses/MGYA00350780/file/ERR1896835_FASTQ_SSU_OTU.tsv	Root-Environmental-Aquatic-Freshwater-Lake
ERR658174	MGYA00103478	https://www.ebi.ac.uk/metagenomics/api/v1/analyses/MGYA00103478/file/ERR658174_FASTQ_otu.tsv	Root-Environmental-Aquatic-Freshwater-Lake
ERR1582858	MGYA00353095	https://www.ebi.ac.uk/metagenomics/api/v1/analyses/MGYA00353095/file/ERR1582858_FASTQ_SSU_OTU.tsv	Root-Environmental-Aquatic-Freshwater-Lake
ERR1896781	MGYA00350726	https://www.ebi.ac.uk/metagenomics/api/v1/analyses/MGYA00350726/file/ERR1896781_FASTQ_SSU_OTU.tsv	Root-Environmental-Aquatic-Freshwater-Lake
ERR1897666	MGYA00351611	https://www.ebi.ac.uk/metagenomics/api/v1/analyses/MGYA00351611/file/ERR1897666_FASTQ_SSU_OTU.tsv	Root-Environmental-Aquatic-Freshwater-Lake
ERR1897110	MGYA00351055	https://www.ebi.ac.uk/metagenomics/api/v1/analyses/MGYA00351055/file/ERR1897110_FASTQ_SSU_OTU.tsv	Root-Environmental-Aquatic-Freshwater-Lake
ERR1896750	MGYA00350695	https://www.ebi.ac.uk/metagenomics/api/v1/analyses/MGYA00350695/file/ERR1896750_FASTQ_SSU_OTU.tsv	Root-Environmental-Aquatic-Freshwater-Lake
ERR1429279	MGYA00107663	https://www.ebi.ac.uk/metagenomics/api/v1/analyses/MGYA00107663/file/ERR1429279_FASTQ_otu.tsv	Root-Environmental-Aquatic-Freshwater-Lake
ERR1582889	MGYA00353126	https://www.ebi.ac.uk/metagenomics/api/v1/analyses/MGYA00353126/file/ERR1582889_FASTQ_SSU_OTU.tsv	Root-Environmental-Aquatic-Freshwater-Lake

ERR1897571	MGYA00351516	https://www.ebi.ac.uk/metagenomics/api/v1/analyses/MGYA00351516/file/ERR1897571_FASTQ_SSU_OTU.tsv	Root-Environmental-Aquatic-Freshwater-Lake
ERR1582204	MGYA00352441	https://www.ebi.ac.uk/metagenomics/api/v1/analyses/MGYA00352441/file/ERR1582204_FASTQ_SSU_OTU.tsv	Root-Environmental-Aquatic-Freshwater-Lake
ERR1583444	MGYA00353681	https://www.ebi.ac.uk/metagenomics/api/v1/analyses/MGYA00353681/file/ERR1583444_FASTQ_SSU_OTU.tsv	Root-Environmental-Aquatic-Freshwater-Lake
ERR1897048	MGYA00350993	https://www.ebi.ac.uk/metagenomics/api/v1/analyses/MGYA00350993/file/ERR1897048_FASTQ_SSU_OTU.tsv	Root-Environmental-Aquatic-Freshwater-Lake
ERR1583025	MGYA00353262	https://www.ebi.ac.uk/metagenomics/api/v1/analyses/MGYA00353262/file/ERR1583025_FASTQ_SSU_OTU.tsv	Root-Environmental-Aquatic-Freshwater-Lake
ERR1582665	MGYA00352902	https://www.ebi.ac.uk/metagenomics/api/v1/analyses/MGYA00352902/file/ERR1582665_FASTQ_SSU_OTU.tsv	Root-Environmental-Aquatic-Freshwater-Lake
ERR1897429	MGYA00351374	https://www.ebi.ac.uk/metagenomics/api/v1/analyses/MGYA00351374/file/ERR1897429_FASTQ_SSU_OTU.tsv	Root-Environmental-Aquatic-Freshwater-Lake
ERR1582113	MGYA00352350	https://www.ebi.ac.uk/metagenomics/api/v1/analyses/MGYA00352350/file/ERR1582113_FASTQ_SSU_OTU.tsv	Root-Environmental-Aquatic-Freshwater-Lake
ERR1897099	MGYA00351044	https://www.ebi.ac.uk/metagenomics/api/v1/analyses/MGYA00351044/file/ERR1897099_FASTQ_SSU_OTU.tsv	Root-Environmental-Aquatic-Freshwater-Lake
ERR1583495	MGYA00353732	https://www.ebi.ac.uk/metagenomics/api/v1/analyses/MGYA00353732/file/ERR1583495_FASTQ_SSU_OTU.tsv	Root-Environmental-Aquatic-Freshwater-Lake
ERR1583332	MGYA00353569	https://www.ebi.ac.uk/metagenomics/api/v1/analyses/MGYA00353569/file/ERR1583332_FASTQ_SSU_OTU.tsv	Root-Environmental-Aquatic-Freshwater-Lake
ERR1582572	MGYA00352809	https://www.ebi.ac.uk/metagenomics/api/v1/analyses/MGYA00352809/file/ERR1582572_FASTQ_SSU_OTU.tsv	Root-Environmental-Aquatic-Freshwater-Lake

ERR1582900	MGYA00353137	https://www.ebi.ac.uk/metagenomics/api/v1/analyses/MGYA00353137/file/ERR1582900_FASTQ_SSU_OTU.tsv	Root-Environmental-Aquatic-Freshwater-Lake
ERR1582039	MGYA00352276	https://www.ebi.ac.uk/metagenomics/api/v1/analyses/MGYA00352276/file/ERR1582039_FASTQ_SSU_OTU.tsv	Root-Environmental-Aquatic-Freshwater-Lake
ERR1314359	MGYA00095152	https://www.ebi.ac.uk/metagenomics/api/v1/analyses/MGYA00095152/file/ERR1314359_MERGED_FASTQ_otu.tsv	Root-Environmental-Aquatic-Freshwater-Lake
ERR1896847	MGYA00350792	https://www.ebi.ac.uk/metagenomics/api/v1/analyses/MGYA00350792/file/ERR1896847_FASTQ_SSU_OTU.tsv	Root-Environmental-Aquatic-Freshwater-Lake
ERR1897275	MGYA00351220	https://www.ebi.ac.uk/metagenomics/api/v1/analyses/MGYA00351220/file/ERR1897275_FASTQ_SSU_OTU.tsv	Root-Environmental-Aquatic-Freshwater-Lake
ERR1582458	MGYA00352695	https://www.ebi.ac.uk/metagenomics/api/v1/analyses/MGYA00352695/file/ERR1582458_FASTQ_SSU_OTU.tsv	Root-Environmental-Aquatic-Freshwater-Lake
ERR1583218	MGYA00353455	https://www.ebi.ac.uk/metagenomics/api/v1/analyses/MGYA00353455/file/ERR1583218_FASTQ_SSU_OTU.tsv	Root-Environmental-Aquatic-Freshwater-Lake
ERR1897614	MGYA00351559	https://www.ebi.ac.uk/metagenomics/api/v1/analyses/MGYA00351559/file/ERR1897614_FASTQ_SSU_OTU.tsv	Root-Environmental-Aquatic-Freshwater-Lake
ERR1896722	MGYA00350667	https://www.ebi.ac.uk/metagenomics/api/v1/analyses/MGYA00350667/file/ERR1896722_FASTQ_SSU_OTU.tsv	Root-Environmental-Aquatic-Freshwater-Lake
ERR1897162	MGYA00351107	https://www.ebi.ac.uk/metagenomics/api/v1/analyses/MGYA00351107/file/ERR1897162_FASTQ_SSU_OTU.tsv	Root-Environmental-Aquatic-Freshwater-Lake
ERR1582489	MGYA00352726	https://www.ebi.ac.uk/metagenomics/api/v1/analyses/MGYA00352726/file/ERR1582489_FASTQ_SSU_OTU.tsv	Root-Environmental-Aquatic-Freshwater-Lake
ERR1897503	MGYA00351448	https://www.ebi.ac.uk/metagenomics/api/v1/analyses/MGYA00351448/file/ERR1897503_FASTQ_SSU_OTU.tsv	Root-Environmental-Aquatic-Freshwater-Lake

SRR7081318	MGYA00424824	https://www.ebi.ac.uk/metagenomics/api/v1/analyses/MGYA00424824/file/SRR7081318_MERGED_FASTQ_SSU_OTU.tsv	Root-Environmental-Aquatic-Freshwater-Lake
ERR1896896	MGYA00350841	https://www.ebi.ac.uk/metagenomics/api/v1/analyses/MGYA00350841/file/ERR1896896_FASTQ_SSU_OTU.tsv	Root-Environmental-Aquatic-Freshwater-Lake
ERR658211	MGYA00103491	https://www.ebi.ac.uk/metagenomics/api/v1/analyses/MGYA00103491/file/ERR658211_FASTQ_otu.tsv	Root-Environmental-Aquatic-Freshwater-Lake
ERR1314388	MGYA00095183	https://www.ebi.ac.uk/metagenomics/api/v1/analyses/MGYA00095183/file/ERR1314388_MERGED_FASTQ_otu.tsv	Root-Environmental-Aquatic-Freshwater-Lake
ERR1583391	MGYA00353628	https://www.ebi.ac.uk/metagenomics/api/v1/analyses/MGYA00353628/file/ERR1583391_FASTQ_SSU_OTU.tsv	Root-Environmental-Aquatic-Freshwater-Lake
ERR1583436	MGYA00353673	https://www.ebi.ac.uk/metagenomics/api/v1/analyses/MGYA00353673/file/ERR1583436_FASTQ_SSU_OTU.tsv	Root-Environmental-Aquatic-Freshwater-Lake
ERR1582276	MGYA00352513	https://www.ebi.ac.uk/metagenomics/api/v1/analyses/MGYA00352513/file/ERR1582276_FASTQ_SSU_OTU.tsv	Root-Environmental-Aquatic-Freshwater-Lake
ERR1582617	MGYA00352854	https://www.ebi.ac.uk/metagenomics/api/v1/analyses/MGYA00352854/file/ERR1582617_FASTQ_SSU_OTU.tsv	Root-Environmental-Aquatic-Freshwater-Lake
ERR1583057	MGYA00353294	https://www.ebi.ac.uk/metagenomics/api/v1/analyses/MGYA00353294/file/ERR1583057_FASTQ_SSU_OTU.tsv	Root-Environmental-Aquatic-Freshwater-Lake
SRR7081240	MGYA00425691	https://www.ebi.ac.uk/metagenomics/api/v1/analyses/MGYA00425691/file/SRR7081240_MERGED_FASTQ_SSU_OTU.tsv	Root-Environmental-Aquatic-Freshwater-Lake
ERR1547117	MGYA00320828	https://www.ebi.ac.uk/metagenomics/api/v1/analyses/MGYA00320828/file/ERR1547117_FASTQ_SSU_OTU.tsv	Root-Environmental-Aquatic-Freshwater-Lake
ERR1583086	MGYA00353323	https://www.ebi.ac.uk/metagenomics/api/v1/analyses/MGYA00353323/file/ERR1583086_FASTQ_SSU_OTU.tsv	Root-Environmental-Aquatic-Freshwater-Lake

ERR1582161	MGYA00352398	https://www.ebi.ac.uk/metagenomics/api/v1/analyses/MGYA00352398/file/ERR1582161_FASTQ_SSU_OTU.tsv	Root-Environmental-Aquatic-Freshwater-Lake
SRR2443008	MGYA00359012	https://www.ebi.ac.uk/metagenomics/api/v1/analyses/MGYA00359012/file/SRR2443008_MERGED_FASTQ_SSU_OTU.tsv	Root-Environmental-Aquatic-Freshwater-Lake
ERR1582500	MGYA00352737	https://www.ebi.ac.uk/metagenomics/api/v1/analyses/MGYA00352737/file/ERR1582500_FASTQ_SSU_OTU.tsv	Root-Environmental-Aquatic-Freshwater-Lake
ERR1582972	MGYA00353209	https://www.ebi.ac.uk/metagenomics/api/v1/analyses/MGYA00353209/file/ERR1582972_FASTQ_SSU_OTU.tsv	Root-Environmental-Aquatic-Freshwater-Lake
ERR1583340	MGYA00353577	https://www.ebi.ac.uk/metagenomics/api/v1/analyses/MGYA00353577/file/ERR1583340_FASTQ_SSU_OTU.tsv	Root-Environmental-Aquatic-Freshwater-Lake
ERR1897209	MGYA00351154	https://www.ebi.ac.uk/metagenomics/api/v1/analyses/MGYA00351154/file/ERR1897209_FASTQ_SSU_OTU.tsv	Root-Environmental-Aquatic-Freshwater-Lake
SRR7081412	MGYA00416199	https://www.ebi.ac.uk/metagenomics/api/v1/analyses/MGYA00416199/file/SRR7081412_MERGED_FASTQ_SSU_OTU.tsv	Root-Environmental-Aquatic-Freshwater-Lake
ERR1582045	MGYA00352282	https://www.ebi.ac.uk/metagenomics/api/v1/analyses/MGYA00352282/file/ERR1582045_FASTQ_SSU_OTU.tsv	Root-Environmental-Aquatic-Freshwater-Lake
ERR1897668	MGYA00351613	https://www.ebi.ac.uk/metagenomics/api/v1/analyses/MGYA00351613/file/ERR1897668_FASTQ_SSU_OTU.tsv	Root-Environmental-Aquatic-Freshwater-Lake
ERR1582383	MGYA00352620	https://www.ebi.ac.uk/metagenomics/api/v1/analyses/MGYA00352620/file/ERR1582383_FASTQ_SSU_OTU.tsv	Root-Environmental-Aquatic-Freshwater-Lake
ERR1582856	MGYA00353093	https://www.ebi.ac.uk/metagenomics/api/v1/analyses/MGYA00353093/file/ERR1582856_FASTQ_SSU_OTU.tsv	Root-Environmental-Aquatic-Freshwater-Lake
ERR1582424	MGYA00352661	https://www.ebi.ac.uk/metagenomics/api/v1/analyses/MGYA00352661/file/ERR1582424_FASTQ_SSU_OTU.tsv	Root-Environmental-Aquatic-Freshwater-Lake

ERR1333513	MGYA00107607	https://www.ebi.ac.uk/metagenomics/api/v1/analyses/MGYA00107607/file/ERR1333513_FASTQ_otu.tsv	Root-Environmental-Aquatic-Freshwater-Lake
ERR1583264	MGYA00353501	https://www.ebi.ac.uk/metagenomics/api/v1/analyses/MGYA00353501/file/ERR1583264_FASTQ_SSU_OTU.tsv	Root-Environmental-Aquatic-Freshwater-Lake
ERR1583512	MGYA00353749	https://www.ebi.ac.uk/metagenomics/api/v1/analyses/MGYA00353749/file/ERR1583512_FASTQ_SSU_OTU.tsv	Root-Environmental-Aquatic-Freshwater-Lake
ERR1582352	MGYA00352589	https://www.ebi.ac.uk/metagenomics/api/v1/analyses/MGYA00352589/file/ERR1582352_FASTQ_SSU_OTU.tsv	Root-Environmental-Aquatic-Freshwater-Lake
ERR1429277	MGYA00107649	https://www.ebi.ac.uk/metagenomics/api/v1/analyses/MGYA00107649/file/ERR1429277_FASTQ_otu.tsv	Root-Environmental-Aquatic-Freshwater-Lake
ERR1582887	MGYA00353124	https://www.ebi.ac.uk/metagenomics/api/v1/analyses/MGYA00353124/file/ERR1582887_FASTQ_SSU_OTU.tsv	Root-Environmental-Aquatic-Freshwater-Lake
ERR1582733	MGYA00352970	https://www.ebi.ac.uk/metagenomics/api/v1/analyses/MGYA00352970/file/ERR1582733_FASTQ_SSU_OTU.tsv	Root-Environmental-Aquatic-Freshwater-Lake
ERR1582094	MGYA00352331	https://www.ebi.ac.uk/metagenomics/api/v1/analyses/MGYA00352331/file/ERR1582094_FASTQ_SSU_OTU.tsv	Root-Environmental-Aquatic-Freshwater-Lake
ERR1583173	MGYA00353410	https://www.ebi.ac.uk/metagenomics/api/v1/analyses/MGYA00353410/file/ERR1583173_FASTQ_SSU_OTU.tsv	Root-Environmental-Aquatic-Freshwater-Lake
SRR7081364	MGYA00423214	https://www.ebi.ac.uk/metagenomics/api/v1/analyses/MGYA00423214/file/SRR7081364_MERGED_FASTQ_SSU_OTU.tsv	Root-Environmental-Aquatic-Freshwater-Lake
ERR1547033	MGYA00320744	https://www.ebi.ac.uk/metagenomics/api/v1/analyses/MGYA00320744/file/ERR1547033_FASTQ_SSU_OTU.tsv	Root-Environmental-Aquatic-Freshwater-Lake
ERR1897046	MGYA00350991	https://www.ebi.ac.uk/metagenomics/api/v1/analyses/MGYA00350991/file/ERR1897046_FASTQ_SSU_OTU.tsv	Root-Environmental-Aquatic-Freshwater-Lake

ERR1897427	MGYA00351372	https://www.ebi.ac.uk/metagenomics/api/v1/analyses/MGYA00351372/file/ERR1897427_FASTQ_SSU_OTU.tsv	Root-Environmental-Aquatic-Freshwater-Lake
ERR1743354	MGYA00087194	https://www.ebi.ac.uk/metagenomics/api/v1/analyses/MGYA00087194/file/ERR1743354_MERGED_FASTQ_otu.tsv	Root-Environmental-Aquatic-Freshwater-Lake
SRR2442951	MGYA00358955	https://www.ebi.ac.uk/metagenomics/api/v1/analyses/MGYA00358955/file/SRR2442951_MERGED_FASTQ_SSU_OTU.tsv	Root-Environmental-Aquatic-Freshwater-Lake
ERR1896963	MGYA00350908	https://www.ebi.ac.uk/metagenomics/api/v1/analyses/MGYA00350908/file/ERR1896963_FASTQ_SSU_OTU.tsv	Root-Environmental-Aquatic-Freshwater-Lake
ERR1743385	MGYA00087144	https://www.ebi.ac.uk/metagenomics/api/v1/analyses/MGYA00087144/file/ERR1743385_MERGED_FASTQ_otu.tsv	Root-Environmental-Aquatic-Freshwater-Lake
SRR2442980	MGYA00358984	https://www.ebi.ac.uk/metagenomics/api/v1/analyses/MGYA00358984/file/SRR2442980_MERGED_FASTQ_SSU_OTU.tsv	Root-Environmental-Aquatic-Freshwater-Lake
ERR1897351	MGYA00351296	https://www.ebi.ac.uk/metagenomics/api/v1/analyses/MGYA00351296/file/ERR1897351_FASTQ_SSU_OTU.tsv	Root-Environmental-Aquatic-Freshwater-Lake
ERR589324	MGYA00103460	https://www.ebi.ac.uk/metagenomics/api/v1/analyses/MGYA00103460/file/ERR589324_FASTQ_otu.tsv	Root-Environmental-Aquatic-Freshwater-Lake
ERR1897730	MGYA00351675	https://www.ebi.ac.uk/metagenomics/api/v1/analyses/MGYA00351675/file/ERR1897730_FASTQ_SSU_OTU.tsv	Root-Environmental-Aquatic-Freshwater-Lake
ERR1897097	MGYA00351042	https://www.ebi.ac.uk/metagenomics/api/v1/analyses/MGYA00351042/file/ERR1897097_FASTQ_SSU_OTU.tsv	Root-Environmental-Aquatic-Freshwater-Lake
ERR1582042	MGYA00352279	https://www.ebi.ac.uk/metagenomics/api/v1/analyses/MGYA00352279/file/ERR1582042_FASTQ_SSU_OTU.tsv	Root-Environmental-Aquatic-Freshwater-Lake
ERR1896788	MGYA00350733	https://www.ebi.ac.uk/metagenomics/api/v1/analyses/MGYA00350733/file/ERR1896788_FASTQ_SSU_OTU.tsv	Root-Environmental-Aquatic-Freshwater-Lake

SRR7081074	MGYA00414129	https://www.ebi.ac.uk/metagenomics/api/v1/analyses/MGYA00414129/file/SRR7081074_MERGED_FASTQ_SSU_OTU.tsv	Root-Environmental-Aquatic-Freshwater-Lake
ERR1583263	MGYA00353500	https://www.ebi.ac.uk/metagenomics/api/v1/analyses/MGYA00353500/file/ERR1583263_FASTQ_SSU_OTU.tsv	Root-Environmental-Aquatic-Freshwater-Lake
ERR1333514	MGYA00107597	https://www.ebi.ac.uk/metagenomics/api/v1/analyses/MGYA00107597/file/ERR1333514_FASTQ_otu.tsv	Root-Environmental-Aquatic-Freshwater-Lake
ERR1582423	MGYA00352660	https://www.ebi.ac.uk/metagenomics/api/v1/analyses/MGYA00352660/file/ERR1582423_FASTQ_SSU_OTU.tsv	Root-Environmental-Aquatic-Freshwater-Lake
ERR1582851	MGYA00353088	https://www.ebi.ac.uk/metagenomics/api/v1/analyses/MGYA00353088/file/ERR1582851_FASTQ_SSU_OTU.tsv	Root-Environmental-Aquatic-Freshwater-Lake
ERR1582384	MGYA00352621	https://www.ebi.ac.uk/metagenomics/api/v1/analyses/MGYA00352621/file/ERR1582384_FASTQ_SSU_OTU.tsv	Root-Environmental-Aquatic-Freshwater-Lake
ERR1582880	MGYA00353117	https://www.ebi.ac.uk/metagenomics/api/v1/analyses/MGYA00353117/file/ERR1582880_FASTQ_SSU_OTU.tsv	Root-Environmental-Aquatic-Freshwater-Lake
ERR1429270	MGYA00107630	https://www.ebi.ac.uk/metagenomics/api/v1/analyses/MGYA00107630/file/ERR1429270_FASTQ_otu.tsv	Root-Environmental-Aquatic-Freshwater-Lake
ERR1582355	MGYA00352592	https://www.ebi.ac.uk/metagenomics/api/v1/analyses/MGYA00352592/file/ERR1582355_FASTQ_SSU_OTU.tsv	Root-Environmental-Aquatic-Freshwater-Lake
ERR1583515	MGYA00353752	https://www.ebi.ac.uk/metagenomics/api/v1/analyses/MGYA00353752/file/ERR1583515_FASTQ_SSU_OTU.tsv	Root-Environmental-Aquatic-Freshwater-Lake
ERR1897119	MGYA00351064	https://www.ebi.ac.uk/metagenomics/api/v1/analyses/MGYA00351064/file/ERR1897119_FASTQ_SSU_OTU.tsv	Root-Environmental-Aquatic-Freshwater-Lake
ERR1896759	MGYA00350704	https://www.ebi.ac.uk/metagenomics/api/v1/analyses/MGYA00350704/file/ERR1896759_FASTQ_SSU_OTU.tsv	Root-Environmental-Aquatic-Freshwater-Lake

ERR1535992	MGYA00320283	https://www.ebi.ac.uk/metagenomics/api/v1/analyses/MGYA00320283/file/ERR1535992_FASTQ_SSU_OTU.tsv	Root-Environmental-Aquatic-Freshwater-Lake
ERR1583174	MGYA00353411	https://www.ebi.ac.uk/metagenomics/api/v1/analyses/MGYA00353411/file/ERR1583174_FASTQ_SSU_OTU.tsv	Root-Environmental-Aquatic-Freshwater-Lake
ERR1582093	MGYA00352330	https://www.ebi.ac.uk/metagenomics/api/v1/analyses/MGYA00352330/file/ERR1582093_FASTQ_SSU_OTU.tsv	Root-Environmental-Aquatic-Freshwater-Lake
ERR1582734	MGYA00352971	https://www.ebi.ac.uk/metagenomics/api/v1/analyses/MGYA00352971/file/ERR1582734_FASTQ_SSU_OTU.tsv	Root-Environmental-Aquatic-Freshwater-Lake
ERR1547034	MGYA00320745	https://www.ebi.ac.uk/metagenomics/api/v1/analyses/MGYA00320745/file/ERR1547034_FASTQ_SSU_OTU.tsv	Root-Environmental-Aquatic-Freshwater-Lake
ERR1897578	MGYA00351523	https://www.ebi.ac.uk/metagenomics/api/v1/analyses/MGYA00351523/file/ERR1897578_FASTQ_SSU_OTU.tsv	Root-Environmental-Aquatic-Freshwater-Lake
ERR1897041	MGYA00350986	https://www.ebi.ac.uk/metagenomics/api/v1/analyses/MGYA00350986/file/ERR1897041_FASTQ_SSU_OTU.tsv	Root-Environmental-Aquatic-Freshwater-Lake
SRR2442956	MGYA00358960	https://www.ebi.ac.uk/metagenomics/api/v1/analyses/MGYA00358960/file/SRR2442956_MERGED_FASTQ_SSU_OTU.tsv	Root-Environmental-Aquatic-Freshwater-Lake
ERR1743353	MGYA00087150	https://www.ebi.ac.uk/metagenomics/api/v1/analyses/MGYA00087150/file/ERR1743353_MERGED_FASTQ_otu.tsv	Root-Environmental-Aquatic-Freshwater-Lake
ERR1897420	MGYA00351365	https://www.ebi.ac.uk/metagenomics/api/v1/analyses/MGYA00351365/file/ERR1897420_FASTQ_SSU_OTU.tsv	Root-Environmental-Aquatic-Freshwater-Lake
ERR1897387	MGYA00351332	https://www.ebi.ac.uk/metagenomics/api/v1/analyses/MGYA00351332/file/ERR1897387_FASTQ_SSU_OTU.tsv	Root-Environmental-Aquatic-Freshwater-Lake
ERR589323	MGYA00103418	https://www.ebi.ac.uk/metagenomics/api/v1/analyses/MGYA00103418/file/ERR589323_FASTQ_otu.tsv	Root-Environmental-Aquatic-Freshwater-Lake

ERR1897356	MGYA00351301	https://www.ebi.ac.uk/metagenomics/api/v1/analyses/MGYA00351301/file/ERR1897356_FASTQ_SSU_OTU.tsv	Root-Environmental-Aquatic-Freshwater-Lake
SRR2442987	MGYA00358991	https://www.ebi.ac.uk/metagenomics/api/v1/analyses/MGYA00358991/file/SRR2442987_MERGED_FASTQ_SSU_OTU.tsv	Root-Environmental-Aquatic-Freshwater-Lake
ERR1743382	MGYA00087164	https://www.ebi.ac.uk/metagenomics/api/v1/analyses/MGYA00087164/file/ERR1743382_MERGED_FASTQ_otu.tsv	Root-Environmental-Aquatic-Freshwater-Lake
ERR1896964	MGYA00350909	https://www.ebi.ac.uk/metagenomics/api/v1/analyses/MGYA00350909/file/ERR1896964_FASTQ_SSU_OTU.tsv	Root-Environmental-Aquatic-Freshwater-Lake
ERR1582909	MGYA00353146	https://www.ebi.ac.uk/metagenomics/api/v1/analyses/MGYA00353146/file/ERR1582909_FASTQ_SSU_OTU.tsv	Root-Environmental-Aquatic-Freshwater-Lake
ERR1897090	MGYA00351035	https://www.ebi.ac.uk/metagenomics/api/v1/analyses/MGYA00351035/file/ERR1897090_FASTQ_SSU_OTU.tsv	Root-Environmental-Aquatic-Freshwater-Lake
ERR1897737	MGYA00351682	https://www.ebi.ac.uk/metagenomics/api/v1/analyses/MGYA00351682/file/ERR1897737_FASTQ_SSU_OTU.tsv	Root-Environmental-Aquatic-Freshwater-Lake
ERR1582799	MGYA00353036	https://www.ebi.ac.uk/metagenomics/api/v1/analyses/MGYA00353036/file/ERR1582799_FASTQ_SSU_OTU.tsv	Root-Environmental-Aquatic-Freshwater-Lake
ERR1897272	MGYA00351217	https://www.ebi.ac.uk/metagenomics/api/v1/analyses/MGYA00351217/file/ERR1897272_FASTQ_SSU_OTU.tsv	Root-Environmental-Aquatic-Freshwater-Lake
ERR1896840	MGYA00350785	https://www.ebi.ac.uk/metagenomics/api/v1/analyses/MGYA00350785/file/ERR1896840_FASTQ_SSU_OTU.tsv	Root-Environmental-Aquatic-Freshwater-Lake
ERR1547099	MGYA00320810	https://www.ebi.ac.uk/metagenomics/api/v1/analyses/MGYA00320810/file/ERR1547099_FASTQ_SSU_OTU.tsv	Root-Environmental-Aquatic-Freshwater-Lake
ERR1897613	MGYA00351558	https://www.ebi.ac.uk/metagenomics/api/v1/analyses/MGYA00351558/file/ERR1897613_FASTQ_SSU_OTU.tsv	Root-Environmental-Aquatic-Freshwater-Lake

ERR1897165	MGYA00351110	https://www.ebi.ac.uk/metagenomics/api/v1/analyses/MGYA00351110/file/ERR1897165_FASTQ_SSU_OTU.tsv	Root-Environmental-Aquatic-Freshwater-Lake
ERR1896725	MGYA00350670	https://www.ebi.ac.uk/metagenomics/api/v1/analyses/MGYA00350670/file/ERR1896725_FASTQ_SSU_OTU.tsv	Root-Environmental-Aquatic-Freshwater-Lake
ERR1582329	MGYA00352566	https://www.ebi.ac.uk/metagenomics/api/v1/analyses/MGYA00352566/file/ERR1582329_FASTQ_SSU_OTU.tsv	Root-Environmental-Aquatic-Freshwater-Lake
ERR1896891	MGYA00350836	https://www.ebi.ac.uk/metagenomics/api/v1/analyses/MGYA00350836/file/ERR1896891_FASTQ_SSU_OTU.tsv	Root-Environmental-Aquatic-Freshwater-Lake
ERR1743277	MGYA00087115	https://www.ebi.ac.uk/metagenomics/api/v1/analyses/MGYA00087115/file/ERR1743277_MERGED_FASTQ_otu.tsv	Root-Environmental-Aquatic-Freshwater-Lake
ERR1547048	MGYA00320759	https://www.ebi.ac.uk/metagenomics/api/v1/analyses/MGYA00320759/file/ERR1547048_FASTQ_SSU_OTU.tsv	Root-Environmental-Aquatic-Freshwater-Lake
ERR1897504	MGYA00351449	https://www.ebi.ac.uk/metagenomics/api/v1/analyses/MGYA00351449/file/ERR1897504_FASTQ_SSU_OTU.tsv	Root-Environmental-Aquatic-Freshwater-Lake
ERR1583108	MGYA00353345	https://www.ebi.ac.uk/metagenomics/api/v1/analyses/MGYA00353345/file/ERR1583108_FASTQ_SSU_OTU.tsv	Root-Environmental-Aquatic-Freshwater-Lake
ERR658216	MGYA00103473	https://www.ebi.ac.uk/metagenomics/api/v1/analyses/MGYA00103473/file/ERR658216_FASTQ_otu.tsv	Root-Environmental-Aquatic-Freshwater-Lake
ERR1582748	MGYA00352985	https://www.ebi.ac.uk/metagenomics/api/v1/analyses/MGYA00352985/file/ERR1582748_FASTQ_SSU_OTU.tsv	Root-Environmental-Aquatic-Freshwater-Lake
ERR1582271	MGYA00352508	https://www.ebi.ac.uk/metagenomics/api/v1/analyses/MGYA00352508/file/ERR1582271_FASTQ_SSU_OTU.tsv	Root-Environmental-Aquatic-Freshwater-Lake
ERR1583431	MGYA00353668	https://www.ebi.ac.uk/metagenomics/api/v1/analyses/MGYA00353668/file/ERR1583431_FASTQ_SSU_OTU.tsv	Root-Environmental-Aquatic-Freshwater-Lake

ERR1583396	MGYA00353633	https://www.ebi.ac.uk/metagenomics/api/v1/analyses/MGYA00353633/file/ERR1583396_FASTQ_SSU_OTU.tsv	Root-Environmental-Aquatic-Freshwater-Lake
ERR1583050	MGYA00353287	https://www.ebi.ac.uk/metagenomics/api/v1/analyses/MGYA00353287/file/ERR1583050_FASTQ_SSU_OTU.tsv	Root-Environmental-Aquatic-Freshwater-Lake
ERR1582610	MGYA00352847	https://www.ebi.ac.uk/metagenomics/api/v1/analyses/MGYA00352847/file/ERR1582610_FASTQ_SSU_OTU.tsv	Root-Environmental-Aquatic-Freshwater-Lake
ERR1547110	MGYA00320821	https://www.ebi.ac.uk/metagenomics/api/v1/analyses/MGYA00320821/file/ERR1547110_FASTQ_SSU_OTU.tsv	Root-Environmental-Aquatic-Freshwater-Lake
ERR1896918	MGYA00350863	https://www.ebi.ac.uk/metagenomics/api/v1/analyses/MGYA00350863/file/ERR1896918_FASTQ_SSU_OTU.tsv	Root-Environmental-Aquatic-Freshwater-Lake
ERR1582166	MGYA00352403	https://www.ebi.ac.uk/metagenomics/api/v1/analyses/MGYA00352403/file/ERR1582166_FASTQ_SSU_OTU.tsv	Root-Environmental-Aquatic-Freshwater-Lake
ERR1583081	MGYA00353318	https://www.ebi.ac.uk/metagenomics/api/v1/analyses/MGYA00353318/file/ERR1583081_FASTQ_SSU_OTU.tsv	Root-Environmental-Aquatic-Freshwater-Lake
SRR7081150	MGYA00413201	https://www.ebi.ac.uk/metagenomics/api/v1/analyses/MGYA00413201/file/SRR7081150_MERGED_FASTQ_SSU_OTU.tsv	Root-Environmental-Aquatic-Freshwater-Lake
ERR1583347	MGYA00353584	https://www.ebi.ac.uk/metagenomics/api/v1/analyses/MGYA00353584/file/ERR1583347_FASTQ_SSU_OTU.tsv	Root-Environmental-Aquatic-Freshwater-Lake
ERR1582975	MGYA00353212	https://www.ebi.ac.uk/metagenomics/api/v1/analyses/MGYA00353212/file/ERR1582975_FASTQ_SSU_OTU.tsv	Root-Environmental-Aquatic-Freshwater-Lake
ERR1582507	MGYA00352744	https://www.ebi.ac.uk/metagenomics/api/v1/analyses/MGYA00352744/file/ERR1582507_FASTQ_SSU_OTU.tsv	Root-Environmental-Aquatic-Freshwater-Lake
ERR1583141	MGYA00353378	https://www.ebi.ac.uk/metagenomics/api/v1/analyses/MGYA00353378/file/ERR1583141_FASTQ_SSU_OTU.tsv	Root-Environmental-Aquatic-Freshwater-Lake

ERR1582701	MGYA00352938	https://www.ebi.ac.uk/metagenomics/api/v1/analyses/MGYA00352938/file/ERR1582701_FASTQ_SSU_OTU.tsv	Root-Environmental-Aquatic-Freshwater-Lake
SRR7081356	MGYA00423818	https://www.ebi.ac.uk/metagenomics/api/v1/analyses/MGYA00423818/file/SRR7081356_MERGED_FASTQ_SSU_OTU.tsv	Root-Environmental-Aquatic-Freshwater-Lake
ERR1582360	MGYA00352597	https://www.ebi.ac.uk/metagenomics/api/v1/analyses/MGYA00352597/file/ERR1582360_FASTQ_SSU_OTU.tsv	Root-Environmental-Aquatic-Freshwater-Lake
ERR1429245	MGYA00107643	https://www.ebi.ac.uk/metagenomics/api/v1/analyses/MGYA00107643/file/ERR1429245_FASTQ_otu.tsv	Root-Environmental-Aquatic-Freshwater-Lake
ERR658199	MGYA00103509	https://www.ebi.ac.uk/metagenomics/api/v1/analyses/MGYA00103509/file/ERR658199_FASTQ_otu.tsv	Root-Environmental-Aquatic-Freshwater-Lake
ERR1583520	MGYA00353757	https://www.ebi.ac.uk/metagenomics/api/v1/analyses/MGYA00353757/file/ERR1583520_FASTQ_SSU_OTU.tsv	Root-Environmental-Aquatic-Freshwater-Lake
ERR1583287	MGYA00353524	https://www.ebi.ac.uk/metagenomics/api/v1/analyses/MGYA00353524/file/ERR1583287_FASTQ_SSU_OTU.tsv	Root-Environmental-Aquatic-Freshwater-Lake
ERR1742281	MGYA00311582	https://www.ebi.ac.uk/metagenomics/api/v1/analyses/MGYA00311582/file/ERR1742281_FASTQ_SSU_OTU.tsv	Root-Environmental-Aquatic-Freshwater-Lake
ERR1535976	MGYA00320267	https://www.ebi.ac.uk/metagenomics/api/v1/analyses/MGYA00320267/file/ERR1535976_FASTQ_SSU_OTU.tsv	Root-Environmental-Aquatic-Freshwater-Lake
ERR1333521	MGYA00107609	https://www.ebi.ac.uk/metagenomics/api/v1/analyses/MGYA00107609/file/ERR1333521_FASTQ_otu.tsv	Root-Environmental-Aquatic-Freshwater-Lake
ERR1583256	MGYA00353493	https://www.ebi.ac.uk/metagenomics/api/v1/analyses/MGYA00353493/file/ERR1583256_FASTQ_SSU_OTU.tsv	Root-Environmental-Aquatic-Freshwater-Lake
ERR1582416	MGYA00352653	https://www.ebi.ac.uk/metagenomics/api/v1/analyses/MGYA00352653/file/ERR1582416_FASTQ_SSU_OTU.tsv	Root-Environmental-Aquatic-Freshwater-Lake

ERR1582864	MGYA00353101	https://www.ebi.ac.uk/metagenomics/api/v1/analyses/MGYA00353101/file/ERR1582864_FASTQ_SSU_OTU.tsv	Root-Environmental-Aquatic-Freshwater-Lake
SRR7081387	MGYA00425684	https://www.ebi.ac.uk/metagenomics/api/v1/analyses/MGYA00425684/file/SRR7081387_MERGED_FASTQ_SSU_OTU.tsv	Root-Environmental-Aquatic-Freshwater-Lake
ERR1896809	MGYA00350754	https://www.ebi.ac.uk/metagenomics/api/v1/analyses/MGYA00350754/file/ERR1896809_FASTQ_SSU_OTU.tsv	Root-Environmental-Aquatic-Freshwater-Lake
ERR1582077	MGYA00352314	https://www.ebi.ac.uk/metagenomics/api/v1/analyses/MGYA00352314/file/ERR1582077_FASTQ_SSU_OTU.tsv	Root-Environmental-Aquatic-Freshwater-Lake
ERR1583190	MGYA00353427	https://www.ebi.ac.uk/metagenomics/api/v1/analyses/MGYA00353427/file/ERR1583190_FASTQ_SSU_OTU.tsv	Root-Environmental-Aquatic-Freshwater-Lake
SRR2443046	MGYA00359050	https://www.ebi.ac.uk/metagenomics/api/v1/analyses/MGYA00359050/file/SRR2443046_MERGED_FASTQ_SSU_OTU.tsv	Root-Environmental-Aquatic-Freshwater-Lake
ERR1897702	MGYA00351647	https://www.ebi.ac.uk/metagenomics/api/v1/analyses/MGYA00351647/file/ERR1897702_FASTQ_SSU_OTU.tsv	Root-Environmental-Aquatic-Freshwater-Lake
ERR1582688	MGYA00352925	https://www.ebi.ac.uk/metagenomics/api/v1/analyses/MGYA00352925/file/ERR1582688_FASTQ_SSU_OTU.tsv	Root-Environmental-Aquatic-Freshwater-Lake
ERR1897363	MGYA00351308	https://www.ebi.ac.uk/metagenomics/api/v1/analyses/MGYA00351308/file/ERR1897363_FASTQ_SSU_OTU.tsv	Root-Environmental-Aquatic-Freshwater-Lake
ERR589316	MGYA00103438	https://www.ebi.ac.uk/metagenomics/api/v1/analyses/MGYA00103438/file/ERR589316_FASTQ_otu.tsv	Root-Environmental-Aquatic-Freshwater-Lake
ERR1896951	MGYA00350896	https://www.ebi.ac.uk/metagenomics/api/v1/analyses/MGYA00350896/file/ERR1896951_FASTQ_SSU_OTU.tsv	Root-Environmental-Aquatic-Freshwater-Lake
ERR1896980	MGYA00350925	https://www.ebi.ac.uk/metagenomics/api/v1/analyses/MGYA00350925/file/ERR1896980_FASTQ_SSU_OTU.tsv	Root-Environmental-Aquatic-Freshwater-Lake

SRR2442963	MGYA00358967	https://www.ebi.ac.uk/metagenomics/api/v1/analyses/MGYA00358967/file/SRR2442963_MERGED_FASTQ_SSU_OTU.tsv	Root-Environmental-Aquatic-Freshwater-Lake
ERR1743366	MGYA00087124	https://www.ebi.ac.uk/metagenomics/api/v1/analyses/MGYA00087124/file/ERR1743366_MERGED_FASTQ_otu.tsv	Root-Environmental-Aquatic-Freshwater-Lake
ERR1897415	MGYA00351360	https://www.ebi.ac.uk/metagenomics/api/v1/analyses/MGYA00351360/file/ERR1897415_FASTQ_SSU_OTU.tsv	Root-Environmental-Aquatic-Freshwater-Lake
ERR1583019	MGYA00353256	https://www.ebi.ac.uk/metagenomics/api/v1/analyses/MGYA00353256/file/ERR1583019_FASTQ_SSU_OTU.tsv	Root-Environmental-Aquatic-Freshwater-Lake
ERR1582659	MGYA00352896	https://www.ebi.ac.uk/metagenomics/api/v1/analyses/MGYA00352896/file/ERR1582659_FASTQ_SSU_OTU.tsv	Root-Environmental-Aquatic-Freshwater-Lake
ERR1897074	MGYA00351019	https://www.ebi.ac.uk/metagenomics/api/v1/analyses/MGYA00351019/file/ERR1897074_FASTQ_SSU_OTU.tsv	Root-Environmental-Aquatic-Freshwater-Lake
ERR1582238	MGYA00352475	https://www.ebi.ac.uk/metagenomics/api/v1/analyses/MGYA00352475/file/ERR1582238_FASTQ_SSU_OTU.tsv	Root-Environmental-Aquatic-Freshwater-Lake
ERR1583478	MGYA00353715	https://www.ebi.ac.uk/metagenomics/api/v1/analyses/MGYA00353715/file/ERR1583478_FASTQ_SSU_OTU.tsv	Root-Environmental-Aquatic-Freshwater-Lake
ERR2868524	MGYA00377406	https://www.ebi.ac.uk/metagenomics/api/v1/analyses/MGYA00377406/file/ERR2868524_FASTQ_SSU_OTU.tsv	Root-Environmental-Aquatic-Freshwater-Lake
ERR1897531	MGYA00351476	https://www.ebi.ac.uk/metagenomics/api/v1/analyses/MGYA00351476/file/ERR1897531_FASTQ_SSU_OTU.tsv	Root-Environmental-Aquatic-Freshwater-Lake
ERR1897296	MGYA00351241	https://www.ebi.ac.uk/metagenomics/api/v1/analyses/MGYA00351241/file/ERR1897296_FASTQ_SSU_OTU.tsv	Root-Environmental-Aquatic-Freshwater-Lake
ERR1897150	MGYA00351095	https://www.ebi.ac.uk/metagenomics/api/v1/analyses/MGYA00351095/file/ERR1897150_FASTQ_SSU_OTU.tsv	Root-Environmental-Aquatic-Freshwater-Lake

ERR1896710	MGYA00350655	https://www.ebi.ac.uk/metagenomics/api/v1/analyses/MGYA00350655/file/ERR1896710_FASTQ_SSU_OTU.tsv	Root-Environmental-Aquatic-Freshwater-Lake
ERR1990500	MGYA00128204	https://www.ebi.ac.uk/metagenomics/api/v1/analyses/MGYA00128204/file/ERR1990500_MERGED_FASTQ_otu.tsv	Root-Environmental-Aquatic-Freshwater-Lake
ERR1429239	MGYA00107621	https://www.ebi.ac.uk/metagenomics/api/v1/analyses/MGYA00107621/file/ERR1429239_FASTQ_otu.tsv	Root-Environmental-Aquatic-Freshwater-Lake
ERR1582818	MGYA00353055	https://www.ebi.ac.uk/metagenomics/api/v1/analyses/MGYA00353055/file/ERR1582818_FASTQ_SSU_OTU.tsv	Root-Environmental-Aquatic-Freshwater-Lake
ERR1897181	MGYA00351126	https://www.ebi.ac.uk/metagenomics/api/v1/analyses/MGYA00351126/file/ERR1897181_FASTQ_SSU_OTU.tsv	Root-Environmental-Aquatic-Freshwater-Lake
ERR1897626	MGYA00351571	https://www.ebi.ac.uk/metagenomics/api/v1/analyses/MGYA00351571/file/ERR1897626_FASTQ_SSU_OTU.tsv	Root-Environmental-Aquatic-Freshwater-Lake
ERR1897247	MGYA00351192	https://www.ebi.ac.uk/metagenomics/api/v1/analyses/MGYA00351192/file/ERR1897247_FASTQ_SSU_OTU.tsv	Root-Environmental-Aquatic-Freshwater-Lake
ERR1743293	MGYA00087132	https://www.ebi.ac.uk/metagenomics/api/v1/analyses/MGYA00087132/file/ERR1743293_MERGED_FASTQ_otu.tsv	Root-Environmental-Aquatic-Freshwater-Lake
ERR1896875	MGYA00350820	https://www.ebi.ac.uk/metagenomics/api/v1/analyses/MGYA00350820/file/ERR1896875_FASTQ_SSU_OTU.tsv	Root-Environmental-Aquatic-Freshwater-Lake
ERR1896699	MGYA00350644	https://www.ebi.ac.uk/metagenomics/api/v1/analyses/MGYA00350644/file/ERR1896699_FASTQ_SSU_OTU.tsv	Root-Environmental-Aquatic-Freshwater-Lake
ERR1583372	MGYA00353609	https://www.ebi.ac.uk/metagenomics/api/v1/analyses/MGYA00353609/file/ERR1583372_FASTQ_SSU_OTU.tsv	Root-Environmental-Aquatic-Freshwater-Lake
ERR1582940	MGYA00353177	https://www.ebi.ac.uk/metagenomics/api/v1/analyses/MGYA00353177/file/ERR1582940_FASTQ_SSU_OTU.tsv	Root-Environmental-Aquatic-Freshwater-Lake

ERR1582532	MGYA00352769	https://www.ebi.ac.uk/metagenomics/api/v1/analyses/MGYA00352769/file/ERR1582532_FASTQ_SSU_OTU.tsv	Root-Environmental-Aquatic-Freshwater-Lake
ERR1990489	MGYA00128207	https://www.ebi.ac.uk/metagenomics/api/v1/analyses/MGYA00128207/file/ERR1990489_MERGED_FASTQ_otu.tsv	Root-Environmental-Aquatic-Freshwater-Lake
ERR1582295	MGYA00352532	https://www.ebi.ac.uk/metagenomics/api/v1/analyses/MGYA00352532/file/ERR1582295_FASTQ_SSU_OTU.tsv	Root-Environmental-Aquatic-Freshwater-Lake
ERR1582153	MGYA00352390	https://www.ebi.ac.uk/metagenomics/api/v1/analyses/MGYA00352390/file/ERR1582153_FASTQ_SSU_OTU.tsv	Root-Environmental-Aquatic-Freshwater-Lake
ERR1583065	MGYA00353302	https://www.ebi.ac.uk/metagenomics/api/v1/analyses/MGYA00353302/file/ERR1583065_FASTQ_SSU_OTU.tsv	Root-Environmental-Aquatic-Freshwater-Lake
ERR1582182	MGYA00352419	https://www.ebi.ac.uk/metagenomics/api/v1/analyses/MGYA00352419/file/ERR1582182_FASTQ_SSU_OTU.tsv	Root-Environmental-Aquatic-Freshwater-Lake
ERR1582625	MGYA00352862	https://www.ebi.ac.uk/metagenomics/api/v1/analyses/MGYA00352862/file/ERR1582625_FASTQ_SSU_OTU.tsv	Root-Environmental-Aquatic-Freshwater-Lake
ERR1547125	MGYA00320836	https://www.ebi.ac.uk/metagenomics/api/v1/analyses/MGYA00320836/file/ERR1547125_FASTQ_SSU_OTU.tsv	Root-Environmental-Aquatic-Freshwater-Lake
ERR1897469	MGYA00351414	https://www.ebi.ac.uk/metagenomics/api/v1/analyses/MGYA00351414/file/ERR1897469_FASTQ_SSU_OTU.tsv	Root-Environmental-Aquatic-Freshwater-Lake
ERR1582991	MGYA00353228	https://www.ebi.ac.uk/metagenomics/api/v1/analyses/MGYA00353228/file/ERR1582991_FASTQ_SSU_OTU.tsv	Root-Environmental-Aquatic-Freshwater-Lake
ERR1582244	MGYA00352481	https://www.ebi.ac.uk/metagenomics/api/v1/analyses/MGYA00352481/file/ERR1582244_FASTQ_SSU_OTU.tsv	Root-Environmental-Aquatic-Freshwater-Lake
ERR1583404	MGYA00353641	https://www.ebi.ac.uk/metagenomics/api/v1/analyses/MGYA00353641/file/ERR1583404_FASTQ_SSU_OTU.tsv	Root-Environmental-Aquatic-Freshwater-Lake

ERR1897008	MGYA00350953	https://www.ebi.ac.uk/metagenomics/api/v1/analyses/MGYA00350953/file/ERR1897008_FASTQ_SSU_OTU.tsv	Root-Environmental-Aquatic-Freshwater-Lake
ERR1896648	MGYA00350593	https://www.ebi.ac.uk/metagenomics/api/v1/analyses/MGYA00350593/file/ERR1896648_FASTQ_SSU_OTU.tsv	Root-Environmental-Aquatic-Freshwater-Lake
ERR1897291	MGYA00351236	https://www.ebi.ac.uk/metagenomics/api/v1/analyses/MGYA00351236/file/ERR1897291_FASTQ_SSU_OTU.tsv	Root-Environmental-Aquatic-Freshwater-Lake
ERR1897536	MGYA00351481	https://www.ebi.ac.uk/metagenomics/api/v1/analyses/MGYA00351481/file/ERR1897536_FASTQ_SSU_OTU.tsv	Root-Environmental-Aquatic-Freshwater-Lake
ERR2868523	MGYA00377405	https://www.ebi.ac.uk/metagenomics/api/v1/analyses/MGYA00377405/file/ERR2868523_FASTQ_SSU_OTU.tsv	Root-Environmental-Aquatic-Freshwater-Lake
ERR1896717	MGYA00350662	https://www.ebi.ac.uk/metagenomics/api/v1/analyses/MGYA00350662/file/ERR1896717_FASTQ_SSU_OTU.tsv	Root-Environmental-Aquatic-Freshwater-Lake
ERR1897157	MGYA00351102	https://www.ebi.ac.uk/metagenomics/api/v1/analyses/MGYA00351102/file/ERR1897157_FASTQ_SSU_OTU.tsv	Root-Environmental-Aquatic-Freshwater-Lake
ERR1897621	MGYA00351566	https://www.ebi.ac.uk/metagenomics/api/v1/analyses/MGYA00351566/file/ERR1897621_FASTQ_SSU_OTU.tsv	Root-Environmental-Aquatic-Freshwater-Lake
ERR1897186	MGYA00351131	https://www.ebi.ac.uk/metagenomics/api/v1/analyses/MGYA00351131/file/ERR1897186_FASTQ_SSU_OTU.tsv	Root-Environmental-Aquatic-Freshwater-Lake
ERR1896872	MGYA00350817	https://www.ebi.ac.uk/metagenomics/api/v1/analyses/MGYA00350817/file/ERR1896872_FASTQ_SSU_OTU.tsv	Root-Environmental-Aquatic-Freshwater-Lake
ERR1743294	MGYA00087104	https://www.ebi.ac.uk/metagenomics/api/v1/analyses/MGYA00087104/file/ERR1743294_MERGED_FASTQ_otu.tsv	Root-Environmental-Aquatic-Freshwater-Lake
ERR1897240	MGYA00351185	https://www.ebi.ac.uk/metagenomics/api/v1/analyses/MGYA00351185/file/ERR1897240_FASTQ_SSU_OTU.tsv	Root-Environmental-Aquatic-Freshwater-Lake

ERR1582292	MGYA00352529	https://www.ebi.ac.uk/metagenomics/api/v1/analyses/MGYA00352529/file/ERR1582292_FASTQ_SSU_OTU.tsv	Root-Environmental-Aquatic-Freshwater-Lake
ERR1582535	MGYA00352772	https://www.ebi.ac.uk/metagenomics/api/v1/analyses/MGYA00352772/file/ERR1582535_FASTQ_SSU_OTU.tsv	Root-Environmental-Aquatic-Freshwater-Lake
ERR1582947	MGYA00353184	https://www.ebi.ac.uk/metagenomics/api/v1/analyses/MGYA00353184/file/ERR1582947_FASTQ_SSU_OTU.tsv	Root-Environmental-Aquatic-Freshwater-Lake
ERR1583375	MGYA00353612	https://www.ebi.ac.uk/metagenomics/api/v1/analyses/MGYA00353612/file/ERR1583375_FASTQ_SSU_OTU.tsv	Root-Environmental-Aquatic-Freshwater-Lake
ERR1897318	MGYA00351263	https://www.ebi.ac.uk/metagenomics/api/v1/analyses/MGYA00351263/file/ERR1897318_FASTQ_SSU_OTU.tsv	Root-Environmental-Aquatic-Freshwater-Lake
ERR1582154	MGYA00352391	https://www.ebi.ac.uk/metagenomics/api/v1/analyses/MGYA00352391/file/ERR1582154_FASTQ_SSU_OTU.tsv	Root-Environmental-Aquatic-Freshwater-Lake
ERR1582622	MGYA00352859	https://www.ebi.ac.uk/metagenomics/api/v1/analyses/MGYA00352859/file/ERR1582622_FASTQ_SSU_OTU.tsv	Root-Environmental-Aquatic-Freshwater-Lake
ERR1582185	MGYA00352422	https://www.ebi.ac.uk/metagenomics/api/v1/analyses/MGYA00352422/file/ERR1582185_FASTQ_SSU_OTU.tsv	Root-Environmental-Aquatic-Freshwater-Lake
ERR1583062	MGYA00353299	https://www.ebi.ac.uk/metagenomics/api/v1/analyses/MGYA00353299/file/ERR1583062_FASTQ_SSU_OTU.tsv	Root-Environmental-Aquatic-Freshwater-Lake
ERR1547122	MGYA00320833	https://www.ebi.ac.uk/metagenomics/api/v1/analyses/MGYA00320833/file/ERR1547122_FASTQ_SSU_OTU.tsv	Root-Environmental-Aquatic-Freshwater-Lake
ERR1583403	MGYA00353640	https://www.ebi.ac.uk/metagenomics/api/v1/analyses/MGYA00353640/file/ERR1583403_FASTQ_SSU_OTU.tsv	Root-Environmental-Aquatic-Freshwater-Lake
ERR1582243	MGYA00352480	https://www.ebi.ac.uk/metagenomics/api/v1/analyses/MGYA00352480/file/ERR1582243_FASTQ_SSU_OTU.tsv	Root-Environmental-Aquatic-Freshwater-Lake

ERR1582996	MGYA00353233	https://www.ebi.ac.uk/metagenomics/api/v1/analyses/MGYA00353233/file/ERR1582996_FASTQ_SSU_OTU.tsv	Root-Environmental-Aquatic-Freshwater-Lake
ERR430930	MGYA00103252	https://www.ebi.ac.uk/metagenomics/api/v1/analyses/MGYA00103252/file/ERR430930_FASTQ_otu.tsv	Root-Environmental-Aquatic-Freshwater-Lake
ERR1582706	MGYA00352943	https://www.ebi.ac.uk/metagenomics/api/v1/analyses/MGYA00352943/file/ERR1582706_FASTQ_SSU_OTU.tsv	Root-Environmental-Aquatic-Freshwater-Lake
ERR1583146	MGYA00353383	https://www.ebi.ac.uk/metagenomics/api/v1/analyses/MGYA00353383/file/ERR1583146_FASTQ_SSU_OTU.tsv	Root-Environmental-Aquatic-Freshwater-Lake
ERR589298	MGYA00103421	https://www.ebi.ac.uk/metagenomics/api/v1/analyses/MGYA00103421/file/ERR589298_FASTQ_otu.tsv	Root-Environmental-Aquatic-Freshwater-Lake
ERR1583280	MGYA00353517	https://www.ebi.ac.uk/metagenomics/api/v1/analyses/MGYA00353517/file/ERR1583280_FASTQ_SSU_OTU.tsv	Root-Environmental-Aquatic-Freshwater-Lake
ERR1429242	MGYA00107668	https://www.ebi.ac.uk/metagenomics/api/v1/analyses/MGYA00107668/file/ERR1429242_FASTQ_otu.tsv	Root-Environmental-Aquatic-Freshwater-Lake
ERR1582367	MGYA00352604	https://www.ebi.ac.uk/metagenomics/api/v1/analyses/MGYA00352604/file/ERR1582367_FASTQ_SSU_OTU.tsv	Root-Environmental-Aquatic-Freshwater-Lake
ERR1535971	MGYA00320262	https://www.ebi.ac.uk/metagenomics/api/v1/analyses/MGYA00320262/file/ERR1535971_FASTQ_SSU_OTU.tsv	Root-Environmental-Aquatic-Freshwater-Lake
ERR1742286	MGYA00311587	https://www.ebi.ac.uk/metagenomics/api/v1/analyses/MGYA00311587/file/ERR1742286_FASTQ_SSU_OTU.tsv	Root-Environmental-Aquatic-Freshwater-Lake
ERR1582863	MGYA00353100	https://www.ebi.ac.uk/metagenomics/api/v1/analyses/MGYA00353100/file/ERR1582863_FASTQ_SSU_OTU.tsv	Root-Environmental-Aquatic-Freshwater-Lake
ERR1582411	MGYA00352648	https://www.ebi.ac.uk/metagenomics/api/v1/analyses/MGYA00352648/file/ERR1582411_FASTQ_SSU_OTU.tsv	Root-Environmental-Aquatic-Freshwater-Lake

ERR1583251	MGYA00353488	https://www.ebi.ac.uk/metagenomics/api/v1/analyses/MGYA00353488/file/ERR1583251_FASTQ_SSU_OTU.tsv	Root-Environmental-Aquatic-Freshwater-Lake
ERR1583197	MGYA00353434	https://www.ebi.ac.uk/metagenomics/api/v1/analyses/MGYA00353434/file/ERR1583197_FASTQ_SSU_OTU.tsv	Root-Environmental-Aquatic-Freshwater-Lake
ERR1582070	MGYA00352307	https://www.ebi.ac.uk/metagenomics/api/v1/analyses/MGYA00352307/file/ERR1582070_FASTQ_SSU_OTU.tsv	Root-Environmental-Aquatic-Freshwater-Lake
ERR1582549	MGYA00352786	https://www.ebi.ac.uk/metagenomics/api/v1/analyses/MGYA00352786/file/ERR1582549_FASTQ_SSU_OTU.tsv	Root-Environmental-Aquatic-Freshwater-Lake
ERR1583309	MGYA00353546	https://www.ebi.ac.uk/metagenomics/api/v1/analyses/MGYA00353546/file/ERR1583309_FASTQ_SSU_OTU.tsv	Root-Environmental-Aquatic-Freshwater-Lake
SRR2443041	MGYA00359045	https://www.ebi.ac.uk/metagenomics/api/v1/analyses/MGYA00359045/file/SRR2443041_MERGED_FASTQ_SSU_OTU.tsv	Root-Environmental-Aquatic-Freshwater-Lake
ERR1897705	MGYA00351650	https://www.ebi.ac.uk/metagenomics/api/v1/analyses/MGYA00351650/file/ERR1897705_FASTQ_SSU_OTU.tsv	Root-Environmental-Aquatic-Freshwater-Lake
ERR1582128	MGYA00352365	https://www.ebi.ac.uk/metagenomics/api/v1/analyses/MGYA00352365/file/ERR1582128_FASTQ_SSU_OTU.tsv	Root-Environmental-Aquatic-Freshwater-Lake
ERR1896956	MGYA00350901	https://www.ebi.ac.uk/metagenomics/api/v1/analyses/MGYA00350901/file/ERR1896956_FASTQ_SSU_OTU.tsv	Root-Environmental-Aquatic-Freshwater-Lake
ERR589311	MGYA00103432	https://www.ebi.ac.uk/metagenomics/api/v1/analyses/MGYA00103432/file/ERR589311_FASTQ_otu.tsv	Root-Environmental-Aquatic-Freshwater-Lake
ERR1897364	MGYA00351309	https://www.ebi.ac.uk/metagenomics/api/v1/analyses/MGYA00351309/file/ERR1897364_FASTQ_SSU_OTU.tsv	Root-Environmental-Aquatic-Freshwater-Lake
ERR1897412	MGYA00351357	https://www.ebi.ac.uk/metagenomics/api/v1/analyses/MGYA00351357/file/ERR1897412_FASTQ_SSU_OTU.tsv	Root-Environmental-Aquatic-Freshwater-Lake

ERR1743361	MGYA00087159	https://www.ebi.ac.uk/metagenomics/api/v1/analyses/MGYA00087159/file/ERR1743361_MERGED_FASTQ_otu.tsv	Root-Environmental-Aquatic-Freshwater-Lake
SRR2442964	MGYA00358968	https://www.ebi.ac.uk/metagenomics/api/v1/analyses/MGYA00358968/file/SRR2442964_MERGED_FASTQ_SSU_OTU.tsv	Root-Environmental-Aquatic-Freshwater-Lake
ERR1896987	MGYA00350932	https://www.ebi.ac.uk/metagenomics/api/v1/analyses/MGYA00350932/file/ERR1896987_FASTQ_SSU_OTU.tsv	Root-Environmental-Aquatic-Freshwater-Lake
ERR1897073	MGYA00351018	https://www.ebi.ac.uk/metagenomics/api/v1/analyses/MGYA00351018/file/ERR1897073_FASTQ_SSU_OTU.tsv	Root-Environmental-Aquatic-Freshwater-Lake
ERR1582598	MGYA00352835	https://www.ebi.ac.uk/metagenomics/api/v1/analyses/MGYA00352835/file/ERR1582598_FASTQ_SSU_OTU.tsv	Root-Environmental-Aquatic-Freshwater-Lake
ERR1583134	MGYA00353371	https://www.ebi.ac.uk/metagenomics/api/v1/analyses/MGYA00353371/file/ERR1583134_FASTQ_SSU_OTU.tsv	Root-Environmental-Aquatic-Freshwater-Lake
ERR1582774	MGYA00353011	https://www.ebi.ac.uk/metagenomics/api/v1/analyses/MGYA00353011/file/ERR1582774_FASTQ_SSU_OTU.tsv	Root-Environmental-Aquatic-Freshwater-Lake
ERR1547074	MGYA00320785	https://www.ebi.ac.uk/metagenomics/api/v1/analyses/MGYA00320785/file/ERR1547074_FASTQ_SSU_OTU.tsv	Root-Environmental-Aquatic-Freshwater-Lake
SRR7081323	MGYA00424203	https://www.ebi.ac.uk/metagenomics/api/v1/analyses/MGYA00424203/file/SRR7081323_MERGED_FASTQ_SSU_OTU.tsv	Root-Environmental-Aquatic-Freshwater-Lake
ERR1897538	MGYA00351483	https://www.ebi.ac.uk/metagenomics/api/v1/analyses/MGYA00351483/file/ERR1897538_FASTQ_SSU_OTU.tsv	Root-Environmental-Aquatic-Freshwater-Lake
ERR1582315	MGYA00352552	https://www.ebi.ac.uk/metagenomics/api/v1/analyses/MGYA00352552/file/ERR1582315_FASTQ_SSU_OTU.tsv	Root-Environmental-Aquatic-Freshwater-Lake
ERR1429230	MGYA00107675	https://www.ebi.ac.uk/metagenomics/api/v1/analyses/MGYA00107675/file/ERR1429230_FASTQ_otu.tsv	Root-Environmental-Aquatic-Freshwater-Lake

ERR1897159	MGYA00351104	https://www.ebi.ac.uk/metagenomics/api/v1/analyses/MGYA00351104/file/ERR1897159_FASTQ_SSU_OTU.tsv	Root-Environmental-Aquatic-Freshwater-Lake
ERR1896719	MGYA00350664	https://www.ebi.ac.uk/metagenomics/api/v1/analyses/MGYA00350664/file/ERR1896719_FASTQ_SSU_OTU.tsv	Root-Environmental-Aquatic-Freshwater-Lake
ERR1897188	MGYA00351133	https://www.ebi.ac.uk/metagenomics/api/v1/analyses/MGYA00351133/file/ERR1897188_FASTQ_SSU_OTU.tsv	Root-Environmental-Aquatic-Freshwater-Lake
ERR1583223	MGYA00353460	https://www.ebi.ac.uk/metagenomics/api/v1/analyses/MGYA00353460/file/ERR1583223_FASTQ_SSU_OTU.tsv	Root-Environmental-Aquatic-Freshwater-Lake
ERR1582811	MGYA00353048	https://www.ebi.ac.uk/metagenomics/api/v1/analyses/MGYA00353048/file/ERR1582811_FASTQ_SSU_OTU.tsv	Root-Environmental-Aquatic-Freshwater-Lake
ERR1582463	MGYA00352700	https://www.ebi.ac.uk/metagenomics/api/v1/analyses/MGYA00352700/file/ERR1582463_FASTQ_SSU_OTU.tsv	Root-Environmental-Aquatic-Freshwater-Lake
ERR1314362	MGYA00095212	https://www.ebi.ac.uk/metagenomics/api/v1/analyses/MGYA00095212/file/ERR1314362_MERGED_FASTQ_otu.tsv	Root-Environmental-Aquatic-Freshwater-Lake
ERR389723	MGYA00103374	https://www.ebi.ac.uk/metagenomics/api/v1/analyses/MGYA00103374/file/ERR389723_FASTQ_otu.tsv	Root-Environmental-Aquatic-Freshwater-Lake
ERR1582949	MGYA00353186	https://www.ebi.ac.uk/metagenomics/api/v1/analyses/MGYA00353186/file/ERR1582949_FASTQ_SSU_OTU.tsv	Root-Environmental-Aquatic-Freshwater-Lake
ERR1990480	MGYA00128201	https://www.ebi.ac.uk/metagenomics/api/v1/analyses/MGYA00128201/file/ERR1990480_MERGED_FASTQ_otu.tsv	Root-Environmental-Aquatic-Freshwater-Lake
ERR1896690	MGYA00350635	https://www.ebi.ac.uk/metagenomics/api/v1/analyses/MGYA00350635/file/ERR1896690_FASTQ_SSU_OTU.tsv	Root-Environmental-Aquatic-Freshwater-Lake
ERR1897777	MGYA00351722	https://www.ebi.ac.uk/metagenomics/api/v1/analyses/MGYA00351722/file/ERR1897777_FASTQ_SSU_OTU.tsv	Root-Environmental-Aquatic-Freshwater-Lake

SRR2443033	MGYA00359037	https://www.ebi.ac.uk/metagenomics/api/v1/analyses/MGYA00359037/file/SRR2443033_MERGED_FASTQ_SSU_OTU.tsv	Root-Environmental-Aquatic-Freshwater-Lake
ERR1897316	MGYA00351261	https://www.ebi.ac.uk/metagenomics/api/v1/analyses/MGYA00351261/file/ERR1897316_FASTQ_SSU_OTU.tsv	Root-Environmental-Aquatic-Freshwater-Lake
ERR1896924	MGYA00350869	https://www.ebi.ac.uk/metagenomics/api/v1/analyses/MGYA00350869/file/ERR1896924_FASTQ_SSU_OTU.tsv	Root-Environmental-Aquatic-Freshwater-Lake
ERR1897460	MGYA00351405	https://www.ebi.ac.uk/metagenomics/api/v1/analyses/MGYA00351405/file/ERR1897460_FASTQ_SSU_OTU.tsv	Root-Environmental-Aquatic-Freshwater-Lake
ERR1897001	MGYA00350946	https://www.ebi.ac.uk/metagenomics/api/v1/analyses/MGYA00350946/file/ERR1897001_FASTQ_SSU_OTU.tsv	Root-Environmental-Aquatic-Freshwater-Lake
ERR1896641	MGYA00350586	https://www.ebi.ac.uk/metagenomics/api/v1/analyses/MGYA00350586/file/ERR1896641_FASTQ_SSU_OTU.tsv	Root-Environmental-Aquatic-Freshwater-Lake
ERR1582998	MGYA00353235	https://www.ebi.ac.uk/metagenomics/api/v1/analyses/MGYA00353235/file/ERR1582998_FASTQ_SSU_OTU.tsv	Root-Environmental-Aquatic-Freshwater-Lake
ERR589296	MGYA00103409	https://www.ebi.ac.uk/metagenomics/api/v1/analyses/MGYA00103409/file/ERR589296_FASTQ_otu.tsv	Root-Environmental-Aquatic-Freshwater-Lake
ERR1897544	MGYA00351489	https://www.ebi.ac.uk/metagenomics/api/v1/analyses/MGYA00351489/file/ERR1897544_FASTQ_SSU_OTU.tsv	Root-Environmental-Aquatic-Freshwater-Lake
ERR1583148	MGYA00353385	https://www.ebi.ac.uk/metagenomics/api/v1/analyses/MGYA00353385/file/ERR1583148_FASTQ_SSU_OTU.tsv	Root-Environmental-Aquatic-Freshwater-Lake
ERR1437513	MGYA00107957	https://www.ebi.ac.uk/metagenomics/api/v1/analyses/MGYA00107957/file/ERR1437513_FASTQ_otu.tsv	Root-Environmental-Aquatic-Freshwater-Lake
ERR1582708	MGYA00352945	https://www.ebi.ac.uk/metagenomics/api/v1/analyses/MGYA00352945/file/ERR1582708_FASTQ_SSU_OTU.tsv	Root-Environmental-Aquatic-Freshwater-Lake

ERR1897125	MGYA00351070	https://www.ebi.ac.uk/metagenomics/api/v1/analyses/MGYA00351070/file/ERR1897125_FASTQ_SSU_OTU.tsv	Root-Environmental-Aquatic-Freshwater-Lake
ERR1897682	MGYA00351627	https://www.ebi.ac.uk/metagenomics/api/v1/analyses/MGYA00351627/file/ERR1897682_FASTQ_SSU_OTU.tsv	Root-Environmental-Aquatic-Freshwater-Lake
ERR1896765	MGYA00350710	https://www.ebi.ac.uk/metagenomics/api/v1/analyses/MGYA00350710/file/ERR1896765_FASTQ_SSU_OTU.tsv	Root-Environmental-Aquatic-Freshwater-Lake
ERR658190	MGYA00103480	https://www.ebi.ac.uk/metagenomics/api/v1/analyses/MGYA00103480/file/ERR658190_FASTQ_otu.tsv	Root-Environmental-Aquatic-Freshwater-Lake
ERR1582369	MGYA00352606	https://www.ebi.ac.uk/metagenomics/api/v1/analyses/MGYA00352606/file/ERR1582369_FASTQ_SSU_OTU.tsv	Root-Environmental-Aquatic-Freshwater-Lake
ERR1742288	MGYA00311589	https://www.ebi.ac.uk/metagenomics/api/v1/analyses/MGYA00311589/file/ERR1742288_FASTQ_SSU_OTU.tsv	Root-Environmental-Aquatic-Freshwater-Lake
SRR7081048	MGYA00427349	https://www.ebi.ac.uk/metagenomics/api/v1/analyses/MGYA00427349/file/SRR7081048_MERGED_FASTQ_SSU_OTU.tsv	Root-Environmental-Aquatic-Freshwater-Lake
ERR1897653	MGYA00351598	https://www.ebi.ac.uk/metagenomics/api/v1/analyses/MGYA00351598/file/ERR1897653_FASTQ_SSU_OTU.tsv	Root-Environmental-Aquatic-Freshwater-Lake
ERR1583199	MGYA00353436	https://www.ebi.ac.uk/metagenomics/api/v1/analyses/MGYA00353436/file/ERR1583199_FASTQ_SSU_OTU.tsv	Root-Environmental-Aquatic-Freshwater-Lake
ERR1897595	MGYA00351540	https://www.ebi.ac.uk/metagenomics/api/v1/analyses/MGYA00351540/file/ERR1897595_FASTQ_SSU_OTU.tsv	Root-Environmental-Aquatic-Freshwater-Lake
ERR1897232	MGYA00351177	https://www.ebi.ac.uk/metagenomics/api/v1/analyses/MGYA00351177/file/ERR1897232_FASTQ_SSU_OTU.tsv	Root-Environmental-Aquatic-Freshwater-Lake
ERR1896800	MGYA00350745	https://www.ebi.ac.uk/metagenomics/api/v1/analyses/MGYA00350745/file/ERR1896800_FASTQ_SSU_OTU.tsv	Root-Environmental-Aquatic-Freshwater-Lake

ERR1583307	MGYA00353544	https://www.ebi.ac.uk/metagenomics/api/v1/analyses/MGYA00353544/file/ERR1583307_FASTQ_SSU_OTU.tsv	Root-Environmental-Aquatic-Freshwater-Lake
ERR1582547	MGYA00352784	https://www.ebi.ac.uk/metagenomics/api/v1/analyses/MGYA00352784/file/ERR1582547_FASTQ_SSU_OTU.tsv	Root-Environmental-Aquatic-Freshwater-Lake
ERR1582935	MGYA00353172	https://www.ebi.ac.uk/metagenomics/api/v1/analyses/MGYA00353172/file/ERR1582935_FASTQ_SSU_OTU.tsv	Root-Environmental-Aquatic-Freshwater-Lake
ERR1896958	MGYA00350903	https://www.ebi.ac.uk/metagenomics/api/v1/analyses/MGYA00350903/file/ERR1896958_FASTQ_SSU_OTU.tsv	Root-Environmental-Aquatic-Freshwater-Lake
ERR1582126	MGYA00352363	https://www.ebi.ac.uk/metagenomics/api/v1/analyses/MGYA00352363/file/ERR1582126_FASTQ_SSU_OTU.tsv	Root-Environmental-Aquatic-Freshwater-Lake
ERR1582681	MGYA00352918	https://www.ebi.ac.uk/metagenomics/api/v1/analyses/MGYA00352918/file/ERR1582681_FASTQ_SSU_OTU.tsv	Root-Environmental-Aquatic-Freshwater-Lake
ERR1583010	MGYA00353247	https://www.ebi.ac.uk/metagenomics/api/v1/analyses/MGYA00353247/file/ERR1583010_FASTQ_SSU_OTU.tsv	Root-Environmental-Aquatic-Freshwater-Lake
ERR1582650	MGYA00352887	https://www.ebi.ac.uk/metagenomics/api/v1/analyses/MGYA00352887/file/ERR1582650_FASTQ_SSU_OTU.tsv	Root-Environmental-Aquatic-Freshwater-Lake
ERR1896989	MGYA00350934	https://www.ebi.ac.uk/metagenomics/api/v1/analyses/MGYA00350934/file/ERR1896989_FASTQ_SSU_OTU.tsv	Root-Environmental-Aquatic-Freshwater-Lake
SRR7081207	MGYA00425943	https://www.ebi.ac.uk/metagenomics/api/v1/analyses/MGYA00425943/file/SRR7081207_MERGED_FASTQ_SSU_OTU.tsv	Root-Environmental-Aquatic-Freshwater-Lake
ERR1582596	MGYA00352833	https://www.ebi.ac.uk/metagenomics/api/v1/analyses/MGYA00352833/file/ERR1582596_FASTQ_SSU_OTU.tsv	Root-Environmental-Aquatic-Freshwater-Lake
ERR1582231	MGYA00352468	https://www.ebi.ac.uk/metagenomics/api/v1/analyses/MGYA00352468/file/ERR1582231_FASTQ_SSU_OTU.tsv	Root-Environmental-Aquatic-Freshwater-Lake

ERR1583471	MGYA00353708	https://www.ebi.ac.uk/metagenomics/api/v1/analyses/MGYA00353708/file/ERR1583471_FASTQ_SSU_OTU.tsv	Root-Environmental-Aquatic-Freshwater-Lake
SRR7081358	MGYA00423816	https://www.ebi.ac.uk/metagenomics/api/v1/analyses/MGYA00423816/file/SRR7081358_MERGED_FASTQ_SSU_OTU.tsv	Root-Environmental-Aquatic-Freshwater-Lake
ERR1897543	MGYA00351488	https://www.ebi.ac.uk/metagenomics/api/v1/analyses/MGYA00351488/file/ERR1897543_FASTQ_SSU_OTU.tsv	Root-Environmental-Aquatic-Freshwater-Lake
ERR589291	MGYA00103408	https://www.ebi.ac.uk/metagenomics/api/v1/analyses/MGYA00103408/file/ERR589291_FASTQ_otu.tsv	Root-Environmental-Aquatic-Freshwater-Lake
ERR1437514	MGYA00107948	https://www.ebi.ac.uk/metagenomics/api/v1/analyses/MGYA00107948/file/ERR1437514_FASTQ_otu.tsv	Root-Environmental-Aquatic-Freshwater-Lake
ERR1896762	MGYA00350707	https://www.ebi.ac.uk/metagenomics/api/v1/analyses/MGYA00350707/file/ERR1896762_FASTQ_SSU_OTU.tsv	Root-Environmental-Aquatic-Freshwater-Lake
ERR1897685	MGYA00351630	https://www.ebi.ac.uk/metagenomics/api/v1/analyses/MGYA00351630/file/ERR1897685_FASTQ_SSU_OTU.tsv	Root-Environmental-Aquatic-Freshwater-Lake
ERR1897122	MGYA00351067	https://www.ebi.ac.uk/metagenomics/api/v1/analyses/MGYA00351067/file/ERR1897122_FASTQ_SSU_OTU.tsv	Root-Environmental-Aquatic-Freshwater-Lake
ERR1583289	MGYA00353526	https://www.ebi.ac.uk/metagenomics/api/v1/analyses/MGYA00353526/file/ERR1583289_FASTQ_SSU_OTU.tsv	Root-Environmental-Aquatic-Freshwater-Lake
ERR658197	MGYA00103468	https://www.ebi.ac.uk/metagenomics/api/v1/analyses/MGYA00103468/file/ERR658197_FASTQ_otu.tsv	Root-Environmental-Aquatic-Freshwater-Lake
ERR1582418	MGYA00352655	https://www.ebi.ac.uk/metagenomics/api/v1/analyses/MGYA00352655/file/ERR1582418_FASTQ_SSU_OTU.tsv	Root-Environmental-Aquatic-Freshwater-Lake
ERR1583258	MGYA00353495	https://www.ebi.ac.uk/metagenomics/api/v1/analyses/MGYA00353495/file/ERR1583258_FASTQ_SSU_OTU.tsv	Root-Environmental-Aquatic-Freshwater-Lake

ERR1897654	MGYA00351599	https://www.ebi.ac.uk/metagenomics/api/v1/analyses/MGYA00351599/file/ERR1897654_FASTQ_SSU_OTU.tsv	Root-Environmental-Aquatic-Freshwater-Lake
ERR1535978	MGYA00320269	https://www.ebi.ac.uk/metagenomics/api/v1/analyses/MGYA00320269/file/ERR1535978_FASTQ_SSU_OTU.tsv	Root-Environmental-Aquatic-Freshwater-Lake
ERR1582079	MGYA00352316	https://www.ebi.ac.uk/metagenomics/api/v1/analyses/MGYA00352316/file/ERR1582079_FASTQ_SSU_OTU.tsv	Root-Environmental-Aquatic-Freshwater-Lake
ERR1896807	MGYA00350752	https://www.ebi.ac.uk/metagenomics/api/v1/analyses/MGYA00350752/file/ERR1896807_FASTQ_SSU_OTU.tsv	Root-Environmental-Aquatic-Freshwater-Lake
ERR1897235	MGYA00351180	https://www.ebi.ac.uk/metagenomics/api/v1/analyses/MGYA00351180/file/ERR1897235_FASTQ_SSU_OTU.tsv	Root-Environmental-Aquatic-Freshwater-Lake
ERR1897592	MGYA00351537	https://www.ebi.ac.uk/metagenomics/api/v1/analyses/MGYA00351537/file/ERR1897592_FASTQ_SSU_OTU.tsv	Root-Environmental-Aquatic-Freshwater-Lake
ERR1582932	MGYA00353169	https://www.ebi.ac.uk/metagenomics/api/v1/analyses/MGYA00353169/file/ERR1582932_FASTQ_SSU_OTU.tsv	Root-Environmental-Aquatic-Freshwater-Lake
ERR1582540	MGYA00352777	https://www.ebi.ac.uk/metagenomics/api/v1/analyses/MGYA00352777/file/ERR1582540_FASTQ_SSU_OTU.tsv	Root-Environmental-Aquatic-Freshwater-Lake
ERR1583300	MGYA00353537	https://www.ebi.ac.uk/metagenomics/api/v1/analyses/MGYA00353537/file/ERR1583300_FASTQ_SSU_OTU.tsv	Root-Environmental-Aquatic-Freshwater-Lake
ERR589318	MGYA00103434	https://www.ebi.ac.uk/metagenomics/api/v1/analyses/MGYA00103434/file/ERR589318_FASTQ_otu.tsv	Root-Environmental-Aquatic-Freshwater-Lake
ERR1582686	MGYA00352923	https://www.ebi.ac.uk/metagenomics/api/v1/analyses/MGYA00352923/file/ERR1582686_FASTQ_SSU_OTU.tsv	Root-Environmental-Aquatic-Freshwater-Lake
ERR1582121	MGYA00352358	https://www.ebi.ac.uk/metagenomics/api/v1/analyses/MGYA00352358/file/ERR1582121_FASTQ_SSU_OTU.tsv	Root-Environmental-Aquatic-Freshwater-Lake

ERR1582657	MGYA00352894	https://www.ebi.ac.uk/metagenomics/api/v1/analyses/MGYA00352894/file/ERR1582657_FASTQ_SSU_OTU.tsv	Root-Environmental-Aquatic-Freshwater-Lake
ERR1583017	MGYA00353254	https://www.ebi.ac.uk/metagenomics/api/v1/analyses/MGYA00353254/file/ERR1583017_FASTQ_SSU_OTU.tsv	Root-Environmental-Aquatic-Freshwater-Lake
ERR1743368	MGYA00087204	https://www.ebi.ac.uk/metagenomics/api/v1/analyses/MGYA00087204/file/ERR1743368_MERGED_FASTQ_otu.tsv	Root-Environmental-Aquatic-Freshwater-Lake
ERR1583476	MGYA00353713	https://www.ebi.ac.uk/metagenomics/api/v1/analyses/MGYA00353713/file/ERR1583476_FASTQ_SSU_OTU.tsv	Root-Environmental-Aquatic-Freshwater-Lake
ERR1582236	MGYA00352473	https://www.ebi.ac.uk/metagenomics/api/v1/analyses/MGYA00352473/file/ERR1582236_FASTQ_SSU_OTU.tsv	Root-Environmental-Aquatic-Freshwater-Lake
ERR1582591	MGYA00352828	https://www.ebi.ac.uk/metagenomics/api/v1/analyses/MGYA00352828/file/ERR1582591_FASTQ_SSU_OTU.tsv	Root-Environmental-Aquatic-Freshwater-Lake
ERR1582773	MGYA00353010	https://www.ebi.ac.uk/metagenomics/api/v1/analyses/MGYA00353010/file/ERR1582773_FASTQ_SSU_OTU.tsv	Root-Environmental-Aquatic-Freshwater-Lake
ERR1583133	MGYA00353370	https://www.ebi.ac.uk/metagenomics/api/v1/analyses/MGYA00353370/file/ERR1583133_FASTQ_SSU_OTU.tsv	Root-Environmental-Aquatic-Freshwater-Lake
ERR1897298	MGYA00351243	https://www.ebi.ac.uk/metagenomics/api/v1/analyses/MGYA00351243/file/ERR1897298_FASTQ_SSU_OTU.tsv	Root-Environmental-Aquatic-Freshwater-Lake
SRR7081324	MGYA00424816	https://www.ebi.ac.uk/metagenomics/api/v1/analyses/MGYA00424816/file/SRR7081324_MERGED_FASTQ_SSU_OTU.tsv	Root-Environmental-Aquatic-Freshwater-Lake
ERR1547073	MGYA00320784	https://www.ebi.ac.uk/metagenomics/api/v1/analyses/MGYA00320784/file/ERR1547073_FASTQ_SSU_OTU.tsv	Root-Environmental-Aquatic-Freshwater-Lake
ERR1429237	MGYA00107648	https://www.ebi.ac.uk/metagenomics/api/v1/analyses/MGYA00107648/file/ERR1429237_FASTQ_otu.tsv	Root-Environmental-Aquatic-Freshwater-Lake

ERR1582312	MGYA00352549	https://www.ebi.ac.uk/metagenomics/api/v1/analyses/MGYA00352549/file/ERR1582312_FASTQ_SSU_OTU.tsv	Root-Environmental-Aquatic-Freshwater-Lake
SRR7081033	MGYA00427362	https://www.ebi.ac.uk/metagenomics/api/v1/analyses/MGYA00427362/file/SRR7081033_MERGED_FASTQ_SSU_OTU.tsv	Root-Environmental-Aquatic-Freshwater-Lake
ERR1897628	MGYA00351573	https://www.ebi.ac.uk/metagenomics/api/v1/analyses/MGYA00351573/file/ERR1897628_FASTQ_SSU_OTU.tsv	Root-Environmental-Aquatic-Freshwater-Lake
ERR1582464	MGYA00352701	https://www.ebi.ac.uk/metagenomics/api/v1/analyses/MGYA00352701/file/ERR1582464_FASTQ_SSU_OTU.tsv	Root-Environmental-Aquatic-Freshwater-Lake
ERR1582816	MGYA00353053	https://www.ebi.ac.uk/metagenomics/api/v1/analyses/MGYA00353053/file/ERR1582816_FASTQ_SSU_OTU.tsv	Root-Environmental-Aquatic-Freshwater-Lake
ERR1583224	MGYA00353461	https://www.ebi.ac.uk/metagenomics/api/v1/analyses/MGYA00353461/file/ERR1583224_FASTQ_SSU_OTU.tsv	Root-Environmental-Aquatic-Freshwater-Lake
ERR1897249	MGYA00351194	https://www.ebi.ac.uk/metagenomics/api/v1/analyses/MGYA00351194/file/ERR1897249_FASTQ_SSU_OTU.tsv	Root-Environmental-Aquatic-Freshwater-Lake
ERR389724	MGYA00103375	https://www.ebi.ac.uk/metagenomics/api/v1/analyses/MGYA00103375/file/ERR389724_FASTQ_otu.tsv	Root-Environmental-Aquatic-Freshwater-Lake
ERR1314365	MGYA00095151	https://www.ebi.ac.uk/metagenomics/api/v1/analyses/MGYA00095151/file/ERR1314365_MERGED_FASTQ_otu.tsv	Root-Environmental-Aquatic-Freshwater-Lake
ERR1990487	MGYA00128219	https://www.ebi.ac.uk/metagenomics/api/v1/analyses/MGYA00128219/file/ERR1990487_MERGED_FASTQ_otu.tsv	Root-Environmental-Aquatic-Freshwater-Lake
ERR1897770	MGYA00351715	https://www.ebi.ac.uk/metagenomics/api/v1/analyses/MGYA00351715/file/ERR1897770_FASTQ_SSU_OTU.tsv	Root-Environmental-Aquatic-Freshwater-Lake
SRR2443034	MGYA00359038	https://www.ebi.ac.uk/metagenomics/api/v1/analyses/MGYA00359038/file/SRR2443034_MERGED_FASTQ_SSU_OTU.tsv	Root-Environmental-Aquatic-Freshwater-Lake

ERR1896697	MGYA00350642	https://www.ebi.ac.uk/metagenomics/api/v1/analyses/MGYA00350642/file/ERR1896697_FASTQ_SSU_OTU.tsv	Root-Environmental-Aquatic-Freshwater-Lake
ERR1896923	MGYA00350868	https://www.ebi.ac.uk/metagenomics/api/v1/analyses/MGYA00350868/file/ERR1896923_FASTQ_SSU_OTU.tsv	Root-Environmental-Aquatic-Freshwater-Lake
ERR1897311	MGYA00351256	https://www.ebi.ac.uk/metagenomics/api/v1/analyses/MGYA00351256/file/ERR1897311_FASTQ_SSU_OTU.tsv	Root-Environmental-Aquatic-Freshwater-Lake
ERR1897467	MGYA00351412	https://www.ebi.ac.uk/metagenomics/api/v1/analyses/MGYA00351412/file/ERR1897467_FASTQ_SSU_OTU.tsv	Root-Environmental-Aquatic-Freshwater-Lake
ERR1743314	MGYA00087118	https://www.ebi.ac.uk/metagenomics/api/v1/analyses/MGYA00087118/file/ERR1743314_MERGED_FASTQ_otu.tsv	Root-Environmental-Aquatic-Freshwater-Lake
ERR1896646	MGYA00350591	https://www.ebi.ac.uk/metagenomics/api/v1/analyses/MGYA00350591/file/ERR1896646_FASTQ_SSU_OTU.tsv	Root-Environmental-Aquatic-Freshwater-Lake
ERR1897006	MGYA00350951	https://www.ebi.ac.uk/metagenomics/api/v1/analyses/MGYA00350951/file/ERR1897006_FASTQ_SSU_OTU.tsv	Root-Environmental-Aquatic-Freshwater-Lake
SRR5983431	MGYA00153083	https://www.ebi.ac.uk/metagenomics/api/v1/analyses/MGYA00153083/file/SRR5983431_MERGED_FASTQ_SSU_OTU.tsv	Root-Host_associated-Human-Digestive_system-Intestine
SRR5983396	MGYA00152857	https://www.ebi.ac.uk/metagenomics/api/v1/analyses/MGYA00152857/file/SRR5983396_MERGED_FASTQ_SSU_OTU.tsv	Root-Host_associated-Human-Digestive_system-Intestine
ERR2784816	MGYA00215550	https://www.ebi.ac.uk/metagenomics/api/v1/analyses/MGYA00215550/file/ERR2784816_MERGED_FASTQ_SSU_OTU.tsv	Root-Host_associated-Human-Digestive_system-Intestine
SRR5983347	MGYA00153102	https://www.ebi.ac.uk/metagenomics/api/v1/analyses/MGYA00153102/file/SRR5983347_MERGED_FASTQ_SSU_OTU.tsv	Root-Host_associated-Human-Digestive_system-Intestine
ERR2784773	MGYA00215505	https://www.ebi.ac.uk/metagenomics/api/v1/analyses/MGYA00215505/file/ERR2784773_MERGED_FASTQ_SSU_OTU.tsv	Root-Host_associated-Human-Digestive_system-Intestine

ERR2784686	MGYA00215438	https://www.ebi.ac.uk/metagenomics/api/v1/analyses/MGYA00215438/file/ERR2784686_MERGED_FASTQ_SSU_OTU.tsv	Root-Host_associated-Human-Digestive_system-Intestine
SRR5983264	MGYA00153035	https://www.ebi.ac.uk/metagenomics/api/v1/analyses/MGYA00153035/file/SRR5983264_MERGED_FASTQ_SSU_OTU.tsv	Root-Host_associated-Human-Digestive_system-Intestine
ERR2784681	MGYA00215468	https://www.ebi.ac.uk/metagenomics/api/v1/analyses/MGYA00215468/file/ERR2784681_MERGED_FASTQ_SSU_OTU.tsv	Root-Host_associated-Human-Digestive_system-Intestine
ERR2784708	MGYA00215598	https://www.ebi.ac.uk/metagenomics/api/v1/analyses/MGYA00215598/file/ERR2784708_MERGED_FASTQ_SSU_OTU.tsv	Root-Host_associated-Human-Digestive_system-Intestine
SRR5947921	MGYA00153075	https://www.ebi.ac.uk/metagenomics/api/v1/analyses/MGYA00153075/file/SRR5947921_MERGED_FASTQ_SSU_OTU.tsv	Root-Host_associated-Human-Digestive_system-Intestine
ERR2784811	MGYA00215504	https://www.ebi.ac.uk/metagenomics/api/v1/analyses/MGYA00215504/file/ERR2784811_MERGED_FASTQ_SSU_OTU.tsv	Root-Host_associated-Human-Digestive_system-Intestine
SRR5983391	MGYA00152793	https://www.ebi.ac.uk/metagenomics/api/v1/analyses/MGYA00152793/file/SRR5983391_MERGED_FASTQ_SSU_OTU.tsv	Root-Host_associated-Human-Digestive_system-Intestine
SRR5983436	MGYA00152987	https://www.ebi.ac.uk/metagenomics/api/v1/analyses/MGYA00152987/file/SRR5983436_MERGED_FASTQ_SSU_OTU.tsv	Root-Host_associated-Human-Digestive_system-Intestine
SRR5947879	MGYA00152826	https://www.ebi.ac.uk/metagenomics/api/v1/analyses/MGYA00152826/file/SRR5947879_MERGED_FASTQ_SSU_OTU.tsv	Root-Host_associated-Human-Digestive_system-Intestine
SRR5983340	MGYA00152868	https://www.ebi.ac.uk/metagenomics/api/v1/analyses/MGYA00152868/file/SRR5983340_MERGED_FASTQ_SSU_OTU.tsv	Root-Host_associated-Human-Digestive_system-Intestine
ERR2784774	MGYA00215506	https://www.ebi.ac.uk/metagenomics/api/v1/analyses/MGYA00215506/file/ERR2784774_MERGED_FASTQ_SSU_OTU.tsv	Root-Host_associated-Human-Digestive_system-Intestine
SRR5947953	MGYA00152807	https://www.ebi.ac.uk/metagenomics/api/v1/analyses/MGYA00152807/file/SRR5947953_MERGED_FASTQ_SSU_OTU.tsv	Root-Host_associated-Human-Digestive_system-Intestine

SRR5983444	MGYA00152936	https://www.ebi.ac.uk/metagenomics/api/v1/analyses/MGYA00152936/file/SRR5983444_MERGED_FASTQ_SSU_OTU.tsv	Root-Host_associated-Human-Digestive_system-Intestine
SRR5983332	MGYA00152859	https://www.ebi.ac.uk/metagenomics/api/v1/analyses/MGYA00152859/file/SRR5983332_MERGED_FASTQ_SSU_OTU.tsv	Root-Host_associated-Human-Digestive_system-Intestine
ERR2784706	MGYA00215527	https://www.ebi.ac.uk/metagenomics/api/v1/analyses/MGYA00215527/file/ERR2784706_MERGED_FASTQ_SSU_OTU.tsv	Root-Host_associated-Human-Digestive_system-Intestine
SRR5947877	MGYA00152996	https://www.ebi.ac.uk/metagenomics/api/v1/analyses/MGYA00152996/file/SRR5947877_MERGED_FASTQ_SSU_OTU.tsv	Root-Host_associated-Human-Digestive_system-Intestine
SRR5983438	MGYA00153123	https://www.ebi.ac.uk/metagenomics/api/v1/analyses/MGYA00153123/file/SRR5983438_MERGED_FASTQ_SSU_OTU.tsv	Root-Host_associated-Human-Digestive_system-Intestine
SRR5947928	MGYA00153111	https://www.ebi.ac.uk/metagenomics/api/v1/analyses/MGYA00153111/file/SRR5947928_MERGED_FASTQ_SSU_OTU.tsv	Root-Host_associated-Human-Digestive_system-Intestine
SRR5947870	MGYA00152825	https://www.ebi.ac.uk/metagenomics/api/v1/analyses/MGYA00152825/file/SRR5947870_MERGED_FASTQ_SSU_OTU.tsv	Root-Host_associated-Human-Digestive_system-Intestine
ERR2784818	MGYA00215599	https://www.ebi.ac.uk/metagenomics/api/v1/analyses/MGYA00215599/file/ERR2784818_MERGED_FASTQ_SSU_OTU.tsv	Root-Host_associated-Human-Digestive_system-Intestine
SRR5983398	MGYA00153110	https://www.ebi.ac.uk/metagenomics/api/v1/analyses/MGYA00153110/file/SRR5983398_MERGED_FASTQ_SSU_OTU.tsv	Root-Host_associated-Human-Digestive_system-Intestine
SRR5983349	MGYA00152794	https://www.ebi.ac.uk/metagenomics/api/v1/analyses/MGYA00152794/file/SRR5983349_MERGED_FASTQ_SSU_OTU.tsv	Root-Host_associated-Human-Digestive_system-Intestine
ERR2784688	MGYA00215434	https://www.ebi.ac.uk/metagenomics/api/v1/analyses/MGYA00215434/file/ERR2784688_MERGED_FASTQ_SSU_OTU.tsv	Root-Host_associated-Human-Digestive_system-Intestine
SRR5983443	MGYA00153038	https://www.ebi.ac.uk/metagenomics/api/v1/analyses/MGYA00153038/file/SRR5983443_MERGED_FASTQ_SSU_OTU.tsv	Root-Host_associated-Human-Digestive_system-Intestine

SRR5983335	MGYA00152946	https://www.ebi.ac.uk/metagenomics/api/v1/analyses/MGYA00152946/file/SRR5983335_MERGED_FASTQ_SSU_OTU.tsv	Root-Host_associated-Human-Digestive_system-Intestine
ERR2784701	MGYA00215486	https://www.ebi.ac.uk/metagenomics/api/v1/analyses/MGYA00215486/file/ERR2784701_MERGED_FASTQ_SSU_OTU.tsv	Root-Host_associated-Human-Digestive_system-Intestine
ERR2784748	MGYA00215568	https://www.ebi.ac.uk/metagenomics/api/v1/analyses/MGYA00215568/file/ERR2784748_MERGED_FASTQ_SSU_OTU.tsv	Root-Host_associated-Human-Digestive_system-Intestine
SRR5947845	MGYA00152879	https://www.ebi.ac.uk/metagenomics/api/v1/analyses/MGYA00152879/file/SRR5947845_MERGED_FASTQ_SSU_OTU.tsv	Root-Host_associated-Human-Digestive_system-Intestine
ERR2784799	MGYA00215622	https://www.ebi.ac.uk/metagenomics/api/v1/analyses/MGYA00215622/file/ERR2784799_MERGED_FASTQ_SSU_OTU.tsv	Root-Host_associated-Human-Digestive_system-Intestine
SRR5983289	MGYA00152963	https://www.ebi.ac.uk/metagenomics/api/v1/analyses/MGYA00152963/file/SRR5983289_MERGED_FASTQ_SSU_OTU.tsv	Root-Host_associated-Human-Digestive_system-Intestine
ERR2784734	MGYA00215499	https://www.ebi.ac.uk/metagenomics/api/v1/analyses/MGYA00215499/file/ERR2784734_MERGED_FASTQ_SSU_OTU.tsv	Root-Host_associated-Human-Digestive_system-Intestine
SRR5983300	MGYA00152945	https://www.ebi.ac.uk/metagenomics/api/v1/analyses/MGYA00152945/file/SRR5983300_MERGED_FASTQ_SSU_OTU.tsv	Root-Host_associated-Human-Digestive_system-Intestine
ERR2784851	MGYA00215448	https://www.ebi.ac.uk/metagenomics/api/v1/analyses/MGYA00215448/file/ERR2784851_MERGED_FASTQ_SSU_OTU.tsv	Root-Host_associated-Human-Digestive_system-Intestine
SRR5983476	MGYA00152852	https://www.ebi.ac.uk/metagenomics/api/v1/analyses/MGYA00152852/file/SRR5983476_MERGED_FASTQ_SSU_OTU.tsv	Root-Host_associated-Human-Digestive_system-Intestine
SRR5947839	MGYA00153153	https://www.ebi.ac.uk/metagenomics/api/v1/analyses/MGYA00153153/file/SRR5947839_MERGED_FASTQ_SSU_OTU.tsv	Root-Host_associated-Human-Digestive_system-Intestine
ERR2784733	MGYA00215511	https://www.ebi.ac.uk/metagenomics/api/v1/analyses/MGYA00215511/file/ERR2784733_MERGED_FASTQ_SSU_OTU.tsv	Root-Host_associated-Human-Digestive_system-Intestine

SRR5983307	MGYA00152893	https://www.ebi.ac.uk/metagenomics/api/v1/analyses/MGYA00152893/file/SRR5983307_MERGED_FASTQ_SSU_OTU.tsv	Root-Host_associated-Human-Digestive_system-Intestine
SRR5983471	MGYA00152816	https://www.ebi.ac.uk/metagenomics/api/v1/analyses/MGYA00152816/file/SRR5983471_MERGED_FASTQ_SSU_OTU.tsv	Root-Host_associated-Human-Digestive_system-Intestine
SRR5947842	MGYA00153129	https://www.ebi.ac.uk/metagenomics/api/v1/analyses/MGYA00153129/file/SRR5947842_MERGED_FASTQ_SSU_OTU.tsv	Root-Host_associated-Human-Digestive_system-Intestine
SRR5983280	MGYA00153099	https://www.ebi.ac.uk/metagenomics/api/v1/analyses/MGYA00153099/file/SRR5983280_MERGED_FASTQ_SSU_OTU.tsv	Root-Host_associated-Human-Digestive_system-Intestine
SRR5947968	MGYA00152927	https://www.ebi.ac.uk/metagenomics/api/v1/analyses/MGYA00152927/file/SRR5947968_MERGED_FASTQ_SSU_OTU.tsv	Root-Host_associated-Human-Digestive_system-Intestine
ERR2784665	MGYA00215565	https://www.ebi.ac.uk/metagenomics/api/v1/analyses/MGYA00215565/file/ERR2784665_MERGED_FASTQ_SSU_OTU.tsv	Root-Host_associated-Human-Digestive_system-Intestine
SRR5983309	MGYA00152888	https://www.ebi.ac.uk/metagenomics/api/v1/analyses/MGYA00152888/file/SRR5983309_MERGED_FASTQ_SSU_OTU.tsv	Root-Host_associated-Human-Digestive_system-Intestine
SRR5947830	MGYA00152865	https://www.ebi.ac.uk/metagenomics/api/v1/analyses/MGYA00152865/file/SRR5947830_MERGED_FASTQ_SSU_OTU.tsv	Root-Host_associated-Human-Digestive_system-Intestine
SRR5947914	MGYA00153022	https://www.ebi.ac.uk/metagenomics/api/v1/analyses/MGYA00153022/file/SRR5947914_MERGED_FASTQ_SSU_OTU.tsv	Root-Host_associated-Human-Digestive_system-Intestine
ERR2784741	MGYA00215459	https://www.ebi.ac.uk/metagenomics/api/v1/analyses/MGYA00215459/file/ERR2784741_MERGED_FASTQ_SSU_OTU.tsv	Root-Host_associated-Human-Digestive_system-Intestine
SRR5983375	MGYA00153138	https://www.ebi.ac.uk/metagenomics/api/v1/analyses/MGYA00153138/file/SRR5983375_MERGED_FASTQ_SSU_OTU.tsv	Root-Host_associated-Human-Digestive_system-Intestine
ERR2784824	MGYA00215576	https://www.ebi.ac.uk/metagenomics/api/v1/analyses/MGYA00215576/file/ERR2784824_MERGED_FASTQ_SSU_OTU.tsv	Root-Host_associated-Human-Digestive_system-Intestine

SRR5983403	MGYA00153067	https://www.ebi.ac.uk/metagenomics/api/v1/analyses/MGYA00153067/file/SRR5983403_MERGED_FASTQ_SSU_OTU.tsv	Root-Host_associated-Human-Digestive_system-Intestine
ERR2784790	MGYA00215571	https://www.ebi.ac.uk/metagenomics/api/v1/analyses/MGYA00215571/file/ERR2784790_MERGED_FASTQ_SSU_OTU.tsv	Root-Host_associated-Human-Digestive_system-Intestine
SRR5947913	MGYA00152798	https://www.ebi.ac.uk/metagenomics/api/v1/analyses/MGYA00152798/file/SRR5947913_MERGED_FASTQ_SSU_OTU.tsv	Root-Host_associated-Human-Digestive_system-Intestine
ERR2784746	MGYA00215615	https://www.ebi.ac.uk/metagenomics/api/v1/analyses/MGYA00215615/file/ERR2784746_MERGED_FASTQ_SSU_OTU.tsv	Root-Host_associated-Human-Digestive_system-Intestine
SRR5983372	MGYA00152912	https://www.ebi.ac.uk/metagenomics/api/v1/analyses/MGYA00152912/file/SRR5983372_MERGED_FASTQ_SSU_OTU.tsv	Root-Host_associated-Human-Digestive_system-Intestine
SRR5983404	MGYA00152840	https://www.ebi.ac.uk/metagenomics/api/v1/analyses/MGYA00152840/file/SRR5983404_MERGED_FASTQ_SSU_OTU.tsv	Root-Host_associated-Human-Digestive_system-Intestine
ERR2784823	MGYA00215616	https://www.ebi.ac.uk/metagenomics/api/v1/analyses/MGYA00215616/file/ERR2784823_MERGED_FASTQ_SSU_OTU.tsv	Root-Host_associated-Human-Digestive_system-Intestine
ERR2784797	MGYA00215554	https://www.ebi.ac.uk/metagenomics/api/v1/analyses/MGYA00215554/file/ERR2784797_MERGED_FASTQ_SSU_OTU.tsv	Root-Host_associated-Human-Digestive_system-Intestine
SRR5983287	MGYA00153014	https://www.ebi.ac.uk/metagenomics/api/v1/analyses/MGYA00153014/file/SRR5983287_MERGED_FASTQ_SSU_OTU.tsv	Root-Host_associated-Human-Digestive_system-Intestine
ERR2784662	MGYA00215586	https://www.ebi.ac.uk/metagenomics/api/v1/analyses/MGYA00215586/file/ERR2784662_MERGED_FASTQ_SSU_OTU.tsv	Root-Host_associated-Human-Digestive_system-Intestine
SRR5947837	MGYA00152853	https://www.ebi.ac.uk/metagenomics/api/v1/analyses/MGYA00152853/file/SRR5947837_MERGED_FASTQ_SSU_OTU.tsv	Root-Host_associated-Human-Digestive_system-Intestine
SRR5983478	MGYA00152903	https://www.ebi.ac.uk/metagenomics/api/v1/analyses/MGYA00152903/file/SRR5983478_MERGED_FASTQ_SSU_OTU.tsv	Root-Host_associated-Human-Digestive_system-Intestine

SRR5983415	MGYA00152925	https://www.ebi.ac.uk/metagenomics/api/v1/analyses/MGYA00152925/file/SRR5983415_MERGED_FASTQ_SSU_OTU.tsv	Root-Host_associated-Human-Digestive_system-Intestine
ERR2784832	MGYA00215436	https://www.ebi.ac.uk/metagenomics/api/v1/analyses/MGYA00215436/file/ERR2784832_MERGED_FASTQ_SSU_OTU.tsv	Root-Host_associated-Human-Digestive_system-Intestine
ERR2784786	MGYA00215545	https://www.ebi.ac.uk/metagenomics/api/v1/analyses/MGYA00215545/file/ERR2784786_MERGED_FASTQ_SSU_OTU.tsv	Root-Host_associated-Human-Digestive_system-Intestine
ERR2784757	MGYA00215483	https://www.ebi.ac.uk/metagenomics/api/v1/analyses/MGYA00215483/file/ERR2784757_MERGED_FASTQ_SSU_OTU.tsv	Root-Host_associated-Human-Digestive_system-Intestine
SRR5983363	MGYA00153050	https://www.ebi.ac.uk/metagenomics/api/v1/analyses/MGYA00153050/file/SRR5983363_MERGED_FASTQ_SSU_OTU.tsv	Root-Host_associated-Human-Digestive_system-Intestine
SRR5947902	MGYA00153095	https://www.ebi.ac.uk/metagenomics/api/v1/analyses/MGYA00153095/file/SRR5947902_MERGED_FASTQ_SSU_OTU.tsv	Root-Host_associated-Human-Digestive_system-Intestine
SRR5983469	MGYA00152805	https://www.ebi.ac.uk/metagenomics/api/v1/analyses/MGYA00152805/file/SRR5983469_MERGED_FASTQ_SSU_OTU.tsv	Root-Host_associated-Human-Digestive_system-Intestine
SRR5947826	MGYA00153010	https://www.ebi.ac.uk/metagenomics/api/v1/analyses/MGYA00153010/file/SRR5947826_MERGED_FASTQ_SSU_OTU.tsv	Root-Host_associated-Human-Digestive_system-Intestine
ERR2784673	MGYA00215614	https://www.ebi.ac.uk/metagenomics/api/v1/analyses/MGYA00215614/file/ERR2784673_MERGED_FASTQ_SSU_OTU.tsv	Root-Host_associated-Human-Digestive_system-Intestine
SRR5983296	MGYA00153036	https://www.ebi.ac.uk/metagenomics/api/v1/analyses/MGYA00153036/file/SRR5983296_MERGED_FASTQ_SSU_OTU.tsv	Root-Host_associated-Human-Digestive_system-Intestine
ERR2784849	MGYA00215488	https://www.ebi.ac.uk/metagenomics/api/v1/analyses/MGYA00215488/file/ERR2784849_MERGED_FASTQ_SSU_OTU.tsv	Root-Host_associated-Human-Digestive_system-Intestine
SRR5947821	MGYA00152792	https://www.ebi.ac.uk/metagenomics/api/v1/analyses/MGYA00152792/file/SRR5947821_MERGED_FASTQ_SSU_OTU.tsv	Root-Host_associated-Human-Digestive_system-Intestine

SRR5983318	MGYA00152861	https://www.ebi.ac.uk/metagenomics/api/v1/analyses/MGYA00152861/file/SRR5983318_MERGED_FASTQ_SSU_OTU.tsv	Root-Host_associated-Human-Digestive_system-Intestine
ERR2784674	MGYA00215547	https://www.ebi.ac.uk/metagenomics/api/v1/analyses/MGYA00215547/file/ERR2784674_MERGED_FASTQ_SSU_OTU.tsv	Root-Host_associated-Human-Digestive_system-Intestine
SRR5983291	MGYA00152871	https://www.ebi.ac.uk/metagenomics/api/v1/analyses/MGYA00152871/file/SRR5983291_MERGED_FASTQ_SSU_OTU.tsv	Root-Host_associated-Human-Digestive_system-Intestine
ERR2784835	MGYA00215521	https://www.ebi.ac.uk/metagenomics/api/v1/analyses/MGYA00215521/file/ERR2784835_MERGED_FASTQ_SSU_OTU.tsv	Root-Host_associated-Human-Digestive_system-Intestine
SRR5983412	MGYA00152934	https://www.ebi.ac.uk/metagenomics/api/v1/analyses/MGYA00152934/file/SRR5983412_MERGED_FASTQ_SSU_OTU.tsv	Root-Host_associated-Human-Digestive_system-Intestine
ERR2784781	MGYA00215494	https://www.ebi.ac.uk/metagenomics/api/v1/analyses/MGYA00215494/file/ERR2784781_MERGED_FASTQ_SSU_OTU.tsv	Root-Host_associated-Human-Digestive_system-Intestine
ERR2784750	MGYA00215496	https://www.ebi.ac.uk/metagenomics/api/v1/analyses/MGYA00215496/file/ERR2784750_MERGED_FASTQ_SSU_OTU.tsv	Root-Host_associated-Human-Digestive_system-Intestine
SRR5983364	MGYA00153004	https://www.ebi.ac.uk/metagenomics/api/v1/analyses/MGYA00153004/file/SRR5983364_MERGED_FASTQ_SSU_OTU.tsv	Root-Host_associated-Human-Digestive_system-Intestine
SRR5947905	MGYA00153112	https://www.ebi.ac.uk/metagenomics/api/v1/analyses/MGYA00153112/file/SRR5947905_MERGED_FASTQ_SSU_OTU.tsv	Root-Host_associated-Human-Digestive_system-Intestine
SRR5983460	MGYA00152891	https://www.ebi.ac.uk/metagenomics/api/v1/analyses/MGYA00152891/file/SRR5983460_MERGED_FASTQ_SSU_OTU.tsv	Root-Host_associated-Human-Digestive_system-Intestine
ERR2784847	MGYA00215530	https://www.ebi.ac.uk/metagenomics/api/v1/analyses/MGYA00215530/file/ERR2784847_MERGED_FASTQ_SSU_OTU.tsv	Root-Host_associated-Human-Digestive_system-Intestine
ERR2784722	MGYA00215561	https://www.ebi.ac.uk/metagenomics/api/v1/analyses/MGYA00215561/file/ERR2784722_MERGED_FASTQ_SSU_OTU.tsv	Root-Host_associated-Human-Digestive_system-Intestine

SRR5983316	MGYA00152993	https://www.ebi.ac.uk/metagenomics/api/v1/analyses/MGYA00152993/file/SRR5983316_MERGED_FASTQ_SSU_OTU.tsv	Root-Host_associated-Human-Digestive_system-Intestine
SRR5947853	MGYA00152883	https://www.ebi.ac.uk/metagenomics/api/v1/analyses/MGYA00152883/file/SRR5947853_MERGED_FASTQ_SSU_OTU.tsv	Root-Host_associated-Human-Digestive_system-Intestine
SRR5947882	MGYA00153068	https://www.ebi.ac.uk/metagenomics/api/v1/analyses/MGYA00153068/file/SRR5947882_MERGED_FASTQ_SSU_OTU.tsv	Root-Host_associated-Human-Digestive_system-Intestine
SRR5947854	MGYA00152897	https://www.ebi.ac.uk/metagenomics/api/v1/analyses/MGYA00152897/file/SRR5947854_MERGED_FASTQ_SSU_OTU.tsv	Root-Host_associated-Human-Digestive_system-Intestine
ERR2784788	MGYA00215489	https://www.ebi.ac.uk/metagenomics/api/v1/analyses/MGYA00215489/file/ERR2784788_MERGED_FASTQ_SSU_OTU.tsv	Root-Host_associated-Human-Digestive_system-Intestine
ERR2784759	MGYA00215558	https://www.ebi.ac.uk/metagenomics/api/v1/analyses/MGYA00215558/file/ERR2784759_MERGED_FASTQ_SSU_OTU.tsv	Root-Host_associated-Human-Digestive_system-Intestine
SRR5947828	MGYA00152944	https://www.ebi.ac.uk/metagenomics/api/v1/analyses/MGYA00152944/file/SRR5947828_MERGED_FASTQ_SSU_OTU.tsv	Root-Host_associated-Human-Digestive_system-Intestine
ERR2784840	MGYA00215518	https://www.ebi.ac.uk/metagenomics/api/v1/analyses/MGYA00215518/file/ERR2784840_MERGED_FASTQ_SSU_OTU.tsv	Root-Host_associated-Human-Digestive_system-Intestine
SRR5983467	MGYA00152977	https://www.ebi.ac.uk/metagenomics/api/v1/analyses/MGYA00152977/file/SRR5983467_MERGED_FASTQ_SSU_OTU.tsv	Root-Host_associated-Human-Digestive_system-Intestine
ERR2784725	MGYA00215585	https://www.ebi.ac.uk/metagenomics/api/v1/analyses/MGYA00215585/file/ERR2784725_MERGED_FASTQ_SSU_OTU.tsv	Root-Host_associated-Human-Digestive_system-Intestine
SRR5983311	MGYA00152882	https://www.ebi.ac.uk/metagenomics/api/v1/analyses/MGYA00152882/file/SRR5983311_MERGED_FASTQ_SSU_OTU.tsv	Root-Host_associated-Human-Digestive_system-Intestine
SRR5983298	MGYA00152862	https://www.ebi.ac.uk/metagenomics/api/v1/analyses/MGYA00152862/file/SRR5983298_MERGED_FASTQ_SSU_OTU.tsv	Root-Host_associated-Human-Digestive_system-Intestine

SRR5947970	MGYA00153113	https://www.ebi.ac.uk/metagenomics/api/v1/analyses/MGYA00153113/file/SRR5947970_MERGED_FASTQ_SSU_OTU.tsv	Root-Host_associated-Human-Digestive_system-Intestine
SRR5983358	MGYA00153033	https://www.ebi.ac.uk/metagenomics/api/v1/analyses/MGYA00153033/file/SRR5983358_MERGED_FASTQ_SSU_OTU.tsv	Root-Host_associated-Human-Digestive_system-Intestine
ERR2784809	MGYA00215446	https://www.ebi.ac.uk/metagenomics/api/v1/analyses/MGYA00215446/file/ERR2784809_MERGED_FASTQ_SSU_OTU.tsv	Root-Host_associated-Human-Digestive_system-Intestine
SRR5983389	MGYA00152851	https://www.ebi.ac.uk/metagenomics/api/v1/analyses/MGYA00152851/file/SRR5983389_MERGED_FASTQ_SSU_OTU.tsv	Root-Host_associated-Human-Digestive_system-Intestine
SRR5947861	MGYA00153127	https://www.ebi.ac.uk/metagenomics/api/v1/analyses/MGYA00153127/file/SRR5947861_MERGED_FASTQ_SSU_OTU.tsv	Root-Host_associated-Human-Digestive_system-Intestine
SRR5947939	MGYA00152968	https://www.ebi.ac.uk/metagenomics/api/v1/analyses/MGYA00152968/file/SRR5947939_MERGED_FASTQ_SSU_OTU.tsv	Root-Host_associated-Human-Digestive_system-Intestine
SRR5983483	MGYA00152810	https://www.ebi.ac.uk/metagenomics/api/v1/analyses/MGYA00152810/file/SRR5983483_MERGED_FASTQ_SSU_OTU.tsv	Root-Host_associated-Human-Digestive_system-Intestine
SRR5983324	MGYA00152866	https://www.ebi.ac.uk/metagenomics/api/v1/analyses/MGYA00152866/file/SRR5983324_MERGED_FASTQ_SSU_OTU.tsv	Root-Host_associated-Human-Digestive_system-Intestine
ERR2784710	MGYA00215548	https://www.ebi.ac.uk/metagenomics/api/v1/analyses/MGYA00215548/file/ERR2784710_MERGED_FASTQ_SSU_OTU.tsv	Root-Host_associated-Human-Digestive_system-Intestine
SRR5983452	MGYA00152971	https://www.ebi.ac.uk/metagenomics/api/v1/analyses/MGYA00152971/file/SRR5983452_MERGED_FASTQ_SSU_OTU.tsv	Root-Host_associated-Human-Digestive_system-Intestine
ERR2784699	MGYA00215465	https://www.ebi.ac.uk/metagenomics/api/v1/analyses/MGYA00215465/file/ERR2784699_MERGED_FASTQ_SSU_OTU.tsv	Root-Host_associated-Human-Digestive_system-Intestine
SRR5947945	MGYA00152784	https://www.ebi.ac.uk/metagenomics/api/v1/analyses/MGYA00152784/file/SRR5947945_MERGED_FASTQ_SSU_OTU.tsv	Root-Host_associated-Human-Digestive_system-Intestine

SRR5983323	MGYA00152812	https://www.ebi.ac.uk/metagenomics/api/v1/analyses/MGYA00152812/file/SRR5983323_MERGED_FASTQ_SSU_OTU.tsv	Root-Host_associated-Human-Digestive_system-Intestine
SRR5983484	MGYA00153148	https://www.ebi.ac.uk/metagenomics/api/v1/analyses/MGYA00153148/file/SRR5983484_MERGED_FASTQ_SSU_OTU.tsv	Root-Host_associated-Human-Digestive_system-Intestine
ERR2784717	MGYA00215490	https://www.ebi.ac.uk/metagenomics/api/v1/analyses/MGYA00215490/file/ERR2784717_MERGED_FASTQ_SSU_OTU.tsv	Root-Host_associated-Human-Digestive_system-Intestine
SRR5983455	MGYA00152900	https://www.ebi.ac.uk/metagenomics/api/v1/analyses/MGYA00152900/file/SRR5983455_MERGED_FASTQ_SSU_OTU.tsv	Root-Host_associated-Human-Digestive_system-Intestine
SRR5947942	MGYA00152796	https://www.ebi.ac.uk/metagenomics/api/v1/analyses/MGYA00152796/file/SRR5947942_MERGED_FASTQ_SSU_OTU.tsv	Root-Host_associated-Human-Digestive_system-Intestine
SRR5983429	MGYA00152902	https://www.ebi.ac.uk/metagenomics/api/v1/analyses/MGYA00152902/file/SRR5983429_MERGED_FASTQ_SSU_OTU.tsv	Root-Host_associated-Human-Digestive_system-Intestine
SRR5947866	MGYA00152952	https://www.ebi.ac.uk/metagenomics/api/v1/analyses/MGYA00152952/file/SRR5947866_MERGED_FASTQ_SSU_OTU.tsv	Root-Host_associated-Human-Digestive_system-Intestine
ERR2784719	MGYA00215460	https://www.ebi.ac.uk/metagenomics/api/v1/analyses/MGYA00215460/file/ERR2784719_MERGED_FASTQ_SSU_OTU.tsv	Root-Host_associated-Human-Digestive_system-Intestine
SRR5947814	MGYA00152836	https://www.ebi.ac.uk/metagenomics/api/v1/analyses/MGYA00152836/file/SRR5947814_MERGED_FASTQ_SSU_OTU.tsv	Root-Host_associated-Human-Digestive_system-Intestine
ERR2784690	MGYA00215502	https://www.ebi.ac.uk/metagenomics/api/v1/analyses/MGYA00215502/file/ERR2784690_MERGED_FASTQ_SSU_OTU.tsv	Root-Host_associated-Human-Digestive_system-Intestine
SRR5983275	MGYA00152931	https://www.ebi.ac.uk/metagenomics/api/v1/analyses/MGYA00152931/file/SRR5983275_MERGED_FASTQ_SSU_OTU.tsv	Root-Host_associated-Human-Digestive_system-Intestine
SRR5983351	MGYA00153044	https://www.ebi.ac.uk/metagenomics/api/v1/analyses/MGYA00153044/file/SRR5983351_MERGED_FASTQ_SSU_OTU.tsv	Root-Host_associated-Human-Digestive_system-Intestine

ERR2784765	MGYA00215555	https://www.ebi.ac.uk/metagenomics/api/v1/analyses/MGYA00215555/file/ERR2784765_MERGED_FASTQ_SSU_OTU.tsv	Root-Host_associated-Human-Digestive_system-Intestine
SRR5947868	MGYA00152880	https://www.ebi.ac.uk/metagenomics/api/v1/analyses/MGYA00152880/file/SRR5947868_MERGED_FASTQ_SSU_OTU.tsv	Root-Host_associated-Human-Digestive_system-Intestine
ERR2784800	MGYA00215560	https://www.ebi.ac.uk/metagenomics/api/v1/analyses/MGYA00215560/file/ERR2784800_MERGED_FASTQ_SSU_OTU.tsv	Root-Host_associated-Human-Digestive_system-Intestine
SRR5983427	MGYA00153074	https://www.ebi.ac.uk/metagenomics/api/v1/analyses/MGYA00153074/file/SRR5983427_MERGED_FASTQ_SSU_OTU.tsv	Root-Host_associated-Human-Digestive_system-Intestine
SRR5983380	MGYA00153154	https://www.ebi.ac.uk/metagenomics/api/v1/analyses/MGYA00153154/file/SRR5983380_MERGED_FASTQ_SSU_OTU.tsv	Root-Host_associated-Human-Digestive_system-Intestine
SRR5983356	MGYA00152948	https://www.ebi.ac.uk/metagenomics/api/v1/analyses/MGYA00152948/file/SRR5983356_MERGED_FASTQ_SSU_OTU.tsv	Root-Host_associated-Human-Digestive_system-Intestine
ERR2784762	MGYA00215451	https://www.ebi.ac.uk/metagenomics/api/v1/analyses/MGYA00215451/file/ERR2784762_MERGED_FASTQ_SSU_OTU.tsv	Root-Host_associated-Human-Digestive_system-Intestine
SRR5983387	MGYA00152854	https://www.ebi.ac.uk/metagenomics/api/v1/analyses/MGYA00152854/file/SRR5983387_MERGED_FASTQ_SSU_OTU.tsv	Root-Host_associated-Human-Digestive_system-Intestine
SRR5983420	MGYA00153150	https://www.ebi.ac.uk/metagenomics/api/v1/analyses/MGYA00153150/file/SRR5983420_MERGED_FASTQ_SSU_OTU.tsv	Root-Host_associated-Human-Digestive_system-Intestine
ERR2784807	MGYA00215583	https://www.ebi.ac.uk/metagenomics/api/v1/analyses/MGYA00215583/file/ERR2784807_MERGED_FASTQ_SSU_OTU.tsv	Root-Host_associated-Human-Digestive_system-Intestine
SRR5947937	MGYA00153096	https://www.ebi.ac.uk/metagenomics/api/v1/analyses/MGYA00153096/file/SRR5947937_MERGED_FASTQ_SSU_OTU.tsv	Root-Host_associated-Human-Digestive_system-Intestine
SRR5947813	MGYA00152790	https://www.ebi.ac.uk/metagenomics/api/v1/analyses/MGYA00152790/file/SRR5947813_MERGED_FASTQ_SSU_OTU.tsv	Root-Host_associated-Human-Digestive_system-Intestine

ERR2784697	MGYA00215454	https://www.ebi.ac.uk/metagenomics/api/v1/analyses/MGYA00215454/file/ERR2784697_MERGED_FASTQ_SSU_OTU.tsv	Root-Host_associated-Human-Digestive_system-Intestine
SRR5983272	MGYA00152940	https://www.ebi.ac.uk/metagenomics/api/v1/analyses/MGYA00152940/file/SRR5983272_MERGED_FASTQ_SSU_OTU.tsv	Root-Host_associated-Human-Digestive_system-Intestine
SRR5947912	MGYA00153002	https://www.ebi.ac.uk/metagenomics/api/v1/analyses/MGYA00153002/file/SRR5947912_MERGED_FASTQ_SSU_OTU.tsv	Root-Host_associated-Human-Digestive_system-Intestine
ERR2784747	MGYA00215602	https://www.ebi.ac.uk/metagenomics/api/v1/analyses/MGYA00215602/file/ERR2784747_MERGED_FASTQ_SSU_OTU.tsv	Root-Host_associated-Human-Digestive_system-Intestine
SRR5983373	MGYA00152830	https://www.ebi.ac.uk/metagenomics/api/v1/analyses/MGYA00152830/file/SRR5983373_MERGED_FASTQ_SSU_OTU.tsv	Root-Host_associated-Human-Digestive_system-Intestine
SRR5983405	MGYA00152842	https://www.ebi.ac.uk/metagenomics/api/v1/analyses/MGYA00152842/file/SRR5983405_MERGED_FASTQ_SSU_OTU.tsv	Root-Host_associated-Human-Digestive_system-Intestine
ERR2784822	MGYA00215471	https://www.ebi.ac.uk/metagenomics/api/v1/analyses/MGYA00215471/file/ERR2784822_MERGED_FASTQ_SSU_OTU.tsv	Root-Host_associated-Human-Digestive_system-Intestine
ERR2784796	MGYA00215538	https://www.ebi.ac.uk/metagenomics/api/v1/analyses/MGYA00215538/file/ERR2784796_MERGED_FASTQ_SSU_OTU.tsv	Root-Host_associated-Human-Digestive_system-Intestine
SRR5983286	MGYA00153019	https://www.ebi.ac.uk/metagenomics/api/v1/analyses/MGYA00153019/file/SRR5983286_MERGED_FASTQ_SSU_OTU.tsv	Root-Host_associated-Human-Digestive_system-Intestine
ERR2784663	MGYA00215569	https://www.ebi.ac.uk/metagenomics/api/v1/analyses/MGYA00215569/file/ERR2784663_MERGED_FASTQ_SSU_OTU.tsv	Root-Host_associated-Human-Digestive_system-Intestine
SRR5983479	MGYA00152788	https://www.ebi.ac.uk/metagenomics/api/v1/analyses/MGYA00152788/file/SRR5983479_MERGED_FASTQ_SSU_OTU.tsv	Root-Host_associated-Human-Digestive_system-Intestine
SRR5947836	MGYA00153103	https://www.ebi.ac.uk/metagenomics/api/v1/analyses/MGYA00153103/file/SRR5947836_MERGED_FASTQ_SSU_OTU.tsv	Root-Host_associated-Human-Digestive_system-Intestine

SRR5983281	MGYA00152924	https://www.ebi.ac.uk/metagenomics/api/v1/analyses/MGYA00152924/file/SRR5983281_MERGED_FASTQ_SSU_OTU.tsv	Root-Host_associated-Human-Digestive_system-Intestine
ERR2784664	MGYA00215567	https://www.ebi.ac.uk/metagenomics/api/v1/analyses/MGYA00215567/file/ERR2784664_MERGED_FASTQ_SSU_OTU.tsv	Root-Host_associated-Human-Digestive_system-Intestine
SRR5983308	MGYA00153048	https://www.ebi.ac.uk/metagenomics/api/v1/analyses/MGYA00153048/file/SRR5983308_MERGED_FASTQ_SSU_OTU.tsv	Root-Host_associated-Human-Digestive_system-Intestine
SRR5947831	MGYA00153043	https://www.ebi.ac.uk/metagenomics/api/v1/analyses/MGYA00153043/file/SRR5947831_MERGED_FASTQ_SSU_OTU.tsv	Root-Host_associated-Human-Digestive_system-Intestine
SRR5947915	MGYA00153140	https://www.ebi.ac.uk/metagenomics/api/v1/analyses/MGYA00153140/file/SRR5947915_MERGED_FASTQ_SSU_OTU.tsv	Root-Host_associated-Human-Digestive_system-Intestine
ERR2784740	MGYA00215577	https://www.ebi.ac.uk/metagenomics/api/v1/analyses/MGYA00215577/file/ERR2784740_MERGED_FASTQ_SSU_OTU.tsv	Root-Host_associated-Human-Digestive_system-Intestine
SRR5983374	MGYA00152991	https://www.ebi.ac.uk/metagenomics/api/v1/analyses/MGYA00152991/file/SRR5983374_MERGED_FASTQ_SSU_OTU.tsv	Root-Host_associated-Human-Digestive_system-Intestine
ERR2784825	MGYA00215546	https://www.ebi.ac.uk/metagenomics/api/v1/analyses/MGYA00215546/file/ERR2784825_MERGED_FASTQ_SSU_OTU.tsv	Root-Host_associated-Human-Digestive_system-Intestine
SRR5983402	MGYA00152913	https://www.ebi.ac.uk/metagenomics/api/v1/analyses/MGYA00152913/file/SRR5983402_MERGED_FASTQ_SSU_OTU.tsv	Root-Host_associated-Human-Digestive_system-Intestine
ERR2784791	MGYA00215563	https://www.ebi.ac.uk/metagenomics/api/v1/analyses/MGYA00215563/file/ERR2784791_MERGED_FASTQ_SSU_OTU.tsv	Root-Host_associated-Human-Digestive_system-Intestine
SRR5947967	MGYA00152809	https://www.ebi.ac.uk/metagenomics/api/v1/analyses/MGYA00152809/file/SRR5947967_MERGED_FASTQ_SSU_OTU.tsv	Root-Host_associated-Human-Digestive_system-Intestine
ERR2784732	MGYA00215592	https://www.ebi.ac.uk/metagenomics/api/v1/analyses/MGYA00215592/file/ERR2784732_MERGED_FASTQ_SSU_OTU.tsv	Root-Host_associated-Human-Digestive_system-Intestine

SRR5983306	MGYA00152855	https://www.ebi.ac.uk/metagenomics/api/v1/analyses/MGYA00152855/file/SRR5983306_MERGED_FASTQ_SSU_OTU.tsv	Root-Host_associated-Human-Digestive_system-Intestine
SRR5983470	MGYA00152958	https://www.ebi.ac.uk/metagenomics/api/v1/analyses/MGYA00152958/file/SRR5983470_MERGED_FASTQ_SSU_OTU.tsv	Root-Host_associated-Human-Digestive_system-Intestine
SRR5947843	MGYA00153066	https://www.ebi.ac.uk/metagenomics/api/v1/analyses/MGYA00153066/file/SRR5947843_MERGED_FASTQ_SSU_OTU.tsv	Root-Host_associated-Human-Digestive_system-Intestine
ERR2784749	MGYA00215519	https://www.ebi.ac.uk/metagenomics/api/v1/analyses/MGYA00215519/file/ERR2784749_MERGED_FASTQ_SSU_OTU.tsv	Root-Host_associated-Human-Digestive_system-Intestine
SRR5947895	MGYA00152885	https://www.ebi.ac.uk/metagenomics/api/v1/analyses/MGYA00152885/file/SRR5947895_MERGED_FASTQ_SSU_OTU.tsv	Root-Host_associated-Human-Digestive_system-Intestine
SRR5947844	MGYA00153070	https://www.ebi.ac.uk/metagenomics/api/v1/analyses/MGYA00153070/file/SRR5947844_MERGED_FASTQ_SSU_OTU.tsv	Root-Host_associated-Human-Digestive_system-Intestine
ERR2784798	MGYA00215517	https://www.ebi.ac.uk/metagenomics/api/v1/analyses/MGYA00215517/file/ERR2784798_MERGED_FASTQ_SSU_OTU.tsv	Root-Host_associated-Human-Digestive_system-Intestine
SRR5983288	MGYA00152932	https://www.ebi.ac.uk/metagenomics/api/v1/analyses/MGYA00152932/file/SRR5983288_MERGED_FASTQ_SSU_OTU.tsv	Root-Host_associated-Human-Digestive_system-Intestine
ERR2784735	MGYA00215503	https://www.ebi.ac.uk/metagenomics/api/v1/analyses/MGYA00215503/file/ERR2784735_MERGED_FASTQ_SSU_OTU.tsv	Root-Host_associated-Human-Digestive_system-Intestine
SRR5983301	MGYA00153124	https://www.ebi.ac.uk/metagenomics/api/v1/analyses/MGYA00153124/file/SRR5983301_MERGED_FASTQ_SSU_OTU.tsv	Root-Host_associated-Human-Digestive_system-Intestine
SRR5947838	MGYA00152943	https://www.ebi.ac.uk/metagenomics/api/v1/analyses/MGYA00152943/file/SRR5947838_MERGED_FASTQ_SSU_OTU.tsv	Root-Host_associated-Human-Digestive_system-Intestine
ERR2784850	MGYA00215589	https://www.ebi.ac.uk/metagenomics/api/v1/analyses/MGYA00215589/file/ERR2784850_MERGED_FASTQ_SSU_OTU.tsv	Root-Host_associated-Human-Digestive_system-Intestine

SRR5983477	MGYA00152981	https://www.ebi.ac.uk/metagenomics/api/v1/analyses/MGYA00152981/file/SRR5983477_MERGED_FASTQ_SSU_OTU.tsv	Root-Host_associated-Human-Digestive_system-Intestine
SRR5947929	MGYA00152916	https://www.ebi.ac.uk/metagenomics/api/v1/analyses/MGYA00152916/file/SRR5947929_MERGED_FASTQ_SSU_OTU.tsv	Root-Host_associated-Human-Digestive_system-Intestine
ERR2784819	MGYA00215620	https://www.ebi.ac.uk/metagenomics/api/v1/analyses/MGYA00215620/file/ERR2784819_MERGED_FASTQ_SSU_OTU.tsv	Root-Host_associated-Human-Digestive_system-Intestine
SRR5983399	MGYA00152783	https://www.ebi.ac.uk/metagenomics/api/v1/analyses/MGYA00152783/file/SRR5983399_MERGED_FASTQ_SSU_OTU.tsv	Root-Host_associated-Human-Digestive_system-Intestine
SRR5947871	MGYA00152848	https://www.ebi.ac.uk/metagenomics/api/v1/analyses/MGYA00152848/file/SRR5947871_MERGED_FASTQ_SSU_OTU.tsv	Root-Host_associated-Human-Digestive_system-Intestine
SRR5983348	MGYA00152975	https://www.ebi.ac.uk/metagenomics/api/v1/analyses/MGYA00152975/file/SRR5983348_MERGED_FASTQ_SSU_OTU.tsv	Root-Host_associated-Human-Digestive_system-Intestine
ERR2784689	MGYA00215581	https://www.ebi.ac.uk/metagenomics/api/v1/analyses/MGYA00215581/file/ERR2784689_MERGED_FASTQ_SSU_OTU.tsv	Root-Host_associated-Human-Digestive_system-Intestine
SRR5947955	MGYA00152884	https://www.ebi.ac.uk/metagenomics/api/v1/analyses/MGYA00152884/file/SRR5947955_MERGED_FASTQ_SSU_OTU.tsv	Root-Host_associated-Human-Digestive_system-Intestine
SRR5983442	MGYA00153027	https://www.ebi.ac.uk/metagenomics/api/v1/analyses/MGYA00153027/file/SRR5983442_MERGED_FASTQ_SSU_OTU.tsv	Root-Host_associated-Human-Digestive_system-Intestine
SRR5983334	MGYA00153149	https://www.ebi.ac.uk/metagenomics/api/v1/analyses/MGYA00153149/file/SRR5983334_MERGED_FASTQ_SSU_OTU.tsv	Root-Host_associated-Human-Digestive_system-Intestine
ERR2784700	MGYA00215562	https://www.ebi.ac.uk/metagenomics/api/v1/analyses/MGYA00215562/file/ERR2784700_MERGED_FASTQ_SSU_OTU.tsv	Root-Host_associated-Human-Digestive_system-Intestine
SRR5947952	MGYA00152930	https://www.ebi.ac.uk/metagenomics/api/v1/analyses/MGYA00152930/file/SRR5947952_MERGED_FASTQ_SSU_OTU.tsv	Root-Host_associated-Human-Digestive_system-Intestine

SRR5983445	MGYA00152837	https://www.ebi.ac.uk/metagenomics/api/v1/analyses/MGYA00152837/file/SRR5983445_MERGED_FASTQ_SSU_OTU.tsv	Root-Host_associated-Human-Digestive_system-Intestine
SRR5983333	MGYA00153137	https://www.ebi.ac.uk/metagenomics/api/v1/analyses/MGYA00153137/file/SRR5983333_MERGED_FASTQ_SSU_OTU.tsv	Root-Host_associated-Human-Digestive_system-Intestine
ERR2784707	MGYA00215587	https://www.ebi.ac.uk/metagenomics/api/v1/analyses/MGYA00215587/file/ERR2784707_MERGED_FASTQ_SSU_OTU.tsv	Root-Host_associated-Human-Digestive_system-Intestine
SRR5983439	MGYA00153121	https://www.ebi.ac.uk/metagenomics/api/v1/analyses/MGYA00153121/file/SRR5983439_MERGED_FASTQ_SSU_OTU.tsv	Root-Host_associated-Human-Digestive_system-Intestine
SRR5947876	MGYA00153085	https://www.ebi.ac.uk/metagenomics/api/v1/analyses/MGYA00153085/file/SRR5947876_MERGED_FASTQ_SSU_OTU.tsv	Root-Host_associated-Human-Digestive_system-Intestine
SRR5983265	MGYA00152921	https://www.ebi.ac.uk/metagenomics/api/v1/analyses/MGYA00152921/file/SRR5983265_MERGED_FASTQ_SSU_OTU.tsv	Root-Host_associated-Human-Digestive_system-Intestine
ERR2784680	MGYA00215513	https://www.ebi.ac.uk/metagenomics/api/v1/analyses/MGYA00215513/file/ERR2784680_MERGED_FASTQ_SSU_OTU.tsv	Root-Host_associated-Human-Digestive_system-Intestine
ERR2784709	MGYA00215522	https://www.ebi.ac.uk/metagenomics/api/v1/analyses/MGYA00215522/file/ERR2784709_MERGED_FASTQ_SSU_OTU.tsv	Root-Host_associated-Human-Digestive_system-Intestine
SRR5947920	MGYA00152856	https://www.ebi.ac.uk/metagenomics/api/v1/analyses/MGYA00152856/file/SRR5947920_MERGED_FASTQ_SSU_OTU.tsv	Root-Host_associated-Human-Digestive_system-Intestine
SRR5947878	MGYA00152896	https://www.ebi.ac.uk/metagenomics/api/v1/analyses/MGYA00152896/file/SRR5947878_MERGED_FASTQ_SSU_OTU.tsv	Root-Host_associated-Human-Digestive_system-Intestine
ERR2784810	MGYA00215525	https://www.ebi.ac.uk/metagenomics/api/v1/analyses/MGYA00215525/file/ERR2784810_MERGED_FASTQ_SSU_OTU.tsv	Root-Host_associated-Human-Digestive_system-Intestine
SRR5983437	MGYA00153049	https://www.ebi.ac.uk/metagenomics/api/v1/analyses/MGYA00153049/file/SRR5983437_MERGED_FASTQ_SSU_OTU.tsv	Root-Host_associated-Human-Digestive_system-Intestine

SRR5983390	MGYA00153051	https://www.ebi.ac.uk/metagenomics/api/v1/analyses/MGYA00153051/file/SRR5983390_MERGED_FASTQ_SSU_OTU.tsv	Root-Host_associated-Human-Digestive_system-Intestine
SRR5983341	MGYA00152786	https://www.ebi.ac.uk/metagenomics/api/v1/analyses/MGYA00152786/file/SRR5983341_MERGED_FASTQ_SSU_OTU.tsv	Root-Host_associated-Human-Digestive_system-Intestine
ERR2784775	MGYA00215531	https://www.ebi.ac.uk/metagenomics/api/v1/analyses/MGYA00215531/file/ERR2784775_MERGED_FASTQ_SSU_OTU.tsv	Root-Host_associated-Human-Digestive_system-Intestine
SRR5947927	MGYA00153132	https://www.ebi.ac.uk/metagenomics/api/v1/analyses/MGYA00153132/file/SRR5947927_MERGED_FASTQ_SSU_OTU.tsv	Root-Host_associated-Human-Digestive_system-Intestine
SRR5983397	MGYA00153072	https://www.ebi.ac.uk/metagenomics/api/v1/analyses/MGYA00153072/file/SRR5983397_MERGED_FASTQ_SSU_OTU.tsv	Root-Host_associated-Human-Digestive_system-Intestine
SRR5983430	MGYA00152821	https://www.ebi.ac.uk/metagenomics/api/v1/analyses/MGYA00152821/file/SRR5983430_MERGED_FASTQ_SSU_OTU.tsv	Root-Host_associated-Human-Digestive_system-Intestine
ERR2784817	MGYA00215475	https://www.ebi.ac.uk/metagenomics/api/v1/analyses/MGYA00215475/file/ERR2784817_MERGED_FASTQ_SSU_OTU.tsv	Root-Host_associated-Human-Digestive_system-Intestine
SRR5983346	MGYA00152803	https://www.ebi.ac.uk/metagenomics/api/v1/analyses/MGYA00152803/file/SRR5983346_MERGED_FASTQ_SSU_OTU.tsv	Root-Host_associated-Human-Digestive_system-Intestine
ERR2784772	MGYA00215476	https://www.ebi.ac.uk/metagenomics/api/v1/analyses/MGYA00215476/file/ERR2784772_MERGED_FASTQ_SSU_OTU.tsv	Root-Host_associated-Human-Digestive_system-Intestine
ERR2784687	MGYA00215447	https://www.ebi.ac.uk/metagenomics/api/v1/analyses/MGYA00215447/file/ERR2784687_MERGED_FASTQ_SSU_OTU.tsv	Root-Host_associated-Human-Digestive_system-Intestine
SRR5983357	MGYA00153158	https://www.ebi.ac.uk/metagenomics/api/v1/analyses/MGYA00153158/file/SRR5983357_MERGED_FASTQ_SSU_OTU.tsv	Root-Host_associated-Human-Digestive_system-Intestine
ERR2784763	MGYA00215520	https://www.ebi.ac.uk/metagenomics/api/v1/analyses/MGYA00215520/file/ERR2784763_MERGED_FASTQ_SSU_OTU.tsv	Root-Host_associated-Human-Digestive_system-Intestine

SRR5983421	MGYA00152909	https://www.ebi.ac.uk/metagenomics/api/v1/analyses/MGYA00152909/file/SRR5983421_MERGED_FASTQ_SSU_OTU.tsv	Root-Host_associated-Human-Digestive_system-Intestine
SRR5983386	MGYA00153143	https://www.ebi.ac.uk/metagenomics/api/v1/analyses/MGYA00153143/file/SRR5983386_MERGED_FASTQ_SSU_OTU.tsv	Root-Host_associated-Human-Digestive_system-Intestine
ERR2784806	MGYA00215456	https://www.ebi.ac.uk/metagenomics/api/v1/analyses/MGYA00215456/file/ERR2784806_MERGED_FASTQ_SSU_OTU.tsv	Root-Host_associated-Human-Digestive_system-Intestine
SRR5947936	MGYA00153144	https://www.ebi.ac.uk/metagenomics/api/v1/analyses/MGYA00153144/file/SRR5947936_MERGED_FASTQ_SSU_OTU.tsv	Root-Host_associated-Human-Digestive_system-Intestine
SRR5947812	MGYA00153040	https://www.ebi.ac.uk/metagenomics/api/v1/analyses/MGYA00153040/file/SRR5947812_MERGED_FASTQ_SSU_OTU.tsv	Root-Host_associated-Human-Digestive_system-Intestine
ERR2784696	MGYA00215491	https://www.ebi.ac.uk/metagenomics/api/v1/analyses/MGYA00215491/file/ERR2784696_MERGED_FASTQ_SSU_OTU.tsv	Root-Host_associated-Human-Digestive_system-Intestine
SRR5983273	MGYA00153109	https://www.ebi.ac.uk/metagenomics/api/v1/analyses/MGYA00153109/file/SRR5983273_MERGED_FASTQ_SSU_OTU.tsv	Root-Host_associated-Human-Digestive_system-Intestine
ERR2784718	MGYA00215435	https://www.ebi.ac.uk/metagenomics/api/v1/analyses/MGYA00215435/file/ERR2784718_MERGED_FASTQ_SSU_OTU.tsv	Root-Host_associated-Human-Digestive_system-Intestine
SRR5947815	MGYA00152949	https://www.ebi.ac.uk/metagenomics/api/v1/analyses/MGYA00152949/file/SRR5947815_MERGED_FASTQ_SSU_OTU.tsv	Root-Host_associated-Human-Digestive_system-Intestine
ERR2784691	MGYA00215610	https://www.ebi.ac.uk/metagenomics/api/v1/analyses/MGYA00215610/file/ERR2784691_MERGED_FASTQ_SSU_OTU.tsv	Root-Host_associated-Human-Digestive_system-Intestine
SRR5983274	MGYA00152822	https://www.ebi.ac.uk/metagenomics/api/v1/analyses/MGYA00152822/file/SRR5983274_MERGED_FASTQ_SSU_OTU.tsv	Root-Host_associated-Human-Digestive_system-Intestine
SRR5983350	MGYA00153003	https://www.ebi.ac.uk/metagenomics/api/v1/analyses/MGYA00153003/file/SRR5983350_MERGED_FASTQ_SSU_OTU.tsv	Root-Host_associated-Human-Digestive_system-Intestine

ERR2784764	MGYA00215472	https://www.ebi.ac.uk/metagenomics/api/v1/analyses/MGYA00215472/file/ERR2784764_MERGED_FASTQ_SSU_OTU.tsv	Root-Host_associated-Human-Digestive_system-Intestine
ERR2784801	MGYA00215526	https://www.ebi.ac.uk/metagenomics/api/v1/analyses/MGYA00215526/file/ERR2784801_MERGED_FASTQ_SSU_OTU.tsv	Root-Host_associated-Human-Digestive_system-Intestine
SRR5983381	MGYA00153053	https://www.ebi.ac.uk/metagenomics/api/v1/analyses/MGYA00153053/file/SRR5983381_MERGED_FASTQ_SSU_OTU.tsv	Root-Host_associated-Human-Digestive_system-Intestine
SRR5983426	MGYA00153042	https://www.ebi.ac.uk/metagenomics/api/v1/analyses/MGYA00153042/file/SRR5983426_MERGED_FASTQ_SSU_OTU.tsv	Root-Host_associated-Human-Digestive_system-Intestine
SRR5947869	MGYA00153125	https://www.ebi.ac.uk/metagenomics/api/v1/analyses/MGYA00153125/file/SRR5947869_MERGED_FASTQ_SSU_OTU.tsv	Root-Host_associated-Human-Digestive_system-Intestine
SRR5947931	MGYA00153062	https://www.ebi.ac.uk/metagenomics/api/v1/analyses/MGYA00153062/file/SRR5947931_MERGED_FASTQ_SSU_OTU.tsv	Root-Host_associated-Human-Digestive_system-Intestine
SRR5983485	MGYA00152914	https://www.ebi.ac.uk/metagenomics/api/v1/analyses/MGYA00152914/file/SRR5983485_MERGED_FASTQ_SSU_OTU.tsv	Root-Host_associated-Human-Digestive_system-Intestine
SRR5983322	MGYA00153011	https://www.ebi.ac.uk/metagenomics/api/v1/analyses/MGYA00153011/file/SRR5983322_MERGED_FASTQ_SSU_OTU.tsv	Root-Host_associated-Human-Digestive_system-Intestine
ERR2784716	MGYA00215462	https://www.ebi.ac.uk/metagenomics/api/v1/analyses/MGYA00215462/file/ERR2784716_MERGED_FASTQ_SSU_OTU.tsv	Root-Host_associated-Human-Digestive_system-Intestine
SRR5983454	MGYA00153025	https://www.ebi.ac.uk/metagenomics/api/v1/analyses/MGYA00153025/file/SRR5983454_MERGED_FASTQ_SSU_OTU.tsv	Root-Host_associated-Human-Digestive_system-Intestine
SRR5947943	MGYA00153031	https://www.ebi.ac.uk/metagenomics/api/v1/analyses/MGYA00153031/file/SRR5947943_MERGED_FASTQ_SSU_OTU.tsv	Root-Host_associated-Human-Digestive_system-Intestine
SRR5947867	MGYA00153013	https://www.ebi.ac.uk/metagenomics/api/v1/analyses/MGYA00153013/file/SRR5947867_MERGED_FASTQ_SSU_OTU.tsv	Root-Host_associated-Human-Digestive_system-Intestine

SRR5983428	MGYA00152832	https://www.ebi.ac.uk/metagenomics/api/v1/analyses/MGYA00152832/file/SRR5983428_MERGED_FASTQ_SSU_OTU.tsv	Root-Host_associated-Human-Digestive_system-Intestine
SRR5983359	MGYA00152799	https://www.ebi.ac.uk/metagenomics/api/v1/analyses/MGYA00152799/file/SRR5983359_MERGED_FASTQ_SSU_OTU.tsv	Root-Host_associated-Human-Digestive_system-Intestine
SRR5947860	MGYA00152876	https://www.ebi.ac.uk/metagenomics/api/v1/analyses/MGYA00152876/file/SRR5947860_MERGED_FASTQ_SSU_OTU.tsv	Root-Host_associated-Human-Digestive_system-Intestine
ERR2784808	MGYA00215612	https://www.ebi.ac.uk/metagenomics/api/v1/analyses/MGYA00215612/file/ERR2784808_MERGED_FASTQ_SSU_OTU.tsv	Root-Host_associated-Human-Digestive_system-Intestine
SRR5983388	MGYA00153141	https://www.ebi.ac.uk/metagenomics/api/v1/analyses/MGYA00153141/file/SRR5983388_MERGED_FASTQ_SSU_OTU.tsv	Root-Host_associated-Human-Digestive_system-Intestine
SRR5947938	MGYA00153091	https://www.ebi.ac.uk/metagenomics/api/v1/analyses/MGYA00153091/file/SRR5947938_MERGED_FASTQ_SSU_OTU.tsv	Root-Host_associated-Human-Digestive_system-Intestine
SRR5983325	MGYA00152908	https://www.ebi.ac.uk/metagenomics/api/v1/analyses/MGYA00152908/file/SRR5983325_MERGED_FASTQ_SSU_OTU.tsv	Root-Host_associated-Human-Digestive_system-Intestine
SRR5983482	MGYA00153000	https://www.ebi.ac.uk/metagenomics/api/v1/analyses/MGYA00153000/file/SRR5983482_MERGED_FASTQ_SSU_OTU.tsv	Root-Host_associated-Human-Digestive_system-Intestine
ERR2784711	MGYA00215539	https://www.ebi.ac.uk/metagenomics/api/v1/analyses/MGYA00215539/file/ERR2784711_MERGED_FASTQ_SSU_OTU.tsv	Root-Host_associated-Human-Digestive_system-Intestine
SRR5983453	MGYA00152829	https://www.ebi.ac.uk/metagenomics/api/v1/analyses/MGYA00152829/file/SRR5983453_MERGED_FASTQ_SSU_OTU.tsv	Root-Host_associated-Human-Digestive_system-Intestine
ERR2784698	MGYA00215484	https://www.ebi.ac.uk/metagenomics/api/v1/analyses/MGYA00215484/file/ERR2784698_MERGED_FASTQ_SSU_OTU.tsv	Root-Host_associated-Human-Digestive_system-Intestine
SRR5947944	MGYA00153108	https://www.ebi.ac.uk/metagenomics/api/v1/analyses/MGYA00153108/file/SRR5947944_MERGED_FASTQ_SSU_OTU.tsv	Root-Host_associated-Human-Digestive_system-Intestine

SRR5947855	MGYA00153107	https://www.ebi.ac.uk/metagenomics/api/v1/analyses/MGYA00153107/file/SRR5947855_MERGED_FASTQ_SSU_OTU.tsv	Root-Host_associated-Human-Digestive_system-Intestine
ERR2784789	MGYA00215549	https://www.ebi.ac.uk/metagenomics/api/v1/analyses/MGYA00215549/file/ERR2784789_MERGED_FASTQ_SSU_OTU.tsv	Root-Host_associated-Human-Digestive_system-Intestine
ERR2784758	MGYA00215464	https://www.ebi.ac.uk/metagenomics/api/v1/analyses/MGYA00215464/file/ERR2784758_MERGED_FASTQ_SSU_OTU.tsv	Root-Host_associated-Human-Digestive_system-Intestine
SRR5947884	MGYA00152858	https://www.ebi.ac.uk/metagenomics/api/v1/analyses/MGYA00152858/file/SRR5947884_MERGED_FASTQ_SSU_OTU.tsv	Root-Host_associated-Human-Digestive_system-Intestine
ERR2784841	MGYA00215590	https://www.ebi.ac.uk/metagenomics/api/v1/analyses/MGYA00215590/file/ERR2784841_MERGED_FASTQ_SSU_OTU.tsv	Root-Host_associated-Human-Digestive_system-Intestine
SRR5983466	MGYA00153081	https://www.ebi.ac.uk/metagenomics/api/v1/analyses/MGYA00153081/file/SRR5983466_MERGED_FASTQ_SSU_OTU.tsv	Root-Host_associated-Human-Digestive_system-Intestine
SRR5947829	MGYA00152990	https://www.ebi.ac.uk/metagenomics/api/v1/analyses/MGYA00152990/file/SRR5947829_MERGED_FASTQ_SSU_OTU.tsv	Root-Host_associated-Human-Digestive_system-Intestine
ERR2784724	MGYA00215440	https://www.ebi.ac.uk/metagenomics/api/v1/analyses/MGYA00215440/file/ERR2784724_MERGED_FASTQ_SSU_OTU.tsv	Root-Host_associated-Human-Digestive_system-Intestine
SRR5983310	MGYA00153078	https://www.ebi.ac.uk/metagenomics/api/v1/analyses/MGYA00153078/file/SRR5983310_MERGED_FASTQ_SSU_OTU.tsv	Root-Host_associated-Human-Digestive_system-Intestine
SRR5983299	MGYA00153005	https://www.ebi.ac.uk/metagenomics/api/v1/analyses/MGYA00153005/file/SRR5983299_MERGED_FASTQ_SSU_OTU.tsv	Root-Host_associated-Human-Digestive_system-Intestine
SRR5983461	MGYA00153146	https://www.ebi.ac.uk/metagenomics/api/v1/analyses/MGYA00153146/file/SRR5983461_MERGED_FASTQ_SSU_OTU.tsv	Root-Host_associated-Human-Digestive_system-Intestine
ERR2784846	MGYA00215611	https://www.ebi.ac.uk/metagenomics/api/v1/analyses/MGYA00215611/file/ERR2784846_MERGED_FASTQ_SSU_OTU.tsv	Root-Host_associated-Human-Digestive_system-Intestine

ERR2784723	MGYA00215514	https://www.ebi.ac.uk/metagenomics/api/v1/analyses/MGYA00215514/file/ERR2784723_MERGED_FASTQ_SSU_OTU.tsv	Root-Host_associated-Human-Digestive_system-Intestine
SRR5983317	MGYA00153119	https://www.ebi.ac.uk/metagenomics/api/v1/analyses/MGYA00153119/file/SRR5983317_MERGED_FASTQ_SSU_OTU.tsv	Root-Host_associated-Human-Digestive_system-Intestine
SRR5947852	MGYA00153118	https://www.ebi.ac.uk/metagenomics/api/v1/analyses/MGYA00153118/file/SRR5947852_MERGED_FASTQ_SSU_OTU.tsv	Root-Host_associated-Human-Digestive_system-Intestine
SRR5947883	MGYA00152827	https://www.ebi.ac.uk/metagenomics/api/v1/analyses/MGYA00152827/file/SRR5947883_MERGED_FASTQ_SSU_OTU.tsv	Root-Host_associated-Human-Digestive_system-Intestine
SRR5947820	MGYA00153145	https://www.ebi.ac.uk/metagenomics/api/v1/analyses/MGYA00153145/file/SRR5947820_MERGED_FASTQ_SSU_OTU.tsv	Root-Host_associated-Human-Digestive_system-Intestine
ERR2784848	MGYA00215619	https://www.ebi.ac.uk/metagenomics/api/v1/analyses/MGYA00215619/file/ERR2784848_MERGED_FASTQ_SSU_OTU.tsv	Root-Host_associated-Human-Digestive_system-Intestine
SRR5983319	MGYA00153147	https://www.ebi.ac.uk/metagenomics/api/v1/analyses/MGYA00153147/file/SRR5983319_MERGED_FASTQ_SSU_OTU.tsv	Root-Host_associated-Human-Digestive_system-Intestine
ERR2784675	MGYA00215523	https://www.ebi.ac.uk/metagenomics/api/v1/analyses/MGYA00215523/file/ERR2784675_MERGED_FASTQ_SSU_OTU.tsv	Root-Host_associated-Human-Digestive_system-Intestine
SRR5983290	MGYA00153064	https://www.ebi.ac.uk/metagenomics/api/v1/analyses/MGYA00153064/file/SRR5983290_MERGED_FASTQ_SSU_OTU.tsv	Root-Host_associated-Human-Digestive_system-Intestine
ERR2784834	MGYA00215528	https://www.ebi.ac.uk/metagenomics/api/v1/analyses/MGYA00215528/file/ERR2784834_MERGED_FASTQ_SSU_OTU.tsv	Root-Host_associated-Human-Digestive_system-Intestine
SRR5983413	MGYA00152867	https://www.ebi.ac.uk/metagenomics/api/v1/analyses/MGYA00152867/file/SRR5983413_MERGED_FASTQ_SSU_OTU.tsv	Root-Host_associated-Human-Digestive_system-Intestine
ERR2784780	MGYA00215474	https://www.ebi.ac.uk/metagenomics/api/v1/analyses/MGYA00215474/file/ERR2784780_MERGED_FASTQ_SSU_OTU.tsv	Root-Host_associated-Human-Digestive_system-Intestine

ERR2784751	MGYA00215594	https://www.ebi.ac.uk/metagenomics/api/v1/analyses/MGYA00215594/file/ERR2784751_MERGED_FASTQ_SSU_OTU.tsv	Root-Host_associated-Human-Digestive_system-Intestine
SRR5983365	MGYA00153026	https://www.ebi.ac.uk/metagenomics/api/v1/analyses/MGYA00153026/file/SRR5983365_MERGED_FASTQ_SSU_OTU.tsv	Root-Host_associated-Human-Digestive_system-Intestine
SRR5947904	MGYA00152933	https://www.ebi.ac.uk/metagenomics/api/v1/analyses/MGYA00152933/file/SRR5947904_MERGED_FASTQ_SSU_OTU.tsv	Root-Host_associated-Human-Digestive_system-Intestine
SRR5983414	MGYA00152802	https://www.ebi.ac.uk/metagenomics/api/v1/analyses/MGYA00152802/file/SRR5983414_MERGED_FASTQ_SSU_OTU.tsv	Root-Host_associated-Human-Digestive_system-Intestine
ERR2784833	MGYA00215580	https://www.ebi.ac.uk/metagenomics/api/v1/analyses/MGYA00215580/file/ERR2784833_MERGED_FASTQ_SSU_OTU.tsv	Root-Host_associated-Human-Digestive_system-Intestine
ERR2784787	MGYA00215512	https://www.ebi.ac.uk/metagenomics/api/v1/analyses/MGYA00215512/file/ERR2784787_MERGED_FASTQ_SSU_OTU.tsv	Root-Host_associated-Human-Digestive_system-Intestine
ERR2784756	MGYA00215613	https://www.ebi.ac.uk/metagenomics/api/v1/analyses/MGYA00215613/file/ERR2784756_MERGED_FASTQ_SSU_OTU.tsv	Root-Host_associated-Human-Digestive_system-Intestine
SRR5983362	MGYA00152860	https://www.ebi.ac.uk/metagenomics/api/v1/analyses/MGYA00152860/file/SRR5983362_MERGED_FASTQ_SSU_OTU.tsv	Root-Host_associated-Human-Digestive_system-Intestine
SRR5947903	MGYA00152869	https://www.ebi.ac.uk/metagenomics/api/v1/analyses/MGYA00152869/file/SRR5947903_MERGED_FASTQ_SSU_OTU.tsv	Root-Host_associated-Human-Digestive_system-Intestine
SRR5947827	MGYA00152985	https://www.ebi.ac.uk/metagenomics/api/v1/analyses/MGYA00152985/file/SRR5947827_MERGED_FASTQ_SSU_OTU.tsv	Root-Host_associated-Human-Digestive_system-Intestine
SRR5983468	MGYA00153082	https://www.ebi.ac.uk/metagenomics/api/v1/analyses/MGYA00153082/file/SRR5983468_MERGED_FASTQ_SSU_OTU.tsv	Root-Host_associated-Human-Digestive_system-Intestine
ERR2784672	MGYA00215443	https://www.ebi.ac.uk/metagenomics/api/v1/analyses/MGYA00215443/file/ERR2784672_MERGED_FASTQ_SSU_OTU.tsv	Root-Host_associated-Human-Digestive_system-Intestine

SRR5983297	MGYA00152838	https://www.ebi.ac.uk/metagenomics/api/v1/analyses/MGYA00152838/file/SRR5983297_MERGED_FASTQ_SSU_OTU.tsv	Root-Host_associated-Human-Digestive_system-Intestine
SRR5983459	MGYA00153052	https://www.ebi.ac.uk/metagenomics/api/v1/analyses/MGYA00153052/file/SRR5983459_MERGED_FASTQ_SSU_OTU.tsv	Root-Host_associated-Human-Digestive_system-Intestine
SRR5947816	MGYA00152961	https://www.ebi.ac.uk/metagenomics/api/v1/analyses/MGYA00152961/file/SRR5947816_MERGED_FASTQ_SSU_OTU.tsv	Root-Host_associated-Human-Digestive_system-Intestine
ERR2784692	MGYA00215439	https://www.ebi.ac.uk/metagenomics/api/v1/analyses/MGYA00215439/file/ERR2784692_MERGED_FASTQ_SSU_OTU.tsv	Root-Host_associated-Human-Digestive_system-Intestine
SRR5983277	MGYA00152972	https://www.ebi.ac.uk/metagenomics/api/v1/analyses/MGYA00152972/file/SRR5983277_MERGED_FASTQ_SSU_OTU.tsv	Root-Host_associated-Human-Digestive_system-Intestine
ERR2784767	MGYA00215466	https://www.ebi.ac.uk/metagenomics/api/v1/analyses/MGYA00215466/file/ERR2784767_MERGED_FASTQ_SSU_OTU.tsv	Root-Host_associated-Human-Digestive_system-Intestine
SRR5983353	MGYA00153046	https://www.ebi.ac.uk/metagenomics/api/v1/analyses/MGYA00153046/file/SRR5983353_MERGED_FASTQ_SSU_OTU.tsv	Root-Host_associated-Human-Digestive_system-Intestine
SRR5983382	MGYA00152791	https://www.ebi.ac.uk/metagenomics/api/v1/analyses/MGYA00152791/file/SRR5983382_MERGED_FASTQ_SSU_OTU.tsv	Root-Host_associated-Human-Digestive_system-Intestine
SRR5983425	MGYA00153120	https://www.ebi.ac.uk/metagenomics/api/v1/analyses/MGYA00153120/file/SRR5983425_MERGED_FASTQ_SSU_OTU.tsv	Root-Host_associated-Human-Digestive_system-Intestine
ERR2784802	MGYA00215524	https://www.ebi.ac.uk/metagenomics/api/v1/analyses/MGYA00215524/file/ERR2784802_MERGED_FASTQ_SSU_OTU.tsv	Root-Host_associated-Human-Digestive_system-Intestine
SRR5947932	MGYA00152970	https://www.ebi.ac.uk/metagenomics/api/v1/analyses/MGYA00152970/file/SRR5947932_MERGED_FASTQ_SSU_OTU.tsv	Root-Host_associated-Human-Digestive_system-Intestine
ERR2784760	MGYA00215535	https://www.ebi.ac.uk/metagenomics/api/v1/analyses/MGYA00215535/file/ERR2784760_MERGED_FASTQ_SSU_OTU.tsv	Root-Host_associated-Human-Digestive_system-Intestine

SRR5983354	MGYA00153134	https://www.ebi.ac.uk/metagenomics/api/v1/analyses/MGYA00153134/file/SRR5983354_MERGED_FASTQ_SSU_OTU.tsv	Root-Host_associated-Human-Digestive_system-Intestine
ERR2784805	MGYA00215495	https://www.ebi.ac.uk/metagenomics/api/v1/analyses/MGYA00215495/file/ERR2784805_MERGED_FASTQ_SSU_OTU.tsv	Root-Host_associated-Human-Digestive_system-Intestine
SRR5983422	MGYA00152787	https://www.ebi.ac.uk/metagenomics/api/v1/analyses/MGYA00152787/file/SRR5983422_MERGED_FASTQ_SSU_OTU.tsv	Root-Host_associated-Human-Digestive_system-Intestine
SRR5983385	MGYA00153086	https://www.ebi.ac.uk/metagenomics/api/v1/analyses/MGYA00153086/file/SRR5983385_MERGED_FASTQ_SSU_OTU.tsv	Root-Host_associated-Human-Digestive_system-Intestine
SRR5947935	MGYA00153088	https://www.ebi.ac.uk/metagenomics/api/v1/analyses/MGYA00153088/file/SRR5947935_MERGED_FASTQ_SSU_OTU.tsv	Root-Host_associated-Human-Digestive_system-Intestine
SRR5983328	MGYA00152846	https://www.ebi.ac.uk/metagenomics/api/v1/analyses/MGYA00152846/file/SRR5983328_MERGED_FASTQ_SSU_OTU.tsv	Root-Host_associated-Human-Digestive_system-Intestine
SRR5947811	MGYA00153133	https://www.ebi.ac.uk/metagenomics/api/v1/analyses/MGYA00153133/file/SRR5947811_MERGED_FASTQ_SSU_OTU.tsv	Root-Host_associated-Human-Digestive_system-Intestine
ERR2784695	MGYA00215609	https://www.ebi.ac.uk/metagenomics/api/v1/analyses/MGYA00215609/file/ERR2784695_MERGED_FASTQ_SSU_OTU.tsv	Root-Host_associated-Human-Digestive_system-Intestine
SRR5983270	MGYA00152823	https://www.ebi.ac.uk/metagenomics/api/v1/analyses/MGYA00152823/file/SRR5983270_MERGED_FASTQ_SSU_OTU.tsv	Root-Host_associated-Human-Digestive_system-Intestine
SRR5947863	MGYA00152904	https://www.ebi.ac.uk/metagenomics/api/v1/analyses/MGYA00152904/file/SRR5947863_MERGED_FASTQ_SSU_OTU.tsv	Root-Host_associated-Human-Digestive_system-Intestine
ERR2784712	MGYA00215509	https://www.ebi.ac.uk/metagenomics/api/v1/analyses/MGYA00215509/file/ERR2784712_MERGED_FASTQ_SSU_OTU.tsv	Root-Host_associated-Human-Digestive_system-Intestine
SRR5983326	MGYA00152980	https://www.ebi.ac.uk/metagenomics/api/v1/analyses/MGYA00152980/file/SRR5983326_MERGED_FASTQ_SSU_OTU.tsv	Root-Host_associated-Human-Digestive_system-Intestine

SRR5983481	MGYA00153006	https://www.ebi.ac.uk/metagenomics/api/v1/analyses/MGYA00153006/file/SRR5983481_MERGED_FASTQ_SSU_OTU.tsv	Root-Host_associated-Human-Digestive_system-Intestine
SRR5983450	MGYA00153122	https://www.ebi.ac.uk/metagenomics/api/v1/analyses/MGYA00153122/file/SRR5983450_MERGED_FASTQ_SSU_OTU.tsv	Root-Host_associated-Human-Digestive_system-Intestine
SRR5947947	MGYA00152890	https://www.ebi.ac.uk/metagenomics/api/v1/analyses/MGYA00152890/file/SRR5947947_MERGED_FASTQ_SSU_OTU.tsv	Root-Host_associated-Human-Digestive_system-Intestine
ERR2784715	MGYA00215584	https://www.ebi.ac.uk/metagenomics/api/v1/analyses/MGYA00215584/file/ERR2784715_MERGED_FASTQ_SSU_OTU.tsv	Root-Host_associated-Human-Digestive_system-Intestine
SRR5983321	MGYA00152797	https://www.ebi.ac.uk/metagenomics/api/v1/analyses/MGYA00152797/file/SRR5983321_MERGED_FASTQ_SSU_OTU.tsv	Root-Host_associated-Human-Digestive_system-Intestine
SRR5947818	MGYA00153041	https://www.ebi.ac.uk/metagenomics/api/v1/analyses/MGYA00153041/file/SRR5947818_MERGED_FASTQ_SSU_OTU.tsv	Root-Host_associated-Human-Digestive_system-Intestine
SRR5983457	MGYA00152808	https://www.ebi.ac.uk/metagenomics/api/v1/analyses/MGYA00152808/file/SRR5983457_MERGED_FASTQ_SSU_OTU.tsv	Root-Host_associated-Human-Digestive_system-Intestine
SRR5947940	MGYA00152969	https://www.ebi.ac.uk/metagenomics/api/v1/analyses/MGYA00152969/file/SRR5947940_MERGED_FASTQ_SSU_OTU.tsv	Root-Host_associated-Human-Digestive_system-Intestine
SRR5983279	MGYA00152951	https://www.ebi.ac.uk/metagenomics/api/v1/analyses/MGYA00152951/file/SRR5983279_MERGED_FASTQ_SSU_OTU.tsv	Root-Host_associated-Human-Digestive_system-Intestine
ERR2784769	MGYA00215532	https://www.ebi.ac.uk/metagenomics/api/v1/analyses/MGYA00215532/file/ERR2784769_MERGED_FASTQ_SSU_OTU.tsv	Root-Host_associated-Human-Digestive_system-Intestine
SRR5947864	MGYA00152789	https://www.ebi.ac.uk/metagenomics/api/v1/analyses/MGYA00152789/file/SRR5947864_MERGED_FASTQ_SSU_OTU.tsv	Root-Host_associated-Human-Digestive_system-Intestine
ERR2784845	MGYA00215556	https://www.ebi.ac.uk/metagenomics/api/v1/analyses/MGYA00215556/file/ERR2784845_MERGED_FASTQ_SSU_OTU.tsv	Root-Host_associated-Human-Digestive_system-Intestine

SRR5983462	MGYA00153069	https://www.ebi.ac.uk/metagenomics/api/v1/analyses/MGYA00153069/file/SRR5983462_MERGED_FASTQ_SSU_OTU.tsv	Root-Host_associated-Human-Digestive_system-Intestine
SRR5983314	MGYA00153077	https://www.ebi.ac.uk/metagenomics/api/v1/analyses/MGYA00153077/file/SRR5983314_MERGED_FASTQ_SSU_OTU.tsv	Root-Host_associated-Human-Digestive_system-Intestine
ERR2784720	MGYA00215566	https://www.ebi.ac.uk/metagenomics/api/v1/analyses/MGYA00215566/file/ERR2784720_MERGED_FASTQ_SSU_OTU.tsv	Root-Host_associated-Human-Digestive_system-Intestine
ERR2784678	MGYA00215596	https://www.ebi.ac.uk/metagenomics/api/v1/analyses/MGYA00215596/file/ERR2784678_MERGED_FASTQ_SSU_OTU.tsv	Root-Host_associated-Human-Digestive_system-Intestine
ERR2784839	MGYA00215497	https://www.ebi.ac.uk/metagenomics/api/v1/analyses/MGYA00215497/file/ERR2784839_MERGED_FASTQ_SSU_OTU.tsv	Root-Host_associated-Human-Digestive_system-Intestine
SRR5947851	MGYA00153090	https://www.ebi.ac.uk/metagenomics/api/v1/analyses/MGYA00153090/file/SRR5947851_MERGED_FASTQ_SSU_OTU.tsv	Root-Host_associated-Human-Digestive_system-Intestine
SRR5947880	MGYA00152877	https://www.ebi.ac.uk/metagenomics/api/v1/analyses/MGYA00152877/file/SRR5947880_MERGED_FASTQ_SSU_OTU.tsv	Root-Host_associated-Human-Digestive_system-Intestine
SRR5983368	MGYA00152917	https://www.ebi.ac.uk/metagenomics/api/v1/analyses/MGYA00152917/file/SRR5983368_MERGED_FASTQ_SSU_OTU.tsv	Root-Host_associated-Human-Digestive_system-Intestine
SRR5947909	MGYA00152892	https://www.ebi.ac.uk/metagenomics/api/v1/analyses/MGYA00152892/file/SRR5947909_MERGED_FASTQ_SSU_OTU.tsv	Root-Host_associated-Human-Digestive_system-Intestine
SRR5983419	MGYA00153016	https://www.ebi.ac.uk/metagenomics/api/v1/analyses/MGYA00153016/file/SRR5983419_MERGED_FASTQ_SSU_OTU.tsv	Root-Host_associated-Human-Digestive_system-Intestine
SRR5947856	MGYA00152938	https://www.ebi.ac.uk/metagenomics/api/v1/analyses/MGYA00152938/file/SRR5947856_MERGED_FASTQ_SSU_OTU.tsv	Root-Host_associated-Human-Digestive_system-Intestine
SRR5983465	MGYA00152920	https://www.ebi.ac.uk/metagenomics/api/v1/analyses/MGYA00152920/file/SRR5983465_MERGED_FASTQ_SSU_OTU.tsv	Root-Host_associated-Human-Digestive_system-Intestine

ERR2784842	MGYA00215533	https://www.ebi.ac.uk/metagenomics/api/v1/analyses/MGYA00215533/file/ERR2784842_MERGED_FASTQ_SSU_OTU.tsv	Root-Host_associated-Human-Digestive_system-Intestine
SRR5983313	MGYA00152873	https://www.ebi.ac.uk/metagenomics/api/v1/analyses/MGYA00152873/file/SRR5983313_MERGED_FASTQ_SSU_OTU.tsv	Root-Host_associated-Human-Digestive_system-Intestine
ERR2784727	MGYA00215473	https://www.ebi.ac.uk/metagenomics/api/v1/analyses/MGYA00215473/file/ERR2784727_MERGED_FASTQ_SSU_OTU.tsv	Root-Host_associated-Human-Digestive_system-Intestine
SRR5947972	MGYA00152881	https://www.ebi.ac.uk/metagenomics/api/v1/analyses/MGYA00152881/file/SRR5947972_MERGED_FASTQ_SSU_OTU.tsv	Root-Host_associated-Human-Digestive_system-Intestine
ERR2784784	MGYA00215481	https://www.ebi.ac.uk/metagenomics/api/v1/analyses/MGYA00215481/file/ERR2784784_MERGED_FASTQ_SSU_OTU.tsv	Root-Host_associated-Human-Digestive_system-Intestine
SRR5947858	MGYA00152947	https://www.ebi.ac.uk/metagenomics/api/v1/analyses/MGYA00152947/file/SRR5947858_MERGED_FASTQ_SSU_OTU.tsv	Root-Host_associated-Human-Digestive_system-Intestine
ERR2784830	MGYA00215621	https://www.ebi.ac.uk/metagenomics/api/v1/analyses/MGYA00215621/file/ERR2784830_MERGED_FASTQ_SSU_OTU.tsv	Root-Host_associated-Human-Digestive_system-Intestine
SRR5983417	MGYA00152841	https://www.ebi.ac.uk/metagenomics/api/v1/analyses/MGYA00152841/file/SRR5983417_MERGED_FASTQ_SSU_OTU.tsv	Root-Host_associated-Human-Digestive_system-Intestine
SRR5983361	MGYA00152833	https://www.ebi.ac.uk/metagenomics/api/v1/analyses/MGYA00152833/file/SRR5983361_MERGED_FASTQ_SSU_OTU.tsv	Root-Host_associated-Human-Digestive_system-Intestine
ERR2784755	MGYA00215582	https://www.ebi.ac.uk/metagenomics/api/v1/analyses/MGYA00215582/file/ERR2784755_MERGED_FASTQ_SSU_OTU.tsv	Root-Host_associated-Human-Digestive_system-Intestine
SRR5947900	MGYA00152898	https://www.ebi.ac.uk/metagenomics/api/v1/analyses/MGYA00152898/file/SRR5947900_MERGED_FASTQ_SSU_OTU.tsv	Root-Host_associated-Human-Digestive_system-Intestine
SRR5947824	MGYA00153047	https://www.ebi.ac.uk/metagenomics/api/v1/analyses/MGYA00153047/file/SRR5947824_MERGED_FASTQ_SSU_OTU.tsv	Root-Host_associated-Human-Digestive_system-Intestine

ERR2784729	MGYA00215572	https://www.ebi.ac.uk/metagenomics/api/v1/analyses/MGYA00215572/file/ERR2784729_MERGED_FASTQ_SSU_OTU.tsv	Root-Host_associated-Human-Digestive_system-Intestine
ERR2784671	MGYA00215482	https://www.ebi.ac.uk/metagenomics/api/v1/analyses/MGYA00215482/file/ERR2784671_MERGED_FASTQ_SSU_OTU.tsv	Root-Host_associated-Human-Digestive_system-Intestine
SRR5983294	MGYA00153100	https://www.ebi.ac.uk/metagenomics/api/v1/analyses/MGYA00153100/file/SRR5983294_MERGED_FASTQ_SSU_OTU.tsv	Root-Host_associated-Human-Digestive_system-Intestine
SRR5947823	MGYA00153007	https://www.ebi.ac.uk/metagenomics/api/v1/analyses/MGYA00153007/file/SRR5947823_MERGED_FASTQ_SSU_OTU.tsv	Root-Host_associated-Human-Digestive_system-Intestine
ERR2784676	MGYA00215537	https://www.ebi.ac.uk/metagenomics/api/v1/analyses/MGYA00215537/file/ERR2784676_MERGED_FASTQ_SSU_OTU.tsv	Root-Host_associated-Human-Digestive_system-Intestine
SRR5983293	MGYA00152850	https://www.ebi.ac.uk/metagenomics/api/v1/analyses/MGYA00152850/file/SRR5983293_MERGED_FASTQ_SSU_OTU.tsv	Root-Host_associated-Human-Digestive_system-Intestine
ERR2784783	MGYA00215564	https://www.ebi.ac.uk/metagenomics/api/v1/analyses/MGYA00215564/file/ERR2784783_MERGED_FASTQ_SSU_OTU.tsv	Root-Host_associated-Human-Digestive_system-Intestine
SRR5983410	MGYA00152901	https://www.ebi.ac.uk/metagenomics/api/v1/analyses/MGYA00152901/file/SRR5983410_MERGED_FASTQ_SSU_OTU.tsv	Root-Host_associated-Human-Digestive_system-Intestine
ERR2784837	MGYA00215623	https://www.ebi.ac.uk/metagenomics/api/v1/analyses/MGYA00215623/file/ERR2784837_MERGED_FASTQ_SSU_OTU.tsv	Root-Host_associated-Human-Digestive_system-Intestine
SRR5983366	MGYA00152960	https://www.ebi.ac.uk/metagenomics/api/v1/analyses/MGYA00152960/file/SRR5983366_MERGED_FASTQ_SSU_OTU.tsv	Root-Host_associated-Human-Digestive_system-Intestine
ERR2784752	MGYA00215605	https://www.ebi.ac.uk/metagenomics/api/v1/analyses/MGYA00215605/file/ERR2784752_MERGED_FASTQ_SSU_OTU.tsv	Root-Host_associated-Human-Digestive_system-Intestine
SRR5947907	MGYA00152889	https://www.ebi.ac.uk/metagenomics/api/v1/analyses/MGYA00152889/file/SRR5947907_MERGED_FASTQ_SSU_OTU.tsv	Root-Host_associated-Human-Digestive_system-Intestine

SRR5983282	MGYA00152922	https://www.ebi.ac.uk/metagenomics/api/v1/analyses/MGYA00152922/file/SRR5983282_MERGED_FASTQ_SSU_OTU.tsv	Root-Host_associated-Human-Digestive_system-Intestine
ERR2784667	MGYA00215480	https://www.ebi.ac.uk/metagenomics/api/v1/analyses/MGYA00215480/file/ERR2784667_MERGED_FASTQ_SSU_OTU.tsv	Root-Host_associated-Human-Digestive_system-Intestine
SRR5947832	MGYA00152906	https://www.ebi.ac.uk/metagenomics/api/v1/analyses/MGYA00152906/file/SRR5947832_MERGED_FASTQ_SSU_OTU.tsv	Root-Host_associated-Human-Digestive_system-Intestine
SRR5947916	MGYA00153001	https://www.ebi.ac.uk/metagenomics/api/v1/analyses/MGYA00153001/file/SRR5947916_MERGED_FASTQ_SSU_OTU.tsv	Root-Host_associated-Human-Digestive_system-Intestine
SRR5983377	MGYA00152939	https://www.ebi.ac.uk/metagenomics/api/v1/analyses/MGYA00152939/file/SRR5983377_MERGED_FASTQ_SSU_OTU.tsv	Root-Host_associated-Human-Digestive_system-Intestine
ERR2784743	MGYA00215463	https://www.ebi.ac.uk/metagenomics/api/v1/analyses/MGYA00215463/file/ERR2784743_MERGED_FASTQ_SSU_OTU.tsv	Root-Host_associated-Human-Digestive_system-Intestine
ERR2784792	MGYA00215445	https://www.ebi.ac.uk/metagenomics/api/v1/analyses/MGYA00215445/file/ERR2784792_MERGED_FASTQ_SSU_OTU.tsv	Root-Host_associated-Human-Digestive_system-Intestine
SRR5983401	MGYA00152923	https://www.ebi.ac.uk/metagenomics/api/v1/analyses/MGYA00152923/file/SRR5983401_MERGED_FASTQ_SSU_OTU.tsv	Root-Host_associated-Human-Digestive_system-Intestine
ERR2784826	MGYA00215597	https://www.ebi.ac.uk/metagenomics/api/v1/analyses/MGYA00215597/file/ERR2784826_MERGED_FASTQ_SSU_OTU.tsv	Root-Host_associated-Human-Digestive_system-Intestine
SRR5947911	MGYA00152910	https://www.ebi.ac.uk/metagenomics/api/v1/analyses/MGYA00152910/file/SRR5947911_MERGED_FASTQ_SSU_OTU.tsv	Root-Host_associated-Human-Digestive_system-Intestine
SRR5947898	MGYA00152843	https://www.ebi.ac.uk/metagenomics/api/v1/analyses/MGYA00152843/file/SRR5947898_MERGED_FASTQ_SSU_OTU.tsv	Root-Host_associated-Human-Digestive_system-Intestine
SRR5983370	MGYA00152887	https://www.ebi.ac.uk/metagenomics/api/v1/analyses/MGYA00152887/file/SRR5983370_MERGED_FASTQ_SSU_OTU.tsv	Root-Host_associated-Human-Digestive_system-Intestine

ERR2784744	MGYA00215552	https://www.ebi.ac.uk/metagenomics/api/v1/analyses/MGYA00215552/file/ERR2784744_MERGED_FASTQ_SSU_OTU.tsv	Root-Host_associated-Human-Digestive_system-Intestine
ERR2784795	MGYA00215433	https://www.ebi.ac.uk/metagenomics/api/v1/analyses/MGYA00215433/file/ERR2784795_MERGED_FASTQ_SSU_OTU.tsv	Root-Host_associated-Human-Digestive_system-Intestine
ERR2784821	MGYA00215500	https://www.ebi.ac.uk/metagenomics/api/v1/analyses/MGYA00215500/file/ERR2784821_MERGED_FASTQ_SSU_OTU.tsv	Root-Host_associated-Human-Digestive_system-Intestine
SRR5983406	MGYA00153055	https://www.ebi.ac.uk/metagenomics/api/v1/analyses/MGYA00153055/file/SRR5983406_MERGED_FASTQ_SSU_OTU.tsv	Root-Host_associated-Human-Digestive_system-Intestine
SRR5947849	MGYA00152999	https://www.ebi.ac.uk/metagenomics/api/v1/analyses/MGYA00152999/file/SRR5947849_MERGED_FASTQ_SSU_OTU.tsv	Root-Host_associated-Human-Digestive_system-Intestine
SRR5983285	MGYA00153128	https://www.ebi.ac.uk/metagenomics/api/v1/analyses/MGYA00153128/file/SRR5983285_MERGED_FASTQ_SSU_OTU.tsv	Root-Host_associated-Human-Digestive_system-Intestine
ERR2784660	MGYA00215536	https://www.ebi.ac.uk/metagenomics/api/v1/analyses/MGYA00215536/file/ERR2784660_MERGED_FASTQ_SSU_OTU.tsv	Root-Host_associated-Human-Digestive_system-Intestine
ERR2784738	MGYA00215458	https://www.ebi.ac.uk/metagenomics/api/v1/analyses/MGYA00215458/file/ERR2784738_MERGED_FASTQ_SSU_OTU.tsv	Root-Host_associated-Human-Digestive_system-Intestine
SRR5947835	MGYA00153028	https://www.ebi.ac.uk/metagenomics/api/v1/analyses/MGYA00153028/file/SRR5947835_MERGED_FASTQ_SSU_OTU.tsv	Root-Host_associated-Human-Digestive_system-Intestine
SRR5947896	MGYA00152835	https://www.ebi.ac.uk/metagenomics/api/v1/analyses/MGYA00152835/file/SRR5947896_MERGED_FASTQ_SSU_OTU.tsv	Root-Host_associated-Human-Digestive_system-Intestine
SRR5947847	MGYA00153023	https://www.ebi.ac.uk/metagenomics/api/v1/analyses/MGYA00153023/file/SRR5947847_MERGED_FASTQ_SSU_OTU.tsv	Root-Host_associated-Human-Digestive_system-Intestine
SRR5983408	MGYA00152953	https://www.ebi.ac.uk/metagenomics/api/v1/analyses/MGYA00152953/file/SRR5983408_MERGED_FASTQ_SSU_OTU.tsv	Root-Host_associated-Human-Digestive_system-Intestine

SRR5947963	MGYA00152894	https://www.ebi.ac.uk/metagenomics/api/v1/analyses/MGYA00152894/file/SRR5947963_MERGED_FASTQ_SSU_OTU.tsv	Root-Host_associated-Human-Digestive_system-Intestine
SRR5983302	MGYA00153136	https://www.ebi.ac.uk/metagenomics/api/v1/analyses/MGYA00153136/file/SRR5983302_MERGED_FASTQ_SSU_OTU.tsv	Root-Host_associated-Human-Digestive_system-Intestine
ERR2784736	MGYA00215544	https://www.ebi.ac.uk/metagenomics/api/v1/analyses/MGYA00215544/file/ERR2784736_MERGED_FASTQ_SSU_OTU.tsv	Root-Host_associated-Human-Digestive_system-Intestine
SRR5983474	MGYA00152844	https://www.ebi.ac.uk/metagenomics/api/v1/analyses/MGYA00152844/file/SRR5983474_MERGED_FASTQ_SSU_OTU.tsv	Root-Host_associated-Human-Digestive_system-Intestine
ERR2784669	MGYA00215469	https://www.ebi.ac.uk/metagenomics/api/v1/analyses/MGYA00215469/file/ERR2784669_MERGED_FASTQ_SSU_OTU.tsv	Root-Host_associated-Human-Digestive_system-Intestine
SRR5983305	MGYA00153130	https://www.ebi.ac.uk/metagenomics/api/v1/analyses/MGYA00153130/file/SRR5983305_MERGED_FASTQ_SSU_OTU.tsv	Root-Host_associated-Human-Digestive_system-Intestine
ERR2784731	MGYA00215508	https://www.ebi.ac.uk/metagenomics/api/v1/analyses/MGYA00215508/file/ERR2784731_MERGED_FASTQ_SSU_OTU.tsv	Root-Host_associated-Human-Digestive_system-Intestine
SRR5983473	MGYA00152950	https://www.ebi.ac.uk/metagenomics/api/v1/analyses/MGYA00152950/file/SRR5983473_MERGED_FASTQ_SSU_OTU.tsv	Root-Host_associated-Human-Digestive_system-Intestine
SRR5947918	MGYA00153039	https://www.ebi.ac.uk/metagenomics/api/v1/analyses/MGYA00153039/file/SRR5947918_MERGED_FASTQ_SSU_OTU.tsv	Root-Host_associated-Human-Digestive_system-Intestine
SRR5983379	MGYA00152847	https://www.ebi.ac.uk/metagenomics/api/v1/analyses/MGYA00152847/file/SRR5983379_MERGED_FASTQ_SSU_OTU.tsv	Root-Host_associated-Human-Digestive_system-Intestine
SRR5947840	MGYA00152942	https://www.ebi.ac.uk/metagenomics/api/v1/analyses/MGYA00152942/file/SRR5947840_MERGED_FASTQ_SSU_OTU.tsv	Root-Host_associated-Human-Digestive_system-Intestine
ERR2784828	MGYA00215543	https://www.ebi.ac.uk/metagenomics/api/v1/analyses/MGYA00215543/file/ERR2784828_MERGED_FASTQ_SSU_OTU.tsv	Root-Host_associated-Human-Digestive_system-Intestine

SRR5983268	MGYA00152955	https://www.ebi.ac.uk/metagenomics/api/v1/analyses/MGYA00152955/file/SRR5983268_MERGED_FASTQ_SSU_OTU.tsv	Root-Host_associated-Human-Digestive_system-Intestine
SRR5983446	MGYA00152874	https://www.ebi.ac.uk/metagenomics/api/v1/analyses/MGYA00152874/file/SRR5983446_MERGED_FASTQ_SSU_OTU.tsv	Root-Host_associated-Human-Digestive_system-Intestine
SRR5947809	MGYA00152817	https://www.ebi.ac.uk/metagenomics/api/v1/analyses/MGYA00152817/file/SRR5947809_MERGED_FASTQ_SSU_OTU.tsv	Root-Host_associated-Human-Digestive_system-Intestine
ERR2784704	MGYA00215557	https://www.ebi.ac.uk/metagenomics/api/v1/analyses/MGYA00215557/file/ERR2784704_MERGED_FASTQ_SSU_OTU.tsv	Root-Host_associated-Human-Digestive_system-Intestine
SRR5983330	MGYA00153139	https://www.ebi.ac.uk/metagenomics/api/v1/analyses/MGYA00153139/file/SRR5983330_MERGED_FASTQ_SSU_OTU.tsv	Root-Host_associated-Human-Digestive_system-Intestine
SRR5947875	MGYA00153104	https://www.ebi.ac.uk/metagenomics/api/v1/analyses/MGYA00153104/file/SRR5947875_MERGED_FASTQ_SSU_OTU.tsv	Root-Host_associated-Human-Digestive_system-Intestine
ERR2784778	MGYA00215516	https://www.ebi.ac.uk/metagenomics/api/v1/analyses/MGYA00215516/file/ERR2784778_MERGED_FASTQ_SSU_OTU.tsv	Root-Host_associated-Human-Digestive_system-Intestine
SRR5947872	MGYA00152834	https://www.ebi.ac.uk/metagenomics/api/v1/analyses/MGYA00152834/file/SRR5947872_MERGED_FASTQ_SSU_OTU.tsv	Root-Host_associated-Human-Digestive_system-Intestine
SRR5983441	MGYA00153009	https://www.ebi.ac.uk/metagenomics/api/v1/analyses/MGYA00153009/file/SRR5983441_MERGED_FASTQ_SSU_OTU.tsv	Root-Host_associated-Human-Digestive_system-Intestine
ERR2784703	MGYA00215542	https://www.ebi.ac.uk/metagenomics/api/v1/analyses/MGYA00215542/file/ERR2784703_MERGED_FASTQ_SSU_OTU.tsv	Root-Host_associated-Human-Digestive_system-Intestine
SRR5983337	MGYA00152813	https://www.ebi.ac.uk/metagenomics/api/v1/analyses/MGYA00152813/file/SRR5983337_MERGED_FASTQ_SSU_OTU.tsv	Root-Host_associated-Human-Digestive_system-Intestine
SRR5947924	MGYA00153008	https://www.ebi.ac.uk/metagenomics/api/v1/analyses/MGYA00153008/file/SRR5947924_MERGED_FASTQ_SSU_OTU.tsv	Root-Host_associated-Human-Digestive_system-Intestine

ERR2784814	MGYA00215510	https://www.ebi.ac.uk/metagenomics/api/v1/analyses/MGYA00215510/file/ERR2784814_MERGED_FASTQ_SSU_OTU.tsv	Root-Host_associated-Human-Digestive_system-Intestine
SRR5983394	MGYA00152815	https://www.ebi.ac.uk/metagenomics/api/v1/analyses/MGYA00152815/file/SRR5983394_MERGED_FASTQ_SSU_OTU.tsv	Root-Host_associated-Human-Digestive_system-Intestine
SRR5983433	MGYA00153116	https://www.ebi.ac.uk/metagenomics/api/v1/analyses/MGYA00153116/file/SRR5983433_MERGED_FASTQ_SSU_OTU.tsv	Root-Host_associated-Human-Digestive_system-Intestine
ERR2784771	MGYA00215452	https://www.ebi.ac.uk/metagenomics/api/v1/analyses/MGYA00215452/file/ERR2784771_MERGED_FASTQ_SSU_OTU.tsv	Root-Host_associated-Human-Digestive_system-Intestine
SRR5983345	MGYA00152907	https://www.ebi.ac.uk/metagenomics/api/v1/analyses/MGYA00152907/file/SRR5983345_MERGED_FASTQ_SSU_OTU.tsv	Root-Host_associated-Human-Digestive_system-Intestine
ERR2784684	MGYA00215607	https://www.ebi.ac.uk/metagenomics/api/v1/analyses/MGYA00215607/file/ERR2784684_MERGED_FASTQ_SSU_OTU.tsv	Root-Host_associated-Human-Digestive_system-Intestine
SRR5983339	MGYA00152915	https://www.ebi.ac.uk/metagenomics/api/v1/analyses/MGYA00152915/file/SRR5983339_MERGED_FASTQ_SSU_OTU.tsv	Root-Host_associated-Human-Digestive_system-Intestine
SRR5983266	MGYA00153152	https://www.ebi.ac.uk/metagenomics/api/v1/analyses/MGYA00153152/file/SRR5983266_MERGED_FASTQ_SSU_OTU.tsv	Root-Host_associated-Human-Digestive_system-Intestine
ERR2784683	MGYA00215437	https://www.ebi.ac.uk/metagenomics/api/v1/analyses/MGYA00215437/file/ERR2784683_MERGED_FASTQ_SSU_OTU.tsv	Root-Host_associated-Human-Digestive_system-Intestine
SRR5947807	MGYA00152976	https://www.ebi.ac.uk/metagenomics/api/v1/analyses/MGYA00152976/file/SRR5947807_MERGED_FASTQ_SSU_OTU.tsv	Root-Host_associated-Human-Digestive_system-Intestine
SRR5983448	MGYA00153012	https://www.ebi.ac.uk/metagenomics/api/v1/analyses/MGYA00153012/file/SRR5983448_MERGED_FASTQ_SSU_OTU.tsv	Root-Host_associated-Human-Digestive_system-Intestine
SRR5947923	MGYA00152962	https://www.ebi.ac.uk/metagenomics/api/v1/analyses/MGYA00152962/file/SRR5947923_MERGED_FASTQ_SSU_OTU.tsv	Root-Host_associated-Human-Digestive_system-Intestine

SRR5983434	MGYA00153157	https://www.ebi.ac.uk/metagenomics/api/v1/analyses/MGYA00153157/file/SRR5983434_MERGED_FASTQ_SSU_OTU.tsv	Root-Host_associated-Human-Digestive_system-Intestine
SRR5983393	MGYA00152941	https://www.ebi.ac.uk/metagenomics/api/v1/analyses/MGYA00152941/file/SRR5983393_MERGED_FASTQ_SSU_OTU.tsv	Root-Host_associated-Human-Digestive_system-Intestine
ERR2784813	MGYA00215493	https://www.ebi.ac.uk/metagenomics/api/v1/analyses/MGYA00215493/file/ERR2784813_MERGED_FASTQ_SSU_OTU.tsv	Root-Host_associated-Human-Digestive_system-Intestine
ERR2784776	MGYA00215442	https://www.ebi.ac.uk/metagenomics/api/v1/analyses/MGYA00215442/file/ERR2784776_MERGED_FASTQ_SSU_OTU.tsv	Root-Host_associated-Human-Digestive_system-Intestine
SRR5983342	MGYA00152795	https://www.ebi.ac.uk/metagenomics/api/v1/analyses/MGYA00152795/file/SRR5983342_MERGED_FASTQ_SSU_OTU.tsv	Root-Host_associated-Human-Digestive_system-Intestine
SRR5947822	MGYA00152982	https://www.ebi.ac.uk/metagenomics/api/v1/analyses/MGYA00152982/file/SRR5947822_MERGED_FASTQ_SSU_OTU.tsv	Root-Host_associated-Human-Digestive_system-Intestine
ERR2784677	MGYA00215588	https://www.ebi.ac.uk/metagenomics/api/v1/analyses/MGYA00215588/file/ERR2784677_MERGED_FASTQ_SSU_OTU.tsv	Root-Host_associated-Human-Digestive_system-Intestine
SRR5983292	MGYA00152965	https://www.ebi.ac.uk/metagenomics/api/v1/analyses/MGYA00152965/file/SRR5983292_MERGED_FASTQ_SSU_OTU.tsv	Root-Host_associated-Human-Digestive_system-Intestine
ERR2784782	MGYA00215540	https://www.ebi.ac.uk/metagenomics/api/v1/analyses/MGYA00215540/file/ERR2784782_MERGED_FASTQ_SSU_OTU.tsv	Root-Host_associated-Human-Digestive_system-Intestine
SRR5983411	MGYA00152984	https://www.ebi.ac.uk/metagenomics/api/v1/analyses/MGYA00152984/file/SRR5983411_MERGED_FASTQ_SSU_OTU.tsv	Root-Host_associated-Human-Digestive_system-Intestine
ERR2784836	MGYA00215470	https://www.ebi.ac.uk/metagenomics/api/v1/analyses/MGYA00215470/file/ERR2784836_MERGED_FASTQ_SSU_OTU.tsv	Root-Host_associated-Human-Digestive_system-Intestine
SRR5983367	MGYA00153063	https://www.ebi.ac.uk/metagenomics/api/v1/analyses/MGYA00153063/file/SRR5983367_MERGED_FASTQ_SSU_OTU.tsv	Root-Host_associated-Human-Digestive_system-Intestine

ERR2784753	MGYA00215487	https://www.ebi.ac.uk/metagenomics/api/v1/analyses/MGYA00215487/file/ERR2784753_MERGED_FASTQ_SSU_OTU.tsv	Root-Host_associated-Human-Digestive_system-Intestine
SRR5947906	MGYA00152886	https://www.ebi.ac.uk/metagenomics/api/v1/analyses/MGYA00152886/file/SRR5947906_MERGED_FASTQ_SSU_OTU.tsv	Root-Host_associated-Human-Digestive_system-Intestine
ERR2784785	MGYA00215618	https://www.ebi.ac.uk/metagenomics/api/v1/analyses/MGYA00215618/file/ERR2784785_MERGED_FASTQ_SSU_OTU.tsv	Root-Host_associated-Human-Digestive_system-Intestine
ERR2784831	MGYA00215534	https://www.ebi.ac.uk/metagenomics/api/v1/analyses/MGYA00215534/file/ERR2784831_MERGED_FASTQ_SSU_OTU.tsv	Root-Host_associated-Human-Digestive_system-Intestine
SRR5983416	MGYA00153020	https://www.ebi.ac.uk/metagenomics/api/v1/analyses/MGYA00153020/file/SRR5983416_MERGED_FASTQ_SSU_OTU.tsv	Root-Host_associated-Human-Digestive_system-Intestine
SRR5947859	MGYA00153037	https://www.ebi.ac.uk/metagenomics/api/v1/analyses/MGYA00153037/file/SRR5947859_MERGED_FASTQ_SSU_OTU.tsv	Root-Host_associated-Human-Digestive_system-Intestine
SRR5983360	MGYA00152964	https://www.ebi.ac.uk/metagenomics/api/v1/analyses/MGYA00152964/file/SRR5983360_MERGED_FASTQ_SSU_OTU.tsv	Root-Host_associated-Human-Digestive_system-Intestine
ERR2784754	MGYA00215515	https://www.ebi.ac.uk/metagenomics/api/v1/analyses/MGYA00215515/file/ERR2784754_MERGED_FASTQ_SSU_OTU.tsv	Root-Host_associated-Human-Digestive_system-Intestine
SRR5947901	MGYA00152899	https://www.ebi.ac.uk/metagenomics/api/v1/analyses/MGYA00152899/file/SRR5947901_MERGED_FASTQ_SSU_OTU.tsv	Root-Host_associated-Human-Digestive_system-Intestine
SRR5947825	MGYA00153030	https://www.ebi.ac.uk/metagenomics/api/v1/analyses/MGYA00153030/file/SRR5947825_MERGED_FASTQ_SSU_OTU.tsv	Root-Host_associated-Human-Digestive_system-Intestine
ERR2784728	MGYA00215603	https://www.ebi.ac.uk/metagenomics/api/v1/analyses/MGYA00215603/file/ERR2784728_MERGED_FASTQ_SSU_OTU.tsv	Root-Host_associated-Human-Digestive_system-Intestine
ERR2784670	MGYA00215606	https://www.ebi.ac.uk/metagenomics/api/v1/analyses/MGYA00215606/file/ERR2784670_MERGED_FASTQ_SSU_OTU.tsv	Root-Host_associated-Human-Digestive_system-Intestine

SRR5983295	MGYA00153098	https://www.ebi.ac.uk/metagenomics/api/v1/analyses/MGYA00153098/file/SRR5983295_MERGED_FASTQ_SSU_OTU.tsv	Root-Host_associated-Human-Digestive_system-Intestine
SRR5947857	MGYA00152806	https://www.ebi.ac.uk/metagenomics/api/v1/analyses/MGYA00152806/file/SRR5947857_MERGED_FASTQ_SSU_OTU.tsv	Root-Host_associated-Human-Digestive_system-Intestine
SRR5983418	MGYA00152989	https://www.ebi.ac.uk/metagenomics/api/v1/analyses/MGYA00152989/file/SRR5983418_MERGED_FASTQ_SSU_OTU.tsv	Root-Host_associated-Human-Digestive_system-Intestine
SRR5983464	MGYA00153054	https://www.ebi.ac.uk/metagenomics/api/v1/analyses/MGYA00153054/file/SRR5983464_MERGED_FASTQ_SSU_OTU.tsv	Root-Host_associated-Human-Digestive_system-Intestine
ERR2784843	MGYA00215478	https://www.ebi.ac.uk/metagenomics/api/v1/analyses/MGYA00215478/file/ERR2784843_MERGED_FASTQ_SSU_OTU.tsv	Root-Host_associated-Human-Digestive_system-Intestine
SRR5983312	MGYA00152957	https://www.ebi.ac.uk/metagenomics/api/v1/analyses/MGYA00152957/file/SRR5983312_MERGED_FASTQ_SSU_OTU.tsv	Root-Host_associated-Human-Digestive_system-Intestine
ERR2784726	MGYA00215450	https://www.ebi.ac.uk/metagenomics/api/v1/analyses/MGYA00215450/file/ERR2784726_MERGED_FASTQ_SSU_OTU.tsv	Root-Host_associated-Human-Digestive_system-Intestine
ERR2784844	MGYA00215541	https://www.ebi.ac.uk/metagenomics/api/v1/analyses/MGYA00215541/file/ERR2784844_MERGED_FASTQ_SSU_OTU.tsv	Root-Host_associated-Human-Digestive_system-Intestine
SRR5983463	MGYA00153079	https://www.ebi.ac.uk/metagenomics/api/v1/analyses/MGYA00153079/file/SRR5983463_MERGED_FASTQ_SSU_OTU.tsv	Root-Host_associated-Human-Digestive_system-Intestine
SRR5983315	MGYA00153089	https://www.ebi.ac.uk/metagenomics/api/v1/analyses/MGYA00153089/file/SRR5983315_MERGED_FASTQ_SSU_OTU.tsv	Root-Host_associated-Human-Digestive_system-Intestine
ERR2784721	MGYA00215529	https://www.ebi.ac.uk/metagenomics/api/v1/analyses/MGYA00215529/file/ERR2784721_MERGED_FASTQ_SSU_OTU.tsv	Root-Host_associated-Human-Digestive_system-Intestine
ERR2784679	MGYA00215604	https://www.ebi.ac.uk/metagenomics/api/v1/analyses/MGYA00215604/file/ERR2784679_MERGED_FASTQ_SSU_OTU.tsv	Root-Host_associated-Human-Digestive_system-Intestine

SRR5947850	MGYA00153017	https://www.ebi.ac.uk/metagenomics/api/v1/analyses/MGYA00153017/file/SRR5947850_MERGED_FASTQ_SSU_OTU.tsv	Root-Host_associated-Human-Digestive_system-Intestine
ERR2784838	MGYA00215498	https://www.ebi.ac.uk/metagenomics/api/v1/analyses/MGYA00215498/file/ERR2784838_MERGED_FASTQ_SSU_OTU.tsv	Root-Host_associated-Human-Digestive_system-Intestine
SRR5983369	MGYA00152811	https://www.ebi.ac.uk/metagenomics/api/v1/analyses/MGYA00152811/file/SRR5983369_MERGED_FASTQ_SSU_OTU.tsv	Root-Host_associated-Human-Digestive_system-Intestine
SRR5947881	MGYA00152979	https://www.ebi.ac.uk/metagenomics/api/v1/analyses/MGYA00152979/file/SRR5947881_MERGED_FASTQ_SSU_OTU.tsv	Root-Host_associated-Human-Digestive_system-Intestine
SRR5947908	MGYA00152988	https://www.ebi.ac.uk/metagenomics/api/v1/analyses/MGYA00152988/file/SRR5947908_MERGED_FASTQ_SSU_OTU.tsv	Root-Host_associated-Human-Digestive_system-Intestine
ERR2784714	MGYA00215551	https://www.ebi.ac.uk/metagenomics/api/v1/analyses/MGYA00215551/file/ERR2784714_MERGED_FASTQ_SSU_OTU.tsv	Root-Host_associated-Human-Digestive_system-Intestine
SRR5983320	MGYA00153151	https://www.ebi.ac.uk/metagenomics/api/v1/analyses/MGYA00153151/file/SRR5983320_MERGED_FASTQ_SSU_OTU.tsv	Root-Host_associated-Human-Digestive_system-Intestine
SRR5983456	MGYA00153106	https://www.ebi.ac.uk/metagenomics/api/v1/analyses/MGYA00153106/file/SRR5983456_MERGED_FASTQ_SSU_OTU.tsv	Root-Host_associated-Human-Digestive_system-Intestine
SRR5947819	MGYA00152872	https://www.ebi.ac.uk/metagenomics/api/v1/analyses/MGYA00152872/file/SRR5947819_MERGED_FASTQ_SSU_OTU.tsv	Root-Host_associated-Human-Digestive_system-Intestine
SRR5947941	MGYA00152978	https://www.ebi.ac.uk/metagenomics/api/v1/analyses/MGYA00152978/file/SRR5947941_MERGED_FASTQ_SSU_OTU.tsv	Root-Host_associated-Human-Digestive_system-Intestine
SRR5983278	MGYA00153135	https://www.ebi.ac.uk/metagenomics/api/v1/analyses/MGYA00153135/file/SRR5983278_MERGED_FASTQ_SSU_OTU.tsv	Root-Host_associated-Human-Digestive_system-Intestine
ERR2784768	MGYA00215593	https://www.ebi.ac.uk/metagenomics/api/v1/analyses/MGYA00215593/file/ERR2784768_MERGED_FASTQ_SSU_OTU.tsv	Root-Host_associated-Human-Digestive_system-Intestine

SRR5947865	MGYA00153080	https://www.ebi.ac.uk/metagenomics/api/v1/analyses/MGYA00153080/file/SRR5947865_MERGED_FASTQ_SSU_OTU.tsv	Root-Host_associated-Human-Digestive_system-Intestine
SRR5947862	MGYA00153024	https://www.ebi.ac.uk/metagenomics/api/v1/analyses/MGYA00153024/file/SRR5947862_MERGED_FASTQ_SSU_OTU.tsv	Root-Host_associated-Human-Digestive_system-Intestine
ERR2784713	MGYA00215617	https://www.ebi.ac.uk/metagenomics/api/v1/analyses/MGYA00215617/file/ERR2784713_MERGED_FASTQ_SSU_OTU.tsv	Root-Host_associated-Human-Digestive_system-Intestine
SRR5983480	MGYA00152824	https://www.ebi.ac.uk/metagenomics/api/v1/analyses/MGYA00152824/file/SRR5983480_MERGED_FASTQ_SSU_OTU.tsv	Root-Host_associated-Human-Digestive_system-Intestine
SRR5983327	MGYA00153159	https://www.ebi.ac.uk/metagenomics/api/v1/analyses/MGYA00153159/file/SRR5983327_MERGED_FASTQ_SSU_OTU.tsv	Root-Host_associated-Human-Digestive_system-Intestine
SRR5983451	MGYA00152820	https://www.ebi.ac.uk/metagenomics/api/v1/analyses/MGYA00152820/file/SRR5983451_MERGED_FASTQ_SSU_OTU.tsv	Root-Host_associated-Human-Digestive_system-Intestine
SRR5947946	MGYA00152967	https://www.ebi.ac.uk/metagenomics/api/v1/analyses/MGYA00152967/file/SRR5947946_MERGED_FASTQ_SSU_OTU.tsv	Root-Host_associated-Human-Digestive_system-Intestine
ERR2784761	MGYA00215457	https://www.ebi.ac.uk/metagenomics/api/v1/analyses/MGYA00215457/file/ERR2784761_MERGED_FASTQ_SSU_OTU.tsv	Root-Host_associated-Human-Digestive_system-Intestine
SRR5983355	MGYA00153101	https://www.ebi.ac.uk/metagenomics/api/v1/analyses/MGYA00153101/file/SRR5983355_MERGED_FASTQ_SSU_OTU.tsv	Root-Host_associated-Human-Digestive_system-Intestine
ERR2784804	MGYA00215461	https://www.ebi.ac.uk/metagenomics/api/v1/analyses/MGYA00215461/file/ERR2784804_MERGED_FASTQ_SSU_OTU.tsv	Root-Host_associated-Human-Digestive_system-Intestine
SRR5983384	MGYA00152801	https://www.ebi.ac.uk/metagenomics/api/v1/analyses/MGYA00152801/file/SRR5983384_MERGED_FASTQ_SSU_OTU.tsv	Root-Host_associated-Human-Digestive_system-Intestine
SRR5983423	MGYA00152998	https://www.ebi.ac.uk/metagenomics/api/v1/analyses/MGYA00152998/file/SRR5983423_MERGED_FASTQ_SSU_OTU.tsv	Root-Host_associated-Human-Digestive_system-Intestine

SRR5947934	MGYA00153117	https://www.ebi.ac.uk/metagenomics/api/v1/analyses/MGYA00153117/file/SRR5947934_MERGED_FASTQ_SSU_OTU.tsv	Root-Host_associated-Human-Digestive_system-Intestine
SRR5983329	MGYA00153021	https://www.ebi.ac.uk/metagenomics/api/v1/analyses/MGYA00153021/file/SRR5983329_MERGED_FASTQ_SSU_OTU.tsv	Root-Host_associated-Human-Digestive_system-Intestine
SRR5947810	MGYA00153131	https://www.ebi.ac.uk/metagenomics/api/v1/analyses/MGYA00153131/file/SRR5947810_MERGED_FASTQ_SSU_OTU.tsv	Root-Host_associated-Human-Digestive_system-Intestine
ERR2784694	MGYA00215591	https://www.ebi.ac.uk/metagenomics/api/v1/analyses/MGYA00215591/file/ERR2784694_MERGED_FASTQ_SSU_OTU.tsv	Root-Host_associated-Human-Digestive_system-Intestine
SRR5983271	MGYA00152974	https://www.ebi.ac.uk/metagenomics/api/v1/analyses/MGYA00152974/file/SRR5983271_MERGED_FASTQ_SSU_OTU.tsv	Root-Host_associated-Human-Digestive_system-Intestine
SRR5947817	MGYA00152983	https://www.ebi.ac.uk/metagenomics/api/v1/analyses/MGYA00152983/file/SRR5947817_MERGED_FASTQ_SSU_OTU.tsv	Root-Host_associated-Human-Digestive_system-Intestine
SRR5983458	MGYA00153061	https://www.ebi.ac.uk/metagenomics/api/v1/analyses/MGYA00153061/file/SRR5983458_MERGED_FASTQ_SSU_OTU.tsv	Root-Host_associated-Human-Digestive_system-Intestine
ERR2784693	MGYA00215574	https://www.ebi.ac.uk/metagenomics/api/v1/analyses/MGYA00215574/file/ERR2784693_MERGED_FASTQ_SSU_OTU.tsv	Root-Host_associated-Human-Digestive_system-Intestine
SRR5983276	MGYA00153093	https://www.ebi.ac.uk/metagenomics/api/v1/analyses/MGYA00153093/file/SRR5983276_MERGED_FASTQ_SSU_OTU.tsv	Root-Host_associated-Human-Digestive_system-Intestine
ERR2784766	MGYA00215595	https://www.ebi.ac.uk/metagenomics/api/v1/analyses/MGYA00215595/file/ERR2784766_MERGED_FASTQ_SSU_OTU.tsv	Root-Host_associated-Human-Digestive_system-Intestine
SRR5983352	MGYA00153092	https://www.ebi.ac.uk/metagenomics/api/v1/analyses/MGYA00153092/file/SRR5983352_MERGED_FASTQ_SSU_OTU.tsv	Root-Host_associated-Human-Digestive_system-Intestine
SRR5983424	MGYA00153058	https://www.ebi.ac.uk/metagenomics/api/v1/analyses/MGYA00153058/file/SRR5983424_MERGED_FASTQ_SSU_OTU.tsv	Root-Host_associated-Human-Digestive_system-Intestine

SRR5983383	MGYA00152937	https://www.ebi.ac.uk/metagenomics/api/v1/analyses/MGYA00152937/file/SRR5983383_MERGED_FASTQ_SSU_OTU.tsv	Root-Host_associated-Human-Digestive_system-Intestine
ERR2784803	MGYA00215579	https://www.ebi.ac.uk/metagenomics/api/v1/analyses/MGYA00215579/file/ERR2784803_MERGED_FASTQ_SSU_OTU.tsv	Root-Host_associated-Human-Digestive_system-Intestine
SRR5947933	MGYA00153160	https://www.ebi.ac.uk/metagenomics/api/v1/analyses/MGYA00153160/file/SRR5947933_MERGED_FASTQ_SSU_OTU.tsv	Root-Host_associated-Human-Digestive_system-Intestine
SRR5983267	MGYA00153065	https://www.ebi.ac.uk/metagenomics/api/v1/analyses/MGYA00153065/file/SRR5983267_MERGED_FASTQ_SSU_OTU.tsv	Root-Host_associated-Human-Digestive_system-Intestine
ERR2784682	MGYA00215575	https://www.ebi.ac.uk/metagenomics/api/v1/analyses/MGYA00215575/file/ERR2784682_MERGED_FASTQ_SSU_OTU.tsv	Root-Host_associated-Human-Digestive_system-Intestine
SRR5983449	MGYA00153115	https://www.ebi.ac.uk/metagenomics/api/v1/analyses/MGYA00153115/file/SRR5983449_MERGED_FASTQ_SSU_OTU.tsv	Root-Host_associated-Human-Digestive_system-Intestine
SRR5947922	MGYA00152959	https://www.ebi.ac.uk/metagenomics/api/v1/analyses/MGYA00152959/file/SRR5947922_MERGED_FASTQ_SSU_OTU.tsv	Root-Host_associated-Human-Digestive_system-Intestine
SRR5983392	MGYA00153032	https://www.ebi.ac.uk/metagenomics/api/v1/analyses/MGYA00153032/file/SRR5983392_MERGED_FASTQ_SSU_OTU.tsv	Root-Host_associated-Human-Digestive_system-Intestine
SRR5983435	MGYA00152986	https://www.ebi.ac.uk/metagenomics/api/v1/analyses/MGYA00152986/file/SRR5983435_MERGED_FASTQ_SSU_OTU.tsv	Root-Host_associated-Human-Digestive_system-Intestine
ERR2784812	MGYA00215467	https://www.ebi.ac.uk/metagenomics/api/v1/analyses/MGYA00215467/file/ERR2784812_MERGED_FASTQ_SSU_OTU.tsv	Root-Host_associated-Human-Digestive_system-Intestine
ERR2784777	MGYA00215449	https://www.ebi.ac.uk/metagenomics/api/v1/analyses/MGYA00215449/file/ERR2784777_MERGED_FASTQ_SSU_OTU.tsv	Root-Host_associated-Human-Digestive_system-Intestine
SRR5983343	MGYA00153155	https://www.ebi.ac.uk/metagenomics/api/v1/analyses/MGYA00153155/file/SRR5983343_MERGED_FASTQ_SSU_OTU.tsv	Root-Host_associated-Human-Digestive_system-Intestine

SRR5947925	MGYA00152845	https://www.ebi.ac.uk/metagenomics/api/v1/analyses/MGYA00152845/file/SRR5947925_MERGED_FASTQ_SSU_OTU.tsv	Root-Host_associated-Human-Digestive_system-Intestine
ERR2784815	MGYA00215507	https://www.ebi.ac.uk/metagenomics/api/v1/analyses/MGYA00215507/file/ERR2784815_MERGED_FASTQ_SSU_OTU.tsv	Root-Host_associated-Human-Digestive_system-Intestine
SRR5983432	MGYA00152954	https://www.ebi.ac.uk/metagenomics/api/v1/analyses/MGYA00152954/file/SRR5983432_MERGED_FASTQ_SSU_OTU.tsv	Root-Host_associated-Human-Digestive_system-Intestine
SRR5983395	MGYA00152864	https://www.ebi.ac.uk/metagenomics/api/v1/analyses/MGYA00152864/file/SRR5983395_MERGED_FASTQ_SSU_OTU.tsv	Root-Host_associated-Human-Digestive_system-Intestine
ERR2784770	MGYA00215600	https://www.ebi.ac.uk/metagenomics/api/v1/analyses/MGYA00215600/file/ERR2784770_MERGED_FASTQ_SSU_OTU.tsv	Root-Host_associated-Human-Digestive_system-Intestine
SRR5983344	MGYA00153094	https://www.ebi.ac.uk/metagenomics/api/v1/analyses/MGYA00153094/file/SRR5983344_MERGED_FASTQ_SSU_OTU.tsv	Root-Host_associated-Human-Digestive_system-Intestine
SRR5947959	MGYA00153156	https://www.ebi.ac.uk/metagenomics/api/v1/analyses/MGYA00153156/file/SRR5947959_MERGED_FASTQ_SSU_OTU.tsv	Root-Host_associated-Human-Digestive_system-Intestine
ERR2784685	MGYA00215573	https://www.ebi.ac.uk/metagenomics/api/v1/analyses/MGYA00215573/file/ERR2784685_MERGED_FASTQ_SSU_OTU.tsv	Root-Host_associated-Human-Digestive_system-Intestine
SRR5983338	MGYA00152839	https://www.ebi.ac.uk/metagenomics/api/v1/analyses/MGYA00152839/file/SRR5983338_MERGED_FASTQ_SSU_OTU.tsv	Root-Host_associated-Human-Digestive_system-Intestine
SRR5947873	MGYA00153097	https://www.ebi.ac.uk/metagenomics/api/v1/analyses/MGYA00153097/file/SRR5947873_MERGED_FASTQ_SSU_OTU.tsv	Root-Host_associated-Human-Digestive_system-Intestine
SRR5983440	MGYA00152800	https://www.ebi.ac.uk/metagenomics/api/v1/analyses/MGYA00152800/file/SRR5983440_MERGED_FASTQ_SSU_OTU.tsv	Root-Host_associated-Human-Digestive_system-Intestine
ERR2784702	MGYA00215455	https://www.ebi.ac.uk/metagenomics/api/v1/analyses/MGYA00215455/file/ERR2784702_MERGED_FASTQ_SSU_OTU.tsv	Root-Host_associated-Human-Digestive_system-Intestine

SRR5983336	MGYA00152818	https://www.ebi.ac.uk/metagenomics/api/v1/analyses/MGYA00152818/file/SRR5983336_MERGED_FASTQ_SSU_OTU.tsv	Root-Host_associated-Human-Digestive_system-Intestine
SRR5983269	MGYA00152973	https://www.ebi.ac.uk/metagenomics/api/v1/analyses/MGYA00152973/file/SRR5983269_MERGED_FASTQ_SSU_OTU.tsv	Root-Host_associated-Human-Digestive_system-Intestine
SRR5947950	MGYA00153029	https://www.ebi.ac.uk/metagenomics/api/v1/analyses/MGYA00153029/file/SRR5947950_MERGED_FASTQ_SSU_OTU.tsv	Root-Host_associated-Human-Digestive_system-Intestine
SRR5947808	MGYA00152814	https://www.ebi.ac.uk/metagenomics/api/v1/analyses/MGYA00152814/file/SRR5947808_MERGED_FASTQ_SSU_OTU.tsv	Root-Host_associated-Human-Digestive_system-Intestine
SRR5983447	MGYA00153045	https://www.ebi.ac.uk/metagenomics/api/v1/analyses/MGYA00153045/file/SRR5983447_MERGED_FASTQ_SSU_OTU.tsv	Root-Host_associated-Human-Digestive_system-Intestine
ERR2784705	MGYA00215570	https://www.ebi.ac.uk/metagenomics/api/v1/analyses/MGYA00215570/file/ERR2784705_MERGED_FASTQ_SSU_OTU.tsv	Root-Host_associated-Human-Digestive_system-Intestine
SRR5983331	MGYA00153056	https://www.ebi.ac.uk/metagenomics/api/v1/analyses/MGYA00153056/file/SRR5983331_MERGED_FASTQ_SSU_OTU.tsv	Root-Host_associated-Human-Digestive_system-Intestine
SRR5947874	MGYA00152785	https://www.ebi.ac.uk/metagenomics/api/v1/analyses/MGYA00152785/file/SRR5947874_MERGED_FASTQ_SSU_OTU.tsv	Root-Host_associated-Human-Digestive_system-Intestine
ERR2784779	MGYA00215492	https://www.ebi.ac.uk/metagenomics/api/v1/analyses/MGYA00215492/file/ERR2784779_MERGED_FASTQ_SSU_OTU.tsv	Root-Host_associated-Human-Digestive_system-Intestine
ERR2784668	MGYA00215477	https://www.ebi.ac.uk/metagenomics/api/v1/analyses/MGYA00215477/file/ERR2784668_MERGED_FASTQ_SSU_OTU.tsv	Root-Host_associated-Human-Digestive_system-Intestine
SRR5983304	MGYA00153059	https://www.ebi.ac.uk/metagenomics/api/v1/analyses/MGYA00153059/file/SRR5983304_MERGED_FASTQ_SSU_OTU.tsv	Root-Host_associated-Human-Digestive_system-Intestine
ERR2784730	MGYA00215624	https://www.ebi.ac.uk/metagenomics/api/v1/analyses/MGYA00215624/file/ERR2784730_MERGED_FASTQ_SSU_OTU.tsv	Root-Host_associated-Human-Digestive_system-Intestine

SRR5983472	MGYA00152875	https://www.ebi.ac.uk/metagenomics/api/v1/analyses/MGYA00152875/file/SRR5983472_MERGED_FASTQ_SSU_OTU.tsv	Root-Host_associated-Human-Digestive_system-Intestine
SRR5947919	MGYA00152926	https://www.ebi.ac.uk/metagenomics/api/v1/analyses/MGYA00152926/file/SRR5947919_MERGED_FASTQ_SSU_OTU.tsv	Root-Host_associated-Human-Digestive_system-Intestine
SRR5983378	MGYA00153105	https://www.ebi.ac.uk/metagenomics/api/v1/analyses/MGYA00153105/file/SRR5983378_MERGED_FASTQ_SSU_OTU.tsv	Root-Host_associated-Human-Digestive_system-Intestine
ERR2784829	MGYA00215485	https://www.ebi.ac.uk/metagenomics/api/v1/analyses/MGYA00215485/file/ERR2784829_MERGED_FASTQ_SSU_OTU.tsv	Root-Host_associated-Human-Digestive_system-Intestine
SRR5947841	MGYA00152995	https://www.ebi.ac.uk/metagenomics/api/v1/analyses/MGYA00152995/file/SRR5947841_MERGED_FASTQ_SSU_OTU.tsv	Root-Host_associated-Human-Digestive_system-Intestine
SRR5947897	MGYA00153034	https://www.ebi.ac.uk/metagenomics/api/v1/analyses/MGYA00153034/file/SRR5947897_MERGED_FASTQ_SSU_OTU.tsv	Root-Host_associated-Human-Digestive_system-Intestine
SRR5983409	MGYA00152935	https://www.ebi.ac.uk/metagenomics/api/v1/analyses/MGYA00152935/file/SRR5983409_MERGED_FASTQ_SSU_OTU.tsv	Root-Host_associated-Human-Digestive_system-Intestine
SRR5947846	MGYA00152911	https://www.ebi.ac.uk/metagenomics/api/v1/analyses/MGYA00152911/file/SRR5947846_MERGED_FASTQ_SSU_OTU.tsv	Root-Host_associated-Human-Digestive_system-Intestine
SRR5947962	MGYA00152918	https://www.ebi.ac.uk/metagenomics/api/v1/analyses/MGYA00152918/file/SRR5947962_MERGED_FASTQ_SSU_OTU.tsv	Root-Host_associated-Human-Digestive_system-Intestine
SRR5983303	MGYA00153142	https://www.ebi.ac.uk/metagenomics/api/v1/analyses/MGYA00153142/file/SRR5983303_MERGED_FASTQ_SSU_OTU.tsv	Root-Host_associated-Human-Digestive_system-Intestine
ERR2784737	MGYA00215578	https://www.ebi.ac.uk/metagenomics/api/v1/analyses/MGYA00215578/file/ERR2784737_MERGED_FASTQ_SSU_OTU.tsv	Root-Host_associated-Human-Digestive_system-Intestine
SRR5983475	MGYA00153087	https://www.ebi.ac.uk/metagenomics/api/v1/analyses/MGYA00153087/file/SRR5983475_MERGED_FASTQ_SSU_OTU.tsv	Root-Host_associated-Human-Digestive_system-Intestine

SRR5947910	MGYA00152831	https://www.ebi.ac.uk/metagenomics/api/v1/analyses/MGYA00152831/file/SRR5947910_MERGED_FASTQ_SSU_OTU.tsv	Root-Host_associated-Human-Digestive_system-Intestine
SRR5983371	MGYA00153071	https://www.ebi.ac.uk/metagenomics/api/v1/analyses/MGYA00153071/file/SRR5983371_MERGED_FASTQ_SSU_OTU.tsv	Root-Host_associated-Human-Digestive_system-Intestine
SRR5947899	MGYA00153015	https://www.ebi.ac.uk/metagenomics/api/v1/analyses/MGYA00153015/file/SRR5947899_MERGED_FASTQ_SSU_OTU.tsv	Root-Host_associated-Human-Digestive_system-Intestine
ERR2784745	MGYA00215444	https://www.ebi.ac.uk/metagenomics/api/v1/analyses/MGYA00215444/file/ERR2784745_MERGED_FASTQ_SSU_OTU.tsv	Root-Host_associated-Human-Digestive_system-Intestine
ERR2784794	MGYA00215553	https://www.ebi.ac.uk/metagenomics/api/v1/analyses/MGYA00215553/file/ERR2784794_MERGED_FASTQ_SSU_OTU.tsv	Root-Host_associated-Human-Digestive_system-Intestine
SRR5947848	MGYA00152919	https://www.ebi.ac.uk/metagenomics/api/v1/analyses/MGYA00152919/file/SRR5947848_MERGED_FASTQ_SSU_OTU.tsv	Root-Host_associated-Human-Digestive_system-Intestine
ERR2784820	MGYA00215608	https://www.ebi.ac.uk/metagenomics/api/v1/analyses/MGYA00215608/file/ERR2784820_MERGED_FASTQ_SSU_OTU.tsv	Root-Host_associated-Human-Digestive_system-Intestine
SRR5983407	MGYA00152828	https://www.ebi.ac.uk/metagenomics/api/v1/analyses/MGYA00152828/file/SRR5983407_MERGED_FASTQ_SSU_OTU.tsv	Root-Host_associated-Human-Digestive_system-Intestine
SRR5983284	MGYA00153076	https://www.ebi.ac.uk/metagenomics/api/v1/analyses/MGYA00153076/file/SRR5983284_MERGED_FASTQ_SSU_OTU.tsv	Root-Host_associated-Human-Digestive_system-Intestine
ERR2784661	MGYA00215453	https://www.ebi.ac.uk/metagenomics/api/v1/analyses/MGYA00215453/file/ERR2784661_MERGED_FASTQ_SSU_OTU.tsv	Root-Host_associated-Human-Digestive_system-Intestine
ERR2784739	MGYA00215501	https://www.ebi.ac.uk/metagenomics/api/v1/analyses/MGYA00215501/file/ERR2784739_MERGED_FASTQ_SSU_OTU.tsv	Root-Host_associated-Human-Digestive_system-Intestine
SRR5947834	MGYA00152905	https://www.ebi.ac.uk/metagenomics/api/v1/analyses/MGYA00152905/file/SRR5947834_MERGED_FASTQ_SSU_OTU.tsv	Root-Host_associated-Human-Digestive_system-Intestine

SRR5983283	MGYA00152997	https://www.ebi.ac.uk/metagenomics/api/v1/analyses/MGYA00152997/file/SRR5983283_MERGED_FASTQ_SSU_OTU.tsv	Root-Host_associated-Human-Digestive_system-Intestine
ERR2784666	MGYA00215601	https://www.ebi.ac.uk/metagenomics/api/v1/analyses/MGYA00215601/file/ERR2784666_MERGED_FASTQ_SSU_OTU.tsv	Root-Host_associated-Human-Digestive_system-Intestine
SRR5947833	MGYA00153060	https://www.ebi.ac.uk/metagenomics/api/v1/analyses/MGYA00153060/file/SRR5947833_MERGED_FASTQ_SSU_OTU.tsv	Root-Host_associated-Human-Digestive_system-Intestine
SRR5983376	MGYA00152929	https://www.ebi.ac.uk/metagenomics/api/v1/analyses/MGYA00152929/file/SRR5983376_MERGED_FASTQ_SSU_OTU.tsv	Root-Host_associated-Human-Digestive_system-Intestine
ERR2784742	MGYA00215441	https://www.ebi.ac.uk/metagenomics/api/v1/analyses/MGYA00215441/file/ERR2784742_MERGED_FASTQ_SSU_OTU.tsv	Root-Host_associated-Human-Digestive_system-Intestine
ERR2784793	MGYA00215479	https://www.ebi.ac.uk/metagenomics/api/v1/analyses/MGYA00215479/file/ERR2784793_MERGED_FASTQ_SSU_OTU.tsv	Root-Host_associated-Human-Digestive_system-Intestine
SRR5983400	MGYA00153018	https://www.ebi.ac.uk/metagenomics/api/v1/analyses/MGYA00153018/file/SRR5983400_MERGED_FASTQ_SSU_OTU.tsv	Root-Host_associated-Human-Digestive_system-Intestine
ERR2784827	MGYA00215559	https://www.ebi.ac.uk/metagenomics/api/v1/analyses/MGYA00215559/file/ERR2784827_MERGED_FASTQ_SSU_OTU.tsv	Root-Host_associated-Human-Digestive_system-Intestine
ERR1713376	MGYA00216579	https://www.ebi.ac.uk/metagenomics/api/v1/analyses/MGYA00216579/file/ERR1713376_MERGED_FASTQ_SSU_OTU.tsv	Root-Engineered-Wastewater-Water_and_sludge
ERR1713400	MGYA00216588	https://www.ebi.ac.uk/metagenomics/api/v1/analyses/MGYA00216588/file/ERR1713400_MERGED_FASTQ_SSU_OTU.tsv	Root-Engineered-Wastewater-Water_and_sludge
ERR982958	MGYA00104300	https://www.ebi.ac.uk/metagenomics/api/v1/analyses/MGYA00104300/file/ERR982958_FASTQ_otu.tsv	Root-Engineered-Wastewater-Water_and_sludge
ERR1725971	MGYA00216559	https://www.ebi.ac.uk/metagenomics/api/v1/analyses/MGYA00216559/file/ERR1725971_MERGED_FASTQ_SSU_OTU.tsv	Root-Engineered-Wastewater-Water_and_sludge

ERR1201416	MGYA00107516	https://www.ebi.ac.uk/metagenomics/api/v1/analyses/MGYA00107516/file/ERR1201416_FASTQ_otu.tsv	Root-Engineered-Wastewater-Water_and_sludge
ERR1201944	MGYA00107525	https://www.ebi.ac.uk/metagenomics/api/v1/analyses/MGYA00107525/file/ERR1201944_FASTQ_otu.tsv	Root-Engineered-Wastewater-Water_and_sludge
ERR982924	MGYA00104243	https://www.ebi.ac.uk/metagenomics/api/v1/analyses/MGYA00104243/file/ERR982924_FASTQ_otu.tsv	Root-Engineered-Wastewater-Water_and_sludge
ERR1201995	MGYA00107563	https://www.ebi.ac.uk/metagenomics/api/v1/analyses/MGYA00107563/file/ERR1201995_FASTQ_otu.tsv	Root-Engineered-Wastewater-Water_and_sludge
ERR1201411	MGYA00107497	https://www.ebi.ac.uk/metagenomics/api/v1/analyses/MGYA00107497/file/ERR1201411_FASTQ_otu.tsv	Root-Engineered-Wastewater-Water_and_sludge
ERR1201943	MGYA00107557	https://www.ebi.ac.uk/metagenomics/api/v1/analyses/MGYA00107557/file/ERR1201943_FASTQ_otu.tsv	Root-Engineered-Wastewater-Water_and_sludge
ERR2592245	MGYA00216493	https://www.ebi.ac.uk/metagenomics/api/v1/analyses/MGYA00216493/file/ERR2592245_MERGED_FASTQ_SSU_OTU.tsv	Root-Engineered-Wastewater-Water_and_sludge
ERR982923	MGYA00104296	https://www.ebi.ac.uk/metagenomics/api/v1/analyses/MGYA00104296/file/ERR982923_FASTQ_otu.tsv	Root-Engineered-Wastewater-Water_and_sludge
ERR1201992	MGYA00107546	https://www.ebi.ac.uk/metagenomics/api/v1/analyses/MGYA00107546/file/ERR1201992_FASTQ_otu.tsv	Root-Engineered-Wastewater-Water_and_sludge
ERR1713407	MGYA00216540	https://www.ebi.ac.uk/metagenomics/api/v1/analyses/MGYA00216540/file/ERR1713407_MERGED_FASTQ_SSU_OTU.tsv	Root-Engineered-Wastewater-Water_and_sludge
ERR1725978	MGYA00216541	https://www.ebi.ac.uk/metagenomics/api/v1/analyses/MGYA00216541/file/ERR1725978_MERGED_FASTQ_SSU_OTU.tsv	Root-Engineered-Wastewater-Water_and_sludge
ERR2808665	MGYA00377455	https://www.ebi.ac.uk/metagenomics/api/v1/analyses/MGYA00377455/file/ERR2808665_FASTQ_SSU_OTU.tsv	Root-Engineered-Wastewater-Water_and_sludge

ERR1726002	MGYA00216458	https://www.ebi.ac.uk/metagenomics/api/v1/analyses/MGYA00216458/file/ERR1726002_MERGED_FASTQ_SSU_OTU.tsv	Root-Engineered-Wastewater-Water_and_sludge
ERR1713409	MGYA00216548	https://www.ebi.ac.uk/metagenomics/api/v1/analyses/MGYA00216548/file/ERR1713409_MERGED_FASTQ_SSU_OTU.tsv	Root-Engineered-Wastewater-Water_and_sludge
ERR2097564	MGYA00245882	https://www.ebi.ac.uk/metagenomics/api/v1/analyses/MGYA00245882/file/ERR2097564_MERGED_FASTQ_SSU_OTU.tsv	Root-Engineered-Wastewater-Water_and_sludge
ERR982951	MGYA00104256	https://www.ebi.ac.uk/metagenomics/api/v1/analyses/MGYA00104256/file/ERR982951_FASTQ_otu.tsv	Root-Engineered-Wastewater-Water_and_sludge
ERR1713378	MGYA00216571	https://www.ebi.ac.uk/metagenomics/api/v1/analyses/MGYA00216571/file/ERR1713378_MERGED_FASTQ_SSU_OTU.tsv	Root-Engineered-Wastewater-Water_and_sludge
ERR1726005	MGYA00216445	https://www.ebi.ac.uk/metagenomics/api/v1/analyses/MGYA00216445/file/ERR1726005_MERGED_FASTQ_SSU_OTU.tsv	Root-Engineered-Wastewater-Water_and_sludge
ERR2097563	MGYA00245881	https://www.ebi.ac.uk/metagenomics/api/v1/analyses/MGYA00245881/file/ERR2097563_MERGED_FASTQ_SSU_OTU.tsv	Root-Engineered-Wastewater-Water_and_sludge
ERR982956	MGYA00104260	https://www.ebi.ac.uk/metagenomics/api/v1/analyses/MGYA00104260/file/ERR982956_FASTQ_otu.tsv	Root-Engineered-Wastewater-Water_and_sludge
ERR1201418	MGYA00107517	https://www.ebi.ac.uk/metagenomics/api/v1/analyses/MGYA00107517/file/ERR1201418_FASTQ_otu.tsv	Root-Engineered-Wastewater-Water_and_sludge
ERR2808662	MGYA00377452	https://www.ebi.ac.uk/metagenomics/api/v1/analyses/MGYA00377452/file/ERR2808662_FASTQ_SSU_OTU.tsv	Root-Engineered-Wastewater-Water_and_sludge
ERR299251	MGYA00102797	https://www.ebi.ac.uk/metagenomics/api/v1/analyses/MGYA00102797/file/ERR299251_FASTQ_otu.tsv	Root-Engineered-Wastewater-Water_and_sludge
ERR1726030	MGYA00216616	https://www.ebi.ac.uk/metagenomics/api/v1/analyses/MGYA00216616/file/ERR1726030_MERGED_FASTQ_SSU_OTU.tsv	Root-Engineered-Wastewater-Water_and_sludge

ERR2097587	MGYA00245905	https://www.ebi.ac.uk/metagenomics/api/v1/analyses/MGYA00245905/file/ERR2097587_MERGED_FASTQ_SSU_OTU.tsv	Root-Engineered-Wastewater-Water_and_sludge
ERR982963	MGYA00104281	https://www.ebi.ac.uk/metagenomics/api/v1/analyses/MGYA00104281/file/ERR982963_FASTQ_otu.tsv	Root-Engineered-Wastewater-Water_and_sludge
ERR2808657	MGYA00377447	https://www.ebi.ac.uk/metagenomics/api/v1/analyses/MGYA00377447/file/ERR2808657_FASTQ_SSU_OTU.tsv	Root-Engineered-Wastewater-Water_and_sludge
ERR982896	MGYA00104225	https://www.ebi.ac.uk/metagenomics/api/v1/analyses/MGYA00104225/file/ERR982896_FASTQ_otu.tsv	Root-Engineered-Wastewater-Water_and_sludge
ERR2200208	MGYA00245860	https://www.ebi.ac.uk/metagenomics/api/v1/analyses/MGYA00245860/file/ERR2200208_FASTQ_SSU_OTU.tsv	Root-Engineered-Wastewater-Water_and_sludge
ERR1713331	MGYA00216627	https://www.ebi.ac.uk/metagenomics/api/v1/analyses/MGYA00216627/file/ERR1713331_MERGED_FASTQ_SSU_OTU.tsv	Root-Engineered-Wastewater-Water_and_sludge
ERR2592279	MGYA00216608	https://www.ebi.ac.uk/metagenomics/api/v1/analyses/MGYA00216608/file/ERR2592279_MERGED_FASTQ_SSU_OTU.tsv	Root-Engineered-Wastewater-Water_and_sludge
ERR2808650	MGYA00377440	https://www.ebi.ac.uk/metagenomics/api/v1/analyses/MGYA00377440/file/ERR2808650_FASTQ_SSU_OTU.tsv	Root-Engineered-Wastewater-Water_and_sludge
ERR982891	MGYA00104235	https://www.ebi.ac.uk/metagenomics/api/v1/analyses/MGYA00104235/file/ERR982891_FASTQ_otu.tsv	Root-Engineered-Wastewater-Water_and_sludge
ERR1713336	MGYA00216587	https://www.ebi.ac.uk/metagenomics/api/v1/analyses/MGYA00216587/file/ERR1713336_MERGED_FASTQ_SSU_OTU.tsv	Root-Engineered-Wastewater-Water_and_sludge
ERR982918	MGYA00104280	https://www.ebi.ac.uk/metagenomics/api/v1/analyses/MGYA00104280/file/ERR982918_FASTQ_otu.tsv	Root-Engineered-Wastewater-Water_and_sludge
ERR1201978	MGYA00107539	https://www.ebi.ac.uk/metagenomics/api/v1/analyses/MGYA00107539/file/ERR1201978_FASTQ_otu.tsv	Root-Engineered-Wastewater-Water_and_sludge

ERR2097580	MGYA00245898	https://www.ebi.ac.uk/metagenomics/api/v1/analyses/MGYA00245898/file/ERR2097580_MERGED_FASTQ_SSU_OTU.tsv	Root-Engineered-Wastewater-Water_and_sludge
ERR982964	MGYA00104277	https://www.ebi.ac.uk/metagenomics/api/v1/analyses/MGYA00104277/file/ERR982964_FASTQ_otu.tsv	Root-Engineered-Wastewater-Water_and_sludge
ERR2592328	MGYA00216674	https://www.ebi.ac.uk/metagenomics/api/v1/analyses/MGYA00216674/file/ERR2592328_MERGED_FASTQ_SSU_OTU.tsv	Root-Engineered-Wastewater-Water_and_sludge
ERR1725992	MGYA00216494	https://www.ebi.ac.uk/metagenomics/api/v1/analyses/MGYA00216494/file/ERR1725992_MERGED_FASTQ_SSU_OTU.tsv	Root-Engineered-Wastewater-Water_and_sludge
ERR1713338	MGYA00216545	https://www.ebi.ac.uk/metagenomics/api/v1/analyses/MGYA00216545/file/ERR1713338_MERGED_FASTQ_SSU_OTU.tsv	Root-Engineered-Wastewater-Water_and_sludge
ERR1725943	MGYA00216530	https://www.ebi.ac.uk/metagenomics/api/v1/analyses/MGYA00216530/file/ERR1725943_MERGED_FASTQ_SSU_OTU.tsv	Root-Engineered-Wastewater-Water_and_sludge
ERR982916	MGYA00104274	https://www.ebi.ac.uk/metagenomics/api/v1/analyses/MGYA00104274/file/ERR982916_FASTQ_otu.tsv	Root-Engineered-Wastewater-Water_and_sludge
ERR2592270	MGYA00216629	https://www.ebi.ac.uk/metagenomics/api/v1/analyses/MGYA00216629/file/ERR2592270_MERGED_FASTQ_SSU_OTU.tsv	Root-Engineered-Wastewater-Water_and_sludge
ERR1201976	MGYA00107547	https://www.ebi.ac.uk/metagenomics/api/v1/analyses/MGYA00107547/file/ERR1201976_FASTQ_otu.tsv	Root-Engineered-Wastewater-Water_and_sludge
ERR1713395	MGYA00216619	https://www.ebi.ac.uk/metagenomics/api/v1/analyses/MGYA00216619/file/ERR1713395_MERGED_FASTQ_SSU_OTU.tsv	Root-Engineered-Wastewater-Water_and_sludge
ERR1713344	MGYA00216475	https://www.ebi.ac.uk/metagenomics/api/v1/analyses/MGYA00216475/file/ERR1713344_MERGED_FASTQ_SSU_OTU.tsv	Root-Engineered-Wastewater-Water_and_sludge
ERR1713392	MGYA00216500	https://www.ebi.ac.uk/metagenomics/api/v1/analyses/MGYA00216500/file/ERR1713392_MERGED_FASTQ_SSU_OTU.tsv	Root-Engineered-Wastewater-Water_and_sludge

ERR2097589	MGYA00245907	https://www.ebi.ac.uk/metagenomics/api/v1/analyses/MGYA00245907/file/ERR2097589_MERGED_FASTQ_SSU_OTU.tsv	Root-Engineered-Wastewater-Water_and_sludge
ERR1725938	MGYA00216529	https://www.ebi.ac.uk/metagenomics/api/v1/analyses/MGYA00216529/file/ERR1725938_MERGED_FASTQ_SSU_OTU.tsv	Root-Engineered-Wastewater-Water_and_sludge
ERR1713343	MGYA00216613	https://www.ebi.ac.uk/metagenomics/api/v1/analyses/MGYA00216613/file/ERR1713343_MERGED_FASTQ_SSU_OTU.tsv	Root-Engineered-Wastewater-Water_and_sludge
ERR982898	MGYA00104263	https://www.ebi.ac.uk/metagenomics/api/v1/analyses/MGYA00104263/file/ERR982898_FASTQ_otu.tsv	Root-Engineered-Wastewater-Water_and_sludge
ERR1725995	MGYA00216597	https://www.ebi.ac.uk/metagenomics/api/v1/analyses/MGYA00216597/file/ERR1725995_MERGED_FASTQ_SSU_OTU.tsv	Root-Engineered-Wastewater-Water_and_sludge
ERR2808659	MGYA00377449	https://www.ebi.ac.uk/metagenomics/api/v1/analyses/MGYA00377449/file/ERR2808659_FASTQ_SSU_OTU.tsv	Root-Engineered-Wastewater-Water_and_sludge
ERR1725944	MGYA00216660	https://www.ebi.ac.uk/metagenomics/api/v1/analyses/MGYA00216660/file/ERR1725944_MERGED_FASTQ_SSU_OTU.tsv	Root-Engineered-Wastewater-Water_and_sludge
ERR982911	MGYA00104226	https://www.ebi.ac.uk/metagenomics/api/v1/analyses/MGYA00104226/file/ERR982911_FASTQ_otu.tsv	Root-Engineered-Wastewater-Water_and_sludge
ERR2592277	MGYA00216542	https://www.ebi.ac.uk/metagenomics/api/v1/analyses/MGYA00216542/file/ERR2592277_MERGED_FASTQ_SSU_OTU.tsv	Root-Engineered-Wastewater-Water_and_sludge
ERR1201971	MGYA00107569	https://www.ebi.ac.uk/metagenomics/api/v1/analyses/MGYA00107569/file/ERR1201971_FASTQ_otu.tsv	Root-Engineered-Wastewater-Water_and_sludge
ERR2097598	MGYA00245916	https://www.ebi.ac.uk/metagenomics/api/v1/analyses/MGYA00245916/file/ERR2097598_MERGED_FASTQ_SSU_OTU.tsv	Root-Engineered-Wastewater-Water_and_sludge
ERR1713352	MGYA00216490	https://www.ebi.ac.uk/metagenomics/api/v1/analyses/MGYA00216490/file/ERR1713352_MERGED_FASTQ_SSU_OTU.tsv	Root-Engineered-Wastewater-Water_and_sludge

ERR2592342	MGYA00216621	https://www.ebi.ac.uk/metagenomics/api/v1/analyses/MGYA00216621/file/ERR2592342_MERGED_FASTQ_SSU_OTU.tsv	Root-Engineered-Wastewater-Water_and_sludge
ERR1713383	MGYA00216503	https://www.ebi.ac.uk/metagenomics/api/v1/analyses/MGYA00216503/file/ERR1713383_MERGED_FASTQ_SSU_OTU.tsv	Root-Engineered-Wastewater-Water_and_sludge
ERR2592266	MGYA00216526	https://www.ebi.ac.uk/metagenomics/api/v1/analyses/MGYA00216526/file/ERR2592266_MERGED_FASTQ_SSU_OTU.tsv	Root-Engineered-Wastewater-Water_and_sludge
ERR1201960	MGYA00107575	https://www.ebi.ac.uk/metagenomics/api/v1/analyses/MGYA00107575/file/ERR1201960_FASTQ_otu.tsv	Root-Engineered-Wastewater-Water_and_sludge
ERR982900	MGYA00104252	https://www.ebi.ac.uk/metagenomics/api/v1/analyses/MGYA00104252/file/ERR982900_FASTQ_otu.tsv	Root-Engineered-Wastewater-Water_and_sludge
ERR2200217	MGYA00245869	https://www.ebi.ac.uk/metagenomics/api/v1/analyses/MGYA00245869/file/ERR2200217_FASTQ_SSU_OTU.tsv	Root-Engineered-Wastewater-Water_and_sludge
ERR1725955	MGYA00216583	https://www.ebi.ac.uk/metagenomics/api/v1/analyses/MGYA00216583/file/ERR1725955_MERGED_FASTQ_SSU_OTU.tsv	Root-Engineered-Wastewater-Water_and_sludge
ERR1725984	MGYA00216455	https://www.ebi.ac.uk/metagenomics/api/v1/analyses/MGYA00216455/file/ERR1725984_MERGED_FASTQ_SSU_OTU.tsv	Root-Engineered-Wastewater-Water_and_sludge
ERR2808648	MGYA00377438	https://www.ebi.ac.uk/metagenomics/api/v1/analyses/MGYA00377438/file/ERR2808648_FASTQ_SSU_OTU.tsv	Root-Engineered-Wastewater-Water_and_sludge
ERR982889	MGYA00104269	https://www.ebi.ac.uk/metagenomics/api/v1/analyses/MGYA00104269/file/ERR982889_FASTQ_otu.tsv	Root-Engineered-Wastewater-Water_and_sludge
ERR1201395	MGYA00107521	https://www.ebi.ac.uk/metagenomics/api/v1/analyses/MGYA00107521/file/ERR1201395_FASTQ_otu.tsv	Root-Engineered-Wastewater-Water_and_sludge
ERR2592261	MGYA00216558	https://www.ebi.ac.uk/metagenomics/api/v1/analyses/MGYA00216558/file/ERR2592261_MERGED_FASTQ_SSU_OTU.tsv	Root-Engineered-Wastewater-Water_and_sludge

ERR1201967	MGYA00107553	https://www.ebi.ac.uk/metagenomics/api/v1/analyses/MGYA00107553/file/ERR1201967_FASTQ_otu.tsv	Root-Engineered-Wastewater-Water_and_sludge
ERR982907	MGYA00104245	https://www.ebi.ac.uk/metagenomics/api/v1/analyses/MGYA00104245/file/ERR982907_FASTQ_otu.tsv	Root-Engineered-Wastewater-Water_and_sludge
ERR2200210	MGYA00245862	https://www.ebi.ac.uk/metagenomics/api/v1/analyses/MGYA00245862/file/ERR2200210_FASTQ_SSU_OTU.tsv	Root-Engineered-Wastewater-Water_and_sludge
ERR1725952	MGYA00216451	https://www.ebi.ac.uk/metagenomics/api/v1/analyses/MGYA00216451/file/ERR1725952_MERGED_FASTQ_SSU_OTU.tsv	Root-Engineered-Wastewater-Water_and_sludge
ERR1725983	MGYA00216675	https://www.ebi.ac.uk/metagenomics/api/v1/analyses/MGYA00216675/file/ERR1725983_MERGED_FASTQ_SSU_OTU.tsv	Root-Engineered-Wastewater-Water_and_sludge
ERR2592339	MGYA00216640	https://www.ebi.ac.uk/metagenomics/api/v1/analyses/MGYA00216640/file/ERR2592339_MERGED_FASTQ_SSU_OTU.tsv	Root-Engineered-Wastewater-Water_and_sludge
ERR1201392	MGYA00107515	https://www.ebi.ac.uk/metagenomics/api/v1/analyses/MGYA00107515/file/ERR1201392_FASTQ_otu.tsv	Root-Engineered-Wastewater-Water_and_sludge
ERR1726028	MGYA00216528	https://www.ebi.ac.uk/metagenomics/api/v1/analyses/MGYA00216528/file/ERR1726028_MERGED_FASTQ_SSU_OTU.tsv	Root-Engineered-Wastewater-Water_and_sludge
ERR1713355	MGYA00216489	https://www.ebi.ac.uk/metagenomics/api/v1/analyses/MGYA00216489/file/ERR1713355_MERGED_FASTQ_SSU_OTU.tsv	Root-Engineered-Wastewater-Water_and_sludge
ERR1713384	MGYA00216485	https://www.ebi.ac.uk/metagenomics/api/v1/analyses/MGYA00216485/file/ERR1713384_MERGED_FASTQ_SSU_OTU.tsv	Root-Engineered-Wastewater-Water_and_sludge
ERR1201969	MGYA00107541	https://www.ebi.ac.uk/metagenomics/api/v1/analyses/MGYA00107541/file/ERR1201969_FASTQ_otu.tsv	Root-Engineered-Wastewater-Water_and_sludge
ERR982909	MGYA00104217	https://www.ebi.ac.uk/metagenomics/api/v1/analyses/MGYA00104217/file/ERR982909_FASTQ_otu.tsv	Root-Engineered-Wastewater-Water_and_sludge

ERR2592337	MGYA00216567	https://www.ebi.ac.uk/metagenomics/api/v1/analyses/MGYA00216567/file/ERR2592337_MERGED_FASTQ_SSU_OTU.tsv	Root-Engineered-Wastewater-Water_and_sludge
ERR982975	MGYA00104247	https://www.ebi.ac.uk/metagenomics/api/v1/analyses/MGYA00104247/file/ERR982975_FASTQ_otu.tsv	Root-Engineered-Wastewater-Water_and_sludge
ERR1726026	MGYA00216553	https://www.ebi.ac.uk/metagenomics/api/v1/analyses/MGYA00216553/file/ERR1726026_MERGED_FASTQ_SSU_OTU.tsv	Root-Engineered-Wastewater-Water_and_sludge
ERR2097591	MGYA00245909	https://www.ebi.ac.uk/metagenomics/api/v1/analyses/MGYA00245909/file/ERR2097591_MERGED_FASTQ_SSU_OTU.tsv	Root-Engineered-Wastewater-Water_and_sludge
ERR982972	MGYA00104244	https://www.ebi.ac.uk/metagenomics/api/v1/analyses/MGYA00104244/file/ERR982972_FASTQ_otu.tsv	Root-Engineered-Wastewater-Water_and_sludge
ERR1726021	MGYA00216643	https://www.ebi.ac.uk/metagenomics/api/v1/analyses/MGYA00216643/file/ERR1726021_MERGED_FASTQ_SSU_OTU.tsv	Root-Engineered-Wastewater-Water_and_sludge
ERR2097596	MGYA00245914	https://www.ebi.ac.uk/metagenomics/api/v1/analyses/MGYA00245914/file/ERR2097596_MERGED_FASTQ_SSU_OTU.tsv	Root-Engineered-Wastewater-Water_and_sludge
ERR2592268	MGYA00216609	https://www.ebi.ac.uk/metagenomics/api/v1/analyses/MGYA00216609/file/ERR2592268_MERGED_FASTQ_SSU_OTU.tsv	Root-Engineered-Wastewater-Water_and_sludge
ERR2200219	MGYA00245871	https://www.ebi.ac.uk/metagenomics/api/v1/analyses/MGYA00245871/file/ERR2200219_FASTQ_SSU_OTU.tsv	Root-Engineered-Wastewater-Water_and_sludge
ERR982887	MGYA00104285	https://www.ebi.ac.uk/metagenomics/api/v1/analyses/MGYA00104285/file/ERR982887_FASTQ_otu.tsv	Root-Engineered-Wastewater-Water_and_sludge
ERR2808646	MGYA00377436	https://www.ebi.ac.uk/metagenomics/api/v1/analyses/MGYA00377436/file/ERR2808646_FASTQ_SSU_OTU.tsv	Root-Engineered-Wastewater-Water_and_sludge
SRR851933	MGYA00127096	https://www.ebi.ac.uk/metagenomics/api/v1/analyses/MGYA00127096/file/SRR851933_FASTQ_otu.tsv	Root-Engineered-Wastewater-Water_and_sludge

ERR982947	MGYA00104255	https://www.ebi.ac.uk/metagenomics/api/v1/analyses/MGYA00104255/file/ERR982947_FASTQ_otu.tsv	Root-Engineered-Wastewater-Water_and_sludge
ERR2097572	MGYA00245890	https://www.ebi.ac.uk/metagenomics/api/v1/analyses/MGYA00245890/file/ERR2097572_MERGED_FASTQ_SSU_OTU.tsv	Root-Engineered-Wastewater-Water_and_sludge
ERR1713369	MGYA00216487	https://www.ebi.ac.uk/metagenomics/api/v1/analyses/MGYA00216487/file/ERR1713369_MERGED_FASTQ_SSU_OTU.tsv	Root-Engineered-Wastewater-Water_and_sludge
ERR1726014	MGYA00216510	https://www.ebi.ac.uk/metagenomics/api/v1/analyses/MGYA00216510/file/ERR1726014_MERGED_FASTQ_SSU_OTU.tsv	Root-Engineered-Wastewater-Water_and_sludge
ERR574418	MGYA00103544	https://www.ebi.ac.uk/metagenomics/api/v1/analyses/MGYA00103544/file/ERR574418_FASTQ_otu.tsv	Root-Engineered-Wastewater-Water_and_sludge
ERR1201409	MGYA00107519	https://www.ebi.ac.uk/metagenomics/api/v1/analyses/MGYA00107519/file/ERR1201409_FASTQ_otu.tsv	Root-Engineered-Wastewater-Water_and_sludge
ERR1725969	MGYA00216665	https://www.ebi.ac.uk/metagenomics/api/v1/analyses/MGYA00216665/file/ERR1725969_MERGED_FASTQ_SSU_OTU.tsv	Root-Engineered-Wastewater-Water_and_sludge
ERR982940	MGYA00104273	https://www.ebi.ac.uk/metagenomics/api/v1/analyses/MGYA00104273/file/ERR982940_FASTQ_otu.tsv	Root-Engineered-Wastewater-Water_and_sludge
ERR2097575	MGYA00245893	https://www.ebi.ac.uk/metagenomics/api/v1/analyses/MGYA00245893/file/ERR2097575_MERGED_FASTQ_SSU_OTU.tsv	Root-Engineered-Wastewater-Water_and_sludge
ERR1726013	MGYA00216664	https://www.ebi.ac.uk/metagenomics/api/v1/analyses/MGYA00216664/file/ERR1726013_MERGED_FASTQ_SSU_OTU.tsv	Root-Engineered-Wastewater-Water_and_sludge
ERR574411	MGYA00103540	https://www.ebi.ac.uk/metagenomics/api/v1/analyses/MGYA00103540/file/ERR574411_FASTQ_otu.tsv	Root-Engineered-Wastewater-Water_and_sludge
ERR982932	MGYA00104270	https://www.ebi.ac.uk/metagenomics/api/v1/analyses/MGYA00104270/file/ERR982932_FASTQ_otu.tsv	Root-Engineered-Wastewater-Water_and_sludge

ERR1201983	MGYA00107580	https://www.ebi.ac.uk/metagenomics/api/v1/analyses/MGYA00107580/file/ERR1201983_FASTQ_otu.tsv	Root-Engineered-Wastewater-Water_and_sludge
ERR1201952	MGYA00107545	https://www.ebi.ac.uk/metagenomics/api/v1/analyses/MGYA00107545/file/ERR1201952_FASTQ_otu.tsv	Root-Engineered-Wastewater-Water_and_sludge
ERR1201400	MGYA00107502	https://www.ebi.ac.uk/metagenomics/api/v1/analyses/MGYA00107502/file/ERR1201400_FASTQ_otu.tsv	Root-Engineered-Wastewater-Water_and_sludge
ERR1725967	MGYA00216573	https://www.ebi.ac.uk/metagenomics/api/v1/analyses/MGYA00216573/file/ERR1725967_MERGED_FASTQ_SSU_OTU.tsv	Root-Engineered-Wastewater-Water_and_sludge
ERR2200225	MGYA00245877	https://www.ebi.ac.uk/metagenomics/api/v1/analyses/MGYA00245877/file/ERR2200225_FASTQ_SSU_OTU.tsv	Root-Engineered-Wastewater-Water_and_sludge
ERR1713360	MGYA00216457	https://www.ebi.ac.uk/metagenomics/api/v1/analyses/MGYA00216457/file/ERR1713360_MERGED_FASTQ_SSU_OTU.tsv	Root-Engineered-Wastewater-Water_and_sludge
ERR982949	MGYA00104293	https://www.ebi.ac.uk/metagenomics/api/v1/analyses/MGYA00104293/file/ERR982949_FASTQ_otu.tsv	Root-Engineered-Wastewater-Water_and_sludge
ERR1713411	MGYA00216595	https://www.ebi.ac.uk/metagenomics/api/v1/analyses/MGYA00216595/file/ERR1713411_MERGED_FASTQ_SSU_OTU.tsv	Root-Engineered-Wastewater-Water_and_sludge
ERR1713367	MGYA00216670	https://www.ebi.ac.uk/metagenomics/api/v1/analyses/MGYA00216670/file/ERR1713367_MERGED_FASTQ_SSU_OTU.tsv	Root-Engineered-Wastewater-Water_and_sludge
ERR574416	MGYA00103541	https://www.ebi.ac.uk/metagenomics/api/v1/analyses/MGYA00103541/file/ERR574416_FASTQ_otu.tsv	Root-Engineered-Wastewater-Water_and_sludge
ERR982935	MGYA00104291	https://www.ebi.ac.uk/metagenomics/api/v1/analyses/MGYA00104291/file/ERR982935_FASTQ_otu.tsv	Root-Engineered-Wastewater-Water_and_sludge
ERR2592282	MGYA00216614	https://www.ebi.ac.uk/metagenomics/api/v1/analyses/MGYA00216614/file/ERR2592282_MERGED_FASTQ_SSU_OTU.tsv	Root-Engineered-Wastewater-Water_and_sludge

ERR1201984	MGYA00107552	https://www.ebi.ac.uk/metagenomics/api/v1/analyses/MGYA00107552/file/ERR1201984_FASTQ_otu.tsv	Root-Engineered-Wastewater-Water_and_sludge
ERR1201955	MGYA00107567	https://www.ebi.ac.uk/metagenomics/api/v1/analyses/MGYA00107567/file/ERR1201955_FASTQ_otu.tsv	Root-Engineered-Wastewater-Water_and_sludge
ERR2592253	MGYA00216628	https://www.ebi.ac.uk/metagenomics/api/v1/analyses/MGYA00216628/file/ERR2592253_MERGED_FASTQ_SSU_OTU.tsv	Root-Engineered-Wastewater-Water_and_sludge
ERR1201407	MGYA00107514	https://www.ebi.ac.uk/metagenomics/api/v1/analyses/MGYA00107514/file/ERR1201407_FASTQ_otu.tsv	Root-Engineered-Wastewater-Water_and_sludge
ERR1725960	MGYA00216538	https://www.ebi.ac.uk/metagenomics/api/v1/analyses/MGYA00216538/file/ERR1725960_MERGED_FASTQ_SSU_OTU.tsv	Root-Engineered-Wastewater-Water_and_sludge
ERR2200222	MGYA00245874	https://www.ebi.ac.uk/metagenomics/api/v1/analyses/MGYA00245874/file/ERR2200222_FASTQ_SSU_OTU.tsv	Root-Engineered-Wastewater-Water_and_sludge
ERR1713393	MGYA00216601	https://www.ebi.ac.uk/metagenomics/api/v1/analyses/MGYA00216601/file/ERR1713393_MERGED_FASTQ_SSU_OTU.tsv	Root-Engineered-Wastewater-Water_and_sludge
ERR1725939	MGYA00216625	https://www.ebi.ac.uk/metagenomics/api/v1/analyses/MGYA00216625/file/ERR1725939_MERGED_FASTQ_SSU_OTU.tsv	Root-Engineered-Wastewater-Water_and_sludge
ERR2097588	MGYA00245906	https://www.ebi.ac.uk/metagenomics/api/v1/analyses/MGYA00245906/file/ERR2097588_MERGED_FASTQ_SSU_OTU.tsv	Root-Engineered-Wastewater-Water_and_sludge
ERR1713342	MGYA00216576	https://www.ebi.ac.uk/metagenomics/api/v1/analyses/MGYA00216576/file/ERR1713342_MERGED_FASTQ_SSU_OTU.tsv	Root-Engineered-Wastewater-Water_and_sludge
ERR2808658	MGYA00377448	https://www.ebi.ac.uk/metagenomics/api/v1/analyses/MGYA00377448/file/ERR2808658_FASTQ_SSU_OTU.tsv	Root-Engineered-Wastewater-Water_and_sludge
ERR1725994	MGYA00216663	https://www.ebi.ac.uk/metagenomics/api/v1/analyses/MGYA00216663/file/ERR1725994_MERGED_FASTQ_SSU_OTU.tsv	Root-Engineered-Wastewater-Water_and_sludge

ERR982899	MGYA00104240	https://www.ebi.ac.uk/metagenomics/api/v1/analyses/MGYA00104240/file/ERR982899_FASTQ_otu.tsv	Root-Engineered-Wastewater-Water_and_sludge
ERR1725945	MGYA00216495	https://www.ebi.ac.uk/metagenomics/api/v1/analyses/MGYA00216495/file/ERR1725945_MERGED_FASTQ_SSU_OTU.tsv	Root-Engineered-Wastewater-Water_and_sludge
ERR982910	MGYA00104253	https://www.ebi.ac.uk/metagenomics/api/v1/analyses/MGYA00104253/file/ERR982910_FASTQ_otu.tsv	Root-Engineered-Wastewater-Water_and_sludge
ERR2592276	MGYA00216460	https://www.ebi.ac.uk/metagenomics/api/v1/analyses/MGYA00216460/file/ERR2592276_MERGED_FASTQ_SSU_OTU.tsv	Root-Engineered-Wastewater-Water_and_sludge
ERR1201970	MGYA00107565	https://www.ebi.ac.uk/metagenomics/api/v1/analyses/MGYA00107565/file/ERR1201970_FASTQ_otu.tsv	Root-Engineered-Wastewater-Water_and_sludge
ERR1725993	MGYA00216536	https://www.ebi.ac.uk/metagenomics/api/v1/analyses/MGYA00216536/file/ERR1725993_MERGED_FASTQ_SSU_OTU.tsv	Root-Engineered-Wastewater-Water_and_sludge
ERR2592329	MGYA00216520	https://www.ebi.ac.uk/metagenomics/api/v1/analyses/MGYA00216520/file/ERR2592329_MERGED_FASTQ_SSU_OTU.tsv	Root-Engineered-Wastewater-Water_and_sludge
ERR1713339	MGYA00216672	https://www.ebi.ac.uk/metagenomics/api/v1/analyses/MGYA00216672/file/ERR1713339_MERGED_FASTQ_SSU_OTU.tsv	Root-Engineered-Wastewater-Water_and_sludge
ERR1725942	MGYA00216492	https://www.ebi.ac.uk/metagenomics/api/v1/analyses/MGYA00216492/file/ERR1725942_MERGED_FASTQ_SSU_OTU.tsv	Root-Engineered-Wastewater-Water_and_sludge
ERR982917	MGYA00104238	https://www.ebi.ac.uk/metagenomics/api/v1/analyses/MGYA00104238/file/ERR982917_FASTQ_otu.tsv	Root-Engineered-Wastewater-Water_and_sludge
ERR2592271	MGYA00216464	https://www.ebi.ac.uk/metagenomics/api/v1/analyses/MGYA00216464/file/ERR2592271_MERGED_FASTQ_SSU_OTU.tsv	Root-Engineered-Wastewater-Water_and_sludge
ERR1201977	MGYA00107556	https://www.ebi.ac.uk/metagenomics/api/v1/analyses/MGYA00107556/file/ERR1201977_FASTQ_otu.tsv	Root-Engineered-Wastewater-Water_and_sludge

ERR1713394	MGYA00216563	https://www.ebi.ac.uk/metagenomics/api/v1/analyses/MGYA00216563/file/ERR1713394_MERGED_FASTQ_SSU_OTU.tsv	Root-Engineered-Wastewater-Water_and_sludge
ERR1713345	MGYA00216635	https://www.ebi.ac.uk/metagenomics/api/v1/analyses/MGYA00216635/file/ERR1713345_MERGED_FASTQ_SSU_OTU.tsv	Root-Engineered-Wastewater-Water_and_sludge
ERR982890	MGYA00104248	https://www.ebi.ac.uk/metagenomics/api/v1/analyses/MGYA00104248/file/ERR982890_FASTQ_otu.tsv	Root-Engineered-Wastewater-Water_and_sludge
ERR2808651	MGYA00377441	https://www.ebi.ac.uk/metagenomics/api/v1/analyses/MGYA00377441/file/ERR2808651_FASTQ_SSU_OTU.tsv	Root-Engineered-Wastewater-Water_and_sludge
ERR1713337	MGYA00216444	https://www.ebi.ac.uk/metagenomics/api/v1/analyses/MGYA00216444/file/ERR1713337_MERGED_FASTQ_SSU_OTU.tsv	Root-Engineered-Wastewater-Water_and_sludge
ERR1201979	MGYA00107577	https://www.ebi.ac.uk/metagenomics/api/v1/analyses/MGYA00107577/file/ERR1201979_FASTQ_otu.tsv	Root-Engineered-Wastewater-Water_and_sludge
ERR2097581	MGYA00245899	https://www.ebi.ac.uk/metagenomics/api/v1/analyses/MGYA00245899/file/ERR2097581_MERGED_FASTQ_SSU_OTU.tsv	Root-Engineered-Wastewater-Water_and_sludge
ERR982965	MGYA00104282	https://www.ebi.ac.uk/metagenomics/api/v1/analyses/MGYA00104282/file/ERR982965_FASTQ_otu.tsv	Root-Engineered-Wastewater-Water_and_sludge
ERR299250	MGYA00102794	https://www.ebi.ac.uk/metagenomics/api/v1/analyses/MGYA00102794/file/ERR299250_FASTQ_otu.tsv	Root-Engineered-Wastewater-Water_and_sludge
ERR1726031	MGYA00216603	https://www.ebi.ac.uk/metagenomics/api/v1/analyses/MGYA00216603/file/ERR1726031_MERGED_FASTQ_SSU_OTU.tsv	Root-Engineered-Wastewater-Water_and_sludge
ERR2097586	MGYA00245904	https://www.ebi.ac.uk/metagenomics/api/v1/analyses/MGYA00245904/file/ERR2097586_MERGED_FASTQ_SSU_OTU.tsv	Root-Engineered-Wastewater-Water_and_sludge
ERR982962	MGYA00104232	https://www.ebi.ac.uk/metagenomics/api/v1/analyses/MGYA00104232/file/ERR982962_FASTQ_otu.tsv	Root-Engineered-Wastewater-Water_and_sludge

ERR982897	MGYA00104249	https://www.ebi.ac.uk/metagenomics/api/v1/analyses/MGYA00104249/file/ERR982897_FASTQ_otu.tsv	Root-Engineered-Wastewater-Water_and_sludge
ERR2808656	MGYA00377446	https://www.ebi.ac.uk/metagenomics/api/v1/analyses/MGYA00377446/file/ERR2808656_FASTQ_SSU_OTU.tsv	Root-Engineered-Wastewater-Water_and_sludge
ERR2200209	MGYA00245861	https://www.ebi.ac.uk/metagenomics/api/v1/analyses/MGYA00245861/file/ERR2200209_FASTQ_SSU_OTU.tsv	Root-Engineered-Wastewater-Water_and_sludge
ERR2592278	MGYA00216589	https://www.ebi.ac.uk/metagenomics/api/v1/analyses/MGYA00216589/file/ERR2592278_MERGED_FASTQ_SSU_OTU.tsv	Root-Engineered-Wastewater-Water_and_sludge
ERR1713379	MGYA00216533	https://www.ebi.ac.uk/metagenomics/api/v1/analyses/MGYA00216533/file/ERR1713379_MERGED_FASTQ_SSU_OTU.tsv	Root-Engineered-Wastewater-Water_and_sludge
ERR1726004	MGYA00216650	https://www.ebi.ac.uk/metagenomics/api/v1/analyses/MGYA00216650/file/ERR1726004_MERGED_FASTQ_SSU_OTU.tsv	Root-Engineered-Wastewater-Water_and_sludge
ERR982957	MGYA00104261	https://www.ebi.ac.uk/metagenomics/api/v1/analyses/MGYA00104261/file/ERR982957_FASTQ_otu.tsv	Root-Engineered-Wastewater-Water_and_sludge
ERR1201419	MGYA00107512	https://www.ebi.ac.uk/metagenomics/api/v1/analyses/MGYA00107512/file/ERR1201419_FASTQ_otu.tsv	Root-Engineered-Wastewater-Water_and_sludge
ERR2808663	MGYA00377453	https://www.ebi.ac.uk/metagenomics/api/v1/analyses/MGYA00377453/file/ERR2808663_FASTQ_SSU_OTU.tsv	Root-Engineered-Wastewater-Water_and_sludge
ERR1725979	MGYA00216561	https://www.ebi.ac.uk/metagenomics/api/v1/analyses/MGYA00216561/file/ERR1725979_MERGED_FASTQ_SSU_OTU.tsv	Root-Engineered-Wastewater-Water_and_sludge
ERR2808664	MGYA00377454	https://www.ebi.ac.uk/metagenomics/api/v1/analyses/MGYA00377454/file/ERR2808664_FASTQ_SSU_OTU.tsv	Root-Engineered-Wastewater-Water_and_sludge
ERR1726003	MGYA00216649	https://www.ebi.ac.uk/metagenomics/api/v1/analyses/MGYA00216649/file/ERR1726003_MERGED_FASTQ_SSU_OTU.tsv	Root-Engineered-Wastewater-Water_and_sludge

ERR1713408	MGYA00216605	https://www.ebi.ac.uk/metagenomics/api/v1/analyses/MGYA00216605/file/ERR1713408_MERGED_FASTQ_SSU_OTU.tsv	Root-Engineered-Wastewater-Water_and_sludge
ERR2097565	MGYA00245883	https://www.ebi.ac.uk/metagenomics/api/v1/analyses/MGYA00245883/file/ERR2097565_MERGED_FASTQ_SSU_OTU.tsv	Root-Engineered-Wastewater-Water_and_sludge
ERR982950	MGYA00104286	https://www.ebi.ac.uk/metagenomics/api/v1/analyses/MGYA00104286/file/ERR982950_FASTQ_otu.tsv	Root-Engineered-Wastewater-Water_and_sludge
ERR1725977	MGYA00216509	https://www.ebi.ac.uk/metagenomics/api/v1/analyses/MGYA00216509/file/ERR1725977_MERGED_FASTQ_SSU_OTU.tsv	Root-Engineered-Wastewater-Water_and_sludge
ERR2097600	MGYA00245918	https://www.ebi.ac.uk/metagenomics/api/v1/analyses/MGYA00245918/file/ERR2097600_MERGED_FASTQ_SSU_OTU.tsv	Root-Engineered-Wastewater-Water_and_sludge
ERR1201410	MGYA00107501	https://www.ebi.ac.uk/metagenomics/api/v1/analyses/MGYA00107501/file/ERR1201410_FASTQ_otu.tsv	Root-Engineered-Wastewater-Water_and_sludge
[truncated: 411,720 more chars]
