# Supplementary material for: Meta-Prism 2.0: Enabling algorithm and web server for ultra-fast, memory-efficient, and accurate analysis among millions of microbial community samples
Source: Gigascience. 2022 Jul 28;11:giac073. doi: 10.1093/gigascience/giac073 (PMC9334027; doi:10.1093/gigascience/giac073)
Supplement: giac073_Response_to_Reviewer_Comments_Original_Submission [file giac073_response_to_reviewer_comments_original_submission.pdf]

Author's Response To Reviewer Comments

Close

We thank editor for these suggestions, and thank reviewers for questions and comments. We have updated the manuscript for clarity, as well as deployed all real data applications' workflow to the Code Ocean platform (https://codeocean.com/capsule/3103931). We also modified the program naming, added program documentation and did more tests to improve program robustness.

However, performance and accuracy analysis require similarity matrix calculations for the entire database, which require, as we show in Figure 4, more than 353 hours of CPU time to calculate. Therefore, we did not deploy this part into Code Ocean, but provided step introductions, intermediate data and supporting scripts in Google Drive (the FTP temporary account provided before seems to have been used by others), and the sharing link is https://drive.google.com/drive/folders/1BB7jyUBigw7ysjY_LCmVEdpaIZSlod-l?usp=sharing.

We have submitted a point to point docx formatted response file as "Respond.docx", please check it for details.

Close
