# Supplementary material for: Meta-Prism 2.0: Enabling algorithm and web server for ultra-fast, memory-efficient, and accurate analysis among millions of microbial community samples
Source: Gigascience. 2022 Jul 28;11:giac073. doi: 10.1093/gigascience/giac073 (PMC9334027; doi:10.1093/gigascience/giac073)
Supplement: giac073_Response_to_Reviewer_Comments_Revision_1 [file giac073_response_to_reviewer_comments_revision_1.pdf]

Author's Response To Reviewer Comments

Close

We thank editor and reviewer for suggestion and comment. We have updated the manuscript according to this comment and reformatting manuscript as "Technical Note".

The FEAST dataset is built based on the biome annotations of the EBI MGnify database (https://docs.mgnify.org/en/latest/glossary.html?highlight=biome#term-Biome). About the definitions of three biomes from FEAST (Fecal, Human, and Mixed). Precise categorization of Fecal is "Root-Host_associated-Human-Digestive_system-Large_intestine-Fecal". Precise categorization of Human is "Root-Host_associated-Human", which is a broad range of human-related samples,such as forehead, skin, oral, sebum, etc. Precise categorization of Mixed is "Root-Mixed", which covers broader samples, such as door knobs, kitchen counter, light switch etc. The detailed names of Human, Fecal, Mixed and the IDs, URLs and notes of all samples in the FEAST dataset are provided in Supplementary Table 1. We also updated the manuscript to make it easier for readers to understand these biomes.

We have submitted a point to point docx formatted response file as "Respond.docx", please check it for details.

Close
