# Supplementary material for: Meta-Prism 2.0: Enabling algorithm and web server for ultra-fast, memory-efficient, and accurate analysis among millions of microbial community samples
Source: Gigascience. 2022 Jul 28;11:giac073. doi: 10.1093/gigascience/giac073 (PMC9334027; doi:10.1093/gigascience/giac073)
Supplement: giac073_Reviewer_1_Report_Original_Submission — Siyuan Ma -- 12/29/2021 Reviewed [file giac073_reviewer_1_report_original_submission.pdf]

Reviewer Report

Title: Meta-Prism 2.0: Enabling algorithm and web server for ultra-fast, memory-efficient, and accurate analysis among millions of microbial community samples

Version: Original Submission **Date:** 12/29/2021

Reviewer name: Siyuan Ma

Reviewer Comments to Author:

In Kang, Chong, and Ning, the authors present Meta-Prism 2, a microbial community analysis framework, which calculates sample-sample dissimilarities and queries microbial profiles similar to those of user-provided targets. Meta-Prism 2 adopts efficient algorithms to achieve the time and memory efficiency required for modern microbiome "big data" application scenarios. The authors evaluated Meta-Prism 2's performance, both in terms of separating different biomes' microbial profiles and time/memory usage, on a variety of real-world studies.

I find the application target of Meta-Prism appealing: achieving efficient dissimilarity profiling is increasingly relevant for modern microbiome applications. However, I'm afraid the manuscript appears to be in poor state, with insufficient details for crucial methods and results components. Some display items are either missing or mis-referenced. As such, I cannot recommend for its acceptance, unless major improvements are made. My comments are detailed below.

Major

1. The authors claim that from its previous iteration, the biggest improvements are: (1) removal of redundant nodes in 1-against-N sample comparisons. (2) functionality for similarity matrix calculation (3) exhaustive search among all available samples.

a. (1) seems the most crucial for the method's improved efficiency. However, the details on why these nodes can be eliminated, and how dissimilarity calculation is achieved post-elimination are not sufficient. The caption for Figure 1C, and relevant Methods texts (lines 173-188) should be expanded, to at least explain i) why it is valid to calculate (dis)similarity post elimination based on aggregation, ii) how aggregation is achieved for the target samples.

b. I may not have understood the authors on (2), but this improvement seems trivial? Is it simply that Meta-Prism 2 has a new function to calculate all pair-wise dissimilarities on a collection of microbial profiles?

c. For (3), it should be made clearer that Meta-Prism 1 does not do this. I needed to read the authors' previous paper to understand the comment about better flexibility in customized datasets. I assume that this improvement is enabled because Meta-Prism 2 is vastly faster compared to 1? If so, it might be helpful to point this out explicitly.

2. I am lost on the accuracy evaluation results for predicting different biomes (Figure 2).

a. How are biomes predicted for each microbial sample?

b. What is the varying classification threshold that generates different sensitivities and specificities?

c. Does "cross-validation" refer to e.g. selection of tuning parameters during model training, or for evaluation model performances?

d. What are the "Fecal", "Human", and "Combined" biomes for the Feast cohort? Such details were not provided in Shenhav et al.

Moderate

1. I understand that this was previously published, but could the authors comment on the intuitions behind their dissimilarity measure, and how it compares to similar measures such as the weighted UniFrac?

a. Does Meta-Storm and Meta-Prism share the same similarity definition? If so, why would they differ in terms of prediction accuracies?

2. There seems to be some mis-referencing on the panels of Figure 1.

a. Panel B was not explained at all in the figure caption.

b. Line 185 references Figure 1E, which does not exist.

Minor

1. The Meta-Prism 1 publication was referenced with duplicates (#16 and 24)

2. There are minor language issues throughout the manuscript, but for they do not affect understanding of the materials. Examples:

a. Line 94: analysis -> analyze

b. Line 193: We also obtained a dataset that consists of …

Level of Interest

Please indicate how interesting you found the manuscript: Choose an item.

Quality of Written English

Please indicate the quality of language in the manuscript: Choose an item.

Declaration of Competing Interests

Please complete a declaration of competing interests, considering the following questions:

- Have you in the past five years received reimbursements, fees, funding, or salary from an organisation that may in any way gain or lose financially from the publication of this manuscript, either now or in the future?
- Do you hold any stocks or shares in an organisation that may in any way gain or lose financially from the publication of this manuscript, either now or in the future?
- Do you hold or are you currently applying for any patents relating to the content of the manuscript?
- Have you received reimbursements, fees, funding, or salary from an organization that holds or has applied for patents relating to the content of the manuscript?
- Do you have any other financial competing interests?
- Do you have any non-financial competing interests in relation to this paper?

If you can answer no to all of the above, write 'I declare that I have no competing interests' below. If your reply is yes to any, please give details below.

I declare that I have no competing interests

I agree to the open peer review policy of the journal. I understand that my name will be included on my report to the authors and, if the manuscript is accepted for publication, my named report including any attachments I upload will be posted on the website along with the authors' responses. I agree for my report to be made available under an Open Access Creative Commons CC-BY license (http://creativecommons.org/licenses/by/4.0/). I understand that any comments which I do not wish to be included in my named report can be included as confidential comments to the editors, which will not be published.

Choose an item.

To further support our reviewers, we have joined with Publons, where you can gain additional credit to further highlight your hard work (see: https://publons.com/journal/530/gigascience). On publication of this paper, your review will be automatically added to Publons, you can then choose whether or not to claim your Publons credit. I understand this statement.

Yes Choose an item.
